# Supplementary material for: Factors influencing unmet need for contraception amongst adolescent girls and women in Cambodia
Source: PeerJ. 2020 Oct 7;8:e10065. doi: 10.7717/peerj.10065 (PMC7547592; doi:10.7717/peerj.10065)
Supplement: Supplemental Information 1 [file peerj-08-10065-s001.pdf]

RE: DHS Download Account Application

Wellington, Bridgette <Bridgette.Wellington@icf.com>

Wed 6/20/2018 7:45 PM

To: FARWA RIZVI <rizvifa@deakin.edu.au>;

Dear Farwa,

1. Questionnaires are in the appendix of the final country report: https://www.dhsprogram.com/pubs/pdf/FR312/FR312.pdf
2. The codebook is within the zip file you downloaded from our website – the file with a .MAP extension. Open this with any text editor
3. All DHS data user support is provided through [userforum.dhsprogram.com]The DHS Program User Forum. The User Forum is a great resource, and provides a space for DHS data users to interact, ask questions, and help each other. DHS review posts periodically, and respond to any questions that data users cannot answer. Before posting question(s), please search the forum to see if your topic has already been covered.

Thanks – Bridgette

-----Original Message-----
From: FARWA RIZVI <rizvifa@deakin.edu.au>
Sent: Wednesday, June 20, 2018 3:00 AM
To: archive@dhsprogram.com
Subject: Re: DHS Download Account Application

Hi,

My name is Farwa Rizvi and I'm in my second year of PhD at the Deakin University, VIC, Australia.

I'd received the permission to access CDHS 2014 data for the secondary data analysis in September 2017.

I have started the secondary data analysis of the Cambodia DHS data 2014 this year. I'm new to STATA 15 SE and just finding my way around the ways to apply logistic regression analysis.

I'd like to request you to please email me a booklet of the Cambodia DHS 2014 data questionnaire and codebook which was used for the data analysis. Also, if possible, could I request for some statistical help for the analysis in STATA from one of your experts? I could share my research question for one of my papers.

Thanks,
Farwa

Dr Farwa Rizvi
PhD candidate
School of Health and Social Development
Burwood campus, Deakin University

From: archive@dhsprogram.com <archive@dhsprogram.com>
Sent: Tuesday, July 11, 2017 1:27 AM
To: FARWA RIZVI

Subject: DHS Download Account Application

Please see attached.

You have been authorized to download "Survey" data from the Demographic and Health Surveys (DHS) Program. To begin downloading, please login at: http://www.dhsprogram.com/data/dataset_admin/login_main.cfm . If you are approved for a large number of countries, please consider using the Bulk Download System. For instructions on bulk downloading, please go to: http://userforum.dhsprogram.com/index.php?t=msg&th=5246 .

The requested data should only be used for the purpose of the registered research or study. To use the data for another purpose, a new research project must be "created" in your account. All DHS data should be treated as confidential, and no effort should be made to identify any household or individual respondent interviewed in the survey. The data must not be passed on to other researchers (other than co-researchers listed in your account), without the written consent of DHS. Users are required to submit a copy of any reports/publications resulting from using the DHS data files to: archive@dhsprogram.com.

The files you will download are in zipped format and must be unzipped before analysis. After unzipping, please print the file with the .DOC/DOCX extension (found in the Individual and Male Recode Zips). This file contains useful information on country specific variables and differences in the Standard Recode definition. You will also need the DHS Recode Manual: http://dhsprogram.com/publications/publication-dhsg4-dhs-questionnaires-and-manuals.cfm . This manual contains a general description of the recode data file, including the rationale for recoding; a description of coding standards and recode variables, and a listing of the standard dictionary, with basic information relating to each variable.

It is essential that you consult the questionnaire for the country, when using the data files. Questionnaires are in the appendices of each survey's final report: http://dhsprogram.com/publications/publications-by-type.cfm . We also recommend that you make use of the Data Tools and Manuals at:
http://www.dhsprogram.com/accesssurveys/technical_assistance.cfm .

For problems with your user account, please email archive@dhsprogram.com. For data related questions, please register to participate in the DHS Program User Forum at: http://userforum.dhsprogram.com .

The Demographic and Health Surveys (DHS) Program ICF
530 Gaither Road
Suite 500
Rockville, MD 20850
USA

LOGIN INFORMATION:
Login Email: rizvifa@deakin.edu.au
Password: (use password selected when you registered)
