# Supplementary material for: Factors influencing unmet need for contraception amongst adolescent girls and women in Cambodia
Source: PeerJ. 2020 Oct 7;8:e10065. doi: 10.7717/peerj.10065 (PMC7547592; doi:10.7717/peerj.10065)
Supplement: Supplemental Information 2 [file peerj-08-10065-s002.pdf]

Figure 2: Percent distribution of unmet need for contraception in sexually active Cambodian females (urban and rural)

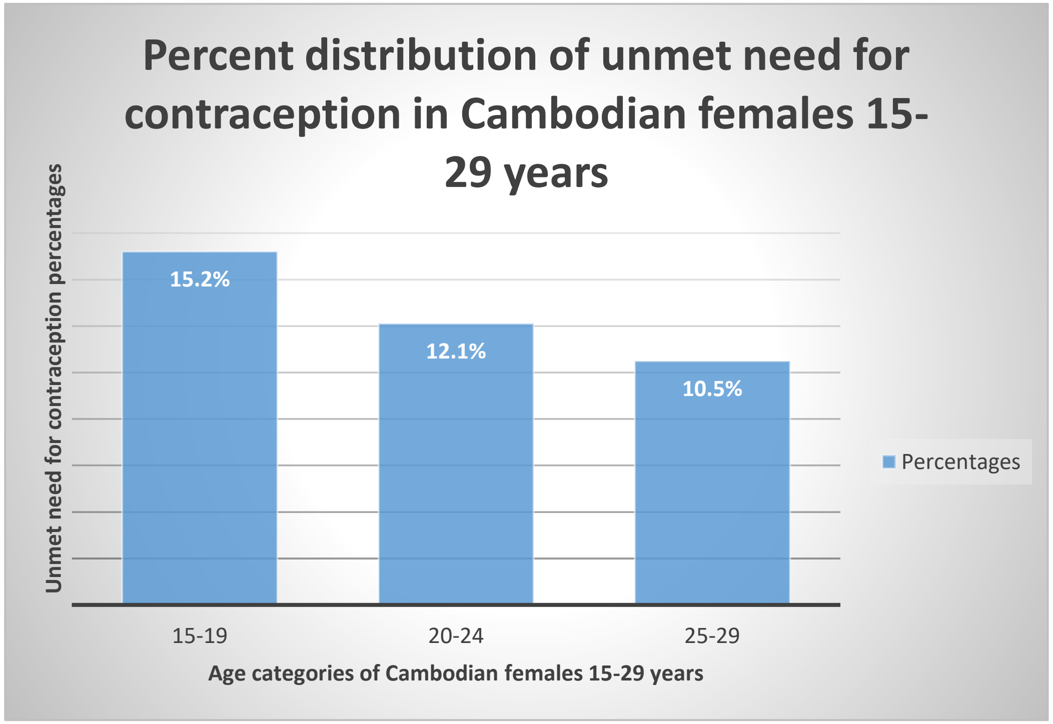

*Prevalence of unmet need for contraception was highest (15.2%) amongst the adolescent girls aged 15-19 years

** Dataset obtained from 2014 Cambodian Demographic and Health Survey (n= 4823 sexually active females aged 15-29 years)

1. Bronfenbrenner U. The ecology of human development: Harvard university press; 1979.

1. Bronfenbrenner U. The ecology of human development: Harvard university press; 1979.
