# Supplementary material for: Factors influencing unmet need for contraception amongst adolescent girls and women in Cambodia
Source: PeerJ. 2020 Oct 7;8:e10065. doi: 10.7717/peerj.10065 (PMC7547592; doi:10.7717/peerj.10065)
Supplement: Supplemental Information 3 [file peerj-08-10065-s003.docx]

**Questions and Filters for Unmet Need Definition**Note that question text has been modified slightly from the DHS questionnaire to reflect the information needed for
the definition of unmet need and avoid extraneous questions. Skip patterns have been modified to reflect the flow of
questions in this set of questions. For the original questions and skip patterns, please see the DHS Model
Questionnaire (Phase 6) (http://www.measuredhs.com/publications/publication-DHSQ6-DHS-Questionnaires-andManuals.cfm)

| NO. | QUESTIONS AND FILTERS | CODING CATEGORIES | SKIP |
| --- | --- | --- | --- |
| 226 | YES . . . . . . . . . . . . . . . . . . . . . . . . . . . . . 1 NO . . . . . . . . . . . . . . . . . . . . . . . . . . . . . 2 UNSURE . . . . . . . . . . . . . . . . . . . . . . . . . 8 | 238 | Are you pregnant now? |

| 228 | YES . . . . . . . . . . . . . . . . . . . . . . . . . . . . . 1  NO . . . . . . . . . . . . . . . . . . . . . . . . . . . . . 2 | 238 | When you got pregnant, did you want to get pregnant at that time? |
| --- | --- | --- | --- |
| 229 | LATER . . . . . . . . . . . . . . . . . . . . . . . . . . . 1 NO MORE . . . . . . . . . . . . . . . . . . . . . . 2 | Did you want to have a baby later on or did you not want any (more) children? |  |

| 238 | DAYS AGO . . . . . . . . . . . . 1 WEEKS AGO . . . . . . . . . . 2 MONTHS AGO . . . . . . . . . . 3 YEARS AGO . . . . . . . . . . 4 IN MENOPAUSE/ HAS HAD HYSTERECTOMY . . . 994 BEFORE LAST BIRTH . . . . . . . . . . . . 995 NEVER MENSTRUATED . . . . . . . . . . 996 | (DATE, IF GIVEN)  When did your last menstrual period start? |
| --- | --- | --- |

| 302 | CHECK 226: NOT PREGNANT PREGNANT OR UNSURE | 313 |
| --- | --- | --- |
| 303 | YES . . . . . . . . . . . . . . . . . . . . . . . . . . . . . 1 NO . . . . . . . . . . . . . . . . . . . . . . . . . . . . . 2 | Are you currently doing something or using any method to delay or avoid getting pregnant? |

| 313 | YES . . . . . . . . . . . . . . . . . . . . . . . . . . . . . 1 NO . . . . . . . . . . . . . . . . . . . . . . . . . . . . . 2 | Have you ever used anything or tried in any way to delay or avoid getting pregnant? |
| --- | --- | --- |

| 212 | NAME | What name was given to your (last) baby? RECORD NAME |
| --- | --- | --- |

| 215 | MONTH . . . . . . . . . . . . . . . . . . YEAR . . . . . . . . . . . . | In what month and year was (NAME) born? PROBE: When is his/her birthday? |
| --- | --- | --- |

| NO. | QUESTIONS AND FILTERS | CODING CATEGORIES | SKIP |
| --- | --- | --- | --- |
| 401 | CHECK 215: BIRTH BIRTH  IN 2006 BEFORE OR LATER 2006 | 601 |  |

| 405 | YES . . . . . . . . . . . . . . . . . . . . . . . . . . . . . 1  NO . . . . . . . . . . . . . . . . . . . . . . . . . . . . . 2 | 447 | When you got pregnant with (NAME), did you want to get pregnant at that time? |
| --- | --- | --- | --- |
| 406 | LATER . . . . . . . . . . . . . . . . . . . . . . . . . 1 NO MORE . . . . . . . . . . . . . . . . . . . . . . 2 | Did you want to have a baby later on, or did you not want any (more) children? |  |

| 447 | YES . . . . . . . . . . . . . . . . . . . . . . . . . . . . . 1 NO . . . . . . . . . . . . . . . . . . . . . . . . . . . . . 2 | Has your menstrual period returned since the birth of (NAME)? |
| --- | --- | --- |

| 601 | YES, CURRENTLY MARRIED 1 YES, LIVING WITH A MAN . . . . . . . . . . 2 NO, NOT IN UNION . . . . . . . . . . . . . . . . 3 . . . . . . . . | Are you currently married or living together with a man as if married? |
| --- | --- | --- |

| 610 | MONTH . . . . . . . . . . . . . . . . . . DON'T KNOW MONTH . . . . . . . . . . . . . . 98 YEAR . . . . . . . . . . . . DON'T KNOW YEAR . . . . . . . . . . . . 9998 | Now I would like to ask about your (first) (husband/partner). In what month and year did you start living with him? |
| --- | --- | --- |

| 615 | DAYS AGO . . . . . . . . . . . . 1 WEEKS AGO . . . . . . . . . . 2 MONTHS AGO . . . . . . . . 3 YEARS AGO . . . . . . . . . . 4 | IF LESS THAN 12 MONTHS, ANSWER MUST BE RECORDED IN DAYS, WEEKS OR MONTHS.  IF 12 MONTHS (ONE YEAR) OR MORE, ANSWER MUST BE RECORDED IN YEARS.  When was the last time you had sexual intercourse? |
| --- | --- | --- |

| 703 | HAVE ANOTHER CHILD . . . . . . . . . . . . 1  NO MORE . . . . . . . . . . . . . . . . . . . . . . 2 UNDECIDED/DON'T KNOW . . . . . . . . . . 8 | 705 END | Now I have some questions about the future. After the child you are expecting now, would you like to have another child, or would you prefer not to have any more children? |
| --- | --- | --- | --- |
| 704 | HAVE (A/ANOTHER) CHILD . . . . . . . . . . 1 NO MORE/NONE . . . . . . . . . . . . . . . . . . 2  SAYS SHE CAN'T GET PREGNANT 3 UNDECIDED/DON'T KNOW . . . . . . . . . . 8 | 707 END | Now I have some questions about the future. Would you like to have (a/another) child, or would you prefer not to have any (more) children? |
| 705 | CHECK 226: NOT PREGNANT PREGNANT OR UNSURE  How long would you like to wait from now before the birth of (a/another) child? After the birth of the child you are expecting now, how long would you like to wait before the birth of another child? | MONTHS . . . . . . . . . . . . . . 1 YEARS . . . . . . . . . . . . . . 2 SOON/NOW . . . . . . . . . . . . . . . . . . 993  SAYS SHE CAN'T GET PREGNANT 994  AFTER MARRIAGE . . . . . . . . . . . . . . 995 OTHER _______________________ 996  (SPECIFY) DON'T KNOW . . . . . . . . . . . . . . . . . . 998 | END END END |

| NO. | QUESTIONS AND FILTERS | CODING CATEGORIES | SKIP |
| --- | --- | --- | --- |
| 706 | CHECK 226: NOT PREGNANT PREGNANT OR UNSURE | END |  |
| 707 | CHECK 303: USING A CONTRACEPTIVE METHOD? USING | END |  |
| 708 | CHECK 705: NOT 24 OR MORE MONTHS 00-23 MONTHS ASKED OR 02 OR MORE YEARS OR 00-01 YEAR | END |  |
| 709 | CHECK 704:  WANTS TO HAVE WANTS NO MORE/  A/ANOTHER CHILD NONE  RECORD ALL REASONS MENTIONED.  Any other reason? Any other reason? You have said that you do not want (a/another) child soon. You have said that you do not want any (more) children. Can you tell me why you are not using a method to prevent pregnancy? Can you tell me why you are not using a method to prevent pregnancy? | NOT MARRIED . . . . . . . . . . . . . . . . . . A FERTILITY-RELATED REASONS NOT HAVING SEX . . . . . . . . . . . . . . B INFREQUENT SEX . . . . . . . . . . . . . . C MENOPAUSAL/HYSTERECTOMY D CAN'T GET PREGNANT . . . . . . . . . . E NOT MENSTRUATED SINCE LAST BIRTH . . . . . . . . . . . . . . . . F BREASTFEEDING . . . . . . . . . . . . . . G UP TO GOD/FATALISTIC . . . . . . . . . . H OPPOSITION TO USE RESPONDENT OPPOSED . . . . . . . . I HUSBAND/PARTNER OPPOSED . . . J OTHERS OPPOSED . . . . . . . . . . . . K RELIGIOUS PROHIBITION . . . . . . . . L LACK OF KNOWLEDGE KNOWS NO METHOD . . . . . . . . . . . . M KNOWS NO SOURCE . . . . . . . . . . . . N METHOD-RELATED REASONS SIDE EFFECTS/HEALTH CONCERNS . . . . . . . . . . . . . . . . . . O LACK OF ACCESS/TOO FAR . . . . . P COSTS TOO MUCH . . . . . . . . . . . . Q PREFERRED METHOD NOT AVAILABLE . . . . . . . . . . . . . . R NO METHOD AVAILABLE . . . . . . . . S INCONVENIENT TO USE . . . . . . . . T INTERFERES WITH BODY'S NORMAL PROCESSES . . . . . . . . U OTHER _______________________ X (SPECIFY) DON'T KNOW . . . . . . . . . . . . . . . . . . . . Z |  |

NOT CURRENTLY
CURRENTLY USING
