# Supplementary material for: Factors influencing unmet need for contraception amongst adolescent girls and women in Cambodia
Source: PeerJ. 2020 Oct 7;8:e10065. doi: 10.7717/peerj.10065 (PMC7547592; doi:10.7717/peerj.10065)
Supplement: Supplemental Information 4 [file peerj-08-10065-s004.doc]

STROBE Statement—Checklist of items that should be included in reports of ***cross-sectional studies***

|  | Item No | Recommendation |
| --- | --- | --- |
| **Title and abstract** | 1 | (*a*) Indicate the study’s design with a commonly used term in the title or the abstract **√** |
|  |  | (*b*) Provide in the abstract an informative and balanced summary of what was done and what was found √ |
| Introduction | | |
| Background/rationale | 2 | Explain the scientific background and rationale for the investigation being reported√ |
| Objectives | 3 | State specific objectives, including any prespecified hypotheses√ |
| Methods | | |
| Study design | 4 | Present key elements of study design early in the paper√ |
| Setting | 5 | Describe the setting, locations, and relevant dates, including periods of recruitment, exposure, follow-up, and data collection√ |
| Participants | 6 | (*a*) Give the eligibility criteria, and the sources and methods of selection of participants√ |
| Variables | 7 | Clearly define all outcomes, exposures, predictors, potential confounders, and effect modifiers. Give diagnostic criteria, if applicable√ |
| Data sources/ measurement | 8* | For each variable of interest, give sources of data and details of methods of assessment (measurement). Describe comparability of assessment methods if there is more than one group √ |
| Bias | 9 | Describe any efforts to address potential sources of bias √ |
| Study size | 10 | Explain how the study size was arrived at √ |
| Quantitative variables | 11 | Explain how quantitative variables were handled in the analyses. If applicable, describe which groupings were chosen and why √ |
| Statistical methods | 12 | (*a*) Describe all statistical methods, including those used to control for confounding √ |
|  |  | (*b*) Describe any methods used to examine subgroups and interactions √ |
|  |  | (*c*) Explain how missing data were addressed √ |
|  |  | (*d*) If applicable, describe analytical methods taking account of sampling strategy√ |
|  |  | (*e*) Describe any sensitivity analyses N/A |
| Results | | |
| Participants | 13* | (a) Report numbers of individuals at each stage of study—eg numbers potentially eligible, examined for eligibility, confirmed eligible, included in the study, completing follow-up, and analysed√ |
|  |  | (b) Give reasons for non-participation at each stage√ |
|  |  | (c) Consider use of a flow diagram√ |
| Descriptive data | 14* | (a) Give characteristics of study participants (eg demographic, clinical, social) and information on exposures and potential confounders√ |
|  |  | (b) Indicate number of participants with missing data for each variable of interest√ |
| Outcome data | 15* | Report numbers of outcome events or summary measures√ |
| Main results | 16 | (*a*) Give unadjusted estimates and, if applicable, confounder-adjusted estimates and their precision (eg, 95% confidence interval). Make clear which confounders were adjusted for and why they were included (Descriptive analyses)- Yes, AOR were reported |
|  |  | (*b*) Report category boundaries when continuous variables were categorized N/A |
|  |  | (*c*) If relevant, consider translating estimates of relative risk into absolute risk for a meaningful time period N/A |
| Other analyses | 17 | Report other analyses done—eg analyses of subgroups and interactions, and sensitivity analyses N/A |
| Discussion | | |
| Key results | 18 | Summarise key results with reference to study objectives √ |
| Limitations | 19 | Discuss limitations of the study, taking into account sources of potential bias or imprecision. Discuss both direction and magnitude of any potential bias √ |
| Interpretation | 20 | Give a cautious overall interpretation of results considering objectives, limitations, multiplicity of analyses, results from similar studies, and other relevant evidence √ |
| Generalisability | 21 | Discuss the generalisability (external validity) of the study results √ |
| Other information | | |
| Funding | 22 | Give the source of funding and the role of the funders for the present study and, if applicable, for the original study on which the present article is based √ |

*Give information separately for exposed and unexposed groups.

**Note:** An Explanation and Elaboration article discusses each checklist item and gives methodological background and published examples of transparent reporting. Information on the STROBE Initiative is available at www.strobe-statement.org.
