# Supplementary material for: Factors influencing unmet need for contraception amongst adolescent girls and women in Cambodia
Source: PeerJ. 2020 Oct 7;8:e10065. doi: 10.7717/peerj.10065 (PMC7547592; doi:10.7717/peerj.10065)
Supplement: Supplemental Information 5 [file peerj-08-10065-s005.docx]

Variable names

| v394 | Visited health facility in last 12 months |
| --- | --- |
| V467d | Distance to health facility for getting medical help for self |
| regionnew | Urban/rural region |
| agenew | Age groups |
| v201 | Total children even born (parity) |
| newwhc | Person who decides on respondent’s health care |
| newwhhp | Person who decides on large household purchases |
| v714 | Respondent currently working |
| v621 | Husband’s desire for children |
| V384a | Heard about family planning on radio in the last few months |
| V384b | Heard about family planning on TV in the last few months |
| v394 | Visited health facility in the last 12 months |

Bivariate descriptive analyse
