# Supplementary material for: Factors influencing unmet need for contraception amongst adolescent girls and women in Cambodia
Source: PeerJ. 2020 Oct 7;8:e10065. doi: 10.7717/peerj.10065 (PMC7547592; doi:10.7717/peerj.10065)
Supplement: Supplemental Information 8 [file peerj-08-10065-s008.docx]

**Outcome (dependent) variable**

The indicator unmet need for contraception is calculated as; unmet need = unmet need spacing + unmet need limiting and used as a binary variable ‘Yes/No’ (Bradley et al. 2012; USAID 2015). The 2014 CDHS with the revised definition of unmet need provides the following categories (Bradley et al. 2012).

- ***No*** unmet need if women want a birth within two years
- ***‘Unmet need for spacing’*** for all women who are sexually active, and want to space their next pregnancy but are not using any contraceptive methods
- ***‘Unmet need for limiting’*** for women who want to limit the next pregnancy but are not using any contraceptive methods
- ‘***No*** unmet need’ for women who want to delay next pregnancy and are using a family planning method so termed *‘****using for spacing’***
- ‘***No*** unmet need’ if women do not want to have any more births and are using any form of contraception so termed as *‘****using for limiting’***
- ‘***No*** unmet need’ if women are using contraception but they say that they are sterilized or do not want any more births or cannot get pregnant, they are categorized as ‘using for limiting’
- ‘***No*** unmet need’ if women are using any contraception but they say that they want to become pregnant shortly, or after some time, or are unsure about either the timing of a pregnancy, or not certain if they want to have a baby, are all categorized as ‘using for spacing’

**References**

Bradley, SE, Croft, TN, Fishel, JD & Westoff, CF 2012, *Revising Unmet Need for Family Planning*, United States Agency for International Development, Calverton, Maryland, USA, <[https://dhsprogram.com/pubs/pdf/AS25/AS25[12June2012].pdf](https://dhsprogram.com/pubs/pdf/AS25/AS25%5b12June2012%5d.pdf)>.

USAID 2015, *Unmet Need for Family Planning-Family Planning and Reproductive Health Indicators*, , <<https://www.measureevaluation.org/prh/rh_indicators/family-planning/fp/unmet-need-for-family-planning>>.
