# Supplementary material for: Factors influencing unmet need for contraception amongst adolescent girls and women in Cambodia
Source: PeerJ. 2020 Oct 7;8:e10065. doi: 10.7717/peerj.10065 (PMC7547592; doi:10.7717/peerj.10065)
Supplement: Supplemental Information 9 [file peerj-08-10065-s009.docx]

**What is already known**

- In Cambodia in 2014, 33% Cambodian females aged 15-24 years have been using modern contraception methods and 13% females have been using traditional methods.
- This shows a big gap between knowledge and use of contraception. The World Health Organisation (WHO) reports that 30% of married Cambodian women aged 15-24 years do not want to become pregnant, but they either do not use any contraception methods or use traditional methods.
- Little information is available about the multiple factors associated with ‘unmet need for contraception’ among sexually active adolescent girls and women under 30 years in urban and rural Cambodia.

**What this paper adds**

- There was an increased likelihood of unmet need for contraception in adolescent girls 15-19 years and young women 20-24 years compared to 25-29-year-old women.
- Unmet need for contraception in Cambodian females 15-29 years is associated with increased parity, unemployment, difficulty in accessing a nearby health centre, low personal autonomy for healthcare and deciding about the family size, but not with education or wealth status.

**What insights does this paper provide for informing policy decision-making**

- There is a need to implement culturally appropriate sexual and reproductive health literacy policies and programs to improve informed decision making in adolescent girls and women for accessing modern contraception.
- Cambodia can achieve the targets set for Sustainable Development Goal 3 as proposed by the United Nations for gender equality, and improved personal, financial and reproductive health autonomy of women by increasing social awareness amongst youth, women, and couples in Cambodian society.
