# Supplementary material for: Factors influencing unmet need for contraception amongst adolescent girls and women in Cambodia
Source: PeerJ. 2020 Oct 7;8:e10065. doi: 10.7717/peerj.10065 (PMC7547592; doi:10.7717/peerj.10065)
Supplement: Supplemental Information 10 [file peerj-08-10065-s010.pdf]

USAID
FROM THE AMERICAN PEOPLE

REVISING UNMET NEED FOR FAMILY PLANNING

DHS ANALYTICAL STUDIES 25

JANUARY 2012

This publication was produced for review by the United States Agency for International Development. It was prepared by Sarah E.K. Bradley, Trevor N. Croft, and Joy D. Fishel of ICF International and Charles F. Westoff of the Office of Population Research, Princeton University.

MEASURE DHS assists countries worldwide in the collection and use of data to monitor and evaluate population, health, and nutrition programs. Additional information about the MEASURE DHS project can be obtained by contacting MEASURE DHS, ICF International, 11785 Beltsville Drive, Suite 300, Calverton, MD 20705 (telephone: 301-572-0200; fax: 301-572-0999; e-mail: reports@measuredhs.com; internet: www.measuredhs.com).

The main objectives of the MEASURE DHS project are:

- to provide decisionmakers in survey countries with information useful for informed policy choices;
- to expand the international population and health database;
- to advance survey methodology; and
- to develop in participating countries the skills and resources necessary to conduct high-quality demographic and health surveys.

DHS Analytical Studies No. 25

Revising Unmet Need for Family Planning

Sarah E.K. Bradley [1]

Trevor N. Croft [1]

Joy D. Fishel [1]

Charles F. Westoff [2]

With acknowledgment to the Technical Expert Working Group (TEWG) on Unmet Need for Family Planning, including technical experts John Bongaarts, Population Council; John Casterline, Ohio State University; Amy O. Tsui, Johns Hopkins Bloomberg School of Public Health; and Charles F. Westoff, Princeton University; USAID participants Jacob Adetunji, Yoonjoung Choi, and Scott Radloff; UNFPA participants Stan Bernstein and Edilberto Loaiza; and MEASURE DHS participants Sunita Kishor, Shea Rutstein, and Ann Way

ICF International
Calverton, Maryland, USA

January 2012

Corresponding author: Sarah E.K. Bradley. Demographic and Health Research Division, ICF International, 11785 Beltsville Drive, Calverton, Maryland 20705, USA; Phone 301-572-0282; Fax 301-572-0999; Email: SBradley@icfi.com

[1] ICF International
[2] Office of Population Research, Princeton University

Editor: Bryant Robey
Document Production: Yuan Cheng

This study was carried out with support provided by the United States Agency for International Development (USAID) through the MEASURE DHS project (#GPO-C-00-08-00008-00). The views expressed are those of the authors and do not necessarily reflect the views of USAID or the United States Government.

Recommended citation:

Bradley, Sarah E.K., Trevor N. Croft, Joy D. Fishel, and Charles F. Westoff. 2012. *Revising Unmet Need for Family Planning*. DHS Analytical Studies No. 25. Calverton, Maryland, USA: ICF International.

Contents

List of Tables v

List of Figures vi

Preface xi

Executive Summary xiii

Introduction 1

1. The Original Definition of Unmet Need for Family Planning 3

1.1 Changes over Time in the Definition of Unmet Need for Family Planning 3
1.1.1 Spacing and Limiting 3
1.1.2 Infecundity 3
1.1.3 Pregnancy and Postpartum Amenorrhea 4
1.1.4 Calendar Data 5
1.1.5 Other Questionnaire Changes 6

1.2 Examining the Original Definition 7
1.2.1 Women Using Contraception (Group 1) 7
1.2.2 Women Who are Pregnant or Postpartum Amenorrheic (Groups 2a & 2b) 7
1.2.3 Women Who are Not Pregnant and Not Postpartum Amenorrheic (Groups 3 & 4) 8
1.2.4 Alterations in Surveys without Calendar Data on Reasons for Discontinuation and/or Marital Status 9
1.2.5 Application of Algorithm to All Women 12

2. The Revised Definition of Unmet Need for Family Planning 13

2.1 Rationale for Revising the Definition of Unmet Need 13

2.2 The Process of Revising the Definition of Unmet Need 13

2.3 Revisions to the Definition of Unmet Need 14

3. The Impact of Revising Unmet Need 23

3.1 Changes in Total Unmet Need 23
3.1.1 Countries That Implemented the Calendar in Every Survey 29
3.1.2 Countries That Did Not Collect Calendar Data in Any Survey 30
3.1.3 Countries That Collected Calendar Data in Some Surveys but Not Others 31

3.2 Changes in Unmet Need for Spacing and Limiting 33

4. New Estimates of Unmet Need and Demand for Family Planning Using the Revised Definition 39

4.1 Demand for Family Planning and Proportion of Demand Satisfied......................................39
4.1.1 Trends in the Proportion of Demand Satisfied 44
4.1.2 The Proportion of Demand Satisfied by Modern Methods.......................................44
4.2 Unmet Need by Background Characteristics 46
4.2.1 Urban-Rural Residence..46
4.2.2 Education 47
4.2.3 Household Wealth..48
4.2.4 Parity and Age...49
4.3 Unmet Need among Sexually Active Unmarried Women 52

5. Estimating the Demographic Impact of Fulfilling Unmet Need....................................55

6. Conclusions...59

References 61

Appendix A. Questions and Filters Needed for Unmet Need Definition.................................63

List of Tables

Table 1. Total unmet need for family planning, summary 24

Table 2. Total unmet need for family planning, all surveys 25

Table 3. Unmet need for spacing and limiting 33

Table 4. Unmet need, contraceptive use, and demand for family planning, currently married women 40

Table 5. Unmet need, contraceptive use, and demand for family planning, sexually active unmarried women 54

Table 6. Current contraceptive use, unmet need, and total demand for limiting 54

Appendix Table 1. Unmet need by residence, education, and wealth quintile 66

Appendix Table 2. Unmet need by parity and age 69

List of Figures

Figure 1. Original definition of unmet need, currently married women 10

Figure 2. Revised definition of unmet need, currently married women..11

Figure 3. Egypt 1992-2008, total unmet need Original and Revised definitions, married women 15-49 29

Figure 4. Zimbabwe 1994-2006, total unmet need Original and Revised definitions, married women 15-49 29

Figure 5. Indonesia 1991-2007, total unmet need Original and Revised definitions, married women 15-49 29

Figure 6. Peru 1991-2008, total unmet need Original and Revised definitions, married women 15-49 29

Figure 7. Haiti 1994-2006, total unmet need Original and Revised definitions, married women 15-49 30

Figure 8. Madagascar 1992-2009, total unmet need Original and Revised definitions, married women 15-49 30

Figure 9. Nepal 1996-2006, total unmet need Original and Revised definitions, married women 15-49 30

Figure 10. Niger 1992-2006, total unmet need Original and Revised definitions, married women 15-49 30

Figure 11. Kenya 1993-2009, total unmet need Original and Revised definitions, married women 15-49 32

Figure 12. Bolivia 1994-2008, total unmet need Original and Revised definitions, married women 15-49 32

Figure 13. Dominican Republic 1991-2007, total unmet need Original and Revised definitions, married women 15-49..32

Figure 14. Colombia 1990-2010, total unmet need Original and Revised definitions, married women 15-49 32

Figure 15. Philippines 1993-2008, total unmet need Original and Revised definitions, married women 15-49 32

Figure 16. Jordan 1990-2009, total unmet need Original and Revised definitions, married women 15-49 32

Figure 17a. Bolivia 1994-2008, unmet need for spacing Original and Revised definitions, married women 15-49 38

Figure 17b. Bolivia 1994-2008, unmet need for limiting Original and Revised definitions, married women 15-49 38

Figure 18a. Dominican Republic 1991-2007, unmet need for spacing Original and Revised definitions, married women 15-49 38

Figure 18b. Dominican Republic 1991-2007, unmet need for limiting Original and Revised definitions, married women 15-49 38

Figure 19a. Philippines1993-2008, unmet need for spacing Original and Revised definitions, married women 15-49 38

Figure 19b. Philippines 1993-2008, unmet need for limiting Original and Revised definitions, married women 15-49 38

Figure 20a. Madagascar 1992-2009, proportion of total demand satisfied, married women 15-49 45

Figure 20b. Madagascar 1992-2009, contraceptive prevalence and unmet need, married women 15-49 45

Figure 21a. Jordan 1990-2009, proportion of total demand satisfied, married women 15-49 45

Figure 21b. Jordan 1990-2009, contraceptive prevalence and unmet need, married women 15-49 45

Figure 22a. Tanzania 1991-2010, proportion of total demand satisfied, married women 15-49 45

Figure 22b. Tanzania 1991-2010, contraceptive prevalence and unmet need, married women 15-49 45

Figure 23. Kenya 2008-09, unmet need by residence, married women 15-49 47

Figure 24. Egypt 2008, unmet need by residence, married women 15-49 47

Figure 25. Albania 2008-09, unmet need by residence, married women 15-49 47

Figure 26. Mozambique 2003, unmet need by residence, married women 15-49 47

Figure 27. Moldova 2005, unmet need by residence, married women 15-49 47

Figure 28. Guyana 2009, unmet need by residence, married women 15-49 47

Figure 29. Sierra Leone 2008, unmet need by residence, married women 15-49 47

Figure 30. Niger 2006, unmet need by residence, married women 15-49 47

Figure 31. Ghana 2008, unmet need by residence, married women 15-49 47

Figure 32. Jordan 2009, unmet need by education, married women 15-49 48

Figure 33. Bolivia 2008, unmet need by education, married women 15-49 48

Figure 34. Liberia 2007, unmet need by education, married women 15-49 48

Figure 35. D. R. Congo 2007, unmet need by education, married women 15-49..........................48

Figure 36. Ethiopia 2005, unmet need by education, married women 15-49 48

Figure 37. Swaziland 2006-07, unmet need by education, married women 15-49........................48

Figure 38. Kenya 2008-09, unmet need by wealth quintile, married women 15-49.......................49

Figure 39. Haiti 2005-06, unmet need by wealth quintile, married women 15-49 49

Figure 40. Armenia 2005, unmet need by wealth quintile, married women 15-49.........................49

Figure 41. Azerbaijan 2006, unmet need by wealth quintile, married women 15-49 49

Figure 42. Chad 2004, unmet need by wealth quintile, married women 15-49 49

Figure 43. Niger 2006, unmet need by wealth quintile, married women 15-49 49

Figure 44. Madagascar 2008-09, unmet need for spacing and limiting by parity, married women 15-49 50

Figure 45. Colombia 2010, unmet need for spacing and limiting by parity, married women 15-49 50

Figure 46. Turkey 2003, unmet need for spacing and limiting by parity, married women 15-49 50

Figure 47. Bangladesh 2007, unmet need for spacing and limiting by parity, married women 15-49 50

Figure 48. Pakistan 2006-07, unmet need for spacing and limiting by parity, married women 15-49 50

Figure 49. Ghana 2008, unmet need for spacing and limiting by parity, married women 15-49 50

Figure 50. Haiti 2005-06, unmet need for spacing and limiting by age group, married women 15-49 51

Figure 51. Nepal 2006, unmet need for spacing and limiting by age group, married women 15-49 51

Figure 52. Samoa 2009, unmet need for spacing and limiting by age group, married women 15-49 51

Figure 53. Namibia 2006-07, unmet need for spacing and limiting by age group, married women 15-49 51

Figure 54. Contraceptive prevalence and unmet need for spacing and limiting, sexually active unmarried women 15-49 53

Figure 55. Correlation of current contraceptive use for limiting and total fertility rates 56

Figure 56. Current and estimated total fertility rates with all unmet need for limiting satisfied (Original and Revised definitions of unmet need) 58

Preface

One of the most significant contributions of the MEASURE DHS program is the creation of an internationally comparable body of data on the demographic and health characteristics of populations in developing countries.

The *DHS Comparative Reports* series examines these data across countries in a comparative framework. The *DHS Analytical Studies* series focuses on analysis of specific topics. The principal objectives of both series are to provide information for policy formulation at the international level and to examine individual country results in an international context.

While *Comparative Reports* are primarily descriptive, *Analytical Studies* comprise in-depth, focused studies on a variety of substantive topics. The studies are based on a variable number of data sets, depending on the topic being examined. A range of methodologies is used in these studies including multivariate statistical techniques.

The topics covered in *Analytical Studies* are selected by MEASURE DHS staff in conjunction with the U.S. Agency for International Development.

It is anticipated that the *DHS Analytical Studies* will enhance the understanding of analysts and policymakers regarding significant issues in the fields of international population and health.

Ann Way
Project Director

Executive Summary

Unmet need for family planning, defined on the basis of survey data to measure the percentage of women who do not want to become pregnant but are not using contraception, is a valuable concept for family planning programs and policies. Continued refinements in the definition of unmet need, however, have made its calculation extraordinarily complex, now incorporating data from 15 separate survey questions, as well as use of the contraceptive calendar. Not all of these questions have been consistently included in every survey. As a result:

- Unmet need has not been calculated consistently.
- Unmet need is not calculated the same way in all Demographic and Health Surveys (DHS) and in other international survey programs, including the Reproductive Health Surveys (RHS) and the Multiple Indicator Cluster Surveys (MICS).
- Levels of unmet need are not comparable across countries or over time.

Unmet need has received an unprecedented level of scrutiny since it became a Millennium Development Goal indicator in 2008. Now more than ever, ensuring that the indicator can be calculated in a consistent way has become crucial.

This report presents a new standard definition of unmet need that can be consistently applied over time and across countries, and shows the impact of the revising the definition on estimated levels of unmet need. The analyses use data from 169 DHS conducted in 70 countries over the last 20 years.

To achieve this standard definition, the authors first proposed a series of modifications to the original definition of unmet need to address the inconsistencies and complexity of the indicator. To review these proposed changes, MEASURE DHS convened a Technical Expert Working Group (TEWG), whose members have a wealth of experience in research on unmet need: technical experts John Bongaarts, John Casterline, Amy Tsui, and Charles Westoff; USAID participants Jacob Adetunji, Yoonjoung Choi, and Scott Radloff; UNFPA participants Stan Bernstein and Edilberto Loaiza; and MEASURE DHS participants Sunita Kishor, Shea Rutstein, and Ann Way. The TEWG felt that, while the basic elements of the definition of unmet need should remain unchanged, some changes were needed to promote standardization across surveys and to enable valid comparisons of unmet need.

The TEWG agreed on six changes that allow unmet need to be calculated in a consistent way, over time and across surveys:

1. Exclude inconsistently collected data.
    - Remove calendar data from the calculation.
    - Remove data based on “happy” and “problem” survey questions.
2. Do not assume an unmet need status for women missing key data.
3. Simplify classification of unmet need for spacing versus unmet need for limiting.
4. Shorten the duration for which women are considered to be postpartum amenorrheic.
    - Women can be considered postpartum amenorrheic for only two years (previously any woman whose period had not resumed since her last birth was considered postpartum amenorrheic for up to five years).
5. Standardize the calculation of infecundity.
    - Harmonize the algorithm for calculating infecundity with MICS and DHS surveys by adding a question on ever-use of contraception to the MICS questionnaire.
    - Restrict the use of the infecundity condition, “Women who were first married five or more years ago, never used contraception, and have not had a birth in past five years = infecund,” to currently married women only.

- Use data on hysterectomy and menopause from the survey question on reasons not currently using a method rather than from a question on reasons for not intending to use a method in the future, since the latter question has been removed from the DHS VI questionnaire.

6. Explicitly handle inconsistencies (e.g., women reporting in one part of the survey questionnaire that her last period was before her last birth, but never had a birth).

The recommended change with the largest impact on estimated levels of unmet need relates to use of data from the contraceptive calendar in the unmet need algorithm. Some DHS surveys, mostly in countries with higher contraceptive prevalence, included a calendar with a month-by-month retrospective history of all births, pregnancies, terminations, and episodes of contraceptive use in the five years prior to the interview. Other surveys did not include such information. The **Original** calculation of unmet need incorporates data from the calendar in countries where a calendar was used, but follows a different algorithm where a calendar was not used. This practice has resulted in an inconsistent calculation of unmet need across countries, and sometimes for different surveys within the same country. According to the **Revised** definition proposed by the authors and agreed upon by the TEWG, calendar data are excluded from the calculation of unmet need.

In terms of the impact of the revised definition on estimated levels of unmet need, countries fit into three categories: (1) countries in which calendar data were collected in every survey, (2) countries in which calendar data were collected in none of the surveys, and (3) countries in which calendar data were collected in some, but not all surveys.

1. In countries that included the calendar in every survey, the overall level of unmet need using the **Revised** definition is consistently higher than the **Original** definition in every survey, but the trend over time remains the same.
2. In countries that never collected calendar data, the change between the **Original** estimates and the **Revised** estimates of unmet need is negligible.
3. In countries that included the calendar in some surveys but not all, results are mixed. In some countries in this group, applying the **Revised** definition of unmet need across all surveys reveals a different trend than the **Original** definition, which changed over time. The **Revised** indicator more accurately reflects the actual trends in unmet need.

In sum, the **Revised** definition of unmet need for family planning produces similar, although slightly higher, levels of unmet need compared with the **Original** definition. In contrast to the **Original** definition, the **Revised** definition can be applied consistently to compare estimates across countries and to reliably measure trends over time.

Introduction

Unmet need for family planning is a valuable concept that is widely used for advocacy, the development of family planning policies, and the implementation and monitoring of family planning programs worldwide. Unmet need is defined on the basis of survey data as the percentage of women who are not currently using a method of contraception and want to stop or delay childbearing. Despite seeming straightforward, the definition of unmet need[1] requires data from 15 separate survey questions, plus the contraceptive calendar, and includes detailed algorithms to calculate postpartum amenorrhea and infecundity. Additionally, the definition of unmet need has changed over time and has been applied inconsistently across surveys. These changes have made comparison across countries and interpretation of trends difficult and potentially misleading.

Unmet need has received an unprecedented level of scrutiny since it became a Millennium Development Goal (MDG) indicator (indicator 5.6) in 2008. Now more than ever, ensuring that the indicator can be calculated in a consistent way has become crucial. To address this need, the USAID-funded MEASURE Demographic and Health Surveys (DHS) program convened a Technical Expert Working Group (TEWG) on unmet need to review suggestions for the revision of the unmet need indicator. After a series of consultations and revisions, all members of the TEWG agreed upon a revised definition of unmet need for family planning, which this report presents.

In the first section, we review the history of unmet need and explain how it reached its current level of complexity. We describe the complete definition of unmet need as implemented in the DHS, including variations over time and between surveys that used different versions of the DHS questionnaire. In the second section, we describe the rationale, process, and result of revising the definition of unmet need. The third section demonstrates the impact of revising the definition of unmet need for currently married women. In the fourth section, we use the **Revised** definition to show estimates of demand for family planning and proportion of demand satisfied, differentials in unmet need by background characteristics, and estimates of unmet need for sexually active unmarried women. In the fifth section, we estimate the potential impact that fulfilling all unmet need could have on fertility rates, comparing the **Original** and **Revised** definitions. The analyses use data from 169 DHS surveys from 70 countries conducted over the last 20 years.

[1] Refers to the unmet need algorithm used to compute estimates of unmet need that are shown in DHS final reports, STATcompiler, and included in the MDG database. This definition has varied over time.

1

The Original Definition of Unmet Need for Family Planning

In this section, we first describe the history of unmet need and the many refinements that brought the indicator to its current level of complexity. We then lay out the definition of unmet need prior to revision, setting the stage for the revisions explained in Section 2.

1.1 Changes over Time in the Definition of Unmet Need for Family Planning

The definition of unmet need for family planning has been under continuous revision and development since the 1960s, when researchers first observed that surveys of contraceptive knowledge, attitudes, and practices (KAP) showed a gap between some women's reproductive intentions and their contraceptive behavior (Robey, Ross, and Bhushan 1996), which became known as the "KAP-gap" (Bogue 1974, Bongaarts 1991). The term "unmet need" was coined in the late 1970s to describe the seemingly discrepant behavior of women who want to avoid pregnancy but are not using contraception.

Early measurement of unmet need employed a basic definition based on data available at the time. At first, unmet need was defined as the percentage of currently married women who want no more children but are not using contraception (the numerator), out of all currently married women (the denominator). In 1978, using data from the World Fertility Surveys (WFS), Westoff published the first comparative estimates of unmet need for family planning to limit births. The WFS questionnaire did not ask women about their desire to space births (Westoff 1978). In 1981, Westoff and Pebley, using WFS data from 18 countries, showed that different definitions of unmet need produced widely differing estimates. Also, they recommended that the unmet need concept be extended to cover desire to space births, as soon as the data could be collected (Westoff and Pebley 1981).

1.1.1 Spacing and Limiting

In 1982 Nortman introduced an expanded calculation of unmet need based on data from the Contraceptive Prevalence Surveys (CPS), which included data on women's preferences for timing births, as well as for limiting. In addition to women who did not want to have any more children, women who wanted to delay a pregnancy, or who were unsure if or when they wanted to become pregnant, were added to the definition of unmet need. These women were considered to have "unmet need for spacing births," while women who did not want more children were considered to have "unmet need for limiting births" (Nortman 1982).

1.1.2 Infecundity

Attempts to identify women who were not at risk of becoming pregnant and exclude them from the calculation of unmet need introduced a new level of complexity. A primary reason for this exclusion was the goal of estimating the effect on fertility levels if all unmet need were converted to contraceptive use. To do so, analysts needed to exclude women for whom contraceptive use would have no demographic impact: that is, women who could not give birth, or were infecund. Infecund women were considered to have no need for contraception and so were removed from the numerator of the unmet need calculation.

Determining women's infecundity based on survey data proved to be complicated. In 1988, Westoff published revised estimates of unmet need that considered women to be infecund either if they had no birth in the last five years despite having been married for longer than five years and never having used contraception, or if they had not menstruated in the last six weeks but were not pregnant or amenorrheic (Westoff 1988). The cutoff date for last menstruation used in determining infecundity was later expanded to the last 12 weeks (Westoff and Ochoa 1991), and then six months (Westoff and Bankole 1996). The definition of infecundity was later refined to include women who reported that they were menopausal or who, when asked if they wanted a/another child, said they could not get pregnant (Westoff and Bankole 1995). Additionally, although we could not find this documented in a research paper, examination of the code used by the DHS to calculate unmet need shows that, in approximately 1990, women who had a hysterectomy or said they had never menstruated but were not postpartum amenorrheic were added to the infecund category.

1.1.3 Pregnancy and Postpartum Amenorrhea

Pregnant and postpartum amenorrheic women have been treated differently in different definitions of unmet need. Initial estimates treated these women as having no need for contraception because they are currently not at risk of becoming pregnant (Westoff 1978). This approach was criticized because these women may soon be in need of contraception, even if they were not at risk of pregnancy at the moment of the survey. Nortman (1982) recommended treating women who were pregnant or breastfeeding (used as a proxy for postpartum insusceptibility) as potentially having an unmet need because they would return to being at risk of pregnancy within a year if they did not use contraception. Westoff and Ochoa (1991) argued that many pregnant and postpartum amenorrheic women might be in that state at the time of survey because they were not using contraception but did not want to become pregnant—that is, they had a prior need for family planning that was not met. They suggested that women who are pregnant or postpartum amenorrheic be assigned an unmet need status based on the retrospective wantedness of their current pregnancy or last birth. If a woman reported that she had wanted to become pregnant when she did, then she had no need for contraception; if she had wanted to become pregnant later, then she had an unmet need for spacing births; if she had not wanted to become pregnant at all, then she had an unmet need for limiting births. Despite critiques of using retrospective fertility intentions as a measure of unmet need status for pregnant and postpartum amenorrheic women (e.g., Ross and Winfrey 2001), this approach has been used by the DHS since approximately 1990.

Around 1995, an adjustment to the treatment of some pregnant and postpartum amenorrheic women was incorporated. If pregnant or postpartum amenorrheic women said they had not wanted their current pregnancy/last birth at all, but also reported wanting another child in the future, they were shifted from having an unmet need for limiting to having an unmet need for spacing (Westoff and Bankole 1995). This adjustment affected levels of unmet need for spacing and for limiting but did not affect the total level of unmet need.

In addition to changes in *how* postpartum amenorrheic women are treated in the unmet need algorithm, the determination of *who* is considered postpartum amenorrheic has also changed over time. To determine whether or not a woman is postpartum amenorrheic, the DHS has consistently used the question from the maternity history *"Has your period returned since the birth of (NAME OF YOUNGEST CHILD)?"* However, the group of women who are asked this question has changed. In surveys from DHS rounds II, IV, and V,[2] this question was asked of all women who gave birth in the five years prior to the survey. In DHS III, it was asked only of women who gave birth in the prior three years. The algorithm for

[2] The DHS project is currently in its 6th round of data collection. The previous survey rounds were approximately DHS I (1984-89), DHS II (1989-93), DHS III (1993-97), DHS IV (1997-2003), and DHS V (2003-2008).

determining whether a woman was postpartum amenorrheic does not limit the duration of postpartum amenorrhea. The maximum duration of postpartum amenorrhea is therefore different in different surveys: 35 months in surveys with three-year maternity histories, and 59 months in surveys with five-year maternity histories.

1.1.4 Calendar Data

Use of a contraceptive calendar in the DHS questionnaires has also affected the definition of unmet need. The contraceptive calendar is a month-by-month retrospective history of births, pregnancies, terminations, and episodes of contraceptive use that each surveyed woman experienced in the five years prior to being interviewed. In addition, the full calendar includes columns to collect information on reasons for discontinuation of each contraceptive method, and on marital status in each month (see DHS IV Model A questionnaire, ORC Macro 2001). During DHS rounds II, III and IV, the DHS core questionnaire was split into two core questionnaires: the Model A questionnaire for high contraceptive prevalence countries and the Model B questionnaire for low contraceptive prevalence countries. The full contraceptive calendar was included in the Model A questionnaires in DHS rounds II, III and IV, from approximately 1990 to 2003, while Model B questionnaires did not include the calendar. In DHS V, the standard questionnaire for all countries included a one-column calendar covering only births and contraceptive use.[3] It should be noted that the calendar was simplified as part of an effort to reduce the length and the complexity of the entire survey instrument, and not with the explicit intention of revising the unmet need definition.

When the full calendar was implemented in a survey, the unmet need algorithm incorporated data from the calendar in two ways. First, if the marital status column (column 4 in the DHS IV Model A questionnaire, ORC Macro 2001) was included, these data were used in the estimation of infecundity. Second, if the reasons for discontinuation column (column 3 in the DHS IV Model A questionnaire, ORC Macro 2001) was included, the data were used to estimate contraceptive failure.

Data from the Marital Status Column of the Calendar

Different definitions of infecundity were used depending on whether or not the survey included column 4 of the calendar on marital status. As described above, several criteria were used to determine whether a woman was able to bear children. In surveys with the marital status column, one criterion was that if women had been continuously married for the last five years, had not used contraception in the last five years, and had no births in the last five years, they were considered to be infecund. In surveys without calendar data, this criterion was adjusted to consider women to be infecund if they had no births in the last five years, were first married more than five years before the survey, and had never used contraception. Infecund women (by either definition) were removed from the numerator of the unmet need calculation.

Data from the Reasons for Discontinuation Column of the Calendar

In surveys that collected column 3, the reasons for contraceptive discontinuation column in the calendar, the unmet need algorithm was altered to incorporate information on contraceptive failure for women who are pregnant or postpartum amenorrheic at the time of the survey. If information from the calendar indicates they are pregnant or postpartum amenorrheic as a result of contraceptive failure, they are considered not to have an unmet need because they were using contraception when they became

[3] Some countries (e.g., Egypt, Indonesia) that are interested in contraceptive use dynamics have continued to implement the full calendar.

pregnant[4] (Westoff and Ochoa 1991). Treating women who are pregnant or postpartum amenorrheic as a result of contraceptive failure as not in need of family planning decreases the level of unmet need.

In contrast, in surveys that did not include column 3 of the calendar, it is not possible to determine whether a pregnancy resulted from contraceptive failure, so all pregnant or postpartum amenorrheic women are assigned an unmet need status based on the retrospective wantedness of their current pregnancy/last birth. As a result, in surveys without column 3, more women are counted as having an unmet need because women cannot be counted as experiencing contraceptive failure. Thus, when unmet need estimates are calculated using calendar data, they are consistently lower than estimates calculated without using calendar data. The magnitude of the difference depends on the contraceptive prevalence rate, method mix, and failure rates in the country at the time of survey.

Inclusion of the Calendar

Even within countries with either high or low contraceptive prevalence, the inclusion or exclusion of the calendar has been inconsistent, partly due to changes in the core questionnaire and partly due to requests of the individual countries. Bangladesh, Bolivia, Cambodia, Ethiopia, India, Kenya, Malawi, the Philippines, Tanzania, and several other countries all included a calendar in some of their DHS surveys, but not in others. Even within surveys that included the calendar, implementation has been inconsistent: surveys included some parts of the calendar but not others. Some surveys (e.g., Azerbaijan 2006; Colombia 2010; Jordan 2007 and 2009; Ukraine 2007) included the reasons for discontinuation column but not the marital status column, and therefore calendar data were used to determine contraceptive failure but not infecundity.

1.1.5 Other Questionnaire Changes

Several other changes to the definition of unmet need for family planning have been incorporated due to changes in survey questions. From approximately 1993 to 1997 (DHS II), the DHS core questionnaire included the question *"If you became pregnant in the next few weeks, would you be happy, unhappy, or would it not matter very much?"* (Macro International 1995). This question was used to determine the unmet need status of fecund women who were not using contraception and said they were unsure if or when they wanted a/another child. If a woman who fit these criteria said she would be happy if she became pregnant in the next few weeks, she was classified as having no unmet need; if she said she would be unhappy or that it would not matter, she was classified as having an unmet need for spacing births (Macro International 1996).

In DHS IV, this question about happiness with pregnancy was removed from the core questionnaire and replaced with a similar question about pregnancy as a problem: *"In the next few weeks, if you discovered that you were pregnant, would that be a big problem, a small problem, or no problem for you?"* (ORC Macro 2001). This question was also used to determine the unmet need status of fecund women who were not using contraception and said they were unsure if or when they wanted a/another child. If a woman said it would be "no problem" if she became pregnant, she was treated as having no need for contraception; if she gave any other response, she was treated as having an unmet need for spacing births (ORC Macro 2005). More recent DHS surveys have not included either question. Subsequently, all fecund women who are not using contraception and are undecided when or if they want (more) children are treated as having an unmet need for spacing births (ICF Macro 2010).

---

[4] Some consider these women to have a need for more effective contraception; this concept, however, has never been incorporated in the DHS definition of unmet need.

1.2 Examining the Original Definition

To help readers understand the new changes made to the definition of unmet need (see "The Revised definition of unmet need," Section 2) we first explain the complete definition of unmet need prior to revision as it has been implemented since approximately 2003. We refer to this as the **Original** definition of unmet need. The unmet need algorithm essentially acts as a large flowchart (Figure 1), using data from 15 questions in different sections of the DHS questionnaire and information from the calendar to classify every woman into one of the following 10 categories: using contraception to space, using to limit, unmet need to space, unmet need to limit, spacing failure, limiting failure, desires a birth within two years, never had sex, no sex/want to wait, or infecund. The following text explains how women are classified into one of these 10 mutually exclusive categories, using the **Original** definition as applied to currently married women, including calendar data. At the end of this section, we describe the variations of the definition of unmet need used for all women and sexually active unmarried women, as well as changes when calendar data were not collected.

1.2.1 Women Using Contraception (Group 1)

The first selection in the unmet need algorithm is whether or not a woman is currently using contraception. All women currently using any contraceptive method are considered to have a met need for family planning.

Women who are currently using contraception (Group 1 in Figure 1) are then disaggregated into "using for spacing" (to delay a/another birth) and "using for limiting" (to avoid having any [more] births). If women report that they are sterilized, or say they want no more children or can't get pregnant (but are using contraception), they are classified as **using to limit.** If they say they want a/another child soon, later, or are undecided about the timing of a/another child, or they are undecided whether they want a/another child, they are classified as **using to space**. Women who are using contraception but whose response on their desire for more children is missing are also classified as **using to space**.

1.2.2 Women Who are Pregnant or Postpartum Amenorrheic (Groups 2a & 2b)

The next selection in the algorithm is to determine whether women are pregnant or postpartum amenorrheic. If they report that they are currently pregnant or that their monthly period has not returned since the birth of their last child, they are treated as pregnant/postpartum amenorrheic. Once women have been categorized as pregnant or postpartum amenorrheic, calendar data are checked to see if they reported that their current pregnancy or last birth was the result of contraceptive failure. If they are pregnant or postpartum amenorrheic due to failure, they fall into Group 2a; otherwise, they fall into Group 2b. Women who are not identified as pregnant or postpartum amenorrheic are then put through an algorithm to check whether they should be considered infecund (Group 3) or fecund (Group 4).

Women Who are Pregnant or Postpartum Amenorrheic as a Result of Contraceptive Failure (Group 2a)

Women who are pregnant or postpartum amenorrheic due to contraceptive failure (Group 2a) are classified into limiting or spacing failures based on the wantedness of their current pregnancy (for pregnant women) or last birth (for postpartum amenorrheic women). They are not considered to have an unmet need (regardless of the wantedness of their pregnancy) because they were using contraception at the time they became pregnant:

a. Women who report that their current pregnancy/last birth was wanted *at that time* or *later* are classified as having a **spacing failure.**
b. Women who report that their current pregnancy/last birth was *not wanted at all* are put through an additional algorithm, based on their desire for another child in the future.
    - Women who want another child in the future are categorized as having a **spacing failure.**
    - Women who want no more children or are undecided are categorized as having a **limiting failure**.
    - Women for whom the data on wantedness of future children are missing are categorized as having a **limiting failure**.
c. Women for whom the data on the wantedness of their current pregnancy/last birth is missing are categorized as having a **spacing failure.**

Women Who are Pregnant or Postpartum Amenorrheic, Not Classified as Due to Contraceptive Failure (Group 2b)

Women who are pregnant or postpartum amenorrheic, but not as the result of contraceptive failure, fall into Group 2b. In surveys without calendar data that included reasons for discontinuation, *all* pregnant or postpartum amenorrheic women are included in this group, because data are not available to determine whether their pregnancy resulted from contraceptive failure. Their responses to whether they wanted their current pregnancy/last birth at that time, later, or not at all are analyzed. Based on those responses, they are put into one of the following categories:
a. Women who report that their current pregnancy/last birth was wanted *at that time* are categorized as having no unmet need (coded as **desiring a birth within two years**).
b. Women who report they had wanted their current pregnancy/last birth *later* are categorized as having an **unmet need for spacing**.
c. Women who report that their current pregnancy/last birth was *not wanted at all* are put through an additional algorithm based on their desire for another child in the future.
    - Women who want another child in the future are categorized as having an **unmet need for spacing.**
    - Women who want no more children or are undecided are categorized as having an **unmet need for limiting**.
    - Women for whom the data on wantedness of future children are missing are categorized as having an **unmet need for limiting**.
d. Women for whom the data on the wantedness of their current pregnancy/last birth is missing are categorized as having an **unmet need for spacing.**

1.2.3 Women Who are Not Pregnant and Not Postpartum Amenorrheic (Groups 3 & 4)

Women who are not selected as pregnant or postpartum amenorrheic are then put through an algorithm to check whether they should be considered fecund (able to bear children) (Group 4) or infecund (Group 3).

Women Who are Not Pregnant and Not Postpartum Amenorrheic, Infecund (Group 3)

Women are classified as **infecund** by the algorithm and treated as not in need of contraception if they are neither pregnant nor postpartum amenorrheic and any of the following conditions apply:
a. Women who have been continuously married and not using contraception for the past five years (from calendar data—see below for surveys without calendar data on marital status) and have not had a birth in the past five years; or

b. Women who responded to the question about wantedness of future children by saying that they cannot get pregnant; or
c. Women who, when asked why they do not intend to use a contraceptive method in the future, responded that they are menopausal or hysterectomized; or
d. Women who answered the question about time since last menstrual period with any of the following responses:
    - Six months ago or longer (calculated from numeric response); or
    - Menopausal/had a hysterectomy; or
    - Never menstruated; or
    - Before last birth (and last birth was 5 or more years ago).

Women who do not meet any of the above conditions are categorized as fecund, and flow through these checks into Group 4.

Women Who are Not Pregnant and Not Postpartum Amenorrheic, Fecund (Group 4)

Women who are not pregnant or postpartum amenorrheic and who do not satisfy the criteria for infecundity are considered fecund, and thus at risk of pregnancy. These women are potentially in need of family planning, and their need for family planning is assessed on the basis of their desire to have children in the future:

a. Women who want a child within the next two years are categorized as **desiring a birth within two years** and treated as having no need for contraception.
b. Women who want no more children are categorized as having an **unmet need for limiting.**
c. Women who want a child after two years or more, who want a child but are undecided about timing, or who are undecided if they want a child are categorized as having an unmet need for spacing.
d. Women who are in this group but for whom data on their wantedness of future children are missing are coded as missing.

1.2.4 Alterations in Surveys without Calendar Data on Reasons for Discontinuation and/or Marital Status

In surveys that did not collect calendar data on reasons for discontinuation and/or marital status (see "Calendar data," above), the algorithm in Figure 1 is altered in two ways:

1. If the survey did not collect the reasons for discontinuation column, Group 2a is eliminated from the algorithm, and all pregnant or postpartum amenorrheic women are treated according to the Group 2b diagram.
2. If the survey did not collect the marital status column, condition (a) for checking fecundity status is removed, and replaced with the condition:
    a. Women who were first married five or more years ago, never used contraception, and did not have a birth in the past five years (are treated as **infecund**).

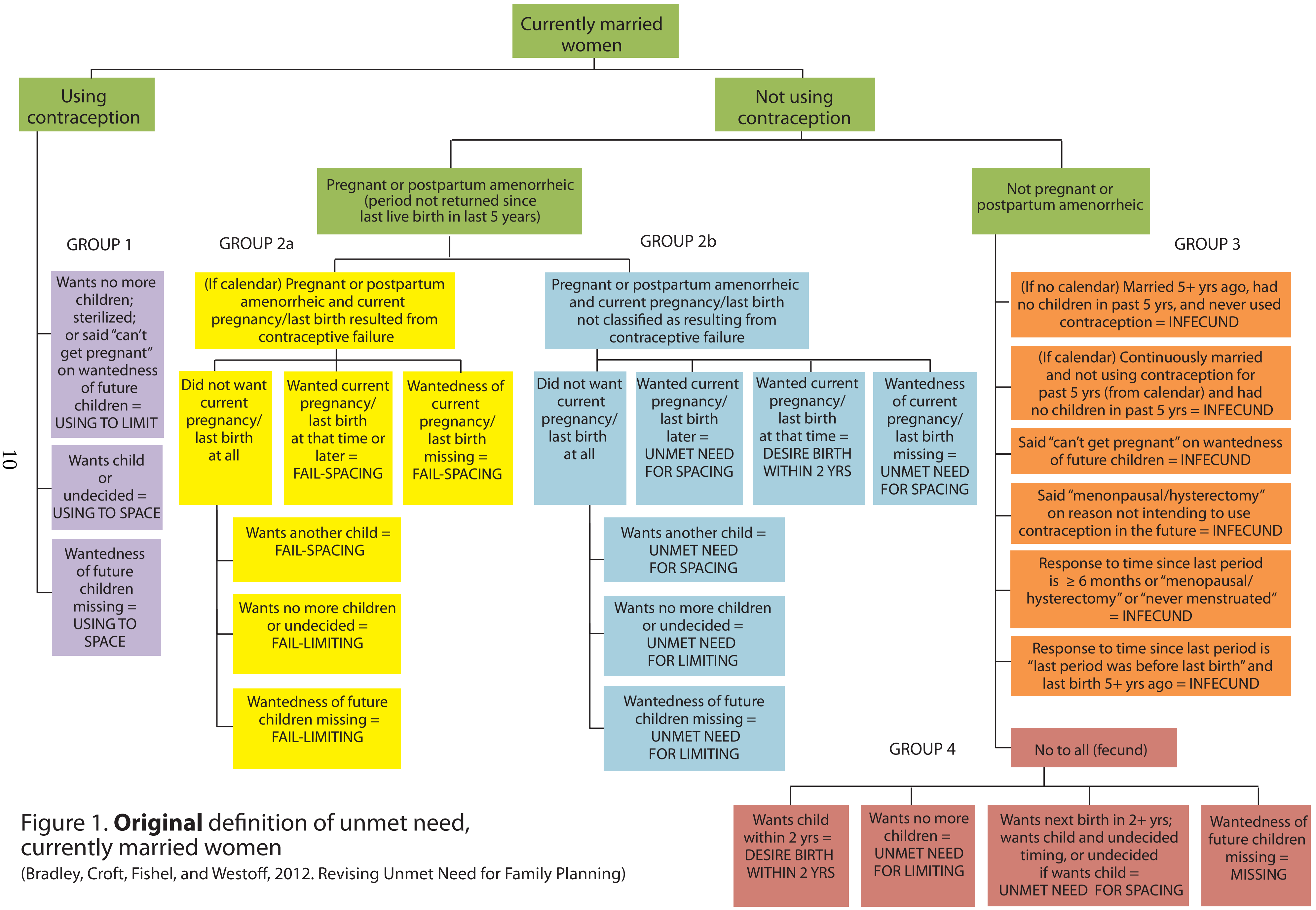

Figure 1. **Original** definition of unmet need, currently married women
(Bradley, Croft, Fishel, and Westoff, 2012. Revising Unmet Need for Family Planning)

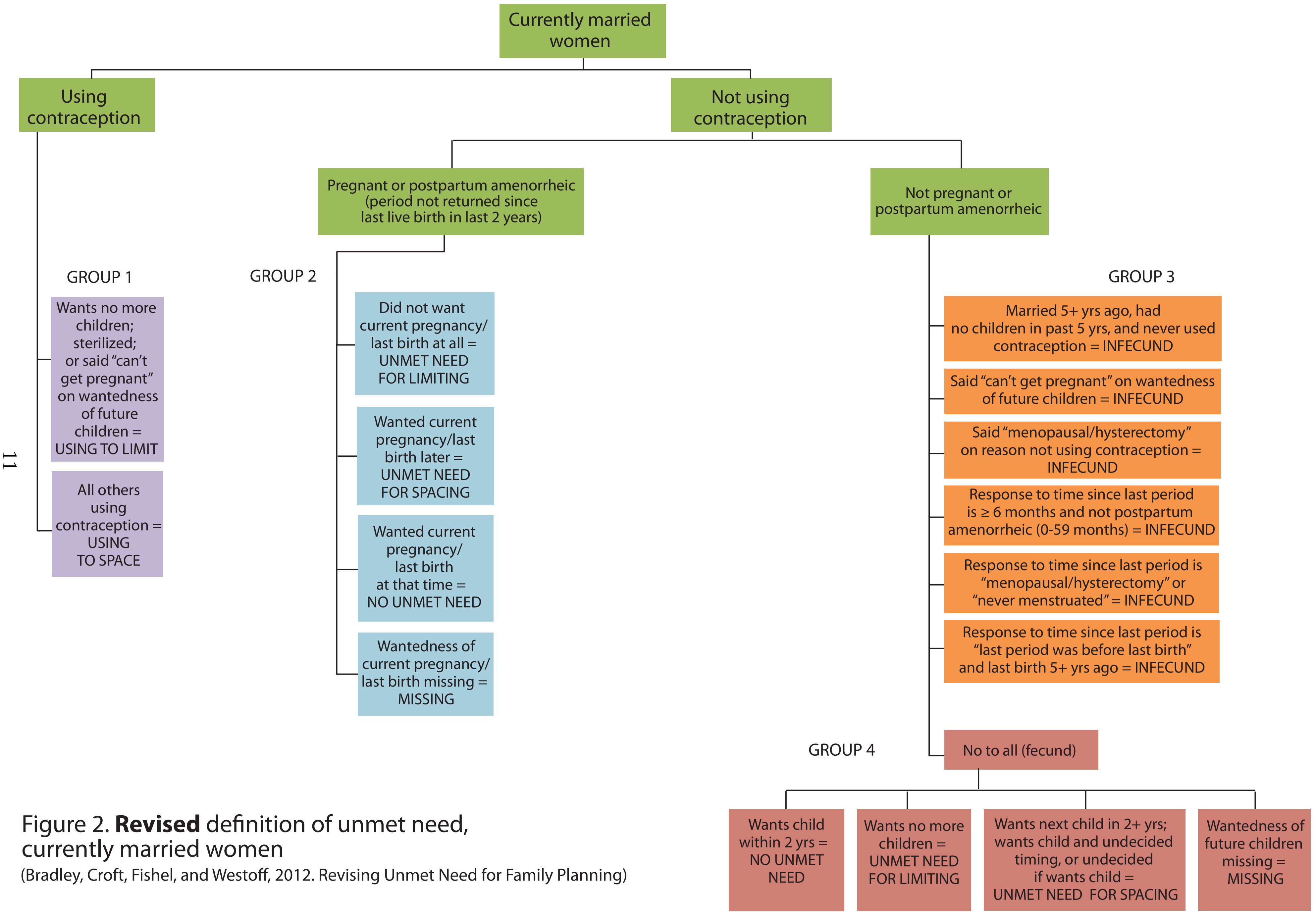

Figure 2. **Revised** definition of unmet need, currently married women
(Bradley, Croft, Fishel, and Westoff, 2012. Revising Unmet Need for Family Planning)

1.2.5 Application of Algorithm to All Women

The standard unmet need indicator is calculated with a denominator of currently married women[5] (UN 2007; UN 2008). When the unmet need algorithm is applied to all women, a few modifications are made to account for the fact that many unmarried women are not exposed to the risk of pregnancy. This is generally done by assuming that unmarried women who are not sexually active—that is, who report no sexual intercourse in the 30 days prior to the survey—are not exposed to the risk of pregnancy, and therefore have no need for family planning. All currently married women, regardless of their sexual activity, are assumed to be exposed to the risk of pregnancy.

As applied to Figure 1, including all women in the algorithm changes nothing for Group 1 (current contraceptive users) or Group 2a (women who are pregnant or postpartum amenorrheic as the result of contraceptive failure). The check for recent sexual activity of unmarried women is made in two places:

1. It acts as a filter for Group 2b (women who are pregnant or postpartum amenorrheic, not as a result of contraceptive failure). Women who are currently married or sexually active flow into Group 2b if they are pregnant or postpartum amenorrheic and were not captured in Group 2a; otherwise they flow into the series of checks for fecundity status for Groups 3 and 4. Women who are unmarried and not sexually active skip Group 2b (even if they are pregnant or postpartum amenorrheic for reasons other than failure) and go straight to Groups 3 and, if not categorized as infecund, Group 4.
2. At the end of the infecundity checks, women who are considered to be fecund and are either currently married or sexually active flow into Group 4 (fecund women). Women who are considered to be fecund but are unmarried and not sexually active do not flow into Group 4. Rather, they are then categorized according to whether they a) never had sex, or b) have had sex, but not in the last 30 days. In the DHS recode variable for unmet need (v626), the labels given to these groups are a) **never had sex**, or (rather inexplicably) b) **no sex, want to wait.**

The treatment of each of the above categories in the unmet need indicator is summarized below.

Category	Treatment in unmet need indicator
Using to space	Met need
Using to limit	Met need
Unmet need for spacing	Unmet need
Unmet need for limiting	Unmet need
Spacing failure	No unmet need
Limiting failure	No unmet need
Desiring a birth within 2 years	No unmet need
Infecund	No unmet need
Never had sex	No unmet need
No sex/want to wait	No unmet need

[5] As with all other DHS indicators, "currently married" here refers to women who are either formally married or living together with a man as though married.

2

The Revised Definition of Unmet Need for Family Planning

In the previous section, we outlined the changes that have been made over time to the definition of unmet need for family planning, and described the complete, complex definition that is currently used in DHS final reports and MDG monitoring. In this section, we present the rationale for revising the unmet need definition and describe the revision process.

2.1 Rationale for Revising the Definition of Unmet Need

Many of the changes to the definition of unmet need over the years resulted from a quite reasonable goal: to estimate in the most precise way possible the level of unmet need for family planning and the impact that fulfilling all demand for contraception would have on fertility rates. Using all available data to achieve this goal, even if the data available are not always the same from one survey to the next, makes sense when the objective is to produce estimates for a given country at a single time. However, the varying definitions of unmet need that resulted from pursuing this goal have produced estimates that are not comparable across all surveys, and therefore are not useful for tracking trends or comparing countries.

The changes in definition that have been incorporated over the years have altered the directionality of trends in unmet need in several countries. In the Philippines, for example, an apparent sharp increase in the level of unmet need between 2003 and 2008 is attributable to inclusion of a contraceptive calendar in the 1993-2003 surveys, and exclusion of the calendar in the 2008 survey. The apparent increase in unmet need in the Philippines has been remarked upon and analyzed (NSO and ICF Macro 2009). If levels of unmet need are re-calculated using a consistent definition that excludes calendar data, however, there is no increase; unmet need remained at the same level between 2003 and 2008.

In Bolivia, trends in unmet need show an inverted V-shape, with an increase between 1994 and 1998, and a steady decrease thereafter. The spike in 1998, however, is due to inclusion of calendar data in the definition of unmet need in 1994 but not in later surveys. When a consistent definition is applied, it becomes clear that unmet need has decreased steadily in Bolivia, and there is no V-shaped trend. Similar problems interpreting trends resulting from inconsistent inclusion of calendar data can be seen in several other countries as well, particularly Bangladesh, Colombia, the Dominican Republic, Jordan, Kenya, Malawi, Morocco, and Tanzania.

Many publications, and the MDG indicator database, treat unmet need as though the calculation has remained unchanged and assume that valid comparisons can be made over time and across countries. Clearly, however, variations in the definition of unmet need among surveys substantially alter reported levels of unmet need and change the direction of trends. Based on these findings, the authors proposed that a revised unmet need indicator was urgently needed to produce consistent estimates that are comparable across time and among countries.

2.2 The Process of Revising the Definition of Unmet Need

Particularly because the **Original** definition of unmet need is so widely used as an indicator, any revision to the definition must be undertaken with care. In July 2010, MEASURE DHS convened a Technical

Expert Working Group (TEWG) on unmet need for family planning to consider the details of a revision. Technical Experts included John Bongaarts, John Casterline, Amy Tsui, and Charles Westoff. USAID participants included Jacob Adetunji, Yoonjoung Choi, and Scott Radloff, and UNFPA was represented by Stan Bernstein and Edilberto Loaiza. In addition to the authors of this report, Ann Way and Sunita Kishor from MEASURE DHS participated in the discussions. Several others within MEASURE DHS, notably Shea Rutstein, also provided guidance.

A number of options for revising the definition of unmet need were considered. With the aim of reducing the complexity of the **Original** unmet need definition, the authors proposed a radical simplification using only current-status data, without consideration of pregnancy or postpartum amenorrhea. This proposal would have required only 4 questions instead of the 15 questions and calendar used by the **Original** algorithm (Bradley, Croft, and Fishel 2009). The proposed definition produced notably higher levels of unmet need than the **Original** definition and was therefore deemed unsuitable by the TEWG, although it did produce comparable trends. Other suggestions to achieve a more clearly current-status measure, such as using only women's self-reported exposure to the risk of pregnancy in place of the behavioral infecundity measure currently used, and treating pregnant women as having no need, were also rejected as too different from the **Original** definition. The TEWG came to an agreement that, while changes were needed to make the definition consistent for every survey, the conceptual underpinnings (such as measuring unmet need using retrospective data for pregnant or postpartum amenorrheic women and prospective data for others) should remain the same as in the **Original** definition.

In calculating a consistent definition, the authors and TEWG found several other problematic issues with the **Original** definition. For example, the **Original** definition treats women as postpartum amenorrheic for up to five years after their most recent birth, categorizes women with missing data as having an unmet need for spacing, and is extremely complex and difficult to understand and to calculate. The **Original** definition also cannot be replicated using data collected in Multiple Indicator Cluster Surveys (MICS), carried out by UNICEF. MICS uses a different algorithm than the DHS to estimate unmet need. As shown above, using different definitions can lead to invalid comparisons among countries and incorrect conclusions about trends. The MDG database also includes unmet need estimates from the Reproductive Health Surveys (RHS), implemented by the Centers for Disease Control and Prevention (CDC), and the surveys of the Pan Arab Project for Family Health (PAPFAM), funded by the Arab League; each of these survey programs uses a different definition of unmet need.

To address these concerns, the authors investigated changes to the **Original** definition of unmet need that would enable the definition to be consistently applied to all DHS surveys, would be simpler to understand and implement than the **Original** definition, and could be calculated using data from MICS and other survey programs.

2.3 Revisions to the Definition of Unmet Need

Based on discussion and examination of the impact on 160 DHS surveys of each change to the definition of unmet need for family planning, the TEWG agreed on six changes that allow unmet need to be calculated in a consistent way, over time and across surveys:

1. Exclude inconsistently collected data.
    - Remove calendar data from the calculation.
    - Remove data based on "happy" and "problem" survey questions.
2. Do not assume an unmet need status for women missing key data.
3. Simplify classification of unmet need for spacing versus unmet need for limiting.
4. Shorten the duration for which women are considered to be postpartum amenorrheic.

- Women can be considered postpartum amenorrheic for only two years (previously any woman whose period had not resumed since her last birth was considered postpartum amenorrheic for up to five years).

5. Standardize the calculation of infecundity.
   - Harmonize the algorithm for calculating infecundity with MICS and DHS surveys by adding a question on ever-use of contraception to the MICS questionnaire.
   - Restrict the use of the infecundity condition, "Women who were first married five or more years ago, never used contraception, and not had a birth in past five years = infecund," to currently married women only.
   - Use data on hysterectomy and menopause from the survey question on reasons not currently using a method rather than from a question on reasons for not intending to use a method in the future, since the latter question has been removed from the DHS VI questionnaire.
6. Explicitly handle inconsistencies (e.g., women reporting in one part of the survey questionnaire that her last period was before her last birth, but never had a birth).

Each change is described below, followed by a box highlighting how the new **Revised** definition differs from the **Original** definition described above. The flowchart of the **Revised** unmet definition is shown in Figure 2.

1. Exclude inconsistently collected data: Calendar data will no longer be used in the calculation of unmet need, either to determine if a woman's current pregnancy or last live birth was due to contraceptive failure, or to determine her fecundity status. Removing calendar data removes the entire Group 2a from the **Revised** definition (see Figure 2). The "happy" question and "problem" question will also be excluded from the algorithm. Only information that has been collected in all DHS surveys since 1990 will be used to calculate unmet need.

Original	Revised
Calendar data, where available, are used to determine if current pregnancy or last live birth was due to contraceptive failure. If yes, these women are NOT considered to have an unmet need, and instead are categorized as having a **spacing failure** or **limiting failure**.	Calendar data not used.<br>• Current pregnancy/last birth wanted later = **unmet need for spacing** (even if pregnancy/birth was due to contraceptive failure).<br>• Current pregnancy/last birth unwanted = **unmet need for limiting** (even if pregnancy/birth was due to contraceptive failure).
Calendar data, where available, are used to determine infecundity: if a woman has been continuously married and not using contraception for past 5 years and has not had a birth in the past 5 years, that woman is considered **infecund**.	Calendar data not used. Condition replaced by: if a woman was first married five or more years ago, never used contraception, and has not had a birth in the past 5 years, that woman is considered **infecund**.
Happy question "*If you became pregnant in the next few weeks, would you be happy, unhappy, or would it not matter very much?"* was used where available (mostly in surveys between 1994 and 1998) to further categorize fecund women who want a/another birth in 2+ years or are undecided if or when they want a/another birth. If women in this group said they would be "happy" if they become pregnant, they were categorized as having **no need**. Otherwise, they were categorized as having an **unmet need for spacing**.	Happy question not used. All fecund women who want a/another birth in 2+ years or are undecided if or when they want a/another birth are categorized as having an **unmet need for spacing**.
Problem question *"In the next few weeks, if you discovered that you were pregnant, would that be a big problem, a small problem, or no problem for you?"* was used where available (mostly in surveys between 1998 and 2003) to further categorize fecund women who want a/another birth in 2+ years or are undecided if or when they want a/another birth. If women in this group said it would be "no problem" if they become pregnant, they were categorized as having **no need**. Otherwise, they were categorized as having an **unmet need for spacing**.	Problem question not used. All fecund women who want a/another birth in 2+ years or are undecided if or when they want a/another birth are categorized as having an **unmet need for spacing**.

Impact of excluding inconsistently collected data
• In countries that collected complete calendar data, removing calendar data from the algorithm can increase estimated levels of unmet need in two ways:<br>1. Women who were previously in contraceptive failure categories can now be in an unmet need category.<br>2. A less restrictive version of the infecundity definition categorizes fewer women as infecund, allowing them to be categorized as having an unmet need.<br>• Removing the "happy" and "problem" questions can increase estimated levels of unmet need by putting fewer women into the "no need" category.<br>• Estimates of unmet need are comparable among surveys that did versus did not include full calendar data, the "happy" question, or the "problem" question.

2. Do not assume an unmet need status for women missing key data: In the **Original** definition of unmet need, if data are missing on key questions (because women did not respond to the question or due to interviewer error, or, in rare cases, because of data entry error), assumptions are made to give women with missing data an unmet need status. Pregnant or postpartum amenorrheic women whose response on the wantedness of their current pregnancy/last birth was missing were categorized as having an unmet need for spacing. Fecund women whose response on desire for future births was missing were also categorized as having an unmet need for spacing. The TEWG agreed to changes in how to treat missing data. If responses to the wantedness of the last birth (for postpartum amenorrheic women), wantedness of the current pregnancy (for pregnant women), or desire for a future birth (for fecund women) are missing, these women will be assigned a value of missing on the unmet need variable.

Original	Revised
Pregnant, missing on wantedness of current pregnancy = **unmet need for spacing.**	Pregnant, missing on wantedness of current pregnancy = **missing.**
Postpartum amenorrheic, missing on wantedness of last birth = **unmet need for spacing.**	Postpartum amenorrheic, missing on wantedness of last birth = **missing.**
Fecund, missing on desire for future birth **= unmet need for spacing.**	Fecund, missing on desire for future birth = **missing.**

Impact of not assuming an unmet need status for women missing key data
• Estimated levels of unmet need decrease slightly, because women who were categorized as having an unmet need now are categorized as missing.<br>• The impact is minimal in most surveys, because few women have missing data on these key questions.<br>• No assumptions are made about the unmet need status of women who did not answer key questions.

3. Simplify classification of unmet need for spacing versus unmet need for limiting: As described above, an addition to the handling of women who were a) pregnant or postpartum amenorrheic, and b) did

not want their current pregnancy/last birth at all, was introduced into the unmet need algorithm around 1995. Previously, all women who fit both these criteria were treated as having an unmet need for limiting. The 1995 modification used more information to classify these women: if their current pregnancy/last birth was unwanted, but in a separate question they say they want more children in the future, they were classified as having an unmet need for spacing rather than limiting. The TEWG agreed that this change was problematic, for two reasons. First, it had not been implemented consistently in the past. Second, it required both retrospective and prospective data from women who are currently pregnant or postpartum amenorrheic and say their current pregnancy/last birth was unwanted. All other women are given an unmet need status on the basis of either retrospective or prospective data, but not both. The TEWG decided this modification was inconsistent and unnecessarily complex, and removed the modification from the algorithm. This change has no effect on estimates of total unmet need, but shifts some women who were classified as having an unmet need for spacing in the **Original** algorithm to having an unmet need for limiting.

Original	Revised
Women who are pregnant/postpartum amenorrheic and said their current pregnancy/ last birth was not wanted at all = **unmet need for limiting,** unless they want another child in the future, in which case = **unmet need for spacing.**	Women who are pregnant/postpartum amenorrheic and said their current pregnancy/last birth was not wanted at all = **unmet need for limiting**, irrespective of whether they want another child in the future.
Women who are pregnant/postpartum amenorrheic and said their current pregnancy/last birth was not wanted at all and are undecided whether they want another child in the future = **unmet need for limiting.**	No change.

Impact of simplifying the classification of unmet need for spacing versus limiting
• No impact on estimates of total unmet need.<br>• In surveys since approximately 1995, some women are shifted from having an unmet need for spacing to having an unmet need for limiting.<br>• Estimates of unmet need for spacing and limiting are comparable over time.

4. Shorten the duration for which women are considered to be postpartum amenorrheic: As described above, women who are postpartum amenorrheic are assigned to an unmet need category based on the wantedness of their last birth in Group 2 (and in some cases also according to their desire for future children, see change #3 above), while women who are not postpartum amenorrheic are assigned to an unmet need category based on their fecundity and/or future fertility intentions in Groups 3 and 4. The **Original** algorithm allowed women to be considered postpartum amenorrheic for up to five years after their last birth (although this duration changed over the course of the survey program).

   The TEWG agreed that assigning a woman's current unmet need status should not be based on the wantedness of a birth that occurred up to five years ago, and that this duration needed to be shorter. Several analyses were undertaken considering shorter cutoffs for the duration of postpartum amenorrhea (i.e., 6, 12, 18, or 24 months). After much discussion, the TEWG agreed that women whose monthly period has not returned since their last birth should be considered postpartum

amenorrheic for up to 23 months (where month 0 is the month of birth) after that birth. In the **Revised** unmet need algorithm, women whose period has not returned since their last birth and whose last birth occurred two or more years ago are no longer considered to have postpartum amenorrhea. Instead of following the flowchart pattern for Group 2 (pregnant or postpartum amenorrheic women), women whose last period has not returned since their last birth, and their last birth was two or more years ago, will follow the flowchart pattern for Groups 3 and 4 (not pregnant or postpartum amenorrheic) to determine whether they should be categorized as infecund.

This change, however, caused a small problem in the algorithm for determining infecundity. In the **Original** definition, all women who were not postpartum amenorrheic and had not menstruated in the last six months were considered infecund, shown in the infecundity criterion "Response to time since last period is ≥ 6 months = infecund" (Group 3, Figure 1). With the shorter duration of postpartum amenorrhea in the **Revised** definition, women whose periods have not returned since the birth of their last child and who gave birth in the last 3-5 years would have been classified as infecund because they had not menstruated in the last six months. These women may still resume menstruation, so the TEWG felt that they should not be considered infecund. Following these changes, in the **Revised** definition, women with a birth in the last 3-5 years whose period had not yet returned since their last birth are considered fecund, unless one of the other fecundity checks in Group 3 identifies them as infecund. Women whose periods have resumed since the birth of their last child but who have not had a period in the past six months are considered infecund[6].

This decision revealed yet another complication, which is that the unmet need algorithm does not capture in all surveys information on whether or not a woman's menses had returned since her last birth in the past five years. The information used in the algorithm comes from the question *"Has your period returned since the birth of [NAME OF CHILD]?"* Although most DHS surveys do ask this question of all women with a birth in the past five years, some DHS surveys ask this question only of women who gave birth in the past three years, and in MICS surveys this question is asked only of women who had a birth in the past two years. For the women who were not asked or did not answer this direct question, the TEWG agreed to take information from another question available in both DHS and MICS *"When did your last menstrual period start?"* in combination with the time since last birth to determine whether or not a woman's menses had returned since her last birth.[7]

---

[6] The algorithm for Group 3 was changed to avoid categorizing women whose period has not returned after a birth in the last 3-5 years as infecund. The condition "Women whose last menstrual period was ≥ 6 months ago = infecund" is replaced by the condition "Women whose last menstrual period was ≥ 6 months ago AND are not postpartum amenorrheic (0-59 months) = infecund." The alteration of the condition can be seen by comparing Group 3 in Figure 1 and Figure 2.

[7] It should be noted that this difference in the reference period for which women are asked about the return of menses since their last birth also created an inconsistency in the **Original** definition. Take, for example, a woman who gave birth 4 years ago and whose period has not yet returned. In a survey with a 5-year reference period, she would have been considered postpartum amenorrheic, and her unmet need status would have been assigned based on the wantedness of her last birth. However, in a survey with a 3-year reference period, this woman would have been considered infecund, based on the criterion "last menstrual period was ≥ 6 months ago = infecund."

<table>
<tr><th>Original</th><th>Revised</th></tr>
<tr><td rowspan="2">Women whose menstrual period has not returned since their most recent birth, and who gave birth in the last 5, 4, or 3 years in DHS, or 2 years in MICS, are considered postpartum amenorrheic and assigned an unmet need status based on the wantedness of their last birth.</td><td>Women whose menstrual period has not returned since their most recent birth, and who gave birth in the last 2 years (0-23 months), are considered postpartum amenorrheic and assigned an unmet need status based on the wantedness of their last birth.</td></tr>
<tr><td>Women whose menstrual period has not returned since their most recent birth, and who gave birth in the last 3-5 years (24-59 months), are not considered postpartum amenorrheic and pass through the part of the unmet need algorithm to determine if they are fecund and whether they desire children in the future.</td></tr>
</table>

Impact of shortening the duration for which women are considered postpartum amenorrheic
• In most countries, this change has little impact because few women whose most recent birth was 2 or more years ago report that their period has not returned since the birth.<br>• In countries where many women report that their period has not returned since their most recent birth 2 or more years ago, unmet need estimates may change in either direction. Previously, unmet need status for women whose period did not return for 3-5 years after a birth was based on retrospective data (whether their last birth was wanted at that time, later, or not at all). Now, unmet need status for women who report that their period did not return for 3-5 years after a birth will be based on whether/when they want a birth in the future. Some of these women may instead be categorized as infecund if they report, for example, that they are menopausal or had a hysterectomy.

5. Standardize the calculation of infecundity: The process of determining which women are infecund was surprisingly complex. Due to differences in the survey questionnaires described in the preceding section, the ways in which women are classified as infecund are different in DHS and MICS surveys, and have changed over time. To make the calculation of infecundity as consistent as possible, several small changes were made.
   a. Most MICS4 surveys do not collect information on ever-use of contraception, data needed for the infecundity criterion “Women who were first married five or more years ago, never used contraception, and have not had a birth in the past five years = infecund” (Group 3). After consideration of alternatives (for example, deleting the criterion for DHS surveys), the TEWG agreed that the DHS would keep this condition and would request the MICS program to add the required question on ever-use of contraception to the MICS questionnaire. Some MICS4 surveys (e.g., Thailand) already have incorporated the question on ever-use of contraception, for the best comparability with DHS data.
   b. In the **Original** definition, the condition “Women who were first married five or more years ago, never used contraception, and have not had a birth in the past five years = infecund” applied in the same way to currently married women and formerly married women. If a woman is no longer married, however, having married (for the first time) at least five years ago but not having children in the past five years is not a good indicator of her current fecundity. In the **Revised** definition, this criterion is limited to currently married women.

c. The algorithm for determining which women are infecund includes the criterion “Women who responded that they were menopausal or hysterectomized when asked why they are not intending to use a contraceptive method *in the future* = infecund.” The question about why women are not intending to use a contraceptive method in the future is no longer included in the DHS questionnaire, as of DHS VI, part of an overall effort to streamline the survey instrument. To make the infecundity algorithm as consistent as possible in the future, this criterion will now be based on responses to why women are not *currently* using a contraceptive method. This question has been asked starting in DHS III. For DHS II surveys, only the question about intended future use was asked, and so the algorithm will be slightly different for DHS II surveys. Making the algorithm slightly different for DHS II surveys, and comparable for DHS III surveys onwards, was determined to be a better option than altering the algorithm between the most recent surveys (DHS V and DHS VI). For MICS surveys, information on menopause/hysterectomy will come from the question *“Why do you think you are not physically able to get pregnant?”*

Original	Revised
Infecundity criterion “Women who were first married five or more years ago, never used contraception, and have not had a birth in the past five years = infecund” is applicable only to DHS, not MICS, because MICS does not collect information on ever-use of contraception.	No change to DHS. For MICS, the infecundity criterion “Women who were first married five or more years ago, never used contraception, and have not had a birth in the past five years = infecund” can be applied when MICS collects information on ever-use of contraception.
Infecundity criterion “Women who were first married five or more years ago, never used contraception, and have not had a birth in the past five years = infecund” applied to all women, including those not currently married.	Infecundity criterion “Women who were first married five or more years ago, never used contraception, and have not had a birth in the past five years = infecund” applied to currently married women only.
Infecundity criterion “Women who responded that they were menopausal or hysterectomized when asked why they are not *intending to use a contraceptive method in the future* = infecund.” Question about reason not intending to use contraception in the future not asked in DHSVI.	Infecundity criterion “Women who responded that they were menopausal or hysterectomized when asked why they are not *currently using a method of contraception* = infecund.” Question about reason for not currently using contraception asked in DHS III-DHS VI.

Impact of standardizing the calculation of infecundity
• Depending on the reference period, duration of amenorrhea, level of contraceptive use, percentage of women who do not intend to use contraception in the future, and percentage of women who report menopause or hysterectomy, estimates of unmet need may change slightly in either direction.<br>• Estimates of unmet need are comparable over time within DHS III-DHS VI surveys.<br>• Comparability between MICS and DHS surveys is improved.

6. Explicitly handle inconsistencies: In analyzing 160 surveys, we came across several inconsistencies in the data. These may be due to women giving inconsistent responses, or possibly due to an error on the part of the interviewer or data entry staff. To ensure comparability, the TEWG agreed that inconsistencies should be explicitly handled within the unmet need algorithm so that they are treated the same way in all surveys. The TEWG agreed to the following changes:
   a. Women who said their last period was before their last birth but have never given birth: in the **Original** calculation, these women were treated as fecund unless classified as infecund elsewhere in the algorithm. In the **Revised** definition, these women are treated as infecund, assuming that the response "before last birth" was a recording error and should have been either "menopausal/hysterectomy" or "never menstruated"—response codes that are on either side of "before last birth" in the questionnaire.
   b. Women who said they never menstruated, but also reported that their period returned after their last birth: the **Original** algorithm treated all these women as infecund. All of the women who were asked if their period returned since their last birth had given birth in the last five years, and in many cases more recently, implying that they are fecund. For the **Revised** definition, the TEWG agreed to treat these women as fecund unless classified as infecund elsewhere in the algorithm.
   c. Women who reported never having menstruated, but had children: the **Original** definition treated these women as infecund because they never menstruated. But since they had children, all of them obviously were fecund at one time. In the **Revised** definition, the TEWG agreed to treat these women as fecund if they had given birth in the last five years (unless classified as infecund elsewhere in the algorithm), and to treat them as infecund if they had not given birth in the last five years (on the assumption that they are no longer menstruating).

Original	Revised
Last period was before their last birth, but have never given birth = **fecund**.	Last period was before their last birth, but have never given birth = **infecund**.
Never menstruated, but their period returned after their last birth and gave birth in the last 5 years = **infecund**.	Never menstruated, but their period returned after their last birth and gave birth in the last 5 years = **fecund**.
Never menstruated, but had children = **infecund**.	Never menstruated, but gave birth in the last 5 years = **fecund**.
	Never menstruated, no birth in the last 5 years = **infecund**.

Impact of explicitly handling inconsistencies
• Because there are relatively few inconsistencies in the majority of surveys, estimates of unmet need are only slightly affected by these changes.
• Explicitly handling missing and inconsistent data in the unmet need algorithm will help ensure that the **Revised** definition of unmet need can be applied consistently to all DHS surveys, as well as MICS and other surveys.

All of the changes described above have been implemented in the **Revised** definition of unmet need for family planning. For reference, all of the questions used to define unmet need are shown in Appendix A.

3

The Impact of Revising Unmet Need

This section examines the impact of implementing all of the changes to the definition of unmet need that were approved by the TEWG, as described above. We compare the **Original** and **Revised** definitions of unmet need for family planning:

- The **Original** definition, shown in Figure 1, was calculated using the definition applied when the survey was implemented. This definition includes the "happy" and "problem" questions and calendar data, if collected, and corrects errors,[8] if found, in the original calculation.
- The **Revised** definition, shown in Figure 2, was calculated using the consistent definition, including all of the changes described above.

3.1 Changes in Total Unmet Need

As Table 1 shows, the impact of incorporating all of the changes above to the definition of unmet need increases the total level of unmet need among currently married women age 15-49 from an unweighted average[9] across 169[10] surveys of 21.4 percent, using the **Original** definition, to 23.1 percent, using the **Revised** definition. The average change per survey is 1.7 percentage points, with a range from -1.3 to 6.3 percentage points across all 169 surveys (see Table 2).

The majority of this change is due to the removal of calendar data. The impact of the removal of calendar data can be seen by comparing the impact of changes on calendar surveys versus non-calendar surveys. In surveys that collected calendar data (including the reasons for discontinuation column, the marital status column, or both), implementing all of the changes increases unmet need by an average of 3.3 percentage points, from 13.9 to 17.3 percent. In comparison, in non-calendar surveys, moving from the **Original** to the **Revised** definition increases total unmet need by only 0.7 percentage points, from 26.0 to 26.7 percent.

Primarily because the calendar was implemented in countries with high contraceptive prevalence, the greatest differences in levels of unmet need between the **Original** and **Revised** definitions are in countries with relatively high contraceptive prevalence rates (CPR). Implementing all changes approved by the TEWG increases unmet need by an average of 2.6 percentage points in countries with the highest levels of contraceptive use, compared with an average of 1.1 percentage point in countries with low CPR. Much

[8] Errors in the original calculation of unmet need were found in several surveys, all of which have been corrected in the tables in this report. Errors that changed estimates of unmet need by more than 2 percentage points were corrected in Azerbaijan 2006, Cambodia 2010, Chad 1996-97, Mali 2006, India 1992-93, Benin 2006, and Uganda 2006. Smaller errors were corrected in the Congo Democratic Republic 2007, Indonesia 1991, Bangladesh 2004, Turkey 2008, and Niger 1992 (changed unmet need by 0.5 to 2 percentage points). Very small errors in calculation (changed unmet need by 0.5 percentage points or less) were corrected in Bangladesh 2007, Indonesia 2007, India 2005-06, Colombia 2010, Dominican Republic 1996, Congo (Brazzaville) 2005, Niger 2006, Sao Tome and Principe 2008-09, Colombia 2005, Ukraine 2000, Egypt 2000, Nigeria 1990, Bolivia 1998, and Peru 2004-08.

[9] While sampling weights were used to calculate the percentage of women with an unmet need within each survey, the results from each survey were not weighted by the size of the population of each country. Each survey therefore represents one observation when averages are calculated across multiple surveys.

[10] The 169 surveys analyzed in this section include the 160 analyzed during the revision process, plus 9 newer surveys for which data have recently become available.

of the variety in the impact of changes (e.g. by region or unmet need tercile) can be explained by the inconsistent collection of calendar data. For example, the impact of moving from the **Original** to the **Revised** definition is largest in the Middle East/North Africa and Eastern Europe/NIS regions. In these regions more than two-thirds of surveys collected calendar data (data not shown). In West and Central Africa, where no surveys included the complete calendar, the impact is 1 percentage point.

Table 1. Total unmet need for family planning, summary

Total unmet need among currently married women 15-49 using the Original and Revised definitions, unweighted averages by survey characteristics, DHS surveys 1990-2010

	Total unmet need, Original definition	Total unmet need, Revised definition	Percentage point difference	Number of surveys
**Survey type**				
Calendar	13.9	17.3	3.3	65
Non-calendar	26.0	26.7	0.7	104
**Region**				
West and Central Africa	25.4	26.4	1.0	40
East and Southern Africa	26.6	27.7	1.1	44
Middle East/North Africa	13.5	16.7	3.1	13
Eastern Europe/NIS	11.3	13.8	2.4	13
Asia	19.9	21.9	2.1	30
Latin America and Caribbean	17.4	19.6	2.2	29
**CPR tercile**				
CPR - lower tercile (<25)	27.8	28.8	1.1	57
CPR - mid tercile (25-51)	24.5	26.0	1.5	56
CPR - upper tercile (>51)	11.7	14.3	2.6	56
**Unmet need tercile**				
Unmet need - upper tercile (>26)	31.7	32.3	0.7	55
Unmet need - mid tercile (16-26)	21.5	23.1	1.6	56
Unmet need - lower tercile (<16)	11.4	14.2	2.8	58
**Total**				
Average, 169 surveys	**21.4**	**23.1**	**1.7**	**169**

Note: while sampling weights were used to calculate the percentage of unmet need within each survey, the results from each survey were not weighted by the population of each country. Each survey therefore represents one observation; all averages are simple arithmetic means.

Unmet need tercile is based on the Original definition.

Table 2. Total unmet need for family planning, all surveys

Total unmet need among currently married women 15-49 using the Original and Revised definitions, DHS surveys 1990-2010

Survey Year	Total unmet need, Original definition	Total unmet need, Revised definition	Percentage point difference	Number of married women 15-49
**Countries with calendar data in every survey**				
Armenia 2000	11.8	18.1	6.3	4,125
Armenia 2005	13.3	19.3	6.1	4,044
Azerbaijan 2006	15.1	15.4	0.3	5,269
Brazil 1996	7.3	10.8	3.5	7,584
Egypt 1992	19.8	22.9	3.0	9,153
Egypt 1995	16.0	20.2	4.3	13,710
Egypt 2000	10.6	13.7	3.1	14,382
Egypt 2003	9.5	11.8	2.3	8,445
Egypt 2005	10.3	12.3	2.0	18,187
Egypt 2008	9.2	11.6	2.4	15,396
Guatemala 1995	24.3	28.1	3.7	7,984
Guatemala 1998-99	23.1	26.8	3.7	3,964
Indonesia 1991	14.1	17.0	2.9	21,109
Indonesia 1994	10.6	15.3	4.7	26,186
Indonesia 1997	9.2	13.6	4.4	26,886
Indonesia 2002-03	8.6	13.2	4.6	27,857
Indonesia 2007	8.8	13.1	4.3	30,931
Moldova 2005	6.7	11.4	4.7	4,937
Paraguay 1990	15.0	17.4	2.4	3,574
Peru 1991-92	15.5	21.6	6.1	8,741
Peru 1996	12.1	17.7	5.6	16,885
Peru 2000	10.2	14.4	4.2	15,628
Peru 2004-08	8.2	12.4	4.2	22,564
Turkey 1993	11.2	14.6	3.3	6,271
Turkey 1998	10.1	14.0	3.8	5,921
Turkey 2003	6.3	9.5	3.2	3,902
Ukraine 2007	10.3	10.1	-0.1	4,116
Vietnam 1997	6.9	8.4	1.4	5,340
Vietnam 2002	4.8	6.6	1.8	5,338
Zimbabwe 1994	14.9	19.1	4.2	3,788
Zimbabwe 1999	12.9	16.7	3.8	3,609
Zimbabwe 2005-06	12.0	15.5	3.5	5,143
**Countries with no calendar data**				
Albania 2008-09	12.8	12.9	0.1	5,001
Benin 1996	25.7	27.7	1.9	4,198
Benin 2001	27.2	27.9	0.7	4,563
Benin 2006	26.4	27.3	0.9	13,403
Burkina Faso 1993	24.5	24.6	0.1	5,326
Burkina Faso 1998-99	25.8	30.3	4.5	5,181
Burkina Faso 2003	28.8	29.8	1.0	9,655
Cameroon 1991	21.7	22.4	0.6	2,868
Cameroon 1998	19.7	20.7	1.0	3,676
Cameroon 2004	20.2	20.5	0.3	7,166
Central African Republic 1994-95	16.2	19.1	2.9	4,083

(Continued…)

Table 2 – *Continued*

Survey Year	Total unmet need, Original definition	Total unmet need, Revised definition	Percentage point difference	Number of married women 15-49
Chad 1996-97	15.1	17.4	2.3	5,832
Chad 2004	19.1	20.6	1.5	4,663
Comoros 1996	34.6	35.6	0.9	1,634
Congo (Brazzaville) 2005	19.3	19.5	0.2	3,979
Congo Democratic Republic 2007	26.2	26.9	0.7	6,622
Cote D'Ivoire 1994	27.1	30.4	3.3	5,271
Cote D'Ivoire 1998-99	27.7	28.9	1.3	1,863
Eritrea 1995	27.5	29.7	2.2	3,371
Eritrea 2002	27.0	28.5	1.4	5,733
Gabon 2000	28.0	27.9	-0.1	3,348
Ghana 1993	36.5	36.9	0.3	3,204
Ghana 1998	33.5	34.7	1.2	3,131
Ghana 2003	34.0	34.5	0.5	3,549
Ghana 2008	35.3	35.7	0.3	2,876
Guinea 1999	24.2	24.8	0.7	5,561
Guinea 2005	21.2	21.9	0.7	6,292
Guyana 2009	28.5	28.5	0.0	2,920
Haiti 1994-95	44.5	44.7	0.2	3,113
Haiti 2000	39.6	39.6	0.0	5,958
Haiti 2005-06	37.5	37.3	-0.1	6,323
Honduras 2005-06	16.9	16.8	-0.1	11,613
Kyrgyz Republic 1997	11.6	11.8	0.2	2,675
Lesotho 2004	31.0	31.0	0.0	3,709
Lesotho 2009	23.0	23.3	0.3	4,049
Liberia 2007	35.6	35.7	0.1	4,540
Madagascar 1992	32.4	32.3	-0.1	3,736
Madagascar 1997	25.6	27.7	2.1	4,435
Madagascar 2003-04	23.6	24.5	0.8	5,140
Madagascar 2008-09	18.9	19.0	0.1	12,039
Maldives 2009	28.1	28.6	0.5	6,500
Mali 1995-96	25.7	27.5	1.8	8,222
Mali 2001	28.5	29.6	1.1	10,723
Mali 2006	26.7	27.6	0.9	12,365
Mauritania 2000-01	31.6	32.1	0.5	4,541
Mozambique 1997	22.5	24.9	2.4	6,530
Mozambique 2003	18.4	18.9	0.5	8,736
Namibia 1992	21.9	21.8	-0.1	2,259
Namibia 2000	22.1	23.9	1.7	2,610
Namibia 2006-07	20.6	20.7	0.2	3,451
Nepal 1996	31.4	32.4	1.0	7,982
Nepal 2001	27.8	27.8	0.0	8,342
Nepal 2006	24.6	24.7	0.1	8,257
Niger 1992	18.1	18.7	0.5	5,561
Niger 1998	16.6	17.7	1.1	6,382
Niger 2006	15.7	16.1	0.4	7,941
Nigeria 1990	20.5	21.5	1.0	6,880
Nigeria 1999	17.5	20.0	2.5	5,757

(Continued…)

Table 2 – *Continued*

Survey Year	Total unmet need, Original definition	Total unmet need, Revised definition	Percentage point difference	Number of married women 15-49
Nigeria 2003	16.9	17.5	0.6	5,336
Nigeria 2008	20.2	20.2	0.0	23,578
Pakistan 1990-91	31.8	30.5	-1.3	6,364
Pakistan 2006-07	24.9	25.2	0.3	9,556
Rwanda 1992	38.8	38.2	-0.5	3,785
Rwanda 2000	35.6	36.4	0.8	5,052
Rwanda 2005	37.9	38.5	0.6	5,510
Samoa 2009	45.6	47.7	2.2	1,554
Sao Tome and Principe 2008-09	37.1	37.6	0.5	1,718
Senegal 1992-93	29.3	28.8	-0.4	4,450
Senegal 2005	31.6	32.0	0.4	9,866
Sierra Leone 2008	27.6	28.4	0.8	5,525
South Africa 1998	15.0	16.5	1.6	5,077
Swaziland 2006-07	23.8	24.7	0.9	2,062
Timor-Leste 2009	30.8	31.5	0.7	7,906
Togo 1998	32.3	35.0	2.7	5,819
Uganda 1995	29.0	30.0	1.0	5,136
Uganda 2000-01	34.6	35.0	0.4	4,881
Uganda 2006	37.8	38.0	0.2	5,337
Uzbekistan 1996	13.7	13.7	0.0	3,102
Zambia 1992	30.7	30.0	-0.7	4,457
Zambia 1996	26.5	25.2	-1.3	4,902
Zambia 2001-02	27.4	27.5	0.1	4,694
Zambia 2007	26.5	26.6	0.1	4,402
**Countries with inconsistent collection of calendar data**				
Bangladesh 1993-94	17.9	21.6	3.7	8,840
Bangladesh 1996-97	15.7	19.7	4.0	8,307
Bangladesh 1999-2000	15.0	18.2	3.2	9,540
Bangladesh 2004	11.8	15.0	3.2	10,436
Bangladesh 2007	16.8	16.8	0.0	10,192
Bolivia 1994	23.2	28.9	5.7	5,334
Bolivia 1998	26.0	26.6	0.5	6,649
Bolivia 2003	22.7	22.8	0.2	10,569
Bolivia 2008	20.2	20.1	-0.1	10,162
Cambodia 2000	29.7	33.0	3.4	9,071
Cambodia 2005	25.1	25.3	0.2	10,087
Cambodia 2010	16.4	16.9	0.6	11,626
Colombia 1990	11.1	13.7	2.6	4,450
Colombia 1995	7.7	11.4	3.6	6,097
Colombia 2000	6.1	10.0	3.9	5,935
Colombia 2005	5.7	8.6	2.9	19,762
Colombia 2010	6.9	8.0	1.1	26,247
Dominican Republic 1991	17.2	19.4	2.2	4,083
Dominican Republic 1996	12.3	14.3	2.0	4,983
Dominican Republic 1999	11.9	13.8	2.0	728
Dominican Republic 2002	10.9	12.4	1.5	13,996
Dominican Republic 2007	11.4	11.1	-0.2	15,417

(Continued…)

Table 2 – *Continued*

Survey Year	Total unmet need, Original definition	Total unmet need, Revised definition	Percentage point difference	Number of married women 15-49
Ethiopia 2000	35.2	36.6	1.4	9,789
Ethiopia 2005	33.8	36.1	2.3	9,066
India 1992-93	20.1	20.3	0.2	84,328
India 1998-99	15.8	16.1	0.3	84,682
India 2005-06	12.6	13.9	1.3	93,089
Jordan 1990	22.2	26.5	4.3	6,168
Jordan 1997	14.2	20.1	5.9	5,337
Jordan 2002	11.0	14.9	3.9	5,706
Jordan 2007	11.9	13.8	1.9	10,354
Jordan 2009	11.2	13.4	2.2	9,651
Kazakhstan 1995	15.7	16.3	0.6	2,507
Kazakhstan 1999	8.7	11.9	3.2	3,018
Kenya 1993	35.5	35.3	-0.2	4,629
Kenya 1998	23.9	28.0	4.1	4,834
Kenya 2003	24.5	27.4	2.9	4,919
Kenya 2008-09	25.6	25.6	0.0	4,928
Malawi 1992	35.7	36.5	0.8	3,492
Malawi 2000	29.7	29.9	0.3	9,452
Malawi 2004	27.6	30.3	2.7	8,312
Malawi 2010	26.1	26.2	0.0	15,528
Morocco 1992	19.7	23.5	3.8	5,118
Morocco 2003-04	10.0	11.9	1.9	8,782
Nicaragua 1998	14.7	17.9	3.1	8,045
Nicaragua 2001	14.6	14.6	0.1	7,424
Philippines 1993	25.9	30.2	4.2	8,961
Philippines 1998	18.8	24.6	5.8	8,336
Philippines 2003	17.3	22.5	5.2	8,671
Philippines 2008	22.3	22.0	-0.3	8,418
Tanzania 1991-92	27.9	27.8	-0.1	6,038
Tanzania 1996	23.9	26.0	2.1	5,411
Tanzania 1999	21.8	22.3	0.5	2,653
Tanzania 2004-05	21.8	24.3	2.4	6,950
Tanzania 2010	25.3	25.3	0.0	6,412
**Unweighted Average**	**21.4**	**23.1**	**1.7**	
Range of differences:				
Minimum difference			-1.3	
Maximum difference			6.3	

Table 2 shows the impact of moving from the **Original** to the **Revised** definition for each survey. Although the impact of the revision in definition varies by survey, trends in the impact can be seen in three broad groups: 1) countries that implemented the calendar in every survey, shown in Figures 3 through 6; 2) countries that did not collect calendar data in any survey, shown in Figures 7 through 10; and 3) countries that collected calendar data in some surveys but not others, shown in Figures 11 through 16.

3.1.1 Countries That Implemented the Calendar in Every Survey

Egypt, Zimbabwe, Indonesia, and Peru (Figures 3 through 6), all collected calendar data including discontinuation and/or marriage data in every DHS survey. Removing calendar data, along with making the other changes approved by the TEWG, consistently increases the level of unmet need. In Egypt, Zimbabwe, and Indonesia the difference between the two estimates is largest in surveys conducted between 1995 and 2002, all of which included either the “happy” or “problem” question, which decreased the level of unmet need. Later surveys in these countries did not include either question, and the differences between levels of unmet need are smaller, comparing the **Original** and **Revised** definitions.

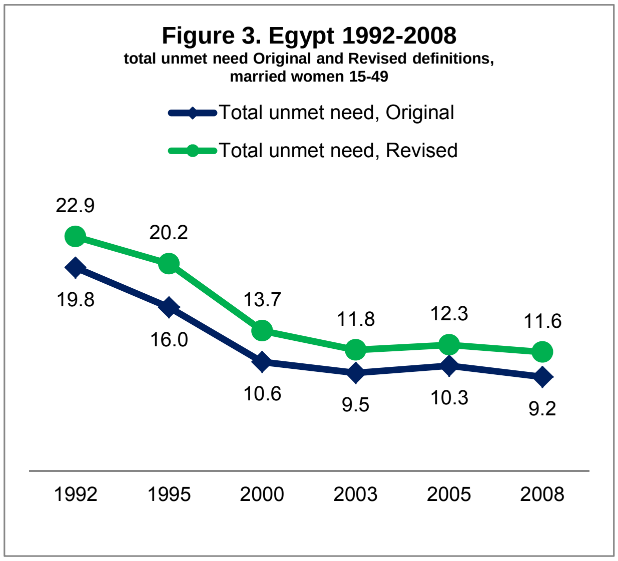

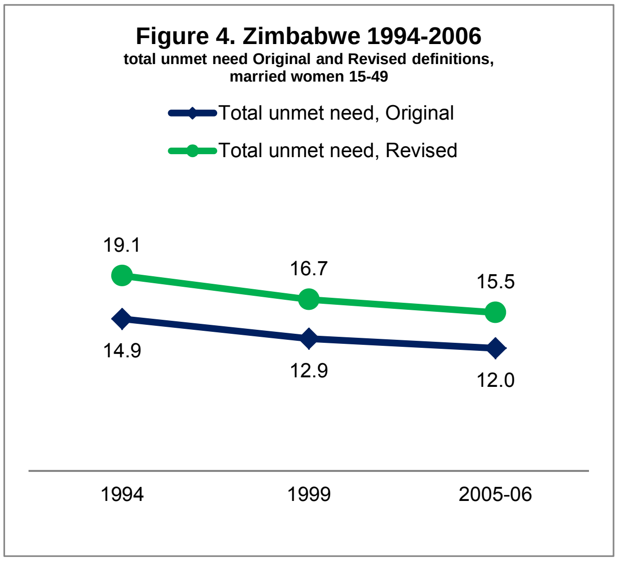

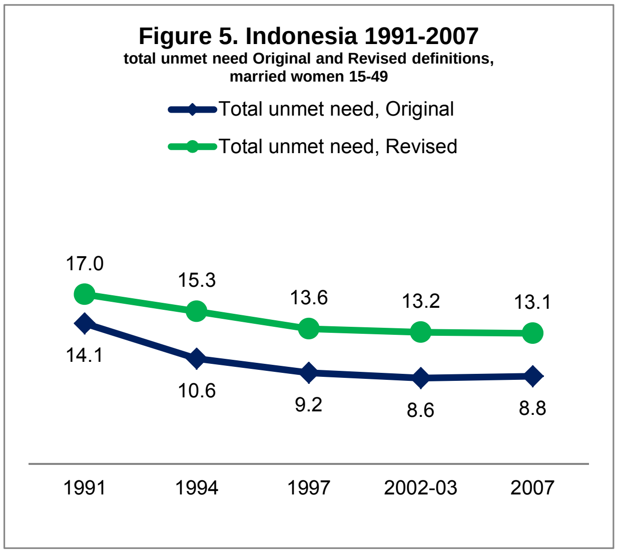

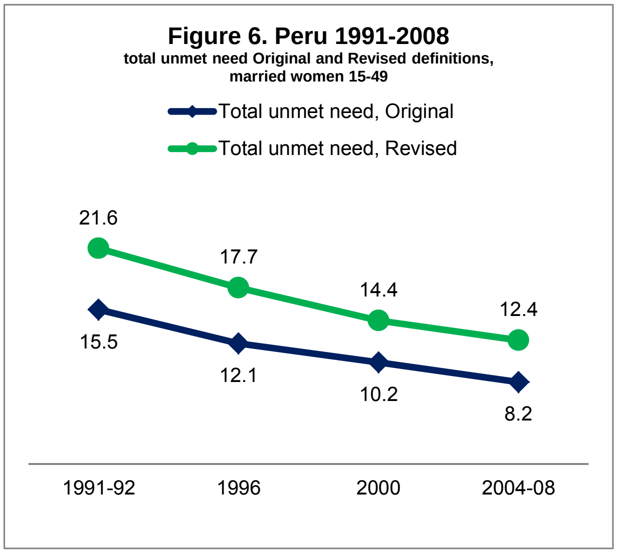

In Peru, the largest difference is in the 1991-92 survey, where the estimated level of unmet need is 6.1 percentage points higher using the **Revised** compared with the **Original** definition. In this survey the difference is due to a particularly high level of contraceptive failure. In the 1991-92 Peru survey, 6.4 percent of married women were pregnant or postpartum amenorrheic due to contraceptive failure and so could not have an unmet need, according to the **Original** definition. The **Revised** definition does not use contraceptive failure in the algorithm, so women who were treated as having contraceptive failure (and

thus as having no need) in the **Original** definition are treated in the **Revised** definition as having an unmet need if their current pregnancy or last birth was wanted later or not wanted at all. In later surveys in Peru, as use of modern contraception increased, failure rates decreased, and the gap between the two definitions of unmet need narrowed.

3.1.2 Countries That Did Not Collect Calendar Data in Any Survey

Figures 7 through 10 show trends in both definitions of unmet need in four countries that did not include calendar data in any survey: Haiti, Madagascar, Nepal, and Niger. In these countries there is almost no difference in the levels of unmet need calculated using the **Original** and the **Revised** definitions. Slight differences are introduced with use of the "happy" question in some surveys (Madagascar 1997, Nepal 1996, Niger 1998) and with missing data. In these four countries the definitional changes approved by the TEWG had little or no effect on the total unmet need estimates.

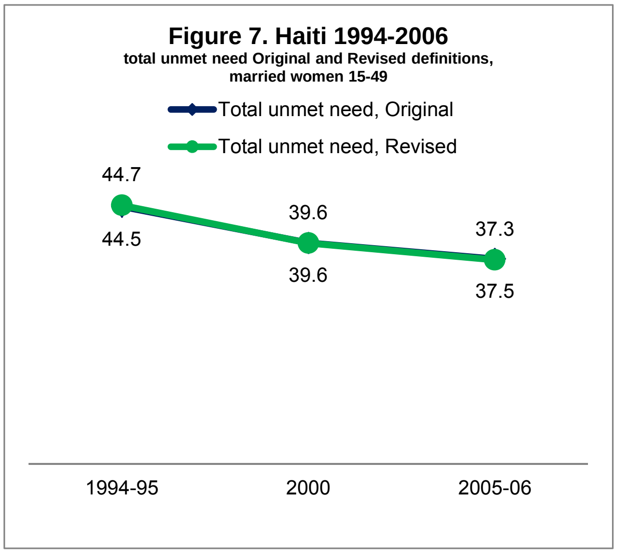

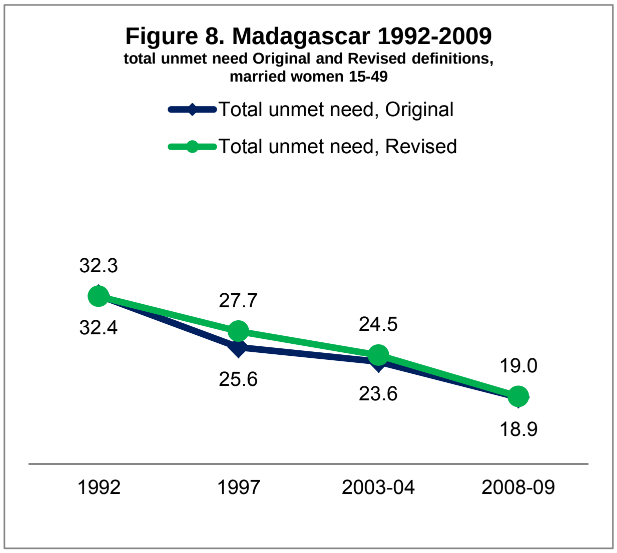

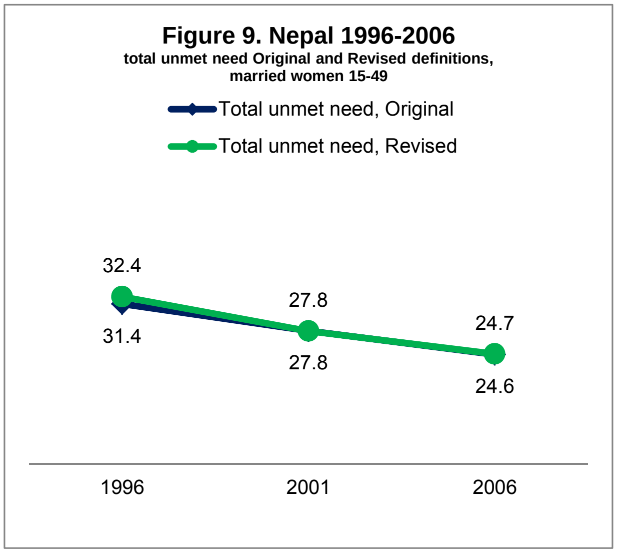

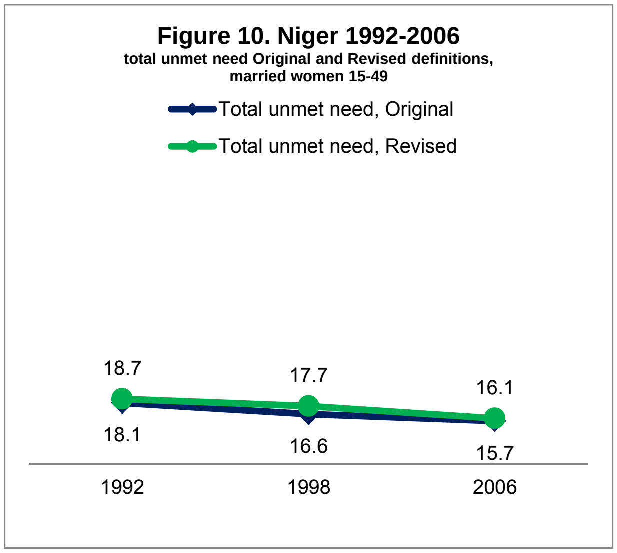

3.1.3 Countries That Collected Calendar Data in Some Surveys but Not Others

Figures 11 through 16 show trends in total unmet need using each definition in six countries that included the calendar inconsistently: Kenya, Bolivia, the Dominican Republic, Colombia, the Philippines, and Jordan. Kenya (Figure 11) collected calendar data in the 1998 and 2003 surveys but not in the 1993 or 2008 surveys. Including the calendar in the 1998 and 2003 surveys seemed to decrease the level of unmet need, but that was only an artifact of the change in definitions. Inclusion of the "happy" question in the 1998 survey also decreased unmet need compared with the other Kenya surveys, none of which included that question. When the consistent **Revised** definition is used, unmet need decreased sharply with an increase in contraceptive use between 1993 and 1998, remained stable between 1998 and 2003, and then decreased between 2003 and 2008-9, mirroring changes in contraceptive prevalence. The **Revised** definition gives a much different—and much easier to interpret—picture of trends in unmet need than the inconsistent **Original** definition.

In Bolivia (Figure 12), trends in unmet need show an inverted V-shape, with an increase between 1994 and 1998, and a steady decrease thereafter. The spike in 1998, however, is due to calendar data having been included in the definition of unmet need in 1994, but not in later surveys. When the consistent, **Revised** definition is applied, it is clear that unmet need has decreased steadily. Given other indicators, such as the steady increase in Bolivia's CPR during this period, a steady decrease in unmet need makes much more sense than the inverted V-shaped trend.

The Dominican Republic (Figure 13) and Colombia (Figure 14) both included calendar data in the first four surveys, conducted between 1990 and 2005. In the Dominican Republic, no calendar data were collected in 2007. Colombia used a modified calendar in 2010, collecting information on reasons for discontinuation (used to collect data on contraceptive failure), but excluding the marital status column (used to measure infecundity). In both countries the outcome is the same: an apparent increase in unmet need between the last two surveys is instead a slight decrease, which can be seen using the consistent, **Revised** definition.

In the Philippines (Figure 15), in the **Original** definition the apparent sharp increase in unmet need between 2003 and 2008 is attributable to the inclusion of a contraceptive calendar in the 1993, 1998, and 2003 surveys, and its exclusion in 2008. If the **Revised** definition of unmet need is consistently applied to all surveys, however, there is no increase in unmet need over this period. Instead, it remained at the same level between 2003 and 2008. The reported apparent increase in unmet need between 2003 and 2008, which has been analyzed as a cause for concern (NSO and ICF Macro 2009), is due solely to the removal of calendar data.

In Jordan the calendar has been implemented in every survey (Figure 16), but in slightly different formats. Similar to Colombia, in the first three surveys shown (1990 to 2002), a full calendar was used, including columns on marital status and reasons for discontinuation. In the 2007 and 2009 surveys, the marital status column was not collected. Additionally, the 1997 survey included the "happy" question and the 2002 survey included the "problem" question, while the 2007 and 2009 surveys included neither. The inclusion of inconsistently collected data makes it appear as though the level of unmet need increased between 2002 and 2007 and remained flat between 2007 and 2009. Although the apparent increase in unmet need from 11.0 percent in 2002 to 11.9 percent in 2007 is small, it has been considered a significant program issue in Jordan (Department of Statistics [Jordan] and Macro International Inc., 2008). When the **Revised** definition, which excludes any inconsistently collected data, is used in every survey, however, it is clear that unmet need has decreased consistently in every survey in Jordan, albeit more slowly in recent years.

Figure 11. Kenya 1993-2009
total unmet need Original and Revised definitions, married women 15-49

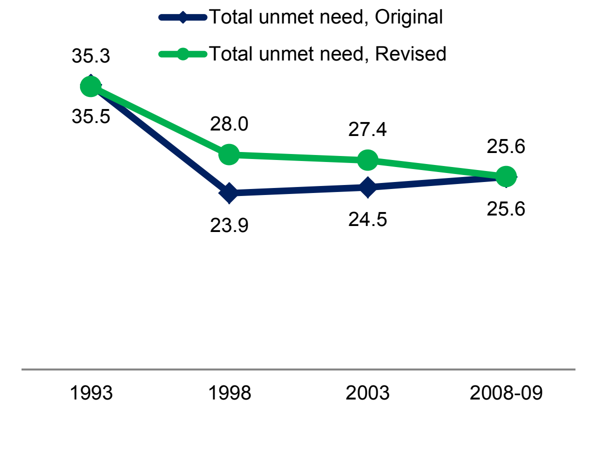

Figure 12. Bolivia 1994-2008
total unmet need Original and Revised definitions, married women 15-49

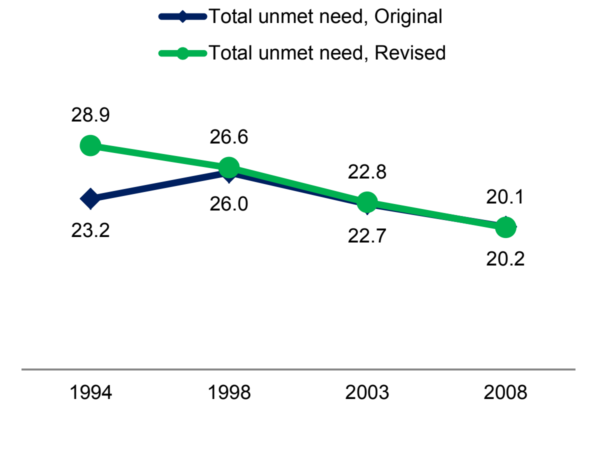

Figure 13. Dominican Republic 1991-2007
total unmet need Original and Revised definitions, married women 15-49

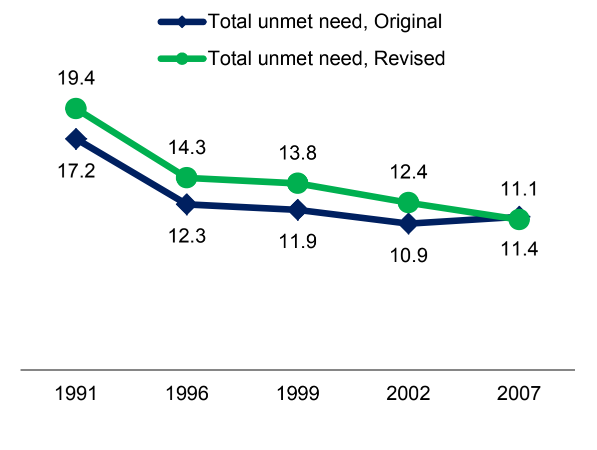

Figure 14. Colombia 1990-2010
total unmet need Original and Revised definitions, married women 15-49

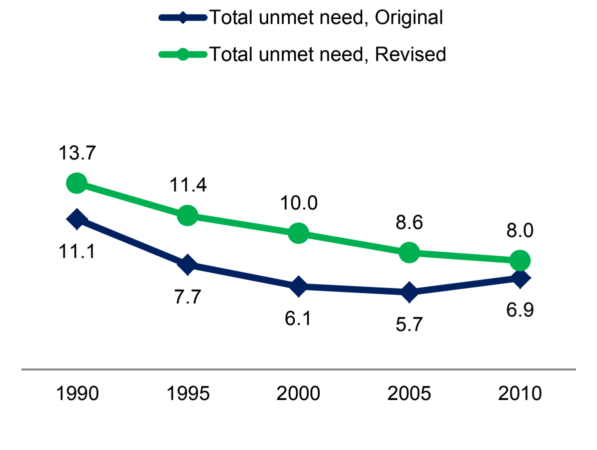

Figure 15. Philippines 1993-2008
total unmet need Original and Revised definitions, married women 15-49

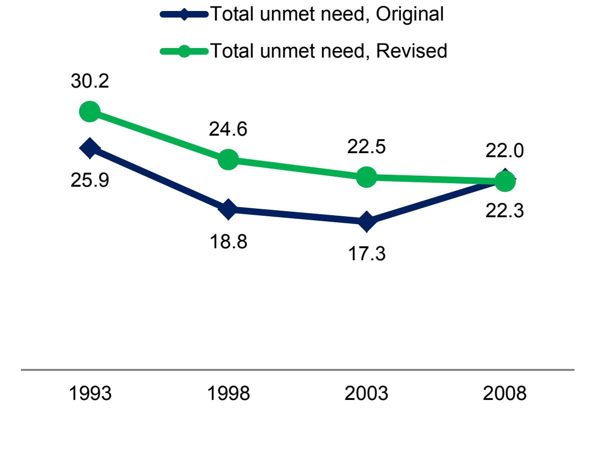

Figure 16. Jordan 1990-2009
total unmet need Original and Revised definitions, married women 15-49

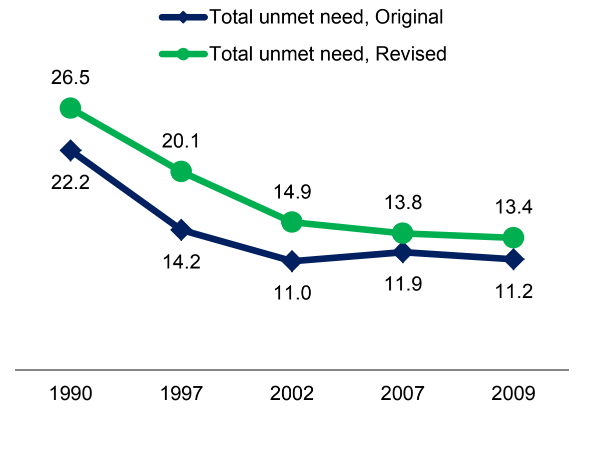

3.2 Changes in Unmet Need for Spacing and Limiting

On average across all surveys, implementing all revisions approved by the TEWG increases unmet need for spacing from 12.0 to 12.4 percent, and increases unmet need for limiting from 9.3 to 10.7 percent. The increase in unmet need for limiting is greater than the increase for spacing primarily because the **Revised** definition removes the modification added in recent surveys to the treatment of pregnant and postpartum amenorrheic women who want no more children, which had shifted some women from having an unmet need for limiting to having an unmet need for spacing instead, based on their intentions for future births. As with trends in total unmet need, the trends in unmet need for spacing and limiting are clearer and easier to interpret using the consistent **Revised** definition compared with the **Original** definition.

Table 3. Unmet need for spacing and limiting

Unmet need for spacing and unmet need for limiting among currently married women 15-49 using the Original and Revised definitions, DHS surveys 1990-2010

	Original definition		Revised definition			
Survey Year	Unmet need for spacing	Unmet need for limiting	Unmet need for spacing	Unmet need for limiting	Difference in spacing	Difference in limiting
**Countries with calendar data in every survey**						
Armenia 2000	2.6	9.3	3.5	14.6	0.9	5.3
Armenia 2005	3.6	9.7	3.9	15.5	0.3	5.8
Azerbaijan 2006	2.9	12.2	3.0	12.5	0.0	0.3
Brazil 1996	2.6	4.7	4.0	6.8	1.4	2.1
Egypt 1992	6.9	12.9	7.1	15.8	0.2	2.9
Egypt 1995	5.3	10.7	6.4	13.8	1.1	3.2
Egypt 2000	3.1	7.6	3.8	9.9	0.7	2.3
Egypt 2003	3.5	6.0	3.7	8.1	0.2	2.1
Egypt 2005	3.6	6.7	3.5	8.8	-0.1	2.1
Egypt 2008	3.4	5.8	3.4	8.2	0.0	2.4
Guatemala 1995	12.4	12.0	14.4	13.7	2.0	1.7
Guatemala 1998-99	11.8	11.3	13.8	13.1	1.9	1.8
Indonesia 1991	7.9	6.2	8.6	8.4	0.8	2.2
Indonesia 1994	4.8	5.8	6.6	8.7	1.8	2.9
Indonesia 1997	4.2	4.9	5.9	7.7	1.7	2.8
Indonesia 2002-03	4.0	4.6	4.7	8.5	0.7	4.0
Indonesia 2007	4.1	4.7	4.8	8.3	0.7	3.7
Moldova 2005	2.5	4.2	3.1	8.2	0.6	4.0
Paraguay 1990	8.9	6.1	10.3	7.1	1.4	1.0
Peru 1991-92	4.3	11.2	6.2	15.4	1.9	4.2
Peru 1996	3.5	8.6	5.5	12.2	2.0	3.6
Peru 2000	3.5	6.7	4.9	9.4	1.4	2.8
Peru 2004-08	3.1	5.1	5.0	7.4	1.9	2.3
Turkey 1993	3.8	7.5	4.4	10.2	0.7	2.7
Turkey 1998	3.8	6.3	5.0	9.0	1.1	2.7
Turkey 2003	2.4	3.9	3.1	6.4	0.6	2.6
Ukraine 2007	3.8	6.4	3.7	6.4	-0.1	0.0
Vietnam 1997	3.5	3.5	3.6	4.8	0.1	1.3
Vietnam 2002	2.0	2.8	2.3	4.4	0.3	1.5
Zimbabwe 1994	9.2	5.6	10.6	8.5	1.4	2.8
Zimbabwe 1999	7.3	5.6	8.5	8.2	1.2	2.6
Zimbabwe 2005-06	7.0	5.0	7.2	8.2	0.2	3.3

(Continued…)

Table 3 – *Continued*

Survey Year	Original definition: Unmet need for spacing	Original definition: Unmet need for limiting	Revised definition: Unmet need for spacing	Revised definition: Unmet need for limiting	Difference in spacing	Difference in limiting
**Countries with no calendar data**						
Albania 2008-09	3.4	9.4	3.5	9.4	0.1	0.0
Benin 1996	17.2	8.6	18.7	9.0	1.5	0.4
Benin 2001	17.5	9.7	18.1	9.8	0.6	0.1
Benin 2006	17.0	9.4	17.4	9.9	0.4	0.5
Burkina Faso 1993	18.3	6.2	18.0	6.6	-0.3	0.4
Burkina Faso 1998-99	19.0	6.8	22.8	7.5	3.8	0.7
Burkina Faso 2003	21.8	7.0	22.3	7.5	0.5	0.5
Cameroon 1991	17.1	4.6	17.4	4.9	0.3	0.3
Cameroon 1998	13.3	6.4	13.9	6.8	0.6	0.4
Cameroon 2004	14.2	6.0	14.1	6.5	-0.1	0.4
Central African Republic 1994-95	11.6	4.6	13.7	5.3	2.1	0.8
Chad 1996-97	12.1	3.1	13.9	3.6	1.8	0.5
Chad 2004	16.6	2.5	17.9	2.7	1.3	0.2
Comoros 1996	21.8	12.9	22.0	13.5	0.2	0.7
Congo (Brazzaville) 2005	14.3	5.0	14.0	5.5	-0.3	0.5
Congo Democratic Republic 2007	20.1	6.1	19.9	7.0	-0.2	0.9
Cote D'Ivoire 1994	20.0	7.1	20.8	9.6	0.8	2.4
Cote D'Ivoire 1998-99	20.0	7.6	21.0	7.9	1.0	0.3
Eritrea 1995	21.4	6.1	22.7	7.0	1.3	0.9
Eritrea 2002	21.0	6.0	21.6	6.8	0.6	0.8
Gabon 2000	19.9	8.0	19.7	8.1	-0.2	0.1
Ghana 1993	25.2	11.4	24.8	12.1	-0.4	0.7
Ghana 1998	21.7	11.8	22.5	12.3	0.7	0.5
Ghana 2003	21.7	12.3	20.7	13.7	-1.0	1.4
Ghana 2008	22.5	12.9	21.5	14.2	-1.0	1.3
Guinea 1999	16.0	8.2	16.1	8.7	0.1	0.5
Guinea 2005	13.1	8.1	13.4	8.5	0.3	0.4
Guyana 2009	9.5	19.0	9.4	19.1	-0.1	0.1
Haiti 1994-95	18.4	26.1	17.0	27.7	-1.4	1.6
Haiti 2000	15.8	23.8	15.6	24.0	-0.3	0.2
Haiti 2005-06	17.0	20.4	16.5	20.8	-0.5	0.4
Honduras 2005-06	8.4	8.4	8.0	8.8	-0.5	0.4
Kyrgyz Republic 1997	4.5	7.2	4.7	7.1	0.2	0.0
Lesotho 2004	11.0	20.0	9.6	21.4	-1.3	1.4
Lesotho 2009	10.6	12.3	10.9	12.4	0.2	0.1
Liberia 2007	24.6	11.0	24.2	11.4	-0.4	0.4
Madagascar 1992	17.5	14.9	16.2	16.2	-1.4	1.2
Madagascar 1997	14.1	11.4	14.7	12.9	0.6	1.5
Madagascar 2003-04	11.3	12.3	11.8	12.7	0.5	0.4
Madagascar 2008-09	10.4	8.5	10.2	8.8	-0.2	0.3
Maldives 2009	14.9	13.2	15.0	13.6	0.1	0.4
Mali 1995-96	20.1	5.7	21.0	6.5	0.9	0.8
Mali 2001	20.9	7.6	21.5	8.2	0.6	0.5
Mali 2006	20.0	6.7	20.3	7.3	0.3	0.6
Mauritania 2000-01	22.9	8.6	23.2	8.9	0.3	0.3
Mozambique 1997	16.9	5.6	18.7	6.3	1.8	0.6
Mozambique 2003	10.8	7.5	10.9	8.0	0.1	0.5
Namibia 1992	15.1	6.8	14.6	7.1	-0.4	0.3
Namibia 2000	9.3	12.8	9.9	14.0	0.6	1.1

(Continued…)

Table 3 – *Continued*

Survey Year	Original definition: Unmet need for spacing	Original definition: Unmet need for limiting	Revised definition: Unmet need for spacing	Revised definition: Unmet need for limiting	Difference in spacing	Difference in limiting
Namibia 2006-07	9.1	11.5	8.6	12.1	-0.5	0.6
Nepal 1996	14.3	17.1	14.8	17.5	0.6	0.4
Nepal 2001	11.4	16.4	11.1	16.7	-0.3	0.3
Nepal 2006	9.4	15.2	9.3	15.4	0.0	0.1
Niger 1992	15.8	2.3	15.6	3.1	-0.2	0.7
Niger 1998	14.0	2.7	14.7	3.0	0.7	0.3
Niger 2006	13.2	2.5	13.4	2.7	0.2	0.2
Nigeria 1990	15.8	4.8	16.5	5.1	0.7	0.3
Nigeria 1999	12.9	4.6	15.1	4.9	2.2	0.3
Nigeria 2003	11.8	5.1	12.0	5.5	0.2	0.4
Nigeria 2008	15.0	5.2	14.5	5.7	-0.5	0.5
Pakistan 1990-91	16.6	15.2	15.0	15.4	-1.5	0.2
Pakistan 2006-07	10.9	14.0	10.8	14.4	-0.1	0.4
Rwanda 1992	25.0	13.8	20.2	18.0	-4.8	4.2
Rwanda 2000	24.0	11.6	22.8	13.5	-1.1	1.9
Rwanda 2005	24.5	13.4	23.5	14.9	-1.0	1.5
Samoa 2009	20.1	25.4	20.6	27.2	0.4	1.7
Sao Tome and Principe 2008-09	19.1	18.0	18.0	19.6	-1.1	1.6
Senegal 1992-93	22.6	6.7	21.7	7.1	-0.9	0.4
Senegal 2005	24.2	7.3	24.3	7.7	0.1	0.3
Sierra Leone 2008	16.4	11.2	16.1	12.2	-0.3	1.1
South Africa 1998	4.7	10.3	5.7	10.8	1.0	0.5
Swaziland 2006-07	7.3	16.5	6.7	18.1	-0.6	1.5
Timor-Leste 2009	20.5	10.2	20.9	10.6	0.3	0.4
Togo 1998	21.4	10.9	23.6	11.4	2.2	0.4
Uganda 1995	18.3	10.7	18.9	11.1	0.6	0.3
Uganda 2000-01	20.7	13.9	20.3	14.7	-0.4	0.8
Uganda 2006	24.1	13.7	23.7	14.3	-0.4	0.6
Uzbekistan 1996	6.6	7.0	6.5	7.2	-0.1	0.1
Zambia 1992	22.9	7.8	20.8	9.2	-2.1	1.4
Zambia 1996	18.7	7.8	16.9	8.3	-1.7	0.4
Zambia 2001-02	16.8	10.6	15.0	12.5	-1.8	1.9
Zambia 2007	17.1	9.4	15.9	10.6	-1.1	1.3
**Countries with inconsistent collection of calendar data**						
Bangladesh 1993-94	8.8	9.0	10.7	10.9	1.8	1.9
Bangladesh 1996-97	7.7	8.0	9.7	10.0	2.0	1.9
Bangladesh 1999-2000	7.6	7.4	8.5	9.7	0.9	2.3
Bangladesh 2004	5.6	6.3	6.7	8.3	1.1	2.1
Bangladesh 2007	6.6	10.2	6.7	10.1	0.0	-0.1
Bolivia 1994	5.5	17.7	7.5	21.4	1.9	3.8
Bolivia 1998	6.8	19.3	6.7	19.9	-0.1	0.6
Bolivia 2003	6.1	16.6	6.0	16.9	-0.1	0.3
Bolivia 2008	6.4	13.8	6.2	14.0	-0.2	0.1
Cambodia 2000	14.4	15.2	17.2	15.9	2.7	0.6
Cambodia 2005	8.9	16.2	8.5	16.8	-0.4	0.6
Cambodia 2010	6.0	10.4	6.1	10.8	0.1	0.4
Colombia 1990	4.2	6.9	4.8	9.0	0.6	2.0
Colombia 1995	3.2	4.6	4.8	6.6	1.6	2.0
Colombia 2000	2.6	3.5	4.4	5.7	1.8	2.2
Colombia 2005	2.5	3.2	3.7	4.9	1.2	1.7

(Continued…)

Table 3 – *Continued*

Survey Year	Original definition: Unmet need for spacing	Original definition: Unmet need for limiting	Revised definition: Unmet need for spacing	Revised definition: Unmet need for limiting	Difference in spacing	Difference in limiting
Colombia 2010	3.0	4.0	3.6	4.4	0.6	0.5
Dominican Republic 1991	8.8	8.3	9.1	10.3	0.3	2.0
Dominican Republic 1996	7.1	5.2	8.2	6.0	1.1	0.8
Dominican Republic 1999	7.4	4.5	8.2	5.7	0.8	1.2
Dominican Republic 2002	6.7	4.2	6.9	5.5	0.3	1.3
Dominican Republic 2007	7.0	4.4	6.7	4.4	-0.3	0.1
Ethiopia 2000	21.3	13.8	20.9	15.7	-0.4	1.8
Ethiopia 2005	20.1	13.7	19.5	16.6	-0.6	2.9
India 1992-93	8.8	7.6	12.2	8.1	3.3	0.5
India 1998-99	8.3	7.5	8.3	7.8	0.0	0.3
India 2005-06	6.0	6.5	6.1	7.8	0.0	1.3
Jordan 1990	9.4	12.8	9.7	16.8	0.3	4.0
Jordan 1997	7.4	6.8	9.9	10.2	2.5	3.4
Jordan 2002	5.6	5.5	7.0	7.9	1.5	2.4
Jordan 2007	4.9	7.0	5.7	8.2	0.8	1.1
Jordan 2009	4.7	6.5	6.0	7.4	1.3	0.9
Kazakhstan 1995	4.0	11.8	4.6	11.7	0.6	-0.1
Kazakhstan 1999	3.6	5.1	4.1	7.7	0.5	2.7
Kenya 1993	22.0	13.5	20.7	14.6	-1.3	1.1
Kenya 1998	14.0	9.9	16.0	11.9	2.1	2.0
Kenya 2003	14.4	10.1	15.2	12.2	0.8	2.1
Kenya 2008-09	12.9	12.8	12.5	13.1	-0.3	0.4
Malawi 1992	25.8	9.9	24.3	12.2	-1.6	2.4
Malawi 2000	17.2	12.5	14.9	15.0	-2.3	2.5
Malawi 2004	17.2	10.4	16.2	14.1	-1.1	3.7
Malawi 2010	14.2	11.9	12.4	13.8	1.9	-1.9
Morocco 1992	8.6	11.1	9.6	13.9	1.0	2.8
Morocco 2003-04	3.5	6.6	4.4	7.4	1.0	0.9
Nicaragua 1998	6.3	8.4	7.3	10.5	1.0	2.1
Nicaragua 2001	5.9	8.7	5.6	9.0	-0.2	0.3
Philippines 1993	12.5	13.4	13.5	16.7	1.0	3.3
Philippines 1998	8.2	10.6	10.2	14.3	2.1	3.7
Philippines 2003	7.9	9.4	9.1	13.5	1.1	4.0
Philippines 2008	9.0	13.4	8.5	13.5	-0.4	0.1
Tanzania 1991-92	19.9	7.9	18.4	9.4	-1.5	1.4
Tanzania 1996	15.4	8.5	15.8	10.1	0.4	1.6
Tanzania 1999	13.8	8.0	13.3	9.0	-0.5	0.9
Tanzania 2004-05	15.1	6.7	16.1	8.2	1.0	1.5
Tanzania 2010	15.9	9.5	15.9	9.4	0.0	-0.1
**Unweighted Averages**	**12.0**	**9.3**	**12.4**	**10.7**	**0.4**	**1.4**
Range of differences:						
Minimum difference					-4.8	-0.1
Maximum difference					3.8	5.8

Figures 17 through 19 show trends in unmet need for spacing and limiting, comparing the **Original** and **Revised** definitions, in Bolivia, the Dominican Republic, and the Philippines. In Bolivia (Figures 17a and 17b), unmet need for spacing decreases consistently (or remains stable) using the **Revised** definition, while the **Original** definition shows an increase between 1994 and 1998. The increase is even sharper in

unmet need for limiting using the **Original** definition, which shows an inverted V-shaped pattern. The **Revised** definition, however, shows a steady decrease in unmet need for limiting with each survey.

In the Dominican Republic, unmet need for spacing calculated using the **Original** definition shows no clear pattern, changing between slight increases or decreases with each survey (Figure 18a). The **Revised** definition shows a pattern that decreases or remains steady with each successive survey. Similarly, unmet need for limiting (Figure 18b) shows a steady decrease with the **Revised** definition, but the trend is less clear with the **Original** definition due to inconsistent inclusion of calendar data in this definition.

Unmet need for both spacing and limiting appears to increase in the Philippines between 2003 and 2008 (Figures 19a and 19b). However, this is an artifact of the changes in the **Original** definition from one survey to the next. Using the **Revised** definition, unmet need for limiting decreases steadily with each survey, and unmet need for spacing decreases or does not change.

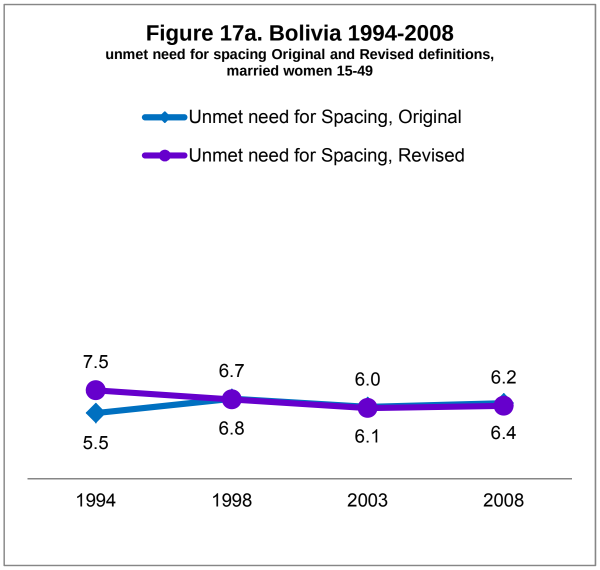
Figure 17a. Bolivia 1994-2008
unmet need for spacing Original and Revised definitions, married women 15-49
Unmet need for Spacing, Original
Unmet need for Spacing, Revised
7.5
6.7
6.0
6.2
5.5
6.8
6.1
6.4
1994
1998
2003
2008

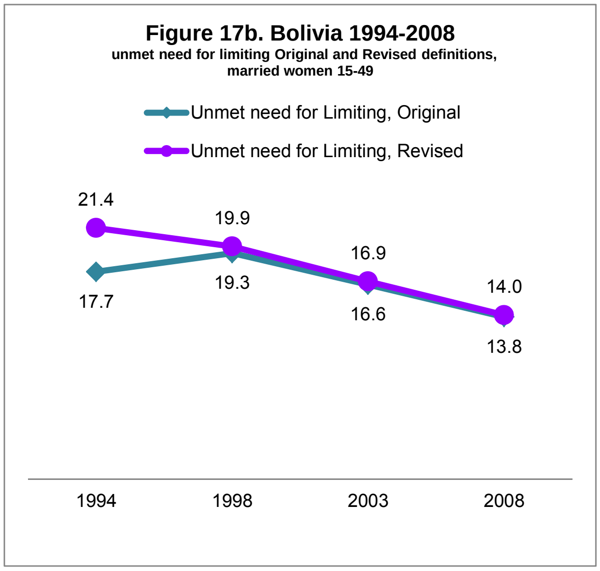
Figure 17b. Bolivia 1994-2008
unmet need for limiting Original and Revised definitions, married women 15-49
Unmet need for Limiting, Original
Unmet need for Limiting, Revised
21.4
19.9
16.9
14.0
17.7
19.3
16.6
13.8
1994
1998
2003
2008

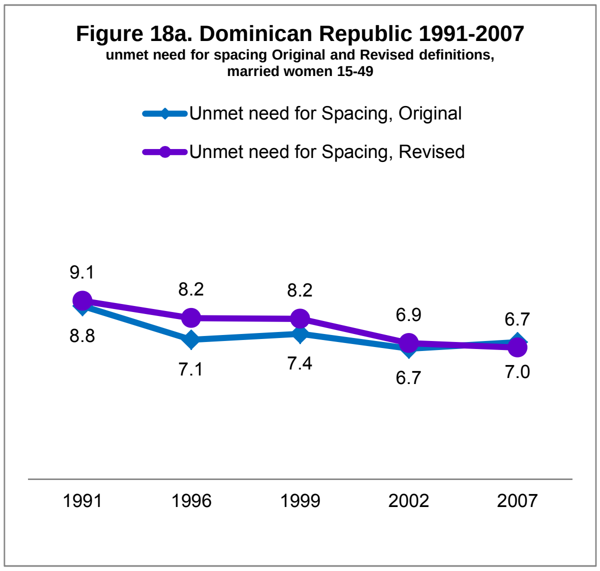
Figure 18a. Dominican Republic 1991-2007
unmet need for spacing Original and Revised definitions, married women 15-49
Unmet need for Spacing, Original
Unmet need for Spacing, Revised
9.1
8.2
8.2
6.9
6.7
8.8
7.1
7.4
6.7
7.0
1991
1996
1999
2002
2007

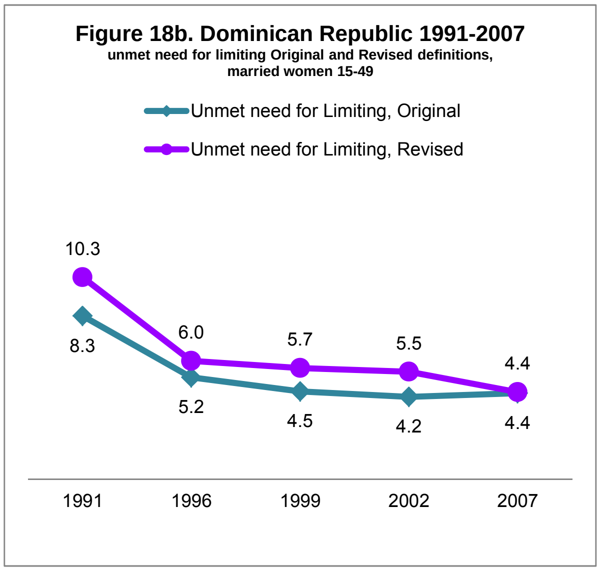
Figure 18b. Dominican Republic 1991-2007
unmet need for limiting Original and Revised definitions, married women 15-49
Unmet need for Limiting, Original
Unmet need for Limiting, Revised
10.3
6.0
5.7
5.5
4.4
8.3
5.2
4.5
4.2
4.4
1991
1996
1999
2002
2007

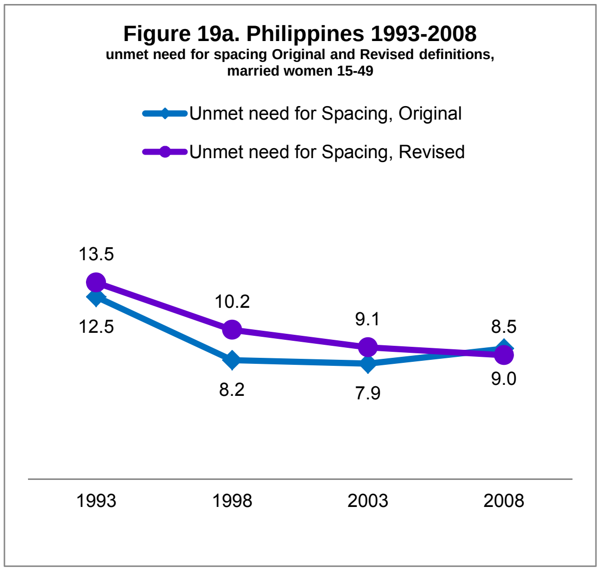
Figure 19a. Philippines 1993-2008
unmet need for spacing Original and Revised definitions, married women 15-49
Unmet need for Spacing, Original
Unmet need for Spacing, Revised
13.5
10.2
9.1
8.5
12.5
8.2
7.9
9.0
1993
1998
2003
2008

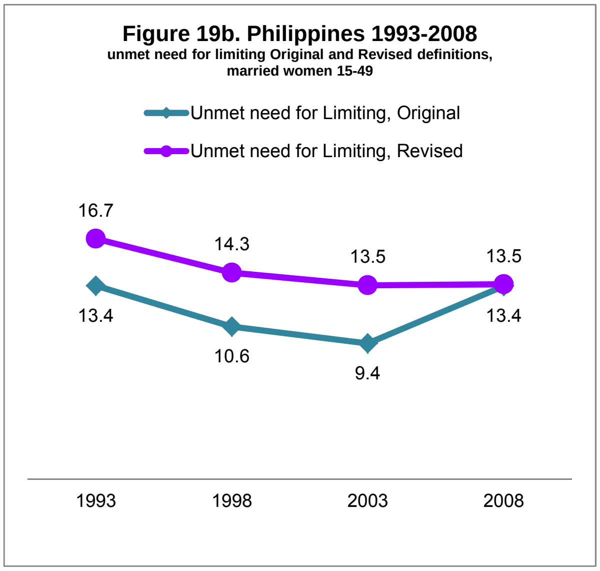
Figure 19b. Philippines 1993-2008
unmet need for limiting Original and Revised definitions, married women 15-49
Unmet need for Limiting, Original
Unmet need for Limiting, Revised
16.7
14.3
13.5
13.5
13.4
10.6
9.4
13.4
1993
1998
2003
2008

4

New Estimates of Unmet Need and Demand for Family Planning Using the Revised Definition

In this section, we examine estimates of unmet need using the **Revised** definition in greater detail. First we use unmet need to calculate total demand for family planning and the proportion of demand satisfied, overall and by modern contraceptive methods. Next, we disaggregate unmet need by background characteristics: urban-rural residence, education, household wealth, parity, and age. Finally, we present estimates of unmet need for sexually active unmarried women.

4.1 Demand for Family Planning and Proportion of Demand Satisfied

We follow the convention of referring to the percentage of women with an unmet need plus the percentage currently using contraception (representing “met need”) as *demand for family planning*, and referring to the percentage of women using contraception divided by the percentage of women with demand for family planning as the *proportion of demand satisfied* (see box, below). The indicator *proportion of demand satisfied* is useful in assessing overall levels of coverage for family planning programs. As levels of contraceptive use increase, the proportion of demand satisfied increases. In contrast, levels of unmet need can either rise or fall with changes in contraceptive use and desired family size and spacing. For example, if contraceptive use increases and desired family size stays the same, unmet need will generally decrease. But if contraceptive use increases at the same time that desired family size decreases, the level of unmet need may go up, making it difficult to assess the progress of family planning programs.

Calculation of unmet need for family planning and proportion of demand satisfied can also be modified to focus on modern contraceptive methods, rather than any contraceptive use, by using the *proportion of demand satisfied by modern methods* as an indicator. In this indicator, women who are using a traditional method of family planning are considered to have an unmet need for a better (modern) contraceptive method.

**Definitions of demand for family planning and the proportion of demand satisfied**	
Demand for Family Planning:	Unmet need for family planning<br>+ current contraceptive use (any method)
Proportion of demand satisfied:	$\frac{\text{Current contraceptive use (any method)}}{\text{Unmet need + current contraceptive use (any method)}}$
Proportion of demand satisfied by modern methods:	$\frac{\text{Current contraceptive use (modern methods)}}{\text{Unmet need + current contraceptive use (any method)}}$

Table 4. Unmet need, contraceptive use, and demand for family planning, currently married women

Unmet need, current contraceptive use, total demand, proportion of demand satisfied, and proportion of demand satisfied by modern methods among currently married women 15-49 using the Revised definition of unmet need, DHS surveys 1990-2010

Survey Year	Unmet need	Current use	Total demand	Proportion of total demand satisfied	Unmet need for modern methods	Using modern methods[1]	Proportion of total demand satisfied by modern methods
**West and Central Africa**							
Benin 1996	27.7	16.4	44.1	37.2	40.6	3.4	7.8
Benin 2001	27.9	18.6	46.5	40.0	39.3	7.2	15.4
Benin 2006	27.3	17.0	44.3	38.4	38.2	6.2	13.9
Burkina Faso 1993	24.6	24.9	49.5	50.3	45.3	4.2	8.5
Burkina Faso 1998-99	30.3	11.9	42.2	28.2	37.3	4.8	11.5
Burkina Faso 2003	29.8	13.8	43.6	31.6	34.8	8.8	20.1
Cameroon 1991	22.4	16.1	38.4	41.8	34.1	4.3	11.1
Cameroon 1998	20.7	19.3	40.1	48.3	33.0	7.1	17.7
Cameroon 2004	20.5	26.0	46.5	55.9	33.4	13.0	28.1
Central African Republic 1994-95	19.1	14.8	33.8	43.6	30.6	3.2	9.6
Chad 1996-97	17.4	4.1	21.6	19.2	20.4	1.2	5.4
Chad 2004	20.6	11.1	31.6	35.0	21.7	9.9	31.4
Congo (Brazzaville) 2005	19.5	44.3	63.8	69.4	51.1	12.7	19.8
Congo Democratic Republic 2007	26.9	20.6	47.6	43.4	41.8	5.8	12.2
Cote D'Ivoire 1994	30.4	11.4	41.8	27.3	37.5	4.3	10.3
Cote D'Ivoire 1998-99	28.9	15.0	43.9	34.2	36.7	7.3	16.5
Gabon 2000	27.9	32.7	60.6	54.0	47.2	13.4	22.1
Ghana 1993	36.9	20.3	57.1	35.4	47.0	10.1	17.7
Ghana 1998	34.7	22.0	56.7	38.7	43.4	13.3	23.5
Ghana 2003	34.5	25.2	59.7	42.2	41.0	18.7	31.3
Ghana 2008	35.7	23.5	59.2	39.7	42.6	16.6	28.0
Guinea 1999	24.8	6.2	31.0	20.1	26.8	4.2	13.6
Guinea 2005	21.9	9.1	31.0	29.3	25.4	5.7	18.3
Liberia 2007	35.7	11.4	47.1	24.3	36.8	10.3	21.8
Mali 1995-96	27.5	6.7	34.2	19.7	29.7	4.5	13.1
Mali 2001	29.6	8.1	37.7	21.4	30.7	7.0	18.5
Mali 2006	27.6	8.2	35.8	23.0	29.0	6.9	19.2
Mauritania 2000-01	32.1	8.0	40.1	19.9	34.6	5.4	13.6
Niger 1992	18.7	4.4	23.1	19.2	20.8	2.3	9.8
Niger 1998	17.7	8.2	25.9	31.7	21.3	4.6	17.8
Niger 2006	16.1	11.2	27.3	41.1	17.6	9.7	35.4
Nigeria 1990	21.5	6.0	27.6	21.9	24.0	3.5	12.9
Nigeria 1999	20.0	15.3	35.3	43.4	26.8	8.6	24.2
Nigeria 2003	17.5	12.6	30.1	41.8	21.8	8.2	27.4
Nigeria 2008	20.2	14.6	34.8	41.9	25.1	9.7	27.8
Sao Tome and Principe 2008-09	37.6	38.4	76.0	50.5	42.3	33.7	44.3
Senegal 1992-93	28.8	7.5	36.3	20.6	31.5	4.8	13.2
Senegal 2005	32.0	11.8	43.8	27.0	33.5	10.3	23.5
Sierra Leone 2008	28.4	8.2	36.6	22.4	29.8	6.7	18.4
Togo 1998	35.0	23.5	58.4	40.2	51.5	7.0	11.9
**East and Southern Africa**							
Comoros 1996	35.6	21.0	56.5	37.1	45.2	11.4	20.1
Eritrea 1995	29.7	8.0	37.6	21.1	33.6	4.0	10.6

(Continued…)

Table 4 – *Continued*

Survey Year	Unmet need	Current use	Total demand	Proportion of total demand satisfied	Unmet need for modern methods	Using modern methods[1]	Proportion of total demand satisfied by modern methods
Eritrea 2002	28.5	8.0	36.5	22.0	29.3	7.3	19.9
Ethiopia 2000	36.6	8.1	44.6	18.1	38.3	6.3	14.2
Ethiopia 2005	36.1	14.7	50.8	29.0	36.9	13.9	27.4
Kenya 1993	35.3	32.7	68.1	48.1	40.8	27.3	40.1
Kenya 1998	28.0	39.0	67.0	58.2	35.5	31.5	47.0
Kenya 2003	27.4	39.3	66.7	58.9	35.2	31.5	47.3
Kenya 2008-09	25.6	45.5	71.1	63.9	31.7	39.4	55.5
Lesotho 2004	31.0	37.3	68.3	54.6	33.1	35.2	51.5
Lesotho 2009	23.3	47.0	70.3	66.9	24.7	45.6	64.9
Madagascar 1992	32.3	16.7	49.0	34.1	43.9	5.1	10.5
Madagascar 1997	27.7	19.4	47.1	41.3	37.4	9.7	20.7
Madagascar 2003-04	24.5	27.1	51.6	52.6	33.3	18.3	35.5
Madagascar 2008-09	19.0	39.9	58.9	67.8	29.7	29.2	49.5
Malawi 1992	36.5	13.0	49.5	26.3	42.2	7.4	14.9
Malawi 2000	29.9	30.6	60.6	50.6	34.5	26.1	43.1
Malawi 2004	30.3	32.5	62.8	51.7	34.6	28.1	44.8
Malawi 2010	26.2	46.1	72.3	63.8	30.1	42.2	58.4
Mozambique 1997	24.9	5.6	30.5	18.4	25.5	5.1	16.6
Mozambique 2003	18.9	25.5	44.4	57.5	23.6	20.8	46.8
Namibia 1992	21.8	28.9	50.6	57.1	24.6	26.0	51.4
Namibia 2000	23.9	43.7	67.6	64.7	25.0	42.6	63.1
Namibia 2006-07	20.7	55.1	75.8	72.7	22.3	53.4	70.5
Rwanda 1992	38.2	21.2	59.4	35.7	46.5	12.9	21.7
Rwanda 2000	36.4	13.2	49.6	26.7	44.0	5.7	11.4
Rwanda 2005	38.5	17.4	55.8	31.1	45.6	10.3	18.4
South Africa 1998	16.5	56.3	72.8	77.3	17.7	55.1	75.7
Swaziland 2006-07	24.7	50.6	75.4	67.2	27.7	47.7	63.3
Tanzania 1991-92	27.8	10.4	38.2	27.3	31.6	6.6	17.2
Tanzania 1996	26.0	18.4	44.4	41.5	31.1	13.3	30.0
Tanzania 1999	22.3	25.4	47.7	53.2	28.9	18.7	39.3
Tanzania 2004-05	24.3	26.4	50.6	52.1	30.6	20.0	39.5
Tanzania 2010	25.3	34.4	59.7	57.6	32.3	27.4	45.9
Uganda 1995	30.0	14.8	44.8	33.1	37.0	7.8	17.4
Uganda 2000-01	35.0	22.8	57.8	39.4	39.6	18.2	31.5
Uganda 2006	38.0	23.7	61.7	38.4	43.8	17.9	29.0
Zambia 1992	30.0	15.2	45.2	33.6	36.3	8.9	19.6
Zambia 1996	25.2	25.9	51.1	50.7	36.7	14.4	28.1
Zambia 2001-02	27.5	34.2	61.7	55.4	36.4	25.3	41.0
Zambia 2007	26.6	40.8	67.4	60.5	34.7	32.7	48.5
Zimbabwe 1994	19.1	48.1	67.2	71.6	25.0	42.2	62.7
Zimbabwe 1999	16.7	53.5	70.3	76.2	19.9	50.4	71.6
Zimbabwe 2005-06	15.5	60.2	75.7	79.6	17.3	58.4	77.2
**Middle East/North Africa**							
Egypt 1992	22.9	47.1	70.0	67.3	25.2	44.8	64.0
Egypt 1995	20.2	47.9	68.1	70.3	22.6	45.5	66.8
Egypt 2000	13.7	56.1	69.8	80.4	15.9	53.9	77.2
Egypt 2003	11.8	60.0	71.8	83.6	15.2	56.6	78.8
Egypt 2005	12.3	59.2	71.5	82.8	15.0	56.5	79.0

(Continued…)

Table 4 – *Continued*

Survey Year	Unmet need	Current use	Total demand	Proportion of total demand satisfied	Unmet need for modern methods	Using modern methods[1]	Proportion of total demand satisfied by modern methods
Egypt 2008	11.6	60.3	71.9	83.9	14.3	57.6	80.1
Jordan 1990	26.5	40.0	66.4	60.2	39.6	26.9	40.4
Jordan 1997	20.1	52.6	72.7	72.4	35.0	37.7	51.9
Jordan 2002	14.9	55.8	70.7	78.9	29.5	41.2	58.3
Jordan 2007	13.8	57.1	70.9	80.5	29.0	41.9	59.1
Jordan 2009	13.4	59.3	72.6	81.6	30.6	42.0	57.9
Morocco 1992	23.5	41.5	65.0	63.9	29.4	35.5	54.7
Morocco 2003-04	11.9	63.0	74.9	84.1	20.1	54.8	73.2
**Eastern Europe/NIS**							
Albania 2008-09	12.9	69.3	82.2	84.4	71.6	10.6	12.9
Armenia 2000	18.1	60.5	78.6	77.0	56.3	22.3	28.3
Armenia 2005	19.3	53.1	72.4	73.3	52.9	19.5	26.9
Azerbaijan 2006	15.4	51.1	66.5	76.8	52.2	14.3	21.5
Kazakhstan 1995	16.3	59.1	75.4	78.4	29.4	46.1	61.1
Kazakhstan 1999	11.9	66.1	77.9	84.8	23.5	54.4	69.8
Kyrgyz Republic 1997	11.8	59.5	71.4	83.4	22.5	48.9	68.5
Moldova 2005	11.4	67.8	79.1	85.6	35.3	43.8	55.4
Turkey 1993	14.6	62.6	77.2	81.1	42.6	34.5	44.7
Turkey 1998	14.0	63.9	77.9	82.0	40.1	37.7	48.5
Turkey 2003	9.5	72.2	81.7	88.4	37.9	43.8	53.6
Ukraine 2007	10.1	66.7	76.8	86.8	29.2	47.5	61.9
Uzbekistan 1996	13.7	55.6	69.3	80.3	18.0	51.3	74.1
**Asia**							
Bangladesh 1993-94	21.6	44.9	66.5	67.5	29.9	36.6	55.0
Bangladesh 1996-97	19.7	49.8	69.5	71.7	27.4	42.1	60.6
Bangladesh 1999-2000	18.2	54.3	72.5	74.9	28.5	44.0	60.6
Bangladesh 2004	15.0	58.5	73.5	79.5	25.9	47.6	64.8
Bangladesh 2007	16.8	55.8	72.6	76.9	25.1	47.5	65.4
Cambodia 2000	33.0	23.8	56.9	41.9	38.1	18.8	33.0
Cambodia 2005	25.3	40.0	65.3	61.3	38.1	27.2	41.6
Cambodia 2010	16.9	50.5	67.5	74.9	32.6	34.9	51.7
India 1992-93	20.3	40.7	61.0	66.7	24.6	36.5	59.7
India 1998-99	16.1	48.2	64.3	75.0	21.5	42.8	66.6
India 2005-06	13.9	56.3	70.2	80.2	21.7	48.5	69.1
Indonesia 1991	17.0	49.7	66.7	74.5	19.7	47.1	70.5
Indonesia 1994	15.3	54.7	70.1	78.1	18.0	52.1	74.3
Indonesia 1997	13.6	57.4	71.0	80.9	16.3	54.7	77.1
Indonesia 2002-03	13.2	60.3	73.6	82.0	16.9	56.7	77.1
Indonesia 2007	13.1	61.4	74.5	82.4	17.1	57.4	77.0
Maldives 2009	28.6	34.7	63.4	54.8	36.4	27.0	42.6
Nepal 1996	32.4	28.5	60.8	46.8	34.8	26.0	42.8
Nepal 2001	27.8	39.3	67.1	58.6	31.7	35.4	52.8
Nepal 2006	24.7	48.0	72.6	66.0	28.4	44.2	60.9
Pakistan 1990-91	30.5	11.8	42.3	28.0	33.3	9.0	21.3
Pakistan 2006-07	25.2	29.6	54.8	54.1	33.0	21.7	39.7
Philippines 1993	30.2	40.0	70.1	57.0	45.3	24.9	35.4
Philippines 1998	24.6	47.8	72.4	66.1	44.2	28.2	39.0
Philippines 2003	22.5	48.9	71.4	68.5	38.0	33.4	46.7

(Continued…)

Table 4 – *Continued*

Survey Year	Unmet need	Current use	Total demand	Proportion of total demand satisfied	Unmet need for modern methods	Using modern methods[1]	Proportion of total demand satisfied by modern methods
Philippines 2008	22.0	50.7	72.7	69.7	38.7	34.0	46.8
Samoa 2009	47.7	28.7	76.4	37.5	49.7	26.7	35.0
Timor-Leste 2009	31.5	22.3	53.8	41.5	32.7	21.1	39.2
Vietnam 1997	8.4	75.3	83.7	90.0	27.9	55.8	66.7
Vietnam 2002	6.6	78.5	85.2	92.2	28.5	56.7	66.6
**Latin America and Caribbean**							
Bolivia 1994	28.9	45.3	74.2	61.1	56.4	17.8	24.0
Bolivia 1998	26.6	48.3	74.9	64.5	49.7	25.2	33.7
Bolivia 2003	22.8	58.4	81.2	71.9	46.3	34.9	43.0
Bolivia 2008	20.1	60.6	80.8	75.1	46.2	34.6	42.8
Brazil 1996	10.8	76.7	87.6	87.6	17.3	70.3	80.2
Colombia 1990	13.7	66.1	79.8	82.8	25.2	54.6	68.4
Colombia 1995	11.4	72.2	83.6	86.4	24.2	59.3	71.0
Colombia 2000	10.0	76.9	87.0	88.5	23.0	64.0	73.6
Colombia 2005	8.6	78.2	86.8	90.1	18.7	68.2	78.5
Colombia 2010	8.0	79.1	87.1	90.8	14.1	72.9	83.8
Dominican Republic 1991	19.4	56.4	75.8	74.4	24.1	51.7	68.2
Dominican Republic 1996	14.3	63.7	77.9	81.7	18.5	59.5	76.3
Dominican Republic 1999	13.8	69.2	83.1	83.3	19.0	64.1	77.1
Dominican Republic 2002	12.4	69.8	82.2	84.9	16.4	65.8	80.1
Dominican Republic 2007	11.1	72.9	84.0	86.7	14.0	70.0	83.3
Guatemala 1995	28.1	31.4	59.5	52.8	32.6	26.9	45.2
Guatemala 1998-99	26.8	38.2	65.1	58.7	34.2	30.9	47.5
Guyana 2009	28.5	42.5	70.9	59.9	31.0	40.0	56.3
Haiti 1994-95	44.7	18.0	62.6	28.7	49.4	13.2	21.1
Haiti 2000	39.6	28.1	67.7	41.5	44.8	22.8	33.8
Haiti 2005-06	37.3	32.0	69.4	46.2	44.5	24.8	35.8
Honduras 2005-06	16.8	65.2	82.0	79.5	25.7	56.4	68.7
Nicaragua 1998	17.9	60.3	78.2	77.2	20.8	57.4	73.4
Nicaragua 2001	14.6	68.6	83.3	82.4	17.1	66.1	79.4
Paraguay 1990	17.4	48.4	65.8	73.5	30.6	35.2	53.5
Peru 1991-92	21.6	59.0	80.6	73.2	47.8	32.8	40.7
Peru 1996	17.7	64.2	81.9	78.4	40.6	41.3	50.5
Peru 2000	14.4	68.9	83.3	82.7	32.8	50.4	60.6
Peru 2004-08	12.4	72.0	84.4	85.3	36.1	48.3	57.2
**Unweighted Averages**							
West and Central Africa	26.4	15.7	42.1	35.3	33.9	8.2	18.7
East and Southern Africa	27.7	29.5	57.2	48.7	33.0	24.1	38.9
Middle East/North Africa	16.7	53.8	70.5	76.1	24.7	45.8	64.7
Eastern Europe/NIS	13.8	62.1	75.9	81.7	39.4	36.5	48.3
Asia	21.9	46.4	68.3	66.4	30.4	38.0	54.5
Latin America and Caribbean	19.6	58.3	77.9	73.4	31.1	46.9	58.9
**Total**	**23.1**	**38.5**	**61.6**	**57.6**	**32.3**	**29.3**	**43.0**

[1] Includes LAM which has been excluded as a modern method in some DHS final reports

4.1.1 Trends in the Proportion of Demand Satisfied

Total demand for family planning varies from 22 percent in the Chad 1996-97 DHS survey to 88 percent in Brazil 1996, and averages 62 percent (unweighted) across the 169 surveys analyzed. The proportion of demand satisfied ranges from 18 percent in Ethiopia 2000 to 92 percent in Vietnam 2002.

In the majority of surveys the proportion of demand satisfied increases over time. Madagascar and Jordan are examples of this pattern. In Madagascar the proportion of demand satisfied doubled, from 34 percent in 1992 to 68 percent in 2008-09 (Figure 20a). Total demand increased by just 10 percentage points, from 49 to 59 percent, while contraceptive prevalence (CPR) increased from 17 to 40 percent, leading to a reduction in unmet need, from 32 percent in 1992 to 19 percent in 2008-09 (Figure 20b).

Jordan also shows a similar—albeit less steep—increase in the proportion of demand satisfied, from 60 to 82 percent between 1990 and 2009 (Figure 21a), with an increase in CPR from 40 to 59 percent and a reduction in unmet need from 27 to 13 percent over this period (Figure 21b).

In Tanzania the proportion of demand satisfied increased from 27 percent in 1992 to 58 percent in 2010 (Figure 22a), but followed a different pattern than in Madagascar and Jordan. Total demand increased from 38 to 60 percent, and CPR tripled from 10 percent to 34 percent (Figure 22b). However, unmet need has not decreased consistently and declined only slightly over the entire period, from 28 percent in 1992 to 25 percent in 2010. Generally, the picture is positive for family planning, with total demand increasing and the proportion of demand satisfied also increasing. This example highlights the problems of relying solely on estimates of unmet need for monitoring family planning programs and polices, and demonstrates the need to assess unmet need in conjunction with both contraceptive prevalence and the proportion of demand satisfied.

Although no country shows a consistent decrease in the proportion of demand satisfied, in some countries the proportion has decreased between two successive surveys. Examples of recent decreases in the proportion of demand satisfied are Armenia (from 77 percent in 2000 to 73 percent in 2005), Bangladesh (80 percent in 2004 to 77 percent in 2007), and Ghana (42 percent in 2003 to 40 percent in 2008), but all of these differences are small[11]. Rwanda is an exception, where the proportion of demand satisfied dropped from 36 percent in 1992 to 27 percent in 2000 (presumably due in no small part to the 1994 genocide), but this decline was followed by an increase to 31 percent in 2005.

4.1.2 The Proportion of Demand Satisfied by Modern Methods

The proportion of demand satisfied by modern contraceptive methods is much lower than the proportion of demand satisfied by all methods, at an average of 43 percent for modern methods compared with an average of 58 percent for all methods. The proportion of demand satisfied by modern methods ranges from 5 percent in the Chad 2004 survey to 84 percent in Colombia 2010. Where traditional methods are a large part of the contraceptive method mix, the proportion of demand satisfied by modern methods is substantially lower than the proportion satisfied by any method. In Albania 2008-09, for example, 13 percent of demand is satisfied by modern methods compared with 84 percent for by method, and in Congo (Brazzaville) 20 percent of demand is satisfied by modern methods compared with 69 percent satisfied by any method of contraception.

[11] A steep decline in the proportion of demand satisfied is seen between the Burkina Faso 1993 survey and the 1998-99 survey, but this is largely due to high reporting of long-term abstinence (17 percent) as a contraceptive method in 1993 and lower reporting (2 percent) in 1998-99. Recalculating CPR and unmet need ignoring long-term abstinence as a current contraceptive method would eliminate this difference.

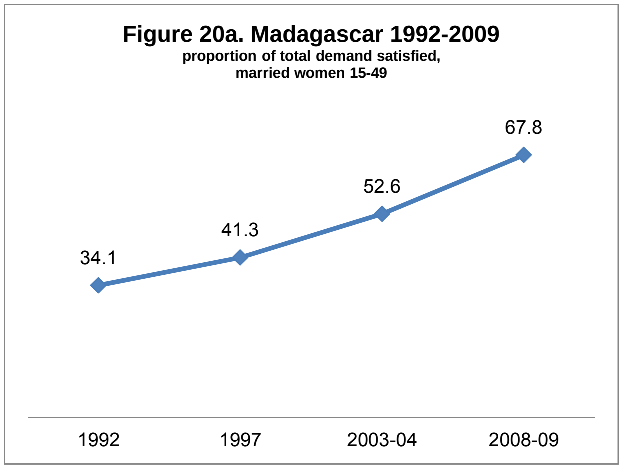
Figure 20a. Madagascar 1992-2009
proportion of total demand satisfied,
married women 15-49
34.1
41.3
52.6
67.8
1992
1997
2003-04
2008-09

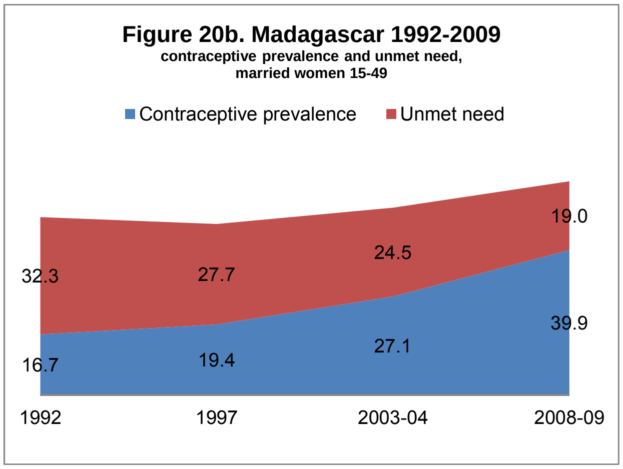
Figure 20b. Madagascar 1992-2009
contraceptive prevalence and unmet need,
married women 15-49
Contraceptive prevalence
Unmet need
32.3
27.7
24.5
19.0
16.7
19.4
27.1
39.9
1992
1997
2003-04
2008-09

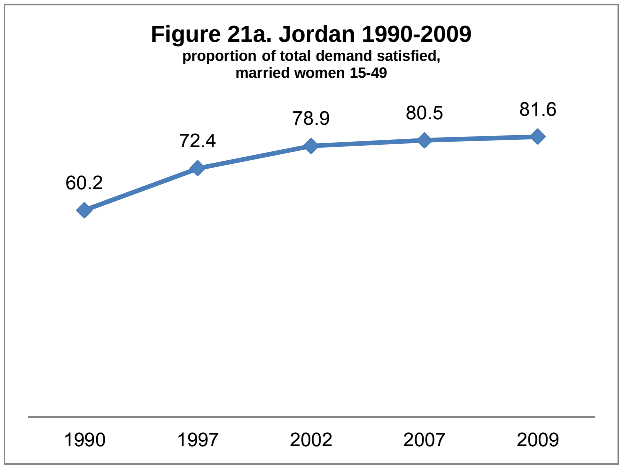
Figure 21a. Jordan 1990-2009
proportion of total demand satisfied,
married women 15-49
60.2
72.4
78.9
80.5
81.6
1990
1997
2002
2007
2009

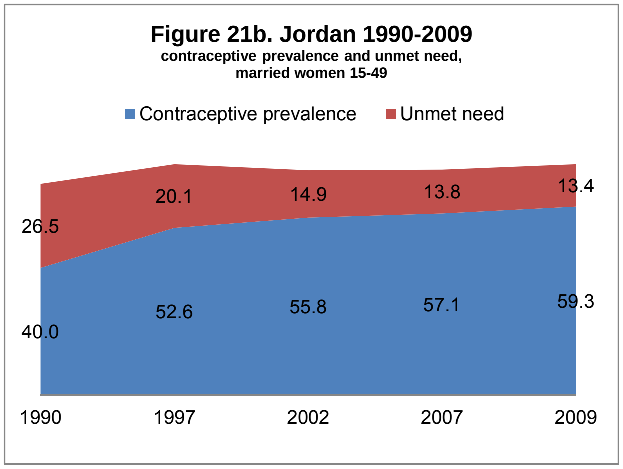
Figure 21b. Jordan 1990-2009
contraceptive prevalence and unmet need,
married women 15-49
Contraceptive prevalence
Unmet need
26.5
20.1
14.9
13.8
13.4
40.0
52.6
55.8
57.1
59.3
1990
1997
2002
2007
2009

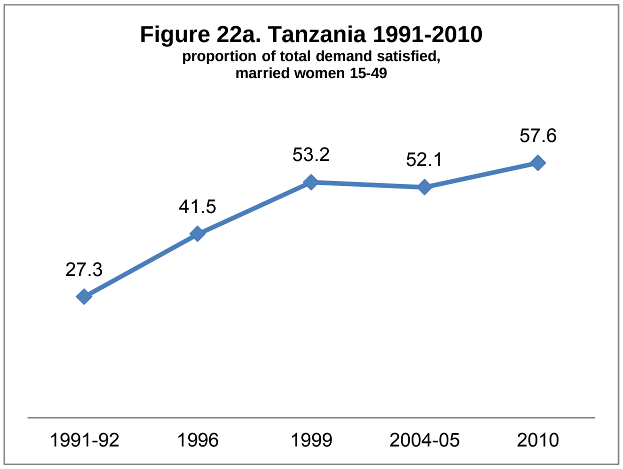
Figure 22a. Tanzania 1991-2010
proportion of total demand satisfied,
married women 15-49
27.3
41.5
53.2
52.1
57.6
1991-92
1996
1999
2004-05
2010

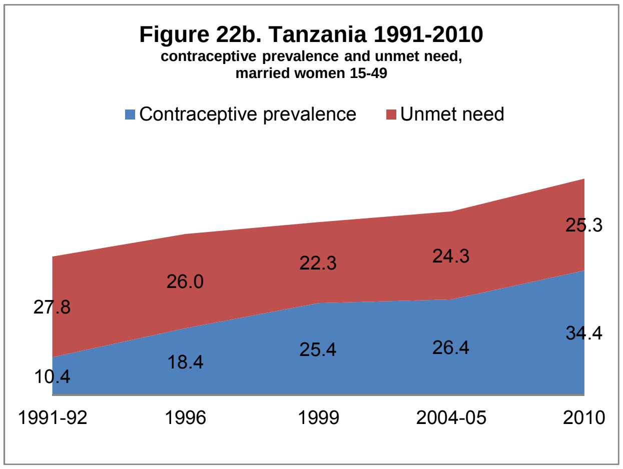
Figure 22b. Tanzania 1991-2010
contraceptive prevalence and unmet need,
married women 15-49
Contraceptive prevalence
Unmet need
27.8
26.0
22.3
24.3
25.3
10.4
18.4
25.4
26.4
34.4
1991-92
1996
1999
2004-05
2010

4.2 Unmet Need by Background Characteristics

Using the **Revised** definition, we present estimates of total unmet need by women's background characteristics—urban-rural residence, education, wealth, parity, and age—for the most recent DHS survey conducted since 2000. This analysis covers 59 countries; countries in which the only surveys were pre-2000 have been excluded. Figures exemplifying each pattern are shown below; estimates for each country are shown in Appendix Tables 1 and 2.

Overall, relationships between unmet need and background characteristics are similar regardless of whether the **Revised** or **Original** definition is used. Unmet need does not always follow a consistent pattern with background characteristics, but when there is a relationship, unmet need tends to be highest among women in rural areas, those with no formal education, and those in the poorest wealth quintiles. Unmet need for spacing is usually highest among the youngest women and those with few children and decreases as age and parity increase, while unmet need for limiting generally increases with age and parity. There are exceptions to these generalizations for several countries.

4.2.1 Urban-Rural Residence

Using the **Revised** definition, in the majority of countries outside West and Central Africa, unmet need is higher in rural than urban areas (Appendix Table 1). Most countries follow the patterns of Kenya, Egypt, and Albania (Figures 23 through 25), with substantially higher levels of unmet need in rural than in urban areas. There are a few exceptions to this pattern: in Mozambique, Moldova, Guyana (Figures 26 through 28), Samoa, and the Dominican Republic, unmet need is the same or slightly higher in urban than rural areas.

Within West and Central Africa, there is no clear pattern in unmet need by urban-rural residence. For example, in Sierra Leone (Figure 29), levels of unmet need are virtually identical in urban and rural areas, while in Niger (Figure 30) unmet need is much higher in urban than rural areas. In Ghana (Figure 31), unmet need is higher in rural areas.

The (nearly) consistent increase in unmet need in rural areas in countries outside West and Central Africa, and the lack of a consistent pattern within West and Central Africa, replicate Westoff's findings in 2006 using the **Original** definition (Westoff 2006).

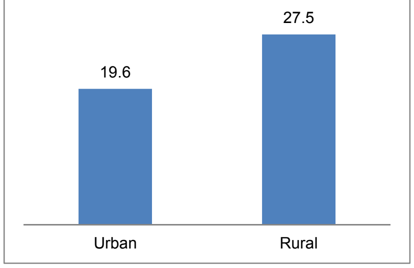

Figure 23. Kenya 2008-09
unmet need by residence, married women 15-49

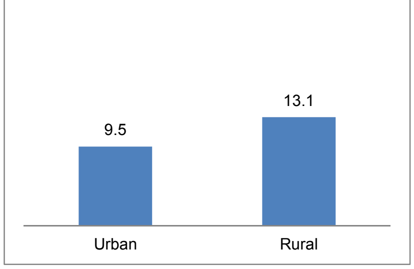

Figure 24. Egypt 2008
unmet need by residence, married women 15-49

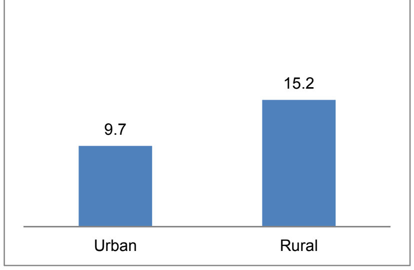

Figure 25. Albania 2008-09
unmet need by residence, married women 15-49

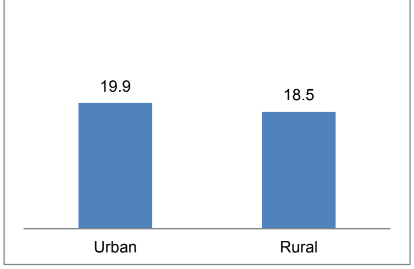

Figure 26. Mozambique 2003
unmet need by residence, married women 15-49

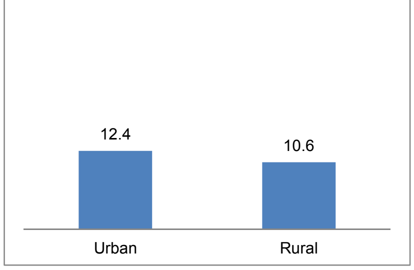

Figure 27. Moldova 2005
unmet need by residence, married women 15-49

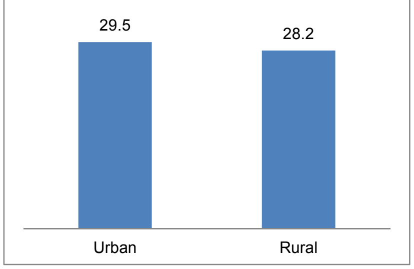

Figure 28. Guyana 2009
unmet need by residence, married women 15-49

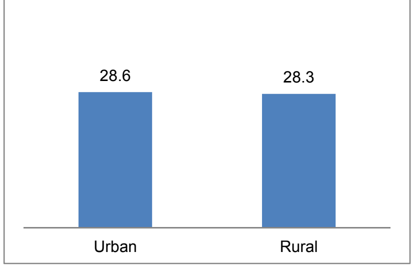

Figure 29. Sierra Leone 2008
unmet need by residence, married women 15-49

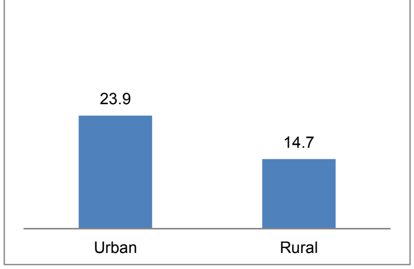

Figure 30. Niger 2006
unmet need by residence, married women 15-49

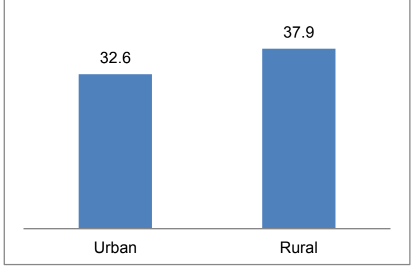

Figure 31. Ghana 2008
unmet need by residence, married women 15-49

4.2.2 Education

In the majority of countries analyzed, unmet need decreases as women’s education increases (Appendix Table 1). This inverse relationship between education and unmet need is seen in all of the Middle East/North African countries and most of the Latin American and Caribbean countries. Jordan and Bolivia (Figures 32 and 33) show examples of this pattern of decreasing unmet need with increasing levels of women’s education.

The relationship between unmet need and education is far from consistent, however. In several countries in West and Central Africa, there is an inverted V-shaped relationship, as in Liberia (Figure 34). In these countries unmet need is highest among women with primary education, and lower among women with either no education or a secondary or higher education. In four other countries in the region, the Democratic Republic of the Congo (Figure 35), Guinea, Mali, and Niger, unmet need increases with women’s education. In all four countries, levels of unmet need also are higher among urban women. In these countries, women with more education are more likely to live in urban areas and to have similar levels of unmet need for family planning. In East and Southern Africa, unmet need either follows the inverted V-shape mentioned above (exemplified by Ethiopia, Figure 36) or decreases with education (exemplified by Swaziland, Figure 37).

Patterns in unmet need by women's education are consistent between the **Revised** definition and the **Original** definition used in Westoff 2006. However, the relationship between unmet need and education seems to be changing in some countries. In 2006, Westoff found that unmet need consistently decreased with education in Asia and in Latin America and the Caribbean. His analysis did not include the more recent surveys of Bangladesh 2007, India 2005-6, Maldives 2009, Nepal 2006, the Dominican Republic 2007, and Haiti 2005-06, all of which demonstrate exceptions to this pattern.

Figure 32. Jordan 2009
unmet need by education, married women 15-49

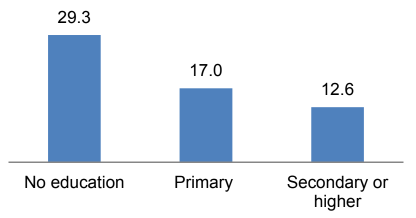

Figure 33. Bolivia 2008
unmet need by education, married women 15-49

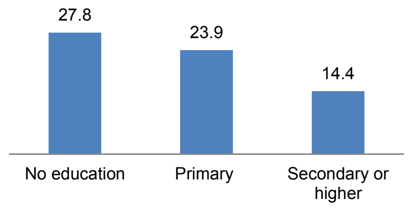

Figure 34. Liberia 2007
unmet need by education, married women 15-49

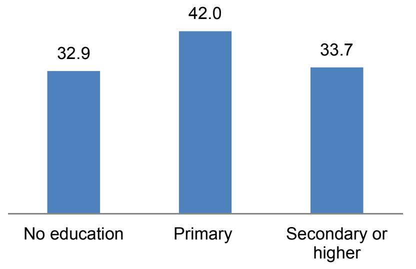

Figure 35. D. R. Congo 2007
unmet need by education, married women 15-49

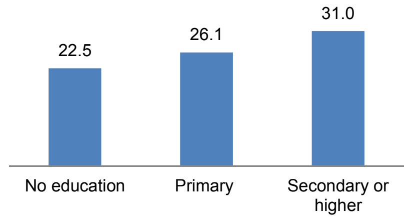

Figure 36. Ethiopia 2005
unmet need by education, married women 15-49

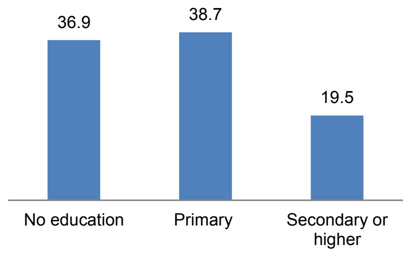

Figure 37. Swaziland 2006-07
unmet need by education, married women 15-49

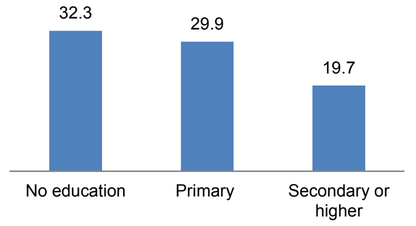

4.2.3 Household Wealth

Unmet need is lowest among women in the wealthiest quintile in almost every survey. Beyond that, the relationship between unmet need and wealth is inconsistent, as can be seen in Appendix Table 1. The most common pattern is that unmet need decreases as wealth increases. Examples of this inverse relationship are seen in Kenya and Haiti (Figures 38 and 39). The same pattern is seen across the majority of surveys in Asia and Latin America, although there is no clear association between unmet need and wealth in several Eastern European/NIS countries, including Armenia and Azerbaijan (Figures 40 and 41).

In a few surveys, instead of an inverse relationship, unmet need increases as wealth increases. Unmet need is highest in the wealthiest quintile in Chad (Figure 42), the Democratic Republic of the Congo,

Mali, and Niger (Figure 43). These surveys also show unusual patterns with other background characteristics described above, and are consistent with Westoff's findings using the **Original** definition of unmet need (Westoff 2006).

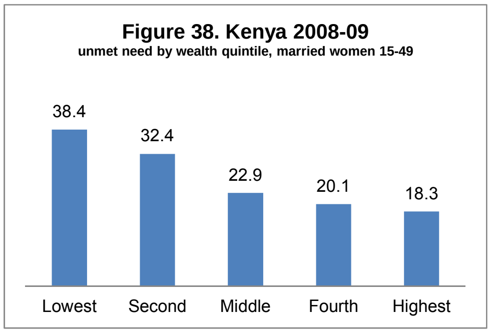

Figure 38. Kenya 2008-09
unmet need by wealth quintile, married women 15-49

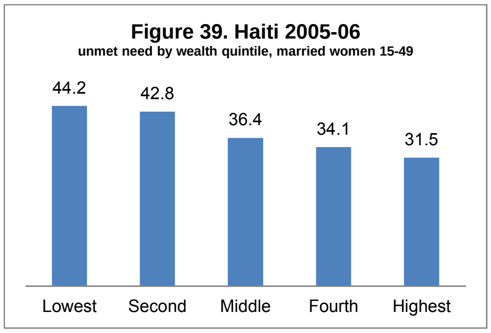

Figure 39. Haiti 2005-06
unmet need by wealth quintile, married women 15-49

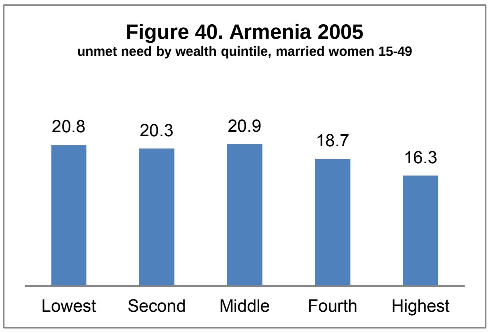

Figure 40. Armenia 2005
unmet need by wealth quintile, married women 15-49

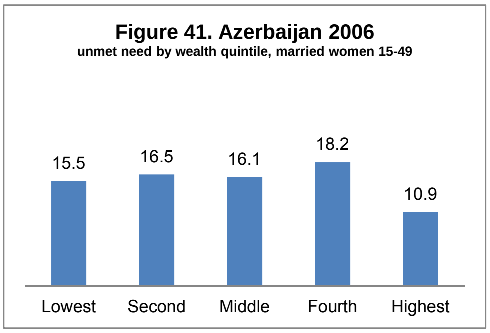

Figure 41. Azerbaijan 2006
unmet need by wealth quintile, married women 15-49

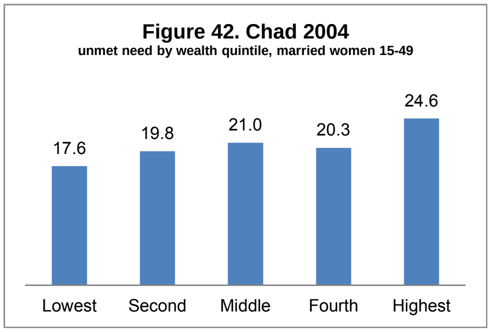

Figure 42. Chad 2004
unmet need by wealth quintile, married women 15-49

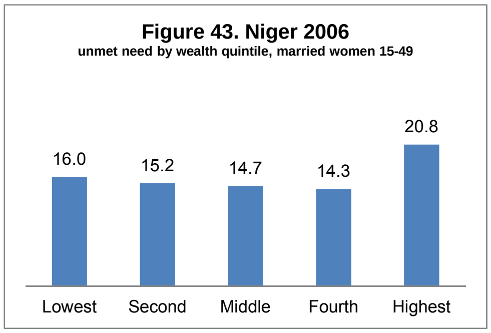

Figure 43. Niger 2006
unmet need by wealth quintile, married women 15-49

4.2.4 Parity and Age

As women have more children, their unmet need for spacing births tends to decrease, while unmet need for limiting increases. These patterns can be examined in detail in Appendix Table 2. Examples are Madagascar, Colombia, Turkey, and Bangladesh (Figures 44 through 47). This pattern of increasing unmet need for limiting and decreasing unmet need for spacing as parity increases is most consistent in surveys within Latin America and the Caribbean.

In almost every survey, unmet need for limiting increases with parity, but the trend is less consistent for unmet need for spacing. Specifically, there is an exception to the pattern for women with no children in countries where marriage is quickly followed by a first birth (note that these tabulations are limited to currently married women). In Middle Eastern/North African, Asian, and sub-Saharan African countries, unmet need for spacing is almost universally lowest for women at parity 0, and does not increase until they have one child. Pakistan and Ghana are examples of this pattern in which unmet need for limiting increases with parity, and unmet need for spacing decreases with parity only after the birth of the first child (Figures 48 and 49).

Figure 44. Madagascar 2008-09
unmet need for spacing and limiting by parity, married women 15-49

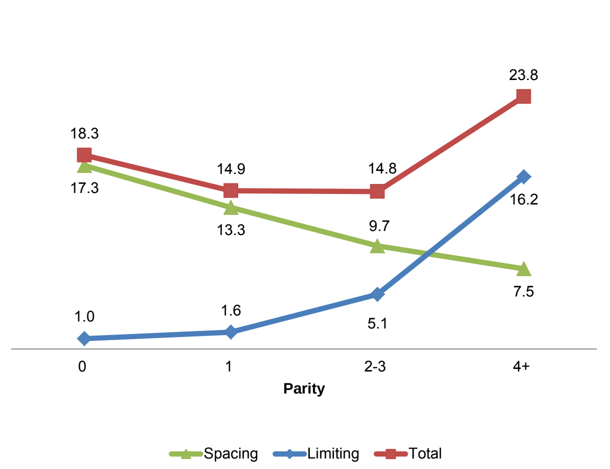

Figure 45. Colombia 2010
unmet need for spacing and limiting by parity, married women 15-49

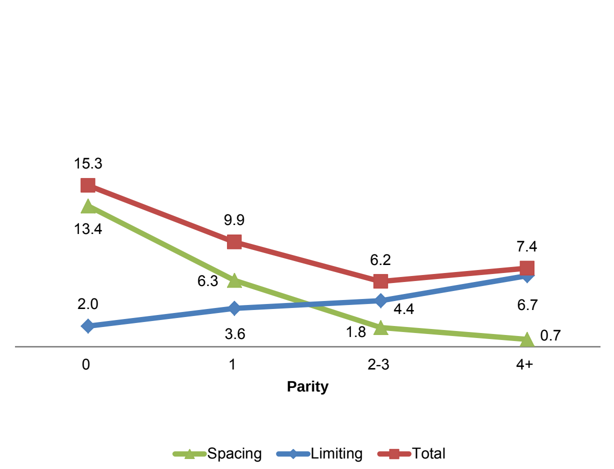

Figure 46. Turkey 2003
unmet need for spacing and limiting by parity, married women 15-49

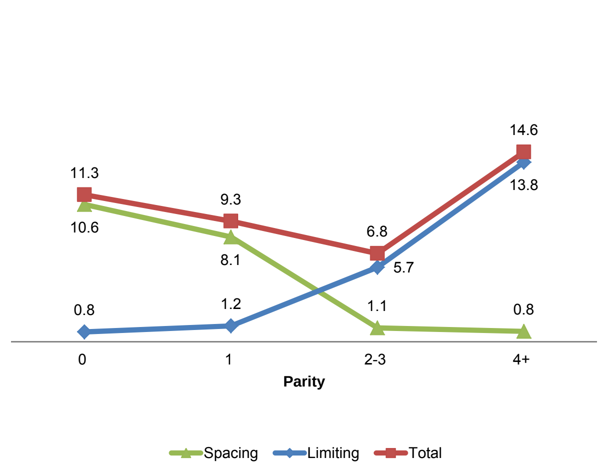

Figure 47. Bangladesh 2007
unmet need for spacing and limiting by parity, married women 15-49

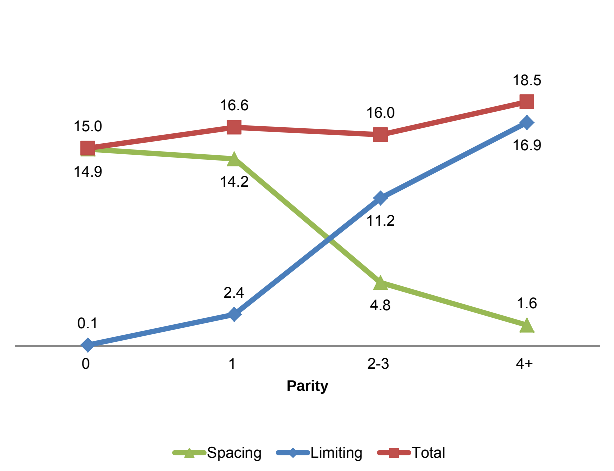

Figure 48. Pakistan 2006-07
unmet need for spacing and limiting by parity, married women 15-49

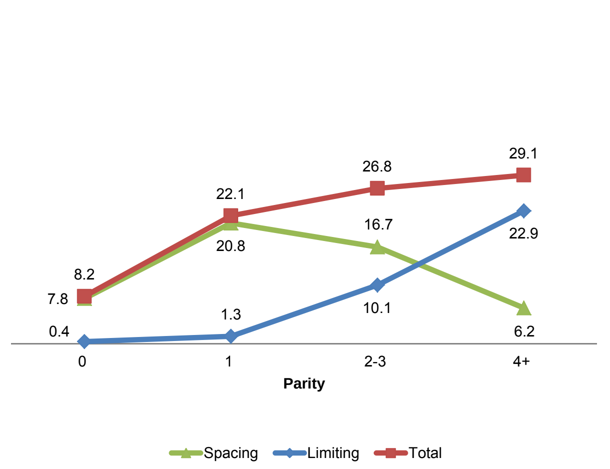

Figure 49. Ghana 2008
unmet need for spacing and limiting by parity, married women 15-49

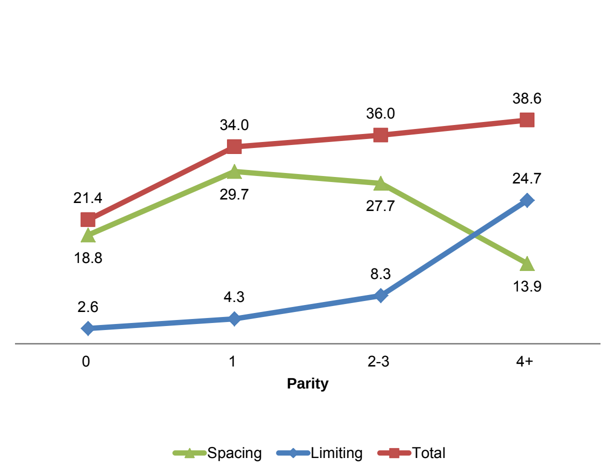

In most countries, patterns for unmet need by parity are similar to those by age because, as would be expected, age and parity are closely linked. In general, most surveys show that unmet need for spacing decreases with age while unmet need for limiting increases, with slightly lower levels of unmet need among the oldest group of women, who have reached menopause, at which point they no longer need family planning at all. These patterns are demonstrated in Haiti and Nepal (Figures 50 and 51).

The relationship between age and unmet need for spacing or for limiting is remarkably consistent across countries. Nearly every survey follows the patterns described above, and exemplified in Haiti and Nepal, albeit at different levels of unmet need. A few exceptions are noted, as in Samoa and Namibia (Figures 52 and 53). The primary reason these two surveys do not follow the usual pattern is that fewer women in the older age groups are categorized as infecund and thus not in need of family planning.

Figure 50. Haiti 2005-06
unmet need for spacing and limiting by age group, married women 15-49

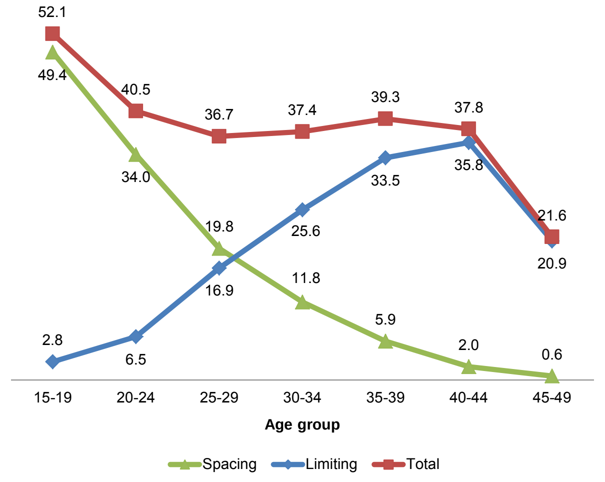

Figure 51. Nepal 2006
unmet need for spacing and limiting by age group, married women 15-49

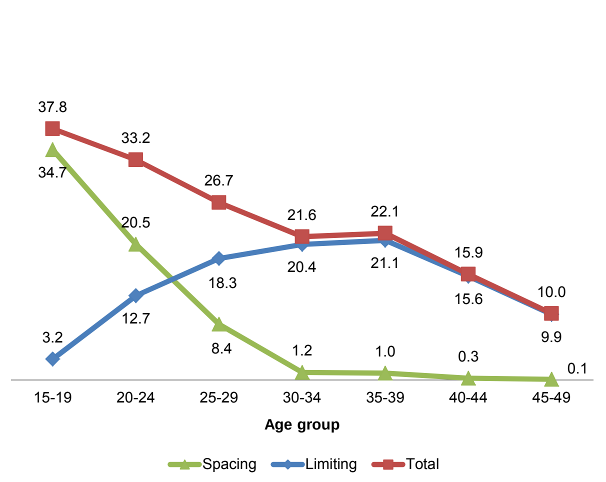

Figure 52. Samoa 2009
unmet need for spacing and limiting by age group, married women 15-49

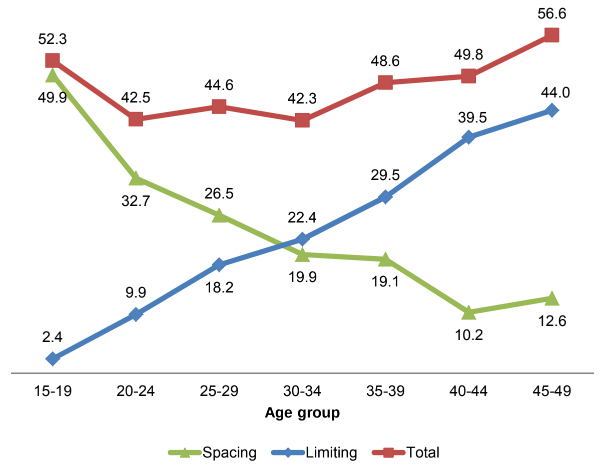

Figure 53. Namibia 2006-07
unmet need for spacing and limiting by age group, married women 15-49

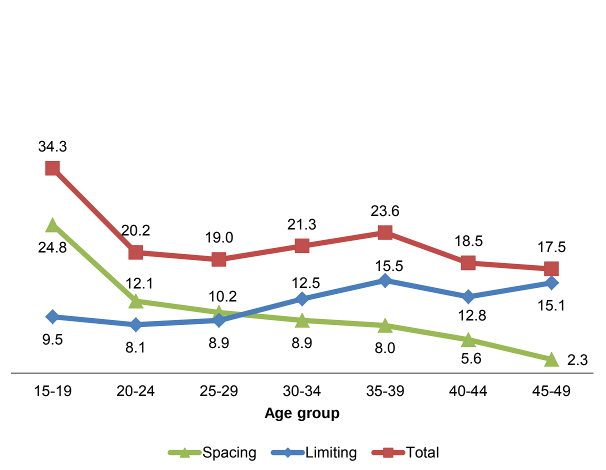

4.3 Unmet Need among Sexually Active Unmarried Women

While unmet need is most commonly measured among currently married women, unmet need can be calculated for unmarried women as well[12]. Here we present estimates of unmet need for sexually active unmarried women, defined as women who are not currently married and who had sexual intercourse in the 30 days preceding the survey. In the **Revised** definition we have followed the assumption in the **Original** definition that unmarried women who are not sexually active do not need family planning. The unmet need algorithm for sexually active unmarried women is the same as the algorithm for currently married women, with one exception. The infecundity criterion “first married five or more years ago, never used contraception, and not had a birth in past five years = infecund” is omitted from the algorithm for sexually active unmarried women.

Table 5 shows levels of unmet need among sexually active unmarried women using the **Revised** definition for the most recent DHS survey conducted in each country since 2000. The table excludes surveys that only interviewed ever-married women and surveys with a sample of fewer than 150 sexually active unmarried women.

Levels of unmet need among sexually active unmarried women should be interpreted relative to the contraceptive prevalence and the total demand for family planning of this group. Unmarried women are less likely to desire a birth than currently married women, and unintended pregnancies may be more problematic, so it is expected that sexually active unmarried women are more motivated than married women to use contraception to prevent pregnancy.

Overall, 59 percent of sexually active unmarried women are using a contraceptive method (unweighted average across the 32 surveys analyzed) compared with 39 percent of currently married women (unweighted average across all 169 surveys analyzed—see Table 4). Unmet need for family planning averages 28 percent for the unmarried group, giving a total demand for family planning of 87 percent. In comparison, average unmet need for currently married women is 23 percent and total demand for family planning is 62 percent. The proportion of demand satisfied for sexually active unmarried women (68 percent) is slightly higher than for currently married women (58 percent).

Among sexually active unmarried women, unmet need is highest in West and Central Africa, at 33 percent, and East and Southern Africa, at 30 percent. Unmet need exceeds 40 percent in Guinea 2005 (42 percent), Liberia 2007 (52 percent), and Sierra Leone 2008 (45 percent). However, the pattern of unmet need differs substantially between the two regions. In all countries in West and Central Africa, more than 80 percent of unmet need is for spacing, while in East and Southern Africa unmet need for spacing accounts for between 39 and 73 percent of total unmet need, and in Lesotho and Swaziland unmet need for limiting exceeds unmet need for spacing (Figure 54). Unmet need is lower in Latin America and the Caribbean (21 percent) and Eastern Europe and the Newly Independent States (15 percent); in both regions more than two-thirds of unmet need is for spacing.[13]

[12] Note that the data needed to calculate unmet need were not collected for unmarried women in DHS II surveys, surveys with ever-married samples, and a small number of more recent surveys.

[13] Estimates for Asia and Middle East/North Africa were not possible due to the exclusion of surveys from those regions because of 1) using an ever-married sample, or 2) having a sample of sexually active unmarried women that was too small to analyze.

Figure 54. Contraceptive prevalence and unmet need for spacing and limiting, sexually active unmarried women 15-49

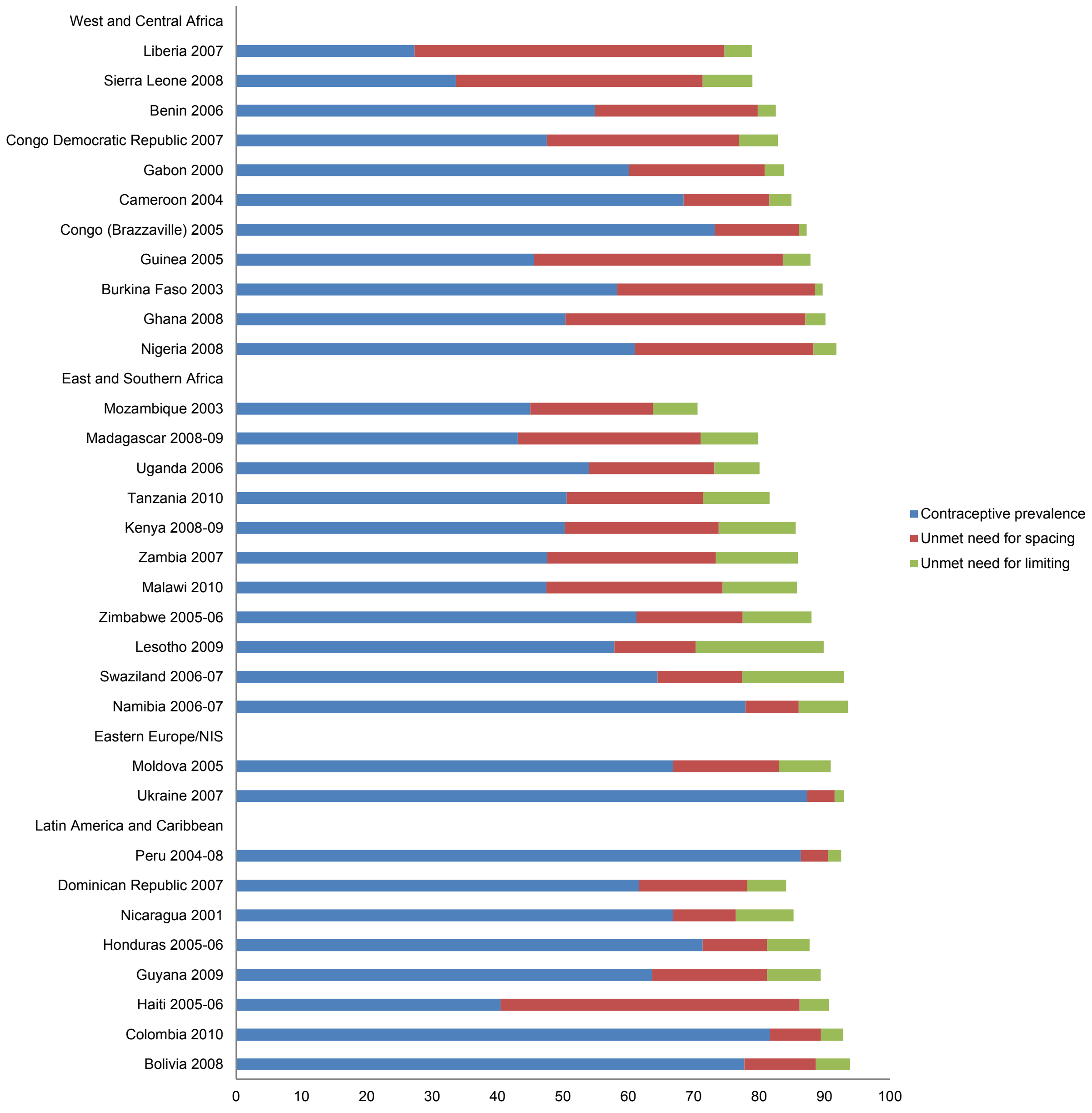

In all but two surveys analyzed, contraceptive prevalence for sexually active unmarried women is higher than for currently married women; only Moldova 2005 (67 versus 68 percent) and the Dominican Republic 2007 (62 versus 73 percent) had a lower CPR for sexually active unmarried women compared to currently married women. In two-thirds of surveys, unmet need is higher for sexually active unmarried women than for currently married women. The difference is greatest in Guinea 2005, where 42 percent of sexually active unmarried women have an unmet need for family planning compared with 9 percent of currently married women.

Table 5. Unmet need, contraceptive use, and demand for family planning, sexually active unmarried women

Current contraceptive use, unmet need (total, spacing and limiting), total demand, and proportion of demand satisfied among sexually active unmarried women age 15-49 using the Revised definition of unmet need, most recent survey from each country, DHS 2000-2010

Survey, Year	Current use	Unmet need total	Unmet need for spacing	Unmet need for limiting	Total demand	Proportion of demand satisfied	Number of women
**West and Central Africa**							
Benin 2006	54.9	27.7	24.9	2.7	82.6	66.5	672
Burkina Faso 2003	58.3	31.4	30.3	1.2	89.7	65.0	295
Cameroon 2004	68.5	16.5	13.1	3.4	84.9	80.6	597
Congo (Brazzaville) 2005	73.3	14.0	12.9	1.2	87.3	83.9	989
Congo Democratic Republic 2007	47.5	35.3	29.4	5.9	82.9	57.4	653
Gabon 2000	60.1	23.8	20.8	3.0	83.8	71.7	977
Ghana 2008	50.4	39.8	36.7	3.1	90.1	55.9	284
Guinea 2005	45.5	42.3	38.1	4.2	87.8	51.8	283
Liberia 2007	27.3	51.6	47.4	4.2	78.9	34.6	1,045
Nigeria 2008	61.0	30.8	27.3	3.5	91.8	66.5	1,607
Sierra Leone 2008	33.6	45.3	37.7	7.6	79.0	42.6	551
**East and Southern Africa**							
Kenya 2008-09	50.3	35.3	23.6	11.7	85.6	58.7	318
Lesotho 2009	57.8	32.0	12.4	19.6	89.9	64.4	466
Madagascar 2008-09	43.1	36.8	28.0	8.8	79.9	54.0	815
Malawi 2010	47.4	38.3	27.0	11.4	85.8	55.3	523
Mozambique 2003	45.0	25.6	18.8	6.8	70.6	63.7	1,065
Namibia 2006-07	77.9	15.7	8.2	7.5	93.6	83.2	1,343
Swaziland 2006-07	64.5	28.5	12.9	15.5	93.0	69.4	573
Tanzania 2010	50.6	31.0	20.8	10.2	81.6	62.0	742
Uganda 2006	54.0	26.1	19.1	6.9	80.1	67.5	359
Zambia 2007	47.6	38.4	25.8	12.5	85.9	55.4	320
Zimbabwe 2005-06	61.2	26.7	16.2	10.5	88.0	69.6	191
**Eastern Europe/NIS**							
Moldova 2005	66.8	24.2	16.3	7.9	90.9	73.4	305
Ukraine 2007	87.3	5.7	4.2	1.5	93.0	93.9	637
**Latin America and Caribbean**							
Bolivia 2008	77.7	16.2	10.9	5.2	93.9	82.8	618
Colombia 2010	81.6	11.2	7.8	3.4	92.9	87.9	6,335
Dominican Republic 2007	61.6	22.5	16.6	5.9	84.1	73.2	2,171
Guyana 2009	63.6	25.8	17.6	8.2	89.4	71.1	408
Haiti 2005-06	40.5	50.2	45.7	4.5	90.7	44.6	444
Honduras 2005-06	71.4	16.4	9.8	6.5	87.7	81.4	581
Nicaragua 2001	66.8	18.4	9.6	8.9	85.3	78.4	402
Peru 2004-08	86.4	6.2	4.2	1.9	92.5	93.3	2,328
**Unweighted Averages**							
West and Central Africa	52.8	32.6	29.0	3.6	85.3	61.5	723
East and Southern Africa	54.5	30.4	19.3	11.1	84.9	63.9	611
Eastern Europe/NIS	77.1	14.9	10.2	4.7	92.0	83.7	471
Latin America and Caribbean	68.7	20.9	15.3	5.6	89.6	76.6	1,661
**Total**	**58.9**	**27.8**	**21.1**	**6.7**	**86.7**	**67.5**	

Note: Excludes surveys with less than 150 sexually active unmarried women

5

Estimating the Demographic Impact of Fulfilling Unmet Need

An important use of the unmet need indicator is to estimate how reducing levels of unmet need would affect fertility rates. If all demand for family planning were to be satisfied, how much might fertility be expected to decline? To answer this question, we use data from the most recent survey in each country with a DHS conducted since 2000, a total of 59 countries.

The section examines the impact on fertility of satisfying the unmet need for limiting only. Earlier approaches concerning the impact on fertility of fulfilling unmet need differentiated unmet need for spacing from unmet need for limiting. In theory, contraceptive use for spacing should have a weaker association with fertility than use for limiting, because contraceptive use for spacing is temporary, and couples will discontinue use to have another child. In the 59 countries analyzed, the correlation of contraceptive use for spacing with fertility is low (-0.26) compared with use for limiting births (-0.87). When the two components are considered simultaneously, the independent contribution of the spacing variable is negligible. This does not mean that contraception for spacing is irrelevant for future fertility, since spacers tend to become limiters at some point, as well as experiencing health-related advantages of family planning use (Westoff and Koffman 2010). For this cross-sectional analysis, however, contraceptive use for limiting births is considered to be the primary predictor of the total fertility rate (TFR).

The procedure for estimating the impact on fertility of satisfying all unmet need for limiting is straightforward. Using DHS data for the 59 countries, we calculated a regression equation including TFR and contraceptive use for limiting. Figure 55 shows TFR plotted against current contraceptive use for limiting for the 59 countries and includes the regression equation.

With this equation, we can predict the level of TFR for a given level of contraceptive use for limiting births. In order to estimate the impact of satisfying the unmet need for limiting on the TFR, the level of contraceptive use selected is equal to the total demand for contraception for limiting. The total demand for limiting is estimated by summing the proportion of married women who are currently using a contraceptive method and who want no more children (i.e., current use for limiting) and the proportion of married women with an unmet need for limiting. Two predicted TFRs are calculated, the first based on the **Original** definition of unmet need and the second on the **Revised** definition, to assess the difference between them in predicting the demographic impact of satisfying unmet need.

These predictions reflect a maximum estimate of the effect of satisfying unmet need for limiting on fertility since it assumes that all unmet need for limiting is satisfied. We are not intending to forecast fertility but rather to demonstrate the impact of reducing unmet need. These estimates do not account for changes in other factors that would be important in any fertility forecast such as the number of children wanted, the effectiveness of the contraceptive methods used, and the abortion rate.

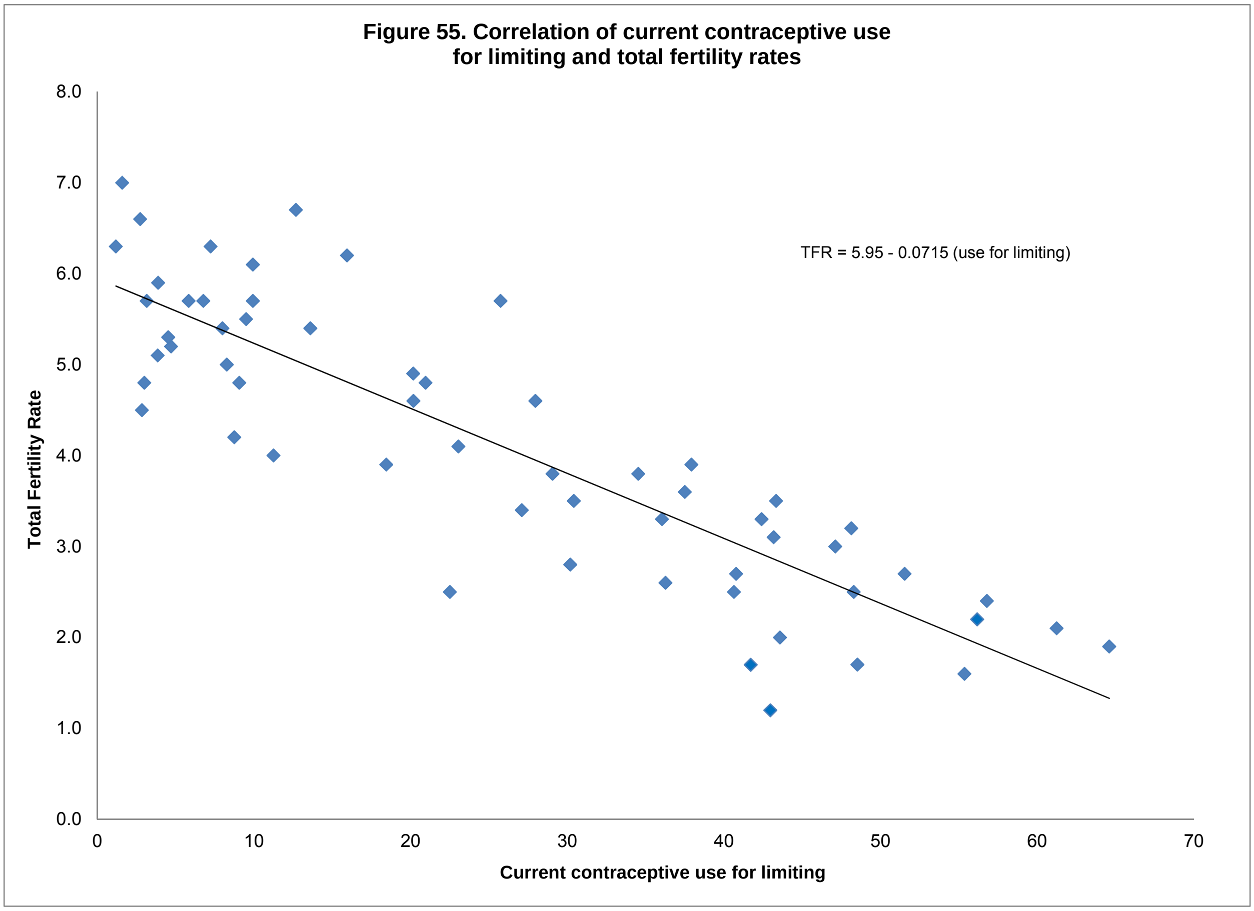

The regression analyses rely on the TFR, the use of contraception, and the measure of unmet need. One possible issue here is that the TFR is based on all women regardless of marital status, while the measurements of contraceptive use and unmet need were calculated for married women only. In order to investigate whether this inconsistency would bias the results, we estimated two kinds of fertility rates for ever-married women. One is the sum of marriage-duration specific fertility rates, and the other is the sum of age-specific fertility rates for married women only. These two rates are so highly correlated with each other and with the conventional TFR that hardly any differences in the outcome are discernible (data not shown).

The results are shown in Table 6 and Figure 56 for countries aggregated in five world regions.[14] The **Original** and **Revised** definitions of unmet need yield similar levels of total demand for limiting. If all unmet need for limiting were satisfied, contraceptive use for limiting for all countries combined would increase from the current level of 26 percent to 35 percent according to the **Original** definition, and to 36 percent according to the **Revised** definition.

Looking at the estimated total demand for limiting according to the **Revised** definition, the pattern of increase is similar for countries in Asia, Latin America/Caribbean, and North Africa/Middle East/Europe. In these three regions, current contraceptive use among married women to limit births is 34, 44, and 46 percent, respectively. If all unmet need for limiting were satisfied, the percentages would increase to 47 percent in Asia and 55 percent in each of the other two regions.

[14] The Middle East/North Africa and Eastern Europe/NIS regions were combined to provide a large enough sample to analyze.

In West and Central Africa, where only 6 percent of married women are currently using contraception for limiting, contraceptive use would increase to 15 percent if all unmet need for limiting were satisfied. In Eastern and Southern Africa, where 20 percent of married women are currently using contraception for limiting, contraceptive use would rise to 32 percent if all unmet need for limiting were satisfied.

Table 6. Current contraceptive use, unmet need, and total demand for limiting

Current contraceptive use for limiting births, unmet need for limiting, and total demand for limiting among currently married women 15-49 using the Original and Revised definitions, most recent surv ey from eac h country, DHS 2000-2010

		Original		Revised		
	Current use for limiting	Unmet need for limiting	Total demand for limiting	Unmet need for limiting	Total demand for limiting	Number of countries
West and Central Africa	6.2	8.0	14.2	8.6	14.8	17
East and Southern Africa	20.2	10.8	31.0	11.9	32.1	14
Middle East/North Africa, Eastern Europe/NIS	45.6	7.2	52.8	9.0	54.7	9
Asia	34.1	12.0	46.1	12.9	47.0	11
Latin America and Caribbean	43.6	10.4	54.0	11.0	54.6	8
**Total**	**25.8**	**9.6**	**35.4**	**10.6**	**36.4**	**59**

Figure 57 shows the fertility implications of completely satisfying all unmet need for limiting. Using the **Revised** definition of unmet need, the predicted TFR for these countries combined would decline from 4.1 children per woman to 3.3, a relative decline of 20 percent. The smallest decline would be in North Africa/Middle East/Europe, where the average TFR would decline from 2.2 children per woman to 2.0. The estimated declines are higher in the other regions but smaller in West and Central Africa, where the expected decrease is from 5.4 children per woman to 4.9. In this region, the smaller estimated decline in fertility reflects the low level of demand for limiting births and implies that a significant reduction in the number of children wanted will have to occur there, and to a lesser extent in East and Southern Africa, if fertility is to fall substantially. The estimated Wanted Total Fertility Rates for these two sub-Saharan regions are 5.0 and 4.0, respectively (Westoff and Koffman, 2010). In contrast, this rate averages 2.2 in the other regions studied.

Figure 56 also shows that the predicted TFRs using the **Revised** definition of unmet need are almost identical to those predicted using the **Original** definition. This finding indicates that the revisions in the calculation of the indicator do not have a significant impact on the estimated demographic impact of satisfying unmet need.

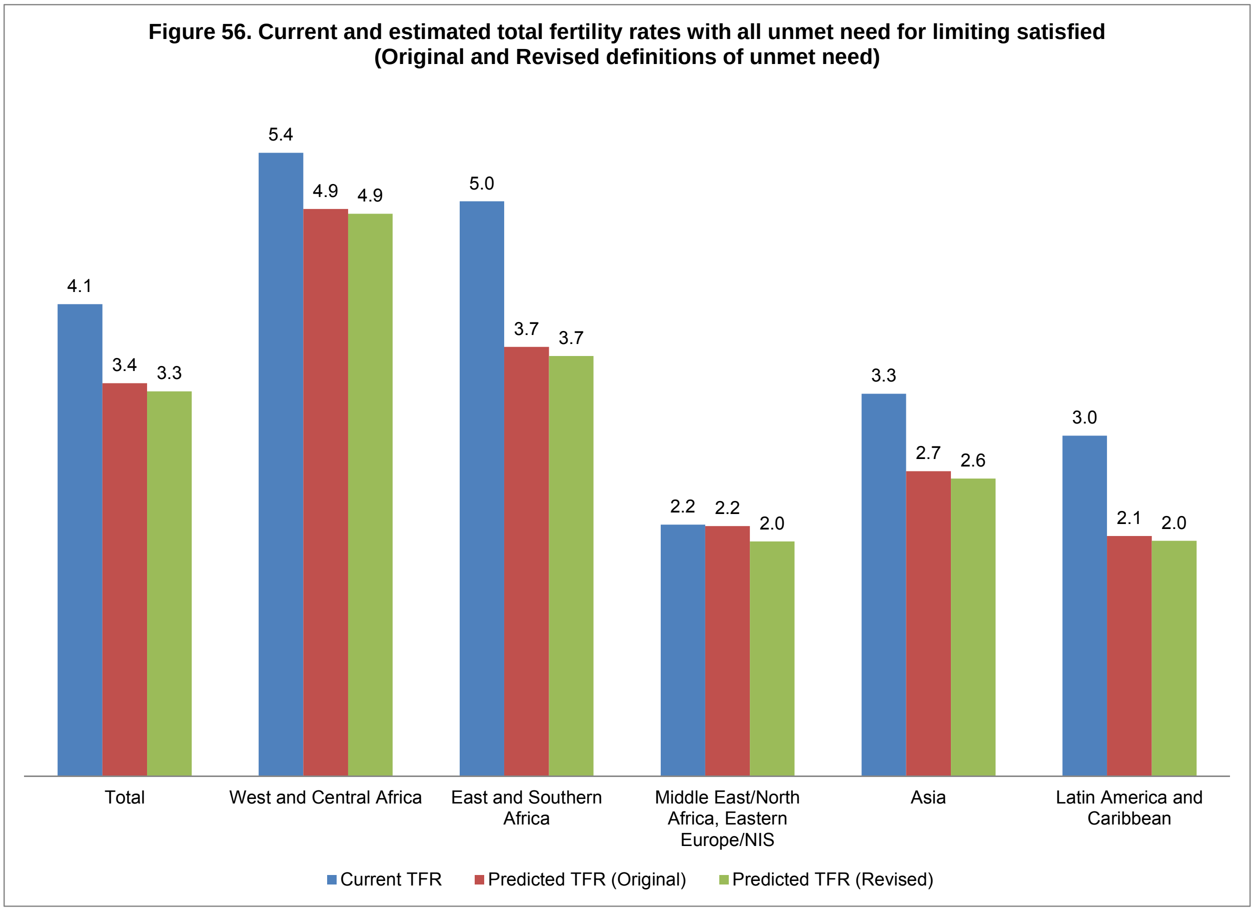

Figure 56. Current and estimated total fertility rates with all unmet need for limiting satisfied
(Original and Revised definitions of unmet need)
4.1
3.4
3.3
5.4
4.9
4.9
5.0
3.7
3.7
2.2
2.2
2.0
3.3
2.7
2.6
3.0
2.1
2.0
Total
West and Central Africa
East and Southern Africa
Middle East/North Africa, Eastern Europe/NIS
Asia
Latin America and Caribbean
Current TFR
Predicted TFR (Original)
Predicted TFR (Revised)

6

Conclusions

While the concept of unmet need for family planning has existed for several decades, its definition and calculation are now gaining an unprecedented level of attention from donors as the family planning movement is revitalized. Policymakers and program planners are monitoring information on unmet need as never before, in part due to its recent inclusion as an MDG indicator. As a result, there has never been a more crucial time to ensure that unmet need is measured and reported consistently.

Despite acknowledgment that levels of unmet need can vary widely with the many changes made in its definition (Govindasamy and Boadi 2000; Westoff and Pebley 1981), publications often assume that the definition of unmet need has remained constant and that survey estimates of unmet need can be tracked and compared over time and across countries. As shown in this report, this assumption is untrue.

This research demonstrates that the varying definitions of unmet need that have been used over time have resulted in estimates that are not comparable with each other and have led to incorrect interpretation of trends in several countries. Previously calculated levels of unmet need also cannot be compared across countries or survey programs, as variations in the definition make such comparisons unreliable and misleading.

This research also demonstrates that unmet need is an extremely complex indicator that is difficult to fully understand, and even more difficult to calculate. The indicator provides an aggregate-level measure of unmet need for family planning in a population but is not a reliable measure of need for any individual woman. Unmet need does not indicate a woman’s access to family planning information or services, her desire to use contraception, or other factors that may affect contraceptive use.

In this paper we present a simplified, standard definition of unmet need that can be consistently applied over time and across countries. This **Revised** definition results in a small increase in estimated levels of unmet need. In the majority of surveys, the impact is minimal. There are no major changes in patterns of unmet need by women’s background characteristics, and estimates of the demographic impact of satisfying all unmet need using the **Original** and **Revised** definitions are similar.

In some countries that have high levels of contraceptive use and that have collected calendar data, however, the impact of the **Revised** definition is larger. In surveys that calculated unmet need using calendar data, the **Revised** definition produces estimates of unmet need that are consistently higher than the **Original** definition.

We hope that simplification and standardization of the unmet need definition will help ensure the quality and comparability of a key MDG indicator. In turn, better data on unmet need should help to inform advocacy efforts for family planning and maternal and child health policies and programs across the globe.

References

Bogue, D.J. 1974. Population perspectives: Some views from a sociologist. *Population Dynamics Quarterly* 2(2c): 2.

Bongaarts, J. 1991. The KAP-gap and the unmet need for contraception. *Population and Development Review* 17(2): 293-313.

Bradley, S.E.K., Croft, T., and Fishel, J. 2009. Understanding unmet need: Changes in measurement over time in the Demographic and Health Surveys. Unpublished presentation made at the International Conference on Family Planning: Research and Best Practices, Kampala, Uganda.

Department of Statistics [Jordan] and Macro International Inc. 2008. *Jordan Population and Family Health Survey 2007.* Calverton, Maryland, USA: Department of Statistics and Macro International Inc.

Govindasamy, P. and Boadi, E. 2000. A decade of unmet need for contraception in Ghana: Programmatic and Policy Implications. Calverton, Maryland: Macro International Inc. and National Population Council Secretariat [Ghana].

ICF Macro. 2010. RECODE5.APP. Computer program written in CSPro for creation of DHS recode dated 2/10/2010.

Macro International Inc. 1995. Model “A” Questionnaire with Commentary for High Contraceptive Prevalence Countries. DHS-III Basic Documentation Number 1. Calverton, Maryland: Macro International Inc.

Macro International Inc. 1996. RECODE3.APP. Computer program written in ISSA for creation of DHS recode dated 11/1/1996.

National Statistics Office (NSO) [Philippines] and ICF Macro. 2009. *National Demographic and Health Survey 2008.* Calverton, Maryland: National Statistics Office and ICF Macro.

Nortman, D.L. 1982. Measuring the unmet need for contraception to space and limit births. *International Family Planning Perspectives* 8(4): 125-134.

ORC Macro. 2001. Model “A” Questionnaire with Commentary for High Contraceptive Prevalence Countries. MEASURE DHS+ Basic Documentation Number 1. Calverton, Maryland: ORC Macro.

ORC Macro. 2005. RECODE4.APP. Computer program written in CSPro for creation of DHS recode dated 11/18/2005.

Robey, B., Ross, J.A., and Bhushan, I. 1996. Meeting unmet need: New strategies. *Population Reports***,** *Series J,* No. 43. Baltimore, Johns Hopkins School of Public Health, Population Information Program.

Ross, J.A. and Winfrey, W.L. 2001. Contraceptive Use, Intention to Use and Unmet Need during the Extended Postpartum Period. *International Family Planning Perspectives* 27(1):20–27.

UN. 2007. Metadata for “Unmet need for family planning, total, percentage.” Available at http://unstats.un.org/unsd/mdg/Metadata.aspx (viewed 11/7/11).

UN. 2008. Official list of MDG indicators. Available at http://unstats.un.org/unsd/mdg/host.aspx?content =indicators/officiallist.htm (viewed 11/7/11).

Westoff, C.F. 1978. The unmet need for birth control in five Asian countries. *Family Planning Perspectives* 10(3):173-81.

Westoff, C.F. and Pebley, A.R. 1981. Alternative measures of unmet need for family planning in developing countries. *International Family Planning Perspectives* 7(4): 126-136.

Westoff, C.F. 1988. The potential demand for family planning: A new measure of unmet need and estimates for five Latin American countries. *International Family Planning Perspectives* 14(2): 45-53.

Westoff, C.F. and Ochoa, L.H. 1991. Unmet need and the demand for family planning. Demographic and Health Surveys Comparative Studies No. 5. Columbia, Maryland: Institute for Resource Development.

Westoff, C.F. and Bankole, A. 1995. Unmet need: 1990-1994. DHS Comparative Studies No. 16. Calverton, Maryland: Macro International Inc.

Westoff, C.F. and Bankole, A. 1996. The potential demographic significance of unmet need. *International Family Planning Perspectives* 22(1): 16-20.

Westoff, C.F. 2006. New Estimates of Unmet Need and the Demand for Family Planning. DHS Comparative Reports No. 14. Calverton, Maryland, USA. Macro International Inc.

Westoff, C.F. and Koffman, D. 2010. Birth Spacing and Limiting Connections. DHS Analytical Studies No. 21. Calverton, Maryland, USA: ICF Macro.

Appendix A. Questions and Filters Needed for Unmet Need Definition

NO.	QUESTIONS AND FILTERS	CODING CATEGORIES	SKIP
226	Are you pregnant now?	YES . . . . . 1<br>NO . . . . . 2<br>UNSURE 8	 2, 8 → 238
228	When you got pregnant, did you want to get pregnant at that time?	YES . . . . . 1<br>NO 2	1 → 238
229	Did you want to have a baby later on or did you not want any (more) children?	LATER . . . . . 1<br>NO MORE 2	
238	When did your last menstrual period start?<br><br>_______________<br>(DATE, IF GIVEN)	DAYS AGO . . . . . 1 [ ][ ]<br>WEEKS AGO . . . . . 2 [ ][ ]<br>MONTHS AGO . . . . . 3 [ ][ ]<br>YEARS AGO . . . . . 4 [ ][ ]<br>IN MENOPAUSE/<br>HAS HAD HYSTERECTOMY . . . 994<br>BEFORE LAST BIRTH . . . . . 995<br>NEVER MENSTRUATED 996	
302	CHECK 226:<br>NOT PREGNANT OR UNSURE [ ] ↓ PREGNANT [ ] →		313
303	Are you currently doing something or using any method to delay or avoid getting pregnant?	YES . . . . . 1<br>NO 2	
313	Have you ever used anything or tried in any way to delay or avoid getting pregnant?	YES . . . . . 1<br>NO 2	
212	What name was given to your (last) baby?<br>RECORD NAME	NAME _______________	
215	In what month and year was (NAME) born?<br><br>PROBE:<br>When is his/her birthday?	MONTH . . . . . [ ][ ]<br>YEAR . . . . . [ ][ ][ ][ ]	

NO.	QUESTIONS AND FILTERS	CODING CATEGORIES	SKIP
401	CHECK 215:<br>BIRTH IN 2006 OR LATER ☐ ↓<br>BIRTH BEFORE 2006 ☐ → 601		→ 601
405	When you got pregnant with (NAME), did you want to get pregnant at that time?	YES . . . . . 1<br>NO 2	(1) → 447
406	Did you want to have a baby later on, or did you not want any (more) children?	LATER . . . . . 1<br>NO MORE 2	
447	Has your menstrual period returned since the birth of (NAME)?	YES . . . . . 1<br>NO 2	
601	Are you currently married or living together with a man as if married?	YES, CURRENTLY MARRIED . . . . . 1<br>YES, LIVING WITH A MAN . . . . . 2<br>NO, NOT IN UNION 3	
610	Now I would like to ask about your (first) (husband/partner). In what month and year did you start living with him?	MONTH . . . . . [ ][ ]<br>DON'T KNOW MONTH . . . . . 98<br>YEAR . . . . . [ ][ ][ ][ ]<br>DON'T KNOW YEAR 9998	
615	When was the <u>last</u> time you had sexual intercourse?<br>IF LESS THAN 12 MONTHS, ANSWER MUST BE RECORDED IN DAYS, WEEKS OR MONTHS.<br>IF 12 MONTHS (ONE YEAR) OR MORE, ANSWER MUST BE RECORDED IN YEARS.	DAYS AGO . . . . . 1 [ ][ ]<br>WEEKS AGO . . . . . 2 [ ][ ]<br>MONTHS AGO . . . . . 3 [ ][ ]<br>YEARS AGO . . . . . 4 [ ][ ]	
703	Now I have some questions about the future. After the child you are expecting now, would you like to have another child, or would you prefer not to have any more children?	HAVE ANOTHER CHILD . . . . . 1<br>NO MORE . . . . . 2<br>UNDECIDED/DON'T KNOW 8	(1) → 705<br>(2, 8) → END
704	Now I have some questions about the future. Would you like to have (a/another) child, or would you prefer not to have any (more) children?	HAVE (A/ANOTHER) CHILD . . . . . 1<br>NO MORE/NONE . . . . . 2<br>SAYS SHE CAN'T GET PREGNANT 3<br>UNDECIDED/DON'T KNOW 8	(2) → 707<br>(3, 8) → END
705	CHECK 226:<br>NOT PREGNANT OR UNSURE ☐ ↓<br>How long would you like to wait from now before the birth of (a/another) child?<br>PREGNANT ☐ ↓<br>After the birth of the child you are expecting now, how long would you like to wait before the birth of another child?	MONTHS . . . . . 1 [ ][ ]<br>YEARS . . . . . 2 [ ][ ]<br>SOON/NOW . . . . . 993<br>SAYS SHE CAN'T GET PREGNANT 994<br>AFTER MARRIAGE . . . . . 995<br>OTHER ______________ 996<br>(SPECIFY)<br>DON'T KNOW 998	(993) → END<br>(994) → END<br>(995, 996, 998) → END

NO.	QUESTIONS AND FILTERS	CODING CATEGORIES	SKIP
706	CHECK 226:<br>NOT PREGNANT OR UNSURE ☐ ↓ PREGNANT ☐ →		END
707	CHECK 303: USING A CONTRACEPTIVE METHOD?<br>NOT CURRENTLY USING ☐ ↓ CURRENTLY USING ☐ →		END
708	CHECK 705:<br>NOT ASKED ☐ ↓ 24 OR MORE MONTHS OR 02 OR MORE YEARS ☐ ↓ 00-23 MONTHS OR 00-01 YEAR ☐ →		END
709	CHECK 704:<br>WANTS TO HAVE A/ANOTHER CHILD ☐ ↓ WANTS NO MORE/ NONE ☐ ↓<br>You have said that you do not want (a/another) child soon.<br>Can you tell me why you are not using a method to prevent pregnancy?<br>Any other reason?<br>You have said that you do not want any (more) children.<br>Can you tell me why you are not using a method to prevent pregnancy?<br>Any other reason?<br>RECORD ALL REASONS MENTIONED.	NOT MARRIED . . . . A<br>FERTILITY-RELATED REASONS<br>NOT HAVING SEX . . . . B<br>INFREQUENT SEX . . . . C<br>MENOPAUSAL/HYSTERECTOMY D<br>CAN'T GET PREGNANT . . . . E<br>NOT MENSTRUATED SINCE LAST BIRTH . . . . F<br>BREASTFEEDING . . . . G<br>UP TO GOD/FATALISTIC . . . . H<br>OPPOSITION TO USE<br>RESPONDENT OPPOSED . . . . I<br>HUSBAND/PARTNER OPPOSED . . . J<br>OTHERS OPPOSED . . . . K<br>RELIGIOUS PROHIBITION . . . . L<br>LACK OF KNOWLEDGE<br>KNOWS NO METHOD . . . . M<br>KNOWS NO SOURCE . . . . N<br>METHOD-RELATED REASONS<br>SIDE EFFECTS/HEALTH CONCERNS . . . . O<br>LACK OF ACCESS/TOO FAR . . . . . P<br>COSTS TOO MUCH . . . . Q<br>PREFERRED METHOD NOT AVAILABLE . . . . R<br>NO METHOD AVAILABLE . . . . S<br>INCONVENIENT TO USE . . . . T<br>INTERFERES WITH BODY'S NORMAL PROCESSES . . . . U<br>OTHER ______________________ X<br>(SPECIFY)<br>DON'T KNOW . . . . Z	

Note that question texts have been modified slightly from the DHS questionnaire to reflect the information needed for the definition of unmet need and avoid extraneous questions. Skips have been modified to reflect the flow of questions in this set of questions. For the original questions and skips, please see the DHS Model Questionnaire (Phase 6) [http://www.measuredhs.com/publications/publication-DHSQ6-DHS-Questionnaires-and-Manuals.cfm]

Appendix Table 1. Unmet need by residence, education, and wealth quintile

Unmet need (total) among currently married women 15-49 by place of residence, highest education level, and household wealth quintile using the Revised definition of unmet need, most recent survey from each country, DHS 2000-2010

		Place of residence		Highest level of education			Wealth index quintiles				
Survey Year	Total	Urban	Rural	No education	Primary	Secondary or higher	Lowest quintile	Second quintile	Middle quintile	Fourth quintile	Highest quintile
**West and Central Africa**											
Benin 2006	27.3	26.3	27.9	27.3	29.5	22.8	28.8	27.8	27.9	28.3	23.5
Burkina Faso 2003	29.8	23.2	31.0	31.0	24.9	16.0	29.4	30.5	32.6	32.2	23.1
Cameroon 2004	20.5	20.0	21.0	20.1	23.0	17.7	19.8	22.3	23.4	20.8	16.6
Chad 2004	20.6	25.3	19.5	18.7	28.1	25.1	17.6	19.8	21.0	20.3	24.6
Congo (Brazzaville) 2005	19.5	19.4	19.7	20.6	22.5	17.9	22.1	22.3	19.6	17.4	16.6
Congo Democratic Republic 2007	26.9	28.1	26.1	22.5	26.1	31.0	27.3	25.5	26.9	27.2	28.0
Gabon 2000	27.9	27.2	30.0	32.9	33.2	23.3	33.2	31.0	27.8	26.5	22.2
Ghana 2008	35.7	32.6	37.9	35.6	41.1	33.3	36.9	42.9	39.9	35.2	24.3
Guinea 2005	21.9	23.0	21.5	20.9	25.4	30.3	19.6	20.3	23.7	23.5	23.1
Liberia 2007	35.7	34.4	36.3	32.9	42.0	33.7	32.5	37.6	38.2	38.0	31.3
Mali 2006	27.6	28.4	27.2	26.9	29.5	32.8	28.6	27.0	26.6	25.8	29.9
Mauritania 2000-01	32.1	35.2	30.0	29.8	38.1	34.8	29.9	33.2	32.4	33.9	30.9
Niger 2006	16.1	23.9	14.7	15.6	18.5	22.8	16.0	15.2	14.7	14.3	20.8
Nigeria 2008	20.2	19.4	20.6	19.3	22.5	20.0	18.5	20.4	22.0	22.8	18.2
Sao Tome and Principe 2008-09	37.6	42.2	32.4	40.3	36.3	40.2	44.0	39.4	40.9	32.8	32.2
Senegal 2005	32.0	32.6	31.6	31.6	35.1	27.4	30.5	31.9	33.8	34.2	29.3
Sierra Leone 2008	28.4	28.6	28.3	27.7	30.8	30.2	27.6	28.5	28.7	30.3	26.6
**East and Southern Africa**											
Eritrea 2002	28.5	25.9	29.8	28.3	30.5	25.2	29.2	29.2	32.8	28.9	21.4
Ethiopia 2005	36.1	19.8	38.0	36.9	38.7	19.5	35.8	39.2	39.0	39.2	26.3
Kenya 2008-09	25.6	19.6	27.5	26.5	30.2	16.5	38.4	32.4	22.9	20.1	18.3
Lesotho 2009	23.3	15.2	26.7	24.1	27.3	18.4	37.2	29.5	22.3	20.7	13.3
Madagascar 2008-09	19.0	17.1	19.3	20.0	20.1	16.2	23.1	21.5	17.9	16.9	16.4
Malawi 2010	26.2	23.3	26.8	27.8	27.0	21.0	30.0	27.6	27.2	24.8	22.0
Mozambique 2003	18.9	19.9	18.5	18.0	20.2	14.5	17.8	18.7	18.5	21.5	18.7
Namibia 2006-07	20.7	15.7	25.8	30.3	26.4	15.8	32.8	23.4	25.3	16.6	11.0

(Continued…)

Appendix Table 1 – *Continued*

Survey Year	Total	Place of residence		Highest level of education			Wealth index quintiles				
		Urban	Rural	No education	Primary	Secondary or higher	Lowest quintile	Second quintile	Middle quintile	Fourth quintile	Highest quintile
Rwanda 2005	38.5	34.3	39.1	40.3	38.9	29.2	41.2	37.5	40.0	38.6	34.5
Swaziland 2006-07	24.7	20.9	26.1	32.3	29.9	19.7	34.5	26.2	24.8	24.9	17.2
Tanzania 2010	25.3	19.6	27.2	30.0	24.6	17.0	31.4	27.2	28.5	23.4	16.2
Uganda 2006	38.0	26.0	39.8	38.3	40.9	26.1	42.8	43.6	39.5	38.1	25.2
Zambia 2007	26.6	23.1	28.5	27.9	28.5	21.4	27.0	31.4	29.8	25.5	18.9
Zimbabwe 2005-06	15.5	10.8	17.9	32.4	18.4	12.0	23.6	16.5	15.1	12.6	9.9
**Middle East/North Africa**											
Egypt 2008	11.6	9.5	13.1	14.2	13.7	9.8	15.3	12.7	11.7	10.6	8.2
Jordan 2009	13.4	13.2	14.5	29.3	17.0	12.6	15.9	14.2	11.5	13.7	11.6
Morocco 2003-04	11.9	11.4	12.5	12.7	11.3	9.6	12.9	12.7	13.1	11.6	9.1
Eastern Europe/NIS											
Albania 2008-09	12.9	9.7	15.2	16.3	15.1	9.9	15.9	13.6	13.8	12.3	8.6
Armenia 2005	19.3	18.2	21.2	4.9	16.4	19.4	20.8	20.3	20.9	18.7	16.3
Azerbaijan 2006	15.4	14.8	16.1	15.2	27.5	15.3	15.5	16.5	16.1	18.2	10.9
Moldova 2005	11.4	12.4	10.6	20.6	8.1	11.4	8.5	13.1	12.0	10.9	12.1
Turkey 2003	9.5	8.0	13.2	17.3	8.9	6.1	18.3	10.6	9.6	5.9	6.2
Ukraine 2007	10.1	8.7	13.2	0.0	14.7	10.1	14.0	9.9	12.0	10.4	7.0
**Asia**											
Bangladesh 2007	16.8	14.1	17.6	16.7	16.0	17.5	17.1	18.2	16.8	16.9	15.1
India 2005-06	13.9	11.0	15.2	14.7	13.2	13.2	19.1	15.8	13.7	11.8	9.6
Maldives 2009	28.6	27.0	29.4	24.2	27.4	32.2	28.8	29.8	29.1	29.0	26.5
Nepal 2006	24.7	19.7	25.6	21.7	27.7	31.2	32.1	26.8	22.7	23.3	19.2
Pakistan 2006-07	25.2	22.0	26.7	26.7	24.7	20.8	31.4	27.9	26.6	20.2	20.2
Cambodia 2010	16.9	12.1	18.0	18.0	18.0	13.7	21.1	19.9	16.3	15.6	12.0
Indonesia 2007	13.1	12.9	13.3	18.9	14.1	11.3	16.7	12.9	13.2	10.5	12.6
Philippines 2008	22.0	20.6	23.5	28.6	24.6	21.0	28.1	22.6	20.5	18.9	20.1
Samoa 2009	47.7	48.2	47.6	72.5	53.8	47.3	47.5	50.5	52.6	44.5	43.2
Timor-Leste 2009	31.5	30.3	31.9	31.6	31.0	31.7	35.7	31.1	34.2	28.8	28.4
Vietnam 2002	6.6	6.0	6.8	10.4	7.7	5.8	9.2	7.2	6.3	5.5	5.2

(Continued…)

Appendix Table 1 – *Continued*

Survey Year	Total	Place of residence		Highest level of education			Wealth index quintiles				
		Urban	Rural	No education	Primary	Secondary or higher	Lowest quintile	Second quintile	Middle quintile	Fourth quintile	Highest quintile
**Latin America and Caribbean**											
Bolivia 2008	20.1	15.5	27.4	27.8	23.9	14.4	34.2	24.3	19.9	14.4	9.4
Colombia 2010	8.0	7.6	9.2	14.1	8.1	7.8	11.8	8.0	7.8	6.8	5.5
Dominican Republic 2007	11.1	11.2	11.0	12.2	10.7	11.4	15.1	11.6	10.9	9.7	8.9
Guyana 2009	28.5	29.5	28.2	40.2	29.4	27.8	38.4	28.3	24.9	27.0	24.3
Haiti 2005-06	37.3	34.9	39.1	38.1	40.1	33.1	44.2	42.8	36.4	34.1	31.5
Honduras 2005-06	16.8	14.2	19.2	21.8	17.2	14.2	24.4	17.8	16.2	14.7	12.2
Nicaragua 2001	14.6	11.5	18.9	23.3	13.8	11.5	25.2	15.9	11.9	11.7	9.5
Peru 2004-08	12.4	10.7	15.7	16.4	14.8	10.8	21.4	14.5	12.1	9.2	9.7

Appendix Table 2. Unmet need by parity and age

Total unmet need and unmet need for spacing and limiting among currently married women age 15-49 by parity and age in 5 year groups using the Revised definition of unmet need, most recent survey from each country, DHS 2000-2010

			Parity				Age in 5 year groups						
**Survey Year**		**Total**	**0**	**1**	**2-3**	**4+**	**15-19**	**20-24**	**25-29**	**30-34**	**35-39**	**40-44**	**45-49**
**West and Central Africa**													
Benin 2006	Spacing	17.4	7.5	24.6	22.0	13.6	26.2	27.8	22.5	17.2	11.7	5.4	1.8
Benin 2006	Limiting	9.9	0.0	0.3	3.4	17.6	0.4	1.0	3.7	9.8	18.8	23.4	19.3
Benin 2006	Total	27.3	7.5	25.0	25.4	31.2	26.6	28.8	26.2	27.0	30.6	28.7	21.1
Burkina Faso 2003	Spacing	22.3	8.4	24.8	27.9	20.9	19.4	30.2	29.4	25.3	22.3	10.7	3.4
Burkina Faso 2003	Limiting	7.5	0.2	0.1	1.4	13.9	0.5	0.4	1.1	6.2	16.3	19.5	16.3
Burkina Faso 2003	Total	29.8	8.7	25.0	29.2	34.7	19.9	30.7	30.5	31.5	38.6	30.1	19.6
Cameroon 2004	Spacing	14.1	6.7	14.9	17.4	13.7	17.6	19.9	16.6	14.7	9.2	6.7	1.9
Cameroon 2004	Limiting	6.5	0.4	0.7	2.8	12.7	0.9	1.5	2.6	6.3	15.7	15.1	12.7
Cameroon 2004	Total	20.5	7.1	15.6	20.1	26.5	18.5	21.4	19.2	21.0	24.9	21.8	14.6
Chad 2004	Spacing	17.9	11.2	19.4	21.1	16.9	18.8	21.9	22.3	17.8	16.9	10.7	4.9
Chad 2004	Limiting	2.7	0.2	0.0	0.5	4.7	0.2	0.3	0.8	2.6	5.8	10.0	4.4
Chad 2004	Total	20.6	11.4	19.4	21.5	21.6	19.0	22.1	23.1	20.4	22.7	20.6	9.2
Congo (Brazzaville) 2005	Spacing	14.0	12.2	16.4	16.5	11.0	28.3	20.4	13.4	13.3	11.0	6.1	3.8
Congo (Brazzaville) 2005	Limiting	5.5	0.5	1.9	2.9	10.8	2.0	1.3	2.1	5.9	6.0	16.3	14.0
Congo (Brazzaville) 2005	Total	19.5	12.7	18.2	19.4	21.7	30.3	21.7	15.4	19.1	17.0	22.4	17.8
Congo Democratic Republic 2007	Spacing	19.9	11.4	22.6	24.1	17.9	24.9	29.1	23.3	21.6	16.1	7.3	2.7
Congo Democratic Republic 2007	Limiting	7.0	0.6	3.0	4.3	10.9	4.0	3.4	4.2	7.0	10.3	15.4	9.6
Congo Democratic Republic 2007	Total	26.9	12.0	25.7	28.4	28.8	28.9	32.5	27.5	28.5	26.4	22.7	12.2
Gabon 2000	Spacing	19.7	14.5	20.2	21.0	20.1	27.8	27.5	23.1	16.3	19.3	9.8	5.3
Gabon 2000	Limiting	8.1	0.3	1.0	3.8	16.6	1.3	2.6	2.8	6.3	12.0	21.3	22.5
Gabon 2000	Total	27.9	14.8	21.3	24.8	36.7	29.1	30.1	25.8	22.7	31.3	31.0	27.8
Ghana 2008	Spacing	21.5	18.8	29.7	27.7	13.9	49.0	34.4	33.8	21.2	14.1	7.3	3.5
Ghana 2008	Limiting	14.2	2.6	4.3	8.3	24.7	12.7	8.1	6.7	12.5	20.7	23.9	17.3
Ghana 2008	Total	35.7	21.4	34.0	36.0	38.6	61.7	42.4	40.5	33.7	34.8	31.2	20.7
Guinea 2005	Spacing	13.4	9.1	16.8	16.0	12.0	18.4	20.6	17.9	16.6	11.2	4.7	0.9
Guinea 2005	Limiting	8.5	0.6	0.6	2.2	14.7	1.2	0.9	2.7	6.7	13.7	19.6	16.5
Guinea 2005	Total	21.9	9.7	17.4	18.1	26.8	19.5	21.6	20.6	23.3	24.8	24.3	17.4

(Continued…)

Appendix Table 2 – *Continued*

Survey Year		Total	Parity				Age in 5 year groups						
			0	1	2-3	4+	15-19	20-24	25-29	30-34	35-39	40-44	45-49
Liberia 2007	Spacing	24.2	16.9	34.5	28.7	19.4	38.8	39.4	33.2	26.7	16.7	9.8	4.6
Liberia 2007	Limiting	11.4	0.0	1.0	4.1	20.1	1.9	2.7	5.7	10.8	17.5	23.5	16.6
Liberia 2007	Total	35.7	16.9	35.5	32.7	39.5	40.7	42.2	38.9	37.5	34.2	33.4	21.2
Mali 2006	Spacing	20.3	25.0	24.0	22.4	17.4	33.3	25.7	23.2	23.1	14.0	7.8	2.3
Mali 2006	Limiting	7.3	0.4	1.4	2.3	12.7	1.5	1.3	2.4	7.1	13.8	21.5	13.7
Mali 2006	Total	27.6	25.3	25.3	24.7	30.1	34.8	26.9	25.6	30.2	27.9	29.3	16.0
Mauritania 2000-01	Spacing	23.2	14.2	30.2	28.7	20.9	31.6	30.2	31.2	24.6	18.4	10.1	6.1
Mauritania 2000-01	Limiting	8.9	1.9	4.4	5.7	13.4	3.9	4.5	7.0	9.8	11.4	13.2	14.2
Mauritania 2000-01	Total	32.1	16.1	34.5	34.5	34.4	35.5	34.7	38.2	34.3	29.8	23.2	20.3
Niger 2006	Spacing	13.4	8.1	14.6	14.8	13.4	11.1	17.0	15.6	14.9	13.9	9.3	4.0
Niger 2006	Limiting	2.7	0.1	0.1	0.3	4.6	0.1	0.2	0.3	1.0	4.3	9.0	11.7
Niger 2006	Total	16.1	8.2	14.7	15.1	18.0	11.2	17.2	15.9	15.9	18.1	18.3	15.7
Nigeria 2008	Spacing	14.5	10.2	16.1	16.3	13.9	16.2	18.8	17.9	15.7	13.1	9.3	5.6
Nigeria 2008	Limiting	5.7	0.8	1.7	2.3	9.5	1.8	1.9	2.3	4.6	8.7	12.6	11.3
Nigeria 2008	Total	20.2	10.9	17.8	18.5	23.4	18.0	20.7	20.3	20.3	21.8	21.9	16.9
Sao Tome and Principe 2008-09	Spacing	18.0	20.5	36.3	18.7	11.0	38.2	31.7	20.5	13.6	15.3	7.6	1.8
Sao Tome and Principe 2008-09	Limiting	19.6	14.1	6.3	14.9	28.6	10.1	9.7	19.1	18.7	21.4	33.5	23.8
Sao Tome and Principe 2008-09	Total	37.6	34.6	42.6	33.6	39.6	48.3	41.4	39.6	32.3	36.7	41.1	25.6
Senegal 2005	Spacing	24.3	19.7	28.1	30.4	20.8	32.3	32.3	33.6	27.7	18.6	8.1	1.6
Senegal 2005	Limiting	7.7	0.1	0.6	2.2	15.3	1.0	0.4	1.5	5.9	14.5	21.0	19.4
Senegal 2005	Total	32.0	19.9	28.7	32.7	36.1	33.3	32.7	35.1	33.6	33.1	29.2	21.0
Sierra Leone 2008	Spacing	16.1	5.2	16.6	21.1	13.8	19.9	23.9	21.8	17.7	11.3	4.0	3.6
Sierra Leone 2008	Limiting	12.2	1.9	3.0	6.5	21.4	3.2	4.4	7.2	16.9	19.1	19.2	16.0
Sierra Leone 2008	Total	28.4	7.1	19.5	27.6	35.2	23.0	28.3	29.1	34.7	30.4	23.1	19.6
**East and Southern Africa**													
Eritrea 2002	Spacing	21.6	24.6	25.7	23.2	18.5	41.4	27.9	20.0	24.6	18.8	11.4	4.9
Eritrea 2002	Limiting	6.8	0.9	1.7	1.9	13.4	2.1	1.6	2.2	5.2	12.1	14.3	16.9
Eritrea 2002	Total	28.5	25.5	27.4	25.1	31.9	43.6	29.5	22.2	29.7	30.9	25.7	21.8
Ethiopia 2005	Spacing	19.5	18.5	24.3	24.4	16.3	27.7	27.4	25.1	21.2	16.0	6.8	1.4
Ethiopia 2005	Limiting	16.6	5.9	6.9	10.2	23.2	10.1	8.9	13.0	17.8	24.6	27.7	17.6
Ethiopia 2005	Total	36.1	24.4	31.2	34.5	39.5	37.8	36.2	38.1	39.0	40.6	34.5	19.0

(Continued…)

Appendix Table 2 – *Continued*

Survey Year		Total	Parity 0	1	2-3	4+	Age in 5 year groups 15-19	20-24	25-29	30-34	35-39	40-44	45-49
Kenya 2008-09	Spacing	12.5	11.8	17.7	14.1	9.7	25.2	23.4	15.8	11.4	5.9	2.3	0.9
Kenya 2008-09	Limiting	13.1	2.5	3.4	8.2	21.3	4.5	6.9	11.1	11.1	19.1	22.1	19.0
Kenya 2008-09	Total	25.6	14.2	21.0	22.3	31.0	29.7	30.4	26.9	22.5	25.1	24.4	19.9
Lesotho 2009	Spacing	10.9	9.0	16.5	9.8	7.1	26.3	18.5	12.2	8.0	5.1	3.2	0.8
Lesotho 2009	Limiting	12.4	1.0	5.2	12.7	24.1	3.3	9.4	11.0	13.1	18.3	18.0	13.4
Lesotho 2009	Total	23.3	10.0	21.7	22.6	31.2	29.6	27.9	23.2	21.1	23.4	21.2	14.3
Madagascar 2008-09	Spacing	10.2	17.3	13.3	9.7	7.5	24.9	15.8	11.4	8.2	5.0	2.1	2.0
Madagascar 2008-09	Limiting	8.8	1.0	1.6	5.1	16.2	1.9	1.7	5.3	8.1	14.4	20.0	15.6
Madagascar 2008-09	Total	19.0	18.3	14.9	14.8	23.8	26.8	17.5	16.7	16.3	19.4	22.1	17.6
Malawi 2010	Spacing	12.4	12.1	16.7	16.4	8.2	17.2	18.4	15.2	11.6	6.9	3.9	1.4
Malawi 2010	Limiting	13.8	6.7	4.9	10.2	19.8	8.1	8.2	11.2	16.6	20.8	22.2	16.9
Malawi 2010	Total	26.2	18.8	21.6	26.6	28.1	25.2	26.6	26.4	28.1	27.7	26.1	18.3
Mozambique 2003	Spacing	10.9	6.3	12.7	13.0	9.9	16.1	15.1	12.8	10.6	7.4	4.3	3.4
Mozambique 2003	Limiting	8.0	0.4	1.1	3.4	14.6	0.8	2.0	3.3	8.9	13.1	19.4	19.9
Mozambique 2003	Total	18.9	6.7	13.8	16.4	24.5	16.9	17.1	16.1	19.5	20.5	23.7	23.3
Namibia 2006-07	Spacing	8.6	12.9	9.2	7.9	8.2	24.8	12.1	10.2	8.9	8.0	5.6	2.3
Namibia 2006-07	Limiting	12.1	3.8	7.1	10.4	18.1	9.5	8.1	8.9	12.5	15.5	12.8	15.1
Namibia 2006-07	Total	20.7	16.7	16.4	18.3	26.3	34.3	20.2	19.0	21.3	23.6	18.5	17.5
Rwanda 2005	Spacing	23.5	4.7	26.1	31.6	20.4	17.9	30.6	32.8	30.8	19.4	8.0	2.8
Rwanda 2005	Limiting	14.9	0.6	3.3	7.1	23.0	4.0	4.7	7.7	11.2	24.0	31.9	22.3
Rwanda 2005	Total	38.5	5.3	29.4	38.7	43.4	21.9	35.2	40.5	42.1	43.4	39.9	25.1
Swaziland 2006-07	Spacing	6.7	12.1	12.8	6.6	3.8	13.6	16.8	8.3	6.1	2.2	1.5	0.4
Swaziland 2006-07	Limiting	18.1	2.9	9.4	15.4	25.6	10.9	12.6	16.1	14.3	23.2	28.4	17.7
Swaziland 2006-07	Total	24.7	15.0	22.2	21.9	29.4	24.6	29.4	24.4	20.3	25.5	30.0	18.1
Tanzania 2010	Spacing	15.9	3.2	16.8	18.3	15.6	15.3	23.8	21.5	16.8	13.7	5.4	2.1
Tanzania 2010	Limiting	9.4	0.0	0.8	3.1	17.8	1.0	1.0	2.6	5.1	14.1	24.3	30.3
Tanzania 2010	Total	25.3	3.2	17.6	21.4	33.3	16.3	24.8	24.0	21.9	27.9	29.7	32.4
Uganda 2006	Spacing	23.7	15.4	25.1	30.8	21.1	32.7	32.6	32.1	25.8	15.1	5.0	2.1
Uganda 2006	Limiting	14.3	1.2	1.0	4.2	22.3	1.7	2.9	7.9	17.3	29.1	32.0	18.8
Uganda 2006	Total	38.0	16.7	26.1	35.0	43.5	34.4	35.5	39.9	43.1	44.1	37.0	20.9

(Continued…)

Appendix Table 2 – *Continued*

			Parity				Age in 5 year groups						
**Survey Year**		**Total**	**0**	**1**	**2-3**	**4+**	**15-19**	**20-24**	**25-29**	**30-34**	**35-39**	**40-44**	**45-49**
Zambia 2007	Spacing	15.9	10.0	14.8	18.5	15.3	15.9	21.6	20.7	19.0	10.3	6.4	1.2
Zambia 2007	Limiting	10.6	1.9	2.0	6.0	16.5	6.7	3.3	5.3	8.7	19.7	25.3	20.8
Zambia 2007	Total	26.6	12.0	16.8	24.6	31.8	22.6	24.9	26.0	27.6	29.9	31.6	22.0
Zimbabwe 2005-06	Spacing	7.2	12.8	8.4	5.9	6.6	13.1	10.3	6.8	6.0	4.8	4.1	2.9
Zimbabwe 2005-06	Limiting	8.2	2.9	4.4	5.3	16.0	5.6	3.4	3.7	7.1	10.6	19.9	23.6
Zimbabwe 2005-06	Total	15.5	15.7	12.8	11.3	22.6	18.8	13.7	10.4	13.2	15.4	24.0	26.6
**Middle East/North Africa**													
Egypt 2008	Spacing	3.4	0.6	9.0	3.7	1.2	6.0	6.9	5.4	3.2	1.5	0.7	0.1
Egypt 2008	Limiting	8.2	0.0	1.2	8.0	14.6	1.0	2.3	5.4	8.2	10.4	13.3	14.7
Egypt 2008	Total	11.6	0.6	10.2	11.7	15.8	7.0	9.2	10.8	11.4	11.9	14.0	14.8
Jordan 2009	Spacing	6.0	2.6	13.5	9.1	2.8	8.1	11.5	11.8	6.9	3.2	1.0	0.5
Jordan 2009	Limiting	7.4	1.5	2.1	4.1	11.8	0.3	3.2	2.0	4.4	7.9	11.8	20.1
Jordan 2009	Total	13.4	4.2	15.6	13.3	14.6	8.4	14.7	13.7	11.3	11.1	12.7	20.6
Morocco 2003-04	Spacing	4.4	5.7	8.1	5.3	2.0	10.1	8.7	8.0	5.6	2.7	1.0	0.5
Morocco 2003-04	Limiting	7.4	0.1	2.0	5.9	12.6	0.2	1.3	3.6	6.1	8.8	11.4	13.7
Morocco 2003-04	Total	11.9	5.8	10.1	11.2	14.7	10.3	10.0	11.6	11.7	11.5	12.4	14.2
**Eastern Europe/NIS**													
Albania 2008-09	Spacing	3.5	8.6	11.6	1.9	1.2	16.6	14.9	9.3	3.6	1.5	0.4	0.1
Albania 2008-09	Limiting	9.4	0.0	5.1	10.8	11.1	0.0	2.9	9.7	9.4	10.3	11.5	9.0
Albania 2008-09	Total	12.9	8.6	16.7	12.7	12.3	16.6	17.7	19.0	13.0	11.8	11.9	9.1
Armenia 2005	Spacing	3.9	4.1	13.4	2.4	0.6	15.7	13.2	6.5	3.1	1.4	0.2	0.5
Armenia 2005	Limiting	15.5	0.8	5.6	17.7	22.9	0.0	6.1	12.8	12.3	15.7	20.7	22.6
Armenia 2005	Total	19.3	4.9	19.0	20.1	23.6	15.7	19.4	19.2	15.4	17.1	20.9	23.1
Azerbaijan 2006	Spacing	3.0	2.6	10.5	1.9	0.4	11.4	9.2	5.8	2.5	0.6	0.1	0.0
Azerbaijan 2006	Limiting	12.5	0.4	4.1	14.3	19.8	5.0	6.6	9.0	12.5	12.0	16.5	17.6
Azerbaijan 2006	Total	15.4	3.0	14.7	16.2	20.2	16.4	15.8	14.8	15.0	12.7	16.6	17.6
Moldova 2005	Spacing	3.1	7.8	6.0	1.3	0.2	12.7	9.1	6.4	2.7	0.6	0.2	0.3
Moldova 2005	Limiting	8.2	1.4	6.1	10.2	11.0	1.5	3.7	3.9	4.9	8.5	12.6	14.5
Moldova 2005	Total	11.4	9.2	12.1	11.5	11.2	14.2	12.8	10.2	7.5	9.0	12.8	14.8

(Continued…)

Appendix Table 2 – *Continued*

Survey Year		Total	Parity: 0	1	2-3	4+	Age in 5 year groups: 15-19	20-24	25-29	30-34	35-39	40-44	45-49
Turkey 2003	Spacing	3.1	10.6	8.1	1.1	0.8	16.1	11.0	3.7	1.2	0.9	0.1	0.0
Turkey 2003	Limiting	6.4	0.8	1.2	5.7	13.8	4.7	4.1	4.3	4.9	6.3	9.3	11.0
Turkey 2003	Total	9.5	11.3	9.3	6.8	14.6	20.9	15.1	8.0	6.1	7.2	9.4	11.0
Ukraine 2007	Spacing	3.7	5.3	4.8	2.2	3.8	29.2	8.9	7.7	3.7	1.9	0.3	0.2
Ukraine 2007	Limiting	6.4	0.7	5.2	9.1	8.4	1.4	1.3	3.0	3.4	6.8	11.0	11.3
Ukraine 2007	Total	10.1	6.0	9.9	11.3	12.3	30.6	10.3	10.7	7.1	8.7	11.3	11.5
**Asia**													
Bangladesh 2007	Spacing	6.7	14.9	14.2	4.8	1.6	19.0	12.2	5.9	2.1	1.1	0.4	0.0
Bangladesh 2007	Limiting	10.1	0.1	2.4	11.2	16.9	0.5	4.9	11.7	16.1	15.4	15.7	8.5
Bangladesh 2007	Total	16.8	15.0	16.6	16.0	18.5	19.5	17.1	17.5	18.2	16.5	16.1	8.5
Cambodia 2010	Spacing	6.1	7.0	13.2	6.4	1.5	14.1	13.1	10.0	5.8	2.4	1.4	0.4
Cambodia 2010	Limiting	10.8	0.6	3.4	9.1	19.6	1.9	4.1	6.4	9.5	13.9	17.3	17.9
Cambodia 2010	Total	16.9	7.6	16.6	15.4	21.0	16.0	17.2	16.4	15.3	16.3	18.7	18.3
India 2005-06	Spacing	6.1	13.0	16.6	3.9	1.8	24.7	14.7	5.9	2.0	0.6	0.1	0.1
India 2005-06	Limiting	7.8	0.5	4.1	7.0	13.3	2.4	6.8	10.5	10.1	8.7	6.8	3.6
India 2005-06	Total	13.9	13.5	20.7	11.0	15.1	27.1	21.5	16.5	12.1	9.3	6.9	3.7
Indonesia 2007	Spacing	4.8	3.8	7.3	4.4	3.3	6.4	7.9	8.1	5.8	3.4	2.1	1.1
Indonesia 2007	Limiting	8.3	1.5	2.8	7.8	18.2	2.9	2.1	2.2	4.6	9.0	16.7	17.8
Indonesia 2007	Total	13.1	5.3	10.1	12.2	21.5	9.3	10.0	10.3	10.4	12.4	18.7	18.9
Maldives 2009	Spacing	15.0	13.1	30.1	15.3	2.7	33.6	26.3	25.6	13.9	6.3	2.4	0.3
Maldives 2009	Limiting	13.6	0.3	5.5	17.3	23.1	3.3	5.8	9.0	17.1	18.5	21.0	15.7
Maldives 2009	Total	28.6	13.3	35.6	32.6	25.8	36.9	32.0	34.6	31.0	24.8	23.4	16.0
Nepal 2006	Spacing	9.3	26.4	25.5	5.7	1.5	34.7	20.5	8.4	1.2	1.0	0.3	0.1
Nepal 2006	Limiting	15.4	0.1	7.4	17.7	20.4	3.2	12.7	18.3	20.4	21.1	15.6	9.9
Nepal 2006	Total	24.7	26.6	32.9	23.4	21.9	37.8	33.2	26.7	21.6	22.1	15.9	10.0
Pakistan 2006-07	Spacing	10.8	7.8	20.8	16.7	6.2	18.8	21.9	16.6	9.4	5.3	2.1	0.8
Pakistan 2006-07	Limiting	14.4	0.4	1.3	10.1	22.9	1.4	5.3	10.5	19.3	21.7	21.0	14.7
Pakistan 2006-07	Total	25.2	8.2	22.1	26.8	29.1	20.2	27.1	27.1	28.7	26.9	23.1	15.5
Philippines 2008	Spacing	8.5	9.9	17.0	7.8	4.1	27.8	19.1	13.7	8.0	5.3	1.5	0.7
Philippines 2008	Limiting	13.5	0.8	6.2	13.9	20.4	6.0	5.5	11.5	12.2	16.9	18.4	16.5
Philippines 2008	Total	22.0	10.7	23.2	21.7	24.5	33.7	24.6	25.2	20.2	22.3	19.9	17.2

(Continued…)

Appendix Table 2 – *Continued*

Survey Year		Total	Parity: 0	1	2-3	4+	Age in 5 year groups: 15-19	20-24	25-29	30-34	35-39	40-44	45-49
Samoa 2009	Spacing	20.6	49.0	37.0	21.2	9.8	49.9	32.7	26.5	19.9	19.1	10.2	12.6
Samoa 2009	Limiting	27.2	2.8	14.1	28.7	34.6	2.4	9.9	18.2	22.4	29.5	39.5	44.0
Samoa 2009	Total	47.7	51.8	51.2	49.9	44.4	52.3	42.5	44.6	42.3	48.6	49.8	56.6
Timor-Leste 2009	Spacing	20.9	12.7	31.6	25.6	17.3	27.2	34.0	27.9	22.7	19.0	11.7	4.9
Timor-Leste 2009	Limiting	10.6	0.0	0.8	3.9	16.7	0.2	1.3	4.7	9.3	15.2	19.8	16.3
Timor-Leste 2009	Total	31.5	12.7	32.5	29.4	34.0	27.4	35.2	32.6	32.0	34.2	31.5	21.2
Vietnam 2002	Spacing	2.3	5.9	7.2	1.1	0.1	13.6	8.7	3.6	1.3	1.2	0.2	0.1
Vietnam 2002	Limiting	4.4	0.0	1.6	4.6	7.1	1.3	1.6	4.0	5.3	3.5	4.1	7.8
Vietnam 2002	Total	6.6	5.9	8.8	5.7	7.2	14.9	10.3	7.6	6.6	4.6	4.2	7.9
**Latin America and Caribbean**													
Bolivia 2008	Spacing	6.2	17.0	12.1	6.5	2.0	26.6	14.3	8.7	5.3	2.2	0.1	0.3
Bolivia 2008	Limiting	14.0	3.3	5.2	11.4	21.5	11.3	12.9	15.3	14.9	15.4	15.5	8.8
Bolivia 2008	Total	20.1	20.4	17.3	17.9	23.5	37.9	27.2	23.9	20.2	17.6	15.7	9.0
Colombia 2010	Spacing	3.6	13.4	6.3	1.8	0.7	19.2	9.9	4.5	2.4	0.9	0.4	0.4
Colombia 2010	Limiting	4.4	2.0	3.6	4.4	6.7	4.5	4.3	3.5	3.7	4.5	5.1	5.7
Colombia 2010	Total	8.0	15.3	9.9	6.2	7.4	23.7	14.1	8.0	6.1	5.4	5.5	6.1
Dominican Republic 2007	Spacing	6.7	17.6	14.8	4.7	1.8	25.0	15.7	10.5	3.8	1.4	0.2	0.6
Dominican Republic 2007	Limiting	4.4	0.7	2.7	4.6	6.5	2.2	3.0	4.9	5.2	4.5	5.8	3.7
Dominican Republic 2007	Total	11.1	18.3	17.5	9.2	8.3	27.2	18.7	15.5	9.0	5.9	6.0	4.4
Guyana 2009	Spacing	9.4	13.9	18.4	9.1	2.6	23.0	21.6	17.0	7.9	3.9	2.4	0.4
Guyana 2009	Limiting	19.1	0.6	5.2	21.5	30.5	11.9	8.5	12.4	18.4	25.6	23.7	26.8
Guyana 2009	Total	28.5	14.5	23.7	30.6	33.1	34.9	30.1	29.4	26.3	29.5	26.1	27.2
Haiti 2005-06	Spacing	16.5	28.8	32.2	15.5	5.1	49.4	34.0	19.8	11.8	5.9	2.0	0.6
Haiti 2005-06	Limiting	20.8	0.2	3.0	19.7	37.8	2.8	6.5	16.9	25.6	33.5	35.8	20.9
Haiti 2005-06	Total	37.3	28.9	35.3	35.2	42.8	52.1	40.5	36.7	37.4	39.3	37.8	21.6
Honduras 2005-06	Spacing	8.0	16.5	15.6	7.3	3.5	20.7	15.5	10.1	5.9	2.6	0.8	0.2
Honduras 2005-06	Limiting	8.8	1.4	3.4	8.0	13.6	5.1	6.9	8.3	9.1	12.6	9.8	8.9
Honduras 2005-06	Total	16.8	17.8	19.0	15.3	17.1	25.8	22.4	18.3	15.0	15.2	10.6	9.1
Nicaragua 2001	Spacing	5.6	13.9	10.2	4.5	2.9	13.4	10.7	6.8	4.0	2.4	1.0	0.1
Nicaragua 2001	Limiting	9.0	5.2	3.8	7.5	13.5	6.5	6.5	9.4	8.4	9.9	12.7	10.2
Nicaragua 2001	Total	14.6	19.1	14.0	12.1	16.4	19.9	17.2	16.2	12.5	12.3	13.7	10.3

(Continued…)

Appendix Table 2 – *Continued*

			Parity				Age in 5 year groups						
**Survey Year**		**Total**	**0**	**1**	**2-3**	**4+**	**15-19**	**20-24**	**25-29**	**30-34**	**35-39**	**40-44**	**45-49**
Peru 2004-08	Spacing	5.0	16.0	9.1	4.0	1.7	22.0	13.4	7.5	5.2	2.1	0.7	0.2
Peru 2004-08	Limiting	7.4	0.6	2.4	7.1	12.4	3.3	4.6	6.1	8.2	8.6	9.2	7.5
Peru 2004-08	Total	12.4	16.6	11.6	11.2	14.1	25.3	18.0	13.6	13.4	10.7	9.8	7.7

DHS Analytical Studies Series

1. Westoff, Charles F. 2000. *The Substitution of Contraception for Abortion in Kazakhstan in the 1990s.*
2. Rafalimanana, Hantamalala, and Charles F. Westoff. 2001. *Gap between Preferred and Actual Birth Intervals in Sub-Saharan Africa: Implications for Fertility and Child Health.*
3. Mahy, Mary, and Neeru Gupta. 2002. *Trends and Differentials in Adolescent Reproductive Behavior in Sub-Saharan Africa.*
4. Westoff, Charles F., and Akinrinola Bankole. 2001. *The Contraception- Fertility Link in Sub-Saharan Africa and in Other Developing Countries.*
5. Yoder, P. Stanley, and Mary Mahy. 2001. *Female Genital Cutting in Guinea: Qualitative and Quantitative Research Strategies.*
6. Westoff, Charles F., Jeremiah M. Sullivan, Holly A. Newby, and Albert R. Themme. 2002. *Contraception-Abortion Connections in Armenia.*
7. Bell, Jacqueline, Siân L. Curtis, and Silvia Alayón. 2003. *Trends in Delivery Care in Six Countries.*
8. Westoff, Charles F. 2005. *Recent Trends in Abortion and Contraception in 12 Countries.*
9. Westoff, Charles F., and Anne R. Cross. 2006. *The Stall in the Fertility Transition in Kenya.*
10. Gebreselassie, Tesfayi. 2008. *Spousal Agreement on Reproductive Preferences in Sub-Saharan Africa.*
11. Gebreselassie, Tesfayi, and Vinod Mishra. 2007. *Spousal Agreement on Family Planning in Sub-Saharan Africa.*
12. Mishra, Vinod, Rathavuth Hong, Shane Khan, Yuan Gu, and Li Liu. 2008. *Evaluating HIV Estimates from National Population-Based Surveys for Bias Resulting from Non-Response.*
13. Westoff, Charles F. 2008. *A New Approach to Estimating Abortion Rates.*
14. Gebreselassie, Tesfayi, Shea O. Rutstein, and Vinod Mishra. 2008. *Contraceptive Use, Breastfeeding, Amenorrhea and Abstinence during the Postpartum Period: An Analysis of Four Countries.*
15. Mishra, Vinod, and Simona Bignami-Van Assche. 2008. *Orphans and Vulnerable Children in High HIV-Prevalence Countries in Sub-Saharan Africa.*
16. Bradley, Sarah E.K., and Vinod Mishra. 2008. *HIV and Nutrition among Women in Sub-Saharan Africa.*
17. Johnson, Kiersten, and Amber Peterman. 2008. *Incontinence Data from the Demographic and Health Surveys: Comparative Analysis of a Proxy Measurement of Vaginal Fistula and Recommendations for Future Population-Based Data Collection.*
18. Hindin, Michelle J., Sunita Kishor, and Donna L. Ansara. 2008. *Intimate Partner Violence among Couples in 10 DHS Countries: Predictors and Health Outcomes.*
19. Johnson, Kiersten, Monica Grant, Shane Khan, Zhuzhi Moore, Avril Armstrong, and Zhihong Sa. 2009. *Fieldwork-Related Factors and Data Quality in the Demographic and Health Surveys Program.*
20. Bradley, Sarah E.K., Hilary M. Schwandt, and Shane Khan. 2009. *Levels, Trends, and Reasons for Contraceptive Discontinuation.*
21. Westoff, Charles F. and Dawn Koffman. 2010. *Birth Spacing and Limiting Connections.*
22. Bradley, Sarah E.K., Trevor N. Croft, and Shea O. Rutstein. 2011. *The Impact of Contraceptive Failure on Unintended Births and Induced Abortions: Estimates and Strategies for Reduction*.
23. Johnson, Kiersten, Noureddine Abderrahim, and Shea Rutstein. 2011. *Changes in the Direct and Indirect Determinants of Fertility in Sub-Saharan Africa.*
24. Westoff, Charles F., Dawn A. Koffman and Caroline Moreau. 2011. *The Impact of Television and Radio on Reproductive Behavior and on HIV/AIDS Knowledge and Behavior.*
25. Bradley, Sarah E.K., Trevor N. Croft, Joy D. Fishel, and Charles F. Westoff. 2012. *Revising Unmet Need for Family Planning.*
