# Supplementary material for: Factors influencing unmet need for contraception amongst adolescent girls and women in Cambodia
Source: PeerJ. 2020 Oct 7;8:e10065. doi: 10.7717/peerj.10065 (PMC7547592; doi:10.7717/peerj.10065)
Supplement: Supplemental Information 11 [file peerj-08-10065-s011.pdf]

Cambodia

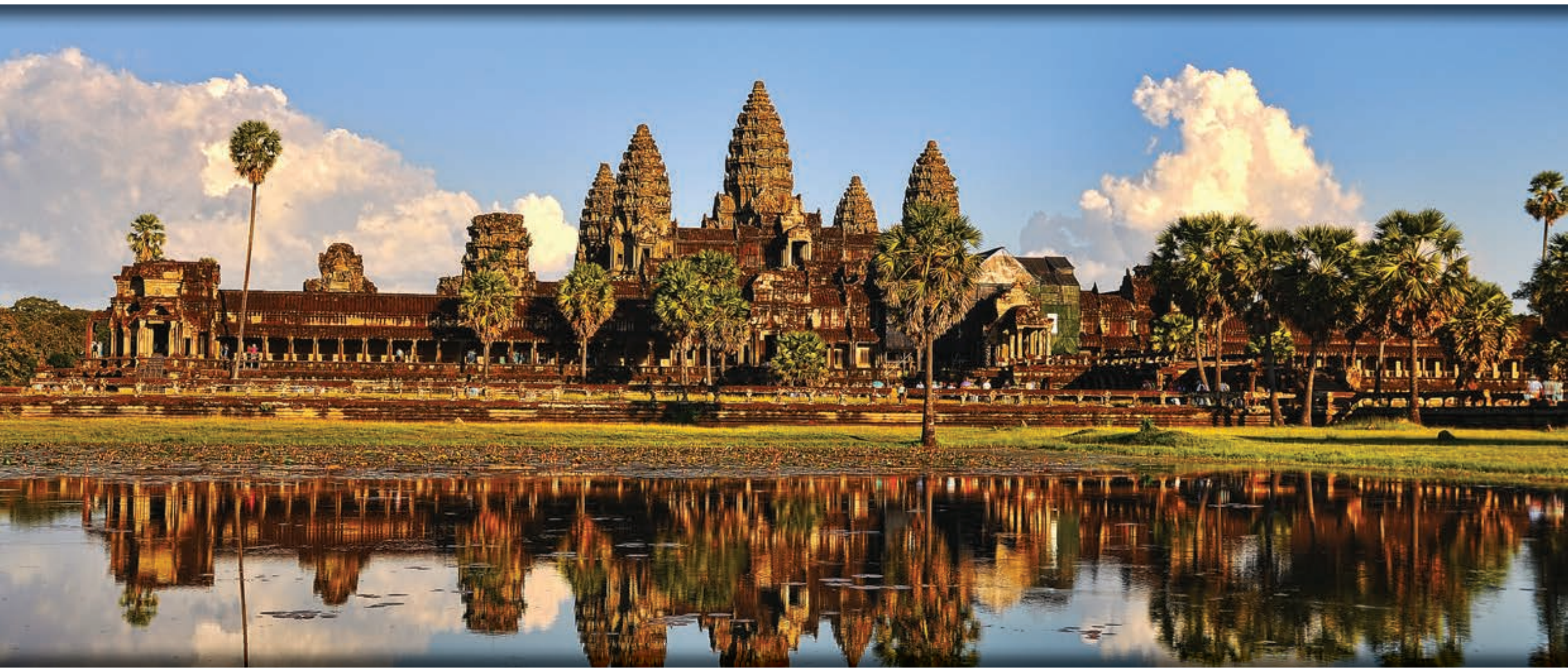

Demographic and Health Survey

2014

Cambodia
Demographic and Health Survey
2014

National Institute of Statistics
Ministry of Planning
Phnom Penh, Cambodia

Directorate General for Health
Ministry of Health
Phnom Penh, Cambodia

The DHS Program
ICF International
Rockville, Maryland, USA

September 2015

The analysis of the *Cambodia Demographic and Health Survey 2014* was achieved through the joint efforts of:

Sok Kosal, NIS/MoP
Chhay Satia, NIS/MoP
They Kheam, NIS/MoP
Phan Chinda, NIS/MoP
Loun Mondol, DGH/MoH
Lam Phirun, DGH/MoH
Rathavuth Hong, ICF International
Bernard Barrère, ICF International
Anne Cross, ICF International
Sunita Kishor, ICF International

See Appendix D for a list of contributors to the implementation of the CDHS.

The 2014 Cambodia Demographic and Health Survey (2014 CDHS) is part of The DHS Program, a worldwide project which assists countries in the collection of data to monitor and evaluate population, health, and nutrition programs. Funding was provided by the Royal Government of Cambodia (RGC), the United States Agency for International Development (USAID), the Australian Department of Foreign Affairs and Trade (Australia-DFAT), the United Nations Population Fund (UNFPA), the United Nations Children's Fund (UNICEF), the Japan International Cooperation Agency (JICA), the Korean International Cooperation Agency (KOICA), and the Health Sector Support Program—Second Phase (HSSP-2).

Additional information about the survey can be obtained from the National Institute of Statistics; 386 Monivong Boulevard, Sangkat Beong Keng Kang 1, Chamkar Mon, Phnom Penh, Cambodia; Telephone: (855) 23-213650; E-mail: ssythan@hotmail.com; Internet: www.nis.gov.kh and the Directorate General for Health, Ministry of Health 80 Samdech Penn Nouth Boulevard (289), Sangkat Boeungkak 2, Tuol Kork, Phnom Penh, Cambodia; Telephone: (855) 23-885970/23-884909; E-mail: webmaster@moh.gov.kh; Internet: www.moh.gov.kh. Additional information about The DHS Program can be obtained from ICF International, 530 Gaither Road, Suite 500, Rockville, MD 20850 USA; Telephone: 301-572-0200, Fax: 301-572-0999, E-mail: info@DHSprogram.com, Internet: www.DHSprogram.com.

Suggested citation:

National Institute of Statistics, Directorate General for Health, and ICF International, 2015. *Cambodia Demographic and Health Survey 2014.* Phnom Penh, Cambodia, and Rockville, Maryland, USA: National Institute of Statistics, Directorate General for Health, and ICF International.

Cover photo of Angkor Wat temple ©2014 J.H. Tan.

CONTENTS

TABLES AND FIGURES .... **ix**
FOREWORD .... **xvii**
ACKNOWLEDGMENTS .... **xix**
MAP OF CAMBODIA .... **xx**

1 INTRODUCTION **1**
1.1 Geodemography, History, and Economy 1
1.1.1 Geodemography 1
1.1.2 History 2
1.1.3 Economy 2
1.2 Health Status and Policy 2
1.3 Objective and Survey Organization 4
1.4 Sample Design 4
1.5 Questionnaires 5
1.6 Training and Fieldwork 7
1.7 Biomarker Testing 7
1.7.1 Anthropometric Measurement 7
1.7.2 Hemoglobin Testing 8
1.7.3 Micronutrient Testing 8
1.8 Data Processing 8
1.9 Sample Coverage 8

2 HOUSEHOLD POPULATION AND HOUSING CHARACTERISTICS **11**
2.1 Characteristics of the Household Population 11
2.1.1 Age and Sex Composition 11
2.1.2 Household Composition 13
2.2 Education of the Household Population 14
2.3 Housing Characteristics 17
2.3.1 Water Supply 18
2.3.2 Sanitation Facilities 20
2.3.3 Hand Washing 21
2.3.4 Flooring Material and Cooking Arrangements 22
2.4 Household Possessions 23
2.5 Household Wealth 23
2.6 Birth Registration 24
2.7 Children's Living Arrangements, Orphanhood, and School Attendance by Survivorship of Parents 25
2.7.1 Children's Living Arrangements and Orphanhood 25
2.7.2 School Attendance by Survivorship of Parents 27

3 UTILIZATION OF HEALTH SERVICES FOR ACCIDENT, ILLNESS, OR INJURY **29**
3.1 Accidental Death or Injury 29
3.1.1 Frequency of Accidental Death or Injury 29
3.1.2 Type of Accident 30
3.2 Prevalence and Severity of Illness or Injury 32
3.3 Treatment Sought for Illness or Injury 33
3.4 Utilization of Health Care Facilities 34

3.5 Cost for Health Care 36
3.5.1 Distribution of Cost for Health Care 36
3.5.2 Expenditures for Health Care 36
3.5.3 Sources of Money for Health Care Expenditures 38

4 DISABILITY 41
4.1 Disability among the General Household Population 41
4.2 Disability among Ill or Injured Household Members 43
4.3 Disability and Employment 44

5 RESPONDENT CHARACTERISTICS 47
5.1 Characteristics of Survey Respondents 47
5.2 Educational Attainment and Literacy 49
5.3 Access to Mass Media 52
5.4 Employment 55
5.4.1 Employment Status 55
5.4.2 Occupation 57
5.4.3 Earnings, Employers, and Continuity of Employment 59
5.5 Health Insurance 60
5.6 Use of Tobacco 62

6 FERTILITY 67
6.1 Current Fertility Levels and Differentials 67
6.2 Fertility Trends 70
6.2.1 Comparison of Current and Cumulative Fertility Levels 70
6.2.2 Retrospective Data 70
6.2.3 Comparison with Previous CDHS 71
6.3 Children Ever Born and Living 72
6.4 Birth Intervals 73
6.5 Age at First Birth 75
6.6 Teenage Pregnancy and Motherhood 76

7 PRACTICE OF ABORTION 79
7.1 Number of Lifetime Induced Abortions 79
7.2 Practice of Abortion in the Past Five Years 81
7.2.1 Pregnancy Duration at the Time of Abortion 81
7.2.2 Place of Abortion 82
7.2.3 Persons Who Helped with the Abortion 83
7.2.4 Method Used for the Abortion 83

8 FAMILY PLANNING 85
8.1 Knowledge of Contraceptive Methods 85
8.2 Current Use of Contraceptive Methods 86
8.3 Use of Social Marketing Brands 89
8.4 Knowledge of Fertile Period 90
8.5 Timing of Sterilization 91
8.6 Source of Family Planning Methods 91
8.7 Informed Choice 92
8.8 Future Use of Contraception 93
8.9 Exposure to Family Planning Messages 94
8.10 Contact of Nonusers with Family Planning Providers 95

9 OTHER PROXIMATE DETERMINANTS OF FERTILITY 97
9.1 Marital Status 97
9.2 Polygamy 98
9.3 Age at First Union 99
9.4 Age at First Sexual Intercourse 102
9.5 Recent Sexual Activity 105
9.6 Postpartum Amenorrhea, Abstinence, and Insusceptibility 108
9.7 Termination of Exposure to Pregnancy 109

10 FERTILITY PREFERENCES 111
10.1 Desire for More Children 111
10.2 Need and Demand for Family Planning Services 114
10.3 Ideal Family Size 116
10.4 Fertility Planning 118

11 ADULT AND MATERNAL MORTALITY 121
11.1 Data Quality Issues 121
11.2 Adult Mortality 123
11.3 Maternal Mortality 123

12 INFANT AND CHILD MORTALITY 127
12.1 Assessment of Data Quality 128
12.2 Levels and Trends in Childhood Mortality 128
12.3 Socioeconomic Differentials in Childhood Mortality 129
12.4 Demographic Differentials in Mortality 131
12.5 Perinatal Mortality 132
12.6 High-Risk Fertility Behavior 133

13 MATERNAL HEALTH 137
13.1 Antenatal Care 137
13.1.1 Source of Antenatal Care 137
13.1.2 Components of Antenatal Care 139
13.1.3 Tetanus Toxoid Vaccinations 141
13.2 Childbirth and Delivery 141
13.2.1 Place of Delivery 142
13.2.2 Assistance at Delivery 143
13.3 Postnatal Care and Practices 144
13.4 Perceived Problems in Accessing Women’s Health Care 149

14 CHILD HEALTH 151
14.1 Child’s Size at Birth 151
14.2 Immunization of Children 152
14.3 Acute Respiratory Infection 155
14.4 Fever 157
14.5 Diarrhea 158
14.6 Feeding Practices 161
14.7 Knowledge of ORS Packets 163
14.8 Stool Disposal 163

15 EARLY CHILDHOOD EDUCATION AND DEVELOPMENT 165
15.1 Early Childhood Education and Learning 165
15.2 Adequate Care for Young Children 169
15.3 Early Childhood Development 170

16 NUTRITION OF CHILDREN AND WOMEN 173
16.1 Nutritional Status of Children 174
16.1.1 Measurement of Nutritional Status among Young Children 174
16.1.2 Measures of Child Nutritional Status 175
16.1.3 Trends in Children's Nutritional Status 177
16.2 Initiation of Breastfeeding 178
16.3 Breastfeeding Status by Age 180
16.4 Duration of Breastfeeding 181
16.5 Types of Complementary Foods 183
16.6 Infant and Young Child Feeding (IYCF) Practices 184
16.7 Prevalence of Anemia in Children 187
16.8 Micronutrient Intake among Children 189
16.9 Use of Iodized Salt 191
16.10 Nutritional Status of Women 192
16.11 Prevalence of Anemia in Women 194
16.12 Micronutrient Intake among Mothers 196

17 MICRONUTRIENTS 199
17.1 Coverage of Micronutrient Testing 200
17.2 Iron, Hemoglobin, and Parasitic Infections 200
17.2.1 Anemia and Iron Status in Mothers 201
17.2.2 Anemia and Iron Status in Children 201
17.2.3 Intestinal Parasite Infection 203
17.3 Vitamin and Calcium Deficiency 203
17.3.1 Vitamin and Calcium Deficiency among Mothers 203
17.3.2 Vitamin and Calcium Deficiency among Children 203
17.4 Urine Iodine Concentration 205

18 HIV/AIDS-RELATED KNOWLEDGE, ATTITUDES, AND BEHAVIOR 207
18.1 Knowledge of HIV/AIDS and of Transmission and Prevention Methods 207
18.1.1 Awareness of AIDS 207
18.1.2 HIV Prevention Methods 208
18.1.3 Knowledge about Transmission 210
18.1.4 Knowledge of Mother-to-Child Transmission 213
18.2 Stigma Associated with AIDS and Attitudes Related to HIV/AIDS 215
18.3 Attitudes towards Negotiating Safer Sex 217
18.4 Multiple Sexual Partnerships 218
18.5 Testing for HIV 223
18.6 Reports of Recent Sexually Transmitted Infections 227
18.7 Injections 229
18.8 HIV/AIDS-Related Knowledge and Behavior among Youth 230
18.8.1 Knowledge about HIV/AIDS and Source for Condoms 231
18.8.2 Age at First Sex and Condom Use at First Sexual Intercourse 232
18.8.3 Recent Sexual Activity 234
18.8.4 Multiple Sexual Partnerships 235
18.8.5 HIV Testing 236

19 WOMEN'S EMPOWERMENT AND DEMOGRAPHIC AND HEALTH OUTCOMES ... 239
19.1 Employment and Forms of Earnings 239
19.2 Control over Women's and Men's Earnings 240
19.3 Participation in Household Decision Making 243
19.4 Ownership of Assets 247
19.5 Attitudes towards Wife Beating 249

19.6 Women's Empowerment Indicators 252
19.7 Current Use of Contraception by Women's Status 253
19.8 Ideal Family Size and Unmet Need by Women's Status 254
19.9 Reproductive Health Care and Women's Empowerment Status 255

20 DOMESTIC VIOLENCE 257
20.1 Measurement of Violence 257
20.1.1 Use of Valid Measures of Violence 257
20.1.2 Ethical Considerations in the 2014 CDHS 258
20.1.3 Subsample for the Violence Module 259
20.2 Experience of Physical Violence 259
20.3 Perpetrators of Physical Violence 261
20.4 Experience of Sexual Violence 262
20.5 Perpetrators of Sexual Violence 262
20.6 Age at First Experience of Sexual Violence 263
20.7 Experience of Different Forms of Violence 263
20.8 Violence during Pregnancy 264
20.9 Marital Control by Spouse 266
20.10 Forms of Spousal Violence 268
20.11 Spousal Violence by Background Characteristics 269
20.12 Violence by Spousal Characteristics and Women's Empowerment Indicators 271
20.13 Recent Spousal Violence by Any Husband or Partner 273
20.14 Onset of Spousal Violence 273
20.15 Physical Consequences of Spousal Violence 274
20.16 Violence by Women against Their Husband 274
20.17 Help-seeking Behavior by Women Who Experience Violence 276

REFERENCES 279

APPENDIX A: SAMPLE IMPLEMENTATION 281
A.1 Introduction 281
A.2 Sampling Frame 281
A.3 Sampling Methodology and Procedure 282
A.4 Sampling Probabilities 284

APPENDIX B: ESTIMATES OF SAMPLING ERRORS 291

APPENDIX C: DATA QUALITY TABLES 317

APPENDIX D: PERSONS INVOLVED IN THE 2014 CAMBODIA DEMOGRAPHIC AND HEALTH SURVEY 323

APPENDIX E: QUESTIONNAIRES 327

TABLES AND FIGURES

1	**INTRODUCTION**		**1**
	Table 1.1	Results of the household and individual interviews	9
2	**HOUSEHOLD POPULATION AND HOUSING CHARACTERISTICS**		**11**
	Table 2.1	Household population by age, sex, and residence	12
	Table 2.2	Population by age according to selected sources	13
	Table 2.3	Household composition	13
	Table 2.4.1	Educational attainment of the female household population	14
	Table 2.4.2	Educational attainment of the male household population	15
	Table 2.5	School attendance ratios	16
	Table 2.6	Household drinking water	19
	Table 2.7	Household sanitation facilities	20
	Table 2.8	Hand washing	21
	Table 2.9	Household characteristics	22
	Table 2.10	Household possessions	23
	Table 2.11	Wealth quintiles	24
	Table 2.12	Birth registration of children under age 5	25
	Table 2.13	Children's living arrangements and orphanhood	26
	Table 2.14	School attendance by survivorship of parents	27
	Figure 2.1	Population pyramid	12
	Figure 2.2	Age-specific attendance rates	17
3	**UTILIZATION OF HEALTH SERVICES FOR ACCIDENT, ILLNESS, OR INJURY**		**29**
	Table 3.1	Injury or death in an accident	30
	Table 3.2	Injury or death in an accident by type of accident	31
	Table 3.3	Prevalence and severity of illness or injury in previous 30 days	32
	Table 3.4	Percentage of ill or injured population who sought treatment	33
	Table 3.5	Percentage of ill or injured population who sought treatment	35
	Table 3.6	Distribution of cost for health care	36
	Table 3.7	Expenditures for health care	37
	Table 3.8	Source of money (United States dollars) spent by persons who sought treatment for health care	39
	Figure 3.1	Percentage of ill or injured household members seeking treatment by order of treatment and sector of health care	35
4	**DISABILITY**		**41**
	Table 4.1	Disability among the household population	42
	Table 4.2	Disability among the ill or injured population	43
	Table 4.3	Disability and employment	44
5	**RESPONDENT CHARACTERISTICS**		**47**
	Table 5.1	Background characteristics of respondents	48
	Table 5.2.1	Educational attainment: Women	49
	Table 5.2.2	Educational attainment: Men	50
	Table 5.3.1	Literacy: Women	51
	Table 5.3.2	Literacy: Men	52

Table 5.4.1 Exposure to mass media: Women ... 53
Table 5.4.2 Exposure to mass media: Men ... 54
Table 5.5.1 Employment status: Women ... 55
Table 5.5.2 Employment status: Men ... 56
Table 5.6.1 Occupation: Women ... 58
Table 5.6.2 Occupation: Men ... 59
Table 5.7 Type of employment: Women ... 60
Table 5.8.1 Health insurance coverage: Women ... 61
Table 5.8.2 Health insurance coverage: Men ... 62
Table 5.9.1 Use of tobacco: Women ... 63
Table 5.9.2 Use of tobacco: Men ... 64

6 FERTILITY ... 67
Table 6.1 Current fertility ... 68
Table 6.2 Fertility by background characteristics ... 69
Table 6.3.1 Trends in age-specific fertility rates ... 70
Table 6.3.2 Trends in fertility ... 71
Table 6.4 Children ever born and living ... 73
Table 6.5 Birth intervals ... 74
Table 6.6 Age at first birth ... 75
Table 6.7 Median age at first birth ... 76
Table 6.8 Teenage pregnancy and motherhood ... 77

Figure 6.1 Age-specific fertility rates for five-year periods preceding the survey ... 71
Figure 6.2 Trends in age-specific fertility rates, Cambodia 2005, 2010, and 2014 ... 72

7 PRACTICE OF ABORTION ... 79
Table 7.1 Number of induced abortions ... 80
Table 7.2 Pregnancy duration at the time of abortion ... 82
Table 7.3 Place of abortion ... 82
Table 7.4 Persons who helped with abortion ... 83
Table 7.5 Method used for the abortion ... 84

Figure 7.1 Distribution of women who have had an abortion by number of abortions ... 81

8 FAMILY PLANNING ... 85
Table 8.1 Knowledge of contraceptive methods ... 85
Table 8.2 Knowledge of contraceptive methods by background characteristics ... 86
Table 8.3 Current use of contraception by age ... 87
Table 8.4.1 Current use of contraception by background characteristics ... 88
Table 8.4.2 Trends in current use of contraception ... 89
Table 8.5 Use of social marketing brand pills and condoms ... 90
Table 8.6 Knowledge of fertile period ... 91
Table 8.7 Timing of sterilization ... 91
Table 8.8 Source of modern contraception methods ... 92
Table 8.9 Informed choice ... 93
Table 8.10 Future use of contraception ... 94
Table 8.11 Exposure to family planning messages ... 95
Table 8.12 Contact of nonusers with family planning providers ... 96

9 OTHER PROXIMATE DETERMINANTS OF FERTILITY ... 97
Table 9.1 Current marital status ... 98
Table 9.2 Number of women’s co-wives ... 99
Table 9.3 Age at first marriage ... 100

Table 9.4.1 Median age at first marriage: Women ... 101
Table 9.4.2 Median age at first marriage: Men ... 102
Table 9.5 Age at first sexual intercourse ... 103
Table 9.6.1 Median age at first intercourse: Women ... 104
Table 9.6.2 Median age at first intercourse: Men ... 105
Table 9.7.1 Recent sexual activity: Women ... 106
Table 9.7.2 Recent sexual activity: Men ... 107
Table 9.8 Postpartum amenorrhea, abstinence, and insusceptibility ... 108
Table 9.9 Median duration of amenorrhea, postpartum abstinence, and postpartum insusceptibility ... 109
Table 9.10 Menopause ... 110

10 FERTILITY PREFERENCES ... 111
Table 10.1 Fertility preferences by number of living children ... 112
Table 10.2.1 Desire to limit childbearing: Women ... 113
Table 10.2.2 Desire to limit childbearing: Men ... 114
Table 10.3 Need and demand for family planning among currently married women ... 116
Table 10.4 Ideal number of children ... 117
Table 10.5 Mean ideal number of children ... 118
Table 10.6 Fertility planning status ... 118
Table 10.7 Wanted fertility rates ... 119

11 ADULT AND MATERNAL MORTALITY ... 121
Table 11.1 Completeness of information on siblings ... 122
Table 11.2 Sibship size and sex ratio of siblings ... 122
Table 11.3 Adult mortality rates ... 123
Table 11.4 Maternal mortality ... 124

Figure 11.1 Confidence intervals for maternal mortality rates, Cambodia 2005, 2010, and 2014 ... 125

12 INFANT AND CHILD MORTALITY ... 127
Table 12.1 Early childhood mortality rates ... 129
Table 12.2 Early childhood mortality rates by socioeconomic characteristics ... 130
Table 12.3 Early childhood mortality rates by demographic characteristics ... 131
Table 12.4 Perinatal mortality ... 133
Table 12.5 High-risk fertility behavior ... 134

Figure 12.1 Trends in childhood mortality, 2000-2014 ... 129
Figure 12.2 Infant mortality rates by socioeconomic characteristics ... 130
Figure 12.3 Infant mortality rates by demographic characteristics ... 132

13 MATERNAL HEALTH ... 137
Table 13.1 Antenatal care ... 138
Table 13.2 Number of antenatal care visits and timing of first visit ... 139
Table 13.3 Components of antenatal care ... 140
Table 13.4 Tetanus toxoid injections ... 141
Table 13.5 Place of delivery ... 142
Table 13.6 Assistance during delivery ... 144
Table 13.7.1 Timing of first postnatal checkup ... 145
Table 13.7.2 Type of provider of first postnatal checkup for the mother ... 146
Table 13.8.1 Timing of first postnatal checkup for the newborn ... 147
Table 13.8.2 Type of provider of first postnatal checkup for the newborn ... 148
Table 13.9 Problems in accessing health care ... 149

**14**	**CHILD HEALTH**		**151**
	Table 14.1	Child's size and weight at birth	152
	Table 14.2	Vaccinations by source of information	153
	Table 14.3	Vaccinations by background characteristics	154
	Table 14.4	Prevalence and treatment of symptoms of ARI	156
	Table 14.5	Prevalence and treatment of fever	158
	Table 14.6	Prevalence of diarrhea	159
	Table 14.7	Diarrhea treatment	161
	Table 14.8	Feeding practices during diarrhea	162
	Table 14.9	Knowledge of ORS packets or pre-packaged liquids	163
	Table 14.10	Disposal of children's stools	164
	Figure 14.1	Trends in vaccination by age 12 months among children age 12-23 months, 2000-2014	155
**15**	**EARLY CHILDHOOD EDUCATION AND DEVELOPMENT**		**165**
	Table 15.1	Early childhood education	166
	Table 15.2	Support for learning	167
	Table 15.3	Learning materials	168
	Table 15.4	Inadequate care	169
	Table 15.5	Early Child Development Index	171
**16**	**NUTRITION OF CHILDREN AND WOMEN**		**173**
	Table 16.1	Nutritional status of children	176
	Table 16.2	Initial breastfeeding	179
	Table 16.3	Breastfeeding status by age	180
	Table 16.4	Median duration of breastfeeding	182
	Table 16.5	Foods and liquids consumed by children in the day or night preceding the interview	184
	Table 16.6	Infant and young child feeding (IYCF) practices	185
	Table 16.7	Prevalence of anemia in children	188
	Table 16.8	Micronutrient intake among children	190
	Table 16.9	Presence of iodized salt in household	192
	Table 16.10	Nutritional status of women	193
	Table 16.11	Prevalence of anemia in women	195
	Table 16.12	Micronutrient intake among mothers	197
	Figure 16.1	Nutritional status of children by age	177
	Figure 16.2	Trends in nutritional status of children under age 5	178
	Figure 16.3	Infant feeding practices by age	181
	Figure 16.4	IYCF indicators on breastfeeding status	182
	Figure 16.5	Trends in infant and young child feeding (IYCF) practices	186
	Figure 16.6	Trends in anemia status among children under age 5	189
	Figure 16.7	Trends in nutritional status among women age 15-49	194
	Figure 16.8	Trends in anemia status among women age 15-49	196
**17**	**MICRONUTRIENTS**		**199**
	Table 17.1	Coverage of micronutrient testing by residence	200
	Table 17.2	Anemia, iron status, and soluble transferrin receptors among mothers	201
	Table 17.3	Type of hemoglobin among mothers by residence	201
	Table 17.4	Anemia, iron status, and soluble transferrin receptors (sTfRs) among children born since January 2009	202
	Table 17.5	Iron status among children by age	202

Table 17.6 Type of hemoglobin among children born since January 2009 by residence 202
Table 17.7 Intestinal parasitic infection in women and children 203
Table 17.8 Blood level of vitamins A, B12, B9, and D and calcium in mothers 203
Table 17.9.1 Blood level of vitamins A, B12, B9, and D and calcium in children 204
Table 17.9.2 Blood level of vitamins A, B12, B9, and D and calcium in children by age 204
Table 17.10 Urinary iodine excretion in mothers and children by residence 205

18 HIV/AIDS-RELATED KNOWLEDGE, ATTITUDES, AND BEHAVIOR 207

Table 18.1 Knowledge of AIDS 208
Table 18.2 Knowledge of HIV prevention methods 209
Table 18.3.1 Comprehensive knowledge about AIDS: Women 211
Table 18.3.2 Comprehensive knowledge about AIDS: Men 212
Table 18.4 Knowledge of prevention of mother-to-child transmission of HIV 214
Table 18.5.1 Accepting attitudes toward those living with HIV/AIDS: Women 215
Table 18.5.2 Accepting attitudes toward those living with HIV/AIDS: Men 216
Table 18.6 Attitudes toward negotiating safer sexual relations with husband 218
Table 18.7.1 Multiple sexual partners: Women 219
Table 18.7.2 Multiple sexual partners: Men 221
Table 18.8 Payment for sexual intercourse and condom use at last paid sexual intercourse 222
Table 18.9.1 Coverage of prior HIV testing: Women 224
Table 18.9.2 Coverage of prior HIV testing: Men 225
Table 18.10 Pregnant women counseled and tested for HIV 226
Table 18.11 Self-reported prevalence of sexually transmitted infections (STIs) and STI symptoms 228
Table 18.12 Prevalence of medical injections 230
Table 18.13 Comprehensive knowledge about AIDS and of a source of condoms among youth 232
Table 18.14 Age at first sexual intercourse among young people 233
Table 18.15 Premarital sexual intercourse and condom use during premarital sexual intercourse among youth 235
Table 18.16 Multiple sexual partners in the past 12 months among youth 236
Table 18.17 Recent HIV tests among youth 237

19 WOMEN'S EMPOWERMENT AND DEMOGRAPHIC AND HEALTH OUTCOMES 239

Table 19.1 Employment and cash earnings of currently married women and men 240
Table 19.2.1 Control over women's cash earnings and relative magnitude of women's cash earnings 241
Table 19.2.2 Control over men's cash earnings 242
Table 19.3 Women's control over their own earnings and over those of their husbands 243
Table 19.4 Participation in decision making 244
Table 19.5.1 Women's participation in decision making by background characteristics 245
Table 19.5.2 Men's participation in decision making by background characteristics 246
Table 19.6.1 Ownership of assets: Women 248
Table 19.6.2 Ownership of assets: Men 249
Table 19.7.1 Attitude toward wife beating: Women 250
Table 19.7.2 Attitude toward wife beating: Men 252
Table 19.8 Indicators of women's empowerment 253
Table 19.9 Current use of contraception by women's empowerment 254
Table 19.10 Ideal number of children and unmet need for family planning by women's empowerment 254

20 DOMESTIC VIOLENCE 257
Table 20.1 Experience of physical violence 260
Table 20.2 Persons committing physical violence 261
Table 20.3 Experience of sexual violence 262
Table 20.4 Persons committing sexual violence 263
Table 20.5 Age at first experience of sexual violence 263
Table 20.6 Experience of different forms of violence 264
Table 20.7 Experience of violence during pregnancy 265
Table 20.8 Marital control exercised by husbands 267
Table 20.9 Forms of spousal violence 268
Table 20.10 Spousal violence by background characteristics 270
Table 20.11 Spousal violence by husband's characteristics and empowerment indicators ... 272
Table 20.12 Physical or sexual violence in the past 12 months by any husband/partner 273
Table 20.13 Experience of spousal violence by duration of marriage 274
Table 20.14 Injuries to women due to spousal violence 274
Table 20.15 Women's violence against their spouse 275
Table 20.16 Help seeking to stop violence 276
Table 20.17 Sources for help to stop the violence 277

APPENDIX A: SAMPLE IMPLEMENTATION 281
Table A.1 Distribution of households in the sampling frame (2008 GPC, updated) by survey domain and by residence, Cambodia 2014 282
Table A.2 Distribution of enumeration areas in the sampling frame (2008 GPC, updated) and average size of EAs by survey domain and by residence, Cambodia 2014 282
Table A.3 Sample allocation of EAs and households by domain and by type of residence, Cambodia 2014 283
Table A.4 Sample allocation of expected number of interviews of women and men by domain and by type of residence, Cambodia 2014 284
Table A.5 Sample implementation: Women 286
Table A.6 Sample implementation: Men 288

APPENDIX B: ESTIMATES OF SAMPLING ERRORS 291
Table B.1 List of selected variables for sampling errors, Cambodia 2014 293
Table B.2 Sampling errors: Total sample, Cambodia 2014 294
Table B.3 Sampling errors: Urban sample, Cambodia 2014 295
Table B.4 Sampling errors: Rural sample, Cambodia 2014 296
Table B.5 Sampling errors: Banteay Meanchey sample, Cambodia 2014 297
Table B.6 Sampling errors: Kampong Cham sample, Cambodia 2014 298
Table B.7 Sampling errors: Kampong Chhnang sample, Cambodia 2014 299
Table B.8 Sampling errors: Kampong Speu sample, Cambodia 2014 300
Table B.9 Sampling errors: Kampong Thom sample, Cambodia 2014 301
Table B.10 Sampling errors: Kandal sample, Cambodia 2014 302
Table B.11 Sampling errors: Kratie sample, Cambodia 2014 303
Table B.12 Sampling errors: Phnom Penh sample, Cambodia 2014 304
Table B.13 Sampling errors: Prey Veng sample, Cambodia 2014 305
Table B.14 Sampling errors: Pursat sample, Cambodia 2014 306
Table B.15 Sampling errors: Siem Reap sample, Cambodia 2014 307
Table B.16 Sampling errors: Svay Rieng sample, Cambodia 2014 308
Table B.17 Sampling errors: Takeo sample, Cambodia 2014 309
Table B.18 Sampling errors: Otdar Meanchey sample, Cambodia 2014 310
Table B.19 Sampling errors: Battambang and Pailin sample, Cambodia 2014 311
Table B.20 Sampling errors: Kampot and Kep sample, Cambodia 2014 312

Table B.21 Sampling errors: Preah Sihanouk and Koh Kong sample, Cambodia 2014 313
Table B.22 Sampling errors: Preah Vihear and Stung Treng sample, Cambodia 2014 314
Table B.23 Sampling errors: Mondul Kiri and Ratanak Kiri sample, Cambodia 2014 315

APPENDIX C: DATA QUALITY TABLES 317
Table C.1 Household age distribution 317
Table C.2.1 Age distribution of eligible and interviewed women 318
Table C.2.2 Age distribution of eligible and interviewed men 318
Table C.3 Completeness of reporting 318
Table C.4 Births by calendar years 319
Table C.5 Reporting of age at death in days 319
Table C.6 Reporting of age at death in months 320
Table C.7 Nutritional status of children based on the NCHS/CDC/WHO International Reference Population 321

FOREWORD

The 2014 Cambodia Demographic and Health Survey (2014 CDHS) is the fourth survey of its kind to be conducted successfully in Cambodia. Sponsors are the United States Agency for International Development (USAID), the Australian Department of Foreign Affairs and Trade (Australia-DFAT), United Nations Population Fund (UNFPA), United Nations Children's Fund (UNICEF), Japan International Cooperation Agency (JICA), Korean International Cooperation Agency (KOICA) and the Health Sector Support Program-Second Phase (HSSP-2). Technical assistance is provided by ICF International. The Directorate General for Health (DGH) of the Ministry of Health and the National Institute of Statistics (NIS) of the Ministry of Planning are the project implementation agencies.

This report includes information on demography, family planning, maternal mortality, infant and child mortality, and women's health care status, including related information, such as breastfeeding, antenatal care, children's immunization, childhood diseases, HIV/AIDS, and domestic violence. The questionnaires (Household, Man's, and Woman's questionnaires) are designed to evaluate the nutritional status of mothers and children and to measure the prevalence of anemia.

The 2014 CDHS findings are expected to be used by policymakers and program managers to evaluate Cambodia's demographic and health status and then to formulate appropriate population and health policies and programs. The programs of reproductive health and child health and health facilities need to be expanded and improved based on the survey findings.

We would like to thank USAID, Australia-DFAT, UNFPA, UNICEF, JICA, KOICA, and HSSP2 for sponsoring this survey project and ICF International for providing technical assistance. We gratefully acknowledge the support and encouragement extended by the Minister of Health and Minister of Planning; and other members of the 2014 CDHS Executive Committee and Technical Committee who contributed to the survey activities.

We express our sincere thanks to all persons involved in the implementation, analysis, and writing of the 2014 CDHS and especially thank the survey respondents, whose contributions made the survey a success.

H.E. Prof. Eng Huot
Secretary of State
For Minister of Health

H.E. San Sy Than
Secretary of State
For Senior Minister
Minister of Planning

ACKNOWLEDGMENTS

The 2014 Cambodia Demographic and Health Survey (2014 CDHS) represents the continuing commitment and efforts in Cambodia to obtain data on population and health. The survey also reflects interest in obtaining information on maternal health, child health, and anemia prevalence. The 2014 CDHS was sponsored by the United States Agency for International Development (USAID), the Australian Department of Foreign Affairs and Trade (Australia-OF AT), United Nations Population Fund (UNFPA), United Nations Children's Fund (UNICEF), Japan International Cooperation Agency (JICA), Korean International Cooperation Agency (KOICA) and the Health Sector Support Program-Second Phase (HSSP-2). The survey was implemented by the Directorate General for Health (DGH) of the Ministry of Health (MOH) and by the National Institute of Statistics (NIS) of the Ministry of Planning (MOP). This survey could not have been completed without the active support and the efforts of many institutions and individuals. The active support and guidance of the Excellencies Secretaries of State; H.E. Prof. Eng Huot, Ministry of Health, and H.E. San Sy Than, Ministry of Planning, are acknowledged with deep gratitude. We also gratefully acknowledge the representatives of USAID, Australia-OF AT, UNFPA, UNICEF, JICA, KOICA, and HSSP-2 and their staff for their support and valuable comments throughout the survey activities.

Our deep appreciation also goes to the ICF International team led by Mr. Bernard Barrère, Dr. Rathavuth Hong, and others. They are acknowledged with gratitude for their support as they facilitated the survey and ensured its success.

We would like to express our appreciation for all team leaders, field editors, and interviewers from NIS, DGH, and the central and local offices of the Ministry of Planning and Ministry of Health, whose dedicated efforts ensured the quality and timeliness of the survey, and to all respondents for contributing their time and for giving the required information, enabling us to produce high-quality data for the country.

Finally, we would like to thank members of the 2014 CDHS Executive Committee and Technical Committee who contributed to the survey activities.

His Excellency Hang Lina
Director General
National Institute of Statistics

Lo Veasnakiry, M.D.; M.A (HMPP)
Director
Department of Planning and Health Information
Directorate General for Health

CAMBODIA

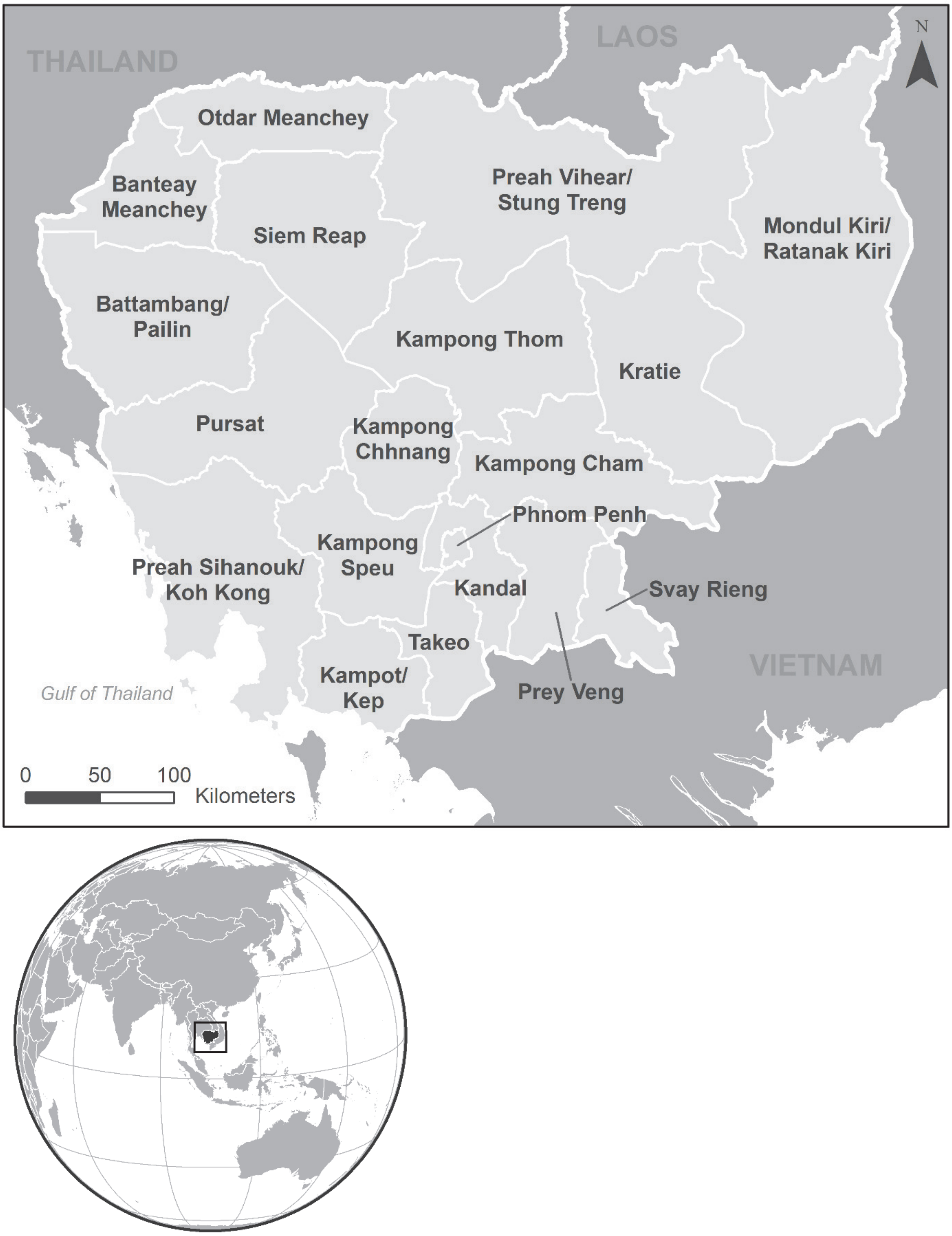
THAILAND
LAOS
N
Otdar Meanchey
Banteay Meanchey
Siem Reap
Preah Vihear/ Stung Treng
Mondul Kiri/ Ratanak Kiri
Battambang/ Pailin
Kampong Thom
Kratie
Pursat
Kampong Chhnang
Kampong Cham
Phnom Penh
Kampong Speu
Preah Sihanouk/ Koh Kong
Kandal
Svay Rieng
Takeo
VIETNAM
Kampot/ Kep
Prey Veng
Gulf of Thailand
0
50
100
Kilometers

INTRODUCTION 1

Key Findings

- The 2014 Cambodia Demographic and Health Survey (CDHS) is a nationally representative survey of 15,825 households with 17,578 women age 15-49 and 5,190 men age 15-49.
- The 2014 CDHS is the fourth Demographic and Health Survey conducted in Cambodia as part of the worldwide Demographic and Health Surveys project.
- The primary purpose of the CDHS is to furnish policymakers and planners with detailed information on fertility and family planning; infant, child, adult, and maternal mortality; maternal and child health; nutrition; and knowledge of HIV/AIDS and other sexually transmitted infections.
- In all selected households, women age 15-49 and children age 6-59 months were tested for anemia.

1.1 Geodemography, History, and Economy

1.1.1 Geodemography

Cambodia is an agricultural country located in Southeast Asia. It borders with Thailand to the west, Laos and Thailand to the north, the Gulf of Thailand to the southwest, and Vietnam to the east and the south. It has a total land area of 181,035 square kilometers.

Cambodia has a tropical climate with two distinct seasons that set the rhythm of rural life. From November to February, the cool, dry northeastern monsoon brings little rain, whereas from May to October the southwestern monsoon carries strong winds, high humidity, and heavy rains. The mean annual temperature for Phnom Penh, the capital city, is 27°C.

The 1962 population census was the last official census to be conducted prior to 1998; it revealed a population of 5.7 million. The population census in 1998 recorded a population of 11.4 million with an annual growth rate of 2.5 percent (National Institute of Statistics, 1999). The 2004 Inter-Censal Population Survey showed that the annual growth rate had declined to 1.8 percent, with a total population of 13.1 million (National Institute of Statistics, 2004). The 2008 General Population Census (GPC) showed a further decrease in the annual growth rate to 1.54, with a total population of 13.4 million (National Institute of Statistics, 2009).

The proportion of the population living in rural areas is 80.5 percent; only 19.5 percent of the country's residents live in urban areas. The population density in the country as a whole is 75 per square kilometer, with approximately 1.3 million inhabitants living in Phnom Penh. The average size of the Cambodian household is 4.7. The total male to female sex ratio is 94.7. The literacy rate among adult males is 84 percent, considerably higher than the rate among females (76 percent). Currently, it is estimated that the percentage of the total population living below the poverty line fell to 21.1 percent in 2010 and decreased further to 19.8 percent in 2011 (MOP, 2012).

1.1.2 History

Cambodia gained complete independence from France under the leadership of Prince Norodom Sihanouk on November 9, 1953. In March 1970, a military coup led by General Lon Nol overthrew Prince Sihanouk.

On April 17, 1975, the Khmer Rouge ousted the Lon Nol regime and took control of the country. Under the new regime, the country was renamed Democratic Kampuchea. Nearly 2 million Cambodian people died during the Khmer Rouge's radical and genocidal regime.

On January 7, 1979, the revolutionary army of the National Front for Solidarity and Liberation of Cambodia defeated the Khmer Rouge regime and proclaimed the country the People's Republic of Kampuchea and later, in 1989, the State of Cambodia.

The country's most important political event was the free elections held in May 1993 under the close supervision of the United Nations Transitional Authority in Cambodia (UNTAC). At that time Cambodia was proclaimed the Kingdom of Cambodia, and it is a constitutional monarchy. Four additional free and fair elections took place in 1998, 2003, 2008, and 2013. Now Cambodia is stable and well on its way to democracy and a promising future.

1.1.3 Economy

Since the 1991 Paris Peace Accord, Cambodia's economy has made significant progress after more than two decades of political unrest. However, Cambodia still remains one of the poorest and least developed countries in Asia, with the gross domestic product per capita estimated at approximately 4.4 million Riel or $1,088 in 2014 (US$1 = 4,087 Riel) (International Monetary Fund, 2011). Agriculture, mainly rice production, is still the main economic activity in Cambodia. Small-scale subsistence agriculture, such as fisheries, forestry, and livestock, is another important sector. Garment factories and tourism services are also important components of foreign direct investments.

1.2 HEALTH STATUS AND POLICY

Health outcomes have improved recently. The infant mortality rate has decreased from 45 per 1,000 live births in 2010 to 27 per 1,000 live births in 2014. The under-5 mortality rate decreased from 54 per 1,000 live births to 35 per 1,000 live births in the same period. Life expectancy at birth is 67.1 years for males and 70.1 years for females (NIS, 2013). General government expenditures on health per capita increased from US$8 in 2008 to US$11 in 2010, US$13 in 2012, and US$16 in 2014 (MOH, 2015). The health status of the Cambodian people has steadily improved in a number of key areas. Nonetheless, challenges remain in many other areas.

To improve the health status of the Cambodian people, the Ministry of Health developed the Health Sector Strategic Plan for 2008-2015 (Ministry of Health, 2008). Its policy direction is as follows:

- Make services more responsive and closer to the public through implementation of a decentralized service delivery function and a management function guided by the national "Policy on Service Delivery" and the policy on "Decentralization and Deconcentration."

- Strengthen sector-wide governance through implementation of a sector-wide approach, focusing on increased national ownership and accountability to improved health outcomes, harmonization and alignment, greater coordination, and effective partnerships among all stakeholders.

- Scale up access to and coverage of health services, especially comprehensive reproductive, maternal, newborn, and child health services, both demand and supply side, through

mechanisms such as institutionalization and expansion of contracting through Special Operating Agencies, exemptions for the poor, health equity funds, and health insurance.

- Implement pro-poor health financing systems, including exemptions for the poor and expansion of health equity funds, in combination with other forms of social assistance mechanisms.

- Reinforce health legislation, professional ethics, and codes of conduct and strengthen regulatory mechanisms, including for the production and distribution of pharmaceuticals, drug quality control, cosmetics, and food safety and hygiene, to protect providers and consumers' rights and their health.

- Improve quality in service delivery and management through establishment of and compliance with national protocols, clinical practice guidelines, and quality standards, in particular establishment of accreditation systems.

- Increase the competency and skills of the health workforce to deal with increased demands for accountability and high-quality care, including through strengthening allied technical skills and advanced technology through increased quality of training, career development, appropriate incentives, and a good working environment.

- Strengthen and invest in health information systems and health research for evidence-based policy-making, planning, performance monitoring, and evaluation.

- Increase investments in physical infrastructures, medical care equipment, and advanced technology, as well as in improvement of non-medical support services including management, maintenance, blood safety, and supply systems for drugs and commodities.

- Promote quality of life and healthy lifestyles by raising health awareness and creating supportive environments, including through strengthening institutional structures, financial and human resources, and IEC (information, education, and communication) materials for health promotion, behavior change communication, and appropriate health-seeking practices.

- Prevent and control communicable and selected chronic and noncommunicable diseases and strengthen disease surveillance systems for an effective response to emerging and reemerging diseases.

- Strengthen public health interventions to deal with cross-cutting challenges, especially those related to gender, health of minorities, hygiene and sanitation, school health, environmental health risks, substance abuse/mental health, injury, occupational health, and disaster, through timely responses and effective collaboration and coordination with other sectors.

- Promote effective public and private partnerships in service provision based on policy, regulation, legislation, and technical standards.

- Encourage community engagement in health service delivery activities, management of health facilities, and continuous quality improvement.

- Systematically strengthen institutions at all levels of the health system to implement the policy agenda listed under the previous 14 elements.

1.3 OBJECTIVE AND SURVEY ORGANIZATION

The 2014 Cambodia Demographic and Health Survey (CDHS) is the fourth nationally representative survey conducted in Cambodia on population and health issues. It uses the same methodology as its predecessors, the 2000, 2005, and 2010 Cambodia Demographic and Health Surveys, allowing policymakers to use these surveys to assess trends over time.

The primary objective of the CDHS is to provide the Ministry of Health (MOH), Ministry of Planning (MOP), and other relevant institutions and users with updated and reliable data on infant and child mortality, fertility preferences, family planning behavior, maternal mortality, utilization of maternal and child health services, health expenditures, women's status, and knowledge and behavior regarding HIV/AIDS and other sexually transmitted infections. This information contributes to policy decisions, planning, monitoring, and program evaluation for the development of Cambodia at both the national and local government levels.

The long-term objectives of the survey are to build the capacity of the Ministry of Health and the National Institute of Statistics (NIS) of the Ministry of Planning for planning, conducting, and analyzing the results of further surveys.

The 2014 CDHS survey was conducted by the Directorate General for Health (DGH) of the Ministry of Health and the National Institute of Statistics of the Ministry of Planning. The CDHS executive committee and technical committee were established to oversee all technical aspects of implementation. They consisted of representatives from the Ministry of Health, the Ministry of Planning, the National Institute of Statistics, the U.S. Agency for International Development (USAID), the Australian Department of Foreign Affairs and Trade (Australia-DFAT), the United Nations Population Fund (UNFPA), the United Nations Children's Fund (UNICEF), the Japan International Cooperation Agency (JICA), and the Korean International Cooperation Agency (KOICA). Funding for the survey came from USAID, Australia-DFAT, UNFPA, UNICEF, JICA, KOICA, and the Health Sector Support Program–Second Phase (HSSP-2). Technical assistance was provided by ICF International.

1.4 SAMPLE DESIGN

The 2014 CDHS sample is a nationally representative sample of women and men between age 15 and 49 who completed interviews. To achieve a balance between the ability to provide estimates at the subnational level and limiting the sample size, 19 sampling domains were defined, 14 of which correspond to individual provinces and 5 of which correspond to grouped provinces:

- Fourteen individual provinces: Banteay Meanchey, Kampong Cham, Kampong Chhnang, Kampong Speu, Kampong Thom, Kandal, Kratie, Phnom Penh, Prey Veng, Pursat, Siem Reap, Svay Rieng, Takeo, and Otdar Meanchey

- Five groups of provinces: Battambang and Pailin, Kampot and Kep, Preah Sihanouk and Koh Kong, Preah Vihear and Stung Treng, and Mondul Kiri and Ratanak Kiri

The sample of households was allocated to the sampling domains in such a way that estimates of indicators could be produced with precision at the national level, as well as separately for urban and rural areas of the country and for each of the 19 sampling domains.

The sampling frame used for the 2014 CDHS was derived from the list of all enumeration areas (EAs) created for the 2008 Cambodia General Population Census (GPC), provided by NIS. The list had been updated in 2012, and it excluded 241 EAs that are special settlement areas and not ordinary residential areas. It included 28,455 EAs for the entire country. The GPC also created maps that delimited the boundaries of each EA. Overall, 4,245 EAs were designated as urban and 24,210 as rural, with an average size of 99 households per EA.

The survey used a stratified sample selected in two stages. Stratification was achieved by separating every reporting domain into urban and rural areas. Thus, the 19 domains were stratified into a total of 38 sampling strata. Samples were selected independently in every stratum through a two-stage selection process. Implicit stratifications were achieved at each of the lower geographical or administrative levels by sorting the sampling frame according to geographical/administrative order before sample selection and by using a probability proportional to size selection strategy at the first stage of selection.

In the first stage, 611 EAs (188 in urban areas and 423 in rural areas) were selected with probability proportional to size. The size of an EA was defined as the number of households residing in the EA. Some of the largest EAs (more than 200 households) were divided into segments; only one segment was selected randomly to be included in the survey. Thus, the 611 CDHS clusters were either an EA or a segment of an EA. A listing of all households was carried out in each of the 611 clusters during the months of February through April 2014. Listing teams also drew fresh maps delineating EA boundaries and identifying all households. These maps and lists were used by field teams during data collection. The household listings provided the frame from which households were selected in the second stage. In the second stage selection, a fixed number of 24 households were selected from every urban cluster, and a fixed number of 28 households were selected from every rural cluster, through equal probability systematic sampling. Small areas and urban areas were oversampled, and this oversampling was corrected in the analysis using sampling weights to ensure the natural representation of the sample for all 38 strata (19 domains by urban or rural area). Appendix A provides a complete description of the sample design and weighting procedures.

All women age 15-49 who were either usual residents of the selected households or visitors present in the household on the night before the survey were eligible to be interviewed. In addition, in a subsample of one-third of the households selected for the survey, all men age 15-49 were eligible to be interviewed (if they were either usual residents of the selected households or visitors present in the household on the night before the survey). This was a cost-effective strategy given that the minimum sample size required for the women's survey was larger than that for the men's survey because complex indicators (such as total fertility and infant and child mortality rates) require larger sample sizes to achieve a reasonable level of precision, and these data are derived from interviews with women.

In the subsample of households chosen for the male interviews (one-third of the total sample), all women eligible for interviews and all children under age 5 were eligible for anemia testing. These same women and children were also eligible for height and weight measurements to determine their nutritional status.

In a subsample consisting of one in every six of the selected clusters, a survey component focusing on micronutrient indicators was implemented among all eligible women age 15-49 who had children under age born since January 2009, as well as among the children themselves. Since data on micronutrient indicators are reported only at the national level and for urban and rural areas, a subsample of clusters was cost-effective, producing a sample size large enough to provide estimations with adequate precision.

1.5 QUESTIONNAIRES

Four questionnaires were used in the 2014 CDHS: the Household Questionnaire, the Woman's Questionnaire, the Man's Questionnaire, and the Micronutrient Questionnaire. These questionnaires are based on the questionnaires developed by the worldwide Demographic and Health Surveys (DHS) Program and on the questionnaires used during the 2010 CDHS survey. To reflect relevant population and health issues in Cambodia, the questionnaires were adapted during a series of technical meetings with various stakeholders from government ministries and agencies, nongovernmental organizations, and international donors. The final drafts of the questionnaires were discussed at a stakeholders' meeting

organized by the National Institute of Statistics. The adapted questionnaires were translated from English into Khmer and pretested in February and March 2014.

The Household Questionnaire was used to list all of the usual members and visitors in the selected households. Basic information was collected on the characteristics of each person listed, including age, sex, education, and relationship to the head of the household. For children under age 18, parents' survival status was determined. The Household Questionnaire also collected information on the following topics:

- Dwelling characteristics
- Accidental death and injury
- Physical impairment
- Utilization of health services and health expenditures for recent illness and injury
- Disability
- Possession of iodized salt
- Height and weight of women and children
- Hemoglobin measurements among women and children for diagnosing anemia

The Household Questionnaire was used to identify women and men eligible for an individual interview. The Woman's Questionnaire was used to collect information from all women age 15-49 and was organized into the following sections:

- Respondent background characteristics
- Reproduction, including a complete birth and death history of respondents' live births and information on abortion
- Contraception
- Pregnancy, postnatal care, and women's nutrition
- Immunization, health, children's nutrition, and early childhood development
- Marriage and sexual activity
- Fertility preferences
- Husbands' background and women's work
- Domestic violence
- HIV/AIDS and other sexually transmitted infections
- Maternal mortality

The Man's Questionnaire was administered to all men age 15-49 living in one-third of the households in the CDHS sample. The Man's Questionnaire was organized into the following sections:

- Respondent background characteristics
- Reproduction
- Marriage and sexual activity
- HIV/AIDS
- Other health issues

The Micronutrient Questionnaire was implemented in a subsample of one-sixth of the sampled clusters for the collection of micronutrient specimens among eligible women and children. Specimens collected included venous blood, urine, and stool samples.

The CDHS underwent a full pretest before commencement of the main data collection. All aspects of data collection were pretested in February and March 2014. Forty-four women and men were trained from February 27 to March 17, 2014, in the administration of the CDHS survey instruments, taking of anthropometric measurements, and hemoglobin testing. Five days of fieldwork were followed by three days of interviewer debriefing and correction of questionnaires. Pretest fieldwork was conducted in 79

households in two rural and two urban villages. Constructive input from interviewers was used to refine the survey instruments and survey logistics. These pretest activities were used to finalize the questionnaires. The majority of pretest participants also attended the training for the main survey, with many of them serving as field editors and team leaders for the survey.

1.6 Training and Fieldwork

The goal of training was to create 19 field teams capable of collecting data for the 2014 CDHS. Each team was responsible for data collection in one of the 19 survey domains (comprising the 23 provinces and the capital city of Phnom Penh). Field teams were composed of five people (5 teams) or six people (14 teams): a team leader, a field editor, two or three female interviewers, and one male interviewer. Nineteen fully staffed field teams would require 114 field personnel, and at the end of training 109 field personnel were retained. Twenty-six days of training included four days of field practice in Kandal province. Data processing personnel (3 data processing supervisors, 10 office editors/coders, 19 data entry operators, and 5 reserves) also attended classroom training.

Training began with the Household Questionnaire and was followed by the Woman's Questionnaire. Additional time was spent reviewing the Household Questionnaire, including consent statements for hemoglobin testing, and conversion of ages and dates of birth from the Khmer calendar to the Gregorian calendar. One week was devoted to additional activities, including the Man's Questionnaire, measurement of women's and children's height and weight, sample implementation and household selection, testing of household salt for iodine, and organization of documents and materials for return to the head office. After completion of training, including field practice, fieldwork was launched and teams disbursed to their assigned provinces.

During the training period, the 19 CDHS team leaders were provided with the cluster information for the provinces in which they would be working so that they could devise a data collection sequence for their sample points. Team leaders were best equipped to perform this task because they hailed from their own provinces. They also conducted the CDHS household listing operation (described in Appendix A) and therefore were well acquainted with the areas in which they would be working. The progression of fieldwork by geographic location had to take into account weather conditions during the rainy season.

Fieldwork supervision was carried out regularly by three CDHS survey coordinators from NIS and MOH along with an ICF Macro consultant. Supervision visits were conducted throughout the six months of data collection and included retrieval of questionnaires from the field. In addition, a quality control program was run by the data processing team to detect key data collection errors for each team. These data checks were used to provide regular feedback to each team based on its specific performance. Data collection was conducted from June 2 to December 12, 2014.

The training and fieldwork for collection of stool, urine, and venous blood samples were conducted separately by UNICEF in collaboration with the Institut de Recherche pour le Développement (France) and Cambodia's Ministry of Agriculture, Forestry, and Fisheries. Details are provided in the micronutrient chapter.

1.7 Biomarker Testing

1.7.1 Anthropometric Measurement

The 2014 CDHS included an anthropometric component in which children under age 5 in a subsample of two-thirds of the households were measured for height and weight. Weight measurements were taken using a lightweight, electronic SECA scale designed and manufactured under the guidance of UNICEF. The scale allowed for the weighing of very young children through an automatic mother-child adjustment that eliminates the mother's weight while she is standing on the scale with her baby. Height measurements were carried out using a SECA measuring board, also produced under the guidance of

UNICEF. Children younger than age 24 months were measured lying down (recumbent length) on the board, whereas standing height was measured for older children. Three nutritional indices were calculated using children's age, height, and weight: height-for-age (stunting), weight-for-height (wasting), and weight-for-age (underweight). The height and weight of women age 15-49 were also measured among the two-thirds subsample of households selected in the 2014 CDHS.

1.7.2 Hemoglobin Testing

Hemoglobin testing is the primary method for anemia diagnosis. The 2014 CDHS included anemia testing of children age 6 to 59 months and women age 15-49 in the two-thirds of CDHS households that were not selected for the men's interview. A consent statement was read to the eligible respondent or, in the case of children and young unmarried women age 15-17, the parent or responsible adult. This statement explained the purpose of the test, informed the individual that the results would be made available as soon as the test was completed, and requested permission for the test to be carried out. Anemia levels were determined by measuring the level of hemoglobin in the blood; a decreased concentration characterizes anemia. The concentration of hemoglobin in the blood was measured in the field using the HemoCue system. The HemoCue instrument is a special purpose photometer designed specifically for the determination of hemoglobin levels. A capillary blood sample was taken from the palm side of the end of a finger, by puncturing with a sterile, non-reusable, self-retractable lancet. The blood drop was collected in a HemoCue microcuvette, which serves as a measuring tool, and placed in the HemoCue photometer to determine the level of hemoglobin in the blood. A pamphlet was given to each respondent explaining symptoms of anemia, prevention methods, and the individual results of the hemoglobin measurement of the respondent and any children for whom she gave permission to be measured. Each person whose hemoglobin level was lower than the recommended cutoff point (testing severely anemic) was advised to visit a health facility for follow-up with a health professional.

1.7.3 Micronutrient Testing

The 2014 CDHS included a micronutrient component that was implemented in one out of six clusters selected for the main survey. In these clusters, blood, urine, and stool samples were collected by separate data collection teams from women who had had children born since January 2009 and from the children themselves. The blood/urine/stool samples were sent to several laboratories inside and outside of Cambodia.

1.8 DATA PROCESSING

Completed questionnaires were returned from the field to NIS headquarters, where they were entered and edited by data processing personnel who were specially trained for this task and had also attended questionnaire training of field staff. Data processing personnel included a data processing chief, two assistants, four secondary editors and coordinators, 25 entry operators, and eight office editors.

Data processing for the 2014 CDHS began on 25 personal computers on July 6, 2014, five weeks after the first interviews were conducted. Processing the data concurrently with data collection allowed for regular monitoring of team performance and data quality. Field check tables were generated regularly during the data processing to check various data quality parameters. As a result, feedback was given on a regular basis, encouraging teams to continue in areas of high quality and to correct areas of needed improvement. Feedback was individually tailored to each team. Data entry, which included 100 percent double entry to minimize keying errors, and data editing were completed on January 8, 2015. Data cleaning and finalization were completed on January 23, 2015.

1.9 SAMPLE COVERAGE

All of the 611 clusters selected for the sample were surveyed in the 2014 CDHS. A total of 16,356 households were selected, of which 15,937 were found to be occupied during data collection. Among these

households, 15,825 completed the Household Questionnaire, yielding a response rate of 99 percent (Table 1.1).

In these interviewed households, 18,012 women were identified as eligible for the individual interview. Interviews were completed with 98 percent of these women. Of the 5,484 eligible men identified in every third household, 95 percent were successfully interviewed. There was little variation in response rates by urban-rural residence.

Table 1.1 Results of the household and individual interviews

Number of households, number of interviews, and response rates, according to residence (unweighted), Cambodia 2014

	Residence		
Result	Urban	Rural	Total
**Household interviews**			
Households selected	4,512	11,844	16,356
Households occupied	4,399	11,538	15,937
Households interviewed	4,366	11,459	15,825
Household response rate[1]	99.2	99.3	99.3
**Interviews with women age 15-49**			
Number of eligible women	5,842	12,170	18,012
Number of eligible women interviewed	5,667	11,911	17,578
Eligible women response rate[2]	97.0	97.9	97.6
**Interviews with men age 15-49**			
Number of eligible men	1,641	3,843	5,484
Number of eligible men interviewed	1,540	3,650	5,190
Eligible men response rate[2]	93.8	95.0	94.6

[1] Households interviewed/households occupied
[2] Respondents interviewed/eligible respondents

HOUSEHOLD POPULATION AND HOUSING CHARACTERISTICS 2

Key Findings

- Forty-three percent of the population in Cambodia is age 19 or younger.
- Twenty-seven percent of household heads are women.
- Sixty-five percent of households use an improved source of drinking water during the dry season and 84 percent during the rainy season.
- Two in three households (67 percent) use an appropriate method of treating their drinking water, primarily boiling it (55 percent).
- Forty-six percent of households have an improved, not shared sanitation facility.
- Slightly more than half of households (56 percent) have electricity.
- Nine in 10 Cambodians own a mobile phone.
- Nearly three-quarters of children (73 percent) under age 5 have their birth registered.

This chapter summarizes the socioeconomic characteristics of households and respondents surveyed, including age, sex, residence (urban-rural), educational status, household facilities, and household characteristics. The profile of the households provided in this chapter will help in understanding the results of the 2014 CDHS in the following chapters. In addition, it may provide useful information for social and economic development planning.

Throughout this report, numbers in the tables reflect weighted numbers. Due to the way the sample was designed, the number of weighted cases in some regions appears small, because they are weighted to make the regional distribution nationally representative. However, roughly the same number of households and women and men were interviewed in each province or group of provinces, and the number of unweighted cases is always large enough to calculate the presented estimates. Estimates based on an insufficient number of cases are shown in parentheses or not shown at all.

The 2014 CDHS collected information from all usual residents of a selected household (*de jure* population) and persons who had stayed in the selected household the night before the interview (*de facto* population). Although the difference between these two populations is small, to avoid double counting all tables in this report refer to the de facto population unless otherwise specified. The CDHS used the same definition of households as the 2008 census conducted by the National Institute of Statistics. A household was defined as a person or group of related and unrelated persons who live together in the same dwelling unit(s) or in connected premises, who acknowledge one adult member as the head of the household, and who have common arrangements for cooking and eating meals.

2.1 CHARACTERISTICS OF THE HOUSEHOLD POPULATION

2.1.1 Age and Sex Composition

Age and sex are important demographic variables and are the primary basis of demographic classification in vital statistics, censuses, and surveys. They are also important variables in the study of mortality, fertility, and nuptiality. The effect of variations in sex composition from one population group to another should be taken into account in comparative studies of mortality. In general, a cross-classification with sex is useful for the effective analysis of all forms of data obtained in surveys.

The survey collected information on age in completed years for each household member. When the age was not known, interviewers inquired further for dates of birth in the Gregorian calendar, the Khmer calendar, and/or a historical calendar. Age was then calculated using conversion charts specifically designed for this purpose.

Table 2.1 presents the percent distribution of the household population by age, according to urban-rural residence and sex. The population spending the night before the survey in the households selected for the survey included 69,471 individuals, of whom 48 percent were males and 52 percent were females.

The age structure of the household population is typical of a society with a young population and recently declining fertility. The sex and age distribution of the population is also shown in the population pyramid in Figure 2.1. Cambodia has a relatively broad-based pyramid structure because 43 percent of the population is less than age 20.

Table 2.1 Household population by age, sex, and residence

Percent distribution of the de facto household population by five-year age groups, according to sex and residence, Cambodia 2014

	Urban			Rural					
Age	Male	Female	Total	Male	Female	Total	Male	Female	Total
<5	10.2	8.9	9.5	12.2	10.8	11.5	11.8	10.5	11.2
5-9	10.2	8.7	9.4	13.4	11.6	12.5	12.9	11.2	12.0
10-14	9.5	9.1	9.3	12.3	11.2	11.7	11.9	10.9	11.4
15-19	9.5	8.9	9.2	9.2	7.8	8.4	9.2	7.9	8.6
20-24	10.0	10.9	10.5	8.1	7.9	8.0	8.4	8.4	8.4
25-29	9.6	9.5	9.5	7.5	7.4	7.5	7.9	7.8	7.8
30-34	9.4	9.7	9.6	7.9	8.0	7.9	8.2	8.3	8.2
35-39	4.8	4.7	4.8	5.0	5.0	5.0	4.9	5.0	5.0
40-44	5.7	5.7	5.7	5.2	5.5	5.4	5.3	5.5	5.4
45-49	5.0	5.2	5.1	4.8	5.2	5.0	4.9	5.2	5.0
50-54	5.0	4.9	5.0	4.4	5.2	4.9	4.5	5.2	4.9
55-59	3.8	4.4	4.1	2.9	4.4	3.7	3.0	4.4	3.7
60-64	2.7	3.5	3.1	2.3	3.4	2.9	2.4	3.4	2.9
65-69	1.5	2.5	2.0	1.9	2.5	2.2	1.8	2.5	2.2
70-74	1.4	1.3	1.3	1.3	1.7	1.5	1.3	1.6	1.5
75-79	0.8	1.2	1.0	0.9	1.2	1.0	0.8	1.2	1.0
80+	0.7	1.0	0.9	0.8	1.1	1.0	0.8	1.1	0.9
Total	100.0	100.0	100.0	100.0	100.0	100.0	100.0	100.0	100.0
Number	5,248	5,932	11,180	27,818	30,473	58,291	33,066	36,405	69,471

Figure 2.1 **Population pyramid**

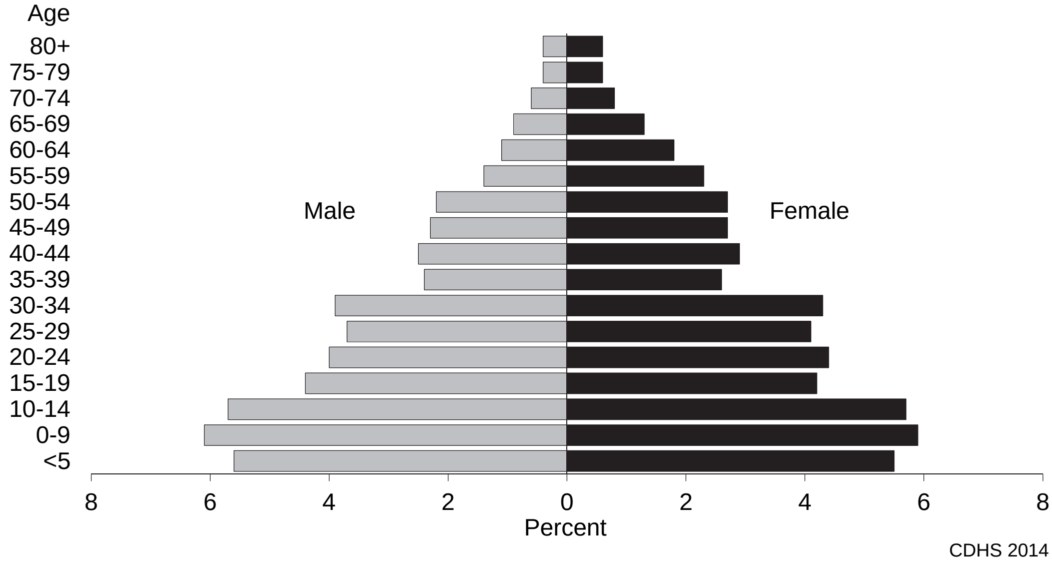

Above the age of 10 years, the pyramid follows a typical pattern of decreasing numbers as age increases. However, the percentage of people age 35 to 44 is less than would be expected because these are the two age groups born in the decade of the 1970s. The early 1970s saw escalating civil war, and in the

late 1970s the Khmer Rouge ruled. This period of time was characterized by few births and high mortality, including high infant and child mortality.

Cambodia has a large dependent population of children and adolescents, although with decreasing fertility the proportion of the population under age 15 has recently declined. The proportion of those age 50 or older has slightly increased. Table 2.2 shows that the proportion of children under age 15 has remained constant over the past four years, with this age group accounting for 35 percent of the population. Sixty percent of the population is in the 15-64 age group, and 6 percent are age 65 or older.

Table 2.2 Population by age according to selected sources

Percent distribution of the de facto population by age group, according to selected sources, Cambodia 2014

Age	1998 census[1]	2000 CDHS[2]	2004 CIPS[3]	2005 CDHS[4]	2008 census[5]	2010 CDHS[6]	2014 CDHS
<15	42.8	42.7	38.6	38.9	33.7	34.5	34.5
15-49	46.9	46.3	49.5	47.9	53.4	50.5	48.4
50-64	6.8	7.4	8.0	8.6	8.6	10.0	11.5
65+	3.5	3.6	3.9	4.6	4.3	5.0	5.6
Total	100.0	100.0	100.0	100.0	100.0	100.0	100.0

[1] General Population Census of Cambodia, 1998 (National Institute of Statistics, 1999)
[2] Cambodia Demographic and Health Survey, 2000 (National Institute of Statistics and ORC Macro, 2001)
[3] Cambodia Inter-Censal Population Survey, 2004 (National Institute of Statistics, 2004)
[4] Cambodia Demographic and Health Survey, 2005 (National Institute of Statistics and ORC Macro, 2006)
[5] General Population Census of Cambodia, 2008 (National Institute of Statistics, 2009)
[6] Cambodia Demographic and Health Survey, 2010 (National Institute of Statistics, Directorate General for Health, and ICF Macro, 2011)

2.1.2 Household Composition

Table 2.3 shows the distribution of households in the survey by the sex of the head of the household and the number of household members, according to urban and rural residence. Households in Cambodia are predominantly male-headed. However, 27 percent of households are headed by women (28 percent and 27 percent in urban and rural areas, respectively).

The average household size is 4.6 persons, about the same as that observed in the 2010 CDHS (4.7 persons per household). Urban households have 5.0 persons per household on average and are slightly larger than rural households (4.5 persons).

Table 2.3 also shows that 17 percent of households include foster and/or orphaned children. Overall, 13 percent of households have foster children, 6 percent have single orphans, and 1 percent have double orphans. The variation between rural and urban areas is small.

Table 2.3 Household composition

Percent distribution of households by sex of head of household and by household size, mean size of household, and percentage of households with orphans and foster children under age 18, according to residence, Cambodia 2014

Characteristic	Residence		Total
	Urban	Rural	
**Household headship**			
Male	71.7	73.4	73.1
Female	28.3	26.6	26.9
Total	100.0	100.0	100.0
**Number of usual members**			
1	3.2	3.4	3.4
2	8.2	9.4	9.2
3	13.5	17.0	16.5
4	20.8	24.0	23.5
5	19.6	19.5	19.6
6	13.8	12.8	12.9
7	7.9	7.4	7.5
8	5.5	3.3	3.7
9+	7.5	3.2	3.8
Total	100.0	100.0	100.0
Mean size of households	5.0	4.5	4.6
**Percentage of households with orphans and foster children under age 18**			
Foster children[1]	14.6	12.9	13.1
Double orphans	1.3	1.0	1.0
Single orphans[2]	4.5	5.6	5.5
Foster and/or orphan children	17.3	16.7	16.8
Number of households	2,284	13,541	15,825

Note: Table is based on de jure household members, i.e., usual residents.
[1] Foster children are those under age 18 living in households with neither their mother nor their father present.
[2] Includes children with one dead parent and an unknown survival status of the other parent

2.2 EDUCATION OF THE HOUSEHOLD POPULATION

Many behaviors, including those in the realms of reproduction, contraceptive use, child health, and proper hygiene, are affected by the education of household members. Information on the educational level of the female and male population age 6 and above is presented in Tables 2.4.1 and 2.4.2. Survey results show that although the majority of Cambodians have not completed primary school, the country has experienced strong improvement in educational attainment over time. Overall, 19 percent of females have never attended school, as compared with 10 percent of males. Improvements over time have resulted in only 2 percent of girls and 3 percent of boys age 10-14 having never attended school at all.

Table 2.4.1 Educational attainment of the female household population

Percent distribution of the de facto female household population age 6 and over by highest level of schooling attended or completed and median years completed, according to background characteristics, Cambodia 2014

Background characteristic	No education	Some primary	Completed primary[1]	Some secondary	Completed secondary[2]	More than secondary	Don't know/ missing	Total	Number	Median years completed
**Age**										
6-9	15.5	84.4	0.0	0.0	0.0	0.0	0.0	100.0	3,318	0.3
10-14	1.9	64.8	3.6	29.6	0.0	0.0	0.0	100.0	3,957	4.2
15-19	3.1	20.2	9.2	62.3	2.3	3.0	0.0	100.0	2,891	7.1
20-24	5.7	25.0	11.2	38.8	7.3	12.0	0.0	100.0	3,054	6.5
25-29	11.5	36.1	10.8	29.2	4.6	7.8	0.0	100.0	2,825	5.2
30-34	18.0	46.8	6.5	22.0	3.2	3.5	0.0	100.0	3,005	3.8
35-39	17.8	53.0	6.0	20.0	2.3	1.0	0.0	100.0	1,806	3.2
40-44	16.6	51.7	5.2	22.5	2.3	1.8	0.0	100.0	2,019	3.3
45-49	25.1	55.5	2.9	13.8	1.8	0.9	0.0	100.0	1,883	2.3
50-54	37.1	53.7	3.0	5.3	0.6	0.2	0.0	100.0	1,889	1.2
55-59	33.0	52.4	4.3	9.2	0.6	0.4	0.1	100.0	1,613	1.6
60-64	36.6	47.8	6.5	7.5	1.2	0.4	0.0	100.0	1,248	1.4
65+	63.8	27.7	3.0	5.0	0.3	0.2	0.0	100.0	2,316	0.0
**Residence**										
Urban	10.7	35.5	5.4	31.8	5.8	10.8	0.0	100.0	5,307	5.5
Rural	20.6	50.5	5.7	20.7	1.4	1.1	0.0	100.0	26,521	2.7
**Province**										
Banteay Meanchey	19.6	52.8	4.7	19.6	2.2	1.1	0.0	100.0	1,340	2.6
Kampong Cham	19.9	51.9	6.6	19.2	1.3	1.1	0.0	100.0	3,985	2.4
Kampong Chhnang	15.3	50.3	7.0	23.0	2.5	1.9	0.0	100.0	1,218	3.2
Kampong Speu	18.7	46.7	7.1	25.4	1.2	0.9	0.1	100.0	2,027	3.5
Kampong Thom	19.1	54.0	6.0	17.5	1.7	1.6	0.0	100.0	1,589	2.5
Kandal	16.4	48.1	6.2	26.1	1.4	1.9	0.0	100.0	2,454	3.5
Kratie	20.1	55.4	5.0	17.1	1.4	1.0	0.0	100.0	903	2.2
Phnom Penh	9.9	34.6	5.0	31.7	5.5	13.3	0.0	100.0	3,135	5.7
Prey Veng	22.1	49.4	5.3	21.3	1.1	0.7	0.1	100.0	2,172	2.8
Pursat	22.5	51.1	6.5	17.0	2.0	1.0	0.0	100.0	1,239	2.3
Siem Reap	26.3	47.8	4.8	17.0	2.0	2.1	0.0	100.0	2,015	2.2
Svay Rieng	12.4	59.5	4.9	20.9	1.0	1.4	0.0	100.0	1,229	2.8
Takeo	22.7	40.0	5.1	27.5	2.7	2.0	0.0	100.0	2,023	3.6
Otdar Meanchey	28.4	45.5	5.3	18.9	1.5	0.5	0.0	100.0	509	1.9
Battambang/Pailin	16.5	47.0	5.5	25.1	2.9	2.9	0.0	100.0	2,446	3.6
Kampot/Kep	15.9	51.3	4.9	24.3	2.0	1.7	0.0	100.0	1,449	3.3
Preah Sihanouk/Koh Kong	15.8	46.2	6.8	25.2	2.3	3.6	0.0	100.0	696	3.9
Preah Vihear/Stung Treng	26.2	52.1	3.8	15.4	1.1	1.5	0.0	100.0	760	1.8
Mondul Kiri/Ratanak Kiri	33.9	43.6	4.2	16.7	0.8	0.9	0.0	100.0	639	1.2
**Wealth quintile**										
Lowest	28.9	56.8	4.3	9.6	0.3	0.1	0.0	100.0	6,013	1.4
Second	23.7	54.1	5.5	16.0	0.5	0.2	0.0	100.0	6,370	2.2
Middle	18.9	50.9	5.6	23.1	1.0	0.4	0.0	100.0	6,313	3.0
Fourth	15.0	44.7	6.8	28.2	2.9	2.5	0.0	100.0	6,507	4.0
Highest	9.2	34.8	6.0	34.5	5.7	9.9	0.0	100.0	6,625	5.7
Total	18.9	48.0	5.6	22.6	2.1	2.7	0.0	100.0	31,828	3.1

Note: Totals include 4 women with information on age missing.
[1] Completed grade 6 at the primary level
[2] Completed grade 12 at the secondary level

Table 2.4.2 Educational attainment of the male household population

Percent distribution of the de facto male household population age 6 and over by highest level of schooling attended or completed and median years completed, according to background characteristics, Cambodia 2014

Background characteristic	No education	Some primary	Completed primary[1]	Some secondary	Completed secondary[2]	More than secondary	Don't know/ missing	Total	Number	Median years completed
**Age**										
6-9	19.3	80.6	0.0	0.0	0.0	0.0	0.0	100.0	3,486	0.1
10-14	2.5	70.8	2.4	24.2	0.0	0.0	0.0	100.0	3,930	3.8
15-19	4.7	25.2	7.9	57.6	1.8	2.7	0.0	100.0	3,053	6.7
20-24	5.1	25.2	9.7	41.0	8.0	11.0	0.0	100.0	2,770	6.7
25-29	7.3	29.6	8.4	34.5	7.9	12.2	0.1	100.0	2,602	6.3
30-34	9.7	36.2	7.7	30.4	8.1	8.0	0.0	100.0	2,698	5.4
35-39	11.5	41.6	6.7	28.6	6.8	4.8	0.0	100.0	1,634	4.7
40-44	8.6	35.9	6.5	34.8	8.5	5.8	0.0	100.0	1,746	5.7
45-49	10.3	39.3	7.3	33.6	5.6	4.0	0.0	100.0	1,606	5.1
50-54	17.7	49.3	8.4	19.3	2.7	2.6	0.0	100.0	1,496	2.9
55-59	18.8	48.6	10.0	17.4	3.5	1.6	0.0	100.0	992	3.1
60-64	16.2	47.4	11.4	21.0	2.8	1.2	0.0	100.0	791	3.5
65+	23.4	40.5	11.8	20.4	2.5	1.3	0.1	100.0	1,572	3.6
**Residence**										
Urban	4.3	28.7	5.6	35.9	8.9	16.6	0.0	100.0	4,623	7.2
Rural	11.6	49.0	6.8	27.2	3.3	2.0	0.0	100.0	23,756	3.9
**Province**										
Banteay Meanchey	10.3	50.0	5.9	28.5	3.6	1.9	0.0	100.0	1,112	3.9
Kampong Cham	11.1	54.2	7.5	23.2	1.7	2.3	0.0	100.0	3,489	3.5
Kampong Chhnang	8.5	50.9	7.5	25.3	4.4	3.4	0.0	100.0	1,006	4.2
Kampong Speu	9.3	43.9	7.0	33.2	4.6	1.8	0.0	100.0	1,851	4.7
Kampong Thom	12.5	51.7	8.4	22.2	1.9	3.2	0.1	100.0	1,365	3.7
Kandal	8.9	45.3	6.7	32.2	4.4	2.6	0.0	100.0	2,287	4.6
Kratie	12.8	55.9	5.0	22.5	2.7	1.1	0.0	100.0	839	3.0
Phnom Penh	4.0	25.5	4.6	36.6	9.0	20.3	0.0	100.0	2,750	7.7
Prey Veng	9.8	45.9	6.8	33.4	2.3	1.6	0.1	100.0	1,922	4.5
Pursat	12.0	52.4	7.7	22.7	4.1	1.1	0.0	100.0	1,100	3.3
Siem Reap	20.1	47.9	5.1	19.3	4.5	3.0	0.1	100.0	1,807	2.7
Svay Rieng	3.8	46.4	6.3	34.5	5.3	3.7	0.0	100.0	1,053	5.0
Takeo	11.6	37.0	6.5	36.0	4.3	4.6	0.0	100.0	1,857	5.1
Otdar Meanchey	13.9	50.8	6.3	24.5	3.5	0.9	0.0	100.0	518	3.1
Battambang/Pailin	6.9	45.5	8.9	30.3	5.0	3.4	0.0	100.0	2,216	4.7
Kampot/Kep	8.3	48.0	6.8	29.9	4.4	2.7	0.0	100.0	1,245	4.3
Preah Sihanouk/Koh Kong	9.0	41.2	7.3	30.9	5.1	6.4	0.0	100.0	655	5.0
Preah Vihear/Stung Treng	19.3	55.5	3.9	16.0	2.5	2.9	0.0	100.0	651	2.3
Mondul Kiri/Ratanak Kiri	23.8	44.2	3.6	22.5	3.4	2.6	0.0	100.0	655	2.4
**Wealth quintile**										
Lowest	19.9	58.9	5.9	14.2	1.0	0.2	0.0	100.0	5,424	2.3
Second	12.9	56.3	6.9	21.6	1.5	0.8	0.0	100.0	5,669	3.2
Middle	9.5	47.4	8.0	30.5	2.9	1.7	0.0	100.0	5,614	4.3
Fourth	6.9	41.2	6.7	36.6	5.5	3.0	0.0	100.0	5,876	5.2
Highest	3.6	26.0	5.6	39.2	9.7	15.9	0.0	100.0	5,796	7.5
Total	10.4	45.7	6.6	28.7	4.2	4.4	0.0	100.0	28,379	4.3

[1] Completed grade 6 at the primary level
[2] Completed grade 12 at the secondary level

Forty-eight percent of females and 46 percent of males in the household population have had some primary schooling without having completed primary school. However, 37 percent of the male population has gone on to attend secondary or higher schooling, compared with only 27 percent of females. Sixty-two percent of males and 68 percent of females age 15-19 have gone on to secondary school. Sixty percent of males and 58 percent of females age 20-24 have done so. As would be expected, higher percentages of males and females in urban areas than rural areas have gone on to secondary schooling. There is a great deal of variation in educational attainment across provinces. The outliers are Mondul Kiri/Ratanak Kiri and Phnom Penh, where 24 percent and 4 percent of males, respectively, and 34 percent and 10 percent of females, respectively, have never been to school.

Data on net attendance ratios (NARs) and gross attendance ratios (GARs) by school level, sex, residence, and province are shown in Table 2.5. The NAR indicates participation in primary schooling for the population age 6-12 and secondary schooling for the population age 13-18. The GAR measures participation at each level of schooling among those age 6-24. The GAR is nearly always higher than the NAR for the same level because the GAR includes participation by those who may be older or younger than the official age range for that level. An NAR of 100 percent would indicate that all of those in the official age range for the level are attending at that level. The GAR can exceed 100 percent if there is significant overage or underage participation at a given level of schooling. Overage participation for a given level of schooling occurs when students start school earlier, repeat one or more grades, or drop out of school and later return.

Table 2.5 School attendance ratios

Net attendance ratios (NARs) and gross attendance ratios (GARs) for the de facto household population by sex and level of schooling, and the gender parity index (GPI), according to background characteristics, Cambodia 2014

Background characteristic	Net attendance ratio[1]				Gross attendance ratio[2]			
	Male	Female	Total	Gender parity index[3]	Male	Female	Total	Gender parity index[3]
PRIMARY SCHOOL								
**Residence**								
Urban	85.0	83.0	84.0	0.98	94.9	93.0	94.0	0.98
Rural	80.9	83.4	82.1	1.03	95.8	94.8	95.3	0.99
**Province**								
Banteay Meanchey	78.5	82.1	80.5	1.05	92.9	88.2	90.4	0.95
Kampong Cham	80.6	88.3	84.5	1.10	98.2	97.3	97.7	0.99
Kampong Chhnang	85.5	83.8	84.6	0.98	99.2	98.2	98.7	0.99
Kampong Speu	80.8	84.5	82.6	1.05	97.5	98.1	97.8	1.01
Kampong Thom	80.9	82.0	81.5	1.01	95.1	93.9	94.5	0.99
Kandal	77.4	81.0	79.0	1.05	87.8	87.8	87.8	1.00
Kratie	74.8	77.6	76.2	1.04	91.1	90.4	90.7	0.99
Phnom Penh	82.3	82.7	82.5	1.00	92.3	94.0	93.1	1.02
Prey Veng	87.1	79.6	83.7	0.91	98.4	92.7	95.8	0.94
Pursat	81.4	80.5	80.9	0.99	98.8	96.9	97.9	0.98
Siem Reap	78.8	82.7	80.7	1.05	88.2	92.6	90.3	1.05
Svay Rieng	87.0	88.5	87.8	1.02	98.9	96.4	97.6	0.97
Takeo	83.6	86.4	85.0	1.03	100.6	96.2	98.4	0.96
Otdar Meanchey	81.3	78.8	80.2	0.97	101.3	89.4	95.7	0.88
Battambang/Pailin	86.7	84.2	85.5	0.97	100.4	96.4	98.4	0.96
Kampot/Kep	85.0	86.9	85.9	1.02	98.2	97.1	97.6	0.99
Preah Sihanouk/Koh Kong	83.6	82.2	82.9	0.98	99.0	96.1	97.6	0.97
Preah Vihear/Stung Treng	72.6	78.5	75.5	1.08	94.5	96.2	95.3	1.02
Mondul Kiri/Ratanak Kiri	67.1	75.0	71.2	1.12	88.5	94.0	91.4	1.06
**Wealth quintile**								
Lowest	75.5	82.0	78.6	1.09	91.4	96.6	93.9	1.06
Second	81.7	82.9	82.3	1.01	96.8	97.2	97.0	1.00
Middle	82.9	83.5	83.3	1.01	97.2	93.0	95.1	0.96
Fourth	84.7	86.6	85.6	1.02	100.6	94.4	97.5	0.94
Highest	85.0	82.1	83.6	0.97	93.6	89.9	91.7	0.96
Total	81.4	83.4	82.4	1.02	95.7	94.5	95.1	0.99
SECONDARY SCHOOL								
**Residence**								
Urban	58.9	54.3	56.5	0.92	75.5	67.7	71.4	0.90
Rural	39.5	42.3	40.9	1.07	48.6	51.5	50.0	1.06
**Province**								
Banteay Meanchey	46.6	43.0	44.8	0.92	53.7	52.4	53.1	0.98
Kampong Cham	35.1	46.7	40.9	1.33	43.3	55.6	49.4	1.28
Kampong Chhnang	46.7	49.9	48.4	1.07	52.3	58.1	55.4	1.11
Kampong Speu	41.4	30.6	36.2	0.74	50.0	35.9	43.2	0.72
Kampong Thom	36.8	43.6	40.1	1.18	47.6	54.0	50.8	1.13
Kandal	40.8	37.9	39.3	0.93	53.1	42.5	47.9	0.80
Kratie	29.7	38.0	33.7	1.28	38.0	46.7	42.2	1.23
Phnom Penh	57.9	49.9	53.9	0.86	73.0	61.1	67.1	0.84
Prey Veng	50.8	51.5	51.1	1.01	58.8	65.5	61.9	1.11
Pursat	24.4	33.5	28.9	1.37	36.7	43.4	40.0	1.18
Siem Reap	34.6	34.9	34.8	1.01	44.1	44.3	44.2	1.00
Svay Rieng	58.0	47.3	53.2	0.82	71.5	61.9	67.3	0.87
Takeo	58.1	66.8	62.1	1.15	74.9	82.1	78.2	1.10
Otdar Meanchey	29.8	32.6	31.1	1.09	36.1	41.3	38.5	1.14
Battambang/Pailin	40.5	52.8	46.8	1.30	48.2	65.4	57.0	1.36
Kampot/Kep	47.4	54.8	50.9	1.16	59.0	66.0	62.3	1.12
Preah Sihanouk/Koh Kong	45.4	43.3	44.3	0.95	53.1	56.2	54.7	1.06
Preah Vihear/Stung Treng	21.2	27.1	24.2	1.28	28.7	34.2	31.6	1.19
Mondul Kiri/Ratanak Kiri	23.6	20.2	21.8	0.85	30.2	24.2	27.1	0.80
**Wealth quintile**								
Lowest	17.0	25.0	20.8	1.47	23.1	29.6	26.2	1.28
Second	30.7	34.4	32.5	1.12	37.2	41.8	39.4	1.12
Middle	45.9	45.6	45.8	0.99	58.5	56.3	57.4	0.96
Fourth	53.1	55.4	54.2	1.04	64.6	65.3	64.9	1.01
Highest	66.5	58.9	62.5	0.88	81.4	75.1	78.1	0.92
Total	42.4	44.3	43.3	1.05	52.6	54.2	53.4	1.03

[1] The NAR for primary school is the percentage of the primary school age (6-12 years) population that is attending primary school. The NAR for secondary school is the percentage of the secondary school age (13-18 years) population that is attending secondary school. By definition the NAR cannot exceed 100 percent.
[2] The GAR for primary school is the total number of primary school students, expressed as a percentage of the official primary school age population. The GAR for secondary school is the total number of secondary school students, expressed as a percentage of the official secondary school age population. If there are significant numbers of overage and underage students at a given level of schooling, the GAR can exceed 100 percent.
[3] The gender parity index for primary school is the ratio of the primary school NAR (GAR) for females to the NAR (GAR) for males. The gender parity index for secondary school is the ratio of the secondary school NAR (GAR) for females to the NAR (GAR) for males.

Of those children who should be attending primary school, 81 percent of females and 83 percent of males are currently doing so. In 2010, 85 percent of children who should have been attending primary school were doing so. The NAR is significantly lower at the secondary school level and at about the same level found in 2010. Forty-three percent of secondary school-age youths are in school at that level (this figure was 44 percent in 2010). Similar to 2010, there is little difference between the NAR of males and females at both the primary and the secondary level.

Table 2.5 also shows the gender parity index (GPI) for primary and secondary school. The GPI for primary school is the ratio of the primary school NAR/GAR for females to the NAR/GAR for males. The GPI for secondary school is the ratio of the secondary school NAR/GAR for females to the NAR/GAR for males. The primary school GPI for NAR of 1.02 indicates gender parity at the primary level, reflecting the fact that about the same proportions of girls and boys attend primary school. The GPI for NAR of 1.05 at the secondary school level indicates near parity at the secondary level. The GPIs for NAR in urban areas and rural areas indicate parity or near parity at the primary level (0.98 and 1.03, respectively). However, the GPI for NAR at the secondary level in urban areas is 0.92, reflecting the fact that a smaller proportion of girls than boys in urban areas attend secondary school. The GPI for NAR varies across provinces, and this variation is far more evident at the secondary school level than at the primary school level (Table 2.5). The GPI for GAR at the primary level (0.99) and at the secondary level (1.03) indicates near parity. The primary school GPI and the secondary school GPI for GAR follow patterns of the GPIs for NAR.

Figure 2.2 illustrates age-specific attendance rates, that is, the percentage of a given age cohort attending school regardless of the level attended (primary, secondary, or higher). Although the minimum age for schooling in Cambodia is 6 years, some children enroll prior to this age, and only about three in every five children age 6 are attending school.

Figure 2.2 **Age-specific attendance rates**

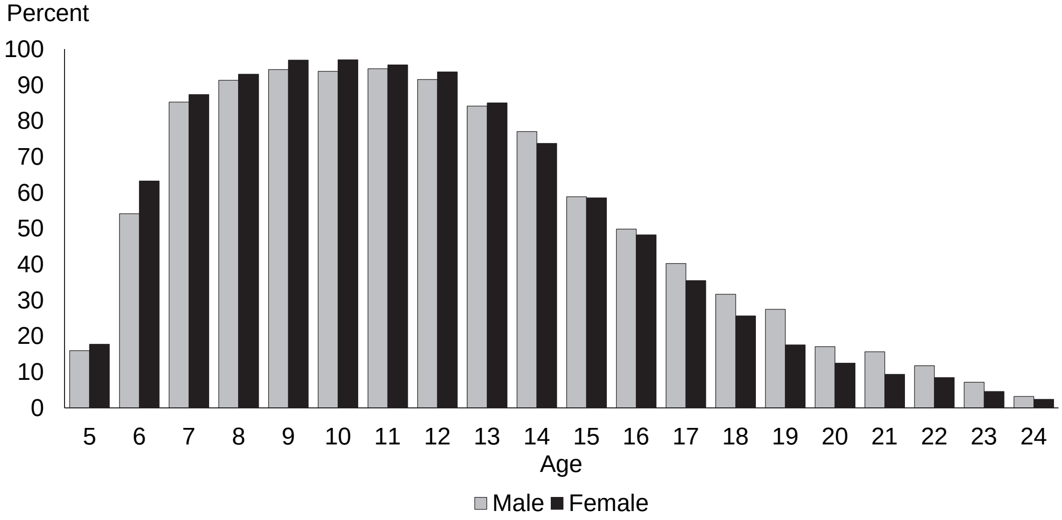

Note: Figure shows percentage of the de jure household population age 5-24 years attending school

CDHS 2014

Similar to 2010, boys and girls attend school in about equal proportions. Up to and including age 13, the proportion of girls attending school is slightly higher than for boys, and then it is slightly lower than for boys at age 14 to 16. From age 17 to 23, young men attend school at a noticeably higher proportion than young women. At age 24, the proportions of men and women attending school are about equal.

2.3 HOUSING CHARACTERISTICS

Types of water sources and sanitation facilities are important determinants of the health status of household members and particularly of children. Proper hygienic and sanitation practices can reduce

exposure to and the seriousness of major childhood diseases such as diarrhea. The CDHS asked respondents about the household source of drinking water, the time required round trip to obtain that water, and the type of sanitation facility used by the household. In Cambodia, the source of drinking water can vary between the dry season and the rainy season, so separate questions were asked for the different seasons. If households had more than one source of drinking water, respondents were asked to identify the most commonly used source.

2.3.1 Water Supply

Table 2.6 shows that sources of drinking water were the same during the dry and rainy seasons for 92 percent of urban households and 67 percent of rural households. The source of drinking water is an indicator of whether it is suitable for drinking. Sources that are considered likely to be of suitable quality are listed under “Improved source” and those that may not be of suitable quality are listed under “Non-improved source,” reflecting the categorizations proposed by the World Health Organization (WHO), the United Nations Children’s Fund (UNICEF), and the Joint Monitoring Programme (JMP) for Water Supply and Sanitation.

During the dry season, 35 percent of households in Cambodia consume drinking water from a non-improved source. This percentage declines to 16 percent of households during the rainy season, when more households utilize rainwater for drinking water. The main source of drinking water during the rainy season is rainwater for nearly two of five households. Rainwater is the most common source of drinking water during the rainy season for rural households.

Even if water is not piped directly into the dwelling or yard, it is common for the source of water to be on the household premises, especially during the rainy season. Seventy-five percent of households report that their source of drinking water during the rainy season is located on the household premises. The variation between urban households and rural households is insignificant. During the dry season, the percentage of households with their source of drinking water on the premises declines to 69 percent and 51 percent among urban and rural households, respectively. Among those households neither having a source of drinking water on the premises nor having water delivered, the majority are within 30 minutes or less in round trip time of obtaining it. During the dry season only 6 percent of households are 30 minutes or longer away from a source, and during the rainy season that number drops to just 2 percent requiring 30 minutes or more.

Table 2.6 Household drinking water

Percent distribution of households and de jure population by source of drinking water, time to obtain drinking water, and treatment of drinking water, according to residence, Cambodia 2014

	Households			Population		
Characteristic	Urban	Rural	Total	Urban	Rural	Total
**Source of drinking water during dry season**						
Improved source	95.0	60.1	65.2	95.1	58.8	64.5
Piped water into dwelling/yard/plot	51.7	5.7	12.3	54.0	5.7	13.3
Public tap/standpipe	1.8	0.5	0.7	1.9	0.5	0.8
Tube well or borehole	7.7	31.1	27.8	7.2	30.0	26.4
Protected dug well	1.4	4.0	3.6	1.4	4.2	3.8
Protected spring	0.2	0.3	0.3	0.2	0.3	0.3
Rainwater	5.5	10.1	9.4	4.9	9.6	8.9
Bottled water	26.7	8.4	11.0	25.7	8.4	11.1
Non-improved source	4.9	39.8	34.8	4.7	41.2	35.4
Unprotected dug well	1.6	13.4	11.7	1.5	13.8	11.9
Unprotected spring	0.1	1.3	1.1	0.1	1.3	1.1
Tanker truck/cart with small tank	1.6	3.9	3.6	1.6	3.9	3.6
Surface water	1.6	21.2	18.4	1.4	22.2	18.9
Other	0.1	0.0	0.0	0.2	0.0	0.0
Total	100.0	100.0	100.0	100.0	100.0	100.0
**Time to obtain drinking water (round trip)**						
Water on premises	69.1	51.3	53.9	70.4	50.9	54.0
Less than 30 minutes	27.6	39.5	37.8	26.1	39.5	37.4
30 minutes or longer	1.3	7.1	6.3	1.6	7.5	6.6
Don’t know/missing	2.0	2.1	2.1	1.9	2.1	2.1
Total	100.0	100.0	100.0	100.0	100.0	100.0
**Source of drinking water during rainy season**						
Improved source	97.6	81.4	83.7	97.5	80.6	83.3
Piped water into dwelling/yard/plot	49.9	4.7	11.2	51.9	4.6	12.1
Public tap/standpipe	1.6	0.5	0.7	1.7	0.5	0.7
Tube well or borehole	6.7	25.4	22.7	6.2	24.1	21.3
Protected dug well	1.2	2.8	2.5	1.2	2.9	2.6
Protected spring	0.2	0.2	0.2	0.1	0.2	0.2
Rainwater	13.4	40.8	36.9	12.7	41.4	36.9
Bottled water	24.7	7.0	9.6	23.6	6.9	9.5
Non-improved source	2.3	18.5	16.2	2.3	19.3	16.6
Unprotected dug well	1.1	9.3	8.1	1.1	9.5	8.2
Unprotected spring	0.0	0.7	0.6	0.0	0.8	0.7
Tanker truck/cart with small tank	0.6	0.9	0.8	0.7	0.9	0.8
Surface water	0.6	7.7	6.6	0.6	8.1	6.9
Other	0.1	0.0	0.0	0.1	0.0	0.0
Missing	0.0	0.1	0.1	0.0	0.1	0.1
Total	100.0	100.0	100.0	100.0	100.0	100.0
**Time to obtain drinking water (round trip) during rainy season**						
Water on premises	73.7	74.9	74.8	74.7	75.2	75.1
Less than 30 minutes	24.0	21.9	22.2	22.8	21.4	21.6
30 minutes or longer	0.8	2.1	1.9	0.9	2.3	2.1
Don’t know/missing	1.5	1.1	1.1	1.5	1.1	1.2
Total	100.0	100.0	100.0	100.0	100.0	100.0
**Percentage using same water within dry and rainy season**	91.5	67.3	70.8	91.7	66.2	70.2
**Water treatment prior to drinking**[1]						
Boiled	56.7	54.9	55.1	56.6	54.4	54.7
Bleach/chlorine added	0.1	0.3	0.2	0.0	0.3	0.2
Strained through cloth	0.3	0.7	0.6	0.3	0.7	0.6
Ceramic, sand, or other filter	15.9	16.7	16.6	17.0	16.9	16.9
Solar disinfection	0.3	0.1	0.1	0.3	0.1	0.1
Stand and settle	0.7	5.3	4.7	0.7	5.3	4.6
Other	0.6	0.2	0.3	0.5	0.2	0.3
No treatment	30.4	30.8	30.8	29.9	31.4	31.2
Percentage using an appropriate treatment method[2]	68.7	67.0	67.3	69.3	66.6	67.0
Number	2,284	13,541	15,825	11,469	61,489	72,958

[1] Respondents may report multiple treatment methods, so the sum of treatment may exceed 100 percent.
[2] Appropriate water treatment methods include boiling, bleaching, filtering, and solar disinfecting.

Fifty-five percent of households boil their water prior to drinking. There is little variation between urban and rural areas in the proportion of households that boil their water prior to drinking. Seventeen percent of households use a ceramic, sand, or other type of filter to filter their water prior to drinking. Among those that do not boil their water, the most common action is to do nothing to treat the water prior to drinking. Overall, 31 percent of households report that they do nothing to treat their drinking water before consuming it. Drinking water without prior treatment is equally likely among urban and rural households. However, the likelihood of drinking water without prior treatment is somewhat higher than in 2010.

2.3.2 Sanitation Facilities

A household's toilet facility is classified as hygienic if it is used only by household members (is not shared by other households) and if the type of toilet effectively separates human waste from human contact. The types of facilities most likely to accomplish this are toilets that flush or pour flush into a piped sewer system, septic tank, or pit latrine; ventilated improved pit (VIP) latrines; pit latrines with a slab; and composting toilets. Households that share their toilet facility or do not effectively separate human waste from human contact are classified as unhygienic. These categories are those proposed by the WHO/UNICEF Joint Monitoring Program.

Table 2.7 Household sanitation facilities

Percent distribution of households and de jure population by type of toilet/latrine facilities, according to residence, Cambodia 2014

	Households			Population		
Type of toilet/latrine facility	Urban	Rural	Total	Urban	Rural	Total
**Improved, not shared facility**						
Flush/pour flush to piped sewer system	37.4	0.7	6.0	38.8	0.7	6.7
Flush/pour flush to septic tank	45.2	37.2	38.4	45.7	38.6	39.7
Flush/pour flush to pit latrine	0.5	1.0	0.9	0.4	0.9	0.9
Ventilated improved pit (VIP) latrine	0.0	0.1	0.0	0.0	0.0	0.0
Pit latrine with slab	0.1	0.5	0.5	0.1	0.6	0.5
Composting toilet	0.0	0.2	0.2	0.0	0.3	0.2
Total	83.2	39.7	46.0	85.0	41.2	48.1
**Shared facility**[1]						
Flush/pour flush to piped sewer system	2.3	0.2	0.5	1.8	0.2	0.5
Flush/pour flush to septic tank	6.1	8.7	8.3	5.5	8.3	7.9
Flush/pour flush to pit latrine	0.1	0.2	0.2	0.1	0.1	0.1
Ventilated improved pit (VIP) latrine	0.0	0.0	0.0	0.0	0.0	0.0
Pit latrine with slab	0.0	0.1	0.1	0.0	0.1	0.1
Composting toilet	0.0	0.0	0.0	0.0	0.0	0.0
Total	8.6	9.1	9.0	7.5	8.7	8.5
**Non-improved facility**						
Flush/pour flush not to sewer/septic tank/pit latrine	1.0	0.2	0.3	1.0	0.2	0.3
Pit latrine without slab/open pit	0.0	0.1	0.1	0.0	0.1	0.1
Bucket	0.0	0.1	0.1	0.0	0.1	0.1
Hanging toilet/hanging latrine	0.3	0.4	0.4	0.3	0.4	0.4
No facility/bush/field	6.9	50.4	44.1	6.2	49.3	42.5
Missing	0.0	0.0	0.0	0.0	0.0	0.0
Total	8.2	51.2	45.0	7.6	50.1	43.4
Total	100.0	100.0	100.0	100.0	100.0	100.0
Number	2,284	13,541	15,825	11,469	61,489	72,958

[1] Facilities that would be considered improved if they were not shared by two or more households

Households vary greatly in access to hygienic facilities by urban and rural residence, as shown in Table 2.7. The majority of households in rural areas have no toilet facility, with half of households (50 percent) reporting no toilet facility and making use of fields or bush areas. This figure was reported among only 7 percent of urban households. Access to hygienic facilities has improved substantially, as the percentage of households which have no facilities declined from 57 percent in 2010 to 44 percent in 2014.

2.3.3 Hand Washing

Washing hands with water and soap before preparing and eating food and after leaving the toilet is a simple and inexpensive practice that protects against many diseases. During the survey, interviewers asked to see the place members of the household used for hand washing and observed whether water and soap or some other cleansing agent was available.

Table 2.8 shows that interviewers observed a place for hand washing in 85 percent of households—a significant increase from 66 percent observed in 2010. Eighty percent of these households had water and soap for hand washing, and 19 percent had water only. In urban areas, nearly all households (97 percent) had a place for hand washing, as compared with 83 percent of households in rural areas. Ninety-four percent of urban households had soap and water available at a hand washing place, compared with only 77 percent of rural households. A higher percentage of households in rural areas than urban areas had water but no soap (22 percent versus 6 percent).

Among the provinces, interviewers observed a place for hand washing in only 42 percent of the households in Mondul Kiri/Ratanak Kiri and 55 percent of the households in Takeo. Among households where a place for hand washing was observed, the lowest proportions with soap and water were in Takeo (59 percent) and Kandal (60 percent). The proportion of households with a place for hand washing increases with increasing wealth, from 74 percent among households in the lowest quintile to 96 percent among those in the highest quintile. Thirty percent of households in the lowest wealth quintile have water but no soap, compared with only 6 percent of households in the highest quintile.

Table 2.8 Hand washing

Percentage of households in which the place most often used for washing hands was observed, and among households in which the place for hand washing was observed, percent distribution by availability of water, soap, and other cleansing agents, Cambodia 2014

Background characteristic	Percentage of households where place for washing hands was observed	Number of households	Among households where place for hand washing was observed, percentage with: Soap and water[1]	Water and cleansing agent[2] other than soap only	Water only	Soap but no water[3]	No water, no soap, no other cleansing agent	Missing	Total	Number of households with place for hand washing observed
**Residence**										
Urban	97.1	2,284	94.1	0.0	5.7	0.0	0.1	0.0	100.0	2,217
Rural	82.6	13,541	77.0	0.1	22.0	0.2	0.8	0.0	100.0	11,189
**Province**										
Banteay Meanchey	98.3	670	94.1	0.2	5.7	0.0	0.0	0.0	100.0	658
Kampong Cham	82.0	1,997	89.4	0.1	10.2	0.2	0.0	0.0	100.0	1,638
Kampong Chhnang	83.1	608	87.2	0.0	12.8	0.0	0.0	0.0	100.0	506
Kampong Speu	99.0	973	69.8	0.0	30.2	0.0	0.0	0.0	100.0	963
Kampong Thom	97.5	801	85.3	0.1	11.5	0.5	2.5	0.0	100.0	781
Kandal	89.5	1,259	59.8	0.0	39.3	0.3	0.6	0.0	100.0	1,127
Kratie	87.9	451	70.9	0.8	26.6	0.8	1.0	0.0	100.0	397
Phnom Penh	98.8	1,293	98.5	0.0	1.4	0.0	0.0	0.0	100.0	1,278
Prey Veng	60.1	1,228	93.0	0.2	6.8	0.0	0.0	0.0	100.0	738
Pursat	96.5	611	61.8	0.0	36.8	0.0	1.4	0.0	100.0	589
Siem Reap	63.6	1,000	95.8	0.0	4.2	0.0	0.0	0.0	100.0	636
Svay Rieng	93.2	678	78.2	0.0	21.5	0.1	0.2	0.0	100.0	632
Takeo	54.9	1,011	59.1	0.1	35.3	0.3	5.3	0.0	100.0	555
Otdar Meanchey	89.9	271	89.2	0.0	10.7	0.0	0.2	0.0	100.0	244
Battambang/Pailin	96.7	1,222	60.9	0.0	37.5	0.2	1.4	0.0	100.0	1,181
Kampot/Kep	90.2	762	70.4	0.0	29.0	0.4	0.2	0.0	100.0	687
Preah Sihanouk/Koh Kong	99.8	320	98.1	0.0	1.9	0.0	0.0	0.0	100.0	319
Preah Vihear/Stung Treng	96.1	361	88.7	0.0	11.2	0.1	0.0	0.0	100.0	346
Mondul Kiri/Ratanak Kiri	41.9	309	77.3	0.4	22.2	0.0	0.0	0.1	100.0	130
**Wealth quintile**										
Lowest	73.7	3,208	68.1	0.1	30.4	0.3	1.1	0.0	100.0	2,364
Second	79.6	3,320	74.0	0.1	24.8	0.2	0.8	0.0	100.0	2,642
Middle	85.1	3,147	77.9	0.1	21.1	0.1	0.8	0.0	100.0	2,677
Fourth	90.0	3,176	82.3	0.0	16.8	0.2	0.6	0.0	100.0	2,859
Highest	96.3	2,975	94.2	0.0	5.8	0.0	0.1	0.0	100.0	2,865
Total	84.7	15,825	79.8	0.1	19.3	0.2	0.7	0.0	100.0	13,406

[1] Soap includes soap or detergent in bar, liquid, powder, or paste form. This column includes households with soap and water only as well as those that had soap and water and another cleansing agent.
[2] Cleansing agents other than soap include locally available materials such as ash, mud, or sand.
[3] Includes households with soap only as well as those with soap and another cleansing agent

2.3.4 Flooring Material and Cooking Arrangements

Table 2.9 presents the distribution of households by dwelling characteristics. Nearly all households in urban areas (97 percent) live in dwellings with electricity, whereas in rural areas only about half of households (49 percent) have electricity. Ceramic tiles are the most common type of flooring material in urban areas, and wood planks are the most common material in rural areas. Thirty-six percent of urban households live in dwellings with ceramic tiles, followed by 26 percent who live in dwellings with wood planks. In rural areas, approximately half of households live in dwellings with wood plank flooring, followed by one-quarter who live in dwellings with palm or bamboo flooring[1]. About two-thirds of rural households (66 percent) sleep together in one room, whereas only 42 percent of urban households do so. In urban areas, 57 percent of households use two or more rooms for sleeping.

Firewood is the most common source of fuel for cooking in rural areas, with 85 percent of rural households using firewood for this purpose. There is more variability in urban areas as to what is used for cooking fuel. Twenty-two percent of urban households use firewood, 59 percent use liquid petroleum gas, and 16 percent use charcoal. Sixty-one percent of urban households and 37 percent of rural households report that they do their cooking in the house.

Table 2.9 Household characteristics

Percent distribution of households by housing characteristics and percentage of households using solid fuel for cooking, according to residence, Cambodia 2014

	Households			Population		
Housing characteristic	Urban	Rural	Total	Urban	Rural	Total
**Electricity**						
Yes	96.9	49.2	56.1	97.3	49.8	57.1
No	3.1	50.8	43.9	2.7	50.2	42.8
Total	100.0	100.0	100.0	100.0	100.0	100.0
**Flooring material**						
Earth, sand	3.1	9.2	8.3	2.9	8.7	7.8
Dung	0.1	0.0	0.0	0.1	0.0	0.0
Wood/planks	26.1	51.1	47.5	26.7	52.7	48.7
Palm/bamboo	3.7	23.6	20.7	3.5	22.7	19.8
Parquet or polished wood	0.1	0.1	0.1	0.1	0.1	0.1
Vinyl or asphalt strips	0.0	0.1	0.1	0.0	0.0	0.0
Ceramic tiles	35.6	5.3	9.6	35.3	5.5	10.1
Cement tiles	19.2	2.8	5.2	20.0	2.8	5.5
Cement	12.1	7.7	8.4	11.4	7.2	7.9
Floating house	0.1	0.1	0.1	0.0	0.2	0.2
Total	100.0	100.0	100.0	100.0	100.0	100.0
**Rooms used for sleeping**						
One	42.2	65.8	62.4	38.1	63.9	59.9
Two	27.4	23.8	24.3	27.5	24.7	25.2
Three or more	29.8	8.7	11.8	33.8	9.8	13.5
Missing	0.6	1.7	1.5	0.6	1.6	1.4
Total	100.0	100.0	100.0	100.0	100.0	100.0
**Cooking fuel**						
Electricity	2.1	0.6	0.8	2.0	0.6	0.8
LPG/natural gas/biogas	58.8	7.7	15.0	59.0	7.2	15.2
Charcoal	16.3	6.5	7.9	16.3	6.5	8.0
Wood	22.1	84.6	75.6	22.3	85.2	75.4
Agricultural crop	0.0	0.3	0.2	0.0	0.3	0.2
Animal dung	0.0	0.1	0.1	0.0	0.1	0.1
No food cooked in household	0.7	0.2	0.3	0.4	0.1	0.1
Total	100.0	100.0	100.0	100.0	100.0	100.0
**Place for cooking**						
In the house	60.9	37.0	40.4	60.6	36.5	40.3
In a separate building	17.1	25.8	24.6	17.8	26.9	25.5
Outdoors	20.4	34.1	32.1	20.4	33.5	31.5
No food cooked in household	1.4	3.1	2.8	1.1	3.0	2.7
Other	0.1	0.0	0.0	0.0	0.0	0.0
Total	100.0	100.0	100.0	100.0	100.0	100.0
Number	2,284	13,541	15,825	13,753	75,030	88,783

LPG = Liquid petroleum gas

[1] If there was more than one type of flooring, interviewers recorded the predominant flooring material.

2.4 HOUSEHOLD POSSESSIONS

Information on ownership of durable goods and other possessions is presented in Table 2.10. The availability of durable consumer goods is a good indicator of a household's socioeconomic level, and particular goods have specific benefits. For example, radio access can increase exposure to innovative ideas, whereas transport vehicles can provide access to services out of the local area.

Sixty-six percent of households in Cambodia own a television, and 87 percent own a mobile telephone. Ownership of mobile telephones is almost universal among urban households (96 percent) and is very common among rural households (86 percent). About two of five households (39 percent) own a generator/battery or a solar panel.

Twenty-six percent of urban households own a car, truck, or van, an increase from 22 percent in 2010. About two-thirds of all households (68 percent) own a motorcycle, an increase from 54 percent of households in 2010. The percentage of households owning a boat remains unchanged at about 8 percent.

Sixty-nine percent of all households own some land, which is about the same as the 2010 figure of 68 percent. Sixty-six percent of households own at least one farm animal, also about the same as the figure reported in 2010 (67 percent).

Table 2.10 Household possessions

Percentage of households possessing various household effects, means of transportation, agricultural land, and livestock/farm animals by residence, Cambodia 2014

	Households			Population		
Possession	Urban	Rural	Total	Urban	Rural	Total
**Household effects**						
Radio	50.4	38.2	40.0	52.9	38.4	40.7
Television	91.4	61.4	65.7	93.6	63.9	68.6
Mobile telephone	96.1	85.7	87.2	97.1	88.4	89.8
Non-mobile telephone	12.2	5.5	6.5	13.2	5.8	7.0
Refrigerator	40.4	2.5	8.0	43.5	2.7	9.1
Wardrobe	72.7	38.5	43.4	75.1	39.7	45.3
Sewing machine	17.6	6.7	8.3	19.2	7.1	9.0
CD/DVD player	47.7	27.3	30.2	51.4	30.0	33.4
Generator/battery/solar	7.9	43.9	38.7	9.2	44.9	39.3
Watch	43.7	14.9	19.0	47.7	16.2	21.1
**Means of transport**						
Bicycle/cyclo	54.9	65.7	64.2	59.8	68.9	67.5
Animal-drawn cart	1.0	14.2	12.3	1.1	15.4	13.2
Motorcycle/scooter	83.2	65.5	68.0	86.6	69.9	72.5
Car/truck	26.0	12.1	14.1	29.1	13.4	15.8
Boat with a motor	1.3	4.5	4.1	1.7	5.3	4.8
Motorcycle cart	6.7	3.3	3.8	8.5	3.7	4.5
Boat without a motor	1.1	4.7	4.1	1.3	5.3	4.6
Ownership of agricultural land	28.7	75.9	69.1	29.5	77.1	69.6
Ownership of farm animals[1]	21.9	73.0	65.6	22.9	76.2	67.8
Number	2,284	13,541	15,825	11,469	61,489	72,958

[1] Water buffaloes, cows, bulls, horses, donkeys, mules, goats, sheep, pigs, chickens, ducks, or elephants

2.5 HOUSEHOLD WEALTH

In addition to standard background characteristics, many of the results in this report are shown by wealth quintiles, an indicator of the economic status of households. The 2014 CDHS did not collect data on consumption or income, but the information collected on dwelling and household characteristics, consumer goods, and assets is used as a measure of socioeconomic status. The resulting wealth index is an indicator of relative level of wealth that is used as a proxy for expenditure and income measures.

Each household asset for which information is collected is assigned a weight or factor score generated through principal components analysis. The resulting asset scores are standardized in relation to a standard normal distribution with a mean of zero and a standard deviation of one.

These standardized scores are then used to create the break points that define wealth quintiles. Each household is assigned a standardized score for each asset, where the score differs depending on whether or not the household owns that asset (or, in the case of sleeping arrangements, the number of people per room). These scores are summed by household, and individuals are ranked according to the total score of the household in which they reside. The sample is then divided into population quintiles (i.e., five groups with the same number of individuals in each). At the national level, approximately 20 percent of the household population is grouped into each wealth quintile.

A single asset index is developed on the basis of data from the entire country sample and used in all of the tabulations presented. The reader should keep in mind that wealth quintiles are expressed in terms of quintiles of individuals in the population rather than quintiles of individuals at risk for any one health or population indicator. For example, quintile rates for infant mortality refer to infant mortality rates per 1,000 live births among all people in the population quintile concerned, as distinct from quintiles of live births or newly born infants, who constitute the only members of the population at risk of mortality during infancy.

The wealth index has been compared against poverty rates and gross domestic product per capita in India and against expenditure data from household surveys in Nepal, Pakistan, and Indonesia (Filmer and Pritchett, 1998) as well as Guatemala (Rutstein, 1999). The evidence from those studies suggests that the asset index is highly comparable to conventionally measured consumption expenditures.

Table 2.11 shows the distribution of the household population into five wealth quintiles (five equally divided levels) based on the wealth index by residence. These distributions indicate the degree to which wealth is evenly (or unevenly) distributed across Cambodia. As expected, urban areas are wealthier than rural areas. For example, 84 percent of Phnom Penh's population falls in the highest wealth quintile. By contrast, Pursat has the lowest representation in the highest wealth quintile, with only 5 percent of its population falling in that quintile.

Table 2.11 Wealth quintiles

Percent distribution of the de jure population by wealth quintiles, according to residence and province, Cambodia 2014

	Wealth quintile						
Residence/region	Lowest	Second	Middle	Fourth	Highest	Total	Number of persons
**Residence**							
Urban	1.3	1.8	4.1	14.9	78.0	100.0	11,469
Rural	23.5	23.4	23.0	20.9	9.2	100.0	61,489
**Province**							
Banteay Meanchey	6.0	12.1	22.6	36.4	22.9	100.0	3,134
Kampong Cham	25.2	21.0	25.3	17.5	11.0	100.0	9,454
Kampong Chhnang	35.5	25.6	17.9	11.2	9.9	100.0	2,574
Kampong Speu	20.4	21.3	25.6	24.4	8.2	100.0	4,665
Kampong Thom	35.9	27.5	15.3	12.7	8.8	100.0	3,632
Kandal	5.3	13.3	27.4	33.7	20.2	100.0	5,674
Kratie	43.0	21.5	14.7	15.0	5.8	100.0	2,160
Phnom Penh	0.3	1.3	3.0	11.0	84.4	100.0	6,814
Prey Veng	22.6	27.6	26.9	15.6	7.3	100.0	4,942
Pursat	34.2	29.6	16.0	15.5	4.7	100.0	2,839
Siem Reap	30.9	25.3	15.8	11.6	16.4	100.0	4,811
Svay Rieng	23.4	30.0	24.7	14.9	6.9	100.0	2,736
Takeo	8.5	21.4	27.2	34.3	8.6	100.0	4,475
Otdar Meanchey	23.5	27.3	19.8	17.2	12.2	100.0	1,203
Battambang/Pailin	9.1	13.6	20.8	29.4	27.1	100.0	5,623
Kampot/Kep	23.2	28.5	23.6	17.2	7.5	100.0	3,220
Preah Sihanouk/Koh Kong	8.0	9.3	11.0	28.3	43.5	100.0	1,622
Preah Vihear/Stung Treng	47.1	27.3	12.9	7.0	5.7	100.0	1,813
Mondul Kiri/Ratanak Kiri	30.8	26.1	11.4	13.5	18.1	100.0	1,567
Total	20.0	20.0	20.0	20.0	20.0	100.0	72,958

2.6 BIRTH REGISTRATION

The registration of births is the inscription of the facts of a birth into an official log. A birth certificate is issued as proof of the registration of the birth. Information on the registration of births was

collected in the household interview by asking whether children under age 5 had a birth certificate. If the interviewer was told that the child did not have a birth certificate, the interviewer probed further to ascertain whether the child's birth had been registered with the civil authority. Nearly two-thirds of children (64 percent) had a birth certificate, and the births of 73 percent of children under age 5 were registered. These figures are significantly higher than those found in the 2010 CDHS (51 percent and 62 percent, respectively). However, levels of registration varied greatly across the country, as shown in Table 2.12.

Table 2.12 Birth registration of children under age 5

Percentage of de jure children under age 5 whose births are registered with the civil authorities, according to background characteristics, Cambodia 2014

Background characteristic	Children whose births are registered			
	Percentage who had a birth certificate	Percentage who did not have a birth certificate	Percentage registered	Number of children
**Age**				
<2	59.4	7.8	67.2	3,125
2-4	66.8	10.5	77.4	4,680
**Sex**				
Male	64.8	8.9	73.7	3,940
Female	62.9	10.0	72.9	3,865
**Residence**				
Urban	75.5	8.8	84.4	1,066
Rural	62.0	9.5	71.6	6,739
**Province**				
Banteay Meanchey	61.7	10.5	72.2	372
Kampong Cham	54.1	15.8	69.9	1,086
Kampong Chhnang	71.5	4.0	75.5	263
Kampong Speu	74.0	4.2	78.1	478
Kampong Thom	59.7	4.0	63.7	364
Kandal	80.1	4.0	84.1	530
Kratie	40.5	4.8	45.3	271
Phnom Penh	84.9	4.5	89.4	607
Prey Veng	73.7	5.5	79.2	592
Pursat	52.0	10.7	62.7	313
Siem Reap	70.5	2.0	72.6	536
Svay Rieng	84.7	2.8	87.5	297
Takeo	60.4	15.3	75.7	408
Otdar Meanchey	73.5	7.9	81.4	140
Battambang/Pailin	32.6	37.8	70.5	613
Kampot/Kep	75.8	1.1	76.9	321
Preah Sihanouk/Koh Kong	72.8	0.8	73.6	170
Preah Vihear/Stung Treng	62.8	3.7	66.5	234
Mondul Kiri/Ratanak Kiri	32.8	7.0	39.7	211
**Wealth quintile**				
Lowest	52.5	6.7	59.1	1,878
Second	60.7	8.8	69.6	1,586
Middle	65.6	9.9	75.4	1,554
Fourth	69.1	11.7	80.8	1,347
Highest	75.5	11.1	86.6	1,439
Total	63.9	9.4	73.3	7,805

2.7 CHILDREN'S LIVING ARRANGEMENTS, ORPHANHOOD, AND SCHOOL ATTENDANCE BY SURVIVORSHIP OF PARENTS

2.7.1 Children's Living Arrangements and Orphanhood

Because the family is the primary safety net for children, any strategy aimed at protecting children must place a high priority on strengthening the family's capacities to care for children. It is therefore essential to identify orphaned children and find out whether those who have one or both parents alive are living with either or both surviving parents. Table 2.13 presents these two types of information for children under age 18, according to background characteristics.

Table 2.13 Children's living arrangements and orphanhood

Percent distribution of de jure children under age 18 by living arrangements and survival status of parents, the percentage of children not living with a biological parent, and the percentage of children with one or both parents dead, according to background characteristics, Cambodia 2014

		Living with mother but not with father		Living with father but not with mother		Not living with either parent								
Background characteristic	Living with both parents	Father alive	Father dead	Mother alive	Mother dead	Both alive	Only father alive	Only mother alive	Both dead	Missing information on father/ mother	Total	Percentage not living with a biological parent	Percentage with one or both parents dead[1]	Number of children
**Age**														
0-4	82.8	6.4	0.9	0.6	0.1	8.5	0.1	0.3	0.3	0.0	100.0	9.2	1.7	7,805
<2	85.9	7.2	0.7	0.3	0.0	5.3	0.2	0.2	0.2	0.0	100.0	5.8	1.2	3,125
2-4	80.7	5.9	1.1	0.8	0.1	10.7	0.1	0.4	0.3	0.0	100.0	11.4	2.0	4,680
5-9	78.3	5.3	2.2	1.1	0.6	10.6	0.4	0.7	0.8	0.0	100.0	12.5	4.6	8,377
10-14	76.1	5.5	4.6	1.2	0.9	9.2	0.6	0.9	0.8	0.1	100.0	11.5	7.8	8,069
15-17	72.3	5.8	7.2	1.3	1.9	8.0	0.8	1.1	1.5	0.1	100.0	11.4	12.5	3,963
**Sex**														
Male	77.9	5.9	3.4	1.0	0.7	9.3	0.5	0.6	0.7	0.1	100.0	11.0	5.8	14,346
Female	78.2	5.5	3.1	1.1	0.8	9.3	0.4	0.8	0.8	0.0	100.0	11.3	5.9	13,868
**Residence**														
Urban	75.1	7.2	2.6	1.5	0.7	10.2	0.7	0.8	0.9	0.2	100.0	12.7	5.6	3,745
Rural	78.5	5.5	3.4	1.0	0.7	9.1	0.4	0.7	0.7	0.0	100.0	10.9	5.9	24,470
**Province**														
Banteay Meanchey	67.6	4.5	2.9	0.8	0.4	23.2	0.3	0.1	0.2	0.0	100.0	23.8	3.8	1,251
Kampong Cham	77.7	5.8	3.4	0.9	0.9	9.0	0.2	0.8	1.2	0.1	100.0	11.2	6.6	3,723
Kampong Chhnang	74.4	7.9	5.7	1.2	0.7	9.0	0.3	0.4	0.4	0.0	100.0	10.1	7.4	1,014
Kampong Speu	85.6	5.1	2.1	0.4	0.8	5.5	0.1	0.2	0.2	0.0	100.0	6.0	3.4	1,832
Kampong Thom	78.9	6.4	3.3	0.8	0.9	8.1	0.4	0.3	0.8	0.0	100.0	9.7	5.7	1,514
Kandal	81.3	6.4	3.9	0.3	0.5	6.3	0.2	0.5	0.7	0.0	100.0	7.6	5.7	2,047
Kratie	84.5	3.7	2.2	0.6	0.4	6.0	1.7	0.5	0.4	0.0	100.0	8.6	5.1	885
Phnom Penh	77.3	7.0	2.2	1.3	0.8	8.4	0.9	0.9	0.9	0.3	100.0	11.1	5.6	2,079
Prey Veng	66.9	10.0	3.3	1.4	0.8	16.1	0.3	0.6	0.5	0.0	100.0	17.5	5.5	1,913
Pursat	81.9	3.6	4.0	1.7	0.2	7.1	0.3	0.6	0.6	0.1	100.0	8.6	5.7	1,156
Siem Reap	80.0	5.1	4.3	0.6	1.0	6.6	0.5	0.8	0.9	0.1	100.0	8.8	7.5	2,037
Svay Rieng	78.7	4.6	2.5	1.3	0.4	10.5	0.2	0.7	1.0	0.0	100.0	12.5	4.9	976
Takeo	73.6	6.5	4.2	1.7	0.7	9.5	0.1	2.3	1.3	0.0	100.0	13.2	8.6	1,678
Otdar Meanchey	83.8	2.3	2.5	1.5	0.4	7.1	0.5	0.4	1.5	0.1	100.0	9.4	5.3	515
Battambang/Pailin	74.9	5.1	1.5	2.0	0.9	13.2	0.7	0.9	0.7	0.2	100.0	15.5	4.7	2,243
Kampot/Kep	79.9	3.6	3.2	0.8	0.8	9.9	0.8	0.7	0.4	0.0	100.0	11.7	5.9	1,246
Preah Sihanouk/ Koh Kong	81.5	5.8	1.9	1.9	0.8	7.1	0.1	0.6	0.3	0.0	100.0	8.1	3.7	612
Preah Vihear/Stung Treng	85.6	3.9	6.1	0.5	0.6	2.3	0.3	0.1	0.5	0.0	100.0	3.2	7.6	808
Mondul Kiri/Ratanak Kiri	86.2	4.5	3.4	0.5	0.6	2.8	0.6	0.3	1.1	0.0	100.0	4.8	5.9	685
**Wealth quintile**														
Lowest	79.5	5.5	4.5	1.0	0.9	7.0	0.3	0.6	0.8	0.0	100.0	8.7	7.0	6,616
Second	77.7	5.7	3.7	1.1	1.0	8.9	0.5	0.5	0.8	0.0	100.0	10.8	6.5	6,023
Middle	77.4	6.1	2.6	1.2	0.5	10.2	0.4	0.8	0.6	0.1	100.0	12.0	5.0	5,574
Fourth	77.2	5.5	3.0	0.7	0.5	11.1	0.5	0.8	0.7	0.0	100.0	13.1	5.5	5,213
Highest	78.3	5.9	2.0	1.1	0.7	9.7	0.4	0.8	0.8	0.2	100.0	11.8	4.7	4,788
Total <15	79.0	5.7	2.6	1.0	0.5	9.5	0.4	0.6	0.6	0.1	100.0	11.1	4.7	24,252
Total <18	78.1	5.7	3.3	1.0	0.7	9.3	0.4	0.7	0.7	0.1	100.0	11.1	5.8	28,215

Note: Table is based on de jure members, i.e., usual residents.
[1] Includes children with father dead, mother dead, both dead, and one parent dead but missing information on survival status of the other parent

The data show that 78 percent of Cambodian children under age 18 live with both of their parents. This proportion declines steadily with age, from a high of 86 percent among children under age 2 to a low of 72 percent among children age 15 to 17. There is little variation according to the child's sex. The proportion of children living with both of their parents is slightly higher in rural areas (79 percent) than in urban areas (75 percent). The lowest proportions of children living with both parents are in Prey Veng (67 percent) and Banteay Meanchey (68 percent). Nine percent of children under age 18 live with their mother only, whether their father is alive (6 percent) or deceased (3 percent), and 2 percent live with their father only. Eleven percent do not live with either parent.

Overall, 6 percent of children under age 18 have lost one or both parents: less than 1 percent have lost both parents, 5 percent have lost their father, and 2 percent have lost their mother. Because a parent's

risk of dying increases with time, the proportion of children who have lost their father and/or mother increases significantly with age, from 1 percent among children less than age 2 and 2 percent among children age 2 to 4 to 5 percent among children age 5 to 9. It increases further to 8 percent among children age 10 to 14 and 13 percent among children age 15 to 17.

2.7.2 School Attendance by Survivorship of Parents

Access to education is considered an "essential service" and is included among the key components of national responses to guarantee orphans access to services on an equal basis with other children.

To assess whether orphans are educationally disadvantaged in relation to other children, an indicator was devised to compare school attendance among orphans and non-orphans. The results are presented in Table 2.14 for children age 10 to 14, the age group in which school attendance is generally assumed for all children.

The data show a clear relationship between parent survivorship and school attendance of children age 10 to 14. According to the 2014 CDHS, 89 percent of children whose parents are both alive and who are living with one or both of their parents attend school, as compared with only 78 percent of children who have lost both parents. The ratio of school attendance for orphaned and non-orphaned children is less than 1 (0.88), indicating an educational disadvantage for orphans.

Table 2.14 School attendance by survivorship of parents

For de jure children 10-14 years of age, the percentage attending school by parental survival and the ratio of the percentage attending, by parental survival, according to background characteristics, Cambodia 2014

Background characteristic	Percentage attending school by survivorship of parents: Both parents deceased	Number	Both parents alive and living with at least one parent	Number	Ratio[1]
**Sex**					
Male	(65.0)	24	88.4	3,360	0.74
Female	(86.0)	39	89.1	3,323	0.97
**Residence**					
Urban	(89.9)	8	93.4	842	0.96
Rural	(76.2)	55	88.1	5,841	0.86
**Province**					
Banteay Meanchey	*	0	85.4	248	1.17
Kampong Cham	*	2	91.0	882	1.10
Kampong Chhnang	*	3	93.5	240	1.07
Kampong Speu	*	3	86.7	504	1.15
Kampong Thom	*	5	84.7	375	0.72
Kandal	*	6	83.4	475	0.35
Kratie	*	2	89.6	202	1.12
Phnom Penh	*	2	94.2	452	1.06
Prey Veng	*	5	93.1	414	0.37
Pursat	*	2	86.2	298	1.10
Siem Reap	*	6	81.5	479	1.23
Svay Rieng	*	1	93.2	246	1.07
Takeo	*	6	95.2	386	0.82
Otdar Meanchey	*	4	86.1	125	0.85
Battambang/Pailin	*	8	90.0	489	1.11
Kampot/Kep	*	3	90.8	326	0.73
Preah Sihanouk/Koh Kong	*	1	92.0	157	0.88
Preah Vihear/Stung Treng	*	1	84.3	196	1.19
Mondul Kiri/Ratanak Kiri	*	2	80.5	189	1.02
**Wealth quintile**					
Lowest	*	18	81.0	1,626	0.81
Second	*	14	86.7	1,446	0.79
Middle	*	10	89.9	1,339	0.83
Fourth	*	11	93.9	1,249	1.04
Highest	*	10	96.1	1,023	0.99
Total	78.0	63	88.7	6,683	0.88

Note: Table is based only on children who usually live in the household.
[1] Ratio of the percentage with both parents deceased to the percentage with both parents alive and living with a parent

UTILIZATION OF HEALTH SERVICES FOR ACCIDENT, ILLNESS, OR INJURY — 3

Key Findings

- Two percent of household members were injured or killed in an accident in the years before the survey.
- Seven in 10 injuries or deaths are attributed to road accidents.
- Thirteen percent of household members had an illness or injury in the month before the survey. Among them 95 percent sought a first treatment, 22 percent a second treatment, and 7 percent a third treatment.

In 1998, the Ministry of Health was beginning to implement a redesigned health coverage plan created to improve the accessibility and quality of government health services. The major aim of the new health care plan was to create a network of health centers throughout the country delivering the "Minimum Package of Activities" services. The data collected in the 1998 National Health Survey provided a baseline of health conditions in the country before implementation of the new health coverage plan. The CDHS surveys implemented in 2000, 2005, and 2010 assessed progress every five years under the coverage plan, and the 2014 CDHS provides updated progress on those health conditions.

Utilization of health services was assessed in the Household Questionnaire. The questions were asked to all households in the sample. First, information was collected to assess the prevalence of injuries and deaths due to accidents in the past year. Second, the respondent was asked whether any household members suffered from any physical impairment. Third, the respondent was asked about the severity of illness or injury and the subsequent utilization of health services among all members of the household who had been ill or injured in the 30 days preceding the interview.

3.1 ACCIDENTAL DEATH OR INJURY

All households reported on whether any household member had suffered accidental injury or death in the 12 months preceding the household interview. If anyone had been injured, the cause of the injury was recorded. The respondent to the Household Questionnaire was further asked whether the accident victim was alive or dead and, if dead, whether the death was the result of the reported accident. The questions were designed in this order to definitively assess the cause of injury and, if a death was noted, the cause of death.

3.1.1 Frequency of Accidental Death or Injury

Accidental injuries and deaths in Cambodia were not common (Table 3.1). Two percent of the population had suffered an injury or death by accident in the past 12 months. Accidental injuries were much more common than accidental deaths; for every 1,000 people in the population, 17 suffered an injury and 1 suffered an accidental death.

The percentage of the population injured in the past 12 months increased with age from 0.7 percent among children age 0-9 to a peak of 2.6 percent among adults age 20-39. The percentage experiencing accidental injury decreased thereafter, to 1.7 percent among adults age 40-59 and 1.5 percent among those age 60 and above.

Males were more than twice as likely as females to be injured in an accident. Overall, 2.4 percent of males had been injured in an accident in the past 12 months, as compared with 1.1 percent of females.

Although there were no differences in accidental injuries by urban-rural residence, there were differences across provinces. The highest percentage of accidental injury was reported in Kratie, with 3.3 percent of the household population experiencing an injury in the preceding 12 months. The lowest rates of accidental injury were in Preah Vihear/Stung Treng (0.2 percent) and Otdar Meanchey (0.8 percent). The percentage of accidental death ranged from 0.0 to 0.2 percent across provinces.

Table 3.1 Injury or death in an accident

Percentage of the de jure household population injured or killed in an accident in the past 12 months, according to background characteristics, Cambodia 2014

Background characteristic	Result of accident		Total injured or killed	Total number of de jure household members
	Injured	Killed		
**Age**				
0-9	0.7	0.2	0.9	16,182
10-19	1.6	0.1	1.6	14,576
20-39	2.6	0.0	2.6	22,161
40-59	1.7	0.0	1.7	13,959
60+	1.5	0.0	1.6	6,079
**Sex**				
Male	2.4	0.0	2.5	35,336
Female	1.1	0.1	1.1	37,622
**Residence**				
Urban	1.7	0.0	1.7	11,469
Rural	1.7	0.1	1.8	61,489
**Province**				
Banteay Meanchey	1.4	0.1	1.5	3,134
Kampong Cham	2.1	0.0	2.1	9,454
Kampong Chhnang	2.9	0.1	3.0	2,574
Kampong Speu	1.4	0.1	1.5	4,665
Kampong Thom	1.4	0.0	1.4	3,632
Kandal	2.1	0.0	2.1	5,674
Kratie	3.3	0.0	3.3	2,160
Phnom Penh	1.7	0.0	1.7	6,814
Prey Veng	1.1	0.1	1.2	4,942
Pursat	1.1	0.1	1.2	2,839
Siem Reap	1.6	0.0	1.7	4,811
Svay Rieng	1.4	0.1	1.5	2,736
Takeo	1.6	0.1	1.7	4,475
Otdar Meanchey	0.8	0.0	0.8	1,203
Battambang/Pailin	2.1	0.1	2.2	5,623
Kampot/Kep	1.7	0.1	1.8	3,220
Preah Sihanouk/Koh Kong	2.3	0.0	2.3	1,622
Preah Vihear/Stung Treng	0.2	0.0	0.3	1,813
Mondul Kiri/Ratanak Kiri	1.6	0.2	1.8	1,567
Total	1.7	0.1	1.8	72,958

3.1.2 Type of Accident

Table 3.2 presents data on accidental injury by type of accident, according to the background characteristics of age, sex, residence, and province. Data on accidental deaths are also included, but these data are not available by age and sex.

Road accidents accounted for the greatest proportion of accidental injuries and deaths. More than 7 of 10 people who had been injured or killed in the previous 12 months were injured as a result of a road accident. Nine percent of injuries/deaths were the result of a fall, and 5 percent were the result of a snake or animal bite. Two percent of injuries/deaths resulted from violence. One percent of injuries/deaths were the result of burning, while less than 1 percent each were the result of a gunshot, drowning, and poisoning. Nine percent of injuries/deaths were due to other or unknown causes.

Table 3.2 Injury or death in an accident by type of accident

Percentage of the de jure household population injured or killed in an accident in the past 12 months by type of accident, according to age and sex, Cambodia 2014

Background characteristic	Type of accident: Gunshot	Road accident	Severe burning	Snake/ animal bite	Fall from tree/ building	Drowning[1]	Poisoning (chemical)	Violence	Other	Don't know/ missing	Total	Number of persons injured
INJURED												
**Age**												
0-9	0.0	48.7	2.0	13.5	22.5	0.0	0.0	1.5	6.6	5.1	100.0	121
10-19	0.0	67.8	2.0	5.1	9.2	0.0	0.0	3.7	12.1	0.0	100.0	230
20-39	0.7	79.4	1.1	3.3	6.1	0.0	0.4	2.7	6.3	0.0	100.0	581
40-59	0.1	73.1	0.9	4.5	11.1	0.0	0.6	1.6	8.2	0.0	100.0	243
60+	0.0	60.3	0.0	6.4	13.1	0.0	0.0	1.1	17.6	1.4	100.0	92
**Sex**												
Male	0.5	74.1	0.2	4.9	8.6	0.0	0.3	3.0	8.2	0.3	100.0	864
Female	0.0	66.8	3.5	5.6	12.0	0.0	0.3	1.3	9.3	1.3	100.0	402
Total	0.3	71.8	1.2	5.1	9.7	0.0	0.3	2.4	8.6	0.6	100.0	1,267
INJURED OR KILLED												
**Residence**												
Urban	1.5	81.8	0.6	2.0	5.5	0.0	0.1	3.1	4.8	0.5	100.0	197
Rural	0.3	69.6	1.3	5.5	10.1	0.9	0.3	2.3	9.2	0.6	100.0	1,109
**Province**												
Banteay Meanchey	0.0	62.5	9.5	4.0	14.4	0.0	0.0	0.0	9.6	0.0	100.0	47
Kampong Cham	0.0	67.3	1.8	6.6	14.4	0.0	0.0	0.0	8.0	1.9	100.0	199
Kampong Chhnang	0.0	64.8	0.0	2.7	15.4	1.1	0.0	3.4	12.6	0.0	100.0	78
Kampong Speu	0.0	90.4	0.0	0.0	4.2	0.0	0.0	0.0	5.4	0.0	100.0	69
Kampong Thom	0.4	63.7	2.9	7.0	14.6	2.3	0.0	2.4	4.5	2.2	100.0	53
Kandal	0.0	71.1	1.5	2.2	6.2	0.0	0.0	5.2	13.8	0.0	100.0	120
Kratie	0.0	56.4	0.8	10.7	15.5	0.0	4.4	0.9	11.4	0.0	100.0	72
Phnom Penh	2.3	84.5	0.8	3.5	2.8	0.0	0.0	2.0	4.1	0.0	100.0	115
Prey Veng	(5.2)	(71.9)	(0.0)	(3.0)	(8.2)	(5.5)	(0.0)	(2.6)	(3.5)	(0.0)	(100.0)	60
Pursat	(0.0)	(59.9)	(0.0)	(6.8)	(19.1)	(8.6)	(0.0)	(0.5)	(5.0)	(0.0)	(100.0)	34
Siem Reap	0.0	81.1	0.0	0.0	8.2	0.0	0.0	5.9	4.9	0.0	100.0	81
Svay Rieng	0.0	75.8	3.8	0.0	12.7	0.0	0.0	0.0	7.8	0.0	100.0	41
Takeo	0.0	68.9	1.7	5.4	6.7	2.0	0.0	1.8	11.9	1.7	100.0	76
Otdar Meanchey	(0.0)	(89.0)	(0.0)	(0.0)	(6.6)	(0.0)	(0.0)	(4.4)	(0.0)	(0.0)	(100.0)	10
Battambang/Pailin	0.0	80.4	0.0	2.9	5.5	0.0	0.0	4.1	7.0	0.0	100.0	123
Kampot/Kep	0.0	56.0	0.0	17.2	6.5	0.0	0.5	3.9	13.9	2.1	100.0	57
Preah Sihanouk/Koh Kong	0.0	67.1	0.0	4.0	8.2	0.0	0.0	6.5	14.1	0.0	100.0	38
Preah Vihear/Stung Treng	*	*	*	*	*	*	*	*	*	*	*	5
Mondul Kiri/Ratanak Kiri	0.0	63.8	0.0	20.5	2.2	2.1	0.0	0.8	10.5	0.0	100.0	28
Total	0.5	71.4	1.2	4.9	9.4	0.8	0.3	2.4	8.5	0.6	100.0	1,306

Note: Figures in parentheses are based on 25-49 unweighted cases. An asterisk indicates that a figure is based on fewer than 25 unweighted cases and has been suppressed.
[1]All drowning cases reported were deceased

Cause of injury varied by age, but road accidents were the most commonly cited source of injury for people of all ages, especially those age 20-39. After road accidents, animal/snake bites and falls from trees/buildings were the most common causes of injuries among children age 0-9, accounting for 14 percent and 23 percent of injuries, respectively. Gunshots accounted for a higher percentage of injuries among people age 20-39 than for any other age group. Severe burning accounted for a higher percentage among children and young adults less than age 20 than among other age groups. Violence as a cause of injury was most common among people age 10-39. There were significant differences in accidental injuries in the preceding 12 months by sex. While males were more likely than females to be injured in road accidents (74 percent versus 67 percent), females were more likely to be injured from severe burning and falls than males.

There were other significant differences in accidental injuries/deaths in the preceding 12 months by urban-rural residence and province. Not surprisingly, road accidents accounted for a higher percentage of injuries/deaths in urban areas (82 percent) than in rural areas (70 percent). Falls accounted for a higher proportion of accidental injuries or deaths in rural areas than in urban areas (10 percent versus 6 percent). The distribution of causes of injuries/deaths by province should be analyzed with caution because sample sizes were small in some provinces.

3.2 PREVALENCE AND SEVERITY OF ILLNESS OR INJURY

All households were asked whether any members had been sick or injured at any time in the 30 days before the interview. If any members had been sick, their names were recorded to ask specifically about their conditions in the questions that followed. The Household Questionnaire allotted space for information to be recorded for up to three household members. Interviewers were instructed to use extra questionnaires to record the information on all household members who were ill or injured. The respondent was asked to judge the illness or injury as slight, moderate, or serious. Finally, questions were asked as to whether ill or injured household members sought care, where they sought care, how much they spent on transport, and how much they spent on treatment. These questions were repeated to collect information on patterns of health care-seeking behavior. For example, a man might first seek treatment from a Kru Khmer traditional healer but later visit a health clinic if the illness continued. Up to three care-seeking attempts were recorded on the questionnaire for each ill or injured person.

Thirteen percent of household members had been ill in the 30 days prior to the interview (Table 3.3). However, this percentage may underrepresent the actual prevalence of morbidity and injury for two reasons. The questions were asked only about living household members at the time of the interview. Therefore, the recorded episodes of illness and injury excluded any cases that ended in the death of a household member in the 30 days prior to the interview. Furthermore, the responses were based on the 30-day recall of one respondent in the household. That respondent might not have been aware of all of the illnesses or injuries that had occurred within the household. It is likely that illnesses or injuries that occurred at the beginning of the 30-day period or that were of mild severity were forgotten and not reported.

Table 3.3 Prevalence and severity of illness or injury in previous 30 days

Percent distribution of the de jure household population ill or injured in the previous 30 days by severity of illness or injury, according to background characteristics, Cambodia 2014

	Severity of illness or injury					
Background characteristics	Not ill or injured	Slight	Moderate	Serious	Any illness or injury	Number of persons
**Age**						
0-9	83.1	10.1	5.7	1.2	16.9	16,182
10-19	93.7	3.0	2.6	0.6	6.3	14,576
20-39	90.6	3.9	4.6	0.9	9.4	22,161
40-59	82.7	6.2	9.3	1.8	17.3	13,959
60+	75.5	7.5	13.4	3.6	24.5	6,079
**Sex**						
Male	88.8	5.1	4.8	1.3	11.2	35,336
Female	84.9	6.5	7.3	1.3	15.1	37,622
**Residence**						
Urban	84.7	9.1	5.2	0.9	15.3	11,469
Rural	87.1	5.2	6.3	1.4	12.9	61,489
**Province**						
Banteay Meanchey	87.8	4.3	6.0	1.8	12.2	3,134
Kampong Cham	86.3	6.2	6.1	1.4	13.7	9,454
Kampong Chhnang	82.0	5.8	10.7	1.5	18.0	2,574
Kampong Speu	85.9	7.2	5.9	0.9	14.1	4,665
Kampong Thom	87.7	5.9	5.3	1.1	12.3	3,632
Kandal	87.3	4.1	7.2	1.3	12.7	5,674
Kratie	82.7	6.6	9.0	1.6	17.3	2,160
Phnom Penh	76.8	15.8	6.8	0.6	23.2	6,814
Prey Veng	92.1	1.8	5.0	1.1	7.9	4,942
Pursat	93.7	2.3	2.8	1.2	6.3	2,839
Siem Reap	90.9	2.9	5.1	1.1	9.1	4,811
Svay Rieng	83.2	7.6	7.6	1.6	16.8	2,736
Takeo	91.3	1.9	4.6	2.2	8.7	4,475
Otdar Meanchey	89.9	4.2	5.2	0.7	10.1	1,203
Battambang/Pailin	86.7	6.6	5.1	1.5	13.3	5,623
Kampot/Kep	86.9	3.0	9.0	1.1	13.1	3,220
Preah Sihanouk/Koh Kong	88.3	5.5	5.0	1.2	11.7	1,622
Preah Vihear/Stung Treng	85.0	5.7	7.1	2.0	15.0	1,813
Mondul Kiri/Ratanak Kiri	91.9	4.8	2.0	1.3	8.1	1,567
Total	86.8	5.8	6.1	1.3	13.2	72,958

The majority (90 percent) of all illnesses or injuries were slight or moderate in severity. Only 1.3 percent of household members experienced a serious illness or injury. The highest percentage of illness or injury was found among persons age 60 and older; 25 percent had an illness or injury. Females and urban residents suffered slightly more illnesses and injuries than males and rural residents. The highest percentages of illness or injury were found in Phnom Penh (23 percent), Kampong Chhnang (18 percent), and Kratie and Svay Rieng (17 percent each).

3.3 Treatment Sought for Illness or Injury

Table 3.4 presents the percentage of ill or injured household members who sought treatment according to the number of times they did so. The type of treatment recorded included, but was not limited to, care provided by medically trained professionals. For example, if a sick child was first given a remedy by a Kru Khmer traditional healer, this was recorded as the first treatment. If the parents later observed that the child was still ill and went to a shop in the market for medicine, this was recorded as the second treatment. If the medicine was not effective and the parents took the child to a doctor at a private clinic, this was recorded as the third treatment.

Table 3.4 Percentage of ill or injured population who sought treatment

Percentage of de jure household members ill or injured in the past 30 days who sought a first, second, and third treatment, according to background characteristics, Cambodia 2014

Background characteristics	Treatment for illness or injury			Number of ill/injured population
	First treatment	Second treatment	Third treatment	
**Severity of illness or injury**[1]				
Slight	93.2	17.8	5.2	4,249
Moderate	96.3	23.5	7.7	4,442
Serious	98.4	36.1	12.6	956
**Age**				
0-9	97.1	21.8	6.1	2,742
10-19	97.0	18.5	5.2	912
20-39	95.0	24.1	7.2	2,094
40-59	93.6	22.9	8.6	2,416
60+	93.1	21.4	7.4	1,492
**Sex**				
Male	95.6	22.3	6.8	3,973
Female	94.8	22.1	7.3	5,683
**Residence**				
Urban	96.1	28.5	11.5	1,755
Rural	94.9	20.8	6.1	7,902
**Province**				
Banteay Meanchey	96.1	16.8	4.9	381
Kampong Cham	93.0	17.3	5.8	1,296
Kampong Chhnang	99.2	26.9	5.1	464
Kampong Speu	96.7	7.5	2.0	657
Kampong Thom	96.2	9.5	1.5	448
Kandal	95.6	31.6	11.5	718
Kratie	94.5	9.2	1.1	373
Phnom Penh	96.8	37.9	16.3	1,582
Prey Veng	98.3	40.4	16.2	390
Pursat	91.1	12.3	0.0	180
Siem Reap	96.5	23.8	6.2	440
Svay Rieng	95.7	18.9	3.4	460
Takeo	94.3	28.0	10.9	389
Otdar Meanchey	87.8	16.6	1.0	121
Battambang/Pailin	91.2	15.0	3.2	748
Kampot/Kep	96.5	16.6	3.5	420
Preah Sihanouk/Koh Kong	97.2	17.1	3.8	189
Preah Vihear/Stung Treng	92.2	16.4	1.5	273
Mondul Kiri/Ratanak Kiri	86.1	13.7	3.2	126
Total	95.1	22.2	7.1	9,656

[1] Includes 10 cases of don't know or missing severity of illness or injury

Ninety-five percent of household members who were ill sought at least one treatment (Table 3.4), a slight increase from the 2010 CDHS. Twenty-two percent of those ill or injured sought at least two treatments, and 7 percent sought at least three treatments. In general, there was a positive relationship between the severity of illness or injury and the number of times treatment was sought. Persons with serious illnesses or injuries were more likely to seek treatment than those with moderate illnesses or injuries. These latter individuals in turn were more likely to seek treatment than those with slight illnesses or injuries. Ninety-three percent of those with a slight illness, 96 percent of those with a moderate illness, and 98 percent of those with a serious illness or injury sought a first treatment. The corresponding percentages among those who sought a second treatment were 18 percent, 24 percent, and 36 percent. Five percent of those with slight illnesses or injuries were treated three times or more, as compared with 13 percent of those with serious illnesses or injuries. There were small differences in health-seeking behavior by sex and age. Urban residents were twice as likely to seek a third treatment as rural residents (12 percent versus 6 percent).

The provinces with the highest percentages of ill or injured persons seeking treatment were Kampong Chhnang (99 percent) and Prey Veng (98 percent), whereas the province with the lowest percentage was Mondul Kiri/Ratanak Kiri (86 percent).

3.4 UTILIZATION OF HEALTH CARE FACILITIES

Information on the location of health care providers was collected to determine where persons who were ill or injured went for treatment. Health care providers were distinguished by public sector, private sector, and non-medical sector. Interviewers were provided with descriptions of the different types of hospitals, clinics, pharmacies, and other health venues. If, during data collection, the interviewer had difficulties distinguishing among the various types, the team supervisor or field editor ascertained the correct designation from local sources.

Table 3.5 presents data on utilization of health services by type of residence (urban-rural). Small differences in patterns of health care use can be observed, with the private sector in general used most often, followed by the public sector and then the non-medical sector.

Within the public sector, health centers were most often visited for treatment of illnesses and injuries in rural areas (13 percent), whereas national hospitals were the most common source for treatment in urban areas (7 percent). Within the private sector, private pharmacies were most often visited for treatment in urban areas (41 percent), and private clinics were the most common source in rural areas (17 percent). Private pharmacies were much more likely to be visited for first treatment in urban areas than in rural areas (41 percent versus 13 percent), whereas trained health workers and nurses were more commonly sought out for first-time treatment in rural areas than in urban areas (29 percent versus 10 percent). Within the non-medical sector, shops or markets were the overwhelming choice as a source of health care.

Table 3.5 Percentage of ill or injured population who sought treatment

Percent distribution of de jure household members who were ill or injured in the past 30 days by place of treatment, according to urban-rural residence, Cambodia 2014

	Residence								
	Urban			Rural			Total		
Place of treatment	First treatment	Second treatment	Third treatment	First treatment	Second treatment	Third treatment	First treatment	Second treatment	Third treatment
**Did not seek treatment**	**3.9**	**71.5**	**88.5**	**5.1**	**79.2**	**93.9**	**4.9**	**77.8**	**92.9**
**Public sector**	**14.9**	**4.0**	**1.3**	**23.5**	**4.8**	**1.4**	**21.9**	**4.7**	**1.4**
National hospital (PP)	6.5	2.2	0.9	3.6	1.0	0.5	4.2	1.2	0.5
Provincial hospital (RH)	2.2	0.2	0.1	3.1	0.6	0.1	3.0	0.5	0.1
District hospital (RH)	0.5	0.4	0.0	2.9	0.7	0.3	2.5	0.7	0.2
Health center	5.0	1.1	0.4	12.8	2.2	0.5	11.4	2.0	0.4
Health post	0.0	0.0	0.0	0.2	0.0	0.0	0.2	0.0	0.0
Outreach	0.0	0.0	0.0	0.0	0.0	0.0	0.0	0.0	0.0
Other public	0.7	0.1	0.0	0.8	0.2	0.1	0.7	0.2	0.1
**Private sector**	**78.1**	**23.9**	**9.9**	**64.7**	**14.5**	**4.3**	**67.1**	**16.2**	**5.3**
Private hospital	3.3	1.0	0.2	3.6	1.0	0.3	3.6	1.0	0.3
Private clinic	22.6	6.7	2.4	17.2	5.0	1.3	18.2	5.3	1.5
Private pharmacy	40.6	13.4	6.1	12.7	2.2	0.8	17.8	4.2	1.8
Home/office of trained health worker/nurse	5.4	1.6	0.4	14.4	3.7	1.0	12.8	3.3	0.9
Visit of trained health worker/nurse	4.7	0.9	0.6	15.0	2.2	0.8	13.1	2.0	0.8
Other private medical	1.5	0.4	0.2	1.7	0.4	0.1	1.7	0.4	0.1
**Non-medical sector**	**1.0**	**0.4**	**0.2**	**5.3**	**1.1**	**0.3**	**4.5**	**1.0**	**0.3**
Shop/market	0.7	0.2	0.1	4.3	0.5	0.1	3.6	0.4	0.1
Kru Khmer/magician	0.3	0.1	0.1	0.9	0.6	0.1	0.8	0.5	0.1
Monk/religious leader	0.0	0.0	0.0	0.1	0.0	0.0	0.1	0.0	0.0
Traditional birth attendant	0.0	0.1	0.0	0.0	0.0	0.0	0.0	0.0	0.0
**Outside of country/other**	**2.1**	**0.2**	**0.1**	**1.5**	**0.3**	**0.1**	**1.6**	**0.3**	**0.1**
Total	100.0	100.0	100.0	100.0	100.0	100.0	100.0	100.0	100.0
Number	1,755	1,755	1,755	7,902	7,902	7,902	9,656	9,656	9,656

Figure 3.1 summarizes the findings detailed in Table 3.5. The private sector is the most popular source for all three types of treatments. After the private sector, people most often choose the public sector for first, second, and third treatments, whereas the non-medical sector is the least popular choice for seeking treatment.

Figure 3.1 **Percentage of ill or injured household members seeking treatment by order of treatment and sector of health care**

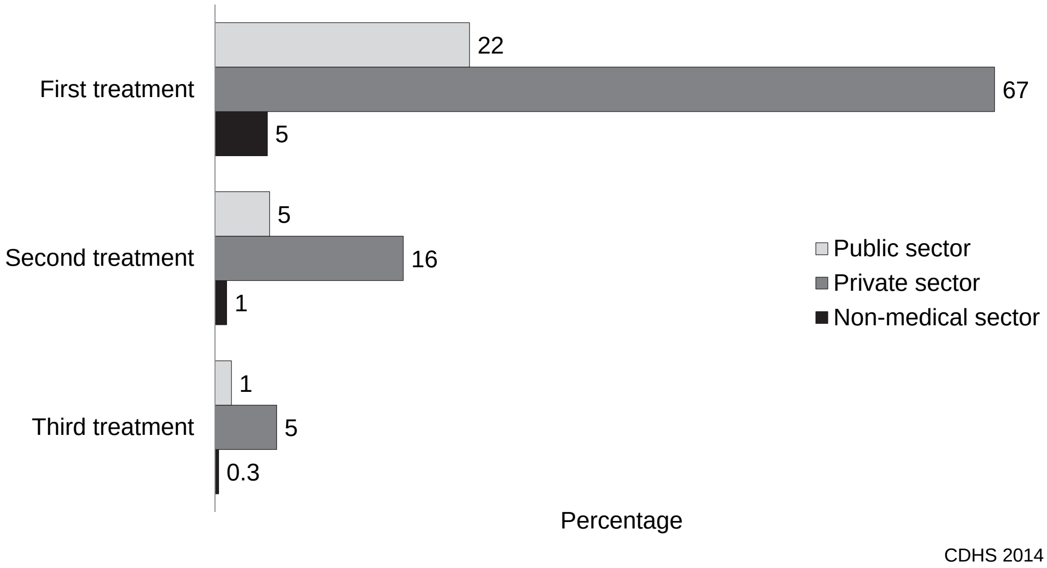

3.5 Cost for Health Care

3.5.1 Distribution of Cost for Health Care

For each ill or injured person, the respondent was asked to state the costs expended for transportation and treatment for each visit to a health care provider. These costs were reported only for living people who had been recently ill or injured and did not include costs incurred for people who had died in the 30 days preceding the interview. Costs are presented in US dollars in Table 3.6. In the case of all treatments, 9 percent of household members spent $1 or less for transportation and treatment for illness or injury, and 21 percent spent $1 to $4. Ten percent of all household members spent $50-$99 for transportation and treatment for illness or injury, and another 10 percent spent $100 or more.

These expenditures varied by type of spending. For transport, 48 percent of household members spent less than $1, 35 percent spent $1 to $4, 8 percent spent $5 to $9, and the rest spent $10 or more. For health care, 6 in 10 household members spent up to $19, 18 percent spent between $20 and $49, 10 percent spent between $50 and $99, and 9 percent spent $100 or more. There were small variations in spending according to order of treatment.

Table 3.6 Distribution of cost for health care

Percent distribution of de jure household members who were ill or injured in the past 30 days and sought treatment by amount of money spent for transport and health care, according to number of treatments, Cambodia 2014

Amount spent for transport and health care	Treatment for illness or injury: First treatment			Second treatment			Third treatment			All treatments		
	Transport	Health care	Total	Transport	Health care	Total	Transport	Health care	Total	Transport	Health care	Total
$0-1	50.4	17.3	10.6	46.0	16.4	9.7	51.6	19.2	11.7	48.4	14.9	9.3
$1-4	35.1	20.9	23.7	37.5	22.1	23.9	32.5	26.5	28.7	34.5	19.1	21.1
$5-9	7.5	13.9	15.1	8.4	14.8	15.4	7.6	15.1	16.0	8.1	13.1	13.9
$10-19	4.1	15.8	16.5	4.1	17.7	18.8	3.5	16.5	17.7	4.6	16.0	16.3
$20-49	1.2	15.9	16.6	2.3	15.8	17.7	1.5	13.5	14.8	2.3	17.5	18.5
$50-99	0.4	7.9	8.3	0.5	7.0	7.6	1.1	2.9	3.9	0.7	9.6	10.0
$100+	0.4	7.7	7.9	0.6	5.6	6.0	1.2	5.1	5.7	0.6	9.2	9.6
Don't know/ missing	0.8	0.6	1.2	0.6	0.4	0.9	1.0	1.1	1.5	0.8	0.7	1.4
Total	100.0	100.0	100.0	100.0	100.0	100.0	100.0	100.0	100.0	100.0	100.0	100.0
Number	9,186	9,186	9,186	2,143	2,143	2,143	684	684	684	9,186	9,186	9,186

3.5.2 Expenditures for Health Care

Table 3.7 presents the mean cost of transport and treatment by order of treatment and background characteristics. Mean total costs for first, second, and third treatments are $41.08, $34.27, and $32.19, respectively. Mean cost of transport increases with treatment order, from $2.78 for the first treatment to $3.59 for the second treatment and then $4.94 for the third treatment.

The mean cost of transport and health care varies according to type of health sector, severity of illness or injury, age group, sex, residence, and province. Examining total costs by type of health sector shows that the highest mean expenditure is for "outside of country/other" treatment, which may include going to Singapore, Thailand, or Vietnam or seeking specialized services. This is true for both costs of transport and costs of health care.

Total cost has continued to increase in the past four years, from a mean of $32.37 in 2010 to $39.36 in 2014. Increases have been observed in both the public and private sectors, in the first and third treatment cycles, and in transport as well as health care costs. Total "outside of country/other" costs have declined from the level reported in the 2010 CDHS, from $324.26 to $234.93. "Outside of country/other" treatment is the most expensive treatment option due to high transport ($33.86) and health care ($201.08) costs.

Table 3.7 Expenditures for health care

Mean expenditures in United States dollars for transport and health care by de jure household members who were ill or injured in the past 30 days and sought treatment by order of treatments, according to background characteristics, Cambodia 2014

Background characteristic	Treatment for illness or injury											
	First treatment			Second treatment			Third treatment			All treatments		
	Transport	Health care	Total	Transport	Health care	Total	Transport	Health care	Total	Transport	Health care	Total
**Type of health sector**												
Public	4.57	48.76	53.33	6.81	27.22	34.03	8.17	32.20	40.36	5.11	44.39	49.51
Private	1.72	33.00	34.72	2.03	30.16	32.19	2.61	21.27	23.88	1.83	31.78	33.61
Non-medical	0.66	8.54	9.20	2.75	18.71	21.45	0.43	5.56	5.98	1.01	10.12	11.12
Outside of country/ other	28.48	202.49	230.97	43.27	155.88	199.15	83.19	293.65	376.83	33.86	201.08	234.93
**Severity of illness or injury**												
Slight	1.22	11.82	13.04	1.23	8.78	10.00	2.51	17.62	20.12	1.28	11.61	12.89
Moderate	2.59	36.97	39.56	2.84	27.13	29.97	2.57	26.32	28.89	2.63	34.50	37.14
Serious	10.22	156.53	166.75	11.16	90.23	101.39	16.27	47.91	64.18	10.96	131.12	142.08
**Age**												
0-9	1.62	10.90	12.52	2.92	11.49	14.41	4.08	5.51	9.59	1.97	10.74	12.71
10-19	1.95	23.69	25.64	3.32	21.87	25.19	1.70	9.37	11.07	2.15	22.78	24.93
20-39	3.28	49.60	52.88	2.72	31.21	33.93	2.60	21.68	24.28	3.14	44.54	47.67
40-59	3.25	47.90	51.15	5.03	45.60	50.63	9.45	45.01	54.46	4.01	47.28	51.28
60+	4.04	68.52	72.55	3.89	44.77	48.66	2.10	41.84	43.94	3.90	62.73	66.62
**Sex**												
Male	2.74	38.20	40.94	3.83	36.14	39.97	3.77	20.93	24.69	2.99	36.89	39.88
Female	2.80	38.38	41.18	3.43	26.86	30.28	5.70	31.36	37.06	3.08	35.90	38.99
**Residence**												
Urban	3.39	48.49	51.88	2.41	22.69	25.09	4.74	32.82	37.56	3.30	41.76	45.05
Rural	2.64	36.00	38.64	3.95	33.12	37.08	5.02	24.93	29.95	2.98	34.95	37.94
**Province**												
Banteay Meanchey	3.63	53.97	57.59	5.14	48.72	53.86	11.01	46.96	57.97	4.15	52.93	57.08
Kampong Cham	2.71	34.11	36.82	5.70	44.42	50.12	13.98	54.87	68.85	3.71	36.67	40.38
Kampong Chhnang	2.21	32.62	34.83	2.28	38.86	41.14	3.00	44.70	47.70	2.26	34.34	36.60
Kampong Speu	2.28	36.44	38.72	2.39	19.64	22.03	1.23	13.07	14.31	2.27	34.79	37.06
Kampong Thom	1.61	26.16	27.77	7.21	94.18	101.39	3.29	23.88	27.17	2.13	32.19	34.32
Kandal	1.84	46.81	48.64	1.36	12.21	13.57	4.04	18.32	22.36	1.91	36.62	38.53
Kratie	2.88	27.83	30.71	4.08	51.43	55.51	3.87	38.15	42.02	3.00	30.02	33.02
Phnom Penh	2.65	33.59	36.24	1.23	13.21	14.44	2.22	18.24	20.46	2.25	26.82	29.06
Prey Veng	3.54	66.03	69.57	3.10	24.54	27.64	2.53	27.14	29.68	3.32	51.15	54.47
Pursat	3.64	71.72	75.36	5.84	63.55	69.40	na	na	na	3.90	70.75	74.65
Siem Reap	3.45	37.77	41.21	6.29	40.46	46.75	10.91	43.36	54.27	4.34	38.55	42.89
Svay Rieng	2.32	42.75	45.07	3.10	61.09	64.19	1.44	19.03	20.47	2.42	45.04	47.47
Takeo	3.97	45.15	49.12	3.16	27.19	30.35	3.09	17.77	20.86	3.73	39.24	42.98
Otdar Meanchey	9.70	36.80	46.50	31.14	51.97	83.11	178.65	178.75	357.40	14.74	40.58	55.33
Battambang/Pailin	2.97	37.06	40.03	8.49	51.72	60.21	5.74	33.05	38.79	3.82	38.99	42.82
Kampot/Kep	1.63	34.82	36.45	1.22	13.26	14.48	0.95	8.17	9.13	1.55	30.99	32.54
Preah Sihanouk/Koh Kong	2.84	29.35	32.19	4.70	33.89	38.59	4.58	46.14	50.72	3.17	30.55	33.72
Preah Vihear/Stung Treng	2.32	21.64	23.96	3.73	19.39	23.13	23.59	139.47	163.05	2.82	22.92	25.74
Mondul Kiri/Ratanak Kiri	7.10	49.35	56.45	18.40	166.02	184.42	1.59	8.02	9.60	8.59	65.33	73.92
Total	2.78	38.30	41.08	3.59	30.68	34.27	4.94	27.25	32.19	3.05	36.31	39.36

na = No third treatment was reported

In general, health care costs increased significantly by severity of illness or injury. The total mean cost of health care increased from $11.61 for slight illness or injury to $131.12 for serious conditions. This followed the same pattern established in the 2010 CDHS.

Overall, average health care costs rise consistently with the patient's age, from $10.74 for children age 0-9 to $62.73 for people age 60 or older. Health care expenditures by sex show that men and women spent about the same on health care ($36.89 and $35.90, respectively). A comparison with the findings of the 2010 CDHS shows that health care spending seems to have become more equitable. In 2010, men spent more than women on health care ($34.28 versus $26.90).

Total health care costs have remained higher in urban areas than in rural areas since the 2010 CDHS. However, the urban-rural difference in health care costs has narrowed considerably due to a decline in costs in urban areas. In urban areas average health care costs decreased from $74.79 in 2010 to

$41.76 in 2014, and in rural areas costs increased from $23.55 to $34.95 over the same period. The average transport cost per treatment has not changed much over the past four years (from $2.38 to $3.05). The difference in transport costs in urban and rural areas is small ($3.30 versus $2.98).

Health care expenditures vary greatly in Cambodia's provinces. The cost of health care is highest in Pursat ($70.75) and lowest in Preah Vihear/Stung Treng ($22.92).

3.5.3 Sources of Money for Health Care Expenditures

Because the health care system in Cambodia is largely fee-based, it is important to know the source of the money used to pay for health care. One goal of the health care system is to have appropriate funding mechanisms for the population to acquire health care without deepening poverty. Table 3.8 shows the different sources of money spent by people seeking treatment for health care. Percentages could sum to greater than 100 because a person could use money from more than one source.

Table 3.8 shows the different sources of money spent by persons who sought treatment for health care. The total percent could be greater than 100 because a person could use money from more than one source. Similar to 2010, the two major sources of money spent on health care are wages or income and savings; in 2014, 64 percent of people who sought health care used money from wages/income and 31 percent used savings. Gifts from relatives or friends and sale of assets were mentioned as a source of funding by 14 percent and 8 percent of those who obtained health care, respectively. Twelve percent of those who had health care treatment said they used money from tontine,[1] and 4 percent used money from a health equity fund. Each of the other sources of funding was mentioned by 1 percent or less of respondents.

There are small differences in the source of money spent on health care by type of health sector. In all sectors, the most common source of funding is wages or income (50 percent to 72 percent), followed by savings (22 percent to 33 percent). Gifts from relatives are the next most common source of funding for health care (13 percent to 17 percent).

As severity of illness or injury increases, dependence on loans, sale of assets, gifts, and savings increases; however, spending of wages or income declines as severity of illness or injury increases.

Wages/income was the most common source of funding regardless of the total cost of treatments; however, as treatment costs increase, the proportion of people who use funds from loans, sale of assets, gifts from relatives, and savings also increases. Health equity funds were used by 15 percent of those spending $0 to $1.

There were no substantial differences in the source of money used for health care costs by the patient's sex. Urban residents were more likely than rural residents to use wages (86 percent versus 59 percent) but less likely to use savings (13 percent versus 35 percent) for health care.

Large differences were found in the sources of money for health care costs by province. Patients in Phnom Penh, Preah Vihear/Stung Treng, and Kandal were most likely to use wages to pay for their health care (92 percent, 89 percent, and 87 percent, respectively) and among the least likely to use their savings (5 percent and 15 percent, respectively).

Conversely, Kampong Chhnang and Kampot/Kep are the provinces in which health care users are most likely to use savings for health care spending (86 percent and 76 percent, respectively). Patients in Prey Veng are least likely to use wages for health care spending (9 percent). Patients in Svay Rieng (34 percent) had the highest reliance on sale of assets for health care spending. Patients in Otdar Meanchey were most likely to use a health equity fund to finance their health care spending. Approximately 1 of 3 patients (32 percent) in Prey Veng reported gifts from relatives or friends as a source of funding for health care costs.

---

[1] Tontine is an informal group saving and loan scheme in Cambodia.

Table 3.8 Source of money (United States dollars) spent by persons who sought treatment for health care

Among de jure household members who were ill or injured in the 30 days before the survey and who sought treatment, percentage who reported specific sources of expenditures for transport and health care, according to background characteristics, Cambodia 2014

	Source of money for health care														
Background characteristic	Health equity fund	Voucher	Free exemp-tion	NGO	National Security Fund	Commu-nity based health insur-ance	Employ-er	Com-mercial health insur-ance	Wages/ income	Loan/ tontine	Sale of assets	Gift from relative	Savings	Other/ missing	Number[1]
**Type of health sector**															
Public	13.1	0.3	3.5	0.8	0.1	0.7	0.2	0.3	50.4	11.1	7.3	13.1	30.9	0.0	1,958
Private	1.4	0.1	0.2	0.2	0.0	0.1	0.3	0.0	67.5	12.9	7.7	14.3	31.5	0.1	6,594
Non-medical	2.4	0.3	0.5	0.6	0.0	0.0	0.0	0.0	72.1	11.3	7.2	16.6	33.4	0.3	472
Other	1.1	0.0	1.9	1.4	0.0	0.0	1.0	0.0	62.6	9.6	7.7	16.9	22.4	2.7	161
**Severity of illness or injury[2]**															
Slight	3.7	0.1	0.8	0.2	0.0	0.1	0.1	0.0	74.6	9.1	4.5	9.6	24.4	0.0	3,961
Moderate	4.1	0.2	1.3	0.4	0.0	0.3	0.4	0.2	57.7	13.1	8.4	16.1	36.3	0.2	4,277
Serious	4.6	0.3	0.4	0.3	0.1	0.1	0.7	0.0	47.5	23.0	16.9	25.6	38.2	0.2	941
**Cost of transport and health care**															
$0-1	15.2	0.1	5.0	1.6	0.2	0.2	0.9	0.0	55.9	2.4	1.5	7.8	25.0	0.7	851
$1-4	4.9	0.2	1.1	0.2	0.1	0.4	0.4	0.1	72.7	4.7	3.8	8.9	26.6	0.1	1,934
$5-9	3.4	0.0	0.9	0.5	0.0	0.2	0.0	0.1	68.7	8.6	6.4	9.7	32.1	0.0	1,274
$10-19	2.8	0.1	0.3	0.2	0.0	0.0	0.1	0.1	64.1	12.4	6.7	13.9	33.0	0.1	1,499
$20-49	1.6	0.1	0.4	0.1	0.0	0.2	0.0	0.1	60.9	15.8	9.2	19.1	34.4	0.0	1,702
$50-99	1.2	0.1	0.0	0.0	0.0	0.0	0.2	0.0	60.7	22.8	12.5	20.1	32.4	0.0	917
$100+	1.5	0.2	0.0	0.1	0.0	0.0	0.2	0.0	56.7	27.9	17.3	23.7	37.1	0.0	883
**Sex**															
Male	3.9	0.1	1.0	0.4	0.0	0.3	0.4	0.0	65.0	11.8	7.3	12.9	31.2	0.2	3,799
Female	4.0	0.2	0.9	0.3	0.0	0.1	0.2	0.1	63.3	12.8	7.8	15.2	31.4	0.0	5,387
**Residence**															
Urban	3.3	0.0	1.1	0.9	0.0	0.1	0.5	0.1	85.7	6.8	1.6	11.2	13.2	0.0	1,686
Rural	4.1	0.2	1.0	0.2	0.0	0.2	0.2	0.1	59.1	13.7	8.9	14.9	35.4	0.1	7,500
**Province**															
Banteay Meanchey	3.1	0.0	0.8	0.3	0.0	0.2	0.0	0.0	57.8	16.1	8.4	31.1	35.0	0.0	366
Kampong Cham	4.0	0.3	0.5	0.2	0.0	0.3	0.5	0.0	62.1	19.9	6.6	12.1	33.8	0.3	1,206
Kampong Chhnang	7.4	0.0	1.4	0.2	0.0	0.0	0.3	0.0	42.2	7.3	13.9	16.0	85.8	0.0	460
Kampong Speu	0.9	0.0	0.2	0.0	0.0	0.0	0.0	0.0	64.5	7.0	11.3	14.7	22.4	0.0	635
Kampong Thom	5.4	0.0	0.3	0.0	0.0	0.7	0.0	0.0	63.2	6.1	6.5	4.8	40.3	0.0	431
Kandal	0.7	0.2	0.4	0.2	0.0	0.0	0.3	0.0	86.6	6.2	1.7	18.6	14.7	0.0	687
Kratie	4.0	0.1	0.7	0.6	0.0	0.0	0.0	0.0	70.8	12.4	5.7	12.6	31.8	0.4	352
Phnom Penh	3.6	0.0	1.7	0.9	0.0	0.1	0.7	0.1	92.1	8.5	1.0	8.6	4.6	0.0	1,532
Prey Veng	2.1	0.0	0.4	0.0	0.0	0.0	0.0	0.0	8.7	20.5	12.2	32.0	64.4	0.0	384
Pursat	9.1	0.0	0.5	0.2	0.9	1.2	0.0	0.4	24.3	12.0	8.0	8.6	59.3	0.0	164
Siem Reap	5.4	0.8	1.1	0.0	0.0	1.4	0.0	0.0	52.6	22.5	5.9	5.3	32.8	0.0	424
Svay Rieng	0.4	0.0	0.5	0.0	0.0	0.0	0.2	0.0	64.1	19.1	34.3	23.9	34.5	0.2	440
Takeo	7.7	1.2	0.8	0.0	0.4	0.0	0.0	1.3	36.1	12.3	19.2	20.5	36.2	0.8	367
Otdar Meanchey	13.3	0.0	1.1	0.2	0.0	0.0	0.0	0.0	61.7	18.9	4.0	5.5	51.5	0.0	106
Battambang/Pailin	6.0	0.1	0.8	0.4	0.0	0.0	0.7	0.0	66.7	15.5	2.0	21.0	13.9	0.0	683
Kampot/Kep	1.9	0.0	2.7	0.5	0.0	0.0	0.0	0.0	36.3	7.6	10.2	8.7	75.6	0.0	406
Preah Sihanouk/ Koh Kong	8.5	0.3	0.6	1.2	0.0	0.0	0.3	0.2	56.2	5.6	1.8	10.3	41.1	0.2	184
Preah Vihear/ Stung Treng	3.6	0.0	3.9	0.0	0.0	0.0	0.0	0.0	89.2	11.3	2.2	4.2	9.9	0.0	251
Mondul Kiri/ Ratanak Kiri	2.7	0.4	0.2	1.7	0.0	0.0	0.0	0.0	74.3	2.8	1.7	3.1	21.8	0.6	109
Total	4.0	0.2	1.0	0.3	0.0	0.2	0.3	0.1	64.0	12.4	7.6	14.2	31.3	0.1	9,186

[1] Total includes 127 non-monetary cases (1 in-kind case and 126 cases of don't know or missing amount of spending)
[2] Includes 7 cases for which information on severity of illness is missing

DISABILITY 4

Key Findings

- Overall, 10 percent of household members age 5 and older suffer with at least one form of disability.
- Twenty-one percent of household members who were ill or injured in the 30 days prior to the interview are disabled.
- The most common types of disabilities reported in the survey are difficulties in seeing, walking or climbing stairs, and concentrating.
- One in 10 men who are not currently employed are disabled, as compared with only 5 percent among other men.

Persons with disabilities are considered vulnerable in Cambodia. The commitment of the Royal Government of Cambodia (RGC) to improving the lives of people with disabilities through recognition of their rights was demonstrated through ratification of the Convention on the Rights of Persons with Disabilities (CRPD) in 2012. The RGC has also enacted a number of disability laws and strategic plans in recent years. The government has developed a National Disability Policy to promote effective service delivery to persons with disabilities, and recently the Disability Rights Initiative Cambodia (DRIC) was jointly developed by the Australian government, the United Nations Development Program (UNDP), the World Health Organization (WHO), and the United Nations Children's Fund (UNICEF). The main objective of this latter initiative is to improve the quality of life of persons with disabilities in Cambodia.

People with disabilities are disadvantaged in workplaces and in other public places. Understanding the prevalence of disabilities in the population and the associated circumstances can improve efforts to remove disabling barriers and provide services that allow people with disabilities to integrate better into society. In the 2014 CDHS, information was collected on each household member age 5 and older about whether he or she had difficulties with seeing, hearing, walking or climbing stairs, remembering or concentrating, performing self-care, or communicating. The survey also collected information as to the severity of these disabilities, that is, whether a disabled person has some difficulty performing the listed activities, a great deal of difficulty, or cannot perform the listed activities at all.

4.1 Disability among the General Household Population

Table 4.1 presents the prevalence of disability in Cambodia according to type of disability and level of difficulty. The first column shows the proportion of the population with no disabilities. The next group of columns shows the proportion of the population with some level of difficulty performing various types of functions, while the final set of columns shows those with a great degree of difficulty or no ability to perform the described functions at all.

According to the survey, 10 percent of persons age 5 and over have some form of disability. Difficulties in seeing, walking or climbing stairs, and concentrating are the most common types of disabilities reported. Five percent of household members have difficulty seeing, 3 percent have difficulty hearing, 4 percent have difficulty walking or climbing stairs, and 4 percent have difficulties with remembering or concentrating. Only 1 percent of the population has at least some difficulty with self-care and 2 percent with communicating.

The prevalence of disability increases with age, from 2 percent among children age 5-14 to 44 percent among those age 60 and above. The prevalence of disability is 13 percent among persons age 35-59.

Table 4.1 Disability among the household population

Percentage of the de jure household population age 5 and over with specific types of physical disabilities, according to background characteristics, Cambodia 2014

		Some difficulty, a lot of difficulty, or cannot do								A lot of difficulty or cannot do						
Background characteristic	No difficulties	Any domain	Seeing	Hearing	Walking	Concentrating	Self-care	Communicating	Any domain	Seeing	Hearing	Walking	Concentrating	Self-care	Communicating	Number
**Age**																
5-14	98.2	1.8	0.3	0.5	0.3	0.7	0.6	0.5	0.5	0.1	0.1	0.1	0.2	0.2	0.3	16,446
15-34	96.5	3.5	1.0	1.0	0.8	1.6	0.3	0.8	0.9	0.1	0.3	0.2	0.4	0.2	0.5	24,987
35-59	86.8	13.2	6.6	2.7	4.4	5.2	0.7	1.2	2.0	0.4	0.4	0.9	0.5	0.3	0.6	17,640
60+	55.7	44.2	30.5	17.0	22.3	21.5	6.9	7.9	11.8	5.3	3.2	5.5	3.6	3.0	2.2	6,079
**Sex**																
Male	91.5	8.5	4.2	2.5	3.1	3.5	1.0	1.3	1.9	0.5	0.6	0.8	0.6	0.4	0.6	31,395
Female	89.5	10.5	5.9	3.1	4.2	4.9	1.2	1.7	2.3	0.8	0.6	0.9	0.8	0.5	0.6	33,757
**Marital status[1]**																
Never married	94.9	5.1	1.5	1.5	1.3	2.3	0.9	1.9	2.2	0.3	0.6	0.6	1.0	0.4	1.3	11,787
Married	88.4	11.6	6.4	3.1	4.4	4.9	0.8	1.1	1.9	0.6	0.5	0.8	0.4	0.3	0.3	31,883
Widowed	63.1	36.7	25.0	14.7	19.2	18.4	6.2	7.1	10.5	4.6	2.8	5.3	3.6	2.8	2.2	3,913
Divorced	86.9	13.1	6.2	2.8	3.6	6.3	1.6	2.7	2.9	0.8	0.8	0.7	1.5	0.9	1.4	1,099
**Education**																
No education	79.4	20.5	12.0	7.7	9.1	10.4	3.8	5.1	6.4	2.3	2.1	2.4	2.5	1.8	2.4	10,587
Primary	91.3	8.7	4.5	2.4	3.2	3.5	0.7	1.0	1.6	0.5	0.4	0.7	0.4	0.3	0.3	33,787
Secondary	94.5	5.5	2.6	1.1	1.9	2.2	0.4	0.5	0.9	0.2	0.1	0.4	0.3	0.1	0.2	18,393
Higher	97.2	2.8	1.3	0.4	0.4	1.7	0.0	0.2	0.4	0.1	0.1	0.1	0.1	0.0	0.1	2,378
**Household size[2]**																
1-4	89.1	10.9	6.2	3.2	4.3	4.9	1.2	1.7	2.3	0.8	0.6	0.9	0.8	0.4	0.6	27,500
5+	91.5	8.5	4.3	2.6	3.2	3.7	1.1	1.4	2.0	0.6	0.6	0.9	0.6	0.5	0.6	37,588
**Region**																
Banteay Meanchey	89.8	10.1	6.4	2.0	4.1	2.8	1.2	1.0	1.5	0.4	0.4	0.8	0.3	0.2	0.4	2,763
Kampong Cham	88.2	11.7	6.6	4.0	4.5	4.4	1.1	1.8	2.6	1.0	0.8	0.8	0.9	0.7	0.8	8,368
Kampong Chhnang	94.7	5.3	3.6	1.8	1.9	1.7	1.1	1.1	1.6	0.6	0.4	0.6	0.5	0.5	0.3	2,311
Kampong Speu	94.9	5.1	1.9	2.2	1.5	1.2	0.7	1.4	1.9	0.4	0.7	0.7	0.7	0.5	0.6	4,187
Kampong Thom	92.5	7.5	4.5	2.7	2.9	2.3	1.1	1.0	2.1	0.6	0.8	1.1	0.5	0.6	0.6	3,268
Kandal	88.9	11.1	6.3	4.0	2.6	5.3	1.4	2.1	2.7	0.9	0.9	1.0	0.7	0.6	0.8	5,144
Kratie	92.7	7.2	3.1	2.1	1.7	3.4	0.8	1.9	1.7	0.4	0.4	0.5	0.6	0.3	0.9	1,889
Phnom Penh	91.0	9.0	4.8	2.4	2.3	4.5	0.7	1.1	2.5	0.9	0.4	0.9	0.7	0.2	0.7	6,206
Prey Veng	90.8	9.2	5.2	3.0	3.2	3.9	1.4	1.8	2.0	0.6	0.5	1.0	0.5	0.5	0.7	4,351
Pursat	91.8	8.1	4.6	2.2	4.7	3.5	1.4	1.7	2.5	0.6	0.5	1.2	0.6	0.7	0.7	2,526
Siem Reap	89.6	10.3	5.0	3.6	2.8	5.3	0.8	1.5	1.3	0.3	0.4	0.4	0.5	0.3	0.4	4,275
Svay Rieng	90.4	9.6	5.4	2.9	2.6	3.9	0.4	1.0	1.5	0.3	0.4	0.5	0.7	0.2	0.5	2,440
Takeo	94.0	6.0	2.8	1.9	2.9	3.3	1.6	1.9	1.5	0.6	0.4	0.8	0.7	0.5	0.6	4,067
Otdar Meanchey	93.3	6.6	4.3	1.8	3.2	2.0	1.0	1.0	2.3	0.9	0.7	1.1	1.0	0.5	0.5	1,063
Battambang/Pailin	80.3	19.7	9.7	2.8	12.4	11.0	1.1	1.2	3.4	1.1	0.6	1.7	1.4	0.4	0.6	5,010
Kampot/Kep	93.8	6.2	2.7	2.4	2.9	2.5	2.6	2.6	2.5	0.9	0.8	1.1	0.9	0.9	0.7	2,900
Preah Sihanouk/ Koh Kong	95.6	4.4	1.9	1.3	1.7	1.7	0.9	1.1	0.7	0.2	0.1	0.3	0.2	0.3	0.1	1,452
Preah Vihear/ Stung Treng	85.0	15.0	8.8	5.1	4.0	7.4	0.8	1.2	1.9	0.5	0.5	0.6	0.6	0.2	0.5	1,579
Mondul Kiri/ Ratanak Kiri	97.8	2.1	1.0	0.9	0.6	0.5	0.2	0.4	0.8	0.3	0.2	0.3	0.2	0.1	0.2	1,356
**Residence**																
Urban	91.3	8.7	4.8	2.3	3.0	4.0	0.9	1.1	2.2	0.7	0.4	0.9	0.6	0.3	0.6	10,403
Rural	90.3	9.7	5.1	2.9	3.8	4.3	1.1	1.6	2.1	0.7	0.6	0.8	0.7	0.5	0.6	54,750
**Ill or injured in the past 30 days**																
Yes	79.3	20.7	11.8	6.3	10.3	10.0	3.1	3.5	5.8	2.2	1.4	2.9	1.6	1.5	1.4	7,852
No	92.0	8.0	4.2	2.3	2.8	3.4	0.8	1.2	1.6	0.5	0.5	0.6	0.6	0.3	0.5	57,301
Total	90.5	9.5	5.1	2.8	3.7	4.2	1.1	1.5	2.1	0.7	0.6	0.9	0.7	0.5	0.6	65,153

Note: Total includes 2 cases for which information on illness or injury in the past 30 days is missing, 21 cases for which information on marital status is missing, and 8 cases for which information on education is missing.
[1] Marital status was asked only for household members age 15 or older.
[2] Households with only de facto member(s) are excluded.

Females are slightly more likely to suffer from some level of disability than their male counterparts (11 percent versus 9 percent). The prevalence of disability is much higher among household members who are widowed (37 percent) than those who are divorced (13 percent), currently in a union (12 percent), or single (5 percent). There is a notable association between disability and education. Household members who have no education (21 percent) are more than twice as likely to suffer from some level of disability as those with a primary education (9 percent) and seven times as likely as those with more than a secondary education (3 percent). There is little difference according to urban or rural residence. However, the level of disability varies substantially by province, from 2 percent in Mondul Kiri/Ratanak Kiri to 20 percent in Battambang/Pailin. Household members who recently suffered an illness or injury (in the 30 days prior to the interview) are more likely than those who did not (21 percent versus 8 percent) to report a disability.

Only 2 percent of the household population suffers from a severe disability (a great degree of difficulty or lack of ability to perform the function at all). This indicates that the majority of disabled people experience a moderate level of disability. Overall, less than 1 percent of the population age 5 and older is severely suffering from each form of disability. The distribution of more severe disabilities by background characteristics follows a pattern similar to that observed among overall disability.

4.2 DISABILITY AMONG ILL OR INJURED HOUSEHOLD MEMBERS

Table 4.2 presents information about disability among household members who were ill or injured in the 30 days prior to the survey. It is worth noting that respondents were not asked the order in which these two morbidities occurred. Therefore, the relationship between disability and illness or injury as discussed here is purely an association and does not indicate cause and effect.

Table 4.2 Disability among the ill or injured population

Among the de jure household population age 5 and over who were ill or injured in the 30 days before the survey, percentage with specific types of physical disabilities, according to background characteristics, Cambodia 2014

Background characteristic	No diffi-culties	Some difficulty, a lot of difficulty, or cannot do							A lot of difficulty or cannot do							Number
		Any domain	Seeing	Hearing	Walking	Concen-trating	Self-care	Com-muni-cating	Any domain	Seeing	Hearing	Walking	Concen-trating	Self-care	Com-muni-cating	
**Sought advice or health facility contact**																
Did not seek treatment	64.4	33.2	20.2	8.9	18.3	18.7	3.9	7.4	7.0	3.5	3.7	3.8	3.0	2.5	4.0	418
Public sector	77.5	21.3	12.3	6.6	11.4	11.0	4.7	5.1	7.0	2.4	2.0	3.3	1.4	2.3	1.9	1,590
Private pharmacy	80.4	17.6	9.7	4.5	6.8	9.2	2.8	2.8	5.5	2.1	1.1	2.4	2.0	1.2	1.2	1,384
Other private facilities	78.1	20.0	11.3	6.7	10.3	9.3	2.7	3.0	5.7	2.2	1.1	2.9	1.6	1.2	1.2	3,903
Other/missing	75.7	21.6	13.6	4.9	9.2	8.2	1.0	1.3	3.2	1.1	0.5	2.0	0.5	0.5	0.4	558
**Transport cost**																
Free/no cost	77.2	20.6	12.5	6.0	9.9	9.7	2.9	2.9	5.8	2.9	1.1	2.9	1.3	1.5	0.9	2,393
Paid money	78.6	19.7	10.9	6.2	9.8	9.6	3.1	3.5	5.7	1.8	1.3	2.8	1.7	1.3	1.5	4,980
Other/don't know/ missing	66.9	30.7	17.9	8.9	16.8	16.7	4.1	6.9	6.8	3.1	3.2	4.0	2.9	2.6	3.9	480
**Treatment cost**																
Free/no cost	74.6	23.7	13.1	7.0	12.7	12.4	4.7	5.0	5.3	1.9	1.9	2.1	1.2	2.1	1.3	542
Paid money	78.5	19.6	11.2	6.1	9.6	9.3	2.9	3.2	5.8	2.2	1.2	2.8	1.6	1.4	1.3	6,842
Other/don't know/ missing	65.1	32.4	19.3	8.3	17.7	17.4	4.1	7.0	7.1	3.1	3.3	4.0	2.7	2.4	4.0	468
**Health care financing mechanism**																
Health equity fund	72.2	25.2	13.0	6.3	16.8	11.1	3.3	3.3	6.2	1.7	1.9	2.8	1.0	1.3	1.0	315
Other subsidy	74.4	20.9	15.6	2.8	5.4	5.7	1.3	2.5	0.0	0.0	0.0	0.0	0.0	0.0	0.0	102
Insurance	(77.2)	22.8	20.8	10.4	10.4	9.0	0.0	0.0	0.0	0.0	0.0	0.0	0.0	0.0	0.0	41
Out of pocket	77.8	20.4	11.7	6.3	10.0	10.0	3.1	3.6	5.9	2.3	1.4	2.9	1.7	1.5	1.5	7,381
Total	77.5	20.7	11.8	6.3	10.3	10.0	3.1	3.5	5.8	2.2	1.4	2.9	1.6	1.5	1.4	7,852

Note: Total includes 14 cases for which information on health care financing mechanism is missing. Figures in parentheses are based on 25-49 unweighted cases.

The prevalence of disability among ill or injured household members is about two times higher than that among the general household population (21 percent versus 10 percent). Difficulties in seeing (12 percent), walking or climbing stairs (10 percent), and remembering or concentrating (10 percent) are the most common types of disabilities reported among the ill and injured population.

According to source of treatment, level of disability is higher among those who did not seek any treatment for their illness or injury (33 percent) than among those who sought treatment in a public health facility (21 percent), a private facility (20 percent), or a pharmacy (18 percent). The percentage of people with a disability is slightly higher among those who received free treatment for their illness or injury than among those who paid for treatment (24 percent versus 20 percent). However, the difference in prevalence by cost of transport is minimal. The prevalence of disability among ill or injured people by type of health care financing shows that the percentage with a disability is slightly higher among those for whom the cost of treatment for their illness or injury was paid by a health equity fund (25 percent) than among those who received other forms of subsidies (21 percent), those who have insurance (23 percent), and those who paid out of their pocket for the treatment of their illness or injury (20 percent).

Six percent of ill and injured household members suffer from more severe disabilities (i.e., they have a great deal of difficulty or cannot perform the function at all). Similar to the general population, this finding indicates that the majority of ill or injured people experience a moderate level of disability. The distribution of more severe disability among the ill or injured population by background characteristics follows somewhat the same pattern observed for overall disability.

4.3 DISABILITY AND EMPLOYMENT

Table 4.3 presents information about disability by type of employment. Since information on employment was collected only among interviewed women and men age 15-49, this table provides data on disability and employment among only household members age 15-49 who were eligible for an individual interview and completed the interview.

Table 4.3 Disability and employment

Percentage of interviewed women and men age 15-49 with a physical disability according to employment status, Cambodia 2014

Employment status	No diffi-culties	Some difficulty, a lot of difficulty, or cannot do: Any domain	Seeing	Hearing	Walking	Concen trating	Self-care	Com-muni-cating	A lot of difficulty or cannot do: Any domain	Seeing	Hearing	Walking	Concen trating	Self-care	Com-muni-cating	Number
WOMEN																
**Employed in the 12 months preceding the survey**																
Currently employed[1]	95.1	4.9	2.3	0.9	1.0	1.8	0.0	0.3	0.3	0.1	0.1	0.1	0.0	0.0	0.1	12,436
Not currently employed	94.4	5.6	2.7	1.0	1.9	2.2	0.0	0.2	0.6	0.1	0.0	0.2	0.3	0.0	0.1	1,542
**Not employed in the 12 months preceding the survey**	93.8	6.2	2.4	0.8	2.0	2.7	0.4	0.8	1.1	0.3	0.1	0.7	0.2	0.1	0.3	3,599
Total	94.7	5.3	2.3	0.9	1.3	2.0	0.1	0.4	0.5	0.2	0.1	0.2	0.1	0.0	0.1	17,578
MEN																
**Employed in the 12 months preceding the survey**																
Currently employed[1]	95.2	4.7	1.8	0.9	1.2	1.6	0.3	0.2	0.4	0.2	0.0	0.2	0.1	0.1	0.0	4,547
Not currently employed	89.8	10.2	2.5	4.4	2.3	3.8	0.9	0.2	0.0	0.0	0.0	0.0	0.0	0.0	0.0	271
**Not employed in the 12 months preceding the survey**	94.8	5.2	1.4	0.2	3.0	1.7	0.3	1.1	2.0	1.0	0.2	0.7	0.3	0.0	0.3	372
Total	94.9	5.0	1.8	1.0	1.4	1.7	0.3	0.3	0.5	0.2	0.0	0.2	0.1	0.1	0.0	5,190

Note: Total includes 1 woman for whom information on employment is missing.
[1] "Currently employed" is defined as having done work in the past 7 days. Includes persons who did not work in the past 7 days but who are regularly employed and were absent from work for leave, illness, vacation, or any other such reason.

According to the 2014 CDHS, only 5 percent of interviewed women and men age 15-49 suffer from at least one form of disability. Difficulties in seeing and concentrating (2 percent each) are the most common types of disabilities reported among these women and men.

The prevalence of disability among women who are currently employed is 5 percent, slightly lower than among those who are not currently employed and those who were not employed in the 12 months preceding the survey (6 percent each). Men who are not currently employed are twice as likely to be disabled as men who are currently employed and those not employed in the 12 months preceding the survey (10 percent versus 5 percent each). Severe disability is only reported for less than 1 percent among this group.

RESPONDENT CHARACTERISTICS 5

Key Findings

- Thirteen percent of women and 6 percent of men age 15-49 have no education; an additional 40 percent and 52 percent have at least some secondary education.
- Twenty-one percent of women and 25 percent of men age 15-49 are exposed to at least one source of mass media once a week.
- Only 16 percent of Cambodian women and 13 percent of men are covered by health insurance.
- Sixty-nine percent of women were employed in the 12 months preceding the survey, with the majority (57 percent of women) employed in the agricultural sector.
- Nearly half of working women (44 percent) work in the agricultural sector, and about three in four of these women are self-employed.

This chapter provides a demographic and socioeconomic profile of respondents interviewed in the 2014 Cambodia Demographic and Health Survey (CDHS). Such background information is essential to interpret the findings and understand the results presented later in the report. Basic characteristics of respondents include age, level of education, marital status, religion, and wealth status. Exposure to mass media and literacy status were examined, and detailed information was collected on employment status, occupation, and earnings. In addition, the CDHS collected data on knowledge and attitudes concerning health insurance coverage and use of tobacco.

5.1 Characteristics of Survey Respondents

Background characteristics of the 17,578 women age 15-49 and the 5,190 men age 15-49 interviewed in the 2014 CDHS are shown in Table 5.1. This table is important because it provides background for interpreting findings presented later in the report.

The distribution of the population of women and men by age reflects recent Cambodian history. It is notable that 16-18 percent of women and men fall into each of the age groups between 15-19 and 30-34. Smaller proportions are found in the older age groups. Between 11 and 12 percent of women and men fall into each of the five-year age groups between 35 and 49. This age distribution of respondents is unusual and reflects the effects of the Khmer Rouge regime (1975-1979), during which fertility rates declined and were coupled with higher than normal mortality. Between one and two million people are estimated to have been killed during the reign of the Khmer Rouge. These events are reflected in the smaller than expected proportions of women and men in the age groups between 35 and 49.

Approximately 68 percent of women and 66 percent of men are married or living with their partner. The proportion not currently married varies by gender, with 25 percent of women never married compared with 32 percent of men. Women are more than three times as likely as men to be divorced, separated, or widowed (7 percent and 2 percent, respectively).

Access to services and exposure to information pertaining to reproductive health and other aspects of life are often determined by one's area of residence. The majority of respondents reside in rural areas, with only 19 percent of women and 17 percent of men residing in urban areas. About 12 percent of women and 13 percent of men live in Kampong Cham, and 11 percent of each live in the capital city of Phnom Penh. Cambodians are predominantly Buddhist (96 percent of women and 95 percent of men). The other two main religions, Islam and Christianity, are practiced by a very small proportion of respondents (Table 5.1).

Table 5.1 Background characteristics of respondents						
Percent distribution of women and men age 15-49 by selected background characteristics, Cambodia 2014						
	Women			Men		
Background characteristic	Weighted percent	Weighted number	Unweighted number	Weighted percent	Weighted number	Unweighted number
**Age**						
15-19	16.5	2,893	3,006	17.8	926	946
20-24	17.2	3,017	3,038	16.1	835	881
25-29	16.1	2,836	2,866	15.7	815	796
30-34	17.3	3,046	2,996	17.5	907	888
35-39	10.5	1,839	1,776	10.7	556	528
40-44	11.6	2,030	1,995	11.5	595	603
45-49	10.9	1,916	1,901	10.7	556	548
**Religion**						
Buddhist	96.0	16,882	16,699	95.4	4,949	4,888
Moslem	1.9	335	338	2.6	133	124
Christian	0.9	157	151	0.9	47	48
Other/missing	1.2	204	390	1.2	61	130
**Marital status**						
Never married	25.2	4,428	4,651	32.0	1,663	1,746
Married	67.2	11,808	11,574	65.3	3,388	3,306
Living together	0.5	91	94	0.3	17	14
Divorced/separated	3.8	664	697	1.8	95	97
Widowed	3.3	588	562	0.5	26	27
**Residence**						
Urban	18.5	3,251	5,667	16.7	869	1,540
Rural	81.5	14,327	11,911	83.3	4,321	3,650
**Province**						
Banteay Meanchey	3.9	689	810	3.7	192	223
Kampong Cham	11.5	2,021	853	12.8	663	300
Kampong Chhnang	3.8	662	899	3.5	182	251
Kampong Speu	6.8	1,196	1,022	6.2	323	269
Kampong Thom	4.8	851	905	4.5	232	261
Kandal	7.6	1,330	875	8.0	413	239
Kratie	2.8	488	874	2.8	143	258
Phnom Penh	11.3	1,994	1,400	10.6	550	391
Prey Veng	6.8	1,188	819	6.6	342	244
Pursat	3.6	631	859	3.5	184	261
Siem Reap	6.5	1,137	943	6.5	337	282
Svay Rieng	3.7	654	822	3.5	183	237
Takeo	6.2	1,082	868	6.4	334	252
Otdar Meanchey	1.7	294	823	1.9	99	277
Battambang/Pailin	7.6	1,333	867	7.8	405	249
Kampot/Kep	4.4	770	880	4.6	241	284
Preah Sihanouk/Koh Kong	2.4	422	1,010	2.3	120	288
Preah Vihear/Stung Treng	2.6	462	1,085	2.2	112	274
Mondul Kiri/Ratanak Kiri	2.1	372	964	2.6	134	350
**Education**						
No education	12.8	2,250	2,233	6.2	324	327
Primary	47.1	8,281	7,826	41.8	2,167	2,026
Secondary and higher	40.1	7,047	7,519	52.0	2,699	2,837
**Wealth quintile**						
Lowest	17.9	3,143	3,050	17.4	901	885
Second	18.9	3,314	3,057	18.4	954	930
Middle	19.2	3,381	2,798	20.0	1,040	867
Fourth	20.6	3,612	3,450	21.7	1,124	1,037
Highest	23.5	4,128	5,223	22.6	1,171	1,471
Total	100.0	17,578	17,578	100.0	5,190	5,190

Note: Education categories refer to the highest level of education attended, whether or not that level was completed.

The majority of Cambodians have some formal schooling, and educational levels of women have improved within the past 10 years. The percentage of women with no schooling declined from 28 percent in the 2000 CDHS to 19 percent in the 2005 CDHS, declined further to 16 percent in the 2010 CDHS, and finished at 13 percent in the 2014 CDHS. Moreover, the percentage of women who had at least some secondary education increased from 25 percent in 2005, to 35 percent in 2010, and reached 40 percent in 2014. However, Table 5.1 shows there are still notable differences in educational attainment between women and men. Twice as many women as men have no schooling (13 percent versus 6 percent), and men are more likely than women to have secondary education or higher (52 percent versus 40 percent).

5.2 EDUCATIONAL ATTAINMENT AND LITERACY

Tables 5.2.1 and 5.2.2 present a detailed distribution of educational attainment among Cambodian women and men, according to background characteristics. The general pattern evident in Table 5.2.1 indicates a decrease in the proportion of women with no schooling from the oldest to the youngest cohorts. Men, with the exception of those in the 40-44 age group, exhibit the same pattern (Table 5.2.2). The data presented in Tables 5.2.1 and 5.2.2 provide evidence of an increase in educational attainment among the youngest age cohort. For example, 68 percent of women age 15-19 have attended secondary school, as compared with only 58 percent of women age 20-24. A similar trend is seen in young men, with 66 percent of those age 15-19 and 62 percent of those age 20-24 having attended some secondary school.

Table 5.2.1 Educational attainment: Women

Percent distribution of women age 15-49 by highest level of schooling attended or completed, and median years completed, according to background characteristics, Cambodia 2014

Background characteristic	Highest level of schooling: No education	Some primary	Completed primary[1]	Some secondary	Completed secondary[2]	More than secondary	Total	Median years completed	Number of women
**Age**									
15-24	4.1	22.6	10.5	50.2	5.4	7.2	100.0	6.9	5,910
15-19	2.8	19.7	9.7	61.8	3.1	2.9	100.0	7.2	2,893
20-24	5.3	25.3	11.3	39.2	7.6	11.3	100.0	6.6	3,017
25-29	11.2	36.2	11.0	29.6	4.5	7.6	100.0	5.2	2,836
30-34	17.8	46.7	6.6	22.2	3.3	3.4	100.0	3.8	3,046
35-39	17.4	53.8	5.8	19.8	2.3	0.9	100.0	3.2	1,839
40-44	17.2	51.0	5.3	22.4	2.3	1.8	100.0	3.3	2,030
45-49	24.9	55.7	2.9	14.0	1.7	0.7	100.0	2.3	1,916
**Residence**									
Urban	5.4	23.5	5.7	39.8	8.9	16.6	100.0	7.5	3,251
Rural	14.5	42.6	8.5	29.8	2.6	1.9	100.0	4.3	14,327
**Province**									
Banteay Meanchey	12.8	46.3	7.1	27.7	4.0	2.1	100.0	4.2	689
Kampong Cham	14.1	43.1	9.9	28.4	2.7	1.8	100.0	4.0	2,021
Kampong Chhnang	8.7	41.1	8.7	33.6	4.3	3.5	100.0	5.0	662
Kampong Speu	11.0	39.2	11.2	35.4	1.9	1.3	100.0	5.0	1,196
Kampong Thom	14.0	46.9	8.8	23.6	3.7	3.0	100.0	3.7	851
Kandal	5.4	42.0	8.5	38.5	2.1	3.6	100.0	5.2	1,330
Kratie	15.6	50.1	7.7	22.1	2.6	1.9	100.0	3.3	488
Phnom Penh	4.1	23.7	5.3	39.6	7.8	19.5	100.0	7.6	1,994
Prey Veng	19.1	42.7	7.6	27.3	2.2	1.0	100.0	3.9	1,188
Pursat	16.3	41.0	10.5	26.5	4.0	1.7	100.0	4.2	631
Siem Reap	25.4	38.4	6.8	22.0	3.9	3.4	100.0	3.3	1,137
Svay Rieng	6.0	50.7	8.1	30.6	2.1	2.5	100.0	4.2	654
Takeo	11.7	32.6	6.1	40.8	5.3	3.5	100.0	5.6	1,082
Otdar Meanchey	26.4	37.2	7.8	24.9	2.8	0.9	100.0	3.3	294
Battambang/Pailin	10.0	35.1	7.9	36.6	5.1	5.3	100.0	5.6	1,333
Kampot/Kep	8.5	39.9	7.4	37.3	3.9	3.0	100.0	5.2	770
Preah Sihanouk/Koh Kong	9.6	38.8	9.6	32.3	4.0	5.6	100.0	5.2	422
Preah Vihear/Stung Treng	23.7	45.1	7.1	19.7	2.2	2.1	100.0	2.8	462
Mondul Kiri/Ratanak Kiri	34.5	32.9	6.0	23.8	1.4	1.3	100.0	2.3	372
**Wealth quintile**									
Lowest	27.9	50.4	7.0	14.0	0.6	0.1	100.0	2.2	3,143
Second	18.1	48.5	8.9	22.9	1.1	0.4	100.0	3.3	3,314
Middle	10.3	46.0	8.0	33.0	1.9	0.8	100.0	4.5	3,381
Fourth	7.1	34.1	9.3	40.2	5.2	4.0	100.0	5.8	3,612
Highest	4.1	21.7	6.9	43.7	8.6	15.1	100.0	7.6	4,128
Total	12.8	39.1	8.0	31.7	3.8	4.6	100.0	4.8	17,578

[1] Completed 6th grade at the primary level
[2] Completed 12th grade at the secondary level

Urban women have higher levels of education than rural women. Almost two-thirds of urban women have attended at least some secondary school, as compared with only about one-third of rural women. Tables 5.2.1 and 5.2.2 show great variation in education across provinces. Mondul Kiri/Ratanak Kiri has an exceptionally low level of educational attainment among women (35 percent of women having no formal education) whereas Siem Reap has the lowest level among men (21 percent of men having no formal education). By contrast, only 4 percent of women and less than 1 percent of men in Phnom Penh have no schooling. Median number of years of education completed is highest in Phnom Penh (7.6 for women and 9.9 for men).

Educational attainment rises dramatically with wealth quintile. Twenty-eight percent of women in the lowest quintile have no formal education, as compared with 4 percent of women in the highest wealth quintile. The percentage of women who have attended some secondary school increases from 15 percent in the lowest wealth quintile to 67 percent in the highest. The pattern of variation in educational attainment by wealth among men is similar to that among women.

Table 5.2.2 Educational attainment: Men

Percent distribution of men age 15-49 by highest level of schooling attended or completed, and median years completed, according to background characteristics, Cambodia 2014

Background characteristic	Highest level of schooling: No education	Some primary	Completed primary[1]	Some secondary	Completed secondary[2]	More than secondary	Total	Median years completed	Number of men
**Age**									
15-24	3.3	24.2	8.2	50.9	5.2	8.2	100.0	7.0	1,760
15-19	2.2	23.6	7.9	59.3	2.8	4.2	100.0	7.0	926
20-24	4.5	25.0	8.6	41.5	7.8	12.6	100.0	7.0	835
25-29	3.9	30.4	9.4	36.6	6.4	13.4	100.0	6.5	815
30-34	8.4	38.4	8.4	30.0	7.0	7.8	100.0	5.3	907
35-39	10.3	45.0	6.9	28.0	5.8	4.0	100.0	4.6	556
40-44	8.1	42.6	6.7	29.3	8.3	5.0	100.0	4.9	595
45-49	9.6	39.5	8.2	34.6	4.9	3.3	100.0	5.1	556
**Residence**									
Urban	1.0	14.9	5.1	42.2	10.0	26.8	100.0	9.0	869
Rural	7.3	37.4	8.7	37.5	5.3	3.7	100.0	5.5	4,321
**Province**									
Banteay Meanchey	7.5	40.0	5.5	38.7	4.2	4.2	100.0	5.3	192
Kampong Cham	5.2	45.5	10.0	30.1	2.5	6.7	100.0	4.9	663
Kampong Chhnang	4.6	41.3	8.9	31.4	7.3	6.5	100.0	5.5	182
Kampong Speu	6.7	24.9	12.4	48.6	5.5	1.9	100.0	6.3	323
Kampong Thom	8.7	42.1	10.6	30.5	3.2	4.9	100.0	4.9	232
Kandal	2.1	39.7	6.8	40.0	6.5	5.0	100.0	5.5	413
Kratie	3.6	45.1	5.2	33.2	8.3	4.5	100.0	5.2	143
Phnom Penh	0.3	11.2	3.7	43.4	10.2	31.2	100.0	9.9	550
Prey Veng	9.2	25.4	9.0	51.6	2.8	2.1	100.0	6.5	342
Pursat	8.0	41.3	12.6	30.6	5.8	1.8	100.0	5.1	184
Siem Reap	20.8	35.5	9.0	20.3	9.8	4.6	100.0	4.2	337
Svay Rieng	1.1	31.6	7.2	48.0	7.8	4.4	100.0	6.7	183
Takeo	4.1	29.9	7.9	45.8	6.9	5.4	100.0	6.9	334
Otdar Meanchey	13.0	38.4	8.7	33.1	6.1	0.8	100.0	4.8	99
Battambang/Pailin	2.3	29.9	9.0	46.3	5.7	6.8	100.0	6.7	405
Kampot/Kep	6.1	33.9	8.0	40.6	8.7	2.7	100.0	6.1	241
Preah Sihanouk/Koh Kong	4.7	29.0	8.1	39.5	8.2	10.5	100.0	6.7	120
Preah Vihear/Stung Treng	11.7	53.1	0.8	26.6	1.5	6.4	100.0	3.7	112
Mondul Kiri/Ratanak Kiri	16.4	36.9	6.3	30.8	3.9	5.7	100.0	4.6	134
**Wealth quintile**									
Lowest	15.7	53.4	8.2	20.5	1.5	0.7	100.0	3.3	901
Second	8.9	47.7	10.8	29.6	1.7	1.2	100.0	4.5	954
Middle	5.7	36.9	9.6	41.2	4.5	2.1	100.0	5.6	1,040
Fourth	2.9	26.3	8.3	49.0	8.2	5.4	100.0	6.9	1,124
Highest	0.6	11.1	4.3	46.3	12.5	25.2	100.0	9.2	1,171
Total	6.2	33.6	8.1	38.3	6.1	7.6	100.0	6.0	5,190

[1] Completed 6th grade at the primary level
[2] Completed 12th grade at the secondary level

The 2014 CDHS assessed literacy levels among respondents who had never been to school or who had attended only primary school by asking them to read all or part of a sentence in whatever language they chose. Those with at least some secondary education were assumed to be literate. Literacy results are shown in Tables 5.3.1 and 5.3.2.

Table 5.3.1 shows that 76 percent of women are literate, and Table 5.3.2 shows that 84 percent of men are literate. For women, those in the younger age groups are more likely to be literate than those in the older age groups. Literacy increases from 62 percent among women age 45-49 to 90 percent among women age 15-19. For men the negative relationship between literacy and age is less evident. The percentage of men who are literate is highest at age group 15-19 (89 percent). It decreases gradually to 76 percent among those age 35-39; then it reverses its pattern and is 83 percent among men age 40-49.

Table 5.3.1 Literacy: Women

Percent distribution of women age 15-49 by level of schooling attended and level of literacy, and percentage literate, according to background characteristics, Cambodia 2014

		No schooling or primary school								
Background characteristic	Secondary school or higher	Can read a whole sentence	Can read part of a sentence	Cannot read at all	No card with required language	Blind/ visually impaired	Missing	Total	Percentage literate[1]	Number of women
**Age**										
15-24	62.8	12.6	12.8	11.7	0.0	0.0	0.0	100.0	88.2	5,910
15-19	67.7	11.8	10.2	10.1	0.0	0.1	0.1	100.0	89.8	2,893
20-24	58.1	13.4	15.2	13.3	0.0	0.0	0.0	100.0	86.7	3,017
25-29	41.6	17.1	20.9	20.2	0.0	0.0	0.1	100.0	79.6	2,836
30-34	28.9	18.7	20.9	31.5	0.0	0.0	0.1	100.0	68.5	3,046
35-39	23.0	16.2	26.2	34.6	0.0	0.0	0.0	100.0	65.4	1,839
40-44	26.5	18.7	24.9	29.8	0.0	0.0	0.1	100.0	70.1	2,030
45-49	16.4	16.6	29.1	37.9	0.0	0.0	0.0	100.0	62.1	1,916
**Residence**										
Urban	65.4	14.8	10.3	9.5	0.0	0.0	0.1	100.0	90.5	3,251
Rural	34.3	16.2	22.3	27.1	0.0	0.0	0.1	100.0	72.8	14,327
**Province**										
Banteay Meanchey	33.8	12.1	26.6	27.5	0.0	0.0	0.0	100.0	72.5	689
Kampong Cham	32.9	12.0	29.9	25.2	0.0	0.0	0.0	100.0	74.8	2,021
Kampong Chhnang	41.5	14.0	24.9	19.5	0.0	0.0	0.0	100.0	80.5	662
Kampong Speu	38.5	15.0	18.6	27.8	0.0	0.0	0.0	100.0	72.2	1,196
Kampong Thom	30.3	18.7	30.1	20.9	0.0	0.0	0.0	100.0	79.1	851
Kandal	44.2	27.2	8.1	20.3	0.0	0.0	0.2	100.0	79.5	1,330
Kratie	26.6	28.4	15.0	29.8	0.0	0.0	0.3	100.0	69.9	488
Phnom Penh	66.9	17.2	7.2	8.6	0.0	0.0	0.1	100.0	91.3	1,994
Prey Veng	30.6	15.0	22.3	31.7	0.0	0.1	0.3	100.0	67.9	1,188
Pursat	32.2	4.7	35.4	27.7	0.0	0.0	0.0	100.0	72.3	631
Siem Reap	29.3	16.4	21.4	33.0	0.0	0.0	0.0	100.0	67.0	1,137
Svay Rieng	35.2	19.8	19.2	25.7	0.0	0.2	0.0	100.0	74.2	654
Takeo	49.5	12.3	18.1	20.0	0.0	0.0	0.0	100.0	80.0	1,082
Otdar Meanchey	28.6	2.3	27.7	41.2	0.2	0.0	0.0	100.0	58.6	294
Battambang/Pailin	47.0	22.2	14.9	15.9	0.0	0.0	0.0	100.0	84.1	1,333
Kampot/Kep	44.2	12.2	21.2	22.5	0.0	0.0	0.0	100.0	77.5	770
Preah Sihanouk/Koh Kong	42.0	14.6	27.3	16.0	0.0	0.0	0.1	100.0	83.9	422
Preah Vihear/Stung Treng	24.1	15.7	14.6	45.2	0.4	0.0	0.0	100.0	54.4	462
Mondul Kiri/Ratanak Kiri	26.5	2.1	24.5	46.9	0.0	0.0	0.0	100.0	53.1	372
**Wealth quintile**										
Lowest	14.7	12.5	27.4	45.3	0.0	0.1	0.0	100.0	54.6	3,143
Second	24.4	17.0	24.4	34.0	0.1	0.0	0.0	100.0	65.8	3,314
Middle	35.7	17.8	23.6	22.8	0.0	0.0	0.1	100.0	77.1	3,381
Fourth	49.5	16.6	17.8	16.0	0.0	0.0	0.1	100.0	83.9	3,612
Highest	67.4	15.5	10.0	7.0	0.0	0.0	0.0	100.0	92.9	4,128
Total	40.1	15.9	20.1	23.9	0.0	0.0	0.1	100.0	76.1	17,578

[1] Refers to women who attended secondary school or higher and women who can read a whole sentence or part of a sentence

Ninety-one percent of women residing in urban areas are literate, as compared with 73 percent of their rural counterparts. Similarly, urban men show higher rates of literacy than rural men (95 percent and 82 percent, respectively). Differences in literacy across provinces are marked, with the highest literacy rate among women in Phnom Penh (91 percent) and the lowest among women in Mondul Kiri/Ratanak Kiri (53 percent). Among men, literacy is also highest in Phnom Penh (96 percent) and lowest in Preah Vihear/Stung Treng (76 percent). Literacy levels increase along with wealth status among both women and men. For example, literacy levels increase from 55 percent among women in the lowest wealth quintile to 93 percent among women in the highest wealth quintile and from 67 percent among men in the lowest wealth quintile to 98 percent among men in the highest wealth quintile.

Women's overall literacy rate has continued to increase since the 2000 CDHS (67 percent in 2000 versus 69 percent in 2005, 74 percent in 2010, and 76 percent in 2014). The difference in the literacy rates among Cambodian men between 2010 (83 percent) and 2014 (84 percent) is very minimal.

Table 5.3.2 Literacy: Men

Percent distribution of men age 15-49 by level of schooling attended and level of literacy, and percentage literate, according to background characteristics, Cambodia 2014

Background characteristic	Secondary school or higher	No schooling or primary school: Can read a whole sentence	Can read part of a sentence	Cannot read at all	No card with required language	Blind/ visually impaired	Missing	Total	Percentage literate[1]	Number of men
**Age**										
15-24	64.3	6.1	17.8	11.8	0.1	0.0	0.0	100.0	88.1	1,760
15-19	66.4	6.1	16.4	11.0	0.2	0.0	0.0	100.0	88.8	926
20-24	61.9	6.1	19.3	12.7	0.0	0.0	0.0	100.0	87.3	835
25-29	56.4	11.3	18.9	13.0	0.0	0.4	0.0	100.0	86.6	815
30-34	44.8	13.3	22.5	19.3	0.1	0.0	0.0	100.0	80.6	907
35-39	37.7	16.4	22.3	23.6	0.0	0.0	0.0	100.0	76.4	556
40-44	42.7	13.8	26.2	17.2	0.0	0.0	0.1	100.0	82.6	595
45-49	42.8	15.6	24.7	16.9	0.0	0.0	0.0	100.0	83.1	556
**Residence**										
Urban	78.9	6.7	9.1	5.3	0.0	0.0	0.0	100.0	94.7	869
Rural	46.6	12.1	23.4	17.8	0.1	0.1	0.0	100.0	82.0	4,321
**Province**										
Banteay Meanchey	47.0	1.1	36.2	15.6	0.0	0.0	0.0	100.0	84.4	192
Kampong Cham	39.3	17.4	24.6	18.2	0.0	0.5	0.0	100.0	81.3	663
Kampong Chhnang	45.2	13.0	25.2	16.1	0.0	0.0	0.5	100.0	83.4	182
Kampong Speu	56.0	5.7	17.8	20.5	0.0	0.0	0.0	100.0	79.5	323
Kampong Thom	38.6	23.4	22.3	15.7	0.0	0.0	0.0	100.0	84.3	232
Kandal	51.5	12.3	14.7	21.5	0.0	0.0	0.0	100.0	78.5	413
Kratie	46.1	13.0	24.6	16.3	0.0	0.0	0.0	100.0	83.7	143
Phnom Penh	84.8	4.5	6.3	4.4	0.0	0.0	0.0	100.0	95.6	550
Prey Veng	56.5	7.8	20.5	15.2	0.0	0.0	0.0	100.0	84.8	342
Pursat	38.2	16.1	30.2	15.5	0.0	0.0	0.0	100.0	84.5	184
Siem Reap	34.7	9.8	36.1	19.0	0.5	0.0	0.0	100.0	80.5	337
Svay Rieng	60.1	11.9	14.6	13.3	0.0	0.0	0.0	100.0	86.7	183
Takeo	58.1	10.5	13.6	17.8	0.0	0.0	0.0	100.0	82.2	334
Otdar Meanchey	40.0	3.1	38.9	17.9	0.0	0.0	0.0	100.0	82.1	99
Battambang/Pailin	58.8	22.5	8.2	10.5	0.0	0.0	0.0	100.0	89.5	405
Kampot/Kep	52.0	0.6	29.2	18.3	0.0	0.0	0.0	100.0	81.7	241
Preah Sihanouk/Koh Kong	58.2	20.6	12.5	8.7	0.0	0.0	0.0	100.0	91.3	120
Preah Vihear/Stung Treng	34.5	5.1	35.9	24.5	0.0	0.0	0.0	100.0	75.5	112
Mondul Kiri/Ratanak Kiri	40.4	0.0	39.8	19.4	0.4	0.0	0.0	100.0	80.2	134
**Wealth quintile**										
Lowest	22.8	11.6	33.0	32.6	0.1	0.0	0.0	100.0	67.4	901
Second	32.6	14.0	31.2	22.0	0.2	0.0	0.0	100.0	77.8	954
Middle	47.7	14.2	21.1	17.0	0.0	0.0	0.1	100.0	83.0	1,040
Fourth	62.5	10.8	17.0	9.5	0.0	0.3	0.0	100.0	90.2	1,124
Highest	84.0	6.3	7.1	2.5	0.0	0.0	0.0	100.0	97.5	1,171
Total	52.0	11.2	21.0	15.7	0.0	0.1	0.0	100.0	84.1	5,190

[1] Refers to men who attended secondary school or higher and men who can read a whole sentence or part of a sentence

5.3 Access to Mass Media

The 2014 CDHS collected information on the exposure of respondents to both broadcast and print media. This information is important because it provides an indication of the exposure of women to mass media that can be used to disseminate family planning, health, and other information. Access to mass media is relatively high in Cambodia. Table 5.4.1 shows that 69 percent of women have some weekly exposure to mass media. Watching television is the most common way of accessing the media: 61 percent of women watch television at least once a week. Listening to the radio is also common (32 percent of women listen at least once a week), with newspapers being the least utilized form of media (8 percent read a newspaper at least once a week).

There is no strong pattern in access to the three types of media by age. The youngest group of women (age 15-19) is most likely to access each form of media. However, women in the oldest age group are not always the least likely to access media. Women age 35-49 are least likely to read a newspaper at least once a week (5 percent), and women age 35-39 are least likely to listen to the radio (25 percent).

Residence, by contrast, is associated with differences in media exposure. Urban women have better access to newspaper, television, and radio sources than their rural counterparts. The percentages of urban women who read newspapers, watch television, and listen to the radio at least once per week are 22

percent, 86 percent, and 43 percent, respectively. In contrast, rural women are significantly less likely than urban women to do so (5 percent read newspapers, 55 percent watch television, and 30 percent listen to the radio).

Media exposure among women varies by province as well. Women residing in Phnom Penh have the greatest exposure to all three media (17 percent). Women residing in Kratie are least likely to be exposed to the media, with 65 percent having no weekly access to media.

Table 5.4.1 Exposure to mass media: Women

Percentage of women age 15-49 who are exposed to specific media on a weekly basis, by background characteristics, Cambodia 2014

Background characteristic	Reads a newspaper at least once a week	Watches television at least once a week	Listens to the radio at least once a week	Accesses all three media at least once a week	Accesses none of the three media at least once a week	Number of women
**Age**						
15-19	11.4	63.6	41.5	7.0	26.3	2,893
20-24	10.2	63.4	35.4	6.2	28.2	3,017
25-29	8.5	61.3	30.5	4.9	30.7	2,836
30-34	7.1	60.4	26.8	4.5	33.3	3,046
35-39	4.7	56.7	25.2	2.8	37.0	1,839
40-44	5.4	57.6	30.3	3.4	34.8	2,030
45-49	4.5	56.5	34.3	2.9	33.2	1,916
**Residence**						
Urban	21.7	85.5	42.5	13.5	10.7	3,251
Rural	4.7	54.8	30.1	2.8	36.1	14,327
**Province**						
Banteay Meanchey	4.6	74.9	24.9	3.1	19.8	689
Kampong Cham	3.8	53.2	26.2	2.2	36.8	2,021
Kampong Chhnang	6.9	41.4	34.3	4.5	48.5	662
Kampong Speu	3.6	59.7	34.8	1.9	31.3	1,196
Kampong Thom	5.5	58.4	36.3	3.9	33.2	851
Kandal	5.6	87.1	45.4	4.3	10.3	1,330
Kratie	2.9	18.8	24.8	1.4	64.5	488
Phnom Penh	26.9	89.5	49.1	16.9	6.8	1,994
Prey Veng	3.4	68.3	34.4	2.0	24.8	1,188
Pursat	3.5	43.0	22.5	2.0	49.0	631
Siem Reap	6.8	40.5	29.6	3.9	46.4	1,137
Svay Rieng	3.6	64.2	21.7	2.4	30.7	654
Takeo	5.8	65.4	24.4	4.5	32.3	1,082
Otdar Meanchey	5.7	36.4	18.8	2.1	53.3	294
Battambang/Pailin	11.8	76.2	41.3	6.9	15.3	1,333
Kampot/Kep	4.5	27.4	17.7	0.1	58.8	770
Preah Sihanouk/Koh Kong	6.7	71.7	16.3	2.9	24.4	422
Preah Vihear/Stung Treng	2.1	21.8	22.5	0.6	59.8	462
Mondul Kiri/Ratanak Kiri	10.8	29.4	32.2	8.2	53.5	372
**Education**						
No education	0.1	33.4	18.2	0.0	58.2	2,250
Primary	3.0	55.9	27.8	1.7	35.2	8,281
Secondary and higher	16.0	74.5	42.2	10.0	18.3	7,047
**Wealth quintile**						
Lowest	1.3	22.8	19.5	0.5	65.0	3,143
Second	1.8	42.4	25.7	0.8	45.6	3,314
Middle	3.9	62.2	33.1	1.9	28.5	3,381
Fourth	7.8	76.4	36.6	5.0	18.3	3,612
Highest	21.1	88.3	43.0	13.4	8.3	4,128
Total	7.9	60.5	32.3	4.8	31.4	17,578

Media exposure increases with both the educational level and wealth quintile of the respondent. For example, 88 percent of women in the highest wealth quintile watch television at least once per week, as compared with 23 percent of women in the lowest wealth quintile. Similarly, 75 percent of women with secondary education compared with 33 percent of women with no schooling watch television once a week. In addition, 16 percent of women with at least some secondary school read a newspaper at least once a week, as compared with 3 percent of women who have attended only primary school.

Table 5.4.2 Exposure to mass media: Men

Percentage of men age 15-49 who are exposed to specific media on a weekly basis, by background characteristics, Cambodia 2014

Background characteristic	Reads a newspaper at least once a week	Watches television at least once a week	Listens to the radio at least once a week	Accesses all three media at least once a week	Accesses none of the three media at least once a week	Number of men
**Age**						
15-19	12.3	65.2	44.7	7.1	23.4	926
20-24	15.5	62.1	46.3	7.8	23.7	835
25-29	16.7	65.9	44.7	11.8	23.3	815
30-34	16.3	62.1	41.4	7.7	26.2	907
35-39	14.7	58.2	43.1	7.1	26.3	556
40-44	14.4	60.9	42.5	9.5	27.0	595
45-49	16.7	56.2	55.3	8.3	22.8	556
**Residence**						
Urban	30.6	74.8	49.8	14.7	11.3	869
Rural	12.1	59.5	44.1	7.2	27.3	4,321
**Province**						
Banteay Meanchey	3.4	70.0	49.0	3.3	19.7	192
Kampong Cham	11.0	58.0	46.8	5.3	26.0	663
Kampong Chhnang	7.4	38.5	24.9	1.9	46.5	182
Kampong Speu	10.2	60.3	49.3	5.1	22.3	323
Kampong Thom	2.9	71.5	48.1	2.3	22.0	232
Kandal	8.4	70.5	33.8	4.4	22.4	413
Kratie	7.6	27.9	30.7	4.2	58.8	143
Phnom Penh	26.7	69.1	44.4	9.6	12.9	550
Prey Veng	2.6	53.8	25.9	0.5	34.1	342
Pursat	5.4	76.4	68.1	5.1	18.1	184
Siem Reap	24.5	31.9	27.2	7.9	46.2	337
Svay Rieng	2.6	50.8	29.3	0.7	36.2	183
Takeo	9.6	84.9	62.9	7.2	5.6	334
Otdar Meanchey	21.0	59.4	52.2	16.0	26.7	99
Battambang/Pailin	38.2	86.7	75.3	32.3	6.1	405
Kampot/Kep	19.9	56.5	42.8	8.6	26.5	241
Preah Sihanouk/Koh Kong	49.0	92.2	61.9	34.9	2.8	120
Preah Vihear/Stung Treng	2.6	35.6	27.7	0.9	45.2	112
Mondul Kiri/Ratanak Kiri	29.2	41.3	42.6	16.1	38.0	134
**Education**						
No education	0.6	35.3	20.6	0.1	54.4	324
Primary	7.8	54.3	39.6	3.5	32.0	2,167
Secondary and higher	22.8	71.5	52.5	13.4	15.1	2,699
**Wealth quintile**						
Lowest	6.1	31.9	28.3	1.3	51.3	901
Second	7.3	47.0	39.1	2.9	34.9	954
Middle	9.3	65.7	49.9	5.9	20.6	1,040
Fourth	16.3	78.8	51.6	10.9	12.9	1,124
Highest	32.8	78.4	52.3	18.5	10.5	1,171
Total	15.2	62.1	45.1	8.5	24.6	5,190

A comparison of Tables 5.4.1 and 5.4.2 shows that women and men have relatively the same access to all three media at least once per week (5 percent of women versus 9 percent of men). The slight difference between the levels of exposure can be explained by greater access of men to printed material: 15 percent of men read a newspaper at least once per week, as compared with 8 percent of women.

In general, rates of media utilization remain more or less similar to those in 2010, when two-thirds of women (68 percent) were exposed to some source of mass media. The differences between media exposure in the 2010 CDHS and the 2014 CDHS are found among women who read a newspaper at least once a week (12 percent versus 8 percent, respectively) and women who watch television at least once per week (58 percent versus 61 percent, respectively). There was some improvement in men's exposure to mass media between 2010 and 2014 due to an increase in the percentage of men who watch television. In 2010, 30 percent of men were not exposed to a mass media source on a weekly basis, whereas this proportion decreased to 25 percent in 2014.

5.4 EMPLOYMENT

5.4.1 Employment Status

The 2014 CDHS included a number of questions regarding respondents' employment status, including whether they worked in the seven days preceding the survey and, if not, whether they worked in the 12 months before the survey. Employment status results for women and men are presented in Tables 5.5.1 and 5.5.2.

Table 5.5.1 Employment status: Women

Percent distribution of women age 15-49 by employment status, according to background characteristics, Cambodia 2014

Background characteristic	Employed in the 12 months preceding the survey: Currently employed[1]	Employed in the 12 months preceding the survey: Not currently employed	Not employed in the 12 months preceding the survey	Total	Number of women
**Age**					
15-19	55.2	7.1	37.7	100.0	2,893
20-24	66.8	10.3	22.9	100.0	3,017
25-29	72.1	8.5	19.4	100.0	2,836
30-34	75.4	8.2	16.5	100.0	3,046
35-39	77.6	9.2	13.1	100.0	1,839
40-44	79.0	9.1	11.9	100.0	2,030
45-49	75.7	9.5	14.8	100.0	1,916
**Marital status**					
Never married	66.0	5.8	28.2	100.0	4,428
Married or living together	71.3	10.1	18.6	100.0	11,898
Divorced/separated/ widowed	82.5	7.1	10.5	100.0	1,252
**Number of living children**					
0	67.1	7.2	25.7	100.0	5,698
1-2	71.1	9.4	19.5	100.0	6,622
3-4	74.9	9.0	16.1	100.0	3,893
5+	72.6	11.5	15.9	100.0	1,365
**Residence**					
Urban	73.1	4.3	22.6	100.0	3,251
Rural	70.2	9.8	20.0	100.0	14,327
**Province**					
Banteay Meanchey	65.5	19.6	14.9	100.0	689
Kampong Cham	71.2	10.4	18.4	100.0	2,021
Kampong Chhnang	88.2	3.7	8.1	100.0	662
Kampong Speu	84.0	4.4	11.6	100.0	1,196
Kampong Thom	79.8	1.2	19.0	100.0	851
Kandal	75.2	5.1	19.7	100.0	1,330
Kratie	74.5	14.3	11.2	100.0	488
Phnom Penh	76.2	3.3	20.5	100.0	1,994
Prey Veng	78.6	5.2	16.2	100.0	1,188
Pursat	56.8	15.9	27.4	100.0	631
Siem Reap	69.4	7.3	23.3	100.0	1,137
Svay Rieng	84.7	5.2	10.1	100.0	654
Takeo	54.8	15.6	29.5	100.0	1,082
Otdar Meanchey	77.8	2.4	19.8	100.0	294
Battambang/Pailin	46.0	8.8	45.1	100.0	1,333
Kampot/Kep	52.5	35.1	12.4	100.0	770
Preah Sihanouk/Koh Kong	69.0	4.3	26.7	100.0	422
Preah Vihear/Stung Treng	70.3	7.1	22.6	100.0	462
Mondul Kiri/Ratanak Kiri	81.5	3.3	15.2	100.0	372
**Education**					
No education	70.4	9.3	20.4	100.0	2,250
Primary	73.0	10.1	16.9	100.0	8,281
Secondary and higher	68.2	7.1	24.7	100.0	7,047
**Wealth quintile**					
Lowest	70.4	12.4	17.2	100.0	3,143
Second	70.9	11.1	18.0	100.0	3,314
Middle	68.5	9.2	22.3	100.0	3,381
Fourth	69.2	9.0	21.7	100.0	3,612
Highest	74.0	3.6	22.4	100.0	4,128
Total	70.7	8.8	20.5	100.0	17,578

[1] *Currently employed* is defined as having done work in the past seven days. Includes persons who did not work in the past seven days but who are regularly employed and were absent from work for leave, illness, vacation, or any other such reason.

Table 5.5.2 Employment status: Men

Percent distribution of men age 15-49 by employment status, according to background characteristics, Cambodia 2014

Background characteristic	Employed in the 12 months preceding the survey: Currently employed[1]	Employed in the 12 months preceding the survey: Not currently employed	Not employed in the 12 months preceding the survey	Total	Number of men
**Age**					
15-19	60.9	8.4	30.7	100.0	926
20-24	87.3	5.1	7.6	100.0	835
25-29	93.8	4.3	1.9	100.0	815
30-34	95.8	4.0	0.2	100.0	907
35-39	94.5	5.5	0.0	100.0	556
40-44	95.7	3.7	0.6	100.0	595
45-49	94.5	4.9	0.6	100.0	556
**Marital status**					
Never married	70.9	7.8	21.3	100.0	1,663
Married or living together	95.6	4.0	0.4	100.0	3,405
Divorced/separated/ widowed	92.9	4.0	3.1	100.0	122
**Number of living children**					
0	75.0	7.5	17.5	100.0	2,043
1-2	96.2	3.3	0.5	100.0	1,725
3-4	95.1	4.5	0.4	100.0	1,058
5+	96.0	3.7	0.3	100.0	364
**Residence**					
Urban	84.1	2.2	13.8	100.0	869
Rural	88.3	5.8	5.8	100.0	4,321
**Province**					
Banteay Meanchey	89.6	8.8	1.6	100.0	192
Kampong Cham	84.0	6.8	9.3	100.0	663
Kampong Chhnang	90.5	1.6	7.8	100.0	182
Kampong Speu	90.3	1.3	8.4	100.0	323
Kampong Thom	98.4	0.5	1.1	100.0	232
Kandal	90.7	0.6	8.8	100.0	413
Kratie	89.7	2.9	7.4	100.0	143
Phnom Penh	81.2	2.9	15.9	100.0	550
Prey Veng	91.2	8.5	0.3	100.0	342
Pursat	86.7	4.7	8.7	100.0	184
Siem Reap	91.6	2.1	6.3	100.0	337
Svay Rieng	88.3	5.0	6.7	100.0	183
Takeo	69.7	26.5	3.8	100.0	334
Otdar Meanchey	91.2	0.0	8.8	100.0	99
Battambang/Pailin	88.6	7.7	3.7	100.0	405
Kampot/Kep	89.0	0.0	11.0	100.0	241
Preah Sihanouk/Koh Kong	88.7	2.8	8.5	100.0	120
Preah Vihear/Stung Treng	95.8	1.4	2.8	100.0	112
Mondul Kiri/Ratanak Kiri	97.6	0.0	2.4	100.0	134
**Education**					
No education	96.1	2.0	2.0	100.0	324
Primary	92.5	5.3	2.2	100.0	2,167
Secondary and higher	82.7	5.5	11.8	100.0	2,699
**Wealth quintile**					
Lowest	90.8	4.5	4.7	100.0	901
Second	91.2	5.1	3.6	100.0	954
Middle	85.6	8.7	5.8	100.0	1,040
Fourth	86.7	5.2	8.1	100.0	1,124
Highest	84.9	2.8	12.3	100.0	1,171
Total	87.6	5.2	7.2	100.0	5,190

[1] *Currently employed* is defined as having done work in the past seven days. Includes persons who did not work in the past seven days but who are regularly employed and were absent from work for leave, illness, vacation, or any other such reason.

At the time of the survey, 71 percent of women were currently employed, and an additional 9 percent were not employed but had worked sometime during the preceding 12 months. The proportion of women currently employed generally increases with increasing age and peaks at age group 40-44 (79 percent) before decreasing to 76 percent at age 45-49. Women who are divorced, separated, or widowed are more likely to be employed than other women. Among men, in contrast, those who are married are more likely to be employed than those who are divorced, separated, or widowed and those who have never married.

Urban and rural women are roughly equally likely to be currently employed (73 percent versus 70 percent). However, rural women are almost two and a half times more likely than urban women to have worked in the past 12 months but not currently (10 percent versus 4 percent). As a result, urban women are slightly more likely than rural women not to have been employed at all in the 12 months preceding the survey (23 percent versus 20 percent). Women in Kampong Chhnang are most likely to be currently employed (88 percent). In contrast, women in Battambang/Pailin are most likely not to have been employed at any time in the 12 months preceding the survey (45 percent). Women who have attended secondary school or higher and those in the three highest wealth quintiles are most likely to have not worked in the 12 months preceding the survey.

The proportion of men currently employed is higher than that of women (88 percent versus 71 percent). Employment status differentials for men are similar to those for women. The proportion of men currently employed generally increases with age and peaks at age 30-34 (from 61 percent to 96 percent). From age 35 to 49, the percentage of men currently employed is relatively constant at 95-96 percent. As with women, urban men are more likely not to have worked in the 12 months preceding the survey, as are men with a secondary education or higher and those in the highest wealth quintile. The proportion of men currently employed ranges from a low of 70 percent in Takeo to a high of 98 percent in Kampong Thom and in Mondul Kiri/Ratanak Kiri. Phnom Penh has the highest percentage of men who are not currently employed (16 percent), and Takeo has the highest percentage of men who worked at some point in the previous 12 months (27 percent), but not currently.

The level of female employment in 2014 is similar to that in 2010. However, there is a difference between the two surveys in the proportions of men employed. In 2010, 81 percent of men in Cambodia were currently employed, whereas this proportion increased to 88 percent of men in 2014.

5.4.2 Occupation

Respondents who were currently employed or had worked in the 12 months preceding the survey were further asked to specify their occupation. Tables 5.6.1 and 5.6.2 show data on occupation of employed women and men, respectively.

Most employed persons are engaged in the agricultural sector, including 44 percent of working women and 51 percent of working men. About one in four working women are employed in sales and services (24 percent), along with 13 percent of men. Twenty-two percent of women are employed in skilled manual labor, and 2 percent are employed in unskilled manual labor. Men are more likely than women to be employed in skilled manual labor, with 26 percent engaged in this type of occupation. Six percent of women and 7 percent of men are employed in professional, technical, and managerial fields.

Table 5.6.1 Occupation: Women

Percent distribution of women age 15-49 employed in the 12 months preceding the survey by occupation, according to background characteristics, Cambodia 2014

Background characteristic	Professional/ technical/ managerial	Clerical	Sales and services	Skilled manual	Unskilled manual	Agriculture	Missing	Total	Number of women
**Age**									
15-19	2.5	1.4	19.8	33.0	1.8	36.4	5.0	100.0	1,802
20-24	8.4	2.6	22.8	28.6	1.7	34.0	1.9	100.0	2,327
25-29	8.0	2.1	24.4	25.9	1.6	36.8	1.2	100.0	2,286
30-34	5.4	1.1	28.6	22.2	1.6	40.5	0.6	100.0	2,545
35-39	3.4	0.3	26.8	18.8	1.9	48.4	0.4	100.0	1,597
40-44	4.9	0.7	25.3	11.1	1.7	55.5	1.0	100.0	1,788
45-49	4.8	0.5	21.7	6.8	2.1	63.2	1.0	100.0	1,632
**Marital status**									
Never married	8.8	3.0	22.1	29.4	2.2	29.6	4.9	100.0	3,177
Married or living together	4.6	0.8	24.9	19.0	1.3	48.8	0.5	100.0	9,679
Divorced/separated/ widowed	5.1	1.2	26.5	22.7	3.5	39.8	1.3	100.0	1,121
**Number of living children**									
0	8.2	2.7	22.3	29.5	2.2	31.2	3.8	100.0	4,231
1-2	5.8	1.0	26.6	23.1	1.4	41.2	0.7	100.0	5,332
3-4	3.4	0.5	26.0	13.9	1.5	54.2	0.5	100.0	3,267
5+	1.0	0.1	16.7	8.0	1.9	72.1	0.3	100.0	1,148
**Residence**									
Urban	15.6	4.6	47.5	19.7	5.3	5.2	2.2	100.0	2,514
Rural	3.4	0.6	19.3	22.1	0.9	52.2	1.4	100.0	11,464
**Province**									
Banteay Meanchey	4.1	1.7	31.3	18.8	2.7	40.5	0.9	100.0	586
Kampong Cham	4.0	0.1	22.0	16.3	0.6	54.3	2.6	100.0	1,649
Kampong Chhnang	3.8	0.3	14.7	26.2	0.4	54.1	0.4	100.0	608
Kampong Speu	2.6	0.2	10.3	45.5	0.6	40.8	0.0	100.0	1,057
Kampong Thom	4.0	0.2	20.7	9.9	1.3	63.5	0.3	100.0	690
Kandal	5.9	0.7	20.9	54.5	1.1	16.9	0.0	100.0	1,068
Kratie	3.6	0.3	18.4	3.5	1.0	72.9	0.2	100.0	434
Phnom Penh	16.8	5.1	39.0	27.8	6.0	3.4	1.8	100.0	1,584
Prey Veng	2.9	0.1	24.0	13.1	0.5	54.7	4.8	100.0	996
Pursat	3.4	0.2	22.5	12.9	1.2	58.8	0.9	100.0	458
Siem Reap	5.5	0.7	29.5	9.0	2.5	51.6	1.1	100.0	872
Svay Rieng	3.3	2.3	16.8	25.8	1.5	47.2	3.2	100.0	589
Takeo	4.5	0.4	24.7	35.2	0.6	29.6	4.9	100.0	763
Otdar Meanchey	1.5	1.2	17.4	1.9	0.5	77.0	0.5	100.0	236
Battambang/Pailin	7.9	4.9	39.2	7.9	1.5	37.2	1.5	100.0	731
Kampot/Kep	4.0	0.6	18.3	7.0	1.2	68.8	0.2	100.0	675
Preah Sihanouk/Koh Kong	5.6	2.4	39.8	30.0	4.5	16.8	0.9	100.0	309
Preah Vihear/Stung Treng	3.2	1.0	14.8	1.9	0.3	78.4	0.3	100.0	358
Mondul Kiri/Ratanak Kiri	2.2	0.6	26.2	1.3	1.3	68.3	0.0	100.0	315
**Education**									
No education	0.5	0.2	16.1	10.7	1.6	70.4	0.5	100.0	1,792
Primary	1.3	0.4	20.9	22.8	1.9	52.4	0.4	100.0	6,882
Secondary and higher	12.8	2.9	31.7	23.9	1.6	23.5	3.5	100.0	5,303
**Wealth quintile**									
Lowest	0.6	0.2	5.8	12.7	0.7	79.0	1.1	100.0	2,602
Second	1.1	0.1	11.8	20.5	1.0	64.6	0.9	100.0	2,718
Middle	2.1	0.3	17.4	28.0	0.7	49.3	2.3	100.0	2,628
Fourth	6.5	0.8	31.5	28.3	2.1	29.5	1.3	100.0	2,827
Highest	15.5	4.6	49.6	18.9	3.7	5.5	2.2	100.0	3,202
Total	5.6	1.3	24.4	21.7	1.7	43.7	1.6	100.0	13,978

Residence has an effect on type of occupation. Employed women and men in urban areas are more likely than those in rural areas to hold jobs in the professional, technical, and managerial; clerical; and sales and services sectors. In contrast, rural women and men are more likely than those in urban areas to be engaged in agricultural work. Those with lower levels of education and those in lower wealth quintiles are also more likely to work in agriculture. For example, 78 percent of employed men with no schooling work in the field of agriculture, whereas only 36 percent of men with a secondary education or higher work in agriculture.

Table 5.6.2 Occupation: Men

Percent distribution of men age 15-49 employed in the 12 months preceding the survey by occupation, according to background characteristics, Cambodia 2014

Background characteristic	Professional/ technical/ managerial	Clerical	Sales and services	Skilled manual	Unskilled manual	Agriculture	Missing	Total	Number of men
**Age**									
15-19	2.1	0.0	9.2	23.9	1.2	63.2	0.4	100.0	641
20-24	5.8	1.1	11.6	31.6	1.1	47.7	1.0	100.0	772
25-29	9.6	1.7	13.0	27.5	2.3	44.3	1.7	100.0	799
30-34	9.7	2.1	13.4	28.7	1.2	44.5	0.4	100.0	906
35-39	6.1	0.6	11.1	27.8	0.6	53.0	0.9	100.0	556
40-44	8.5	2.0	14.1	20.7	0.1	54.1	0.6	100.0	592
45-49	8.5	1.0	16.7	15.1	0.6	57.9	0.2	100.0	553
**Marital status**									
Never married	6.4	1.0	11.9	27.2	1.1	50.7	1.6	100.0	1,309
Married or living together	7.9	1.4	12.8	24.9	1.1	51.5	0.4	100.0	3,391
Divorced/separated/ widowed	3.9	0.3	15.7	31.3	0.5	46.5	1.7	100.0	118
**Number of living children**									
0	6.9	1.2	12.0	27.8	1.3	49.3	1.5	100.0	1,685
1-2	8.3	1.8	13.1	27.4	1.3	47.7	0.4	100.0	1,717
3-4	7.8	0.8	13.7	23.5	0.8	53.1	0.3	100.0	1,053
5+	3.8	0.5	10.6	14.5	0.1	70.1	0.5	100.0	363
**Residence**									
Urban	20.1	4.8	28.7	32.7	2.4	8.6	2.6	100.0	749
Rural	5.0	0.6	9.7	24.4	0.8	59.0	0.4	100.0	4,069
**Province**									
Banteay Meanchey	7.5	1.1	10.9	22.6	1.8	55.7	0.5	100.0	189
Kampong Cham	5.0	0.2	10.9	19.3	1.9	62.3	0.4	100.0	602
Kampong Chhnang	7.7	0.0	7.8	20.0	0.6	62.4	1.5	100.0	168
Kampong Speu	4.9	0.5	7.3	30.7	0.7	55.1	0.9	100.0	296
Kampong Thom	5.5	0.2	9.1	18.1	0.2	66.5	0.5	100.0	230
Kandal	6.8	0.4	11.8	39.0	1.8	40.1	0.1	100.0	377
Kratie	6.3	0.6	5.2	17.8	0.9	68.9	0.3	100.0	132
Phnom Penh	21.9	4.9	29.6	35.5	2.2	2.8	3.1	100.0	462
Prey Veng	4.7	0.6	12.1	30.9	0.0	51.8	0.0	100.0	341
Pursat	2.4	1.3	6.9	13.7	0.0	75.2	0.4	100.0	168
Siem Reap	6.0	0.4	10.4	27.3	0.5	54.8	0.6	100.0	316
Svay Rieng	6.1	2.3	16.4	36.2	3.0	35.3	0.6	100.0	171
Takeo	7.1	0.0	13.8	29.5	0.6	48.6	0.6	100.0	321
Otdar Meanchey	5.1	1.1	8.5	7.1	0.0	78.3	0.0	100.0	91
Battambang/Pailin	5.1	3.9	13.7	23.8	0.4	52.2	0.9	100.0	390
Kampot/Kep	6.9	0.9	6.6	19.4	1.6	63.8	0.7	100.0	214
Preah Sihanouk/Koh Kong	10.5	2.5	17.4	36.7	1.7	29.8	1.4	100.0	110
Preah Vihear/Stung Treng	4.1	0.2	7.6	13.6	0.0	74.1	0.4	100.0	109
Mondul Kiri/Ratanak Kiri	5.6	1.4	14.9	8.4	0.0	69.6	0.0	100.0	131
**Education**									
No education	1.7	0.0	4.3	16.1	0.0	78.0	0.0	100.0	318
Primary	2.4	0.0	8.0	23.6	1.3	64.6	0.1	100.0	2,119
Secondary and higher	12.5	2.6	17.9	28.8	1.0	35.6	1.4	100.0	2,380
**Wealth quintile**									
Lowest	1.1	0.3	3.4	17.7	0.2	77.2	0.2	100.0	859
Second	2.5	0.0	3.8	25.0	0.3	68.4	0.0	100.0	919
Middle	3.7	0.4	8.0	24.8	1.0	61.6	0.5	100.0	980
Fourth	8.7	0.6	16.4	27.6	2.1	44.2	0.4	100.0	1,033
Highest	19.1	4.9	28.9	31.9	1.6	11.0	2.6	100.0	1,027
Total	7.4	1.3	12.7	25.7	1.1	51.2	0.8	100.0	4,818

5.4.3 Earnings, Employers, and Continuity of Employment

Table 5.7 shows the percent distribution of employed women by type of earnings and employment characteristics. Because all of the employment variables in the table are strongly influenced by the sector in which a woman is employed, data are grouped according to agricultural or nonagricultural work.

Table 5.7 Type of employment: Women

Percent distribution of women age 15-49 employed in the 12 months preceding the survey by type of earnings, type of employer, and continuity of employment, according to type of employment (agricultural or nonagricultural), Cambodia 2014

Employment characteristic	Agricultural work	Nonagricultural work	Missing	Total
**Type of earnings**				
Cash only	67.5	97.0	46.7	83.3
Cash and in-kind	15.6	1.3	2.3	7.6
In-kind only	8.9	0.0	0.9	3.9
Not paid	8.1	1.6	49.7	5.2
Total	100.0	100.0	100.0	100.0
**Type of employer**				
Employed by family member	13.8	4.6	5.4	8.6
Employed by nonfamily member	13.8	51.8	35.6	34.9
Self-employed	72.5	43.6	58.7	56.4
Total	100.0	100.0	100.0	100.0
**Continuity of employment**				
All year	16.1	85.9	67.9	55.1
Seasonal	81.0	10.0	19.5	41.2
Occasional	2.8	4.1	12.3	3.7
Total	100.0	100.0	100.0	100.0
Number of women employed during the last 12 months	6,115	7,644	219	13,978

Note: Total includes women with missing information on type of employment who are not shown separately.

One in four women engaged in agricultural work are paid in-kind or through a combination of cash and in-kind, 68 percent are paid in cash only, and 8 percent are unpaid. Women employed in the nonagricultural sector are more likely to be paid in cash only (97 percent). Nationally, across all occupations, 83 percent of employed women are paid in cash and 8 percent are paid in cash and in-kind for their work. Five percent are not paid at all for their work.

In 2014, 56 percent of employed Cambodian women are self-employed, and 9 percent are employed by a family member. Thirty-five percent of employed women work for someone outside the family. Among women working in the agricultural sector, almost three-quarters (73 percent) are working for themselves, as compared with 44 percent of those in the nonagricultural sector. In addition, the proportion of women employed by someone outside the family is nearly four times higher among those working in the nonagricultural sector than among those in the agricultural sector (52 percent versus 14 percent).

Fifty-five percent of employed women work all year, and 41 percent work seasonally. Those who work occasionally account for only 4 percent. Among women working in the agricultural sector, 81 percent are seasonal workers, as compared with only 10 percent of those working in the nonagricultural sector. Continuity of employment is more assured for women engaged in nonagricultural work than for those in agricultural work. For example, 86 percent of women working in the nonagricultural sector work all year, as compared with 16 percent of women engaged in agricultural work.

5.5 Health Insurance

In the 2014 CDHS, women and men age 15-49 were asked whether they were covered by any health insurance and, if so, which type. The choices were the following: a health equity fund, a maternal health voucher, community-based insurance, employer-based insurance, and privately purchased commercial insurance. Tables 5.8.1 and 5.8.2 show health insurance coverage for women and men in Cambodia.

Table 5.8.1 Health insurance coverage: Women

Percentage of women age 15-49 with specific types of health insurance coverage, according to background characteristics, Cambodia 2014

Background characteristic	Health equity fund	Maternal health voucher	Community-based health insurance	Employer-based insurance	Privately purchased commercial insurance	Other	None	Number of women
**Age**								
15-19	9.3	0.3	0.8	2.8	0.1	0.1	86.7	2,893
20-24	8.6	0.7	0.8	4.3	0.1	0.1	85.9	3,017
25-29	12.5	0.5	0.6	3.1	0.4	0.0	83.0	2,836
30-34	12.4	0.4	1.1	2.9	0.3	0.0	83.4	3,046
35-39	15.5	0.4	1.1	1.0	0.3	0.0	81.9	1,839
40-44	13.1	0.8	1.1	1.1	0.3	0.0	84.5	2,030
45-49	13.3	0.2	1.4	0.8	0.1	0.0	84.5	1,916
**Residence**								
Urban	5.9	0.2	1.0	7.0	0.6	0.0	85.6	3,251
Rural	13.1	0.5	0.9	1.5	0.1	0.0	84.1	14,327
**Province**								
Banteay Meanchey	14.7	0.4	0.0	0.0	0.0	0.0	85.0	689
Kampong Cham	14.6	0.7	1.9	0.5	0.5	0.0	82.9	2,021
Kampong Chhnang	26.0	0.0	0.0	0.0	0.0	0.0	74.0	662
Kampong Speu	1.5	0.0	0.2	3.6	0.0	0.5	94.2	1,196
Kampong Thom	17.6	0.0	2.6	0.0	0.0	0.0	79.8	851
Kandal	1.2	0.0	0.0	0.3	0.0	0.0	98.4	1,330
Kratie	19.3	0.2	0.8	0.0	0.0	0.0	79.8	488
Phnom Penh	3.8	0.0	1.5	16.0	0.8	0.0	78.4	1,994
Prey Veng	9.3	0.0	0.1	0.0	0.0	0.0	90.7	1,188
Pursat	18.2	0.1	0.9	0.1	0.1	0.0	80.9	631
Siem Reap	15.5	0.1	4.8	1.4	0.2	0.0	78.9	1,137
Svay Rieng	13.0	0.5	0.0	2.2	0.0	0.0	84.7	654
Takeo	12.3	3.3	0.3	0.4	0.3	0.0	83.6	1,082
Otdar Meanchey	21.2	0.0	0.6	0.0	0.0	0.0	78.2	294
Battambang/Pailin	25.0	0.1	0.3	0.0	0.0	0.0	74.7	1,333
Kampot/Kep	5.1	1.5	0.0	0.0	0.1	0.0	93.6	770
Preah Sihanouk/Koh Kong	18.3	1.8	0.0	6.6	0.0	0.0	73.6	422
Preah Vihear/Stung Treng	0.8	0.4	0.0	0.0	0.0	0.0	98.8	462
Mondul Kiri/Ratanak Kiri	2.9	0.0	0.0	0.0	0.2	0.2	96.8	372
**Education**								
No education	21.0	0.7	1.1	0.4	0.1	0.0	77.1	2,250
Primary	14.5	0.5	0.9	1.7	0.1	0.0	82.8	8,281
Secondary and higher	5.7	0.4	1.0	4.1	0.4	0.0	88.7	7,047
**Wealth quintile**								
Lowest	24.3	0.4	1.7	0.5	0.1	0.1	73.5	3,143
Second	17.8	0.7	0.6	0.6	0.0	0.0	80.6	3,314
Middle	9.8	0.9	0.9	1.3	0.1	0.1	87.3	3,381
Fourth	6.9	0.2	0.9	2.3	0.2	0.0	89.9	3,612
Highest	3.3	0.2	0.7	6.8	0.6	0.0	88.6	4,128
Total	11.8	0.5	0.9	2.5	0.2	0.0	84.4	17,578

The majority of Cambodians, 84 percent of women and 87 percent of men, do not have health insurance. These percentages are slightly improved compared with the 2010 CDHS (89 percent and 92 percent, respectively). As for those who are insured, the gross majority (12 percent of women and 9 percent of men) are insured through a health equity fund. One percent of respondents report having community-based health insurance, and about 3 percent report being covered through employer-based health insurance, privately purchased commercial health insurance, or, in the case of female respondents, maternal health vouchers. These data imply that the health insurance system in the country is not widespread in its reach.

Table 5.8.2 Health insurance coverage: Men

Percentage of men age 15-49 with specific types of health insurance coverage, according to background characteristics, Cambodia 2014

Background characteristic	Health equity fund	Community-based health insurance	Employer-based insurance	Privately purchased commercial insurance	Other	None	Number of men
**Age**							
15-19	7.1	0.3	2.1	0.0	0.0	90.8	926
20-24	5.3	0.6	3.6	0.2	0.0	90.3	835
25-29	8.4	1.9	4.6	0.4	0.0	85.1	815
30-34	10.0	0.8	3.6	0.8	0.1	84.8	907
35-39	9.5	3.0	1.5	0.2	0.0	85.8	556
40-44	9.9	1.0	1.6	0.4	0.0	87.0	595
45-49	10.9	0.6	1.7	0.7	0.0	86.0	556
**Residence**							
Urban	4.7	0.7	10.8	1.0	0.0	82.8	869
Rural	9.3	1.2	1.2	0.3	0.0	88.2	4,321
**Province**							
Banteay Meanchey	5.6	0.0	3.9	0.3	0.0	91.4	192
Kampong Cham	11.6	1.0	0.0	0.0	0.0	88.0	663
Kampong Chhnang	24.2	0.0	0.0	0.0	0.0	75.8	182
Kampong Speu	4.3	0.4	1.6	0.1	0.0	93.5	323
Kampong Thom	1.0	0.1	0.2	0.5	0.0	98.2	232
Kandal	0.2	4.0	0.4	0.5	0.0	95.0	413
Kratie	20.5	0.2	0.5	0.1	0.0	78.7	143
Phnom Penh	3.2	0.9	19.0	1.0	0.0	75.8	550
Prey Veng	2.2	0.0	1.0	0.6	0.0	96.3	342
Pursat	14.2	0.4	0.0	0.0	0.0	85.4	184
Siem Reap	15.9	5.1	1.1	0.9	0.3	76.7	337
Svay Rieng	0.1	0.0	2.9	0.5	0.0	96.5	183
Takeo	11.2	2.2	0.8	0.4	0.0	85.7	334
Otdar Meanchey	4.6	0.0	0.0	0.0	0.0	95.4	99
Battambang/Pailin	20.6	0.0	0.0	0.2	0.0	79.1	405
Kampot/Kep	8.5	0.0	0.0	0.0	0.0	91.5	241
Preah Sihanouk/Koh Kong	9.1	1.6	9.6	1.4	0.3	78.9	120
Preah Vihear/Stung Treng	1.1	0.0	0.1	0.0	0.0	98.8	112
Mondul Kiri/Ratanak Kiri	0.9	0.0	0.0	0.0	0.0	99.1	134
**Education**							
No education	13.3	1.6	0.2	0.6	0.0	84.4	324
Primary	12.0	1.6	0.9	0.1	0.0	85.6	2,167
Secondary and higher	5.1	0.6	4.7	0.6	0.0	89.1	2,699
**Wealth quintile**							
Lowest	17.6	1.1	0.1	0.0	0.1	81.5	901
Second	12.6	1.3	0.5	0.2	0.0	85.4	954
Middle	7.5	1.2	0.7	0.1	0.0	90.5	1,040
Fourth	5.2	1.2	2.5	0.6	0.0	90.8	1,124
Highest	2.3	0.7	9.1	0.9	0.0	87.2	1,171
Total	8.5	1.1	2.8	0.4	0.0	87.3	5,190

There are notable differentials in the proportions of women and men with health equity funds according to background characteristics. Both rural women and rural men are about twice as likely as their urban counterparts to have a health equity fund. The proportion of those with a health equity fund is higher among women and men with no education and in the lower wealth quintiles. For example, 24 percent of women in the lowest wealth quintile are covered through a health equity fund, as compared with only 3 percent of women in the highest wealth quintile. Health equity fund coverage among women varies by province, ranging from about 1 percent in Preah Vihear/Stung Treng and Kandal to 26 percent in Kampong Chhnang.

5.6 USE OF TOBACCO

Smoking or other use of tobacco affects one's health and may adversely affect the health of one's children, especially in terms of vulnerability to respiratory illness. In addition, tobacco use during pregnancy increases a woman's risk of having a small or low-birth-weight baby. All interviewed respondents in the 2014 CDHS were asked about their smoking habits. Tables 5.9.1 and 5.9.2 show the percentage of women and men, respectively, who use various types of tobacco and the percent distribution of cigarette smokers by number of cigarettes smoked in the preceding 24 hours, according to background characteristics.

Table 5.9.1 Use of tobacco: Women

Percentage of women age 15-49 who smoke cigarettes or a pipe or use other tobacco products, according to background characteristics and maternity status, Cambodia 2014

Background characteristic	Uses tobacco: Ciga-rettes	Uses tobacco: Pipe	Uses tobacco: Other tobacco	Does not use tobacco	Number of women	Percent distribution of women who smoke cigarettes by number of cigarettes smoked in the past 24 hours: 0	1-2	3-5	6-9	10+	Don't know/ missing	Total	Number of ciga-rette smokers
**Age**													
15-19	0.5	0.0	0.3	99.3	2,893	*	*	*	*	*	*	100.0	14
20-24	0.6	0.0	0.6	98.9	3,017	*	*	*	*	*	*	100.0	19
25-29	1.6	0.0	1.0	98.0	2,836	0.0	16.0	45.9	5.2	30.8	2.2	100.0	44
30-34	2.0	0.1	2.7	95.9	3,046	0.0	25.8	33.2	4.9	27.1	8.9	100.0	61
35-39	4.6	0.1	5.8	90.9	1,839	0.0	17.2	35.4	12.4	32.4	2.6	100.0	85
40-44	4.8	0.1	10.5	85.7	2,030	0.0	13.3	31.1	10.1	42.5	3.0	100.0	98
45-49	4.7	0.2	13.7	82.6	1,916	0.0	19.9	33.7	11.6	33.3	1.5	100.0	89
**Maternity status**													
Pregnant	1.1	0.0	1.6	97.8	934	*	*	*	*	*	*	100.0	10
Breastfeeding (not pregnant)	3.0	0.2	2.6	95.2	2,348	0.0	23.3	41.5	7.6	26.6	1.1	100.0	70
Neither	2.3	0.1	4.5	93.7	14,296	0.0	16.9	34.2	10.1	34.4	4.4	100.0	330
**Residence**													
Urban	0.6	0.0	0.7	98.8	3,251	(0.0)	(21.9)	(43.3)	(4.7)	(26.1)	(3.9)	100.0	20
Rural	2.7	0.1	4.9	93.1	14,327	0.0	17.9	35.2	10.2	33.0	3.7	100.0	390
**Province**													
Banteay Meanchey	1.1	0.0	3.5	95.6	689	*	*	*	*	*	*	100.0	7
Kampong Cham	4.6	0.0	5.9	90.5	2,021	(0.0)	(17.8)	(24.5)	(10.4)	(43.5)	(3.8)	100.0	93
Kampong Chhnang	0.0	0.0	3.6	96.4	662	*	*	*	*	*	*	0.0	0
Kampong Speu	0.5	0.0	1.6	97.9	1,196	*	*	*	*	*	*	100.0	6
Kampong Thom	1.3	0.0	3.6	95.6	851	*	*	*	*	*	*	100.0	11
Kandal	0.3	0.0	2.5	97.4	1,330	*	*	*	*	*	*	100.0	4
Kratie	12.4	0.0	7.9	81.4	488	0.0	32.6	32.5	9.2	25.7	0.0	100.0	60
Phnom Penh	0.5	0.0	0.7	98.8	1,994	*	*	*	*	*	*	100.0	10
Prey Veng	0.8	0.0	8.4	91.1	1,188	*	*	*	*	*	*	100.0	10
Pursat	2.0	0.0	7.8	90.5	631	*	*	*	*	*	*	100.0	12
Siem Reap	1.4	0.1	4.2	94.4	1,137	*	*	*	*	*	*	100.0	16
Svay Rieng	0.0	0.0	8.3	91.7	654	*	*	*	*	*	*	0.0	0
Takeo	1.1	0.0	1.8	97.8	1,082	*	*	*	*	*	*	100.0	12
Otdar Meanchey	1.5	0.0	3.3	95.7	294	*	*	*	*	*	*	100.0	4
Battambang/Pailin	2.4	0.0	1.8	96.0	1,333	*	*	*	*	*	*	100.0	32
Kampot/Kep	0.9	0.0	1.6	97.7	770	*	*	*	*	*	*	100.0	7
Preah Sihanouk/ Koh Kong	0.8	0.0	0.6	98.7	422	*	*	*	*	*	*	100.0	3
Preah Vihear/ Stung Treng	14.4	0.0	12.4	77.0	462	0.0	24.6	50.3	4.0	17.8	3.2	100.0	67
Mondul Kiri/ Ratanak Kiri	14.9	3.3	11.3	82.1	372	0.0	13.1	53.3	9.3	24.3	0.0	100.0	55
**Education**													
No education	8.2	0.4	11.3	83.4	2,250	0.0	21.6	37.9	11.9	27.6	0.9	100.0	185
Primary	2.4	0.1	5.1	92.9	8,281	0.0	15.9	37.7	9.4	30.9	6.2	100.0	200
Secondary and higher	0.4	0.0	0.6	99.1	7,047	*	*	*	*	*	*	100.0	25
**Wealth quintile**													
Lowest	6.1	0.3	9.7	85.5	3,143	0.0	19.6	35.3	13.5	31.1	0.5	100.0	192
Second	3.2	0.1	5.4	92.6	3,314	0.0	19.4	34.8	9.0	33.5	3.4	100.0	106
Middle	2.1	0.0	3.9	94.4	3,381	0.0	13.8	38.4	4.8	35.9	7.0	100.0	71
Fourth	0.9	0.0	2.5	96.8	3,612	(0.0)	(11.5)	(29.2)	(5.3)	(38.6)	(15.4)	100.0	33
Highest	0.2	0.0	0.4	99.4	4,128	*	*	*	*	*	*	100.0	8
Total	2.3	0.1	4.1	94.2	17,578	0.0	18.1	35.6	9.9	32.7	3.7	100.0	410

Note: Figures in parentheses are based on 25-49 unweighted cases. An asterisk indicates that a figure is based on fewer than 25 unweighted cases and has been suppressed.

Overall, 6 percent of women in Cambodia use some form of tobacco, a slight decrease from self-reported tobacco use among women in the 2010 CDHS. Two percent smoke cigarettes and 4 percent use a form of tobacco other than cigarettes or a pipe (some women use more than one form of tobacco). Only 2 percent of pregnant women and 5 percent of women who are breastfeeding use tobacco. Tobacco use is much higher among men, with 32 percent of Cambodian men reporting that they smoke cigarettes and 5 percent reporting that they use other forms of tobacco.

Tobacco use varies greatly by background characteristics. Older women and men are much more likely to use tobacco than are younger women and men. Cigarette smoking increases from less than 1

percent among women age 15-19 to 5 percent among women 35-49. Similarly, reported cigarette smoking in men increases from 8 percent among those age 15-19 to 53 percent among those in the oldest age cohort. Use of tobacco other than cigarettes also increases with age among both women and men.

Table 5.9.2 Use of tobacco: Men

Percentage of men age 15-49 who smoke cigarettes or a pipe or use other tobacco products and the percent distribution of cigarette smokers by number of cigarettes smoked in preceding 24 hours, according to background characteristics, Cambodia 2014

	Uses tobacco					Percent distribution of men who smoke cigarettes by number of cigarettes smoked in the past 24 hours							
Background characteristic	Ciga-rettes	Pipe	Other tobacco	Does not use tobacco	Number of men	0	1-2	3-5	6-9	10+	Don't know/ missing	Total	Number of ciga-rette smokers
**Age**													
15-19	8.2	0.1	1.1	91.8	926	0.0	20.2	27.2	14.1	38.4	0.0	100.0	76
20-24	23.7	0.0	2.9	76.1	835	0.0	6.7	15.9	8.2	69.2	0.0	100.0	198
25-29	25.0	0.0	3.2	74.6	815	0.1	2.4	12.0	7.7	77.8	0.0	100.0	204
30-34	36.8	0.1	6.8	62.1	907	0.6	3.0	15.5	8.2	72.7	0.0	100.0	334
35-39	45.5	0.1	6.3	53.0	556	0.0	2.5	7.2	4.2	86.1	0.0	100.0	253
40-44	48.7	0.1	7.1	49.2	595	0.0	3.6	7.7	7.7	81.0	0.0	100.0	290
45-49	53.4	0.0	10.5	45.1	556	0.0	1.1	10.7	7.3	80.6	0.3	100.0	297
**Residence**													
Urban	21.6	0.0	1.5	78.4	869	0.1	9.7	17.5	8.8	63.9	0.0	100.0	187
Rural	33.9	0.1	5.7	65.1	4,321	0.1	3.1	11.5	7.4	77.8	0.1	100.0	1,466
**Province**													
Banteay Meanchey	34.7	0.0	0.7	64.7	192	0.0	1.8	10.2	3.0	85.0	0.0	100.0	67
Kampong Cham	37.9	0.0	2.1	61.1	663	0.0	1.6	4.3	8.8	85.3	0.0	100.0	251
Kampong Chhnang	25.4	0.0	10.7	66.6	182	0.0	2.2	18.3	5.8	73.7	0.0	100.0	46
Kampong Speu	30.9	0.0	0.0	69.1	323	0.0	11.2	14.2	7.0	67.6	0.0	100.0	100
Kampong Thom	34.8	0.0	2.4	63.4	232	0.0	0.0	4.8	11.3	83.9	0.0	100.0	81
Kandal	22.4	0.0	1.6	76.5	413	0.0	0.0	36.3	4.7	59.0	0.0	100.0	93
Kratie	37.5	0.0	0.0	62.5	143	0.0	5.5	12.1	9.8	72.6	0.0	100.0	54
Phnom Penh	17.9	0.0	2.6	82.1	550	2.2	18.3	21.7	7.2	50.7	0.0	100.0	99
Prey Veng	31.6	0.0	31.6	67.8	342	0.0	0.0	19.1	7.2	73.7	0.0	100.0	108
Pursat	30.3	0.0	2.1	69.7	184	0.0	9.1	7.1	12.8	69.6	1.3	100.0	56
Siem Reap	40.6	0.0	0.3	59.1	337	0.0	3.5	13.3	2.9	80.3	0.0	100.0	137
Svay Rieng	29.6	0.0	3.4	68.1	183	0.0	6.7	12.2	5.7	75.4	0.0	100.0	54
Takeo	30.3	0.0	1.0	68.7	334	0.1	1.6	6.9	7.6	83.8	0.0	100.0	101
Otdar Meanchey	41.2	0.0	0.4	58.4	99	0.0	1.7	5.8	8.6	83.9	0.0	100.0	41
Battambang/Pailin	31.4	0.0	0.0	68.6	405	0.0	0.0	3.8	8.8	87.4	0.0	100.0	127
Kampot/Kep	37.0	0.0	4.2	63.0	241	0.0	4.5	12.5	5.0	78.0	0.0	100.0	89
Preah Sihanouk/ Koh Kong	37.9	0.0	0.0	62.1	120	0.0	4.7	7.5	12.8	75.0	0.0	100.0	46
Preah Vihear/ Stung Treng	44.7	0.0	8.3	55.3	112	0.0	6.3	17.0	15.2	61.5	0.0	100.0	50
Mondul Kiri/ Ratanak Kiri	40.2	1.7	40.1	58.4	134	0.0	0.7	15.9	5.0	78.4	0.0	100.0	54
**Education**													
No education	59.4	0.2	16.3	38.0	324	0.0	4.8	7.5	4.6	83.1	0.0	100.0	193
Primary	41.8	0.1	5.8	56.9	2,167	0.0	2.0	9.7	8.3	79.9	0.1	100.0	906
Secondary and higher	20.5	0.0	2.9	79.2	2,699	0.4	6.6	17.7	7.3	68.0	0.0	100.0	554
**Wealth quintile**													
Lowest	46.9	0.1	9.6	50.7	901	0.0	1.9	11.8	10.5	75.8	0.0	100.0	422
Second	36.4	0.1	6.7	62.4	954	0.0	2.5	8.7	8.6	80.2	0.0	100.0	348
Middle	34.1	0.1	6.0	65.2	1,040	0.0	3.6	7.4	5.3	83.7	0.0	100.0	355
Fourth	27.2	0.0	2.6	72.6	1,124	0.7	4.5	13.8	5.4	75.3	0.2	100.0	306
Highest	19.0	0.0	1.4	80.9	1,171	0.1	9.2	23.4	6.8	60.5	0.0	100.0	223
Total	31.8	0.0	5.0	67.3	5,190	0.1	3.9	12.2	7.5	76.2	0.0	100.0	1,653

Women and men in rural areas, those with less education, and those in the lower wealth quintiles are more likely to use tobacco. Only 1 percent of women in urban areas use tobacco, as compared with 7 percent of women in rural areas. Likewise, almost 22 percent of urban men report using tobacco, as compared with 35 percent of their rural counterparts. Tobacco use ranges from less than 1 percent among women with a secondary education or higher and those in the highest wealth quintile to 17 percent among women with no education and 14 percent among those in the lowest wealth quintile. Men show a pattern similar to that of women. Tobacco use rates are highest among women in Preah Vihear/Stung Treng, where 23 percent of women use tobacco, in the form of cigarettes (14 percent) or other form of tobacco (12 percent), and in Mondul Kiri/Ratanak Kiri (18 percent), where the proportion of women using cigarettes is 15 percent and that using other forms of tobacco is 11 percent). Among men, tobacco use is highest in

Preah Vihear/Stung Treng (45 percent), where high proportions of men report smoking cigarettes (45 percent). Using other forms of tobacco is highest among men in Mondul Kiri/Ratanak Kiri (40 percent).

Respondents who reported smoking cigarettes were asked to recall the number of cigarettes smoked in the past 24 hours. The differentials in smoking frequency among female smokers are very small due to the small number of women in Cambodia who smoke. However, Table 5.9.1 shows that 36 percent of women who smoke cigarettes report smoking 3-5 cigarettes per day, and 33 percent report smoking 10 or more cigarettes per day. Proportions among women are much lower than those among men, as can be seen in Table 4.9.2. Seventy-six percent of male smokers reported smoking 10 or more cigarettes in the past 24 hours. This proportion is much higher among men living in Battambang/Pailin, where 87 percent report smoking 10 or more cigarettes in the past 24 hours.

FERTILITY 6

Key Findings

- The total fertility rate in Cambodia for the three years preceding the survey is 2.7 children per woman. Rural women have almost one child more than urban women.
- Fertility declined by 0.4 children per woman between 2005 and 2010, from 3.4 children to 3.0 children per woman, and slightly decreased further to 2.7 children in 2014.
- One-tenth of women age 25-49 gave birth by age 18 and 28 percent by age 20. The median age at first birth is 22.4 years.
- Thirty-seven percent of births occur within three years of a previous birth; 13 percent occur within 24 months.
- Twelve percent of young women age 15-19 are already mothers or pregnant with their first child.

Fertility is an important component of population dynamics and plays a large role in changing the size and structure of the population of a given area. In Cambodia, population size and structure were severely affected during the reign of the Khmer Rouge (1975-1979), in terms of both excess mortality and reduced fertility. The CDHS generates detailed information on fertility and fertility patterns over time that will be useful for the formulation of policies and the design of programs.

Current fertility levels, trends and differentials in fertility, cumulative fertility, birth intervals, age at first birth, and adolescent fertility are examined in this chapter. The fertility indicators presented in this chapter are based on information obtained from women age 15-49. All women who were interviewed in the 2014 CDHS were asked to report the total number of daughters and sons they had given birth to in their lifetime. To encourage complete reporting, women were asked separately about children still living at home, those living elsewhere, and those who had died. A complete birth history was then obtained, including information on the sex, date of birth,[1] and survival status of each child and the age at death for deceased children.

6.1 CURRENT FERTILITY LEVELS AND DIFFERENTIALS

The current level of fertility refers to live births in the three-year period preceding the survey. This information was obtained from birth history data and is presented in Table 6.1. The summary measures include age-specific fertility rates (ASFRs),[2] total fertility rates (TFRs) for women age 15-49, the general fertility rate (GFR), and the crude birth rate (CBR). The ASFRs represent the number of live births per 1,000 women in the age group. The TFR is a common measure of current fertility and is defined as the total number of births a woman would have by the end of her childbearing years if she were to pass through those years bearing children at the currently observed age-specific fertility rates. The GFR is

---

[1] During data collection, interviewers recorded Gregorian month and year of birth. However, when the respondent knew only the Khmer month and year of birth, the interviewer used a chart specially designed for the CDHS to convert Khmer dates into Gregorian dates.

[2] Numerators of the three-year ASFRs are calculated by summing the number of live births that occurred in the period 1-36 months preceding the survey (determined by the date of the interview and the date of birth of the child) and classifying them by age (in 5-year groups) of the mother at the time of the birth (determined by the mother's birth date). The denominators of the rates are the number of woman-years lived in each of the specified 5-year age groups during the 1-36 months preceding the survey.

defined as the annual number of births per 1,000 women age 15-44. The CBR is the total number of births occurring in a given year per 1,000 population.

The total fertility rate in Cambodia for the three years preceding the survey indicates that if fertility rates were to remain constant at the level prevailing during the period 2012-2014, a Cambodian woman would bear 2.7 children during her lifetime. The average Cambodian woman will give birth to 1.1 children by age 25[3] and 1.9 children by age 30. The TFR in urban areas is 2.1 births per woman, almost one child lower than the rate in rural areas (2.9 births per woman). An examination of age-specific rates by urban-rural residence indicates that the age pattern of fertility is quite different in urban and rural areas. Fertility rates are higher in nearly every age group for rural women than for urban women. Among women age 15-19, fertility rates are quite low in both urban and rural areas (21 and 66 per 1,000 women, respectively). Among rural women, rates quickly increase to reach their maximum at age 20-24 (179 per 1,000) and remain quite high at age 25-29 (156 per 1,000) before declining regularly above age 29. Among urban women, fertility rates increase from 21 per 1,000 at age 15-19 to 101 per 1,000 at age 20-24 and reach a maximum of 135 per 1,000 at age 25-29. They then decline regularly, similar to rates among rural women.

The CBR, also presented in Table 6.1, is 22.0 per 1,000 population. The GFR, the average annual number of births per 1,000 women age 15-44 for the three years prior to the survey, is 98. As with the TFR, the GFR and CBR vary by urban-rural residence. The GFR for rural women is 103 births per 1,000 women, which is about 36 percent higher than that for urban women (76 births per 1,000 women). Also, the CBR in rural areas (22.4 per 1,000 population) is approximately 10 percent higher than the CBR in urban areas (20.2 per 1,000 population).

Table 6.1 Current fertility

Age-specific and total fertility rates, the general fertility rate, and the crude birth rate for the three years preceding the survey, by residence, Cambodia 2014

	Residence		
Age group	Urban	Rural	Total
15-19	21	66	57
20-24	101	179	162
25-29	135	156	152
30-34	92	104	102
35-39	56	50	51
40-44	11	18	17
45-49	3	5	4
TFR (15-49)	2.1	2.9	2.7
GFR	76	103	98
CBR	20.2	22.4	22.0

Notes: Age-specific fertility rates are per 1,000 women. Rates for age group 45-49 may be slightly biased due to truncation. Rates are for the period 1-36 months prior to the interview.
TFR: Total fertility rate, expressed per woman
GFR: General fertility rate, expressed per 1,000 women age 15-44
CBR: Crude birth rate, expressed per 1,000 population

Table 6.2 presents differentials in fertility by urban-rural residence, province, education, and wealth quintile. There are large differences in fertility levels across provinces. Fertility is lowest in the capital city of Phnom Penh, at 2.0 children per woman, and highest in Preah Vihear/Stung Treng and Kratie, at 3.6 children per woman. Among the remaining provinces, total fertility ranges from 2.4 to 3.3. Fertility is well known to be inversely related to level of education around the world, and Cambodian women demonstrate this universal pattern. A woman with no education (TFR of 3.3) has 0.2 children more

[3] Calculated as the age-specific fertility rate for women age 15-19 plus the age-specific fertility rate for women age 20-24, multiplied by 5 (to take into account the five-year age group) and divided by 1,000.

than a woman with a primary school education (TFR of 3.1) and one child more than a woman with a secondary education or higher (TFR of 2.3). Fertility is also very closely associated with wealth. The disparity in fertility between the poorest women, who have the most children (3.8), and the richest women, who have the fewest (2.2), is 1.6 children per woman.

Table 6.2 Fertility by background characteristics

Total fertility rate for the three years preceding the survey, percentage of women age 15-49 currently pregnant, and mean number of children ever born to women age 40-49, by background characteristics, Cambodia 2014

Background characteristic	Total fertility rate	Percentage of women age 15-49 currently pregnant	Mean number of children ever born to women age 40-49
**Residence**			
Urban	2.1	4.9	3.0
Rural	2.9	5.4	4.0
**Province**			
Banteay Meanchey	2.8	5.2	4.0
Kampong Cham	3.3	3.5	3.9
Kampong Chhnang	2.4	5.4	4.2
Kampong Speu	2.4	6.3	4.1
Kampong Thom	2.9	5.8	4.4
Kandal	2.5	5.7	3.9
Kratie	3.6	7.3	4.5
Phnom Penh	2.0	4.6	2.8
Prey Veng	3.0	4.9	3.5
Pursat	3.1	5.9	4.0
Siem Reap	2.7	5.2	3.9
Svay Rieng	2.5	5.7	3.4
Takeo	2.4	3.9	3.7
Otdar Meanchey	3.0	8.5	4.6
Battambang/Pailin	2.9	5.5	3.8
Kampot/Kep	2.5	4.9	3.9
Preah Sihanouk/Koh Kong	2.7	5.8	4.1
Preah Vihear/Stung Treng	3.6	9.5	5.2
Mondul Kiri/Ratanak Kiri	3.3	6.9	4.8
**Education**			
No education	3.3	4.5	4.3
Primary	3.1	5.3	4.0
Secondary and higher	2.3	5.6	3.1
**Wealth quintile**			
Lowest	3.8	5.2	4.4
Second	2.8	6.4	4.3
Middle	2.8	4.9	3.9
Fourth	2.4	5.2	3.7
Highest	2.2	4.9	3.0
Total	2.7	5.3	3.9

Note: Total fertility rates are for the period 1-36 months prior to the interview.

Table 6.2 includes another indicator of current fertility, the percentage of women who reported being pregnant at the time of the survey. This percentage may be underreported because women may not be aware of a pregnancy, especially at the very early stages, and some women who are early in their pregnancy may not want to reveal that they are pregnant. Five percent of women reported that they were pregnant at the time of the survey. The proportion of pregnant women in urban areas and rural areas is about the same. Kampong Cham and Takeo had the lowest proportion of pregnant women (4 percent), and Preah Vihear/Stung Treng (10 percent) and Otdar/Meanchey (9 percent) had the highest. The proportion of women who are currently pregnant rises slightly as education increases. There is no clear relationship between current pregnancy and wealth quintile.

6.2 FERTILITY TRENDS

The 2014 CDHS data can be used to assess trends in fertility in Cambodia in several ways.

6.2.1 Comparison of Current and Cumulative Fertility Levels

Table 6.2 shows the mean number of live births among women age 40 to 49. This figure is an indicator of completed, or cumulative, fertility. Unlike the TFR, which measures the current or recent fertility of women age 15 to 49, cumulative fertility shows the past fertility of women surveyed at the end of their childbearing years. In a population whose fertility does not change, the level of cumulative fertility more or less coincides with the TFR. But TFRs that are lower than the mean number of children ever born to women at the end of their childbearing years indicate a downward trend in fertility.

In Cambodia, women age 40-49 have given birth to an average of 3.9 children. This is higher than the TFR (2.7). The difference, although small (1.2), suggests a substantial decline in fertility. Data from previous CDHS surveys show a difference between the two rates of 1.4 children in 2000, 1.5 children in 2005, and 1.2 children in 2010.

Fertility results by background characteristics show cumulative fertility rates above the TFR for all categories, indicating that fertility is declining among all women. However, the difference between cumulative fertility (number of children ever born) and the TFR is greatest in Kampong Chhnang (1.8 children).

6.2.2 Retrospective Data

Fertility trends can be investigated using retrospective data from the birth histories collected within the 2014 CDHS. Table 6.3.1 and Figure 6.1 show age-specific fertility rates (ASFRs) for successive five-year periods preceding the 2014 CDHS. Numerators of the rates are classified by five-year segments of time preceding the survey and the mother's age at the time of birth. Because women age 50 and over were not interviewed in the survey, the rates for older age groups become progressively more truncated for periods more distant from the survey date. For example, rates cannot be calculated for women age 35-39 for the period 15-19 years before the survey because these women would have been over age 50 at the time of the survey and were not interviewed.

Table 6.3.1 Trends in age-specific fertility rates

Age-specific fertility rates for five-year periods preceding the survey, by mother's age at the time of the birth, Cambodia 2014

Mother's age at birth	Number of years preceding survey			
	0-4	5-9	10-14	15-19
15-19	56	50	61	65
20-24	159	165	184	212
25-29	149	177	193	219
30-34	105	123	142	[187]
35-39	51	79	[109]	
40-44	18	[48]		
45-49	[4]			

Note: Age-specific fertility rates are per 1,000 women. Estimates in brackets are truncated. Rates exclude the month of the interview.

Figure 6.1 **Age-specific fertility rates for five-year periods preceding the survey**

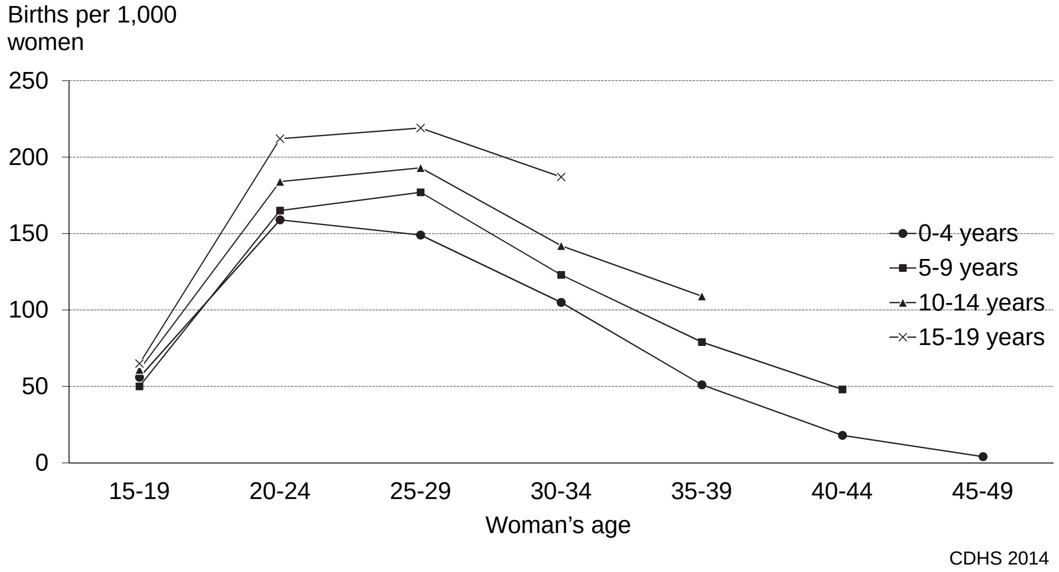

Age-specific fertility rates calculated over time provide further evidence of a substantial decline in fertility at all ages. Among young women age 15-19, the ASFR declined from 65 per 1,000 15-19 years before the survey to 50 per 1,000 5-9 years before the survey and then slightly increased to 56 per 1,000 during the most recent period (0-4 years before the survey). Fertility rates have also progressively declined over time among women age 20-49 at the birth of their child. For instance, among mothers age 20-24 when they gave birth, the ASFR fell from 212 per 1,000 15-19 years before the survey to 159 per 1,000 0-4 years before the survey. A similar pattern was observed among women in the other age groups. The data further show that the decline in fertility rates has slowed slightly in recent years.

6.2.3 Comparison with Previous CDHS

Another way to assess fertility trends is to compare current estimates with earlier surveys. Table 6.3.2 and Figure 6.2 show the ASFRs for the 2005, 2010, and 2014 CDHSs. The current TFR of 2.7 attests to a decline in fertility, from 3.4 children per woman reported in the 2005 CDHS. As mentioned, the decline in fertility has slowed slightly in recent years: the TFR decreased by 0.6 children per woman between 2000 and 2005 (data not shown), by 0.4 between 2005 and 2010, and by 0.3 between 2010 and 2014. Although fertility declined in both urban and rural areas, the change in the TFR between the 2010 CDHS and the 2014 CDHS occurred predominantly as a result of declining fertility among rural women. The TFR decreased by 0.4 children among rural women and by 0.1 children among urban women.

Table 6.3.2 Trends in fertility

Age-specific and total fertility rates (TFR) for the three years preceding the survey, Cambodia 2005, 2010, and 2014

Age group	CDHS 2005[1]	CDHS 2010[2]	CDHS 2014
15-19	47	46	57
20-24	175	173	162
25-29	180	167	152
30-34	142	121	102
35-39	91	71	51
40-44	41	28	28
45-49	5	4	5
Total	3.4	3.0	2.7

Note: Age-specific fertility rates are per 1,000 women.
[1] NIPH, NIS, and ORC Macro, 2006
[2] NIS, DGH, and ICF Macro, 2011

Figure 6.2 Trends in age-specific fertility rates, Cambodia 2005, 2010, and 2014

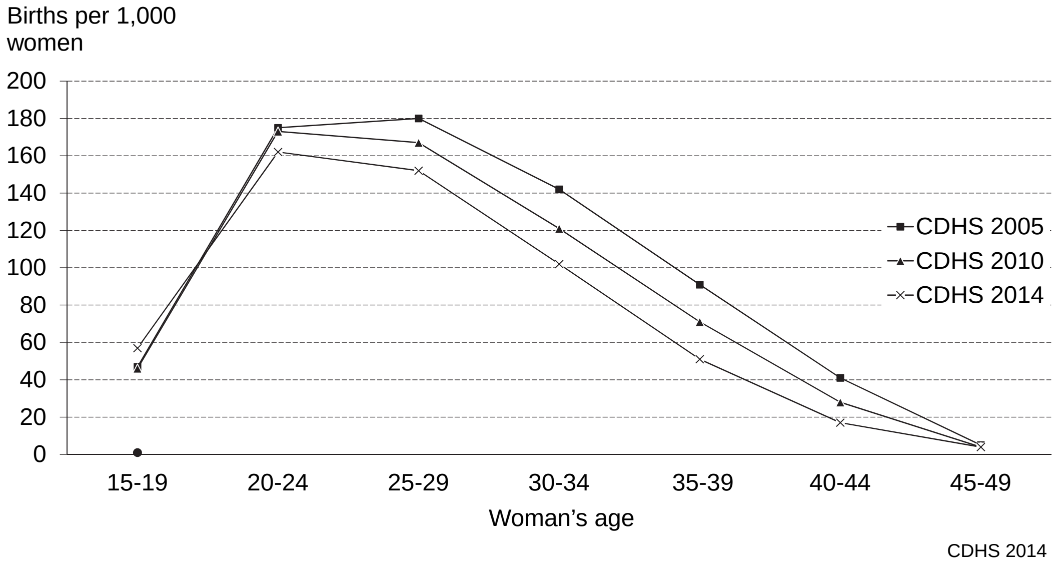

Declines in ASFRs between 2010 and 2014 have occurred among women age 20-24 and older. The age groups in which women have demonstrated the largest decreases in fertility are 25-29, 30-34, and 35-39, with women in these age groups showing a decrease of 15-20 births per 1,000 women. In contrast, there has been a rise in teenage fertility in Cambodia over the past few years, with age-specific fertility rates among young women age 15-19 increasing from 46 children per 1,000 women in 2010 to 57 children per 1,000 women in 2014.

6.3 CHILDREN EVER BORN AND LIVING

Data on the number of children ever born reflect the accumulation of births over the past 30 years and therefore have limited relevance to current fertility levels, particularly when a country has experienced a decline in fertility. Nevertheless, information on children ever born (or parity) is useful in looking at how average family size varies across age groups and in assessing the level of primary infertility, the inability to bear children. A comparison of the differences in the mean number of children ever born and surviving reflects the cumulative effects of mortality levels during the period in which women have been bearing children.

Table 6.4 shows the percent distribution of all women and currently married women by the number of children ever born, the mean number of children ever born, and the mean number of children living. More than 9 in 10 women age 15-19 (93 percent) have never given birth. However, this proportion declines quickly to 23 percent among women age 25-29 and to 9 percent or less among women age 35 and above. On average, Cambodian women have attained a parity of 4.1 children by the end of their reproductive years. This is 1.4 children more than the total fertility rate, a difference brought about by sustained declines in fertility.

Table 6.4 Children ever born and living

Percent distribution of all women and currently married women age 15-49 by number of children ever born, mean number of children ever born, and mean number of living children, according to age group, Cambodia 2014

Age	Number of children ever born											Total	Number of women	Mean number of children ever born	Mean number of living children
	0	1	2	3	4	5	6	7	8	9	10+				
ALL WOMEN															
15-19	92.7	6.7	0.6	0.0	0.0	0.0	0.0	0.0	0.0	0.0	0.0	100.0	2,893	0.08	0.08
20-24	50.6	34.7	13.1	1.4	0.3	0.0	0.0	0.0	0.0	0.0	0.0	100.0	3,017	0.66	0.64
25-29	23.0	28.9	33.3	11.5	2.7	0.6	0.1	0.1	0.0	0.0	0.0	100.0	2,836	1.44	1.39
30-34	11.1	14.7	34.8	23.9	10.3	3.7	1.1	0.2	0.1	0.1	0.0	100.0	3,046	2.25	2.14
35-39	8.7	7.4	21.9	24.0	20.4	9.7	4.0	2.9	0.7	0.3	0.0	100.0	1,839	3.06	2.81
40-44	7.1	6.0	16.3	21.4	19.7	13.2	9.2	4.1	1.3	0.8	0.9	100.0	2,030	3.59	3.23
45-49	6.8	6.4	10.9	16.4	18.6	14.6	12.1	6.1	4.4	1.7	2.2	100.0	1,916	4.13	3.57
Total	32.0	16.4	19.1	13.0	8.7	4.9	3.0	1.5	0.7	0.3	0.3	100.0	17,578	1.93	1.77
CURRENTLY MARRIED WOMEN															
15-19	56.8	39.5	3.6	0.1	0.0	0.0	0.0	0.0	0.0	0.0	0.0	100.0	450	0.47	0.46
20-24	22.3	54.4	20.8	2.1	0.3	0.0	0.0	0.0	0.0	0.0	0.0	100.0	1,833	1.04	1.00
25-29	9.1	32.4	40.4	14.0	3.4	0.7	0.1	0.1	0.0	0.0	0.0	100.0	2,249	1.73	1.67
30-34	3.3	14.3	38.6	26.7	11.5	4.1	1.3	0.2	0.1	0.1	0.0	100.0	2,625	2.48	2.36
35-39	3.4	5.5	22.9	25.7	22.7	10.9	4.4	3.3	0.8	0.3	0.0	100.0	1,573	3.33	3.06
40-44	2.3	3.9	16.4	23.0	21.3	14.4	10.3	4.9	1.5	0.8	1.1	100.0	1,673	3.90	3.52
45-49	2.4	4.4	10.0	17.3	19.0	16.4	13.7	7.5	5.1	1.8	2.5	100.0	1,495	4.53	3.91
Total	9.1	21.0	26.1	17.7	11.6	6.6	4.0	2.1	1.0	0.4	0.5	100.0	11,898	2.61	2.40

The same pattern is observed for currently married women, except that the mean number of children ever born is higher for currently married women (2.6 children) than for all women (1.9 children). The difference between all women and currently married women in mean number of children ever born is due to the substantial proportion of young and unmarried women in the all-women category who exhibit lower fertility. For example, only 7 percent of teenage women overall have given birth to a child, whereas 43 percent of currently married teenage women have begun childbearing.

As would be expected, the mean number of children ever born and the mean number of children surviving rise monotonically as age increases. A comparison of the mean number of children ever born with the mean number of living children reveals the experience of child loss among Cambodian women. By the end of their reproductive years (age 45-49), married women in Cambodia have given birth, on average, to 4.5 children, with 3.9 surviving.

Voluntary childlessness is not common in Cambodia, and currently married women with no children are likely to be those who are unable to bear children (primary infertility). Whereas 57 percent of currently married adolescent women are childless, this proportion decreases to 9 percent among currently married women age 25-29 and continues to decline with increasing age. The percentage of childless women among currently married women at the end of the reproductive period (age 45-49) shows that primary infertility among currently married women is low (2 percent).

6.4 BIRTH INTERVALS

Longer birth intervals contribute to improved health status of both mother and child (Rutstein, 2005). Infants born within two years of the birth of a previous child experience a higher risk of health problems. Table 6.5 shows the distribution of second- and higher-order births that occurred in the five years preceding the survey by the number of months since the previous birth, according to background characteristics.

Table 6.5 Birth intervals

Percent distribution of non-first births in the five years preceding the survey by number of months since preceding birth, and median number of months since preceding birth, according to background characteristics, Cambodia 2014

Background characteristic	Months since preceding birth						Total	Number of non-first births	Median number of months since preceding birth
	7-17	18-23	24-35	36-47	48-59	60+			
**Age**									
15-19	*	*	*	*	*	*	100.0	17	*
20-29	6.8	12.2	28.6	23.7	13.2	15.3	100.0	1,807	37.1
30-39	4.0	5.5	20.8	17.4	15.2	37.1	100.0	2,233	49.6
40-49	4.3	3.1	16.2	11.7	11.1	53.6	100.0	374	63.6
**Sex of preceding birth**									
Male	5.5	8.0	20.9	20.8	13.7	31.1	100.0	2,306	44.5
Female	4.9	8.2	26.5	18.3	14.4	27.7	100.0	2,124	42.7
**Survival of preceding birth**									
Living	4.2	7.9	23.2	20.1	14.2	30.4	100.0	4,189	44.5
Dead	22.5	12.4	31.2	9.8	11.1	13.1	100.0	242	30.6
**Birth order**									
2-3	5.1	8.3	23.8	20.8	14.4	27.6	100.0	3,274	43.4
4-6	4.0	7.7	22.0	15.9	13.2	37.1	100.0	971	48.3
7+	13.8	5.8	29.7	17.4	11.2	22.2	100.0	186	36.3
**Residence**									
Urban	5.1	8.2	21.1	18.9	12.8	33.9	100.0	616	45.7
Rural	5.2	8.1	24.0	19.7	14.2	28.8	100.0	3,815	43.5
**Province**									
Banteay Meanchey	7.2	8.4	15.7	14.9	14.5	39.3	100.0	129	50.5
Kampong Cham	5.2	5.1	24.6	21.5	14.3	29.3	100.0	637	44.9
Kampong Chhnang	4.1	9.6	25.6	18.0	18.5	24.1	100.0	153	41.6
Kampong Speu	3.8	9.2	23.4	22.8	13.4	27.4	100.0	265	43.9
Kampong Thom	5.7	5.8	23.3	17.3	16.5	31.3	100.0	217	45.7
Kandal	5.7	7.2	26.3	21.1	13.4	26.3	100.0	339	42.9
Kratie	2.5	11.8	30.0	18.0	11.2	26.6	100.0	177	37.8
Phnom Penh	4.1	7.8	22.0	17.8	12.9	35.3	100.0	370	46.5
Prey Veng	4.3	6.2	24.4	15.2	13.4	36.5	100.0	284	47.9
Pursat	2.9	8.1	29.2	19.0	19.1	21.6	100.0	191	41.3
Siem Reap	7.6	13.5	25.1	18.8	11.1	23.9	100.0	341	38.6
Svay Rieng	2.4	5.2	14.7	22.2	19.0	36.5	100.0	140	51.1
Takeo	5.4	10.4	19.9	16.9	17.8	29.6	100.0	228	44.6
Otdar Meanchey	3.0	8.7	21.0	19.8	16.6	30.9	100.0	86	45.7
Battambang/Pailin	7.7	5.4	18.7	26.9	12.2	29.2	100.0	313	43.5
Kampot/Kep	2.8	8.0	25.6	16.8	12.1	34.7	100.0	156	46.2
Preah Sihanouk/Koh Kong	6.3	8.3	21.8	17.2	14.8	31.7	100.0	106	46.2
Preah Vihear/Stung Treng	8.8	9.7	23.9	22.9	10.4	24.3	100.0	152	38.7
Mondul Kiri/Ratanak Kiri	6.2	13.6	28.3	15.0	11.4	25.5	100.0	148	36.9
**Education**									
No education	6.1	8.2	26.3	15.8	13.0	30.5	100.0	791	41.4
Primary	5.4	7.9	22.7	19.2	14.0	30.8	100.0	2,518	44.5
Secondary and higher	4.0	8.5	23.7	23.1	14.9	25.8	100.0	1,122	43.4
**Wealth quintile**									
Lowest	7.1	8.6	27.2	20.4	14.4	22.4	100.0	1,214	38.7
Second	3.9	8.4	25.3	17.0	14.4	31.0	100.0	930	44.4
Middle	4.7	9.0	23.2	20.3	12.5	30.3	100.0	772	43.4
Fourth	4.8	8.3	19.4	18.8	16.2	32.6	100.0	681	47.0
Highest	4.5	6.1	20.5	21.2	12.8	35.0	100.0	834	47.1
Total	5.2	8.1	23.6	19.6	14.0	29.5	100.0	4,431	43.8

Note: First-order births are excluded. The interval for multiple births is the number of months since the preceding pregnancy that ended in a live birth. An asterisk indicates that a figure is based on fewer than 25 unweighted cases and has been suppressed.

In 2014, 13 percent of non-first births in Cambodia occurred less than 24 months after the preceding birth (as compared with 16 percent in 2010 and 18 percent in 2005), with 5 percent occurring less than 18 months after the preceding birth. Sixty-three percent of women gave birth at least 36 months after the previous birth, an improvement over the figure from the 2010 CDHS (58 percent). The overall median birth interval was 43.8 months. This means that half of the births in Cambodia occur within 43.8 months of the previous birth, and half occur after an interval of 43.8 months or longer.

The data also indicate that median birth intervals increase as age increases, from 37.1 months among women age 20-29 to 63.6 months among women age 40 and above. Birth intervals do not vary appreciably by sex of the preceding child or urban-rural residence. However, birth intervals vary markedly by the survival status of the preceding birth: 23 percent of births occur within an 18-month interval when the preceding child has died, as compared with 4 percent when the child is still alive. The median birth interval is 44.5 months if the previous child is living but falls to 30.6 months if the preceding child died. Median birth intervals are shortest in Kratie (37.8 months) and Mondul Kiri/Ratanak Kiri (36.9 months) and significantly longer in Banteay Meanchey (50.5 months) and Svay Rieng (51.1 months). Mothers with more education have slightly longer birth intervals: those with no education have a median birth interval of 41.4 months, whereas those with a primary education have a median birth interval of 44.5 months and those with a secondary education or higher have a median birth interval of 43.4 months. The median birth interval is shortest among women in the lowest wealth quintile (38.7 months) and longest among those in the highest wealth quintile (47.1 months).

6.5 Age at First Birth

Early age at childbearing has a detrimental effect on the health of both mother and child. It also frequently leads to a longer reproductive span and a higher level of fertility. Table 6.6 shows the percentage of women age 15-49 who have given birth by exact ages, the percentage who have never given birth, and the median age at first birth, according to current age. The youngest cohort of women for whom median age at first birth can be calculated is 25-29 years. The medians age for women in the 15-19 and 20-24 age groups cannot be determined because fewer than half of these women had a birth before reaching the lowest age of the age group.

Table 6.6 Age at first birth

Percentage of women age 15-49 who gave birth by exact ages, percentage who have never given birth, and median age at first birth, according to current age, Cambodia 2014

Current age	Percentage who gave birth by exact age: 15	18	20	22	25	Percentage who have never given birth	Number of women	Median age at first birth
15-19	0.2	na	na	na	na	92.7	2,893	a
20-24	0.4	7.0	24.1	na	na	50.6	3,017	a
25-29	0.5	6.9	21.9	41.1	65.4	23.0	2,836	22.9
30-34	0.5	10.3	26.4	44.5	66.0	11.1	3,046	22.7
35-39	0.8	13.3	33.3	51.9	73.6	8.7	1,839	21.8
40-44	0.9	11.7	32.0	53.4	75.5	7.1	2,030	21.7
45-49	1.6	12.6	27.7	46.5	69.2	6.8	1,916	22.4
25-49	0.8	10.5	27.6	46.7	69.2	12.2	11,668	22.4

na = Not applicable due to censoring
a = Omitted because less than 50 percent of women had a birth before reaching the beginning of the age group

Whereas less than 1 percent of women in the 25-49 age group had given birth by age 15, 11 percent had given birth by age 18 and 28 percent by age 20. The percentage who had given birth by age 18 does not vary much between ages 30 and 49, but it is slightly lower among the youngest cohorts (7 percent), implying a trend toward postponement of childbearing. Median age at first birth ranged from 21.7 to 22.9 years across the age groups, with no discernible pattern of variation.

Table 6.7 presents the median age at first birth by background characteristics and age at the time of the survey. The median age at first birth (22.4 for all women age 25-49) is higher in urban areas than in rural areas, with a difference of 1.5 years among women age 25-49. Phnom Penh has the highest median age at first birth (23.9), and Mondul Kiri/Ratanak Kiri has the lowest (20.9). There is a positive relationship between educational attainment and median age at first birth. Women with no formal education have a lower median age at first birth (21.3) than women with a primary education (22.0) and those with a secondary education or higher (23.7). There is no clear pattern in median age at first birth by wealth quintile.

Table 6.7 Median age at first birth

Median age at first birth among women age 25-49, according to background characteristics, Cambodia 2014

Background characteristic	Age					Women age 25-49
	25-29	30-34	35-39	40-44	45-49	
**Residence**						
Urban	a	24.2	22.1	22.4	22.9	23.6
Rural	22.5	22.4	21.8	21.6	22.3	22.1
**Province**						
Banteay Meanchey	22.5	22.8	20.9	21.4	21.9	22.0
Kampong Cham	22.4	21.8	21.9	21.6	22.4	22.0
Kampong Chhnang	24.1	22.6	22.8	22.7	22.5	22.9
Kampong Speu	21.9	21.8	20.0	21.4	22.9	21.6
Kampong Thom	22.8	22.4	22.3	20.8	22.2	22.2
Kandal	22.9	23.8	23.7	21.9	23.7	23.2
Kratie	21.6	23.1	21.0	21.6	22.6	22.0
Phnom Penh	a	24.6	22.3	22.5	23.1	23.9
Prey Veng	22.5	22.4	21.0	20.9	21.6	21.7
Pursat	23.4	21.8	21.3	22.3	24.2	22.6
Siem Reap	21.6	22.2	21.8	22.3	23.1	22.2
Svay Rieng	22.7	22.0	21.8	21.4	22.9	22.1
Takeo	23.5	22.6	23.0	21.1	21.6	22.6
Otdar Meanchey	21.9	22.7	21.7	20.6	21.4	21.8
Battambang/Pailin	23.6	23.3	21.9	22.0	21.4	22.5
Kampot/Kep	21.6	21.6	20.9	21.8	22.1	21.7
Preah Sihanouk/Koh Kong	23.4	21.9	20.5	21.2	21.8	22.0
Preah Vihear/Stung Treng	22.3	21.6	21.1	21.5	21.3	21.6
Mondul Kiri/Ratanak Kiri	21.4	20.4	21.2	20.6	21.1	20.9
**Education**						
No education	21.4	21.7	20.9	21.1	21.5	21.3
Primary	22.0	22.3	21.8	21.6	22.2	22.0
Secondary and higher	24.6	24.3	22.3	22.3	23.7	23.7
**Wealth quintile**						
Lowest	22.0	22.0	21.7	22.0	23.4	22.2
Second	21.5	22.2	21.6	21.6	21.9	21.7
Middle	22.6	21.9	21.6	21.3	22.0	21.9
Fourth	23.6	22.8	21.7	21.2	22.2	22.4
Highest	a	23.8	22.5	22.5	22.6	23.5
Total	22.9	22.7	21.8	21.7	22.4	22.4

a = Omitted because less than 50 percent of the women had a birth before reaching the beginning of the age group

6.6 TEENAGE PREGNANCY AND MOTHERHOOD

Teenage fertility is a major health concern because teenage mothers and their children are at high risk of illness and death. Childbearing during the teenage years can have dire social consequences as well, curtailing the educational and employment opportunities of women. Early initiation into childbearing is also often associated with higher lifetime levels of fertility. Table 6.8 presents the proportion of women age 15-19 (teenagers) who are mothers or pregnant with their first child, by background characteristics.

Approximately 1 in 8 women (12 percent) age 15-19 have become mothers or are currently pregnant with their first child. The percentage of women who have begun childbearing at age 15-19 provides further evidence of a sharp increase in teenage fertility in recent years. The level of teenage fertility was relatively stable from 2000 to 2010 at 8 percent.

The percentage of women who have begun childbearing increases with age, from less than 1 percent among women age 15 to 31 percent among women age 19. Six percent of urban women begin childbearing in their teens, as do 13 percent of rural women. The level of teenage fertility is strongly associated with education. More than one-third of teenagers (37 percent) who have never been to school have begun childbearing, as compared with 18 percent who have a primary school education and 8 percent who have a secondary education or higher. The level of teenage fertility is also strongly associated with wealth: 18 percent of the poorest teenagers have begun childbearing, as compared with only 7 percent of the richest. The percentage of teenagers who have begun childbearing varies greatly among provinces, with the lowest in Battambang/Pailin (4 percent) and the highest in Mondul Kiri/Ratanak Kiri (34 percent).

Table 6.8 Teenage pregnancy and motherhood

Percentage of women age 15-19 who have had a live birth or who are pregnant with their first child, and percentage who have begun childbearing, by background characteristics, Cambodia 2014

Background characteristic	Percentage of women age 15-19 who: Have had a live birth	Are pregnant with first child	Percentage who have begun childbearing	Number of women
**Age**				
15	0.2	0.4	0.6	640
16	1.4	2.4	3.8	556
17	3.5	4.4	7.9	577
18	10.0	8.4	18.4	577
19	23.2	8.2	31.3	542
**Residence**				
Urban	4.3	1.9	6.2	532
Rural	8.0	5.2	13.3	2,361
**Province**				
Banteay Meanchey	16.5	2.5	18.9	113
Kampong Cham	13.1	2.9	16.1	327
Kampong Chhnang	6.3	2.9	9.1	129
Kampong Speu	2.0	6.2	8.2	202
Kampong Thom	4.1	4.4	8.6	156
Kandal	8.8	3.1	11.9	225
Kratie	14.5	5.0	19.5	80
Phnom Penh	3.8	2.1	5.9	316
Prey Veng	6.7	4.2	10.9	141
Pursat	3.8	4.8	8.6	100
Siem Reap	4.8	10.5	15.3	191
Svay Rieng	6.5	4.4	10.9	73
Takeo	2.5	5.2	7.8	193
Otdar Meanchey	11.0	5.8	16.8	50
Battambang/Pailin	3.1	1.2	4.3	217
Kampot/Kep	7.3	4.3	11.6	114
Preah Sihanouk/Koh Kong	7.5	5.5	12.9	88
Preah Vihear/Stung Treng	12.1	13.1	25.1	102
Mondul Kiri/Ratanak Kiri	23.2	10.6	33.8	75
**Education**				
No education	25.5	11.6	37.1	82
Primary	11.8	6.7	18.4	852
Secondary and higher	4.7	3.5	8.1	1,959
**Wealth quintile**				
Lowest	11.2	7.0	18.1	458
Second	9.8	5.1	14.9	552
Middle	7.5	6.2	13.8	578
Fourth	4.9	3.7	8.6	630
Highest	4.9	2.2	7.1	675
Total	7.3	4.6	12.0	2,893

PRACTICE OF ABORTION 7

Key Findings

- Twelve percent of women have had at least one abortion in their lifetime, and 7 percent have had an abortion in the past five years.
- Among those who have had an abortion in the past five years, 53 percent have had it within the first two months of pregnancy.
- Forty-four percent of abortions take place in a private health facility and 40 percent occur in the respondent's or someone else's home.
- Sixty-one percent of abortions were assisted by a health care professional. However, 30 percent of women did not receive any assistance.

In many countries in the developing world, there are very few data on the practice of abortion. It is illegal in a number of countries, often has negative social connotations, and is often considered against religious principles. The practice of abortion was legalized in Cambodia in 1997. According to the 1997 law, abortions can be conducted only by medical doctors, medical practitioners, or midwives authorized by the Ministry of Health and can be carried out only in a hospital, health center, health clinic, or maternity ward. In addition, abortions can be legally conducted only before the 12th week of pregnancy unless one of a number of specific conditions that permit later abortions is met (The World Law Guide, accessed on May 23, 2011).

In order to better understand the practice of abortion in Cambodia, questions on the practice were integrated into the reproductive section of the CDHS Woman's Questionnaire. The results in this chapter present an estimation of the frequency of abortion in the past five years. Information was collected on the person who performed the abortion, the pregnancy duration, the place where the abortion took place, and the persons who assisted with the abortion. Pregnancy outside of marriage is not socially acceptable in Cambodia, and thus it is likely that not all women who have had an abortion will be willing to report having done so. As a result, abortion statistics are likely underestimates of the true level of abortion.

7.1 NUMBER OF LIFETIME INDUCED ABORTIONS

Table 7.1 presents the percent distribution of all women age 15 to 49 by the number of induced abortions they have had over their lifetime according to background characteristics. In Cambodia, 12 percent of women age 15 to 49 reported having had one or more abortions in their lifetime.

There is a reverse U-shaped association between abortion and age. The percentage of women who have had at least one abortion increases sharply from less than 1 percent at age 15-19 to a peak of 21 percent at age 35-39 before declining to 20 percent at age 40-44 and 16 percent at age 45-49. The likelihood that a woman has had an abortion increases with number of living children. Less than 1 percent of women with no children and 9 percent of women with one child reported ever having had an abortion. Sixteen percent of women with two children and 22-24 percent of women with three to four children have had at least one abortion. The proportion declines to 20 percent among women with five or more children.

The practice of abortion varies slightly by urban-rural residence (17 percent in urban areas versus 11 percent in rural areas). The percentage of women who have had an abortion varies across provinces as well. The highest percentages are observed among women living in Phnom Penh (19 percent) and Banteay Meanchey (18 percent). By contrast, only 3 percent of women in Mondul Kiri/Ratanak Kiri reported having had an abortion. Less than 1 in 10 women (9 percent) with a secondary education or higher reported ever having had an abortion, as compared with 15 percent of women with a primary education and 12 percent of women with no education. The practice of abortion occurs mostly among ever-married women.

Table 7.1 Number of induced abortions

Percent distribution of women by number of induced abortions during their lifetime, according to background characteristics, Cambodia 2014

Background characteristic	Number of abortions						Total	Number of women
	None	1	2	3	4+	Missing		
**Age**								
15-19	99.2	0.6	0.2	0.0	0.0	0.0	100.0	2,893
20-24	94.6	4.8	0.4	0.1	0.1	0.0	100.0	3,017
25-29	87.4	10.3	1.6	0.5	0.1	0.1	100.0	2,836
30-34	82.6	12.6	3.2	0.7	0.6	0.2	100.0	3,046
35-39	78.8	14.3	4.4	1.1	1.4	0.0	100.0	1,839
40-44	80.4	12.3	3.3	2.5	1.6	0.0	100.0	2,030
45-49	84.0	9.4	3.3	1.5	1.8	0.0	100.0	1,916
**Number of living children**								
0	99.0	0.8	0.1	0.0	0.0	0.0	100.0	5,235
1	90.4	7.8	1.1	0.3	0.2	0.1	100.0	3,236
2	83.5	12.4	2.7	0.9	0.4	0.1	100.0	3,726
3	78.0	14.3	4.8	1.4	1.5	0.0	100.0	2,477
4	76.1	17.0	3.5	2.0	1.4	0.1	100.0	1,519
5	79.6	12.6	3.9	1.8	2.0	0.1	100.0	773
6+	80.3	10.5	3.6	2.6	2.9	0.0	100.0	611
**Residence**								
Urban	82.5	10.3	3.8	1.6	1.7	0.0	100.0	3,251
Rural	88.8	8.3	1.7	0.6	0.4	0.1	100.0	14,327
**Province**								
Banteay Meanchey	82.3	12.9	2.5	0.8	1.3	0.2	100.0	689
Kampong Cham	87.7	9.8	1.3	0.7	0.6	0.0	100.0	2,021
Kampong Chhnang	90.5	7.2	1.4	0.5	0.3	0.0	100.0	662
Kampong Speu	91.5	6.3	1.2	0.5	0.5	0.0	100.0	1,196
Kampong Thom	93.2	5.0	1.2	0.3	0.3	0.0	100.0	851
Kandal	86.0	10.7	2.0	1.1	0.0	0.2	100.0	1,330
Kratie	89.0	8.2	1.6	0.4	0.8	0.0	100.0	488
Phnom Penh	80.6	10.6	5.0	1.8	2.0	0.0	100.0	1,994
Prey Veng	94.2	4.7	0.6	0.2	0.3	0.0	100.0	1,188
Pursat	93.8	5.2	0.7	0.0	0.4	0.0	100.0	631
Siem Reap	85.5	10.2	2.5	1.0	0.6	0.2	100.0	1,137
Svay Rieng	88.1	8.0	2.4	1.0	0.4	0.0	100.0	654
Takeo	87.8	7.9	3.0	1.0	0.3	0.0	100.0	1,082
Otdar Meanchey	92.8	6.0	0.9	0.1	0.0	0.0	100.0	294
Battambang/Pailin	83.9	10.6	3.0	1.2	1.2	0.0	100.0	1,333
Kampot/Kep	85.5	12.2	1.5	0.6	0.1	0.0	100.0	770
Preah Sihanouk/Koh Kong	83.5	11.7	2.0	1.4	1.4	0.0	100.0	422
Preah Vihear/Stung Treng	91.8	6.5	0.8	0.1	0.4	0.4	100.0	462
Mondul Kiri/Ratanak Kiri	97.3	2.2	0.4	0.0	0.0	0.1	100.0	372
**Education**								
No education	87.6	8.5	2.3	0.8	0.7	0.0	100.0	2,250
Primary	84.9	10.9	2.3	1.0	0.9	0.1	100.0	8,281
Secondary and higher	91.0	6.2	1.8	0.6	0.4	0.0	100.0	7,047
**Marital status**								
Never married	99.8	0.2	0.0	0.0	0.0	0.0	100.0	4,428
Ever married	83.6	11.6	2.8	1.1	0.9	0.1	99.9	13,150
Total	87.7	8.7	2.1	0.8	0.7	0.1	100.0	17,578

Figure 7.1 presents the distribution of women who report having at least one abortion in their lifetime according to the number of abortions they have had. The majority of women who have had an abortion have had only one (74 percent). Seventeen percent of women who have had an abortion report having had two abortions, and 10 percent of women who have had an abortion report having had three or more induced abortions.

Figure 7.1 Distribution of women who have had an abortion by number of abortions

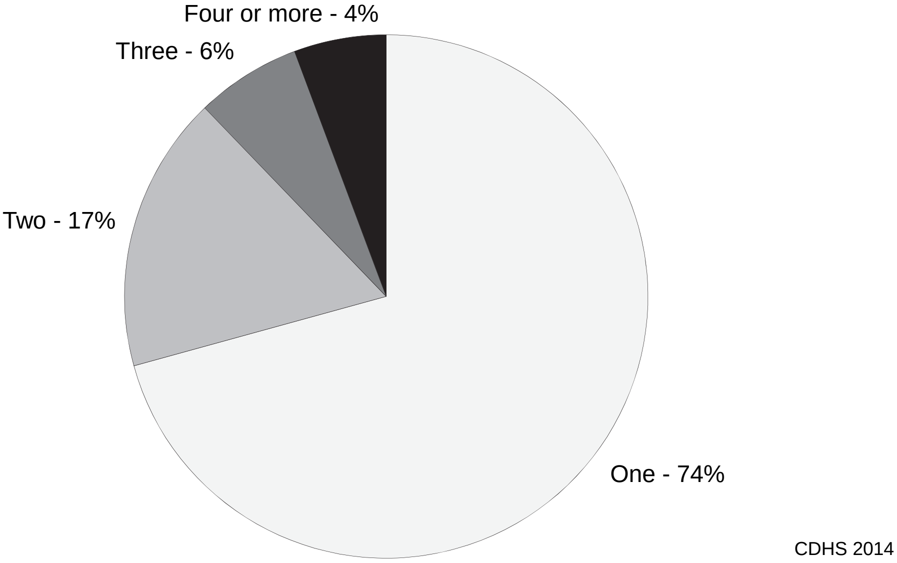

7.2 PRACTICE OF ABORTION IN THE PAST FIVE YEARS

In order to obtain information on the recent practice of abortion, detailed questions concerning abortion were asked to those women who had had an abortion since 2009. In Table 7.2 and subsequent tables, education of the respondent has been grouped into two categories, no schooling and primary education or higher, due to the relatively small number of cases.

Table 7.2 shows that 7 percent of women had an induced abortion in the five years before the survey. This represents an increase from the figure reported in the 2010 CDHS (5 percent).

7.2.1 Pregnancy Duration at the Time of Abortion

Table 7.2 also shows the percentage of women who reported having an abortion in the past five years by their pregnancy duration at the time of abortion. Slightly more than half of these women (53 percent) aborted their pregnancy within the first two months of pregnancy, and 46 percent had the abortion between the second and fourth months of pregnancy.

Women with three or four living children were more likely than other women to have had an abortion (11 percent), and approximately half of these women (53 percent) had their abortion within the first two months of pregnancy. Sixty-nine percent of women with five or more living children had their abortion after the second month of pregnancy. The percentage of women who recently had an abortion also varies by urban-rural residence; urban women are more likely to have had a recent abortion than rural women (10 percent versus 6 percent). Although the likelihood of having a recent abortion did not vary by level of education, there were differences according to education in the duration of pregnancy at the time of the abortion. Women with a primary education or higher were more likely than women with no schooling to have had their abortion within the first two months of pregnancy (55 percent versus 42 percent). In contrast, the percentage of women with no schooling who had their abortion after the second month of pregnancy is higher than that among women with at least a primary education (58 percent versus 45 percent).

Table 7.2 Pregnancy duration at the time of abortion

Percentage of women who had at least one induced abortion and percent distribution of the last termination that was an abortion during the past five years by pregnancy duration at the time of the abortion, according to background characteristics, Cambodia 2014

Background characteristic	Percentage with at least one abortion since January 2009	Number of women	Pregnancy duration at the time of last abortion			Total	Number of women whose last termination was an abortion
			<2 months	2-4 months	5+ months		
**Current age**							
15-24	3.1	5,910	55.1	44.0	0.9	100.0	166
25-34	10.2	5,882	54.7	43.4	1.9	100.0	532
35-49	7.5	5,786	49.6	49.7	0.7	100.0	373
**Number of living children[1]**							
0-2	5.5	12,198	56.6	41.7	1.7	100.0	598
3-4	10.7	3,996	52.8	46.1	1.1	100.0	377
5+	8.2	1,384	30.8	69.2	0.0	100.0	95
**Residence**							
Urban	9.7	3,251	61.5	36.8	1.6	100.0	282
Rural	6.3	14,327	50.0	48.8	1.2	100.0	789
**Education**							
No education	6.9	2,250	41.6	57.4	1.0	100.0	136
Primary and higher	6.9	15,328	54.7	44.0	1.4	100.0	934
Total	6.9	17,578	53.0	45.7	1.3	100.0	1,070

[1] Including current pregnancy

7.2.2 Place of Abortion

Women who had an abortion in the five years before the survey were asked where the most recent abortion took place (Table 7.3). The proportion of women who had their abortion in a health facility (60 percent) was similar to the figure reported in the 2010 CDHS (57 percent). Of facility-based abortions, the majority occur in a private facility. Thirty-two percent of abortions took place in the respondent's home, and 8 percent took place in someone else's home. These figures show that abortions are more likely to have taken place in a health facility than at home.

Among women who had an abortion in the five years before the survey, the percentage who had an abortion in a health facility is slightly higher among those in urban areas than among those in rural areas (64 percent and 58 percent, respectively). However, the percentage of women who had an abortion in a health facility does not differ markedly by education (58 percent among those with no schooling versus 60 percent among those with at least a primary education).

Table 7.3 Place of abortion

Percent distribution of the last termination that was an abortion during the five years before the survey by place of abortion, according to background characteristics, Cambodia 2014

Background characteristic	Place of abortion				Total	Number of women whose last termination was an abortion
	Public health facility	Private health facility	Respondent home	Other home		
**Current age**						
15-34	13.4	44.8	35.4	6.4	100.0	697
35-49	19.4	43.2	26.3	11.2	100.0	373
**Pregnancy duration at the time of last abortion**						
<2 months	12.6	45.5	36.8	5.1	100.0	567
2-4 months	18.7	42.7	27.2	11.3	100.0	489
5+ months	20.0	49.0	20.5	10.5	100.0	14
**Residence**						
Urban	18.2	45.5	29.1	7.2	100.0	282
Rural	14.5	43.8	33.3	8.4	100.0	789
**Education**						
No education	18.5	39.3	34.6	7.5	100.0	136
Primary and higher	15.0	45.0	31.9	8.1	100.0	934
Total	15.5	44.3	32.2	8.0	100.0	1,070

7.2.3 Persons Who Helped with the Abortion

Women who had an abortion in the five years before the survey were asked to identify the type of person or persons who assisted their last abortion. If more than one person assisted with the abortion, only the most qualified person is reported in Table 7.4. The proportion of women receiving help from a qualified health care provider (doctor, nurse, midwife, and/or other health worker) has continued to decline over recent years, from 79 percent in 2005 to 67 percent in 2010 and only 61 percent in 2014. In contrast, the percentage of women who report having no help from anyone has increased in the past few years (from 8 percent in 2005 to 22 percent in 2010 and 30 percent in 2014). Approximately 8 percent of women received help from a relative or friend and 1 percent from a traditional birth attendant, Kru Khmer, or pharmacist.

Women age 15-34 were less likely to seek assistance from a qualified provider than older women (59 percent versus 67 percent). Moreover, women who had their abortion at the early stage of pregnancy (before 2 months) were more likely to have had no help than were those who were 2-4 months pregnant at the time of their abortion (37 percent versus 23 percent). More late-stage abortions (2-4 months) involved assistance from a health professional (67 percent) than early-stage abortions (before 2 months). There were no substantial variations in assistance at abortion by urban-rural residence. The percentage of women who received help from a relative or friend during their last abortion differed by education (13 percent among women with no schooling versus 7 percent among women with a primary education or higher).

Table 7.4 Persons who helped with abortion

Percent distribution of the last termination that was an abortion during the five years before the survey by the most qualified person who helped with the abortion, according to background characteristics, Cambodia 2014

Background characteristic	Person who helped with last abortion: Doctor/nurse/ midwife/other health worker	Traditional birth attendant/ Kru Khmer/ pharmacist	Relative/friend/ other	No one	Total	Number of women whose last termination was an abortion
**Current age**						
15-34	58.5	0.6	8.6	32.3	100.0	697
35-49	67.0	1.5	5.8	25.8	100.0	373
**Pregnancy duration at the time of last abortion**						
<2 months	56.5	1.3	5.1	37.2	100.0	567
2-4 months	66.6	0.4	10.3	22.6	100.0	489
5+ months	*	*	*	*	100.0	14
**Residence**						
Urban	63.4	0.9	5.7	30.0	100.0	282
Rural	60.7	0.9	8.3	30.1	100.0	789
**Education**						
No education	56.6	0.0	13.0	30.4	100.0	136
Primary and higher	62.1	1.0	6.8	30.0	100.0	934
Total	61.4	0.9	7.6	30.1	100.0	1,070

Note: An asterisk indicates that a figure is based on fewer than 25 unweighted cases and had been suppressed.

7.2.4 Method Used for the Abortion

Women who had an abortion in the five years before the survey were asked about the methods they used to induce the last abortion. The percentages shown in Table 7.5 can sum to more than 100 percent because women could list more than one method.

Three in five women (60 percent) who had an abortion in the five years before the survey used a surgical method to induce abortion, whereas 47 percent used a medical method. The majority of women (57 percent) used vacuum aspiration, a surgical method. Oral pill/tablet was the second most popular method, used by 42 percent of women. Only 6 percent of women used the curettage method, and only 4 percent used the dilatation and evacuation method.

There were no substantial differences in the method of abortion according to women's residence or level of education. However, there were slight variations by age and duration of pregnancy. Women age 35-49 were more likely to use a surgical method than women age 15-24 and 25-34 (64 percent versus 54 percent and 59 percent, respectively). In contrast, the proportion of younger women (15-24 and 25-39) who used a medical method for their last abortion was slightly higher than the proportion among their older counterparts (35-49) (55 percent and 50 percent, respectively, versus 42 percent). Women who had their abortions at a later stage of pregnancy (2-4 months) were more likely than those who had their abortions at an early stage of pregnancy (before 2 months) to use a surgical method (65 percent versus 56 percent). In contrast, medical methods were used more often during the early stage of pregnancy (51 percent) than during the late stage of pregnancy (42 percent).

Table 7.5 Method used for the abortion

Among women who had an abortion during the five years before the survey, the percentage who used different methods to induce the abortion, according to background characteristics, Cambodia 2014

Background characteristic	Any surgical method	Surgical methods: Vacuum aspiration	Curettage	Dilatation and evacuation	Any medical method	Medical methods: Oral pill/tablet	Vaginal pill/ tablet	Injectable	Intrauterine	Traditional methods	Other methods	Number of women whose last termination was an abortion
**Current age**												
15-24	53.7	50.1	5.9	3.0	54.6	50.8	12.3	3.4	0.0	0.0	0.0	166
25-34	58.8	55.4	6.3	4.3	47.9	43.3	9.9	3.3	0.5	0.0	1.0	532
35-49	64.3	62.2	5.1	2.5	42.3	37.0	8.0	6.0	0.5	0.1	2.6	373
**Pregnancy duration at the time of last abortion**												
<2 months	56.2	54.9	2.8	2.2	50.8	46.9	9.2	3.9	0.0	0.0	0.7	567
2-4 months	65.1	60.8	9.2	4.6	42.2	37.4	10.3	3.6	1.0	0.1	1.8	489
5+ months	*	*	*	*	*	*	*	*	*	*	*	14
**Residence**												
Urban	61.7	57.6	6.3	4.7	43.2	37.5	6.3	2.6	1.5	0.0	0.7	282
Rural	59.3	56.7	5.7	3.1	48.4	43.9	10.8	4.8	0.1	0.1	1.7	789
**Education**												
No education	61.5	58.6	8.0	1.9	53.3	48.4	6.9	9.4	0.0	0.0	0.0	136
Primary and higher	59.7	56.7	5.5	3.7	46.1	41.4	10.0	3.5	0.5	0.1	1.6	934
Total	59.9	57.0	5.8	3.5	47.0	42.3	9.6	4.2	0.4	0.1	1.4	1,070

Note: An asterisk indicates that a figure is based on fewer than 25 unweighted cases and had been suppressed.

FAMILY PLANNING 8

Key Findings

- Awareness of at least one method of contraception is universal in Cambodia.
- More than half (56 percent) of currently married women are using a method of contraception, with most women using a modern method (39 percent).
- The daily pill remains the most commonly used method of contraception among currently married women (18 percent).
- Use of modern methods of family planning has consistently increased over the past decade, from 19 percent of currently married women in 2000 to 39 percent in 2014.
- The government sector remains the major provider of contraceptive methods for nearly half of the users of modern methods (47 percent).
- Nearly 9 in 10 women (88 percent) who use the rhythm method know correctly when the fertile period occurs.

This chapter presents information from the 2014 CDHS on contraceptive knowledge, attitudes, and behavior. Comparisons are also made, where appropriate, with findings from the 2010 CDHS to evaluate trends over the past four years.

8.1 KNOWLEDGE OF CONTRACEPTIVE METHODS

Acquiring knowledge about family planning is an important step toward gaining access to and using a suitable contraceptive method in a timely and effective manner. Individuals who have adequate information about the available methods of contraception are better able to make choices about planning their families. Thus, one of the main objectives of the 2014 CDHS was to assess the level of knowledge of family planning methods among women of reproductive age. To collect data on knowledge of contraception, the interviewer described each method and probed for whether the respondent recognized it.

Information was collected on several modern contraceptive methods: female and male sterilization, daily and monthly pills, intrauterine devices (IUDs), injectables, implants, male and female condoms, the lactational amenorrhea method (LAM), and emergency contraception. Information was also collected on two traditional methods: rhythm (or periodic abstinence) and withdrawal. In addition, provision was made in the questionnaire to record any other methods named spontaneously by the respondents.

Table 8.1 presents information about knowledge of contraceptive methods among all women and currently married women age 15-49. Knowledge of any contraceptive method and

Table 8.1 Knowledge of contraceptive methods

Percentage of all women and currently married women who know any contraceptive method, by specific method, Cambodia 2014

Method	All women	Currently married women
Any method	99.2	99.8
Any modern method	99.2	99.8
Female sterilization	90.5	94.1
Male sterilization	69.2	74.7
Daily pill	97.7	99.1
Monthly pill	48.9	54.1
IUD	96.9	98.5
Injectables	97.0	98.9
Implants	93.5	96.3
Male condom	95.0	97.0
Female condom	22.8	23.3
Lactational amenorrhea (LAM)	27.1	31.5
Emergency contraception	16.4	17.0
Any traditional method	70.3	82.9
Rhythm	48.2	55.8
Withdrawal	62.3	76.2
Other	0.6	0.7
Mean number of methods known by respondents 15-49	8.7	9.2
Number of respondents	17,578	11,898

any modern method is nearly universal among both all women and currently married women in Cambodia. Knowledge of traditional methods is lower; 70 percent of all women and 83 percent of currently married women know at least one traditional method. Nearly all of the modern methods are widely known to both all women and currently married women. Over 90 percent of all women and currently married women have heard of female sterilization, the daily pill, IUDs, injectables, implants, and male condoms. However, only 69 percent of women overall and 75 percent of currently married women know about male sterilization. Knowledge of female condoms, LAM, and emergency contraception remains very low among both all women and currently married women. About half of women know about monthly pills (also known as Chinese pills).

The mean number of methods known, a rough indicator of the breadth of knowledge of family planning methods, is high in Cambodia. Breadth of contraceptive knowledge is slightly higher among currently married women (9.2 methods) than all women (8.7 methods).

Knowledge of at least one contraceptive method among all women increased from 92 percent in 2000 to 99 percent in 2005 and has remained at this level over the past nine years. Some of the greatest increases in knowledge in the past four years were in knowledge of male sterilization, implants, and emergency contraception. Knowledge of male sterilization increased from 59 percent to 69 percent among all women and from 65 percent to 75 percent among married women. Knowledge of implants increased from 88 percent to 94 percent among all women and from 91 percent to 96 percent among married women. Finally, knowledge of emergency contraception increased from 10 percent among all women and 11 percent among currently married women to 16 percent among all women and 17 percent among currently married women. However, there has been a decrease in the percentage of women reporting that they know about monthly pills. Knowledge of any traditional method has increased over the same period, especially knowledge of withdrawal.

With practically all currently married women knowing at least one method of contraception, there is very little variation in knowledge by background characteristics (Table 8.2). Knowledge of any method of contraception is slightly lower in Mondul Kiri/Ratanak Kiri, where 97 percent of married women are aware of any method or any modern method of contraception.

Table 8.2 Knowledge of contraceptive methods by background characteristics

Percentage of currently married women age 15-49 who have heard of at least one contraceptive method and who have heard of at least one modern method by background characteristics, Cambodia 2014

Background characteristic	Heard of any method	Heard of any modern method[1]	Number
**Age**			
15-19	98.8	98.8	450
20-24	99.8	99.8	1,833
25-29	99.9	99.8	2,249
30-34	100.0	100.0	2,625
35-39	100.0	100.0	1,573
40-44	99.9	99.9	1,673
45-49	99.5	99.5	1,495
**Residence**			
Urban	99.9	99.9	1,818
Rural	99.8	99.8	10,080
**Province**			
Banteay Meanchey	99.8	99.8	503
Kampong Cham	99.7	99.7	1,490
Kampong Chhnang	100.0	100.0	396
Kampong Speu	99.7	99.7	843
Kampong Thom	100.0	100.0	572
Kandal	100.0	100.0	870
Kratie	99.8	99.8	359
Phnom Penh	100.0	100.0	1,084
Prey Veng	100.0	100.0	889
Pursat	100.0	100.0	425
Siem Reap	100.0	100.0	765
Svay Rieng	100.0	100.0	483
Takeo	99.8	99.8	677
Otdar Meanchey	100.0	100.0	218
Battambang/Pailin	99.8	99.8	890
Kampot/Kep	100.0	100.0	574
Preah Sihanouk/ Koh Kong	99.8	99.8	266
Preah Vihear/ Stung Treng	99.7	99.3	314
Mondul Kiri/ Ratanak Kiri	97.3	97.3	281
**Education**			
No education	99.3	99.3	1,774
Primary	99.9	99.9	6,399
Secondary and higher	99.9	99.9	3,431
**Wealth quintile**			
Lowest	99.7	99.6	2,294
Second	99.7	99.7	2,404
Middle	99.8	99.8	2,365
Fourth	99.9	99.9	2,393
Highest	100.0	100.0	2,443
Total	99.8	99.8	11,898

[1] Female sterilization, male sterilization, daily pills, monthly pills, IUD, injectables, implants, male condom, female condom, diaphragm, foam or jelly, lactational amenorrhea method (LAM), and emergency contraception

8.2 Current Use of Contraceptive Methods

The level of current use of contraceptive methods is one of the indicators most frequently used to assess the success of family planning program activities. It is also widely used as a measure in analyzing the

determinants of fertility. This section focuses on the levels of and differentials in current use of family planning in Cambodia.

Current contraceptive use among all women and currently married women is presented in Table 8.3 by age group. Fifty-six percent of married women are currently using a method of family planning. This includes 39 percent who are using a modern method and 18 percent who are using a traditional method. The most widely used method is the daily pill (18 percent), followed by withdrawal (15 percent) and injectables (9 percent).

Table 8.3 Current use of contraception by age

Percent distribution of all women and currently married women age 15-49 by contraceptive method currently used, according to age, Cambodia 2014

			Modern method											Traditional method					
Age	Any method	Any modern method	Female sterili-zation	Male sterili-zation	Daily pill	Monthly pill	IUD	Injecta-bles	Im-plants	Male condom	Female condom	LAM	Any tradi-tional method	Rhythm	With-drawal	Folk method	Not current-ly using	Total	Number of women
ALL WOMEN																			
15-19	4.6	3.2	0.0	0.0	1.1	0.0	0.3	1.2	0.3	0.1	0.0	0.0	1.4	0.1	1.4	0.0	95.4	100.0	2,893
20-24	29.7	21.4	0.0	0.0	9.9	0.0	2.4	6.2	1.5	1.1	0.0	0.1	8.3	0.9	7.4	0.0	70.3	100.0	3,017
25-29	49.2	34.9	0.8	0.0	17.7	0.1	4.1	7.8	2.2	2.1	0.0	0.1	14.3	1.8	12.3	0.1	50.8	100.0	2,836
30-34	58.8	41.1	2.6	0.0	18.8	0.2	4.8	9.4	2.3	2.8	0.0	0.1	17.7	3.0	14.7	0.0	41.1	100.0	3,046
35-39	57.8	40.9	5.7	0.3	16.8	0.1	5.2	8.7	2.0	2.1	0.0	0.1	16.9	3.0	13.8	0.1	41.9	100.0	1,839
40-44	49.6	32.0	5.4	0.2	13.4	0.1	3.0	7.1	1.4	1.4	0.0	0.0	17.5	4.5	13.0	0.0	50.4	100.0	2,030
45-49	25.1	15.1	3.7	0.0	5.7	0.3	1.4	3.1	0.1	0.8	0.0	0.0	10.0	2.0	7.8	0.2	74.9	100.0	1,916
Total	38.5	26.6	2.2	0.1	11.9	0.1	3.0	6.2	1.5	1.5	0.0	0.0	11.9	2.0	9.8	0.1	61.5	100.0	17,578
CURRENTLY MARRIED WOMEN																			
15-19	29.1	20.2	0.0	0.0	7.2	0.0	2.2	8.0	2.1	0.7	0.0	0.0	8.9	0.4	8.5	0.0	70.9	100.0	450
20-24	47.8	34.4	0.1	0.0	16.4	0.1	3.9	10.1	2.5	1.2	0.0	0.2	13.4	1.2	12.2	0.0	52.2	100.0	1,833
25-29	61.6	43.8	0.9	0.0	22.3	0.1	5.2	9.8	2.8	2.5	0.0	0.1	17.9	2.3	15.4	0.2	38.4	100.0	2,249
30-34	67.9	47.4	2.9	0.0	21.8	0.2	5.5	10.9	2.7	3.3	0.0	0.1	20.5	3.5	17.0	0.0	32.0	100.0	2,625
35-39	66.9	47.1	6.2	0.4	19.5	0.1	6.0	9.9	2.4	2.5	0.0	0.1	19.7	3.5	16.1	0.1	32.8	100.0	1,573
40-44	59.6	38.4	6.2	0.2	16.1	0.2	3.7	8.6	1.7	1.7	0.0	0.0	21.3	5.5	15.8	0.0	40.4	100.0	1,673
45-49	31.3	18.6	4.2	0.0	7.1	0.4	1.8	4.0	0.1	0.9	0.0	0.0	12.7	2.6	10.0	0.1	68.7	100.0	1,495
Total	56.3	38.8	3.0	0.1	17.6	0.2	4.4	9.1	2.2	2.1	0.0	0.1	17.5	3.0	14.5	0.1	43.7	100.0	11,898

Note: If more than one method is used, only the most effective method is considered in this tabulation.
LAM = Lactational amenorrhea method

Use of modern contraceptive methods among currently married women varies by age, rising sharply from 20 percent among women age 15-19 to a peak of 47 percent among women age 30-39 before dropping quickly to 19 percent among women age 45-49. There are also differences by age in the methods used by women. For example, among currently married women, the daily pill is the most commonly used method in all age groups other than 15-19, with slightly more women in this group using withdrawal and injectables than the daily pill. As expected, most of the women who have been sterilized are age 35 or older.

As shown in Table 8.4.1, there are marked differences in use of contraceptives by women's background characteristics. Among those currently married, urban women are more likely than rural women to be using any method of contraception (60 percent versus 56 percent) and any traditional method (27 percent versus 16 percent). Meanwhile, rural women are more likely to use modern methods than urban women (40 percent versus 33 percent), particularly injectables (10 percent versus 3 percent) and daily pills (18 percent versus 13 percent). However, urban women are more likely to use male condoms than rural women (5 percent versus 2 percent). There is also substantial variation in current use by province. Current use of any method among married women is highest in Kampong Speu (65 percent); Phnom Penh (63 percent); Banteay Meanchey, Kandal, and Preah Sihanouk/Koh Kong (61 percent each); and Takeo (60 percent). It is lowest in Preah Vihear/Stung Treng (42 percent).

Contraceptive use is associated with the number of living children a woman has; use of any method is highest among married women with three to four children (66 percent) and lowest among women with no children (12 percent). Current contraceptive use increases with increasing education. Fifty-two percent of married women with no schooling are currently using any method of contraception, as compared with 58 percent of married women with a secondary education or higher. Use of contraception rises in an irregular pattern with increasing wealth; the percentage of currently married women using

contraception ranges from 53 percent in the lowest wealth quintile to 62 percent in the highest quintile, with a slight drop at the middle quintile.

Table 8.4.1 Current use of contraception by background characteristics

Percent distribution of currently married women age 15-49 by contraceptive method currently used, according to background characteristics, Cambodia 2014

		Modern method												Traditional method					
Background characteristic	Any method	Any modern method	Female sterili-zation	Male sterili-zation	Daily pill	Monthly pill	IUD	Injecta-bles	Im-plants	Male condom	Female condom	LAM	Any tradi-tional method	Rhythm	With-drawal	Other	Not current-ly using	Total	Number of women
**Number of living children**																			
0	11.8	4.3	0.2	0.0	2.5	0.0	0.2	0.7	0.4	0.4	0.0	0.0	7.5	0.9	6.5	0.0	88.2	100.0	1,128
1-2	60.4	42.3	1.3	0.1	20.6	0.2	5.0	10.1	2.5	2.5	0.0	0.1	18.0	2.9	15.1	0.1	39.6	100.0	5,942
3-4	65.7	45.2	5.4	0.1	18.8	0.2	5.1	10.3	2.7	2.4	0.0	0.1	20.5	4.1	16.4	0.0	34.1	100.0	3,572
5+	49.9	34.6	6.9	0.1	13.1	0.1	3.6	8.9	0.7	1.0	0.0	0.0	15.4	1.9	13.1	0.3	50.1	100.0	1,257
**Residence**																			
Urban	59.8	32.8	3.7	0.1	13.3	0.3	5.0	2.9	2.3	5.1	0.0	0.0	27.0	7.0	19.9	0.1	40.2	100.0	1,818
Rural	55.6	39.8	2.9	0.1	18.3	0.1	4.3	10.3	2.1	1.6	0.0	0.1	15.8	2.2	13.5	0.1	44.3	100.0	10,080
**Province**																			
Banteay Meanchey	61.4	51.0	4.7	0.1	22.9	0.3	3.6	14.7	2.4	2.2	0.0	0.0	10.4	0.8	9.5	0.0	38.6	100.0	503
Kampong Cham	44.3	27.3	2.3	0.0	11.4	0.1	3.6	5.4	2.7	1.8	0.0	0.0	17.0	3.8	13.2	0.0	55.3	100.0	1,490
Kampong Chhnang	56.0	33.5	2.3	0.0	14.7	0.0	5.8	7.7	1.4	1.7	0.0	0.0	22.5	2.0	20.5	0.0	44.0	100.0	396
Kampong Speu	65.4	41.2	4.5	0.2	25.2	0.0	3.4	5.2	0.3	2.3	0.0	0.2	24.3	0.7	23.6	0.0	34.6	100.0	843
Kampong Thom	58.4	44.1	3.8	0.0	15.5	0.2	5.8	14.9	3.1	0.7	0.0	0.0	14.3	4.7	9.6	0.0	41.6	100.0	572
Kandal	61.0	40.4	2.0	0.1	19.5	0.0	4.5	12.2	0.5	1.6	0.0	0.0	20.6	0.8	19.8	0.0	39.0	100.0	870
Kratie	47.9	30.7	2.5	0.0	11.6	0.0	5.0	8.8	1.7	1.0	0.0	0.0	17.2	2.3	14.4	0.5	52.1	100.0	359
Phnom Penh	63.0	28.5	2.7	0.0	11.8	0.5	5.1	1.5	1.3	5.6	0.0	0.0	34.5	7.9	26.4	0.2	36.7	100.0	1,084
Prey Veng	55.4	41.3	2.5	0.2	19.4	0.0	3.7	11.6	2.7	1.2	0.0	0.0	14.1	1.5	12.5	0.0	44.6	100.0	889
Pursat	51.0	40.1	1.6	0.3	18.8	0.3	5.6	11.1	0.7	1.7	0.0	0.0	11.0	1.5	9.5	0.0	49.0	100.0	425
Siem Reap	59.0	46.5	4.2	0.2	19.5	0.0	5.3	10.2	3.3	3.8	0.0	0.0	12.6	2.4	10.2	0.0	41.0	100.0	765
Svay Rieng	57.9	40.7	3.0	0.0	19.7	0.2	3.9	9.3	2.2	1.7	0.0	0.6	17.2	2.6	14.6	0.0	42.1	100.0	483
Takeo	60.1	48.2	4.8	0.2	19.6	1.0	6.8	10.6	3.3	1.7	0.0	0.2	12.0	4.0	7.9	0.0	39.9	100.0	677
Otdar Meanchey	56.6	49.7	3.6	0.0	24.4	0.0	3.2	15.6	1.5	1.3	0.0	0.1	6.9	0.3	6.4	0.2	43.4	100.0	218
Battambang/Pailin	58.3	40.6	4.2	0.1	19.2	0.0	4.7	8.2	2.6	1.5	0.0	0.0	17.7	4.0	13.2	0.4	41.7	100.0	890
Kampot/Kep	53.8	38.3	1.4	0.0	16.3	0.2	4.9	10.7	3.0	1.7	0.0	0.2	15.5	2.3	13.2	0.0	46.2	100.0	574
Preah Sihanouk/ Koh Kong	60.6	41.5	2.7	0.0	18.8	0.4	5.4	6.5	5.5	2.0	0.0	0.1	19.1	3.4	15.5	0.1	39.4	100.0	266
Preah Vihear/ Stung Treng	42.0	34.9	1.2	0.0	17.8	0.0	0.9	13.2	0.3	1.4	0.0	0.0	7.1	1.4	5.7	0.0	58.0	100.0	314
Mondul Kiri/ Ratanak Kiri	50.0	42.7	2.5	0.0	18.5	0.0	1.3	16.3	3.6	0.5	0.0	0.0	7.3	3.1	4.0	0.2	50.0	100.0	281
**Education**																			
No education	52.0	39.9	4.2	0.0	17.8	0.4	2.7	12.2	1.4	1.2	0.0	0.0	12.1	1.1	10.9	0.2	48.0	100.0	1,774
Primary	56.4	39.7	2.9	0.1	18.5	0.1	4.5	9.6	2.2	1.7	0.0	0.0	16.7	2.0	14.7	0.1	43.5	100.0	6,399
Secondary and higher	58.1	36.7	2.8	0.1	15.8	0.1	5.1	6.9	2.5	3.2	0.0	0.1	21.4	5.6	15.8	0.0	41.9	100.0	3,726
**Wealth quintile**																			
Lowest	52.6	39.4	2.0	0.0	18.9	0.1	3.9	11.9	1.8	0.7	0.0	0.0	13.2	1.1	12.0	0.1	47.3	100.0	2,294
Second	55.4	42.4	2.6	0.1	20.1	0.2	4.4	11.5	1.6	1.8	0.0	0.1	13.0	1.4	11.6	0.0	44.6	100.0	2,404
Middle	53.5	38.2	3.2	0.0	18.6	0.1	3.2	9.3	2.3	1.5	0.0	0.0	15.3	1.9	13.2	0.2	46.4	100.0	2,365
Fourth	57.7	39.2	3.2	0.2	18.1	0.3	4.4	8.6	2.7	1.6	0.0	0.1	18.5	3.0	15.5	0.0	42.3	100.0	2,393
Highest	61.7	34.5	4.1	0.1	12.3	0.2	6.1	4.5	2.4	4.8	0.0	0.0	27.2	7.3	19.8	0.1	38.2	100.0	2,443
Total	56.3	38.8	3.0	0.1	17.6	0.2	4.4	9.1	2.2	2.1	0.0	0.1	17.5	3.0	14.5	0.1	43.7	100.0	11,898

Note: If more than one method is used, only the most effective method is considered in this tabulation.
LAM = Lactational amenorrhea method

Current use of contraceptive method among women in Cambodia continues to increase from the levels reported in the first CDHS in 2000. Since the 2010 CDHS, the proportion of currently married women who are using any method of contraception has increased from 51 percent to 56 percent (Table 8.4.2). The proportion of currently married women using any modern method has increased from 35 percent to 39 percent, and the proportion using any traditional method has increased from 16 percent to 18 percent. In the case of individual methods, the largest increases have been achieved among currently married women using pills (16 percent in 2010 to 18 percent in 2014) and withdrawal (12 percent to 15 percent).

Table 8.4.2 Trends in current use of contraception

Percent distribution of currently married women age 15-49 by contraceptive method currently used, according to CDHS 2000, CDHS 2005, CDHS 2010, and 2014 CDHS

Method	CDHS 2000	CDHS 2005	CDHS 2010	CDHS 2014
**Any method**	**23.8**	**40.0**	**50.5**	**56.3**
**Any modern method**	**18.5**	**27.2**	**34.9**	**38.8**
Female sterilization	1.5	1.7	2.4	3.0
Daily/monthly pill	7.2	12.6	15.7	17.8
IUD	1.3	1.8	3.1	4.4
Injectables	7.4	7.9	10.4	9.1
Male condom	0.9	2.9	2.7	2.1
Implant	0.1	0.2	0.4	2.2
Other modern	0.1	0.1	0.1	0.4
**Any traditional method**	**5.3**	**12.8**	**15.7**	**17.5**
Rhythm	2.7	4.5	3.9	3.0
Withdrawal	2.3	8.3	11.7	14.5
Other traditional	0.1	0.1	0.1	0.1
Not currently using	76.2	60.0	49.5	43.7
Total	100.0	100.0	100.0	100.0
Number of women	9,071	10,087	11,626	11,898

8.3 USE OF SOCIAL MARKETING BRANDS

Current users of daily pills and condoms were asked for the brand name of the pills and condoms they last used. This information is useful in monitoring the success of social marketing programs that promote a specific brand.

Socially marketed contraceptive brands are prevalent in Cambodia. Almost all pill and condom users are using a socially marketed product (95 percent of pill users and 88 percent of condom users). Just over half of daily pill users (52 percent) use the “Srey Pich” brand of pills, and 43 percent use “OK” brand pills. About half of condom users (51 percent) use OK condoms and 37 percent use “Number 1” brand condoms (Table 8.5). Srey Pich pills are more popular among rural women than among urban women (54 percent versus 32 percent among pill users). OK pills, OK condoms, and Number 1 condoms are equally popular among urban and rural women. There are large differences by province in use of the two brands of pills; however, the small number of pill users in some provinces indicates that caution should be exercised in interpreting these results.

Table 8.5 Use of social marketing brand pills and condoms

Percentage of daily pill and condom users age 15-49 using specific social marketing brands, by background characteristics, Cambodia 2014

	Among pill users			Among condom users[1]		
Background characteristic	Percentage using Srey Pich pill	Percentage using OK pill	Number of women using the daily pill	Percentage using Number 1	Percentage using OK condom	Number of women using condoms
**Residence**						
Urban	31.8	43.0	247	37.4	50.0	96
Rural	54.2	43.2	1,850	36.1	51.8	144
**Province**						
Banteay Meanchey	46.8	46.0	114	*	*	10
Kampong Cham	31.8	65.2	166	*	*	24
Kampong Chhnang	73.5	25.5	57	*	*	5
Kampong Speu	80.9	18.2	211	*	*	18
Kampong Thom	33.9	62.6	90	*	*	5
Kandal	48.6	49.0	169	*	*	14
Kratie	41.1	57.8	41	*	*	4
Phnom Penh	35.2	33.7	133	(34.6)	(53.1)	62
Prey Veng	51.1	46.7	173	*	*	10
Pursat	57.7	39.0	81	*	*	7
Siem Reap	43.9	47.8	152	(39.1)	(54.8)	29
Svay Rieng	48.1	51.9	96	*	*	8
Takeo	68.0	27.4	139	*	*	11
Otdar Meanchey	45.7	50.4	53	*	*	3
Battambang/Pailin	51.8	43.8	171	*	*	11
Kampot/Kep	52.7	47.1	94	*	*	8
Preah Sihanouk/ Koh Kong	50.8	37.8	50	*	*	5
Preah Vihear/ Stung Treng	57.6	41.4	56	*	*	4
Mondul Kiri/ Ratanak Kiri	46.1	45.1	50	*	*	1
**Education**						
No education	45.0	53.2	317	*	*	18
Primary	53.4	43.8	1,189	38.8	49.6	107
Secondary and higher	51.3	36.4	591	34.6	50.7	115
**Wealth quintile**						
Lowest	52.1	47.1	434	*	*	14
Second	55.1	43.2	483	(34.5)	(51.8)	36
Middle	60.4	37.9	441	(49.0)	(51.0)	35
Fourth	51.0	46.4	437	(46.4)	(43.0)	39
Highest	33.1	40.5	303	33.5)	51.2	115
Total	51.6	43.2	2,097	36.6	51.1	240

Note: Table excludes pill and condom users who do not know the brand name. Condom use is based on women's reports. Figures in parentheses are based on 25-49 unweighted cases. An asterisk indicates that a figure is based on fewer than 25 unweighted cases and has been suppressed.
[1] Among condom users not also using the pill

8.4 KNOWLEDGE OF FERTILE PERIOD

The successful use of natural family planning methods depends largely on an understanding of when during the menstrual cycle a woman is most likely to conceive. All women in the survey were asked about their knowledge of the fertile period. Specifically, they were asked whether there are certain days between two menstrual periods when a woman is more likely to become pregnant if she has sexual intercourse. Those who said yes were further asked whether this time is just before the period begins, during the period, right after the period ends, or halfway between the two periods.

Table 8.6 shows that 64 percent of women do not know when a woman's fertile period is, and only 21 percent correctly state that the fertile time in a woman's menstrual cycle is halfway between two periods. Knowledge of the fertile period is much higher among women who are users of the rhythm method, 88 percent of whom accurately know the timing of the fertile period. However, 8 percent of rhythm method users report that they don't know when the fertile period is, and an additional 3 percent believe it is right after a woman's period has ended. Thus, slightly more than one-tenth of users of the rhythm method are at risk of unwanted pregnancy.

Table 8.6 Knowledge of fertile period

Percent distribution of women age 15-49 by knowledge of the fertile period during the ovulatory cycle, according to current use of the rhythm method, Cambodia 2014

Perceived fertile period	Users of rhythm method	Nonusers of rhythm method	All women
Just before her menstrual period begins	0.5	1.2	1.2
During her menstrual period	0.0	0.5	0.5
Right after her menstrual period has ended	3.4	6.8	6.7
Halfway between two menstrual periods	87.7	19.1	20.5
No specific time	0.9	7.3	7.2
Don't know	7.5	65.1	63.9
Missing	0.0	0.0	0.0
Total	100.0	100.0	100.0
Number of women	357	17,221	17,578

8.5 Timing of Sterilization

Given the effectiveness of female sterilization as a means of preventing pregnancies, family planning programs should emphasize dissemination of information about this method. Trends in the use of sterilization are of interest, especially trends in women's age at the time of the operation.

In Cambodia, 3 percent of married women of reproductive age rely on sterilization as their method of contraception. Table 8.7 shows the distribution of sterilized women age 15-49 by age group at the time of sterilization and median age at sterilization. The data are disaggregated according to number of years since the operation. Thirty-four percent of women who have been sterilized had the operation at age 30-34, with 24 percent each having the operation at age 25-29 and age 35-39. The median age at sterilization is 31.9, a figure that has not varied substantially over time.

Table 8.7 Timing of sterilization

Percent distribution of sterilized women age 15-49 by age at the time of sterilization and median age at sterilization, according to the number of years since the operation, Cambodia 2014

Years since operation	Age at time of sterilization: <25	25-29	30-34	35-39	40-44	45-49	Total	Number of women	Median age[1]
<2	2.4	20.0	34.7	26.9	13.1	3.0	100.0	56	33.7
2-3	5.6	12.9	39.3	28.6	13.5	0.0	100.0	70	32.8
4-5	4.3	27.5	31.5	25.1	11.6	0.0	100.0	88	30.8
6-7	4.9	25.2	29.9	27.1	12.9	0.0	100.0	75	33.1
8-9	(13.5)	(30.8)	(20.1)	(29.6)	(5.9)	(0.0)	100.0	29	(30.4)
10+	17.3	28.8	43.0	11.0	0.0	0.0	100.0	70	a
Total	7.4	23.8	34.3	24.1	9.9	0.4	100.0	387	31.9

Note: Figures in parentheses are based on 25-49 unweighted cases.
a = Not calculated due to censoring
[1] Median age at sterilization is calculated only for women sterilized before age 40 to avoid problems of censoring.

8.6 Source of Family Planning Methods

Data on sources of modern contraceptives are important for family planning program managers and service providers. Women who reported using a modern method of contraception at the time of the survey were asked where they last obtained the method, and interviewers recorded the name and location of the source. To ensure accuracy in reporting, supervisors and editors verified the type of source from the written response.

Table 8.8 shows that users of modern contraceptives obtain their methods from the public sector more than from the private medical sector (47 percent versus 39 percent). Thirty-nine percent of all modern contraceptive users obtain their methods from public health centers, and 20 percent obtain their methods from a private pharmacy. Approximately 12 percent of women who use contraception obtain their methods from private clinics, 8 percent from a community distributor, and 4 percent from a shop.

Table 8.8 Source of modern contraception methods

Percent distribution of users of modern contraceptive methods age 15-49 by most recent source of method, according to method, Cambodia 2014

Source	Female sterilization	Daily pill	IUD	Injectables	Implants	Male condom	Total[1]
**Public sector**	**75.6**	**35.0**	**53.0**	**64.9**	**48.5**	**17.3**	**47.2**
National hospital (PP)	12.2	0.0	0.7	0.0	0.0	0.0	1.1
Provincial hospital (RH)	36.6	0.1	2.6	0.5	3.2	0.1	3.8
District hospital (RH)	21.5	0.3	5.3	0.5	2.0	1.2	2.9
Health center	4.5	34.2	44.1	62.5	42.0	15.6	38.7
Health post	0.0	0.4	0.0	0.8	0.0	0.0	0.3
Military hospital	0.0	0.0	0.0	0.0	0.0	0.5	0.0
Other public sector	0.7	0.1	0.3	0.7	1.3	0.0	0.4
**Private medical sector**	**22.3**	**42.1**	**34.7**	**32.5**	**48.0**	**64.9**	**39.0**
Private hospital	5.8	2.0	0.7	3.2	4.2	1.3	2.5
Private clinic	16.2	5.2	26.1	12.1	34.1	3.1	11.7
Pharmacy	0.3	32.8	0.0	8.1	0.2	58.7	20.3
Other private medical sector	0.0	2.2	7.9	9.0	9.4	1.7	4.6
**Other sources**	**0.0**	**22.8**	**0.6**	**2.4**	**1.5**	**17.8**	**12.0**
Shop	0.0	6.6	0.0	0.1	0.0	13.4	3.8
Community distributor	0.0	16.0	0.6	2.3	1.5	2.9	8.0
Friend/relative	0.0	0.2	0.0	0.1	0.0	1.5	0.2
Other	1.5	0.0	0.2	0.1	0.7	0.0	0.2
Missing	0.6	0.1	11.5	0.2	1.4	0.0	1.5
Total	100.0	100.0	100.0	100.0	100.0	100.0	100.0
Number of women	387	2,098	530	1,094	257	266	4,663

Note: Total includes 10 users of male sterilization and 21 users of monthly pills.
[1] Includes other modern methods but excludes lactational amenorrhea method (LAM)
PP = Phnom Penh
RH = Referral hospital

There is notable variation in source of method by type of contraceptive. Users of daily pills most often obtain them from a public health center (34 percent), a pharmacy (33 percent), or a community distributor (16 percent). The public sector is the largest source of IUDs. More than half of women (53 percent) who use an IUD obtained it from the public sector, primarily a health center (44 percent). An additional 35 percent obtained their IUD from a private medical source. The public sector is also the most common source of contraception among women who use injectables (65 percent). Similar proportions of implant users obtain their methods from public (49 percent) and private (48 percent) sources. Two-thirds of condom users obtain their method from a private source, predominantly pharmacies. Finally, provincial hospitals and district hospitals are the most commonly cited sources of female sterilization.

Since the 2010 CDHS, there have been changes in the most commonly cited sources of contraceptive methods. In 2010, users of daily pills and male condoms were less likely to obtain them from a pharmacy and more likely to obtain them from the public sector than in 2014. Female sterilization was more commonly done in a private hospital or private clinic in 2014 than in 2010.

8.7 Informed Choice

Current users of modern methods who are well informed about the side effects and problems associated with different methods and who know of a range of method options are in a better position to make an informed choice about the method they would like to use. Current users of various modern contraceptive methods were asked whether, at the time they were initiating their use of a particular method, they were informed about the possible side effects or problems they might have with the method and what to do if they experienced side effects. Table 8.9 shows the percentage of current users of modern methods who were informed about side effects or problems with the method used, informed about what to do if they experienced side effects, and informed of other methods they could use, according to the type of method they are currently using and initial source of the method.

Table 8.9 Informed choice

Among current users of modern methods age 15-49 who started the last episode of use within the five years preceding the survey, the percentage who were informed about possible side effects or problems of that method, the percentage who were informed about what to do if they experienced side effects, and the percentage who were informed about other methods they could use, by method and initial source, Cambodia 2014

	Among women who started last episode of modern contraceptive method within five years preceding the survey:			
Method/source	Percentage who were informed about side effects or problems of method used	Percentage who were informed about what to do if side effects experienced	Percentage who were informed by a health or family planning worker of other methods that could be used	Number of women
**Method**				
Female sterilization	88.4	75.5	80.7	166
Daily pill	73.3	68.9	75.1	1,548
IUD	97.3	96.3	91.0	446
Injectables	84.3	78.8	83.0	874
Implants	94.4	92.6	88.6	251
**Initial source of method**[1]				
Public sector	91.9	88.6	87.9	1,797
National hospital (PP)	(88.7)	(73.9)	(84.7)	28
Provincial hospital (RH)	88.3	82.1	80.6	92
District hospital (RH)	95.4	89.9	90.3	81
Health center	92.1	89.2	88.3	1,573
Health post	(77.0)	(77.0)	(67.4)	13
Other public sector	*	*	*	10
Private sector	70.5	65.5	72.1	1,162
Private hospital	79.0	79.0	85.2	71
Private clinic	87.9	83.9	83.6	431
Pharmacy	54.1	48.8	61.0	435
Other private medical sector	66.3	58.4	67.2	225
Other sources	66.4	57.3	72.2	320
Shop	41.8	36.3	57.8	82
Community distributor	75.6	65.5	77.3	225
Friend/relative	*	*	*	13
Other	*	*	*	5
Total	81.9	77.4	80.7	3,284

Note: Table includes users of only the methods listed individually. Figures in parentheses are based on 25-49 unweighted cases. An asterisk indicates that a figure is based on fewer than 25 unweighted cases and has been suppressed.
[1] Source at start of current episode of use

Overall, 82 percent of contraceptive users were informed about side effects of their method when they initiated their current use of that method. More than three-quarters (77 percent) of women were informed about what to do if they experienced side effects, and 81 percent were informed by a health or family planning worker about other methods they could use.

Findings on informed choice varied by method. Users of IUDs and implants were most likely to have received all three types of information relating to informed choice. Unfortunately, users of the most commonly used method—pills—were least likely to be informed; 73 percent of users of the daily pill were informed of side effects, and 69 percent were told about what to do in the event of side effects. Users of pills (75 percent) were also least likely to be informed of other methods.

8.8 Future Use of Contraception

Intention to use a method of contraception is an important indicator of the potential demand for family planning services. Currently married women who were not using contraception at the time of the survey were asked about their intention to use family planning methods in the future. The results are presented in Table 8.10.

Table 8.10 Future use of contraception

Percent distribution of currently married women age 15-49 who are not using a contraceptive method by intention to use in the future, according to number of living children, Cambodia 2014

	Number of living children[1]					
Intention	0	1	2	3	4+	Total
Intends to use	56.4	72.5	61.2	46.8	29.2	54.4
Unsure	7.6	3.5	4.7	5.0	5.5	4.9
Does not intend to use	36.0	24.1	33.8	48.2	65.1	40.6
Missing	0.0	0.0	0.3	0.1	0.2	0.1
Total	100.0	100.0	100.0	100.0	100.0	100.0
Number of women	540	1,472	1,215	775	1,195	5,198

[1] Includes current pregnancy

Fifty-four percent of currently married women who were not using any contraception at the time of the survey reported that they intend to use a family planning method sometime in the future, approximately the same percentage as in 2010 (53 percent). Forty-one percent do not intend to use any method, and 5 percent are unsure of their intention. The proportion of women who intend to use contraception in the future varied by number of living children, increasing from 56 percent among those with no living children to a peak of 73 percent among those with one child. These women are most likely interested in spacing subsequent births.

8.9 Exposure to Family Planning Messages

The media can be a major source of family planning messages. Information about public exposure to messages on a particular type of media allows policymakers to ensure the use of the most effective means of communication for various target groups in the population. To assess the effectiveness of electronic and print sources in disseminating family planning information, women interviewed in the 2014 CDHS were asked whether they had heard or seen family planning messages on the radio or television or read a family planning message in a newspaper or magazine in the months leading up to the survey. The results are shown in Table 8.11.

Media messages about family planning were largely accessed through television and radio, with lesser access through the print media. For example, 51 percent of women had recently heard about family planning on television and 38 percent had recently heard about it on the radio. By contrast, only 17 percent of women obtained such information from newspapers or magazines. There has been a significant decline in access to family planning messages since 2010. In 2014, 40 percent of women were not exposed to a family planning message through any of these three media sources in the months preceding the survey, as compared with only 25 percent of women in 2010. Access to family planning messages declined for all three types of mass media.

Significant variation was observed in exposure to family planning messages by background characteristics. Younger women were more likely to be exposed to family planning messages than older women, and women in rural areas had less exposure to information on family planning through the media than women in urban areas. For example, 43 percent of rural women had not seen or heard family planning messages in any of the three types of media, as compared with 30 percent of urban women. Educational attainment and wealth quintile were both associated with access to family planning messages in the media. For example, only 5 percent of women with no schooling were exposed to a family planning message in a newspaper or magazine, as compared with 28 percent of women with a secondary education or higher. In addition, the proportion of women who had not seen or heard family planning messages in any of the three types of media decreased steadily from 59 percent among those in the lowest wealth quintile to 28 percent among those in the highest wealth quintile.

Table 8.11 Exposure to family planning messages

Percentage of women age 15-49 who heard or saw a family planning message on radio, on television, or in a newspaper or magazine in the past few months, according to background characteristics, Cambodia 2014

Background characteristic	Radio	Television	Newspaper/ magazine	None of these three media sources	Number of women
**Age**					
15-19	40.1	51.1	18.5	39.2	2,893
20-24	38.6	53.6	21.7	37.8	3,017
25-29	39.8	52.8	18.8	37.3	2,836
30-34	37.2	52.0	16.3	40.3	3,046
35-39	33.2	48.3	13.4	45.0	1,839
40-44	36.0	48.6	14.6	43.6	2,030
45-49	40.0	49.4	13.1	42.5	1,916
**Residence**					
Urban	36.0	63.7	28.2	30.2	3,251
Rural	38.5	48.4	14.6	42.6	14,327
**Province**					
Banteay Meanchey	25.4	45.7	11.1	49.9	689
Kampong Cham	22.4	29.6	5.6	60.3	2,021
Kampong Chhnang	64.0	67.7	33.6	19.1	662
Kampong Speu	32.2	49.2	4.2	44.2	1,196
Kampong Thom	48.6	60.2	12.8	29.4	851
Kandal	43.8	67.3	17.9	28.3	1,330
Kratie	29.2	19.6	6.8	63.0	488
Phnom Penh	35.6	65.6	32.1	27.0	1,994
Prey Veng	82.3	88.7	47.0	10.1	1,188
Pursat	32.9	37.2	8.4	52.5	631
Siem Reap	40.6	41.6	16.5	44.1	1,137
Svay Rieng	44.1	62.1	23.8	33.6	654
Takeo	41.3	67.9	19.3	29.7	1,082
Otdar Meanchey	19.2	28.1	11.1	60.3	294
Battambang/Pailin	19.7	34.8	9.3	57.6	1,333
Kampot/Kep	38.1	45.6	6.0	41.3	770
Preah Sihanouk/ Koh Kong	33.5	60.1	16.0	35.6	422
Preah Vihear/ Stung Treng	28.4	18.7	5.6	62.3	462
Mondul Kiri/ Ratanak Kiri	38.1	25.7	18.7	55.2	372
**Education**					
No education	28.3	33.1	5.1	58.7	2,250
Primary	36.2	47.2	11.2	44.0	8,281
Secondary and higher	43.4	61.6	28.0	30.1	7,047
**Wealth quintile**					
Lowest	32.5	27.7	8.4	59.2	3,143
Second	36.9	42.1	11.5	47.5	3,314
Middle	42.0	53.2	14.2	38.5	3,381
Fourth	41.2	61.6	17.5	33.0	3,612
Highest	37.3	65.7	30.5	28.2	4,128
Total	38.1	51.2	17.1	40.3	17,578

Exposure to family planning messages through the media was highest in Prey Veng and lowest in Kratie and Preah Vihear/Stung Treng.

8.10 CONTACT OF NONUSERS WITH FAMILY PLANNING PROVIDERS

Family planning services are important for the improvement of mother and child health. Thus, it is crucial that every opportunity to meet a woman's family planning needs be fully exploited. In reality, however, health care providers miss these opportunities. Information on missed opportunities was gathered in the survey by asking women who were not currently using a modern contraceptive method whether they had visited a health facility in the 12 months preceding the survey. Those who visited a health facility were asked whether anyone at the facility had discussed family planning with them during any of their visits. Women were also asked whether they had been visited by a fieldworker who talked with them about family planning in the 12 months preceding the survey.

Results showed that three-quarters of nonusers did not have any contact with health care providers or fieldworkers with whom family planning was discussed (Table 8.12). Only 18 percent of nonusers

reported being visited by fieldworkers who discussed family planning issues. Thirty-nine percent of nonusers visited a health facility during the 12 months preceding the survey, but the majority of these women did not discuss family planning with any health care provider (25 percent). Younger nonusers, those in urban areas, and those in Mondul Kiri/Ratanak Kiri are particularly disadvantaged. There have not been any improvements in maximizing opportunities to meet a woman's family planning needs over the past four years. Levels of missed opportunities among nonusers remain high and have actually increased somewhat during this period.

Table 8.12 Contact of nonusers with family planning providers

Among all women age 15-49 who are not currently using any contraception, the percentage who during the past 12 months were visited by a fieldworker who discussed family planning, the percentage who visited a health facility and discussed family planning, the percentage who visited a health facility but did not discuss family planning, and the percentage who did not discuss family planning either with a fieldworker or at a health facility, by background characteristics, Cambodia 2014

Background characteristic	Percentage of women who were visited by fieldworker who discussed family planning	Percentage of women who visited a health facility in the past 12 months and who:		Percentage of women who did not discuss family planning either with fieldworker or at a health facility	Number of women
		Discussed family planning	Did not discuss family planning		
**Age**					
15-19	12.0	6.2	18.6	86.0	2,760
20-24	15.8	15.2	29.9	75.9	2,121
25-29	21.8	20.6	28.3	68.5	1,441
30-34	22.4	22.6	28.8	65.8	1,252
35-39	21.1	17.0	28.5	70.6	771
40-44	20.3	14.2	24.3	72.9	1,024
45-49	22.5	9.7	23.4	74.7	1,436
**Residence**					
Urban	7.1	10.2	32.7	84.9	2,124
Rural	20.8	14.7	23.4	73.2	8,681
**Province**					
Banteay Meanchey	13.9	10.3	25.7	79.3	379
Kampong Cham	15.0	11.8	35.1	77.3	1,353
Kampong Chhnang	66.8	56.4	20.9	28.8	440
Kampong Speu	19.7	9.1	24.5	76.6	643
Kampong Thom	7.8	8.0	17.4	87.6	513
Kandal	10.7	4.2	24.8	85.8	798
Kratie	7.3	11.0	25.0	83.7	317
Phnom Penh	4.3	10.8	44.1	86.0	1,279
Prey Veng	21.1	13.5	4.0	76.2	695
Pursat	11.8	7.4	13.2	84.2	411
Siem Reap	17.0	15.3	29.8	72.9	673
Svay Rieng	10.8	11.8	24.9	81.4	373
Takeo	38.5	25.6	13.6	57.1	672
Otdar Meanchey	15.8	7.7	6.5	81.5	171
Battambang/Pailin	26.7	16.1	25.8	66.4	807
Kampot/Kep	16.4	13.0	15.6	75.3	461
Preah Sihanouk/ Koh Kong	15.6	11.4	12.2	76.6	260
Preah Vihear/ Stung Treng	28.1	17.2	37.0	62.3	330
Mondul Kiri/ Ratanak Kiri	7.0	2.1	25.7	91.6	231
**Education**					
No education	22.8	14.4	23.4	71.6	1,316
Primary	19.7	14.8	25.5	73.4	4,632
Secondary and higher	15.4	12.6	25.4	78.5	4,857
**Wealth quintile**					
Lowest	22.4	15.2	24.8	71.9	1,923
Second	20.7	16.4	19.6	72.5	1,973
Middle	21.1	14.4	22.9	73.2	2,109
Fourth	20.2	13.1	24.0	74.1	2,220
Highest	8.7	10.9	32.7	83.5	2,579
Total	18.1	13.8	25.2	75.5	10,805

OTHER PROXIMATE DETERMINANTS OF FERTILITY 9

Key Findings

- The median age at marriage among men age 25-49 is 23 years, two years older than the median age among women (21 years).
- The percentage of women who were first married by age 15 declines from 7 percent among women currently age 45-49 to 1 percent among women age 15-19.
- Among Cambodian women, the median age at first sex is about the same as the median age at first marriage. In contrast, men typically initiate sexual intercourse one full year before their first marriage.
- Overall, although it is illegal, 3 percent of married women in Cambodia are in a polygamous union.

This chapter examines the principal factors, other than contraception, that affect a woman's chances of becoming pregnant. These factors include marriage (including consensual unions), postpartum amenorrhea, abstinence from sexual relations, and termination of exposure to pregnancy. Marriage and sexual relations relate to childbearing; postpartum amenorrhea and abstinence affect the intervals between births; and menopause marks the end of childbearing. This chapter also takes an in-depth look at more direct measures of timing and level of exposure to the risk of pregnancy: age at first sexual intercourse and frequency of intercourse. Marriage is an important fertility indicator because, for most women in Cambodia, it marks the beginning of regular exposure to the risk of pregnancy. Populations in which the age at first marriage is low also tend to experience early childbearing and high fertility. Measures of the onset of menopause are important because the probability of becoming pregnant decreases as women approach the end of their reproductive years and increasing proportions become infecund. Collectively, the above-mentioned factors determine the duration and pace of reproductive activity and hence are important in understanding fertility.

9.1 MARITAL STATUS

Table 9.1 shows the distribution of all women and men age 15-49 by current marital status. The data indicate that, on average, 25 percent of Cambodian women of reproductive age have never been married, and 68 percent are currently married or cohabiting as if married. Four percent of women of reproductive age are divorced or separated, and 3 percent are widows. A higher proportion of men age 15-49 have never been married (32 percent), and, because men tend to marry later than women, fewer men than women in the youngest age groups have ever been married. Almost no men in the two oldest age groups have never been married.

Table 9.1 Current marital status

Percent distribution of women and men age 15-49 by current marital status, according to age, Cambodia 2014

Age	Marital status						Total	Percentage of respondents currently in union	Number of respondents
	Never married	Married	Living together	Divorced	Separated	Widowed			
WOMEN									
15-19	83.4	15.4	0.2	0.6	0.3	0.1	100.0	15.6	2,893
20-24	35.8	59.9	0.8	1.9	0.3	1.2	100.0	60.8	3,017
25-29	15.0	78.7	0.6	4.1	0.3	1.3	100.0	79.3	2,836
30-34	7.6	85.7	0.5	3.5	0.3	2.5	100.0	86.2	3,046
35-39	5.1	84.9	0.6	4.7	0.4	4.3	100.0	85.5	1,839
40-44	5.0	81.9	0.5	5.2	0.4	7.0	100.0	82.4	2,030
45-49	4.2	77.6	0.4	6.1	0.5	11.2	100.0	78.0	1,916
Total	25.2	67.2	0.5	3.4	0.3	3.3	100.0	67.7	17,578
MEN									
15-19	96.8	2.9	0.1	0.0	0.2	0.0	100.0	3.0	926
20-24	59.4	36.1	0.8	1.5	1.8	0.4	100.0	36.9	835
25-29	24.6	72.0	0.6	2.7	0.1	0.0	100.0	72.6	815
30-34	5.5	91.6	0.1	2.3	0.3	0.2	100.0	91.7	907
35-39	2.5	95.2	0.1	2.1	0.0	0.1	100.0	95.2	556
40-44	1.1	96.1	0.0	0.9	0.1	1.8	100.0	96.1	595
45-49	0.0	97.4	0.5	0.3	0.0	1.7	100.0	98.0	556
Total	32.0	65.3	0.3	1.4	0.4	0.5	100.0	65.6	5,190

Table 9.1 also shows that the proportion of women who have never married decreases with age to a low of 4 percent among those age 45-49. This reflects the near universality of marriage in Cambodian society. Consequently, the proportion of women currently married or cohabiting as if married increases with age up to age 30-39 (86 percent) and declines thereafter due to increasing levels of widowhood. Widowhood also increases with age among men, but not to the same extent as among women. Only 2 percent of men age 45-49 are widowed, as compared with 11 percent of women. This is likely due to men's greater propensity to remarry after having been widowed.

9.2 POLYGAMY

The survey asked currently married women (in formal or informal union) whether their partners had other wives. Table 9.2 shows the percent distribution of married women by number of co-wives, according to background characteristics. Polygamy is not very common in Cambodia. However, although it is illegal, it affects 3 percent of women in union.

The proportion of women in a polygamous marriage increases with age, from less than 2 percent at age 15-19 to 5 percent at age 45-49. Although the prevalence of polygamy does not differ by residence, there is substantial variation by province. The percentage of currently married women with co-wives is highest in Kampot/Kep (9 percent) and lowest in Kampong Thom, Kratie, Otdar Meanchey, Battambang/Pailin, Preah Vihear/Stung Treng, and Mondul Kiri/Ratanak Kiri (less than 1 percent). Women's level of education is related to the prevalence of this practice: the percentage of married women with co-wives is twice as high among those with no education (4 percent) as among those with a secondary education or higher (2 percent). The proportion of women with co-wives varies little by wealth quintile, from 2 percent to 3 percent.

Table 9.2 Number of women's co-wives

Percent distribution of currently married women age 15-49 by number of co-wives, according to background characteristics, Cambodia 2014

Background characteristic	Number of co-wives				Total	Number of women
	0	1	2+	Missing		
**Age**						
15-19	95.0	0.6	1.2	3.2	100.0	450
20-24	94.8	1.7	0.3	3.2	100.0	1,833
25-29	96.1	1.3	0.3	2.3	100.0	2,249
30-34	94.7	2.0	0.6	2.8	100.0	2,625
35-39	92.7	3.4	0.5	3.3	100.0	1,573
40-44	93.6	2.9	0.3	3.2	100.0	1,673
45-49	92.8	4.0	0.9	2.4	100.0	1,495
**Residence**						
Urban	94.1	2.6	0.3	3.0	100.0	1,818
Rural	94.4	2.3	0.5	2.8	100.0	10,080
**Province**						
Banteay Meanchey	92.9	5.2	1.4	0.6	100.0	503
Kampong Cham	89.0	5.3	1.3	4.4	100.0	1,490
Kampong Chhnang	97.0	2.2	0.2	0.6	100.0	396
Kampong Speu	97.3	1.2	0.0	1.5	100.0	843
Kampong Thom	96.2	0.0	0.3	3.5	100.0	572
Kandal	85.6	1.5	0.5	12.4	100.0	870
Kratie	98.6	0.4	0.2	0.8	100.0	359
Phnom Penh	94.3	2.5	0.0	3.2	100.0	1,084
Prey Veng	96.8	2.3	0.0	0.8	100.0	889
Pursat	94.3	2.1	1.1	2.5	100.0	425
Siem Reap	94.7	2.4	0.1	2.8	100.0	765
Svay Rieng	93.3	1.0	0.6	5.1	100.0	483
Takeo	98.1	1.1	0.0	0.8	100.0	677
Otdar Meanchey	98.7	0.4	0.0	0.9	100.0	218
Battambang/Pailin	98.5	0.3	0.2	1.0	100.0	890
Kampot/Kep	89.4	7.2	2.0	1.5	100.0	574
Preah Sihanouk/ Koh Kong	96.7	2.2	0.2	0.9	100.0	266
Preah Vihear/ Stung Treng	99.3	0.5	0.0	0.2	100.0	314
Mondul Kiri/ Ratanak Kiri	99.9	0.1	0.0	0.0	100.0	281
**Education**						
No education	93.6	3.1	0.8	2.5	100.0	1,774
Primary	93.9	2.6	0.6	2.8	100.0	6,399
Secondary and higher	95.4	1.5	0.1	3.1	100.0	3,726
**Wealth quintile**						
Lowest	94.6	2.5	0.9	2.0	100.0	2,294
Second	94.9	2.2	0.5	2.5	100.0	2,404
Middle	94.2	1.9	0.3	3.6	100.0	2,365
Fourth	94.0	2.7	0.4	2.9	100.0	2,393
Highest	94.0	2.4	0.3	3.3	100.0	2,443
Total	94.3	2.3	0.5	2.9	100.0	11,898

9.3 Age at First Union

In many societies, age at first marriage marks the point in a woman's life when childbearing becomes socially acceptable. Women who marry early will on average have a longer exposure to the risk of pregnancy. Therefore, early age at first marriage would imply early age at childbearing and a higher societal level of fertility. Information on age at first marriage was obtained by asking all ever-married respondents the month and year they started living with their first spouse or, if they could not remember the month and year, the age at which they started living with their first spouse. This information is presented in Table 9.3.

Table 9.3 Age at first marriage

Percentage of women and men age 15-49 who were first married by specific exact ages and median age at first marriage, according to current age, Cambodia 2014

Current age	Percentage first married by exact age: 15	18	20	22	25	Percentage never married	Number of respondents	Median age at first marriage
				WOMEN				
15-19	1.4	na	na	na	na	83.4	2,893	a
20-24	1.9	18.5	40.8	na	na	35.8	3,017	a
25-29	2.7	18.6	38.7	55.9	76.4	15.0	2,836	21.2
30-34	3.7	24.5	43.9	59.8	76.5	7.6	3,046	20.7
35-39	5.4	29.5	48.8	67.3	79.8	5.1	1,839	20.1
40-44	5.1	30.3	51.7	68.6	81.9	5.0	2,030	19.8
45-49	6.6	28.2	45.6	62.8	79.7	4.2	1,916	20.5
25-49	4.4	25.4	45.1	62.1	78.5	8.0	11,668	20.5
				MEN				
15-19	0.0	na	na	na	na	96.8	926	a
20-24	0.0	3.6	16.1	na	na	59.4	835	a
25-29	0.0	3.9	13.6	31.9	57.4	24.6	815	24.1
30-34	0.2	10.8	23.7	41.5	67.6	5.5	907	22.9
35-39	0.1	10.3	23.2	42.5	68.5	2.5	556	22.7
40-44	0.2	7.8	23.4	46.2	72.5	1.1	595	22.4
45-49	0.7	10.8	27.0	43.5	66.1	0.0	556	22.6
25-49	0.2	8.5	21.7	40.5	65.9	7.9	3,430	23.0

Note: The age at first marriage is defined as the age at which the respondent began living with her/his first spouse/partner.
na = Not applicable due to censoring
a = Omitted because less than 50 percent of the respondents married for the first time before reaching the beginning of the age group

The median age at first marriage among women in Cambodia has increased marginally over the past two decades and is now just under 21 years. Men have a slightly older median age at first marriage of 23 years. The proportion of women married by age 15 has declined in recent years, dropping from 7 percent among women age 45-49 to 1 percent among women age 15-19. Almost half (45 percent) of Cambodian women age 25-49 are married by age 20, and 79 percent are married by age 25. Less than 1 percent of all Cambodian men age 25-49 are married by the age of 15, and only 9 percent are married by age 18. This finding contrasts fairly sharply with the proportion of women married by age 18 (25 percent).

Table 9.4.1 Median age at first marriage: Women

Median age at first marriage among women by five-year age groups, age 25-49, according to background characteristics, Cambodia 2014

Background characteristic	Age 25-29	30-34	35-39	40-44	45-49	Women age 25-49
**Residence**						
Urban	23.6	22.2	20.1	20.5	21.2	21.7
Rural	20.8	20.5	20.1	19.7	20.3	20.3
**Province**						
Banteay Meanchey	20.9	21.6	19.5	19.7	20.3	20.5
Kampong Cham	21.0	20.1	20.8	20.1	20.5	20.5
Kampong Chhnang	22.3	20.4	20.9	20.4	20.4	20.8
Kampong Speu	19.9	19.9	18.4	19.8	21.0	19.8
Kampong Thom	21.1	20.3	20.6	19.2	20.2	20.2
Kandal	21.4	22.4	21.6	20.0	22.0	21.5
Kratie	19.7	21.1	19.0	19.4	20.4	19.9
Phnom Penh	24.3	22.9	20.5	20.6	21.3	22.1
Prey Veng	20.7	19.8	19.1	19.5	19.4	19.8
Pursat	21.7	20.3	20.0	20.3	21.4	21.1
Siem Reap	20.0	20.6	20.5	20.3	21.1	20.5
Svay Rieng	20.9	19.8	19.0	19.7	20.6	20.0
Takeo	22.4	20.6	21.0	18.8	20.1	20.8
Otdar Meanchey	20.5	20.1	20.1	18.9	20.1	20.1
Battambang/Pailin	22.1	21.4	19.9	20.2	19.7	20.7
Kampot/Kep	19.7	19.4	19.4	19.9	20.2	19.8
Preah Sihanouk/ Koh Kong	21.4	19.9	18.7	19.3	20.2	20.1
Preah Vihear/Stung Treng	20.8	20.0	19.3	20.0	19.2	20.0
Mondul Kiri/ Ratanak Kiri	19.4	19.0	19.0	19.4	19.7	19.3
**Education**						
No education	19.7	19.9	19.4	19.4	19.7	19.7
Primary	20.3	20.2	20.1	19.7	20.2	20.1
Secondary and higher	22.7	22.4	20.7	20.4	22.1	21.9
**Wealth quintile**						
Lowest	20.0	20.2	20.3	20.2	20.9	20.3
Second	19.8	20.4	19.9	19.8	19.9	20.0
Middle	21.0	19.9	20.2	19.6	20.2	20.2
Fourth	22.1	20.6	19.8	19.4	20.2	20.4
Highest	23.0	21.8	20.6	20.4	20.8	21.6
Total	21.2	20.7	20.1	19.8	20.5	20.5

Note: The age at first marriage is defined as the age at which the respondent began living with her/his first spouse/partner.

Table 9.4.1 shows the median age at first marriage among women age 25-49 by current age and selected background characteristics. Table 9.4.2 shows the same information among men age 25-49. The median age at first marriage among urban women (22) is older than that among rural women (20). Men demonstrate greater urban-rural differences in median age at marriage than do women. Less than half of urban men are married by age 25, whereas half of rural men are married by age 23. Median age at first marriage among women varies by almost three years across provinces, ranging from 19 in Mondul Kiri/Ratanak Kiri to 22 in Phnom Penh and Kandal. One consistent difference in age at first marriage among Cambodian women of all ages is by education. Women who have attained a high school education or higher tend to marry two years later than women with no education. Men with a high school education or higher tend to marry more than three years later than their counterparts with no education. Among women, there is little difference in median age at marriage in the lowest four wealth quintiles; however, women in the highest wealth quintile marry at least one year later than their less wealthy counterparts. Among men, the median age at first marriage increases incrementally with increasing wealth.

Table 9.4.2 Median age at first marriage: Men

Median age at first marriage among men by five-year age groups, age 25-49, according to background characteristics, Cambodia 2014

Background characteristic	Age 25-29	30-34	35-39	40-44	45-49	Men age 25-49
**Residence**						
Urban	a	26.0	24.9	24.2	24.3	a
Rural	23.6	22.4	22.5	22.1	22.3	22.6
**Province**						
Banteay Meanchey	22.7	23.6	22.2	23.2	21.5	22.8
Kampong Cham	24.0	22.2	22.7	22.7	20.7	22.9
Kampong Chhnang	a	21.3	21.6	22.7	23.2	22.9
Kampong Speu	23.3	22.4	21.9	21.1	22.4	22.4
Kampong Thom	23.7	23.0	22.9	21.4	23.0	23.0
Kandal	23.5	21.9	23.5	23.5	24.1	22.8
Kratie	24.1	23.7	23.8	22.4	27.3	23.9
Phnom Penh	a	25.8	24.2	24.2	24.3	a
Prey Veng	22.9	23.0	22.8	21.2	22.2	22.4
Pursat	a	24.1	23.9	22.5	26.5	24.7
Siem Reap	23.3	23.5	22.5	21.3	22.7	22.7
Svay Rieng	22.5	21.5	20.4	20.4	17.9	21.1
Takeo	24.6	22.7	22.8	22.0	21.7	22.5
Otdar Meanchey	22.8	23.2	23.3	23.7	23.3	23.1
Battambang/Pailin	24.1	24.2	23.6	22.6	22.9	23.7
Kampot/Kep	23.6	21.6	20.4	20.4	20.9	21.1
Preah Sihanouk/ Koh Kong	a	23.7	24.8	23.2	22.8	24.3
Preah Vihear/ Stung Treng	21.8	23.4	23.5	22.6	20.8	22.5
Mondul Kiri/ Ratanak Kiri	21.9	23.4	23.0	22.2	23.6	22.6
**Education**						
No education	21.3	20.9	21.5	20.6	21.0	21.0
Primary	23.0	21.7	21.9	22.4	21.4	22.1
Secondary and higher	a	24.8	24.6	23.2	24.2	24.5
**Wealth quintile**						
Lowest	21.7	20.9	21.2	21.5	25.0	21.7
Second	23.0	21.8	22.2	22.4	22.4	22.3
Middle	24.1	22.5	22.8	21.4	20.9	22.5
Fourth	23.8	23.5	22.6	22.6	21.9	23.0
Highest	a	25.5	24.9	24.0	24.7	a
Total	24.1	22.9	22.7	22.4	22.6	23.0

Note: The age at first marriage is defined as the age at which the respondent began living with her/his first spouse/partner.
a = Omitted because less than 50 percent of men married for the first time before reaching the beginning of the age group

9.4 AGE AT FIRST SEXUAL INTERCOURSE

Age at first marriage is commonly used as a proxy for the onset of women's exposure to sexual intercourse and risk of pregnancy and sexually transmitted infections. However, because some men and women are sexually active before marriage, it is also important to measure the impact of age at first sexual intercourse on fertility. The 2014 CDHS asked women and men how old they were when they first engaged in sexual intercourse. The results are presented in Tables 9.5, 9.6.1, and 9.6.2.

A comparison of the percentage of women who had first sexual intercourse by specific ages (Table 9.5) with the percentage of women first married by those ages (Table 9.3) shows very little variation, implying that women rarely engage in sexual activity prior to marriage. The median age at first intercourse is slightly older than the median age at first marriage among women age 25-49 (20.7 years versus 20.5 years). Eight percent of women age 25-49 have never had intercourse.

Table 9.5 Age at first sexual intercourse

Percentage of women and men age 15-49 who had first sexual intercourse by specific exact ages, percentage who never had intercourse, and median age at first intercourse, according to current age, Cambodia 2014

Current age	Percentage who had first sexual intercourse by exact age: 15	18	20	22	25	Percentage who never had intercourse	Number	Median age at first intercourse
WOMEN								
15-19	1.4	na	na	na	na	82.9	2,893	a
20-24	1.6	17.4	40.2	na	na	35.2	3,017	a
25-29	2.3	17.8	37.2	55.1	74.6	14.7	2,836	21.4
30-34	3.2	22.5	42.3	58.0	74.1	7.5	3,046	20.9
35-39	5.0	28.9	47.8	65.0	76.4	5.0	1,839	20.3
40-44	4.8	29.5	51.2	67.7	80.4	4.8	2,030	19.9
45-49	6.2	27.4	44.5	62.3	77.8	4.1	1,916	20.6
25-49	4.0	24.4	43.8	60.8	76.3	7.8	11,668	20.7
MEN								
15-19	0.3	na	na	na	na	92.5	926	a
20-24	0.4	4.4	24.4	na	na	45.4	835	a
25-29	0.1	6.0	22.8	45.7	71.3	12.1	815	22.4
30-34	0.3	13.0	30.3	50.7	73.1	1.9	907	21.9
35-39	0.1	10.5	33.4	51.6	73.6	1.0	556	21.8
40-44	0.2	8.9	28.3	51.2	75.1	0.7	595	21.9
45-49	1.0	11.3	33.4	49.8	71.0	0.0	556	22.0
25-49	0.3	9.9	29.2	49.6	72.8	3.7	3,430	22.0

na = Not applicable due to censoring
a = Omitted because less than 50 percent of the respondents had intercourse for the first time before reaching the beginning of the age group

A comparison of the percentage of men who had first sexual intercourse by specific ages (Table 9.5) with the percentage of men first married by those ages (Table 9.3) shows some variation, indicating that men are more likely to engage in sexual activity prior to marriage than women. The percentage of men age 25-49 never having had intercourse is 4 percent, half the proportion of men never having married (8 percent). Half of men have had sexual experience by age 22, while only 41 percent have married by that age. Among men, the median age at first intercourse is one year younger than the median age at first marriage (22.0 years versus 23.0 years).

Table 9.6.1 shows differentials in the median age at first sexual intercourse by background characteristics for women, and Table 9.6.2 shows these differentials for men. Among women (Table 9.6.1), there is a one-year difference in age at first sexual intercourse between urban and rural residents (22 and 21, respectively). There is little variation by province with the exception of a notably older median age at first intercourse among women in Phnom Penh (22 years). There is also a two-year difference between those who have a secondary education or higher (22 years) and those who have less education (20 years) and at least a one-year difference between those in the highest wealth quintile (22) and those in the lower four quintiles (20-21 years). Among men (Table 9.6.2), there is a one-year difference in median age at first sexual intercourse between urban and rural residents (23 and 22 years, respectively). There is a difference of two years between those who have a secondary education or higher (23 years) and those who have less education (21 years). Also, there is a difference of one to two years in the median age at first sex between men in the wealthiest quintile (23 years) and men in the other quintiles (21-22 years).

Table 9.6.1 Median age at first intercourse: Women

Median age at first sexual intercourse among women by five-year age groups, age 25-49, according to background characteristics, Cambodia 2014

Background characteristic	Age 25-29	30-34	35-39	40-44	45-49	Women age 25-49
**Residence**						
Urban	23.7	22.2	20.2	20.6	21.3	21.8
Rural	20.9	20.7	20.3	19.8	20.5	20.5
**Province**						
Banteay Meanchey	21.2	21.3	19.5	19.7	20.4	20.6
Kampong Cham	21.5	20.9	21.2	20.3	20.9	21.0
Kampong Chhnang	22.9	21.1	21.5	20.5	20.8	21.3
Kampong Speu	19.9	20.0	18.5	19.7	21.1	19.8
Kampong Thom	21.1	20.5	20.7	19.2	20.0	20.2
Kandal	21.5	22.4	21.6	20.1	21.6	21.4
Kratie	19.6	21.1	18.9	19.1	20.4	19.8
Phnom Penh	24.4	22.8	20.3	20.6	21.4	22.0
Prey Veng	20.7	19.7	19.3	19.3	19.7	19.8
Pursat	21.8	20.6	20.0	20.4	21.8	21.2
Siem Reap	20.2	20.6	20.9	20.4	21.0	20.6
Svay Rieng	20.9	19.9	18.9	19.5	20.8	20.1
Takeo	22.5	21.0	21.7	18.9	20.7	21.3
Otdar Meanchey	20.8	20.9	21.0	20.5	20.6	20.7
Battambang/Pailin	22.1	21.4	20.1	20.4	19.5	20.7
Kampot/Kep	19.8	19.7	19.2	19.8	20.2	19.8
Preah Sihanouk/ Koh Kong	21.8	20.5	19.1	19.6	20.3	20.4
Preah Vihear/ Stung Treng	20.9	19.9	19.2	19.1	19.1	19.8
Mondul Kiri/ Ratanak Kiri	19.6	19.2	19.3	19.4	19.2	19.4
**Education**						
No education	20.0	20.4	19.9	19.5	19.7	19.9
Primary	20.4	20.4	20.1	19.7	20.3	20.2
Secondary and higher	22.8	22.5	20.8	20.5	22.2	22.0
**Wealth quintile**						
Lowest	20.3	20.4	20.4	20.3	21.1	20.5
Second	19.9	20.6	20.0	19.8	20.2	20.1
Middle	21.3	20.2	20.3	19.5	20.2	20.3
Fourth	22.2	20.8	19.9	19.6	20.4	20.6
Highest	23.0	21.9	20.7	20.5	20.9	21.6
Total	21.4	20.9	20.3	19.9	20.6	20.7

Table 9.6.2 Median age at first intercourse: Men

Median age at first sexual intercourse among men by five-year age groups, age 25-49, according to background characteristics, Cambodia 2014

Background characteristic	Age 25-29	30-34	35-39	40-44	45-49	Men age 25-49
**Residence**						
Urban	22.4	23.1	22.4	23.2	23.1	22.8
Rural	22.4	21.6	21.7	21.6	21.8	21.9
**Province**						
Banteay Meanchey	22.0	22.5	20.7	22.5	21.5	22.0
Kampong Cham	22.4	20.8	20.7	22.0	19.8	21.1
Kampong Chhnang	24.2	21.1	18.9	21.2	20.8	21.5
Kampong Speu	21.4	22.0	20.7	20.7	22.5	21.7
Kampong Thom	23.4	22.9	22.9	21.1	22.8	22.8
Kandal	22.1	20.7	22.8	23.6	21.8	22.1
Kratie	23.2	23.4	23.0	22.0	26.0	23.4
Phnom Penh	22.3	22.7	21.5	23.1	22.9	22.6
Prey Veng	22.6	21.5	22.8	20.8	22.3	22.2
Pursat	22.5	20.5	23.0	20.8	24.5	22.6
Siem Reap	23.0	23.6	21.7	21.3	22.8	22.5
Svay Rieng	21.7	20.4	20.3	19.6	17.9	20.7
Takeo	22.9	22.3	21.3	21.5	21.6	22.1
Otdar Meanchey	24.3	23.1	26.1	22.3	21.7	23.6
Battambang/Pailin	21.7	22.2	23.0	24.2	23.1	22.6
Kampot/Kep	22.4	21.7	20.4	19.7	20.8	21.1
Preah Sihanouk/ Koh Kong	21.4	20.8	21.9	20.8	20.1	21.0
Preah Vihear/ Stung Treng	22.1	25.2	23.5	23.6	22.7	23.4
Mondul Kiri/ Ratanak Kiri	21.3	23.8	20.8	22.1	23.7	22.2
**Education**						
No education	20.8	21.3	20.4	20.5	20.8	20.7
Primary	21.7	20.8	21.4	21.9	21.0	21.3
Secondary and higher	22.8	22.9	23.0	22.3	23.0	22.8
**Wealth quintile**						
Lowest	21.6	21.0	21.0	21.2	23.2	21.5
Second	21.5	21.5	20.8	22.0	21.3	21.4
Middle	23.3	21.5	22.6	20.9	20.7	21.9
Fourth	22.6	21.8	21.9	22.3	21.5	22.1
Highest	22.7	23.4	22.8	23.2	23.2	23.1
Total	22.4	21.9	21.8	21.9	22.0	22.0

9.5 RECENT SEXUAL ACTIVITY

In addition to age at first sexual intercourse, in the absence of effective contraception, exposure to pregnancy depends on the pattern of sexual activity. The most important factors are frequency of intercourse, postpartum abstinence, and abstinence for reasons other than being postpartum. Information on recent sexual activity, therefore, can be used to refine measures of exposure to pregnancy. Table 9.7.1 shows patterns of sexual activity among women in the four weeks preceding the survey by background characteristics, and Table 9.7.2 shows patterns among men.

Table 9.7.1 Recent sexual activity: Women

Percent distribution of women age 15-49 by timing of last sexual intercourse, according to background characteristics, Cambodia 2014

Background characteristic	Timing of last sexual intercourse: Within the last 4 weeks	Within 1 year[1]	One or more years	Missing	Never had sexual intercourse	Total	Number of women
**Age**							
15-19	12.4	3.8	0.9	0.1	82.9	100.0	2,893
20-24	48.4	13.2	3.2	0.0	35.2	100.0	3,017
25-29	66.9	12.4	6.0	0.0	14.7	100.0	2,836
30-34	74.8	11.5	6.2	0.1	7.5	100.0	3,046
35-39	73.3	11.4	10.2	0.0	5.0	100.0	1,839
40-44	69.3	11.6	14.3	0.1	4.8	100.0	2,030
45-49	61.6	14.6	19.6	0.2	4.1	100.0	1,916
**Marital status**							
Never married	0.4	0.3	0.5	0.1	98.7	100.0	4,428
Married or living together	83.1	15.1	1.8	0.0	0.0	100.0	11,898
Divorced/separated/ widowed	2.0	9.7	88.0	0.3	0.0	100.0	1,252
**Marital duration[2]**							
0-4 years	85.4	12.8	1.7	0.0	0.0	100.0	8,267
5-9 years	84.7	13.6	1.7	0.0	0.0	100.0	2,244
10-14 years	89.1	10.3	0.6	0.0	0.0	100.0	1,897
15-19 years	86.7	11.7	1.5	0.1	0.0	100.0	1,422
20-24 years	85.6	12.2	2.2	0.0	0.0	100.0	1,399
25+ years	79.5	17.3	3.2	0.0	0.0	100.0	1,306
Married more than once	79.6	18.2	2.2	0.0	0.0	100.0	868
**Residence**							
Urban	46.9	9.7	8.0	0.0	35.4	100.0	3,251
Rural	58.7	11.3	7.5	0.1	22.5	100.0	14,327
**Province**							
Banteay Meanchey	57.9	14.7	6.1	0.0	21.4	100.0	689
Kampong Cham	61.5	11.6	7.3	0.0	19.6	100.0	2,021
Kampong Chhnang	51.3	8.6	8.6	0.0	31.6	100.0	662
Kampong Speu	60.9	10.1	7.2	0.0	21.7	100.0	1,196
Kampong Thom	60.3	7.0	8.0	0.0	24.8	100.0	851
Kandal	56.0	10.0	8.5	0.0	25.4	100.0	1,330
Kratie	64.8	8.9	4.5	0.1	21.7	100.0	488
Phnom Penh	45.3	9.8	7.5	0.0	37.4	100.0	1,994
Prey Veng	64.4	9.7	9.6	0.0	16.3	100.0	1,188
Pursat	54.0	12.0	6.1	0.0	27.8	100.0	631
Siem Reap	53.4	14.0	8.4	0.0	24.3	100.0	1,137
Svay Rieng	57.8	14.7	7.2	0.5	19.8	100.0	654
Takeo	50.4	11.0	10.9	0.5	27.1	100.0	1,082
Otdar Meanchey	63.2	10.5	4.3	0.0	22.0	100.0	294
Battambang/Pailin	53.5	12.3	6.2	0.0	28.0	100.0	1,333
Kampot/Kep	60.2	13.3	6.8	0.0	19.7	100.0	770
Preah Sihanouk/ Koh Kong	50.3	12.9	7.0	0.0	29.9	100.0	422
Preah Vihear/ Stung Treng	58.9	9.9	7.9	0.0	23.3	100.0	462
Mondul Kiri/ Ratanak Kiri	69.2	7.3	6.1	0.0	17.4	100.0	372
**Education**							
No education	65.5	13.4	11.6	0.1	9.4	100.0	2,250
Primary	65.1	11.5	8.4	0.0	15.0	100.0	8,281
Secondary and higher	43.5	9.6	5.4	0.1	41.4	100.0	7,047
**Wealth quintile**							
Lowest	61.3	11.1	8.9	0.1	18.7	100.0	3,143
Second	59.9	12.6	6.7	0.0	20.7	100.0	3,314
Middle	58.4	11.0	7.6	0.1	22.9	100.0	3,381
Fourth	55.0	11.0	7.4	0.1	26.5	100.0	3,612
Highest	49.8	9.7	7.4	0.0	33.0	100.0	4,128
Total	56.5	11.0	7.6	0.1	24.9	100.0	17,578

[1] Excludes women who had sexual intercourse within the last 4 weeks
[2] Excludes women who are not currently married

More than half (57 percent) of all women had been sexually active during the four weeks preceding the survey; 11 percent had not had sex within the past four weeks but had done so within the past year; and 8 percent had not had sex in one year or longer. The remaining 25 percent had never had sexual intercourse. The proportion of women who were sexually active in the four weeks prior to the survey increased with age up to age 30-34 and declined thereafter. With respect to marital duration, the

proportion sexually active in the past four weeks peaked at a duration of 10-14 years and declined thereafter. A higher proportion of rural women (59 percent) than urban women (47 percent) were recently sexually active. The proportion of women who were sexually active in the four weeks before the survey declined with increasing education and wealth.

Table 9.7.2 Recent sexual activity: Men

Percent distribution of men age 15-49 by timing of last sexual intercourse, according to background characteristics, Cambodia 2014

Background characteristic	Timing of last sexual intercourse: Within the last 4 weeks	Within 1 year[1]	One or more years	Missing	Never had sexual intercourse	Total	Number of men
**Age**							
15-19	4.0	2.5	1.0	0.0	92.5	100.0	926
20-24	33.1	13.3	7.9	0.2	45.4	100.0	835
25-29	60.6	18.3	9.0	0.0	12.1	100.0	815
30-34	77.7	15.7	4.7	0.0	1.9	100.0	907
35-39	76.6	18.4	3.9	0.0	1.0	100.0	556
40-44	72.7	22.4	4.1	0.1	0.7	100.0	595
45-49	76.2	20.5	3.3	0.0	0.0	100.0	556
**Marital status**							
Never married	2.4	7.3	8.4	0.1	81.8	100.0	1,663
Married or living together	80.7	18.3	1.0	0.0	0.0	100.0	3,405
Divorced/separated/ widowed	5.8	25.9	66.7	0.5	1.1	100.0	122
**Marital duration[2]**							
0-4 years	80.3	18.7	1.1	0.0	0.0	100.0	2,363
5-9 years	83.1	15.9	1.0	0.0	0.0	100.0	639
10-14 years	84.8	15.0	0.3	0.0	0.0	100.0	592
15-19 years	78.0	21.7	0.4	0.0	0.0	100.0	457
20-24 years	75.1	22.5	2.5	0.0	0.0	100.0	407
25+ years	75.4	22.5	2.0	0.1	0.0	100.0	269
Married more than once	80.8	17.9	1.3	0.0	0.0	100.0	296
**Residence**							
Urban	46.0	16.4	7.4	0.0	30.2	100.0	869
Rural	55.4	14.7	4.4	0.1	25.4	100.0	4,321
**Province**							
Banteay Meanchey	61.5	8.9	4.0	0.0	25.6	100.0	192
Kampong Cham	53.8	21.9	7.0	0.0	17.3	100.0	663
Kampong Chhnang	54.1	10.8	6.9	0.1	28.1	100.0	182
Kampong Speu	54.8	16.4	3.3	0.0	25.5	100.0	323
Kampong Thom	59.4	4.5	3.0	0.0	33.1	100.0	232
Kandal	46.7	25.3	4.3	0.5	23.2	100.0	413
Kratie	63.9	9.3	2.5	0.0	24.3	100.0	143
Phnom Penh	44.1	18.0	6.3	0.0	31.6	100.0	550
Prey Veng	64.8	7.6	5.1	0.0	22.5	100.0	342
Pursat	49.7	12.7	3.8	0.0	33.7	100.0	184
Siem Reap	58.8	10.8	2.4	0.0	27.9	100.0	337
Svay Rieng	58.3	14.1	5.6	0.0	22.0	100.0	183
Takeo	44.5	21.3	7.5	0.0	26.7	100.0	334
Otdar Meanchey	54.8	12.0	2.2	0.2	30.8	100.0	99
Battambang/Pailin	49.1	12.5	6.4	0.0	32.1	100.0	405
Kampot/Kep	61.8	13.0	2.9	0.0	22.4	100.0	241
Preah Sihanouk/ Koh Kong	54.9	13.5	6.5	0.0	25.1	100.0	120
Preah Vihear/ Stung Treng	60.2	8.5	1.5	0.5	29.3	100.0	112
Mondul Kiri/ Ratanak Kiri	57.7	8.7	1.3	0.0	32.3	100.0	134
**Education**							
No education	69.6	16.8	1.9	0.0	11.7	100.0	324
Primary	60.1	16.3	4.4	0.0	19.2	100.0	2,167
Secondary and higher	47.0	13.6	5.7	0.1	33.6	100.0	2,699
**Wealth quintile**							
Lowest	54.9	15.3	3.4	0.1	26.3	100.0	901
Second	57.9	13.3	4.1	0.0	24.8	100.0	954
Middle	55.3	15.0	4.5	0.0	25.2	100.0	1,040
Fourth	53.5	15.3	4.8	0.2	26.2	100.0	1,124
Highest	48.8	15.7	7.1	0.0	28.4	100.0	1,171
Total	53.9	15.0	4.9	0.1	26.2	100.0	5,190

[1] Excludes men who had sexual intercourse within the last 4 weeks
[2] Excludes men who are not currently married

The proportion of men who reported being sexually active in the past four weeks (54 percent) was similar to that of women. Fifteen percent of men had not had sex within the past four weeks but had done so within the past year, and 5 percent had not had sex in one year or longer. Approximately the same proportion of men as women had never had sex (26 percent). The proportion of men who were sexually active in the four weeks prior to the survey increased with age up to age 30-34, with 78 percent of men in that age group reporting sex in the past four weeks. The proportion of men who were sexually active in the four weeks prior to the survey peaked at a marital duration of 10-14 years (85 percent) and declined thereafter. Rural men were more likely to have had sexual intercourse in the four weeks preceding the survey (55 percent) than urban men (46 percent). In terms of education, the proportion recently sexually active fell from 70 percent among men with no education to 47 percent among men with a secondary education or higher. Education was also related to the percentage of men who had never had sexual intercourse, with this percentage rising steadily with increasing education. The proportion of men who were sexually active in the four weeks preceding the survey was lower among those in the highest wealth quintile (49 percent) than among those in the lower quintiles (54-58 percent). Recent sexual activity among men ranged from a low of 44 percent in Phnom Penh to a high of 65 percent in Prey Veng.

9.6 POSTPARTUM AMENORRHEA, ABSTINENCE, AND INSUSCEPTIBILITY

Postpartum amenorrhea refers to the interval between childbirth and the resumption of ovulation, a period during which a woman is temporarily infecund. As shown in various studies, the length and intensity of breastfeeding influence the duration of postpartum amenorrhea. Women are considered insusceptible if they are not exposed to the risk of pregnancy either because they are amenorrheic or because they are abstaining from sexual intercourse after a birth. Table 9.8 shows the percentage of births in the three years prior to the survey for which mothers are amenorrheic, abstaining from sex, and insusceptible, by the number of months since the birth.

Table 9.8 Postpartum amenorrhea, abstinence, and insusceptibility

Percentage of births in the three years preceding the survey for which mothers are postpartum amenorrheic, abstaining, and insusceptible, by number of months since birth, and median and mean durations, Cambodia 2014

Months since birth	Percentage of births for which the mother is: Amenorrheic	Abstaining	Insusceptible[1]	Number of births
<2	95.8	96.5	98.7	216
2-3	77.9	61.2	87.7	267
4-5	61.7	27.9	70.9	254
6-7	50.5	17.5	58.8	269
8-9	35.2	9.7	40.6	256
10-11	34.4	5.3	37.4	240
12-13	23.6	5.7	27.3	225
14-15	13.6	2.3	15.4	259
16-17	8.6	3.6	12.2	225
18-19	4.9	3.7	8.4	255
20-21	7.6	5.5	11.1	276
22-23	6.0	4.3	9.0	251
24-25	3.9	2.9	6.6	236
26-27	3.3	2.5	5.8	233
28-29	2.9	4.4	6.8	240
30-31	2.6	4.5	7.1	250
32-33	1.4	1.7	3.2	235
34-35	2.1	0.9	2.3	211
Total	24.4	14.3	28.6	4,397
Median	6.4	3.3	7.8	na
Mean	9.0	5.5	10.4	na

Note: Estimates are based on status at the time of the survey.
na = Not applicable
[1] Includes births for which mothers are either still amenorrheic or still abstaining (or both) following birth

In Cambodia, the typical duration of postpartum amenorrhea is considerably longer than the typical duration of postpartum abstinence and is the major determinant of postpartum insusceptibility to pregnancy. Cambodian women are insusceptible to pregnancy for a median period of almost eight months after birth. They are amenorrheic for a median period of more than six months, but they abstain after childbirth for only about three months. In the first two months after birth, almost all mothers are postpartum amenorrheic and abstaining from sex. However, by six to seven months after birth, while half of mothers are still amenorrheic only 18 percent are abstaining. Seventy-one percent of mothers remain insusceptible to pregnancy at four to five months postpartum.

Table 9.9 shows the median duration of postpartum amenorrhea, abstinence, and insusceptibility to pregnancy according to background characteristics. The median duration of postpartum insusceptibility is slightly longer for births to older women and births in rural areas. Women in the highest educational and wealth categories have the shortest periods of postpartum insusceptibility.

9.7 Termination of Exposure to Pregnancy

The risk of childbearing declines as age increases. The term infecundity denotes a process rather than a well-defined event. Although the onset of infecundity is difficult to determine for an individual woman, there are ways of estimating it for a group of women. Table 9.10 presents data on menopause, an indicator of decreasing exposure to the risk of pregnancy (infecundity) among women age 30 and over.

Table 9.9 Median duration of amenorrhea, postpartum abstinence, and postpartum insusceptibility

Median number of months of postpartum amenorrhea, postpartum abstinence, and postpartum insusceptibility following births in the three years preceding the survey, by background characteristics, Cambodia 2014

Background characteristic	Postpartum amenorrhea	Postpartum abstinence	Postpartum insusceptibility[1]
**Mother's age**			
15-29	6.1	3.3	7.3
30-49	8.7	3.3	9.7
**Residence**			
Urban	4.5	3.7	6.3
Rural	6.8	3.3	8.1
**Province**			
Banteay Meanchey	5.3	2.4	5.8
Kampong Cham	8.6	3.4	9.3
Kampong Chhnang	4.6	3.2	7.0
Kampong Speu	5.5	2.9	7.3
Kampong Thom	6.4	2.3	8.6
Kandal	4.1	3.0	10.9
Kratie	8.6	3.2	8.8
Phnom Penh	3.6	5.2	7.2
Prey Veng	6.1	2.7	7.0
Pursat	5.6	3.3	7.6
Siem Reap	5.9	3.5	6.5
Svay Rieng	4.7	2.9	5.7
Takeo	7.6	4.5	9.3
Otdar Meanchey	5.8	4.4	6.2
Battambang/Pailin	6.2	2.7	6.4
Kampot/Kep	7.1	4.7	7.9
Preah Sihanouk/ Koh Kong	5.6	2.4	9.0
Preah Vihear/ Stung Treng	7.4	4.8	7.5
Mondul Kiri/ Ratanak Kiri	6.7	2.9	7.8
**Education**			
No education	6.4	3.1	7.6
Primary	7.6	3.1	8.7
Secondary and higher	5.6	3.8	6.6
**Wealth quintile**			
Lowest	6.6	2.8	7.4
Second	7.7	3.8	8.5
Middle	6.4	3.0	8.0
Fourth	7.1	3.7	8.8
Highest	4.3	3.6	5.9
Total	6.4	3.3	7.8

Note: Medians are based on status at the time of the survey (current status).
[1] Includes births for which mothers are either still amenorrheic or still abstaining (or both) following birth

A woman is considered menopausal if she is not pregnant, is not postpartum amenorrheic, and did not have a menstrual period for at least six months before the survey. Twelve percent of Cambodian women age 30-49 are menopausal. As expected, the proportion of women who have reached menopause increases with age, particularly after age 45. It rises from 12 percent among women age 44-45 to 39 percent among women at the end of their reproductive years (age 48-49).

Table 9.10 Menopause

Percentage of women age 30-49 who are menopausal, by age, Cambodia 2014

Age	Percentage menopausal[1]	Number of women
30-34	5.5	3,046
35-39	7.0	1,839
40-41	9.8	742
42-43	10.3	818
44-45	11.9	901
46-47	21.8	730
48-49	39.0	756
Total	11.5	8,832

[1] Percentage of all women who are not pregnant and not postpartum amenorrheic whose last menstrual period occurred six or more months preceding the survey

FERTILITY PREFERENCES 10

Key Findings

- About half of currently married women and men age 15-49 (52 percent and 51 percent, respectively) either want no more children or have been sterilized.
- The ideal number of children is 3.3 among currently married women and 3.4 among currently married men.
- The percentage of planned births has not changed from the figure reported in the 2010 CDHS (84 percent).
- About 12 percent of currently married women have an unmet need for family planning services, with 5 percent in need of spacing and 7 percent in need of limiting.

The 2014 CDHS collected information on fertility preferences to measure the overall attitudes of women and men toward childbearing and the general course of future fertility. Data on fertility preferences are also useful for assessing unmet need for family planning and the number of unwanted or mistimed births in the population. These data, together with information on contraceptive prevalence, provide an estimation of the demand for family planning.

10.1 Desire for More Children

Currently married women and men in Cambodia were asked whether they wanted to have a child (or another child) and, if so, how soon. Table 10.1 presents fertility preferences among currently married women and men age 15-49 by number of living children. Forty-two percent of currently married women state that they want to have another child; this is a slight increase from the 2010 CDHS, in which 38 percent of women stated that they wanted to have another child. Twelve percent of women want to have a child within two years, 27 percent prefer to wait for two years or more to have another child, and 2 percent want another child but are undecided as to when they want to have that child. Slightly more than half of married women want no more children; 52 percent want no more or have been sterilized. This is a slight decrease relative to the percentage in the 2010 CDHS who reported wanting no more children (56 percent). Three percent of married women are undecided about whether they want more children. The information presented in Table 10.1 indicates that, among women who would like to have another child, many prefer to space their pregnancies and are potentially in need of family planning for that purpose, as are the larger proportions of women who express the desire to limit their births.

Fertility preferences among men are similar to those of women. Forty-eight percent of currently married men want to have another child, 51 percent do not want to have another child (or have been sterilized), and less than 1 percent are undecided. Most men who want to have a child want to wait two or more years (33 percent of all currently married men).

Table 10.1 Fertility preferences by number of living children

Percent distribution of currently married women and currently married men age 15-49 by desire for children, according to number of living children, Cambodia 2014

Desire for children	Number of living children[1]							Total
	0	1	2	3	4	5	6+	
**WOMEN**								
Have another soon[2]	73.2	19.2	8.7	4.0	2.5	2.2	0.5	12.4
Have another later[3]	12.1	62.5	30.5	11.0	3.4	1.3	0.6	27.0
Have another, undecided when	4.3	3.3	2.3	1.5	1.0	0.8	0.1	2.1
Undecided	1.3	3.1	4.7	3.2	1.3	1.5	0.5	3.1
Want no more	4.4	9.6	48.4	71.0	79.3	81.8	85.7	48.4
Sterilized[4]	0.4	0.6	1.9	4.9	6.4	7.3	6.5	3.1
Declared infecund	4.3	1.8	3.2	4.5	6.1	5.0	6.2	3.8
Missing	0.0	0.0	0.1	0.0	0.0	0.1	0.0	0.0
Total	100.0	100.0	100.0	100.0	100.0	100.0	100.0	100.0
Number	673	2,824	3,452	2,283	1,392	713	563	11,898
**MEN**								
Have another soon[2]	72.1	18.7	9.0	7.2	4.6	4.9	2.9	13.8
Have another later[3]	14.1	70.0	39.3	17.9	6.8	3.6	0.6	32.5
Have another, undecided when	1.0	1.1	1.9	1.6	0.2	0.4	1.0	1.3
Undecided	0.9	0.9	0.6	0.5	0.5	0.0	0.0	0.6
Want no more	6.3	8.7	48.4	70.2	84.8	85.6	93.8	49.8
Sterilized[4]	0.4	0.2	0.2	1.7	2.1	1.7	0.9	0.9
Declared infecund	5.1	0.3	0.6	0.6	0.9	1.4	0.4	0.9
Missing	0.0	0.1	0.0	0.1	0.0	2.3	0.2	0.2
Total	100.0	100.0	100.0	100.0	100.0	100.0	100.0	100.0
Number	215	754	1,002	669	400	220	145	3,405

[1] The number of living children includes current pregnancy for women.
[2] Wants next birth within 2 years
[3] Wants to delay next birth for 2 or more years
[4] Includes both female and male sterilization

Tables 10.2.1 and 10.2.2 display the percentage of currently married women and men age 15-49 who want no more children by number of living children and background characteristics. As mentioned above, over half of currently married women want no more children (52 percent). Desire to limit childbearing increases with increasing number of living children, from 5 percent among married women with no living children to 92 percent among women with six or more living children. There are particularly notable increases in the proportion of women wanting no more children between parities one and two (a difference of 40 percentage points) and parities two and three (a difference of 26 percentage points). The large proportion of women indicating a desire to have no more children at parities two and three is consistent with an ideal family size of two to three children.

Table 10.2.1 Desire to limit childbearing: Women

Percentage of currently married women age 15-49 who want no more children, by number of living children, according to background characteristics, Cambodia 2014

Background characteristic	Number of living children[1]							Total
	0	1	2	3	4	5	6+	
**Residence**								
Urban	4.2	12.2	54.8	79.5	87.4	90.6	93.6	50.8
Rural	4.9	9.8	49.4	75.2	85.4	88.9	92.1	51.7
**Province**								
Banteay Meanchey	(5.6)	10.4	45.9	82.6	85.1	(95.2)	(92.2)	48.1
Kampong Cham	(20.9)	11.4	40.1	74.7	88.5	(85.1)	(90.2)	51.2
Kampong Chhnang	(0.0)	4.5	52.2	75.7	86.0	(100.0)	(90.9)	52.8
Kampong Speu	(3.9)	6.1	51.2	80.0	92.0	(92.8)	(97.0)	51.8
Kampong Thom	(3.3)	15.9	52.5	78.6	93.6	93.4	(94.2)	59.1
Kandal	(0.0)	6.4	51.6	84.5	89.0	*	*	51.4
Kratie	(1.7)	7.2	48.7	65.0	79.0	89.4	(99.2)	50.8
Phnom Penh	2.5	12.0	57.0	83.4	86.9	*	*	49.8
Prey Veng	(5.1)	18.0	56.7	77.2	85.9	(82.7)	*	54.5
Pursat	(0.0)	6.1	40.6	77.1	90.3	(97.0)	(87.0)	50.7
Siem Reap	(2.5)	12.5	51.8	75.8	78.5	(88.9)	(100.0)	55.0
Svay Rieng	*	20.6	63.8	87.1	93.1	*	*	60.6
Takeo	(0.0)	9.2	43.4	57.9	75.6	(65.2)	*	42.7
Otdar Meanchey	(0.0)	7.4	50.1	78.1	80.9	(92.0)	(89.2)	51.5
Battambang/Pailin	(5.3)	5.4	48.9	74.3	86.0	(92.5)	*	50.5
Kampot/Kep	(3.3)	7.2	61.5	64.0	74.0	(80.7)	(82.3)	50.0
Preah Sihanouk/ Koh Kong	(3.4)	5.7	45.9	79.1	83.1	(96.7)	(89.2)	51.3
Preah Vihear/ Stung Treng	(0.7)	6.9	41.0	66.5	86.1	(95.9)	(96.3)	49.0
Mondul Kiri/ Ratanak Kiri	(4.2)	9.7	38.5	69.2	92.3	(89.1)	92.7	48.2
**Education**								
No education	7.0	17.2	49.7	71.4	85.8	86.1	87.2	62.1
Primary	5.3	11.7	50.2	75.6	86.4	89.1	94.7	55.7
Secondary and higher	3.9	7.3	50.8	79.9	82.9	97.5	(91.8)	39.4
**Wealth quintile**								
Lowest	9.5	11.8	43.6	75.6	85.0	90.2	93.5	55.5
Second	8.1	11.7	49.0	71.5	87.3	89.8	91.5	52.7
Middle	5.3	8.5	51.7	73.7	80.7	85.8	88.8	50.0
Fourth	0.8	9.4	50.9	77.1	90.6	87.9	96.2	50.3
Highest	2.8	9.8	54.6	80.9	83.5	93.3	(87.8)	49.4
Total	4.7	10.1	50.4	75.9	85.6	89.1	92.2	51.6

Note: Women who have been sterilized are considered to want no more children. Figures in parentheses are based on 25-49 unweighted cases. An asterisk indicates that a figure is based on fewer than 25 unweighted cases and has been suppressed.
[1] The number of living children includes the current pregnancy.

It is worth noting that the proportion of women who want no more children is much larger among those with no education (62 percent) than among those with a secondary education or higher (39 percent). There is considerable variation across provinces; Takeo has the smallest proportion of women wishing to curtail their fertility (43 percent), whereas Svay Rieng has the highest proportion (61 percent). The proportion of women who want no more children is similar in urban and rural areas (51 percent and 52 percent, respectively). Desire to limit childbearing generally decreases with increasing household wealth.

As observed for women, the percentage of currently married men age 15-49 who want no more children increases with number of living children. However, men in rural areas are slightly more likely than men in urban areas to want no more children (51 percent and 48 percent, respectively). By province, the percentage of men who want no more children ranges from 38 percent in Battambang/Pailin to 60 percent in Siem Reap. The percentage of men who want no more children is inversely associated with level of education and is lower among those in the highest wealth quintile than among those in the lower four quintiles.

Table 10.2.2 Desire to limit childbearing: Men

Percentage of currently married men age 15-49 who want no more children, by number of living children, according to background characteristics, Cambodia 2014

Background characteristic	Number of living children[1]							Total
	0	1	2	3	4	5	6+	
**Residence**								
Urban	8.1	9.1	53.7	76.7	75.9	*	*	47.9
Rural	6.4	8.8	47.7	71.2	88.4	87.4	96.0	51.2
**Province**								
Banteay Meanchey	*	(7.4)	57.3	(68.8)	*	*	*	50.6
Kampong Cham	*	(2.3)	44.5	(70.9)	(82.5)	*	*	51.5
Kampong Chhnang	*	(6.8)	(35.7)	(71.2)	*	*	*	46.2
Kampong Speu	*	(17.4)	62.6	(67.3)	*	*	*	59.3
Kampong Thom	*	(8.0)	39.9	(76.5)	*	*	*	53.4
Kandal	*	(8.2)	(58.6)	(75.1)	*	*	*	53.2
Kratie	*	(11.0)	57.7	(71.3)	*	*	*	53.8
Phnom Penh	(15.3)	(8.1)	58.6	(83.2)	*	*	*	47.8
Prey Veng	*	(18.7)	54.3	(62.4)	*	*	*	52.6
Pursat	*	(11.2)	(19.8)	(63.0)	(94.1)	*	*	43.0
Siem Reap	*	(19.7)	(61.7)	(71.9)	*	*	*	59.8
Svay Rieng	*	(4.3)	(52.8)	(71.2)	*	*	*	44.7
Takeo	*	(5.6)	(43.5)	(75.8)	(91.6)	*	*	52.9
Otdar Meanchey	*	(17.2)	56.4	(76.7)	*	*	*	53.7
Battambang/Pailin	*	(2.9)	29.0	(79.2)	*	*	*	37.5
Kampot/Kep	*	(9.9)	55.1	(74.2)	(94.1)	*	*	58.9
Preah Sihanouk/ Koh Kong	*	(0.8)	40.0	(59.2)	(86.6)	*	*	44.1
Preah Vihear/ Stung Treng	*	(7.6)	(25.8)	(49.2)	*	*	*	39.5
Mondul Kiri/ Ratanak Kiri	*	(1.4)	21.8	(65.3)	(86.5)	*	*	42.8
**Education**								
No education	*	(20.1)	62.8	56.0	(94.0)	(94.5)	(93.4)	63.3
Primary	7.1	10.3	47.8	73.3	84.9	86.6	95.1	55.0
Secondary and higher	6.8	6.8	47.4	73.0	88.2	(83.8)	(94.9)	43.9
**Wealth quintile**								
Lowest	(12.2)	17.9	43.1	66.0	88.3	82.1	100.0	55.4
Second	(6.0)	6.3	46.8	75.1	90.6	(91.5)	(90.0)	52.0
Middle	(2.0)	8.0	48.3	71.7	78.8	(93.0)	(89.1)	49.9
Fourth	(4.4)	7.6	53.2	68.3	92.4	(82.0)	*	51.3
Highest	9.3	6.1	49.5	77.4	82.1	*	*	45.4
Total	6.8	8.9	48.6	72.0	86.9	87.3	94.8	50.7

Note: Men who have been sterilized or who state in response to the question about desire for children that their wife has been sterilized are considered to want no more children. Figures in parentheses are based on 25-29 unweighted cases. An asterisk indicates that a figure is based on fewer than 25 unweighted cases and has been suppressed.
[1] The number of living children includes one additional child if the respondent's wife is pregnant (or if any wife is pregnant for men with more than one current wife).

10.2 Need and Demand for Family Planning Services

The proportion of women who want to stop childbearing or who want to space their next birth is a crude measure of the extent of the need for family planning, given that not all of these women are exposed to the risk of pregnancy and some may already be using contraception. This section discusses the extent of need and the potential demand for family planning services. Women who want to postpone their next birth for two or more years or who want to stop childbearing altogether but are not using a contraceptive method are said to have an unmet need for family planning. Pregnant women are considered to have an unmet need for spacing or limiting if their pregnancy was mistimed or unwanted. Similarly, amenorrheic women are categorized as having an unmet need if their last birth was mistimed or unwanted. Women who are currently using a family planning method are said to have a met need for family planning. The sum of women with unmet need and met need constitutes the total demand for family planning.

Table 10.3 presents data on unmet need, met need, and total demand for family planning among currently married women age 15-49 by background characteristics. These indicators help evaluate the extent to which family planning programs in Cambodia meet the demand for services. The definition of unmet need for family planning has been revised so that data on levels of unmet need are comparable over time and across surveys. The unmet need estimates for the 2005 and 2010 CDHS surveys have been

recalculated using the revised definition of unmet need but differ only slightly from the numbers published in the previous final reports.

The percentage of currently married women with a met need for family planning has increased over the past decade, from 40 percent in 2005 to 51 percent in 2010 and 56 percent in 2014.[1] This increase in the use of family planning has resulted in a corresponding decrease in unmet need from 25 percent in 2005 and 17 percent in 2010 to 12 percent in 2014. The total demand for family planning among currently married women is 69 percent, and 82 percent of that demand is satisfied. Fifty-six percent of total demand is satisfied by modern methods. Total demand for family planning in 2014 remained about the same as that observed in 2010 (68 percent), while total demand satisfied increased from the 2010 figure (76 percent).

Five percent of currently married women have an unmet need for spacing, and 7 percent have an unmet need for limiting. The level of unmet need for spacing decreases with age, whereas the opposite is true for unmet need for limiting. Unmet need is slightly higher among rural women than among urban women (13 percent and 11 percent, respectively). Across provinces, the overall unmet need for family planning is highest in Preah Vihear/Stung Treng and Kampong Cham (18 percent each) and lowest in Kampong Chhnang and Takeo (8 percent each). Whereas unmet need for spacing increases as level of education increases, unmet need for limiting is negatively associated with education. In general, unmet need decreases with increasing wealth.

The total demand for family planning rises from a low of 44 percent among women age 15-19 to a high of 80 percent among women age 35-39; and then declines to 46 percent among women age 45-49. Total demand for family planning varies inconsistently across wealth quintiles and level of education. The percentage of demand satisfied generally increases with increasing education and wealth.

---

[1] Numbers from the 2005 and 2010 CDHS surveys correspond with the revised definition of unmet need described in Bradley et al., 2012.

Table 10.3 Need and demand for family planning among currently married women

Percentage of currently married women age 15-49 with unmet need for family planning, percentage with met need for family planning, the total demand for family planning, and the percentage of the demand for contraception that is satisfied, by background characteristics, Cambodia 2014

	Unmet need for family planning			Met need for family planning (currently using)			Total demand for family planning[1]					
Background characteristic	For spacing	For limiting	Total	For spacing	For limiting	Total	For spacing	For limiting	Total	Percentage of demand satisfied[2]	Percentage of demand satisfied by modern methods[3]	Number of women
**Age**												
15-19	13.0	1.9	14.9	28.2	0.9	29.1	41.2	2.8	44.0	66.0	45.8	450
20-24	12.4	1.3	13.6	39.6	8.2	47.8	52.0	9.4	61.5	77.8	55.9	1,833
25-29	7.9	3.5	11.4	41.0	20.7	61.6	48.8	24.2	73.0	84.4	59.9	2,249
30-34	4.7	5.0	9.7	25.6	42.4	68.0	30.3	47.4	77.7	87.5	61.1	2,625
35-39	2.8	10.1	12.9	7.8	59.4	67.2	10.6	69.4	80.1	83.9	59.3	1,573
40-44	0.6	13.3	13.9	2.6	57.0	59.6	3.2	70.4	73.6	81.1	52.1	1,673
45-49	0.3	14.2	14.5	0.6	30.7	31.3	0.9	44.9	45.8	68.3	40.6	1,495
**Residence**												
Urban	5.2	5.7	10.8	24.3	35.5	59.8	29.4	41.2	70.6	84.7	46.5	1,818
Rural	5.5	7.3	12.8	21.6	34.1	55.7	27.1	41.3	68.4	81.4	58.3	10,080
**Province**												
Banteay Meanchey	5.8	3.7	9.5	26.7	34.7	61.4	32.4	38.4	70.9	86.6	72.0	503
Kampong Cham	7.7	9.9	17.6	18.8	25.9	44.7	26.5	35.8	62.3	71.7	44.4	1,490
Kampong Chhnang	5.3	2.8	8.2	20.6	35.5	56.0	25.9	38.3	64.2	87.3	52.2	396
Kampong Speu	2.9	6.9	9.8	28.3	37.1	65.4	31.2	44.0	75.2	87.0	54.7	843
Kampong Thom	3.6	6.5	10.1	18.5	39.9	58.4	22.0	46.5	68.5	85.2	64.3	572
Kandal	5.0	7.0	12.0	24.8	36.2	61.0	29.8	43.2	73.0	83.5	55.4	870
Kratie	4.9	7.2	12.1	21.6	26.3	47.9	26.5	33.5	60.0	79.8	51.1	359
Phnom Penh	6.3	4.4	10.7	26.3	37.0	63.3	32.6	41.4	74.0	85.5	38.8	1,084
Prey Veng	5.2	6.2	11.5	22.2	33.2	55.4	27.4	39.4	66.8	82.9	61.8	889
Pursat	6.0	7.8	13.7	19.1	31.9	51.0	25.1	39.7	64.8	78.8	61.8	425
Siem Reap	5.0	6.9	11.9	23.6	35.5	59.0	28.5	42.4	70.9	83.2	65.5	765
Svay Rieng	4.6	9.4	14.1	17.8	40.1	57.9	22.4	49.5	71.9	80.5	56.5	483
Takeo	4.2	4.1	8.3	14.7	45.4	60.1	18.9	49.5	68.4	87.9	70.4	677
Otdar Meanchey	8.0	6.5	14.6	22.9	33.7	56.6	31.0	40.2	71.2	79.5	69.9	218
Battambang/Pailin	5.4	7.1	12.5	24.3	34.0	58.3	29.7	41.0	70.8	82.3	57.3	890
Kampot/Kep	5.4	10.2	15.6	17.3	36.5	53.8	22.7	46.7	69.4	77.5	55.2	574
Preah Sihanouk/ Koh Kong	4.1	7.6	11.7	26.2	34.4	60.6	30.3	42.0	72.3	83.8	57.4	266
Preah Vihear/ Stung Treng	5.2	12.8	17.9	18.4	23.6	42.0	23.5	36.4	59.9	70.1	58.2	314
Mondul Kiri/ Ratanak Kiri	7.9	6.2	14.1	24.0	26.0	50.0	31.9	32.2	64.1	78.0	66.7	281
**Education**												
No education	3.9	9.8	13.7	15.1	36.9	52.0	19.0	46.7	65.7	79.2	60.7	1,774
Primary	5.3	7.9	13.1	20.0	36.4	56.5	25.3	44.3	69.6	81.1	57.1	6,399
Secondary and higher	6.4	4.3	10.7	28.7	29.3	58.1	35.2	33.6	68.8	84.4	53.3	3,726
**Wealth quintile**												
Lowest	6.4	10.7	17.0	19.9	32.8	52.7	26.2	43.5	69.8	75.6	56.7	2,294
Second	4.9	6.3	11.2	22.2	33.2	55.4	27.0	39.6	66.6	83.2	63.7	2,404
Middle	6.1	7.4	13.5	20.9	32.8	53.6	27.0	40.2	67.1	79.9	57.1	2,365
Fourth	5.0	5.8	10.8	21.7	36.1	57.7	26.6	41.9	68.5	84.2	57.3	2,393
Highest	4.9	5.2	10.1	25.4	36.4	61.8	30.3	41.6	71.8	86.0	48.2	2,443
Total	5.4	7.0	12.5	22.0	34.3	56.3	27.5	41.3	68.8	81.9	56.4	11,898

Note: Numbers in this table correspond to the revised definition of unmet need described in Bradley et al., 2012.
[1] Total demand is the sum of unmet need and met need
[2] Percentage of demand satisfied is met need divided by total demand
[3] Modern methods include female sterilization, male sterilization, pill, IUD, injectables, implants, male condom, female condom, and lactational amenorrhea method (LAM)

10.3 IDEAL FAMILY SIZE

Information on ideal family size was collected in two ways. Respondents who had no living children were asked how many children they would like to have if they could choose the number of children to have. Respondents with children were asked how many children they would like to have if they could go back to the time when they did not have any children and could choose exactly the number of children to have. Although these questions are based on hypothetical situations, they give an idea of the total number of children women who have not started childbearing will have in the future, and, among older and high parity women, these data provide a measure of the level of unwanted fertility.

Looking at the data for women, Table 10.4 shows that the majority of respondents were able to provide a numeric response to these questions. Two percent of women gave nonnumeric responses such as “any number,” “depends on fate,” or “do not know.” Among women with no living children, 52 percent

would like to have two children, 26 percent would like to have three children, and 12 percent would like to have four. Only 2 percent of women with no living children want five or more children. Mean ideal family size shows a positive association with number of living children, increasing from 2.5 children among childless women to 4.6 children among women with six or more children. The observed positive association between ideal family size and number of living children may arise for several possible reasons. First, women may tend to rationalize their family size by reporting their actual number of children as their ideal number, or, second, they may have achieved their preferred number of children. A third possibility is that there has been a decrease in the ideal family size among the youngest cohorts. Nevertheless, the results indicate a considerable level of unwanted fertility. For example, among women with six or more children, 71 percent said they would ideally have liked to have fewer. Among those with five children, almost half reported an ideal number of children less than five. The average ideal family size among all women who gave numeric responses is 3.1, whereas it is 3.3 children among currently married women. The data on ideal family size reported by men follow a pattern similar to that seen among women.

Table 10.4 Ideal number of children

Percent distribution of women and men age 15-49 by ideal number of children, and mean ideal number of children for all respondents and for currently married respondents, according to number of living children, Cambodia 2014

	Number of living children[1]							
Ideal number of children	0	1	2	3	4	5	6+	Total
WOMEN								
0	3.0	0.0	0.0	0.5	0.2	0.6	0.2	1.0
1	2.7	3.8	0.5	0.8	0.3	0.3	0.2	1.8
2	51.6	50.4	37.1	13.1	11.4	8.2	5.7	35.9
3	25.8	26.0	30.7	38.7	10.9	17.0	14.6	26.6
4	11.6	15.3	23.9	30.0	56.2	21.4	29.4	22.4
5	2.0	3.5	6.2	13.1	13.4	37.5	20.6	7.9
6+	0.2	0.4	1.3	2.8	6.6	12.4	25.0	2.8
Non-numeric responses	3.2	0.6	0.3	1.0	1.0	2.6	4.2	1.6
Total	100.0	100.0	100.0	100.0	100.0	100.0	100.0	100.0
Number	5,235	3,236	3,726	2,477	1,519	773	611	17,578
**Mean ideal number of children for:**[2]								
All women	2.5	2.7	3.0	3.5	4.0	4.3	4.6	3.1
Number of all women	5,069	3,217	3,714	2,452	1,504	753	585	17,295
Currently married women	2.7	2.6	3.0	3.5	4.0	4.3	4.6	3.3
Number of currently married women	671	2,812	3,444	2,263	1,377	694	539	11,800
MEN								
0	0.6	0.0	0.4	0.6	0.6	1.5	0.3	0.5
1	1.9	2.0	0.6	0.5	0.6	0.0	0.7	1.3
2	43.6	45.2	33.6	10.6	7.7	6.4	3.8	32.0
3	33.3	33.7	31.2	38.9	9.0	21.6	16.5	30.8
4	14.5	14.7	24.4	35.3	55.6	18.4	19.3	22.7
5	2.9	2.7	7.3	9.2	16.2	26.6	15.1	7.0
6+	0.4	0.9	1.3	3.9	8.1	17.8	39.6	3.6
Non-numeric responses	2.8	0.8	1.2	1.0	2.3	7.6	4.7	2.1
Total	100.0	100.0	100.0	100.0	100.0	100.0	100.0	100.0
Number	1,926	788	1,024	674	407	226	147	5,190
**Mean ideal number of children for:**[2]								
All men	2.7	2.7	3.1	3.5	4.1	4.3	5.2	3.1
Number of all men	1,873	782	1,011	667	398	209	140	5,079
Currently married men	2.7	2.7	3.1	3.5	4.1	4.3	5.2	3.4
Number of currently married men	215	748	989	662	391	203	140	3,348

[1] The number of living children includes one additional child if a female respondent or a male respondent's wife is pregnant (or if any wife is pregnant for men with more than one current wife).
[2] Means are calculated excluding respondents who gave non-numeric responses.

The mean ideal number of children for all women by five-year age groups and background characteristics is shown in Table 10.5. The mean ideal number of children increases gradually with increasing age, from 2.5 children among women age 15-19 to 3.9 children among women age 45-49. The mean ideal number of children among rural women is somewhat higher than among their urban counterparts (3.1 children versus 2.8 children). Women in Phnom Penh have the lowest mean ideal number of children (2.8 children), and women in Mondul Kiri/Ratanak Kiri have the highest (3.4 children). Mean ideal family size decreases with increasing education and wealth.

10.4 Fertility Planning

The 2014 CDHS provides an opportunity to estimate levels of unwanted fertility. Unwanted fertility can be estimated in one of two ways. Women were asked a series of questions about each of their children born in the five years preceding the survey, as well as any current pregnancy, to determine whether the pregnancy was wanted then (planned), wanted later (mistimed), or not wanted (unplanned) at the time of conception. This information may underestimate unplanned childbearing given that women may rationalize unplanned births and declare them as planned once they occur. Another way of measuring unwanted fertility utilizes the data on ideal family size to calculate what the total fertility rate would be if all unwanted births were avoided. This measure may also suffer from underestimation to the extent that women are unwilling to report an ideal family size lower than their actual family size.

Table 10.6 shows that 6 percent of births in the five years preceding the survey were not wanted, down from 9 percent of births in the 2010 CDHS. Ten percent of births were mistimed (wanted later), an increase from 7 percent in 2010. The proportion of unwanted births rises with birth order, increasing from less than 1 percent among first-order births to 2 percent among second-order births, 7 percent among third-order births, and, finally, 25 percent among fourth- and higher-order births. The percentage of unwanted births also increases with mother's age.

Table 10.5 Mean ideal number of children

Mean ideal number of children for all women age 15-49 by background characteristics, Cambodia 2014

Background characteristic	Mean	Number of women[1]
**Age**		
15-19	2.5	2,776
20-24	2.6	2,994
25-29	2.8	2,824
30-34	3.1	3,024
35-39	3.4	1,819
40-44	3.6	1,998
45-49	3.9	1,860
**Residence**		
Urban	2.8	3,157
Rural	3.1	14,137
**Province**		
Banteay Meanchey	3.3	682
Kampong Cham	3.3	1,973
Kampong Chhnang	3.2	662
Kampong Speu	3.0	1,152
Kampong Thom	3.1	848
Kandal	3.0	1,328
Kratie	3.2	482
Phnom Penh	2.8	1,918
Prey Veng	3.0	1,183
Pursat	3.2	629
Siem Reap	3.2	1,126
Svay Rieng	2.9	653
Takeo	2.9	1,063
Otdar Meanchey	2.9	294
Battambang/Pailin	3.0	1,312
Kampot/Kep	3.0	767
Preah Sihanouk/ Koh Kong	3.2	411
Preah Vihear/ Stung Treng	3.3	443
Mondul Kiri/ Ratanak Kiri	3.4	369
**Education**		
No education	3.5	2,221
Primary	3.2	8,148
Secondary and higher	2.7	6,925
**Wealth quintile**		
Lowest	3.3	3,105
Second	3.2	3,275
Middle	3.1	3,328
Fourth	3.0	3,563
Highest	2.8	4,024
Total	3.1	17,295

[1] Number of women who gave a numeric response

Table 10.6 Fertility planning status

Percent distribution of births to women age 15-49 in the five years preceding the survey (including current pregnancies), by planning status of the birth, according to birth order and mother's age at birth, Cambodia 2014

	Planning status of birth					
Birth order and mother's age at birth	Wanted then	Wanted later	Wanted no more	Missing	Total	Number of births
**Birth order**						
1	93.0	6.4	0.6	0.1	100.0	3,256
2	84.9	13.0	2.1	0.0	100.0	2,369
3	79.6	12.9	7.4	0.1	100.0	1,282
4+	66.4	9.0	24.6	0.0	100.0	1,279
**Mother's age at birth**						
<20	89.7	9.9	0.4	0.0	100.0	950
20-24	87.3	10.3	2.3	0.1	100.0	2,609
25-29	85.7	10.9	3.3	0.0	100.0	2,405
30-34	81.6	9.0	9.2	0.1	100.0	1,488
35-39	72.6	5.7	21.7	0.0	100.0	516
40-44	59.3	2.9	37.7	0.0	100.0	199
45-49	*	*	*	*	100.0	20
Total	84.4	9.7	5.8	0.0	100.0	8,187

Note: An asterisk indicates that a figure is based on fewer than 25 unweighted cases and has been suppressed.

Table 10.7 shows wanted fertility rates calculated using the second approach to measuring unwanted fertility. The wanted fertility rate is computed in the same way as the total fertility rate, except that unwanted births are excluded from the numerator. In this case, unwanted births are those that exceed the number mentioned as ideal by the respondent. This rate represents the level of fertility that would have prevailed in the three years preceding the survey if all unwanted births had been prevented.

The overall wanted fertility rate is 2.4 children, a decrease from 2.6 children in the 2010 CDHS. The wanted fertility rate is about one-third of a child lower than the actual total fertility rate of 2.7 children. Overall, the gap between wanted and observed fertility is larger when the total fertility rate is still high, as can be observed by comparing figures across provinces. The gap between wanted and actual fertility is about half of a child in Preah Vihear/Stung Treng and Kratie, where the total fertility rate is 3.6, as well as in Otdar Meanchey, where the total fertility rate is 3.0.

The gap between wanted and observed fertility rates among women living in rural areas and among those living in urban areas is small (0.3 children and 0.2 children, respectively). The difference between wanted and actual fertility is almost nonexistent among women with a secondary education or higher (0.1 children). In contrast, the difference is 0.4 children among women with no education and 0.3 children among women with a primary education. The gap between wanted and observed fertility among women in the lowest wealth quintile (0.6 children) is larger than that among other women (0.2 children). This finding suggests that the poorest women are less likely to have access to modern contraceptive methods than women in the other wealth quintiles, thus resulting in difficulty in achieving their desired fertility.

Table 10.7 Wanted fertility rates

Total wanted fertility rates and total fertility rates for the three years preceding the survey, by background characteristics, Cambodia 2014

Background characteristic	Total wanted fertility rate	Total fertility rate
**Residence**		
Urban	1.9	2.1
Rural	2.6	2.9
**Province**		
Banteay Meanchey	2.6	2.8
Kampong Cham	2.9	3.3
Kampong Chhnang	2.3	2.4
Kampong Speu	2.3	2.4
Kampong Thom	2.7	2.9
Kandal	2.2	2.5
Kratie	3.0	3.6
Phnom Penh	1.7	2.0
Prey Veng	2.8	3.0
Pursat	2.8	3.1
Siem Reap	2.3	2.7
Svay Rieng	2.4	2.5
Takeo	2.2	2.4
Otdar Meanchey	2.5	3.0
Battambang/Pailin	2.6	2.9
Kampot/Kep	2.4	2.5
Preah Sihanouk/ Koh Kong	2.5	2.7
Preah Vihear/ Stung Treng	3.1	3.6
Mondul Kiri/ Ratanak Kiri	3.0	3.3
**Education**		
No education	2.9	3.3
Primary	2.8	3.1
Secondary and higher	2.2	2.3
**Wealth quintile**		
Lowest	3.2	3.8
Second	2.6	2.8
Middle	2.6	2.8
Fourth	2.2	2.4
Highest	2.0	2.2
Total	2.4	2.7

Note: Rates are calculated based on births to women age 15-49 in the period 1-36 months preceding the survey. The total fertility rates are the same as those presented in Table 5.2.

ADULT AND MATERNAL MORTALITY 11

Key Findings

- Adult mortality is much higher among men than among women (3.5 deaths and 2.0 deaths per 1,000 population, respectively).
- Maternal deaths account for 9 percent of all deaths to women age 15-49. The maternal mortality rate for the seven-year period preceding the survey was 0.15 maternal deaths per 1,000 woman-years of exposure.
- The maternal mortality ratio was 170 maternal deaths per 100,000 live births for the seven-year period preceding the survey. This ratio is lower than the ratio reported in the 2010 CDHS but is not significantly different.

Estimates of maternal mortality require comprehensive and accurate reporting of maternal deaths. Such reporting can be obtained through vital registration, longitudinal studies of pregnant women, or repeated household surveys. The 2014 CDHS is the fourth population-based national survey (following the 2000, 2005, and 2010 CDHS) to incorporate questions on maternal mortality. The CDHS asked female respondents a series of questions designed with the explicit purpose of providing the necessary information to make direct estimates of maternal mortality.

However, in order to avoid serious misinterpretation of the results of the survey, it is crucial for users of this information to understand the problems inherent in measuring maternal mortality. Direct estimates of maternal mortality rely on data on the age of surviving sisters of survey respondents, the age at death of sisters who have died, and the number of years that have passed since the death of the sisters. CDHS interviewers listed all of the brothers and sisters born to the natural mother of female respondents, in chronological order, starting with the first born. Information was then obtained on the survivorship of each of the siblings, the ages of surviving siblings, the year of death or years since death of deceased siblings, and the age at death of deceased siblings. For each sister who died at age 12 or above, the respondent was asked additional questions to determine whether the death was maternity related, that is, whether the sister was pregnant when she died, and if so, whether the sister died during childbirth, and if not, whether the sister died within six weeks of the termination of a pregnancy or childbirth. Listing all siblings in chronological order of their birth is done with the intention of improving the completeness of reporting. Collecting data on both male and female siblings also allows direct estimation of adult male and female mortality.

11.1 DATA QUALITY ISSUES

Estimation of adult and maternal mortality requires reasonably accurate reporting of the number of sisters and brothers the respondent ever had, the number who have died, and the number of sisters who died of maternity-related causes. There is no definitive procedure for establishing the completeness or accuracy of retrospective data on sibling survivorship. Table 11.1 shows the number of siblings reported by female respondents and the completeness of the reported data on survival status, current age, age at death, and years since death.

Table 11.1 Completeness of information on siblings

Number of siblings reported by female survey respondents and completeness of reported data on sibling survival status, age of living siblings, and age at death (AD) and years since death (YSD) of dead siblings (unweighted), Cambodia 2014

	Sisters		Brothers		All siblings	
	Number	Percent	Number	Percent	Number	Percent
**All siblings**	40,413	100.0	41,661	100.0	82,074	100.0
Living	35,332	87.4	34,543	82.9	69,875	85.1
Dead	5,042	12.5	7,030	16.9	12,072	14.7
Survival status unknown	39	0.1	88	0.2	127	0.2
**Living siblings**	35,332	100.0	34,543	100.0	69,875	100.0
Age reported	35,314	99.9	34,525	99.9	69,839	99.9
Age missing	18	0.1	18	0.1	36	0.1
**Dead siblings**	5,042	100.0	7,030	100.0	12,072	100.0
AD and YSD reported	5,028	99.7	7,005	99.6	12,033	99.7
Missing only AD	6	0.1	11	0.2	17	0.1
Missing only YSD	5	0.1	6	0.1	11	0.1
Missing AD and YSD	3	0.1	8	0.1	11	0.1

As a group, 2014 CDHS female respondents were able to report the survival status of more than 99 percent of their siblings; whether or not a brother or sister was alive or dead was unknown for only 0.2 percent of siblings. Sex ratio is defined as the number of males per 100 females. The sex ratio of siblings who have died is calculated as the number of brothers per 100 sisters (7,030 brothers who died compared with 5,042 sisters who died). The sex ratio of siblings who have died was 139, which is very high and may be the consequence of the higher male mortality during the Khmer Rouge period. Fighting in the post-Khmer Rouge period continued until the signing of the Paris Peace Accord in 1993; this fighting would have also contributed to the high sex ratio of dead siblings. Overall, the data on siblings are nearly complete, with age reported for 99.9 percent of living siblings and age at death and years since death reported for 99.7 percent of siblings who have died, with little difference between brothers and sisters. Rather than excluding siblings with missing information from the analysis, the information on the birth order of siblings, in conjunction with other information, is used to impute the missing data.[1]

Table 11.2 Sibship size and sex ratio of siblings

Mean sibship size and sex ratio of siblings at birth, Cambodia 2014

Age of respondent	Respondent's year of birth	Mean sibship size[1]	Sex ratio of siblings at birth[2]
45-49	1965-1969	6.3	98.4
40-44	1970-1974	6.2	98.9
35-39	1975-1979	6.5	108.6
30-34	1980-1984	6.0	100.2
25-29	1985-1989	5.8	104.0
20-24	1990-1994	5.3	106.4
15-19	1995-1999	4.8	107.0
Total		5.8	103.2

[1] Includes the respondent
[2] Excludes the respondent

[1] The imputation procedure is based on the assumption that the reported birth ordering of the siblings in the birth history is correct. The first step is to calculate birth dates. For each living sibling with a reported age and for each dead sibling with complete information on both age at death and years since death, the birth date is calculated. For a sibling missing these data, a birth date is imputed within the range defined by the birth dates of the bracketing siblings. In the case of living siblings, an age is calculated from the imputed birth date. In the case of dead siblings, if either age at death or years since death is reported, that information is combined with the birth date to provide missing information. If both pieces of information are missing, the age at death is imputed. This imputation is based on the distribution of the ages at death for those whose year of death is unreported but age at death is reported.

Another crude measure of data quality is the mean number of siblings, or the mean sibship size (Table 11.2). Sibship size is expected to decline as fertility declines over time. The monotonic decline in sibship size that would be expected to accompany declining fertility is supportive of more complete reporting of older siblings. The average sex ratio at birth of 103.2 is within the internationally accepted range of 103 to 105, indicating that, as a group, there is no serious underreporting or overreporting of brothers or sisters. However, sibling sex ratios among respondents age 40-49 are somewhat below the range, suggesting that there may be underreporting of brothers among the most senior respondents. Nonetheless, it should be kept in mind that any information that relies on recall will suffer from some degree of misreporting, especially if it pertains to deceased persons and involves events that occurred a long period of time before the survey.

11.2 ADULT MORTALITY

Because maternal mortality is a subset of adult mortality, estimates of overall adult mortality are calculated before estimates of maternal mortality. If overall adult mortality estimates display a general, stable, and plausible pattern, then credence is given to the maternal mortality estimates derived thereafter.

Direct estimates of male and female adult mortality are obtained from information collected in the sibling history. Age-specific death rates are computed by dividing the number of deaths in each age group by the total person-years of exposure in that age group during a specified reference period. In total, female respondents reported 82,074 siblings, of whom 40,413 were sisters and 41,661 were brothers (Table 11.1). Direct estimates of age-specific mortality rates for men and women are shown in Table 11.3. To minimize the impact of possible heaping on years since death ending in zero and five, direct estimates are presented for the period 0-6 years before the survey, which roughly corresponds[2] to June 2008 to December 2014. Aggregating the data over the age range 15-49 will reduce the effects of sampling variability. There are more male than female deaths in the period 0-6 years preceding the survey (616 versus 348). The male mortality rate is 3.50 deaths per 1,000 population, a figure higher than the female mortality rate of 1.96 deaths per 1,000 population.

Table 11.3 Adult mortality rates

Direct estimates of female and male mortality rates for the period 0-6 years preceding the survey, by five-year age groups, Cambodia 2014

Age	Deaths	Exposure years	Mortality rate[1]
FEMALE			
15-19	31	33,159	0.93
20-24	32	40,909	0.78
25-29	40	40,900	0.98
30-34	46	30,606	1.50
35-39	47	23,590	1.99
40-44	76	19,321	3.93
45-49	76	14,071	5.43
15-49	348	202,557	1.96[a]
MALE			
15-19	46	34,258	1.36
20-24	82	41,628	1.98
25-29	71	40,586	1.75
30-34	88	31,515	2.80
35-39	115	22,709	5.05
40-44	125	17,418	7.15
45-49	88	11,866	7.45
15-49	616	199,979	3.50[a]

Note: Exposure years are calculated using a life table technique; here, they represent the number of person-years that men or women are exposed to the probability of dying.
[1] Expressed per 1,000 population
[a] Age-adjusted rate

11.3 MATERNAL MORTALITY

Estimates of maternal mortality for the period 0-6 years before the survey are shown in Table 11.4. This period of time was chosen to reduce possible heaping of reported years since death on five-year intervals. Age-specific mortality rates are calculated by dividing the number of maternal deaths by years of exposure. To remove the effect of truncation bias (the upper boundary for eligibility in the 2014 CDHS is 49 years), the overall rate for women age 15-49 is standardized by the age distribution of the survey respondents. Maternal deaths are defined as any death that occurred during pregnancy, childbirth, or within six weeks after the birth or termination of a pregnancy. This time-specific definition includes all deaths occurring during the specified period even if the death is due to causes that are not pregnancy related. However, this definition is unlikely to result in overreporting of maternal deaths because most deaths to women in the specified period are due to maternal causes, and maternal deaths in general are more likely to

[2] The time period is not exact because, as with all DHS calculations of exposure time, exposure is calculated separately for each respondent, counting back in time from the date of the interview, and dates of interview in the 2014 CDHS spanned a period of six months.

be underreported than overreported. For any given age group, maternal deaths are a relatively rare occurrence, and as such the age-specific pattern should be interpreted with caution.

There were 32 maternal deaths reported by survey respondents in the period 0-6 years preceding the survey. During the period 2008-2014, the maternal mortality rate, which is the annual number of maternal deaths per 1,000 women age 15-49, was 0.15. Maternal deaths accounted for 9 percent of all deaths to women age 15-49; in other words, about 1 in 11 Cambodian women who died in the period 0-6 years preceding the survey died as a result of pregnancy or pregnancy-related causes. Maternal deaths accounted for a similar proportion of overall female deaths as they had in the 2010 CDHS.

The maternal mortality ratio, obtained by dividing the age-standardized maternal mortality rate by the age-standardized general fertility rate, is often considered a more useful measure of maternal mortality because it measures the obstetric risk associated with each live birth. Table 11.4 shows that the maternal mortality ratio for Cambodia for the period 2008-2014 was 170 deaths per 100,000 live births (or, alternatively, 1.70 deaths per 1,000 live births). The 95 percent confidence interval of this estimate ranges from 95 to 246 deaths per 100,000 live births. The maternal mortality ratio can be converted to an estimate of the lifetime risk of dying from maternal causes: 0.005 or, in other words, a risk of dying of 1 in 200.

Table 11.4 Maternal mortality

Direct estimates of maternal mortality rates for the period 0-6 years preceding the survey, by five-year age groups, Cambodia 2014

Age	Percentage of female deaths that are maternal	Maternal deaths	Exposure years	Maternal mortality rate[1]
15-19	0.0	0	33,159	0.00
20-24	13.2	4	40,909	0.10
25-29	27.1	11	40,901	0.27
30-34	10.5	5	30,606	0.16
35-39	16.1	8	23,590	0.32
40-44	2.8	2	19,321	0.11
45-49	3.0	2	14,071	0.16
15-49	9.1	32	202,557	0.15[a]
General fertility rate (GFR)[2]	89			
Maternal mortality ratio (MMR)[3]	170 (±2 SE; CI = 95, 246)			
Lifetime risk of maternal death[4]	0.005			

CI = Confidence interval
[1] Expressed per 1,000 woman-years of exposure
[2] Expressed per 1,000 women age 15-49
[3] Expressed per 100,000 live births; calculated as the age-adjusted maternal mortality rate times 100 divided by the age-adjusted general fertility rate
[4] Calculated as $1-(1-MMR)^{TFR}$, where TFR represents the total fertility rate for the seven years preceding the survey
[a] Age-adjusted rate

A comparison of the maternal mortality ratios from the 2005, 2010, and 2014 CDHS shows a large decline between 2005 and 2010 but a small decline from 2010 to 2014. Although the decline between 2010 and 2014 is far too slight to be significant because of the overlapping confidence intervals of these two data points (Figure 11.1), the results provide confirmation that the maternal mortality ratio has declined over the past decade.

Figure 11.1 Confidence intervals for maternal mortality rates, Cambodia 2005, 2010, and 2014

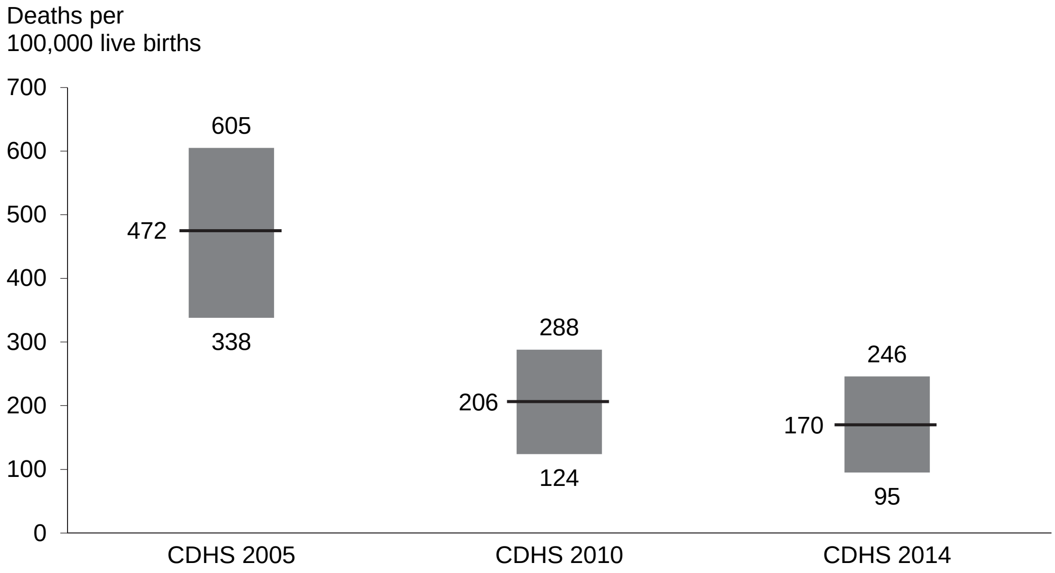

INFANT AND CHILD MORTALITY 12

Key Findings

- One in every 36 Cambodian children die before their first birthday, and one in every 29 do not survive to their fifth birthday.
- Infant mortality declined from 45 deaths to 28 deaths per 1,000 live births between the 2010 CDHS and the 2014 CDHS.
- Under-5 mortality declined from 54 deaths per 1,000 live births to 35 deaths per 1,000 live births between the two survey periods.
- Childhood mortality is higher in rural areas than in urban areas. Mortality rates are lowest among children living in the richest households.
- Neonatal and postneonatal mortality rates are 18 deaths per 1,000 live births and 10 deaths per 1,000 live births, respectively. The perinatal mortality rate is 20 deaths per 1,000 pregnancies.

This chapter describes levels of and trends in neonatal, postneonatal, infant, and child mortality in Cambodia. Infant and child mortality rates reflect a country's socioeconomic situation as well as the quality of life of the population under study. Childhood mortality is affected by socioeconomic conditions and can vary according to the demographic characteristics of children and their mothers. Therefore, differentials in infant and child mortality by socioeconomic and demographic characteristics are also presented in this chapter.

Disaggregation of mortality indicators by economic, social, and demographic categories helps to identify groups of the population at risk. Preparation, implementation, monitoring, and evaluation of population, health, and other socioeconomic programs and policies depend to a large extent on identification of a target population. The data presented here can help identify at-risk populations and provide an indication of the current mortality situation, which can be compared with previously collected data to determine whether improvements in health and quality of life have occurred over time.

The data used to compute the childhood mortality rates presented in this chapter were derived from the birth history section of the Woman's Questionnaire. Each woman age 15-49 was asked whether she had ever given birth, and, if she had, she was asked to report the number of sons and daughters who live with her, the number who live elsewhere, and the number who have died. In addition, she was asked to provide a detailed birth history of her children in chronological order starting with the first child. Women were asked whether a birth was single or multiple, the sex of the child, the date of birth (month and year, according to either the Gregorian or the Khmer calendar system), survival status, age of the child on the date of the interview if alive, and, if not alive, the age at death of each live birth. Childhood mortality rates, expressed as deaths per 1,000 live births, are defined as follows:

Neonatal mortality: the probability of dying within the first month of life

Postneonatal mortality: the probability of dying between the first month of life and first birthday (computed as the difference between infant and neonatal mortality)

Infant mortality: the probability of dying between birth and the first birthday

Child mortality: the probability of dying between the first and the fifth birthday

Under-5 mortality: the probability of dying between birth and the fifth birthday

12.1 ASSESSMENT OF DATA QUALITY

The reliability of mortality estimates depends on sampling errors and nonsampling errors. Sampling errors are discussed in detail in Appendix B. Nonsampling errors depend on the extent to which the date of birth and age at death are accurately reported and recorded and the completeness with which child deaths are reported. Omission of births and deaths affects mortality estimates, displacement of dates of births and of deaths impacts mortality trends, and misreporting of age at death may alter the age pattern of mortality. Typically, the most serious source of nonsampling errors in a survey that collects retrospective information on births and deaths is underreporting of both births and deaths of children who are not alive at the time of the survey. It may be that mothers are generally reluctant to talk about their dead children because of the sorrow associated with any death, or they may live in a culture that discourages discussing the dead. Underreporting of births and deaths is generally more severe the further back in time an event occurred.

An unusual pattern in the distribution of births by calendar years is an indication of omission of children or age displacement. However, Table C.4 in Appendix C shows that the percentage of all births for which a month and year of birth were reported remains stable over time, ranging from 100 percent of births in 2011 to 99.6 percent of births prior to 1992. There is little difference in reporting by whether or not the child is alive (99.9 percent of births) or dead (98.8 percent of births).

Underreporting of deaths is usually assumed to be higher for deaths that occur very early in infancy. An examination of the ratios in Tables C.5 and C.6 shows no significant number of early infant deaths being omitted in the 2014 CDHS. Another problem with survey data is misreporting deaths that occur in the late postneonatal period. Such misreporting results in an underestimate of the infant mortality rate and an overestimate of the child mortality rate. Table C.6 displays some digit preferences in reported deaths at age 12 months. This age "heaping" occurred despite the care taken in the CDHS to minimize such errors by requiring that age at death be recorded in days if the death took place within one month of birth, in months if the child died within 24 months of birth, and in years if the child died between age 2 and 5.

Omissions can also be detected by examining the proportion of neonatal deaths that occur during the first week of life and the proportion of infant deaths that take place during the first month of life. If there is substantial underreporting of deaths, the result would be an abnormally low ratio of deaths before seven days to all neonatal deaths. Because underreporting of deaths is likely to be more common for births that occurred a long period of time before the survey, it is important to explore whether these ratios change markedly over time.

Inspection of the ratio of deaths in the first six days of life to all neonatal deaths (shown in Appendix C, Table C.5) shows that the proportion of neonatal deaths that took place in the first week of life ranges from 82 percent for deaths during the period 0-4 years before the survey to 63 percent for deaths during the period 15-19 years before the survey. There is some variation over time in the proportion of neonatal deaths to all infant deaths (shown in Appendix C, Table C.6), which ranges from 67 percent in the period 0-4 years before the survey to 34 percent during the period 15-19 years before the survey. These ratios are within acceptable limits for the levels of mortality observed during these time periods.

12.2 LEVELS AND TRENDS IN CHILDHOOD MORTALITY

Table 12.1 presents neonatal, postneonatal, infant, child, and under-5 mortality rates for five-year periods preceding the survey. Neonatal mortality in the most recent period is 18 deaths per 1,000 live births. This rate is higher than the postneonatal mortality rate (10 deaths per 1,000 live births) during the same period; that is, the risk of dying is considerably higher in the first month of life than in the next 11 months. Thus, 28 of every 1,000 babies born in Cambodia do not survive to their first birthday. Under-5 mortality in Cambodia is 35 deaths per 1,000 live births.

Table 12.1 Early childhood mortality rates

Neonatal, postneonatal, infant, child, and under-5 mortality rates for five-year periods preceding the survey, Cambodia 2014

Years preceding the survey	Neonatal mortality (NN)	Post-neonatal mortality (PNN)[1]	Infant mortality ($_1q_0$)	Child mortality ($_4q_1$)	Under-5 mortality ($_5q_0$)
0-4	18	10	28	7	35
5-9	24	24	49	12	60
10-14	24	40	63	17	79

[1] Computed as the difference between the infant and neonatal mortality rates

Trends in the childhood mortality rate can be established by comparing the results of the 2014 CDHS with the findings from the 2000, 2005, and 2010 CDHS in which data were collected using the same techniques and estimates were calculated using the same methodology. Figure 12.1 shows that infant mortality has declined gradually and substantially in the past 14 years, from 95 deaths per 1,000 live births in 2000 to 28 per 1,000 in 2014. Under-5 mortality also declined during this period, from 124 deaths per 1,000 live births in 2000 to 35 per 1,000 in 2014.

Figure 12.1 **Trends in childhood mortality, 2000-2014**

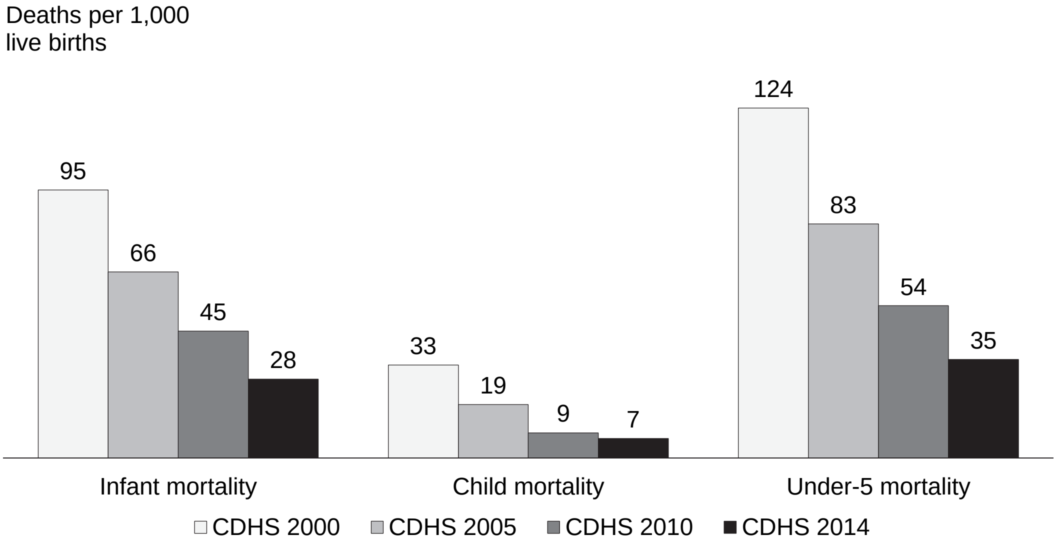

12.3 SOCIOECONOMIC DIFFERENTIALS IN CHILDHOOD MORTALITY

The results presented in Table 12.2 and Figure 12.2 show that childhood mortality in Cambodia varies significantly by the socioeconomic characteristics of households and mothers.[1] Mortality in urban areas is consistently lower than in rural areas. For example, infant mortality and under-5 mortality in rural areas (42 deaths and 52 deaths per 1,000 live births, respectively) are about three times higher than in urban areas (13 deaths and 18 deaths per 1,000 live births, respectively). The urban-rural gap is wider for postneonatal mortality, which is five times higher in rural areas than in urban areas. Differentials in mortality by province are also substantial. Phnom Penh has the lowest rates of both infant mortality (17 deaths per 1,000 live births) and under-5 mortality (23 deaths per 1,000 live births). Preah Vihear/Stung Treng and Mondul Kiri/Ratanak Kiri have the highest rates of infant mortality (70 or more deaths per 1,000 live births), and Kratie, Preah Vihear/Stung Treng, and Mondul Kiri/Ratanak Kiri have the highest rates of under-5 mortality (79 or more deaths per 1,000 live births).

[1] To have a sufficient number of cases to ensure statistically reliable mortality estimates, rates presented in Tables 12.2 and 12.3 are calculated for a 10-year period.

Table 12.2 Early childhood mortality rates by socioeconomic characteristics

Neonatal, postneonatal, infant, child, and under-5 mortality rates for the 10-year period preceding the survey, by background characteristics, Cambodia 2014

Background characteristic	Neonatal mortality (NN)	Post-neonatal mortality (PNN)[1]	Infant mortality ($_1q_0$)	Child mortality ($_4q_1$)	Under-5 mortality ($_5q_0$)
**Residence**					
Urban	10	4	13	5	18
Rural	23	20	42	10	52
**Province**					
Banteay Meanchey	20	9	29	3	32
Kampong Cham	25	14	39	9	48
Kampong Chhnang	27	23	50	6	55
Kampong Speu	19	7	26	5	31
Kampong Thom	29	11	41	20	60
Kandal	17	14	30	10	40
Kratie	30	31	61	20	80
Phnom Penh	13	4	17	6	23
Prey Veng	33	31	64	11	75
Pursat	14	17	31	5	36
Siem Reap	17	23	40	16	56
Svay Rieng	20	26	46	18	63
Takeo	16	12	28	4	31
Otdar Meanchey	17	15	32	9	41
Battambang/Pailin	12	16	28	9	37
Kampot/Kep	20	17	38	6	44
Preah Sihanouk/ Koh Kong	20	15	35	7	42
Preah Vihear/ Stung Treng	25	45	70	9	79
Mondul Kiri/ Ratanak Kiri	36	36	72	9	80
**Mother's education**					
No education	22	41	63	18	79
Primary	22	15	37	9	46
Secondary	19	6	26	5	30
**Wealth quintile**					
Lowest	27	35	62	15	76
Second	23	22	44	13	56
Middle	24	9	33	8	41
Fourth	18	9	27	6	33
Highest	12	4	16	3	19

[1] Computed as the difference between the infant and neonatal mortality rates

Figure 12.2 Infant mortality rates by socioeconomic characteristics

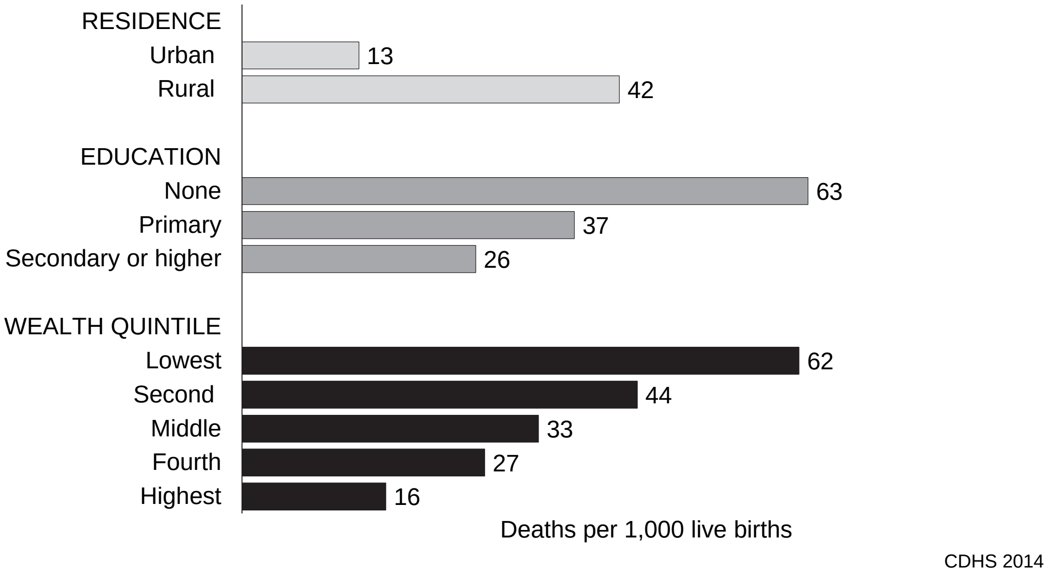

As expected, mortality declines markedly as mother's education increases. Children born to mothers with no schooling have the highest mortality rates. According to the survey results, the infant mortality rate among children of mothers with a secondary education or higher is 26 deaths per 1,000 live births, much lower than the rate of 63 deaths per 1,000 live births among children of mothers with no schooling.

In addition, mortality declines markedly as the wealth of the household increases. Children born in poorer households suffer higher mortality than those born in wealthier households. For example, infant and under-5 mortality rates are approximately four times higher among children living in the poorest households than among those living in the wealthiest households.

12.4 DEMOGRAPHIC DIFFERENTIALS IN MORTALITY

Infant and child mortality varies substantially by the demographic characteristics of mothers and children. Table 12.3 shows childhood mortality rates by selected demographic variables. Childhood mortality rates are higher among male children than among female children during all periods of life before age 5. This excess mortality among boys is not observed only in Cambodia but is a universal phenomenon.

Table 12.3 Early childhood mortality rates by demographic characteristics

Neonatal, postneonatal, infant, child, and under-5 mortality rates for the 10-year period preceding the survey, by demographic characteristics, Cambodia 2014

Demographic characteristic	Neonatal mortality (NN)	Post-neonatal mortality (PNN)[1]	Infant mortality ($_1q_0$)	Child mortality ($_4q_1$)	Under-5 mortality ($_5q_0$)
**Child's sex**					
Male	22	22	44	10	54
Female	20	13	33	9	41
**Mother's age at birth**					
<20	20	10	31	13	44
20-29	17	14	31	7	38
30-39	27	23	50	14	64
40-49	(60)	(68)	(128)	*	*
**Birth order**					
1	20	11	31	9	39
2-3	17	13	30	7	36
4-6	22	28	50	17	66
7+	76	71	147	15	160
**Previous birth interval[2]**					
<2 years	42	44	87	15	100
2 years	19	20	39	13	52
3 years	9	16	25	9	34
4+ years	21	14	35	6	41
**Birth size[3]**					
Small/very small	63	20	82	*	*
Average or larger	10	8	17	na	na

Note: Figures in parentheses are based on 250-499 unweighted person-years of exposure to the risk of death. An asterisk indicates that a figure is based on fewer than 250 unweighted person-years of exposure to the risk of death and has been suppressed.
na = Not available
[1] Computed as the difference between the infant and neonatal mortality rates
[2] Excludes first-order births
[3] Rates for the five-year period before the survey

In general infant mortality increases as the age of the mother at birth increases. The distribution of under-5 mortality by maternal age at birth is a U-shaped curve, being higher among children born to mothers under age 20 and over age 30 than among children born to mothers in the middle age groups. Relationships between infant mortality and specific demographic characteristics are illustrated in Figure 12.3.

First-order births appear to be at the same risk of mortality as second- or third-order births, whereas the risk increases for births of order four to six. However, significant increases in risk are most apparent for births of order seven and higher. Infant mortality rates for children of a seventh or higher birth order are nearly three times the rates for children of a fourth to sixth birth order.

Figure 12.3 Infant mortality rates by demographic characteristics

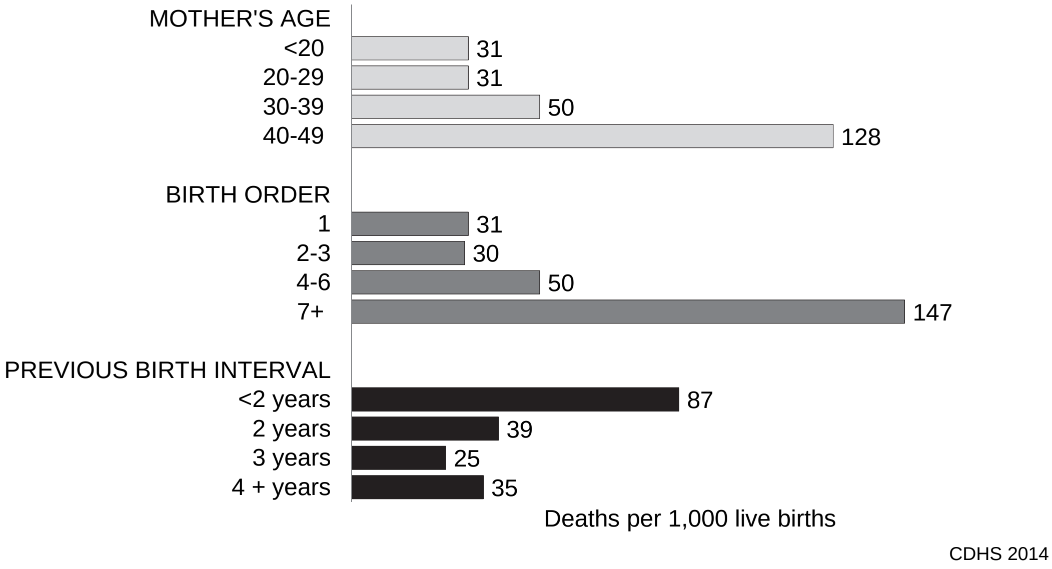

Short birth interval is one of the risk factors for childhood mortality. For example, children born less than two years after a preceding birth are more than twice as likely to die within the first month of life as children born after a two-year interval (42 deaths per 1,000 live births versus 19 per 1,000). There is a similar relationship between short birth interval and postneonatal mortality, infant mortality, and under-5 mortality; mortality rates for children born less than two years after a preceding birth are approximately twice as high as those for children born two or more years after a preceding birth.

Studies have demonstrated that children's weight at birth is an important determinant of their survival chances. Actual birth weights were unavailable for most children; instead, mothers were asked whether their child was very large, larger than average, average, smaller than average, or very small at birth, because this has been found to be a good proxy for a child's weight at birth. Those children reported by their mother to be small or very small were six times more likely to die before age 1 month than those reported to be average or larger.

12.5 PERINATAL MORTALITY

Perinatal deaths include pregnancy losses occurring after seven completed months of gestation (stillbirths) and deaths within the first seven days of life (early neonatal deaths). The perinatal death rate is calculated by dividing the total number of perinatal deaths by the total number of pregnancies reaching seven months of gestation. The distinction between a stillbirth and an early neonatal death may be a fine one, depending often on the observed presence or absence of some faint signs of life after delivery.

The causes of stillbirths and early neonatal deaths overlap, and examining just one or the other can understate the true level of mortality around delivery. For these reasons, both events are usually combined and examined together. Information on stillbirths for the five years preceding the survey was derived from the calendar at the end of the Woman's Questionnaire.

Table 12.4 presents the number of stillbirths, the number of early neonatal deaths, and perinatal mortality rates for the five-year period preceding the 2014 CDHS, by selected demographic and socioeconomic characteristics. The perinatal mortality rate in Cambodia is 20 deaths per 1,000 pregnancies. The perinatal mortality rate is highest among children whose mothers were age 40-49 (73 deaths per 1,000 pregnancies) and for pregnancies that occurred fewer than 15 months after the previous pregnancy (28 deaths per 1,000 pregnancies). Perinatal mortality is higher in rural areas than in urban areas.

Table 12.4 Perinatal mortality

Number of stillbirths and early neonatal deaths, and the perinatal mortality rate for the five-year period preceding the survey, by background characteristics, Cambodia 2014

Background characteristic	Number of stillbirths[1]	Number of early neonatal deaths[2]	Perinatal mortality rate[3]	Number of pregnancies of 7+ months' duration
**Mother's age at birth**				
<20	4	6	12	818
20-29	18	55	16	4,483
30-39	19	27	25	1,796
40-49	2	12	73	198
**Previous pregnancy interval in months[4]**				
First pregnancy	11	40	20	2,562
<15	7	21	28	999
15-26	6	14	20	973
27-38	8	3	14	832
39+	10	22	17	1,929
**Residence**				
Urban	3	4	7	1,044
Rural	39	96	22	6,251
**Province**				
Banteay Meanchey	3	1	15	256
Kampong Cham	5	25	30	1,013
Kampong Chhnang	0	5	22	248
Kampong Speu	3	7	20	471
Kampong Thom	4	6	29	341
Kandal	8	6	27	530
Kratie	1	3	14	270
Phnom Penh	3	7	15	629
Prey Veng	0	9	19	499
Pursat	4	2	20	302
Siem Reap	2	6	16	489
Svay Rieng	3	4	26	264
Takeo	2	5	16	388
Otdar Meanchey	1	0	5	137
Battambang/Pailin	2	6	13	555
Kampot/Kep	1	2	10	277
Preah Sihanouk/ Koh Kong	2	2	21	170
Preah Vihear/ Stung Treng	0	2	9	239
Mondul Kiri/ Ratanak Kiri	1	3	18	218
**Mother's education**				
No education	2	10	11	1,019
Primary	28	55	22	3,823
Secondary	10	34	20	2,251
**Wealth quintile**				
Lowest	7	33	22	1,778
Second	9	18	18	1,462
Middle	10	18	20	1,372
Fourth	10	22	26	1,262
Highest	7	10	12	1,422
Total	42	100	20	7,295

[1] Stillbirths are fetal deaths in pregnancies lasting seven or more months.
[2] Early neonatal deaths are deaths at age 0-6 days among live-born children.
[3] The sum of the number of stillbirths and early neonatal deaths divided by the number of pregnancies of seven or more months' duration, expressed per 1,000
[4] Categories correspond to birth intervals of <24 months, 24-35 months, 36-47 months, and 48+ months.

12.6 HIGH-RISK FERTILITY BEHAVIOR

The survival of infants and children depends in part on the demographic and biological characteristics of their mothers. Typically, the probability of dying in infancy is much greater among children born to mothers who are too young (under age 18) or too old (over age 34), children born after a short birth interval (less than 24 months after the preceding birth), and children born to mothers of high parity (more than three children). The risk is elevated when a child is born to a mother who has a combination of these risk characteristics.

Table 12.5 shows the percent distribution of children born in the five years before the survey by these risk factors. Nearly 2 in 5 births (37 percent) were not in any high-risk category. Thirty-six percent were first births to women between age 18 and 34—considered an unavoidable risk category—whereas 20 percent of births were in a single high-risk category and only 8 percent were in a multiple high-risk category. The most common single high-risk category was births of order three and above (8 percent), and the most common multiple high-risk category was births to mothers older than age 34 and of birth order three and above (6 percent).

Table 12.5 High-risk fertility behavior

Percent distribution of children born in the five years preceding the survey by category of elevated risk of mortality and the risk ratio, and percent distribution of currently married women by category of risk if they were to conceive a child at the time of the survey, Cambodia 2014

Risk category	Births in the 5 years preceding the survey: Percentage of births	Births in the 5 years preceding the survey: Risk ratio	Percentage of currently married women[1]
Not in any high-risk category	36.6	1.00	30.0[a]
**Unavoidable risk category**			
First-order births between age 18 and 34	35.7	1.45	7.6
**Single high-risk category**			
Mother's age <18	2.7	1.22	0.4
Mother's age >34	2.9	1.35	10.0
Birth interval <24 months	5.8	2.34	10.1
Birth order >3	8.3	1.40	8.2
Subtotal	19.8	1.64	28.6
**Multiple high-risk category**			
Age <18 and birth interval <24 months[2]	0.0	*	0.1
Age >34 and birth interval <24 months	0.2	*	0.5
Age >34 and birth order >3	5.6	3.65	29.0
Age >34 and birth interval <24 months and birth order >3	0.5	(8.80)	1.2
Birth interval <24 months and birth order >3	1.6	5.64	3.0
Subtotal	7.8	4.42	33.7
In any avoidable high-risk category	27.6	2.43	62.3
Total	100.0	na	100.0
Number of births/women	7,253	na	11,898

Note: Risk ratio is the ratio of the proportion dead among births in a specific high-risk category to the proportion dead among births not in any high-risk category. Figures in parentheses are based on 25-49 unweighted cases. An asterisk indicates that a ratio is based on fewer than 25 unweighted cases and has been suppressed.
na = Not applicable
[1] Women are assigned to risk categories according to the status they would have at the birth of a child if they were to conceive at the time of the survey: current age less than 17 years and 3 months or older than 34 years and 2 months, latest birth less than 15 months ago, or latest birth being of order 3 or higher.
[2] Includes the category age <18 and birth order >3
[a] Includes sterilized women

The risk ratios displayed in the second column of Table 12.5 denote the relationship between risk factors and mortality. For example, the risk of dying for a child who falls into any of the avoidable high-risk categories is 2.4 times higher than for a child not in any high-risk category. In general, risk ratios are higher for children in a multiple high-risk category than for children in a single high-risk category. Most vulnerable are children born to a mother older than age 34, born less than 24 months after a preceding birth, and of a birth order greater than three; they are nine times as likely to die as children who are not in any high-risk category. However, less than 1 percent of births fall into this category. Among the single high-risk categories, children born after a birth interval shorter than 24 months have 2.3 times the risk of dying of children not in any high-risk category.

The final column of Table 12.5 illustrates the potential currently married women have of experiencing a high-risk birth. A woman's status at the time of the survey with regard to her age, time elapsed since the last birth, and parity is used to classify her into a potential risk category if she were to

become pregnant at the time of the survey. For example, if a respondent who is age 40, has had four births, and had her last birth 12 months ago were to become pregnant, she would fall into the multiple high-risk category of being too old, too high parity (four or more births), and giving birth too soon (less than 24 months) after a previous birth.

Overall, approximately 3 in 5 currently married women (62 percent) have the potential of giving birth to a child at elevated risk of mortality. Twenty-nine percent of women have the potential for having a birth in a single high-risk category, and about one-third of women (34 percent) have the potential for having a birth in a multiple high-risk category (mainly older maternal age and high birth order).

MATERNAL HEALTH 13

> **Key Findings**
> - More than 9 in 10 (95 percent) mothers received antenatal care from a skilled provider.
> - The median duration of pregnancy at the first antenatal visit is 2.5 months.
> - Eighty-nine percent of mothers with a birth in the five years preceding the survey were protected against neonatal tetanus.
> - Nine in 10 (89 percent) births in the five years preceding the survey were assisted by a skilled provider, and 83 percent of births were delivered in a health facility.
> - In the two years before the survey, 90 percent of women received postnatal care for their last birth in the first two days after delivery.

This chapter presents findings on important areas of maternal health: antenatal, delivery, and postnatal care. This information, in combination with data from other chapters, is useful in formulating programs and policies to improve maternal and child health services.

13.1 ANTENATAL CARE

The health care that a mother receives during pregnancy and at the time of delivery is important for the survival and well-being of both the mother and the child. Antenatal care (ANC) from a trained provider is vital in monitoring the pregnancy and reducing morbidity risk for the mother and child during pregnancy and delivery. A well-designed and well-implemented ANC program facilitates detection and treatment of problems during pregnancy, such as anemia and infections, and provides an opportunity to disseminate health messages to women and their families. In the 2014 CDHS, women who had given birth in the five years preceding the survey were asked about the type of ANC provider, number of ANC visits, stage of pregnancy at the time of the first visit, and services and information provided during ANC. For women with two or more live births during the five-year period, data on antenatal care refer to the most recent birth only.

13.1.1 Source of Antenatal Care

Table 13.1 shows the percent distribution of women who had a birth in the five years preceding the survey by source of antenatal care received during pregnancy. Ninety-five percent of women received ANC from trained personnel (doctors, nurses, and midwives) at least once. Nearly 9 in 10 women (88 percent) received care during pregnancy from midwives, 6 percent received care from a doctor, and 1 percent received care from a nurse. Only 5 percent of women received no antenatal care for births in the preceding five years. The 2014 data show continued improvement in antenatal care since the 2010 CDHS, when 89 percent of women had received antenatal care from a trained health professional. In 2010, one-tenth of women received no antenatal care.

Younger women (less than age 35) were more likely than older women (age 35 and older) to receive antenatal care from trained personnel (96 percent versus 89 percent). Women were more likely to receive care from a health professional for first births (98 percent) than for births of order six and higher (72 percent). Urban and rural women differed slightly in their use of antenatal care services. Health professionals provided antenatal care for 99 percent of women in urban areas and 95 percent of women in rural areas. Five percent of women in rural areas received no antenatal care at all, as compared with 1 percent in urban areas.

Table 13.1 Antenatal care

Percent distribution of women age 15-49 who had a live birth in the five years preceding the survey by antenatal care (ANC) provider during pregnancy for the most recent birth and the percentage receiving antenatal care from a skilled provider for the most recent birth, according to background characteristics, Cambodia 2014

Background characteristic	Antenatal care provider									Percentage receiving antenatal care from a skilled provider[1]	Number of women
	Doctor	Nurse	Midwife	Traditional birth attendant	Village health volunteer	Other	No one	Missing	Total		
**Mother's age at birth**											
<20	5.5	0.2	89.8	0.0	0.1	0.0	4.4	0.0	100.0	95.5	620
20-34	5.9	1.4	88.8	0.1	0.0	0.0	3.7	0.0	100.0	96.2	4,749
35-49	4.5	1.1	83.0	0.4	0.0	0.0	11.0	0.0	100.0	88.6	603
**Birth order**											
1	7.0	1.2	90.1	0.0	0.0	0.0	1.6	0.0	100.0	98.4	2,127
2-3	4.9	1.6	90.0	0.1	0.1	0.0	3.2	0.1	100.0	96.5	2,826
4-5	5.8	0.3	84.6	0.3	0.0	0.0	9.0	0.0	100.0	90.7	748
6+	3.9	0.7	67.3	0.8	0.0	0.0	27.2	0.0	100.0	71.9	272
**Place where ANC received**											
Public sector	5.0	1.3	93.6	0.0	0.0	0.0	0.0	0.0	100.0	99.9	5,366
Private sector	24.8	1.7	73.5	0.0	0.0	0.0	0.0	0.0	100.0	100.0	274
Home	(11.8)	(0.0)	(80.8)	(6.3)	(1.2)	(0.0)	(0.0)	(0.0)	100.0	(92.5)	53
Other	*	*	*	*	*	*	*	*	100.0	*	12
No ANC	0.0	0.0	0.0	0.0	0.0	0.0	100.0	0.0	100.0	0.0	267
**Residence**											
Urban	9.1	4.0	85.5	0.0	0.0	0.0	1.4	0.0	100.0	98.6	876
Rural	5.1	0.8	88.8	0.1	0.0	0.0	5.0	0.0	100.0	94.8	5,096
**Province**											
Banteay Meanchey	2.5	0.4	96.0	0.0	0.0	0.0	0.6	0.5	100.0	98.9	219
Kampong Cham	17.6	0.3	79.0	0.0	0.0	0.0	3.1	0.0	100.0	96.9	819
Kampong Chhnang	0.1	0.1	99.2	0.0	0.0	0.0	0.5	0.0	100.0	99.5	203
Kampong Speu	0.2	0.0	97.5	0.0	0.0	0.0	2.2	0.0	100.0	97.8	395
Kampong Thom	1.6	0.0	94.0	0.0	0.0	0.0	4.4	0.0	100.0	95.6	279
Kandal	1.9	2.3	92.1	0.0	0.0	0.0	3.7	0.0	100.0	96.3	420
Kratie	2.0	0.0	70.8	0.6	0.0	0.0	26.6	0.0	100.0	72.8	214
Phnom Penh	10.3	8.0	80.1	0.0	0.0	0.0	1.5	0.0	100.0	98.5	535
Prey Veng	3.8	0.0	95.1	0.0	0.0	0.0	1.0	0.0	100.0	99.0	405
Pursat	0.3	0.0	94.4	0.0	0.0	0.0	5.0	0.3	100.0	94.7	245
Siem Reap	21.6	3.2	71.3	1.3	0.4	0.0	2.2	0.0	100.0	96.1	379
Svay Rieng	0.5	0.0	97.6	0.0	0.0	0.0	1.9	0.0	100.0	98.1	229
Takeo	1.5	0.0	96.1	0.0	0.0	0.0	2.4	0.0	100.0	97.6	321
Otdar Meanchey	0.9	0.0	95.7	0.0	0.0	0.0	2.9	0.4	100.0	96.7	116
Battambang/Pailin	1.5	1.5	94.0	0.0	0.0	0.0	2.9	0.0	100.0	97.1	460
Kampot/Kep	0.7	0.1	93.1	0.0	0.0	0.0	6.1	0.0	100.0	93.9	236
Preah Sihanouk/ Koh Kong	3.4	0.2	94.1	0.0	0.0	0.0	2.4	0.0	100.0	97.6	142
Preah Vihear/ Stung Treng	0.2	0.0	85.3	0.3	0.0	0.0	14.2	0.0	100.0	85.5	188
Mondul Kiri/ Ratanak Kiri	0.3	0.0	75.7	0.0	0.4	0.0	23.7	0.0	100.0	76.0	169
**Mother's education**											
No education	4.2	1.1	81.0	0.2	0.1	0.0	13.3	0.1	100.0	86.3	805
Primary	5.1	0.8	89.4	0.2	0.0	0.0	4.4	0.1	100.0	95.3	3,100
Secondary and higher	7.2	2.1	89.5	0.0	0.1	0.0	1.1	0.0	100.0	98.8	2,068
**Wealth quintile**											
Lowest	4.4	0.5	84.8	0.4	0.0	0.0	9.9	0.0	100.0	89.7	1,359
Second	4.4	0.3	89.9	0.1	0.2	0.0	5.1	0.0	100.0	94.7	1,215
Middle	5.1	0.9	90.2	0.0	0.0	0.0	3.7	0.2	100.0	96.2	1,133
Fourth	4.4	1.0	92.7	0.1	0.0	0.0	1.9	0.0	100.0	98.1	1,069
Highest	10.4	3.8	85.1	0.0	0.0	0.0	0.7	0.0	100.0	99.3	1,196
Total	5.7	1.3	88.3	0.1	0.0	0.0	4.5	0.0	100.0	95.3	5,973

Note: If more than one source of ANC was mentioned, only the provider with the highest qualifications is considered in this tabulation. Figures in parentheses are based on 25-49 unweighted cases. An asterisk indicates that a figure is based on fewer than 25 unweighted cases and has been suppressed.
[1] Skilled provider includes doctor, nurse, and midwife.

Provincial differences in antenatal care coverage were significant. For example, while nearly all women in several provinces received antenatal care from a health professional, only about three-quarters of women in Kratie and Mondul Kiri/Ratanak Kiri received qualified antenatal care (73 percent and 76 percent, respectively).

The use of antenatal care services was strongly associated with a woman's level of education. Women with a secondary education or higher were more likely to receive antenatal care from trained

personnel (99 percent) than women with a primary education (95 percent) and women with no education (86 percent). Thirteen percent of uneducated women received no antenatal care at all, with the proportion decreasing to 4 percent among women with a primary school education and 1 percent among women with a secondary education or higher. The proportion of women who receive ANC from a skilled provider increases steadily with increasing wealth.

Antenatal care is more beneficial in preventing adverse pregnancy outcomes when it is sought early in the pregnancy and is continued throughout pregnancy. Health professionals recommend that the first antenatal visit occur within the first three months of the pregnancy and that visits continue on a monthly basis through week 28 of pregnancy and then every two weeks up to week 36 (or until birth). If the first antenatal visit is made during the third month of pregnancy and then visits occur as regularly as recommended, there will be a total of at least 12 to 13 antenatal visits. Table 13.2 shows that three-quarters of women (76 percent) make four or more antenatal care visits during their entire pregnancy. Table 13.2 includes antenatal care received from any type of provider listed in Table 13.1.

Four in five women (79 percent) make their first antenatal care visit before the fourth month of pregnancy. The median duration of pregnancy at the first antenatal care visit is 2.5 months. This indicates that, overall, women in Cambodia start antenatal care during the first trimester of their pregnancy. Rural women tend to have fewer ANC visits and to start care later in pregnancy than urban women.

Table 13.2 Number of antenatal care visits and timing of first visit

Percent distribution of women age 15-49 who had a live birth in the five years preceding the survey by number of antenatal care (ANC) visits for the most recent live birth, and by the timing of the first visit, and among women with ANC, median months pregnant at first visit, according to residence, Cambodia 2014

Number and timing of ANC visits	Residence		
	Urban	Rural	Total
**Number of ANC visits**			
None	1.4	5.0	4.5
1	1.4	3.1	2.9
2-3	11.5	17.6	16.7
4+	85.4	73.9	75.6
Don't know/missing	0.3	0.3	0.3
Total	100.0	100.0	100.0
**Number of months pregnant at time of first ANC visit**			
No antenatal care	1.4	5.0	4.5
<4	87.6	77.5	79.0
4-5	8.4	13.3	12.6
6-7	2.1	3.6	3.4
8+	0.4	0.5	0.5
Don't know/missing	0.1	0.1	0.1
Total	100.0	100.0	100.0
Number of women	876	5,096	5,973
Median months pregnant at first visit (for those with ANC)	2.1	2.6	2.5
Number of women with ANC	864	4,840	5,704

13.1.2 Components of Antenatal Care

Apart from receiving basic care, every pregnant woman should be monitored for complications. For that reason, pregnant women should receive information on pregnancy complications or danger signs and be screened for complications at all antenatal care visits. The 2014 CDHS asked respondents a number of questions about the care they received during pregnancy for their most recent live birth in the past five years. Table 13.3 presents information on the percentage of women who took iron tablets and intestinal parasite drugs during pregnancy and on the content of ANC services, including the percentage of women who were informed of the symptoms of pregnancy complications.

Nearly all women (96 percent) took iron tablets or syrup during pregnancy, and 72 percent took intestinal parasite drugs. Eighty-two percent of mothers who received antenatal care reported that they were informed about the signs of pregnancy-related complications during their visits. Blood pressure measurements were part of antenatal care for 96 percent of mothers, and 95 percent were weighed as part of their antenatal care. Urine and blood samples were taken from 49 percent and 77 percent of women, respectively.

Table 13.3 Components of antenatal care

Among women age 15-49 with a live birth in the five years preceding the survey, the percentage who took iron tablets or syrup and drugs for intestinal parasites during the pregnancy of the most recent birth, and among women receiving antenatal care (ANC) for the most recent live birth in the five years preceding the survey, the percentage receiving specific antenatal services, according to background characteristics, Cambodia 2014

	Among women with a live birth in the past five years, the percentage who during the pregnancy of their last birth:			Among women who received antenatal care for their most recent birth in the past five years, the percentage with selected services					
Background characteristic	Took iron tablets or syrup	Took intestinal parasite drugs	Number of women with a live birth in the past five years	Informed of signs of pregnancy complica-tions	Weighed	Blood pressure measured	Urine sample taken	Blood sample taken	Number of women with ANC for their most recent birth
**Mother's age at birth**									
<20	94.7	71.5	620	76.4	95.4	95.9	42.5	77.1	592
20-34	96.5	73.2	4,749	82.7	95.7	96.5	50.0	77.8	4,574
35-49	89.6	64.6	603	83.6	91.8	93.3	46.6	71.2	537
**Birth order**									
1	98.1	74.9	2,127	80.6	96.3	96.5	50.3	81.5	2,093
2-3	96.8	73.7	2,826	84.2	96.0	96.7	49.2	76.1	2,733
4-5	91.4	66.1	748	80.5	92.5	95.4	44.2	71.6	680
6+	74.9	51.0	272	75.6	85.8	86.0	46.1	63.6	198
**Residence**									
Urban	97.4	62.0	876	83.1	97.5	97.9	60.8	82.6	864
Rural	95.3	73.9	5,096	82.0	94.9	95.8	46.8	76.1	4,840
**Province**									
Banteay Meanchey	98.7	72.1	219	78.0	98.6	94.5	59.4	90.7	217
Kampong Cham	97.3	64.3	819	70.6	91.3	94.3	33.2	70.6	793
Kampong Chhnang	99.3	96.6	203	96.4	97.0	97.7	83.3	92.9	202
Kampong Speu	98.2	71.7	395	75.7	94.2	97.0	22.6	73.4	386
Kampong Thom	94.7	90.6	279	98.5	97.4	96.1	84.9	92.4	266
Kandal	95.0	71.1	420	86.4	95.7	95.5	52.8	72.3	404
Kratie	77.0	57.0	214	85.0	90.3	91.7	39.8	55.1	157
Phnom Penh	97.9	53.6	535	84.6	98.0	99.4	63.9	78.8	527
Prey Veng	98.9	84.5	405	90.1	93.0	95.1	56.7	75.9	401
Pursat	95.5	89.2	245	93.6	97.6	96.8	54.9	86.0	232
Siem Reap	96.4	74.3	379	80.3	98.4	96.1	82.9	95.8	371
Svay Rieng	98.1	70.1	229	78.7	99.2	97.9	32.7	78.9	224
Takeo	97.9	71.1	321	89.8	96.1	98.0	41.3	78.1	313
Otdar Meanchey	95.6	82.0	116	73.0	98.5	91.5	54.7	73.7	112
Battambang/Pailin	97.5	78.4	460	73.4	98.1	97.9	45.6	91.1	447
Kampot/Kep	92.8	72.7	236	81.8	93.4	94.3	21.1	79.2	222
Preah Sihanouk/ Koh Kong	96.7	66.1	142	83.5	95.9	95.2	55.6	84.2	138
Preah Vihear/ Stung Treng	90.1	59.6	188	76.3	84.1	95.4	18.4	28.8	161
Mondul Kiri/ Ratanak Kiri	77.7	72.0	169	87.5	94.8	95.1	15.1	15.6	129
**Education**									
No education	87.8	63.0	805	76.7	89.5	90.3	43.6	68.4	697
Primary	95.6	72.8	3,100	81.7	94.9	96.0	46.5	75.3	2,961
Secondary and higher	98.6	74.8	2,068	84.5	97.9	98.3	54.2	82.6	2,045
**Wealth quintile**									
Lowest	91.0	70.5	1,359	78.7	92.3	93.0	44.9	73.4	1,224
Second	95.2	71.6	1,215	81.1	91.9	94.5	47.1	74.0	1,153
Middle	96.4	76.3	1,133	83.2	97.0	97.6	44.6	77.9	1,091
Fourth	98.3	74.4	1,069	85.0	97.9	97.8	49.9	79.2	1,049
Highest	98.0	68.7	1,196	83.1	98.0	98.1	57.8	81.3	1,187
Total	95.6	72.2	5,973	82.1	95.3	96.1	48.9	77.1	5,704

Urban-rural differences existed for various components of antenatal care. Urban women and rural women were equally likely to have been informed about signs of pregnancy complications, to have been weighed, and to have their blood pressure measured; however, urban women were more likely than rural women to have blood and urine taken for testing. Women in rural areas were more likely than those in urban areas to take intestinal parasite drugs, but rural and urban were equally likely to take iron tablets or syrup during pregnancy. Antenatal care content was also greatly related to education and wealth. Women with a secondary education or higher and women in the highest wealth quintile were more likely to have received most services than other women.

13.1.3 Tetanus Toxoid Vaccinations

Tetanus toxoid (TT) injections are given to women during pregnancy to prevent deaths from neonatal tetanus. Neonatal tetanus can result when sterile procedures are not followed in cutting the umbilical cord after delivery. In the 2014 CDHS, information was collected on the number of doses of TT vaccine the mother received for her most recent birth during the five-year period prior to the survey. In addition, questions were included to ascertain whether mothers received tetanus injections prior to the last birth as a means of determining whether that birth was fully protected from neonatal tetanus.

Table 13.4 shows the percentage of women with a live birth in the five years preceding the survey who reported receiving TT injections during the pregnancy for the last live birth. Also shown is whether the last birth was fully protected against neonatal tetanus. An infant is considered to be fully protected if the mother had two tetanus toxoid injections during the pregnancy or if she had the requisite number of injections prior to the pregnancy (see footnote in Table 13.4). According to the 2014 CDHS results, 89 percent of last-born children during the five-year period before the survey were fully protected against neonatal tetanus. This figure is slightly higher than that observed in the 2010 CDHS (85 percent). There were provincial differences in the percentage of last-born children who were fully protected against neonatal tetanus. For example, 98 percent of births in Kampong Chhnang were fully protected, as compared with 72 percent of births in Mondul Kiri/Ratanak Kiri. The proportion of births protected against tetanus is higher in urban than rural areas and increases with increasing mother's education and wealth.

For approximately three in five births in the past five years (62 percent), the mother received two or more tetanus toxoid injections. This figure is similar to that reported in 2010, when 61 percent of women received two or more doses of tetanus toxoid vaccine.

Table 13.4 Tetanus toxoid injections

Among mothers age 15-49 with a live birth in the five years preceding the survey, the percentage receiving two or more tetanus toxoid injections during the pregnancy for the last live birth and the percentage whose last live birth was protected against neonatal tetanus, according to background characteristics, Cambodia 2014

Background characteristic	Percentage receiving two or more injections during last pregnancy	Percentage whose last birth was protected against neonatal tetanus[1]	Number of mothers
**Mother's age at birth**			
<20	66.7	83.1	620
20-34	62.4	90.3	4,749
35-49	58.2	80.8	603
**Birth order**			
1	72.3	89.8	2,127
2-3	57.9	90.1	2,826
4-5	55.9	87.4	748
6+	49.3	65.9	272
**Residence**			
Urban	64.2	92.9	876
Rural	62.1	87.8	5,096
**Province**			
Banteay Meanchey	70.2	93.8	219
Kampong Cham	62.9	84.6	819
Kampong Chhnang	79.5	97.9	203
Kampong Speu	53.4	90.5	395
Kampong Thom	73.6	92.7	279
Kandal	61.6	88.3	420
Kratie	50.3	83.6	214
Phnom Penh	69.0	94.3	535
Prey Veng	72.3	91.2	405
Pursat	64.5	89.8	245
Siem Reap	51.6	86.4	379
Svay Rieng	69.1	93.1	229
Takeo	79.4	93.9	321
Otdar Meanchey	62.1	90.9	116
Battambang/Pailin	47.3	79.7	460
Kampot/Kep	59.7	86.7	236
Preah Sihanouk/ Koh Kong	56.8	89.6	142
Preah Vihear/ Stung Treng	45.1	88.8	188
Mondul Kiri/ Ratanak Kiri	53.9	71.8	169
**Education**			
No education	57.0	79.8	805
Primary	60.7	87.6	3,100
Secondary and higher	67.1	93.4	2,068
**Wealth quintile**			
Lowest	57.8	83.2	1,359
Second	61.7	87.5	1,215
Middle	63.0	87.3	1,133
Fourth	64.9	92.4	1,069
Highest	65.6	93.5	1,196
Total	62.4	88.6	5,973

[1] Includes mothers with two injections during the pregnancy of their last birth, or two or more injections (the last within 3 years of the last live birth), or three or more injections (the last within 5 years of the last birth), or four or more injections (the last within 10 years of the last live birth), or five or more injections at any time prior to the last birth.

13.2 CHILDBIRTH AND DELIVERY

An important component of efforts to reduce the health risks of mothers and children is increasing the proportion of babies delivered under the supervision of health professionals. Proper medical attention and hygienic conditions during delivery can reduce the risk of complications and infections that may cause death or serious illness to either the mother or the baby (or both). Data on delivery care were obtained for all births that occurred in the five years preceding the survey.

13.2.1 Place of Delivery

More than four in five births (83 percent) in the five years before the survey were delivered in a health facility, and 17 percent were delivered at home (Table 13.5). The percentage of deliveries occurring in the home has declined dramatically from the figures reported in 2005 (78 percent) and 2010 (45 percent).

Table 13.5 Place of delivery

Percent distribution of live births in the five years preceding the survey by place of delivery and percentage delivered in a health facility, according to background characteristics, Cambodia 2014

Background characteristic	Health facility: Public sector	Health facility: Private sector	Home	Other	Missing	Total	Percentage delivered in a health facility	Number of births
**Mother's age at birth**								
<20	68.5	14.8	16.4	0.3	0.0	100.0	83.3	814
20-34	69.7	14.7	15.2	0.2	0.1	100.0	84.5	5,777
35-49	61.6	10.0	28.4	0.0	0.0	100.0	71.6	662
**Birth order**								
1	72.5	17.5	9.8	0.2	0.1	100.0	90.0	2,822
2-3	69.5	13.5	16.9	0.1	0.1	100.0	83.0	3,274
4-5	60.9	10.4	27.9	0.7	0.0	100.0	71.3	833
6+	51.5	4.6	43.6	0.0	0.3	100.0	56.1	323
**Antenatal care visits[1]**								
None	22.1	6.1	71.2	0.0	0.6	100.0	28.2	269
1-3	62.9	11.1	25.5	0.4	0.0	100.0	74.0	1,169
4+	74.3	16.4	9.2	0.1	0.0	100.0	90.7	4,516
**Residence**								
Urban	65.8	30.2	3.9	0.0	0.0	100.0	96.0	1,041
Rural	69.4	11.6	18.7	0.2	0.1	100.0	81.0	6,212
**Province**								
Banteay Meanchey	70.0	17.9	11.0	0.0	1.1	100.0	87.9	253
Kampong Cham	61.0	23.5	15.2	0.4	0.0	100.0	84.5	1,008
Kampong Chhnang	96.0	1.2	2.9	0.0	0.0	100.0	97.1	248
Kampong Speu	66.9	17.2	15.5	0.4	0.0	100.0	84.1	469
Kampong Thom	67.5	6.9	25.7	0.0	0.0	100.0	74.3	337
Kandal	63.6	17.2	19.2	0.0	0.0	100.0	80.8	523
Kratie	40.3	5.9	53.7	0.0	0.0	100.0	46.3	269
Phnom Penh	65.2	30.7	4.1	0.0	0.0	100.0	95.9	626
Prey Veng	69.4	20.5	10.0	0.0	0.0	100.0	90.0	499
Pursat	72.6	5.8	21.6	0.0	0.0	100.0	78.4	298
Siem Reap	87.0	4.6	8.4	0.0	0.0	100.0	91.6	487
Svay Rieng	74.3	8.2	17.2	0.4	0.0	100.0	82.4	261
Takeo	80.1	12.0	6.9	0.9	0.0	100.0	92.2	386
Otdar Meanchey	85.9	2.5	11.1	0.2	0.3	100.0	88.4	137
Battambang/Pailin	81.3	8.9	9.6	0.0	0.2	100.0	90.2	553
Kampot/Kep	72.2	8.7	18.2	0.8	0.0	100.0	80.9	276
Preah Sihanouk/ Koh Kong	68.6	20.4	10.2	0.0	0.9	100.0	88.9	168
Preah Vihear/ Stung Treng	49.3	1.8	48.8	0.1	0.0	100.0	51.1	239
Mondul Kiri/ Ratanak Kiri	39.3	11.9	48.1	0.7	0.0	100.0	51.2	217
**Mother's education**								
No education	62.2	5.7	31.6	0.5	0.0	100.0	67.8	1,017
Primary	70.3	10.7	18.7	0.2	0.1	100.0	81.0	3,795
Secondary and higher	69.4	23.5	7.0	0.1	0.0	100.0	92.9	2,442
**Wealth quintile**								
Lowest	65.2	3.2	31.2	0.3	0.1	100.0	68.4	1,771
Second	71.6	7.1	20.8	0.3	0.1	100.0	78.8	1,453
Middle	76.0	10.8	13.1	0.0	0.1	100.0	86.8	1,362
Fourth	72.1	18.7	9.0	0.2	0.0	100.0	90.8	1,252
Highest	60.9	35.0	3.8	0.2	0.1	100.0	95.9	1,415
Total	68.9	14.3	16.6	0.2	0.1	100.0	83.2	7,253

Note: Total includes 19 births for which the number of antenatal care visits is missing.
[1] Includes only the most recent birth in the five years preceding the survey

First births are more likely to be delivered in a health facility (90 percent) than are subsequent births. Children born in urban areas (96 percent) are more likely to be delivered in a health facility than children born in rural areas (81 percent). The proportion of births delivered in a health facility is highest in Kampong Chhnang (97 percent) and Phnom Penh (96 percent) and lowest in Kratie (46 percent), Preah Vihear/Stung Treng (51 percent), and Mondul Kiri/Ratanak Kiri (51 percent). Facility-based delivery is positively associated with mother's educational level. About two-thirds of births to women (68 percent) with no education are delivered in a health facility, as compared with 93 percent of births to women with a secondary education or higher. A similar relationship is observed between place of delivery and wealth.

13.2.2 Assistance at Delivery

Obstetric care by a trained provider during delivery is recognized as critical for the reduction of maternal and neonatal mortality. Table 13.6 shows the percent distribution of births in the five years preceding the survey by the person providing assistance at delivery, the percentage of births attended by a skilled health worker, and the percentage of births delivered by cesarean section, according to background characteristics. Eighty-nine percent of births are delivered with the assistance of a trained health professional (i.e., a doctor, nurse, or midwife), an increase from 71 percent in 2010. Only 11 percent are delivered with the assistance of a traditional birth attendant. Six percent of births are delivered via cesarean, an increase from 3 percent in 2010.

First births are more likely to be assisted by a trained health professional (94 percent) than subsequent births. Births to urban women are more likely (98 percent) to be assisted by a trained health professional than births to rural women (88 percent). Conversely, rural births are more likely (12 percent) than urban births (2 percent) to receive assistance from a traditional birth attendant. At least 80 percent of deliveries are assisted by a trained health professional in all provinces other than Kratie (52 percent), Preah Vihear/Stung Treng (55 percent), and Mondul Kiri/Ratanak Kiri (54 percent). As expected, mother's education is related to delivery care. Births to women with a primary school education (89 percent) and women with a secondary education or higher (97 percent) are more likely than births to women with no education (72 percent) to be assisted by a health professional. Household wealth is also positively associated with professionally assisted delivery.

First births and births to older women (age 35-49) are more likely to be delivered via cesarean than other births. The proportion of births delivered by cesarean section is about three times higher in urban areas than rural areas (14 percent versus 5 percent) and is highest in Phnom Penh. Births to women with a secondary education or higher and those to women in the highest wealth quintile are more likely than other births to be delivered via cesarean.

Table 13.6 Assistance during delivery

Percent distribution of live births in the five years preceding the survey by person providing assistance during delivery, percentage of births assisted by a skilled provider, and percentage delivered by cesarean section, according to background characteristics, Cambodia 2014

Background characteristic	Person providing assistance during delivery								Percentage delivered by a skilled provider[1]	Percentage delivered by C-section	Number of births
	Doctor	Nurse	Midwife	Traditional birth attendant	Relative/ other	No one	Don't know/ missing	Total			
**Mother's age at birth**											
<20	11.3	3.6	74.4	10.7	0.0	0.0	0.0	100.0	89.3	4.3	814
20-34	16.2	3.2	70.4	9.9	0.1	0.0	0.1	100.0	89.9	6.4	5,777
35-49	12.5	2.7	66.1	18.2	0.5	0.0	0.0	100.0	81.3	8.0	662
**Birth order**											
1	18.2	2.9	73.1	5.8	0.0	0.0	0.1	100.0	94.1	8.2	2,822
2-3	14.8	3.7	70.9	10.3	0.2	0.0	0.1	100.0	89.4	5.3	3,274
4-5	10.0	3.5	66.3	19.8	0.3	0.2	0.0	100.0	79.8	4.8	833
6+	9.8	0.9	53.9	35.1	0.0	0.0	0.3	100.0	64.6	3.7	323
**Place of delivery**											
Health facility	18.3	3.8	77.8	0.0	0.0	0.0	0.0	100.0	99.9	7.6	6,032
Elsewhere	0.8	0.5	34.2	63.7	0.6	0.2	0.0	100.0	35.5	0.0	1,215
**Residence**											
Urban	30.8	7.2	59.7	2.0	0.2	0.0	0.0	100.0	97.8	14.3	1,041
Rural	12.8	2.6	72.3	12.2	0.1	0.0	0.1	100.0	87.6	4.9	6,212
**Province**											
Banteay Meanchey	6.6	3.9	85.4	3.0	0.5	0.0	0.7	100.0	95.8	8.9	253
Kampong Cham	28.7	1.4	61.4	8.5	0.0	0.0	0.0	100.0	91.5	7.5	1,008
Kampong Chhnang	5.3	0.0	92.2	2.4	0.0	0.0	0.0	100.0	97.6	3.3	248
Kampong Speu	6.1	0.1	83.1	10.4	0.0	0.3	0.0	100.0	89.3	2.2	469
Kampong Thom	9.6	8.3	62.5	19.6	0.0	0.0	0.0	100.0	80.4	5.1	337
Kandal	7.5	1.0	87.2	3.9	0.4	0.0	0.0	100.0	95.7	8.5	523
Kratie	9.1	4.5	38.3	48.1	0.1	0.0	0.0	100.0	51.9	4.5	269
Phnom Penh	34.2	11.2	50.8	3.6	0.3	0.0	0.0	100.0	96.1	14.4	626
Prey Veng	4.8	0.0	92.8	2.4	0.0	0.0	0.0	100.0	97.6	4.4	499
Pursat	2.5	0.0	83.6	13.7	0.3	0.0	0.0	100.0	86.1	2.2	298
Siem Reap	37.1	10.7	45.2	6.7	0.3	0.0	0.0	100.0	93.0	3.8	487
Svay Rieng	12.2	0.0	82.2	5.0	0.4	0.2	0.0	100.0	94.3	6.5	261
Takeo	16.4	3.6	77.4	2.6	0.0	0.0	0.0	100.0	97.4	4.2	386
Otdar Meanchey	7.0	0.3	81.4	11.0	0.0	0.3	0.0	100.0	88.7	3.7	137
Battambang/Pailin	14.9	5.1	74.0	5.5	0.0	0.0	0.5	100.0	94.1	8.3	553
Kampot/Kep	8.7	0.0	81.8	9.5	0.0	0.0	0.0	100.0	90.5	5.0	276
Preah Sihanouk/ Koh Kong	8.5	0.0	88.9	1.6	0.6	0.0	0.3	100.0	97.5	9.4	168
Preah Vihear/ Stung Treng	2.8	0.0	51.8	45.4	0.0	0.0	0.0	100.0	54.6	2.3	239
Mondul Kiri/ Ratanak Kiri	5.2	0.1	48.3	46.0	0.3	0.0	0.1	100.0	53.6	4.0	217
**Mother's education**											
No education	7.6	2.6	61.6	27.8	0.3	0.2	0.0	100.0	71.8	2.6	1,017
Primary	12.0	2.8	73.7	11.3	0.1	0.0	0.1	100.0	88.5	5.1	3,795
Secondary and higher	23.7	4.2	69.1	2.7	0.2	0.0	0.1	100.0	97.0	9.6	2,442
**Wealth quintile**											
Lowest	7.9	2.0	65.3	24.4	0.3	0.0	0.1	100.0	75.2	3.1	1,771
Second	9.6	2.6	74.8	12.7	0.1	0.1	0.1	100.0	87.0	3.0	1,453
Middle	11.9	2.3	78.5	7.2	0.1	0.0	0.0	100.0	92.7	4.4	1,362
Fourth	18.2	4.1	74.1	3.5	0.0	0.0	0.0	100.0	96.5	7.7	1,252
Highest	31.3	5.6	61.5	1.3	0.2	0.0	0.1	100.0	98.4	14.2	1,415
Total	15.3	3.2	70.5	10.7	0.1	0.0	0.1	100.0	89.0	6.3	7,253

Note: If the respondent mentioned more than one person attending during delivery, only the most qualified person is considered in this tabulation. Total includes 6 births for which the place of delivery is missing.
[1] Skilled provider includes doctor, nurse, midwife, and auxiliary nurse/midwife.

13.3 POSTNATAL CARE AND PRACTICES

A large proportion of maternal and neonatal deaths occur during the first 48 hours after delivery. Safe motherhood programs have recently increased their emphasis on the importance of postnatal care, recommending that all women receive a health checkup within two days of delivery. To assess the extent of postnatal care utilization, respondents who had given birth in the five years preceding the survey were asked whether they had received a health check after the delivery of their last birth. Table 13.7.1 shows the timing of the first postnatal checkup for women giving birth in the past two years.

Table 13.7.1 Timing of first postnatal checkup

Among women age 15-49 giving birth in the two years preceding the survey, the percent distribution of the mother's first postnatal checkup for the last live birth by time after delivery, and the percentage of women with a live birth in the two years preceding the survey who received a postnatal checkup in the first two days after giving birth, according to background characteristics, Cambodia 2014

Background characteristic	Time after delivery of mother's first postnatal checkup: Less than 4 hours	4-23 hours	1-2 days	3-41 days	Don't know/ missing	No postnatal checkup[1]	Total	Percentage of women with a postnatal checkup in the first two days after birth	Number of women
**Mother's age at birth**									
<20	74.4	7.5	6.8	0.7	1.0	9.6	100.0	88.7	326
20-34	77.2	9.9	3.8	0.3	0.8	8.1	100.0	90.9	2,380
35-49	67.1	13.9	6.1	0.7	0.7	11.5	100.0	87.1	238
**Birth order**									
1	76.9	10.5	4.3	0.4	1.2	6.7	100.0	91.7	1,204
2-3	77.3	8.8	4.1	0.1	0.5	9.2	100.0	90.2	1,310
4-5	69.4	13.7	4.8	1.4	0.7	10.0	100.0	87.8	346
6+	72.0	4.6	5.7	0.3	0.0	17.3	100.0	82.3	86
**Place of delivery**									
Health facility	80.4	10.6	3.5	0.1	0.9	4.4	100.0	94.5	2,614
Elsewhere	41.7	4.8	10.8	2.2	0.0	40.5	100.0	57.3	329
**Residence**									
Urban	72.9	22.0	3.1	0.1	0.5	1.3	100.0	98.1	414
Rural	76.6	7.9	4.5	0.4	0.8	9.7	100.0	89.1	2,531
**Province**									
Banteay Meanchey	70.7	3.0	15.5	1.0	0.5	9.3	100.0	89.3	120
Kampong Cham	64.9	8.1	9.7	0.0	1.4	15.9	100.0	82.7	418
Kampong Chhnang	98.4	1.6	0.0	0.0	0.0	0.0	100.0	100.0	111
Kampong Speu	83.8	5.9	1.1	0.0	0.8	8.4	100.0	90.8	182
Kampong Thom	96.4	0.3	3.2	0.0	0.0	0.0	100.0	100.0	141
Kandal	66.1	13.9	5.8	1.0	0.0	13.3	100.0	85.7	193
Kratie	74.1	1.5	16.1	0.0	0.0	8.2	100.0	91.8	107
Phnom Penh	61.2	37.1	1.7	0.0	0.0	0.0	100.0	100.0	257
Prey Veng	96.5	2.2	0.0	0.0	1.3	0.0	100.0	98.7	194
Pursat	88.6	4.8	2.4	0.0	0.0	4.2	100.0	95.8	122
Siem Reap	67.0	17.2	3.8	1.3	2.1	8.6	100.0	87.9	182
Svay Rieng	85.2	4.0	0.9	0.9	0.0	9.0	100.0	90.1	108
Takeo	85.4	9.2	1.8	2.0	0.8	0.8	100.0	96.4	164
Otdar Meanchey	91.1	3.1	0.0	0.5	0.0	5.3	100.0	94.2	54
Battambang/Pailin	79.4	15.1	1.0	0.0	2.9	1.6	100.0	95.5	247
Kampot/Kep	78.6	11.3	3.2	0.0	0.0	7.0	100.0	93.0	116
Preah Sihanouk/ Koh Kong	89.4	3.1	2.4	0.0	0.8	4.2	100.0	94.9	61
Preah Vihear/ Stung Treng	57.0	2.4	8.8	0.7	0.0	31.1	100.0	68.2	92
Mondul Kiri/ Ratanak Kiri	37.6	1.6	0.0	0.0	0.0	60.8	100.0	39.2	75
**Education**									
No education	71.5	4.3	4.1	0.6	0.7	18.8	100.0	79.8	366
Primary	76.3	8.8	5.1	0.5	0.7	8.8	100.0	90.1	1,491
Secondary and higher	77.3	13.4	3.4	0.1	1.0	4.7	100.0	94.2	1,088
**Wealth quintile**									
Lowest	74.2	5.2	5.0	0.5	0.2	15.0	100.0	84.3	694
Second	77.8	5.9	3.8	0.9	0.8	10.8	100.0	87.5	589
Middle	82.7	5.4	5.6	0.2	1.2	5.0	100.0	93.6	565
Fourth	75.0	13.2	4.0	0.2	1.1	6.5	100.0	92.2	536
Highest	70.9	21.5	3.2	0.1	0.8	3.5	100.0	95.6	560
Total	76.1	9.9	4.3	0.4	0.8	8.5	100.0	90.3	2,944

Note: Total includes 1 birth for which place of delivery is missing.
[1] Includes women who received a checkup after 41 days

Ninety percent of mothers received postnatal care within the crucial first two days of delivery, with 76 percent receiving care within four hours of delivery. Only 9 percent of mothers received no postnatal care.

Urban women were more likely to receive postnatal care (98 percent) than rural women (89 percent) during the first two days after delivery. Women with a secondary education or higher (94 percent) were more likely to receive postnatal care within two days of delivery than women with either no

schooling (80 percent) or only a primary school education (90 percent). Only 57 percent of women who did not deliver in a health facility received a postnatal checkup.

Table 13.7.2 presents information on the provider of postnatal care for women who delivered in the two years preceding the survey. Eighty-seven percent of women received postnatal care from a health professional (midwife, doctor, or nurse), and only 3 percent received postnatal care from traditional birth attendants. Women in urban areas (98 percent) were more likely than those in rural areas (85 percent) to receive postnatal care from a health professional. Similarly, mothers with a secondary education or higher (93 percent) were much more likely to receive postnatal care from a trained health professional than women with either no schooling (71 percent) or only a primary school education (86 percent).

Table 13.7.2 Type of provider of first postnatal checkup for the mother

Among women age 15-49 giving birth in the two years preceding the survey, the percent distribution by type of provider of the mother's first postnatal health check in the two days after the last live birth, according to background characteristics, Cambodia 2014

Background characteristic	Type of health provider of mother's first postnatal checkup: Doctor	Nurse	Midwife	Traditional birth attendant	Don't know/ missing	No postnatal checkup in the first two days after birth[1]	Total	Number of women
**Mother's age at birth**								
<20	10.3	1.7	71.7	5.1	0.0	11.3	100.0	326
20-34	11.5	2.1	74.2	3.0	0.1	9.0	100.0	2,380
35-49	13.8	0.0	68.5	4.8	0.0	12.9	100.0	238
**Birth order**								
1	13.5	2.2	73.8	2.2	0.2	8.1	100.0	1,204
2-3	10.2	2.2	74.9	2.8	0.0	9.8	100.0	1,310
4-5	9.8	0.0	70.3	7.7	0.0	12.2	100.0	346
6+	11.9	0.0	59.3	11.2	0.0	17.7	100.0	86
**Place of delivery**								
Health facility	12.9	2.0	79.6	0.1	0.1	5.4	100.0	2,614
Elsewhere	1.3	1.0	25.1	30.0	0.0	42.7	100.0	329
**Residence**								
Urban	26.1	7.9	63.7	0.4	0.0	1.9	100.0	414
Rural	9.2	0.9	75.1	3.9	0.1	10.8	100.0	2,531
**Province**								
Banteay Meanchey	6.6	1.1	81.6	0.0	0.0	10.7	100.0	120
Kampong Cham	25.0	1.5	53.7	2.5	0.0	17.3	100.0	418
Kampong Chhnang	1.6	0.0	98.4	0.0	0.0	0.0	100.0	111
Kampong Speu	2.1	0.4	82.8	5.5	0.6	8.6	100.0	182
Kampong Thom	3.8	0.0	82.0	14.2	0.0	0.0	100.0	141
Kandal	8.7	0.3	76.0	0.7	0.0	14.3	100.0	193
Kratie	9.2	0.0	53.8	28.8	0.0	8.2	100.0	107
Phnom Penh	28.6	16.7	52.9	1.8	0.0	0.0	100.0	257
Prey Veng	4.8	0.0	93.9	0.0	0.0	1.3	100.0	194
Pursat	3.4	0.0	89.9	2.5	0.0	4.2	100.0	122
Siem Reap	15.7	0.0	72.1	0.0	0.0	12.1	100.0	182
Svay Rieng	9.3	0.9	79.0	0.9	0.0	9.9	100.0	108
Takeo	20.7	1.0	73.8	0.9	0.0	3.6	100.0	164
Otdar Meanchey	2.2	0.0	87.6	4.3	0.0	5.8	100.0	54
Battambang/Pailin	6.5	0.0	89.0	0.0	0.0	4.5	100.0	247
Kampot/Kep	3.3	0.0	87.3	2.4	0.9	6.1	100.0	116
Preah Sihanouk/ Koh Kong	9.7	1.7	82.8	0.6	0.0	5.1	100.0	61
Preah Vihear/ Stung Treng	2.1	0.0	54.6	11.4	0.0	31.8	100.0	92
Mondul Kiri/ Ratanak Kiri	2.7	0.0	35.4	1.2	0.0	60.8	100.0	75
**Education**								
No education	6.8	0.0	63.9	9.1	0.0	20.2	100.0	366
Primary	8.9	1.2	76.2	3.8	0.1	9.7	100.0	1,491
Secondary and higher	16.8	3.5	73.0	0.9	0.0	5.8	100.0	1,088
**Wealth quintile**								
Lowest	7.4	0.9	66.8	9.2	0.0	15.7	100.0	694
Second	6.7	0.3	77.3	3.2	0.2	12.3	100.0	589
Middle	8.9	0.4	82.0	2.3	0.2	6.3	100.0	565
Fourth	11.7	1.8	78.1	0.6	0.0	7.8	100.0	536
Highest	24.3	6.5	64.7	0.2	0.0	4.4	100.0	560
Total	11.6	1.9	73.5	3.4	0.1	9.6	100.0	2,944

[1] Includes women who received a checkup after 41 days

Table 13.8.1 shows the timing of the first postnatal checkup for newborns born in the past two years. Seventy-seven percent of newborns received postnatal care within the crucial first two days of delivery, with 68 percent receiving care within four hours of delivery. About 21 percent of newborns received no postnatal care.

Postnatal care for newborns was more likely to be provided in urban areas (84 percent) than in rural areas (78 percent) during the first two days after delivery. Newborns whose mothers had a secondary education or higher (84 percent) were more likely to receive postnatal care within two days of delivery than those whose mothers had either no schooling (69 percent) or only a primary school education (78 percent). Only 52 percent of babies who were not delivered in a health facility received a postnatal checkup.

Table 13.8.1 Timing of first postnatal checkup for the newborn

Percent distribution of last births in the two years preceding the survey by time after birth of first postnatal checkup, and the percentage of births with a postnatal checkup in the first two days after birth, according to background characteristics, Cambodia 2014

Background characteristic	Time after birth of newborn's first postnatal checkup						No postnatal checkup[1]	Total	Percentage of births with a postnatal checkup in the first two days after birth	Number of births
	Less than 1 hour	1-3 hours	4-23 hours	1-2 days	3-6 days	Don't know/ missing				
**Mother's age at birth**										
<20	26.4	38.0	6.5	3.8	0.1	0.5	24.9	100.0	74.6	326
20-34	26.9	42.4	8.1	2.0	0.2	1.2	19.1	100.0	79.6	2,380
35-49	21.3	41.6	9.0	5.2	0.5	0.9	21.5	100.0	77.0	238
**Birth order**										
1	27.1	41.3	8.3	2.2	0.2	0.8	20.0	100.0	79.1	1,204
2-3	27.7	41.5	7.8	1.9	0.3	1.4	19.4	100.0	79.0	1,310
4-5	18.2	45.0	9.3	4.2	0.0	1.1	22.3	100.0	76.8	346
6+	28.8	43.4	0.9	7.6	0.3	0.7	18.3	100.0	80.7	86
**Place of delivery**										
Health facility	27.0	44.8	8.7	1.7	0.1	1.1	16.6	100.0	82.3	2,614
Elsewhere	21.5	19.0	2.7	8.1	1.1	1.2	46.3	100.0	51.6	329
**Residence**										
Urban	28.8	32.9	19.8	2.4	0.8	0.5	14.8	100.0	83.8	414
Rural	26.0	43.4	6.1	2.5	0.2	1.2	20.8	100.0	78.0	2,531
**Province**										
Banteay Meanchey	1.2	4.1	0.0	0.6	0.3	1.1	92.7	100.0	5.9	120
Kampong Cham	40.3	18.6	5.4	2.9	0.0	0.0	32.9	100.0	67.1	418
Kampong Chhnang	12.1	85.1	1.6	0.0	0.0	0.0	1.2	100.0	98.8	111
Kampong Speu	31.6	44.8	4.2	0.0	0.0	0.8	18.6	100.0	80.6	182
Kampong Thom	27.1	65.6	0.0	3.1	0.0	2.6	1.5	100.0	97.8	141
Kandal	24.9	38.7	10.0	6.3	0.0	0.0	20.0	100.0	80.0	193
Kratie	46.5	25.0	1.7	12.1	0.2	0.4	14.1	100.0	85.7	107
Phnom Penh	22.2	33.4	37.7	2.2	0.9	0.0	3.5	100.0	95.6	257
Prey Veng	31.7	59.3	1.4	1.0	0.0	1.3	5.3	100.0	93.4	194
Pursat	24.3	51.5	4.1	2.4	0.0	0.5	17.2	100.0	82.3	122
Siem Reap	6.0	53.1	10.0	2.7	0.3	8.6	19.3	100.0	71.8	182
Svay Rieng	19.3	54.1	2.0	1.8	1.8	0.0	21.0	100.0	77.2	108
Takeo	25.2	56.5	8.1	1.0	1.1	0.0	8.1	100.0	90.8	164
Otdar Meanchey	5.6	79.2	2.4	0.2	0.5	0.0	12.1	100.0	87.5	54
Battambang/Pailin	43.0	33.8	12.9	0.8	0.0	2.2	7.3	100.0	90.5	247
Kampot/Kep	10.8	70.7	6.4	1.9	0.0	0.6	9.5	100.0	90.5	116
Preah Sihanouk/ Koh Kong	46.0	40.7	0.7	1.9	0.0	0.8	9.9	100.0	89.3	61
Preah Vihear/ Stung Treng	25.7	30.0	2.3	5.3	0.0	0.0	36.6	100.0	63.4	92
Mondul Kiri/ Ratanak Kiri	6.5	10.4	1.0	0.7	0.0	0.0	81.4	100.0	18.6	75
**Mother's education**										
No education	20.5	42.2	2.6	3.2	0.3	1.3	29.9	100.0	68.5	366
Primary	25.4	42.8	6.8	2.6	0.4	1.0	21.1	100.0	77.7	1,491
Secondary and higher	29.7	40.6	11.5	2.0	0.1	1.2	15.0	100.0	83.8	1,088
**Wealth quintile**										
Lowest	23.2	45.5	3.4	3.0	0.3	0.6	24.0	100.0	75.2	694
Second	25.0	44.7	3.9	2.3	0.3	2.1	21.7	100.0	76.4	589
Middle	29.7	44.6	4.8	2.8	0.0	0.6	17.5	100.0	81.9	565
Fourth	25.9	40.0	9.2	2.8	0.0	1.6	20.6	100.0	77.8	536
Highest	29.0	33.4	19.9	1.4	0.6	0.8	14.9	100.0	83.8	560
Total	26.4	41.9	8.0	2.5	0.2	1.1	19.9	100.0	78.8	2,944

Note: Total includes 1 case for which information on place of delivery is missing.
[1] Includes newborns who received a checkup after the first week

Table 13.8.2 presents information on the provider of postnatal care for newborns who were delivered in the two years preceding the survey. Nearly all newborns receiving postnatal care received it from a health professional (midwife, doctor, or nurse), and only 3 percent received postnatal care from traditional birth attendants. Postnatal care was more likely to be provided by a health professional in urban areas (84 percent) than in rural areas (75 percent). Similarly, babies whose mothers had a secondary education or higher (83 percent) were much more likely to receive postnatal care from a trained health professional than babies whose mothers had either no schooling (60 percent) or only a primary school education (74 percent).

Table 13.8.2 Type of provider of first postnatal checkup for the newborn

Percent distribution of last births in the two years preceding the survey by type of provider of the newborn's first postnatal health check during the two days after the last live birth, according to background characteristics, Cambodia 2014

Background characteristic	Type of health provider of newborn's first postnatal checkup: Doctor/nurse/ midwife	Nurse/midwife	Traditional birth attendant	No postnatal checkup in the first two days after birth, don't know or missing	Total	Number of births
**Mother's age at birth**						
<20	68.2	1.7	4.8	25.4	100.0	326
20-34	74.6	2.2	2.8	20.4	100.0	2,380
35-49	72.0	1.0	4.0	23.0	100.0	238
**Birth order**						
1	74.5	2.6	2.0	20.9	100.0	1,204
2-3	74.2	2.2	2.6	21.0	100.0	1,310
4-5	69.6	0.3	6.9	23.2	100.0	346
6+	69.7	0.0	10.9	19.3	100.0	86
**Place of delivery**						
Health facility	79.9	2.3	0.1	17.7	100.0	2,614
Elsewhere	24.5	0.1	27.0	48.4	100.0	329
**Residence**						
Urban	74.3	9.3	0.3	16.2	100.0	414
Rural	73.6	0.9	3.6	22.0	100.0	2,531
**Province**						
Banteay Meanchey	5.9	0.0	0.0	94.1	100.0	120
Kampong Cham	63.2	0.8	3.1	32.9	100.0	418
Kampong Chhnang	98.8	0.0	0.0	1.2	100.0	111
Kampong Speu	75.2	0.4	4.9	19.4	100.0	182
Kampong Thom	84.2	0.0	13.6	2.2	100.0	141
Kandal	80.0	0.0	0.0	20.0	100.0	193
Kratie	58.3	0.0	27.4	14.3	100.0	107
Phnom Penh	73.7	20.2	1.8	4.4	100.0	257
Prey Veng	93.4	0.0	0.0	6.6	100.0	194
Pursat	80.4	0.0	1.9	17.7	100.0	122
Siem Reap	71.8	0.0	0.0	28.2	100.0	182
Svay Rieng	75.5	1.7	0.0	22.8	100.0	108
Takeo	88.9	1.0	0.9	9.2	100.0	164
Otdar Meanchey	84.5	0.0	3.0	12.5	100.0	54
Battambang/Pailin	90.5	0.0	0.0	9.5	100.0	247
Kampot/Kep	90.5	0.0	0.0	9.5	100.0	116
Preah Sihanouk/ Koh Kong	87.6	0.8	0.9	10.7	100.0	61
Preah Vihear/ Stung Treng	52.0	0.0	11.4	36.6	100.0	92
Mondul Kiri/ Ratanak Kiri	18.3	0.0	0.4	81.4	100.0	75
**Mother's education**						
No education	59.6	0.0	8.9	31.5	100.0	366
Primary	73.5	0.8	3.4	22.3	100.0	1,491
Secondary and higher	78.6	4.4	0.8	16.2	100.0	1,088
**Wealth quintile**						
Lowest	66.2	0.5	8.5	24.8	100.0	694
Second	72.8	0.4	3.2	23.6	100.0	589
Middle	79.9	0.2	1.8	18.1	100.0	565
Fourth	75.6	1.6	0.6	22.2	100.0	536
Highest	75.8	7.9	0.1	16.2	100.0	560
Total	73.7	2.0	3.1	21.2	100.0	2,944

Note: Total includes 1 case for which information on place of delivery is missing.

13.4 PERCEIVED PROBLEMS IN ACCESSING WOMEN'S HEALTH CARE

Many factors can prevent women from getting medical advice or treatment for themselves. In the 2014 CDHS, women were asked about various problems they might face in accessing health care. Table 13.9 shows that 75 percent of women reported having one or more problems in accessing health care for themselves. This figure is similar to that reported by women in 2010 (72 percent).

Table 13.9 Problems in accessing health care

Percentage of women age 15-49 who reported that they have serious problems in accessing health care for themselves when they are sick, by type of problem, according to background characteristics, Cambodia 2014

	Problems in accessing health care					
Background characteristic	Getting permission to go for treatment	Getting money for treatment	Distance to health facility	Not wanting to go alone	At least one problem accessing health care	Number of women
**Age**						
15-19	25.1	64.0	34.4	55.8	78.7	2,893
20-34	20.1	62.0	33.3	42.6	71.8	8,899
35-49	21.5	68.2	37.8	44.4	76.6	5,786
**Number of living children**						
0	21.8	61.2	31.9	49.1	73.9	5,698
1-2	21.3	63.4	35.0	42.8	72.1	6,622
3-4	20.4	66.1	36.5	42.4	75.7	3,893
5+	22.8	77.4	43.0	50.6	85.8	1,365
**Marital status**						
Never married	22.6	61.0	31.4	50.7	74.1	4,428
Married or living together	20.9	64.8	36.1	43.7	74.4	11,898
Divorced/separated/ widowed	21.8	72.1	36.5	41.7	77.3	1,252
**Employed last 12 months**						
Not employed	20.8	63.9	35.6	47.8	75.4	3,600
Employed for cash	21.4	63.7	33.4	43.4	73.3	12,702
Employed not for cash	22.8	72.9	48.6	57.9	84.2	1,273
**Residence**						
Urban	10.6	44.5	14.4	26.5	54.7	3,251
Rural	23.8	68.9	39.6	49.6	79.0	14,327
**Province**						
Banteay Meanchey	76.2	88.8	74.1	77.1	93.1	689
Kampong Cham	27.0	88.4	51.4	72.4	97.7	2,021
Kampong Chhnang	11.0	77.2	15.3	45.9	81.0	662
Kampong Speu	26.6	85.2	43.0	53.4	91.0	1,196
Kampong Thom	33.4	90.0	25.3	26.9	91.1	851
Kandal	5.0	55.5	25.3	39.1	70.2	1,330
Kratie	22.0	67.0	53.3	51.5	82.4	488
Phnom Penh	2.1	33.7	5.4	13.4	41.4	1,994
Prey Veng	38.3	43.8	23.3	24.6	48.0	1,188
Pursat	31.5	84.9	59.4	58.7	90.9	631
Siem Reap	19.4	39.2	25.0	51.4	63.8	1,137
Svay Rieng	5.9	41.3	45.7	56.6	66.1	654
Takeo	37.5	76.0	46.4	50.4	80.7	1,082
Otdar Meanchey	29.5	72.8	50.0	38.4	76.7	294
Battambang/Pailin	3.4	54.8	27.2	38.5	71.1	1,333
Kampot/Kep	11.0	82.5	38.3	47.6	85.1	770
Preah Sihanouk/ Koh Kong	38.1	71.2	49.6	56.4	86.3	422
Preah Vihear/ Stung Treng	14.8	57.4	42.2	54.0	75.0	462
Mondul Kiri/ Ratanak Kiri	7.8	38.3	29.8	33.1	55.6	372
**Education**						
No education	27.3	74.5	49.0	55.3	84.7	2,250
Primary	23.5	70.1	39.2	47.4	79.6	8,281
Secondary and higher	17.0	54.4	25.5	39.7	65.3	7,047
**Wealth quintile**						
Lowest	26.1	79.2	50.9	58.3	87.8	3,143
Second	25.9	72.8	44.2	51.9	82.8	3,314
Middle	24.9	72.9	39.2	49.1	81.4	3,381
Fourth	22.1	62.3	32.0	44.5	72.9	3,612
Highest	10.7	41.2	14.5	27.9	53.5	4,128
Total	21.4	64.4	35.0	45.4	74.5	17,578

The most frequently cited problem in accessing health care was not having money for treatment (64 percent), followed by not wanting to go to the facility alone (45 percent). Thirty-five percent of women cited distance to the health facility as a problem, and 21 percent reported that getting permission to go to a facility was a problem. As expected, rural women were more likely than urban women to report each of the factors as being a problem, especially distance to a health facility and getting money for treatment. The proportion of women reporting each of these problems as a serious obstacle to accessing health care decreases with increasing education and wealth.

CHILD HEALTH 14

Key Findings

- Seventy-three percent of children age 12-23 months are fully immunized.
- Six percent of children under age 5 showed symptoms of acute respiratory infection in the two weeks before the survey, and 81 percent of these children received antibiotics.
- Twenty-eight percent of children under age 5 had a fever in the two weeks before the survey, and 61 percent of them were taken to a health facility or provider for advice or treatment.
- Thirteen percent of children under age 5 had diarrhea in the two weeks before the survey.
- Fifty-six percent of children with diarrhea were taken to a health facility or provider for advice or treatment.

This chapter presents findings on several areas of importance to child health: characteristics of the neonate (birth weight and size at birth), vaccination status of children, and important childhood illnesses and their treatment. Information on birth weight and birth size is important for the design and implementation of programs aimed at reducing neonatal and infant mortality. Many early childhood deaths can be prevented by immunizing children against preventable diseases and by ensuring that children receive prompt and appropriate treatment when they become ill.

14.1 CHILD'S SIZE AT BIRTH

Birth weight is one of the major determinants of infant and child health and mortality. Children whose birth weight is less than 2.5 kilograms, or children reported to be "very small" or "smaller than average," are considered to have a higher than average risk of early childhood death. For births in the five years preceding the 2014 CDHS, birth weight was recorded in the questionnaire if available from either a written record or the mother's recall. Because birth weight may not be known for many babies, the mother's estimate of the baby's size at birth was also obtained. Even though such an estimate is subjective, it can be a useful proxy for the weight of the child. Table 14.1 presents information on child's size at birth according to background characteristics.

Table 14.1 shows that 91 percent of babies were weighed at birth; this represents a significant increase since the 2010 CDHS, which reported that 72 percent of babies were weighed at birth. Among those births for which the mother was able to report the baby's weight, 8 percent were classified as low birth weight (less than 2.5 kilograms at birth), which is the same as the figure reported in 2010. Low birth weight was more common among children of birth order six or higher (13 percent) and first-born children (10 percent) than among children of birth orders two through five (6-7 percent). Children born to mothers who smoke were more likely to be of low birth weight (11 percent) than children born to mothers who do not smoke (8 percent). The proportion of low birth weight births varied somewhat across provinces (from 4 percent to 12 percent). However, the proportion with a reported birth weight varied substantially, from a low of 56 percent in Mondul Kiri/Ratanak Kiri to a high of just under 100 percent in Kampong Chhnang.

Table 14.1 also includes information on the mother's assessment of the baby's size at birth. In the absence of birth weight, a mother's subjective assessment of the size of the baby at birth may be useful. However, this assessment may vary among respondents because it is based on the mother's own perception of what is small, average, or large for a baby and not on a uniform definition. Eighty-eight percent of

infants were considered by their mothers to be average or larger than average in size. Nine percent were perceived as smaller than average, and 3 percent were considered very small. For less than 1 percent of births, mothers did not remember the size of their baby at birth.

Table 14.1 Child's size and weight at birth

Percent distribution of live births in the five years preceding the survey by mother's estimate of baby's size at birth, percentage of live births in the five years preceding the survey that have a reported birth weight, and among live births in the five years preceding the survey with a reported birth weight, percentage less than 2.5 kg, according to background characteristics, Cambodia 2014

	Percent distribution of all live births by size of child at birth					Percentage of all births that have a		Percent distribution of births with a reported birth weight	
Background characteristic	Very small	Smaller than average	Average or larger	Don't know/ missing	Total	reported birth weight[1]	Number of births	Less than 2.5 kg	Number of births
**Mother's age at birth**									
<20	3.3	10.5	85.9	0.3	100.0	89.5	814	8.1	729
20-34	2.5	8.6	88.1	0.8	100.0	91.5	5,777	7.8	5,289
35-49	3.8	8.9	86.8	0.6	100.0	85.6	662	8.8	567
**Birth order**									
1	3.3	11.1	85.2	0.5	100.0	94.6	2,822	9.9	2,670
2-3	1.8	7.4	90.2	0.6	100.0	91.4	3,274	6.1	2,991
4-5	3.4	6.1	89.1	1.3	100.0	84.2	833	6.7	702
6+	5.3	10.8	81.5	2.4	100.0	68.4	323	12.7	221
**Mother's smoking status**									
Smokes cigarettes/tobacco	6.2	17.0	75.7	1.1	100.0	47.0	215	11.1	101
Does not smoke	2.6	8.6	88.1	0.7	100.0	92.1	7,037	7.9	6,482
**Residence**									
Urban	1.7	6.8	91.1	0.4	100.0	98.1	1,041	5.6	1,022
Rural	2.9	9.2	87.1	0.8	100.0	89.5	6,212	8.4	5,562
**Province**									
Banteay Meanchey	7.0	6.8	83.0	3.2	100.0	94.6	253	6.6	240
Kampong Cham	4.3	8.6	85.4	1.7	100.0	92.9	1,008	6.9	936
Kampong Chhnang	1.8	4.6	93.6	0.0	100.0	99.6	248	8.9	247
Kampong Speu	7.4	11.0	81.6	0.0	100.0	90.7	469	9.3	425
Kampong Thom	1.1	3.1	95.8	0.0	100.0	85.0	337	8.4	286
Kandal	0.4	9.1	90.5	0.0	100.0	93.1	523	7.6	486
Kratie	10.3	10.7	79.0	0.0	100.0	70.5	269	11.5	190
Phnom Penh	1.0	6.1	92.9	0.0	100.0	98.2	626	5.7	615
Prey Veng	1.2	6.7	92.1	0.0	100.0	97.8	499	9.9	488
Pursat	0.3	10.7	88.6	0.5	100.0	86.9	298	4.4	259
Siem Reap	2.3	16.6	80.5	0.6	100.0	94.5	487	12.2	460
Svay Rieng	3.5	6.7	89.4	0.4	100.0	89.3	261	11.9	233
Takeo	1.9	3.1	94.5	0.5	100.0	98.4	386	8.2	380
Otdar Meanchey	0.8	9.3	89.3	0.6	100.0	94.2	137	8.5	129
Battambang/Pailin	0.5	9.2	89.0	1.4	100.0	93.5	553	5.2	517
Kampot/Kep	0.9	12.3	85.7	1.1	100.0	87.6	276	6.1	241
Preah Sihanouk/ Koh Kong	1.1	6.3	91.7	0.9	100.0	94.1	168	6.6	158
Preah Vihear/ Stung Treng	2.5	5.2	92.4	0.0	100.0	72.1	239	7.8	172
Mondul Kiri/ Ratanak Kiri	3.7	25.0	68.3	3.1	100.0	55.8	217	9.2	121
**Mother's education**									
No education	3.9	11.7	82.1	2.3	100.0	77.9	1,017	11.9	792
Primary	2.6	8.4	88.3	0.7	100.0	90.1	3,795	7.3	3,419
Secondary and higher	2.4	8.4	89.1	0.1	100.0	97.2	2,442	7.5	2,373
**Wealth quintile**									
Lowest	3.8	10.1	85.0	1.1	100.0	80.2	1,771	10.8	1,420
Second	2.4	9.1	87.6	0.9	100.0	88.1	1,453	8.2	1,279
Middle	2.9	9.7	86.7	0.7	100.0	94.0	1,362	7.3	1,280
Fourth	2.7	8.0	88.8	0.5	100.0	96.5	1,252	7.6	1,209
Highest	1.5	6.9	91.3	0.3	100.0	98.6	1,415	5.6	1,395
Total	2.7	8.8	87.7	0.7	100.0	90.8	7,253	7.9	6,584

Note: Total includes 1 birth with missing information on mother's smoking status
[1] Based on either a written record or the mother's recall

14.2 IMMUNIZATION OF CHILDREN

Universal immunization of children against six vaccine-preventable diseases (namely, tuberculosis, diphtheria, whooping cough, tetanus, polio, and measles) is crucial to reducing infant and child mortality. Data on differences in vaccination coverage among subgroups of the population are of

great assistance for program planning. In addition, information on immunization coverage is important for monitoring and evaluation of the Expanded Program on Immunization.

Similar to the previous CDHS, the 2014 CDHS collected information on vaccination coverage for all living children born in the five years preceding the survey. Guidelines developed by the World Health Organization define children as fully vaccinated when they have received a vaccination against tuberculosis (BCG); three doses each of the diphtheria, pertussis, and tetanus (DPT) and polio vaccines; and a measles vaccination by age 12 months. BCG should be given at birth or at first clinical contact; DPT and polio require three vaccinations at approximately age 4, 8, and 12 weeks. Measles should be given at or soon after age 9 months. In 2006, the Cambodian National Immunization Program replaced the DPT vaccine with a tetravalent vaccine that includes DPT and *Haemophilus influenzae* type b vaccine (Hib) and a pentavalent vaccine that includes DPT, Hib, and hepatitis B vaccine (HepB). The program also administers HepB vaccine at birth or at first clinical contact (HB 0).

Information on vaccination coverage was collected in two ways: from vaccination cards shown to the interviewer and from mothers' verbal reports. If cards were available, the interviewer copied the vaccination dates directly onto the questionnaire. When there was no vaccination card for the child or if a vaccine had not been recorded on the card as being given, the respondent was asked to recall the vaccines given to her child. The top three rows of Table 14.2 show the percentage of children age 12-23 months who have received various vaccinations by source of information, that is, from the vaccination card or a mother's report. Data are presented only for those children who have reached the age by which they should be fully vaccinated (i.e., age 12-23 months) so as to provide estimates of the most recent vaccination coverage.

Table 14.2 Vaccinations by source of information

Percentage of children age 12-23 months who received specific vaccines at any time before the survey, by source of information (vaccination card or mother's report), and percentage vaccinated by age 12 months, Cambodia 2014

Source of information	BCG	Tetravalent/pentavalent 1	2	3	HB 0[1]	Polio 1	2	3	Measles	All basic vaccina-tions[2]	No vaccina-tions	Number of children
**Vaccinated at any time before survey**												
Vaccination card	76.4	75.4	72.8	68.6	65.1	75.9	72.0	67.2	63.4	60.0	0.0	1,129
Mother's report	19.7	18.6	17.6	15.1	17.7	18.9	17.6	15.1	15.2	13.5	2.4	332
Either source	96.1	94.0	90.4	83.7	82.8	94.8	89.5	82.3	78.6	73.4	2.4	1,460
Vaccinated by age 12 months[3]	95.9	93.6	89.7	81.9	82.6	94.5	88.8	80.2	70.3	65.3	2.6	1,460

[1] HB 0 is hepatitis B vaccine given at birth.
[2] BCG, measles, and three doses each of tetravalent/pentavalent and polio vaccine
[3] For children whose information was based on the mother's report, the proportion of vaccinations given during the first year of life was assumed to be the same as for children with a written record of vaccination.

The last row of Table 14.2 shows that two-thirds of children (65 percent) age 12-23 months were fully vaccinated by age 12 months. Nearly all children had received the BCG vaccination and the first two doses of tetravalent/pentavalent vaccine or polio vaccine (89 percent to 96 percent), and 70 percent had been vaccinated against measles. Because the tetravalent/pentavalent and polio vaccines are often administered at the same time, their coverage rates are similar. Eighty-two percent and 80 percent of children received the third doses of tetravalent/pentavalent and polio vaccines, respectively. When looking at the proportion of children who received vaccines at any time before the survey (not necessarily before age 12 months), the percentages are higher, with 73 percent fully vaccinated.

Table 14.3 shows vaccination coverage at any time before the survey among children age 12-23 months by background characteristics. These data may provide certain information for the assessment of the immunization program in reaching out to all population subgroups. The vaccination coverage rates of male and female children are practically the same. Children in urban areas are more likely to be fully vaccinated than those in rural areas (86 percent versus 71 percent). Also, there are substantial differences in coverage across provinces. The percentage of children fully vaccinated is lowest in Mondul

Kiri/Ratanak Kiri (44 percent), Preah Vihear/Stung Treng (56 percent), and Kampong Cham (57 percent). The provinces with the highest proportion of children fully vaccinated are Banteay Meanchey (91 percent), Phnom Penh (89 percent), Battambang/Pailin (89 percent), and Takeo (88 percent).

The percentage of children fully vaccinated increases substantially with increasing mother's education. Children of mothers with a secondary education or higher are much more likely to be fully vaccinated (84 percent) than children whose mothers have no schooling (58 percent). The percentage of children fully vaccinated also increases according to the wealth of the household; children living in the wealthiest households are more likely to be fully vaccinated (91 percent) than children from the poorest households (61 percent).

Table 14.3 Vaccinations by background characteristics

Percentage of children age 12-23 months who received specific vaccines at any time before the survey (according to a vaccination card or the mother's report), and percentage with a vaccination card, by background characteristics, Cambodia 2014

Background characteristic	BCG	Tetravalent/pentavalent			HB 0[1]	Polio			Measles	All basic vaccinations[2]	No vaccinations	Percentage with a vaccination card seen	Number of children
		1	2	3		1	2	3					
**Sex**													
Male	96.2	93.4	90.4	83.0	81.8	94.3	90.3	82.6	79.1	73.9	2.6	77.2	750
Female	96.0	94.5	90.4	84.3	83.8	95.3	88.7	82.0	78.1	73.0	2.2	77.4	711
**Birth order**													
1	98.1	94.6	91.5	87.2	85.1	96.7	91.5	86.5	80.9	76.0	1.0	80.3	586
2-3	94.7	93.9	90.8	83.9	81.9	94.2	89.3	82.2	79.1	74.1	3.3	75.3	648
4-5	95.6	94.9	88.1	75.3	83.2	94.9	87.7	73.1	73.1	68.2	2.2	74.7	181
6+	(91.7)	(82.4)	(80.6)	(67.2)	(63.6)	(77.1)	(73.9)	(65.9)	(63.8)	(52.4)	(6.7)	(78.6)	45
**Residence**													
Urban	97.6	99.1	97.3	92.9	87.9	98.5	96.8	90.0	90.7	86.4	0.5	71.3	217
Rural	95.8	93.1	89.2	82.1	81.9	94.1	88.3	80.9	76.5	71.2	2.7	78.3	1,244
**Province**													
Banteay Meanchey	96.4	96.6	95.4	94.0	86.3	96.6	95.4	94.0	93.4	91.3	1.6	88.8	63
Kampong Cham	95.0	81.1	81.1	70.6	77.1	86.6	78.7	71.1	64.1	56.8	4.7	67.6	182
Kampong Chhnang	100.0	98.2	92.9	86.3	98.3	98.2	92.9	86.3	74.6	74.6	0.0	77.5	52
Kampong Speu	97.1	97.3	90.1	78.2	74.5	97.3	88.4	78.2	66.5	66.5	1.4	87.9	90
Kampong Thom	97.6	94.8	90.3	82.0	96.0	96.4	91.9	82.0	74.1	70.9	2.4	86.3	77
Kandal	100.0	100.0	97.3	81.5	83.9	100.0	91.3	74.1	75.2	64.5	0.0	80.9	89
Kratie	86.5	89.5	81.8	72.8	62.3	88.6	83.9	72.5	79.7	65.1	7.7	72.1	57
Phnom Penh	98.6	100.0	98.6	93.1	86.3	98.6	98.2	90.0	91.0	89.1	0.0	69.6	145
Prey Veng	95.2	89.8	79.2	76.0	60.2	89.8	79.2	76.0	63.2	61.7	4.8	68.8	101
Pursat	95.3	94.0	90.6	83.3	88.0	95.6	92.2	84.5	88.9	79.7	3.0	84.4	60
Siem Reap	98.2	99.0	93.7	90.9	95.4	99.0	93.7	86.6	85.1	78.6	1.0	91.9	100
Svay Rieng	92.4	90.9	90.9	88.8	71.5	90.9	90.9	88.8	86.7	82.7	5.6	72.4	53
Takeo	96.5	99.8	99.5	97.9	98.1	99.8	94.8	93.1	94.2	87.8	0.2	89.7	84
Otdar Meanchey	97.4	92.7	85.6	83.4	77.2	94.8	87.7	81.8	85.3	75.0	2.6	71.7	27
Battambang/Pailin	99.5	98.4	98.4	95.4	88.5	98.4	98.4	95.4	89.6	89.2	0.0	81.2	120
Kampot/Kep	94.0	93.1	88.6	80.0	93.8	93.1	88.6	80.0	81.1	72.0	2.0	70.7	54
Preah Sihanouk/ Koh Kong	98.0	98.0	96.2	92.0	93.3	98.0	96.2	89.9	86.4	82.6	2.0	79.7	28
Preah Vihear/ Stung Treng	91.9	93.1	89.5	79.3	72.8	93.1	87.8	77.7	62.8	55.6	1.2	74.6	44
Mondul Kiri/ Ratanak Kiri	81.4	79.7	67.4	55.9	62.8	83.9	65.2	54.1	56.1	43.9	10.6	41.1	35
**Mother's education**													
No education	91.6	85.4	78.6	69.0	70.1	83.8	76.3	68.9	65.5	58.4	5.9	73.6	197
Primary	96.0	93.2	88.8	80.0	83.0	94.6	88.5	78.7	75.3	69.7	2.8	79.1	732
Secondary and higher	97.9	98.2	97.0	94.1	87.1	99.1	95.9	92.1	88.0	84.2	0.5	76.2	532
**Wealth quintile**													
Lowest	93.1	88.3	81.1	71.6	75.2	89.1	80.5	71.7	65.9	60.9	4.5	72.4	369
Second	92.4	91.9	86.7	78.3	77.9	93.1	86.0	77.0	71.7	65.4	5.2	76.5	285
Middle	98.8	93.1	91.0	82.5	84.7	95.7	89.8	80.2	77.7	70.0	0.4	84.1	267
Fourth	99.3	99.2	98.0	94.3	88.3	98.7	97.7	93.7	87.6	85.2	0.5	83.5	253
Highest	98.3	99.4	98.9	96.2	90.7	99.5	97.2	92.9	94.7	90.5	0.2	72.6	286
Total	96.1	94.0	90.4	83.7	82.8	94.8	89.5	82.3	78.6	73.4	2.4	77.3	1,460

Note: Figures in parentheses are based on 25-49 unweighted cases.
[1] HB 0 is hepatitis B vaccine given at birth.
[2] BCG, measles, and three doses each of tetravalent/pentavalent and polio vaccine

Trends in vaccination coverage can be seen by comparing similarly collected data in the previous CDHS surveys (2000, 2005, and 2010) with data from the 2014 CDHS (Figure 14.1). The data show that full vaccination coverage in Cambodia substantially improved from 2000 to 2005. From 2005 to 2010, vaccination coverage further improved but at a smaller increment than the increase between 2000 and 2005. However, between 2010 and 2014 vaccination coverage has declined, from 74 percent to 65 percent. Examining trends for individual vaccines reveals that the decline in full coverage is largely due to decreases in coverage of measles and polio 3 vaccinations, particularly measles.

Figure 14.1 **Trends in vaccination by age 12 months among children age 12-23 months, 2000-2014**

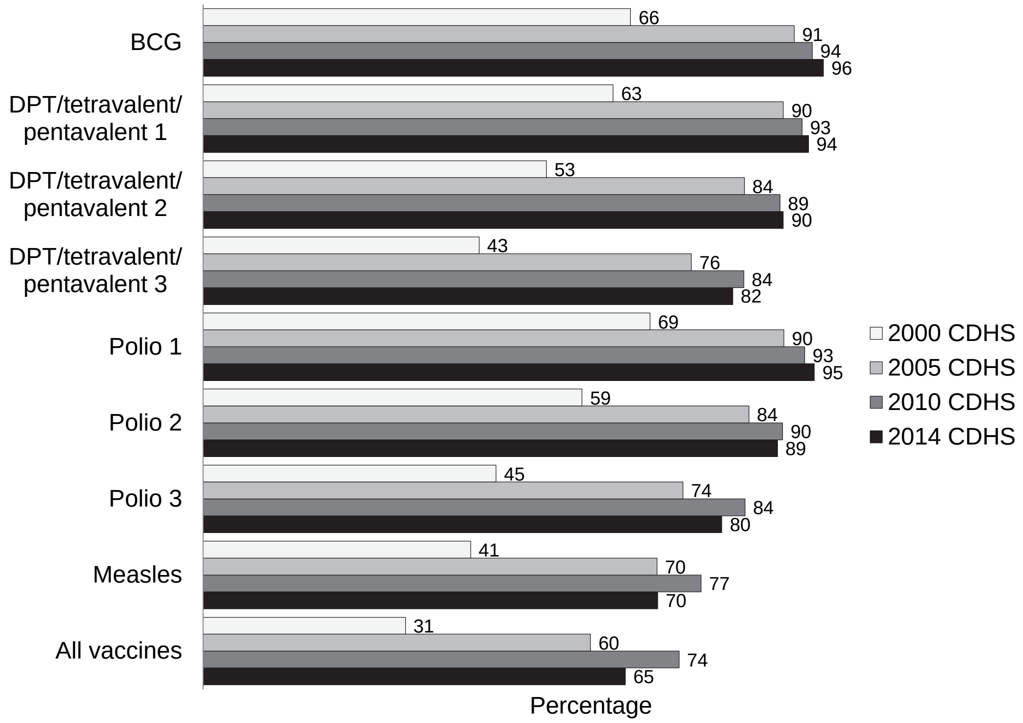

"All vaccines" includes BCG, measles and three doses each of DPT or tetravalent or pentavalent and polio vaccine.

14.3 ACUTE RESPIRATORY INFECTION

Acute respiratory infection (ARI) is one of the leading causes of childhood morbidity and mortality throughout the world. Early diagnosis and treatment with antibiotics can prevent a large proportion of deaths caused by ARI. In the 2014 CDHS, the prevalence of ARI was estimated by asking mothers whether their children under age 5 had been ill with a cough accompanied by short, rapid breathing in the two weeks preceding the survey. These symptoms are compatible with ARI. It should be noted that the morbidity data collected are subjective—that is, they are based on the mother's perception of illness with no validation from medical personnel—and that the prevalence of ARI is subject to seasonality.

Table 14.4 shows the percentage of children under age 5 with symptoms of ARI during the two weeks preceding the survey according to selected background characteristics. Six percent of children under age 5 showed ARI symptoms at some point in the two weeks preceding the survey. Only about 3 percent of children under age 6 months experienced ARI symptoms. The prevalence of ARI increased to 6 percent among children age 6-11 months and 7 percent among those age 12-23 months. After age 23 months, ARI prevalence decreased with increasing age. The prevalence of ARI was significantly higher among children whose mothers smoke (10 percent) than among children whose mothers do not smoke (5 percent). There was only minor variation in the prevalence of ARI symptoms between urban and rural children.

Table 14.4 Prevalence and treatment of symptoms of ARI

Among children under age 5, the percentage who had symptoms of acute respiratory infection (ARI) in the two weeks preceding the survey, and among children with symptoms of ARI, the percentage for whom advice or treatment was sought from a health facility or provider and the percentage who received antibiotics as treatment, according to background characteristics, Cambodia 2014

	Among children under age 5:		Among children under age 5 with symptoms of ARI:		
Background characteristic	Percentage with symptoms of ARI[1]	Number of children	Percentage for whom advice or treatment was sought from a health facility or provider[2]	Percentage who received antibiotics	Number of children
**Age in months**					
<6	2.9	736	*	*	22
6-11	5.9	761	(75.9)	(82.9)	45
12-23	7.4	1,460	68.8	85.2	109
24-35	6.0	1,368	64.6	80.2	82
36-47	5.5	1,343	61.8	77.8	73
48-59	4.1	1,376	78.2	80.2	56
**Sex**					
Male	5.7	3,522	62.2	77.8	201
Female	5.3	3,523	75.9	85.0	186
**Mother's smoking status**					
Smokes cigarettes/ tobacco	10.0	202	*	*	20
Does not smoke	5.4	6,842	70.1	82.1	367
**Cooking fuel**					
Electricity	0.7	47	na	na	0
LPG	5.8	1,150	62.3	77.9	67
Biogas	*	1	na	na	0
Coal/lignite	5.0	554	(78.7)	(86.1)	28
Charcoal	5.5	5,269	69.0	81.6	290
Wood/straw/ agricultural crop[3]	*	19	*	*	2
No food cooked in household	*	3	na	na	0
**Residence**					
Urban	5.4	1,033	69.6	74.4	56
Rural	5.5	6,011	68.6	82.4	332
**Province**					
Banteay Meanchey	5.6	250	*	*	14
Kampong Cham	5.7	974	*	*	56
Kampong Chhnang	11.8	236	(94.6)	(98.3)	28
Kampong Speu	3.9	457	*	*	18
Kampong Thom	4.1	327	*	*	13
Kandal	3.6	506	*	*	18
Kratie	10.5	254	(59.6)	(79.3)	27
Phnom Penh	6.4	618	(62.8)	(72.9)	39
Prey Veng	3.8	478	*	*	18
Pursat	3.5	294	*	*	10
Siem Reap	10.2	470	(75.0)	(82.8)	48
Svay Rieng	5.5	253	*	*	14
Takeo	6.5	374	*	*	24
Otdar Meanchey	3.5	134	*	*	5
Battambang/Pailin	3.4	545	*	*	18
Kampot/Kep	4.0	272	*	*	11
Preah Sihanouk/ Koh Kong	11.8	164	(78.4)	(50.4)	19
Preah Vihear/ Stung Treng	1.7	228	*	*	4
Mondul Kiri/ Ratanak Kiri	1.3	208	*	*	3
**Mother's education**					
No education	5.3	973	(66.1)	(76.9)	52
Primary	6.5	3,687	66.8	82.3	239
Secondary and higher	4.1	2,384	74.9	81.2	97
**Wealth quintile**					
Lowest	7.1	1,689	67.9	89.0	119
Second	5.3	1,403	64.9	77.2	74
Middle	5.0	1,332	78.1	81.1	67
Fourth	5.4	1,217	67.3	74.8	66
Highest	4.4	1,404	66.5	78.3	61
Total	5.5	7,044	68.8	81.3	387

Note: Figures in parentheses are based on 25-49 unweighted cases. An asterisk indicates that a figure is based on fewer than 25 unweighted cases and has been suppressed.
na = Not applicable
LPG = Liquid petroleum gas
[1] Symptoms of ARI (cough accompanied by short, rapid breathing that was chest-related) are considered a proxy for pneumonia.
[2] Excludes pharmacy, shop, and traditional practitioner
[3] Includes grass, shrubs, and crop residues

The proportion of children with ARI symptoms was negatively associated with wealth quintile. Seven percent of children living in households in the lowest wealth quintile experienced ARI symptoms, as compared with 4 percent of children living in households in the highest wealth quintile. There were significant provincial variations in the prevalence of ARI, ranging from a low of 1 percent in Mondul Kiri/Ratanak Kiri to a high of 12 percent in Kampong Chhnang and Preah Sihanouk/Koh Kong.

About 7 of 10 children under age 5 (69 percent) with a cough and rapid breathing were taken to a health facility or provider to seek treatment or advice. Children of mothers with no schooling or with a primary education were less likely to receive treatment for ARI symptoms (66-67 percent) than were children of mothers with a secondary education or higher (75 percent). About 8 in 10 children with ARI symptoms were given antibiotics.

14.4 Fever

Fever is a primary manifestation of several acute infections in children. Fever and other infections can contribute to high levels of malnutrition and mortality. The 2014 CDHS asked mothers whether their children experienced fever during the two weeks preceding the survey.

Table 14.5 shows the percentage of children under age 5 who had a fever during the two weeks preceding the survey according to selected background characteristics. Overall, 28 percent of children under age 5 had a fever at some time in the two weeks preceding the survey. The prevalence of fever varied by the age of the child, and children age 6-11 months and 12-23 months were more commonly sick with fever (35 percent and 36 percent, respectively) than other children. The prevalence of fever among boys was slightly higher than that among girls. There were no significant differences by residence in the prevalence of fever.

Provincial variations, however, were significant; fever prevalence ranged from a low of 11 percent in Kampot/Kep to a high of 40 percent in Battambang/Pailin. Mother’s education and wealth quintile had little association with the prevalence of fever among children less than age 5.

Sixty-one percent of all children under age 5 with a fever were taken to a health facility or provider to seek treatment or advice. Children of mothers with a primary education and a secondary education or higher were more likely to receive treatment for fever (61 percent and 62 percent, respectively) than children of mothers with no schooling (56 percent). The proportion of children for whom treatment was sought from a health facility or provider was highest in Kampong Chhnang (95 percent) and lowest in Mondul Kiri/Ratanak Kiri (44 percent).

Less than 1 percent of children with a fever received antimalarial drugs, whereas 73 percent received antibiotic drugs. Use of antibiotic drugs was more common in urban areas (75 percent) than in rural areas (64 percent). Mothers in Kampong Chhnang, Takeo, Svay Rieng, and Kampong Thom were most likely to use antibiotic drugs to treat fever (90 percent or more).

Table 14.5 Prevalence and treatment of fever

Among children under age 5, the percentage who had a fever in the two weeks preceding the survey, and among children with fever, the percentage for whom advice or treatment was sought from a health facility or provider, the percentage who took antimalarial drugs, and the percentage who received antibiotics as treatment, by background characteristics, Cambodia 2014

	Among children under age 5:		Among children under age 5 with fever:			
Background characteristic	Percentage with fever	Number of children	Percentage for whom advice or treatment was sought from a health facility or provider[1]	Percentage who took antimalarial drugs	Percentage who took antibiotic drugs	Number of children
**Age in months**						
<6	19.9	736	54.5	0.0	73.3	146
6-11	35.1	761	64.5	0.0	82.4	267
12-23	35.7	1,460	61.7	0.4	77.4	521
24-35	30.1	1,368	58.6	0.4	68.3	412
36-47	24.7	1,343	61.8	0.5	71.4	332
48-59	21.0	1,376	59.7	0.2	67.7	289
**Sex**						
Male	29.3	3,522	57.4	0.3	72.8	1,030
Female	26.6	3,523	64.1	0.3	74.1	937
**Residence**						
Urban	28.3	1,033	57.9	0.2	63.8	292
Rural	27.9	6,011	61.1	0.3	75.1	1,675
**Province**						
Banteay Meanchey	23.6	250	48.9	2.2	61.1	59
Kampong Cham	31.0	974	53.6	0.0	75.1	302
Kampong Chhnang	30.1	236	94.9	0.0	96.4	71
Kampong Speu	21.4	457	49.4	0.0	81.1	98
Kampong Thom	20.6	327	76.9	0.0	90.2	67
Kandal	26.0	506	68.2	0.0	79.4	132
Kratie	34.1	254	71.7	1.1	77.1	87
Phnom Penh	37.0	618	59.5	0.0	53.2	229
Prey Veng	14.5	478	(85.2)	(0.0)	(77.5)	69
Pursat	19.6	294	47.0	0.0	52.9	58
Siem Reap	34.6	470	49.5	0.9	55.6	163
Svay Rieng	17.5	253	84.9	0.0	89.8	44
Takeo	33.7	374	53.5	0.0	93.5	126
Otdar Meanchey	16.3	134	53.7	8.4	79.3	22
Battambang/Pailin	40.0	545	57.3	0.0	87.4	218
Kampot/Kep	11.2	272	(68.1)	(0.0)	(57.3)	30
Preah Sihanouk/ Koh Kong	35.7	164	69.3	0.0	58.6	59
Preah Vihear/ Stung Treng	32.6	228	67.4	0.0	77.0	74
Mondul Kiri/ Ratanak Kiri	28.7	208	44.1	0.0	51.7	60
**Mother's education**						
No education	24.3	973	56.2	0.1	73.5	237
Primary	28.9	3,687	60.8	0.4	73.5	1,064
Secondary and higher	28.0	2,384	61.8	0.1	73.3	667
**Wealth quintile**						
Lowest	29.2	1,689	61.8	0.3	74.5	493
Second	25.0	1,403	67.0	0.1	75.5	351
Middle	28.6	1,332	55.8	0.5	78.4	381
Fourth	27.8	1,217	59.1	0.5	71.4	339
Highest	28.8	1,404	59.3	0.0	67.4	404
Total	27.9	7,044	60.6	0.3	73.4	1,967

Note: Figures in parentheses are based on 25-49 unweighted cases.
[1] Excludes pharmacy, shop, and traditional practitioner

14.5 DIARRHEA

Dehydration caused by severe diarrhea is a major cause of morbidity and mortality among young children, although the condition can be easily treated with oral rehydration therapy (ORT). Exposure to diarrhea-causing agents is frequently related to the use of contaminated water and to unhygienic practices in food preparation and disposal of excreta.

Table 14.6 shows the percentage of children under age 5 with diarrhea in the two weeks preceding the survey according to selected background characteristics. Overall, 13 percent of all children under age 5 had diarrhea, and 2 percent had diarrhea with blood.

Table 14.6 Prevalence of diarrhea

Percentage of children under age 5 who had diarrhea in the two weeks preceding the survey, by background characteristics, Cambodia 2014

Background characteristic	Diarrhea in the two weeks preceding the survey: All diarrhea	Diarrhea in the two weeks preceding the survey: Diarrhea with blood	Number of children
**Age in months**			
<6	12.8	0.5	736
6-11	20.0	2.3	761
12-23	19.0	2.4	1,460
24-35	13.7	2.0	1,368
36-47	7.4	1.3	1,343
48-59	6.6	0.6	1,376
**Sex**			
Male	13.4	1.7	3,522
Female	12.2	1.4	3,523
**Residence**			
Urban	12.5	1.4	1,033
Rural	12.9	1.6	6,011
**Province**			
Banteay Meanchey	13.0	3.3	250
Kampong Cham	12.8	2.3	974
Kampong Chhnang	10.6	0.9	236
Kampong Speu	10.5	0.6	457
Kampong Thom	6.1	0.5	327
Kandal	9.0	0.2	506
Kratie	17.0	2.4	254
Phnom Penh	17.2	2.6	618
Prey Veng	4.7	0.7	478
Pursat	8.2	0.3	294
Siem Reap	16.9	2.5	470
Svay Rieng	5.6	0.0	253
Takeo	18.9	4.2	374
Otdar Meanchey	10.2	0.1	134
Battambang/Pailin	20.9	0.5	545
Kampot/Kep	5.1	0.9	272
Preah Sihanouk/ Koh Kong	17.0	3.0	164
Preah Vihear/ Stung Treng	19.3	2.2	228
Mondul Kiri/ Ratanak Kiri	15.6	0.8	208
**Mother's education**			
No education	13.3	2.2	973
Primary	12.9	1.3	3,687
Secondary and higher	12.4	1.6	2,384
**Wealth quintile**			
Lowest	16.1	2.2	1,689
Second	11.8	1.6	1,403
Middle	10.5	1.4	1,332
Fourth	13.6	1.3	1,217
Highest	11.3	1.1	1,404
**Source of drinking water during dry season[1]**			
Improved	11.4	1.5	4,472
Not improved	15.2	1.6	2,571
**Source of drinking water during rainy season[1,2]**			
Improved	12.3	1.6	5,833
Not improved	15.3	1.5	1,208
**Toilet facility[3]**			
Improved, not shared	11.0	1.2	2,941
Non-improved or shared	14.1	1.8	4,103
Total	12.8	1.5	7,044

[1] See Table 2.6 for definition of categories.
[2] Not including 4 missing cases
[3] See Table 2.7 for definition of categories.

The occurrence of diarrhea varies by age of the child. Similar to fever, young children age 6-11 and 12-23 months are more prone to diarrhea (20 percent and 19 percent, respectively) than children in the other age groups. The prevalence of diarrhea is about the same among rural children and urban children, and there is no variation by sex of the child. However, there are significant variations in the prevalence of diarrhea by province. Children living in Battambang/Pailin (21 percent), Preah Vihear/Stung Treng (19 percent), and Takeo (19 percent) are more susceptible to episodes of diarrhea than children living in other provinces. Children living in Kampot/Kep and Prey Veng have the lowest prevalence of diarrhea (5 percent each). The prevalence of diarrhea is higher among children who live in the poorest households, in households without an improved source of drinking water (in both the dry and rainy seasons), and in households with a non-improved or shared toilet facility.

The 2014 CDHS asked mothers of children under age 5 who had diarrhea what was done to treat the illness. Table 14.7 shows the percentage of children with diarrhea who received specific treatments according to background characteristics. Fifty-six percent of children with diarrhea were taken to a health provider. A larger percentage of children in rural areas and children living in the poorest households were taken to a health provider than other children. Children with bloody diarrhea are much more likely to be taken to a health provider. There is little variation by sex of the child in whether or not treatment for diarrhea was sought.

Comparable data from the 2010 CDHS show that the percentage of children with diarrhea taken to a health provider has not changed significantly (59 percent in 2010 versus 56 percent in 2014).

Fifty-seven percent of children with diarrhea were treated with a solution prepared from an oral rehydration salt (ORS) packet or tablet or were given increased fluids. Very few children with diarrhea were treated with antibiotics, antimotility drugs, or other medicines. Almost one in five children (18 percent) with diarrhea did not receive any treatment at all.

Diarrhea treatment varied by age: 39 percent of children less than age 6 months received ORT or increased fluids, as compared with 58-62 percent of children age 6 months and older. Children who had diarrhea with blood were more likely than children with non-bloody diarrhea to receive ORT or increased fluids (68 percent versus 56 percent).

Table 14.7 Diarrhea treatment

Among children under age 5 who had diarrhea in the two weeks preceding the survey, the percentage for whom advice or treatment was sought from a health facility or provider, the percentage given oral rehydration therapy (ORT), the percentage given increased fluids, the percentage given ORT or increased fluids, and the percentage who were given other treatments, by background characteristics, Cambodia 2014

Background characteristic	Percentage of children with diarrhea for whom advice or treatment was sought from a health facility or provider[1]	Oral rehydration therapy (ORT)	Increased fluids	ORT or increased fluids	Other treatments: Antibiotic drugs	Antimotility drugs	Zinc supplements	Intravenous solution	Home remedy/ other	No treatment	Number of children with diarrhea
**Age in months**											
<6	43.6	18.7	29.1	39.1	5.5	5.8	4.1	0.0	27.0	42.0	94
6-11	58.1	39.2	38.3	61.7	4.2	0.0	9.7	2.8	62.4	14.1	152
12-23	57.4	35.9	36.8	57.7	3.9	2.0	6.6	1.0	68.2	13.3	277
24-35	54.8	33.5	38.6	60.1	2.8	2.0	1.7	1.0	62.2	17.5	188
36-47	57.3	38.5	35.3	57.7	7.4	2.3	3.5	0.8	55.2	16.6	100
48-59	56.7	43.5	34.1	59.0	5.4	3.4	5.6	4.2	59.4	12.1	91
**Sex**											
Male	53.1	37.4	35.7	56.2	4.3	2.4	5.6	1.0	57.2	18.6	473
Female	58.1	32.9	36.7	58.0	4.5	2.0	5.2	2.1	61.7	16.4	429
**Type of diarrhea[2]**											
Non-bloody	53.4	32.7	36.4	55.6	3.7	2.3	4.8	1.2	57.9	18.8	786
Bloody	72.6	53.9	34.7	68.3	7.2	0.0	9.9	4.1	72.2	7.9	109
**Residence**											
Urban	47.0	30.2	40.1	59.1	3.8	0.9	3.3	2.7	65.3	19.2	129
Rural	56.9	36.1	35.5	56.7	4.5	2.5	5.8	1.3	58.3	17.3	772
**Province**											
Banteay Meanchey	(52.1)	(34.9)	(18.5)	(41.9)	(13.3)	(0.0)	(0.0)	(0.0)	(39.1)	(33.4)	32
Kampong Cham	(52.6)	(34.1)	(32.3)	(51.5)	(3.1)	(0.0)	(0.0)	(2.3)	(71.5)	(10.5)	124
Kampong Chhnang	(84.7)	(77.2)	(8.3)	(78.3)	(7.0)	(0.0)	(20.4)	(5.4)	(63.9)	(14.2)	25
Kampong Speu	(63.3)	(23.6)	(49.9)	(56.4)	(0.9)	(0.0)	(0.7)	(0.0)	(87.8)	(6.8)	48
Kampong Thom	*	*	*	*	*	*	*	*	*	*	20
Kandal	*	*	*	*	*	*	*	*	*	*	46
Kratie	59.8	20.2	32.8	47.4	1.1	0.0	18.4	0.0	70.5	18.7	43
Phnom Penh	57.5	33.5	52.0	69.2	2.9	0.5	3.1	3.8	73.6	14.9	107
Prey Veng	*	*	*	*	*	*	*	*	*	*	23
Pursat	(31.0)	(49.7)	(54.1)	(57.0)	(0.7)	(4.4)	(11.0)	(0.0)	(49.4)	(8.0)	24
Siem Reap	48.1	54.4	46.9	80.5	10.4	4.0	27.7	0.0	29.1	11.0	79
Svay Rieng	*	*	*	*	*	*	*	*	*	*	14
Takeo	52.5	57.7	37.5	74.3	10.0	0.0	0.0	4.2	56.7	16.3	71
Otdar Meanchey	(29.9)	(22.6)	(11.1)	(22.6)	(2.3)	(3.5)	(3.5)	(2.5)	(69.4)	(23.7)	14
Battambang/Pailin	48.3	22.3	46.5	62.2	7.5	13.1	1.4	0.0	46.9	17.9	114
Kampot/Kep	*	*	*	*	*	*	*	*	*	*	14
Preah Sihanouk/Koh Kong	58.8	47.9	30.4	63.4	2.4	0.0	8.4	0.0	41.1	26.4	28
Preah Vihear/Stung Treng	60.3	28.4	20.7	40.0	0.0	0.0	0.0	4.5	53.0	29.0	44
Mondul Kiri/Ratanak Kiri	53.3	42.8	12.8	45.7	0.0	0.0	0.0	0.0	57.2	25.4	32
**Mother's education**											
No education	58.8	46.3	34.4	61.3	1.7	1.5	7.6	0.0	57.6	15.1	129
Primary	55.2	34.6	33.1	54.9	5.5	2.8	5.3	1.8	55.7	21.0	477
Secondary and higher	54.3	31.4	41.9	58.6	3.9	1.7	4.6	1.7	65.9	13.0	296
**Wealth quintile**											
Lowest	62.0	39.8	33.2	56.3	2.9	2.3	6.3	2.2	62.0	16.2	271
Second	53.6	35.2	34.0	53.5	8.7	2.2	5.5	0.0	57.5	17.8	166
Middle	57.8	41.6	31.0	57.1	0.2	0.8	4.8	1.4	55.3	20.9	140
Fourth	55.7	30.6	39.5	57.6	5.8	5.2	4.4	0.8	60.0	12.2	166
Highest	43.8	26.7	44.7	61.4	4.9	0.3	5.3	2.5	59.6	22.1	158
Total	55.5	35.2	36.2	57.0	4.4	2.2	5.4	1.5	59.3	17.5	902

Note: Figures in parentheses are based on 25-49 unweighted cases. An asterisk indicates that a figure is based on fewer than 25 unweighted cases and has been suppressed. ORT includes fluid prepared from oral rehydration salt (ORS) packets and ORS tablets.
[1] Excludes pharmacy, shop, and traditional practitioner
[2] Excludes 7 cases for which information on type of diarrhea is missing

14.6 FEEDING PRACTICES

Mothers are normally encouraged to continue feeding children with diarrhea and to increase the amount of fluids given. These practices help to reduce dehydration and minimize the adverse consequences of diarrhea on the child's nutritional status. Mothers were asked whether they gave their child less, the same amount, or more fluids and food than usual when the child had diarrhea. Table 14.8 shows the percent distribution of children under age 5 who had diarrhea in the two weeks preceding the survey by feeding practices, according to background characteristics.

Table 14.8 Feeding practices during diarrhea

Percent distribution of children under age 5 who had diarrhea in the two weeks preceding the survey by amount of liquids and food offered compared with normal practice, the percentage of children given increased fluids and continued feeding during the diarrhea episode, and the percentage of children who continued feeding and were given ORT and/or increased fluids during the episode of diarrhea, by background characteristics, Cambodia 2014

	Amount of liquids given							Amount of food given										
Background characteristic	More	Same as usual	Somewhat less	Much less	None	Don't know/ missing	Total	More	Same as usual	Somewhat less	Much less	None	Never gave food	Don't know/ missing	Total	Percentage given increased fluids and continued feeding[1,2]	Percentage who continued feeding and were given ORT and/or increased fluids[3]	Number of children with diarrhea
**Age in months**																		
<6	29.1	63.4	1.3	1.5	4.7	0.0	100.0	4.5	18.1	1.3	0.7	0.0	75.5	0.0	100.0	3.6	5.7	94
6-11	38.3	53.4	4.8	1.6	1.9	0.0	100.0	9.9	70.2	12.3	0.3	0.0	6.7	0.6	100.0	35.8	58.0	152
12-23	36.8	49.4	8.6	4.4	0.2	0.7	100.0	21.7	60.1	14.7	1.6	1.0	0.9	0.0	100.0	35.6	55.6	277
24-35	38.6	48.9	7.0	5.5	0.0	0.0	100.0	15.3	61.3	19.1	4.3	0.0	0.0	0.0	100.0	37.6	58.1	188
36-47	35.3	47.4	13.9	2.3	0.0	1.0	100.0	11.6	62.4	23.2	1.7	0.0	0.0	1.0	100.0	35.3	55.9	100
48-59	34.1	47.7	14.1	2.4	0.0	1.6	100.0	17.3	68.4	13.3	1.0	0.0	0.0	0.0	100.0	34.1	58.3	91
**Sex**																		
Male	35.7	49.6	7.9	5.6	0.7	0.5	100.0	13.2	59.7	17.0	1.7	0.5	7.7	0.2	100.0	31.6	50.0	473
Female	36.7	52.6	8.1	1.1	1.1	0.5	100.0	17.1	57.7	12.0	1.9	0.1	11.0	0.2	100.0	33.6	53.3	429
**Type of diarrhea[4]**																		
Non-bloody	36.4	52.6	7.1	2.6	1.0	0.3	100.0	15.4	59.5	13.2	1.3	0.3	10.1	0.2	100.0	32.4	50.1	786
Bloody	34.7	41.1	12.5	9.9	0.0	1.8	100.0	10.1	53.4	25.8	5.8	0.6	4.4	0.0	100.0	33.3	62.6	109
**Residence**																		
Urban	40.1	48.9	6.4	3.8	0.1	0.8	100.0	10.2	63.5	15.6	0.5	0.0	9.3	0.8	100.0	38.5	55.8	129
Rural	35.5	51.4	8.3	3.4	1.0	0.4	100.0	15.8	57.9	14.5	2.0	0.4	9.3	0.1	100.0	31.5	50.9	772
**Province**																		
Banteay Meanchey	(18.5)	(66.0)	(15.5)	(0.0)	(0.0)	(0.0)	100.0	(8.7)	(59.9)	(24.4)	(0.0)	(0.0)	(7.1)	(0.0)	100.0	(18.5)	(37.8)	32
Kampong Cham	(32.3)	(50.8)	(11.3)	(5.6)	(0.0)	(0.0)	100.0	(2.3)	(80.4)	(17.0)	(0.0)	(0.0)	(0.3)	(0.0)	100.0	(32.3)	(51.5)	124
Kampong Chhnang	(8.3)	(87.4)	(1.1)	(3.2)	(0.0)	(0.0)	100.0	(14.4)	(82.4)	(0.0)	(0.0)	(0.0)	(3.2)	(0.0)	100.0	(8.3)	(78.3)	25
Kampong Speu	(49.9)	(48.6)	(0.7)	(0.7)	(0.0)	(0.0)	100.0	(15.1)	(58.2)	(15.2)	(4.9)	(0.0)	(6.6)	(0.0)	100.0	(39.9)	(45.7)	48
Kampong Thom	*	*	*	*	*	*	100.0	*	*	*	*	*	*	*	100.0	*	*	20
Kandal	*	*	*	*	*	*	100.0	*	*	*	*	*	*	*	100.0	*	*	46
Kratie	32.8	37.7	20.4	6.5	2.6	0.0	100.0	23.4	37.6	28.3	1.1	3.6	6.0	0.0	100.0	32.8	46.3	43
Phnom Penh	52.0	35.7	2.8	8.5	0.0	1.0	100.0	14.3	58.6	17.9	1.4	0.0	6.8	1.0	100.0	49.4	63.3	107
Prey Veng	*	*	*	*	*	*	100.0	*	*	*	*	*	*	*	100.0	*	*	23
Pursat	(54.1)	(36.1)	(0.0)	(6.5)	(3.3)	(0.0)	100.0	(44.2)	(33.1)	(8.0)	(5.8)	(0.0)	(9.0)	(0.0)	100.0	(54.1)	(57.0)	24
Siem Reap	46.9	36.3	12.7	2.2	0.0	1.9	100.0	17.5	49.1	17.1	4.4	0.0	11.9	0.0	100.0	36.7	68.2	79
Svay Rieng	*	*	*	*	*	*	100.0	*	*	*	*	*	*	*	100.0	*	*	14
Takeo	37.5	45.3	13.3	3.9	0.0	0.0	100.0	16.2	68.2	11.3	4.3	0.0	0.0	0.0	100.0	37.5	72.2	71
Otdar Meanchey	(11.1)	(77.9)	(11.0)	(0.0)	(0.0)	(0.0)	100.0	(22.3)	(66.7)	(10.1)	(0.0)	(0.0)	(0.9)	(0.0)	100.0	(11.1)	(22.6)	14
Battambang/Pailin	46.5	51.7	0.0	0.0	0.0	1.7	100.0	21.6	41.6	3.1	0.0	0.9	32.1	0.8	100.0	31.7	43.0	114
Kampot/Kep	*	*	*	*	*	*	100.0	*	*	*	*	*	*	*	100.0	*	*	14
Preah Sihanouk/Koh Kong	30.4	61.8	5.7	2.1	0.0	0.0	100.0	17.8	66.2	9.7	2.1	0.0	4.2	0.0	100.0	30.4	61.5	28
Preah Vihear/Stung Treng	20.7	61.3	9.1	0.0	8.9	0.0	100.0	6.4	64.4	19.9	0.0	0.0	9.3	0.0	100.0	20.7	39.4	44
Mondul Kiri/Ratanak Kiri	12.8	64.8	15.0	7.3	0.0	0.0	100.0	9.3	58.1	23.6	6.6	0.8	1.6	0.0	100.0	12.0	39.4	32
**Mother's education**																		
No education	34.4	47.7	12.5	2.8	1.1	1.5	100.0	13.7	61.9	12.6	4.2	0.2	6.8	0.7	100.0	30.6	54.8	129
Primary	33.1	54.9	7.9	3.0	0.8	0.2	100.0	13.7	57.4	16.0	1.3	0.3	11.1	0.2	100.0	30.0	49.6	477
Secondary and higher	41.9	46.3	6.2	4.3	0.8	0.5	100.0	17.7	59.6	13.2	1.6	0.4	7.4	0.0	100.0	37.4	53.4	296
**Wealth quintile**																		
Lowest	33.2	48.5	10.8	5.5	1.3	0.7	100.0	14.8	57.8	17.2	3.4	0.0	6.8	0.0	100.0	30.7	52.3	271
Second	34.0	55.4	7.4	2.9	0.4	0.0	100.0	14.7	56.3	16.5	2.7	0.4	9.4	0.0	100.0	28.8	47.1	166
Middle	31.0	59.7	7.1	1.8	0.4	0.0	100.0	17.0	55.0	17.9	1.1	0.5	8.5	0.0	100.0	29.0	53.7	140
Fourth	39.5	49.3	7.9	0.6	1.7	0.9	100.0	14.6	61.6	9.0	0.2	0.3	13.8	0.5	100.0	32.4	47.0	166
Highest	44.7	44.9	4.8	4.9	0.1	0.7	100.0	14.4	63.2	11.3	0.4	0.6	9.4	0.7	100.0	42.8	58.1	158
Total	36.2	51.0	8.0	3.4	0.9	0.5	100.0	15.0	58.7	14.6	1.8	0.3	9.3	0.2	100.0	32.5	51.6	902

Note: It is recommended that children be given more liquids to drink during diarrhea and that food not be reduced. Figures in parentheses are based on 25-49 unweighted cases. An asterisk indicates that a figure is based on fewer than 25 unweighted cases and has been suppressed.
[1] Equivalent to the UNICEF/WHO indicator "Home management of diarrhea" (MICS Indicator 34)
[2] Continued feeding includes children who were given more, the same as usual, or somewhat less food during the diarrhea episode.
[3] Equivalent to UNICEF MICS Indicator 35
[4] Excludes 7 cases for which information on type of diarrhea is missing

Fifty-one percent of children who had diarrhea were given the same amount of liquid as usual, and 36 percent were given more. Eight percent of children were given somewhat less than the usual amount, and 3 percent were given much less than the usual amount. Less than 1 percent of children who had diarrhea were given no liquids.

Regarding the amount of food offered to children who had diarrhea, 59 percent were given the same as usual, 15 percent were given more than usual, another 15 percent were given somewhat less than usual, 2 percent were given much less than usual, and less than 1 percent did not receive food during their illness.

Overall, one-third of children with diarrhea were given increased fluids with continued feeding (i.e., more, the same amount as usual, or somewhat less to eat). Just over half of children with diarrhea continued feeding and were given ORT and/or increased fluids.

Children under age 6 months were more likely than older children to receive the same amount of liquid or more during episodes of diarrhea. Children with bloody diarrhea were less likely than those with non-bloody diarrhea to receive the same amount of food or more.

14.7 KNOWLEDGE OF ORS PACKETS

A simple and effective response to dehydration caused by diarrhea is a prompt increase in the child's fluid intake through some form of oral rehydration therapy, which may include the use of a solution prepared from packets of oral rehydration salts. To ascertain how widespread knowledge of ORS is in Cambodia, respondents were asked whether they know about ORS packets or Oralyte/Orasel. Interviewers displayed a sample ORS packet to respondents when asking the question.

Table 14.9 shows that nearly all (96 percent) of the women who gave birth in the five years preceding the survey know about ORS packets. In the 2010 CDHS, almost the same proportion of women reported knowing about ORS packets (95 percent).

Young mothers age 15-19 are less likely than older mothers to know about ORS. Mothers with no schooling are less likely to know about ORS packets (90 percent) than mothers with a primary school education (96 percent) or a secondary education or higher (98 percent). Mothers in Mondul Kiri/Ratanak Kiri (82 percent) are least likely to know about ORS packets.

Table 14.9 Knowledge of ORS packets or pre-packaged liquids

Percentage of women age 15-49 with a live birth in the five years preceding the survey who know about ORS for treatment of diarrhea, by background characteristics, Cambodia 2014

Background characteristic	Percentage of women who know about ORS	Number of women
**Age**		
15-19	82.1	213
20-24	94.9	1,442
25-34	97.2	3,345
35-49	94.9	974
**Residence**		
Urban	98.4	876
Rural	95.3	5,096
**Province**		
Banteay Meanchey	95.1	219
Kampong Cham	92.8	819
Kampong Chhnang	100.0	203
Kampong Speu	95.6	395
Kampong Thom	100.0	279
Kandal	92.6	420
Kratie	90.7	214
Phnom Penh	99.2	535
Prey Veng	99.7	405
Pursat	98.0	245
Siem Reap	99.5	379
Svay Rieng	99.1	229
Takeo	96.3	321
Otdar Meanchey	95.3	116
Battambang/Pailin	98.5	460
Kampot/Kep	89.3	236
Preah Sihanouk/ Koh Kong	96.2	142
Preah Vihear/ Stung Treng	91.4	188
Mondul Kiri/ Ratanak Kiri	82.4	169
**Education**		
No education	89.8	805
Primary	95.8	3,100
Secondary and higher	98.0	2,068
**Wealth quintile**		
Lowest	93.0	1,359
Second	93.9	1,215
Middle	96.0	1,133
Fourth	97.1	1,069
Highest	99.3	1,196
Total	95.8	5,973

ORS = Oral rehydration salts

14.8 STOOL DISPOSAL

If human feces are left uncontained, disease may spread by direct contact or by animal contact with the feces. Hence, the proper disposal of children's stools is extremely important in preventing the spread of disease. Table 14.10 presents information on disposal of the stools of children under age 5, by background characteristics.

Almost 30 percent of children's stools are left uncontained: 5 percent are put or rinsed into a drain or ditch, 6 percent are thrown into the garbage, and 19 percent are rinsed away. Seventy-one percent of

children’s stools are disposed of hygienically: 39 percent are buried in the yard, 15 percent are disposed of in a toilet or latrine, and 16 percent of children under age 5 use a toilet or latrine.

The stools of children less than age 6 months (47 percent) were less likely to be disposed of hygienically than the stools of older children. There are significant differences in stool disposal practices by mother’s level of education. Stools are disposed of hygienically (the child uses a toilet, the child’s stool is thrown in a toilet or buried in the yard) for 75 percent of children whose mothers have a secondary education or higher, as compared with 60 percent of children of mothers with no schooling.

Proper disposal of children’s stools does not differ between urban and rural areas. However, there are large provincial variations in stool disposal practices. The percentage of children whose stools are contained through safe disposal ranges from a low of 35 percent in Mondul Kiri/Ratanak Kiri and 46 percent in Kampot/Kep to a high of 91 percent in Kampong Chhnang.

Table 14.10 Disposal of children’s stools

Percent distribution of youngest children under age 5 living with their mother by the manner of disposal of the child’s last fecal matter, and percentage of children whose stools are disposed of safely, according to background characteristics, Cambodia 2014

	Manner of disposal of children’s stools										
Background characteristic	Child used toilet or latrine	Put/rinsed into toilet or latrine	Buried	Put/rinsed into drain or ditch	Thrown into garbage	Not disposed of, left in the open	Other	Missing	Total	Percentage of children whose stools are disposed of safely[1]	Number of children
**Age in months**											
<6	1.5	14.8	30.8	16.6	16.4	19.4	0.3	0.2	100.0	47.0	717
6-11	4.3	17.9	41.1	7.2	11.0	18.5	0.0	0.0	100.0	63.3	745
12-23	7.5	18.5	43.7	3.7	6.1	20.3	0.1	0.1	100.0	69.7	1,398
24-35	15.5	19.0	41.7	2.4	3.2	18.2	0.0	0.0	100.0	76.2	1,142
36-47	25.9	9.5	42.6	1.3	0.6	19.9	0.0	0.1	100.0	78.1	949
48-59	42.0	9.8	31.3	1.6	0.2	15.1	0.0	0.0	100.0	83.1	874
**Residence**											
Urban	31.0	33.4	6.9	4.2	20.4	4.1	0.0	0.0	100.0	71.3	836
Rural	13.6	12.3	44.8	4.9	3.2	21.2	0.1	0.1	100.0	70.6	4,988
**Province**											
Banteay Meanchey	17.7	18.0	45.1	6.1	6.4	6.7	0.0	0.0	100.0	80.8	218
Kampong Cham	8.2	13.6	51.9	1.9	1.8	22.6	0.0	0.0	100.0	73.6	802
Kampong Chhnang	8.4	11.9	70.6	1.7	1.8	5.6	0.0	0.0	100.0	91.0	198
Kampong Speu	15.5	9.9	45.4	1.1	4.4	23.6	0.0	0.0	100.0	70.9	389
Kampong Thom	17.8	10.3	56.8	1.1	5.0	8.6	0.3	0.0	100.0	85.0	271
Kandal	28.5	16.4	38.8	4.8	2.5	8.9	0.0	0.0	100.0	83.7	412
Kratie	5.2	12.2	43.5	6.9	2.4	29.8	0.1	0.0	100.0	60.9	205
Phnom Penh	32.5	29.6	4.3	5.2	24.4	3.9	0.0	0.0	100.0	66.5	503
Prey Veng	16.5	5.3	46.9	1.0	0.1	30.3	0.0	0.0	100.0	68.7	397
Pursat	8.0	16.9	40.9	8.6	2.2	23.4	0.0	0.0	100.0	65.8	243
Siem Reap	9.7	12.4	57.8	11.2	6.2	2.2	0.0	0.4	100.0	80.0	370
Svay Rieng	25.8	8.5	29.4	6.7	2.1	27.5	0.0	0.0	100.0	63.7	223
Takeo	16.1	30.2	27.6	1.7	2.2	22.1	0.0	0.0	100.0	74.0	317
Otdar Meanchey	14.8	10.4	31.9	0.0	3.0	39.9	0.0	0.0	100.0	57.1	113
Battambang/Pailin	20.0	19.9	38.5	12.6	3.2	5.5	0.4	0.0	100.0	78.5	449
Kampot/Kep	7.4	7.2	31.5	6.2	14.5	32.7	0.0	0.5	100.0	46.1	232
Preah Sihanouk/ Koh Kong	21.2	26.4	15.2	5.2	16.5	15.0	0.0	0.5	100.0	62.8	136
Preah Vihear/ Stung Treng	2.9	12.5	34.6	4.6	2.8	42.6	0.0	0.0	100.0	50.0	183
Mondul Kiri/ Ratanak Kiri	18.0	6.9	10.1	4.6	5.5	54.5	0.4	0.0	100.0	35.0	164
**Mother’s education**											
No education	8.4	5.4	46.2	4.5	1.6	34.0	0.1	0.0	100.0	59.9	774
Primary	14.3	11.6	44.7	5.7	3.9	19.7	0.0	0.1	100.0	70.6	3,041
Secondary and higher	21.8	24.7	28.6	3.6	9.8	11.3	0.1	0.1	100.0	75.1	2,009
**Wealth quintile**											
Lowest	1.5	1.3	57.3	4.7	1.9	33.1	0.0	0.1	100.0	60.2	1,327
Second	7.0	6.2	51.0	6.5	2.1	27.0	0.0	0.0	100.0	64.3	1,181
Middle	13.5	12.9	48.9	5.3	2.6	16.7	0.0	0.1	100.0	75.3	1,119
Fourth	26.2	25.2	28.3	4.8	5.1	10.2	0.2	0.0	100.0	79.8	1,050
Highest	35.6	34.1	7.2	2.7	17.1	3.2	0.1	0.0	100.0	76.9	1,147
Total	16.1	15.3	39.3	4.8	5.7	18.7	0.1	0.1	100.0	70.7	5,824

[1] Children’s stools are considered to be disposed of safely if the child used a toilet or latrine, if the fecal matter was put/rinsed into a toilet or latrine, or if it was buried.

EARLY CHILDHOOD EDUCATION AND DEVELOPMENT 15

Key Findings

- Fifteen percent of children age 36-59 months are attending an organized early childhood education program.
- Fifty-nine percent of children engaged with an adult household member (including parents) in four or more activities that promote learning and school readiness during the three days before the survey.
- Only 4 percent of children under age 5 have at least three children's books.
- One in 10 children under age 5 had been left alone or left in the care of other children under age 10 for one hour or more during the week preceding the interview.

Children are the foundation of sustainable development. The early years of life are crucial not only for individual health and physical development, but also for cognitive and social-emotional development. Events in the first few years of life are formative and play a vital role in building human capital, breaking the cycle of poverty, promoting economic productivity, and eliminating social disparities and inequities. This chapter provides key data on early childhood education and development collected in the 2014 CDHS. These data will help the Cambodian government, civil society, communities, and other stakeholders design and implement programs and policies that help young children reach their full potential by supporting families and communities and increasing access to quality early childhood care and education.

15.1 Early Childhood Education and Learning

The readiness of children for primary school can be improved through early childhood education programs such as preschools. Early childhood education programs include programs that have organized learning components; they do not include those characterized primarily as baby-sitting or day-care programs, which typically do not include organized learning activities. In the 2014 CDHS, women with a child born in the five years before the survey were asked questions regarding early childhood education and learning. In the case of women with more than one child under age 5, questions referred to the youngest child.

The data show that 15 percent of children age 36-59 months are attending an organized early childhood education program (Table 15.1). Children living in urban areas (36 percent) are much more likely to attend an early childhood education program than children living in rural areas (11 percent). Participation in early childhood education varies substantially by province, from a high of 40 percent among children in Phnom Penh to a low of only 5 percent among children in Pursat. Considerable differences are observed by mother's education and household wealth quintile. Only 7 percent of children whose mothers have no education attend an early childhood education program, as compared with 26 percent of children whose mothers have a secondary education or higher. Thirty-eight percent of children living in the richest households attend an early childhood education program, compared with only 7 percent of children in the poorest households.

It is recognized that a period of rapid brain development occurs in the first three to four years of life and that the quality of home care is the major determinant of a child's development during this period. In this context, the amount of "quality time" adults spend with children, the presence of children's books in

the home, opportunities for play to stimulate the imagination, and conditions of care are all important indicators of quality of home care. Children should be physically healthy, mentally alert, emotionally secure, socially competent, and ready to learn.

Information on a number of activities that support early learning was collected for children age 3-4 who were living with their mothers. The activities asked about focused on the involvement of adults with children in the following activities: reading books or looking at picture books; telling stories; singing songs; taking children outside the home, compound, or yard; playing with children; and spending time with children naming, counting, or drawing things.

Table 15.2 shows the percentage of children age 36-59 months who engaged with an adult household member in activities that promote learning and school readiness within the three days prior to the survey. Fifty-nine percent of children engaged with an adult household member (including parents) in four or more such activities. The average number of activities in which adults engaged with children was 4.6. Nearly all (92 percent) children age 36-59 months live with their biological fathers; of these children, only 9 percent engaged with their father in four or more early educational activities. The average number of activities in which fathers involved themselves with children was 1.1. The involvement of mothers in early childhood learning activities was somewhat better than that of fathers. Seventeen percent of children engaged with their mothers in four or more such activities, with an average of 1.7 activities.

There was only a slight difference between boys and girls with respect to adults' engagement in activities that promote learning and school readiness. A larger percentage of children in urban areas (72 percent) than rural areas (57 percent) engaged with adults in early education activities. Strong differentials are observed by educational level of the mother and father, as well as by socioeconomic status. The percentage of children who have an adult engage with them in four or more learning activities rises steadily with increasing parents' education and increasing household wealth. For example, 73 percent of children living in the richest households had an adult engage with them in four or more learning-related activities, as opposed to 48 percent of those living in the poorest households. Patterns by background characteristics in fathers' and mothers' involvement in such activities were similar.

Table 15.1 Early childhood education

Percentage of children age 36-59 months who are attending an organized early childhood education program, according to background characteristics, Cambodia 2014

Background characteristic	Percentage of children age 36-59 months attending early childhood education[1]	Number of children age 36-59 months
**Age in months**		
36-47	7.3	1,303
48-59	21.7	1,314
**Child's sex**		
Male	11.9	1,303
Female	17.1	1,314
**Residence**		
Urban	36.4	348
Rural	11.2	2,269
**Province**		
Banteay Meanchey	18.2	73
Kampong Cham	7.0	395
Kampong Chhnang	14.9	91
Kampong Speu	19.2	178
Kampong Thom	15.9	119
Kandal	12.0	181
Kratie	5.6	93
Phnom Penh	39.8	209
Prey Veng	11.4	188
Pursat	4.7	101
Siem Reap	15.1	184
Svay Rieng	9.7	102
Takeo	8.7	146
Otdar Meanchey	13.9	48
Battambang/Pailin	24.0	178
Kampot/Kep	8.3	104
Preah Sihanouk/ Koh Kong	21.2	60
Preah Vihear/ Stung Treng	11.1	83
Mondul Kiri/ Ratanak Kiri	7.4	85
**Mother's education**		
No education	7.2	413
Primary	10.3	1,423
Secondary and higher	26.1	782
**Wealth quintile**		
Lowest	6.6	661
Second	7.2	564
Middle	7.9	473
Fourth	16.4	423
Highest	38.1	497
Total	14.5	2,617

[1] Not including baby-sitting or day care

Table 15.2 Support for learning

Percentage of children age 36-59 months living with their mothers with whom adult household members engaged in four or more activities that promote learning and school readiness during the last three days, the mean number of such activities, and engagement in such activities by biological fathers and mothers, according to background characteristics, Cambodia 2014

Background characteristic	Percentage of children with whom adult household members[1] have engaged in four or more activities[2]	Mean number of activities with adult household members	Percentage of children living with their biological father	Number of children age 36-59 months	Percentage of children with whom biological fathers have engaged in four or more activities	Mean number of activities with biological fathers	Number of children age 36-59 months living with their biological fathers	Percentage of children with whom biological mothers have engaged in four or more activities	Mean number of activities with biological mothers	Number of children age 36-59 months
**Age in months**										
36-47	59.0	4.5	93.3	1,303	8.3	1.1	1,216	16.0	1.7	1,303
48-59	59.7	4.7	90.4	1,314	9.0	1.2	1,187	18.0	1.7	1,314
**Child's sex**										
Male	56.5	4.4	90.9	1,303	7.5	1.1	1,185	15.7	1.6	1,303
Female	62.2	4.7	92.7	1,314	9.8	1.1	1,218	18.4	1.8	1,314
**Residence**										
Urban	71.9	5.2	88.6	348	11.8	1.4	309	24.0	2.2	348
Rural	57.4	4.5	92.3	2,269	8.2	1.1	2,094	16.0	1.6	2,269
**Province**										
Banteay Meanchey	57.3	4.2	92.0	73	8.8	0.9	67	14.9	1.7	73
Kampong Cham	78.9	5.5	92.2	395	12.4	1.6	365	18.5	2.2	395
Kampong Chhnang	74.7	6.1	86.6	91	7.9	1.3	79	29.3	2.5	91
Kampong Speu	35.6	2.8	95.4	178	3.8	1.0	170	6.3	1.1	178
Kampong Thom	64.8	5.0	97.3	119	4.8	0.8	115	7.4	0.9	119
Kandal	71.5	5.9	93.7	181	11.9	1.4	170	29.4	2.5	181
Kratie	32.1	2.6	94.9	93	1.2	0.5	88	6.7	1.0	93
Phnom Penh	79.9	5.6	88.8	209	10.7	1.6	185	24.1	2.3	209
Prey Veng	79.4	7.0	86.9	188	30.8	2.3	163	49.0	3.3	188
Pursat	55.5	4.2	95.2	101	7.7	1.0	96	17.5	1.9	101
Siem Reap	35.2	2.9	90.3	184	4.2	0.4	166	4.4	0.8	184
Svay Rieng	35.5	2.8	92.6	102	7.2	0.8	94	8.3	1.2	102
Takeo	57.0	4.1	89.4	146	2.4	0.7	131	11.6	1.1	146
Otdar Meanchey	70.2	5.6	95.8	48	10.3	1.1	46	10.5	1.3	48
Battambang/Pailin	45.3	3.0	89.5	178	0.0	0.3	159	1.3	0.4	178
Kampot/Kep	42.5	3.8	94.1	104	1.3	0.7	97	17.7	1.4	104
Preah Sihanouk/ Koh Kong	70.6	6.1	92.1	60	11.6	1.4	55	31.6	2.7	60
Preah Vihear/ Stung Treng	70.2	5.7	93.4	83	8.0	1.1	77	16.0	1.7	83
Mondul Kiri/ Ratanak Kiri	20.0	2.3	91.7	85	5.5	0.7	78	5.1	0.5	85
**Mother's education**										
No education	45.3	3.7	92.2	413	4.8	0.8	380	7.6	1.1	413
Primary	57.2	4.3	92.2	1,423	7.3	1.0	1,312	12.8	1.5	1,423
Secondary and higher	70.6	5.6	90.8	782	13.2	1.5	710	29.7	2.3	782
**Father's education**										
No education	42.6	3.3	100.0	241	1.0	0.6	241	6.9	1.1	241
Primary	55.8	4.2	100.0	1,145	5.2	0.9	1,145	13.5	1.5	1,145
Secondary and higher	68.2	5.3	100.0	1,016	14.3	1.5	1,016	22.4	2.0	1,016
Not living with father	55.1	4.2	0.0	214	0.0	0.0	0	21.6	1.8	214
**Wealth quintile**										
Lowest	48.2	3.9	94.6	661	5.0	0.9	625	10.9	1.4	661
Second	53.7	4.1	92.5	564	5.7	0.9	521	15.3	1.4	564
Middle	60.7	4.6	89.5	473	9.4	1.1	423	17.2	1.7	473
Fourth	66.6	5.1	91.2	423	10.1	1.3	386	18.0	1.8	423
Highest	73.1	5.6	90.1	497	15.3	1.6	447	26.2	2.2	497
Total	59.3	4.6	91.8	2,617	8.6	1.1	2,403	17.0	1.7	2,617

[1] Including parents or other adult members of the household
[2] Including the following activities: reading books or looking at picture books; telling stories; singing songs; taking children outside the home, compound, or yard; playing with children; and spending time with children naming, counting, or drawing things

Exposure to books in the early years not only provides children with a greater understanding of the nature of print but may also give them opportunities to see others reading (e.g., older siblings doing schoolwork). The presence of books is also important for later school performance. Mothers of children age under age 5 were asked about the number of children's books or picture books they have. By stimulating the imagination, play also contributes to brain development. Mothers were asked what items

children play with, including homemade toys, toys purchased from a shop, and other household objects or objects found around or outside the home.

In Cambodia, only 4 percent of children under age 5 have at least three children's books (Table 15.3). One factor that contributes to the relatively low figure is that questions were asked about the woman's youngest child under age 5. Consequently, Table 15.3 does not adequately reflect older children in this age group but, rather, is disproportionally based on younger children.

No differences are observed between boys and girls. A higher percentage of urban than rural children have access to three or more children's books (11 percent and 3 percent, respectively).

Table 15.3 also shows that 34 percent of children under age 5 play with two or more types of playthings: homemade toys (including dolls and cars), toys purchased from a store, and household objects (such as pots and bowls) along with objects and materials found outside the home (such as sticks, rocks, animal shells, and leaves). Nearly 1 in 2 children (48 percent) play with toys that come from a store, while 23 percent play with homemade toys. The percentage of children who play with two or more types of playthings is higher in urban areas than in rural areas. This percentage increases with increasing mother's education and household wealth.

Table 15.3 Learning materials

Percentage of the youngest children under age 5 with three or more children's books and percentage who play with various types of playthings, according to background characteristics, Cambodia 2014

		Percentage of children who play with:				
Background characteristic	Percentage of children who have 3 or more children's books	Homemade toys	Toys from a shop/ manufactured toys	Household objects/objects found outside	Two or more types of playthings	Number of children under age 5
**Age in months**						
0-23	1.6	16.4	37.4	21.5	21.4	2,859
24-59	6.1	29.4	57.7	50.5	45.5	2,964
**Child's sex**						
Male	3.9	24.1	50.7	35.9	34.8	2,912
Female	3.9	21.9	44.9	36.6	32.5	2,912
**Residence**						
Urban	10.8	22.5	73.8	46.1	48.6	836
Rural	2.7	23.1	43.4	34.6	31.2	4,988
**Province**						
Banteay Meanchey	6.3	57.0	51.1	47.1	54.3	218
Kampong Cham	4.5	15.1	53.4	29.3	27.7	802
Kampong Chhnang	1.5	34.8	27.2	16.9	17.5	198
Kampong Speu	2.5	17.3	57.0	50.1	42.9	389
Kampong Thom	0.2	26.8	41.1	50.2	36.2	271
Kandal	4.0	24.2	67.8	30.0	31.3	412
Kratie	1.5	3.7	46.4	14.2	11.8	205
Phnom Penh	11.0	6.9	78.8	57.0	54.3	503
Prey Veng	1.9	38.1	43.1	41.9	40.3	397
Pursat	1.9	32.0	36.6	42.0	33.8	243
Siem Reap	5.5	25.2	30.6	37.9	27.6	370
Svay Rieng	1.6	31.9	29.3	18.8	28.4	223
Takeo	4.3	17.7	28.2	20.3	22.1	317
Otdar Meanchey	6.3	35.6	33.0	41.0	38.0	113
Battambang/Pailin	3.6	8.9	52.6	43.3	33.1	449
Kampot/Kep	1.4	20.6	30.8	18.2	23.0	232
Preah Sihanouk/ Koh Kong	7.0	43.0	64.3	39.8	50.8	136
Preah Vihear/ Stung Treng	0.7	35.4	23.2	45.0	33.6	183
Mondul Kiri/ Ratanak Kiri	1.9	25.7	50.0	21.6	25.2	164
**Mother's education**						
No education	1.6	17.3	27.3	31.6	19.7	774
Primary	1.5	21.9	43.5	36.1	31.3	3,041
Secondary and higher	8.4	26.8	62.0	38.3	42.6	2,009
**Wealth quintile**						
Lowest	1.0	17.5	24.9	31.0	19.6	1,327
Second	1.4	22.1	32.8	32.7	26.4	1,181
Middle	1.3	22.7	46.8	33.7	31.0	1,119
Fourth	4.4	27.8	58.0	38.7	41.2	1,050
Highest	11.9	26.3	81.2	46.2	53.0	1,147
Total	3.9	23.0	47.8	36.2	33.7	5,824

15.2 ADEQUATE CARE FOR YOUNG CHILDREN

Leaving children alone or only in the presence of other young children is known to increase the risk of accidents. In the 2014 CDHS, mothers were asked two questions to establish whether their youngest child age 0-59 months had been left alone during the week preceding the interview for one hour or more and whether the child was left in the care of other children under age 10 for one hour or more.

Table 15.4 shows that 8 percent of children under age 5 had been left in the care of other children under age 10. Five percent of children under age 5 were left completely alone (under the care of no one) for at least one hour during the week preceding the interview. A child under age 5 left only in the care of another child or left alone is considered inadequately cared for. According to the data, 1 in 10 children under age 5 received inadequate care.

Table 15.4 Inadequate care

Percentage of youngest children under age 5 who were left alone or left in the care of another child younger than age 10 for more than one hour at least once during the past week, according to background characteristics, Cambodia 2014

Background characteristic	Left alone in the past week	Left in the care of another child younger than age 10 in the past week	Left with inadequate care in the past week	Number of children under age 5
**Age in months**				
0-23	2.6	5.4	6.3	2,859
24-59	6.9	10.1	13.3	2,964
**Child's sex**				
Male	5.2	7.2	10.1	2,912
Female	4.4	8.4	9.6	2,912
**Residence**				
Urban	2.5	3.2	4.8	836
Rural	5.2	8.6	10.7	4,988
**Province**				
Banteay Meanchey	2.8	0.4	3.3	218
Kampong Cham	11.9	9.4	14.9	802
Kampong Chhnang	7.3	8.1	10.7	198
Kampong Speu	3.4	5.3	6.3	389
Kampong Thom	0.0	0.0	0.0	271
Kandal	1.5	7.4	7.6	412
Kratie	2.3	11.0	11.3	205
Phnom Penh	1.7	2.0	3.3	503
Prey Veng	0.7	0.2	0.7	397
Pursat	10.8	12.4	16.3	243
Siem Reap	7.9	12.6	16.7	370
Svay Rieng	3.8	4.6	5.9	223
Takeo	1.9	4.6	4.6	317
Otdar Meanchey	11.1	17.4	20.1	113
Battambang/Pailin	5.2	21.0	23.8	449
Kampot/Kep	1.9	9.0	9.0	232
Preah Sihanouk/ Koh Kong	4.7	13.2	14.7	136
Preah Vihear/ Stung Treng	3.2	8.0	9.9	183
Mondul Kiri/ Ratanak Kiri	3.1	5.0	5.5	164
**Mother's education**				
No education	10.5	14.5	19.9	774
Primary	4.9	8.5	10.1	3,041
Secondary and higher	2.4	4.2	5.5	2,009
**Wealth quintile**				
Lowest	8.0	12.1	15.6	1,327
Second	5.7	9.6	11.3	1,181
Middle	4.5	6.8	8.9	1,119
Fourth	3.8	6.1	7.9	1,050
Highest	1.3	3.6	4.4	1,147
Total	4.8	7.8	9.8	5,824

Children age 24-59 months were twice as likely to be left without adequate care (13 percent) as children age 0-23 months (6 percent). There was no variation in the proportion left with inadequate care by sex of the child. A higher percentage of rural children (11 percent) than urban children (5 percent) received inadequate care, and there were substantial differences by province. In addition, differences were observed with regard to both the educational level of the mother and the socioeconomic status of the household. The proportion of children who were left with inadequate care was three times higher among those whose mothers had no education than among those whose mothers had a secondary education or higher (20 percent versus 6 percent). Similarly, 16 percent of children living in the poorest households were left with inadequate care, as compared with 4 percent of children living in the wealthiest households.

15.3 Early Childhood Development

Early child development is defined as an orderly, predictable process along a continuous path in which a child learns to handle more complicated levels of moving, thinking, speaking, feeling, and relating to others. Physical growth, literacy and numeracy skills, socio-emotional development, and readiness to learn are vital domains of a child's overall development, which is a basis for overall human development.

A 10-item module was used to calculate the Early Child Development Index (ECDI). The ECDI is based on benchmarks that children are expected to reach if they are progressing in their development at a pace similar to the majority of children in their age group. The primary purpose of the ECDI is to inform public policy regarding the developmental status of children in Cambodia. Each of the 10 items is used in one of four domains to determine whether children are developmentally on track in that domain. The domains in question are as follows.

- **Literacy-numeracy:** Children are identified as being developmentally on track according to whether they can identify/name at least 10 letters of the alphabet; whether they can read at least four simple, popular words; and whether they know the names and recognize the symbols of all numbers from 1 to 10. If at least two of these capabilities are observed, the child is considered developmentally on track.

- **Physical:** If the child can pick up a small object such as a stick or a rock from the ground with two fingers and/or the mother does not indicate that the child is sometimes too sick to play, then the child is regarded as being developmentally on track in the physical domain.

- **Social-emotional:** A child is considered to be developmentally on track if two of the following are true: the child gets along well with other children; the child does not kick, bite, or hit other children; and the child does not become distracted easily.

- **Learning:** If the child follows simple directions on how to do something correctly and/or when given something to do, and is able to do it independently, then the child is considered to be developmentally on track in this domain.

The ECDI score is calculated as the percentage of children who are developmentally on track in at least three of these four domains.

The percentages of children age 36-59 months who are developmentally on track in the literacy-numeracy, physical, social-emotional, and learning domains, as well as ECDI scores, are presented in Table 15.5. Analysis of the four domains of child development shows that at least 7 in 10 children are on track in the physical, social-emotional, and learning domains (70-91 percent). However, only about 1 in 4 children age 36-59 months (27 percent) are developmentally on track in literacy-numeracy. There is practically no difference in literacy-numeracy between boys and girls. A much higher proportion of urban children than rural children are on track in the literacy-numeracy domain (43 percent versus 25 percent). Forty-one percent of children whose mothers have a secondary education or higher are on track in the literacy-numeracy domain, as compared with only 13 percent of children whose mothers have no education. Nearly half of children (46 percent) in the richest households are on track in the literacy-numeracy domain, compared with 18 percent of children in the poorest households.

Table 15.5 Early Child Development Index

Percentage of children age 36-59 months who are developmentally on track in literacy-numeracy, physical, social-emotional, and learning domains, and the Early Child Development Index score, according to background characteristics, Cambodia 2014

Background characteristic	Percentage of children age 36-59 months who are developmentally on track for indicated domains				Early Child Development Index score[2]	Number of children age 36-59 months
	Literacy-numeracy[1]	Physical[1]	Social-emotional[1]	Learning[1]		
**Age in months**						
36-47	21.0	89.2	70.1	88.5	64.7	1,303
48-59	33.6	93.5	69.6	90.8	71.7	1,314
**Child's sex**						
Male	26.6	91.2	69.0	89.7	67.1	1,303
Female	28.1	91.6	70.7	89.7	69.3	1,314
**Residence**						
Urban	42.5	94.9	72.6	95.5	78.2	348
Rural	25.0	90.8	69.4	88.8	66.7	2,269
**Province**						
Banteay Meanchey	55.9	98.8	82.5	98.8	88.7	73
Kampong Cham	28.8	88.3	73.1	93.9	68.4	395
Kampong Chhnang	59.1	100.0	79.5	85.6	85.8	91
Kampong Speu	7.7	97.6	67.5	74.6	49.1	178
Kampong Thom	32.0	85.1	78.6	93.9	69.3	119
Kandal	23.4	77.9	53.4	98.6	49.3	181
Kratie	14.3	81.2	66.3	83.5	55.1	93
Phnom Penh	48.0	97.7	77.3	98.1	84.2	209
Prey Veng	48.9	93.5	89.7	92.8	90.9	188
Pursat	14.7	97.6	70.8	88.7	67.4	101
Siem Reap	15.4	92.0	64.6	86.1	61.0	184
Svay Rieng	18.5	95.2	67.1	90.8	70.1	102
Takeo	25.4	85.3	58.8	76.4	60.0	146
Otdar Meanchey	17.7	75.6	54.1	81.9	48.6	48
Battambang/Pailin	8.3	97.0	76.1	97.5	80.3	178
Kampot/Kep	39.9	97.7	72.6	93.2	76.8	104
Preah Sihanouk/ Koh Kong	49.2	96.7	76.1	94.4	86.6	60
Preah Vihear/ Stung Treng	4.4	96.4	72.9	95.4	68.6	83
Mondul Kiri/ Ratanak Kiri	12.6	83.2	20.7	57.5	24.8	85
**Mother's education**						
No education	12.9	89.0	69.9	88.2	61.4	413
Primary	24.1	90.5	68.7	88.8	65.7	1,423
Secondary and higher	40.8	94.1	71.9	92.0	76.3	782
**Wealth quintile**						
Lowest	18.0	88.5	67.5	85.3	61.0	661
Second	21.4	92.6	70.5	89.9	67.3	564
Middle	23.7	91.5	69.2	89.7	66.2	473
Fourth	32.2	90.4	70.3	89.7	69.4	423
Highest	45.8	94.5	72.5	95.1	79.7	497
Total	27.3	91.4	69.8	89.7	68.2	2,617

[1] See the text for the items included in each domain.
[2] Percentage of children who are developmentally on track for at least three of the four domains

Seven in 10 children age 36-59 months (68 percent) are developmentally on track (i.e., on track in at least three of the four domains). Urban children are more likely than rural children to be developmentally on track (78 percent versus 67 percent). The proportion of children developmentally on track varies substantially by province, from a low of 25 percent in Mondul Kiri/Ratanak Kiri to a high of 91 percent in Prey Veng. ECDI scores are positively associated with mother's education and household wealth (Table 15.5).

NUTRITION OF CHILDREN AND WOMEN 16

Key Findings

- Thirty-two percent of children under age 5 are stunted, 10 percent are wasted, and 24 percent are underweight.
- Breastfeeding is nearly universal in Cambodia. Ninety-six percent of children born in the last two years have been breastfed.
- The median duration of breastfeeding among children born in the three years before the survey is 18 months.
- Sixty-five percent of children less than age 6 months are exclusively breastfed, and the median duration of exclusive breastfeeding is four months.
- More than 8 in 10 (82 percent) children age 6-8 months (both breastfed and nonbreastfed) are introduced to complementary foods at an appropriate time.
- Overall, 30 percent of children age 6-23 months are fed appropriately based on recommended infant and young child feeding (IYCF) practices.
- Fourteen percent of women age 15-49 are underweight, that is, they fall below the body mass index (BMI) cutoff of 18.5. Eighteen percent of women are overweight or obese. The percentage of women who are overweight or obese has increased steadily over the last decade.
- Three-quarters (76 percent) of women age 15-49 with a birth in the last five years took iron tablets or syrup during the pregnancy of their last birth for more than 90 days, 72 percent took deworming medication during their most recent pregnancy, and 49 percent received iron supplementation postpartum.

Nutritional status is the result of complex interactions between food consumption and the overall status of health and care practices. Numerous socioeconomic and cultural factors influence decisions on patterns of feeding and nutritional status. Adequate nutrition is critical to child development. The period from birth to age 2 is important to optimal growth, health, and development. During this period, children who do not receive adequate nutrition can be susceptible to growth faltering, micronutrient deficiencies, and common childhood illnesses such as diarrhea and acute respiratory infections. Among women, malnutrition can result in reduced productivity, an increased susceptibility to infections, slow recovery from illness, and a heightened risk of adverse pregnancy outcomes. A woman who has poor nutritional status, as indicated by a low body mass index (BMI), short stature, anemia, or other micronutrient deficiency, has a greater risk of obstructed labor, of having a baby with a low birth weight, of producing lower quality breast milk, of mortality due to postpartum hemorrhage, and of morbidity for both herself and her baby.

The 2014 CDHS asked questions about early initiation of breastfeeding, exclusive breastfeeding during the first six months of life, continued breastfeeding until at least age 2, timely introduction of complementary foods at age 6 months (with increasing frequency of feeding solid and semisolid foods), and diet diversity. The height and weight of all children under age 5 and women age 15-49 were measured. This chapter presents findings on infant feeding practices, maternal eating patterns, household testing of salt for adequate levels of iodine, and the nutritional status of women and children.

16.1 NUTRITIONAL STATUS OF CHILDREN

Nutritional status of children under age 5 is an important measure of children's health. The anthropometric data on height and weight collected in the 2014 CDHS permit the evaluation of the nutritional status of young children in Cambodia.

16.1.1 Measurement of Nutritional Status among Young Children

In addition to questions about feeding practices of infants and young children, the 2014 CDHS included an anthropometric component in which children under age 5 in a subsample of two-thirds of the survey households were measured for height and weight. Weight measurements were taken using a lightweight electronic SECA scale designed and manufactured under the guidance of the United Nations Children's Fund (UNICEF). The scale allowed for the weighing of very young children through an automatic mother-child adjustment that eliminated the mother's weight while she was standing on the scale with her baby. Height measurements were carried out using a SECA measuring board also produced under the guidance of UNICEF. Children younger than age 24 months were measured lying down (recumbent length) on the board, whereas standing height was measured for older children. Based on these measurements, three internationally accepted indices were constructed and are used to reflect the nutritional status of children. These are:

- Height-for-age (stunting)
- Weight-for-height (wasting)
- Weight-for-age (underweight)

In the 2005 CDHS, children's anthropometric measurements were compared with an international reference population defined by the U.S. National Center for Health Statistics (NCHS) and accepted by the U.S. Centers for Disease Control and Prevention (CDC). However, in the 2010 and 2014 CDHS surveys, as recommended by the World Health Organization (WHO), the nutritional status of children in the survey population was compared with the 2006 WHO child growth standards (WHO, 2006), which are based on an international sample (from Brazil, Ghana, India, Norway, Oman, and the United States) of ethnically, culturally, and genetically diverse healthy children living under optimum conditions conducive to achieving a child's full genetic growth potential. The 1977 NCHS/CDC/WHO reference was replaced with the 2006 WHO child growth standards because of the prescriptive rather than descriptive nature of the WHO standards versus the NCHS reference. Also, the 2006 WHO child growth standards identify the breastfed child as the normative model for growth and development and document how children should grow under optimum conditions and infant feeding and child health practices.

The use of the 2006 WHO child growth standards is based on the finding that well-nourished children in all population groups for which data exist follow very similar growth patterns before puberty. The internationally based standard population serves as a point of comparison, facilitating examination of differences in the anthropometric status of subgroups in a population and of changes in nutritional status over time.

The height-for-age index is an indicator of linear growth retardation and cumulative growth deficits. Children whose height-for-age Z-score is below minus two standard deviations (-2 SD) from the mean of the reference population are considered short for their age (stunted) and are chronically malnourished. Children who are below minus three standard deviations (-3 SD) from the mean of the reference population are considered severely stunted. Stunting reflects failure to receive adequate nutrition over a long period of time and is also affected by recurrent and chronic illness. Height-for-age, therefore, represents the long-term effects of malnutrition in a population and does not vary according to recent dietary intake.

The weight-for-height index measures body mass in relation to body length and describes current nutritional status. Children whose Z-scores are below minus two standard deviations (-2 SD) from the mean of the reference population are considered thin (wasted) for their height and are acutely malnourished. Wasting represents the failure to receive adequate nutrition in the period immediately preceding the survey and may be the result of inadequate food intake or a recent episode of illness causing loss of weight and the onset of malnutrition. Children whose weight-for-height is below minus three standard deviations (-3 SD) from the mean of the reference population are considered severely wasted. Overweight and obesity are other forms of malnutrition that are becoming concerns for some children in developing countries. Children whose Z-score values are more than two standard deviations (+2 SD) above the median for weight-for-height are considered overweight.

Weight-for-age is a composite index of height-for-age and weight-for-height. It takes into account both acute and chronic malnutrition. Children whose weight-for-age is below minus two standard deviations (-2 SD) from the mean of the reference population are classified as underweight. Children whose weight-for-age is below minus three standard deviations (-3 SD) from the mean of the reference population are considered severely underweight.

A total of 5,120 children under age 5 were eligible to be measured for weight and height. Of these children, 96 percent had complete data on their age and on their weight and height measurements. The following analysis focuses on the 4,893 children for whom complete and valid anthropometric data were collected.

16.1.2 Measures of Child Nutritional Status

Overall, 32 percent of Cambodian children under age 5 are stunted, and 9 percent are severely stunted (Table 16.1 and Figure 16.1). Analysis by age group indicates that stunting is apparent even among children less than age 6 months (16 percent). In general, stunting increases with the age of the child, rising from 13 percent among children age 6-8 months to 40 percent among children age 36-47 months before declining to 36 percent among children age 48-59 months. There is very little difference in the level of stunting by gender. Stunting is highest when the birth interval is less than 24 months (37 percent). Size at birth is an important indicator of children's nutritional status. Nearly 2 in 3 children (63 percent) reported to have been very small at birth are stunted. Children whose mothers are underweight are more likely to be stunted (44 percent) than children of normal weight mothers (32 percent). The disparity in stunting prevalence between rural and urban children is substantial: 34 percent of rural children are stunted, as compared with 24 percent of urban children. Variation in the nutritional status of children by province is quite evident, with stunting being highest in Preah Vihear/Stung Treng (44 percent) and Kampong Chhnang (43 percent) and lowest in Phnom Penh (18 percent). Mother's education and wealth quintile have an inverse relationship with stunting levels. For example, the prevalence of stunting is higher among children living in the poorest households (42 percent) than among children in the richest households (19 percent).

Ten percent of children under age 5 are wasted, and 2 percent are severely wasted. There is a substantial correlation between wasting and size at birth. Babies who are very small and small at birth are more likely to be wasted (24 percent and 17 percent, respectively) than those of average or larger size at birth (9 percent). The prevalence of wasting among children of thin mothers (BMI below 18.5) is more than twice that of children whose mothers are either normal weight or overweight/obese. Wasting is higher among rural children than urban children (10 percent versus 8 percent) and is highest in Takeo and Otdar Meanchey (15 percent each). Wasting prevalence varies inconsistently by age of the child and does not differ substantially by sex. It is highest among children whose mothers have no education and those in the lowest two wealth quintiles.

Overweight and obesity affect a very small proportion of children in Cambodia. Overall, 2 percent of children below age 5 are overweight (weight-for-height more than +2 SD). Overweight among children tends to decrease with increasing age. There are no substantial differences by other characteristics.

Table 16.1 Nutritional status of children

Percentage of children under age 5 classified as malnourished according to three anthropometric indices of nutritional status: height-for-age, weight-for-height, and weight-for-age, by background characteristics, Cambodia 2014

	Height-for-age[1]			Weight-for-height				Weight-for-age				
Background characteristic	Percentage below -3 SD	Percentage below -2 SD[2]	Mean Z-score (SD)	Percentage below -3 SD	Percentage below -2 SD[2]	Percentage above +2 SD	Mean Z-score (SD)	Percentage below -3 SD	Percentage below -2 SD[2]	Percentage above +2 SD	Mean Z-score (SD)	Number of children
**Age in months**												
<6	6.1	16.1	(0.4)	5.4	12.8	6.0	(0.3)	3.2	11.5	1.3	(0.6)	426
6-8	1.2	13.1	(0.6)	2.3	6.5	4.8	(0.3)	1.9	8.5	2.2	(0.6)	252
9-11	3.9	16.6	(0.9)	2.3	14.2	3.1	(0.7)	3.0	15.4	0.5	(1.0)	225
12-17	6.4	28.1	(1.3)	3.1	10.6	3.5	(0.7)	2.6	21.2	1.0	(1.1)	515
18-23	11.3	33.8	(1.5)	2.8	10.9	0.7	(0.7)	5.4	19.6	0.3	(1.2)	545
24-35	10.8	38.5	(1.6)	1.8	8.0	1.8	(0.6)	4.3	24.9	0.9	(1.3)	1,013
36-47	10.9	39.8	(1.8)	1.9	9.3	0.7	(0.8)	7.1	31.4	0.1	(1.5)	978
48-59	9.4	36.0	(1.7)	1.2	8.7	0.5	(0.8)	5.1	30.9	0.2	(1.6)	939
**Sex**												
Male	9.4	32.9	(1.4)	2.7	9.9	2.3	(0.6)	4.5	23.2	0.8	(1.2)	2,497
Female	8.4	31.9	(1.4)	1.9	9.3	1.6	(0.7)	4.8	24.6	0.5	(1.3)	2,395
**Birth interval in months[3]**												
First birth[4]	8.3	30.2	(1.4)	2.3	9.6	2.1	(0.7)	3.3	24.3	0.7	(1.3)	1,683
<24	11.7	37.4	(1.5)	2.3	10.5	2.3	(0.8)	9.3	31.1	0.9	(1.4)	336
24-47	9.5	34.1	(1.5)	2.4	9.9	2.0	(0.7)	6.3	25.0	0.6	(1.3)	1,102
48+	7.8	31.0	(1.3)	2.7	9.5	1.8	(0.7)	3.6	21.0	0.5	(1.2)	1,139
**Size at birth[3]**												
Very small	25.6	63.1	(2.3)	3.6	23.6	4.8	(1.0)	16.5	58.9	2.6	(2.0)	96
Small	13.9	40.5	(1.7)	2.3	16.7	1.0	(0.9)	7.1	38.5	0.0	(1.6)	368
Average or larger	7.8	30.2	(1.4)	2.4	8.6	2.0	(0.7)	4.1	21.8	0.7	(1.2)	3,772
**Mother's interview status**												
Interviewed	8.7	32.0	(1.4)	2.4	9.7	2.0	(0.7)	4.6	24.1	0.6	(1.3)	4,261
Not interviewed but in household	2.5	36.0	(1.2)	1.4	11.4	5.1	(0.5)	2.7	23.1	2.1	(1.0)	107
Not interviewed and not in the household[5]	11.9	35.2	(1.5)	1.6	8.4	0.8	(0.5)	5.3	22.3	0.2	(1.2)	525
**Mother's nutritional status[6]**												
Thin (BMI <18.5)	13.7	44.0	(1.8)	2.5	18.5	0.7	(1.0)	11.0	39.7	0.4	(1.7)	461
Normal (BMI 18.5-24.9)	8.5	31.8	(1.4)	2.3	8.6	2.4	(0.6)	4.0	23.9	0.7	(1.3)	2,726
Overweight/obese (BMI ≥25)	6.2	26.2	(1.3)	2.0	6.7	1.7	(0.5)	2.5	15.7	0.9	(1.1)	671
**Residence**												
Urban	5.9	23.7	(1.1)	2.0	7.5	3.1	(0.4)	2.6	14.8	1.9	(0.9)	674
Rural	9.4	33.8	(1.5)	2.4	9.9	1.8	(0.7)	5.0	25.4	0.4	(1.3)	4,219
**Province**												
Banteay Meanchey	6.9	28.6	(1.3)	0.7	7.8	0.7	(0.5)	5.3	17.0	0.0	(1.1)	241
Kampong Cham	8.6	33.5	(1.4)	1.5	8.1	2.2	(0.7)	4.2	25.7	0.8	(1.3)	692
Kampong Chhnang	13.5	42.8	(1.7)	3.1	11.2	2.2	(0.9)	5.1	35.6	0.0	(1.5)	173
Kampong Speu	10.0	40.5	(1.7)	2.5	11.5	1.3	(0.8)	6.9	29.4	0.0	(1.5)	318
Kampong Thom	10.7	36.4	(1.3)	3.1	13.0	3.4	(0.8)	7.1	27.7	1.9	(1.3)	217
Kandal	3.5	28.1	(1.3)	3.2	9.2	0.2	(0.8)	4.7	26.2	0.0	(1.3)	298
Kratie	10.5	38.4	(1.6)	2.7	6.5	0.5	(0.7)	4.4	25.1	0.0	(1.4)	180
Phnom Penh	4.9	17.9	(0.9)	1.0	8.4	3.7	(0.4)	2.2	12.9	3.4	(0.8)	391
Prey Veng	8.7	32.7	(1.5)	2.9	8.6	1.8	(0.6)	3.4	22.2	0.3	(1.3)	379
Pursat	18.4	38.8	(1.8)	5.7	12.3	4.7	(0.6)	7.9	31.6	0.4	(1.4)	200
Siem Reap	11.3	35.9	(1.5)	2.3	9.5	1.0	(0.7)	6.9	26.2	0.4	(1.3)	323
Svay Rieng	8.2	32.8	(1.4)	2.7	7.6	3.6	(0.6)	3.6	20.8	0.5	(1.2)	190
Takeo	6.4	30.7	(1.3)	5.0	14.6	1.5	(0.8)	4.4	22.7	0.0	(1.3)	258
Otdar Meanchey	14.0	36.3	(1.3)	7.2	15.1	5.3	(0.7)	5.2	26.4	0.0	(1.3)	78
Battambang/Pailin	5.0	24.9	(1.2)	0.3	7.9	0.7	(0.6)	1.8	18.2	0.5	(1.1)	388
Kampot/Kep	8.3	25.2	(1.4)	0.9	8.2	1.9	(0.7)	3.5	21.1	0.7	(1.3)	195
Preah Sihanouk/ Koh Kong	10.4	33.4	(1.4)	3.1	10.5	1.8	(0.6)	6.5	22.0	0.0	(1.2)	105
Preah Vihear/ Stung Treng	14.0	44.3	(1.8)	1.3	13.8	2.3	(0.7)	5.9	30.7	0.1	(1.5)	142
Mondul Kiri/ Ratanak Kiri	14.6	39.8	(1.6)	1.4	8.2	1.2	(0.6)	6.0	26.2	0.3	(1.4)	125
**Mother's education[7]**												
No education	13.3	38.5	(1.6)	2.8	12.2	1.5	(0.7)	7.4	29.7	0.4	(1.4)	577
Primary	8.7	34.1	(1.5)	2.1	9.3	2.1	(0.7)	4.7	24.6	0.6	(1.3)	2,397
Secondary and higher	6.8	26.8	(1.3)	2.7	9.9	2.0	(0.7)	3.5	22.3	0.6	(1.2)	1,278
**Wealth quintile**												
Lowest	14.1	41.9	(1.7)	2.8	11.0	1.1	(0.8)	7.1	31.0	0.0	(1.5)	1,182
Second	9.8	37.1	(1.6)	2.3	11.4	2.1	(0.7)	5.7	27.5	0.5	(1.4)	998
Middle	7.8	31.7	(1.4)	2.1	8.4	1.3	(0.7)	4.3	23.3	0.2	(1.3)	978
Fourth	6.9	29.1	(1.3)	2.5	9.3	2.4	(0.7)	3.5	22.0	0.8	(1.2)	844
Highest	4.1	18.5	(0.9)	1.9	7.4	3.3	(0.4)	1.7	13.0	2.1	(0.8)	891
Total	8.9	32.4	(1.4)	2.3	9.6	2.0	(0.7)	4.7	23.9	0.6	(1.3)	4,893

Note: Table is based on children who stayed in the household on the night before the interview. Each of the indices is expressed in standard deviation units (SD) from the median of the WHO child growth standards adopted in 2006. The indices in this table are NOT comparable to those based on the previously used NCHS/CDC/WHO reference. Table is based on children with valid dates of birth (month and year) and valid measurement of both height and weight. Total includes 25 cases with missing information on size at birth.

[1] Recumbent length is measured for children under age 2 and in the few cases when the age of the child is unknown and the child is less than 85 cm; standing height is measured for all other children.

[2] Includes children who are below -3 standard deviations (SD) from the WHO child growth standards population median

[3] Excludes children whose mothers were not interviewed

[4] First-born twins (triplets, etc.) are counted as first births because they do not have a previous birth interval.

[5] Includes children whose mothers are deceased

[6] Excludes children whose mothers were not weighed and measured, children whose mothers were not interviewed, and children whose mothers are pregnant or gave birth within the preceding 2 months. Mother's nutritional status in terms of BMI (body mass index) is presented in Table 16.10.

[7] For women who are not interviewed, information is taken from the Household Questionnaire. Excludes children whose mothers are not listed in the Household Questionnaire.

Twenty-four percent of children under age 5 are underweight (low weight-for-age), and 5 percent are severely underweight. Figure 16.1 shows that the percentage of children underweight increases steadily from 5 percent among children younger than age 2 months to more than 10 percent among children age 2-4 months, followed by a small decline among children age 6-8 months. The percentage then increases with increasing age and peaks among children age 40-42 months. This may be due to inappropriate and/or inadequate feeding practices because the percentage of underweight children begins to increase at the age when normal complementary feeding starts. The prevalence of underweight is 10 percentage points higher among rural children (25 percent) than among urban children (15 percent) (Table 16.1). More than half of the provinces in Cambodia (11 of 19) have percentages of underweight children above the national average. A mother's wealth status and educational level are negatively correlated with the likelihood that her child is underweight. Children born to mothers in the lowest wealth quintile are more than twice as likely (31 percent) to be underweight as children born to mothers in the highest wealth quintile (13 percent).

Figure 16.1 **Nutritional status of children by age**

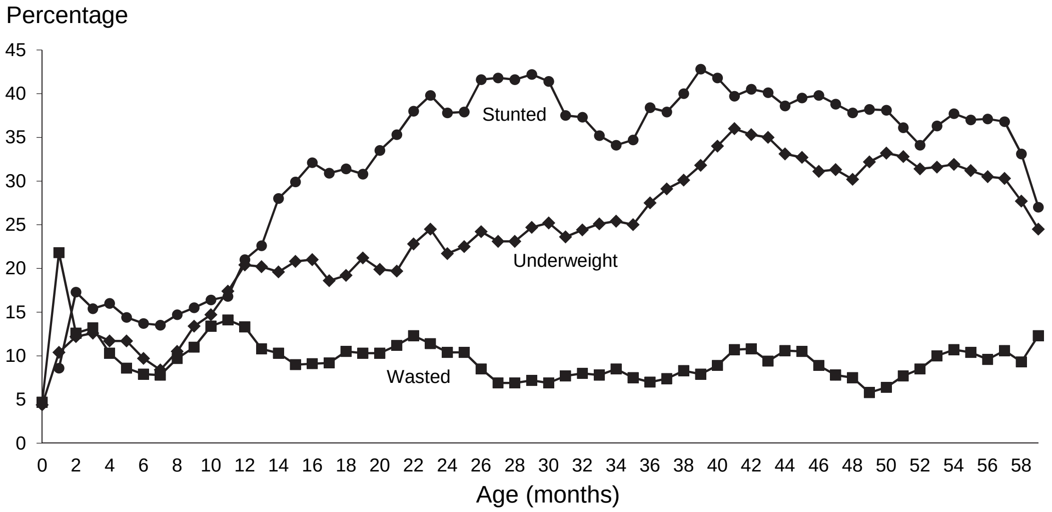

Note: *Stunting* reflects chronic malnutrition; *wasting* reflects acute malnutrition; *underweight* reflects chronic or acute malnutrition or a combination of both. Plotted values are smoothed by a five-month moving average.

CDHS 2014

16.1.3 Trends in Children's Nutritional Status

Trends in children's nutritional status for the period 2000 to 2014 are shown in Figure 16.2. To allow assessment of trends, the data for 2000 and 2005 were recalculated using the 2006 WHO child growth standards. Figure 16.2 shows that there have been improvements in the nutritional status of children in the past 14 years. The percentage of children stunted fell consistently from 50 percent in 2000 to 32 percent in 2014. The percentage of children wasted declined from 17 percent in 2000 to 8 percent in 2005 before increasing to 11 percent in 2010 and subsequently dropping slightly to 10 percent in 2014. Underweight declined from 39 percent in 2000 to 28 percent in 2005 and 2010 and then decreased to 24 percent in 2014.

Although there have been improvements in the nutritional status of Cambodian children in the past decade and a half, there is still a need for more intensive interventions.

Figure 16.2 **Trends in nutritional status of children under age 5**

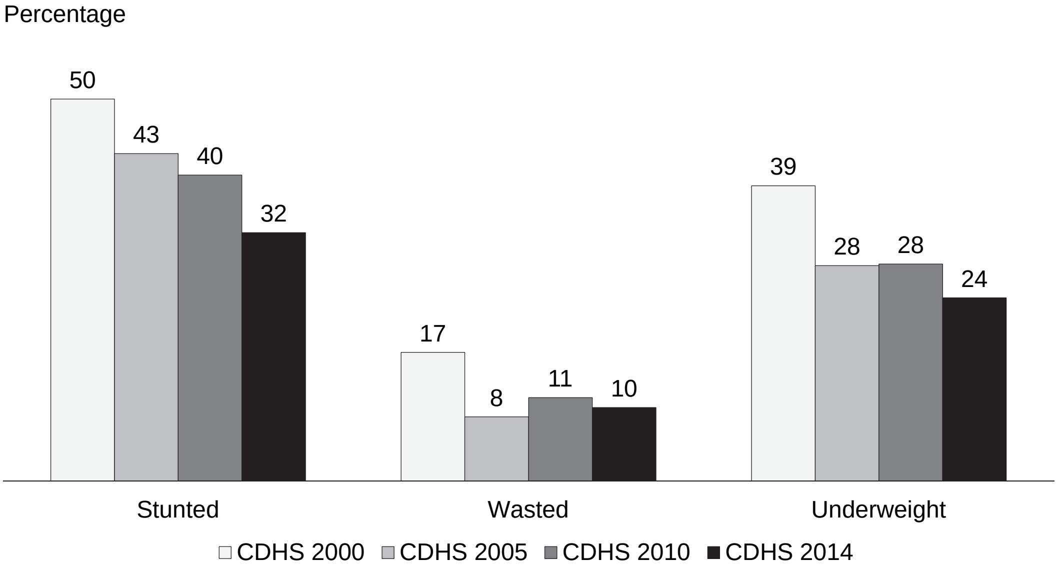

16.2 Initiation of Breastfeeding

Early initiation of breastfeeding is encouraged for a number of reasons. Mothers benefit from early suckling because it stimulates breast milk production and facilitates the release of oxytocin, which helps the uterus contract and reduces postpartum blood loss. The first breast milk contains colostrum, which is highly nutritious and has antibodies that protect the newborn from diseases. Early initiation of breastfeeding also fosters bonding between mother and child.

Table 16.2 shows the percentage of all children born in the two years before the survey by breastfeeding status and the timing of initial breastfeeding, according to background characteristics. In the 2010 CDHS, initial breastfeeding data were collected for children of the same age (0-2 years) as in the 2014 survey; however, in the 2000 and 2005 CDHS surveys, initial breastfeeding data were collected for all children less than age 5, and thus caution should be exercised in comparing the results of the 2010 and 2014 surveys with previous survey results.

Ninety-six percent of children born in the two years preceding the survey were breastfed at some point of time. Young children living in rural areas at the time of the survey are more likely to have ever been breastfed than children living in urban areas. The proportion of children ever breastfed ranges from a low of 91 percent in Phnom Penh to a high of over 99 percent in Mondul Kiri/Ratanak Kiri and Prey Veng. Children in the lowest wealth quintile are more likely to have ever been breastfed (98 percent) than children in the highest wealth quintile (92 percent).

Sixty-three percent of children are breastfed within one hour of birth, and 87 percent are breastfed within one day of birth.

Several background characteristics have important influences on early breastfeeding practices. For example, early initiation of breastfeeding is more common among children whose mothers delivered in a heath facility and whose birth was assisted by a health professional than among children delivered at home or by a traditional birth attendant. In addition, the proportion of children breastfed within one hour of birth is highest in Kampong Thom (85 percent) and lowest in Mondul Kiri/Ratanak Kiri (16 percent). There is no consistent association between early breastfeeding and mother’s education and wealth.

Table 16.2 Initial breastfeeding

Among last-born children who were born in the two years preceding the survey, the percentage who were ever breastfed and the percentages who started breastfeeding within one hour and within one day of birth, and among last-born children born in the two years preceding the survey who were ever breastfed, the percentage who received a prelacteal feed, by background characteristics, Cambodia 2014

	Among last-born children born in the past two years:				Among last-born children born in the past two years who were ever breastfed:	
Background characteristic	Percentage ever breastfed	Percentage who started breastfeeding within 1 hour of birth	Percentage who started breastfeeding within 1 day of birth[1]	Number of last-born children	Percentage who received a prelacteal feed[2]	Number of last-born children ever breastfed
**Sex**						
Male	96.8	60.9	86.7	1,471	27.0	1,425
Female	95.6	64.2	87.8	1,473	28.3	1,409
**Assistance at delivery**						
Health professional[3]	96.3	63.9	88.1	2,730	27.6	2,628
Traditional birth attendant	98.0	45.1	78.4	207	29.1	202
**Place of delivery**						
Health facility	96.3	64.3	88.1	2,614	27.5	2,516
At home	96.8	49.1	81.0	325	28.6	314
**Residence**						
Urban	91.7	50.6	79.1	414	50.2	380
Rural	97.0	64.5	88.6	2,531	24.2	2,454
**Province**						
Banteay Meanchey	94.2	67.7	78.4	120	28.7	113
Kampong Cham	96.9	72.6	82.9	418	38.4	404
Kampong Chhnang	97.7	47.5	95.6	111	10.6	108
Kampong Speu	97.3	56.2	95.8	182	26.0	177
Kampong Thom	95.9	84.7	95.1	141	8.7	135
Kandal	95.5	69.5	86.1	193	39.8	184
Kratie	98.4	64.0	82.7	107	27.8	106
Phnom Penh	91.0	37.2	75.8	257	61.0	234
Prey Veng	99.4	65.8	94.1	194	22.0	193
Pursat	96.8	72.5	93.9	122	7.6	118
Siem Reap	97.2	65.6	86.2	182	23.7	177
Svay Rieng	93.3	68.8	82.5	108	25.8	101
Takeo	93.8	58.1	92.0	164	16.3	154
Otdar Meanchey	98.8	49.2	97.3	54	5.6	54
Battambang/Pailin	96.9	72.4	87.8	247	29.9	239
Kampot/Kep	96.1	37.9	85.3	116	24.6	112
Preah Sihanouk/ Koh Kong	96.9	76.6	89.8	61	24.7	60
Preah Vihear/ Stung Treng	98.9	77.3	97.7	92	8.4	91
Mondul Kiri/ Ratanak Kiri	99.7	15.9	76.2	75	17.3	75
**Mother's education**						
No education	96.7	58.4	86.1	366	21.6	354
Primary	96.3	66.6	88.3	1,491	23.2	1,436
Secondary and higher	96.0	58.4	86.2	1,088	35.9	1,044
**Wealth quintile**						
Lowest	97.6	62.8	90.6	694	14.5	677
Second	96.1	62.2	86.0	589	20.7	566
Middle	97.7	71.0	89.9	565	25.7	552
Fourth	97.4	64.1	89.1	536	31.9	522
Highest	92.2	52.8	80.1	560	50.4	516
Total	96.2	62.6	87.3	2,944	27.7	2,834

Note: Table is based on last-born children born in the two years preceding the survey regardless of whether the children are living or dead at the time of the interview. Total includes cases for which information on place of delivery and assistance at delivery is missing.
[1] Includes children who started breastfeeding within one hour of birth
[2] Children given something other than breast milk during the first three days of life
[3] Doctor, nurse, or midwife

Twenty-eight percent of children receive a prelacteal feed, that is, something other than breast milk during the first three days of life. The proportions of children who receive a prelacteal feed in the first three days of life do not differ significantly by sex of the child, assistance at delivery, or place of delivery. Children residing in urban areas are twice as likely as children residing in rural areas to receive a prelacteal feed. More than 3 in 5 children living in Phnom Penh (61 percent) receive a prelacteal feed after birth. Prelacteal feeding increases as the level of mother's education and wealth increase. The percentage of children who receive a prelacteal feed is lower among those whose mothers have no schooling (22 percent)

than among those whose mothers have a primary education (23 percent) or a secondary education or higher (36 percent). Fifteen percent of children in the lowest quintile receive a prelacteal feed, as compared with 50 percent of children in the highest wealth quintile.

16.3 Breastfeeding Status by Age

UNICEF and WHO recommend that children be exclusively breastfed during the first six months of life and that children be given solid or semisolid complementary food in addition to continued breastfeeding from six months to 24 months. Exclusive breastfeeding is recommended because breast milk is uncontaminated and contains all of the nutrients necessary for children in the first few months of life. In addition, the mother's antibodies in breast milk provide immunity to disease. Early supplementation is discouraged for several reasons. First, it exposes infants to pathogens and increases their risk of infection, especially disease. Second, it decreases infants' intake of breast milk and therefore suckling, which reduces breast milk production. Third, in a harsh socioeconomic environment, supplementary food is often nutritionally inferior.

Information on complementary feeding was obtained by asking mothers about the current breastfeeding status of all children under age 2 and food (liquids or solids) given to the child the day and night before the survey.

Table 16.3 Breastfeeding status by age

Percent distribution of youngest children under age 2 who are living with their mother by breastfeeding status and the percentage currently breastfeeding, and the percentage of all children under age 2 using a bottle with a nipple, according to age in months, Cambodia 2014

		Breastfeeding status									
Age in months	Not breast-feeding	Exclusively breastfed	Breast-feeding and consuming plain water only	Breast-feeding and consuming non-milk liquids[1]	Breast-feeding and consuming other milk	Breast-feeding and consuming comple-mentary foods	Total	Percentage currently breast-feeding	Number of youngest children under age 2 living with their mother	Percentage using a bottle with a nipple	Number of all children under age 2
0-1	3.6	79.9	5.6	0.4	7.6	2.9	100.0	96.4	210	8.6	213
2-3	6.6	67.1	14.7	2.1	6.2	3.3	100.0	93.4	257	16.2	262
4-5	9.4	50.9	12.5	0.3	7.9	18.9	100.0	90.6	249	32.5	260
6-8	7.2	9.0	5.8	0.4	1.7	75.8	100.0	92.8	396	38.4	403
9-11	11.4	0.4	1.3	0.8	1.9	84.3	100.0	88.6	349	38.3	357
12-17	22.2	0.3	0.6	0.2	0.4	76.2	100.0	77.8	673	34.1	689
18-23	60.3	0.0	0.0	0.0	0.2	39.4	100.0	39.7	724	33.0	771
0-3	5.2	72.9	10.6	1.3	6.9	3.1	100.0	94.8	467	12.8	475
0-5	6.7	65.2	11.3	1.0	7.2	8.6	100.0	93.3	717	19.8	736
6-9	8.8	7.1	4.5	0.3	1.3	78.0	100.0	91.2	514	38.8	523
12-15	20.0	0.4	0.6	0.4	0.6	78.0	100.0	80.0	461	34.9	467
12-23	42.0	0.1	0.3	0.1	0.3	57.2	100.0	58.0	1,398	33.5	1,460
20-23	62.9	0.0	0.0	0.0	0.1	37.0	100.0	37.1	484	32.3	519

Note: Breastfeeding status refers to a "24-hour" period (yesterday and last night). Children who are classified as breastfeeding and consuming plain water only consumed no liquid or solid supplements. The categories of not breastfeeding, exclusively breastfed, and breastfeeding and consuming plain water, non-milk liquids, other milk, and complementary foods (solids and semisolids) are hierarchical and mutually exclusive, and their percentages sum to 100 percent. Thus, children who receive breast milk and non-milk liquids and who do not receive other milk and who do not receive complementary foods are classified in the non-milk liquid category even though they may also get plain water. Any children who get complementary food are classified in that category as long as they are breastfeeding as well.

[1] Non-milk liquids include juice, juice drinks, clear broth, or other liquids.

Table 16.3 shows the percent distribution of youngest children under age 2 living with their mother by breastfeeding status and the percentage of all children under age 2 using a bottle with a nipple, according to age in months. The data presented in Table 16.3 and Figure 16.3 show that, contrary to WHO's recommendations, not all children under age 6 months are exclusively breastfed. Seventy-three percent of Cambodian children age 0-3 months are exclusively breastfed, and only 65 percent of children age 0-5 months are exclusively breastfed.

Among children less than age 6 months, 11 percent consume breast milk and plain water and 7 percent consume other milk in addition to breast milk. Although 76 percent of children begin eating

complementary foods at age 6-8 months, 9 percent of children continue to be exclusively breastfed and 6 percent receive just plain water in addition to breast milk. Only 37 percent of Cambodian children continue to breastfeed until age 2 (Table 16.3), and thus 63 percent are deprived of valuable nutrients during this period. Exclusive breastfeeding quickly declines from birth to age 6-8 months. Although other liquids are not needed before six months, 12 percent of infants under age 6 months receive water or other liquids with milk.

The prevalence of bottle feeding among Cambodian children age 6 months and above has increased substantially in comparison with data from the 2010 CDHS. Thirty-four percent of children age 12-23 months were fed with a bottle in 2014, as compared with 25 percent in 2010. In Cambodia the bottle is used for feeding breast milk substitutes (which are most often formula or sweetened condensed milk or other canned milk usually thinned out with water) or very watery rice porridge (borbor), both of which are contraindicated.

Figure 16.3 **Infant feeding practices by age**

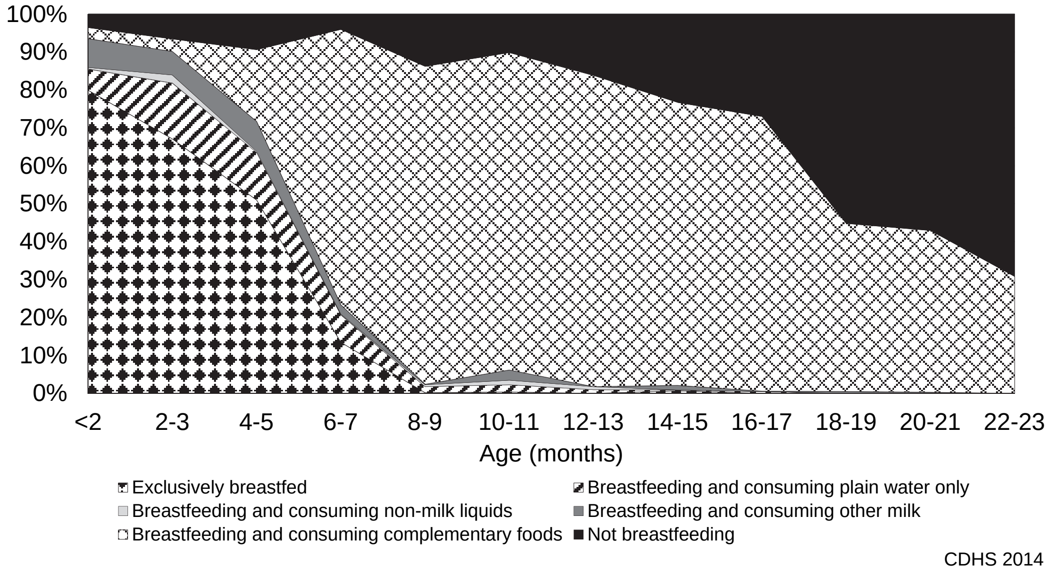

16.4 DURATION OF BREASTFEEDING

Table 16.4 shows the median duration of breastfeeding by selected background characteristics. The estimates of median and mean durations of breastfeeding are based on current status data, that is, the proportion of last-born children in the three years preceding the survey who were being breastfed at the time of the survey.

The median duration of any breastfeeding is 18.4 months, and the mean duration is 19.0 months. There is little difference in duration of breastfeeding by sex of the child. Urban children are breastfed for a much shorter duration than rural children (13.5 months versus 18.8 months). Highly educated mothers breastfeed their children for about one month less than mothers with little or no education. Mothers from the highest wealth quintile breastfeed their children for only 14 months, as compared with 19 months among mothers in all other wealth quintiles.

The median duration of exclusive breastfeeding among Cambodian children is 3.7 months, and the mean duration is 4.5 months. In comparison with the 2010 CDHS, the median durations of any breastfeeding and exclusive breastfeeding have decreased by about one month.

Table 16.4 Median duration of breastfeeding

Median duration of any breastfeeding, exclusive breastfeeding, and predominant breastfeeding among children born in the three years preceding the survey, by background characteristics, Cambodia 2014

Background characteristic	Median duration (months) of breastfeeding among children born in the past three years[1]		
	Any breastfeeding	Exclusive breastfeeding	Predominant breastfeeding[2]
**Sex**			
Male	18.6	3.5	4.8
Female	18.1	3.9	4.8
**Residence**			
Urban	13.5	a	1.6
Rural	18.8	4.0	5.0
**Mother's education**			
No education	19.3	3.7	5.0
Primary	18.4	3.8	4.8
Secondary and higher	18.0	3.8	4.8
**Wealth quintile**			
Lowest	19.3	4.3	5.0
Second	18.9	4.4	5.2
Middle	18.5	3.7	4.9
Fourth	18.9	4.0	5.2
Highest	14.1	(1.2)	2.8
Total	18.4	3.7	4.8
Mean for all children	19.0	4.5	5.4

Note: Median and mean durations are based on the distributions at the time of the survey of the proportion of births by months since birth. Includes children living and deceased at the time of the survey. Figures in parentheses are based on 25-49 unweighted cases in the duration category in which the median value fell.
a = Omitted because less than 50 percent of children were breastfed before reaching the reference period
[1] It is assumed that non-last-born children and last-born children not currently living with their mother are not currently breastfeeding.
[2] Either exclusively breastfed or received breast milk and plain water and/or non-milk liquids only

Figure 16.4 presents a number of indicators summarizing the extent to which Cambodian children are being fed according to recommended infant and young child feeding (IYCF) practices. The exclusive breastfeeding indicators included in the figure highlight the fact that the majority of children are not exclusively breastfed for the recommended six months. Overall, only 65 percent of all children under age 6 months are being exclusively breastfed, and at age 4-5 months only half of children (51 percent) are receiving only breast milk.

Figure 16.4 **IYCF indicators on breastfeeding status**

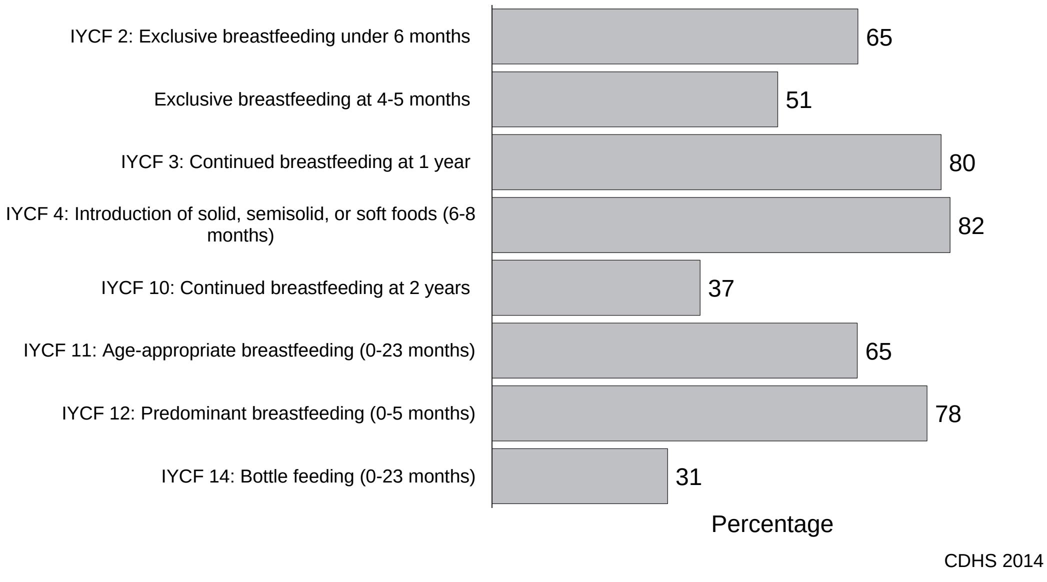

Figure 16.4 also provides information on the prevalence of predominant breastfeeding. Three quarters of children (78 percent) under age 6 months are in this category, that is, they are exclusively breastfed or they are breastfed and receive either plain water or non-milk liquids. In addition, Figure 16.4 includes data on the timely introduction of complementary feeding; as recommended, 82 percent of children age 6-8 months are being given solid, semisolid, or soft food. The continued breastfeeding indicators in Figure 16.4 show that breastfeeding continues well into the first year of life for most children. However, by age 2, the majority of children are weaned. Although bottle feeding is discouraged, 31 percent of Cambodian children age 0-23 months are bottle fed.

Finally, the age-appropriate breastfeeding indicator in Figure 16.4 provides an overall measure of the extent to which recommendations with respect to exclusive breastfeeding and timely introduction of complementary foods are being observed. Children are classified as receiving age-appropriate breastfeeding if they are age 0-5 months and exclusively breastfed or age 6-23 months and breastfeeding and consuming complementary foods. Around two-thirds of Cambodian children (65 percent) are being breastfed appropriately.

16.5 TYPES OF COMPLEMENTARY FOODS

UNICEF and WHO recommend the introduction of solid food to infants at approximately age 6 months because by that age breast milk alone is no longer sufficient to maintain a child’s optimal growth. In the transition to eating the family diet, children from age 6 months should be fed small quantities of solid and semisolid foods throughout the day. During this transition period (age 6-23 months), the prevalence of malnutrition increases substantially in many countries because of increased infections and poor feeding practices.

Table 16.5 provides information from mothers on the types of food given to their youngest child under age 2 living with the mother on the day and night preceding the survey, according to breastfeeding status. The data show that 7 percent and 13 percent of breastfeeding infants receive infant formula and any other kinds of milk, respectively. However, 16 percent of younger breastfeeding infants (age 4-5 months) are already consuming food made from grains; 3 percent consume food made from meat, fish, or poultry; and 2 percent consume eggs.

Overall, two-thirds of breastfed children (68 percent) under age 2 received solid or semisolid complementary foods in addition to breast milk. Consumption of foods made from grains (63 percent), animal sources of food (meat, fish, and poultry) (50 percent), and fruits and vegetables rich in vitamin A (38 percent) is high. Consumption of food made from roots or tubers is low (8 percent).

Comparing dietary intake of children by their breastfeeding status, a higher proportion of solid and semisolid foods are being consumed by nonbreastfed children. Thirty-one percent of nonbreastfeeding children receive infant formula and 32 percent receive other types of milk, both of which are essential because these children are not benefiting from breast milk. A larger percentage of nonbreastfed children under age 2 than breastfed children in the same age group are receiving grains, fruits and vegetables rich in vitamin A, and meat, fish, poultry, and eggs.

Table 16.5 Foods and liquids consumed by children in the day or night preceding the interview

Percentage of youngest children under age 2 who are living with their mother by type of foods consumed in the day or night preceding the interview, according to breastfeeding status and age, Cambodia 2014

	Liquids			Solid or semisolid foods										
Age in months	Infant formula	Other milk[1]	Other liquids[2]	Fortified baby foods	Food made from grains[3]	Fruits and vege-tables rich in vitamin A[4]	Other fruits and vege-tables	Food made from roots and tubers	Food made from legumes and nuts	Meat, fish, poultry	Eggs	Cheese, yogurt, other milk products	Any solid or semi-solid food	Number of children
BREASTFEEDING CHILDREN														
0-1	8.1	2.8	2.3	0.7	1.4	1.3	1.3	1.3	1.3	1.3	0.7	2.3	3.0	203
2-3	4.0	3.0	3.1	0.7	3.5	0.9	0.7	0.7	0.7	0.9	0.7	0.0	3.5	240
4-5	10.0	3.9	4.9	2.1	16.0	3.0	0.7	1.3	0.0	3.4	2.2	0.4	20.9	226
6-8	10.0	8.5	20.8	3.4	74.3	28.4	8.6	5.7	3.5	37.8	19.5	1.1	81.7	367
9-11	5.5	10.8	30.7	2.6	89.2	51.7	17.9	9.8	4.5	71.0	34.0	1.4	95.1	309
12-17	6.4	21.2	41.0	4.6	94.9	66.6	30.0	14.3	8.1	83.6	42.4	3.3	98.0	524
18-23	5.1	27.1	51.0	3.6	93.6	70.5	42.4	15.0	10.2	90.5	47.2	3.9	99.3	287
6-23	6.8	17.1	35.8	3.7	88.4	54.8	24.6	11.4	6.6	71.0	35.9	2.5	93.6	1,488
Total	7.0	12.8	25.8	2.9	63.2	38.4	17.3	8.2	4.8	49.6	25.2	2.0	67.5	2,156
NONBREASTFEEDING CHILDREN														
0-1	*	*	*	*	*	*	*	*	*	*	*	*	*	8
2-3	*	*	*	*	*	*	*	*	*	*	*	*	*	17
4-5	(85.2)	(12.2)	(14.7)	(6.5)	(16.9)	(12.5)	(0.0)	(0.0)	(7.6)	(11.9)	(4.3)	(0.0)	(27.6)	24
6-8	(80.1)	(8.0)	(28.2)	(9.6)	(66.3)	(44.6)	(26.2)	(8.0)	(0.0)	(62.7)	(41.1)	(7.0)	(79.9)	29
9-11	(65.4)	(29.3)	(30.7)	(11.4)	(85.0)	(61.0)	(45.2)	(16.0)	(2.7)	(74.2)	(49.2)	(0.0)	(91.5)	40
12-17	48.4	32.8	44.9	5.5	92.9	62.2	37.9	13.5	11.9	90.4	42.8	6.3	97.8	150
18-23	14.9	35.2	51.8	7.2	95.6	73.5	46.3	18.0	13.7	95.2	47.6	6.4	99.2	437
6-23	28.5	33.1	47.9	7.2	93.1	68.9	43.4	16.4	12.0	91.4	46.3	6.0	97.5	655
Total	31.3	31.5	45.2	6.9	88.1	65.3	40.8	15.5	11.8	86.3	43.9	5.8	92.6	703

Note: Breastfeeding status and food consumed refer to a "24-hour" period (yesterday and last night). Figures in parentheses are based on 25-49 unweighted cases. An asterisk indicates that a figure is based on fewer than 25 unweighted cases and has been suppressed.
[1] Other milk includes fresh, tinned, and powdered cow or other animal milk.
[2] Does not include plain water
[3] Includes fortified baby food
[4] Includes pumpkin, carrots, squash or sweet potatoes that are yellow or orange inside, dark green leafy vegetables, mangoes, and papayas.

16.6 INFANT AND YOUNG CHILD FEEDING (IYCF) PRACTICES

Appropriate IYCF practices include timely initiation of feeding solid and semisolid foods from age 6 months and increasing the amount and variety of foods and frequency of feeding as the child gets older while maintaining frequent breastfeeding (WHO, 2008).

The age ranges of various indicators of IYCF practices presented in this chapter have been updated based on the most recent definitions of breastfeeding and complementary feeding indicators (WHO, 2010). Therefore, to compare results with the previous CDHS surveys, one needs to first check that indicator definitions and age ranges of sampled children are the same across surveys.

Table 16.6 presents a summary indicator of IYCF practices. The indicator takes into account the percentages of children for whom feeding practices meet minimum standards with respect to food diversity (i.e., the number of food groups consumed), feeding frequency (i.e., the number of times the child is fed), and consumption of breast milk or other types of milk or milk products (accounting for number of milk feedings for nonbreastfed children). Breastfed children are considered to be fed within the minimum standards if they consume at least four food groups and receive food other than breast milk two to three times per day in the case of infants age 6-8 months and three to four times per day in the case of children age 9-23 months (Arimond and Ruel, 2003). Nonbreastfed children are considered to be fed in accordance with the minimum standards if they consume milk or milk products at least twice a day, are fed four food groups each day, and are fed at least four to five times per day (including milk feeds). Meal frequency is considered a proxy for energy intake from foods other than breast milk; therefore, the feeding frequency indicator for nonbreastfed children includes both milk and solid and semisolid foods (WHO, 2008).

Table 16.6 Infant and young child feeding (IYCF) practices

Percentage of youngest children age 6-23 months living with their mother who are fed according to three IYCF feeding practices based on breastfeeding status, number of food groups, and times they are fed during the day or night preceding the survey, by background characteristics, Cambodia 2014

	Among breastfed children 6-23 months, percentage fed:				Among non-breastfed children 6-23 months, percentage fed:					Among all children 6-23 months, percentage fed:				
Background characteristic	4+ food groups[1]	Minimum meal frequency[2]	Both 4+ food groups and minimum meal frequency	Number of breastfed children 6-23 months	Milk or milk products[3]	4+ food groups[1]	Minimum meal frequency[4]	With 3 IYCF practices[5]	Number of non-breastfed children 6-23 months	Breast milk, milk, or milk products[6]	4+ food groups[1]	Minimum meal frequency[7]	With 3 IYCF practices	Number of all children 6-23 months
**Age in months**														
6-8	15.0	71.0	14.8	367	(78.2)	(55.1)	(81.7)	(30.5)	29	98.4	17.9	71.8	15.9	396
9-11	35.5	68.1	25.3	309	(79.8)	(65.5)	(82.8)	(39.8)	40	97.7	39.0	69.8	27.0	349
12-17	51.2	77.1	41.0	524	64.9	66.3	76.3	28.8	150	92.2	54.6	76.9	38.3	673
18-23	56.1	79.5	45.9	287	37.8	64.8	62.3	24.1	437	62.5	61.4	69.1	32.7	724
**Sex**														
Male	39.9	73.6	31.9	760	54.1	67.1	71.6	25.6	326	86.2	48.1	73.0	30.0	1,086
Female	40.0	74.7	32.6	727	42.6	62.5	63.7	27.2	329	82.1	47.0	71.3	30.9	1,057
**Residence**														
Urban	54.7	77.5	47.2	140	81.5	83.8	89.3	49.6	161	90.1	70.3	83.9	48.5	300
Rural	38.4	73.8	30.7	1,348	37.5	58.6	60.5	18.9	494	83.2	43.9	70.2	27.5	1,842
**Province**														
Banteay Meanchey	44.2	83.9	43.1	59	(51.4)	(86.4)	(91.5)	(29.7)	30	83.6	58.5	86.5	38.6	89
Kampong Cham	26.7	67.4	19.8	215	(26.1)	(44.1)	(42.3)	(14.3)	77	80.6	31.2	60.8	18.4	292
Kampong Chhnang	39.6	86.1	35.5	58	(20.3)	(44.2)	(56.0)	(11.1)	23	77.7	40.9	77.7	28.7	81
Kampong Speu	54.9	81.0	50.5	104	(49.3)	(58.5)	(54.2)	(21.2)	42	85.5	55.9	73.4	42.1	146
Kampong Thom	45.0	75.7	38.0	77	(30.8)	(71.6)	(42.6)	(5.1)	30	80.8	52.4	66.5	28.8	107
Kandal	43.1	61.6	25.2	98	*	*	*	*	37	87.0	43.1	64.3	23.1	135
Kratie	20.1	67.5	14.9	57	(11.1)	(23.7)	(54.7)	(3.4)	19	77.3	21.0	64.2	12.0	76
Phnom Penh	69.7	84.9	59.9	76	94.7	91.4	97.2	61.8	110	96.9	82.6	92.2	61.0	186
Prey Veng	31.1	83.6	29.6	113	*	*	*	*	32	83.9	34.6	74.4	25.0	146
Pursat	28.0	83.7	24.0	69	(33.0)	(38.6)	(60.9)	(12.8)	27	81.1	31.0	77.3	20.8	96
Siem Reap	46.5	83.8	38.6	77	(41.1)	(77.8)	(84.2)	(32.7)	52	76.4	59.1	84.0	36.2	129
Svay Rieng	63.3	59.0	40.5	55	(52.7)	(72.3)	(58.8)	(31.5)	19	87.7	65.7	59.0	38.1	75
Takeo	47.3	89.2	45.6	84	(43.6)	(81.6)	(83.8)	(32.4)	37	82.7	57.8	87.5	41.6	121
Otdar Meanchey	37.0	64.5	30.1	33	(21.0)	(43.7)	(35.6)	(2.0)	9	83.3	38.4	58.4	24.2	42
Battambang/Pailin	40.5	53.9	22.3	125	(56.9)	(74.2)	(64.0)	(31.4)	48	88.1	49.8	56.7	24.8	173
Kampot/Kep	30.3	91.2	30.3	56	(35.1)	(51.3)	(72.0)	(4.6)	26	79.2	37.0	85.0	22.0	82
Preah Sihanouk/ Koh Kong	54.2	80.1	46.6	31	(53.0)	(64.5)	(72.3)	(18.3)	15	84.6	57.6	77.5	37.3	46
Preah Vihear/ Stung Treng	20.3	74.3	16.8	54	(37.7)	(66.7)	(60.5)	(14.6)	10	89.9	27.8	72.0	16.5	65
Mondul Kiri/ Ratanak Kiri	37.7	51.3	24.1	45	(45.8)	(69.5)	(41.9)	(25.2)	12	88.7	44.3	49.3	24.4	57
**Mother's education**														
No education	28.9	71.1	23.2	200	22.9	49.0	53.7	12.1	70	80.0	34.1	66.6	20.3	270
Primary	36.6	72.8	28.6	806	44.6	63.9	64.4	21.7	290	85.3	43.8	70.6	26.7	1,097
Secondary and higher	50.3	77.7	42.1	481	58.1	69.4	74.1	34.4	295	84.1	57.5	76.3	39.2	776
**Wealth quintile**														
Lowest	29.1	72.5	22.5	382	17.2	45.2	42.9	7.5	132	78.8	33.2	64.9	18.6	514
Second	37.4	72.5	30.8	324	25.1	53.5	59.0	10.8	103	82.0	41.3	69.2	26.0	427
Middle	39.7	76.0	30.2	289	36.2	56.0	66.1	13.4	109	82.5	44.1	73.3	25.6	397
Fourth	47.6	75.5	37.9	276	51.7	71.6	65.5	29.5	105	86.7	54.2	72.7	35.6	382
Highest	53.8	75.3	46.8	216	84.4	84.0	89.5	51.4	207	92.4	68.6	82.2	49.1	423
Total	40.0	74.2	32.2	1,488	48.3	64.8	67.6	26.4	655	84.2	47.6	72.2	30.4	2,143

Note: Figures in parentheses are based on 25-49 unweighted cases. An asterisk indicates that a figure is based on fewer than 25 unweighted cases and has been suppressed.
[1] Food groups: a. infant formula, milk other than breast milk, cheese or yogurt or other milk products; b. foods made from grains, roots, and tubers, including porridge and fortified baby food from grains; c. vitamin A-rich fruits and vegetables (and red palm oil); d. other fruits and vegetables; e. eggs; f. meat, poultry, fish, and shellfish (and organ meats); g. legumes and nuts.
[2] For breastfed children, minimum meal frequency is receiving solid or semisolid food at least twice a day for infants age 6-8 months and at least three times a day for children age 9-23 months.
[3] Includes two or more feedings of commercial infant formula; fresh, tinned, and powdered animal milk; and yogurt
[4] For nonbreastfed children age 6-23 months, minimum meal frequency is receiving solid or semisolid food or milk feeds at least four times a day.
[5] Nonbreastfed children age 6-23 months are considered to be fed with a minimum standard of three IYCF practices if they receive other milk or milk products at least twice a day, receive the minimum meal frequency, and receive solid or semisolid foods from at least four food groups not including the milk or milk products food group.
[6] Breastfeeding, or not breastfeeding and receiving two or more feedings of commercial infant formula; fresh, tinned, and powdered animal milk; and yogurt
[7] Children are fed the minimum recommended number of times per day according to their age and breastfeeding status as described in notes 2 and 4.

According to the results presented in Table 16.6, 40 percent of breastfed children age 6-23 months were given foods from four or more food groups in the 24 hours preceding the survey, and 74 percent were fed the minimum number of times in the preceding 24 hours. Almost 1 in 3 (32 percent) breastfed children fell into both categories; that is, their feeding practices met minimum standards with respect to food diversity as well as feeding frequency.

Among nonbreastfed children age 6-23 months, 48 percent were given milk or milk products, 65 percent were given food from at least four food groups, and 68 percent were fed four or more times per day. However, only about 1 in 4 children (26 percent) were fed in accordance with all three IYCF practices. Appropriate feeding practices were more common among breastfed children than nonbreastfed children.

Overall, 30 percent of Cambodian children age 6-23 months met the minimum standard with respect to all three IYCF feeding practices (Table 16.6). Eighty-four percent of all children age 6-23 months received breast milk or other milk or milk products during the 24-hour period before the survey, and 72 percent were fed the minimum number of times in the preceding 24 hours. The most common problem with feeding practices was an inadequate number of food groups; only 48 percent of children received foods from the minimum number of food groups for their age.

The proportion of children age 6-23 months meeting all three recommended IYCF standards increased from 15 percent among children age 6-8 months to 38 percent among those age 12-17 months and then fell to 33 percent among those age 18-23 months. The proportions of children who met the criteria did not vary by sex of the child. Urban children were more likely to be fed according to all of the IYCF practices than rural children (49 percent versus 28 percent). There were large regional differences in feeding practices. Children residing in Phnom Penh were most likely to be fed according to the recommended IYCF practices (61 percent), whereas children in Kratie were least likely to be fed according to the recommendations (12 percent). The proportions of children fed in accordance with the IYCF criteria were highest among children of mothers with a secondary education or higher (39 percent) and those in the highest wealth quintile (49 percent).

Figure 16.5 presents a comparison of the IYCF data from the 2010 and 2014 CDHS surveys. Since 2010, there have been improvements in infant and young child feeding practices. The percentage of children fed according to the IYCF practices increased from 24 percent to 30 percent between 2010 and 2014, with larger improvements observed among nonbreastfed than breastfed children (Figure 16.5).

Figure 16.5 **Trends in infant and young child feeding (IYCF) practices**

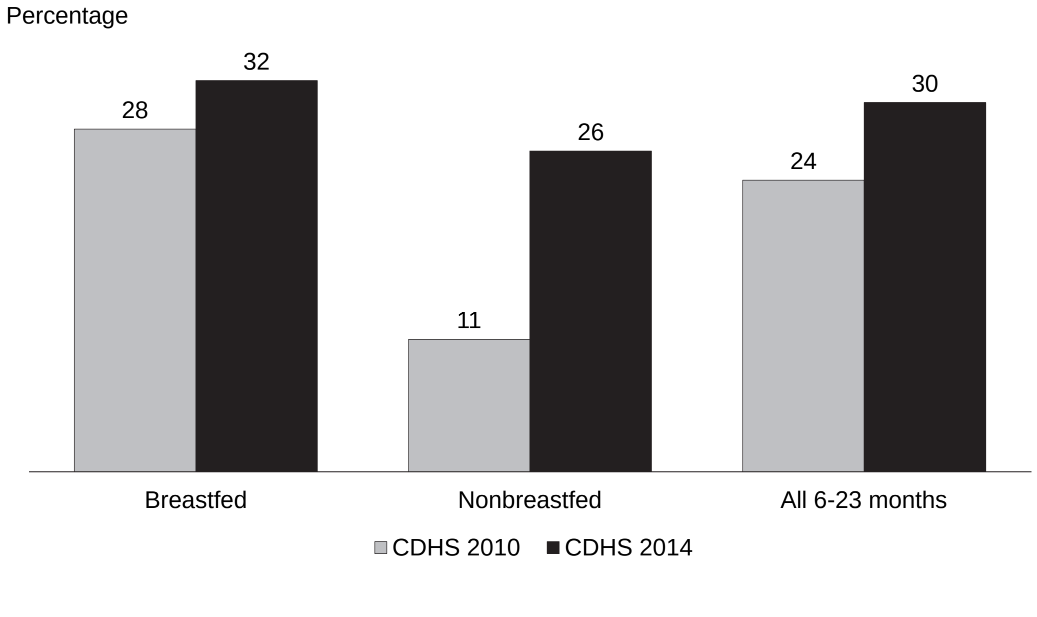

16.7 Prevalence of Anemia in Children

Common causes of anemia, characterized by a low level of hemoglobin in the blood, include inadequate intake of iron, folate, vitamin B12, and other nutrients. Anemia can also result from thalassemia, sickle cell disease, malaria, and intestinal worm infestation. Anemia may be an underlying cause of maternal mortality, spontaneous abortion, premature birth, and low birth weight. Iron and folic acid supplementation and antimalarial prophylaxis for pregnant women, promotion of the use of insecticide-treated bednets by pregnant women and children under age 5, and six-month deworming for children are some important measures used to reduce anemia prevalence among vulnerable groups.

Table 16.7 shows the prevalence of anemia among children age 6 to 59 months according to selected background characteristics. Unadjusted (i.e., measured) values of hemoglobin were obtained using the HemoCue instrument. Given that hemoglobin requirements differ substantially depending on altitude, an adjustment to sea-level equivalents is typically made before classifying children by level of anemia. Based on the altitude information derived from the clusters surveyed for the 2014 CDHS, no adjustment was required in the measured hemoglobin values.

The results show that anemia is a critical public health problem in Cambodia, where more than half (56 percent) of children age 6-59 months are anemic, with 30 percent mildly anemic, 25 percent moderately anemic, and less than 1 percent severely anemic. Anemia is highest among children age 9-11 months (83 percent) and declines gradually among older children, reaching a low of 40 percent among children age 48-59 months. Rural children are more likely (57 percent) to be anemic than urban children (43 percent). The prevalence of anemia in the different provinces ranges from 40 percent among children in Banteay Meanchey to 69 percent among children in Preah Vihear/Stung Treng. Children residing in the poorest households are more likely than other children to be anemic. For example, 64 percent of children in the lowest wealth quintile are anemic, as compared with 43 percent of children in the highest wealth quintile. Children whose mothers have a secondary education or higher are less likely to be anemic than other children.

Table 16.7 Prevalence of anemia in children

Percentage of children age 6-59 months classified as having anemia, by background characteristics, Cambodia 2014

Background characteristic	Anemia status by hemoglobin level: Any anemia (<11.0 g/dl)	Mild anemia (10.0-10.9 g/dl)	Moderate anemia (7.0-9.9 g/dl)	Severe anemia (<7.0 g/dl)	Number of children
**Age in months**					
6-8	77.2	30.3	46.3	0.6	244
9-11	82.8	31.4	50.7	0.7	230
12-17	76.4	29.1	45.8	1.5	515
18-23	68.5	30.2	37.7	0.5	542
24-35	50.5	28.2	21.9	0.4	1,013
36-47	45.3	31.0	14.0	0.3	976
48-59	40.3	29.9	10.3	0.1	936
**Sex**					
Male	56.7	28.8	27.4	0.5	2,280
Female	54.2	30.8	23.0	0.5	2,176
**Mother's interview status**					
Interviewed	56.6	29.7	26.4	0.5	3,836
Not interviewed but in household	54.8	30.6	24.1	0.0	103
Not interviewed and not in the household[1]	47.7	30.4	16.7	0.6	516
**Residence**					
Urban	43.4	25.7	17.5	0.2	591
Rural	57.4	30.4	26.4	0.5	3,864
**Province**					
Banteay Meanchey	39.7	21.1	17.2	1.4	222
Kampong Cham	62.7	40.3	22.4	0.0	625
Kampong Chhnang	59.2	27.9	31.2	0.0	161
Kampong Speu	63.9	35.2	27.8	0.9	301
Kampong Thom	66.0	36.2	29.3	0.4	197
Kandal	58.6	24.9	33.7	0.0	267
Kratie	50.2	28.5	21.7	0.0	157
Phnom Penh	41.0	24.5	16.5	0.0	335
Prey Veng	51.3	26.5	24.2	0.5	345
Pursat	64.8	25.7	36.8	2.3	192
Siem Reap	52.3	29.1	22.8	0.4	306
Svay Rieng	49.8	22.9	26.3	0.6	168
Takeo	53.1	29.0	23.5	0.6	245
Otdar Meanchey	64.3	37.1	27.3	0.0	77
Battambang/Pailin	49.0	26.8	22.2	0.0	344
Kampot/Kep	57.3	31.8	25.0	0.5	177
Preah Sihanouk/ Koh Kong	58.1	29.4	27.5	1.2	92
Preah Vihear/ Stung Treng	68.8	27.1	41.0	0.7	128
Mondul Kiri/ Ratanak Kiri	57.7	30.3	25.8	1.6	117
**Mother's education[2]**					
No education	56.6	28.1	27.4	1.2	537
Primary	58.8	30.5	28.0	0.4	2,192
Secondary and higher	52.4	28.6	23.5	0.3	1,116
**Wealth quintile**					
Lowest	64.1	31.0	32.4	0.6	1,104
Second	60.6	32.9	26.9	0.8	929
Middle	53.5	27.3	25.8	0.3	890
Fourth	51.9	29.9	21.5	0.5	756
Highest	43.2	27.0	16.1	0.1	777
Total	55.5	29.8	25.2	0.5	4,456

Note: Table is based on children who stayed in the household on the night before the interview and who were tested for anemia. Prevalence of anemia, based on hemoglobin levels, is adjusted for altitude using formulas in CDC, 1998. Hemoglobin is in grams per deciliter (g/dl).
[1] Includes children whose mothers are deceased
[2] For women who are not interviewed, information is taken from the Household Questionnaire. Excludes children whose mothers are not listed in the Household Questionnaire.

A comparison with earlier CDHS surveys shows that the prevalence of anemia decreased between 2005 and 2010 but has remained relatively unchanged over the past four years (Figure 16.6).

Figure 16.6 **Trends in anemia status among children under age 5**

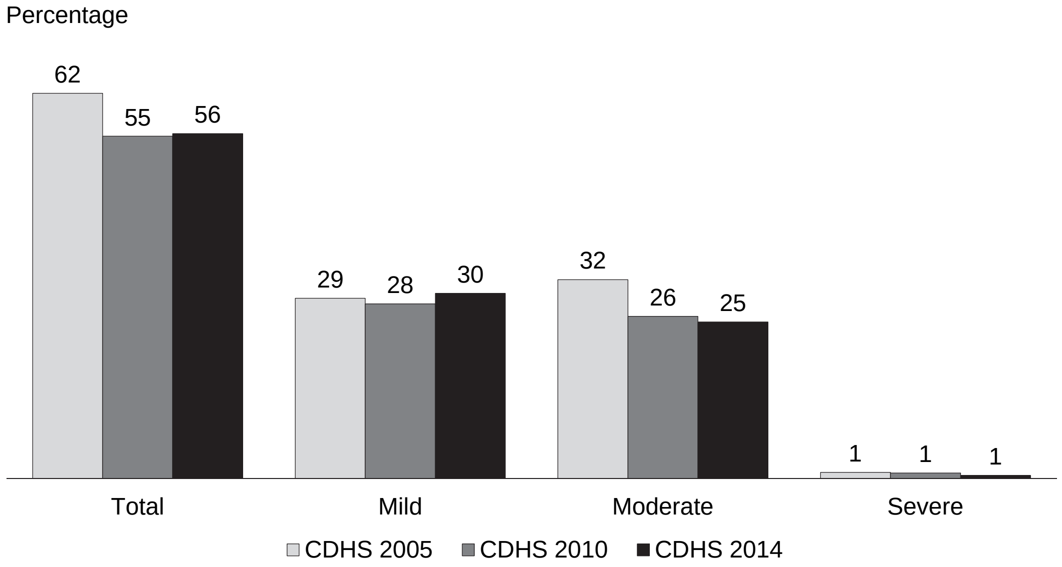

16.8 MICRONUTRIENT INTAKE AMONG CHILDREN

A serious contributor to childhood morbidity and mortality is micronutrient deficiency. Children can receive micronutrients from foods, food fortification, and direct supplementation. Table 16.8 looks at measures relating to intake of several key micronutrients among children.

Vitamin A is an essential micronutrient for the immune system and plays an important role in maintaining the epithelial tissue in the body. Severe vitamin A deficiency (VAD) can cause eye damage. VAD can also increase the severity of infections such as measles and diarrheal diseases in children and slows recovery from illness. Vitamin A is found in breast milk, other milks, liver, eggs, fish, butter, red palm oil, mangoes, papayas, carrots, pumpkins, and dark green leafy vegetables. The liver can store an adequate amount of the vitamin for four to six months. Periodic dosing (usually every six months) of vitamin A supplements is one method of ensuring that children at risk do not develop VAD.

In 2009, the National Nutrition Program developed a National Vitamin A Policy that ensures uniform provision of vitamin A to children age 6-59 months. Mebendazol, a deworming drug, is also given to children age 12-59 months. Currently, the vitamin A capsules, together with the Mebendazol, are distributed through outreach sessions held twice annually in May and November. The provisions of iodine and iron are made through salt that is iodized and in fish sauce, soy milk, and common children's snacks that are fortified with iron.

The CDHS collected information on the consumption of foods rich in vitamin A and iron and on the coverage of supplements. Table 16.8 shows that 85 percent of last-born children age 6-23 months living with their mother consumed foods rich in vitamin A in the 24-hour period before the survey. Consumption of foods rich in vitamin A increases from 50 percent among children age 6-8 months to 98 percent among children age 18-23 months. There is no gender difference in the consumption of foods rich in vitamin A. Nonbreastfeeding children are more likely to consume foods rich in vitamin A than breastfeeding children (95 percent versus 80 percent). At least 90 percent of children living in Kampong Chhnang, Kampong Speu, Kampong Thom, Kratie, and Phnom Penh consumed foods rich in vitamin A the day or night preceding the survey. Vitamin A consumption was lowest in Kampong Cham (73 percent).

Eighty-two percent of children consume foods rich in iron. The differences in consumption of iron-rich foods by background characteristics are similar to those seen for consumption of foods rich in vitamin A.

Table 16.8 Micronutrient intake among children

Among youngest children age 6-23 months who are living with their mother, the percentages who consumed vitamin A-rich and iron-rich foods in the day or night preceding the survey; among all children age 6-59 months, the percentages who were given vitamin A supplements in the six months preceding the survey, who were given iron supplements in the past seven days, and who were given deworming medication in the six months preceding the survey; and among all children age 6-59 months who live in households that were tested for iodized salt, the percentage who live in households with iodized salt, by background characteristics, Cambodia 2014

	Among youngest children age 6-23 months living with their mother:			Among all children age 6-59 months:				Among children age 6-59 months living in households tested for iodized salt:	
Background characteristic	Percentage who consumed foods rich in vitamin A in last 24 hours[1]	Percentage who consumed foods rich in iron in last 24 hours[2]	Number of children	Percentage given vitamin A supplements in last 6 months	Percentage given iron supplements in last 7 days	Percentage given deworming medication in last 6 months[3]	Number of children	Percentage living in households with iodized salt[4]	Number of children
**Age in months**									
6-8	49.9	45.5	396	39.3	3.6	11.7	403	59.8	389
9-11	81.1	77.5	349	58.9	4.6	29.6	357	70.8	357
12-17	93.1	89.8	673	70.0	7.3	44.9	689	69.1	684
18-23	97.5	95.9	724	71.1	5.9	62.6	771	68.2	762
24-35	na	na	na	72.6	8.1	65.3	1,368	70.7	1,349
36-47	na	na	na	74.6	6.6	70.3	1,343	67.6	1,330
48-59	na	na	na	72.2	4.7	67.1	1,376	69.7	1,365
**Sex**									
Male	85.2	82.3	1,086	70.8	6.1	59.7	3,170	68.6	3,133
Female	84.0	81.1	1,057	68.4	6.3	57.8	3,139	68.8	3,103
**Breastfeeding status**									
Breastfeeding	80.1	76.3	1,488	60.1	7.0	40.9	1,720	65.5	1,693
Not breastfeeding	94.9	93.9	655	73.1	5.9	65.4	4,578	69.9	4,533
**Mother's age at birth**									
15-19	77.7	75.7	110	57.4	4.4	33.7	158	65.2	153
20-29	84.2	81.1	1,299	69.3	6.2	58.1	3,426	66.8	3,391
30-39	87.1	84.1	666	71.8	6.9	62.0	2,373	71.0	2,343
40-49	79.1	78.0	67	62.8	2.0	54.4	351	73.2	348
**Residence**									
Urban	90.3	88.5	300	63.7	3.5	49.5	929	82.0	920
Rural	83.7	80.6	1,842	70.6	6.6	60.3	5,379	66.4	5,316
**Province**									
Banteay Meanchey	82.2	79.7	89	84.5	2.4	71.3	220	90.1	217
Kampong Cham	73.0	70.2	292	67.2	3.7	51.1	865	33.5	855
Kampong Chhnang	94.6	92.4	81	85.5	2.9	77.7	210	68.6	210
Kampong Speu	89.5	89.5	146	58.7	6.0	47.1	424	89.3	412
Kampong Thom	92.3	89.0	107	90.8	8.4	81.5	299	87.5	299
Kandal	77.4	77.4	135	66.2	18.5	54.3	450	84.6	449
Kratie	93.0	86.5	76	57.6	0.2	52.6	225	90.4	224
Phnom Penh	95.8	94.7	186	60.2	3.5	44.0	554	77.5	546
Prey Veng	85.4	79.6	146	69.6	20.1	55.1	435	63.3	433
Pursat	77.1	70.8	96	77.9	2.8	75.2	272	47.3	268
Siem Reap	86.6	81.4	129	73.4	3.1	59.5	419	77.4	406
Svay Rieng	84.0	82.5	75	67.0	3.6	55.7	223	94.9	223
Takeo	85.1	81.3	121	85.8	10.5	79.1	335	87.2	331
Otdar Meanchey	79.6	75.6	42	65.2	1.2	56.1	122	77.9	120
Battambang/Pailin	85.6	82.6	173	75.1	2.1	67.9	471	46.8	467
Kampot/Kep	89.2	86.5	82	51.1	0.3	49.3	240	14.7	237
Preah Sihanouk/ Koh Kong	81.5	80.1	46	72.6	1.9	62.5	149	95.9	147
Preah Vihear/ Stung Treng	78.6	75.1	65	73.6	13.1	62.2	205	81.0	199
Mondul Kiri/ Ratanak Kiri	85.8	83.4	57	46.1	0.0	44.6	192	81.6	191
**Mother's education**									
No education	82.8	78.8	270	65.1	4.5	54.9	887	64.1	871
Primary	83.5	80.5	1,097	70.5	6.4	60.0	3,322	67.2	3,271
Secondary and higher	86.9	84.4	776	70.0	6.5	58.4	2,099	73.0	2,094
**Wealth quintile**									
Lowest	82.0	78.4	514	68.9	4.2	57.5	1,526	61.9	1,499
Second	86.5	83.3	427	68.9	4.7	58.6	1,258	63.6	1,238
Middle	86.8	82.6	397	69.9	8.8	60.6	1,179	64.3	1,173
Fourth	82.7	80.5	382	73.6	8.3	63.9	1,068	75.9	1,060
Highest	85.7	84.3	423	67.5	5.7	54.4	1,277	79.8	1,266
Total	84.6	81.7	2,143	69.6	6.2	58.7	6,308	68.7	6,236

Note: Information on vitamin A is based on both mother's recall and the immunization card (where available). Information on iron supplements and deworming medication is based on the mother's recall. Total includes 10 cases for which information on breastfeeding is missing.
na = Not applicable
[1] Includes meat (and organ meat), fish, poultry, eggs, pumpkin, red or yellow yams or squash, carrots, red sweet potatoes, dark green leafy vegetables, mango, papaya, and other locally grown fruits and vegetables that are rich in vitamin A
[2] Includes meat (and organ meat), fish, poultry, and eggs
[3] Deworming for intestinal parasites is commonly done for helminthes and for schistosomiasis.
[4] Excludes children in households in which salt was not tested

Seventy percent of children age 6-59 months received a vitamin A supplement in the six months before the survey, about the same percentage observed in the 2010 CDHS (71 percent). The difference in consumption of vitamin A supplements between boys and girls is small (71 percent versus 68 percent). Children who were not breastfeeding were more likely to receive vitamin A supplements (73 percent) than children who were breastfeeding (60 percent). Seventy-one percent of rural children received vitamin A supplements, as compared with 64 percent of urban children. The proportion of children who received vitamin A supplements was lowest in Mondul Kiri/Ratanak Kiri (46 percent) and highest in Kampong Thom (91 percent).

Only 6 percent of children age 6-59 months received iron supplementation in the seven days preceding the survey. However, 59 percent of children received deworming medication in the six months before the survey.

Inadequate amounts of iodine in the diet are related to serious health risks for young children. The 2014 CDHS results show that 69 percent of children age 6-59 months live in households using iodized salt. Differences are sizable by area of residence; 82 percent of urban children live in households with iodized salt, as compared with 66 percent of rural children. At least 90 percent of children living in Banteay Meanchey, Kratie, Svay Rieng, and Preah Sihanouk/Koh Kong live in households using iodized salt. Children whose mothers have a secondary education or higher are more likely to live in households with iodized salt (73 percent) than are children whose mothers do not have any formal schooling (64 percent). The percentage of children living in households with iodized salt is positively associated with wealth quintile.

16.9 USE OF IODIZED SALT

Iodine is an important micronutrient. Dietary iodine deficiencies are a major public health concern worldwide. A lack of sufficient iodine is known to cause goiter, cretinism (a severe form of neurological defect), spontaneous abortion, premature birth, infertility, stillbirth, and increased child mortality. Iodine deficiency disorder is the most common cause of preventable mental retardation and brain damage in the world.

In the 2014 CDHS, a rapid test was used to determine the presence or absence of iodine in the salt used for cooking in the household.

Table 16.9 shows the percentage of households using iodized salt. Overall, 69 percent of households have salt with some iodine. This figure is substantially lower than that found in 2010 (83 percent) but is only slightly lower than the figure reported in the 2005 CDHS (73 percent). A higher percentage of urban households (82 percent) than rural households (67 percent) are using iodized salt. The consumption of iodized salt is lowest in Kampot/Kep (14 percent) and Kampong Cham (34 percent). Households in the highest wealth quintile are more likely (82 percent) than households in the lower wealth quintiles to use salt that is adequately iodized.

Table 16.9 Presence of iodized salt in household

Among all households, the percentage with salt tested for iodine content and the percentage with no salt in the household, and among households with salt tested, the percentage with iodized salt, according to background characteristics, Cambodia 2014

Background characteristic	Among all households: Percentage with salt tested	Percentage with no salt in the household	Number of households	Among households with tested salt: Percentage with iodized salt	Number of households
**Residence**					
Urban	98.9	1.1	2,284	82.3	2,257
Rural	98.8	1.2	13,541	66.6	13,376
**Province**					
Banteay Meanchey	99.2	0.8	670	86.4	664
Kampong Cham	98.3	1.7	1,997	33.6	1,964
Kampong Chhnang	99.7	0.3	608	64.1	607
Kampong Speu	98.6	1.4	973	89.4	959
Kampong Thom	100.0	0.0	801	85.5	801
Kandal	99.5	0.5	1,259	87.8	1,253
Kratie	99.2	0.8	451	92.3	448
Phnom Penh	98.2	1.8	1,293	80.7	1,270
Prey Veng	98.2	1.8	1,228	62.0	1,206
Pursat	98.8	1.2	611	45.1	603
Siem Reap	98.2	1.8	1,000	79.4	982
Svay Rieng	99.7	0.3	678	94.5	676
Takeo	98.7	1.3	1,011	87.2	998
Otdar Meanchey	98.4	1.6	271	80.5	267
Battambang/Pailin	99.0	1.0	1,222	48.6	1,209
Kampot/Kep	98.4	1.6	762	14.1	750
Preah Sihanouk/Koh Kong	99.3	0.7	320	95.9	318
Preah Vihear/Stung Treng	97.7	2.3	361	85.3	352
Mondul Kiri/Ratanak Kiri	99.0	1.0	309	79.8	306
**Wealth quintile**					
Lowest	98.2	1.8	3,208	59.0	3,149
Second	98.5	1.5	3,320	63.6	3,271
Middle	99.1	0.9	3,147	67.3	3,119
Fourth	99.0	1.0	3,176	74.0	3,146
Highest	99.1	0.9	2,975	81.6	2,949
Total	98.8	1.2	15,825	68.9	15,633

16.10 NUTRITIONAL STATUS OF WOMEN

The height and weight of women age 15-49 were measured among a two-thirds subsample of households selected in the 2014 CDHS. In this report, two indicators of nutritional status are presented: height and body mass index.

The height of a woman is associated with past socioeconomic status and nutrition during childhood and adolescence. A woman's height is used to predict the risk of difficulty in delivery because small stature is often associated with small pelvis size and the potential for obstructed labor. The risk of giving birth to a low birth weight baby is influenced by the mother's nutritional status. The cutoff point for the height at which mothers can be considered at risk varies between populations but normally falls between 140 and 150 centimeters. As in other DHS surveys, a cutoff point of 145 cm was used for the 2014 CDHS.

The index used to measure thinness or obesity is known as the body mass index, or the Quetelet index. BMI is defined as weight in kilograms divided by height squared in meters (kg/m^2). A BMI of 18.5 or lower indicates thinness or acute undernutrition, a BMI of 25.0-29.9 indicates overweight, and a BMI of 30.0 or higher indicates obesity.

Table 16.10 presents the mean values of the two indicators of nutritional status and the proportions of women falling into high-risk categories, according to background characteristics. Women for whom there was no information on height and/or weight and for whom a BMI could not be estimated are excluded from this analysis. The BMI data analysis is based on 10,624 women, whereas the height analysis is based on 11,380 women.

Table 16.10 Nutritional status of women

Among women age 15-49, the percentage with height under 145 cm, mean body mass index (BMI), and the percentage with specific BMI levels, by background characteristics, Cambodia 2014

	Height		Body mass index[1]								
Background characteristic	Percentage below 145 cm	Number of women	Mean BMI	18.5-24.9 (total normal)	<18.5 (total thin)	17.0-18.4 (mildly thin)	<17 (moderately and severely thin)	≥25.0 (total overweight or obese)	25.0-29.9 (overweight)	≥30.0 (obese)	Number of women
**Age**											
15-19	5.9	1,826	19.9	69.6	27.5	18.4	9.1	2.9	2.8	0.1	1,724
20-29	4.5	3,927	21.3	72.0	17.1	12.8	4.3	10.9	9.3	1.6	3,458
30-39	6.2	3,085	22.9	68.8	7.2	5.6	1.5	24.0	20.2	3.8	2,917
40-49	5.3	2,543	23.4	60.3	8.4	5.4	3.0	31.3	26.1	5.2	2,526
**Residence**											
Urban	3.8	2,187	22.3	64.0	13.5	9.9	3.6	22.5	18.4	4.2	2,056
Rural	5.7	9,194	21.9	68.9	14.1	10.0	4.1	17.0	14.5	2.5	8,569
**Province**											
Banteay Meanchey	4.0	448	23.0	62.9	10.7	8.3	2.4	26.4	21.3	5.1	418
Kampong Cham	8.8	1,252	22.2	70.0	10.2	7.4	2.8	19.8	16.9	2.9	1,176
Kampong Chhnang	6.8	418	21.4	67.5	18.0	14.3	3.8	14.5	12.6	1.9	389
Kampong Speu	7.3	793	21.1	68.4	20.9	14.5	6.4	10.7	10.0	0.7	737
Kampong Thom	2.3	568	22.0	71.2	12.5	8.7	3.8	16.4	13.6	2.8	526
Kandal	4.4	867	21.7	65.1	17.9	14.0	4.0	17.0	14.5	2.5	807
Kratie	4.7	320	21.7	71.5	15.8	11.1	4.6	12.7	10.7	2.0	287
Phnom Penh	3.2	1,342	22.2	65.6	14.0	10.3	3.7	20.4	17.3	3.1	1,269
Prey Veng	4.3	758	22.2	69.1	12.5	7.8	4.7	18.4	15.2	3.2	708
Pursat	5.6	419	21.7	70.5	13.1	8.5	4.6	16.4	15.6	0.9	384
Siem Reap	5.4	731	22.1	71.4	11.6	8.3	3.4	17.0	14.0	3.0	686
Svay Rieng	5.7	430	22.0	67.7	13.1	9.3	3.8	19.2	17.9	1.3	405
Takeo	4.2	688	21.9	65.8	13.9	8.1	5.8	20.3	17.5	2.8	653
Otdar Meanchey	3.4	191	22.0	72.0	12.8	11.4	1.4	15.2	11.7	3.5	170
Battambang/Pailin	3.8	848	22.7	65.7	11.9	9.6	2.4	22.4	17.1	5.2	800
Kampot/Kep	4.4	481	21.6	69.1	17.0	10.0	7.0	14.0	12.0	2.0	455
Preah Sihanouk/ Koh Kong	4.5	274	22.4	60.5	15.6	11.3	4.4	23.8	18.6	5.2	254
Preah Vihear/ Stung Treng	8.0	314	21.1	73.7	16.4	11.2	5.2	9.9	8.9	1.0	282
Mondul Kiri/ Ratanak Kiri	16.8	240	22.2	70.6	10.4	8.8	1.6	18.9	15.6	3.4	219
**Education**											
No education	8.5	1,479	22.6	66.7	10.9	7.8	3.1	22.5	18.2	4.3	1,396
Primary	5.6	5,379	22.3	67.2	12.4	9.1	3.2	20.4	17.2	3.2	5,030
Secondary and higher	4.1	4,523	21.4	69.3	17.0	11.8	5.2	13.7	11.9	1.8	4,198
**Wealth quintile**											
Lowest	7.6	2,060	21.4	73.7	15.3	10.3	5.0	11.0	9.7	1.3	1,917
Second	6.6	2,118	21.9	68.8	14.6	10.4	4.2	16.6	14.2	2.4	1,944
Middle	5.5	2,168	21.9	67.7	14.3	10.0	4.3	18.0	14.9	3.1	2,026
Fourth	4.3	2,308	22.1	66.6	13.5	10.2	3.3	20.0	17.4	2.5	2,168
Highest	3.6	2,728	22.5	64.5	12.7	9.2	3.5	22.7	18.5	4.3	2,569
Total	5.4	11,380	22.0	68.0	14.0	10.0	4.0	18.0	15.2	2.8	10,624

Note: Body mass index is expressed as the ratio of weight in kilograms to the square of height in meters (kg/m^2).
[1] Excludes pregnant women and women with a birth in the preceding 2 months

Overall, 5 percent of women are shorter than 145 cm. A larger percentage of women in Mondul Kiri/Ratanak Kiri are below 145 cm (17 percent) than women in other provinces. As expected, women with no schooling and those in the lowest wealth quintile are more likely to be shorter than 145 cm.

Table 16.10 shows that 14 percent of women are underweight or thin (BMI less than 18.5), and 18 percent are overweight or obese (BMI 25.0 or higher). There are large differentials across background characteristics in the percentage of women assessed as thin and overweight or obese. For example, the proportion of women who are thin generally decreases with age group, while the proportion who are overweight or obese increases with age. The percentage of overweight or obese women is higher in urban areas (23 percent) than in rural areas (17 percent). Comparisons across provinces show that Kampong Speu (21 percent), Kampong Chhnang (18 percent), and Kandal (18 percent) have the highest percentages of undernourished women, whereas the lowest proportion of undernourished women is found in Kampong Cham (10 percent). The percentage of overweight or obese women in the highest wealth quintile is more than two times that of the lowest quintile (23 percent versus 11 percent).

A comparison with the previous CDHS surveys shows that the proportion of undernourished women in the reproductive age group has declined more substantially between the most recent two surveys than the previous ones. However, the prevalence of overweight and obesity, another form of malnutrition, has increased remarkably (Figure 16.7).

Figure 16.7 **Trends in nutritional status among women age 15-49**

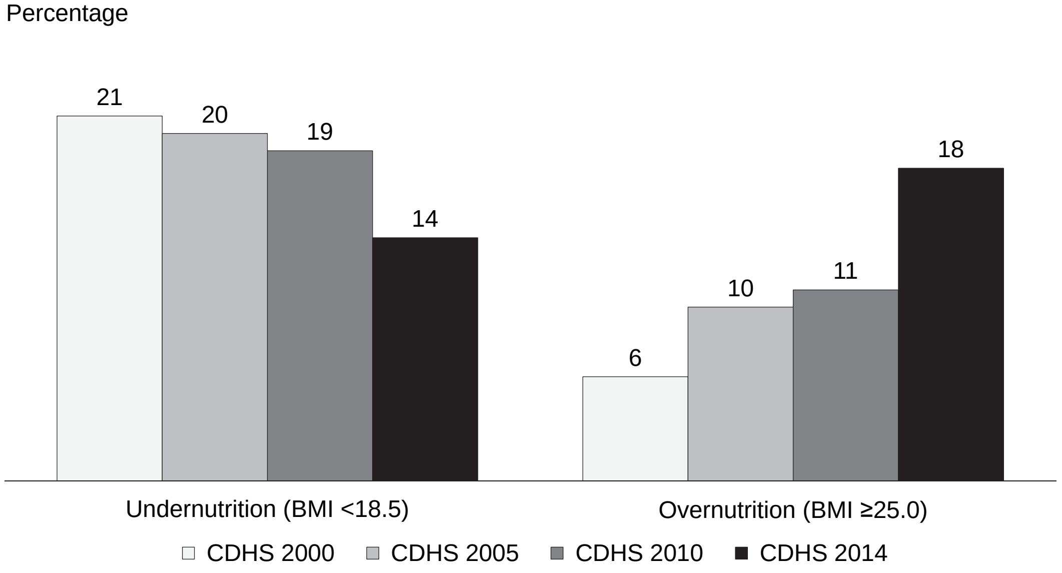

16.11 PREVALENCE OF ANEMIA IN WOMEN

Table 16.11 shows the prevalence of anemia among women age 15-49, adjusted for smoking status. Forty-five percent of Cambodian women are anemic, including 38 percent with mild anemia and 7 percent with moderate anemia. Less than 1 percent of women suffer from severe anemia.

Anemia shows a U-shaped pattern with respect to age, with younger and older women more likely to be anemic than their counterparts in the other age groups. A similar pattern is seen according to number of children. The prevalence of anemia is higher among women who have only a primary education or less, those who are pregnant, and those who live in poorer households. Also, the prevalence is higher among rural women (47 percent) than urban women (39 percent). Women residing in Banteay Meanchey have the lowest prevalence of anemia (31 percent), and women residing in Kampong Chhnang, Kampong Speu, and Preah Vihear/Stung Treng have the highest prevalence (53-54 percent). Anemia prevalence is higher among women who smoke (52 percent) than among women who do not smoke (45 percent) and is slightly higher among women who use an IUD than those who do not.

Table 16.11 Prevalence of anemia in women

Percentage of women age 15-49 with anemia, by background characteristics, Cambodia 2014

Background characteristic	Anemia status by hemoglobin level: Any anemia	Mild anemia	Moderate anemia	Severe anemia	Number of women
**Age**					
15-19	49.4	42.1	6.9	0.4	1,811
20-29	42.5	36.6	5.7	0.2	3,897
30-39	44.3	37.4	6.8	0.1	3,055
40-49	48.2	39.6	8.1	0.4	2,524
**Number of children ever born**					
0	46.5	39.3	6.9	0.3	3,585
1	44.7	37.5	6.9	0.4	1,865
2-3	43.2	37.5	5.4	0.2	3,653
4-5	46.4	38.4	7.7	0.3	1,541
6+	51.2	40.6	10.6	0.0	641
**Maternity status**					
Pregnant	53.2	30.4	22.4	0.4	615
Breastfeeding	51.5	44.6	6.8	0.2	1,566
Neither	43.8	37.9	5.6	0.3	9,106
**Using IUD**					
Yes	49.4	43.3	6.1	0.0	334
No	45.2	38.2	6.7	0.3	10,952
**Smoking status**					
Smokes cigarettes/tobacco	51.5	41.2	9.5	0.8	631
Does not smoke	45.0	38.2	6.6	0.2	10,655
**Residence**					
Urban	39.4	34.9	4.3	0.2	2,156
Rural	46.8	39.2	7.3	0.3	9,130
**Province**					
Banteay Meanchey	30.5	26.3	3.7	0.6	450
Kampong Cham	52.0	42.2	9.6	0.2	1,226
Kampong Chhnang	53.0	45.2	6.9	0.9	418
Kampong Speu	53.3	44.2	9.1	0.0	784
Kampong Thom	44.6	36.6	7.2	0.8	567
Kandal	49.4	41.3	7.9	0.2	867
Kratie	46.2	36.3	8.8	1.0	318
Phnom Penh	41.7	37.4	4.0	0.3	1,316
Prey Veng	46.9	39.8	7.1	0.0	749
Pursat	46.6	37.6	8.8	0.2	418
Siem Reap	41.1	34.3	6.3	0.4	722
Svay Rieng	45.7	38.2	7.3	0.3	427
Takeo	35.4	32.2	3.2	0.0	693
Otdar Meanchey	48.3	40.7	7.6	0.0	189
Battambang/Pailin	42.5	38.2	4.3	0.0	843
Kampot/Kep	44.1	39.1	4.7	0.3	479
Preah Sihanouk/Koh Kong	43.7	37.4	6.2	0.1	267
Preah Vihear/Stung Treng	53.7	41.4	12.2	0.0	313
Mondul Kiri/Ratanak Kiri	41.7	34.4	7.2	0.1	239
**Education**					
No education	47.8	38.8	8.4	0.5	1,473
Primary	47.5	39.5	7.8	0.3	5,337
Secondary and higher	42.0	37.0	4.9	0.2	4,476
**Wealth quintile**					
Lowest	53.3	42.2	10.5	0.6	2,055
Second	48.9	40.1	8.6	0.1	2,103
Middle	44.5	39.1	5.3	0.2	2,157
Fourth	43.1	36.7	6.3	0.1	2,292
Highest	39.1	35.1	3.8	0.2	2,680
Total	45.4	38.4	6.7	0.2	11,286

Note: Prevalence is adjusted for altitude and for smoking status if known using formulas in CDC, 1998.

Figure 16.8 indicates that the overall prevalence of anemia has remained more or less the same since the 2005 CDHS. This persistence of a high level of anemia requires more rigorous study to identify the causes and effective interventions.

Figure 16.8 **Trends in anemia status among women age 15-49**

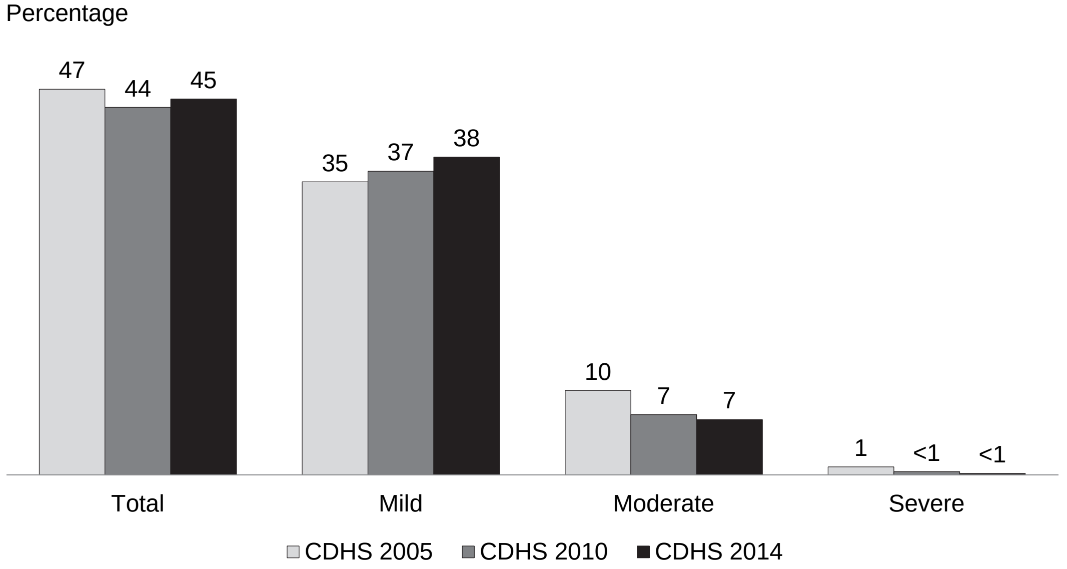

16.12 MICRONUTRIENT INTAKE AMONG MOTHERS

Adequate micronutrient intake by women has important benefits for both women and their children. Breastfeeding children benefit from micronutrient supplementation that mothers receive. Iron supplementation of women during pregnancy protects the mother and infant against anemia, which results in an increased risk of premature delivery and low birth weight. Finally, iodine deficiency is also related to a number of adverse pregnancy outcomes.

The Ministry of Health has developed and adopted a number of policies and guidelines addressing micronutrient deficiencies in women, including the National Guidelines for the Use of Iron/Folate Supplementation to Prevent and Treat Anemia in Pregnant and Postpartum Women (2007 revision). Iron/folate supplementation is provided to women during pregnancy (for 90 days) and in the postpartum period (for 42 days). In addition, Mebendazol is given to pregnant women during a prenatal care visit.

Table 16.12 presents the extent to which women receive iron supplements following delivery. Forty-nine percent of women reported that they had received iron/folate supplements in the six-week period following the delivery of their last-born child. With regard to iron supplementation during pregnancy, 94 percent of women who gave birth during the five-year period before the 2014 CDHS reported that they had taken iron tablets or syrup during the pregnancy preceding their last live birth; 76 percent indicated that they took the supplements for 90 days or more, which is recommended. The prevalence of iron supplement intake for 90 days or more varies little by area of residence. The lowest level of recommended iron supplementation was in Kratie (39 percent), and the highest level was in Kampong Chhnang (94 percent). The percentage of women who took iron supplements for the recommended 90 days increased with increasing education and wealth.

Almost three-quarters of women reported that they took deworming medicine (drugs for intestinal parasites) during pregnancy. Deworming medicine was more commonly used by rural than urban women and by women in Kampong Chhnang. The proportion of women who took drugs for intestinal parasites during pregnancy increases with increasing education; however, it shows no steady pattern by wealth quintile.

As was the case among children, about 7 in 10 mothers (69 percent) live in households with iodized salt. Women in Kampot/Kep (13 percent) are least likely to be living in households consuming iodized salt.

Table 16.12 Micronutrient intake among mothers

Among women age 15-49 with a child born in the past five years, the percentage who received iron tablets in the first six weeks after the birth of the last child, the percent distribution by number of days they took iron tablets or syrup during the pregnancy of the last child, and the percentage who took deworming medication during the pregnancy of the last child, and among women age 15-49 with a child born in the past five years and who live in households that were tested for iodized salt, the percentage who live in households with iodized salt, by background characteristics, Cambodia 2014

		Number of days women took iron tablets or syrup during pregnancy of last birth							Among women with a child born in the last five years who live in households that were tested for iodized salt:	
Background characteristic	Percentage who received iron postpartum[1]	None	<60	60-89	90+	Don't know/ missing	Percentage of women who took deworming medication during pregnancy of last birth	Number of women	Percentage living in households with iodized salt[2]	Number of women
**Age**										
15-19	42.1	5.7	13.7	6.7	74.0	0.0	71.8	213	63.1	207
20-29	49.0	3.2	7.7	11.1	76.5	1.6	74.1	3,227	67.5	3,193
30-39	50.0	4.4	8.2	9.2	76.2	2.1	70.5	2,175	70.9	2,151
40-49	43.6	14.2	9.6	8.9	64.2	3.1	64.3	357	74.6	351
**Residence**										
Urban	47.2	2.5	8.0	9.3	77.7	2.6	62.0	876	80.8	867
Rural	49.0	4.7	8.2	10.3	75.2	1.6	73.9	5,096	67.0	5,035
**Province**										
Banteay Meanchey	38.0	0.8	3.8	4.4	88.3	2.8	72.1	219	88.7	216
Kampong Cham	34.6	2.7	14.3	17.5	65.5	0.0	64.3	819	36.2	802
Kampong Chhnang	84.9	0.7	0.9	2.7	94.4	1.3	96.6	203	64.7	203
Kampong Speu	30.4	1.8	8.9	6.9	79.2	3.3	71.7	395	88.7	387
Kampong Thom	82.2	5.3	6.1	10.2	78.3	0.0	90.6	279	88.2	279
Kandal	45.0	5.0	11.4	4.5	79.0	0.0	71.1	420	84.6	419
Kratie	29.9	23.0	24.9	13.6	38.5	0.0	57.0	214	91.4	212
Phnom Penh	44.5	2.1	7.3	10.4	79.2	1.0	53.6	535	76.7	526
Prey Veng	59.0	1.1	1.8	9.3	87.8	0.0	84.5	405	63.2	404
Pursat	63.4	4.5	6.0	7.8	80.6	1.0	89.2	245	49.2	241
Siem Reap	62.7	3.1	9.2	13.2	73.1	1.4	74.3	379	79.3	370
Svay Rieng	31.5	1.9	6.6	12.0	78.2	1.2	70.1	229	95.4	229
Takeo	72.4	2.1	2.8	5.5	86.7	2.9	71.1	321	86.9	318
Otdar Meanchey	62.9	4.0	6.1	12.5	76.8	0.7	82.0	116	78.8	114
Battambang/Pailin	43.7	2.5	2.1	6.3	80.0	9.0	78.4	460	46.9	456
Kampot/Kep	37.8	7.1	8.4	13.4	66.7	4.5	72.7	236	13.3	234
Preah Sihanouk/ Koh Kong	62.7	3.2	11.1	8.2	74.5	3.0	66.1	142	95.9	141
Preah Vihear/ Stung Treng	38.8	9.8	13.4	12.9	63.3	0.6	59.6	188	82.3	183
Mondul Kiri/ Ratanak Kiri	42.5	22.3	6.9	14.8	55.9	0.1	72.0	169	81.4	168
**Education**										
No education	44.4	12.2	11.9	11.3	63.0	1.6	63.0	805	64.8	788
Primary	48.0	4.3	9.2	10.5	73.7	2.2	72.8	3,100	67.4	3,054
Secondary and higher	51.7	1.4	5.2	9.1	83.1	1.2	74.8	2,068	73.1	2,059
**Wealth quintile**										
Lowest	46.4	8.8	12.3	12.5	65.0	1.3	70.5	1,359	62.0	1,332
Second	50.9	4.8	9.5	9.5	74.8	1.5	71.6	1,215	65.1	1,198
Middle	48.0	3.5	6.3	11.8	76.7	1.7	76.3	1,133	64.7	1,127
Fourth	51.1	1.7	6.4	8.5	81.3	2.2	74.4	1,069	75.7	1,061
Highest	48.0	2.0	5.7	8.0	81.9	2.3	68.7	1,196	79.0	1,185
Total	48.8	4.4	8.2	10.1	75.5	1.8	72.2	5,973	69.0	5,902

[1] In the first six weeks after delivery of last birth
[2] Excludes women in households where salt was not tested

MICRONUTRIENTS 17

Key Findings

- Lack of iron storage is relatively rare in Cambodia. Only 3 percent each of mothers age 15-49 and their children born since January 2009 are affected.
- Nineteen percent of mothers and 10 percent of children are infected with at least one intestinal parasite. Hookworm is the most commonly found intestinal parasite, present in 15 percent of mothers and 7 percent of children.
- Three percent of mothers and 9 percent of children have vitamin A deficiency.
- Insufficient urinary iodine concentrations were found in 78 percent of mothers and 66 percent of children.

Micronutrient data at the national level for women and their children were collected for the first time in the 2014 CDHS. Micronutrient data collection and analysis were implemented with support and collaboration from UNICEF; the Institut de Recherche pour le Développement; the International Life Science Institute; World Vision; the World Food Programme; the Cambodian Fisheries Administration of the Ministry of Agriculture, Forestry and Fisheries; and ICF International. Data on micronutrients were collected in a subsample of one in every six clusters selected for the main survey (102 of the overall 611 clusters). All children in the selected households who were born after January 2009 and their mothers were eligible. It was estimated that this sampling design would allow collection of biological samples for approximately 1,000 mothers and 1,000 children under age 5. The samples collected from the women and children were a venous blood sample, a spot urine sample, and a stool sample. In order not to disrupt the CDHS survey procedures, collection of specimens was planned as a follow-on activity in which households were revisited after the DHS team had left the clusters. Due to logistic complications, the micronutrient teams were able to revisit the clusters only about one to three months after the DHS team had left. During the DHS data collection, all households in the eligible clusters were asked for consent to be revisited for the micronutrient survey. Informed consent was obtained from women and from the parent or adult responsible for the children before the collection of micronutrient samples.

Blood samples were tested for iron status (plasma ferritin and plasma soluble transferrin receptor concentrations), vitamin A status (plasma retinol-binding protein concentration), vitamin D status (plasma 25-hydroxy vitamin D3 concentration), calcium status (plasma calcium concentration), vitamin B9 (plasma folate concentration) and vitamin B12 (plasma vitamin B12 concentration), and type of hemoglobin. Urine samples were tested for iodine status (urinary iodine concentration). Stool samples were tested for intestinal parasites (egg counts).

Iron status and vitamin A status were analyzed at the VitMin laboratory in Germany using the sandwich ELISA technique. Vitamin D, calcium, vitamin B9, and vitamin B12 were analyzed at the Institut Pasteur in Phnom Penh, Cambodia, using electrochemiluminescence immunoassay with a Cobas system. Urinary iodine concentrations were analyzed at the laboratory of the Mahidol University Institute of Nutrition in Bangkok, Thailand, using spectrophotometry. Stool samples were analyzed for intestinal parasite eggs at the National Centre for Malaria and Parasitology in Phnom Penh Cambodia, using the Flotac technique.

17.1 COVERAGE OF MICRONUTRIENT TESTING

The survey identified 1,048 mothers and 1,358 children who were eligible for the micronutrient study. However, about one in four mothers and their children (27 percent and 24 percent, respectively) refused to participate in the micronutrient survey (Table 17.1). Refusal rates were considerably higher in urban areas than in rural areas for both mothers and children.

Table 17.1 Coverage of micronutrient testing by residence

Among mothers age 15-49 who have at least one child born since January 2009 and children born since January 2009, percent distribution by biological specimen testing status (unweighted), according to residence, Cambodia 2014

	Mothers age 15-49			Children under age 5		
Specimen	Urban	Rural	Total	Urban	Rural	Total
**Stool**						
Tested with results	52.5	64.6	61.6	52.5	63.6	60.8
Tested, no results	8.5	12.0	11.2	13.3	16.1	15.4
**Blood[1]**						
Tested with results	57.5	73.8	69.8	48.7	61.0	58.0
Tested, no results	3.5	2.9	3.1	17.1	18.7	18.3
**Blood[2]**						
Tested with results	57.9	73.9	69.9	49.3	62.6	59.3
Tested, no results	3.1	2.8	2.9	16.5	17.1	16.9
**Urinary iodine**						
Tested with results	58.3	74.1	70.2	59.3	73.5	70.0
Tested, no results	3.1	1.8	2.1	5.3	5.6	5.5
**Not tested**						
Refused	39.0	22.8	26.8	34.2	20.0	23.6
Absent/missing	0.0	0.5	0.4	0.0	0.3	0.2
Total	100.0	100.0	100.0	100.0	100.0	100.0
Number	259	789	1,048	339	1,019	1,358

[1] Tests for vitamins and calcium
[2] Tests for ferritin and soluble transferrin receptors

Moreover, the quantity and/or quality of the specimens were not always sufficient for the laboratory analyses, especially in the case of children. For example, 15 percent of children's stool specimens and 17-18 percent of their blood specimens were not testable or were tested without valid results (Table 17.1). Thus, the analyses are based on only about 60-70 percent of eligible respondents.

17.2 IRON, HEMOGLOBIN, AND PARASITIC INFECTIONS

It has generally been assumed that, in Cambodia, the majority of anemia is associated with insufficient iron intake. However, preliminary data suggested that, in the Cambodian context, factors other than iron deficiency might play an important role in the pathogenesis of anemia (Karakochuk et al., 2015; George et al., 2012), prompting an in-depth assessment of iron status as well as other micronutrients. Iron deficiency can be assessed via serum ferritin concentrations (indicating iron stores) and soluble transferrin receptor (sTfR) concentrations (indicating tissue iron needs). A serum ferritin concentration of less than 15 µg/L in women and less than 12 µg/L in children is a highly specific indicator of iron deficiency. Normal sTfR concentrations are 8.3 mg/L or below, and an sTfR above 8.3 mg/L indicates tissue iron deficiency.

17.2.1 Anemia and Iron Status in Mothers

The data indicate that more than 4 in 10 mothers are anemic (Table 17.2). Iron deficiency was relatively rare among mothers (Table 17.2), with only 3 percent having no iron storage (ferritin concentrations <15 μg/L). In contrast, 34 percent of women showed tissue iron deficiency, as indicated by elevated sTfR concentrations (>8.3 mg/L). Caution is needed in interpreting this indicator, however, as the cutoff level is currently being discussed among experts and has not yet been formally established. Also, other factors such as hemoglobin disorders (hemoglobinopathy) might influence this indicator.

Table 17.2 Anemia, iron status, and soluble transferrin receptors among mothers

Among mothers aged 15-49 years who have at least one child born since January 2009, percentage with anemia, no iron storage (low ferritin) and tissue iron deficiency (high soluble transferrin receptor [sTfR] concentration), according to urban-rural residence, Cambodia 2014

	Urban	Rural	Total
**Hemoglobin level**			
Anemia[1]	46.23	43.50	43.89
Number of mothers	71	415	485
Ferritin <15 mg/L	3.61	2.40	2.55
sTfR >8.3 mg/L	31.57	34.23	33.89
Number of mothers	96	642	738

[1]All pregnant mothers with hemoglobin <11.0 gram per deciliter (g/dl) and all nonpregnant mothers with hemoglobin <12.0 g/dl, after adjustments for altitude and for smoking status, if known, using formulas in CDC (1998), are classified as anemic. The hemoglobin level was measured in two-thirds of the households in each cluster during the main DHS data collection. This is why the number of women in the table is lower for hemoglobin level.

Hemoglobin is the oxygen-transporting protein in red blood cells. Hemoglobin A1 is the normal form of hemoglobin. A person who has an HbA1 level above 95 percent is considered to have normal hemoglobin. However, many genetic disorders induce different types of hemoglobin. Some of these disorders, such as hemoglobin E (HbE), result in a structurally different hemoglobin protein, leading to shorter red blood cell life spans and lower hemoglobin concentrations. Other disorders, such as α- and β-thalassemia, are caused by deletion of part of the protein and result in lower production of hemoglobin. A combination of different disorders, such as HbE and β-thalassemia, is also possible and even quite common in Cambodia. HbE can be present in one of the two genes (heterozygote HbE) or in both genes (homozygote HbE). Heterozygote HbE is expected when HbE levels are between 20 percent and 30 percent of total Hb. Homozygote HbE is expected when levels are above 80 percent of total Hb.

The results showed that 41 percent of mothers had HbA1 levels above 95 percent, indicating that they had normal hemoglobin, and 28 percent had heterozygote HbE (Table 17.3). Another 6 percent had homozygote HbE. Other forms of hemoglobin were found among 23percent of mothers.

Table 17.3 Type of hemoglobin among mothers by residence

Among mothers age 15-49 who have at least one child born since January 2009, percent distribution by type of hemoglobin, according to residence, Cambodia 2014

Type of hemoglobin	Urban	Rural	Total
Normal hemoglobin[1]	46.6	39.6	40.5
Heterozygote hemoglobin E[2]	25.5	28.4	28.0
Homozygote hemoglobin E[3]	7.9	5.7	6.0
Other forms of hemoglobin[4]	19.3	23.2	22.7
Missing	0.8	3.1	2.8
Total	100.0	100.0	100.0
Number	96	643	739

[1] Hemoglobin A1 >95 percent
[2] Hemoglobin E between 20 percent and 30 percent
[3] Hemoglobin E >80 percent
[4] Any other forms of hemoglobin spectrum

17.2.2 Anemia and Iron Status in Children

The overall prevalence of iron deficiency among children (3 percent) is about the same as among mothers (Table 17.4). The prevalence of iron deficiency is very low relative to the high prevalence of anemia (53 percent) among children. Again, as for mothers, the prevalence of tissue iron deficiency was much higher, with nearly half (48 percent) of the children affected. However, the same caution as

mentioned above concerning use of the sTfR cutoff in this population is needed when interpreting these data. The prevalence of children with no iron stores (a ferritin concentration <12 μg/L) was highly dependent on age, with the prevalence being higher among children age 6-11 months (9 percent) and age 12-23 months (12 percent) than among children age 2 and older (1 percent) (Table 17.5). Tissue iron deficiency (as indicated by an sTfR >8.3 mg/L) showed a similar pattern, with a higher prevalence of deficiency among children less than age 2 than among older children.

Table 17.4 Anemia, iron status, and soluble transferrin receptors (sTfRs) among children born since January 2009

Percentage of children born since 2009 according to their anemia status (hemoglobin concentration <11.0 g/dl) and iron status (ferritin concentration <15 μg/L; soluble transferrin receptor [sTfR] concentration <8.3 mg/L), by residence, Cambodia 2014

	Urban	Rural	Total
**Hemoglobin level**			
Anemia[1]	42.2	55.1	53.4
Number of children	87	573	659
Ferritin <12 μg/L	5.0	3.1	3.3
sTfR >8.3 mg/L	44.0	47.9	47.5
Number of children	88	705	793

[1] All children with hemoglobin levels below 11.0 g/dl (after adjustment for altitude using formulas in CDC, 1998) are classified as anemic. Hemoglobin levels were tested in two-thirds of the households in each cluster during the main DHS data collection.

Table 17.5 Iron status among children by age

Among children born since 2009, percentage with no iron storage (low ferritin) and tissue iron deficiency (high sTfR) according to age group, Cambodia 2014

	6-11 months	12-23 months	24-59 months	60+ months	Total
Ferritin <12 μg/L	8.6	12.3	1.3	1.2	3.3
sTfR >8.3 mg/L	59.4	60.3	45.7	39.1	47.5
Number of children	53	110	491	139	793

Hemoglobin patterns among children were more or less similar to those among mothers, with the majority of children (32 percent) having normal hemoglobin (HbA1), 24 percent having heterozygote HbE, and approximately 3 percent having homozygote HbE (Table 17.6). Another 23 percent of children have other forms of hemoglobinopathy. Being a carrier of the HbE gene is usually strongly associated with anemia.

Table 17.6 Type of hemoglobin among children born since January 2009 by residence

Among children born since January 2009, percent distribution by type of hemoglobin, according to residence, Cambodia 2014

Type of hemoglobin	Urban	Rural	Total
Normal hemoglobin[1]	31.8	32.0	32.0
Heterozygote hemoglobin[2]	27.6	23.6	24.1
Homozygote hemoglobin[3]	3.7	2.8	2.9
Other hemoglobin[4]	15.6	24.3	23.3
Missing	21.2	17.2	17.7
Total	100.0	100.0	100.0
Number of children	88	705	793

[1] Hemoglobin A1 >95 percent
[2] Hemoglobin E between 20 percent and 30 percent
[3] Hemoglobin E >80 percent
[4] Any other forms of hemoglobin spectrum

17.2.3 Intestinal Parasite Infection

The data showed that nearly 1 in 5 mothers (19 percent) were infected with at least one intestinal parasite, approximately twice the proportion found among children (10 percent). By far the most prevalent intestinal parasite infection was hookworm, present in 15 percent of mothers and 7 percent of children (Table 17.7). The prevalence of hookworm infection among both mothers and children was much higher in rural areas than in urban areas.

Table 17.7 Intestinal parasitic infection in women and children

Among mothers age 15-49 who have at least one child born since 2009 and all children born since 2009, percentage with various intestinal parasitic infections, according to residence, Cambodia 2014

Type of intestinal parasitic infection	Mothers age 15-49			Children born since 2009		
	Urban	Rural	Total	Urban	Rural	Total
**Any infection**	**9.6**	**19.7**	**18.5**	**2.3**	**11.4**	**10.4**
Ascaris	0.0	0.1	0.1	1.6	0.8	0.9
Trichuris	0.0	0.3	0.2	0.0	0.1	0.1
Hookworm	6.8	16.6	15.4	1.8	7.8	7.1
Enterobius	0.0	0.9	0.8	0.2	2.3	2.1
Taenia	0.0	0.0	0.0	0.0	0.2	0.2
Hymenolepis nana	1.8	1.2	1.3	0.0	0.8	0.7
Other	1.0	1.1	1.0	0.0	0.9	0.8
Number	77	555	632	93	719	811

17.3 VITAMIN AND CALCIUM DEFICIENCY

17.3.1 Vitamin and Calcium Deficiency among Mothers

Serum retinol is commonly used as an indicator of vitamin A status. Retinol is transported in a one-to-one complex with retinol-binding protein (RBP). Studies have shown a high correlation between concentrations of RBP and concentrations of retinol. Marginal vitamin A status is assumed when retinol or RBP concentrations are below 1.05 µmol/L, and vitamin A deficiency is assumed when retinol or RBP concentrations are below 0.70 µmol/L.

Table 17.8 shows that 9 percent of mothers were classified as having marginal vitamin A status, while 3 percent had vitamin A deficiency. The prevalence of vitamin B12 deficiency (<150 pmol/L) and calcium deficiency (<1.15 mmol/L) was low, with approximately 1 percent of women affected by each deficiency. In contrast, almost 1 in 5 women had folic acid (also known as vitamin B9) deficiency. Folic acid deficiency in pregnancy is linked to neural tube defects and cleft palate. The prevalence of vitamin D deficiency was also high among women, depending on the cutoff used. Overall, 31 percent and 60 percent of women had vitamin D levels less than 50 nmol/L and 70 nmol/L, respectively.

Table 17.8 Blood level of vitamins A, B12, B9, and D and calcium in mothers

Among mothers age 15-49 who have at least one child born since 2009, percentage with deficiencies of vitamin A, vitamin B12, vitamin B9, vitamin D, and calcium, according to residence, Cambodia 2014

	Urban	Rural	Total
**Vitamin A deficiency**			
Deficient (RBP <0.70 µmol/L)	4.6	3.0	3.2
Marginal (RBP <1.05 µmol/L)	9.8	8.6	8.7
**Vitamin B12 deficiency**			
Deficient (<150 pmol/L)	1.7	1.0	1.1
**Vitamin B9 deficiency**			
Deficient (<10 nmol/L)	19.1	19.2	19.2
**Vitamin D deficiency**			
<50 nmol/L	26.6	31.6	30.9
<70 nmol/L	51.5	61.1	59.9
**Calcium deficiency**			
<1.15 mmol/L	0.3	0.9	0.8
<0.90 mmol/L	0.0	0.2	0.2
Number of mothers	96	643	739

Note: Blood level of RBP is measured in micromoles per liter (µmol/L), vitamin B12 in picomoles per liter (pmol/L), vitamin B9 and vitamin D in nanomoles per liter (nmol/L), and calcium in millimoles per liter (mmol/L).

17.3.2 Vitamin and Calcium Deficiency among Children

Among children, the overall prevalence of vitamin A deficiency (RBP <0.70 µmol/L) is 9 percent, just below the threshold for a major public health problem (Table 17.9.1). Moreover, the high prevalence of children with marginal vitamin A status (29 percent) is a significant public health concern. Similar to

mothers, the prevalence of vitamin B12 and calcium deficiency was low (2 percent and 1 percent, respectively). In contrast, 8 percent of children suffer from folic acid (vitamin B9) deficiency. The prevalence of folic acid deficiency among children is higher in urban areas (18 percent) than rural areas (7 percent). The prevalence of vitamin D deficiency is also high, with 15 percent of children having a vitamin D level below 50 nmol/L and 33 percent having a level below 70 nmol/L.

Table 17.9.1 Blood level of vitamins A, B12, B9, and D and calcium in children

Among children born since January 2009, percentage with deficiencies of vitamin A, vitamin B12, vitamin B9, vitamin D, and calcium, according to residence, Cambodia 2014

	Urban	Rural	Total
**Vitamin A deficiency**			
Deficient (RBP <0.70 μmol/L)	13.7	8.7	9.2
Marginal (RBP <1.05 μmol/L)	39.9	27.9	29.2
**Vitamin B12 deficiency**			
Deficient (<150 pmol/L)	0.8	1.8	1.7
**Vitamin B9 deficiency**			
Deficient (<10 nmol/L)	17.5	6.8	8.0
**Vitamin D deficiency**			
<50 nmol/L	17.9	15.0	15.3
<70 nmol/L	36.7	32.7	33.1
**Calcium deficiency**			
<1.15 mmol/L	0.4	0.6	0.6
<0.90 mmol/L	0.4	0.5	0.5
Number of children	87	688	775

Note: Blood level of vitamin A is measured in micromoles per liter (μmol/L), vitamin B12 in picomoles per liter (pmol/L), vitamin B9 and vitamin D in nanomoles per liter (nmol/L), and calcium in millimoles per liter (mmol/L).

Table 17.9.2 presents levels of deficiency for different vitamins among children by age. The prevalence of vitamin B12 deficiency and calcium deficiency is substantially higher among children age 6-11 months than among children age 12 months and older. In contrast, the proportion of children with vitamin D deficiency increases with age (Table 17.9.2). The proportions of children who suffer from vitamin A and folic acid deficiency vary only slightly by age.

Table 17.9.2 Blood level of vitamins A, B12, B9, and D and calcium in children by age

Among children born since January 2009, percentage with deficiencies of vitamin A, vitamin B12, vitamin B9, vitamin D, and calcium, according to age group, Cambodia 2014

	6-11 months	12-23 months	24-59 months	60+ months	Total
**Vitamin A deficiency**					
Deficient (RBP <0.70 μmol/L)	11.1	8.8	9.3	8.7	9.2
Marginal (RBP <1.05 μmol/L)	33.8	26.1	27.7	35.5	29.2
**Vitamin B12 deficiency**					
Deficient (<150 pmol/L)	11.4	1.0	1.3	0.3	1.7
**Vitamin B9 deficiency**					
Deficient (<10 nmol/L)	8.4	4.3	7.8	11.3	8.0
**Vitamin D deficiency**					
<50 nmol/L	9.1	12.9	14.8	21.2	15.3
<70 nmol/L	9.1	27.4	34.9	39.5	33.1
**Calcium deficiency**					
<1.15 mmol/L	4.2	1.1	0.3	0.0	0.6
<0.90 mmol/L	4.2	0.4	0.3	0.0	0.5
Number of children	46	105	486	138	775

Note: Blood level of vitamin A is measured in micromoles per liter (μmol/L), vitamin B12 in picomoles per liter (pmol/L), vitamin B9 and vitamin D in nanomoles per liter (nmol/L), and calcium in millimoles per liter (mmol/L).

17.4 URINE IODINE CONCENTRATION

Urinary iodine concentration (UIC) is the prime indicator of nutritional iodine status and is used to evaluate population-based iodine supplementation. A UIC of less than 100 µg/L indicates insufficient iodine in urine, and a value below 50 µg/L indicates a severe insufficiency. A UIC of more than 300 µg/L is considered excessive. The data show high levels of insufficiency among both mothers and children, with 78 percent of mothers and 66 percent of children having a urinary iodine concentration below 100 µg/L (Table 17.10). Severely insufficient iodine concentrations (<50 µg/L) are more prevalent among children and mothers who live in rural areas than among those who live in urban areas.

Excess iodine concentrations were found in 4 percent of children and 1 percent of mothers.

Table 17.10 Urinary iodine excretion in mothers and children by residence

Percent distribution of mothers age 15-49 and children born since January 2009 by urinary iodine concentration, according to residence, Cambodia 2014

	Mothers age 15-49			Children born since January 2009		
Urinary iodine[1]	Urban	Rural	Total	Urban	Rural	Total
<50 µg/L	27.8	44.8	42.7	19.2	40.8	38.2
50-99 µg/L	36.7	35.2	35.3	24.8	28.4	28.0
100-299 µg/L	34.7	18.6	20.5	51.2	27.0	29.9
≥300 µg/L	0.9	1.5	1.4	4.7	3.9	4.0
Total	100.0	100.0	100.0	100.0	100.0	100.0
Number	90	648	737	114	840	953

[1] Iodine status was assessed via UIC as recommended by WHO. Targets differ according to age (WHO-VMNIS, 2013).

HIV/AIDS-RELATED KNOWLEDGE, ATTITUDES, AND BEHAVIOR 18

Key Findings

- Knowledge of HIV/AIDS in Cambodia is universal; almost all women and men age 15-49 have heard of AIDS.
- Overall, 39 percent of women and 48 percent of men age 15-49 have comprehensive knowledge about HIV/AIDS.
- Women are more aware than men that HIV can be transmitted through breastfeeding and that this risk can be reduced by taking special drugs (60 percent versus 51 percent).
- Women age 15-49 are less likely to have multiple sexual partners than their male counterparts (less than 1 percent versus 3 percent).
- Eighteen percent of women and 5 percent of men age 18-24 reported having sexual intercourse before age 18.
- Among never-married youth age 15-24, only 1 percent of young women and 7 percent of young men reported that they had sexual intercourse in the past 12 months.
- Only two-thirds (66 percent) of young men age 15-24 who had sexual intercourse in the past 12 months reported using a condom during their last sexual encounter.

This chapter presents current levels of HIV/AIDS knowledge, attitudes, and related behaviors for the general adult population. The chapter then focuses on HIV/AIDS knowledge and patterns of sexual activity among young people. The findings in this chapter will assist the AIDS control program in Cambodia to identify particular groups of people most in need of information and services and most vulnerable to the risk of HIV infection.

18.1 Knowledge of HIV/AIDS and of Transmission and Prevention Methods

18.1.1 Awareness of AIDS

Ninety-eight percent of women and men age 15-49 have heard of AIDS (Table 18.1), almost identical to the 99 percent found in 2010. Knowledge of AIDS exceeds 95 percent among women and men in all age groups, in all marital status categories, and by urban and rural residence. However, only 94 percent of women and 93 percent of men with no schooling have heard of AIDS. Women in Mondul Kiri/Ratanak Kiri (75 percent) and men in Preah Vihear/Stung Treng (83 percent) are least likely to be aware of AIDS.

Table 18.1 Knowledge of AIDS

Percentage of women and men age 15-49 who have heard of AIDS, by background characteristics, Cambodia 2014

Background characteristic	Women		Men	
	Has heard of AIDS	Number of respondents	Has heard of AIDS	Number of respondents
**Age**				
15-24	97.9	5,910	96.8	1,760
15-19	97.0	2,893	95.2	926
20-24	98.8	3,017	98.5	835
25-29	98.5	2,836	99.0	815
30-39	98.6	4,886	98.6	1,463
40-49	97.5	3,947	98.8	1,152
**Marital status**				
Never married	97.8	4,428	96.8	1,663
Ever had sex	99.1	56	99.6	303
Never had sex	97.7	4,372	96.2	1,360
Married/living together	98.2	11,898	98.7	3,405
Divorced/separated/ widowed	97.9	1,252	99.1	122
**Residence**				
Urban	99.6	3,251	99.4	869
Rural	97.7	14,327	97.8	4,321
**Province**				
Banteay Meanchey	99.5	689	100.0	192
Kampong Cham	95.6	2,021	97.9	663
Kampong Chhnang	100.0	662	100.0	182
Kampong Speu	99.9	1,196	99.6	323
Kampong Thom	100.0	851	100.0	232
Kandal	99.0	1,330	96.9	413
Kratie	99.3	488	97.0	143
Phnom Penh	99.9	1,994	99.8	550
Prey Veng	99.7	1,188	98.6	342
Pursat	99.7	631	99.5	184
Siem Reap	97.6	1,137	98.3	337
Svay Rieng	99.4	654	100.0	183
Takeo	98.9	1,082	98.0	334
Otdar Meanchey	89.0	294	99.4	99
Battambang/Pailin	99.2	1,333	99.6	405
Kampot/Kep	99.8	770	97.4	241
Preah Sihanouk/ Koh Kong	99.2	422	100.0	120
Preah Vihear/ Stung Treng	92.7	462	83.3	112
Mondul Kiri/ Ratanak Kiri	74.7	372	85.3	134
**Education**				
No education	93.7	2,250	92.5	324
Primary	98.1	8,281	97.1	2,167
Secondary and higher	99.5	7,047	99.6	2,699
**Wealth quintile**				
Lowest	95.8	3,143	93.7	901
Second	96.6	3,314	97.7	954
Middle	98.6	3,381	98.9	1,040
Fourth	99.2	3,612	99.5	1,124
Highest	99.6	4,128	99.7	1,171
Total	98.1	17,578	98.1	5,190

18.1.2 HIV Prevention Methods

HIV/AIDS prevention programs focus their messages and efforts on two important aspects of behavior: limiting the number of sexual partners or staying faithful to one partner and use of condoms. To ascertain whether programs have effectively communicated these messages, the 2014 CDHS prompted respondents with specific questions about HIV/AIDS prevention methods (limiting sexual intercourse to one uninfected faithful sexual partner and using condoms).

Table 18.2 presents knowledge of these HIV/AIDS prevention methods among women and men age 15-49, by background characteristics. Eighty-five percent of women and 93 percent of men are aware that the chances of contracting the AIDS virus can be reduced by limiting sex to one uninfected partner who has no other partners; women (84 percent) and men (90 percent) are somewhat less likely to know that using condoms can prevent transmission of the AIDS virus. Overall, 77 percent of women and 87 percent of men have knowledge of both HIV prevention methods. These levels are slightly higher than those found in the 2010 CDHS (75 percent of women and 80 percent of men).

Table 18.2 Knowledge of HIV prevention methods

Percentage of women and men age 15-49 who, in response to prompted questions, say that people can reduce the risk of getting the AIDS virus by using condoms every time they have sexual intercourse and by having one sex partner who is not infected and has no other partners, by background characteristics, Cambodia 2014

	Women				Men			
Background characteristic	Using condoms[1]	Limiting sexual intercourse to one uninfected partner[2]	Using condoms and limiting sexual intercourse to one uninfected partner[1,2]	Number of women	Using condoms[1]	Limiting sexual intercourse to one uninfected partner[2]	Using condoms and limiting sexual intercourse to one uninfected partner[1,2]	Number of men
**Age**								
15-24	83.2	84.5	76.2	5,910	88.1	90.6	84.1	1,760
15-19	80.1	81.0	72.4	2,893	86.0	88.0	82.0	926
20-24	86.2	87.9	79.9	3,017	90.3	93.5	86.5	835
25-29	86.6	87.5	80.4	2,836	93.2	96.8	91.4	815
30-39	85.2	86.4	78.6	4,886	90.9	94.3	88.0	1,463
40-49	80.6	82.5	73.7	3,947	88.9	94.3	86.3	1,152
**Marital status**								
Never married	81.9	83.1	75.6	4,428	88.9	90.7	85.0	1,663
Ever had sex	87.2	85.7	80.8	56	94.9	95.0	90.6	303
Never had sex	81.8	83.1	75.6	4,372	87.6	89.8	83.8	1,360
Married/living together	84.7	85.9	77.8	11,898	90.2	94.8	87.7	3,405
Divorced/separated/ widowed	81.3	84.3	74.0	1,252	92.8	93.4	89.6	122
**Residence**								
Urban	90.4	91.1	85.7	3,251	96.8	97.4	95.2	869
Rural	82.2	83.7	75.0	14,327	88.5	92.6	85.2	4,321
**Province**								
Banteay Meanchey	56.9	40.1	33.3	689	85.7	87.7	79.2	192
Kampong Cham	74.5	77.9	66.4	2,021	93.7	89.6	87.6	663
Kampong Chhnang	88.8	99.0	88.6	662	93.6	98.4	92.5	182
Kampong Speu	80.6	85.9	73.3	1,196	86.7	90.7	80.2	323
Kampong Thom	96.3	93.7	90.5	851	96.7	99.6	96.5	232
Kandal	92.1	93.3	87.3	1,330	83.2	95.6	82.8	413
Kratie	84.4	87.1	76.9	488	75.4	83.7	68.3	143
Phnom Penh	95.7	97.0	93.7	1,994	98.7	99.0	98.4	550
Prey Veng	84.4	91.6	81.5	1,188	94.9	97.3	93.7	342
Pursat	73.7	71.4	62.0	631	87.6	96.9	87.6	184
Siem Reap	79.8	80.6	70.0	1,137	68.9	89.5	63.8	337
Svay Rieng	81.0	90.8	76.5	654	87.2	93.0	84.0	183
Takeo	84.9	91.0	81.6	1,082	93.1	89.3	85.8	334
Otdar Meanchey	69.7	70.3	62.4	294	97.5	98.9	96.9	99
Battambang/Pailin	91.2	87.8	83.5	1,333	97.5	99.6	97.5	405
Kampot/Kep	93.4	90.5	86.9	770	90.1	94.3	88.3	241
Preah Sihanouk/ Koh Kong	86.1	83.6	77.2	422	98.2	97.1	95.4	120
Preah Vihear/ Stung Treng	74.8	68.1	61.1	462	81.1	79.9	77.7	112
Mondul Kiri/ Ratanak Kiri	61.7	66.5	57.4	372	76.6	80.8	74.1	134
**Education**								
No education	70.9	74.6	63.1	2,250	70.1	84.8	66.6	324
Primary	82.7	83.0	74.8	8,281	87.1	90.3	82.6	2,167
Secondary and higher	89.1	90.8	84.0	7,047	94.5	97.0	92.7	2,699
**Wealth quintile**								
Lowest	76.8	78.2	68.4	3,143	78.2	87.6	74.8	901
Second	79.5	82.2	72.3	3,314	86.6	90.7	82.2	954
Middle	82.2	83.6	74.5	3,381	93.3	94.4	89.7	1,040
Fourth	86.3	86.5	79.3	3,612	93.0	94.8	89.7	1,124
Highest	91.3	92.5	87.4	4,128	95.5	98.0	94.6	1,171
Total	83.7	85.1	77.0	17,578	89.9	93.4	86.9	5,190

[1] Using condoms every time they have sexual intercourse
[2] Partner who has no other partners

Knowledge of both HIV prevention methods is higher among women age 20-29 (80 percent) than among younger or older women. Men age 25-29 are somewhat more likely to have knowledge about prevention of HIV/AIDS than men in other age groups. There is little variation in knowledge of the two HIV prevention methods by marital status, although knowledge is slightly higher among women and men who have never been married but have had sex (81 percent and 91 percent, respectively) and among formerly married men (90 percent).

Knowledge of HIV prevention methods is higher among respondents in urban than rural areas, and there is considerable variability across provinces. Among women, knowledge of the two HIV prevention methods is highest in Phnom Penh (94 percent) and lowest in Banteay Meanchey (33 percent). Among men, knowledge of the two methods is highest in Kampong Thom, Phnom Penh, Otdar Meanchey, Battambang/Pailin, and Preah Sihanouk/Koh Kong (95 percent or higher) and lowest in Siem Reap (64 percent).

Level of educational attainment strongly relates to a respondent’s knowledge of HIV prevention methods. Women and men with higher levels of schooling are more likely than those with less schooling to be aware of various preventive methods. The data also show that men and women in the higher wealth quintiles are more likely than those in the lower quintiles to be aware of ways to prevent transmission of the HIV virus.

18.1.3 Knowledge about Transmission

The 2014 CDHS included questions on common misconceptions about AIDS and HIV transmission. Respondents were asked whether they think it is possible for a healthy-looking person to have the AIDS virus and whether a person can contract AIDS from mosquito bites, by supernatural means, or by sharing food with a person who has AIDS.

The results in Tables 18.3.1 and 18.3.2 indicate that many Cambodian adults lack accurate knowledge about the ways in which the AIDS virus can and cannot be transmitted. Particularly critical is the fact that only 62 percent of women and 66 percent of men know that a healthy-looking person can have (and thus transmit) the virus that causes AIDS. Many women and men also erroneously believe that AIDS can be transmitted by mosquito bites; only 70 percent of women and 73 percent of men reject this common misconception. Larger proportions of women and men are aware that the AIDS virus cannot be transmitted by supernatural means (89 percent and 93 percent, respectively) or by sharing food with a person who has AIDS (88 percent and 90 percent, respectively). Overall, only about half of women (46 percent) and men (51 percent) are able to reject two of the more common misconceptions about AIDS—that AIDS can be transmitted by mosquito bites and that a person can become infected with the AIDS virus by sharing food with someone who is infected—and know that a healthy-looking person can have the AIDS virus.

Table 18.3.1 Comprehensive knowledge about AIDS: Women

Percentage of women age 15-49 who say that a healthy-looking person can have the AIDS virus and who, in response to prompted questions, correctly reject local misconceptions about transmission or prevention of the AIDS virus, and the percentage with comprehensive knowledge about AIDS, by background characteristics, Cambodia 2014

Background characteristic	Percentage of respondents who say that:				Percentage who say that a healthy-looking person can have the AIDS virus and who reject the two most common local misconceptions[1]	Percentage with comprehensive knowledge about AIDS[2]	Number of women
	A healthy-looking person can have the AIDS virus	The AIDS virus cannot be transmitted by mosquito bites	The AIDS virus cannot be transmitted by supernatural means	A person cannot become infected by sharing food with a person who has AIDS			
**Age**							
15-24	61.6	69.0	89.0	84.1	44.1	37.6	5,910
15-19	57.0	66.1	86.0	80.3	39.6	32.7	2,893
20-24	66.0	71.8	91.8	87.7	48.4	42.4	3,017
25-29	65.3	77.5	91.0	92.0	53.2	45.9	2,836
30-39	64.4	71.7	91.2	91.3	48.6	41.7	4,886
40-49	58.4	62.7	86.1	85.3	39.6	33.0	3,947
**Marital status**							
Never married	62.0	72.0	89.4	85.1	46.9	40.0	4,428
Ever had sex	65.7	70.5	89.0	85.9	47.2	42.5	56
Never had sex	61.9	72.1	89.5	85.1	46.9	40.0	4,372
Married/living together	62.4	69.0	89.5	88.7	45.5	38.8	11,898
Divorced/separated/ widowed	61.7	67.8	86.4	86.9	45.1	38.0	1,252
**Residence**							
Urban	79.0	83.1	95.1	93.4	66.2	59.2	3,251
Rural	58.4	66.7	88.0	86.3	41.2	34.5	14,327
**Province**							
Banteay Meanchey	52.1	64.4	86.7	87.5	36.4	8.9	689
Kampong Cham	58.3	61.9	89.1	85.1	39.2	26.9	2,021
Kampong Chhnang	49.3	96.9	98.8	99.0	46.7	44.7	662
Kampong Speu	46.2	63.5	85.1	85.4	30.0	25.5	1,196
Kampong Thom	71.5	91.9	98.8	97.1	64.8	59.3	851
Kandal	54.3	70.8	93.0	87.7	38.7	36.4	1,330
Kratie	73.5	56.0	82.4	81.6	44.1	39.3	488
Phnom Penh	90.6	83.0	96.3	92.3	74.5	71.2	1,994
Prey Veng	48.3	70.1	91.2	88.3	34.1	29.0	1,188
Pursat	57.1	77.9	90.8	93.1	44.3	29.7	631
Siem Reap	51.9	61.5	85.9	87.4	36.7	29.8	1,137
Svay Rieng	45.7	61.3	90.2	87.0	25.5	21.4	654
Takeo	79.2	75.9	92.4	90.8	63.1	54.5	1,082
Otdar Meanchey	33.3	64.2	69.1	73.2	28.6	24.8	294
Battambang/Pailin	81.2	69.2	87.7	89.4	58.1	52.9	1,333
Kampot/Kep	72.0	60.3	91.9	89.6	44.1	40.9	770
Preah Sihanouk/ Koh Kong	53.2	71.1	93.4	91.3	42.3	36.2	422
Preah Vihear/ Stung Treng	46.0	42.5	64.1	62.0	25.0	20.7	462
Mondul Kiri/ Ratanak Kiri	47.1	51.0	61.9	59.8	35.4	32.1	372
**Education**							
No education	47.3	49.8	75.3	74.8	27.3	21.6	2,250
Primary	59.0	64.3	87.9	86.2	39.7	32.9	8,281
Secondary and higher	70.9	82.4	95.4	93.4	58.8	51.9	7,047
**Wealth quintile**							
Lowest	51.3	56.3	79.7	79.3	32.1	25.8	3,143
Second	54.8	62.1	86.2	84.6	35.5	28.1	3,314
Middle	57.5	67.1	89.4	86.8	39.9	32.5	3,381
Fourth	65.3	75.8	93.1	92.0	50.7	43.7	3,612
Highest	77.8	82.8	95.5	93.3	65.1	59.3	4,128
Total	62.2	69.7	89.3	87.6	45.8	39.1	17,578

[1] Two most common local misconceptions: the AIDS virus can be transmitted by mosquito bites and a person can become infected by sharing food with a person who has AIDS.
[2] Comprehensive knowledge means knowing that consistent use of condoms during sexual intercourse and having just one uninfected faithful partner can reduce the chance of getting the AIDS virus, knowing that a healthy-looking person can have the AIDS virus, and rejecting the two most common local misconceptions about AIDS transmission or prevention.

Table 18.3.2 Comprehensive knowledge about AIDS: Men

Percentage of men age 15-49 who say that a healthy-looking person can have the AIDS virus and who, in response to prompted questions, correctly reject local misconceptions about transmission or prevention of the AIDS virus, and the percentage with comprehensive knowledge about AIDS, by background characteristics, Cambodia 2014

Background characteristic	Percentage of respondents who say that:				Percentage who say that a healthy-looking person can have the AIDS virus and who reject the two most common local misconceptions[1]	Percentage with comprehensive knowledge about AIDS[2]	Number of men
	A healthy-looking person can have the AIDS virus	The AIDS virus cannot be transmitted by mosquito bites	The AIDS virus cannot be transmitted by supernatural means	A person cannot become infected by sharing food with a person who has AIDS			
**Age**							
15-24	63.2	72.0	90.7	85.7	48.8	45.9	1,760
15-19	59.1	70.2	87.4	81.9	45.2	42.4	926
20-24	67.7	74.1	94.4	90.0	52.9	49.9	835
25-29	70.0	80.4	95.3	94.1	57.5	55.4	815
30-39	67.0	74.3	93.7	92.6	52.5	50.2	1,463
40-49	65.9	67.4	91.7	89.6	47.9	44.9	1,152
**Marital status**							
Never married	64.4	74.4	91.3	86.5	51.7	49.2	1,663
Ever had sex	79.2	79.4	96.3	90.2	66.8	64.5	303
Never had sex	61.1	73.3	90.1	85.7	48.4	45.8	1,360
Married/living together	66.3	72.4	93.0	91.6	50.6	47.9	3,405
Divorced/separated/ widowed	76.5	70.4	95.5	86.4	54.8	52.7	122
**Residence**							
Urban	80.6	87.9	97.0	93.9	72.3	70.2	869
Rural	63.0	70.0	91.6	89.0	46.8	44.0	4,321
**Province**							
Banteay Meanchey	66.3	60.9	92.9	83.1	41.3	36.8	192
Kampong Cham	74.0	58.8	89.6	87.8	48.2	47.2	663
Kampong Chhnang	81.3	68.1	97.3	83.9	55.2	52.3	182
Kampong Speu	66.7	69.8	86.4	87.1	47.7	43.7	323
Kampong Thom	85.0	97.9	98.0	99.3	83.4	81.4	232
Kandal	6.7	67.6	90.9	85.7	4.0	3.2	413
Kratie	48.6	63.8	82.1	84.2	33.4	29.3	143
Phnom Penh	91.2	90.0	98.0	95.0	82.0	81.3	550
Prey Veng	93.1	69.8	97.3	95.4	67.6	66.6	342
Pursat	80.9	80.1	96.0	91.9	61.7	54.6	184
Siem Reap	63.7	55.8	91.8	85.6	38.4	27.9	337
Svay Rieng	64.2	61.0	89.0	88.7	47.7	45.2	183
Takeo	72.2	76.0	92.1	90.3	57.2	54.6	334
Otdar Meanchey	3.5	80.3	95.6	96.2	3.2	2.5	99
Battambang/Pailin	73.7	88.5	99.3	96.7	67.3	66.4	405
Kampot/Kep	50.4	84.7	94.2	96.2	44.4	39.6	241
Preah Sihanouk/ Koh Kong	76.3	82.8	98.3	96.1	64.3	63.2	120
Preah Vihear/ Stung Treng	22.7	70.2	74.4	73.1	18.6	18.3	112
Mondul Kiri/ Ratanak Kiri	47.4	58.1	73.5	71.1	39.7	38.0	134
**Education**							
No education	50.4	40.9	74.8	75.5	24.2	21.9	324
Primary	58.9	62.4	88.8	85.2	39.3	36.7	2,167
Secondary and higher	73.4	85.3	97.6	95.3	63.7	61.0	2,699
**Wealth quintile**							
Lowest	53.0	56.8	84.2	81.3	33.7	30.4	901
Second	63.5	66.8	90.3	86.2	45.7	42.4	954
Middle	64.5	70.8	93.9	89.7	47.4	45.6	1,040
Fourth	67.4	77.3	94.4	93.6	53.6	50.5	1,124
Highest	77.8	88.2	97.7	95.8	69.4	67.6	1,171
Total	65.9	73.0	92.5	89.8	51.0	48.4	5,190

[1] Two most common local misconceptions: the AIDS virus can be transmitted by mosquito bites and a person can become infected by sharing food with a person who has AIDS.
[2] Comprehensive knowledge means knowing that consistent use of condoms during sexual intercourse and having just one uninfected faithful partner can reduce the chance of getting the AIDS virus, knowing that a healthy-looking person can have the AIDS virus, and rejecting the two most common local misconceptions about AIDS transmission or prevention.

Tables 18.3.1 and 18.3.2 also provide an assessment of the level of comprehensive knowledge of HIV/AIDS prevention and transmission. People are considered to have comprehensive knowledge about AIDS when they know that both condom use and limiting sex partners to one uninfected person are HIV/AIDS prevention methods, they are aware that a healthy-looking person can have HIV, and they reject the two most common local misconceptions. In Cambodia, 39 percent of women and 48 percent of men have comprehensive knowledge of HIV/AIDS prevention and transmission, very close to the figures reported in 2010 (41 percent and 44 percent, respectively).

Tables 18.3.1 and 18.3.2 show that there is considerable variation in HIV/AIDS knowledge by background characteristics. Sexually active, never-married respondents tend to be slightly more knowledgeable than those in other marital status categories. For all indicators, the proportion of women and men with correct knowledge about HIV/AIDS prevention and transmission is much higher in urban than rural areas and among women and men with higher levels of schooling. Similarly, men and women in the higher wealth quintiles are more likely than those in the lower quintiles to have comprehensive knowledge about HIV/AIDS. Variations in knowledge levels by province are marked among both women and men, with the highest levels of comprehensive knowledge about AIDS observed among women and men from Kampong Thom (59 percent and 81 percent, respectively) and Phnom Penh (71 percent and 81 percent, respectively).

18.1.4 Knowledge of Mother-to-Child Transmission

Educating people about the ways in which HIV can be transmitted from mother to child during pregnancy, delivery, and breastfeeding is critical to reducing mother-to-child transmission (MTCT) of HIV. To obtain information on these issues, respondents were asked whether the virus that causes AIDS can be transmitted from a mother to a child during pregnancy, delivery, or breastfeeding and whether a mother who is infected with HIV can reduce the risk of transmission of the virus to the baby by taking certain drugs (antiretrovirals) during pregnancy (see Table 18.4).

Although 86 percent of women and 84 percent of men know that HIV can be transmitted by breastfeeding, only 62 percent of women and 54 percent of men know that the risk of MTCT can be reduced through the use of certain drugs during pregnancy. Sixty percent of women and 51 percent of men are aware of both aspects of MTCT. This represents an increase from the figures reported in the 2010 CDHS.

MTCT knowledge is slightly higher among women and men age 25-29 and those who are currently in a union. There is considerable variation by education and wealth among both women and men, with MTCT knowledge increasing as education and wealth increase. Among women, MTCT knowledge does not differ markedly by pregnancy status or by urban-rural residence. However, male urban residents have higher levels of knowledge about mother-to-child transmission than their rural counterparts. Most respondents know that HIV can be transmitted by breastfeeding; lack of knowledge about antiretrovirals accounts for most of the variation by background characteristics. More than 4 of 5 women living in Kampong Thom and Prey Veng have comprehensive knowledge of MTCT. Kampong Thom also has the highest percentage of men with comprehensive MTCT knowledge (91 percent).

Particularly notable is the comparatively low level of knowledge among pregnant women; just 3 in 5 pregnant women are aware that HIV can be transmitted from mother to child during breastfeeding and that mother-to-child transmission can be reduced by taking certain drugs during pregnancy. This indicates incomplete coverage of MTCT counseling during prenatal care visits in Cambodia.

Table 18.4 Knowledge of prevention of mother-to-child transmission of HIV

Percentage of women and men age 15-49 who know that HIV can be transmitted from mother to child by breastfeeding and that the risk of mother-to-child transmission (MTCT) of HIV can be reduced by the mother taking special drugs during pregnancy, by background characteristics, Cambodia 2014

	Women				Men			
Background characteristic	HIV can be transmitted by breastfeeding	Risk of MTCT can be reduced by mother taking special drugs during pregnancy	HIV can be transmitted by breastfeeding and risk of MTCT can be reduced by mother taking special drugs during pregnancy	Number of women	HIV can be transmitted by breastfeeding	Risk of MTCT can be reduced by mother taking special drugs during pregnancy	HIV can be transmitted by breastfeeding and risk of MTCT can be reduced by mother taking special drugs during pregnancy	Number of men
**Age**								
15-24	83.3	56.9	53.5	5,910	80.2	51.7	47.9	1,760
15-19	81.0	51.1	47.4	2,893	78.0	48.8	45.7	926
20-24	85.5	62.5	59.3	3,017	82.7	55.0	50.5	835
25-29	87.0	67.1	64.1	2,836	84.1	58.3	54.1	815
30-39	88.2	65.7	63.4	4,886	86.4	55.2	51.8	1,463
40-49	86.2	63.2	60.6	3,947	86.3	54.4	51.6	1,152
**Marital status**								
Never married	81.5	54.5	50.5	4,428	81.4	52.2	48.5	1,663
Ever had sex	82.2	56.3	50.6	56	84.3	52.5	47.4	303
Never had sex	81.5	54.5	50.5	4,372	80.7	52.1	48.7	1,360
Married/living together	87.6	65.4	62.9	11,898	85.3	55.6	52.2	3,405
Divorced/separated/ widowed	85.3	61.6	59.7	1,252	79.0	49.7	44.2	122
**Currently pregnant**								
Pregnant	85.3	63.9	60.4	934	na	na	na	na
Not pregnant or not sure	86.0	62.3	59.5	16,644	na	na	na	na
**Residence**								
Urban	85.7	64.8	60.2	3,251	87.8	61.0	58.3	869
Rural	86.0	61.8	59.4	14,327	83.2	53.0	49.3	4,321
**Province**								
Banteay Meanchey	88.5	63.1	61.1	689	84.8	46.2	43.9	192
Kampong Cham	86.0	54.9	52.7	2,021	82.1	62.4	56.3	663
Kampong Chhnang	95.7	77.8	76.2	662	93.1	65.5	65.0	182
Kampong Speu	90.9	58.9	56.4	1,196	77.3	29.2	25.1	323
Kampong Thom	97.8	87.4	87.2	851	99.0	91.2	90.7	232
Kandal	84.1	63.5	59.8	1,330	66.3	45.6	40.1	413
Kratie	93.8	53.1	51.1	488	72.7	20.5	18.4	143
Phnom Penh	84.1	61.5	55.8	1,994	91.4	62.6	61.0	550
Prey Veng	93.1	85.3	84.7	1,188	91.3	61.9	58.4	342
Pursat	89.6	62.3	60.8	631	91.8	32.3	31.5	184
Siem Reap	86.9	57.5	53.8	1,137	74.2	47.1	42.7	337
Svay Rieng	91.2	70.5	69.0	654	83.8	24.5	21.6	183
Takeo	74.6	62.6	61.2	1,082	81.6	40.7	35.8	334
Otdar Meanchey	76.5	50.8	49.0	294	97.5	74.9	74.1	99
Battambang/Pailin	81.6	55.6	49.8	1,333	89.4	64.8	61.7	405
Kampot/Kep	87.7	65.4	61.7	770	93.7	78.2	76.5	241
Preah Sihanouk/ Koh Kong	83.2	58.7	55.8	422	85.3	46.8	42.4	120
Preah Vihear/ Stung Treng	71.7	33.6	30.9	462	62.7	67.4	52.7	112
Mondul Kiri/ Ratanak Kiri	57.1	35.3	34.0	372	78.2	47.3	47.3	134
**Education**								
No education	80.6	53.8	52.8	2,250	79.2	46.4	45.0	324
Primary	87.0	62.1	59.9	8,281	80.9	49.9	46.5	2,167
Secondary and higher	86.3	65.4	61.3	7,047	86.9	58.9	55.0	2,699
**Wealth quintile**								
Lowest	85.4	56.1	54.5	3,143	79.1	47.1	43.9	901
Second	85.1	61.2	59.2	3,314	85.2	50.1	48.4	954
Middle	86.7	61.8	59.5	3,381	86.5	54.1	49.8	1,040
Fourth	86.6	64.8	61.6	3,612	79.9	55.2	49.8	1,124
Highest	85.8	66.6	61.9	4,128	88.2	62.8	60.0	1,171
Total	85.9	62.4	59.6	17,578	83.9	54.3	50.8	5,190

na = Not applicable

18.2 STIGMA ASSOCIATED WITH AIDS AND ATTITUDES RELATED TO HIV/AIDS

Knowledge and beliefs about HIV infection affect how people treat those they know to be living with HIV or AIDS. In the 2014 CDHS, a number of questions were posed to respondents to measure their attitudes towards HIV-infected people, including questions about their willingness to buy vegetables from an infected shopkeeper, to let others know the HIV status of family members, and to take care of relatives who have the AIDS virus in their own household. They were also asked whether an HIV-positive female teacher who is not sick should be allowed to continue teaching. Tables 18.5.1 and 18.5.2 show the percentages of women and men who have heard of HIV/AIDS and who express positive attitudes towards people with HIV, by background characteristics.

Table 18.5.1 Accepting attitudes toward those living with HIV/AIDS: Women

Among women age 15-49 who have heard of AIDS, percentage expressing specific accepting attitudes toward people with HIV/AIDS, by background characteristics, Cambodia 2014

	Percentage of respondents who:					
Background characteristic	Are willing to care for a family member with AIDS in the respondent's home	Would buy fresh vegetables from a shopkeeper who has the AIDS virus	Say that a female teacher who has the AIDS virus but is not sick should be allowed to continue teaching	Would not want to keep secret that a family member got infected with the AIDS virus	Percentage expressing accepting attitudes on all four indicators	Number of respondents who have heard of AIDS
**Age**						
15-24	89.1	76.5	88.7	40.9	25.8	5,786
15-19	87.2	70.7	85.7	38.7	22.1	2,806
20-24	90.9	82.0	91.4	42.9	29.4	2,979
25-29	91.0	88.0	94.3	44.4	33.0	2,794
30-39	89.4	82.7	91.8	47.6	33.6	4,816
40-49	86.8	71.5	85.8	51.8	28.5	3,847
**Marital status**						
Never married	88.9	77.3	89.7	39.1	24.9	4,328
Ever had sex	90.0	83.9	94.3	42.8	32.1	55
Never had sex	88.9	77.2	89.7	39.1	24.8	4,273
Married/living together	89.1	79.7	90.1	48.1	31.6	11,689
Divorced/separated/ widowed	87.8	77.9	87.5	46.9	28.8	1,226
**Residence**						
Urban	93.1	88.9	95.0	37.4	29.8	3,238
Rural	88.0	76.7	88.6	47.7	29.7	14,005
**Province**						
Banteay Meanchey	72.4	76.8	82.4	36.7	16.7	686
Kampong Cham	90.6	72.1	91.3	54.2	35.3	1,933
Kampong Chhnang	88.6	95.1	98.3	36.4	26.7	662
Kampong Speu	95.2	80.0	92.2	44.2	32.5	1,194
Kampong Thom	98.8	83.6	93.9	31.3	20.1	851
Kandal	92.5	74.2	89.7	50.4	35.9	1,317
Kratie	82.9	62.2	76.3	51.8	27.4	485
Phnom Penh	94.4	89.0	95.1	32.4	26.7	1,992
Prey Veng	64.0	82.2	93.6	67.6	27.7	1,184
Pursat	89.6	86.8	92.5	30.5	20.1	629
Siem Reap	80.1	68.6	87.0	61.4	32.3	1,110
Svay Rieng	97.6	69.0	88.1	26.7	13.2	651
Takeo	93.1	86.7	90.4	38.8	30.8	1,070
Otdar Meanchey	90.2	69.5	77.0	29.8	13.3	262
Battambang/Pailin	97.8	86.5	93.0	57.2	48.0	1,323
Kampot/Kep	86.5	77.1	87.1	66.1	46.8	768
Preah Sihanouk/ Koh Kong	96.5	91.7	96.0	38.9	33.0	419
Preah Vihear/ Stung Treng	77.2	41.1	56.7	45.1	11.6	428
Mondul Kiri/ Ratanak Kiri	92.9	77.9	76.8	12.8	5.6	278
**Education**						
No education	84.4	64.6	80.5	48.1	23.6	2,108
Primary	88.2	76.0	87.8	48.1	29.4	8,122
Secondary and higher	91.3	86.9	94.9	42.4	32.0	7,013
**Wealth quintile**						
Lowest	85.2	66.0	82.3	46.6	22.4	3,010
Second	86.7	73.9	87.9	47.0	27.5	3,202
Middle	88.0	78.3	89.9	49.7	31.9	3,334
Fourth	90.8	83.2	92.3	47.9	34.2	3,585
Highest	92.8	89.4	94.5	39.1	31.2	4,112
Total	89.0	79.0	89.8	45.8	29.8	17,243

The large majority of women and men age 15-49 (90 percent each) say that an HIV-positive female teacher should be allowed to continue teaching. Comparatively fewer (79 percent of women and 82 percent of men) would buy fresh food from a shopkeeper with the AIDS virus. Although 89 percent of women and 95 percent of men say they would be willing to care for a family member with the AIDS virus in their home, only 46 percent of women and 49 percent of men would not want to keep secret that a family member has HIV. Overall, 3 in 10 women (30 percent) and nearly 4 in 10 men (37 percent) express accepting attitudes on all four indicators, approximately the same figures reported in 2010 (34 percent of women and 35 percent of men).

Table 18.5.2 Accepting attitudes toward those living with HIV/AIDS: Men

Among men age 15-49 who have heard of HIV/AIDS, percentage expressing specific accepting attitudes toward people with HIV/AIDS, by background characteristics, Cambodia 2014

Background characteristic	Percentage of respondents who:				Percentage expressing accepting attitudes on all four indicators	Number of respondents who have heard of AIDS
	Are willing to care for a family member with AIDS in the respondent's home	Would buy fresh vegetables from a shopkeeper who has the AIDS virus	Say that a female teacher who has the AIDS virus but is not sick should be allowed to continue teaching	Would not want to keep secret that a family member got infected with the AIDS virus		
**Age**						
15-24	94.6	78.1	88.9	40.6	28.9	1,703
15-19	92.4	73.8	86.6	42.0	27.3	881
20-24	96.9	82.7	91.2	39.2	30.7	822
25-29	95.4	90.3	94.2	46.0	39.2	806
30-39	94.8	86.0	92.2	54.6	44.4	1,444
40-49	94.4	76.4	87.7	56.1	38.5	1,138
**Marital status**						
Never married	94.6	80.0	90.1	40.8	30.9	1,610
Ever had sex	94.8	88.9	93.1	39.7	33.2	302
Never had sex	94.5	78.0	89.3	41.1	30.3	1,309
Married/living together	94.8	82.8	90.5	52.6	40.0	3,360
Divorced/separated/ widowed	94.2	81.9	90.4	53.5	37.4	121
**Residence**						
Urban	95.3	93.0	94.5	42.1	34.9	864
Rural	94.6	79.6	89.6	50.3	37.5	4,227
**Province**						
Banteay Meanchey	88.6	77.0	88.1	39.3	29.4	192
Kampong Cham	97.8	65.9	93.2	51.1	32.2	649
Kampong Chhnang	100.0	81.6	84.9	56.0	45.9	182
Kampong Speu	94.4	73.2	83.8	52.2	35.9	322
Kampong Thom	99.4	98.4	99.8	61.3	59.5	232
Kandal	80.0	84.4	85.6	51.9	31.9	400
Kratie	86.7	51.2	71.3	35.6	10.5	139
Phnom Penh	96.1	95.3	96.1	37.6	32.0	548
Prey Veng	99.4	76.4	86.9	44.9	36.1	337
Pursat	97.5	85.5	93.1	48.7	38.8	183
Siem Reap	87.6	85.0	89.6	61.3	40.6	331
Svay Rieng	98.5	71.4	76.6	44.4	33.3	183
Takeo	98.5	88.5	87.9	38.5	32.5	327
Otdar Meanchey	100.0	88.4	95.4	48.4	40.0	99
Battambang/Pailin	94.8	94.8	100.0	71.0	65.7	403
Kampot/Kep	97.9	81.3	94.0	43.2	31.9	235
Preah Sihanouk/ Koh Kong	95.4	90.5	91.8	38.4	31.5	120
Preah Vihear/ Stung Treng	94.4	86.3	86.0	67.6	52.8	93
Mondul Kiri/ Ratanak Kiri	100.0	73.5	92.8	9.3	5.9	114
**Education**						
No education	90.1	65.9	80.0	51.1	27.0	300
Primary	93.9	76.2	87.3	49.3	34.5	2,104
Secondary and higher	95.9	88.1	93.9	48.4	40.2	2,687
**Wealth quintile**						
Lowest	94.2	69.5	84.8	49.3	30.4	844
Second	95.5	80.7	89.4	51.5	38.8	932
Middle	94.5	76.7	89.5	48.6	35.7	1,029
Fourth	93.7	84.0	91.5	51.5	40.3	1,118
Highest	95.7	94.3	94.9	44.4	38.6	1,168
Total	94.7	81.9	90.4	48.9	37.1	5,091

In general, urban residents are more willing to buy fresh vegetables from a shopkeeper who has the AIDS virus than their rural counterparts. However, they are less likely to say that they would not want to keep secret that a family member is HIV positive. Overall, better educated respondents are more likely to express accepting attitudes on all four measures. There is no significant variation in accepting attitudes on all four measures by urban-rural residence, and there is no linear relationship with wealth.

Tables 18.5.1 and 18.5.2 document considerable variation in accepting attitudes by province. Forty-eight percent of women in Battambang/Pailin and 47 percent in Kampot/Kep express accepting attitudes on all four measures, as compared with only 6 percent in Mondul Kiri/Ratanak Kiri. Among men, 66 percent in Battambang/Pailin and 60 percent in Kampong Thom express accepting attitudes on all four measures, compared with only 6 percent in Mondul Kiri/Ratanak Kiri.

18.3 ATTITUDES TOWARDS NEGOTIATING SAFER SEX

Knowledge about HIV transmission and ways to prevent it is useless if people feel powerless to negotiate safer sex practices with their partners. To gauge attitudes towards safer sex, respondents in the 2014 CDHS were asked whether they think a woman is justified in refusing to have sex with her husband if she knows he has sex with other women. They were also asked whether they think that a woman is justified in asking her husband to use a condom if she knows that he has a sexually transmitted infection (STI). The results from these questions are shown in Table 18.6.

Seventy-two percent of women and 70 percent of men believe that a woman is justified in refusing to have sex with her husband if she knows he has sex with other women, and 95 percent of women and 98 percent of men believe that a woman is justified in asking her husband to use a condom if he has an STI.

Although a large majority of respondents in all groups support a woman's right to refuse to have sex with her husband if she knows he has sex with other women, some differences by background characteristics stand out. For example, among both women and men, the percentage who agree with a woman's right to refuse to have sex with her husband is lower in urban areas than in rural areas; this percentage is also lower among those with a secondary education or higher than among those with a primary education or less. The percentage of women who support a woman's right to refuse to have sex with her husband if she knows he has sex with other women ranges from a low of 52 percent in Preah Sihanouk/Koh Kong to a high of 90 percent in Prey Veng. Among men, support for a woman's right to refuse sex when her husband has sex with other women is lowest in Preah Vihear/Stung Treng (6 percent) and highest in Mondul Kiri/Ratanak Kiri (97 percent).

There are only small differences by background characteristics in support for a woman's right to propose using a condom if she knows that her husband has an STI. For example, the percentage of women and men supporting this is somewhat lower in the 15-19 age group than in the other age groups. Also, the higher a respondent's educational attainment and wealth quintile, the more likely he or she is to say that a woman can propose using a condom.

Table 18.6 Attitudes toward negotiating safer sexual relations with husband

Percentage of women and men age 15-49 who believe that a woman is justified in refusing to have sexual intercourse with her husband if she knows that he has sexual intercourse with other women, and percentage who believe that a woman is justified in asking that they use a condom if she knows that her husband has a sexually transmitted infection (STI), by background characteristics, Cambodia 2014

Background characteristic	Women: Refusing to have sexual intercourse with her husband if she knows he has sex with other women	Women: Asking that they use a condom if she knows that her husband has an STI	Number of women	Men: Refusing to have sexual intercourse with her husband if she knows he has sex with other women	Men: Asking that they use a condom if she knows that her husband has an STI	Number of men
**Age**						
15-24	68.2	91.3	5,910	68.2	96.1	1,760
15-19	65.7	86.3	2,893	69.6	94.5	926
20-24	70.7	96.1	3,017	66.7	97.8	835
25-29	73.6	96.9	2,836	68.5	98.1	815
30-39	75.8	97.0	4,886	72.0	98.6	1,463
40-49	73.6	94.5	3,947	71.0	98.5	1,152
**Marital status**						
Never married	63.9	87.9	4,428	66.4	96.0	1,663
Ever had sex	65.2	92.2	56	55.0	98.4	303
Never had sex	63.9	87.9	4,372	69.0	95.5	1,360
Married/living together	75.2	97.1	11,898	71.8	98.5	3,405
Divorced/separated/ widowed	75.9	93.6	1,252	66.1	96.7	122
**Residence**						
Urban	64.7	97.6	3,251	57.1	98.7	869
Rural	74.1	93.8	14,327	72.5	97.4	4,321
**Province**						
Banteay Meanchey	54.9	95.3	689	77.8	99.3	192
Kampong Cham	72.3	93.9	2,021	67.2	97.3	663
Kampong Chhnang	86.8	99.1	662	61.6	99.0	182
Kampong Speu	75.8	93.8	1,196	66.5	92.8	323
Kampong Thom	79.9	97.1	851	93.4	93.0	232
Kandal	79.4	96.1	1,330	80.9	96.8	413
Kratie	76.4	97.0	488	56.4	92.0	143
Phnom Penh	61.1	99.0	1,994	44.0	99.8	550
Prey Veng	89.5	96.4	1,188	52.4	99.7	342
Pursat	72.0	93.7	631	83.1	96.8	184
Siem Reap	66.3	94.3	1,137	88.8	97.3	337
Svay Rieng	72.2	96.8	654	60.7	98.5	183
Takeo	68.3	82.2	1,082	76.0	97.7	334
Otdar Meanchey	78.4	83.2	294	88.3	99.4	99
Battambang/Pailin	71.1	97.3	1,333	91.6	100.0	405
Kampot/Kep	81.4	97.1	770	60.4	99.6	241
Preah Sihanouk/ Koh Kong	52.1	92.7	422	81.2	99.2	120
Preah Vihear/ Stung Treng	74.0	88.2	462	5.7	96.2	112
Mondul Kiri/ Ratanak Kiri	61.5	82.8	372	96.7	98.1	134
**Education**						
No education	72.2	90.3	2,250	73.4	95.6	324
Primary	74.4	95.0	8,281	73.8	96.8	2,167
Secondary and higher	70.1	95.3	7,047	66.4	98.5	2,699
**Wealth quintile**						
Lowest	75.1	91.5	3,143	69.4	94.3	901
Second	76.2	93.4	3,314	72.8	97.0	954
Middle	73.6	94.0	3,381	72.9	98.1	1,040
Fourth	72.6	95.0	3,612	74.4	98.8	1,124
Highest	66.1	97.7	4,128	61.2	99.2	1,171
Total	72.4	94.5	17,578	69.9	97.6	5,190

18.4 Multiple Sexual Partnerships

Given that most HIV infections in Cambodia are contracted through heterosexual contact, information on sexual behavior is important when designing and monitoring intervention programs to control the spread of the epidemic. In the context of HIV/AIDS prevention, limiting the number of sexual partners and encouraging protected sex are crucial to combating the epidemic. The 2014 CDHS included questions on respondents' lifetime sexual partners as well as the partners respondents had in the 12 months preceding the survey. Male respondents were also asked whether they had paid for sex in the 12 months preceding the interview. Information on use of condoms during the last sexual encounter with each of these types of partners was collected from both women and men. Given that questions about sexual activity are

sensitive, it is important to remember that respondents' answers are likely subject to at least some reporting bias when interpreting the results in this section.

Tables 18.7.1 and 18.7.2 show the percentages of women and men age 15-49 who had engaged in sexual intercourse with more than one partner in the past 12 months along with their mean number of lifetime sexual partners. Table 18.7.2 also shows the percentage of men who used a condom during their most recent intercourse (among those with more than one partner in the past 12 months). Because the number of women reporting more than one partner in the past 12 months is very small, condom use among these women is not presented.

Table 18.7.1 Multiple sexual partners: Women

Among all women age 15-49, the percentage who had sexual intercourse with more than one sexual partner in the past 12 months and the mean number of sexual partners during their lifetime for women who ever had sexual intercourse, by background characteristics, Cambodia 2014

	Among all women:		Among women who ever had sexual intercourse[1]:	
Background characteristic	Percentage who had 2+ partners in the past 12 months	Number of women	Mean number of sexual partners in lifetime	Number of women
**Age**				
15-24	0.1	5,910	1.2	2,443
15-19	0.0	2,893	1.0	490
20-24	0.3	3,017	1.2	1,953
25-29	0.1	2,836	1.1	2,411
30-39	0.0	4,886	1.1	4,561
40-49	0.0	3,947	1.2	3,762
**Marital status**				
Never married	0.0	4,428	1.4	48
Married/living together	0.0	11,898	1.1	11,883
Divorced/separated/ widowed	0.4	1,252	1.4	1,246
**Residence**				
Urban	0.2	3,251	1.3	2,095
Rural	0.0	14,327	1.1	11,082
**Province**				
Banteay Meanchey	0.0	689	1.1	541
Kampong Cham	0.0	2,021	1.1	1,624
Kampong Chhnang	0.0	662	1.1	453
Kampong Speu	0.2	1,196	1.1	933
Kampong Thom	0.2	851	1.1	638
Kandal	0.0	1,330	1.1	987
Kratie	0.0	488	1.1	383
Phnom Penh	0.3	1,994	1.5	1,248
Prey Veng	0.0	1,188	1.1	995
Pursat	0.0	631	1.1	454
Siem Reap	0.0	1,137	1.1	858
Svay Rieng	0.0	654	1.1	525
Takeo	0.0	1,082	1.2	781
Otdar Meanchey	0.0	294	1.1	229
Battambang/Pailin	0.0	1,333	1.1	960
Kampot/Kep	0.0	770	1.1	616
Preah Sihanouk/ Koh Kong	0.1	422	1.1	293
Preah Vihear/ Stung Treng	0.0	462	1.1	354
Mondul Kiri/ Ratanak Kiri	0.1	372	1.1	306
**Education**				
No education	0.1	2,250	1.2	2,034
Primary	0.1	8,281	1.2	7,032
Secondary and higher	0.0	7,047	1.1	4,111
**Wealth quintile**				
Lowest	0.0	3,143	1.1	2,555
Second	0.0	3,314	1.1	2,620
Middle	0.0	3,381	1.1	2,601
Fourth	0.0	3,612	1.1	2,645
Highest	0.2	4,128	1.3	2,756
Total	0.1	17,578	1.1	13,177

[1] Means are calculated excluding respondents who gave non-numeric responses.

The data show that almost no women and less than 3 percent of men reportedly had two or more sexual partners during the 12 months preceding the survey. Among men, the proportion with multiple sexual partners increases as age increases, from less than 1 percent among those age 15-19 to 4 percent among those age 40-49. Men who are married and divorced, separated, or widowed and those living in urban areas and in households in the highest wealth quintiles are more likely than other respondents to have had multiple partners over the past year. There is no clear association between educational level and having multiple partners over the past year. The percentage of men who report having had two or more sexual partners in the past 12 months varies according to province. One in 10 men in Kratie and Phnom Penh reported having had multiple partners over the past year.

Among men with two or more partners in the past 12 months, 30 percent report having used a condom during their last encounter. Condom use is more pronounced among urban than rural men (48 percent and 16 percent, respectively).

On average, men report having 3.5 lifetime sexual partners, more than three times the average reported by women (1.1 partners). Among women, there is almost no variation according to background characteristics. Never-married men report 4.7 lifetime sexual partners, as compared with 3.4 among currently married men and 2.8 among formerly married men. The number of sexual partners is also higher among urban than rural men (8.0 versus 2.7). More educated and well-off men report a higher number of sexual partners. Men with no schooling report an average of 1.4 partners, as compared with 4.8 partners among men with a secondary education or higher, and the average number of lifetime partners ranges from 1.9 among men in the lowest wealth quintile to 7.0 in the highest quintile.

Table 18.7.2 Multiple sexual partners: Men

Among all men age 15-49, the percentage who had sexual intercourse with more than one sexual partner in the past 12 months; among those having more than one partner in the past 12 months, the percentage reporting that a condom was used at last intercourse; and the mean number of sexual partners during their lifetime for men who ever had sexual intercourse, by background characteristics, Cambodia 2014

Background characteristic	All men		Among men who had 2+ partners in the past 12 months:		Among men who ever had sexual intercourse[1]:	
	Percentage who had 2+ partners in the past 12 months	Number of men	Percentage who reported using a condom during last sexual intercourse	Number of men	Mean number of sexual partners in lifetime	Number of men
**Age**						
15-24	1.1	1,760	(46.2)	19	2.1	524
15-19	0.2	926	*	2	1.4	69
20-24	2.1	835	(43.4)	18	2.2	455
25-29	2.9	815	*	24	3.0	715
30-39	3.2	1,463	(28.9)	47	3.6	1,438
40-49	4.3	1,152	(18.8)	50	4.4	1,144
**Marital status**						
Never married	1.5	1,663	(84.2)	25	4.7	302
Married/living together	3.1	3,405	14.9	107	3.4	3,400
Divorced/separated/ widowed	6.2	122	*	8	2.8	120
**Residence**						
Urban	7.1	869	47.7	61	8.0	605
Rural	1.8	4,321	16.3	78	2.7	3,216
**Province**						
Banteay Meanchey	1.9	192	*	4	4.1	143
Kampong Cham	2.0	663	*	13	5.4	548
Kampong Chhnang	3.6	182	*	7	4.0	130
Kampong Speu	1.5	323	*	5	1.6	241
Kampong Thom	0.0	232	*	0	1.2	155
Kandal	1.0	413	*	4	1.4	317
Kratie	10.5	143	(24.7)	15	2.0	107
Phnom Penh	10.0	550	(47.5)	55	9.9	376
Prey Veng	4.2	342	*	14	2.6	263
Pursat	0.7	184	*	1	3.2	121
Siem Reap	1.4	337	*	5	1.1	243
Svay Rieng	1.6	183	*	3	3.0	143
Takeo	1.3	334	*	4	2.3	244
Otdar Meanchey	0.0	99	*	0	1.4	69
Battambang/Pailin	0.2	405	*	1	2.9	275
Kampot/Kep	1.7	241	*	4	2.2	187
Preah Sihanouk/ Koh Kong	4.5	120	*	5	6.9	90
Preah Vihear/ Stung Treng	0.0	112	*	0	1.2	78
Mondul Kiri/ Ratanak Kiri	0.0	134	*	0	1.9	91
**Education**						
No education	2.7	324	*	9	1.4	284
Primary	1.9	2,167	(14.4)	41	2.5	1,747
Secondary and higher	3.3	2,699	36.1	90	4.8	1,790
**Wealth quintile**						
Lowest	1.6	901	*	14	1.9	663
Second	0.9	954	*	9	2.3	716
Middle	2.0	1,040	*	20	2.7	778
Fourth	2.4	1,124	*	26	3.1	827
Highest	6.0	1,171	41.5	70	7.0	837
Total	2.7	5,190	30.1	140	3.5	3,821

Note: Figures in parentheses are based on 25-49 unweighted cases. An asterisk indicates that a figure is based on fewer than 25 unweighted cases and has been suppressed.
[1] Means are calculated excluding respondents who gave non-numeric responses.

Male respondents in the 2014 CDHS who had had sex in the past 12 months were asked whether they had paid anyone in exchange for sex in the past 12 months or ever in their lifetime and whether any of their last three partners in the past 12 months was a commercial sex worker.

The results in Table 18.8 show that 10 percent of men have ever paid for sexual intercourse and that 3 percent had done so in the 12 months before the survey. Men age 25-29 (15 percent); men who are divorced, separated, or widowed (18 percent); men living in urban areas (14 percent); and men living in Preah Sihanouk/Koh Kong and Svay Rieng (36 percent and 22 percent, respectively) are most likely to have ever paid for sexual intercourse.

Table 18.8 Payment for sexual intercourse and condom use at last paid sexual intercourse

Percentage of men age 15-49 who ever paid for sexual intercourse and percentage reporting payment for sexual intercourse in the past 12 months, and among them, the percentage reporting that a condom was used the last time they paid for sexual intercourse, by background characteristics, Cambodia 2014

	Among all men:			Among men who paid for sex in the past 12 months:	
Background characteristic	Percentage who ever paid for sexual intercourse	Percentage who paid for sexual intercourse in the past 12 months	Number of men	Percentage reporting condom use at last paid sexual intercourse	Number of men
**Age**					
15-24	4.3	2.0	1,760	91.1	36
15-19	1.7	1.0	926	*	9
20-24	7.1	3.2	835	(93.6)	27
25-29	14.9	3.4	815	(67.8)	28
30-39	11.8	3.2	1,463	90.1	46
40-49	12.9	4.3	1,152	75.2	50
**Marital status**					
Never married	6.4	2.9	1,663	93.4	49
Married/living together	11.4	2.8	3,405	75.5	94
Divorced/separated/ widowed	18.1	12.9	122	(83.4)	16
**Residence**					
Urban	14.3	6.6	869	84.5	57
Rural	9.1	2.4	4,321	80.3	102
**Province**					
Banteay Meanchey	18.6	2.1	192	*	4
Kampong Cham	19.8	2.5	663	*	16
Kampong Chhnang	4.3	4.2	182	*	8
Kampong Speu	1.5	1.2	323	*	4
Kampong Thom	0.6	0.0	232	*	0
Kandal	1.8	1.3	413	*	5
Kratie	15.4	15.3	143	84.6	22
Phnom Penh	9.7	7.6	550	(88.2)	42
Prey Veng	10.3	3.6	342	*	12
Pursat	17.5	2.2	184	*	4
Siem Reap	4.5	3.8	337	*	13
Svay Rieng	21.6	1.8	183	*	3
Takeo	17.2	2.9	334	*	10
Otdar Meanchey	2.4	0.0	99	*	0
Battambang/Pailin	2.3	0.4	405	*	2
Kampot/Kep	2.4	2.4	241	*	6
Preah Sihanouk/ Koh Kong	35.7	4.3	120	*	5
Preah Vihear/ Stung Treng	1.8	1.8	112	*	2
Mondul Kiri/ Ratanak Kiri	9.2	0.7	134	*	1
**Education**					
No education	4.4	1.9	324	*	6
Primary	8.8	2.6	2,167	76.6	57
Secondary and higher	11.6	3.6	2,699	83.8	96
**Wealth quintile**					
Lowest	4.3	2.1	901	*	19
Second	7.2	1.8	954	*	17
Middle	9.9	2.6	1,040	*	27
Fourth	12.7	3.0	1,124	(93.4)	34
Highest	14.1	5.3	1,171	79.0	62
Total 15-54	10.0	3.1	5,190	81.8	159

Note: Figures in parentheses are based on 25-49 unweighted cases. An asterisk indicates that a figure is based on fewer than 25 unweighted cases and has been suppressed.

Men in Kratie (15 percent) are most likely to report having engaged in paid sex in the past 12 months. Men who are divorced, separated, or widowed are more likely than those in other marital status categories to report having recently paid money for sex, with 13 percent having engaged in such a transaction in the past year. Urban men (7 percent), men with a secondary education or higher (4 percent), and the wealthiest men (5 percent) are more likely than their counterparts to report having paid for sex in the past year. Eighty-two percent of men who paid for sex in the past year reported using a condom during their most recent paid sex; due to the small number of cases, differentials between subgroups should be interpreted with caution.

18.5 TESTING FOR HIV

Knowledge of HIV status helps HIV-negative individuals make specific decisions to reduce risk and increase safer sex practices so they can remain disease free. For those who are HIV infected, knowledge of their status allows them to take action to protect their sexual partners, to access treatment, and to plan for the future. Testing of pregnant women is especially important so that action can be taken to prevent mother-to-child transmission.

To obtain information on the prevalence of HIV testing, all respondents were asked whether they had ever been tested for HIV. If they said that they had, they were asked whether they had received the results of their last test. Women giving birth in the two-year period before the survey were asked additional questions regarding testing that may have occurred as part of any antenatal care they received prior to the birth.

Tables 18.9.1 and 18.9.2 show that, among the adult population age 15-49, 42 percent of women and 36 percent of men have been tested for HIV at some time. These figures are substantially higher than those reported in 2010, when only 25 percent of both women and men had ever been tested. Forty-one percent of women and 35 percent of men were tested indicated that they had received the results of their test. Ten percent of women and 9 percent of men said that they been tested and received results during the 12 months prior to the survey.

The proportions of both women and men ever tested were higher among those age 20 and older than among those younger than age 20. Testing levels were highest among currently married women (54 percent), whereas levels were approximately the same among never-married men who had ever had sex (47 percent), currently married men (46 percent), and widowed, divorced, and separated men (48 percent). Unmarried women and men who had never had sex were least likely to have ever been tested for HIV (11 percent and 9 percent, respectively). Urban residents, those with a secondary education or higher, and those in the highest wealth quintile had higher testing levels than their counterparts. Women and men residing in Preah Vihear/Stung Treng and Mondul Kiri/Ratanak Kiri are least likely to have ever been tested for HIV.

Table 18.9.1 Coverage of prior HIV testing: Women

Percentage of women age 15-49 who know where to get an HIV test, percent distribution of women age 15-49 by testing status and by whether they received the results of the last test, the percentage of women ever tested, and the percentage of women age 15-49 who were tested in the past 12 months and received the results of the last test, according to background characteristics, Cambodia 2014

Background characteristic	Percentage who know where to get an HIV test	Percent distribution of women by testing status and by whether they received the results of the last test: Ever tested and received results	Ever tested, did not receive results	Never tested[1]	Total	Percentage ever tested	Percentage who have been tested for HIV in the past 12 months and received the results of the last test	Number of women
**Age**								
15-24	76.4	32.4	1.9	65.7	100.0	34.3	12.1	5,910
15-19	67.2	13.6	1.0	85.3	100.0	14.7	6.7	2,893
20-24	85.2	50.5	2.7	46.8	100.0	53.2	17.3	3,017
25-29	87.7	63.2	1.9	34.9	100.0	65.1	14.8	2,836
30-39	81.0	50.3	2.0	47.6	100.0	52.4	8.2	4,886
40-49	70.0	24.0	0.6	75.4	100.0	24.6	3.2	3,947
**Marital status**								
Never married	70.2	10.8	0.8	88.4	100.0	11.6	3.8	4,428
Ever had sex	81.8	41.8	0.0	58.2	100.0	41.8	24.6	56
Never had sex	70.0	10.4	0.8	88.8	100.0	11.2	3.6	4,372
Married/living together	81.5	51.6	2.0	46.4	100.0	53.6	11.9	11,898
Divorced/separated/ widowed	73.7	39.7	1.0	59.3	100.0	40.7	6.6	1,252
**Residence**								
Urban	90.3	51.4	1.2	47.4	100.0	52.6	12.8	3,251
Rural	75.3	38.0	1.7	60.3	100.0	39.7	8.7	14,327
**Province**								
Banteay Meanchey	80.0	44.9	1.0	54.1	100.0	45.9	12.1	689
Kampong Cham	75.5	37.1	1.2	61.7	100.0	38.3	8.7	2,021
Kampong Chhnang	99.6	42.6	1.9	55.5	100.0	44.5	13.5	662
Kampong Speu	71.1	39.7	3.3	56.9	100.0	43.1	8.3	1,196
Kampong Thom	78.3	42.3	0.3	57.4	100.0	42.6	8.9	851
Kandal	74.8	34.7	2.4	62.9	100.0	37.1	7.7	1,330
Kratie	61.5	24.1	1.8	74.0	100.0	26.0	3.5	488
Phnom Penh	92.3	51.7	1.1	47.1	100.0	52.9	13.6	1,994
Prey Veng	80.4	34.6	0.2	65.2	100.0	34.8	4.4	1,188
Pursat	75.3	43.0	1.2	55.8	100.0	44.2	9.4	631
Siem Reap	77.4	46.4	3.2	50.4	100.0	49.6	10.6	1,137
Svay Rieng	87.6	40.0	4.5	55.4	100.0	44.6	11.3	654
Takeo	72.3	41.3	1.6	57.1	100.0	42.9	9.2	1,082
Otdar Meanchey	60.5	31.3	1.2	67.5	100.0	32.5	9.1	294
Battambang/Pailin	89.7	54.0	0.8	45.2	100.0	54.8	12.0	1,333
Kampot/Kep	72.4	33.5	1.7	64.8	100.0	35.2	6.6	770
Preah Sihanouk/ Koh Kong	88.8	50.2	1.9	47.9	100.0	52.1	16.3	422
Preah Vihear/ Stung Treng	48.2	16.6	1.4	82.0	100.0	18.0	4.9	462
Mondul Kiri/ Ratanak Kiri	37.3	14.1	0.4	85.6	100.0	14.4	4.1	372
**Education**								
No education	59.1	29.8	1.6	68.6	100.0	31.4	6.3	2,250
Primary	76.8	40.6	1.8	57.5	100.0	42.5	8.5	8,281
Secondary and higher	85.6	43.7	1.4	54.8	100.0	45.2	11.6	7,047
**Wealth quintile**								
Lowest	65.0	32.9	1.5	65.5	100.0	34.5	7.0	3,143
Second	72.0	34.5	2.0	63.5	100.0	36.5	9.0	3,314
Middle	77.3	38.1	1.9	60.0	100.0	40.0	8.2	3,381
Fourth	80.8	40.6	1.8	57.6	100.0	42.4	9.5	3,612
Highest	91.2	52.9	1.1	46.0	100.0	54.0	12.7	4,128
Total	78.1	40.5	1.6	57.9	100.0	42.1	9.5	17,578

[1] Includes “don’t know/missing”

Table 18.9.2 Coverage of prior HIV testing: Men

Percentage of men age 15-49 who know where to get an HIV test, percent distribution of men age 15-49 by testing status and by whether they received the results of the last test, the percentage of men ever tested, and the percentage of men age 15-49 who were tested in the past 12 months and received the results of the last test, according to background characteristics, Cambodia 2014

Background characteristic	Percentage who know where to get an HIV test	Percent distribution of men by testing status and by whether they received the results of the last test: Ever tested and received results	Ever tested, did not receive results	Never tested[1]	Total	Percentage ever tested	Percentage who have been tested for HIV in the past 12 months and received the results of the last test	Number of men
**Age**								
15-24	70.8	18.1	0.8	81.1	100.0	18.9	6.6	1,760
15-19	62.4	6.7	0.8	92.5	100.0	7.5	2.9	926
20-24	80.0	30.8	0.8	68.5	100.0	31.5	10.7	835
25-29	86.7	56.9	0.4	42.6	100.0	57.4	17.3	815
30-39	80.5	48.5	1.6	49.9	100.0	50.1	8.2	1,463
40-49	73.1	29.9	1.0	69.1	100.0	30.9	6.4	1,152
**Marital status**								
Never married	70.4	15.0	1.0	84.0	100.0	16.0	5.4	1,663
Ever had sex	88.2	45.0	2.2	52.8	100.0	47.2	17.7	303
Never had sex	66.4	8.4	0.7	90.9	100.0	9.1	2.6	1,360
Married/living together	79.3	44.9	1.1	54.0	100.0	46.0	10.2	3,405
Divorced/separated/ widowed	82.3	47.8	0.0	52.2	100.0	47.8	12.4	122
**Residence**								
Urban	90.8	52.7	1.0	46.3	100.0	53.7	12.4	869
Rural	73.7	31.9	1.0	67.1	100.0	32.9	7.9	4,321
**Province**								
Banteay Meanchey	78.6	39.4	0.0	60.6	100.0	39.4	5.2	192
Kampong Cham	57.9	31.8	2.1	66.1	100.0	33.9	13.0	663
Kampong Chhnang	97.5	41.6	1.7	56.8	100.0	43.2	12.1	182
Kampong Speu	59.5	33.3	1.5	65.2	100.0	34.8	6.8	323
Kampong Thom	70.8	36.9	0.0	63.1	100.0	36.9	1.8	232
Kandal	89.8	36.5	0.3	63.2	100.0	36.8	12.3	413
Kratie	67.4	28.7	0.4	70.8	100.0	29.2	6.4	143
Phnom Penh	92.4	55.6	0.9	43.5	100.0	56.5	11.7	550
Prey Veng	80.2	28.8	0.9	70.3	100.0	29.7	9.0	342
Pursat	75.5	33.7	0.9	65.4	100.0	34.6	5.6	184
Siem Reap	85.2	27.4	1.0	71.6	100.0	28.4	5.1	337
Svay Rieng	73.3	38.3	0.0	61.7	100.0	38.3	9.7	183
Takeo	79.6	34.0	2.2	63.8	100.0	36.2	10.5	334
Otdar Meanchey	58.0	32.0	0.6	67.4	100.0	32.6	5.3	99
Battambang/Pailin	88.9	40.0	1.2	58.8	100.0	41.2	8.8	405
Kampot/Kep	64.0	22.3	0.0	77.7	100.0	22.3	3.3	241
Preah Sihanouk/ Koh Kong	84.8	40.7	2.9	56.4	100.0	43.6	13.9	120
Preah Vihear/ Stung Treng	71.2	18.8	0.3	80.9	100.0	19.1	1.5	112
Mondul Kiri/ Ratanak Kiri	55.3	22.0	0.0	78.0	100.0	22.0	2.5	134
**Education**								
No education	53.0	17.1	0.2	82.7	100.0	17.3	3.1	324
Primary	68.4	29.9	1.1	68.9	100.0	31.1	6.9	2,167
Secondary and higher	85.9	42.0	1.0	57.0	100.0	43.0	10.8	2,699
**Wealth quintile**								
Lowest	59.8	22.4	1.1	76.5	100.0	23.5	5.4	901
Second	68.6	26.4	1.0	72.6	100.0	27.4	4.9	954
Middle	73.2	29.2	0.6	70.2	100.0	29.8	7.2	1,040
Fourth	83.7	39.8	1.2	59.0	100.0	41.0	10.3	1,124
Highest	92.0	54.0	1.2	44.9	100.0	55.1	14.1	1,171
Total	76.5	35.4	1.0	63.6	100.0	36.4	8.7	5,190

[1] Includes "don't know/missing"

More than three-quarters of women and men in Cambodia know where to get an HIV test. Knowledge about where to get an HIV test is more common among women and men in urban areas than rural areas. It is also higher among educated women and men and among those living in richer households.

Table 18.10 presents data on HIV/AIDS information and counseling during antenatal care. Among women who had given birth in the two years before the survey, 57 percent received information and counseling about HIV/AIDS during antenatal care for their most recent birth. Sixty-two percent of women who had given birth in the past two years reported that they were tested for HIV during antenatal care and

received the test results and post-test counseling; 8 percent were tested and received the test results but not post-test counseling; and 3 percent were tested but received neither the results nor post-test counseling. Taking these elements into account, the 2014 CDHS results indicate that 51 percent of women giving birth during the two-year period prior to the survey were counseled about HIV, were tested for HIV, and received the test results.

Table 18.10 Pregnant women counseled and tested for HIV

Among all women age 15-49 who gave birth in the two years preceding the survey, the percentage who received HIV pretest counseling, the percentage who received an HIV test during antenatal care for their most recent birth by whether they received their results and post-test counseling, and the percentage who received an HIV test during ANC or labor for their most recent birth by whether they received their test results, according to background characteristics, Cambodia 2014

Background characteristic	Percentage who received counseling on HIV during antenatal care[1]	Percentage who were tested for HIV during antenatal care and who: Received results and received post-test counseling	Received results and did not receive post-test counseling	Did not receive results	Percentage who received counseling on HIV and an HIV test during ANC, and the results	Percentage who had an HIV test during ANC or labor and who:[2] Received results	Did not receive results	Number of women who gave birth in the past two years[3]
**Age**								
15-24	54.1	60.7	9.7	3.3	47.7	72.0	3.9	1,099
15-19	53.4	50.1	9.4	4.2	43.0	61.8	4.2	180
20-24	54.3	62.7	9.7	3.1	48.6	74.0	3.8	919
25-29	63.8	67.5	6.3	2.5	57.1	75.9	2.8	885
30-39	54.8	60.7	7.6	3.3	48.4	70.9	3.4	879
40-49	48.5	50.7	4.3	1.2	41.6	56.3	1.7	82
**Marital status**								
Married/living together	56.7	62.4	8.0	3.1	50.1	72.4	3.4	2,831
Divorced/separated/ widowed	67.0	63.6	5.1	0.8	61.1	71.1	2.5	114
**Residence**								
Urban	61.4	70.7	8.8	1.5	56.3	83.0	3.3	414
Rural	56.4	61.1	7.7	3.2	49.6	70.7	3.3	2,531
**Province**								
Banteay Meanchey	62.7	62.1	20.7	4.1	56.5	82.8	4.1	120
Kampong Cham	45.6	53.6	7.7	3.1	40.8	64.5	3.1	418
Kampong Chhnang	90.8	93.0	0.0	0.8	87.3	93.0	2.0	111
Kampong Speu	49.2	54.2	15.4	5.6	43.7	73.0	5.8	182
Kampong Thom	89.2	87.4	0.4	0.0	85.4	89.4	0.0	141
Kandal	56.3	54.8	3.8	3.7	43.5	60.7	3.7	193
Kratie	37.6	35.2	2.2	3.7	28.8	38.7	4.4	107
Phnom Penh	58.3	66.2	7.0	1.4	51.6	79.4	3.6	257
Prey Veng	74.6	67.0	0.0	0.9	59.5	69.1	0.9	194
Pursat	82.8	79.4	1.3	3.3	73.7	82.1	3.3	122
Siem Reap	57.6	76.6	9.9	8.2	52.5	86.6	8.2	182
Svay Rieng	67.3	70.7	5.5	8.9	58.5	77.1	8.9	108
Takeo	72.8	74.9	2.5	2.6	68.4	78.5	2.6	164
Otdar Meanchey	61.7	63.7	1.1	3.0	53.5	66.2	3.0	54
Battambang/Pailin	34.9	58.3	28.0	1.2	33.4	88.9	1.2	247
Kampot/Kep	43.4	64.8	9.0	0.8	38.9	74.7	2.5	116
Preah Sihanouk/ Koh Kong	70.0	75.7	6.5	3.7	65.6	83.3	4.2	61
Preah Vihear/ Stung Treng	27.6	27.9	2.3	1.2	23.6	30.1	1.2	92
Mondul Kiri/ Ratanak Kiri	25.3	13.3	3.5	0.8	14.4	17.1	1.5	75
**Education**								
No education	42.6	47.6	7.2	3.2	37.2	55.7	3.2	366
Primary	56.5	60.2	7.2	3.2	48.8	69.1	3.5	1,491
Secondary and higher	62.8	70.5	9.1	2.6	57.5	82.5	3.2	1,088
**Wealth quintile**								
Lowest	52.7	55.8	6.1	3.5	46.0	62.8	3.6	694
Second	60.0	61.9	6.6	4.0	51.6	69.9	4.5	589
Middle	59.2	64.0	8.4	2.9	53.5	74.5	3.0	565
Fourth	56.9	63.8	10.9	2.6	51.6	76.2	3.1	536
Highest	57.5	68.3	7.9	1.7	51.0	81.1	2.5	560
Total	57.1	62.4	7.9	3.0	50.5	72.4	3.3	2,944

[1] In this context, "pretest counseling" means that someone talked with the respondent about all three of the following topics: (1) babies getting the AIDS virus from their mother, (2) preventing the virus, and (3) getting tested for the virus.
[2] Women were asked whether they received an HIV test during labor only if they were not tested for HIV during ANC.
[3] The denominator for percentages includes women who did not receive antenatal care for their last birth in the past two years.

Women living in urban areas were more likely than those living in rural areas to have received counseling, testing, and results during antenatal care. According to province, pregnant women living in Mondul Kiri/Ratanak Kiri (14 percent) and Preah Vihear/Stung Treng (24 percent) were least likely to have received HIV/AIDS counseling and testing services. Women with a secondary education or higher are more likely than those with no education to receive full counseling and testing services during antenatal care.

Some women are tested for HIV after labor starts. Including such women, 72 percent were tested for HIV and received results at some time during pregnancy.

18.6 REPORTS OF RECENT SEXUALLY TRANSMITTED INFECTIONS

Information about the incidence of sexually transmitted infections is useful not only as a marker of unprotected sexual intercourse but also as a cofactor for HIV transmission. The 2014 CDHS asked respondents who had ever had sex whether they had had an STI in the past 12 months. They were also asked whether, in the past year, they had experienced a genital sore or ulcer and whether they had any genital discharge. These symptoms have been shown useful in identifying STIs in men. They are less easily interpreted in women because women are likely to experience more non-STI conditions of the reproductive tract that produce a discharge.

Table 18.11 shows the self-reported prevalence of STIs and STI symptoms among women and men age 15-49 who have ever had sexual intercourse. Six percent of women and less than 1 percent of men who have ever had sex reported having had an STI in the 12 months before the survey. A higher proportion of women (10 percent) than men (1 percent) reported having had an abnormal genital discharge. Furthermore, 4 percent of women and 1 percent of men reported having had a genital sore or ulcer in the past 12 months. Overall, 12 percent of women and 2 percent of men had either an STI or symptoms of an STI in the 12 months preceding the survey.

The results presented in Table 18.11 indicate that the proportion of respondents who reported having had an STI or an STI symptom varied considerably across provinces. Among women, the self-reported prevalence of STIs and STI symptoms ranged from a low of 5 percent in Kampong Thom and Otdar Meanchey to a high of 22 percent in Preah Vihear/Stung Treng and 20 percent in Battambang/Pailin and Preah Sihanouk/Koh Kong. Among men, the prevalence of reported STIs or symptoms of STIs is highest in Svay Rieng (4 percent) and Kandal (5 percent). Differences in the prevalence of STIs or their symptoms by other background characteristics are not large.

Table 18.11 Self-reported prevalence of sexually transmitted infections (STIs) and STI symptoms

Among women and men age 15-49 who ever had sexual intercourse, the percentage reporting having an STI and/or symptoms of an STI in the past 12 months, by background characteristics, Cambodia 2014

Background characteristic	Percentage of women who reported having in the past 12 months: STI	Bad smelling/ abnormal genital discharge	Genital sore/ulcer	STI/genital discharge/ sore or ulcer	Number of women who ever had sexual intercourse	Percentage of men who reported having in the past 12 months: STI	Bad smelling/ abnormal discharge from penis	Genital sore/ulcer	STI/ abnormal discharge from penis/ sore or ulcer	Number of men who ever had sexual intercourse
**Age**										
15-24	4.4	10.3	2.8	11.8	2,452	0.6	2.1	1.3	2.7	525
15-19	2.2	8.7	2.4	10.1	496	0.0	2.6	2.6	2.6	69
20-24	5.0	10.7	2.9	12.2	1,956	0.6	2.0	1.1	2.7	455
25-29	6.6	11.3	4.5	13.6	2,417	0.6	1.1	0.5	1.2	716
30-39	6.4	10.1	3.5	12.0	4,566	0.4	0.5	0.8	1.1	1,440
40-49	4.8	8.1	3.7	9.8	3,768	0.7	0.5	1.3	1.8	1,147
**Marital status**										
Never married	5.0	5.0	10.0	10.6	56	0.5	1.8	1.2	1.8	303
Married/living together	5.6	9.8	3.6	11.7	11,897	0.5	0.7	1.0	1.5	3,405
Divorced/separated/ widowed	5.6	9.6	3.3	10.7	1,250	1.8	0.0	0.0	1.8	120
**Residence**										
Urban	4.5	8.6	4.4	10.8	2,098	1.4	0.9	1.1	1.9	606
Rural	5.8	10.0	3.5	11.8	11,105	0.4	0.8	0.9	1.5	3,221
**Province**										
Banteay Meanchey	4.0	6.5	1.9	8.7	542	1.5	0.6	1.2	1.8	143
Kampong Cham	8.7	15.8	1.1	17.9	1,624	0.7	1.2	1.2	1.8	548
Kampong Chhnang	3.5	7.8	3.4	8.1	453	0.0	0.2	1.0	1.0	131
Kampong Speu	13.0	10.8	1.7	15.5	936	0.6	0.2	0.6	0.7	241
Kampong Thom	3.7	3.6	0.9	4.5	639	0.0	0.0	0.0	0.0	155
Kandal	2.5	5.5	3.4	6.6	991	0.8	2.9	2.5	4.5	317
Kratie	0.6	8.5	1.4	8.7	383	0.9	0.0	0.5	0.9	108
Phnom Penh	3.5	8.7	4.5	11.0	1,249	1.8	0.8	1.4	2.4	376
Prey Veng	6.4	7.8	2.5	8.2	995	0.0	0.0	0.0	0.0	265
Pursat	2.4	6.7	2.1	6.8	455	0.5	0.0	0.5	0.5	122
Siem Reap	4.4	9.5	3.9	10.0	861	0.0	0.0	0.0	0.0	243
Svay Rieng	3.7	12.6	6.4	13.0	525	0.8	2.1	2.3	4.3	143
Takeo	5.5	7.1	3.8	8.9	786	0.0	1.3	2.5	3.0	244
Otdar Meanchey	1.2	4.8	2.6	4.9	230	0.2	0.0	0.0	0.2	69
Battambang/Pailin	8.9	17.5	8.3	20.4	960	0.3	0.9	0.3	0.9	275
Kampot/Kep	3.9	1.5	1.1	5.7	618	0.5	0.5	0.0	0.5	187
Preah Sihanouk/ Koh Kong	8.5	17.6	13.1	19.9	296	0.4	0.7	1.5	1.9	90
Preah Vihear/ Stung Treng	1.2	17.3	14.5	22.0	354	0.0	0.0	0.0	0.0	78
Mondul Kiri/ Ratanak Kiri	9.3	10.5	0.5	11.4	307	0.0	0.0	0.0	0.0	91
**Education**										
No education	6.9	11.7	4.3	13.6	2,038	0.0	0.2	0.2	0.2	286
Primary	5.9	10.1	3.8	11.8	7,042	0.7	1.2	1.4	2.3	1,750
Secondary and higher	4.5	8.3	3.0	10.3	4,124	0.5	0.5	0.7	1.1	1,791
**Wealth quintile**										
Lowest	5.9	11.8	3.9	13.4	2,555	0.0	0.6	1.6	1.8	663
Second	6.2	11.1	4.1	12.7	2,625	0.8	1.4	0.3	1.6	718
Middle	5.4	9.2	2.7	11.2	2,605	0.4	0.6	0.6	0.8	778
Fourth	5.8	8.6	3.7	10.7	2,654	0.4	0.9	0.9	1.7	830
Highest	4.9	8.5	3.6	10.2	2,764	1.1	0.6	1.3	1.8	839
Total	5.6	9.8	3.6	11.6	13,204	0.6	0.8	1.0	1.6	3,828

18.7 INJECTIONS

Injection overuse in a health care setting can contribute to the transmission of blood-borne pathogens to the extent that it encourages unsafe practices such as reuse of injection equipment. The proportion of injections given with reused injection equipment is an important prevention indicator in initiatives designed to control the spread of HIV/AIDS.

Table 18.12 presents data on the prevalence of injections among respondents. Respondents were asked whether they had had any injections given by a health worker in the 12 months preceding the survey and, if so, the number of injections they had received and whether their last injection was given with a syringe from a new, unopened package. It should be noted that medical injections can be self-administered (e.g., insulin for diabetes). These injections were not included in the calculations.

Women were more likely than men to report having received at least one injection from a health provider in the previous 12 months (37 percent and 27 percent, respectively). On average, women had received two injections, and men had received one injection.

The largest variations in injection prevalence were across provinces. Among women, for example, the percentage reporting that they had received at least one injection from a health worker during the 12 months prior to the survey varied from a low of 15 percent in Mondul Kiri/Ratanak Kiri to a high of 45 percent in Phnom Penh. Among men, the likelihood of having received an injection was also lowest in Mondul Kiri/Ratanak Kiri (15 percent), and it was highest in Kampong Chhnang (43 percent). There is practically no difference between urban and rural residents in terms of receiving at least one injection from a health provider. The associations between receiving at least one injection from a health provider and other background characteristics such as education and wealth were not consistent.

The majority of recent injections (99 percent among both women and men) were administered with a needle and syringe taken from a newly opened package.

Table 18.12 Prevalence of medical injections

Percentage of women and men age 15-49 who received at least one medical injection in the last 12 months, the average number of medical injections per person in the last 12 months, and among those who received a medical injection, the percentage of last medical injections for which the syringe and needle were taken from a new, unopened package, by background characteristics, Cambodia 2014

	Women					Men				
Background characteristic	Percentage who received a medical injection in the last 12 months	Average number of medical injections per person in the last 12 months	Number of respondents	For last injection, syringe and needle taken from a new, unopened package	Number of respondents receiving medical injections in the last 12 months	Percentage who received a medical injection in the last 12 months	Average number of medical injections per person in the last 12 months	Number of respondents	For last injection, syringe and needle taken from a new, unopened package	Number of respondents receiving medical injections in the last 12 months
**Age**										
15-24	38.0	1.7	5,910	98.2	2,245	27.9	0.8	1,760	99.3	491
15-19	32.6	1.2	2,893	98.2	943	28.7	0.8	926	100.0	266
20-24	43.2	2.2	3,017	98.2	1,302	26.9	0.9	835	98.5	225
25-29	41.5	2.6	2,836	98.6	1,176	28.0	1.2	815	99.3	228
30-39	37.2	2.4	4,886	99.2	1,817	25.6	1.1	1,463	99.5	374
40-49	33.6	2.0	3,947	98.1	1,325	27.6	1.6	1,152	98.3	318
**Marital status**										
Never married	31.0	1.2	4,428	98.3	1,374	28.1	0.9	1,663	99.6	468
Ever had sex	25.4	0.6	56	*	14	33.0	1.1	303	98.0	100
Never had sex	31.1	1.2	4,372	98.4	1,360	27.1	0.8	1,360	100.0	368
Married/living together	40.3	2.4	11,898	98.6	4,798	26.7	1.2	3,405	99.2	909
Divorced/separated/ widowed	31.2	2.1	1,252	98.8	391	27.0	0.9	122	(91.2)	33
**Residence**										
Urban	37.1	2.1	3,251	97.7	1,206	26.1	1.0	869	99.1	227
Rural	37.4	2.1	14,327	98.7	5,357	27.4	1.1	4,321	99.1	1,183
**Province**										
Banteay Meanchey	39.7	2.0	689	97.2	273	17.3	0.4	192	(100.0)	33
Kampong Cham	39.3	2.0	2,021	99.5	794	29.3	1.0	663	99.6	194
Kampong Chhnang	41.1	3.8	662	99.5	272	43.0	2.4	182	100.0	79
Kampong Speu	43.9	2.2	1,196	96.8	525	29.3	1.2	323	97.0	95
Kampong Thom	23.5	1.5	851	98.5	200	19.7	0.6	232	100.0	46
Kandal	41.6	2.2	1,330	99.6	553	32.3	1.4	413	98.8	134
Kratie	39.3	2.0	488	97.5	192	42.1	1.3	143	100.0	60
Phnom Penh	44.8	2.7	1,994	98.3	893	29.7	1.1	550	99.5	163
Prey Veng	37.5	3.2	1,188	100.0	445	17.5	1.6	342	(100.0)	60
Pursat	32.5	1.7	631	97.4	205	21.7	0.6	184	100.0	40
Siem Reap	38.4	1.2	1,137	100.0	436	16.2	0.5	337	97.5	55
Svay Rieng	43.5	2.0	654	99.2	285	40.3	1.2	183	98.5	74
Takeo	29.5	2.3	1,082	99.6	320	33.2	1.0	334	96.9	111
Otdar Meanchey	24.3	1.5	294	99.6	72	26.2	1.2	99	98.8	26
Battambang/Pailin	30.2	1.6	1,333	99.1	403	29.1	1.7	405	100.0	118
Kampot/Kep	38.2	2.0	770	93.0	294	20.6	0.7	241	100.0	50
Preah Sihanouk/ Koh Kong	34.8	1.6	422	95.6	147	23.9	1.8	120	100.0	29
Preah Vihear/ Stung Treng	43.1	1.7	462	99.7	199	22.9	0.5	112	100.0	26
Mondul Kiri/ Ratanak Kiri	14.6	0.6	372	98.8	54	15.2	0.3	134	100.0	20
**Education**										
No education	32.5	1.9	2,250	98.8	731	21.2	0.8	324	100.0	69
Primary	38.6	2.2	8,281	98.5	3,197	25.8	1.1	2,167	99.2	560
Secondary and higher	37.4	2.1	7,047	98.5	2,636	29.0	1.2	2,699	99.0	782
**Wealth quintile**										
Lowest	36.7	1.9	3,143	98.7	1,155	22.9	0.8	901	99.3	206
Second	35.8	1.8	3,314	98.7	1,185	27.2	1.2	954	99.4	259
Middle	38.1	2.4	3,381	98.3	1,289	30.5	1.3	1,040	99.5	317
Fourth	38.9	2.2	3,612	98.5	1,404	26.1	1.0	1,124	97.6	294
Highest	37.1	2.1	4,128	98.6	1,531	28.5	1.2	1,171	99.8	333
Total	37.3	2.1	17,578	98.5	6,563	27.2	1.1	5,190	99.1	1,410

Note: Medical injections are those given by a doctor, nurse, pharmacist, dentist, or other health worker. Figures in parentheses are based on 25-49 unweighted cases. An asterisk indicates that a figure is based on fewer than 25 unweighted cases and has been suppressed.

18.8 HIV/AIDS-Related Knowledge and Behavior among Youth

Knowledge of HIV/AIDS issues and related sexual behavior among youth age 15-24 is of particular interest because the period between sexual initiation and marriage is, for many young people, a time of sexual experimentation that may involve high-risk behaviors. This section considers a number of issues that relate to both transmission and prevention of HIV/AIDS among youth, including the extent to which youth have comprehensive knowledge of HIV/AIDS transmission and prevention modes and knowledge of a source where they can obtain condoms. Issues such as abstinence, age at sexual debut, and condom use are also covered in this section.

18.8.1 Knowledge about HIV/AIDS and Source for Condoms

Knowledge of how HIV is transmitted is crucial in enabling young people to avoid AIDS. Young people are often at greater risk because they may have shorter relationships with more partners or engage in other risky behaviors. As discussed earlier, comprehensive knowledge is defined as knowing that people can reduce their chances of getting the AIDS virus by having sex with only one uninfected faithful partner and by using condoms consistently, that a healthy-looking person can have the AIDS virus, and that HIV cannot be transmitted by mosquito bites or by sharing food with a person who has AIDS.

Table 18.13 shows that only 38 percent of young women and 46 percent of young men age 15-24 know all of these facts about HIV/AIDS. The level of comprehensive knowledge about HIV/AIDS slightly increases with age in the youth population. Young women and men who have never been married but have had sex (49 percent and 54 percent, respectively) are more likely than never-married (39 percent and 46 percent, respectively) and ever-married (36 percent and 44 percent, respectively) young adults to have comprehensive knowledge about HIV/AIDS.

As expected, comprehensive HIV/AIDS knowledge is much more common among urban than rural youth. Young adults with a secondary education or higher are about three times as likely as those with no schooling to have comprehensive knowledge of HIV/AIDS.

Because condoms play an important role in combating the transmission of HIV, young women were asked whether they knew where condoms could be obtained. Only “formal” sources of condoms were counted; friends and family and other similar sources were not included.

As shown in Table 18.13, 65 percent of young women know where to obtain a condom. Knowledge of a condom source tends to increase with age. Ever-married young women are more likely to know about a source for condoms than those who have never been married. Women in urban areas are more likely than those in rural areas to know of a condom source. Knowledge of a condom source among women is lowest in Kampong Speu (38 percent) and Kratie (42 percent) and highest in Kampong Chhnang (100 percent). Consistent with the patterns observed for other indicators, young women who are better educated and live in wealthier households are more likely than their counterparts to know a source of condoms.

Table 18.13 Comprehensive knowledge about AIDS and of a source of condoms among youth

Percentage of young women and young men age 15-24 with comprehensive knowledge about AIDS and percentage of young women with knowledge of a source of condoms, by background characteristics, Cambodia 2014

Background characteristic	Women			Men	
	Percentage with comprehensive knowledge of AIDS[1]	Percentage who know a condom source[2]	Number of respondents	Percentage with comprehensive knowledge of AIDS[1]	Number of respondents
**Age**					
15-19	32.7	56.7	2,893	42.4	926
15-17	31.7	53.2	1,774	42.0	581
18-19	34.2	62.3	1,119	43.0	345
20-24	42.4	73.7	3,017	49.9	835
20-22	38.6	71.6	1,811	50.3	545
23-24	48.0	76.8	1,206	49.2	289
**Marital status**					
Never married	38.5	59.5	3,495	46.4	1,392
Ever had sex	(48.5)	(86.0)	37	53.5	158
Never had sex	38.4	59.2	3,458	45.5	1,234
Ever married	36.4	73.9	2,415	44.2	368
**Residence**					
Urban	55.0	79.8	1,171	63.9	324
Rural	33.3	61.9	4,739	41.9	1,436
**Province**					
Banteay Meanchey	10.1	56.4	233	39.7	64
Kampong Cham	26.7	61.5	628	47.3	193
Kampong Chhnang	43.1	100.0	238	49.2	66
Kampong Speu	23.6	37.9	413	39.2	114
Kampong Thom	59.2	79.0	271	75.4	86
Kandal	34.9	68.1	426	0.2	120
Kratie	38.3	41.6	175	22.8	53
Phnom Penh	65.4	85.3	744	75.3	217
Prey Veng	26.4	75.0	310	67.3	85
Pursat	28.0	58.1	227	59.3	71
Siem Reap	27.9	70.7	391	27.9	120
Svay Rieng	15.9	49.0	176	40.2	61
Takeo	49.3	52.8	367	45.4	109
Otdar Meanchey	29.0	53.0	99	0.3	37
Battambang/Pailin	49.7	63.9	486	58.6	149
Kampot/Kep	38.6	66.5	236	46.9	78
Preah Sihanouk/ Koh Kong	36.4	71.5	161	50.9	36
Preah Vihear/ Stung Treng	20.2	52.9	183	18.9	47
Mondul Kiri/ Ratanak Kiri	33.9	60.1	145	30.6	55
**Education**					
No education	16.3	46.1	243	17.8	57
Primary	24.7	58.2	1,955	28.8	572
Secondary and higher	45.8	70.5	3,712	56.0	1,131
**Wealth quintile**					
Lowest	26.6	56.8	956	28.2	323
Second	27.7	59.0	1,057	40.0	304
Middle	28.5	61.4	1,139	44.1	354
Fourth	42.1	61.1	1,262	50.0	384
Highest	54.9	82.1	1,496	62.7	396
Total	37.6	65.4	5,910	45.9	1,760

Note: Figures in parentheses are based on 25-49 unweighted cases.
[1] Comprehensive knowledge means knowing that consistent use of condoms during sexual intercourse and having just one uninfected faithful partner can reduce the chance of getting the AIDS virus, knowing that a healthy-looking person can have the AIDS virus, and rejecting the two most common local misconceptions about AIDS transmission or prevention of the AIDS virus. The components of comprehensive knowledge are presented in Tables 18.2, 18.3.1, and 18.3.2.
[2] For this table, the following responses are not considered a source for condoms: friends, family members, and home.

18.8.2 Age at First Sex and Condom Use at First Sexual Intercourse

Information from the 2014 CDHS can be used to look at several important issues related to the initiation of sexual activity among youth, such as age at first sex and condom use at first sexual intercourse.

Table 18.14 shows the proportion of women and men in the 15-24 age cohort who had sex before age 15 and before age 18. Approximately 2 percent of young women and less than 1 percent of young men had sex by age 15, whereas 18 percent of young women and 5 percent of young men had sex by age 18.

Table 18.14 Age at first sexual intercourse among young people

Percentage of young women and young men age 15-24 who had sexual intercourse before age 15 and percentage of young women and young men age 18-24 who had sexual intercourse before age 18, by background characteristics, Cambodia 2014

	Women				Men			
Background characteristic	Percentage who had sexual intercourse before age 15	Number of respondents (15-24)	Percentage who had sexual intercourse before age 18	Number of respondents (18-24)	Percentage who had sexual intercourse before age 15	Number of respondents (15-24)	Percentage who had sexual intercourse before age 18	Number of respondents (18-24)
**Age**								
15-19	1.4	2,893	na	na	0.3	926	na	na
15-17	0.7	1,774	na	na	0.5	581	na	na
18-19	2.4	1,119	20.6	1,119	0.0	345	4.9	345
20-24	1.6	3,017	17.4	3,017	0.4	835	4.4	835
20-22	1.6	1,811	17.6	1,811	0.6	545	5.2	545
23-24	1.7	1,206	17.0	1,206	0.0	289	3.1	289
**Marital status**								
Never married	0.0	3,495	0.5	1,837	0.4	1,392	2.4	819
Ever married	3.7	2,415	32.4	2,299	0.0	368	9.4	361
**Knows condom source**[1]								
Yes	1.5	3,865	19.0	2,921	na	na	na	na
No	1.6	2,045	16.5	1,215	na	na	na	na
**Residence**								
Urban	0.9	1,171	8.5	873	0.0	324	3.0	237
Rural	1.6	4,739	20.8	3,263	0.4	1,436	5.0	942
**Province**								
Banteay Meanchey	0.4	233	13.6	168	0.0	64	(10.0)	43
Kampong Cham	2.0	628	29.2	423	3.1	193	12.2	132
Kampong Chhnang	0.4	238	13.3	155	0.0	66	4.0	44
Kampong Speu	1.6	413	18.5	292	0.0	114	0.0	77
Kampong Thom	0.0	271	20.5	155	0.0	86	0.0	44
Kandal	3.0	426	16.8	285	0.0	120	0.9	81
Kratie	3.1	175	29.4	128	0.0	53	5.4	36
Phnom Penh	0.9	744	6.2	577	0.0	217	1.5	163
Prey Veng	0.5	310	26.1	207	0.0	85	(0.0)	48
Pursat	1.2	227	11.8	168	0.0	71	3.9	48
Siem Reap	0.1	391	24.3	285	0.0	120	8.3	84
Svay Rieng	0.7	176	15.6	130	0.0	61	(2.2)	37
Takeo	1.3	367	12.1	239	0.0	109	3.6	77
Otdar Meanchey	1.7	99	20.7	69	0.0	37	2.4	23
Battambang/Pailin	0.6	486	14.8	349	0.0	149	5.9	94
Kampot/Kep	2.9	236	22.9	161	0.0	78	5.1	52
Preah Sihanouk/ Koh Kong	1.1	161	14.2	117	0.0	36	4.9	24
Preah Vihear/ Stung Treng	3.8	183	25.9	123	0.0	47	5.5	30
Mondul Kiri/ Ratanak Kiri	8.7	145	35.2	104	0.0	55	6.5	43
**Education**								
No education	5.2	243	38.0	201	0.0	57	9.0	50
Primary	2.3	1,955	26.4	1,468	0.5	572	6.3	372
Secondary and higher	0.9	3,712	11.8	2,467	0.3	1,131	3.4	758
**Wealth quintile**								
Lowest	2.1	956	29.3	662	0.0	323	7.3	210
Second	1.9	1,057	21.9	717	1.0	304	6.3	200
Middle	1.7	1,139	17.8	769	0.8	354	5.8	228
Fourth	1.2	1,262	15.7	891	0.0	384	2.5	250
Highest	1.0	1,496	11.5	1,098	0.0	396	2.2	292
Total	1.5	5,910	18.2	4,136	0.3	1,760	4.6	1,180

Note: Figures in parentheses are based on 25-49 unweighted cases.
na = Not applicable
[1] For this table, the following responses are not considered a source for condoms: friends, family members, and home.

In Cambodia, it is rare for women to have sex prior to marriage; therefore, given that the median age at first marriage among Cambodian women is 21 years, very few women report that they have had sex before age 15. Young women who live in Mondul Kiri/Ratanak Kiri, perhaps by virtue of having a comparatively younger median age at first marriage of 19 years, are most likely to report having had sexual intercourse before age 15 (9 percent).

Among young women in the 18-24 age group, those in urban areas were less likely to have had sex by age 18 than those in rural areas (9 percent versus 21 percent). The proportion of women age 18-24 who reported having had sex before age 18 ranged from 6 percent in Phnom Penh to 35 percent in Mondul Kiri/Ratanak Kiri. Education and wealth showed a negative association with early initiation of sexual activity: as education and wealth increased, the proportion of women reporting sex before age 18 decreased.

Differentials in these indicators among young men tend to be more muted than those among young women. This is in part because the proportions of men initiating sexual activity before age 18 were not large in most subgroups. As was the case with young women, education and wealth were related to initiation of sexual activity before age 18 among young men. For example, young men with no education were more likely to have had sex by age 18 than young men with a secondary education or higher (9 percent versus 3 percent).

18.8.3 Recent Sexual Activity

The period between first sex and marriage is often a time of sexual experimentation. Unfortunately, in the era of HIV/AIDS, it can also be a risky time. Table 18.15 presents data on the percentage of never-married young women and men age 15-24 who have never had sexual intercourse, the percentage who had sex in the 12 months preceding the survey, and, among men who have had sexual intercourse, the percentage who used a condom during their most recent sexual intercourse.

The majority of never-married young women (99 percent) and men (89 percent) reported that they had never had sex, and as a result the proportions reporting recent sexual activity (i.e., within the 12-month period before the survey) are low (less than 1 percent among young women and 7 percent among young men).

Given the comparatively small proportion of never-married young women reporting premarital sexual intercourse, differentials in this indicator are for the most part minimal. Among never-married young men, the proportion reporting premarital sexual activity generally increases with age and wealth and is higher among urban than rural residents. Phnom Penh and Kampong Cham (16 percent each) have the highest proportion of never-married young men reporting premarital sex in the past 12 months. Among men reporting premarital sex, only two-thirds (66 percent) used a condom during their last sexual intercourse.

Table 18.15 Premarital sexual intercourse and condom use during premarital sexual intercourse among youth

Among never-married women and men age 15-24, the percentage who have never had sexual intercourse, the percentage who had sexual intercourse in the past 12 months, and, among men who had premarital sexual intercourse in the past 12 months, the percentage who used a condom at the last sexual intercourse, by background characteristics, Cambodia 2014

Background characteristic	Women			Men				
	Percentage who have never had sexual intercourse	Percentage who had sexual intercourse in the past 12 months	Number of never-married respondents	Percentage who have never had sexual intercourse	Percentage who had sexual intercourse in the past 12 months	Number of never-married respondents	Percentage who used a condom at last sexual intercourse	Number of respondents
**Age**								
15-19	99.3	0.3	2,414	95.6	3.5	896	(50.6)	31
15-17	99.8	0.1	1,658	97.7	1.7	574	*	10
18-19	98.2	0.6	756	91.7	6.6	323	*	21
20-24	98.1	1.6	1,081	76.2	14.0	496	72.8	69
20-22	98.0	1.9	762	79.4	11.9	378	64.4	45
23-24	98.5	1.0	319	65.9	20.6	118	(88.3)	24
**Knows condom source**[1]								
Yes	98.5	1.1	2,081	na	na	na	na	na
No	99.6	0.1	1,414	na	na	na	na	na
**Residence**								
Urban	97.4	2.4	870	79.2	13.8	294	85.3	41
Rural	99.4	0.1	2,625	91.2	5.4	1,099	(52.8)	60
**Province**								
Banteay Meanchey	100.0	0.0	122	91.4	4.6	50	*	2
Kampong Cham	99.3	0.0	322	74.4	15.6	141	*	22
Kampong Chhnang	100.0	0.0	158	89.0	5.0	49	*	2
Kampong Speu	99.4	0.0	230	94.7	5.3	81	*	4
Kampong Thom	100.0	0.0	172	98.1	0.9	73	*	1
Kandal	97.0	0.6	258	87.8	7.3	102	*	7
Kratie	100.0	0.0	84	85.4	10.2	38	*	4
Phnom Penh	97.2	2.7	550	78.1	15.9	200	(78.3)	32
Prey Veng	100.0	0.0	153	94.8	0.0	69	*	0
Pursat	100.0	0.0	136	91.6	7.2	59	*	4
Siem Reap	96.6	2.9	225	95.1	2.4	93	*	2
Svay Rieng	100.0	0.0	94	88.1	7.1	46	*	3
Takeo	99.4	0.0	242	85.6	6.4	92	*	6
Otdar Meanchey	99.1	0.9	56	98.8	1.2	28	*	0
Battambang/Pailin	100.0	0.0	308	98.3	1.0	114	*	1
Kampot/Kep	100.0	0.0	125	90.9	7.4	55	*	4
Preah Sihanouk/ Koh Kong	99.4	0.3	107	87.7	10.1	30	*	3
Preah Vihear/ Stung Treng	99.6	0.4	95	100.0	0.0	32	*	0
Mondul Kiri/ Ratanak Kiri	99.8	0.2	58	94.9	3.5	42	*	1
**Education**								
No education	99.4	0.6	77	(89.4)	(10.6)	35	*	4
Primary	99.1	0.5	886	89.4	6.5	415	(68.2)	27
Secondary and higher	98.9	0.7	2,532	88.3	7.4	943	68.6	70
**Wealth quintile**								
Lowest	100.0	0.0	470	93.7	5.3	234	*	12
Second	99.8	0.0	574	91.7	3.5	234	*	8
Middle	99.4	0.2	631	87.7	6.8	274	*	19
Fourth	98.4	0.6	776	88.7	8.3	298	*	25
Highest	98.2	1.7	1,044	84.0	10.3	353	80.9	36
Total	98.9	0.7	3,495	88.7	7.2	1,392	66.0	100

Note: Figures in parentheses are based on 25-49 unweighted cases. An asterisk indicates that a figure is based on fewer than 25 unweighted cases and has been suppressed.
na = Not applicable
[1] For this table, the following responses are not considered a source for condoms: friends, family members, and home.

18.8.4 Multiple Sexual Partnerships

The most common mode of HIV transmission in Cambodia is through unprotected sex with an infected person. To prevent HIV/AIDS transmission, it is important for young people to be faithful to one uninfected partner. Table 18.16 shows the percentage of all young women and men age 15-24 who had sexual intercourse with more than one partner in the 12 months before the survey, by background characteristics.

Table 18.16 Multiple sexual partners in the past 12 months among youth

Among all young women and men age 15-24, the percentage who had sexual intercourse with more than one sexual partner in the past 12 months, and among those having more than one partner in the past 12 months, the percentage reporting that a condom was used at last intercourse, by background characteristics, Cambodia 2014

Background characteristic	Women age 15-24		Men age 15-24	
	Percentage who had 2+ partners in the past 12 months	Number of women	Percentage who had 2+ partners in the past 12 months	Number of men
**Age**				
15-19	0.0	2,893	0.2	926
15-17	0.0	1,774	0.0	581
18-19	0.0	1,119	0.5	345
20-24	0.3	3,017	2.1	835
20-22	0.3	1,811	2.1	545
23-24	0.2	1,206	2.2	289
**Marital status**				
Never married	0.0	3,495	0.7	1,392
Ever married	0.3	2,415	2.4	368
**Knows condom source**[1]				
No	0.2	3,865	na	na
Yes	0.0	2,045	na	na
**Residence**				
Urban	0.3	1,171	3.5	324
Rural	0.1	4,739	0.6	1,436
**Education**				
No education	0.6	243	0.0	57
Primary	0.3	1,955	0.9	572
Secondary and higher	0.0	3,712	1.3	1,131
Total	0.1	5,910	1.1	1,760

na = Not available
[1] For this table, the following responses are not considered a source for condoms: friends, family members, and home.

Overall, less than 1 percent of young women and 1 percent of young men reported having had two or more sexual partners in the past 12 months. Given the comparatively small proportions of young women and men having had multiple sexual partners in the past 12 months, differentials in this indicator are generally not significant.

18.8.5 HIV Testing

Young people may believe there are barriers to accessing and using many health services and facilities, and this is particularly true for sensitive concerns relating to sexual health, such as HIV/AIDS and other STIs. Table 18.17 presents data on the percentage of sexually active youth who had been tested and received their results within the past year. Young women who had had sexual intercourse in the 12 months before the survey were more likely than young men to have been tested for HIV (25 percent and 18 percent, respectively). In the case of young women, testing levels were higher among those living in urban areas, those with a secondary education or higher, those in the highest wealth quintile, and those living in Kampong Chhnang. Among young men, testing rates were higher among those with a secondary education or higher. There was no clear association among young men between HIV testing and other background characteristics.

Table 18.17 Recent HIV tests among youth

Among young women and young men age 15-24 who have had sexual intercourse in the past 12 months, the percentage who were tested for HIV in the past 12 months and received the results of the last test, by background characteristics, Cambodia 2014

Background characteristic	Women age 15-24 who have had sexual intercourse in the past 12 months: Percentage who have been tested for HIV in the past 12 months and received the results of the last test	Women age 15-24 who have had sexual intercourse in the past 12 months: Number of women	Men age 15-24 who have had sexual intercourse in the past 12 months: Percentage who have been tested for HIV in the past 12 months and received the results of the last test	Men age 15-24 who have had sexual intercourse in the past 12 months: Number of men
**Age**				
15-19	29.0	468	19.7	60
15-17	36.2	116	*	17
18-19	26.7	353	(27.3)	44
20-24	24.1	1,859	17.6	388
20-22	27.5	1,016	19.0	202
23-24	20.1	843	16.1	186
**Marital status**				
Never married	(39.3)	24	16.2	100
Ever married	25.0	2,303	18.4	348
**Knows condom source**[1]				
Yes	27.1	1,726	na	na
No	19.6	601	na	na
**Residence**				
Urban	36.8	305	17.6	70
Rural	23.4	2,022	18.0	378
**Province**				
Banteay Meanchey	35.7	102	*	17
Kampong Cham	22.7	293	(22.8)	74
Kampong Chhnang	45.0	72	*	18
Kampong Speu	22.7	181	(20.4)	35
Kampong Thom	29.5	98	*	14
Kandal	23.6	159	*	26
Kratie	4.3	87	(7.0)	18
Phnom Penh	35.6	199	(16.8)	48
Prey Veng	11.1	150	*	12
Pursat	26.5	88	*	16
Siem Reap	28.6	168	*	30
Svay Rieng	34.5	78	*	17
Takeo	27.8	115	*	22
Otdar Meanchey	21.6	42	(20.8)	9
Battambang/Pailin	34.6	172	*	29
Kampot/Kep	20.0	106	(5.8)	26
Preah Sihanouk/ Koh Kong	37.0	52	*	8
Preah Vihear/ Stung Treng	8.4	83	(0.0)	15
Mondul Kiri/ Ratanak Kiri	5.2	82	(0.0)	15
**Education**				
No education	21.0	154	(0.0)	25
Primary	21.7	1,022	13.4	179
Secondary and higher	28.7	1,151	23.0	245
**Wealth quintile**				
Lowest	18.6	466	8.3	99
Second	25.1	469	19.2	75
Middle	22.8	479	22.5	98
Fourth	26.7	471	20.2	100
Highest	32.9	442	20.3	77
Total	25.1	2,327	17.9	448

Note: Figures in parentheses are based on 25-49 unweighted cases. An asterisk indicates that a figure is based on fewer than 25 unweighted cases and has been suppressed.
na = Not available
[1] For this table, the following responses are not considered a source for condoms: friends, family members, and home.

WOMEN'S EMPOWERMENT AND DEMOGRAPHIC AND HEALTH OUTCOMES 19

Key Findings

- Seventy-four percent of currently married employed women who earn cash make independent decisions about how to spend their earnings.
- About 4 in 10 women own a house and/or land, mostly jointly with their husband.
- A large majority of currently married women (86 percent) participate in three specified decisions pertaining to their own health care, major household purchases, and visits to their family or relatives.
- Fifty percent of women believe that wife beating is justified for at least one of six specified reasons.

The 2014 CDHS collected information on the general background characteristics of respondents (age, education, wealth quintile, and employment status) but also information specific to women's empowerment, such as receipt of cash earnings, the magnitude of a woman's earnings relative to those of her husband, and control over the use of her own earnings and those of her spouse.[1]

In addition, the 2014 CDHS collected information on women's participation in household decision making and their attitude, as well as that of their husband, towards wife beating. This report uses the two DHS-developed indices of women's empowerment to measure women's and men's responses to the questions. The first index is based on the number of household decisions in which the woman participates, and the second is based on the respondent's opinion regarding the number of reasons that justify wife beating. The ranking of women on these two indices is then related to selected demographic and health outcomes, including use of contraception, ideal family size, and the use of reproductive health care services during pregnancy, childbirth, and the postnatal period.

19.1 EMPLOYMENT AND FORMS OF EARNINGS

Employment can be a source of empowerment for both women and men. It is particularly so for women if it puts them in control of the household income. In the 2014 CDHS, respondents were asked whether they were employed at the time of the survey and, if not, whether they were employed in the 12 months preceding the survey.

Table 19.1 shows that 81 percent of currently married women age 15-49 were employed at the time of the survey or within the 12 months preceding the survey, as compared with practically all men. Younger married women (age 15-24) were less likely to be employed than older respondents.

Among currently married respondents who had been employed in the past 12 months, 92 percent of women and 95 percent of men received earnings in cash or cash and in-kind. About 3 percent of women and 2 percent of men employed in the past 12 months were not paid. The proportion not paid was highest among young respondents.

[1] The questions were phrased in terms of "husband/partner" (for women) and "wife/partner" (for men), referring to marital partners; however, in this report, the word "partner" has been dropped to simplify the text and tables.

Table 19.1 Employment and cash earnings of currently married women and men

Percentage of currently married women and men age 15-49 who were employed at any time in the past 12 months and the percent distribution of currently married women and men employed in the past 12 months by type of earnings, according to age, Cambodia 2014

	Among currently married respondents:		Percent distribution of currently married respondents employed in the past 12 months, by type of earnings						
Age	Percentage employed in past 12 months	Number of respondents	Cash only	Cash and in-kind	In-kind only	Not paid	Missing/don't know	Total	Number of women
				WOMEN					
15-19	70.6	450	86.6	3.8	3.1	6.5	0.0	100.0	318
20-24	73.8	1,833	84.5	7.7	2.3	5.4	0.0	100.0	1,352
25-29	77.8	2,249	85.5	7.5	4.0	3.0	0.0	100.0	1,750
30-34	82.5	2,625	87.1	7.7	3.2	2.0	0.0	100.0	2,166
35-39	85.9	1,573	82.5	8.7	5.8	3.0	0.0	100.0	1,351
40-44	87.8	1,673	79.9	10.2	6.8	3.1	0.0	100.0	1,469
45-49	85.2	1,495	78.3	11.4	7.1	3.3	0.0	100.0	1,273
Total	81.3	11,898	83.6	8.5	4.6	3.3	0.0	100.0	9,679
				MEN					
15-19	*	27	*	*	*	*	*	*	25
20-24	100.0	308	81.5	13.5	0.2	4.9	0.0	100.0	308
25-29	99.3	591	88.7	7.3	2.1	1.8	0.0	100.0	587
30-34	99.9	832	88.4	8.5	1.7	1.5	0.0	100.0	831
35-39	100.0	529	80.2	12.8	5.7	1.4	0.0	100.0	529
40-44	99.4	572	84.0	12.0	1.5	2.5	0.0	100.0	569
45-49	99.4	545	77.3	18.7	2.5	1.5	0.0	100.0	542
Total	99.6	3,405	83.9	11.5	2.4	2.1	0.0	100.0	3,391

Note: An asterisk indicates that a figure is based on fewer than 25 unweighted cases and has been suppressed.

19.2 Control over Women's and Men's Earnings

Currently married women who were employed and received cash for their work were asked to identify the main decision maker in the family regarding use of their earnings. They were also asked the relative magnitude of their earnings in comparison with those of their husband. Women whose husbands were employed for cash were asked who usually decides how his earnings are used. Men were also asked who mainly decides how their earnings are used. These pieces of information provide insight into women's level of empowerment in the family and the extent of their control over decision making regarding the use of household income. It is expected that employment and cash earnings are more likely to empower women if they control their own earnings and perceive their earnings as important relative to those of their husband and important to the welfare of the household.

Table 19.2.1 shows the results on women's control over their cash earnings and the relative magnitude of their earnings relative to those of their husband for currently married women who had cash earnings in the 12 months preceding the survey. Seventy-four percent of married women who are employed say that they mainly control their cash earnings; 25 percent say that decisions regarding how their earnings are used are made jointly with their husband, and only 2 percent say that their husband mainly controls their cash earnings.

Women's control over their cash earnings is highest among those in Kampot/Kep (92 percent), Kampong Thom (90 percent), and Svay Rieng (90 percent) and lowest among those in Mondul Kiri/Ratanak Kiri (24 percent) and Banteay Meanchey (36 percent). Younger women and women with no education are less likely than other women to control their cash earnings. There is little variation in control of cash income by other background characteristics.

Nearly one in two (48 percent) currently married, employed women in Cambodia say they earn less than their husband; 35 percent say they earn about the same amount, and 17 percent say either that they earn more than their husband or that their husband has no earnings. Thus, half of currently married, employed women earn at least as much as their husband. Employed women in urban areas are more likely than employed women in rural areas to earn more than their husbands, as are better educated women and those in higher wealth quintiles.

Table 19.2.1 Control over women's cash earnings and relative magnitude of women's cash earnings

Percent distribution of currently married women age 15-49 who received cash earnings for employment in the 12 months preceding the survey by person who decides how wife's cash earnings are used and by whether she earned more or less than her husband, according to background characteristics, Cambodia 2014

	Person who decides how the wife's cash earnings are used:						Wife's cash earnings compared with husband's cash earnings:						
Background characteristic	Mainly wife	Wife and husband jointly	Mainly husband	Other	Missing	Total	More	Less	About the same	Husband has no earnings	Don't know/ missing	Total	Number of women
**Age**													
15-19	63.7	33.6	2.1	0.7	0.0	100.0	9.8	64.0	26.2	0.0	0.0	100.0	287
20-24	71.1	27.0	1.0	0.9	0.0	100.0	14.6	55.2	29.8	0.4	0.0	100.0	1,247
25-29	71.0	27.5	1.4	0.1	0.0	100.0	15.9	49.0	34.4	0.6	0.2	100.0	1,628
30-34	74.2	23.7	1.9	0.2	0.0	100.0	16.2	49.9	33.2	0.5	0.2	100.0	2,054
35-39	75.1	23.5	1.3	0.0	0.0	100.0	18.9	45.4	35.0	0.7	0.0	100.0	1,232
40-44	77.1	20.9	1.8	0.0	0.2	100.0	17.4	42.7	38.8	0.7	0.4	100.0	1,324
45-49	75.4	23.4	1.2	0.0	0.0	100.0	13.7	41.2	42.6	2.2	0.3	100.0	1,141
**Number of living children**													
0	71.0	26.5	1.6	0.9	0.0	100.0	18.1	50.8	30.6	0.5	0.0	100.0	849
1-2	73.1	25.4	1.2	0.2	0.1	100.0	16.5	49.2	33.5	0.6	0.2	100.0	4,403
3-4	74.8	23.2	1.9	0.1	0.0	100.0	15.5	45.7	37.8	0.8	0.2	100.0	2,716
5+	74.1	24.1	1.7	0.0	0.0	100.0	12.6	48.0	37.8	1.5	0.1	100.0	945
**Residence**													
Urban	71.7	27.1	1.1	0.2	0.0	100.0	20.2	41.7	36.8	1.2	0.1	100.0	1,414
Rural	73.9	24.3	1.6	0.2	0.1	100.0	15.1	49.3	34.7	0.7	0.2	100.0	7,499
**Province**													
Banteay Meanchey	35.8	62.7	1.0	0.0	0.5	100.0	6.8	67.5	25.1	0.0	0.6	100.0	412
Kampong Cham	87.6	10.8	1.6	0.0	0.0	100.0	11.0	46.1	42.4	0.6	0.0	100.0	1,065
Kampong Chhnang	43.5	54.6	1.9	0.0	0.0	100.0	13.8	48.4	37.8	0.0	0.0	100.0	381
Kampong Speu	77.5	20.7	0.9	0.9	0.0	100.0	22.4	37.4	39.0	0.6	0.6	100.0	765
Kampong Thom	90.3	9.2	0.2	0.2	0.0	100.0	11.5	47.4	40.4	0.6	0.0	100.0	477
Kandal	77.6	22.1	0.2	0.0	0.0	100.0	27.1	46.7	26.1	0.1	0.0	100.0	708
Kratie	80.6	16.8	2.0	0.0	0.6	100.0	9.7	68.1	20.3	1.2	0.7	100.0	200
Phnom Penh	70.7	28.0	0.8	0.4	0.0	100.0	20.0	45.4	33.2	1.5	0.0	100.0	862
Prey Veng	78.9	20.7	0.3	0.0	0.0	100.0	12.2	53.3	33.7	0.8	0.0	100.0	732
Pursat	80.4	18.7	0.8	0.0	0.0	100.0	12.9	58.8	28.0	0.3	0.0	100.0	300
Siem Reap	66.5	31.5	1.7	0.3	0.0	100.0	24.8	39.7	35.0	0.6	0.0	100.0	460
Svay Rieng	89.7	7.7	1.4	1.2	0.0	100.0	12.6	41.0	44.6	1.8	0.0	100.0	421
Takeo	70.8	18.4	10.4	0.4	0.0	100.0	27.0	47.2	24.7	1.1	0.0	100.0	395
Otdar Meanchey	66.7	31.5	1.6	0.0	0.2	100.0	6.5	44.1	48.4	0.9	0.2	100.0	175
Battambang/Pailin	79.4	18.8	1.8	0.0	0.0	100.0	15.8	43.0	39.7	0.4	1.0	100.0	490
Kampot/Kep	91.5	7.8	0.7	0.0	0.0	100.0	12.7	64.0	20.6	2.4	0.3	100.0	451
Preah Sihanouk/ Koh Kong	53.1	46.3	0.5	0.0	0.0	100.0	16.7	44.2	38.5	0.6	0.0	100.0	187
Preah Vihear/ Stung Treng	55.8	44.0	0.0	0.0	0.1	100.0	11.3	57.4	30.4	0.0	0.9	100.0	193
Mondul Kiri/ Ratanak Kiri	24.0	72.3	3.6	0.0	0.1	100.0	5.4	36.6	57.9	0.0	0.2	100.0	239
**Education**													
No education	69.7	27.8	2.5	0.0	0.0	100.0	12.2	45.6	40.3	1.6	0.3	100.0	1,261
Primary	75.2	23.2	1.3	0.2	0.1	100.0	15.6	48.8	34.7	0.7	0.3	100.0	4,763
Secondary and higher	72.5	25.9	1.3	0.3	0.0	100.0	18.1	48.2	33.1	0.5	0.0	100.0	2,889
**Wealth quintile**													
Lowest	73.0	25.2	1.6	0.3	0.0	100.0	11.0	53.1	34.4	1.2	0.3	100.0	1,653
Second	75.7	22.9	1.2	0.1	0.1	100.0	13.8	51.5	33.9	0.7	0.2	100.0	1,777
Middle	75.5	23.2	0.9	0.3	0.1	100.0	15.3	51.1	32.8	0.6	0.2	100.0	1,681
Fourth	72.6	24.4	2.7	0.3	0.0	100.0	18.7	44.5	36.2	0.4	0.1	100.0	1,872
Highest	71.2	27.6	1.1	0.1	0.0	100.0	20.0	41.7	37.2	0.9	0.2	100.0	1,930
Total	73.5	24.7	1.5	0.2	0.0	100.0	15.9	48.1	35.0	0.7	0.2	100.0	8,913

Currently married men age 15-49 who receive cash earnings and currently married women age 15-49 whose husbands receive cash earnings were asked who decides how the husband's cash earnings are spent. Table 19.2.2 shows that 50 percent of men and 61 percent of women say that the wife mainly decides how the husband's earnings are used. Forty-one percent of men and 35 percent of women say the husband and wife decide jointly how the man's cash earnings are used.

Table 19.2.2 Control over men's cash earnings

Percent distributions of currently married men age 15-49 who receive cash earnings and of currently married women age 15-49 whose husbands receive cash earnings, by person who decides how husband's cash earnings are used, according to background characteristics, Cambodia 2014

	Men							Women						
Background characteristic	Mainly wife	Husband and wife jointly	Mainly husband	Other	Missing	Total	Number	Mainly wife	Husband and wife jointly	Mainly husband	Other	Missing	Total	Number
**Age**														
15-19	*	*	*	*	*	100.0	20	54.5	39.6	5.3	0.5	0.1	100.0	449
20-24	49.4	44.1	6.0	0.4	0.1	100.0	292	59.2	37.8	2.7	0.3	0.0	100.0	1,828
25-29	48.5	41.6	9.7	0.2	0.0	100.0	564	61.7	35.5	2.8	0.1	0.0	100.0	2,240
30-34	50.3	42.1	7.6	0.1	0.0	100.0	805	60.8	34.8	4.4	0.0	0.0	100.0	2,614
35-39	46.9	42.6	10.4	0.0	0.1	100.0	492	60.7	35.6	3.7	0.0	0.0	100.0	1,563
40-44	53.2	38.0	8.8	0.0	0.0	100.0	546	63.9	31.7	4.3	0.1	0.0	100.0	1,660
45-49	50.9	40.6	8.4	0.0	0.1	100.0	520	63.2	32.6	4.2	0.0	0.0	100.0	1,463
**Number of living children**														
0	46.1	45.3	8.1	0.5	0.0	100.0	307	56.3	40.1	3.1	0.4	0.0	100.0	1,122
1-2	50.8	41.0	8.1	0.1	0.1	100.0	1,593	61.5	34.6	3.8	0.1	0.0	100.0	5,911
3-4	50.0	40.4	9.6	0.0	0.0	100.0	1,004	62.1	34.1	3.7	0.0	0.0	100.0	3,546
5+	49.4	42.9	7.6	0.0	0.1	100.0	334	61.5	34.4	4.1	0.1	0.0	100.0	1,239
**Residence**														
Urban	39.4	50.5	10.0	0.0	0.1	100.0	462	53.5	39.8	6.6	0.1	0.0	100.0	1,799
Rural	51.7	39.9	8.3	0.1	0.0	100.0	2,776	62.6	34.1	3.2	0.1	0.0	100.0	10,019
**Province**														
Banteay Meanchey	93.7	4.5	1.8	0.0	0.0	100.0	127	23.9	74.9	1.1	0.0	0.0	100.0	503
Kampong Cham	43.2	42.9	13.9	0.0	0.0	100.0	437	60.2	36.2	3.6	0.0	0.0	100.0	1,484
Kampong Chhnang	13.5	73.6	12.4	0.0	0.4	100.0	115	27.4	68.8	3.7	0.0	0.0	100.0	396
Kampong Speu	41.7	50.9	7.4	0.0	0.0	100.0	221	53.2	43.7	2.7	0.5	0.0	100.0	837
Kampong Thom	91.2	6.9	1.9	0.0	0.0	100.0	145	84.7	14.2	1.1	0.0	0.0	100.0	569
Kandal	72.7	8.6	18.6	0.0	0.0	100.0	288	76.0	21.8	2.1	0.0	0.0	100.0	869
Kratie	35.3	45.0	18.2	0.6	1.0	100.0	86	71.4	27.5	0.7	0.2	0.2	100.0	356
Phnom Penh	30.8	60.4	8.8	0.0	0.0	100.0	278	49.8	41.3	8.8	0.1	0.0	100.0	1,070
Prey Veng	66.4	25.2	7.9	0.4	0.0	100.0	246	73.6	25.9	0.5	0.0	0.0	100.0	883
Pursat	53.8	36.7	9.4	0.0	0.0	100.0	102	71.2	26.7	1.9	0.2	0.0	100.0	424
Siem Reap	1.4	98.6	0.0	0.0	0.0	100.0	188	53.7	41.8	4.3	0.2	0.0	100.0	754
Svay Rieng	9.0	86.1	4.0	0.9	0.0	100.0	124	80.0	17.4	2.6	0.0	0.0	100.0	474
Takeo	62.0	23.9	14.1	0.0	0.0	100.0	180	62.3	21.9	15.5	0.3	0.0	100.0	673
Otdar Meanchey	0.0	100.0	0.0	0.0	0.0	100.0	58	53.9	45.9	0.2	0.0	0.0	100.0	216
Battambang/Pailin	81.5	17.4	1.1	0.0	0.0	100.0	242	77.0	19.5	3.3	0.2	0.0	100.0	886
Kampot/Kep	64.0	30.5	5.5	0.0	0.0	100.0	173	90.4	7.6	2.0	0.0	0.0	100.0	563
Preah Sihanouk/ Koh Kong	66.7	21.8	11.5	0.0	0.0	100.0	74	43.0	56.1	0.9	0.0	0.0	100.0	264
Preah Vihear/ Stung Treng	44.7	51.5	3.8	0.0	0.0	100.0	72	30.9	67.8	1.3	0.0	0.0	100.0	314
Mondul Kiri/ Ratanak Kiri	13.0	84.0	3.0	0.0	0.0	100.0	82	22.1	73.4	4.3	0.0	0.2	100.0	281
**Education**														
No education	50.0	45.3	4.7	0.0	0.0	100.0	240	58.0	36.5	5.5	0.0	0.0	100.0	1,746
Primary	53.1	38.1	8.7	0.0	0.1	100.0	1,528	62.9	33.7	3.3	0.2	0.0	100.0	6,363
Secondary and higher	46.6	44.3	8.9	0.2	0.0	100.0	1,470	59.8	36.5	3.7	0.0	0.0	100.0	3,709
**Wealth quintile**														
Lowest	53.4	39.3	7.1	0.0	0.1	100.0	572	60.7	36.0	3.1	0.1	0.0	100.0	2,270
Second	49.4	41.6	8.9	0.2	0.0	100.0	615	64.7	32.8	2.4	0.1	0.0	100.0	2,390
Middle	52.9	37.1	9.7	0.2	0.1	100.0	664	62.7	33.8	3.3	0.1	0.0	100.0	2,352
Fourth	51.1	41.5	7.5	0.0	0.0	100.0	720	61.2	34.1	4.6	0.1	0.0	100.0	2,381
Highest	43.3	47.4	9.3	0.0	0.0	100.0	667	56.8	38.1	5.1	0.1	0.0	100.0	2,424
Total	49.9	41.4	8.5	0.1	0.0	100.0	3,238	61.2	35.0	3.7	0.1	0.0	100.0	11,818

Note: An asterisk indicates that a figure is based on fewer than 25 unweighted cases and has been suppressed.

Men in Kandal and Kratie (19 percent and 18 percent, respectively) are more likely to make decisions themselves on how to use their cash earnings than their counterparts in other provinces. More women in Takeo (16 percent) whose husbands receive cash earnings report that their husbands usually have sole authority over the use of their earnings than in other provinces. All of the married, working men interviewed in Otdar Meanchey reported that decisions on how to use their earnings were made jointly with their wives.

Table 19.3 shows the percent distribution of currently married women age 15-49 who received cash earnings in the past 12 months by the person who decides how their cash earnings are used and the percent distribution of currently married women age 15-49 whose husbands received cash earnings in the

past 12 months by the person who decides how the husband's cash earnings are used, according to the relative magnitude of the earnings of the woman and her husband.

Women who earn more than their husbands are more likely to decide how their cash earnings are used (79 percent) than women whose cash earnings are the same as those of their husband (69 percent). Women who say they earn about the same amount as their husband are more likely to make joint decisions with their husband about how their cash earnings and those of their husbands are used (30 percent and 42 percent, respectively).

Table 19.3 Women's control over their own earnings and over those of their husbands

Percent distribution of currently married women age 15-49 with cash earnings in the last 12 months by person who decides how the wife's cash earnings are used and percent distribution of currently married women age 15-49 whose husbands have cash earnings by person who decides how the husband's cash earnings are used, according to the relation between wife's and husband's cash earnings, Cambodia 2014

	Person who decides how the wife's cash earnings are used:							Person who decides how the husband's cash earnings are used:						
Women's earnings relative to husband's earnings	Mainly wife	Wife and husband jointly	Mainly husband	Other	Missing	Total	Number	Mainly wife	Wife and husband jointly	Mainly husband	Other	Missing	Total	Number of women
More than husband	78.7	19.4	1.8	0.1	0.0	100.0	1,422	67.9	27.2	4.9	0.0	0.0	100.0	1,422
Less than husband	75.1	23.1	1.6	0.2	0.0	100.0	4,290	60.4	36.0	3.5	0.0	0.0	100.0	4,290
Same as husband	68.8	29.7	1.3	0.3	0.0	100.0	3,119	55.6	42.1	2.1	0.2	0.0	100.0	3,119
Husband has no cash earnings or did not work	86.3	13.7	0.0	0.0	0.0	100.0	67	na	na	na	na	na	na	0
Woman worked but has no cash earnings	na	na	na	na	na	na	0	64.3	33.1	2.6	0.0	0.0	100.0	760
Woman did not work	na	na	na	na	na	na	0	65.6	28.6	5.6	0.2	0.0	100.0	2,211
Total	73.5	24.7	1.5	0.2	0.0	100.0	8,913	61.2	35.0	3.7	0.1	0.0	100.0	11,818

Note: Total includes 17 cases where a woman does not know whether she earned more or less than her husband.
na = Not applicable

19.3 PARTICIPATION IN HOUSEHOLD DECISION MAKING

The ability to make decisions about their own life is important to women's empowerment. In addition to information on women's control over cash earnings, the 2014 CDHS collected information from both women and men on other measures of women's empowerment. Respondents were asked about women's role in household decision making and acceptance of wife beating. Such information provides insight into women's control over their environment and their attitudes towards gender roles, both of which are relevant to understanding women's ability to make independent decisions about their own health care and that of their children.

To assess women's decision-making autonomy, information was collected on their participation in three types of household decisions: their own health care, making large household purchases, and visiting their family or relatives. Having a final say in the decision-making process represents the highest degree of autonomy. Women are considered to participate in a decision if they usually make that decision alone or jointly with their husband. Table 19.4 shows the percent distribution of currently married women age 15-49 by the person in the household who usually makes decisions concerning these matters.

Cambodian women are usually involved in all three specific decisions, although the extent of their involvement depends on the issue being decided. Forty-six percent of women say they alone make decisions about their own health care. However, decisions about major household purchases and visits to the wife's family or relatives are usually made jointly by the husband and wife.

Married men are somewhat less likely than women to mainly make decisions about their own health care (29 percent versus 46 percent). However, 73 percent of men say they make decisions about major household purchases jointly with their wives, about the same proportion as among women (76 percent).

Table 19.4 Participation in decision making

Percent distribution of currently married women and currently married men age 15-49 by person who usually makes decisions about various issues, Cambodia 2014

Decision	Mainly wife	Wife and husband jointly	Mainly husband	Someone else	Other	Missing	Total	Number
WOMEN								
Own health care	46.4	45.1	8.0	0.5	0.1	0.0	100.0	11,898
Major household purchases	17.2	76.3	5.5	0.9	0.1	0.0	100.0	11,898
Visits to her family or relatives	22.8	73.6	3.4	0.2	0.0	0.0	100.0	11,898
MEN								
Own health care	24.9	46.0	28.9	0.1	0.0	0.0	100.0	3,405
Major household purchases	17.6	73.2	8.9	0.3	0.0	0.0	100.0	3,405

Table 19.5.1 shows the percentage of married women who participate in the three decisions specified for female respondents, according to background characteristics. As noted above, a woman is considered to participate in a decision if she says she usually makes the decision alone or jointly with her husband.

Ninety-two percent of currently married women age 15-49 say they make decisions about their own health care either by themselves or jointly with their husbands, and 94 percent say they participate in decisions about major household purchases. Ninety-six percent say they participate in decisions about visits to their family or relatives. Overall, 86 percent of currently married women participate in all three decisions, and less than 2 percent do not participate in any of the three decisions.

Younger women are less likely than older women to participate in all three kinds of decisions. In addition, women employed for cash are more likely to participate in all three decisions (88 percent) than women who are employed but not for cash (79 percent). Women with children are more likely to participate in all three decisions (86-88 percent) than women with no children (80 percent).

Women in Phnom Penh and Kratie are less likely to participate in all three decisions. Almost one in five women in Takeo say they do not participate in any of the three types of decisions. Women in rural areas are more likely than those in urban areas to participate in all three decisions. Women’s participation in decision making varies slightly by education and wealth quintile.

Table 19.5.1 Women's participation in decision making by background characteristics

Percentage of currently married women age 15-49 who usually make specific decisions either by themselves or jointly with their husband, by background characteristics, Cambodia 2014

Background characteristic	Specific decisions: Woman's own health care	Making major household purchases	Visits to her family or relatives	All three decisions	None of the three decisions	Number of women
**Age**						
15-19	89.2	86.7	95.3	77.3	1.3	450
20-24	90.7	92.0	95.7	82.9	1.2	1,833
25-29	92.1	94.2	97.4	87.8	1.0	2,249
30-34	90.4	93.6	96.0	86.0	1.9	2,625
35-39	92.5	94.7	96.8	87.5	1.0	1,573
40-44	92.0	93.5	95.6	87.4	1.7	1,673
45-49	92.3	94.9	97.3	89.1	1.6	1,495
**Employment (last 12 months)**						
Not employed	88.8	90.0	92.7	81.5	3.3	2,218
Employed for cash	92.6	94.5	97.2	88.2	1.1	8,913
Employed not for cash	86.4	92.3	98.0	78.8	0.3	766
**Number of living children**						
0	88.7	90.4	95.9	80.3	1.0	1,128
1-2	91.3	93.5	96.4	86.0	1.5	5,942
3-4	92.5	94.5	96.5	88.2	1.4	3,572
5+	91.8	93.6	96.9	88.1	1.7	1,257
**Residence**						
Urban	84.5	92.8	96.4	79.4	1.0	1,818
Rural	92.7	93.6	96.4	87.6	1.5	10,080
**Province**						
Banteay Meanchey	99.5	99.1	99.9	98.6	0.0	503
Kampong Cham	98.4	98.2	99.9	97.0	0.0	1,490
Kampong Chhnang	98.0	97.1	96.8	94.7	0.4	396
Kampong Speu	94.3	96.0	98.2	91.3	0.7	843
Kampong Thom	98.9	98.7	99.4	97.9	0.3	572
Kandal	98.9	95.3	97.1	92.9	0.3	870
Kratie	69.2	94.0	96.6	63.7	0.9	359
Phnom Penh	70.8	90.5	95.6	65.0	1.0	1,084
Prey Veng	99.5	98.7	99.8	98.1	0.0	889
Pursat	93.0	93.0	97.8	86.6	0.4	425
Siem Reap	89.3	90.6	94.6	77.8	0.0	765
Svay Rieng	89.8	90.4	97.7	80.6	0.2	483
Takeo	80.1	80.6	79.8	76.1	17.0	677
Otdar Meanchey	98.2	90.2	99.6	89.5	0.2	218
Battambang/Pailin	89.9	88.4	92.4	79.0	1.3	890
Kampot/Kep	95.8	89.1	97.8	85.9	0.6	574
Preah Sihanouk/ Koh Kong	99.4	99.3	99.6	98.8	0.0	266
Preah Vihear/ Stung Treng	81.0	89.8	96.0	75.1	1.6	314
Mondul Kiri/ Ratanak Kiri	95.7	95.0	95.3	91.5	1.7	281
**Education**						
No education	91.5	92.2	94.8	86.1	2.7	1,774
Primary	92.3	94.2	96.6	87.2	1.1	6,399
Secondary and higher	90.0	92.9	96.9	85.0	1.4	3,726
**Wealth quintile**						
Lowest	92.6	94.1	96.7	86.9	0.8	2,294
Second	92.3	93.7	96.6	87.3	1.4	2,404
Middle	93.8	93.4	95.9	88.7	1.8	2,365
Fourth	92.5	92.9	96.1	87.7	2.4	2,393
Highest	86.3	93.4	96.8	81.3	0.7	2,443
Total	91.5	93.5	96.4	86.3	1.4	11,898

Note: Total includes 1 missing case for employment.

Table 19.5.2 presents similar data on men's participation in decisions about their own health care and about major household purchases by background characteristics. Seventy-five percent of men participate in decisions about their own health care, and 82 percent participate in decisions about major household purchases; only 8 percent do not make decisions on either of the two issues.

Participation in both decisions does not have a clear association with age. Men who are employed for cash are less likely than those who are not employed for cash to participate in both decisions. Men who

live in urban areas and have a secondary education or higher are more likely than other men to participate in both decisions. There is no clear pattern in decision making by level of wealth. Only 16 percent of men from Banteay Meanchey participate in making decisions on both issues, while more than 90 percent of men from Kampong Chhnang, Kampong Thom, Pursat, Siem Reap, and Otdar Meanchey say they make decisions on both issues.

Table 19.5.2 Men's participation in decision making by background characteristics

Percentage of currently married men age 15-49 who usually make specific decisions either alone or jointly with their wife, by background characteristics, Cambodia 2014

Background characteristic	Specific decisions: Man's own health	Specific decisions: Making major household purchases	Both decisions	Neither of the two decisions	Number of men
**Age**					
15-19	*	*	*	*	27
20-24	75.0	83.8	66.0	7.3	308
25-29	74.4	80.8	62.7	7.6	591
30-34	76.1	83.1	65.6	6.3	832
35-39	78.3	78.5	66.1	9.3	529
40-44	72.6	84.0	64.7	8.1	572
45-49	73.2	83.1	65.7	9.3	545
**Employment (last 12 months)**					
Not employed	*	*	*	*	14
Employed for cash	74.3	81.9	64.2	8.1	3,238
Employed not for cash	88.4	84.6	76.6	3.6	153
**Number of living children**					
0	70.3	81.5	60.3	8.4	333
1-2	75.8	83.5	66.2	6.9	1,670
3-4	74.6	79.6	63.3	9.2	1,046
5+	76.4	83.5	67.8	7.9	357
**Residence**					
Urban	77.9	86.4	69.3	5.0	465
Rural	74.5	81.4	64.2	8.3	2,940
**Province**					
Banteay Meanchey	15.6	94.0	15.6	6.0	131
Kampong Cham	86.5	75.8	70.8	8.5	474
Kampong Chhnang	98.9	96.9	96.0	0.2	115
Kampong Speu	89.0	70.8	63.0	3.2	221
Kampong Thom	97.1	99.0	97.0	0.8	146
Kandal	47.7	58.0	32.3	26.6	288
Kratie	70.1	94.8	68.6	3.7	95
Phnom Penh	83.0	87.5	73.0	2.5	279
Prey Veng	37.9	82.8	37.4	16.8	248
Pursat	98.2	94.7	93.6	0.7	109
Siem Reap	99.7	98.9	98.9	0.3	229
Svay Rieng	95.9	89.2	86.5	1.5	130
Takeo	66.4	69.0	48.1	12.6	214
Otdar Meanchey	99.5	93.6	93.1	0.0	67
Battambang/Pailin	46.8	79.4	42.3	16.0	247
Kampot/Kep	85.9	96.9	84.0	1.1	173
Preah Sihanouk/ Koh Kong	93.6	65.2	63.3	4.4	75
Preah Vihear/ Stung Treng	66.8	80.6	55.0	7.6	77
Mondul Kiri/ Ratanak Kiri	98.4	71.8	70.9	0.7	87
**Education**					
No education	74.7	77.6	62.3	10.0	273
Primary	71.8	81.6	61.8	8.5	1,618
Secondary and higher	78.4	83.4	68.7	6.8	1,513
**Wealth quintile**					
Lowest	77.6	81.0	67.4	8.8	618
Second	76.3	84.6	67.3	6.4	665
Middle	69.8	81.0	61.1	10.3	709
Fourth	72.7	77.9	59.1	8.5	735
Highest	79.1	86.3	70.5	5.1	677
Total	75.0	82.1	64.9	7.8	3,405

Note: An asterisk indicates that a figure is based on fewer than 25 unweighted cases and has been suppressed.

19.4 OWNERSHIP OF ASSETS

Ownership and control of assets by women and men influence their individual participation in development processes at all levels. Lack of assets makes women vulnerable to various forms of violence and lessens their decision-making power in the household. Tradition and low economic status limit women's ownership of productive assets such as land and housing. Ownership of assets confers additional economic value, status, and bargaining power.

Table 19.6.1 shows the percent distribution of women age 15-49 by ownership of a house and land, according to background characteristics. Owning a house is more common among women than owning land. Overall, 62 percent of women own a house and 54 percent own land. The majority of women who do own assets own them jointly; 43 percent of women own a house jointly, and 35 percent own land jointly.

There are variations in ownership of a house and land by age, residence, region, education, and wealth. Ownership of houses and land increases with age. Eighty percent of young women age 15-19 do not own a house, and 82 percent do not own land. Ownership of a house or land is more common in rural than in urban areas: 58 percent and 34 percent of urban and rural women, respectively, do not own a house, and 65 percent and 41 percent, respectively, do not own land.

Fifteen percent of women in Kandal own a house alone and 15 percent own land alone, among the highest percentages in the country. Women in Phnom Penh are most likely not to own a house (65 percent), and women in Preah Sihanouk/Koh Kong are most likely not to own land (80 percent). The likelihood of owning either a house or land decreases with increasing education. The percentage of women with a secondary education or higher who do not own a house (54 percent) is more than double that of women with no education (21 percent). Fifty-three percent of women in the highest wealth quintile do not own a house, as compared with 29 percent of women in the lowest quintile. In addition, 60 percent of women in the highest quintile do not own land, compared with 39 percent in the lowest quintile.

The results indicate that tradition is likely to play a larger role in asset ownership than women's socioeconomic status. These findings might be explained by the fact that in general respondents who live in urban areas are more educated and wealthier than their rural counterparts, and they are probably also more likely to rent a place to live and to not own any land.

The pattern of ownership of land among men is similar to that among women with the exception that slightly fewer men than women own a house and land (Table 19.6.2). Overall, 49 percent of men age 15-49 do not own a house (compared with 39 percent of women) and 49 percent do not own land (compared with 46 percent of women). As is true for women, ownership of both housing and land increases with age among men. A larger proportion of men in rural areas own a house and land than their counterparts in urban areas. Men in Phnom Penh are most likely not to own a house (72 percent) or land (64 percent). Men in Banteay Meanchey are least likely not to own a house (34 percent), and men in Kampong Speu are least likely not to own land (32 percent). Similar to women, education and wealth are negatively associated with land and house ownership among men.

Table 19.6.1 Ownership of assets: Women

Percent distribution of women age 15-49 by ownership of housing and land, according to background characteristics, Cambodia 2014

Background characteristic	Percentage who own a house: Alone	Jointly	Alone and jointly	Percent-age who do not own a house	Missing	Total	Percentage who own land: Alone	Jointly	Alone and jointly	Percent-age who do not own land	Missing	Total	Number
**Age**													
15-19	1.9	13.8	4.1	80.2	0.0	100.0	3.0	11.2	3.8	82.0	0.0	100.0	2,893
20-24	4.2	25.3	7.2	63.3	0.0	100.0	7.8	22.6	5.9	63.8	0.0	100.0	3,017
25-29	5.8	43.2	9.8	41.2	0.0	100.0	10.1	35.3	7.4	47.3	0.0	100.0	2,836
30-34	7.2	55.5	13.1	24.2	0.0	100.0	10.2	44.6	11.3	33.9	0.0	100.0	3,046
35-39	12.4	61.8	12.5	13.3	0.0	100.0	12.8	49.3	10.2	27.6	0.0	100.0	1,839
40-44	13.8	61.5	13.8	10.9	0.0	100.0	15.0	50.0	11.5	23.6	0.0	100.0	2,030
45-49	16.7	59.9	15.0	8.3	0.1	100.0	17.8	48.7	13.1	20.2	0.2	100.0	1,916
**Residence**													
Urban	6.2	30.0	5.7	58.1	0.0	100.0	7.1	24.2	3.6	65.1	0.0	100.0	3,251
Rural	8.3	46.3	11.3	34.0	0.0	100.0	10.9	37.9	9.7	41.4	0.0	100.0	14,327
**Province**													
Banteay Meanchey	7.1	52.7	7.9	32.3	0.0	100.0	8.4	48.1	7.9	35.6	0.0	100.0	689
Kampong Cham	8.8	43.9	3.4	43.8	0.0	100.0	9.2	36.5	3.1	51.0	0.1	100.0	2,021
Kampong Chhnang	5.3	43.5	19.7	31.6	0.0	100.0	3.9	28.8	18.9	48.4	0.0	100.0	662
Kampong Speu	8.7	70.9	7.2	13.1	0.0	100.0	16.3	62.1	6.0	15.5	0.0	100.0	1,196
Kampong Thom	7.1	53.9	3.7	35.3	0.0	100.0	6.4	48.2	1.7	43.8	0.0	100.0	851
Kandal	15.1	47.0	15.9	22.0	0.0	100.0	15.3	27.3	10.4	46.9	0.0	100.0	1,330
Kratie	3.4	41.7	22.2	32.8	0.0	100.0	6.1	37.8	21.3	34.9	0.0	100.0	488
Phnom Penh	6.5	25.7	3.0	64.8	0.0	100.0	7.8	22.5	1.5	68.3	0.0	100.0	1,994
Prey Veng	8.1	14.8	39.4	37.7	0.0	100.0	7.8	15.0	38.5	38.6	0.0	100.0	1,188
Pursat	8.4	36.4	12.7	42.5	0.0	100.0	16.8	28.1	10.6	44.5	0.0	100.0	631
Siem Reap	8.3	45.7	15.6	30.3	0.1	100.0	12.5	19.2	11.7	56.4	0.1	100.0	1,137
Svay Rieng	6.2	59.8	7.2	26.9	0.0	100.0	19.4	49.6	5.2	25.8	0.0	100.0	654
Takeo	7.7	47.2	7.1	38.1	0.0	100.0	9.0	45.5	6.4	39.1	0.0	100.0	1,082
Otdar Meanchey	6.8	54.1	12.5	26.6	0.0	100.0	5.9	45.9	10.7	37.5	0.0	100.0	294
Battambang/Pailin	7.6	42.1	1.2	49.1	0.0	100.0	11.8	36.0	0.9	51.2	0.0	100.0	1,333
Kampot/Kep	7.5	47.7	10.6	34.3	0.0	100.0	9.3	44.9	8.9	36.9	0.0	100.0	770
Preah Sihanouk/ Koh Kong	4.5	22.4	14.3	58.8	0.0	100.0	3.9	9.8	6.7	79.6	0.0	100.0	422
Preah Vihear/ Stung Treng	8.2	48.6	1.7	41.5	0.0	100.0	10.1	48.5	1.1	40.4	0.0	100.0	462
Mondul Kiri/ Ratanak Kiri	3.5	51.7	2.1	42.7	0.0	100.0	4.0	51.5	1.8	42.7	0.0	100.0	372
**Education**													
No education	11.0	55.0	12.5	21.3	0.1	100.0	10.7	41.4	10.8	37.0	0.1	100.0	2,250
Primary	8.7	49.4	12.1	29.8	0.0	100.0	11.3	40.6	10.2	37.9	0.0	100.0	8,281
Secondary and higher	6.0	32.3	7.5	54.2	0.0	100.0	8.8	27.3	6.1	57.8	0.0	100.0	7,047
**Wealth quintile**													
Lowest	9.8	50.5	10.9	28.8	0.0	100.0	11.3	40.1	9.6	39.0	0.0	100.0	3,143
Second	8.5	47.5	11.8	32.2	0.0	100.0	10.5	39.1	10.0	40.4	0.1	100.0	3,314
Middle	7.3	44.7	11.6	36.3	0.0	100.0	11.1	36.6	10.5	41.8	0.0	100.0	3,381
Fourth	8.3	42.8	11.1	37.8	0.0	100.0	11.2	35.0	9.4	44.3	0.0	100.0	3,612
Highest	6.1	33.8	6.9	53.2	0.0	100.0	7.6	28.2	4.6	59.7	0.0	100.0	4,128
Total	7.9	43.3	10.3	38.5	0.0	100.0	10.2	35.4	8.6	45.8	0.0	100.0	17,578

Table 19.6.2 Ownership of assets: Men

Percent distribution of men age 15-49 by ownership of housing and land, according to background characteristics, Cambodia 2014

	Percentage who own a house:					Percentage who own land:						
Background characteristic	Alone	Jointly	Alone and jointly	Percent-age who do not own a house	Total	Alone	Jointly	Alone and jointly	Percent-age who do not own land	Missing	Total	Number
**Age**												
15-19	0.1	2.8	1.6	95.5	100.0	2.9	2.4	1.9	92.8	0.0	100.0	926
20-24	2.2	11.1	2.0	84.7	100.0	7.9	11.2	4.0	76.9	0.0	100.0	835
25-29	2.1	32.0	6.7	59.2	100.0	9.9	30.5	5.5	54.1	0.0	100.0	815
30-34	5.4	47.6	15.0	32.0	100.0	11.1	42.1	14.4	32.3	0.0	100.0	907
35-39	5.0	63.7	16.6	14.8	100.0	8.3	54.6	14.8	22.3	0.0	100.0	556
40-44	8.5	63.7	19.0	8.8	100.0	8.9	60.3	14.8	15.5	0.4	100.0	595
45-49	5.3	68.6	21.4	4.7	100.0	12.2	58.5	15.8	13.5	0.0	100.0	556
**Residence**												
Urban	1.9	15.5	15.1	67.5	100.0	5.3	14.6	16.5	63.6	0.0	100.0	869
Rural	4.1	41.4	9.6	44.9	100.0	9.1	37.2	7.9	45.7	0.0	100.0	4,321
**Province**												
Banteay Meanchey	0.4	45.0	20.4	34.2	100.0	1.5	42.7	20.8	34.9	0.0	100.0	192
Kampong Cham	11.0	40.3	4.2	44.5	100.0	15.6	27.6	0.9	55.6	0.3	100.0	663
Kampong Chhnang	3.8	35.4	14.4	46.4	100.0	7.2	35.9	13.0	43.9	0.0	100.0	182
Kampong Speu	3.9	42.5	12.2	41.4	100.0	35.1	19.3	13.9	31.6	0.0	100.0	323
Kampong Thom	1.1	46.6	5.6	46.7	100.0	1.1	44.4	5.2	49.2	0.0	100.0	232
Kandal	9.3	37.8	10.9	42.0	100.0	8.3	36.0	7.7	48.0	0.0	100.0	413
Kratie	3.3	28.9	15.3	52.5	100.0	4.8	31.3	9.4	54.5	0.0	100.0	143
Phnom Penh	1.3	3.8	22.5	72.4	100.0	5.3	5.3	25.6	63.8	0.0	100.0	550
Prey Veng	4.1	10.6	45.3	40.0	100.0	18.7	9.7	33.4	38.3	0.0	100.0	342
Pursat	4.8	39.1	4.2	51.8	100.0	7.7	37.0	2.1	53.3	0.0	100.0	184
Siem Reap	1.0	41.6	7.0	50.3	100.0	1.8	40.2	6.1	51.9	0.0	100.0	337
Svay Rieng	0.0	54.8	1.1	44.1	100.0	1.2	65.8	0.1	32.9	0.0	100.0	183
Takeo	3.1	51.5	0.4	45.0	100.0	9.6	40.9	4.8	44.7	0.0	100.0	334
Otdar Meanchey	0.3	52.8	0.3	46.6	100.0	0.3	50.4	0.6	48.7	0.0	100.0	99
Battambang/Pailin	0.9	42.1	0.0	57.0	100.0	2.4	43.2	0.0	54.4	0.0	100.0	405
Kampot/Kep	0.5	60.5	1.4	37.6	100.0	0.5	59.0	1.8	38.7	0.0	100.0	241
Preah Sihanouk/ Koh Kong	1.9	28.8	9.4	60.0	100.0	2.0	27.9	8.4	61.7	0.0	100.0	120
Preah Vihear/ Stung Treng	1.6	51.5	2.9	44.0	100.0	1.6	51.9	2.6	43.9	0.0	100.0	112
Mondul Kiri/ Ratanak Kiri	0.8	46.6	1.4	51.2	100.0	0.5	47.4	0.9	51.2	0.0	100.0	134
**Education**												
No education	3.8	53.3	12.4	30.5	100.0	6.3	45.3	11.4	37.0	0.0	100.0	324
Primary	5.0	45.7	10.1	39.3	100.0	9.2	40.5	8.8	41.4	0.1	100.0	2,167
Secondary and higher	2.7	28.3	10.6	58.3	100.0	8.2	26.3	9.6	55.9	0.0	100.0	2,699
**Wealth quintile**												
Lowest	6.1	45.3	8.5	40.2	100.0	8.7	39.2	7.4	44.4	0.2	100.0	901
Second	5.1	45.9	9.1	39.9	100.0	8.5	40.9	6.6	44.0	0.0	100.0	954
Middle	2.2	39.7	10.0	48.0	100.0	12.2	34.7	8.5	44.6	0.0	100.0	1,040
Fourth	3.3	37.3	9.6	49.8	100.0	8.6	34.2	7.7	49.5	0.0	100.0	1,124
Highest	2.6	21.1	14.7	61.6	100.0	4.9	20.9	15.6	58.6	0.0	100.0	1,171
Total	3.7	37.1	10.5	48.6	100.0	8.5	33.4	9.4	48.7	0.0	100.0	5,190

19.5 ATTITUDES TOWARDS WIFE BEATING

Another measure of women's empowerment derives from the idea that gender equity is essential to empowerment. Attitudes that view the beating of wives by husbands as justified are indicative of women's lower status. They signify acceptance of norms that give men the right to use force against women, which is a violation of women's human rights. Violence against women has serious consequences for their mental and physical well-being, including their reproductive and sexual health (Heise et al., 1999).

The 2014 CDHS gathered information on women's and men's attitudes toward wife beating, a proxy for women's status. Respondents who believe that a husband is justified in hitting or beating his wife for any of the specified reasons may believe that women are low in status both absolutely and relative to men. Such a perception could act as a barrier for women in accessing health care for themselves and their children; moreover, it could affect women's attitudes towards contraceptive use and affect their general well-being. Respondents were asked whether a husband is justified in beating his wife under a series of circumstances: if the wife burns the food, if she argues with him, if she goes out without telling him, if she

neglects the children, if she refuses to have sex with him, and if she asks him to use a condom. Table 19.7.1 summarizes women's attitudes towards wife beating in these six specific circumstances. Table 19.7.2 summarizes men's attitudes.

Table 19.7.1 Attitude toward wife beating: Women

Percentage of all women age 15-49 who agree that a husband is justified in hitting or beating his wife for specific reasons, by background characteristics, Cambodia 2014

Background characteristic	Husband is justified in hitting or beating his wife if she:						Percentage who agree with at least one specified reason	Number
	Burns the food	Argues with him	Goes out without telling him	Neglects the children	Refuses to have sexual intercourse with him	Asks him to use a condom		
**Age**								
15-19	10.3	21.6	24.8	38.9	9.5	1.9	45.9	2,893
20-24	8.6	18.8	25.8	39.1	8.3	1.7	44.8	3,017
25-29	8.6	20.2	28.0	40.2	9.6	1.9	46.4	2,836
30-34	11.6	23.2	32.5	46.6	12.2	1.9	51.9	3,046
35-39	13.3	26.8	36.6	47.0	15.9	1.9	54.4	1,839
40-44	15.7	30.7	38.7	50.2	18.9	2.1	57.9	2,030
45-49	18.3	32.0	40.0	48.8	21.0	1.1	57.4	1,916
**Employment (last 12 months)**								
Not employed	11.4	21.5	28.0	41.7	10.8	1.6	48.0	3,599
Employed for cash	11.8	24.4	32.1	43.5	13.1	1.6	50.2	12,702
Employed not for cash	11.9	24.9	33.3	51.2	15.6	4.3	59.1	1,273
**Number of living children**								
0	9.8	19.3	23.5	36.4	9.2	1.6	43.0	5,698
1-2	10.4	22.8	31.4	44.3	11.9	1.9	50.4	6,622
3-4	14.0	28.0	38.6	50.3	16.2	1.9	57.1	3,893
5+	19.7	36.1	42.8	52.5	22.5	2.3	62.1	1,365
**Marital status**								
Never married	9.3	18.0	21.7	34.9	8.3	1.5	41.1	4,428
Married or living together	12.4	26.0	35.1	47.2	14.1	2.0	53.9	11,898
Divorced/separated/ widowed	13.9	24.9	29.7	42.1	16.0	1.5	50.1	1,252
**Residence**								
Urban	6.5	14.4	21.5	32.7	6.3	0.3	37.2	3,251
Rural	12.9	26.0	33.5	46.2	14.2	2.2	53.4	14,327
**Province**								
Banteay Meanchey	29.3	47.1	59.9	76.2	40.4	2.4	82.1	689
Kampong Cham	12.2	28.4	33.5	49.6	13.0	3.9	59.4	2,021
Kampong Chhnang	4.5	12.6	24.8	30.8	6.3	3.2	36.8	662
Kampong Speu	15.6	29.8	36.8	45.3	18.6	1.8	54.6	1,196
Kampong Thom	10.1	13.0	20.0	20.4	8.7	0.0	22.9	851
Kandal	8.7	28.2	35.5	45.8	14.9	1.1	52.2	1,330
Kratie	15.6	27.9	45.1	61.0	12.5	9.6	71.8	488
Phnom Penh	5.1	9.5	16.8	27.6	4.0	0.0	32.2	1,994
Prey Veng	1.2	22.5	27.3	28.3	2.8	0.9	30.0	1,188
Pursat	26.5	38.1	46.7	60.1	26.0	2.2	68.0	631
Siem Reap	4.8	10.9	11.9	39.5	10.4	2.9	47.5	1,137
Svay Rieng	21.4	38.8	50.8	62.0	21.3	6.5	67.3	654
Takeo	7.4	19.5	35.6	41.8	10.6	1.7	47.9	1,082
Otdar Meanchey	8.0	37.2	22.0	33.9	10.4	0.1	50.3	294
Battambang/Pailin	11.8	19.5	24.6	50.7	9.6	0.0	56.8	1,333
Kampot/Kep	18.7	26.6	32.9	49.0	14.6	0.0	56.5	770
Preah Sihanouk/Koh Kong	11.8	21.6	34.9	44.9	10.5	0.5	50.0	422
Preah Vihear/Stung Treng	23.9	33.5	41.9	55.8	28.3	0.3	65.9	462
Mondul Kiri/Ratanak Kiri	21.1	35.5	41.1	42.0	3.2	0.0	46.0	372
**Education**								
No education	17.6	31.5	38.0	50.3	20.1	2.1	58.4	2,250
Primary	14.4	27.7	36.6	49.4	15.5	2.1	56.6	8,281
Secondary and higher	6.8	17.0	22.9	34.9	7.2	1.5	40.5	7,047
**Wealth quintile**								
Lowest	17.5	30.3	37.3	50.1	17.3	3.3	57.6	3,143
Second	14.3	28.8	34.1	47.9	15.5	2.1	54.9	3,314
Middle	12.4	26.0	33.2	46.0	13.9	2.4	54.1	3,381
Fourth	10.3	22.9	33.8	44.8	12.5	1.2	51.5	3,612
Highest	6.1	14.2	20.8	32.6	6.5	0.6	37.3	4,128
Total	11.7	23.9	31.3	43.7	12.8	1.8	50.4	17,578

Note: Total includes 4 missing cases on employment.

Half of women believe that a husband is justified in beating his wife for at least one of the six specified reasons. The proportions of women who believe that wife beating is justified if the wife argues with her husband, goes out without telling him, or neglects the children are 24 percent, 31 percent, and 44 percent, respectively. Slightly more than 1 in 10 women say wife beating is justified if the wife burns the food (12 percent) and refuses to have sexual intercourse with her husband (13 percent). Very few women (2 percent) believe that wife beating is justified if the wife asks her husband to use a condom.

Overall, women in rural areas, those with no schooling or a primary school education, and those in the lower wealth quintiles are more likely than other women to agree with at least one reason for wife beating. In addition, women who are employed but not paid in cash and those with five or more children are more likely than other women to agree with at least one of the reasons for wife beating. Women living in Banteay Meanchey and Kratie (82 percent and 72 percent, respectively) are most likely to agree with at least one specified reason for wife beating, and those living in Kampong Thom and Prey Veng (23 percent and 30 percent, respectively) are least likely.

Table 19.7.2 shows that men are about half as likely as women to believe that a husband is justified in beating his wife for any of the specified reasons (27 percent versus 50 percent). Only 19 percent of men age 15-49 believe that a husband is justified in beating his wife if she neglects the children, as compared with 44 percent of women. Four percent of men agree that wife beating is justified if the wife refuses to have sex with her husband, and only 2 percent believe that a husband is justified in beating his wife if she burns the food or asks him to use a condom. Eleven percent and 13 percent of men, respectively, say that a husband is justified in beating his wife if she argues with him or goes out without telling him.

There are differences among men in the percentage who believe wife beating is justified for any of the specified reasons. Formerly married men, men with five or more children, men with no schooling, men in rural areas, and men in the lowest household wealth quintile are more likely to agree with at least one of the reasons for wife beating than other men. Men who reside in Banteay Meanchey are most likely to agree with at least one of the reasons for wife beating.

Table 19.7.2 Attitude toward wife beating: Men

Percentage of all men age 15-49 who agree that a husband is justified in hitting or beating his wife for specific reasons, by background characteristics, Cambodia 2014

Background characteristic	Husband is justified in hitting or beating his wife if she: Burns the food	Argues with him	Goes out without telling him	Neglects the children	Refuses to have sexual intercourse with him	Asks him to use a condom	Percentage who agree with at least one specified reason	Number
**Age**								
15-19	2.2	11.3	10.8	18.5	4.8	3.4	26.4	926
20-24	2.0	9.9	10.1	15.9	2.3	2.0	24.8	835
25-29	0.6	7.0	8.9	15.4	1.7	1.5	20.8	815
30-34	1.4	10.6	14.2	20.7	2.9	2.0	27.4	907
35-39	2.1	16.3	14.9	19.9	5.5	1.5	29.0	556
40-44	2.7	13.1	14.8	22.7	5.1	3.1	30.9	595
45-49	2.5	12.9	16.6	23.2	6.6	1.3	29.2	556
**Employment (last 12 months)**								
Not employed	2.1	5.9	5.7	12.1	3.0	2.9	17.2	372
Employed for cash	1.8	11.8	12.9	19.4	3.8	2.1	27.2	4,395
Employed not for cash	1.9	9.3	13.9	22.8	5.4	1.7	27.9	423
**Number of living children**								
0	1.9	9.5	9.6	16.3	3.6	2.4	24.0	2,043
1-2	1.7	10.1	12.2	18.2	3.3	1.8	24.8	1,725
3-4	1.5	14.5	15.4	24.3	4.2	2.0	31.0	1,058
5+	3.4	15.9	21.7	24.7	7.3	3.0	35.3	364
**Marital status**								
Never married	2.0	10.3	9.9	16.5	4.0	2.8	24.4	1,663
Married or living together	1.7	11.5	13.8	20.3	3.7	1.9	27.4	3,405
Divorced/separated/ widowed	4.0	15.6	12.2	22.8	7.2	1.8	29.7	122
**Residence**								
Urban	1.0	4.3	5.6	8.3	1.6	0.5	12.9	869
Rural	2.0	12.6	13.9	21.3	4.3	2.5	29.3	4,321
**Province**								
Banteay Meanchey	6.5	41.6	23.6	49.6	19.6	4.0	64.2	192
Kampong Cham	3.1	14.2	14.0	20.9	5.4	4.4	35.1	663
Kampong Chhnang	3.2	19.3	14.5	26.2	3.5	2.0	31.3	182
Kampong Speu	3.0	11.9	14.9	21.0	5.9	2.3	28.6	323
Kampong Thom	0.0	1.4	3.8	1.0	0.0	0.0	5.7	232
Kandal	1.0	16.2	24.6	28.1	4.0	2.3	30.3	413
Kratie	0.0	3.7	11.2	6.4	5.2	10.0	25.9	143
Phnom Penh	0.7	1.4	2.9	3.9	0.8	0.3	6.5	550
Prey Veng	1.0	10.4	22.9	29.6	1.2	1.0	36.1	342
Pursat	3.2	20.6	22.7	39.3	3.2	1.3	46.7	184
Siem Reap	0.0	8.8	8.6	5.8	2.0	2.9	18.8	337
Svay Rieng	1.5	18.1	11.7	21.3	4.5	5.9	31.8	183
Takeo	5.2	16.3	13.0	41.0	11.1	2.9	49.4	334
Otdar Meanchey	0.1	1.9	0.8	1.6	0.8	0.1	1.9	99
Battambang/Pailin	1.2	4.7	5.6	9.7	0.9	0.3	11.7	405
Kampot/Kep	0.4	7.5	11.1	21.1	0.9	0.0	26.7	241
Preah Sihanouk/Koh Kong	2.7	11.0	16.5	20.9	3.8	0.8	27.3	120
Preah Vihear/Stung Treng	0.9	1.4	0.4	2.7	0.8	0.4	4.6	112
Mondul Kiri/Ratanak Kiri	0.3	3.5	6.9	4.1	0.0	0.0	8.8	134
**Education**								
No education	0.7	16.1	20.3	25.0	2.7	2.1	35.8	324
Primary	2.2	14.6	15.6	22.9	5.6	2.5	32.6	2,167
Secondary and higher	1.7	7.9	9.1	15.4	2.7	1.9	20.5	2,699
**Wealth quintile**								
Lowest	1.3	15.9	14.8	23.2	4.4	3.4	34.7	901
Second	1.8	13.4	12.4	21.3	3.2	2.3	29.5	954
Middle	2.2	12.2	16.2	24.4	5.3	2.3	31.7	1,040
Fourth	3.1	11.9	14.8	21.2	5.2	2.6	28.2	1,124
Highest	0.8	4.1	5.4	7.5	1.6	0.5	11.6	1,171
Total	1.9	11.2	12.5	19.1	3.9	2.2	26.5	5,190

19.6 WOMEN'S EMPOWERMENT INDICATORS

Responses from women as to their participation in making household decisions and their attitude towards wife beating can be summarized into two separate indices.

The first index is the number of decisions in which women participate alone or jointly with their husband (see Table 19.5.1 for the list of decisions). This index ranges in value from 0 to 3 and is positively related to women's empowerment. It reflects the degree of decision-making control that women are able to exercise in areas that affect their lives and environments.

The second index is the number of reasons for which the respondent believes that a husband is justified in beating his wife (see Table 19.7.1 for the list of reasons). This index ranges in value from 0 to 6. A lower score on this indicator is interpreted as reflecting a greater sense of entitlement and self-esteem and higher status.

Table 19.8 presents these two indicators of women's empowerment and how they relate to each other. It shows the percentage of married women age 15-49 who participate in all decision making and the percentage of women who disagree with all of the specified reasons justifying wife beating, by the value on each of the indicators. In general, the expectation is that women who participate in making household decisions are more likely to have gender-egalitarian beliefs.

The findings on women's empowerment are mixed. Looking at the top panel of Table 19.8, one would expect the proportion of women who disagree with all reasons justifying wife beating to increase with the number of decisions in which women participate. However, the percentage is highest among the small number of women who do not participate in any decisions. The second indicator (shown in the bottom panel) shows the expected pattern; women who do not support wife beating for any reason are most likely to participate in all of the decision making in the household (87 percent).

Table 19.8 Indicators of women's empowerment

Percentage of currently married women age 15-49 who participate in all decision making and the percentage who disagree with all of the reasons justifying wife beating, by value on each of the indicators of women's empowerment, Cambodia 2014

Empowerment indicator	Percentage who participate in all decision making	Percentage who disagree with all of the reasons justifying wife beating	Number of women
**Number of decisions in which women participate[1]**			
0	na	70.5	169
1-2	na	40.5	1,456
3	na	47.1	10,273
**Number of reasons for which wife beating is justified[2]**			
0	87.2	na	5,543
1-2	85.3	na	3,360
3-4	86.5	na	2,228
5-6	84.0	na	767
Total	86.3	46.6	11,898

na = Not applicable
[1] See Table 19.5.1 for the list of decisions.
[2] See Table 19.7.1 for the list of reasons.

19.7 Current Use of Contraception by Women's Status

A woman's desire and ability to control her fertility and her choice of contraceptive method are in part affected by her status in the household and her own sense of empowerment. A woman who feels that she is unable to control her life may be less likely to feel she can make and carry out decisions about her fertility. She may also feel the need to choose methods that are less obvious or do not depend on her husband's cooperation. Table 19.9 shows the distribution of currently married women by contraceptive method used, according to the two empowerment indicators.

The findings indicate that there is a positive relationship between use of contraception and participation in household decision making. For example, current use of any contraceptive method increases from 54 percent among women who do not participate in any of the household decisions to 57 percent among women who participate in all three household decisions. Women who believe that wife beating is justified for five or six specified reasons are slightly less likely to use any method of contraception than other women.

Table 19.9 Current use of contraception by women's empowerment

Percent distribution of currently married women age 15-49 by current contraceptive method, according to selected indicators of women's status, Cambodia 2014

Empowerment indicator	Any method	Any modern method	Modern methods: Female sterilization	Male sterilization	Temporary modern female methods[1]	Male condom	Any traditional method	Not currently using	Total	Number of women
**Number of decisions in which women participate[2]**										
0	53.7	43.6	4.8	0.0	35.6	3.1	10.2	46.3	100.0	169
1-2	54.7	34.4	2.7	0.1	29.8	1.9	20.3	45.3	100.0	1,456
3	56.6	39.4	3.1	0.1	34.1	2.1	17.2	43.4	100.0	10,273
**Number of reasons for which wife beating is justified[3]**										
0	55.9	37.8	2.9	0.1	32.5	2.4	18.1	44.1	100.0	5,543
1-2	57.2	39.6	3.8	0.0	33.5	2.3	17.6	42.8	100.0	3,360
3-4	57.1	39.6	2.4	0.3	35.4	1.4	17.5	42.9	100.0	2,228
5-6	53.4	40.7	2.7	0.1	36.7	1.2	12.8	46.6	100.0	767
Total	56.3	38.8	3.0	0.1	33.6	2.1	17.5	43.7	100.0	11,898

Note: If more than one method is used, only the most effective method is considered in this tabulation.
[1] Pill, IUD, injectables, implants, female condom, diaphragm, foam/jelly, and lactational amenorrhea method
[2] See Table 19.5.1 for the list of decisions.
[3] See Table 19.7.1 for the list of reasons.

19.8 IDEAL FAMILY SIZE AND UNMET NEED BY WOMEN'S STATUS

The ability of women to make household decisions has important implications for their fertility preferences and the practice of family planning. Increases in women's status and empowerment are recognized as important in efforts to reduce fertility.

Table 19.10 shows how women's ideal family size and unmet need for family planning are related to the two indicators of women's status. The findings indicate that there is little variation in ideal family size and decision making; however, mean ideal family size increases slightly as number of reasons justifying wife beating increases. With respect to the relationship between unmet need and women's empowerment indicators, the findings show that unmet need is higher among women who participate in at least one of the household decisions than among women who do not participate in any. Women who believe that wife beating is justified for five or six specified reasons are more likely to have an unmet need for family planning than other women.

Table 19.10 Ideal number of children and unmet need for family planning by women's empowerment

Mean ideal number of children for women age 15-49 and the percentage of currently married women age 15-49 with an unmet need for family planning, by indicators of women's empowerment, Cambodia 2014

Empowerment indicator	Mean ideal number of children[1]	Number of women	Percentage of currently married women with an unmet need for family planning[2]: For spacing	For limiting	Total	Number of women
**Number of decisions in which women participate[3]**						
0	3.1	169	3.9	5.6	9.5	169
1-2	3.2	1,445	6.7	6.9	13.5	1,456
3	3.3	10,186	5.3	7.1	12.4	10,273
**Number of reasons for which wife beating is justified[4]**						
0	2.9	8,601	5.9	6.3	12.2	5,543
1-2	3.1	4,765	5.6	6.6	12.2	3,360
3-4	3.2	2,931	4.2	8.0	12.3	2,228
5-6	3.3	998	4.5	11.6	16.0	767
Total	3.1	17,295	5.4	7.0	12.5	11,898

[1] Mean excludes respondents who gave non-numeric responses.
[2] See Table 10.3 for the definition of unmet need for family planning.
[3] Restricted to currently married women. See Table 19.5.1 for the list of decisions.
[4] See Table 19.7.1 for the list of reasons.

19.9 REPRODUCTIVE HEALTH CARE AND WOMEN'S EMPOWERMENT STATUS

Table 19.11 examines whether women's use of antenatal, delivery, and postnatal care services from health professionals varies by their status as measured on the two indicators of women's empowerment. In societies where health care is widespread, women's empowerment may not affect their access to reproductive health services; in other societies, however, increased empowerment is likely to enhance women's ability to seek out and use health services to better meet their own reproductive health goals, including the goal of safe motherhood.

Table 19.11 shows that use of reproductive health services is not clearly related to the number of decisions in which women participate. However, mothers who agree with none of the reasons justifying wife beating are more likely to have received antenatal care from health personnel (97 percent) and assistance at delivery (93 percent) than women who agree with at least one reason. Similarly, the proportion of women who received postnatal care from a skilled provider soon after delivery is highest among those who do not justify wife beating for any reason (91 percent) and lowest among those who feel wife beating is justified for five or six reasons (83 percent).

Table 19.11 Reproductive health care by women's empowerment

Percentage of women age 15-49 with a live birth in the five years preceding the survey who received antenatal care, delivery assistance, and postnatal care from health personnel for the most recent birth, by indicators of women's empowerment, Cambodia 2014

Empowerment indicator	Percentage receiving antenatal care from a skilled provider[1]	Percentage receiving delivery care from a skilled provider[1]	Received postnatal care from health personnel within the first two days since delivery[2]	Number of women with a child born in the last five years
**Number of decisions in which women participate[3]**				
0	97.9	96.4	94.4	75
1-2	94.7	89.4	88.0	722
3	95.5	90.4	88.6	4,881
**Number of reasons for which wife beating is justified[4]**				
0	97.3	92.7	90.5	2,874
1-2	94.5	90.5	88.9	1,712
3-4	92.7	86.4	85.2	1,080
5-6	90.6	82.4	82.6	306
Total	95.3	90.4	88.7	5,973

[1] "Skilled provider" includes doctor, nurse, midwife, or auxiliary nurse/midwife.
[2] Includes women who received a postnatal checkup from a doctor, nurse, midwife, community health worker, or traditional birth attendant in the first two days after the birth. Includes women who gave birth in a health facility and those who did not give birth in a health facility.
[3] Restricted to currently married women. See Table 19.5.1 for the list of decisions.
[4] See Table 19.7.1 for the list of reasons.

DOMESTIC VIOLENCE 20

Key Findings

- One in five women age 15-49 have experienced physical violence at least once since age 15, and 9 percent experienced physical violence within the 12 months prior to the survey.
- Six percent of women age 15-49 report having experienced sexual violence at least once in their lifetime.
- Overall, 18 percent of ever-married women age 15-49 report having experienced physical or sexual violence from a spouse.
- Among ever-married women who have experienced spousal violence (physical or sexual), 48 percent reported experiencing physical injuries.
- About two in five women have sought assistance to stop the violence they have experienced.

Gender-based violence is defined as any act that results in, or is likely to result in, physical, sexual, or psychological harm or suffering among women, including threats of such acts and coercion or arbitrary deprivations of liberty, whether occurring in public or in private life (United Nations, 1993; United Nations, 1995). Domestic violence has negative health consequences for victims, especially with respect to the reproductive health of women and the physical, emotional, and mental health of their children. The 2014 CDHS included a domestic violence module for women to assess the problem of domestic violence in Cambodia.

20.1 Measurement of Violence

Collecting valid, reliable, and ethical data on domestic violence poses particular challenges because what constitutes violence or abuse varies across cultures and among individuals. In addition, a culture of silence usually surrounds domestic violence and can affect reporting. The sensitivity of the topic is another issue. Assuring the safety of respondents and interviewers when asking about domestic violence in a familial setting, protecting women who disclose violence, and reducing the risk of double victimization of respondents as they relive their experiences are all specific ethical concerns. The responses to these challenges in the 2014 CDHS are described in the sections that follow.

20.1.1 Use of Valid Measures of Violence

In the 2014 CDHS, information was obtained from ever-married women on violence committed by their current and former husbands and by others. Information was collected from never-married women on violence committed by anyone. Since international research shows that intimate partner violence is one of the most common forms of violence, especially against women, information on spousal violence was measured in more detail than violence committed by other perpetrators. These measurements were made using a shortened, modified version of the Conflict Tactics Scale (Strauss, 1990). Specifically, violence by the current husband/partner for currently married women and by the most recent husband/partner for formerly married women was measured by asking all ever-married women the following set of questions:

Does (did) your (last) husband/partner ever:

(a) Push you, shake you, or throw something at you?

(b) Slap you?

(c) Twist your arm or pull your hair?

(d) Punch you with his/her fist or with something that could hurt you?

(e) Kick you, drag you, or beat you up?

(f) Try to choke you or burn you on purpose?

(g) Threaten or attack you with a knife, gun, or any other weapon?

(h) Physically force you to have sexual intercourse with him/her even when you did not want to?

(i) Physically force you to perform any other sexual acts you did not want to?

(j) Force you with threats or in any other way to perform sexual acts you did not want to?

For every question that a woman answered "yes," she was asked about the frequency of the act in the 12 months preceding the survey (often, sometimes, not at all). A yes answer to one or more of items (a) to (g) above constitutes evidence of physical violence, and a yes answer to item (h), (i), or (j) constitutes evidence of sexual violence.

Similarly, emotional violence among ever-married women was measured with the following questions:

Does (did) your (last) husband/partner ever:

(a) Say or do something to humiliate you in front of others?

(b) Threaten to hurt or harm you or someone close to you?

(c) Insult you or make you feel bad about yourself?

This approach of asking about specific acts to measure different forms of violence has the advantage of not being affected by different understandings of what constitutes a summary term such as "violence." By including a wide range of acts, this approach has the additional advantage of giving the respondent multiple opportunities to disclose any experience of violence.

In addition to these questions asked only of ever-married respondents, all women were asked about physical and sexual violence from persons other than the current or most recent husband/partner. Respondents who answered yes to this question were asked who committed violence against them and the frequency of such violence during the 12 months preceding the survey. Respondents who reported experiencing different forms of violence were asked for the perpetrators of the violence.

Although this approach to questioning is generally considered to be optimal, the possibility of underreporting of violence, particularly sexual violence, cannot be entirely ruled out in any survey, and this survey is no exception.

20.1.2 Ethical Considerations in the 2014 CDHS

In recognition of the challenges in collecting data on violence, the interviewers in the 2014 CDHS were given special training. The training focused on how to ask sensitive questions, ensure privacy, and

build rapport between interviewer and respondent. Rapport with the interviewer, confidentiality, and privacy are all keys to building respondents' confidence so that they can safely share their experiences with the interviewer. Also, placement of questions about violence at the end of the questionnaire provides time for the interviewer to develop a certain degree of intimacy that should further encourage respondents to share their experiences of violence, if any. In addition, the following protections were built into the survey in keeping with the World Health Organization's ethical and safety recommendations for research on domestic violence (WHO, 2001):

1. To maintain confidentiality, only one woman per household was administered the questions on violence. The random selection of one woman was done through a simple selection procedure based on the Kish grid, which was built into the Household Questionnaire (Kish, 1965).

2. As a means of obtaining additional consent beyond the initial consent provided at the start of the interview, the respondent was informed that the questions could be sensitive and was reassured regarding the confidentiality of her responses.

3. The violence module was implemented only if privacy could be obtained. The interviewers were instructed to skip the module, thank the respondent, and end the interview if they could not maintain privacy.

4. A brochure that included information on domestic violence and contact information for service centers across the country was provided to all eligible respondents after the interview was completed, irrespective of whether or not they were selected for the module. This was done to safeguard against identifying the woman selected for the module and to provide information to all respondents so that they could access the services and be informed about what to do in the event of domestic violence.

20.1.3 Subsample for the Violence Module

The domestic violence module was implemented only in the subsample of households selected for the men's survey. Furthermore, in keeping with ethical requirements, only one woman per household was selected for the module, as mentioned above. As a result of these restrictions, a total of 4,307 women age 15-49 (3,245 ever-married women) completed the domestic violence module. Specially constructed weights were used to adjust for the selection of only one woman per household and to ensure that the domestic violence subsample was nationally representative. In total, only 28 women, or 0.6 percent of the subsample selected for the domestic violence module, could not be interviewed because privacy was not possible.

20.2 EXPERIENCE OF PHYSICAL VIOLENCE

Table 20.1 shows the percentages of women who have ever experienced physical violence since age 15 and the percentages who experienced violence during the 12 months preceding the survey, by background characteristics. Twenty percent of women have experienced physical violence since age 15, and 8 percent experienced physical violence in the 12 months prior to the survey. Overall, 2 percent of women reported that they had experienced physical violence often in the past 12 months and 6 percent said they had experienced physical violence sometimes during the same period.

Table 20.1 Experience of physical violence

Percentage of women age 15-49 who have ever experienced physical violence since age 15 and percentage who have experienced violence during the 12 months preceding the survey, by background characteristics, Cambodia 2014

Background characteristic	Percentage who have ever experienced physical violence since age 15[1]	Percentage who have experienced physical violence in the past 12 months: Often	Sometimes	Often or sometimes[2]	Number of women
**Age**					
15-19	6.8	0.9	2.3	3.1	729
20-24	16.1	0.6	5.2	5.8	687
25-29	15.1	0.6	5.1	5.7	647
30-39	26.9	2.7	8.9	11.5	1,260
40-49	27.2	2.8	6.9	9.7	985
**Religion**					
Buddhist	20.1	1.8	6.0	7.7	4,117
Moslem	22.0	0.0	12.9	12.9	96
Christian	(11.7)	(0.0)	(2.0)	(2.0)	47
Other/missing	23.0	3.9	12.0	15.8	46
**Residence**					
Urban	18.4	1.0	2.5	3.6	740
Rural	20.4	1.9	6.9	8.8	3,567
**Province**					
Banteay Meanchey	13.4	0.7	12.0	12.8	166
Kampong Cham	33.2	3.0	14.0	17.0	527
Kampong Chhnang	11.0	0.5	2.6	3.1	173
Kampong Speu	21.0	2.2	4.4	6.6	285
Kampong Thom	15.5	2.2	4.5	6.7	196
Kandal	20.1	1.5	5.6	7.0	325
Kratie	24.9	4.7	5.2	9.9	119
Phnom Penh	18.8	1.2	1.2	2.4	453
Prey Veng	11.8	0.0	4.3	4.3	304
Pursat	19.0	0.8	5.0	5.7	144
Siem Reap	22.6	2.2	9.2	11.4	280
Svay Rieng	11.1	0.4	3.2	3.7	159
Takeo	18.7	4.1	4.4	8.5	267
Otdar Meanchey	17.8	3.8	8.7	12.5	73
Battambang/Pailin	19.3	0.5	4.4	4.9	336
Kampot/Kep	18.9	0.6	6.3	6.9	200
Preah Sihanouk/ Koh Kong	22.4	0.8	6.9	7.8	105
Preah Vihear/ Stung Treng	31.8	3.4	6.8	10.2	103
Mondul Kiri/ Ratanak Kiri	13.9	1.5	5.2	6.7	92
**Marital status**					
Never married	6.6	0.1	1.7	1.8	1,062
Married or living together	23.3	1.9	7.9	9.7	2,977
Divorced/separated/ widowed	37.8	7.0	4.8	11.8	268
**Number of living children**					
0	9.0	0.5	2.2	2.7	1,377
1-2	21.5	1.7	7.2	8.9	1,593
3-4	27.0	2.6	8.3	10.9	968
5+	37.3	4.4	10.3	14.7	370
**Employment**					
Employed for cash	19.6	2.2	6.1	8.3	3,068
Employed not for cash	29.4	1.4	7.8	9.2	342
Not employed	18.2	0.3	5.6	5.9	897
**Education**					
No education	29.9	3.0	11.7	14.6	561
Primary	24.7	2.4	7.9	10.3	1,991
Secondary and higher	11.7	0.6	2.5	3.0	1,755
**Wealth quintile**					
Lowest	28.2	3.2	11.6	14.8	755
Second	22.2	2.9	6.3	9.3	823
Middle	19.3	1.1	7.3	8.4	851
Fourth	16.9	1.1	4.5	5.6	898
Highest	15.7	0.8	2.3	3.1	980
Total	20.1	1.7	6.2	7.9	4,307

Note: Figures in parentheses are based on 25-49 unweighted cases.
[1] Includes violence in the past 12 months. For women who were married before age 15 and who reported physical violence by a spouse, the violence could have occurred before age 15.
[2] Includes women for whom frequency in the past 12 months is not known

The experience of physical violence differs by background characteristics. The percentage of women who have experienced physical violence since age 15 does not vary greatly by urban-rural residence or religion. However, it increases by age from 7 percent among those age 15-19 to 27 percent among those age 30-49. The percentage is higher among women who have five or more children (37 percent) and those who have no education (30 percent) than among other women. Women who are employed but not for cash are more likely than women who are employed for cash and those who are not employed to have experienced physical violence. Thirty-eight percent of women who are divorced, separated, or widowed and 23 percent of currently married women have experienced physical violence since age 15, as compared with 7 percent of never-married women. The percentage of women who have experienced physical violence since age 15 ranges from 16 percent among those in the highest wealth quintile to 28 percent among those in the lowest quintile. Women from Svay Rieng and Kampong Chhnang are least likely to have experienced physical violence since age 15 (11 percent each), and women from Kampong Cham and Preah Vihear/Stung Treng are most likely to have experienced violence (33 percent and 32 percent, respectively).

The percentage of women who experienced physical violence in the past 12 months (often or sometimes) follows a similar pattern by background characteristics as that for physical violence since age 15. It increases as age increases and decreases with increasing education and wealth. Recent physical violence is substantially higher among women who work but not for cash, those who have five or more children, and those who are formerly or currently in a union. However, unlike physical violence since age 15, recent physical violence is considerably higher among women who live in rural areas than those who live in urban areas.

20.3 Perpetrators of Physical Violence

Table 20.2 shows perpetrators of physical violence, according to women's marital status, among those who have experienced physical violence since age 15. Among ever-married women, the most commonly reported perpetrator of physical violence is their current husband or partner (56 percent), followed by their mother/stepmother (23 percent) and former husband/partner (20 percent). These findings indicate a high proportion of spousal violence.

Table 20.2 Persons committing physical violence

Among women age 15-49 who have experienced physical violence since age 15, percentage who report specific persons who committed the violence, according to the respondent's current marital status, Cambodia 2014

Person	Marital status: Ever married	Marital status: Never married	Total
Current husband/partner	56.3	na	51.7
Former husband/partner	19.6	na	18.0
Current boyfriend	0.4	3.1	0.6
Former boyfriend	0.2	0.4	0.2
Father/stepfather	14.3	37.0	16.1
Mother/stepmother	22.7	26.2	23.0
Sister/brother	6.4	30.5	8.4
Daughter/son	0.3	0.0	0.3
Other relative	5.4	8.7	5.7
Mother-in-law	0.1	na	0.1
Other in-law	1.1	na	1.6
Teacher	1.4	3.6	1.6
Employer/someone at work	0.3	0.0	0.3
Police/soldier	0.0	0.2	0.0
Other	1.1	0.9	1.0
Number women who have experienced physical violence since age 15	795	70	865

na = Not applicable

Among never-married women who have experienced physical violence since age 15, the most common perpetrators of violence are their father/ stepfather (37 percent), sister/brother (31 percent), and mother/stepmother (26 percent).

20.4 Experience of Sexual Violence

Table 20.3 shows the percentage of women who have experienced sexual violence ever and in the past 12 months, according to background characteristics.

The results show that 6 percent of women age 15-49 have ever experienced sexual violence, and 3 percent experienced sexual violence in the past 12 months. There are notable variations in the experience of sexual violence by age. Younger women (age 15-19) are less likely to report sexual violence ever (1 percent) and in the past 12 months (less than 1 percent) than older women. Women who have no education are more likely than other women to have experienced sexual violence ever and in the past year. The percentage of women who have ever experienced sexual violence ranges from 1 percent among those in Kampot/Kep to 13 percent among those in Kandal. Women in Kandal are more likely to have experienced recent sexual violence (10 percent) than women in other provinces.

Experience of sexual violence ever and in the past 12 months is lowest among never-married women (2 percent and less than 1 percent, respectively), women with no living children (3 percent and 1 percent, respectively), and those with a secondary education or higher (5 percent and 2 percent, respectively). There is no clear relationship between sexual violence and wealth.

20.5 Perpetrators of Sexual Violence

Table 20.4 shows perpetrators of sexual violence among those who have ever experienced such violence, according to women's marital status.

Among ever-married women, current husbands/ partners are the most commonly reported perpetrators of sexual violence (61 percent), followed by former husbands/partners (23 percent).

The number of never-married women who have experienced sexual violence is too small to provide any meaningful analysis.

Table 20.3 Experience of sexual violence

Percentage of women age 15-49 who have ever experienced sexual violence and percentage who have experienced sexual violence in the 12 months preceding the survey, by background characteristics, Cambodia 2014

Background characteristic	Percentage who have experienced sexual violence: Ever[1]	Percentage who have experienced sexual violence: In the past 12 months	Number of women
**Age**			
15-19	1.0	0.2	729
20-24	6.2	2.6	687
25-29	5.7	3.2	647
30-39	6.9	4.1	1,260
40-49	9.0	4.0	985
**Religion**			
Buddhist	6.2	3.2	4,117
Moslem	1.1	0.0	96
Christian	(11.9)	(1.5)	47
Other/missing	3.4	2.4	46
**Residence**			
Urban	5.6	2.4	740
Rural	6.2	3.2	3,567
**Province**			
Banteay Meanchey	5.8	3.8	166
Kampong Cham	5.5	4.0	527
Kampong Chhnang	3.8	1.9	173
Kampong Speu	3.8	1.6	285
Kampong Thom	2.6	1.2	196
Kandal	13.2	9.8	325
Kratie	8.2	4.4	119
Phnom Penh	7.3	2.9	453
Prey Veng	1.5	1.1	304
Pursat	5.2	0.4	144
Siem Reap	9.1	5.4	280
Svay Rieng	0.4	0.4	159
Takeo	5.7	2.0	267
Otdar Meanchey	7.8	5.7	73
Battambang/Pailin	10.2	0.8	336
Kampot/Kep	1.2	0.9	200
Preah Sihanouk/ Koh Kong	5.3	2.6	105
Preah Vihear/ Stung Treng	11.9	6.0	103
Mondul Kiri/ Ratanak Kiri	1.6	1.4	92
**Marital status**			
Never married	2.0	0.4	1,062
Married or living together	6.8	3.9	2,977
Divorced/separated/ widowed	14.1	4.4	268
**Employment**			
Employed for cash	6.4	3.6	3,068
Employed not for cash	8.2	3.7	342
Not employed	4.0	1.1	897
**Number of living children**			
0	3.3	1.1	1,377
1-2	6.2	3.6	1,593
3-4	7.9	3.8	968
5+	11.2	6.1	370
**Education**			
No education	8.8	5.7	561
Primary	6.3	3.2	1,991
Secondary and higher	5.0	2.1	1,755
**Wealth quintile**			
Lowest	7.2	5.1	755
Second	6.0	3.6	823
Middle	5.6	2.2	851
Fourth	6.4	2.3	898
Highest	5.4	2.5	980
Total	6.1	3.1	4,307

Note: Figures in parentheses are based on 25-49 unweighted cases.
[1] Includes violence in the past 12 months

Table 20.4 Persons committing sexual violence

Among women age 15-49 who have experienced sexual violence, percentage who report specific persons who committed the violence, according to the respondent's current marital status, Cambodia 2014

Person	Marital status		Total
	Ever married	Never married	
Current husband/partner	60.6	na	55.7
Former husband/partner	22.9	na	21.1
Current/former boyfriend	2.4	*	5.6
Father/step father	1.0	*	1.1
Brother/step brother	0.0	*	0.0
Other relative	5.4	*	5.2
In-law	2.9	na	2.7
Own friend/acquaintance	6.6	*	7.3
Family friend	1.0	*	0.9
Police/soldier	0.2	*	0.2
Stranger	3.8	*	6.1
Other	0.0	*	0.5
Number women who have experienced sexual violence	241	21	262

Note: An asterisk indicates that a figure is based on fewer than 25 unweighted cases and has been suppressed. Women can report more than one person who committed the violence. Women can report more than one person who committed the violence.
na = Not applicable

20.6 Age at First Experience of Sexual Violence

Table 20.5 shows the percent distribution of women age 15-49 who experienced sexual violence by specific exact ages, according to current age and current marital status. Overall, 94 percent of women have not experienced sexual violence.

Less than 1 percent of women experienced sexual violence before age 15. One percent of women experienced sexual violence by age 18, and 3 percent experienced sexual violence by age 22.

In general, higher percentages of ever-married women than never-married women experienced sexual violence by the specified ages.

Table 20.5 Age at first experience of sexual violence

Percentage of women age 15-49 who experienced sexual violence by specific exact ages, according to current age and current marital status, Cambodia 2014

Background characteristic	Percentage who first experienced sexual violence by exact age:					Percentage who have not experienced sexual violence	Number of women
	10	12	15	18	22		
**Age**							
15-19	0.0	0.0	0.3	na	na	99.0	729
20-24	0.4	0.4	0.6	1.7	na	93.8	687
25-29	0.0	0.4	0.5	1.4	2.5	94.3	647
30-39	0.1	0.2	0.4	1.0	2.2	93.1	1,260
40-49	0.0	0.0	0.2	1.0	3.4	91.0	985
**Marital status**							
Never married	0.0	0.0	0.4	0.7	1.0	98.0	1,062
Ever married	0.1	0.2	0.4	1.3	3.4	92.6	3,245
Total	0.1	0.2	0.4	1.2	2.8	93.9	4,307

na = Not applicable

20.7 Experience of Different Forms of Violence

Table 20.6 presents information on the experience of various forms of violence among women age 15-49.

Twenty-two percent of women reported that they have experienced either physical or sexual violence. Sixteen percent have experienced physical violence only, 2 percent have experienced sexual violence only, and 4 percent have experienced both physical and sexual violence. The percentage of women who have experienced physical or sexual violence generally increases with age.

Table 20.6 Experience of different forms of violence

Percentage of women age 15-49 who have ever experienced different forms of violence by current age, Cambodia 2014

Age	Physical violence only	Sexual violence only	Physical and sexual violence	Physical or sexual violence	Number of women
15-19	6.4	0.7	0.4	7.5	729
15-17	7.5	0.0	0.0	7.5	430
18-19	4.9	1.6	0.9	7.4	299
20-24	12.7	2.8	3.4	18.9	687
25-29	11.8	2.4	3.3	17.6	647
30-39	22.6	2.6	4.3	29.5	1,260
40-49	20.1	1.8	7.2	29.0	985
Total	16.1	2.1	4.0	22.2	4,307

20.8 VIOLENCE DURING PREGNANCY

Respondents who had ever been pregnant were asked specifically whether they had ever experienced physical violence while pregnant and, if yes, who the perpetrators of the violence were.

Table 20.7 shows that 4 percent of women experienced physical violence during pregnancy. This percentage is higher among women age 15-19 (5 percent) and those age 40-49 (6 percent). Rural women are twice as likely as their urban counterparts to have experienced physical violence while pregnant (4 percent versus 2 percent). Women who are divorced, separated, or widowed are more likely to report experiencing violence during pregnancy (10 percent) than women who are currently married (4 percent).

Women with five or more children (10 percent) are substantially more likely to report violence during pregnancy than women with fewer or no children. The experience of violence during pregnancy declines with increasing education, from 6 percent among women with no education to 3 percent among women with a secondary education or higher. Women in the two lowest wealth quintiles are more likely than those in the three higher quintiles to have experienced violence during pregnancy (6 percent versus 2-4 percent).

Table 20.7 Experience of violence during pregnancy

Among women age 15-49 who have ever been pregnant, percentage who have ever experienced physical violence during pregnancy, by background characteristics, Cambodia 2014

Background characteristic	Percentage who experienced violence during pregnancy	Number of women who have ever been pregnant
**Age**		
15-19	5.1	77
20-24	4.1	415
25-29	2.0	548
30-39	3.2	1,138
40-49	6.1	913
**Religion**		
Buddhist	4.1	2,953
Moslem	0.0	71
Christian	(0.6)	28
Other/missing	3.8	38
**Residence**		
Urban	2.3	449
Rural	4.3	2,642
**Province**		
Banteay Meanchey	2.8	118
Kampong Cham	4.0	423
Kampong Chhnang	2.2	103
Kampong Speu	8.9	226
Kampong Thom	4.3	149
Kandal	2.0	222
Kratie	6.5	90
Phnom Penh	1.4	277
Prey Veng	2.7	236
Pursat	5.2	97
Siem Reap	5.7	188
Svay Rieng	1.3	114
Takeo	3.8	190
Otdar Meanchey	6.2	61
Battambang/Pailin	4.8	221
Kampot/Kep	3.7	149
Preah Sihanouk/ Koh Kong	3.6	73
Preah Vihear/ Stung Treng	7.4	83
Mondul Kiri/ Ratanak Kiri	2.1	72
**Marital status**		
Never married	*	4
Married or living together	3.5	2,828
Divorced/separated/ widowed	9.7	258
**Number of living children**		
0	0.0	160
1-2	3.6	1,593
3-4	3.3	968
5+	9.5	370
**Education**		
No education	5.9	478
Primary	4.3	1,673
Secondary and higher	2.6	939
**Wealth quintile**		
Lowest	6.0	602
Second	5.7	591
Middle	2.9	649
Fourth	3.9	626
Highest	1.9	623
Total	4.0	3,090

Note: Figures in parentheses are based on 25-49 unweighted cases. An asterisk indicates that a figure is based on fewer than 25 unweighted cases and has been suppressed.

20.9 MARITAL CONTROL BY SPOUSE

Attempts by husbands to closely control and monitor their wives' behavior may be an important warning sign and correlate of violence in a relationship. A series of questions were included in the 2014 CDHS to elicit the degree of marital control exercised by husbands over their wives. Controlling behaviors most often manifest themselves in terms of extreme possessiveness, jealousy, and attempts to isolate the spouse from her family and friends. To determine degree of marital control, ever-married women were asked whether their current or former husband or partner exhibited each of the following controlling behaviors: (1) he is jealous or gets angry if she talks to other men, (2) he frequently accuses her of being unfaithful, (3) he does not permit meetings with female friends, (4) he tries to limit contact with her family, (5) he insists on knowing where she is at all times, (6) he does not give her money to cover household expenses, and (7) he does not trust her with money. Because the concentration of such behaviors is more significant than the display of any single behavior, the proportion of respondents whose husbands or partners display at least three of the specified behaviors is highlighted. Table 20.8 presents the percentage of ever-married women whose husbands or partners display each of the listed behaviors, by selected background characteristics.

The main controlling behaviors women experienced from their husbands or partners were jealousy or anger if they talked to other men (23 percent) and being accused of being unfaithful (12 percent). About 1 in 20 women experienced each of the other remaining controlling behaviors (4-5 percent each).

Seven percent of ever-married women say that their husbands or partners display three or more of these controlling behaviors. Women living in Preah Sihanouk/Koh Kong (31 percent), those in urban areas (10 percent), and those who have been previously married (18 percent) are more likely than other women to report that their husbands or partners display at least three of these controlling behaviors. Women with 0-2 living children (6 percent) and those who are not employed (5 percent) are least likely to have a husband or partner who displays three or more controlling behaviors. There is no clear pattern in the percentage of women whose husbands display at least three controlling behaviors according to education, wealth, or age. Women who say they are afraid of their husbands most of the time are much more likely to say their husbands display three or more controlling behaviors (31 percent) than women who are never afraid of their husbands (6 percent).

Table 20.8 Marital control exercised by husbands

Percentage of ever-married women age 15-49 whose husbands/partners have ever demonstrated specific types of controlling behaviors, by background characteristics, Cambodia 2014

Background characteristic	Percentage of women whose husband/partner:									
	Is jealous or angry if she talks to other men	Frequently accuses her of being unfaithful	Does not permit her to meet her female friends	Tries to limit her contact with her family	Insists on knowing where she is at all times	Does not give her enough money	Does not trust her with any money	Displays 3 or more of the specific behaviors	Displays none of the specific behaviors	Number of ever-married women
**Age**										
15-19	29.0	10.8	4.1	2.6	4.6	4.6	4.6	5.8	69.2	108
20-24	29.4	11.8	9.3	3.0	4.1	5.7	5.1	7.3	64.6	461
25-29	20.1	7.9	2.7	2.1	2.1	3.6	3.9	4.3	77.8	577
30-39	23.8	13.7	4.8	4.1	4.1	4.8	4.0	7.2	71.4	1,161
40-49	19.8	13.4	6.2	5.7	7.5	4.5	5.3	8.7	74.4	938
**Religion**										
Buddhist	23.0	12.5	5.4	4.1	4.8	4.6	4.6	7.2	72.4	3,095
Moslem	19.8	2.0	3.4	0.5	0.5	5.7	3.4	3.9	72.5	75
Christian	(29.0)	(8.4)	(14.3)	(0.0)	(2.0)	(0.0)	(0.0)	(2.9)	(57.2)	32
Other/missing	21.0	12.4	5.2	3.7	6.7	4.0	6.0	8.2	78.2	43
**Residence**										
Urban	24.8	11.8	10.7	4.6	5.5	9.0	7.8	9.7	67.7	489
Rural	22.6	12.3	4.5	3.9	4.6	3.8	3.9	6.6	73.2	2,756
**Province**										
Banteay Meanchey	19.1	8.1	3.0	3.9	3.1	1.3	1.7	5.9	80.5	123
Kampong Cham	23.6	11.1	2.6	3.6	4.1	5.3	4.3	4.2	70.2	441
Kampong Chhnang	26.4	17.2	2.5	2.4	2.0	4.0	3.6	4.1	73.0	109
Kampong Speu	23.4	10.4	4.5	4.1	7.2	2.0	2.4	6.8	74.4	238
Kampong Thom	24.1	10.1	2.4	3.3	1.7	3.0	3.0	4.9	73.4	153
Kandal	25.9	8.6	4.7	3.0	4.5	4.8	3.3	4.7	69.9	237
Kratie	25.3	18.0	7.0	5.5	3.1	2.4	2.2	8.4	72.4	95
Phnom Penh	24.1	10.4	10.5	5.3	4.9	7.7	6.2	7.9	69.1	301
Prey Veng	15.0	6.2	2.4	1.3	1.4	2.1	1.9	3.0	82.6	250
Pursat	20.0	13.3	1.9	1.7	3.0	3.4	4.3	4.2	73.4	101
Siem Reap	27.7	21.8	6.0	7.8	9.0	2.8	1.9	12.2	66.1	196
Svay Rieng	21.9	15.5	3.7	1.9	7.2	3.8	2.5	7.9	75.1	118
Takeo	21.8	14.0	4.2	3.4	2.2	4.5	5.2	6.7	76.7	194
Otdar Meanchey	12.9	11.4	6.1	4.9	5.7	3.7	6.1	8.2	84.9	63
Battambang/Pailin	25.2	13.5	8.2	4.0	6.6	2.2	5.7	7.5	68.7	234
Kampot/Kep	23.3	11.0	3.8	3.7	4.0	3.2	4.7	7.2	73.6	154
Preah Sihanouk/Koh Kong	21.9	15.8	28.0	8.3	6.0	27.8	25.1	30.7	50.0	75
Preah Vihear/Stung Treng	29.2	20.9	9.2	5.0	10.4	7.4	8.7	13.7	63.6	87
Mondul Kiri/Ratanak Kiri	17.7	8.9	6.0	6.6	7.2	8.7	8.1	7.8	79.6	77
**Marital status**										
Married or living together	21.8	10.8	5.1	3.5	4.1	3.8	3.6	6.1	74.0	2,977
Divorced/separated/ widowed	35.8	28.0	9.7	9.8	11.5	13.7	14.2	17.5	54.0	268
**Number of living children**										
0	25.2	9.3	5.9	3.1	4.8	3.0	3.6	5.5	72.3	314
1-2	22.8	10.5	4.8	2.7	3.5	5.3	4.7	6.0	72.5	1,593
3-4	20.5	13.3	5.7	4.3	5.5	4.3	3.9	8.0	75.1	968
5+	27.9	19.0	7.3	9.1	8.0	3.7	6.1	10.5	64.5	370
**Employment**										
Employed for cash	22.9	12.2	5.6	3.9	5.1	5.0	4.5	7.1	72.3	2,485
Employed not for cash	28.5	19.5	7.2	7.3	5.5	3.2	4.1	10.9	67.8	238
Not employed	20.7	8.8	3.9	2.9	2.7	3.4	4.9	5.4	75.0	522
**Education**										
No education	25.4	16.8	5.6	4.6	4.2	3.7	4.6	8.1	70.1	495
Primary	22.7	12.7	5.2	4.5	5.4	5.1	4.3	7.8	72.1	1,725
Secondary and higher	22.2	9.1	5.7	2.8	3.8	4.1	4.9	5.3	73.8	1,025
**Wealth quintile**										
Lowest	27.3	19.0	5.9	4.8	5.8	3.5	4.7	9.1	68.3	620
Second	26.7	16.2	5.0	4.0	4.7	4.2	3.7	7.4	69.2	621
Middle	18.0	10.2	3.9	3.9	4.4	4.2	4.6	6.3	78.3	676
Fourth	21.4	6.6	2.8	3.4	4.0	4.2	3.7	4.7	74.1	659
Highest	22.0	9.8	9.5	3.8	4.8	6.7	5.9	8.0	71.4	670
**Woman afraid of husband/ partner**										
Afraid most of the time	49.5	39.6	14.9	17.8	20.7	19.5	23.4	31.4	40.5	106
Sometimes afraid	25.4	13.8	5.7	4.0	4.0	4.3	4.0	7.0	69.5	1,637
Never afraid	18.5	8.6	4.6	3.1	4.4	3.9	3.8	5.5	77.6	1,495
Total	23.0	12.2	5.4	4.0	4.7	4.6	4.5	7.1	72.4	3,245

Note: Husband/partner refers to the current husband/partner for currently married women and the most recent husband/partner for divorced, separated, or widowed women. Total includes 7 women with missing information about fear of husband. Figures in parentheses are based on 25-49 unweighted cases. Figures in parentheses are based on 25-49 unweighted cases.

20.10 FORMS OF SPOUSAL VIOLENCE

Different types of violence are not mutually exclusive, and women may report multiple forms of violence. Research suggests that physical violence in intimate relationships is often accompanied by psychological abuse and, in one-third to more than one-half of cases, by sexual abuse (Krug et al., 2002). Table 20.9 shows the percentage of ever-married women age 15-49 who have experienced various forms of violence by their spouse over the course of the marriage and in the 12 months preceding the survey. Note that respondents who are currently married reported on violence by their current spouse, and respondents who are widowed, divorced, or separated reported on violence by their most recent spouse. The lower panel of the table provides information on violence by any husband/partner, as opposed to only the current or most recent husband/partner.

Table 20.9 Forms of spousal violence

Percentage of ever-married women age 15-49 who have experienced various forms of violence ever or in the 12 months preceding the survey committed by their husband/partner, Cambodia 2014

		In the past 12 months		
Type of violence	Ever	Often	Sometimes	Often or sometimes
SPOUSAL VIOLENCE COMMITTED BY CURRENT OR MOST RECENT HUSBAND/PARTNER				
**Physical violence**				
Any physical violence	16.2	2.1	7.3	9.3
Pushed her, shook her, or threw something at her	10.7	1.6	4.7	6.3
Slapped her	11.9	0.9	5.6	6.5
Twisted her arm or pulled her hair	6.2	0.9	2.6	3.5
Punched her with his fist or with something that could hurt her	4.7	0.6	2.1	2.7
Kicked her, dragged her, or beat her up	5.3	0.6	2.4	3.0
Tried to choke her or burn her on purpose	0.8	0.1	0.4	0.5
Threatened her or attacked her with a knife, gun, or other weapon	3.7	0.6	1.7	2.3
**Sexual violence**				
Any sexual violence	5.5	0.7	3.3	3.9
Physically forced her to have sexual intercourse with him when she did not want to	4.6	0.6	2.7	3.3
Physically forced her to perform any other sexual acts she did not want to	2.7	0.2	2.0	2.2
Forced her with threats or in any other way to perform sexual acts she did not want to	1.7	0.2	1.1	1.3
**Emotional violence**				
Any emotional violence	24.8	3.5	13.8	17.3
Said or did something to humiliate her in front of others	13.1	1.5	7.9	9.3
Threatened to hurt or harm her or someone she cared about	9.5	1.5	4.7	6.2
Insulted her or made her feel bad about herself	20.4	3.0	10.8	13.8
**Economic violence**				
Any economic violence	5.8	na	na	na
Does/did not give her money to cover household expenses	4.6	na	na	na
Does/did not trust her with money	4.5	na	na	na
Any form of physical and/or sexual violence	18.2	2.3	8.7	10.9
Any form of emotional and/or physical and/or sexual violence	28.7	4.5	15.1	19.6
Any form of emotional and/or physical and/or sexual violence and/or economic violence	30.8	na	na	na
SPOUSAL VIOLENCE COMMITTED BY ANY HUSBAND/PARTNER				
Physical violence	17.6	0.0	9.3	9.3
Sexual violence	5.8	na	na	3.9
Economical violence	5.8	na	na	0.0
Physical and/or sexual violence	19.6	na	na	10.9
Number of ever-married women	3,245	3,245	3,245	3,245

na = Not applicable

Sixteen percent of ever-married women reported ever experiencing physical violence committed by their current or most recent husband or partner, 6 percent reported sexual violence, and 25 percent reported emotional violence. Eighteen percent of women have experienced physical and/or sexual violence, and 29 percent have experienced at least one of the three forms of spousal violence. Thirty-one percent of women have experienced at least one of the four forms of spousal violence (physical, sexual, emotional and economic).

The most common form of spousal violence reported by ever-married women is being insulted or made to feel bad about themselves (20 percent) and being humiliated in front of others (13 percent). Twelve percent of women report having been slapped, and 11 percent report having been pushed, been shaken, or had something thrown at them.

Nine percent of ever-married women reported experiencing spousal physical violence in the past 12 months, with 7 percent having experienced violence sometimes and 2 percent having experienced it often. Four percent reported having experienced spousal sexual violence in the past 12 months (3 percent sometimes and 1 percent often), and 17 percent reported having experienced spousal emotional violence during that period (14 percent sometimes and 4 percent often). Overall, 20 percent of ever-married women experienced at least one of the three forms (physical, sexual and emotional) of violence by their current or most recent husband or partner in the past year.

Some women have been married or partnered more than once. When previous husbands or partners are included as perpetrators, the percentage of ever-married women who report having ever experienced physical and/or sexual violence by any husband or partner increases to 20 percent.

20.11 Spousal Violence by Background Characteristics

Table 20.10 shows the percentages of ever-married women age 15-49 who have experienced spousal, emotional, economic, physical, or sexual violence from their current or most recent husband by selected background characteristics. Overall, 31 percent of ever-married women have experienced at least one forms of violence.

The percentage of women who have experienced at least one form of spousal violence tends to increase with age and number of living children. It is higher among women from Siem Reap (59 percent) and Preah Vihear/Stung Treng (54 percent); among rural women (31 percent); among women who are divorced, separated, or widowed (47 percent); and among women who are employed but not for cash (45 percent). Women with a secondary education or higher and those in the wealthiest quintile are least likely to have experienced at least one form of spousal violence.

Table 20.10 Spousal violence by background characteristics

Percentage of ever-married women age 15-49 who have ever experienced emotional, physical, sexual, or economic violence committed by their husband/partner, by background characteristics, Cambodia 2014

Background characteristic	Emotional violence	Physical violence	Sexual violence	Economic violence	Physical and sexual	Physical and sexual and emotional	Physical or sexual	Physical or sexual or emotional	Physical or sexual or emotional or economic	Number of ever-married women
**Age**										
15-19	13.5	7.1	1.5	4.9	1.2	1.2	7.5	14.9	15.2	108
20-24	15.2	11.7	4.7	6.3	2.0	1.9	14.4	19.2	22.3	461
25-29	18.7	11.1	4.2	4.4	2.2	2.1	13.1	22.4	24.3	577
30-39	27.7	18.0	5.7	6.2	3.2	3.1	20.5	32.2	33.9	1,161
40-49	30.9	20.3	6.9	6.2	5.4	4.9	21.7	34.4	36.7	938
**Religion**										
Buddhist	24.8	16.2	5.5	5.9	3.5	3.3	18.2	28.7	30.8	3,095
Moslem	27.1	17.9	1.4	5.7	0.0	0.0	19.3	28.9	32.4	75
Christian	(15.2)	(1.6)	(13.6)	(0.0)	(0.0)	(0.0)	(15.1)	(17.7)	(17.7)	32
Other/missing	23.2	22.2	3.7	6.0	3.7	3.7	22.2	32.8	35.7	43
**Residence**										
Urban	18.2	12.2	3.9	9.7	2.7	2.6	13.4	23.2	28.4	489
Rural	25.9	16.9	5.8	5.1	3.6	3.3	19.1	29.7	31.2	2,756
**Province**										
Banteay Meanchey	19.2	14.2	5.1	1.7	4.6	4.6	14.6	23.8	24.3	123
Kampong Cham	34.7	20.5	4.9	8.2	3.2	3.2	22.2	35.3	36.7	441
Kampong Chhnang	16.6	9.3	5.6	4.0	2.9	1.2	12.0	21.7	25.4	109
Kampong Speu	19.3	15.3	3.5	2.4	3.1	3.1	15.7	23.8	23.8	238
Kampong Thom	17.9	13.2	2.3	3.4	2.3	1.8	13.2	18.9	19.6	153
Kandal	22.7	13.9	16.8	4.8	7.2	6.7	23.5	28.3	29.2	237
Kratie	25.5	20.4	7.8	2.6	7.1	5.8	21.1	31.7	32.2	95
Phnom Penh	15.5	11.4	3.8	8.1	2.5	2.5	12.7	22.1	26.4	301
Prey Veng	7.8	10.4	1.9	2.5	1.0	1.0	11.3	13.7	14.3	250
Pursat	24.3	11.2	1.8	7.7	1.8	1.8	11.2	26.2	31.5	101
Siem Reap	55.4	28.7	10.8	2.8	7.7	7.7	31.8	58.0	58.8	196
Svay Rieng	13.9	8.4	0.6	4.3	0.6	0.6	8.4	17.1	19.5	118
Takeo	32.3	20.1	5.6	5.2	5.0	4.3	20.7	35.7	37.3	194
Otdar Meanchey	17.1	15.5	6.6	7.2	4.8	3.6	17.3	18.8	19.9	63
Battambang/Pailin	22.3	12.8	5.4	6.1	0.9	0.9	17.3	26.3	27.3	234
Kampot/Kep	28.2	21.9	1.1	4.7	0.1	0.1	22.9	32.1	32.1	154
Preah Sihanouk/ Koh Kong	26.5	20.3	4.9	28.7	4.1	4.1	21.0	31.1	52.1	75
Preah Vihear/ Stung Treng	45.2	25.7	12.1	9.4	7.6	6.8	30.2	51.2	53.5	87
Mondul Kiri/ Ratanak Kiri	17.5	13.7	1.7	8.7	1.5	1.5	14.0	23.3	29.9	77
**Marital status**										
Married or living together	23.9	14.9	4.8	4.8	2.8	2.6	16.9	27.6	29.3	2,977
Divorced/separated/ widowed	34.2	30.0	12.6	16.9	10.1	9.8	32.5	40.1	46.7	268
**Number of living children**										
0	9.8	7.7	3.9	3.6	2.3	2.3	9.3	12.7	14.8	314
1-2	21.0	13.9	4.6	6.3	2.5	2.4	16.0	24.9	27.5	1,593
3-4	30.3	18.7	6.8	5.4	4.4	4.0	21.1	34.4	36.0	968
5+	39.0	26.7	7.3	6.7	5.9	5.1	28.1	43.5	44.8	370
**Employment**										
Employed for cash	22.8	15.1	5.4	6.1	3.2	3.1	17.3	26.7	29.0	2,485
Employed not for cash	41.8	26.8	8.8	4.1	7.8	6.9	27.7	44.9	45.0	238
Not employed	26.1	16.6	4.2	5.3	2.3	1.9	18.5	30.5	32.7	522
**Education**										
No education	31.2	24.8	7.8	5.3	5.8	5.2	26.9	37.9	39.9	495
Primary	29.2	17.8	5.4	6.2	3.3	3.2	19.9	32.6	34.4	1,725
Secondary and higher	14.2	9.3	4.5	5.4	2.4	2.3	11.3	17.7	20.2	1,025
**Wealth quintile**										
Lowest	31.8	23.7	7.3	5.9	5.2	4.5	25.9	37.1	38.5	620
Second	30.3	18.7	6.6	5.4	4.1	4.0	21.1	33.1	33.8	621
Middle	23.8	16.4	3.9	5.1	2.5	2.4	17.8	27.3	29.1	676
Fourth	23.2	13.5	5.3	5.4	3.6	3.2	15.2	26.2	28.1	659
Highest	15.6	9.4	4.5	7.3	1.9	1.9	12.0	20.6	25.0	670
Total	24.8	16.2	5.5	5.8	3.4	3.2	18.2	28.7	30.8	3,245

Note: Husband/partner refers to the current husband/partner for currently married women and the most recent husband/partner for divorced, separated, or widowed women. Figures in parentheses are based on 25-49 unweighted cases.

20.12 VIOLENCE BY SPOUSAL CHARACTERISTICS AND WOMEN'S EMPOWERMENT INDICATORS

Table 20.11 presents information on ever-married women age 15-49 who have experienced emotional, economic, physical, or sexual violence committed by their current or most recent husband according to the husband's characteristics and empowerment indicators. It is worth noting that about 8 percent of women have been married more than once.

Spousal violence is highest among women whose husbands have no education or only a primary education (42 percent and 36 percent, respectively), women whose husbands get drunk very often (61 percent), and women who are not educated and have husbands who are not educated (39 percent).

Spousal violence increases linearly with the number of controlling behaviors displayed by the husband. Among women whose husbands exhibit three or more types of controlling behaviors, at least three in four (89-91 percent) have experienced one or more forms of violence. In contrast, among women whose husbands display none of the seven controlling behaviors, about one-fifth (19 percent) have experienced any form of spousal violence. There is no clear association between women's experience of violence and the number of decisions in which they participate or the number of reasons they give for which wife beating is justified. Women whose father did not beat their mother are much less likely to experience any type of violence by their husband than women whose father beat their mother (26 percent versus 44 percent). Finally, women who are afraid of their husband/partner most of the time are much more likely to experience any type of violence than women who are never afraid (86 percent versus 24 percent).

Table 20.11 Spousal violence by husband's characteristics and empowerment indicators

Percentage of ever-married women age 15-49 who have ever experienced emotional, physical, or sexual violence committed by their husband/partner, by husband's characteristics and empowerment indicators, Cambodia 2014

Background characteristic	Emotional violence	Physical violence	Sexual violence	Economic violence	Physical and sexual	Physical and sexual and emotional	Physical or sexual	Physical or sexual or emotional	Physical or sexual or emotional or economic	Number of ever-married women
**Husband's/partner's education**										
No education	33.4	23.1	11.8	5.3	8.1	7.8	26.7	40.1	42.0	327
Primary	29.5	20.1	5.8	6.0	3.7	3.4	22.2	34.3	36.0	1,464
Secondary and higher	17.7	10.6	3.7	5.8	2.0	1.9	12.2	20.2	22.7	1,435
**Husband's/partner's alcohol consumption**										
Does not drink	13.3	8.7	1.8	4.0	1.3	1.0	9.1	15.5	17.7	490
Drinks/never gets drunk	11.1	8.0	2.1	2.6	0.0	0.0	10.1	14.4	15.6	108
Gets drunk sometimes	20.6	12.3	3.7	4.6	2.1	2.0	13.9	24.4	26.7	2,103
Gets drunk very often	53.9	39.6	16.2	12.9	11.1	10.4	44.7	60.0	61.3	543
**Spousal education difference**										
Husband better educated	24.4	16.3	4.8	6.2	2.7	2.6	18.3	28.5	30.7	1,699
Wife better educated	22.5	14.7	4.8	4.4	3.4	3.2	16.1	26.5	27.8	876
Both equally educated	27.1	17.0	7.7	6.7	4.9	4.4	19.9	30.3	32.7	474
Neither educated	30.2	19.4	9.9	5.9	6.4	5.9	22.8	35.3	38.5	170
Don't know/missing	(41.7)	(22.0)	(5.8)	(16.7)	(3.2)	(3.2)	(24.6)	(41.7)	(47.6)	26
**Spousal age difference**[1]										
Wife older	24.3	17.1	6.2	3.0	4.0	3.6	19.3	28.7	29.4	540
Wife is same age	22.8	11.0	5.8	6.3	1.2	1.2	15.7	27.8	28.4	253
Wife is 1-4 years younger	25.2	15.6	4.8	4.6	3.5	3.2	17.0	28.9	31.1	1,219
Wife is 5-9 years younger	22.9	15.0	3.5	5.9	1.9	1.8	16.6	26.5	28.3	699
Wife is 10+ years younger	20.5	10.4	4.7	5.9	1.3	1.2	13.8	22.1	24.3	263
**Number of marital control behaviors displayed by husband/partner**[2]										
0	15.5	8.6	2.1	0.9	1.3	1.1	9.4	18.6	19.1	2,371
1-2	42.6	30.3	10.3	11.3	5.0	4.7	35.6	49.3	54.2	675
3-4	70.5	57.1	23.1	37.2	20.7	20.3	59.4	74.9	89.4	147
5-6	85.1	63.5	45.2	68.4	31.5	31.5	77.2	87.6	91.0	53
**Number of decisions in which women participate**[3]										
0	(30.1)	(12.9)	(0.0)	(0.9)	(0.0)	(0.0)	(12.9)	(30.1)	(30.1)	28
1-2	25.4	15.8	6.2	8.4	3.3	2.4	18.7	32.0	34.5	362
3	23.6	14.8	4.7	4.4	2.8	2.6	16.7	27.0	28.6	2,586
**Number of reasons for which wife beating is justified**[4]										
0	20.5	14.1	4.3	6.6	3.1	2.9	15.3	23.4	26.2	1,482
1-2	29.6	18.5	7.7	4.8	3.9	3.6	22.4	34.9	36.3	934
3-4	26.1	18.4	5.7	5.9	3.8	3.3	20.3	31.3	32.9	662
5-6	30.0	12.6	2.9	3.8	2.5	2.3	13.1	30.8	31.2	166
**Woman's father beat her mother**										
Yes	35.6	25.2	7.3	6.9	4.2	4.1	28.3	41.6	43.8	595
No	20.5	12.6	4.9	5.0	3.1	2.8	14.5	23.6	25.8	2,426
Don't know/missing	42.2	30.5	6.8	11.9	5.2	5.0	32.1	49.1	50.0	224
**Woman afraid of husband/ partner**										
Afraid most of the time	74.9	77.2	30.4	23.8	29.9	28.3	77.7	83.9	86.1	106
Sometimes afraid	27.8	17.4	5.9	5.5	3.7	3.3	19.6	32.0	33.8	1,637
Never afraid	18.0	10.6	3.3	4.9	1.3	1.3	12.6	21.3	23.7	1,495
Total	24.8	16.2	5.5	5.8	3.4	3.2	18.2	28.7	30.8	3,245

Note: Husband/partner refers to the current husband/partner for currently married women and the most recent husband/partner for divorced, separated, or widowed women. Total includes women with missing information on husband's/partner's education, husband's/partner's alcohol consumption, spousal age difference, and fear of husband who are not shown separately. Figures in parentheses are based on 25-49 unweighted cases.

[1] Includes only women who have been married only once

[2] According to the wife's report. See Table 20.8 for list of behaviors.

[3] According to the wife's report. Includes only currently married women. See Table 19.5.1 for list of decisions.

[4] According to the wife's report. See Table 19.7.1 for list of reasons.

20.13 RECENT SPOUSAL VIOLENCE BY ANY HUSBAND OR PARTNER

Table 20.12 shows the percentage of ever-married women who have experienced physical or sexual violence by any husband or partner (current, most recent, or previous) in the past 12 months, by background characteristics.

Overall, 11 percent of women experienced physical or sexual violence by any husband or partner in the past 12 months. The percentage of women who have experienced recent physical or sexual violence by any spouse or partner is higher among those age 30-39 (13 percent), those from Kampong Cham (20 percent) and Siem Reap (19 percent), and those from rural areas (12 percent). The percentage is lowest among women who have no living children (6 percent), those who are not employed (9 percent), those with a secondary education or higher (6 percent), and those in the highest wealth quintile (5 percent). The experience of physical or sexual violence by any husband or partner in the past 12 months varies only minimally by marital status.

20.14 ONSET OF SPOUSAL VIOLENCE

To obtain information on the onset of marital violence, the 2014 CDHS asked ever-married women how long after marriage the onset of spousal violence occurred, if ever.

Table 20.13 shows that the majority of women (84 percent) have never experienced spousal physical or sexual violence. Four percent experienced violence in the first two years of marriage, 9 percent experienced it in the first five years, and 12 percent experienced it within the first 10 years of marriage. These data clearly suggest that, for a considerable percentage of women who have experienced spousal physical or sexual violence, the violence began relatively early in their marriage.

Table 20.12 Physical or sexual violence in the past 12 months by any husband/partner

Percentage of ever-married women who have experienced physical or sexual violence by any husband/partner in the past 12 months, by background characteristics, Cambodia 2014

Background characteristic	Physical or sexual violence in the past 12 months from any husband/partner	Number of ever-married women
**Age**		
15-19	7.0	108
20-24	9.8	461
25-29	7.8	577
30-39	13.3	1,161
40-49	11.0	938
**Religion**		
Buddhist	10.8	3,095
Moslem	15.3	75
Christian	(2.3)	32
Other/missing	17.1	43
**Residence**		
Urban	5.5	489
Rural	11.9	2,756
**Province**		
Banteay Meanchey	14.6	123
Kampong Cham	20.0	441
Kampong Chhnang	5.5	109
Kampong Speu	7.9	238
Kampong Thom	8.5	153
Kandal	16.0	237
Kratie	13.7	95
Phnom Penh	4.2	301
Prey Veng	4.7	250
Pursat	7.4	101
Siem Reap	19.1	196
Svay Rieng	4.9	118
Takeo	9.3	194
Otdar Meanchey	15.4	63
Battambang/Pailin	7.0	234
Kampot/Kep	9.4	154
Preah Sihanouk/ Koh Kong	8.7	75
Preah Vihear/ Stung Treng	15.7	87
Mondul Kiri/ Ratanak Kiri	7.9	77
**Marital status**		
Married or living together	10.8	2,977
Divorced/separated/ widowed	12.0	268
**Number of living children**		
0	6.3	314
1-2	10.3	1,593
3-4	11.4	968
5+	16.6	370
**Employment**		
Employed for cash	11.2	2,485
Employed not for cash	12.3	238
Not employed	9.3	522
**Education**		
No education	17.5	495
Primary	12.1	1,725
Secondary and higher	5.8	1,025
**Wealth quintile**		
Lowest	18.2	620
Second	13.0	621
Middle	11.1	676
Fourth	7.7	659
Highest	5.4	670
Total	10.9	3,245

Note: Any husband/partner includes all current, most recent, and former husbands/partners. Total includes 7 women with missing information about fear of husband/partner. Figures in parentheses are based on 25-49 unweighted cases.

Table 20.13 Experience of spousal violence by duration of marriage						

Among currently married women age 15-49 who have been married only once, the percentage who first experienced physical or sexual violence committed by their current husband/partner by specific exact years since marriage, according to marital duration, Cambodia 2014

Duration of marriage	Percentage who first experienced spousal physical or sexual violence by exact marital duration: Before marriage	2 years	5 years	10 years	Percentage who have not experienced spousal sexual or physical violence	Number of currently married women who have been married only once
**Years since marriage**						
<2	0.2	na	na	na	95.5	297
2-4	0.0	9.8	na	na	86.9	385
5-9	0.7	3.4	8.7	na	86.7	550
10+	0.0	3.1	8.7	12.7	80.6	1,520
Total	0.2	4.2	8.8	11.9	84.3	2,752

na = Not applicable

20.15 Physical Consequences of Spousal Violence

In the 2014 CDHS, ever-married women were asked whether they had sustained some form of injury as a result of physical or sexual violence inflicted by their spouse. About half of women (44 percent) who reported ever having experienced spousal physical or sexual violence suffered cuts, bruises, or aches; 22 percent had eye injuries, sprains, dislocations, or burns; and 5 percent had deep wounds, broken bones, broken teeth, or other serious injuries (Table 20.14). Overall, 48 percent of women who had ever experienced spousal physical or sexual violence suffered one or more of these injuries. The prevalence of all forms of injury is similar among women who had experienced violence in the past 12 months.

Table 20.14 Injuries to women due to spousal violence

Percentage of ever-married women age 15-49 who have experienced specific types of spousal violence by types of injuries resulting from the violence, according to the type of violence and whether they experienced the violence ever and in the 12 months preceding the survey, Cambodia 2014

Type of violence	Cuts, bruises, or aches	Eye injuries, sprains, dislocations, or burns	Deep wounds, broken bones, broken teeth, or any other serious injury	Any of these injuries	Number of ever-married women who have ever experienced any physical or sexual violence
**Experienced physical violence[1]**					
Ever[2]	48.0	23.4	5.1	51.5	525
In the past 12 months	51.5	27.6	4.7	56.2	303
**Experienced sexual violence**					
Ever[2]	47.9	25.5	2.7	50.8	178
In the past 12 months	47.0	29.2	2.4	50.6	128
**Experienced physical or sexual violence[1]**					
Ever[2]	44.4	21.8	4.5	48.1	592
In the past 12 months	46.7	25.3	4.0	51.6	355

Note: Husband/partner refers to the current husband/partner for currently married women and the most recent husband/partner for divorced, separated, or widowed women.
[1] Excludes women who reported violence only in response to a direct question on violence during pregnancy
[2] Includes in the past 12 months

20.16 Violence by Women against Their Husband

In cases of domestic violence, either person, the husband or the wife, can be the perpetrator of violence. In the 2014 CDHS, ever-married women were asked about instances when they were the instigator of spousal violence. Specifically, they were asked whether they had ever tried to initiate physical violence against their current or most recent husband or partner when he was not already hitting or beating them.

Table 20.15 shows the percentage of ever-married women age 15-49 who reported initiating physical violence against their husbands ever and in the 12 months prior to the survey, by background characteristics.

Overall, 9 percent of women reported that they had initiated physical violence against their husbands or partners, and 5 percent had done so in the past 12 months. Women who have been physically abused by their current or most recent husband or partner ever or in the past 12 months (31 percent each) are more likely to have initiated spousal physical abuse than women who have never been abused (5 percent). Women's use of violence against their husbands increases somewhat with age. It is higher among women in Preah Vihear/Stung Treng (19 percent) and Preah Sihanouk/Koh Kong (18 percent), formerly married women (11 percent), and women with three or more living children (10 percent). On the other hand, the proportion of women who have initiated physical violence against their husbands decreases with increasing education, from 11 percent among those with no education to 7 percent among those with a secondary education or higher. The percentage who report that they have initiated physical violence does not vary notably by residence or employment and does not have a linear relationship with wealth quintile. Patterns among women who reported initiating physical violence against their husbands in the past 12 months are similar in the case of some but not all background characteristics.

Table 20.15 Women's violence against their spouse

Percentage of ever-married women age 15-49 who have committed physical violence against their current or most recent husband/partner when he was not already beating or physically hurting them, ever and in the past 12 months, according to women's own experience of spousal violence and background characteristics, Cambodia 2014

Background characteristic	Percentage who have committed physical violence against their husband/partner: Ever[1]	In the past 12 months	Number of ever-married women
**Woman's experience of spousal physical violence**			
Ever[1]	30.9	15.8	525
In the past 12 months	31.2	23.2	303
Never	4.7	2.9	2,720
**Age**			
15-19	5.2	4.8	108
20-24	5.9	4.7	461
25-29	9.0	6.3	577
30-39	9.9	5.5	1,161
40-49	9.6	3.9	938
**Religion**			
Buddhist	8.6	4.6	3,095
Moslem	14.1	12.7	75
Christian	(32.5)	(31.8)	32
Other/missing	2.3	0.7	43
**Residence**			
Urban	10.2	5.5	489
Rural	8.7	4.9	2,756
**Province**			
Banteay Meanchey	6.1	3.4	123
Kampong Cham	13.1	11.1	441
Kampong Chhnang	6.4	3.8	109
Kampong Speu	5.9	3.6	238
Kampong Thom	7.0	3.1	153
Kandal	5.5	5.1	237
Kratie	14.3	5.9	95
Phnom Penh	12.1	4.5	301
Prey Veng	6.7	3.7	250
Pursat	9.5	3.9	101
Siem Reap	7.4	2.2	196
Svay Rieng	7.4	2.5	118
Takeo	3.4	1.1	194
Otdar Meanchey	11.2	9.4	63
Battambang/Pailin	11.6	6.4	234
Kampot/Kep	4.9	1.1	154
Preah Sihanouk/Koh Kong	18.0	10.6	75
Preah Vihear/Stung Treng	19.2	8.2	87
Mondul Kiri/Ratanak Kiri	1.9	1.3	77
**Marital status**			
Married or living together	8.8	5.2	2,977
Divorced/separated/widowed	10.6	2.8	268
**Employment**			
Employed for cash	8.7	5.1	2,485
Employed not for cash	10.4	3.7	238
Not employed	9.1	5.1	522
**Number of living children**			
0	8.4	6.6	314
1-2	7.9	5.0	1,593
3-4	10.4	4.6	968
5+	9.9	4.9	370
**Education**			
No education	11.2	6.6	495
Primary	9.6	5.3	1,725
Secondary and higher	6.7	3.7	1,025
**Wealth quintile**			
Lowest	13.6	8.0	620
Second	7.3	3.0	621
Middle	7.8	5.0	676
Fourth	7.3	4.9	659
Highest	8.8	4.2	670
Total	8.9	5.0	3,245

Note: Husband/partner refers to the current husband/partner for currently married women and the most recent husband/partner for divorced, separated, or widowed women. Figures in parentheses are based on 25-49 unweighted cases.
[1] Includes in the past 12 months

20.17 Help-seeking Behavior by Women Who Experience Violence

Table 20.16 shows the percent distribution of women age 15-49 who have ever experienced physical or sexual violence committed by anyone, according to whether they ever sought help to stop the violence and, if not, whether or not they told anyone about the violence.

Table 20.16 Help seeking to stop violence

Percent distribution of women age 15-49 who have ever experienced physical or sexual violence by their help-seeking behavior, according to type of violence and background characteristics, Cambodia 2014

Background characteristic	Sought help to stop violence	Never sought help but told someone	Never sought help, never told anyone	Missing/don't know	Total	Number of women who have ever experienced any physical or sexual violence
**Type of violence experienced**						
Physical only	40.6	19.8	39.0	0.6	100.0	693
Sexual only	33.5	4.7	61.9	0.0	100.0	90
Physical and sexual	55.1	22.0	22.9	0.0	100.0	172
**Age**						
15-19	(40.3)	(22.6)	(37.1)	(0.0)	100.0	54
20-24	29.0	24.6	45.9	0.5	100.0	130
25-29	34.2	12.5	53.3	0.0	100.0	114
30-39	42.5	22.2	34.8	0.5	100.0	372
40-49	52.5	13.6	33.4	0.5	100.0	286
**Residence**						
Urban	32.3	24.4	43.0	0.4	100.0	152
Rural	44.5	17.7	37.3	0.4	100.0	803
**Marital status**						
Never married	40.3	21.3	36.4	2.0	100.0	89
Married or living together	42.8	18.5	38.5	0.3	100.0	756
Divorced/separated/ widowed	42.9	18.9	38.1	0.1	100.0	110
**Number of living children**						
0	37.0	22.4	38.5	2.1	100.0	152
1-2	38.9	18.8	42.2	0.2	100.0	375
3-4	43.1	22.0	34.8	0.1	100.0	282
5+	56.4	9.0	34.5	0.0	100.0	146
**Employment**						
Employed for cash	43.8	17.7	37.9	0.6	100.0	678
Employed not for cash	42.9	14.0	43.1	0.0	100.0	105
Not employed	37.5	26.0	36.5	0.0	100.0	172
**Education**						
No education	44.2	17.8	37.9	0.1	100.0	177
Primary	46.2	19.6	33.6	0.6	100.0	535
Secondary and higher	33.4	17.7	48.6	0.3	100.0	244
**Wealth quintile**						
Lowest	44.4	18.3	37.2	0.1	100.0	225
Second	52.1	9.7	37.5	0.7	100.0	201
Middle	44.9	21.1	34.0	0.0	100.0	176
Fourth	35.8	20.8	42.3	1.0	100.0	179
Highest	33.6	25.5	40.5	0.3	100.0	174
Total	42.6	18.8	38.2	0.4	100.0	955

Note: Women can report more than one source from which they sought help. The distribution by provinces is not shown because there were less than 50 unweighted cases in most provinces. Figures in parentheses are based on 25-49 unweighted cases.

Overall, more than 4 in 10 women (43 percent) who have experienced any type of physical or sexual violence from anyone sought help to stop the violence. Thirty-eight percent of women never sought help and never told anyone, and 19 percent never sought help but told someone. Women who have experienced both physical and sexual violence (55 percent), women age 40-49 (53 percent), rural women (45 percent), and women who have five or more children (56 percent) are more likely than other women to seek help to stop violence. Women who are employed (for cash or not for cash) are more likely than those who are not employed to have ever sought help. Help seeking varies insignificantly by marital status and wealth quintile. It is interesting to note that highly educated women are less likely than women with a primary education or less to seek help from any source to stop the violence.

Table 20.17 shows the percentage of abused women who reported seeking help by the sources from which help was sought. The most common sources of help are the woman's own family (59 percent), neighbors (29 percent), and the police (12 percent). A relatively high percentage of women (11 percent) seek help from their husband's or partner's family.

Table 20.17 Sources for help to stop the violence

Percentage of women age 15-49 who have experienced physical or sexual violence and sought help by sources from which they sought help, according to the type of violence that women reported, Cambodia 2014

	Type of violence experienced			
Person	Physical only	Sexual only	Physical and sexual	Total
Own family	57.2	*	60.1	58.5
Husband/partner's family	11.5	*	13.1	11.4
Husband/partner	0.6	*	0.2	0.5
Boyfriend	0.0	*	0.0	0.6
Friend	7.1	*	6.1	7.8
Neighbor	30.4	*	32.2	29.2
Religious leader	0.2	*	0.0	0.8
Doctor/medical personnel	0.9	*	0.0	0.6
Police	10.7	*	17.8	12.3
Lawyer	0.6	*	1.3	0.7
Social work organization	0.0	*	1.6	0.5
Other	14.7	*	22.2	16.4
Number of women who have experienced violence and sought help	282	22	103	406

Note: An asterisk indicates that a figure is based on fewer than 25 unweighted cases and has been suppressed.

REFERENCES

Arimond, M., and M.T. Ruel. 2003. *Generating indicators of appropriate feeding of children 6 through 23 months from the KPC 2000+*. Washington, DC: Academy for Educational Development, Food and Nutrition Technical Assistance Project (FANTA). http://pdf.usaid.gov/pdf_docs/PNACW465.pdf.

Bradley, E.K, T.N. Croft, J.D. Fishel, and C.F. Westoff. 2012. *Revising unmet need for family planning.* DHS Analytical Studies No. 25. Calverton, Maryland, USA: ICF International; 2012.

Centers for Disease Control and Prevention (CDC). 1998. Recommendations to prevent and control iron deficiency in the United States. *Morbidity and Mortality Weekly Report* 47(RR-3): 1-29.

Filmer, D., and L. Pritchett. 1998. *Estimating wealth effects without expenditure data – or tears: An application to educational enrollments in states of India*. World Bank Policy Research Working Paper No. 1994. Washington, DC: World Bank, Development Economics Research Group.

George, J., M. Yiannakis, B. Main, R. Devenish, C. Anderson C, U.S. An, et al. 2012. Genetic hemoglobin disorders, infection, and deficiencies of iron and vitamin A determine anemia in young Cambodian children. *Journal of Nutrition*. 142: 781-787.

Heise, L., M. Ellsberg, and M. Gottemoeller. 1999. *Ending violence against women*. Population Reports, Series L, No. 11. Baltimore, Maryland: Johns Hopkins University School of Public Health, Population Information Program.

International Monetary Fund. 2011. Cambodia GDP per capita at current prices in U.S. dollars. http://www.tradingeconomics.com/cambodia/gdp-per-capita-at-current-prices-in-us-dollars-imf-data.html.

Karakochuk, C.D., K.C. Whitfield, S.I. Barr, Y. Lamers, A.M. Devlin, S.M. Vercauteren, et al. 2015. Genetic hemoglobin disorders rather than iron deficiency are a major predictor of hemoglobin concentration in women of reproductive age in rural Prey Veng, Cambodia. *Journal of Nutrition*. 145: 134-142.

Kish, L. 1965. *Survey sampling.* New York: John Wiley and Sons Inc.

Krug, E.G., L. Dahlberg, J. Mercy, A. Zwi, and R. Lozano, eds. 2002. *World report on violence and health.* Geneva, Switzerland: World Health Organization.

Ministry of Health (MOH) [Cambodia]. 2008. Health Sector Strategic Plan 2008-2015. Phnom Penh, Cambodia: MOH.

Ministry of Health (MOH) [Cambodia]. 2015. Annual Health Financing Report 2015. Phnom Penh, Cambodia: MOH.

Ministry of Planning (MOP) [Cambodia]. 2012. Poverty estimates in Cambodia: 2012. http://www.mop.gov.kh/Projects/ tabid/148/Default.aspx.

National Institute of Statistics [Cambodia]. 1999. General Population Census 1998. Phnom Penh, Cambodia: National Institute of Statistics.

National Institute of Statistics [Cambodia], Directorate General for Health [Cambodia], and ORC Macro. 2001. *Cambodia Demographic and Health Survey 2000*. Phnom Penh, Cambodia, and Calverton, MD, USA: National Institute of Statistics, Directorate General for Health, and ORC Macro.

National Institute of Statistics [Cambodia]. 2004. Cambodia Inter-Censal Population Survey 2004. General Report. Phnom Penh, Cambodia: National Institute of Statistics.

National Institute of Statistics [Cambodia], Directorate General for Health [Cambodia], and ORC Macro. 2006. *Cambodia Demographic and Health Survey 2005*. Phnom Penh, Cambodia, and Calverton, MD, USA: National Institute of Statistics, Directorate General for Health, and ORC Macro.

National Institute of Statistics [Cambodia]. 2009. General Population Census 2008. Phnom Penh, Cambodia: National Institute of Statistics.

National Institute of Statistics [Cambodia], Directorate General for Health [Cambodia], and ICF Macro. 2011. *Cambodia Demographic and Health Survey 2010.* Phnom Penh, Cambodia, and Calverton, Maryland, USA: National Institute of Statistics, Directorate General for Health, and ICF Macro.

National Institute of Statistics [Cambodia]. 2013. Cambodia Inter-censal Population Survey Results. Report 13: Population Aging in Cambodia 2014. Phnom Penh, Cambodia: National Institute of Statistics.

Rutstein, S. 1999. *Wealth versus expenditure: Comparison between the DHS wealth index and household expenditures in four departments of Guatemala*. Calverton, Maryland, USA: ORC Macro.

Rutstein, S.O. 2005. Effects of preceding birth intervals on neonatal, infant and under-five years mortality and nutritional status in developing countries: Evidence from the Demographic and Health Surveys. *International Journal of Gynecology and Obstetrics* 89(Supplement 1): S7-S24.

Strauss, M.A. 1990. Measuring intra-family conflict and violence: The Conflict Tactics Scale. In *Physical violence in American families: Risk factors and adaptation to violence in 8,145 families*. New Brunswick, New Jersey, USA: Transaction Publications.

World Bank. 2014. Data for Cambodia. http://data.worldbank.org/country/cambodia.

World Health Organization (WHO). 2001. *Putting women first: Ethical and safety recommendations for research on domestic violence against women.* Geneva, Switzerland: Department of Gender and Women's Health, World Health Organization.

World Health Organization (WHO) Multicentre Growth Reference Study Group (MGRS). 2006. *WHO child growth standards: Length/height-for-age, weight-for-age, weight-for-length, weight-for-height and body mass index-for-age: Methods and development.* Geneva, Switzerland: World Health Organization.

World Health Organization (WHO). 2008. *Indicators for assessing infant and young child feeding practices. Part 1: Definitions.* Geneva, Switzerland: WHO. http://whqlibdoc.who.int/publications/2008/9789241596664_eng.pdf.

World Health Organization (WHO). 2010. *Indicators for assessing infant and young child feeding practices. Part 2: Measurement.* Geneva, Switzerland: WHO. http://whqlibdoc.who.int/publications/2010/9789241599290_eng.pdf.

World Health Organization (WHO). 2011. Cambodia National Health Account. http://www.who.int/nha/country/khm/en/.

World Health Organization (WHO) Vitamin and Mineral Nutrition Information System (VMNIS). 2013. WHO Urinary iodine concentrations for determining iodine status in populations. Geneva, Switzerland: World Health Organization.

World Law Guide. 2011. Legislation Cambodia. http://www.lexadin.nl/wlg/legis/nofr/oeur/lxwecam.htm.

SAMPLE IMPLEMENTATION *Appendix* A

A.1 INTRODUCTION

The 2014 Cambodia Demographic and Health Survey is the fourth of its kind and follows similar surveys conducted in 2000, 2005, and 2010. The 2014 CDHS included a nationally representative sample of 15,825 households. All women age 15-49 who were usual members of a sampled household or who slept in the sampled household the night before the survey were eligible to be individually interviewed. The survey resulted in completed interviews with 17,578 de facto women. Similar to previous surveys, the 2014 CDHS was designed to provide information on fertility and childhood mortality, family planning, maternal and child health, and knowledge and behavior regarding AIDS and other sexually transmitted infections (STIs). The survey also collected data on domestic violence among women and micronutrients among mothers and children age 0-5. Survey estimates are reported for 19 study domains, of which 14 are individual provinces (Banteay Meanchey, Kampong Cham, Kampong Chhnang, Kampong Speu, Kampong Thom, Kandal, Kratie, Phnom Penh, Prey Veng, Pursat, Siem Reap, Svay Rieng, Takeo, and Otdar Meanchey) and 5 are groups of provinces:

Group 1: Battambang and Pailin
Group 2: Kampot and Kep
Group 3: Preah Sihanouk and Koh Kong
Group 4: Preah Vihear and Stung Treng
Group 5: Mondul Kiri and Ratanak Kiri

In addition, a male survey was conducted at the same time in a subsample consisting of one-third of the households selected. All men age 15-49 who were usual members of a subsampled household or who slept in the household the night before the survey were eligible for the male survey. The male survey collected information on family planning and knowledge and behavior regarding HIV/AIDS and other STIs. Also in this subsample, one eligible woman per household was selected randomly from all of the eligible women in the household to complete a domestic violence survey. In the other two-thirds of households that were not selected for the male survey, all women age 15-49 and all children under age 5 were eligible for height and weight measurements and for anemia testing. Furthermore, in a subsample consisting of one in every six of the selected clusters, a micronutrient survey was implemented among all women age 15-49 who had children under age 5 as well as among the children themselves.

A.2 SAMPLING FRAME

The sampling frame used for the 2014 CDHS was the complete list of all enumeration areas (EAs) created for the 2008 Cambodian General Population Census (GPC), provided by the National Institute of Statistics (NIS). The list had been updated in 2012 by NIS. The updated list consists of 28,696 EAs that encompass the entire country. An EA is either a village or part of a large village; it is linked to information about its administrative affiliation and its locality, number of residential households, and type of residence (urban or rural). A cartographic map delimiting each EA's boundaries was also created at the time of the 2008 GPC. Among the EAs, 4,307 are urban residences, and 24,389 are rural residences. There are 241 EAs that are special settlement areas and not ordinary residential areas; therefore, they are not eligible for the DHS surveys. These EAs were excluded from the sample selection. The final sampling frame for the 2014 CDHS comprised 28,455 eligible EAs.

The average size of the EAs (number of residential households) is 119 households in urban areas and 95 households in rural areas, with an overall average of 99 households per EA. Tables A.1 and A.2 show the distribution of households and the number of EAs by domain and by type of residence. In

Cambodia, 18 percent of residential households are in urban areas and 82 percent in rural areas. The largest domain (Kampong Cham) represents 13.1 percent of the total number of households in the country; the smallest domains (Otdar Meanchey and Mondul Kiri/Ratanak Kiri) represent just 1.4 percent each of total households. Table A.2 shows that, overall, 4,245 EAs were designated as urban and 24,210 as rural, with (as mentioned) an average size of 99 households per EA.

Table A.1 Distribution of households in the sampling frame (2008 GPC, updated) by survey domain and by residence, Cambodia 2014

		Number of households			Percentage of urban households	Percentage of households
Domain		Urban	Rural	Total		
1	Banteay Meanchey	37,165	107,280	144,445	25.7	5.1
2	Kampong Cham	24,875	342,704	367,579	6.8	13.1
3	Kampong Chhnang	8,294	92,218	100,512	8.3	3.6
4	Kampong Speu	10,530	138,615	149,145	7.1	5.3
5	Kampong Thom	6,674	127,156	133,830	5.0	4.8
6	Kandal	22,087	195,195	217,282	10.2	7.7
7	Kratie	7,400	57,797	65,197	11.4	2.3
8	Phnom Penh	247,135	40,757	287,892	85.8	10.2
9	Prey Veng	6,988	219,272	226,260	3.1	8.0
10	Pursat	5,389	77,899	83,288	6.5	3.0
11	Siem Reap	33,934	144,878	178,812	19.0	6.4
12	Svay Rieng	3,562	111,196	114,758	3.1	4.1
13	Takeo	2,688	181,017	183,705	1.5	6.5
14	Otdar Meanchey	3,573	34,568	38,141	9.4	1.4
15	Battambang/Pailin	38,865	185,112	223,977	17.4	8.0
16	Kampot/Kep	10,778	125,928	136,706	7.9	4.9
17	Preah Sihanouk/Koh Kong	25,702	43,066	68,768	37.4	2.4
18	Preah Vihear/Stung Treng	5,234	48,231	53,465	9.8	1.9
19	Mondul Kiri/Ratanak Kiri	4,673	35,040	39,713	11.8	1.4
20	Cambodia	505,546	2,307,929	2,813,475	18.0	100.0

Table A.2 Distribution of enumeration areas in the sampling frame (2008 GPC, updated) and average size of EAs by survey domain and by residence, Cambodia 2014

		Number of EAs			Average EA size		
Domain		Urban	Rural	Total	Urban	Rural	Total
1	Banteay Meanchey	338	1,138	1,476	110	94	98
2	Kampong Cham	225	3,426	3,651	111	100	101
3	Kampong Chhnang	72	940	1,012	115	98	99
4	Kampong Speu	110	1,631	1,741	96	85	86
5	Kampong Thom	62	1,342	1,404	108	95	95
6	Kandal	187	2,050	2,237	118	95	97
7	Kratie	69	573	642	107	101	102
8	Phnom Penh	2,015	443	2,458	123	92	117
9	Prey Veng	63	2,343	2,406	111	94	94
10	Pursat	58	833	891	93	94	93
11	Siem Reap	235	1,503	1,738	144	96	103
12	Svay Rieng	34	1,214	1,248	105	92	92
13	Takeo	23	1,935	1,958	117	94	94
14	Otdar Meanchey	34	378	412	105	91	93
15	Battambang/Pailin	364	1,858	2,222	107	100	101
16	Kampot/Kep	95	1,241	1,336	113	101	102
17	Preah Sihanouk/Koh Kong	180	425	605	143	101	114
18	Preah Vihear/Stung Treng	45	514	559	116	94	96
19	Mondul Kiri/Ratanak Kiri	36	423	459	130	83	87
20	Cambodia	4,245	24,210	28,455	119	95	99

A.3 Sampling Methodology and Procedure

The sample for the 2014 CDHS was a stratified sample selected in two stages. Stratification was achieved by separating every survey domain into urban and rural areas. As a result, the 19 domains were stratified into 38 sampling strata in total. EAs were selected independently in every stratum via two-stage random selection process, according to the sample allocation shown in Table A.3. Implicit stratification with proportional allocation was achieved at each of the lower level administrative units by sorting the sampling frame before sample selection within each of the 38 sampling strata, according to administrative units, and by using a probability proportional to size selection strategy at the first stage of sampling.

Because of the large number of study domains and the relatively low fertility rate in most of the provinces, an equal size allocation of about 950 completed interviews in each domain among women was adopted with some adjustment to ensure comparable survey precision for fertility across domains. The sample allocated to each domain was then allocated to the urban and rural areas of the domain with a power allocation in order to oversample urban areas, given that Cambodia is predominantly rural. Phnom Penh was allocated a relatively larger sample size because of its low fertility and large proportion of urban residences. The allocated sample in terms of women interviewed was then converted to number of households and number of EAs by taking estimated nonresponse into account, by using the average number of eligible women age 15-49 per household, and by taking into account the fact that the sample design called for 24 households to be interviewed in each urban EA and 28 in each rural EA. Table A.3 shows the sample allocation in terms of number of EAs and number of households by domain and by type of residence. In total, 611 EAs were needed; 188 were selected from urban areas, and 423 were selected from rural areas.

In the first stage, the 611 sample EAs were selected with probability proportional to EA size according to the sample allocation shown in Table A.3. The EA size is the number of households in the EA. After the sample selection of EAs and before the main survey, a household listing operation was carried out in all of the selected EAs, and the resulting lists of households served as the sampling frame for the selection of households in the second stage. Some of the selected EAs were large in size. In order to reduce the task of household listing, selected EAs that had more than 200 households were segmented; only one segment was selected randomly to include in the survey, with a selection probability proportional to segment size. Household listing was conducted only in the selected segment. Thus, in the 2014 CDHS, a sample "cluster" was either an entire EA or a segment of an EA.

Table A.3 Sample allocation of EAs and households by domain and by type of residence, Cambodia 2014

	Allocation of EAs			Allocation of households		
Domain	Urban	Rural	Total	Urban	Rural	Total
Banteay Meanchey	12	20	32	288	560	848
Kampong Cham	8	24	32	192	672	864
Kampong Chhnang	8	23	31	192	644	836
Kampong Speu	8	24	32	192	672	864
Kampong Thom	7	25	32	168	700	868
Kandal	9	23	32	216	644	860
Kratie	9	23	32	216	644	860
Phnom Penh	35	6	41	840	168	1,008
Prey Veng	6	26	32	144	728	872
Pursat	7	24	31	168	672	840
Siem Reap	11	21	32	264	588	852
Svay Rieng	6	26	32	144	728	872
Takeo	4	27	31	96	756	852
Otdar Meanchey	8	23	31	192	644	836
Battambang/Pailin	10	21	31	240	588	828
Kampot/Kep	8	24	32	192	672	864
Preah Sihanouk/Koh Kong	14	18	32	336	504	840
Preah Vihear/Stung Treng	9	23	32	216	644	860
Mondul Kiri/Ratanak Kiri	9	22	31	216	616	832
Cambodia	188	423	611	4,512	11,844	16,356

In the second selection stage, a fixed number of 24 households were selected from every urban cluster, and a fixed number of 28 households were selected from every rural cluster, through an equal probability systematic sampling process. The number of sample households from urban and rural areas was 4,512 and 11,844, respectively, with a total sample size of 16,356 households. The sample households for the 2014 CDHS were preselected in the central office before the main survey, and a household selection spreadsheet was prepared to facilitate this household selection. During the main survey, the interviewers were asked to interview only the preselected households. To prevent bias, no replacement was allowed for unfound or nonresponding households. All women age 15-49 in the selected households and men age 15-49 in the subsample of households for male survey were eligible for the individual survey.

Table A.4 shows the expected number of interviews with women and men by domain and by type of residence. The expected numbers of completed interviews with women were 5,989 and 12,749 for urban and rural areas, respectively, with a total of 18,738 interviews. For the male survey, conducted in one in every three households selected for the female survey, the expected numbers of completed interviews with men were 1,710 and 4,046 for urban and rural areas, respectively, for a total of 5,756 interviews.

Table A.4 Sample allocation of expected number of interviews of women and men by domain and by type of residence, Cambodia 2014

	Women completed			Men completed		
Domain	Urban	Rural	Total	Urban	Rural	Total
Banteay Meanchey	382	604	986	109	192	301
Kampong Cham	254	723	977	72	229	301
Kampong Chhnang	254	693	947	72	220	292
Kampong Speu	254	723	977	72	229	301
Kampong Thom	223	754	977	64	239	303
Kandal	287	693	980	82	220	302
Kratie	287	693	980	82	220	302
Phnom Penh	1,116	180	1,296	319	58	377
Prey Veng	191	784	975	55	249	304
Pursat	223	723	946	64	229	293
Siem Reap	350	633	983	100	201	301
Svay Rieng	191	784	975	55	249	304
Takeo	128	814	942	37	258	295
Otdar Meanchey	254	693	947	72	220	292
Battambang/Pailin	319	633	952	91	201	292
Kampot/Kep	254	723	977	72	229	301
Preah Sihanouk/Koh Kong	448	543	991	128	173	301
Preah Vihear/Stung Treng	287	693	980	82	220	302
Mondul Kiri/Ratanak Kiri	287	663	950	82	210	292
Cambodia	5,989	12,749	18,738	1,710	4,046	5,756

Note: The male survey was conducted in one of every three households selected for the 2014 CDHS.

The number of expected interviews was calculated based on several facts obtained from the 2010 CDHS: (1) there were an average of 1.42 and 1.15 women age 15-49 in each urban and rural household, respectively, with small variations across domains; (2) the household gross response rate was 96 percent for both urban and rural areas; (3) the individual response rate for women was 98 percent in both urban and rural areas; (4) there were on average 1.25 and 1.05 men age 15-49 in each urban and rural household, respectively, with small variations across domains; and (5) the individual response rate for men was 95 percent in both urban and rural areas.

A.4 SAMPLING PROBABILITIES

Due to the nonproportional allocation of the sample to the different domains and to urban and rural areas, along with possible differences in response rates, sampling weights are required for any analysis using the 2014 CDHS data to ensure the actual representativeness of the survey results at the national as well as the regional level. Since the 2014 CDHS sample was a two-stage stratified cluster sample, sampling weights were calculated based on sampling probabilities separately for each sampling stage and for each cluster. The following notations were used:

P_{1hi}: first-stage sampling probability of the ith cluster in stratum h
P_{2hi}: second-stage sampling probability within the ith cluster (household selection)

Let n_h be the number of clusters selected in stratum h, M_{hi} the number of households according to the sampling frame in the ith cluster, and $\sum M_{hi}$ the total number of households in the stratum. The probability of selecting the ith cluster in the 2014 CDHS sample is calculated as follows:

$$\frac{n_h \, M_{hi}}{\sum M_{hi}}$$

Let s_{hi} be the proportion of households in the selected segment compared with the total number of households in the EA *i* in stratum *h* if the EA is segmented; otherwise, $s_{hi} = 1$. Then the probability of selecting cluster *i* in stratum *h* in the sample is:

$$P_{1hi} = \frac{n_h \, M_{hi}}{\sum M_{hi}} \times s_{hi}$$

Let L_{hi} be the number of households listed in the household listing operation in cluster *i* in stratum *h*, and let t_{hi} be the number of households selected in the cluster. The second stage's selection probability for each household in the cluster is calculated as follows:

$$P_{2hi} = \frac{t_{hi}}{L_{hi}}$$

The overall selection probability of each household in cluster *i* of stratum *h* is therefore the product of the two stages of selection probabilities:

$$P_{hi} = P_{1hi} \times P_{2hi}$$

The sampling weight for each household in cluster *i* of stratum *h* is the inverse of its overall selection probability:

$$W_{hi} = 1 / P_{hi}$$

Sampling weights were adjusted for household nonresponse as well as for individual nonresponse in order to calculate the survey weights. A spreadsheet containing all sampling parameters and selection probabilities was prepared to facilitate the calculation of survey weights. Several sets of survey weights were calculated:

- one set for all households and for women's individual surveys
- one set for households selected for the male survey and for men's individual surveys
- one set for women selected for the domestic violence survey
- one set for households in the clusters selected for the micronutrient survey

The differences between the household weights and the individual weights are due to individual nonresponse. The domestic violence survey weight takes the number of eligible women in the household into account because of the selection of only one woman per household. The final survey weights were normalized so that the total number of weighted cases was equal to the total number of unweighted cases at the national level, for both household weights and individual women's and men's weights. The normalized weights are relative weights that are valid for estimating means, proportions, and ratios but are not valid for estimating population totals or pooled data.

Tables A.5 and A.6 show details on response rates for households, women, and men by residence and survey domain.

Table A.5 Sample implementation: Women

Percent distribution of households and eligible women by results of the household and individual interviews, and household, eligible women's, and overall women's response rates, according to urban-rural residence and domain (unweighted), Cambodia 2014

	Residence		Domain									
Result	Urban	Rural	Banteay Meanchey	Kampong Cham	Kampong Chhnang	Kampong Speu	Kampong Thom	Kandal	Kratie	Phnom Penh	Prey Veng	Pursat
**Selected households**												
Completed (C)	96.8	96.7	92.7	96.9	98.9	97.0	97.2	97.3	96.9	96.0	97.6	97.6
Household present but no competent respondent at home (HP)	0.3	0.6	0.1	0.3	0.4	0.7	0.0	0.5	0.2	0.5	1.9	1.1
Postponed (P)	0.0	0.0	0.0	0.0	0.0	0.0	0.0	0.0	0.0	0.0	0.0	0.0
Refused (R)	0.2	0.0	0.0	0.0	0.0	0.0	0.0	0.0	0.0	0.6	0.0	0.0
Dwelling not found (DNF)	0.2	0.1	0.9	0.1	0.0	0.0	0.0	0.0	0.0	0.2	0.0	0.0
Household absent (HA)	1.6	2.3	5.5	2.4	0.7	1.9	2.8	2.1	2.8	2.0	0.2	0.8
Dwelling vacant/address not a dwelling (DV)	0.8	0.2	0.6	0.2	0.0	0.3	0.0	0.1	0.1	0.7	0.2	0.4
Dwelling destroyed (DD)	0.0	0.0	0.0	0.0	0.0	0.0	0.0	0.0	0.0	0.0	0.0	0.0
Other (O)	0.0	0.0	0.1	0.0	0.0	0.1	0.0	0.0	0.0	0.0	0.0	0.1
Total	100.0	100.0	100.0	100.0	100.0	100.0	100.0	100.0	100.0	100.0	100.0	100.0
Number of sampled households	4,512	11,844	848	864	836	864	868	860	860	1,008	872	840
Household response rate (HRR)[1]	99.2	99.3	98.9	99.5	99.6	99.3	100.0	99.5	99.8	98.7	98.0	98.9
**Eligible women**												
Completed (EWC)	97.0	97.9	98.4	98.3	99.2	96.4	99.6	94.9	99.3	94.9	97.8	98.5
Not at home (EWNH)	1.7	1.4	0.9	1.0	0.4	2.0	0.2	4.3	0.2	1.9	1.2	0.6
Refused (EWR)	0.8	0.2	0.0	0.3	0.0	0.8	0.0	0.2	0.0	2.4	0.1	0.5
Partly completed (EWPC)	0.1	0.0	0.0	0.0	0.0	0.1	0.0	0.0	0.0	0.2	0.1	0.2
Incapacitated (EWI)	0.5	0.5	0.7	0.3	0.3	0.8	0.2	0.5	0.5	0.6	0.7	0.2
Other (EWO)	0.0	0.0	0.0	0.0	0.0	0.0	0.0	0.0	0.0	0.1	0.0	0.0
Total	100.0	100.0	100.0	100.0	100.0	100.0	100.0	100.0	100.0	100.0	100.0	100.0
Number of women	5,842	12,170	823	868	906	1,060	909	922	880	1,476	837	872
Eligible women response rate (EWRR)[2]	97.0	97.9	98.4	98.3	99.2	96.4	99.6	94.9	99.3	94.9	97.8	98.5
Overall women response rate (OWRR)[3]	96.3	97.2	97.3	97.8	98.9	95.7	99.6	94.5	99.1	93.6	95.9	97.4

Continued…

Table A.5—*Continued*

Result	Siem Reap	Svay Rieng	Takeo	Otdar Meanchey	Battam-bang/ Pailin	Kampot/ Kep	Preah Sihanouk/ Koh Kong	Preah Vihear/ Stung Treng	Mondul Kiri/ Ratanak Kiri	Total
	Domain									
**Selected households**										
Completed (C)	96.1	97.0	97.1	93.5	96.4	98.4	97.3	96.7	97.7	96.8
Household present but no competent respondent at home (HP)	1.8	0.1	0.1	0.0	0.0	0.3	0.2	0.9	0.8	0.5
Postponed (P)	0.1	0.0	0.0	0.0	0.0	0.0	0.0	0.0	0.0	0.0
Refused (R)	0.1	0.0	0.0	0.0	0.1	0.0	0.0	0.1	0.1	0.1
Dwelling not found (DNF)	0.1	0.1	0.0	0.0	0.1	0.0	0.0	0.0	0.0	0.1
Household absent (HA)	1.3	2.6	2.2	5.9	2.5	1.3	1.1	1.4	1.1	2.1
Dwelling vacant/address not a dwelling (DV)	0.4	0.1	0.6	0.5	0.7	0.0	1.3	0.8	0.2	0.4
Dwelling destroyed (DD)	0.1	0.0	0.0	0.0	0.1	0.0	0.1	0.0	0.0	0.0
Other (O)	0.0	0.0	0.0	0.1	0.0	0.0	0.0	0.0	0.0	0.0
Total	100.0	100.0	100.0	100.0	100.0	100.0	100.0	100.0	100.0	100.0
Number of sampled households	852	872	852	836	828	864	840	860	832	16,356
Household response rate (HRR)[1]	97.8	99.8	99.9	100.0	99.8	99.6	99.8	98.9	99.0	99.3
**Eligible women**										
Completed (EWC)	97.9	98.2	97.0	99.0	98.7	97.2	96.5	97.9	96.9	97.6
Not at home (EWNH)	1.5	1.0	2.1	0.6	1.0	1.9	3.0	1.4	2.4	1.5
Refused (EWR)	0.0	0.1	0.2	0.0	0.1	0.1	0.1	0.2	0.5	0.4
Partly completed (EWPC)	0.0	0.0	0.0	0.0	0.0	0.0	0.0	0.0	0.0	0.0
Incapacitated (EWI)	0.6	0.7	0.7	0.4	0.1	0.8	0.5	0.5	0.2	0.5
Other (EWO)	0.0	0.0	0.0	0.0	0.0	0.0	0.0	0.0	0.0	0.0
Total	100.0	100.0	100.0	100.0	100.0	100.0	100.0	100.0	100.0	100.0
Number of women	963	837	895	831	878	905	1,047	1,108	995	18,012
Eligible women response rate (EWRR)[2]	97.9	98.2	97.0	99.0	98.7	97.2	96.5	97.9	96.9	97.6
Overall women response rate (OWRR)[3]	95.8	98.0	96.9	99.0	98.5	96.9	96.2	96.9	95.9	96.9

[1] Using the number of households falling into specific response categories, the household response rate (HRR) is calculated as:

$$\frac{100 * C}{C + HP + P + R + DNF}$$

[2] The eligible women's response rate (EWRR) is equivalent to the percentage of interviews completed (EWC):

$$\frac{100 * EWC}{EWC + EWNH + EWP + EWR + EWPC + EWI + EWO}$$

[3] The overall women's response rate (OWRR) is calculated as OWRR = HRR * EWRR/100.

Table A.6 Sample implementation: Men

Percent distribution of households and eligible men by results of the household and individual interviews, and household, eligible men's, and overall men's response rates, according to urban-rural residence and domain (unweighted), Cambodia 2014

	Residence		Domain									
Result	Urban	Rural	Banteay Meanchey	Kampong Cham	Kampong Chhnang	Kampong Speu	Kampong Thom	Kandal	Kratie	Phnom Penh	Prey Veng	Pursat
**Selected households**												
Completed (C)	96.4	97.0	91.2	98.4	98.6	96.7	96.4	98.0	96.7	96.5	98.1	98.6
Household present but no competent respondent at home (HP)	0.2	0.4	0.0	0.3	0.3	0.3	0.0	0.0	0.0	0.3	1.3	0.3
Refused (R)	0.2	0.0	0.0	0.0	0.0	0.0	0.0	0.0	0.0	0.3	0.0	0.0
Dwelling not found (DNF)	0.1	0.1	1.4	0.0	0.0	0.0	0.0	0.0	0.0	0.0	0.0	0.0
Household absent (HA)	1.8	2.4	6.4	1.3	1.0	2.3	3.6	2.0	3.0	1.8	0.3	1.0
Dwelling vacant/address not a dwelling (DV)	1.2	0.2	1.0	0.0	0.0	0.7	0.0	0.0	0.3	1.2	0.3	0.0
Dwelling destroyed (DD)	0.1	0.0	0.0	0.0	0.0	0.0	0.0	0.0	0.0	0.0	0.0	0.0
Total	100.0	100.0	100.0	100.0	100.0	100.0	100.0	100.0	100.0	100.0	100.0	100.0
Number of sampled households	1,503	4,229	296	304	294	304	306	302	302	340	308	296
Household response rate (HRR)[1]	99.5	99.5	98.5	99.7	99.7	99.7	100.0	100.0	100.0	99.4	98.7	99.7
**Eligible men**												
Completed (EMC)	93.8	95.0	97.0	96.2	98.0	92.1	99.2	83.0	96.3	90.7	93.5	98.5
Not at home (EMNH)	4.3	3.7	2.2	2.6	2.0	5.5	0.0	14.6	1.9	3.7	5.7	1.1
Postponed (EMP)	0.1	0.1	0.4	0.0	0.0	0.0	0.0	1.4	0.0	0.0	0.0	0.0
Refused (EMR)	1.2	0.3	0.0	0.3	0.0	1.4	0.0	0.3	0.0	4.4	0.0	0.0
Partly completed (EMPC)	0.0	0.1	0.0	0.3	0.0	0.0	0.0	0.0	0.0	0.0	0.0	0.0
Incapacitated (EMI)	0.5	0.8	0.4	0.6	0.0	1.0	0.8	0.7	1.9	0.9	0.8	0.4
Other (EMO)	0.1	0.0	0.0	0.0	0.0	0.0	0.0	0.0	0.0	0.2	0.0	0.0
Total	100.0	100.0	100.0	100.0	100.0	100.0	100.0	100.0	100.0	100.0	100.0	100.0
Number of men	1,641	3,843	230	312	256	292	263	288	268	431	261	265
Eligible men response rate (EMRR)[2]	93.8	95.0	97.0	96.2	98.0	92.1	99.2	83.0	96.3	90.7	93.5	98.5
Overall men response rate (OMRR)[3]	93.3	94.5	95.5	95.8	97.7	91.8	99.2	83.0	96.3	90.2	92.3	98.2

Continued…

Table A.6—*Continued*

Result	Siem Reap	Svay Rieng	Takeo	Otdar Meanchey	Battam-bang/ Pailin	Kampot/ Kep	Preah Sihanouk/ Koh Kong	Preah Vihear/ Stung Treng	Mondul Kiri/ Ratanak Kiri	Total
	Domain									
**Selected households**										
Completed (C)	95.3	95.1	97.0	93.9	95.5	99.3	98.3	97.4	98.6	96.8
Household present but no competent respondent at home (HP)	2.3	0.0	0.0	0.0	0.0	0.0	0.0	0.7	1.0	0.4
Refused (R)	0.0	0.0	0.0	0.0	0.0	0.0	0.0	0.3	0.3	0.1
Dwelling not found (DNF)	0.0	0.0	0.0	0.0	0.3	0.0	0.0	0.0	0.0	0.1
Household absent (HA)	1.7	4.9	2.7	5.4	2.4	0.7	1.0	0.7	0.0	2.2
Dwelling vacant/address not a dwelling (DV)	0.3	0.0	0.3	0.7	1.7	0.0	0.7	1.0	0.0	0.4
Dwelling destroyed (DD)	0.3	0.0	0.0	0.0	0.0	0.0	0.0	0.0	0.0	0.0
Total	100.0	100.0	100.0	100.0	100.0	100.0	100.0	100.0	100.0	100.0
Number of sampled households	298	308	301	294	290	304	291	302	292	5,732
Household response rate (HRR)[1]	97.6	100.0	100.0	100.0	99.6	100.0	100.0	99.0	98.6	99.5
**Eligible men**										
Completed (EMC)	96.6	96.0	90.0	97.2	97.3	95.0	91.1	95.8	98.0	94.6
Not at home (EMNH)	2.1	1.2	8.6	2.8	2.3	3.7	8.5	2.4	2.0	3.9
Postponed (EMP)	0.0	0.0	0.0	0.0	0.0	0.0	0.0	0.3	0.0	0.1
Refused (EMR)	0.0	1.2	0.0	0.0	0.0	0.3	0.0	0.3	0.0	0.5
Partly completed (EMPC)	0.0	0.4	0.0	0.0	0.0	0.0	0.0	0.0	0.0	0.0
Incapacitated (EMI)	1.4	1.2	1.1	0.0	0.4	1.0	0.3	1.0	0.0	0.7
Other (EMO)	0.0	0.0	0.4	0.0	0.0	0.0	0.0	0.0	0.0	0.0
Total	100.0	100.0	100.0	100.0	100.0	100.0	100.0	100.0	100.0	100.0
Number of men	292	247	280	285	256	299	316	286	357	5,484
Eligible men response rate (EMRR)[2]	96.6	96.0	90.0	97.2	97.3	95.0	91.1	95.8	98.0	94.6
Overall men response rate (OMRR)[3]	94.3	96.0	90.0	97.2	96.9	95.0	91.1	94.8	96.7	94.1

[1] Using the number of households falling into specific response categories, the household response rate (HRR) is calculated as:

$$\frac{100 * C}{C + HP + P + R + DNF}$$

[2] The eligible men's response rate (EMRR) is equivalent to the percentage of interviews completed (EMC):

$$\frac{100 * EMC}{EMC + EMNH + EMP + EMR + EMPC + EMI + EMO}$$

[3] The overall men's response rate (OMRR) is calculated as OMRR = HRR * EMRR/100.

ESTIMATES OF SAMPLING ERRORS — Appendix B

The estimates from a sample survey are affected by two types of errors: (1) nonsampling errors and (2) sampling errors. Nonsampling errors are the results of mistakes made in implementing data collection and data processing, such as failure to locate and interview the correct household, misunderstanding of the questions on the part of either the interviewer or the respondent, and data entry errors. Although numerous efforts were made during the implementation of the 2014 Cambodia Demographic and Health Survey (CDHS) to minimize this type of error, nonsampling errors are impossible to avoid and difficult to evaluate statistically.

Sampling errors, on the other hand, can be evaluated statistically. The sample of respondents selected in the 2014 CDHS is only one of many samples that could have been selected from the same population, using the same design and identical size. Each of these samples would yield results that differ somewhat from the results of the actual sample selected. Sampling errors are a measure of the variability between all possible samples. Although the degree of variability is not known exactly, it can be estimated from the survey results.

A sampling error is usually measured in terms of the *standard error* for a particular statistic (mean, percentage, etc.), which is the square root of the variance. The standard error can be used to calculate confidence intervals within which the true value for the population can reasonably be assumed to fall. For example, for any given statistic calculated from a sample survey, the value of that statistic will fall within a range of plus or minus two times the standard error of that statistic in 95 percent of all possible samples of identical size and design.

If the sample of respondents had been selected as a simple random sample, it would have been possible to use straightforward formulas for calculating sampling errors. However, the 2014 CDHS sample is the result of a multistage stratified design, and, consequently, it was necessary to use more complex formulae. The computer software used to calculate sampling errors for the 2014 CDHS is an SAS program. This program used the Taylor linearization method for variance estimation for survey estimates that are means or proportions. The Jackknife repeated replication method is used for variance estimation of more complex statistics such as fertility and mortality rates.

The Taylor linearization method treats any percentage or average as a ratio estimate, $r = y/x$, where y represents the total sample value for variable y, and x represents the total number of cases in the group or subgroup under consideration. The variance of r is computed using the formula given below, with the standard error being the square root of the variance:

$$SE^2(r) = var(r) = \frac{1}{x^2}\sum_{h=1}^{H}\left[(1-f_h)\frac{m_h}{m_h-1}\left(\sum_{i=1}^{m_h} z_{hi}^2 - \frac{z_h^2}{m_h}\right)\right]$$

in which

$$z_{hi} = y_{hi} - rx_{hi}\text{, and } z_h = y_h - rx_h$$

where h represents the stratum which varies from 1 to H,
m_h is the total number of clusters selected in the hth stratum,
y_{hi} is the sum of the weighted values of variable y in the ith cluster in the hth stratum,
x_{hi} is the sum of the weighted number of cases in the ith cluster in the hth stratum, and
f_h is the sampling fraction of PSU in the hth stratum.

The Jackknife repeated replication method derives estimates of complex rates from each of several replications of the parent sample and calculates standard errors for these estimates using simple formulae. Each replication considers *all but one* cluster in the calculation of the estimates. Pseudo-independent replications are thus created. In the 2014 CDHS, there were 611 non-empty clusters. Hence, 611 replications were created. The variance of a rate r is calculated as follows:

$$SE^2(r) = var(r) = \frac{1}{k(k-1)}\sum_{i=1}^{k}(r_i - r)^2$$

in which

$$r_i = kr - (k-1)r_{(i)}$$

where r is the estimate computed from the full sample of 611 clusters,
$r_{(i)}$ is the estimate computed from the reduced sample of 610 clusters (i^{th} cluster excluded), and
k is the total number of clusters.

In addition to the standard error, the program computes the design effect (DEFT) for each estimate, which is defined as the ratio between the standard error using the given sample design and the standard error that would result if a simple random sample had been used. A DEFT value of 1.0 indicates that the sample design is as efficient as a simple random sample, while a value greater than 1.0 indicates the increase in the sampling error due to the use of a more complex and less statistically efficient design, such as multistage and cluster selection. The program also computes the relative standard error and the confidence limits for the estimates.

Sampling errors for the 2014 CDHS are calculated for selected variables considered to be of primary interest for the women's survey and the men's survey. The results are presented in this appendix for the country as a whole, for urban and rural areas, and for each of the 19 study domains. For each variable, the type of statistic (mean, proportion, or rate) and the base population are given in Table B.1. Tables B.2 to B.23 present the value of the statistic (R), its standard error (SE), the number of unweighted (N) and weighted (WN) cases, the design effect (DEFT), the relative standard error (SE/R), and the 95 percent confidence limits (R±2SE) for each variable. The DEFT is considered undefined when the standard error considering a simple random sample is zero (when the estimate is close to 0 or 1). In the case of the total fertility rate, the number of unweighted cases is not relevant, as there is no known unweighted value for woman-years of exposure to childbearing.

The confidence interval (e.g., as calculated for *children ever born to women over age 40*) can be interpreted as follows: the overall average from the national sample is 3.850, and its standard error is 0.051. Therefore, to obtain the 95 percent confidence limits, one adds and subtracts twice the standard error to the sample estimate, that is, 3.850±2×0.051. There is a high probability (95 percent) that the *true* average number of children ever born to all women over age 40 is between 3.748 and 3.952.

For the total sample, the value of the design effect (DEFT), averaged over all variables for the women's survey, is 1.478. This means that, due to multistage clustering of the sample, the average standard error is increased by a factor of 1.478 over that in an equivalent simple random sample.

Table B.1 List of selected variables for sampling errors, Cambodia 2014

Variable	Estimate	Base population
WOMEN		
Urban residence	Proportion	All women 15-49
No schooling	Proportion	All women 15-49
Secondary and higher education	Proportion	All women 15-49
Never married (never in union)	Proportion	All women 15-49
Currently married (in union)	Proportion	All women 15-49
Married before age 20	Proportion	Women 25-49
Had first sexual intercourse before age 18	Proportion	Women 25-49
Children ever born	Mean	All women 15-49
Children surviving	Mean	All women 15-49
Children ever born to women over age 40	Mean	Women age 40-49
Knowing any contraceptive method	Proportion	Currently married women 15-49
Knowing any modern contraceptive method	Proportion	Currently married women 15-49
Currently using any method	Proportion	Currently married women 15-49
Currently using a modern method	Proportion	Currently married women 15-49
Currently using daily pill	Proportion	Currently married women 15-49
Currently using condom	Proportion	Currently married women 15-49
Currently using injectables	Proportion	Currently married women 15-49
Used public sector source	Proportion	Current modern users of women 15-49
Want no more children	Proportion	Currently married women 15-49
Want to delay at least 2 years	Proportion	Currently married women 15-49
Mothers protected against tetanus for last birth	Proportion	Last birth in last 5 years
Mothers received medical assistance at delivery	Proportion	Births in last 5 years
Had diarrhea in last 2 weeks	Proportion	Children under 5
Treated with ORS packets or pre-packed liquid	Proportion	Children under 5 with diarrhea in last 2 weeks
Consulted medical personnel for diarrhea	Proportion	Children under 5 with diarrhea in last 2 weeks
Having health card, seen	Proportion	Children 12-23 months
Received BCG vaccination	Proportion	Children 12-23 months
Received DPT vaccination (3 doses)	Proportion	Children 12-23 months
Received polio vaccination (3 doses)	Proportion	Children 12-23 months
Received measles vaccination	Proportion	Children 12-23 months
Fully immunized	Proportion	Children 12-23 months
Weight-for-height (< -2 SD)	Proportion	Children under 5 who were measured
Height-for-age (< -2 SD)	Proportion	Children under 5 who were measured
Weight-for-age (< -2 SD)	Proportion	Children under 5 who were measured
Prevalence of anemia (children 6-59 months)	Proportion	Children 6-59 months who were tested
Prevalence of anemia (women 15-49)	Proportion	All women 15-49 who were tested
Body mass index (BMI) < 18.5	Proportion	All women 15-49 who were measured
Had an HIV test and received results	Proportion	All women 15-49 who had an HIV test in last 12 months
Accepting attitudes towards people with HIV	Proportion	All women 15-49
Total fertility rate (last 3 years)	Rate	Woman-years of exposure to childbearing
Neonatal mortality rate[1]	Rate	Number of births
Postneonatal mortality rate[1]	Rate	Number of births
Infant mortality rate[1]	Rate	Number of births
Child mortality rate[1]	Rate	Number of births
Under-5 mortality rate[1]	Rate	Number of births
MEN		
Urban residence	Proportion	All men 15-49
No schooling	Proportion	All men 15-49
Secondary and higher education	Proportion	All men 15-49
Never married (never in union)	Proportion	All men 15-49
Currently married (in union)	Proportion	All men 15-49
Had first sexual intercourse before age 18	Proportion	All men 25-49
Want no more children	Proportion	All currently married men 15-49
Want to delay birth at least 2 years	Proportion	All currently married men 15-49
Had paid sex in past 12 months	Proportion	All men 15-49
Had HIV test and received results	Proportion	All men 15-49 who had an HIV test in last 12 months
Accepting attitudes towards people with HIV	Proportion	All men 15-49

[1] Mortality rates are calculated for the last 5 years for the total sample and 10 years for domain samples.

Table B.2 Sampling errors: Total sample, Cambodia 2014

Variable	R	SE	N	WN	DEFT	SE/R	R-2SE	R+2SE
				WOMEN				
Urban residence	0.185	0.007	17,578	17,578	2.444	0.039	0.171	0.199
No schooling	0.128	0.006	17,578	17,578	2.283	0.045	0.117	0.140
Secondary or higher education	0.401	0.008	17,578	17,578	2.281	0.021	0.384	0.418
Never married (never in union)	0.252	0.004	17,578	17,578	1.297	0.017	0.243	0.260
Currently married (in union)	0.677	0.005	17,578	17,578	1.310	0.007	0.668	0.686
Married before age 20	0.451	0.007	11,534	11,668	1.521	0.016	0.437	0.465
Had first sexual intercourse before age 18	0.244	0.006	11,534	11,668	1.476	0.024	0.232	0.256
Currently pregnant	0.053	0.002	17,578	17,578	1.199	0.038	0.049	0.057
Children ever born	1.933	0.022	17,578	17,578	1.479	0.011	1.890	1.977
Children surviving	1.775	0.019	17,578	17,578	1.437	0.011	1.737	1.813
Children ever born to women age 40-49	3.850	0.051	3,896	3,947	1.440	0.013	3.748	3.952
Know any contraceptive method	0.998	0.000	11,668	11,898	1.236	0.000	0.997	0.999
Know any modern contraceptive method	0.998	0.000	11,668	11,898	1.224	0.000	0.997	0.999
Currently using any method	0.563	0.007	11,668	11,898	1.521	0.012	0.549	0.577
Currently using a modern method	0.388	0.007	11,668	11,898	1.553	0.018	0.374	0.402
Currently using daily pill	0.176	0.006	11,668	11,898	1.685	0.034	0.164	0.187
Currently using male condoms	0.021	0.002	11,668	11,898	1.201	0.076	0.018	0.024
Currently using injectables	0.091	0.005	11,668	11,898	1.807	0.053	0.082	0.101
Used public sector source	0.472	0.012	4,583	4,663	1.672	0.026	0.448	0.497
Want no more children	0.516	0.006	11,668	11,898	1.359	0.012	0.503	0.528
Want to delay next birth at least 2 years	0.270	0.006	11,668	11,898	1.355	0.021	0.259	0.281
Mothers protected against tetanus for last birth	0.886	0.007	5,901	5,973	1.608	0.008	0.872	0.899
Mothers received medical assistance at delivery	0.890	0.009	7,165	7,253	2.129	0.010	0.872	0.909
Had diarrhea in the last 2 weeks	0.128	0.006	6,971	7,044	1.357	0.044	0.117	0.139
Treated with ORS packets or pre-packed liquid	0.352	0.024	855	902	1.429	0.068	0.304	0.400
Consulted medical personnel for diarrhea	0.555	0.023	855	902	1.335	0.042	0.508	0.601
Vaccination card seen	0.773	0.014	1,441	1,460	1.300	0.019	0.744	0.802
Received BCG vaccination	0.961	0.006	1,441	1,460	1.115	0.006	0.949	0.972
Received DPT vaccination (3 doses)	0.837	0.014	1,441	1,460	1.443	0.017	0.808	0.865
Received polio vaccination (3 doses)	0.823	0.014	1,441	1,460	1.382	0.017	0.795	0.851
Received measles vaccination	0.786	0.015	1,441	1,460	1.404	0.019	0.756	0.816
Received all basic vaccinations	0.734	0.016	1,441	1,460	1.396	0.022	0.702	0.767
Height-for-age (-2SD)	0.324	0.009	4,889	4,893	1.340	0.029	0.305	0.343
Weight-for-height (-2SD)	0.096	0.006	4,889	4,893	1.314	0.059	0.085	0.107
Weight-for-age (-2SD)	0.239	0.009	4,889	4,893	1.402	0.037	0.222	0.257
Prevalence of anemia (children 6-59 months)	0.555	0.011	4,468	4,456	1.404	0.019	0.534	0.576
Prevalence of anemia (women 15-49)	0.454	0.006	11,390	11,286	1.304	0.013	0.441	0.466
Body mass index (BMI) <18.5	0.140	0.004	10,686	10,624	1.284	0.031	0.131	0.149
Had an HIV test and received results in past 12 months	0.095	0.003	17,578	17,578	1.301	0.030	0.089	0.100
Accepting attitudes towards people with HIV	0.298	0.006	17,121	17,243	1.833	0.022	0.285	0.310
Total fertility rate (last 3 years)	2.724	0.055	49,864	49,875	1.342	0.020	2.614	2.834
Neonatal mortality (last 0-4 years)	17.802	2.444	7,213	7,312	1.456	0.137	12.913	22.691
Postneonatal mortality (last 0-4 years)	10.156	1.502	7,182	7,276	1.256	0.148	7.153	13.159
Infant mortality (last 0-4 years)	27.958	2.911	7,225	7,325	1.396	0.104	22.136	33.780
Child mortality (last 0-4 years)	6.956	1.190	7,011	7,147	1.236	0.171	4.576	9.335
Under-5 mortality (last 0-4 years)	34.719	3.293	7,251	7,350	1.457	0.095	28.134	41.305
				MEN				
Urban residence	0.167	0.008	5,190	5,190	1.530	0.047	0.152	0.183
No education	0.062	0.005	5,190	5,190	1.379	0.074	0.053	0.072
Secondary or higher education	0.520	0.012	5,190	5,190	1.731	0.023	0.496	0.544
Never married (in union)	0.320	0.008	5,190	5,190	1.240	0.025	0.304	0.337
Currently married (in union)	0.656	0.008	5,190	5,190	1.283	0.013	0.639	0.673
Had first sexual intercourse before age 18	0.099	0.007	3,363	3,430	1.367	0.071	0.085	0.114
Want no more children	0.507	0.011	3,320	3,405	1.271	0.022	0.485	0.529
Want to delay birth at least 2 years	0.325	0.011	3,320	3,405	1.323	0.033	0.304	0.347
Had paid sex in past 12 months	0.031	0.003	5,190	5,190	1.119	0.087	0.025	0.036
Had HIV test and received results in past 12 months	0.087	0.006	5,190	5,190	1.490	0.067	0.075	0.099
Accepting attitudes towards people with HIV	0.371	0.009	5,070	5,091	1.381	0.025	0.352	0.390

Table B.3 Sampling errors: Urban sample, Cambodia 2014

Variable	R	SE	N	WN	DEFT	SE/R	R-2SE	R+2SE
				WOMEN				
Urban residence	1.000	0.000	5,667	3,251	na	0.000	1.000	1.000
No schooling	0.054	0.006	5,667	3,251	1.925	0.107	0.042	0.065
Secondary or higher education	0.654	0.014	5,667	3,251	2.226	0.022	0.626	0.682
Never married (never in union)	0.365	0.010	5,667	3,251	1.573	0.028	0.345	0.385
Currently married (in union)	0.559	0.011	5,667	3,251	1.681	0.020	0.537	0.582
Married before age 20	0.362	0.012	3,659	2,080	1.549	0.034	0.338	0.387
Had first sexual intercourse before age 18	0.181	0.008	3,659	2,080	1.273	0.045	0.165	0.198
Currently pregnant	0.049	0.004	5,667	3,251	1.300	0.076	0.042	0.057
Children ever born	1.387	0.033	5,667	3,251	1.557	0.024	1.321	1.454
Children surviving	1.324	0.031	5,667	3,251	1.533	0.023	1.262	1.385
Children ever born to women age 40-49	2.968	0.092	1,171	642	1.691	0.031	2.784	3.152
Know any contraceptive method	0.999	0.001	3,330	1,818	1.057	0.001	0.998	1.000
Know any modern contraceptive method	0.999	0.001	3,330	1,818	1.057	0.001	0.998	1.000
Currently using any method	0.598	0.012	3,330	1,818	1.416	0.020	0.574	0.622
Currently using a modern method	0.328	0.010	3,330	1,818	1.196	0.030	0.309	0.348
Currently using daily pill	0.133	0.010	3,330	1,818	1.660	0.073	0.114	0.153
Currently using male condoms	0.051	0.006	3,330	1,818	1.604	0.120	0.039	0.064
Currently using injectables	0.029	0.004	3,330	1,818	1.276	0.129	0.021	0.036
Used public sector source	0.240	0.016	1,154	626	1.253	0.066	0.208	0.271
Want no more children	0.508	0.013	3,330	1,818	1.484	0.025	0.483	0.534
Want to delay next birth at least 2 years	0.255	0.011	3,330	1,818	1.493	0.044	0.232	0.278
Mothers protected against tetanus for last birth	0.929	0.010	1,628	876	1.509	0.010	0.909	0.948
Mothers received medical assistance at delivery	0.978	0.005	1,950	1,041	1.402	0.005	0.967	0.988
Had diarrhea in the last 2 weeks	0.125	0.011	1,923	1,033	1.412	0.090	0.103	0.148
Treated with ORS packets or pre-packed liquid	0.302	0.044	208	129	1.462	0.144	0.215	0.390
Consulted medical personnel for diarrhea	0.470	0.040	208	129	1.187	0.084	0.390	0.549
Vaccination card seen	0.713	0.032	406	217	1.403	0.045	0.649	0.777
Received BCG vaccination	0.976	0.010	406	217	1.349	0.011	0.955	0.997
Received DPT vaccination (3 doses)	0.929	0.015	406	217	1.190	0.017	0.898	0.959
Received polio vaccination (3 doses)	0.900	0.017	406	217	1.146	0.019	0.865	0.934
Received measles vaccination	0.907	0.017	406	217	1.185	0.019	0.873	0.942
Received all basic vaccinations	0.864	0.020	406	217	1.156	0.023	0.825	0.904
Height-for-age (-2SD)	0.237	0.017	1,311	674	1.425	0.073	0.203	0.272
Weight-for-height (-2SD)	0.075	0.008	1,311	674	1.058	0.106	0.059	0.091
Weight-for-age (-2SD)	0.148	0.013	1,311	674	1.237	0.086	0.123	0.174
Prevalence of anemia (children 6-59 months)	0.434	0.019	1,163	591	1.237	0.043	0.397	0.471
Prevalence of anemia (women 15-49)	0.394	0.010	3,765	2,156	1.266	0.026	0.374	0.414
Body mass index (BMI) <18.5	0.135	0.008	3,583	2,056	1.354	0.057	0.120	0.151
Had an HIV test and received results in past 12 months	0.128	0.006	5,667	3,251	1.461	0.051	0.115	0.141
Accepting attitudes towards people with HIV	0.298	0.010	5,629	3,238	1.649	0.034	0.278	0.319
Total fertility rate (last 3 years)	2.094	0.072	16,169	9,296	1.158	0.034	1.950	2.238
Neonatal mortality (last 0-9 years)	9.621	1.946	3,830	2,027	1.141	0.202	5.730	13.513
Postneonatal mortality (last 0-9 years)	3.870	1.061	3,830	2,021	0.820	0.274	1.748	5.992
Infant mortality (last 0-9 years)	13.491	2.310	3,830	2,027	1.058	0.171	8.871	18.111
Child mortality (last 0-9 years)	4.518	1.583	3,785	1,997	1.391	0.350	1.353	7.683
Under-5 mortality (last 0-9 years)	17.948	2.707	3,834	2,028	1.045	0.151	12.534	23.362
				MEN				
Urban residence	1.000	0.000	1,540	869	na	0.000	1.000	1.000
No education	0.010	0.002	1,540	869	0.869	0.215	0.006	0.015
Secondary or higher education	0.789	0.019	1,540	869	1.859	0.024	0.751	0.828
Never married (in union)	0.444	0.016	1,540	869	1.292	0.037	0.412	0.477
Currently married (in union)	0.536	0.016	1,540	869	1.245	0.030	0.504	0.567
Had first sexual intercourse before age 18	0.058	0.011	981	545	1.462	0.189	0.036	0.079
Want no more children	0.479	0.024	867	465	1.417	0.050	0.431	0.527
Want to delay birth at least 2 years	0.314	0.023	867	465	1.483	0.075	0.267	0.361
Had paid sex in past 12 months	0.066	0.008	1,540	869	1.305	0.125	0.049	0.082
Had HIV test and received results in past 12 months	0.124	0.011	1,540	869	1.326	0.090	0.102	0.147
Accepting attitudes towards people with HIV	0.349	0.016	1,527	864	1.326	0.046	0.317	0.382

Table B.4 Sampling errors: Rural sample, Cambodia 2014

Variable	R	SE	N	WN	DEFT	SE/R	R-2SE	R+2SE
				WOMEN				
Urban residence	0.000	0.000	11,911	14,327	na	na	0.000	0.000
No schooling	0.145	0.007	11,911	14,327	2.149	0.048	0.131	0.159
Secondary or higher education	0.343	0.010	11,911	14,327	2.209	0.028	0.324	0.363
Never married (never in union)	0.226	0.005	11,911	14,327	1.199	0.020	0.217	0.235
Currently married (in union)	0.704	0.005	11,911	14,327	1.184	0.007	0.694	0.713
Married before age 20	0.470	0.008	7,875	9,589	1.443	0.017	0.454	0.486
Had first sexual intercourse before age 18	0.257	0.007	7,875	9,589	1.405	0.027	0.243	0.271
Currently pregnant	0.054	0.002	11,911	14,327	1.131	0.043	0.049	0.059
Children ever born	2.057	0.026	11,911	14,327	1.380	0.012	2.006	2.109
Children surviving	1.877	0.022	11,911	14,327	1.342	0.012	1.833	1.921
Children ever born to women age 40-49	4.022	0.057	2,725	3,305	1.333	0.014	3.908	4.135
Know any contraceptive method	0.998	0.001	8,338	10,080	1.166	0.001	0.997	0.999
Know any modern contraceptive method	0.998	0.001	8,338	10,080	1.153	0.001	0.997	0.999
Currently using any method	0.557	0.008	8,338	10,080	1.460	0.014	0.541	0.573
Currently using a modern method	0.399	0.008	8,338	10,080	1.505	0.020	0.383	0.415
Currently using daily pill	0.183	0.007	8,338	10,080	1.601	0.037	0.170	0.197
Currently using male condoms	0.016	0.002	8,338	10,080	1.139	0.099	0.012	0.019
Currently using injectables	0.103	0.006	8,338	10,080	1.672	0.054	0.092	0.114
Used public sector source	0.508	0.014	3,429	4,038	1.622	0.027	0.481	0.536
Want no more children	0.517	0.007	8,338	10,080	1.289	0.014	0.503	0.531
Want to delay next birth at least 2 years	0.273	0.006	8,338	10,080	1.278	0.023	0.260	0.285
Mothers protected against tetanus for last birth	0.878	0.008	4,273	5,096	1.503	0.009	0.863	0.893
Mothers received medical assistance at delivery	0.876	0.011	5,215	6,212	1.978	0.012	0.855	0.897
Had diarrhea in the last 2 weeks	0.129	0.006	5,048	6,011	1.290	0.049	0.116	0.141
Treated with ORS packets or pre-packed liquid	0.361	0.027	647	772	1.358	0.076	0.306	0.415
Consulted medical personnel for diarrhea	0.569	0.026	647	772	1.287	0.046	0.516	0.621
Vaccination card seen	0.783	0.016	1,035	1,244	1.237	0.020	0.752	0.815
Received BCG vaccination	0.958	0.006	1,035	1,244	1.037	0.007	0.945	0.971
Received DPT vaccination (3 doses)	0.821	0.016	1,035	1,244	1.365	0.020	0.788	0.853
Received polio vaccination (3 doses)	0.809	0.016	1,035	1,244	1.318	0.020	0.777	0.842
Received measles vaccination	0.765	0.018	1,035	1,244	1.324	0.023	0.730	0.800
Received all basic vaccinations	0.712	0.019	1,035	1,244	1.328	0.026	0.674	0.749
Height-for-age (-2SD)	0.338	0.010	3,578	4,219	1.268	0.031	0.317	0.359
Weight-for-height (-2SD)	0.099	0.006	3,578	4,219	1.256	0.064	0.087	0.112
Weight-for-age (-2SD)	0.254	0.010	3,578	4,219	1.331	0.039	0.234	0.273
Prevalence of anemia (children 6-59 months)	0.574	0.012	3,305	3,864	1.352	0.021	0.550	0.597
Prevalence of anemia (women 15-49)	0.468	0.007	7,625	9,130	1.249	0.015	0.453	0.482
Body mass index (BMI) <18.5	0.141	0.005	7,103	8,569	1.220	0.036	0.131	0.151
Had an HIV test and received results in past 12 months	0.087	0.003	11,911	14,327	1.228	0.036	0.081	0.093
Accepting attitudes towards people with HIV	0.297	0.008	11,492	14,005	1.767	0.025	0.282	0.312
Total fertility rate (last 3 years)	2.885	0.061	33,695	40,579	1.269	0.021	2.763	3.008
Neonatal mortality (last 0-9 years)	22.915	1.882	10,747	12,804	1.213	0.082	19.150	26.679
Postneonatal mortality (last 0-9 years)	19.557	1.848	10,745	12,795	1.207	0.095	15.860	23.253
Infant mortality (last 0-9 years)	42.472	2.880	10,758	12,813	1.313	0.068	36.712	48.231
Child mortality (last 0-9 years)	10.393	1.301	10,639	12,648	1.292	0.125	7.792	12.995
Under-5 mortality (last 0-9 years)	52.424	3.302	10,786	12,845	1.376	0.063	45.820	59.027
				MEN				
Urban residence	0.000	0.000	3,650	4,321	na	na	0.000	0.000
No education	0.073	0.006	3,650	4,321	1.291	0.076	0.062	0.084
Secondary or higher education	0.466	0.014	3,650	4,321	1.699	0.030	0.438	0.494
Never married (in union)	0.296	0.009	3,650	4,321	1.217	0.031	0.277	0.314
Currently married (in union)	0.680	0.010	3,650	4,321	1.267	0.014	0.661	0.700
Had first sexual intercourse before age 18	0.107	0.008	2,382	2,885	1.278	0.076	0.091	0.123
Want no more children	0.512	0.012	2,453	2,940	1.210	0.024	0.487	0.536
Want to delay birth at least 2 years	0.327	0.012	2,453	2,940	1.254	0.036	0.303	0.351
Had paid sex in past 12 months	0.024	0.003	3,650	4,321	1.045	0.111	0.018	0.029
Had HIV test and received results in past 12 months	0.079	0.007	3,650	4,321	1.490	0.084	0.066	0.093
Accepting attitudes towards people with HIV	0.375	0.011	3,543	4,227	1.325	0.029	0.354	0.397

Table B.5 Sampling errors: Banteay Meanchey sample, Cambodia 2014

Variable	R	SE	N	WN	DEFT	SE/R	R-2SE	R+2SE
		WOMEN						
Urban residence	0.242	0.036	810	689	2.365	0.147	0.171	0.314
No schooling	0.128	0.027	810	689	2.292	0.211	0.074	0.182
Secondary or higher education	0.338	0.040	810	689	2.420	0.119	0.257	0.418
Never married (never in union)	0.216	0.012	810	689	0.816	0.055	0.192	0.239
Currently married (in union)	0.730	0.012	810	689	0.799	0.017	0.705	0.755
Married before age 20	0.455	0.034	535	457	1.559	0.074	0.387	0.522
Had first sexual intercourse before age 18	0.239	0.024	535	457	1.281	0.099	0.192	0.287
Currently pregnant	0.052	0.008	810	689	1.078	0.163	0.035	0.068
Children ever born	1.950	0.073	810	689	1.064	0.037	1.804	2.096
Children surviving	1.788	0.074	810	689	1.208	0.041	1.641	1.936
Children ever born to women age 40-49	3.997	0.197	201	169	1.245	0.049	3.603	4.390
Know any contraceptive method	0.998	0.002	567	503	1.130	0.002	0.993	1.002
Know any modern contraceptive method	0.998	0.002	567	503	1.130	0.002	0.993	1.002
Currently using any method	0.614	0.031	567	503	1.509	0.050	0.552	0.676
Currently using a modern method	0.510	0.041	567	503	1.958	0.081	0.428	0.593
Currently using daily pill	0.229	0.020	567	503	1.126	0.087	0.189	0.269
Currently using male condoms	0.022	0.007	567	503	1.084	0.303	0.009	0.036
Currently using injectables	0.147	0.046	567	503	3.076	0.314	0.055	0.240
Used public sector source	0.483	0.066	260	258	2.107	0.136	0.351	0.615
Want no more children	0.481	0.024	567	503	1.159	0.051	0.432	0.529
Want to delay next birth at least 2 years	0.325	0.025	567	503	1.249	0.076	0.276	0.374
Mothers protected against tetanus for last birth	0.938	0.013	259	219	0.853	0.014	0.912	0.964
Mothers received medical assistance at delivery	0.958	0.017	300	253	1.184	0.017	0.925	0.991
Had diarrhea in the last 2 weeks	0.130	0.020	297	250	0.987	0.157	0.089	0.170
Treated with ORS packets or pre-packed liquid	0.349	0.092	35	32	1.094	0.264	0.165	0.534
Consulted medical personnel for diarrhea	0.521	0.110	35	32	1.285	0.212	0.300	0.742
Vaccination card seen	0.888	0.037	70	63	0.995	0.042	0.814	0.963
Received BCG vaccination	0.964	0.026	70	63	1.157	0.027	0.912	1.015
Received DPT vaccination (3 doses)	0.940	0.029	70	63	1.042	0.031	0.882	0.999
Received polio vaccination (3 doses)	0.940	0.029	70	63	1.042	0.031	0.882	0.999
Received measles vaccination	0.934	0.030	70	63	1.023	0.032	0.874	0.994
Received all basic vaccinations	0.913	0.036	70	63	1.066	0.039	0.842	0.985
Height-for-age (-2SD)	0.286	0.025	283	241	0.907	0.089	0.235	0.337
Weight-for-height (-2SD)	0.078	0.018	283	241	1.070	0.228	0.043	0.114
Weight-for-age (-2SD)	0.170	0.023	283	241	0.978	0.133	0.125	0.215
Prevalence of anemia (children 6-59 months)	0.397	0.041	261	222	1.289	0.103	0.315	0.479
Prevalence of anemia (women 15-49)	0.305	0.027	528	450	1.332	0.087	0.252	0.359
Body mass index (BMI) <18.5	0.107	0.016	493	418	1.147	0.150	0.075	0.139
Had an HIV test and received results in past 12 months	0.121	0.015	810	689	1.268	0.120	0.092	0.150
Accepting attitudes towards people with HIV	0.167	0.018	805	686	1.332	0.105	0.132	0.202
Total fertility rate (last 3 years)	2.786	0.214	2,299	1,956	1.081	0.077	2.357	3.215
Neonatal mortality (last 0-9 years)	19.821	5.290	641	544	0.905	0.267	9.242	30.400
Postneonatal mortality (last 0-9 years)	9.159	5.084	639	542	1.288	0.555	0.000	19.328
Infant mortality (last 0-9 years)	28.980	5.976	641	544	0.896	0.206	17.028	40.931
Child mortality (last 0-9 years)	2.940	2.133	621	530	0.954	0.725	0.000	7.207
Under-5 mortality (last 0-9 years)	31.835	5.808	642	544	0.844	0.182	20.219	43.450
		MEN						
Urban residence	0.183	0.033	223	192	1.263	0.180	0.117	0.248
No education	0.075	0.020	223	192	1.117	0.264	0.035	0.114
Secondary or higher education	0.470	0.052	223	192	1.544	0.110	0.367	0.574
Never married (in union)	0.299	0.023	223	192	0.752	0.077	0.253	0.345
Currently married (in union)	0.682	0.023	223	192	0.724	0.033	0.637	0.728
Had first sexual intercourse before age 18	0.075	0.028	152	128	1.292	0.370	0.020	0.131
Want no more children	0.506	0.046	152	131	1.117	0.090	0.415	0.597
Want to delay birth at least 2 years	0.287	0.039	152	131	1.046	0.134	0.210	0.364
Had paid sex in past 12 months	0.021	0.009	223	192	0.982	0.450	0.002	0.040
Had HIV test and received results in past 12 months	0.052	0.018	223	192	1.191	0.342	0.016	0.087
Accepting attitudes towards people with HIV	0.294	0.053	223	192	1.711	0.179	0.189	0.399

Table B.6 Sampling errors: Kampong Cham sample, Cambodia 2014

Variable	R	SE	N	WN	DEFT	SE/R	R-2SE	R+2SE
				WOMEN				
Urban residence	0.086	0.012	853	2,021	1.272	0.142	0.062	0.111
No schooling	0.141	0.024	853	2,021	1.997	0.169	0.093	0.189
Secondary or higher education	0.329	0.034	853	2,021	2.115	0.104	0.261	0.397
Never married (never in union)	0.198	0.015	853	2,021	1.067	0.074	0.168	0.227
Currently married (in union)	0.737	0.014	853	2,021	0.909	0.019	0.710	0.765
Married before age 20	0.448	0.028	580	1,393	1.378	0.064	0.391	0.505
Had first sexual intercourse before age 18	0.245	0.025	580	1,393	1.374	0.100	0.196	0.294
Currently pregnant	0.035	0.008	853	2,021	1.240	0.222	0.020	0.051
Children ever born	2.157	0.082	853	2,021	1.186	0.038	1.992	2.322
Children surviving	1.985	0.068	853	2,021	1.128	0.034	1.848	2.121
Children ever born to women age 40-49	3.932	0.206	196	478	1.243	0.052	3.520	4.344
Know any contraceptive method	0.997	0.002	616	1,490	0.996	0.002	0.992	1.001
Know any modern contraceptive method	0.997	0.002	616	1,490	0.996	0.002	0.992	1.001
Currently using any method	0.447	0.028	616	1,490	1.418	0.064	0.390	0.503
Currently using a modern method	0.276	0.027	616	1,490	1.499	0.098	0.222	0.330
Currently using daily pill	0.114	0.021	616	1,490	1.597	0.180	0.073	0.155
Currently using male condoms	0.018	0.006	616	1,490	1.053	0.309	0.007	0.030
Currently using injectables	0.054	0.011	616	1,490	1.173	0.198	0.033	0.076
Used public sector source	0.491	0.045	174	410	1.195	0.093	0.400	0.582
Want no more children	0.512	0.018	616	1,490	0.901	0.036	0.475	0.548
Want to delay next birth at least 2 years	0.257	0.019	616	1,490	1.080	0.074	0.219	0.296
Mothers protected against tetanus for last birth	0.846	0.023	327	819	1.176	0.027	0.799	0.892
Mothers received medical assistance at delivery	0.915	0.028	402	1,008	2.014	0.031	0.859	0.972
Had diarrhea in the last 2 weeks	0.128	0.017	390	974	0.935	0.129	0.095	0.161
Treated with ORS packets or pre-packed liquid	0.341	0.094	47	124	1.273	0.277	0.152	0.530
Consulted medical personnel for diarrhea	0.526	0.084	47	124	1.130	0.160	0.358	0.695
Vaccination card seen	0.676	0.059	74	182	1.089	0.087	0.558	0.793
Received BCG vaccination	0.950	0.026	74	182	1.027	0.027	0.898	1.002
Received DPT vaccination (3 doses)	0.706	0.059	74	182	1.113	0.083	0.588	0.823
Received polio vaccination (3 doses)	0.711	0.055	74	182	1.043	0.077	0.601	0.820
Received measles vaccination	0.641	0.061	74	182	1.099	0.095	0.519	0.763
Received all basic vaccinations	0.568	0.066	74	182	1.151	0.116	0.436	0.700
Height-for-age (-2SD)	0.335	0.026	282	692	0.912	0.077	0.284	0.387
Weight-for-height (-2SD)	0.081	0.020	282	692	1.199	0.251	0.040	0.121
Weight-for-age (-2SD)	0.257	0.030	282	692	1.113	0.115	0.198	0.317
Prevalence of anemia (children 6-59 months)	0.627	0.028	258	625	0.965	0.045	0.571	0.683
Prevalence of anemia (women 15-49)	0.520	0.023	524	1,226	1.032	0.044	0.475	0.565
Body mass index (BMI) <18.5	0.102	0.014	501	1,176	1.017	0.136	0.074	0.130
Had an HIV test and received results in past 12 months	0.087	0.013	853	2,021	1.300	0.144	0.062	0.112
Accepting attitudes towards people with HIV	0.353	0.023	817	1,933	1.360	0.064	0.308	0.399
Total fertility rate (last 3 years)	3.268	0.211	2,412	5,712	1.176	0.065	2.845	3.690
Neonatal mortality (last 0-9 years)	25.467	7.256	774	1,926	1.149	0.285	10.955	39.978
Postneonatal mortality (last 0-9 years)	13.866	6.376	774	1,923	1.423	0.460	1.115	26.618
Infant mortality (last 0-9 years)	39.333	11.320	774	1,926	1.380	0.288	16.693	61.973
Child mortality (last 0-9 years)	8.669	2.974	759	1,883	0.971	0.343	2.722	14.616
Under-5 mortality (last 0-9 years)	47.661	11.318	777	1,933	1.316	0.237	25.024	70.298
				MEN				
Urban residence	0.096	0.011	300	663	0.662	0.117	0.074	0.119
No education	0.052	0.015	300	663	1.183	0.292	0.022	0.082
Secondary or higher education	0.393	0.038	300	663	1.334	0.096	0.318	0.468
Never married (in union)	0.262	0.025	300	663	0.975	0.095	0.213	0.312
Currently married (in union)	0.715	0.027	300	663	1.028	0.038	0.661	0.769
Had first sexual intercourse before age 18	0.137	0.029	201	470	1.181	0.210	0.079	0.194
Want no more children	0.515	0.041	202	474	1.165	0.080	0.432	0.597
Want to delay birth at least 2 years	0.295	0.043	202	474	1.331	0.146	0.209	0.380
Had paid sex in past 12 months	0.025	0.008	300	663	0.920	0.334	0.008	0.041
Had HIV test and received results in past 12 months	0.130	0.019	300	663	0.984	0.147	0.092	0.168
Accepting attitudes towards people with HIV	0.322	0.027	296	649	1.007	0.085	0.267	0.376

Table B.7 Sampling errors: Kampong Chhnang sample, Cambodia 2014

Variable	R	SE	N	WN	DEFT	SE/R	R-2SE	R+2SE
				WOMEN				
Urban residence	0.087	0.011	899	662	1.139	0.123	0.066	0.109
No schooling	0.087	0.017	899	662	1.756	0.190	0.054	0.120
Secondary or higher education	0.415	0.036	899	662	2.200	0.087	0.343	0.488
Never married (never in union)	0.317	0.020	899	662	1.281	0.063	0.277	0.357
Currently married (in union)	0.598	0.017	899	662	1.050	0.029	0.563	0.632
Married before age 20	0.425	0.030	583	423	1.485	0.072	0.364	0.486
Had first sexual intercourse before age 18	0.188	0.024	583	423	1.462	0.126	0.140	0.235
Currently pregnant	0.054	0.008	899	662	1.069	0.150	0.038	0.070
Children ever born	1.893	0.104	899	662	1.472	0.055	1.685	2.100
Children surviving	1.715	0.097	899	662	1.543	0.057	1.521	1.909
Children ever born to women age 40-49	4.158	0.221	207	157	1.369	0.053	3.715	4.600
Know any contraceptive method	1.000	0.000	532	396	na	0.000	1.000	1.000
Know any modern contraceptive method	1.000	0.000	532	396	na	0.000	1.000	1.000
Currently using any method	0.560	0.023	532	396	1.083	0.042	0.514	0.607
Currently using a modern method	0.335	0.028	532	396	1.346	0.082	0.280	0.390
Currently using daily pill	0.147	0.023	532	396	1.515	0.159	0.100	0.193
Currently using male condoms	0.017	0.005	532	396	0.972	0.319	0.006	0.028
Currently using injectables	0.077	0.012	532	396	1.067	0.160	0.052	0.102
Used public sector source	0.659	0.051	175	133	1.412	0.077	0.558	0.761
Want no more children	0.528	0.021	532	396	0.966	0.040	0.486	0.569
Want to delay next birth at least 2 years	0.337	0.018	532	396	0.887	0.054	0.301	0.374
Mothers protected against tetanus for last birth	0.979	0.012	271	203	1.382	0.012	0.955	1.003
Mothers received medical assistance at delivery	0.976	0.010	329	248	1.216	0.010	0.955	0.996
Had diarrhea in the last 2 weeks	0.106	0.021	313	236	1.171	0.196	0.064	0.147
Treated with ORS packets or pre-packed liquid	0.772	0.063	33	25	0.862	0.082	0.645	0.898
Consulted medical personnel for diarrhea	0.847	0.051	33	25	0.821	0.061	0.744	0.950
Vaccination card seen	0.775	0.054	69	52	1.072	0.069	0.668	0.882
Received BCG vaccination	1.000	0.000	69	52	na	0.000	1.000	1.000
Received DPT vaccination (3 doses)	0.863	0.066	69	52	1.603	0.077	0.731	0.995
Received polio vaccination (3 doses)	0.863	0.066	69	52	1.603	0.077	0.731	0.995
Received measles vaccination	0.746	0.063	69	52	1.215	0.085	0.619	0.873
Received all basic vaccinations	0.746	0.063	69	52	1.215	0.085	0.619	0.873
Height-for-age (-2SD)	0.428	0.037	231	173	1.092	0.087	0.353	0.502
Weight-for-height (-2SD)	0.112	0.019	231	173	0.919	0.166	0.075	0.150
Weight-for-age (-2SD)	0.356	0.033	231	173	1.043	0.092	0.290	0.422
Prevalence of anemia (children 6-59 months)	0.592	0.030	216	161	0.908	0.050	0.532	0.652
Prevalence of anemia (women 15-49)	0.530	0.027	568	418	1.308	0.052	0.475	0.584
Body mass index (BMI) <18.5	0.180	0.014	529	389	0.866	0.080	0.151	0.209
Had an HIV test and received results in past 12 months	0.135	0.011	899	662	0.926	0.078	0.114	0.156
Accepting attitudes towards people with HIV	0.267	0.025	899	662	1.715	0.095	0.217	0.318
Total fertility rate (last 3 years)	2.434	0.197	2,521	1,859	1.048	0.081	2.040	2.828
Neonatal mortality (last 0-9 years)	26.548	6.453	704	531	1.092	0.243	13.641	39.454
Postneonatal mortality (last 0-9 years)	23.102	5.897	702	529	0.997	0.255	11.309	34.896
Infant mortality (last 0-9 years)	49.650	8.348	704	531	1.039	0.168	32.954	66.345
Child mortality (last 0-9 years)	6.014	3.781	695	523	1.292	0.629	0.000	13.575
Under-5 mortality (last 0-9 years)	55.365	8.883	705	532	1.076	0.160	37.599	73.130
				MEN				
Urban residence	0.073	0.012	251	182	0.709	0.159	0.050	0.096
No education	0.046	0.016	251	182	1.194	0.345	0.014	0.078
Secondary or higher education	0.452	0.056	251	182	1.756	0.123	0.341	0.563
Never married (in union)	0.334	0.026	251	182	0.864	0.077	0.282	0.385
Currently married (in union)	0.632	0.030	251	182	0.995	0.048	0.572	0.693
Had first sexual intercourse before age 18	0.140	0.033	160	116	1.211	0.238	0.074	0.207
Want no more children	0.462	0.040	153	115	0.990	0.087	0.382	0.542
Want to delay birth at least 2 years	0.382	0.040	153	115	1.018	0.105	0.302	0.463
Had paid sex in past 12 months	0.042	0.014	251	182	1.110	0.336	0.014	0.070
Had HIV test and received results in past 12 months	0.121	0.023	251	182	1.124	0.192	0.075	0.167
Accepting attitudes towards people with HIV	0.459	0.033	251	182	1.042	0.072	0.393	0.525

Table B.8 Sampling errors: Kampong Speu sample, Cambodia 2014

Variable	R	SE	N	WN	DEFT	SE/R	R-2SE	R+2SE
				WOMEN				
Urban residence	0.078	0.007	1,022	1,196	0.782	0.084	0.065	0.091
No schooling	0.110	0.024	1,022	1,196	2.461	0.220	0.062	0.158
Secondary or higher education	0.385	0.032	1,022	1,196	2.067	0.082	0.322	0.448
Never married (never in union)	0.219	0.014	1,022	1,196	1.074	0.063	0.191	0.247
Currently married (in union)	0.705	0.017	1,022	1,196	1.177	0.024	0.671	0.738
Married before age 20	0.519	0.032	672	783	1.659	0.062	0.455	0.583
Had first sexual intercourse before age 18	0.296	0.027	672	783	1.549	0.092	0.242	0.351
Currently pregnant	0.063	0.010	1,022	1,196	1.346	0.162	0.043	0.084
Children ever born	1.989	0.090	1,022	1,196	1.461	0.045	1.808	2.170
Children surviving	1.836	0.083	1,022	1,196	1.491	0.045	1.670	2.002
Children ever born to women age 40-49	4.091	0.228	216	254	1.516	0.056	3.636	4.547
Know any contraceptive method	0.997	0.002	707	843	0.990	0.002	0.993	1.001
Know any modern contraceptive method	0.997	0.002	707	843	0.990	0.002	0.993	1.001
Currently using any method	0.654	0.022	707	843	1.223	0.033	0.610	0.698
Currently using a modern method	0.412	0.023	707	843	1.242	0.056	0.366	0.458
Currently using daily pill	0.252	0.024	707	843	1.482	0.096	0.203	0.300
Currently using male condoms	0.023	0.005	707	843	0.937	0.231	0.012	0.033
Currently using injectables	0.052	0.011	707	843	1.368	0.220	0.029	0.075
Used public sector source	0.326	0.046	277	347	1.617	0.140	0.234	0.417
Want no more children	0.518	0.021	707	843	1.142	0.042	0.475	0.561
Want to delay next birth at least 2 years	0.300	0.023	707	843	1.325	0.076	0.254	0.346
Mothers protected against tetanus for last birth	0.905	0.018	336	395	1.105	0.020	0.869	0.941
Mothers received medical assistance at delivery	0.893	0.036	396	469	2.071	0.041	0.820	0.966
Had diarrhea in the last 2 weeks	0.105	0.017	387	457	1.089	0.163	0.071	0.139
Treated with ORS packets or pre-packed liquid	0.236	0.102	42	48	1.385	0.432	0.032	0.439
Consulted medical personnel for diarrhea	0.633	0.076	42	48	0.943	0.120	0.482	0.784
Vaccination card seen	0.879	0.042	76	90	1.105	0.047	0.796	0.963
Received BCG vaccination	0.971	0.020	76	90	1.047	0.021	0.931	1.012
Received DPT vaccination (3 doses)	0.782	0.062	76	90	1.309	0.080	0.657	0.907
Received polio vaccination (3 doses)	0.782	0.062	76	90	1.309	0.080	0.657	0.907
Received measles vaccination	0.665	0.068	76	90	1.253	0.103	0.528	0.802
Received all basic vaccinations	0.665	0.068	76	90	1.253	0.103	0.528	0.802
Height-for-age (-2SD)	0.405	0.050	275	318	1.631	0.124	0.304	0.505
Weight-for-height (-2SD)	0.115	0.014	275	318	0.745	0.122	0.087	0.143
Weight-for-age (-2SD)	0.294	0.023	275	318	0.817	0.079	0.248	0.341
Prevalence of anemia (children 6-59 months)	0.639	0.041	256	301	1.315	0.064	0.558	0.721
Prevalence of anemia (women 15-49)	0.533	0.024	679	784	1.261	0.046	0.484	0.581
Body mass index (BMI) <18.5	0.209	0.016	629	737	0.979	0.076	0.177	0.241
Had an HIV test and received results in past 12 months	0.083	0.012	1,022	1,196	1.425	0.149	0.058	0.107
Accepting attitudes towards people with HIV	0.325	0.021	1,021	1,194	1.417	0.064	0.283	0.366
Total fertility rate (last 3 years)	2.421	0.187	2,910	3,393	1.278	0.077	2.047	2.795
Neonatal mortality (last 0-9 years)	19.427	5.216	833	994	1.040	0.268	8.995	29.859
Postneonatal mortality (last 0-9 years)	6.729	3.275	830	989	1.172	0.487	0.179	13.280
Infant mortality (last 0-9 years)	26.156	5.961	833	994	1.044	0.228	14.234	38.079
Child mortality (last 0-9 years)	4.549	2.591	837	1,005	1.160	0.570	0.000	9.730
Under-5 mortality (last 0-9 years)	30.586	6.940	834	995	1.135	0.227	16.705	44.467
				MEN				
Urban residence	0.064	0.006	269	323	0.422	0.098	0.052	0.077
No education	0.067	0.018	269	323	1.172	0.268	0.031	0.102
Secondary or higher education	0.560	0.035	269	323	1.165	0.063	0.489	0.631
Never married (in union)	0.287	0.034	269	323	1.222	0.118	0.219	0.354
Currently married (in union)	0.684	0.037	269	323	1.318	0.055	0.609	0.759
Had first sexual intercourse before age 18	0.083	0.022	180	209	1.063	0.264	0.039	0.127
Want no more children	0.593	0.029	183	221	0.802	0.049	0.534	0.651
Want to delay birth at least 2 years	0.296	0.031	183	221	0.916	0.105	0.234	0.358
Had paid sex in past 12 months	0.012	0.007	269	323	0.988	0.537	0.000	0.026
Had HIV test and received results in past 12 months	0.068	0.025	269	323	1.645	0.372	0.017	0.119
Accepting attitudes towards people with HIV	0.359	0.034	268	322	1.159	0.095	0.291	0.427

Table B.9 Sampling errors: Kampong Thom sample, Cambodia 2014

Variable	R	SE	N	WN	DEFT	SE/R	R-2SE	R+2SE
			WOMEN					
Urban residence	0.052	0.004	905	851	0.573	0.081	0.044	0.061
No schooling	0.140	0.019	905	851	1.659	0.137	0.101	0.178
Secondary or higher education	0.303	0.035	905	851	2.271	0.115	0.233	0.373
Never married (never in union)	0.248	0.013	905	851	0.907	0.053	0.222	0.274
Currently married (in union)	0.672	0.016	905	851	1.044	0.024	0.639	0.705
Married before age 20	0.484	0.024	620	580	1.200	0.050	0.436	0.533
Had first sexual intercourse before age 18	0.249	0.020	620	580	1.137	0.079	0.210	0.289
Currently pregnant	0.058	0.007	905	851	0.920	0.123	0.044	0.073
Children ever born	2.177	0.108	905	851	1.497	0.050	1.960	2.394
Children surviving	2.015	0.091	905	851	1.384	0.045	1.833	2.197
Children ever born to women age 40-49	4.417	0.221	224	209	1.418	0.050	3.974	4.860
Know any contraceptive method	1.000	0.000	597	572	na	0.000	1.000	1.000
Know any modern contraceptive method	1.000	0.000	597	572	na	0.000	1.000	1.000
Currently using any method	0.584	0.030	597	572	1.496	0.052	0.524	0.644
Currently using a modern method	0.441	0.026	597	572	1.270	0.059	0.389	0.492
Currently using daily pill	0.155	0.020	597	572	1.376	0.131	0.115	0.196
Currently using male condoms	0.007	0.003	597	572	0.944	0.451	0.001	0.014
Currently using injectables	0.149	0.022	597	572	1.473	0.144	0.106	0.192
Used public sector source	0.533	0.043	264	256	1.409	0.081	0.446	0.620
Want no more children	0.591	0.016	597	572	0.786	0.027	0.560	0.623
Want to delay next birth at least 2 years	0.265	0.016	597	572	0.913	0.062	0.232	0.298
Mothers protected against tetanus for last birth	0.927	0.025	288	279	1.647	0.027	0.876	0.977
Mothers received medical assistance at delivery	0.804	0.051	347	337	2.202	0.063	0.703	0.905
Had diarrhea in the last 2 weeks	0.061	0.014	339	327	1.027	0.233	0.033	0.089
Treated with ORS packets or pre-packed liquid	0.190	0.097	22	20	1.105	0.509	0.000	0.383
Consulted medical personnel for diarrhea	0.803	0.107	22	20	1.206	0.133	0.589	1.017
Vaccination card seen	0.863	0.068	80	77	1.766	0.078	0.728	0.999
Received BCG vaccination	0.976	0.016	80	77	0.942	0.017	0.943	1.008
Received DPT vaccination (3 doses)	0.820	0.034	80	77	0.786	0.041	0.753	0.888
Received polio vaccination (3 doses)	0.820	0.034	80	77	0.786	0.041	0.753	0.888
Received measles vaccination	0.741	0.039	80	77	0.803	0.053	0.663	0.819
Received all basic vaccinations	0.709	0.038	80	77	0.755	0.054	0.633	0.786
Height-for-age (-2SD)	0.364	0.041	228	217	1.237	0.113	0.281	0.446
Weight-for-height (-2SD)	0.130	0.026	228	217	1.124	0.200	0.078	0.183
Weight-for-age (-2SD)	0.277	0.032	228	217	1.040	0.115	0.213	0.340
Prevalence of anemia (children 6-59 months)	0.660	0.042	207	197	1.259	0.064	0.576	0.744
Prevalence of anemia (women 15-49)	0.446	0.019	592	567	0.941	0.043	0.408	0.484
Body mass index (BMI) <18.5	0.125	0.019	554	526	1.337	0.150	0.087	0.162
Had an HIV test and received results in past 12 months	0.089	0.012	905	851	1.275	0.136	0.065	0.113
Accepting attitudes towards people with HIV	0.201	0.028	905	851	2.094	0.139	0.145	0.257
Total fertility rate (last 3 years)	2.935	0.195	2,513	2,353	1.113	0.066	2.545	3.325
Neonatal mortality (last 0-9 years)	29.195	6.656	782	759	1.117	0.228	15.882	42.508
Postneonatal mortality (last 0-9 years)	11.466	4.666	782	760	1.157	0.407	2.134	20.798
Infant mortality (last 0-9 years)	40.661	9.345	782	759	1.317	0.230	21.972	59.350
Child mortality (last 0-9 years)	20.219	8.113	794	770	1.642	0.401	3.993	36.445
Under-5 mortality (last 0-9 years)	60.058	14.874	785	763	1.667	0.248	30.310	89.806
			MEN					
Urban residence	0.055	0.009	261	232	0.612	0.157	0.037	0.072
No education	0.087	0.018	261	232	1.005	0.202	0.052	0.122
Secondary or higher education	0.386	0.056	261	232	1.839	0.144	0.274	0.498
Never married (in union)	0.339	0.026	261	232	0.883	0.076	0.287	0.391
Currently married (in union)	0.629	0.028	261	232	0.929	0.044	0.574	0.685
Had first sexual intercourse before age 18	0.079	0.020	165	146	0.929	0.248	0.040	0.118
Want no more children	0.534	0.044	164	146	1.131	0.083	0.445	0.622
Want to delay birth at least 2 years	0.358	0.044	164	146	1.172	0.123	0.270	0.446
Had paid sex in past 12 months	0.000	0.000	261	232	na	na	0.000	0.000
Had HIV test and received results in past 12 months	0.018	0.007	261	232	0.886	0.401	0.004	0.033
Accepting attitudes towards people with HIV	0.595	0.057	261	232	1.872	0.096	0.481	0.710

Table B.10 Sampling errors: Kandal sample, Cambodia 2014								
Variable	R	SE	N	WN	DEFT	SE/R	R-2SE	R+2SE
				WOMEN				
Urban residence	0.103	0.017	875	1,330	1.688	0.169	0.068	0.138
No schooling	0.054	0.015	875	1,330	1.909	0.271	0.025	0.083
Secondary or higher education	0.442	0.025	875	1,330	1.516	0.058	0.391	0.493
Never married (never in union)	0.262	0.014	875	1,330	0.923	0.052	0.234	0.289
Currently married (in union)	0.654	0.017	875	1,330	1.087	0.027	0.619	0.689
Married before age 20	0.347	0.028	589	904	1.449	0.082	0.290	0.404
Had first sexual intercourse before age 18	0.172	0.023	589	904	1.489	0.135	0.125	0.218
Currently pregnant	0.057	0.008	875	1,330	1.034	0.142	0.041	0.073
Children ever born	1.789	0.087	875	1,330	1.356	0.048	1.616	1.962
Children surviving	1.647	0.069	875	1,330	1.201	0.042	1.510	1.784
Children ever born to women age 40-49	3.885	0.205	178	259	1.273	0.053	3.475	4.294
Know any contraceptive method	1.000	0.000	544	870	na	0.000	1.000	1.000
Know any modern contraceptive method	1.000	0.000	544	870	na	0.000	1.000	1.000
Currently using any method	0.610	0.027	544	870	1.312	0.045	0.555	0.665
Currently using a modern method	0.404	0.031	544	870	1.454	0.076	0.343	0.465
Currently using daily pill	0.195	0.017	544	870	0.998	0.087	0.161	0.229
Currently using male condoms	0.016	0.005	544	870	0.995	0.333	0.005	0.027
Currently using injectables	0.122	0.024	544	870	1.669	0.192	0.075	0.169
Used public sector source	0.424	0.037	208	353	1.070	0.087	0.350	0.497
Want no more children	0.514	0.039	544	870	1.800	0.075	0.437	0.592
Want to delay next birth at least 2 years	0.280	0.026	544	870	1.339	0.092	0.228	0.332
Mothers protected against tetanus for last birth	0.883	0.037	256	420	1.851	0.042	0.809	0.956
Mothers received medical assistance at delivery	0.957	0.021	318	523	1.487	0.022	0.914	1.000
Had diarrhea in the last 2 weeks	0.090	0.024	310	506	1.424	0.271	0.041	0.139
Treated with ORS packets or pre-packed liquid	0.145	0.100	23	46	1.244	0.691	0.000	0.346
Consulted medical personnel for diarrhea	0.486	0.124	23	46	1.226	0.255	0.238	0.734
Vaccination card seen	0.809	0.077	57	89	1.471	0.096	0.654	0.964
Received BCG vaccination	1.000	0.000	57	89	na	0.000	1.000	1.000
Received DPT vaccination (3 doses)	0.815	0.068	57	89	1.315	0.084	0.678	0.952
Received polio vaccination (3 doses)	0.741	0.076	57	89	1.300	0.103	0.588	0.893
Received measles vaccination	0.752	0.071	57	89	1.232	0.095	0.609	0.894
Received all basic vaccinations	0.645	0.070	57	89	1.093	0.108	0.505	0.785
Height-for-age (-2SD)	0.281	0.035	206	298	1.026	0.125	0.211	0.350
Weight-for-height (-2SD)	0.092	0.026	206	298	1.299	0.285	0.040	0.145
Weight-for-age (-2SD)	0.262	0.040	206	298	1.275	0.151	0.183	0.341
Prevalence of anemia (children 6-59 months)	0.586	0.044	188	267	1.193	0.075	0.498	0.674
Prevalence of anemia (women 15-49)	0.494	0.029	579	867	1.405	0.060	0.435	0.553
Body mass index (BMI) <18.5	0.179	0.019	536	807	1.115	0.104	0.142	0.216
Had an HIV test and received results in past 12 months	0.077	0.010	875	1,330	1.113	0.131	0.057	0.097
Accepting attitudes towards people with HIV	0.359	0.024	869	1,317	1.472	0.067	0.311	0.407
Total fertility rate (last 3 years)	2.540	0.163	2,483	3,758	0.979	0.064	2.214	2.866
Neonatal mortality (last 0-9 years)	16.610	6.183	676	1,135	1.322	0.372	4.244	28.976
Postneonatal mortality (last 0-9 years)	13.595	4.343	676	1,133	1.014	0.319	4.909	22.282
Infant mortality (last 0-9 years)	30.206	8.511	676	1,135	1.365	0.282	13.183	47.228
Child mortality (last 0-9 years)	10.094	5.117	669	1,116	1.319	0.507	0.000	20.329
Under-5 mortality (last 0-9 years)	39.995	11.181	677	1,137	1.566	0.280	17.633	62.357
				MEN				
Urban residence	0.088	0.020	239	413	1.108	0.232	0.047	0.128
No education	0.021	0.013	239	413	1.367	0.610	0.000	0.046
Secondary or higher education	0.515	0.048	239	413	1.467	0.093	0.419	0.610
Never married (in union)	0.282	0.028	239	413	0.967	0.100	0.226	0.339
Currently married (in union)	0.697	0.028	239	413	0.948	0.041	0.640	0.753
Had first sexual intercourse before age 18	0.088	0.017	164	293	0.755	0.190	0.055	0.121
Want no more children	0.532	0.044	154	288	1.097	0.083	0.444	0.621
Want to delay birth at least 2 years	0.316	0.042	154	288	1.129	0.135	0.231	0.400
Had paid sex in past 12 months	0.013	0.007	239	413	0.980	0.549	0.000	0.028
Had HIV test and received results in past 12 months	0.123	0.034	239	413	1.590	0.276	0.055	0.191
Accepting attitudes towards people with HIV	0.319	0.040	232	400	1.310	0.126	0.239	0.400

Table B.11 Sampling errors: Kratie sample, Cambodia 2014

Variable	R	SE	N	WN	DEFT	SE/R	R-2SE	R+2SE
				WOMEN				
Urban residence	0.105	0.017	874	488	1.611	0.159	0.072	0.139
No schooling	0.156	0.044	874	488	3.527	0.279	0.069	0.243
Secondary or higher education	0.266	0.037	874	488	2.448	0.138	0.192	0.339
Never married (never in union)	0.218	0.016	874	488	1.124	0.072	0.187	0.250
Currently married (in union)	0.735	0.013	874	488	0.893	0.018	0.708	0.761
Married before age 20	0.513	0.026	582	313	1.249	0.050	0.462	0.565
Had first sexual intercourse before age 18	0.269	0.020	582	313	1.113	0.076	0.228	0.310
Currently pregnant	0.073	0.013	874	488	1.473	0.178	0.047	0.099
Children ever born	2.291	0.121	874	488	1.562	0.053	2.049	2.533
Children surviving	2.042	0.101	874	488	1.516	0.050	1.840	2.245
Children ever born to women age 40-49	4.460	0.313	181	104	1.579	0.070	3.833	5.087
Know any contraceptive method	0.998	0.002	631	359	0.932	0.002	0.994	1.001
Know any modern contraceptive method	0.998	0.002	631	359	0.932	0.002	0.994	1.001
Currently using any method	0.479	0.031	631	359	1.549	0.064	0.417	0.541
Currently using a modern method	0.307	0.026	631	359	1.421	0.085	0.254	0.359
Currently using daily pill	0.116	0.017	631	359	1.346	0.148	0.081	0.150
Currently using male condoms	0.010	0.004	631	359	0.918	0.359	0.003	0.018
Currently using injectables	0.088	0.013	631	359	1.181	0.152	0.061	0.115
Used public sector source	0.392	0.059	207	110	1.721	0.150	0.274	0.509
Want no more children	0.508	0.024	631	359	1.217	0.048	0.459	0.556
Want to delay next birth at least 2 years	0.297	0.027	631	359	1.493	0.092	0.242	0.351
Mothers protected against tetanus for last birth	0.836	0.041	382	214	2.164	0.049	0.754	0.918
Mothers received medical assistance at delivery	0.519	0.080	471	269	3.072	0.154	0.359	0.679
Had diarrhea in the last 2 weeks	0.170	0.020	449	254	1.104	0.119	0.129	0.210
Treated with ORS packets or pre-packed liquid	0.202	0.067	72	43	1.385	0.331	0.068	0.335
Consulted medical personnel for diarrhea	0.598	0.096	72	43	1.625	0.160	0.407	0.790
Vaccination card seen	0.721	0.063	97	57	1.430	0.088	0.594	0.848
Received BCG vaccination	0.865	0.039	97	57	1.154	0.045	0.787	0.943
Received DPT vaccination (3 doses)	0.728	0.067	97	57	1.522	0.092	0.594	0.862
Received polio vaccination (3 doses)	0.725	0.067	97	57	1.517	0.092	0.591	0.859
Received measles vaccination	0.797	0.070	97	57	1.764	0.088	0.656	0.938
Received all basic vaccinations	0.651	0.074	97	57	1.568	0.114	0.503	0.799
Height-for-age (-2SD)	0.384	0.033	319	180	1.161	0.087	0.318	0.451
Weight-for-height (-2SD)	0.065	0.021	319	180	1.378	0.317	0.024	0.106
Weight-for-age (-2SD)	0.251	0.034	319	180	1.351	0.135	0.183	0.319
Prevalence of anemia (children 6-59 months)	0.502	0.035	285	157	1.167	0.069	0.433	0.571
Prevalence of anemia (women 15-49)	0.462	0.026	568	318	1.247	0.056	0.410	0.514
Body mass index (BMI) <18.5	0.158	0.028	516	287	1.726	0.176	0.102	0.213
Had an HIV test and received results in past 12 months	0.035	0.007	874	488	1.187	0.210	0.020	0.050
Accepting attitudes towards people with HIV	0.274	0.029	868	485	1.928	0.107	0.215	0.332
Total fertility rate (last 3 years)	3.609	0.201	2,511	1,399	0.966	0.056	3.206	4.011
Neonatal mortality (last 0-9 years)	29.670	7.431	918	541	1.318	0.250	14.808	44.533
Postneonatal mortality (last 0-9 years)	31.147	9.878	915	537	1.496	0.317	11.391	50.903
Infant mortality (last 0-9 years)	60.817	16.325	918	541	1.894	0.268	28.167	93.468
Child mortality (last 0-9 years)	20.297	11.151	908	532	2.166	0.549	0.000	42.599
Under-5 mortality (last 0-9 years)	79.880	23.546	921	542	2.308	0.295	32.788	126.972
				MEN				
Urban residence	0.097	0.021	258	143	1.159	0.220	0.054	0.140
No education	0.036	0.025	258	143	2.150	0.698	0.000	0.087
Secondary or higher education	0.461	0.045	258	143	1.442	0.097	0.371	0.551
Never married (in union)	0.294	0.036	258	143	1.267	0.122	0.222	0.367
Currently married (in union)	0.666	0.036	258	143	1.213	0.054	0.595	0.738
Had first sexual intercourse before age 18	0.010	0.008	170	90	0.973	0.729	0.000	0.026
Want no more children	0.538	0.054	170	95	1.411	0.101	0.429	0.646
Want to delay birth at least 2 years	0.246	0.032	170	95	0.976	0.131	0.182	0.311
Had paid sex in past 12 months	0.153	0.020	258	143	0.883	0.130	0.113	0.192
Had HIV test and received results in past 12 months	0.064	0.017	258	143	1.100	0.262	0.031	0.098
Accepting attitudes towards people with HIV	0.105	0.023	252	139	1.203	0.221	0.059	0.152

Table B.12 Sampling errors: Phnom Penh sample, Cambodia 2014

Variable	R	SE	N	WN	DEFT	SE/R	R-2SE	R+2SE
				WOMEN				
Urban residence	0.825	0.026	1,400	1,994	2.544	0.031	0.773	0.877
No schooling	0.041	0.007	1,400	1,994	1.417	0.184	0.026	0.056
Secondary or higher education	0.669	0.022	1,400	1,994	1.743	0.033	0.625	0.713
Never married (never in union)	0.386	0.015	1,400	1,994	1.132	0.038	0.356	0.415
Currently married (in union)	0.544	0.016	1,400	1,994	1.233	0.030	0.511	0.577
Married before age 20	0.340	0.018	874	1,250	1.094	0.052	0.305	0.375
Had first sexual intercourse before age 18	0.172	0.011	874	1,250	0.898	0.067	0.149	0.195
Currently pregnant	0.046	0.005	1,400	1,994	0.975	0.119	0.035	0.057
Children ever born	1.263	0.049	1,400	1,994	1.207	0.039	1.165	1.360
Children surviving	1.208	0.044	1,400	1,994	1.146	0.036	1.121	1.296
Children ever born to women age 40-49	2.783	0.135	262	372	1.250	0.049	2.512	3.054
Know any contraceptive method	1.000	0.000	753	1,084	na	0.000	1.000	1.000
Know any modern contraceptive method	1.000	0.000	753	1,084	na	0.000	1.000	1.000
Currently using any method	0.633	0.018	753	1,084	1.038	0.029	0.596	0.669
Currently using a modern method	0.287	0.013	753	1,084	0.812	0.047	0.261	0.314
Currently using daily pill	0.118	0.015	753	1,084	1.277	0.127	0.088	0.148
Currently using male condoms	0.056	0.010	753	1,084	1.154	0.172	0.037	0.076
Currently using injectables	0.015	0.006	753	1,084	1.368	0.399	0.003	0.028
Used public sector source	0.134	0.022	225	326	0.961	0.163	0.091	0.178
Want no more children	0.498	0.021	753	1,084	1.148	0.042	0.456	0.540
Want to delay next birth at least 2 years	0.262	0.018	753	1,084	1.099	0.067	0.227	0.297
Mothers protected against tetanus for last birth	0.943	0.013	370	535	1.073	0.014	0.917	0.969
Mothers received medical assistance at delivery	0.961	0.014	435	626	1.314	0.014	0.934	0.989
Had diarrhea in the last 2 weeks	0.172	0.017	430	618	0.943	0.101	0.138	0.207
Treated with ORS packets or pre-packed liquid	0.335	0.065	74	107	1.194	0.194	0.205	0.465
Consulted medical personnel for diarrhea	0.575	0.059	74	107	1.012	0.102	0.458	0.692
Vaccination card seen	0.696	0.046	103	145	1.003	0.066	0.605	0.788
Received BCG vaccination	0.986	0.013	103	145	1.142	0.013	0.960	1.013
Received DPT vaccination (3 doses)	0.931	0.028	103	145	1.112	0.030	0.874	0.987
Received polio vaccination (3 doses)	0.900	0.033	103	145	1.117	0.037	0.833	0.967
Received measles vaccination	0.910	0.034	103	145	1.199	0.038	0.842	0.979
Received all basic vaccinations	0.891	0.034	103	145	1.105	0.038	0.823	0.960
Height-for-age (-2SD)	0.179	0.025	284	391	1.125	0.140	0.129	0.229
Weight-for-height (-2SD)	0.084	0.013	284	391	0.802	0.155	0.058	0.110
Weight-for-age (-2SD)	0.129	0.022	284	391	1.115	0.173	0.084	0.173
Prevalence of anemia (children 6-59 months)	0.410	0.033	238	335	1.044	0.080	0.345	0.476
Prevalence of anemia (women 15-49)	0.417	0.016	913	1,316	1.006	0.039	0.384	0.449
Body mass index (BMI) <18.5	0.140	0.013	887	1,269	1.114	0.093	0.114	0.165
Had an HIV test and received results in past 12 months	0.136	0.010	1,400	1,994	1.038	0.070	0.117	0.155
Accepting attitudes towards people with HIV	0.267	0.014	1,398	1,992	1.199	0.053	0.238	0.295
Total fertility rate (last 3 years)	1.975	0.115	4,027	5,731	0.920	0.058	1.745	2.206
Neonatal mortality (last 0-9 years)	13.075	6.375	818	1,172	1.440	0.488	0.326	25.824
Postneonatal mortality (last 0-9 years)	3.934	3.948	811	1,160	1.380	1.004	0.000	11.831
Infant mortality (last 0-9 years)	17.009	7.775	818	1,172	1.193	0.457	1.459	32.560
Child mortality (last 0-9 years)	6.250	2.837	796	1,139	1.056	0.454	0.577	11.924
Under-5 mortality (last 0-9 years)	23.154	7.742	818	1,172	1.045	0.334	7.669	38.638
				MEN				
Urban residence	0.815	0.026	391	550	1.336	0.032	0.762	0.867
No education	0.003	0.003	391	550	1.100	0.999	0.000	0.009
Secondary or higher education	0.848	0.027	391	550	1.464	0.031	0.794	0.901
Never married (in union)	0.479	0.023	391	550	0.904	0.048	0.433	0.525
Currently married (in union)	0.507	0.022	391	550	0.869	0.043	0.463	0.551
Had first sexual intercourse before age 18	0.041	0.015	232	333	1.126	0.360	0.011	0.070
Want no more children	0.478	0.037	191	279	1.023	0.077	0.404	0.553
Want to delay birth at least 2 years	0.316	0.038	191	279	1.119	0.119	0.241	0.392
Had paid sex in past 12 months	0.076	0.012	391	550	0.889	0.157	0.052	0.100
Had HIV test and received results in past 12 months	0.117	0.016	391	550	0.981	0.136	0.085	0.149
Accepting attitudes towards people with HIV	0.320	0.026	390	548	1.095	0.081	0.268	0.372

Table B.13 Sampling errors: Prey Veng sample, Cambodia 2014

Variable	R	SE	N	WN	DEFT	SE/R	R-2SE	R+2SE
				WOMEN				
Urban residence	0.032	0.004	819	1,188	0.658	0.127	0.024	0.040
No schooling	0.191	0.028	819	1,188	2.015	0.145	0.136	0.247
Secondary or higher education	0.306	0.029	819	1,188	1.810	0.095	0.247	0.364
Never married (never in union)	0.163	0.016	819	1,188	1.257	0.100	0.130	0.195
Currently married (in union)	0.749	0.018	819	1,188	1.184	0.024	0.713	0.784
Married before age 20	0.521	0.021	606	878	1.010	0.039	0.480	0.562
Had first sexual intercourse before age 18	0.332	0.019	606	878	0.991	0.057	0.294	0.370
Currently pregnant	0.049	0.007	819	1,188	0.901	0.139	0.035	0.062
Children ever born	2.114	0.082	819	1,188	1.306	0.039	1.949	2.279
Children surviving	1.879	0.067	819	1,188	1.234	0.036	1.746	2.013
Children ever born to women age 40-49	3.477	0.164	250	362	1.317	0.047	3.149	3.806
Know any contraceptive method	1.000	0.000	599	889	na	0.000	1.000	1.000
Know any modern contraceptive method	1.000	0.000	599	889	na	0.000	1.000	1.000
Currently using any method	0.554	0.026	599	889	1.262	0.046	0.502	0.605
Currently using a modern method	0.413	0.025	599	889	1.252	0.061	0.363	0.464
Currently using daily pill	0.194	0.022	599	889	1.363	0.114	0.150	0.238
Currently using male condoms	0.012	0.004	599	889	0.949	0.358	0.003	0.020
Currently using injectables	0.116	0.027	599	889	2.032	0.230	0.063	0.170
Used public sector source	0.480	0.060	246	368	1.878	0.125	0.360	0.601
Want no more children	0.545	0.025	599	889	1.214	0.045	0.496	0.595
Want to delay next birth at least 2 years	0.211	0.022	599	889	1.299	0.103	0.168	0.254
Mothers protected against tetanus for last birth	0.912	0.021	264	405	1.250	0.023	0.869	0.955
Mothers received medical assistance at delivery	0.976	0.011	326	499	1.160	0.012	0.954	0.999
Had diarrhea in the last 2 weeks	0.047	0.015	314	478	1.179	0.315	0.018	0.077
Treated with ORS packets or pre-packed liquid	0.215	0.109	16	23	1.013	0.506	0.000	0.432
Consulted medical personnel for diarrhea	0.852	0.076	16	23	0.831	0.090	0.700	1.005
Vaccination card seen	0.688	0.065	62	101	1.145	0.094	0.559	0.818
Received BCG vaccination	0.952	0.028	62	101	1.079	0.029	0.897	1.008
Received DPT vaccination (3 doses)	0.760	0.080	62	101	1.537	0.105	0.600	0.920
Received polio vaccination (3 doses)	0.760	0.080	62	101	1.537	0.105	0.600	0.920
Received measles vaccination	0.632	0.076	62	101	1.281	0.120	0.480	0.783
Received all basic vaccinations	0.617	0.079	62	101	1.333	0.129	0.458	0.775
Height-for-age (-2SD)	0.327	0.043	254	379	1.404	0.133	0.240	0.414
Weight-for-height (-2SD)	0.086	0.023	254	379	1.316	0.269	0.040	0.132
Weight-for-age (-2SD)	0.222	0.041	254	379	1.572	0.185	0.140	0.304
Prevalence of anemia (children 6-59 months)	0.513	0.045	230	345	1.429	0.088	0.422	0.603
Prevalence of anemia (women 15-49)	0.469	0.028	516	749	1.266	0.059	0.414	0.525
Body mass index (BMI) <18.5	0.125	0.019	488	708	1.290	0.155	0.086	0.164
Had an HIV test and received results in past 12 months	0.044	0.007	819	1,188	0.991	0.162	0.030	0.058
Accepting attitudes towards people with HIV	0.277	0.026	816	1,184	1.632	0.093	0.225	0.328
Total fertility rate (last 3 years)	2.960	0.176	2,353	3,407	1.109	0.060	2.607	3.313
Neonatal mortality (last 0-9 years)	33.412	8.731	637	947	1.160	0.261	15.951	50.873
Postneonatal mortality (last 0-9 years)	30.979	6.200	635	945	0.933	0.200	18.579	43.379
Infant mortality (last 0-9 years)	64.391	12.501	637	947	1.280	0.194	39.388	89.393
Child mortality (last 0-9 years)	11.348	5.219	615	918	1.187	0.460	0.910	21.786
Under-5 mortality (last 0-9 years)	75.008	13.786	639	951	1.338	0.184	47.436	102.580
				MEN				
Urban residence	0.037	0.005	244	342	0.372	0.121	0.028	0.046
No education	0.092	0.023	244	342	1.214	0.245	0.047	0.137
Secondary or higher education	0.565	0.042	244	342	1.327	0.075	0.480	0.649
Never married (in union)	0.242	0.033	244	342	1.194	0.136	0.176	0.308
Currently married (in union)	0.724	0.030	244	342	1.032	0.041	0.665	0.783
Had first sexual intercourse before age 18	0.161	0.032	180	257	1.148	0.196	0.098	0.224
Want no more children	0.526	0.047	174	248	1.242	0.090	0.432	0.621
Want to delay birth at least 2 years	0.294	0.043	174	248	1.248	0.147	0.207	0.381
Had paid sex in past 12 months	0.036	0.012	244	342	1.009	0.337	0.012	0.059
Had HIV test and received results in past 12 months	0.090	0.027	244	342	1.470	0.300	0.036	0.144
Accepting attitudes towards people with HIV	0.361	0.036	239	337	1.140	0.099	0.289	0.432

Table B.14 Sampling errors: Pursat sample, Cambodia 2014

Variable	R	SE	N	WN	DEFT	SE/R	R-2SE	R+2SE
				WOMEN				
Urban residence	0.049	0.006	859	631	0.822	0.123	0.037	0.061
No schooling	0.163	0.035	859	631	2.749	0.214	0.093	0.232
Secondary or higher education	0.322	0.050	859	631	3.126	0.156	0.222	0.423
Never married (never in union)	0.278	0.033	859	631	2.133	0.117	0.213	0.344
Currently married (in union)	0.673	0.034	859	631	2.120	0.051	0.605	0.741
Married before age 20	0.428	0.050	561	404	2.391	0.117	0.328	0.529
Had first sexual intercourse before age 18	0.210	0.045	561	404	2.588	0.213	0.121	0.300
Currently pregnant	0.059	0.013	859	631	1.648	0.224	0.033	0.086
Children ever born	1.954	0.111	859	631	1.553	0.057	1.731	2.176
Children surviving	1.815	0.103	859	631	1.602	0.057	1.609	2.021
Children ever born to women age 40-49	4.025	0.232	170	116	1.228	0.058	3.560	4.489
Know any contraceptive method	1.000	0.000	568	425	na	0.000	1.000	1.000
Know any modern contraceptive method	1.000	0.000	568	425	na	0.000	1.000	1.000
Currently using any method	0.510	0.032	568	425	1.542	0.063	0.446	0.575
Currently using a modern method	0.401	0.028	568	425	1.359	0.070	0.345	0.457
Currently using daily pill	0.188	0.031	568	425	1.911	0.167	0.125	0.251
Currently using male condoms	0.017	0.007	568	425	1.225	0.396	0.003	0.030
Currently using injectables	0.111	0.017	568	425	1.291	0.154	0.077	0.145
Used public sector source	0.477	0.047	217	173	1.393	0.100	0.382	0.572
Want no more children	0.507	0.023	568	425	1.113	0.046	0.460	0.553
Want to delay next birth at least 2 years	0.327	0.025	568	425	1.247	0.075	0.278	0.376
Mothers protected against tetanus for last birth	0.898	0.018	314	245	1.079	0.020	0.862	0.934
Mothers received medical assistance at delivery	0.861	0.035	388	298	1.830	0.041	0.790	0.931
Had diarrhea in the last 2 weeks	0.082	0.019	383	294	1.343	0.236	0.043	0.120
Treated with ORS packets or pre-packed liquid	0.497	0.166	27	24	1.803	0.334	0.165	0.830
Consulted medical personnel for diarrhea	0.310	0.114	27	24	1.372	0.367	0.082	0.538
Vaccination card seen	0.844	0.053	71	60	1.317	0.063	0.738	0.950
Received BCG vaccination	0.953	0.025	71	60	1.080	0.027	0.902	1.004
Received DPT vaccination (3 doses)	0.833	0.055	71	60	1.328	0.066	0.724	0.943
Received polio vaccination (3 doses)	0.845	0.055	71	60	1.373	0.065	0.735	0.955
Received measles vaccination	0.889	0.046	71	60	1.309	0.051	0.797	0.980
Received all basic vaccinations	0.797	0.057	71	60	1.272	0.071	0.683	0.911
Height-for-age (-2SD)	0.388	0.062	257	200	2.094	0.159	0.264	0.511
Weight-for-height (-2SD)	0.123	0.021	257	200	1.025	0.173	0.081	0.166
Weight-for-age (-2SD)	0.316	0.057	257	200	1.935	0.180	0.203	0.430
Prevalence of anemia (children 6-59 months)	0.648	0.042	243	192	1.405	0.065	0.563	0.732
Prevalence of anemia (women 15-49)	0.466	0.029	566	418	1.403	0.063	0.408	0.525
Body mass index (BMI) <18.5	0.131	0.023	524	384	1.583	0.179	0.084	0.178
Had an HIV test and received results in past 12 months	0.094	0.012	859	631	1.213	0.128	0.070	0.118
Accepting attitudes towards people with HIV	0.201	0.025	856	629	1.823	0.125	0.151	0.251
Total fertility rate (last 3 years)	3.063	0.219	2,448	1,804	1.468	0.072	2.624	3.502
Neonatal mortality (last 0-9 years)	14.099	4.878	783	605	1.153	0.346	4.343	23.856
Postneonatal mortality (last 0-9 years)	16.562	5.229	776	602	1.281	0.316	6.105	27.019
Infant mortality (last 0-9 years)	30.662	8.837	784	608	1.540	0.288	12.989	48.335
Child mortality (last 0-9 years)	5.031	4.360	771	616	1.360	0.867	0.000	13.751
Under-5 mortality (last 0-9 years)	35.538	9.303	786	610	1.487	0.262	16.931	54.145
				MEN				
Urban residence	0.048	0.008	261	184	0.608	0.169	0.031	0.064
No education	0.080	0.025	261	184	1.461	0.309	0.030	0.129
Secondary or higher education	0.382	0.045	261	184	1.502	0.119	0.291	0.473
Never married (in union)	0.390	0.050	261	184	1.647	0.128	0.290	0.490
Currently married (in union)	0.590	0.049	261	184	1.600	0.083	0.492	0.688
Had first sexual intercourse before age 18	0.089	0.027	172	113	1.252	0.308	0.034	0.143
Want no more children	0.430	0.037	167	109	0.973	0.087	0.355	0.505
Want to delay birth at least 2 years	0.316	0.035	167	109	0.957	0.109	0.247	0.385
Had paid sex in past 12 months	0.022	0.009	261	184	1.036	0.427	0.003	0.041
Had HIV test and received results in past 12 months	0.056	0.017	261	184	1.224	0.312	0.021	0.091
Accepting attitudes towards people with HIV	0.388	0.061	260	183	2.012	0.158	0.265	0.510

Table B.15 Sampling errors: Siem Reap sample, Cambodia 2014

Variable	R	SE	N	WN	DEFT	SE/R	R-2SE	R+2SE
		WOMEN						
Urban residence	0.179	0.022	943	1,137	1.725	0.120	0.136	0.222
No schooling	0.254	0.033	943	1,137	2.322	0.130	0.188	0.321
Secondary or higher education	0.293	0.028	943	1,137	1.915	0.097	0.236	0.350
Never married (never in union)	0.252	0.015	943	1,137	1.086	0.061	0.221	0.283
Currently married (in union)	0.672	0.018	943	1,137	1.169	0.027	0.637	0.708
Married before age 20	0.456	0.031	607	746	1.554	0.069	0.393	0.519
Had first sexual intercourse before age 18	0.223	0.026	607	746	1.549	0.118	0.170	0.275
Currently pregnant	0.052	0.008	943	1,137	1.073	0.149	0.036	0.067
Children ever born	2.096	0.093	943	1,137	1.395	0.044	1.910	2.282
Children surviving	1.902	0.083	943	1,137	1.398	0.044	1.736	2.067
Children ever born to women age 40-49	3.889	0.168	199	259	1.108	0.043	3.552	4.225
Know any contraceptive method	1.000	0.000	604	765	na	0.000	1.000	1.000
Know any modern contraceptive method	1.000	0.000	604	765	na	0.000	1.000	1.000
Currently using any method	0.590	0.025	604	765	1.258	0.043	0.540	0.641
Currently using a modern method	0.465	0.023	604	765	1.131	0.049	0.419	0.511
Currently using daily pill	0.195	0.033	604	765	2.023	0.168	0.130	0.261
Currently using male condoms	0.038	0.008	604	765	0.993	0.204	0.022	0.053
Currently using injectables	0.102	0.019	604	765	1.530	0.185	0.064	0.139
Used public sector source	0.623	0.050	292	367	1.769	0.081	0.522	0.724
Want no more children	0.550	0.031	604	765	1.525	0.056	0.488	0.612
Want to delay next birth at least 2 years	0.268	0.022	604	765	1.215	0.082	0.224	0.311
Mothers protected against tetanus for last birth	0.864	0.027	299	379	1.347	0.031	0.810	0.917
Mothers received medical assistance at delivery	0.930	0.018	380	487	1.324	0.020	0.893	0.966
Had diarrhea in the last 2 weeks	0.169	0.027	369	470	1.357	0.162	0.114	0.223
Treated with ORS packets or pre-packed liquid	0.544	0.063	58	79	1.007	0.116	0.418	0.670
Consulted medical personnel for diarrhea	0.481	0.072	58	79	1.102	0.151	0.336	0.625
Vaccination card seen	0.919	0.029	79	100	0.952	0.032	0.861	0.978
Received BCG vaccination	0.982	0.013	79	100	0.883	0.014	0.955	1.008
Received DPT vaccination (3 doses)	0.909	0.042	79	100	1.306	0.047	0.824	0.994
Received polio vaccination (3 doses)	0.866	0.046	79	100	1.197	0.053	0.773	0.958
Received measles vaccination	0.851	0.040	79	100	0.999	0.047	0.771	0.932
Received all basic vaccinations	0.786	0.047	79	100	1.018	0.060	0.692	0.881
Height-for-age (-2SD)	0.359	0.034	257	323	1.101	0.096	0.290	0.428
Weight-for-height (-2SD)	0.095	0.022	257	323	1.211	0.226	0.052	0.138
Weight-for-age (-2SD)	0.262	0.031	257	323	1.066	0.117	0.201	0.324
Prevalence of anemia (children 6-59 months)	0.523	0.035	243	306	1.100	0.067	0.453	0.593
Prevalence of anemia (women 15-49)	0.411	0.025	615	722	1.252	0.061	0.360	0.461
Body mass index (BMI) <18.5	0.116	0.016	581	686	1.160	0.134	0.085	0.148
Had an HIV test and received results in past 12 months	0.106	0.012	943	1,137	1.172	0.111	0.082	0.129
Accepting attitudes towards people with HIV	0.323	0.020	923	1,110	1.321	0.063	0.282	0.363
Total fertility rate (last 3 years)	2.714	0.245	2,673	3,252	1.346	0.090	2.223	3.205
Neonatal mortality (last 0-9 years)	17.492	6.048	829	1,084	1.095	0.346	5.395	29.589
Postneonatal mortality (last 0-9 years)	22.959	7.613	835	1,092	1.178	0.332	7.732	38.186
Infant mortality (last 0-9 years)	40.451	8.948	829	1,084	1.075	0.221	22.554	58.347
Child mortality (last 0-9 years)	16.453	6.135	838	1,096	1.412	0.373	4.183	28.722
Under-5 mortality (last 0-9 years)	56.238	10.759	833	1,088	1.187	0.191	34.721	77.755
		MEN						
Urban residence	0.143	0.017	282	337	0.819	0.120	0.108	0.177
No education	0.208	0.032	282	337	1.315	0.153	0.145	0.272
Secondary or higher education	0.347	0.057	282	337	1.985	0.163	0.233	0.460
Never married (in union)	0.301	0.029	282	337	1.042	0.095	0.244	0.358
Currently married (in union)	0.680	0.029	282	337	1.038	0.043	0.622	0.738
Had first sexual intercourse before age 18	0.031	0.013	181	217	0.985	0.412	0.005	0.056
Want no more children	0.598	0.034	185	229	0.949	0.057	0.529	0.667
Want to delay birth at least 2 years	0.268	0.032	185	229	0.977	0.119	0.204	0.332
Had paid sex in past 12 months	0.038	0.013	282	337	1.142	0.341	0.012	0.065
Had HIV test and received results in past 12 months	0.051	0.014	282	337	1.095	0.281	0.023	0.080
Accepting attitudes towards people with HIV	0.406	0.028	277	331	0.940	0.068	0.350	0.461

Table B.16 Sampling errors: Svay Rieng sample, Cambodia 2014

Variable	R	SE	N	WN	DEFT	SE/R	R-2SE	R+2SE
				WOMEN				
Urban residence	0.037	0.004	822	654	0.549	0.098	0.030	0.044
No schooling	0.060	0.011	822	654	1.306	0.181	0.038	0.081
Secondary or higher education	0.352	0.027	822	654	1.590	0.075	0.299	0.405
Never married (never in union)	0.200	0.019	822	654	1.380	0.097	0.161	0.238
Currently married (in union)	0.738	0.021	822	654	1.343	0.028	0.696	0.779
Married before age 20	0.497	0.026	604	478	1.283	0.053	0.444	0.549
Had first sexual intercourse before age 18	0.269	0.025	604	478	1.412	0.095	0.218	0.320
Currently pregnant	0.057	0.008	822	654	0.996	0.142	0.041	0.073
Children ever born	1.967	0.096	822	654	1.578	0.049	1.775	2.159
Children surviving	1.789	0.076	822	654	1.418	0.042	1.638	1.940
Children ever born to women age 40-49	3.424	0.183	225	180	1.472	0.054	3.057	3.791
Know any contraceptive method	1.000	0.000	590	483	na	0.000	1.000	1.000
Know any modern contraceptive method	1.000	0.000	590	483	na	0.000	1.000	1.000
Currently using any method	0.579	0.027	590	483	1.325	0.047	0.525	0.633
Currently using a modern method	0.407	0.030	590	483	1.490	0.074	0.346	0.467
Currently using daily pill	0.197	0.028	590	483	1.732	0.144	0.140	0.254
Currently using male condoms	0.017	0.005	590	483	0.927	0.292	0.007	0.027
Currently using injectables	0.093	0.016	590	483	1.324	0.170	0.062	0.125
Used public sector source	0.519	0.046	231	196	1.396	0.089	0.427	0.611
Want no more children	0.606	0.018	590	483	0.912	0.030	0.569	0.643
Want to delay next birth at least 2 years	0.213	0.014	590	483	0.851	0.067	0.184	0.242
Mothers protected against tetanus for last birth	0.931	0.012	282	229	0.795	0.013	0.907	0.955
Mothers received medical assistance at delivery	0.943	0.018	320	261	1.328	0.019	0.907	0.980
Had diarrhea in the last 2 weeks	0.056	0.014	311	253	1.104	0.258	0.027	0.085
Treated with ORS packets or pre-packed liquid	0.412	0.103	17	14	0.873	0.249	0.207	0.617
Consulted medical personnel for diarrhea	0.640	0.125	17	14	1.095	0.196	0.390	0.891
Vaccination card seen	0.724	0.051	70	53	0.934	0.071	0.621	0.827
Received BCG vaccination	0.924	0.034	70	53	1.056	0.037	0.856	0.993
Received DPT vaccination (3 doses)	0.888	0.044	70	53	1.130	0.049	0.800	0.976
Received polio vaccination (3 doses)	0.888	0.044	70	53	1.130	0.049	0.800	0.976
Received measles vaccination	0.867	0.043	70	53	1.037	0.050	0.780	0.953
Received all basic vaccinations	0.827	0.046	70	53	0.981	0.055	0.735	0.918
Height-for-age (-2SD)	0.328	0.026	233	190	0.864	0.080	0.275	0.381
Weight-for-height (-2SD)	0.076	0.019	233	190	1.051	0.251	0.038	0.113
Weight-for-age (-2SD)	0.208	0.027	233	190	0.970	0.128	0.155	0.261
Prevalence of anemia (children 6-59 months)	0.498	0.045	206	168	1.259	0.090	0.409	0.588
Prevalence of anemia (women 15-49)	0.457	0.030	541	427	1.391	0.065	0.397	0.517
Body mass index (BMI) <18.5	0.131	0.021	514	405	1.420	0.162	0.089	0.174
Had an HIV test and received results in past 12 months	0.113	0.013	822	654	1.161	0.114	0.087	0.138
Accepting attitudes towards people with HIV	0.132	0.016	817	651	1.382	0.124	0.099	0.165
Total fertility rate (last 3 years)	2.502	0.167	2,373	1,887	0.974	0.067	2.169	2.835
Neonatal mortality (last 0-9 years)	20.018	6.977	635	519	1.271	0.349	6.064	33.972
Postneonatal mortality (last 0-9 years)	26.091	9.519	639	521	1.258	0.365	7.052	45.129
Infant mortality (last 0-9 years)	46.109	15.385	636	520	1.563	0.334	15.339	76.879
Child mortality (last 0-9 years)	17.581	6.519	633	517	1.101	0.371	4.543	30.619
Under-5 mortality (last 0-9 years)	62.879	20.106	638	521	1.756	0.320	22.667	103.090
				MEN				
Urban residence	0.036	0.008	237	183	0.638	0.215	0.020	0.051
No education	0.011	0.008	237	183	1.126	0.703	0.000	0.026
Secondary or higher education	0.601	0.038	237	183	1.200	0.064	0.525	0.678
Never married (in union)	0.272	0.035	237	183	1.214	0.129	0.202	0.342
Currently married (in union)	0.711	0.037	237	183	1.248	0.052	0.637	0.785
Had first sexual intercourse before age 18	0.231	0.044	157	123	1.291	0.189	0.144	0.319
Want no more children	0.447	0.034	160	130	0.862	0.076	0.379	0.514
Want to delay birth at least 2 years	0.347	0.032	160	130	0.845	0.092	0.283	0.411
Had paid sex in past 12 months	0.018	0.013	237	183	1.476	0.718	0.000	0.043
Had HIV test and received results in past 12 months	0.097	0.021	237	183	1.091	0.217	0.055	0.139
Accepting attitudes towards people with HIV	0.333	0.032	237	183	1.053	0.097	0.268	0.397

Table B.17 Sampling errors: Takeo sample, Cambodia 2014

Variable	R	SE	N	WN	DEFT	SE/R	R-2SE	R+2SE
		WOMEN						
Urban residence	0.019	0.003	868	1,082	0.562	0.136	0.014	0.024
No schooling	0.117	0.019	868	1,082	1.705	0.159	0.080	0.155
Secondary or higher education	0.495	0.031	868	1,082	1.814	0.062	0.434	0.557
Never married (never in union)	0.274	0.016	868	1,082	1.062	0.059	0.242	0.306
Currently married (in union)	0.626	0.018	868	1,082	1.110	0.029	0.589	0.662
Married before age 20	0.436	0.021	572	715	0.996	0.047	0.395	0.478
Had first sexual intercourse before age 18	0.227	0.016	572	715	0.894	0.069	0.196	0.259
Currently pregnant	0.039	0.006	868	1,082	0.947	0.160	0.026	0.051
Children ever born	1.782	0.074	868	1,082	1.237	0.042	1.634	1.930
Children surviving	1.687	0.068	868	1,082	1.231	0.041	1.550	1.824
Children ever born to women age 40-49	3.732	0.156	201	239	1.266	0.042	3.420	4.044
Know any contraceptive method	0.998	0.002	541	677	1.097	0.002	0.993	1.002
Know any modern contraceptive method	0.998	0.002	541	677	1.097	0.002	0.993	1.002
Currently using any method	0.601	0.020	541	677	0.951	0.033	0.561	0.641
Currently using a modern method	0.482	0.024	541	677	1.128	0.050	0.433	0.530
Currently using daily pill	0.196	0.033	541	677	1.934	0.169	0.130	0.263
Currently using male condoms	0.017	0.005	541	677	0.927	0.308	0.006	0.027
Currently using injectables	0.106	0.018	541	677	1.366	0.171	0.069	0.142
Used public sector source	0.534	0.045	248	328	1.403	0.084	0.445	0.624
Want no more children	0.427	0.020	541	677	0.920	0.046	0.388	0.467
Want to delay next birth at least 2 years	0.200	0.014	541	677	0.802	0.069	0.172	0.227
Mothers protected against tetanus for last birth	0.939	0.016	247	321	1.080	0.017	0.906	0.971
Mothers received medical assistance at delivery	0.974	0.010	297	386	0.986	0.010	0.954	0.994
Had diarrhea in the last 2 weeks	0.189	0.033	289	374	1.424	0.176	0.123	0.256
Treated with ORS packets or pre-packed liquid	0.577	0.077	51	71	1.134	0.134	0.422	0.732
Consulted medical personnel for diarrhea	0.525	0.071	51	71	1.024	0.135	0.383	0.668
Vaccination card seen	0.897	0.034	63	84	0.918	0.038	0.829	0.966
Received BCG vaccination	0.965	0.023	63	84	1.026	0.024	0.918	1.011
Received DPT vaccination (3 doses)	0.979	0.017	63	84	0.957	0.017	0.945	1.013
Received polio vaccination (3 doses)	0.931	0.031	63	84	0.988	0.033	0.870	0.993
Received measles vaccination	0.942	0.030	63	84	1.037	0.032	0.882	1.001
Received all basic vaccinations	0.878	0.039	63	84	0.966	0.045	0.799	0.956
Height-for-age (-2SD)	0.307	0.031	207	258	0.953	0.102	0.244	0.369
Weight-for-height (-2SD)	0.146	0.031	207	258	1.245	0.212	0.084	0.208
Weight-for-age (-2SD)	0.227	0.034	207	258	1.185	0.149	0.159	0.295
Prevalence of anemia (children 6-59 months)	0.531	0.045	197	245	1.254	0.085	0.441	0.622
Prevalence of anemia (women 15-49)	0.354	0.025	563	693	1.227	0.070	0.304	0.403
Body mass index (BMI) <18.5	0.139	0.016	531	653	1.032	0.112	0.108	0.170
Had an HIV test and received results in past 12 months	0.092	0.010	868	1,082	1.055	0.113	0.071	0.113
Accepting attitudes towards people with HIV	0.308	0.022	859	1,070	1.388	0.071	0.264	0.352
Total fertility rate (last 3 years)	2.368	0.166	2,437	3,038	1.134	0.070	2.036	2.701
Neonatal mortality (last 0-9 years)	15.751	6.119	625	810	0.981	0.388	3.513	27.989
Postneonatal mortality (last 0-9 years)	12.008	5.572	620	804	1.208	0.464	0.864	23.152
Infant mortality (last 0-9 years)	27.760	7.540	625	810	0.935	0.272	12.679	42.840
Child mortality (last 0-9 years)	3.665	2.574	617	793	1.052	0.702	0.000	8.813
Under-5 mortality (last 0-9 years)	31.322	8.771	625	810	1.027	0.280	13.781	48.864
		MEN						
Urban residence	0.014	0.004	252	334	0.583	0.304	0.006	0.023
No education	0.041	0.013	252	334	1.000	0.304	0.016	0.067
Secondary or higher education	0.581	0.046	252	334	1.464	0.079	0.490	0.672
Never married (in union)	0.339	0.024	252	334	0.793	0.070	0.292	0.387
Currently married (in union)	0.642	0.027	252	334	0.901	0.042	0.587	0.696
Had first sexual intercourse before age 18	0.114	0.025	173	224	1.020	0.217	0.065	0.164
Want no more children	0.529	0.036	163	214	0.910	0.067	0.458	0.600
Want to delay birth at least 2 years	0.297	0.041	163	214	1.148	0.139	0.215	0.380
Had paid sex in past 12 months	0.029	0.010	252	334	0.955	0.346	0.009	0.050
Had HIV test and received results in past 12 months	0.105	0.025	252	334	1.282	0.237	0.055	0.155
Accepting attitudes towards people with HIV	0.325	0.033	248	327	1.115	0.102	0.259	0.392

Table B.18 Sampling errors: Otdar Meanchey sample, Cambodia 2014

Variable	R	SE	N	WN	DEFT	SE/R	R-2SE	R+2SE
WOMEN								
Urban residence	0.070	0.011	823	294	1.234	0.157	0.048	0.092
No schooling	0.264	0.022	823	294	1.429	0.083	0.220	0.308
Secondary or higher education	0.286	0.032	823	294	2.035	0.112	0.222	0.351
Never married (never in union)	0.222	0.018	823	294	1.261	0.082	0.185	0.258
Currently married (in union)	0.739	0.018	823	294	1.155	0.024	0.704	0.775
Married before age 20	0.495	0.023	536	195	1.073	0.047	0.448	0.541
Had first sexual intercourse before age 18	0.247	0.019	536	195	1.018	0.077	0.209	0.285
Currently pregnant	0.085	0.010	823	294	0.981	0.113	0.066	0.104
Children ever born	2.063	0.120	823	294	1.676	0.058	1.823	2.303
Children surviving	1.947	0.109	823	294	1.646	0.056	1.730	2.165
Children ever born to women age 40-49	4.640	0.283	162	58	1.642	0.061	4.075	5.206
Know any contraceptive method	1.000	0.000	603	218	na	0.000	1.000	1.000
Know any modern contraceptive method	1.000	0.000	603	218	na	0.000	1.000	1.000
Currently using any method	0.566	0.030	603	218	1.461	0.052	0.507	0.625
Currently using a modern method	0.497	0.036	603	218	1.760	0.072	0.425	0.569
Currently using daily pill	0.244	0.027	603	218	1.538	0.110	0.190	0.298
Currently using male condoms	0.013	0.005	603	218	0.988	0.349	0.004	0.022
Currently using injectables	0.156	0.027	603	218	1.851	0.176	0.101	0.211
Used public sector source	0.589	0.049	308	109	1.729	0.083	0.492	0.686
Want no more children	0.515	0.025	603	218	1.246	0.049	0.465	0.566
Want to delay next birth at least 2 years	0.240	0.017	603	218	0.991	0.072	0.205	0.274
Mothers protected against tetanus for last birth	0.909	0.023	323	116	1.416	0.025	0.864	0.954
Mothers received medical assistance at delivery	0.887	0.043	382	137	2.569	0.049	0.800	0.974
Had diarrhea in the last 2 weeks	0.102	0.030	375	134	1.803	0.297	0.041	0.162
Treated with ORS packets or pre-packed liquid	0.226	0.073	35	14	1.050	0.322	0.080	0.371
Consulted medical personnel for diarrhea	0.299	0.107	35	14	1.356	0.358	0.085	0.514
Vaccination card seen	0.717	0.050	79	27	0.969	0.070	0.616	0.817
Received BCG vaccination	0.974	0.019	79	27	1.016	0.019	0.937	1.011
Received DPT vaccination (3 doses)	0.834	0.043	79	27	1.010	0.052	0.747	0.921
Received polio vaccination (3 doses)	0.818	0.047	79	27	1.053	0.057	0.724	0.912
Received measles vaccination	0.853	0.050	79	27	1.217	0.058	0.753	0.952
Received all basic vaccinations	0.750	0.058	79	27	1.159	0.077	0.634	0.865
Height-for-age (-2SD)	0.363	0.041	227	78	1.330	0.114	0.280	0.445
Weight-for-height (-2SD)	0.151	0.025	227	78	1.056	0.168	0.100	0.202
Weight-for-age (-2SD)	0.264	0.033	227	78	1.147	0.124	0.199	0.329
Prevalence of anemia (children 6-59 months)	0.643	0.034	227	77	1.037	0.053	0.576	0.711
Prevalence of anemia (women 15-49)	0.483	0.019	535	189	0.856	0.039	0.446	0.520
Body mass index (BMI) <18.5	0.128	0.017	490	170	1.086	0.130	0.095	0.161
Had an HIV test and received results in past 12 months	0.091	0.016	823	294	1.606	0.177	0.059	0.124
Accepting attitudes towards people with HIV	0.133	0.016	747	262	1.325	0.124	0.100	0.166
Total fertility rate (last 3 years)	3.028	0.253	2,341	838	1.278	0.084	2.522	3.535
Neonatal mortality (last 0-9 years)	16.804	4.467	774	283	0.963	0.266	7.869	25.739
Postneonatal mortality (last 0-9 years)	15.408	6.199	775	284	1.180	0.402	3.010	27.806
Infant mortality (last 0-9 years)	32.212	6.949	774	283	0.883	0.216	18.315	46.109
Child mortality (last 0-9 years)	9.289	5.140	761	276	1.402	0.553	0.000	19.569
Under-5 mortality (last 0-9 years)	41.202	10.562	774	283	1.205	0.256	20.078	62.326
MEN								
Urban residence	0.052	0.009	277	99	0.677	0.174	0.034	0.070
No education	0.130	0.020	277	99	1.004	0.157	0.089	0.170
Secondary or higher education	0.400	0.041	277	99	1.388	0.102	0.318	0.482
Never married (in union)	0.316	0.030	277	99	1.073	0.095	0.256	0.376
Currently married (in union)	0.673	0.033	277	99	1.160	0.049	0.607	0.739
Had first sexual intercourse before age 18	0.080	0.024	168	62	1.144	0.300	0.032	0.128
Want no more children	0.537	0.043	182	67	1.155	0.080	0.452	0.623
Want to delay birth at least 2 years	0.299	0.037	182	67	1.097	0.125	0.225	0.374
Had paid sex in past 12 months	0.000	0.000	277	99	na	na	0.000	0.000
Had HIV test and received results in past 12 months	0.053	0.024	277	99	1.766	0.453	0.005	0.100
Accepting attitudes towards people with HIV	0.400	0.042	275	99	1.406	0.104	0.317	0.483

Table B.19 Sampling errors: Battambang and Pailin sample, Cambodia 2014

Variable	R	SE	N	WN	DEFT	SE/R	R-2SE	R+2SE
**WOMEN**								
Urban residence	0.160	0.023	867	1,333	1.820	0.142	0.115	0.205
No schooling	0.100	0.017	867	1,333	1.686	0.172	0.065	0.134
Secondary or higher education	0.470	0.034	867	1,333	1.977	0.071	0.402	0.537
Never married (never in union)	0.280	0.019	867	1,333	1.218	0.066	0.243	0.317
Currently married (in union)	0.668	0.020	867	1,333	1.227	0.029	0.629	0.707
Married before age 20	0.431	0.029	552	847	1.357	0.066	0.374	0.488
Had first sexual intercourse before age 18	0.233	0.019	552	847	1.057	0.082	0.195	0.271
Currently pregnant	0.055	0.007	867	1,333	0.950	0.133	0.041	0.070
Children ever born	1.859	0.078	867	1,333	1.184	0.042	1.703	2.015
Children surviving	1.728	0.071	867	1,333	1.215	0.041	1.586	1.871
Children ever born to women age 40-49	3.785	0.175	203	280	1.166	0.046	3.435	4.135
Know any contraceptive method	0.998	0.002	555	890	0.951	0.002	0.995	1.002
Know any modern contraceptive method	0.998	0.002	555	890	0.951	0.002	0.995	1.002
Currently using any method	0.583	0.018	555	890	0.874	0.031	0.546	0.619
Currently using a modern method	0.406	0.031	555	890	1.482	0.076	0.344	0.468
Currently using daily pill	0.192	0.026	555	890	1.566	0.137	0.140	0.245
Currently using male condoms	0.015	0.006	555	890	1.161	0.407	0.003	0.026
Currently using injectables	0.082	0.017	555	890	1.468	0.209	0.048	0.116
Used public sector source	0.481	0.048	231	369	1.451	0.100	0.385	0.577
Want no more children	0.505	0.029	555	890	1.357	0.057	0.447	0.563
Want to delay next birth at least 2 years	0.304	0.029	555	890	1.488	0.096	0.246	0.362
Mothers protected against tetanus for last birth	0.797	0.033	272	460	1.372	0.041	0.732	0.862
Mothers received medical assistance at delivery	0.941	0.013	329	553	0.970	0.014	0.914	0.967
Had diarrhea in the last 2 weeks	0.209	0.027	322	545	1.255	0.128	0.155	0.262
Treated with ORS packets or pre-packed liquid	0.223	0.063	62	114	1.272	0.283	0.097	0.349
Consulted medical personnel for diarrhea	0.483	0.080	62	114	1.335	0.167	0.322	0.643
Vaccination card seen	0.812	0.039	63	120	0.876	0.049	0.734	0.891
Received BCG vaccination	0.995	0.005	63	120	0.620	0.005	0.985	1.005
Received DPT vaccination (3 doses)	0.954	0.023	63	120	0.939	0.024	0.909	1.000
Received polio vaccination (3 doses)	0.954	0.023	63	120	0.939	0.024	0.909	1.000
Received measles vaccination	0.896	0.043	63	120	1.227	0.048	0.810	0.983
Received all basic vaccinations	0.892	0.044	63	120	1.229	0.049	0.803	0.980
Height-for-age (-2SD)	0.249	0.037	232	388	1.301	0.150	0.174	0.324
Weight-for-height (-2SD)	0.079	0.026	232	388	1.515	0.327	0.027	0.131
Weight-for-age (-2SD)	0.182	0.038	232	388	1.549	0.209	0.106	0.257
Prevalence of anemia (children 6-59 months)	0.490	0.059	210	344	1.815	0.121	0.371	0.609
Prevalence of anemia (women 15-49)	0.425	0.018	557	843	0.841	0.042	0.390	0.461
Body mass index (BMI) <18.5	0.119	0.017	531	800	1.216	0.145	0.085	0.154
Had an HIV test and received results in past 12 months	0.120	0.010	867	1,333	0.906	0.083	0.100	0.141
Accepting attitudes towards people with HIV	0.480	0.044	861	1,323	2.582	0.092	0.392	0.568
Total fertility rate (last 3 years)	2.931	0.232	2,450	3,768	1.402	0.079	2.467	3.396
Neonatal mortality (last 0-9 years)	12.273	4.812	672	1,149	1.181	0.392	2.649	21.898
Postneonatal mortality (last 0-9 years)	15.742	7.959	672	1,153	1.101	0.506	0.000	31.660
Infant mortality (last 0-9 years)	28.015	9.502	673	1,150	1.141	0.339	9.010	47.019
Child mortality (last 0-9 years)	9.246	4.226	650	1,093	1.104	0.457	0.793	17.699
Under-5 mortality (last 0-9 years)	37.002	10.954	674	1,151	1.139	0.296	15.094	58.911
**MEN**								
Urban residence	0.125	0.026	249	405	1.259	0.212	0.072	0.178
No education	0.023	0.012	249	405	1.203	0.494	0.000	0.047
Secondary or higher education	0.588	0.055	249	405	1.767	0.094	0.477	0.699
Never married (in union)	0.355	0.045	249	405	1.479	0.127	0.265	0.446
Currently married (in union)	0.611	0.050	249	405	1.613	0.082	0.511	0.711
Had first sexual intercourse before age 18	0.081	0.039	165	256	1.799	0.476	0.004	0.158
Want no more children	0.375	0.052	157	247	1.329	0.138	0.272	0.478
Want to delay birth at least 2 years	0.480	0.046	157	247	1.156	0.097	0.387	0.572
Had paid sex in past 12 months	0.004	0.003	249	405	0.807	0.776	0.000	0.011
Had HIV test and received results in past 12 months	0.088	0.024	249	405	1.327	0.272	0.040	0.136
Accepting attitudes towards people with HIV	0.657	0.049	247	403	1.629	0.075	0.558	0.756

Table B.20 Sampling errors: Kampot and Kep sample, Cambodia 2014

Variable	R	SE	N	WN	DEFT	SE/R	R-2SE	R+2SE
WOMEN								
Urban residence	0.089	0.008	880	770	0.837	0.090	0.073	0.105
No schooling	0.085	0.016	880	770	1.692	0.187	0.053	0.117
Secondary or higher education	0.442	0.034	880	770	2.006	0.076	0.374	0.509
Never married (never in union)	0.197	0.016	880	770	1.161	0.079	0.166	0.228
Currently married (in union)	0.745	0.018	880	770	1.246	0.025	0.708	0.782
Married before age 20	0.517	0.028	595	534	1.356	0.054	0.462	0.573
Had first sexual intercourse before age 18	0.317	0.029	595	534	1.521	0.092	0.258	0.375
Currently pregnant	0.049	0.007	880	770	0.949	0.141	0.035	0.063
Children ever born	2.172	0.089	880	770	1.300	0.041	1.994	2.349
Children surviving	1.959	0.077	880	770	1.296	0.039	1.805	2.113
Children ever born to women age 40-49	3.910	0.218	251	220	1.570	0.056	3.475	4.346
Know any contraceptive method	1.000	0.000	625	574	na	0.000	1.000	1.000
Know any modern contraceptive method	1.000	0.000	625	574	na	0.000	1.000	1.000
Currently using any method	0.538	0.024	625	574	1.202	0.045	0.490	0.586
Currently using a modern method	0.383	0.024	625	574	1.236	0.063	0.335	0.431
Currently using daily pill	0.163	0.019	625	574	1.262	0.115	0.126	0.200
Currently using male condoms	0.017	0.006	625	574	1.226	0.376	0.004	0.029
Currently using injectables	0.107	0.016	625	574	1.263	0.146	0.076	0.138
Used public sector source	0.508	0.049	226	219	1.468	0.097	0.410	0.606
Want no more children	0.500	0.030	625	574	1.493	0.060	0.440	0.560
Want to delay next birth at least 2 years	0.229	0.017	625	574	0.990	0.073	0.196	0.262
Mothers protected against tetanus for last birth	0.867	0.032	252	236	1.525	0.037	0.803	0.932
Mothers received medical assistance at delivery	0.905	0.032	294	276	1.778	0.036	0.840	0.970
Had diarrhea in the last 2 weeks	0.051	0.012	290	272	0.957	0.238	0.027	0.075
Treated with ORS packets or pre-packed liquid	0.265	0.139	13	14	1.227	0.525	0.000	0.544
Consulted medical personnel for diarrhea	0.759	0.107	13	14	0.978	0.141	0.545	0.974
Vaccination card seen	0.707	0.093	58	54	1.556	0.131	0.522	0.892
Received BCG vaccination	0.940	0.032	58	54	1.036	0.034	0.876	1.004
Received DPT vaccination (3 doses)	0.800	0.058	58	54	1.116	0.073	0.683	0.917
Received polio vaccination (3 doses)	0.800	0.058	58	54	1.116	0.073	0.683	0.917
Received measles vaccination	0.811	0.058	58	54	1.141	0.072	0.694	0.928
Received all basic vaccinations	0.720	0.063	58	54	1.081	0.088	0.593	0.846
Height-for-age (-2SD)	0.252	0.033	217	195	1.093	0.131	0.186	0.318
Weight-for-height (-2SD)	0.082	0.023	217	195	1.138	0.275	0.037	0.128
Weight-for-age (-2SD)	0.211	0.032	217	195	1.147	0.153	0.146	0.276
Prevalence of anemia (children 6-59 months)	0.573	0.040	194	177	1.153	0.069	0.494	0.653
Prevalence of anemia (women 15-49)	0.441	0.034	546	479	1.607	0.077	0.373	0.510
Body mass index (BMI) <18.5	0.170	0.020	518	455	1.191	0.116	0.130	0.209
Had an HIV test and received results in past 12 months	0.066	0.011	880	770	1.273	0.161	0.045	0.088
Accepting attitudes towards people with HIV	0.468	0.026	878	768	1.558	0.056	0.415	0.520
Total fertility rate (last 3 years)	2.515	0.223	2,493	2,186	1.254	0.089	2.068	2.961
Neonatal mortality (last 0-9 years)	20.445	5.176	637	599	0.882	0.253	10.094	30.796
Postneonatal mortality (last 0-9 years)	17.283	6.820	636	599	1.228	0.395	3.642	30.923
Infant mortality (last 0-9 years)	37.728	6.844	637	599	0.875	0.181	24.041	51.415
Child mortality (last 0-9 years)	6.005	3.289	648	610	1.090	0.548	0.000	12.582
Under-5 mortality (last 0-9 years)	43.506	8.671	638	600	1.003	0.199	26.164	60.848
MEN								
Urban residence	0.088	0.015	284	241	0.891	0.170	0.058	0.118
No education	0.061	0.023	284	241	1.643	0.384	0.014	0.108
Secondary or higher education	0.520	0.040	284	241	1.333	0.076	0.440	0.599
Never married (in union)	0.255	0.028	284	241	1.075	0.109	0.199	0.311
Currently married (in union)	0.717	0.029	284	241	1.091	0.041	0.659	0.775
Had first sexual intercourse before age 18	0.137	0.032	188	163	1.256	0.231	0.073	0.200
Want no more children	0.589	0.041	193	173	1.143	0.069	0.508	0.670
Want to delay birth at least 2 years	0.274	0.034	193	173	1.055	0.124	0.206	0.342
Had paid sex in past 12 months	0.024	0.009	284	241	0.947	0.363	0.006	0.041
Had HIV test and received results in past 12 months	0.033	0.010	284	241	0.953	0.306	0.013	0.053
Accepting attitudes towards people with HIV	0.319	0.039	276	235	1.377	0.121	0.242	0.397

Table B.21 Sampling errors: Preah Sihanouk and Koh Kong sample, Cambodia 2014

Variable	R	SE	N	WN	DEFT	SE/R	R-2SE	R+2SE
WOMEN								
Urban residence	0.410	0.045	1,010	422	2.896	0.110	0.320	0.500
No schooling	0.096	0.013	1,010	422	1.418	0.137	0.070	0.122
Secondary or higher education	0.420	0.030	1,010	422	1.915	0.071	0.360	0.479
Never married (never in union)	0.300	0.016	1,010	422	1.134	0.055	0.267	0.333
Currently married (in union)	0.629	0.019	1,010	422	1.245	0.030	0.592	0.667
Married before age 20	0.493	0.017	631	261	0.861	0.035	0.458	0.527
Had first sexual intercourse before age 18	0.240	0.015	631	261	0.892	0.063	0.210	0.270
Currently pregnant	0.058	0.007	1,010	422	0.973	0.124	0.044	0.072
Children ever born	1.820	0.053	1,010	422	0.829	0.029	1.715	1.926
Children surviving	1.688	0.044	1,010	422	0.785	0.026	1.599	1.777
Children ever born to women age 40-49	4.121	0.176	204	83	1.088	0.043	3.769	4.474
Know any contraceptive method	0.998	0.002	636	266	1.007	0.002	0.995	1.002
Know any modern contraceptive method	0.998	0.002	636	266	1.007	0.002	0.995	1.002
Currently using any method	0.606	0.025	636	266	1.293	0.041	0.556	0.656
Currently using a modern method	0.415	0.023	636	266	1.179	0.056	0.369	0.461
Currently using daily pill	0.188	0.016	636	266	1.036	0.085	0.156	0.220
Currently using male condoms	0.020	0.006	636	266	1.088	0.306	0.008	0.031
Currently using injectables	0.065	0.013	636	266	1.288	0.194	0.040	0.090
Used public sector source	0.447	0.041	259	111	1.308	0.091	0.366	0.528
Want no more children	0.513	0.019	636	266	0.960	0.037	0.475	0.552
Want to delay next birth at least 2 years	0.279	0.014	636	266	0.769	0.049	0.252	0.307
Mothers protected against tetanus for last birth	0.896	0.013	336	142	0.789	0.015	0.870	0.922
Mothers received medical assistance at delivery	0.975	0.009	403	168	1.053	0.010	0.956	0.993
Had diarrhea in the last 2 weeks	0.170	0.021	393	164	1.006	0.121	0.129	0.211
Treated with ORS packets or pre-packed liquid	0.479	0.073	64	28	1.166	0.152	0.333	0.624
Consulted medical personnel for diarrhea	0.588	0.058	64	28	0.918	0.099	0.472	0.704
Vaccination card seen	0.797	0.047	67	28	0.959	0.059	0.703	0.891
Received BCG vaccination	0.980	0.015	67	28	0.894	0.016	0.950	1.011
Received DPT vaccination (3 doses)	0.920	0.032	67	28	0.957	0.034	0.857	0.984
Received polio vaccination (3 doses)	0.899	0.037	67	28	0.998	0.041	0.826	0.972
Received measles vaccination	0.864	0.047	67	28	1.127	0.054	0.770	0.959
Received all basic vaccinations	0.826	0.049	67	28	1.069	0.060	0.727	0.924
Height-for-age (-2SD)	0.334	0.023	261	105	0.755	0.068	0.289	0.379
Weight-for-height (-2SD)	0.105	0.019	261	105	1.023	0.180	0.067	0.142
Weight-for-age (-2SD)	0.220	0.023	261	105	0.866	0.106	0.174	0.267
Prevalence of anemia (children 6-59 months)	0.581	0.033	228	92	1.014	0.057	0.514	0.647
Prevalence of anemia (women 15-49)	0.437	0.030	635	267	1.542	0.069	0.376	0.498
Body mass index (BMI) <18.5	0.156	0.019	608	254	1.257	0.119	0.119	0.194
Had an HIV test and received results in past 12 months	0.163	0.015	1,010	422	1.291	0.092	0.133	0.193
Accepting attitudes towards people with HIV	0.330	0.012	1,003	419	0.816	0.037	0.306	0.355
Total fertility rate (last 3 years)	2.719	0.164	2,865	1,197	0.857	0.060	2.390	3.048
Neonatal mortality (last 0-9 years)	19.753	7.113	809	340	1.207	0.360	5.527	33.979
Postneonatal mortality (last 0-9 years)	15.245	4.207	815	341	0.829	0.276	6.832	23.658
Infant mortality (last 0-9 years)	34.998	7.449	812	340	0.955	0.213	20.099	49.896
Child mortality (last 0-9 years)	7.404	3.327	815	339	1.037	0.449	0.750	14.058
Under-5 mortality (last 0-9 years)	42.142	7.038	814	341	0.878	0.167	28.067	56.218
MEN								
Urban residence	0.371	0.046	288	120	1.600	0.123	0.280	0.463
No education	0.047	0.013	288	120	1.026	0.272	0.022	0.073
Secondary or higher education	0.582	0.059	288	120	2.023	0.102	0.463	0.700
Never married (in union)	0.353	0.036	288	120	1.280	0.102	0.281	0.425
Currently married (in union)	0.623	0.038	288	120	1.313	0.060	0.547	0.698
Had first sexual intercourse before age 18	0.077	0.023	196	85	1.179	0.292	0.032	0.122
Want no more children	0.441	0.044	174	75	1.177	0.101	0.352	0.529
Want to delay birth at least 2 years	0.366	0.041	174	75	1.111	0.111	0.285	0.448
Had paid sex in past 12 months	0.043	0.011	288	120	0.898	0.249	0.022	0.065
Had HIV test and received results in past 12 months	0.139	0.023	288	120	1.149	0.169	0.092	0.186
Accepting attitudes towards people with HIV	0.315	0.029	288	120	1.055	0.092	0.257	0.372

Table B.22 Sampling errors: Preah Vihear and Stung Treng sample, Cambodia 2014

Variable	R	SE	N	WN	DEFT	SE/R	R-2SE	R+2SE
				WOMEN				
Urban residence	0.092	0.012	1,085	462	1.409	0.134	0.068	0.117
No schooling	0.237	0.035	1,085	462	2.695	0.147	0.167	0.307
Secondary or higher education	0.241	0.035	1,085	462	2.714	0.147	0.170	0.311
Never married (never in union)	0.234	0.020	1,085	462	1.571	0.086	0.194	0.274
Currently married (in union)	0.679	0.026	1,085	462	1.853	0.039	0.627	0.732
Married before age 20	0.503	0.030	645	279	1.545	0.061	0.442	0.563
Had first sexual intercourse before age 18	0.305	0.033	645	279	1.836	0.109	0.238	0.372
Currently pregnant	0.095	0.012	1,085	462	1.377	0.129	0.070	0.119
Children ever born	2.292	0.100	1,085	462	1.342	0.044	2.092	2.491
Children surviving	1.981	0.090	1,085	462	1.483	0.046	1.800	2.162
Children ever born to women age 40-49	5.230	0.263	202	85	1.397	0.050	4.704	5.757
Know any contraceptive method	0.997	0.003	695	314	1.439	0.003	0.991	1.003
Know any modern contraceptive method	0.993	0.004	695	314	1.343	0.004	0.984	1.001
Currently using any method	0.420	0.021	695	314	1.147	0.051	0.377	0.463
Currently using a modern method	0.349	0.019	695	314	1.053	0.055	0.311	0.387
Currently using daily pill	0.178	0.018	695	314	1.254	0.102	0.141	0.214
Currently using male condoms	0.014	0.004	695	314	0.928	0.296	0.006	0.022
Currently using injectables	0.132	0.018	695	314	1.375	0.134	0.096	0.167
Used public sector source	0.622	0.038	238	109	1.214	0.062	0.546	0.699
Want no more children	0.490	0.022	695	314	1.136	0.044	0.447	0.533
Want to delay next birth at least 2 years	0.314	0.019	695	314	1.087	0.061	0.276	0.353
Mothers protected against tetanus for last birth	0.888	0.031	402	188	1.998	0.035	0.826	0.950
Mothers received medical assistance at delivery	0.546	0.077	510	239	2.961	0.140	0.393	0.699
Had diarrhea in the last 2 weeks	0.193	0.030	489	228	1.691	0.157	0.132	0.254
Treated with ORS packets or pre-packed liquid	0.284	0.069	92	44	1.485	0.244	0.145	0.422
Consulted medical personnel for diarrhea	0.603	0.063	92	44	1.226	0.104	0.478	0.728
Vaccination card seen	0.746	0.051	104	44	1.165	0.069	0.643	0.848
Received BCG vaccination	0.919	0.027	104	44	0.963	0.029	0.865	0.972
Received DPT vaccination (3 doses)	0.793	0.050	104	44	1.228	0.063	0.692	0.893
Received polio vaccination (3 doses)	0.777	0.055	104	44	1.305	0.071	0.667	0.886
Received measles vaccination	0.628	0.065	104	44	1.325	0.103	0.499	0.757
Received all basic vaccinations	0.556	0.061	104	44	1.224	0.110	0.433	0.679
Height-for-age (-2SD)	0.443	0.027	306	142	0.996	0.062	0.388	0.497
Weight-for-height (-2SD)	0.138	0.027	306	142	1.436	0.197	0.084	0.192
Weight-for-age (-2SD)	0.307	0.036	306	142	1.413	0.118	0.235	0.380
Prevalence of anemia (children 6-59 months)	0.688	0.035	275	128	1.209	0.051	0.619	0.758
Prevalence of anemia (women 15-49)	0.537	0.023	741	313	1.235	0.042	0.491	0.582
Body mass index (BMI) <18.5	0.164	0.015	678	282	1.007	0.088	0.135	0.193
Had an HIV test and received results in past 12 months	0.049	0.008	1,085	462	1.181	0.158	0.034	0.065
Accepting attitudes towards people with HIV	0.116	0.015	1,025	428	1.455	0.126	0.087	0.145
Total fertility rate (last 3 years)	3.593	0.255	3,006	1,279	1.508	0.071	3.083	4.102
Neonatal mortality (last 0-9 years)	25.492	7.587	996	481	1.445	0.298	10.319	40.666
Postneonatal mortality (last 0-9 years)	44.525	10.528	1,003	486	1.356	0.236	23.470	65.581
Infant mortality (last 0-9 years)	70.018	13.733	999	483	1.476	0.196	42.551	97.484
Child mortality (last 0-9 years)	9.450	3.186	965	472	1.048	0.337	3.078	15.821
Under-5 mortality (last 0-9 years)	78.806	11.805	1,002	485	1.239	0.150	55.195	102.416
				MEN				
Urban residence	0.085	0.011	274	112	0.678	0.135	0.062	0.108
No education	0.117	0.037	274	112	1.893	0.317	0.043	0.190
Secondary or higher education	0.345	0.041	274	112	1.434	0.120	0.262	0.427
Never married (in union)	0.297	0.042	274	112	1.532	0.143	0.212	0.381
Currently married (in union)	0.684	0.043	274	112	1.534	0.063	0.598	0.771
Had first sexual intercourse before age 18	0.069	0.030	152	65	1.444	0.434	0.009	0.129
Want no more children	0.395	0.062	173	77	1.649	0.156	0.272	0.519
Want to delay birth at least 2 years	0.479	0.053	173	77	1.399	0.112	0.372	0.586
Had paid sex in past 12 months	0.018	0.008	274	112	1.010	0.451	0.002	0.034
Had HIV test and received results in past 12 months	0.015	0.007	274	112	0.993	0.492	0.000	0.029
Accepting attitudes towards people with HIV	0.528	0.041	237	93	1.266	0.078	0.446	0.611

Table B.23 Sampling errors: Mondul Kiri and Ratanak Kiri sample, Cambodia 2014

Variable	R	SE	N	WN	DEFT	SE/R	R-2SE	R+2SE
WOMEN								
Urban residence	0.120	0.036	964	372	3.436	0.302	0.047	0.192
No schooling	0.345	0.051	964	372	3.300	0.147	0.244	0.447
Secondary or higher education	0.265	0.043	964	372	3.027	0.163	0.179	0.352
Never married (never in union)	0.175	0.020	964	372	1.618	0.113	0.136	0.215
Currently married (in union)	0.757	0.021	964	372	1.487	0.027	0.716	0.798
Married before age 20	0.586	0.028	590	227	1.387	0.048	0.530	0.642
Had first sexual intercourse before age 18	0.380	0.032	590	227	1.590	0.084	0.316	0.444
Currently pregnant	0.069	0.013	964	372	1.544	0.183	0.044	0.094
Children ever born	2.299	0.108	964	372	1.522	0.047	2.083	2.516
Children surviving	2.038	0.089	964	372	1.453	0.043	1.861	2.215
Children ever born to women age 40-49	4.808	0.229	164	64	1.189	0.048	4.349	5.267
Know any contraceptive method	0.973	0.012	705	281	1.940	0.012	0.949	0.996
Know any modern contraceptive method	0.973	0.012	705	281	1.940	0.012	0.949	0.996
Currently using any method	0.500	0.032	705	281	1.676	0.063	0.437	0.563
Currently using a modern method	0.427	0.031	705	281	1.675	0.073	0.365	0.490
Currently using daily pill	0.185	0.022	705	281	1.482	0.117	0.142	0.229
Currently using male condoms	0.005	0.003	705	281	1.087	0.570	0.000	0.011
Currently using injectables	0.163	0.019	705	281	1.375	0.118	0.125	0.201
Used public sector source	0.555	0.066	297	120	2.252	0.118	0.424	0.686
Want no more children	0.482	0.023	705	281	1.207	0.047	0.437	0.528
Want to delay next birth at least 2 years	0.339	0.022	705	281	1.231	0.065	0.295	0.383
Mothers protected against tetanus for last birth	0.718	0.043	421	169	1.962	0.059	0.633	0.803
Mothers received medical assistance at delivery	0.536	0.072	538	217	2.747	0.135	0.391	0.680
Had diarrhea in the last 2 weeks	0.156	0.024	521	208	1.447	0.154	0.108	0.204
Treated with ORS packets or pre-packed liquid	0.428	0.088	72	32	1.487	0.205	0.252	0.604
Consulted medical personnel for diarrhea	0.533	0.081	72	32	1.391	0.153	0.370	0.696
Vaccination card seen	0.411	0.071	99	35	1.365	0.173	0.269	0.553
Received BCG vaccination	0.814	0.041	99	35	1.007	0.051	0.732	0.897
Received DPT vaccination (3 doses)	0.559	0.063	99	35	1.200	0.113	0.433	0.686
Received polio vaccination (3 doses)	0.541	0.062	99	35	1.178	0.115	0.417	0.665
Received measles vaccination	0.561	0.075	99	35	1.421	0.133	0.411	0.710
Received all basic vaccinations	0.439	0.064	99	35	1.224	0.146	0.311	0.568
Height-for-age (-2SD)	0.398	0.036	330	125	1.293	0.090	0.326	0.469
Weight-for-height (-2SD)	0.082	0.021	330	125	1.386	0.260	0.039	0.124
Weight-for-age (-2SD)	0.262	0.047	330	125	1.820	0.179	0.168	0.355
Prevalence of anemia (children 6-59 months)	0.577	0.043	306	117	1.508	0.075	0.491	0.663
Prevalence of anemia (women 15-49)	0.417	0.032	624	239	1.617	0.077	0.353	0.481
Body mass index (BMI) <18.5	0.104	0.016	578	219	1.212	0.149	0.073	0.136
Had an HIV test and received results in past 12 months	0.041	0.008	964	372	1.322	0.205	0.024	0.058
Accepting attitudes towards people with HIV	0.056	0.015	754	278	1.786	0.267	0.026	0.086
Total fertility rate (last 3 years)	3.255	0.327	2,748	1,060	1.832	0.100	2.601	3.909
Neonatal mortality (last 0-9 years)	36.099	6.057	1,034	413	1.012	0.168	23.984	48.213
Postneonatal mortality (last 0-9 years)	36.137	7.972	1,040	416	1.276	0.221	20.193	52.081
Infant mortality (last 0-9 years)	72.235	9.740	1,036	414	1.093	0.135	52.756	91.715
Child mortality (last 0-9 years)	8.904	3.785	1,032	416	1.238	0.425	1.334	16.473
Under-5 mortality (last 0-9 years)	80.496	10.279	1,038	415	1.070	0.128	59.938	101.053
MEN								
Urban residence	0.097	0.026	350	134	1.624	0.266	0.045	0.148
No education	0.164	0.029	350	134	1.463	0.177	0.106	0.222
Secondary or higher education	0.404	0.056	350	134	2.135	0.140	0.291	0.517
Never married (in union)	0.344	0.022	350	134	0.875	0.065	0.300	0.389
Currently married (in union)	0.650	0.022	350	134	0.854	0.034	0.606	0.693
Had first sexual intercourse before age 18	0.133	0.028	207	79	1.183	0.211	0.077	0.188
Want no more children	0.428	0.033	223	87	1.002	0.078	0.362	0.495
Want to delay birth at least 2 years	0.445	0.030	223	87	0.898	0.067	0.385	0.505
Had paid sex in past 12 months	0.007	0.005	350	134	1.065	0.668	0.000	0.017
Had HIV test and received results in past 12 months	0.025	0.009	350	134	1.084	0.361	0.007	0.043
Accepting attitudes towards people with HIV	0.059	0.021	313	114	1.599	0.364	0.016	0.101

DATA QUALITY TABLES *Appendix* C

Table C.1 Household age distribution

Single-year age distribution of the de facto household population by sex (weighted), Cambodia 2014

	Women		Men			Women		Men	
Age	Number	Percent	Number	Percent	Age	Number	Percent	Number	Percent
0	771	2.1	752	2.3	37	326	0.9	274	0.8
1	777	2.1	811	2.5	38	296	0.8	264	0.8
2	740	2.0	791	2.4	39	374	1.0	324	1.0
3	762	2.1	799	2.4	40	349	1.0	345	1.0
4	781	2.1	765	2.3	41	389	1.1	338	1.0
5	747	2.1	769	2.3	42	441	1.2	369	1.1
6	846	2.3	911	2.8	43	366	1.0	323	1.0
7	901	2.5	907	2.7	44	474	1.3	371	1.1
8	776	2.1	829	2.5	45	406	1.1	371	1.1
9	794	2.2	839	2.5	46	364	1.0	301	0.9
10	828	2.3	793	2.4	47	367	1.0	315	1.0
11	754	2.1	735	2.2	48	357	1.0	305	0.9
12	808	2.2	790	2.4	49	389	1.1	313	0.9
13	800	2.2	798	2.4	50	289	0.8	292	0.9
14	767	2.1	815	2.5	51	418	1.1	333	1.0
15	628	1.7	701	2.1	52	437	1.2	307	0.9
16	575	1.6	618	1.9	53	384	1.1	279	0.8
17	580	1.6	580	1.8	54	361	1.0	285	0.9
18	564	1.5	563	1.7	55	381	1.0	249	0.8
19	545	1.5	591	1.8	56	333	0.9	222	0.7
20	613	1.7	590	1.8	57	313	0.9	212	0.6
21	632	1.7	570	1.7	58	337	0.9	166	0.5
22	600	1.6	574	1.7	59	249	0.7	144	0.4
23	575	1.6	530	1.6	60	269	0.7	179	0.5
24	633	1.7	506	1.5	61	248	0.7	142	0.4
25	561	1.5	554	1.7	62	242	0.7	159	0.5
26	581	1.6	502	1.5	63	227	0.6	161	0.5
27	576	1.6	536	1.6	64	262	0.7	150	0.5
28	566	1.6	482	1.5	65	250	0.7	139	0.4
29	540	1.5	528	1.6	66	179	0.5	103	0.3
30	579	1.6	520	1.6	67	158	0.4	159	0.5
31	598	1.6	537	1.6	68	194	0.5	112	0.3
32	649	1.8	599	1.8	69	129	0.4	89	0.3
33	579	1.6	502	1.5	70+	1,405	3.9	971	2.9
34	601	1.7	541	1.6	Don't know/ missing	4	0.0	0	0.0
35	518	1.4	439	1.3					
36	291	0.8	332	1.0	Total	36,405	100.0	33,066	100.0

Table C.2.1 Age distribution of eligible and interviewed women

De facto household population of women age 10-54, interviewed women age 15-49; and percent distribution and percentage of eligible women who were interviewed (weighted), by five-year age groups, Cambodia 2014

Age group	Household population of women age 10-54	Interviewed women age 15-49		Percentage of eligible women interviewed
		Number	Percentage	
10-14	3,957	na	na	na
15-19	2,891	2,794	16.4	96.6
20-24	3,054	2,943	17.3	96.4
25-29	2,825	2,747	16.1	97.2
30-34	3,005	2,944	17.3	97.9
35-39	1,806	1,779	10.4	98.5
40-44	2,019	1,985	11.6	98.3
45-49	1,883	1,849	10.9	98.2
50-54	1,889	na	na	na
15-49	17,484	17,041	100.0	97.5

Note: The de facto population includes all residents and nonresidents who stayed in the household the night before the interview. Weights for both household population of women and interviewed women are household weights. Age is based on the household questionnaire.
na = Not applicable

Table C.2.2 Age distribution of eligible and interviewed men

De facto household population of men age 10-54, interviewed men age 15-49 and percent of eligible men who were interviewed (weighted), by five-year age groups, Cambodia 2014

Age group	Household population of men age 10-54	Interviewed men age 15-49		Percentage of eligible men interviewed
		Number	Percentage	
10-14	1,391	na	na	na
15-19	963	911	18.0	94.6
20-24	873	804	15.9	92.1
25-29	867	798	15.8	92.0
30-34	942	883	17.4	93.7
35-39	572	548	10.8	95.8
40-44	596	571	11.3	95.8
45-49	575	548	10.8	95.4
50-54	518	na	na	na
15-49	5,389	5,064	100.0	94.0

Note: The de facto population includes all residents and nonresidents who stayed in the household the night before the interview. Weights for both household population of men and interviewed men are household weights. Age is based on the household questionnaire.
na = Not applicable

Table C.3 Completeness of reporting

Percentage of observations missing information for selected demographic and health questions (weighted), Cambodia 2014

Subject	Percentage with information missing	Number of cases
Month only (births in the 15 years preceding the survey)	0.11	21,687
Month and year (births in the 15 years preceding the survey)	0.00	21,687
Age at death (deceased children born in the 15 years preceding the survey)	0.07	1,217
Age/date at first union[1] (ever married women age 15-49)	0.07	13,150
Age/date at first union[1] (ever married men age 15-49)	0.00	3,527
Respondent's education (all women age 15-49)	0.00	17,578
Respondent's education (all men age 15-49)	0.00	5,190
Diarrhea in last 2 weeks (living children age 0-59 months)	0.48	7,044
Height (living children age 0-59 months from the Household Questionnaire)	2.09	5,092
Weight (living children age 0-59 months from the Household Questionnaire)	1.63	5,092
Height or weight (living children age 0-59 months from the Household Questionnaire)	2.09	5,092
Height (women age 15-49 from the Household Questionnaire)	2.36	11,362
Weight (women age 15-49 from the Household Questionnaire)	2.35	11,362
Height or weight (women age 15-49 from the Household Questionnaire)	2.39	11,362
Anemia (living children age 6-59 months from the Household Questionnaire)	3.45	4,615
Anemia (all women from the Household Questionnaire)	3.29	11,362

[1] Both year and age missing

Table C.4 Births by calendar years

Number of births, percentage with complete birth date, sex ratio at birth, and calendar year ratio by calendar year, according to living, dead, and total children (weighted), Cambodia 2014

Calendar	Number of births			Percentage with complete birth date[1]			Sex ratio at birth[2]			Calendar year ratio[3]		
year	Living	Dead	Total	Living	Dead	Total	Living	Dead	Total	Living	Dead	Total
2014	972	16	988	100.0	100.0	100.0	90.3	83.6	90.2	na	na	na
2013	1,461	44	1,505	100.0	100.0	100.0	110.9	160.6	112.1	120.0	159.6	120.9
2012	1,464	39	1,502	100.0	100.0	100.0	100.7	139.8	101.5	106.4	97.5	106.1
2011	1,290	36	1,326	100.0	100.0	100.0	96.1	95.5	96.1	90.6	78.9	90.3
2010	1,384	52	1,436	100.0	100.0	100.0	108.1	123.6	108.6	105.2	89.3	104.5
2009	1,342	81	1,422	100.0	100.0	100.0	93.2	105.0	93.9	92.8	110.7	93.6
2008	1,509	94	1,602	99.7	98.8	99.7	108.2	230.4	112.8	103.9	111.4	104.4
2007	1,561	87	1,649	99.9	97.4	99.8	97.3	148.7	99.4	109.5	106.1	109.3
2006	1,344	71	1,415	99.9	99.6	99.8	106.2	134.6	107.5	89.8	69.9	88.5
0-4	5,187	134	5,321	100.0	100.0	100.0	100.2	123.9	100.8	na	na	na
5-9	7,140	384	7,525	99.9	99.0	99.9	102.4	146.1	104.3	na	na	na
10-14	6,442	493	6,935	99.9	99.8	99.9	104.6	105.7	104.7	na	na	na
15-19	5,738	727	6,465	99.9	98.4	99.7	105.6	132.0	108.2	na	na	na
20+	6,688	1,052	7,740	99.8	98.3	99.6	99.0	119.8	101.6	na	na	na
All	31,196	2,790	33,986	99.9	98.8	99.8	102.3	123.6	103.9	na	na	na

na = Not applicable
[1] Both year and month of birth given
[2] $(B_m/B_f)x100$, where B_m and B_f are the numbers of male and female births, respectively
[3] $[2B_x/(B_{x-1}+B_{x+1})]x100$, where B_x is the number of births in calendar year x

Table C.5 Reporting of age at death in days

Distribution of reported deaths under 1 month of age by age at death in days and the percentage of neonatal deaths at ages 0-6 days, for five-year periods of birth preceding the survey (weighted), Cambodia 2014

Age at death	Number of years preceding the survey				Total
(days)	0-4	5-9	10-14	15-19	0-19
<1	40	73	56	51	220
1	35	30	30	19	114
2	14	11	5	7	37
3	5	16	20	18	60
4	1	3	8	2	15
5	0	3	3	5	10
6	6	4	5	4	19
7	11	19	16	23	69
8	1	1	0	5	7
9	0	0	2	2	4
10	5	2	3	5	15
11	1	0	0	0	1
12	0	4	0	0	5
13	0	0	0	0	0
14	0	5	1	3	10
15	2	6	9	9	27
16	0	0	0	2	2
17	0	0	0	3	3
18	0	1	1	2	3
20	2	2	3	5	12
21	0	1	0	1	2
23	0	0	2	0	2
25	0	1	2	0	3
27	0	0	0	3	3
28	0	1	0	1	1
29	0	0	0	1	1
30	0	0	2	0	2
Total 0-30	123	184	168	171	646
Percentage of early neonatal death[1]	81.7	76.0	75.5	62.8	73.5

[1] 0-6 days/0-30 days

Table C.6 Reporting of age at death in months

Distribution of reported deaths under age 2 by age at death in months and the percentage of infant deaths under age of 1 month, for five-year periods of birth preceding the survey, Cambodia 2014

Age at death (months)	Number of years preceding the survey				Total
	0-4	5-9	10-14	15-19	0-19
<1[a]	123	184	168	171	646
1	22	52	66	82	221
2	16	36	40	68	160
3	5	38	48	58	148
4	3	16	21	28	67
5	4	9	17	18	48
6	5	7	16	27	54
7	0	6	19	14	39
8	2	5	12	21	40
9	1	7	5	11	24
10	2	5	6	7	20
11	2	2	4	3	11
12	4	9	12	15	40
13	0	1	4	3	7
14	2	0	4	1	7
15	0	4	1	0	5
16	2	2	1	2	7
17	0	0	0	2	2
18	0	6	2	3	11
19	0	2	0	1	3
20	1	2	0	3	5
22	0	0	0	1	1
24+	0	0	0	1	1
1 Year	2	10	4	5	21
Total 0-11	185	366	421	507	1,479
Percentage of neonatal death[1]	66.5	50.3	40.1	33.7	43.7

[a] Includes deaths under one month reported in days
[1] Under one month/under one year

Table C.7 Nutritional status of children based on the NCHS/CDC/WHO International Reference Population

Percentage of children under age 5 classified as malnourished according to three anthropometric indices of nutritional status: height-for-age, weight-for-height, and weight-for-age, by background characteristics, based on the NCHS/CDC/WHO International Reference Population, Cambodia 2014

	Height-for-age[1]			Weight-for-height				Weight-for-age				
Background characteristic	Percentage below -3 SD	Percentage below -2 SD[2]	Mean Z-score (SD)	Percentage below -3 SD	Percentage below -2 SD[2]	Percentage above +2 SD	Mean Z-score (SD)	Percentage below -3 SD	Percentage below -2 SD[2]	Percentage above +2 SD	Mean Z-score (SD)	Number of children
**Age in months**												
<6	0.6	7.3	(0.2)	1.4	7.1	6.3	0.0	0.5	2.6	3.0	(0.0)	420
6-8	0.2	7.5	(0.6)	1.4	2.1	4.9	(0.1)	0.0	6.2	2.2	(0.6)	250
9-11	2.5	14.0	(0.9)	0.6	10.4	2.4	(0.7)	3.3	23.5	0.5	(1.3)	222
12-17	5.7	29.6	(1.4)	2.3	11.1	3.2	(0.8)	5.4	35.1	0.7	(1.5)	516
18-23	9.9	34.0	(1.5)	2.0	16.0	0.4	(1.0)	6.0	32.0	0.0	(1.6)	543
24-35	6.9	24.1	(1.2)	1.2	8.3	1.0	(0.9)	5.4	36.1	0.9	(1.5)	1,013
36-47	7.9	32.5	(1.5)	1.1	8.6	0.5	(0.9)	6.5	37.1	0.3	(1.7)	979
48-59	8.6	33.6	(1.6)	0.6	7.4	0.4	(0.9)	4.6	34.2	0.3	(1.6)	939
**Sex**												
Male	6.4	26.2	(1.3)	1.6	8.9	1.8	(0.8)	4.2	28.8	0.8	(1.4)	2,492
Female	6.7	27.0	(1.3)	0.9	9.2	1.6	(0.8)	5.3	32.0	0.8	(1.4)	2,392
**Birth interval in months[3]**												
First birth[4]	5.6	24.8	(1.3)	1.2	8.6	1.8	(0.8)	3.2	30.5	0.7	(1.4)	1,672
<24	9.0	34.6	(1.4)	1.9	8.7	2.1	(0.9)	8.8	39.1	1.0	(1.6)	337
24-47	7.3	28.8	(1.3)	1.1	9.0	1.7	(0.8)	6.5	32.0	1.0	(1.4)	1,103
48+	5.7	23.5	(1.2)	1.7	9.7	1.5	(0.8)	3.9	27.6	0.7	(1.4)	1,138
**Size at birth[3]**												
Very small	15.0	58.8	(2.0)	2.2	23.8	4.1	(1.0)	17.5	58.1	2.6	(2.0)	94
Small	11.5	33.0	(1.6)	1.1	13.8	0.7	(1.0)	7.4	44.2	0.0	(1.7)	367
Average or larger	5.6	24.7	(1.2)	1.4	8.1	1.8	(0.8)	4.1	28.8	0.8	(1.4)	3,763
**Mother's interview status**												
Interviewed	6.3	26.3	(1.3)	1.4	9.0	1.7	(0.8)	4.7	30.8	0.8	(1.4)	4,250
Not interviewed but in household	3.7	31.7	(1.2)	0.0	11.9	6.6	(0.6)	3.6	27.0	3.3	(1.2)	108
Not interviewed and not in the household[5]	8.8	27.6	(1.3)	0.8	8.1	0.2	(0.8)	5.3	27.4	0.2	(1.4)	525
**Mother's nutritional status[6]**												
Thin (BMI <18.5)	9.1	39.6	(1.7)	1.5	16.0	0.7	(1.1)	11.7	49.2	0.4	(1.9)	462
Normal (BMI 18.5-24.9)	6.5	26.0	(1.3)	1.4	8.2	2.1	(0.8)	4.0	30.8	0.8	(1.4)	2,722
Overweight/obese (BMI ≥25)	4.6	20.8	(1.1)	1.2	6.5	1.5	(0.7)	2.5	21.8	1.0	(1.3)	672
**Residence**												
Urban	3.6	19.6	(0.9)	0.8	6.7	2.3	(0.6)	2.9	20.5	2.2	(1.1)	671
Rural	7.0	27.7	(1.3)	1.3	9.4	1.6	(0.8)	5.1	31.9	0.6	(1.5)	4,213
**Province**												
Banteay Meanchey	5.2	21.9	(1.2)	0.6	8.2	0.0	(0.7)	4.7	23.0	0.0	(1.3)	241
Kampong Cham	6.1	27.5	(1.3)	0.8	8.7	2.1	(0.8)	3.9	30.5	0.8	(1.4)	686
Kampong Chhnang	9.6	35.7	(1.5)	2.4	10.9	0.7	(0.9)	4.7	43.4	0.0	(1.7)	172
Kampong Speu	7.4	34.1	(1.6)	1.9	10.0	0.9	(0.9)	7.2	38.9	0.0	(1.7)	317
Kampong Thom	7.0	32.2	(1.2)	1.7	11.6	2.9	(0.9)	8.5	34.5	1.4	(1.4)	214
Kandal	2.7	19.6	(1.2)	2.4	11.1	1.2	(0.9)	3.9	36.1	0.5	(1.5)	299
Kratie	7.8	28.6	(1.4)	1.0	8.0	0.0	(0.9)	5.1	31.3	0.0	(1.5)	180
Phnom Penh	2.7	14.0	(0.8)	0.3	6.3	3.4	(0.5)	3.5	19.8	3.9	(0.9)	391
Prey Veng	5.7	26.6	(1.3)	0.6	6.2	1.2	(0.7)	3.7	26.0	0.8	(1.4)	381
Pursat	13.9	33.8	(1.6)	2.7	10.0	4.3	(0.7)	8.1	39.8	0.7	(1.5)	200
Siem Reap	10.6	29.3	(1.4)	1.6	9.1	1.1	(0.8)	6.7	34.1	0.4	(1.5)	325
Svay Rieng	5.7	25.6	(1.3)	2.7	8.1	2.6	(0.8)	5.0	28.9	0.5	(1.4)	190
Takeo	5.2	23.2	(1.1)	3.3	17.2	1.0	(0.9)	2.7	28.8	0.5	(1.4)	261
Otdar Meanchey	11.0	35.1	(1.4)	2.6	11.8	4.6	(0.7)	6.8	34.2	0.0	(1.4)	76
Battambang/Pailin	3.3	20.7	(1.1)	0.3	5.6	0.7	(0.7)	2.1	24.9	0.5	(1.3)	389
Kampot/Kep	5.7	23.0	(1.2)	0.2	7.3	2.5	(0.8)	2.3	24.9	1.0	(1.4)	193
Preah Sihanouk/ Koh Kong	6.1	28.4	(1.3)	0.3	8.9	0.9	(0.6)	4.8	25.3	0.0	(1.3)	104
Preah Vihear/ Stung Treng	10.7	39.2	(1.6)	0.2	12.4	1.9	(0.8)	8.2	35.3	0.4	(1.6)	141
Mondul Kiri/ Ratanak Kiri	12.4	34.2	(1.5)	0.3	5.9	0.6	(0.7)	6.6	31.9	0.3	(1.5)	125
**Mother's education**												
No education	10.3	31.8	(1.5)	2.1	11.7	1.6	(0.8)	7.7	34.9	1.0	(1.5)	583
Primary	6.5	28.5	(1.3)	1.2	8.6	1.7	(0.8)	5.0	31.8	0.5	(1.5)	2,386
Secondary and higher	4.4	21.3	(1.1)	1.4	9.0	1.8	(0.8)	3.1	28.6	0.9	(1.3)	1,275
**Wealth quintile**												
Lowest	11.2	34.7	(1.6)	1.3	10.9	0.9	(0.9)	7.4	38.8	0.2	(1.7)	1,179
Second	6.7	30.8	(1.5)	1.5	9.9	1.4	(0.8)	6.1	33.3	0.5	(1.5)	997
Middle	6.1	26.6	(1.3)	1.2	8.0	1.6	(0.8)	4.4	29.5	0.3	(1.4)	974
Fourth	5.0	23.2	(1.1)	1.5	9.4	2.0	(0.8)	3.3	28.9	0.5	(1.4)	845
Highest	2.3	14.1	(0.8)	0.8	6.2	2.8	(0.6)	1.6	18.3	2.5	(1.0)	888
Total	6.6	26.5	(1.3)	1.3	9.0	1.7	(0.8)	4.8	30.4	0.8	(1.4)	4,884

Note: Table is based on children who slept in the household the night before the interview. Each of the indices is expressed in standard deviation units (SD) from the median of the NCHS/CDC/WHO international reference population. Table is based on children with valid dates of birth (month and year) and valid measurement of both height and weight. Total includes 25 cases missing size at birth.

[1] Recumbent length is measured for children under age 2, or in the few cases when the age of the child ids unknown and the child is less than 85cm; standing height is measured for all other children to be consistent with Table 16.1.

[2] Includes children who are below -3 standard deviations (SD) from the international reference population median

[3] Excludes children whose mothers were not interviewed

[4] First born twins (triplets, etc.) are counted as first births because they do not have a previous birth interval

[5] Includes children whose mothers are deceased

[6] Excludes children whose mothers were not interviewed, children whose mothers were not weighed and measured, and children whose mothers are pregnant or gave birth within the preceding 2 months. Mother's nutritional status in terms of BMI (body mass index) is presented in Table 16.10.

[7] For women who are not interviewed, information is taken from the Household Questionnaire. Excludes children whose mothers are not listed in the Household Questionnaire.

PERSONS INVOLVED IN THE 2014 CAMBODIA DEMOGRAPHIC AND HEALTH SURVEY

Appendix **D**

EXECUTIVE COMMITTEE

H.E. Prof. Eng Huot
Dr. Lo Veasnakiry
Dr. Tung Rathavy
Dr. Derveeuw Marc
Ms. Rebecca Black
Mr. David Gordge
Ms. Rana Flowers
H.E. San Sy Than
H.E. Hor Darith
H.E. Ms. Hang Lina
H.E. Phoeuk Sarin
H.E. Chay Satia
Mr. Back Suik Hee
Mr. Izaki Hiroshi

TECHNICAL COMMITTEE

H.E. Ms. Hang Lina
H.E. Chay Satia
Mr. Sok Kosal
Mr. They Kheam
Mr. Phan Chinda
Mr. Saint Lundy
Mr. Chhuon Sothy
Mr. Sok Borith
Mr. Srey Nimol
Mr. Try Mengseang
Mr. Som Bony
Mr. Nit Sarun
Mr. Sam Saroeurn
Mr. Long Lok
Mr. Yi Soktha
Mr. Soun Veasna
Mr. Chap Rathana
Dr. Lo Veasnakiry
Dr. Tung Rathavy
Dr. Prak Sophoannary
Dr. Loun Mondol
Dr. Lam Phirun
Dr. York Dararith
Dr. Thiep Chanthan
Ms. Taing Sokun Phalla
Ms. Leng Monipha
Dr. Chak Chantha
Mr. Arnaud Laillou
Dr. Rathavuth Hong
Representative, AusAID
Representative, WHO
Representative, KOICA
Other Partners

SURVEY DIRECTORS

H.E. Ms. Hang Lina
Dr. Tung Rathavy

ASSISTANT SURVEY DIRECTORS

H.E. Chay Satia
Dr. Prak Sophoannary

SURVEY COORDINATORS

Mr. Sok Kosal
Mr. Phan Chinda
Dr. Loun Mondol
Dr. Lam Phirun

SAMPLING COORDINATORS

Mr. They Kheam
Mr. Sok Borith

ASSISTANT SAMPLING COORDINATORS

Mr. Som Bony
Mr. Nit Sarun

TEAM LEADERS

Mr. Thong Vandeth
Mr. Sin Sereivuth
Ms. Lay Nareth
Mr. Mar Sothar
Mr. Sinn Samnang
Mr. Chan Tara
Mr. In Kimsea
Mr. San Sophat
Mr. Duch Chamrourn
Mr. Cheam Sokha
Ms. Chuon Soda
Mr. Sok Chantha
Mr. Kang Vivath
Mr. Yi Rith
Mr. In Vanorin
Mr. Tom Roeun
Mr. Vong Vuthy
Mr. Det Chamnan
Mr. Sok Borith

FIELD EDITORS

Ms. Nau Puthmakkara
Ms. Ros Thoravy
Ms. Chhun Navy
Ms. Ouk Samean
Ms. Long Leakhena
Ms. Prum Sotha
Ms. Pang Sophany
Ms. Chea Chanthou
Ms. Thong Somaly
Ms. Bou Bundol
Ms. Hun Ley
Ms. Sambath Sorphae
Ms. Hout Soklim
Ms. Kang Sreymom
Ms. Seng Chantheara
Ms. Thok Kimrot
Ms. Ly Nary
Ms. Pheach Borin
Ms. Hun Kamsan

INTERVIEWERS

Ms. Sem Chanthorn
Ms. Thuy Sokha
Ms. Tann Bunary
Mr. Ros Punlork
Ms. Moung Sopheap
Ms. Oung Kaknika
Ms. Long Pharvy
Mr. Seang Darot
Ms. Ou Sophina
Ms. Phlong Sam Oy
Ms. Som Thitha
Mr. Neang Vannrasmey
Ms. Dy Sor
Ms. Chun Ry
Ms. Chhearv chantheary
Mr. Soun Roth
Ms. Phich Somlida
Ms. Khoeum Sokim
Ms. Kong Sarom
Mr. Moeurng Tha
Ms. Chean Neikearng
Ms. Ngeth Sereiroth
Ms. So Samphasvichhka
Mr. Chap Sovannmakara
Ms. Bun Sytho
Ms. Pol Sophea
Ms. Im Sothea
Mr. Lay Sopheap
Ms. Mao Sophon
Ms. Sron Srei On
Ms. Seng Vutha
Mr. Roth Eileven
Ms. Keo Vitheavy
Ms. Samreth Srey
Ms. Cheap Yeang
Mr. Meth Sorphorn
Ms. Chheng Phannary
Ms. Hey Chhorlida

Ms. Vuth Sokhun
Mr. Leang Hour
Ms. Sor Phidor
Ms. Ly Chhengchunn
Ms. Sam Charkrya
Mr. Morn Simun
Ms. Orn Savoeurn
Ms. Som Sariem
Ms. Oun Leakena
Mr. Samean Chanmanith
Ms. Phorn Sokha
Ms. Yin Ngounmorakot
Ms. Touch Samphos
Mr. Phann Sochetra
Ms. Kheuiv Sokheng
Ms. Chea Samphas
Ms. Ky Kimtorng
Mr. Touch Minaer
Ms. Prak Sophalleap
Ms. Vann Mardeth
Ms. Som Sreina
Mr. Chan Niphol
Ms. Ung Nimol
Ms. Tum Tevy
Ms. Chhoun Sereiroth
Mr. Sous Saiy
Ms. Phoung Vichany
Ms. Mao Kolthida
Ms. Ty Rina
Mr. Hor Chandara
Ms. Yours Solida
Ms. Yoeum Sophanna
Ms. Gneh Kunthea
Mr. Chea Eng
Ms. Chin Sreineang
Ms. Doung Chaokarm
Ms. Sa Soknart
Mr. Moeurng Larv

EDITING STAFF

Ms. Chum Puthivan
Mr. Nit Sarun
Ms. Ma Sreyka
Ms. Khuev Madary
Ms. Chan Lekhena
Ms. Chay Dala
Ms. Kry Sopheap
Ms. Meas Linmoniroth
Ms. Meas Rathmony
Mr. Sok Phal

DATA PROCESSING COORDINATOR

Mr. Saint Lundy

ASSISTANT DATA PROCESSING COORDINATORS

Mr. Chao Pheav
Mr. Lay Sophat

DATA PROCESSING STAFF

Mr. Oukchay Panharith
Mrs. Phang Sokmean
Ms. Ros Bundane
Mrs. Meas Linsaosanvar
Ms. Srou Soreirath
Ms. Nou Thavy
Mrs. Nay Seyha
Ms. Po Daly
Ms. Bunny Sonin
Mr. Ly Sophanith
Mr. Chan Mos
Mr. Moeng Tityaroat
Ms. Kim Chanvechhecka
Mrs. Chea Narun
Ms. Nhip Srey Toch
Mr. Eng Eangmeng
Mr. Nul Pheakdeybot
Mrs. Chan Elen
Ms. Ten Sovannary
Mr. Heng Rattanak
Mr. Pen Boran
Mr. Nou Chanthy
Mr. Sin Ranan
Mr. Cheat Sokmeng
Ms. Nheb Reak Smey
Mr. Seng Sovannarith
Mr. Bean Rotha
Mrs. SoeuSamet
Mr. Oeun Chamrern

ACCOUNTANTS

Mr. Chhoun Sothy
Mr. Try Mengseang

ADMINISTRATIVE STAFF

Mr. Try Mengseang
Mr. Chap Ratana

ICF INTERNATIONAL STAFF

Rathavuth Hong
Bernard Barrère
Ruilin Ren
Blake Zakary
Kia Reinis
Abraham Sene
Mianmian Yu
Trevor Croft
Anne Cross
Bunsoth Mao
Sarah Balian
Matt Pagan
Nancy Johnson
Christopher Gramer
Greg Edmondson

QUESTIONNAIRES *Appendix* E

CAMBODIA DEMOGRAPHIC AND HEALTH SURVEYS 2014
HOUSEHOLD QUESTIONNAIRE

MINISTRY OF PLANNING
NATIONAL INSTITUTE OF STATISTICS

MINISTRY OF HEALTH
DIRECTORATE FOR GENERAL HEALTH

IDENTIFICATION

DOMAIN	☐☐
NAME OF HOUSEHOLD HEAD ______	
PROVINCE ______	☐☐
DISTRICT ______	☐☐
COMMUNE ______	☐☐
VILLAGE ______	☐☐
CLUSTER NUMBER	☐☐☐
HOUSEHOLD NUMBER	☐☐
HOUSEHOLD IS IN THE CLUSTER SELECTED FOR MICRONUTRIENT TEST	YES = 1<br>NO = 2 ☐
HOUSEHOLD SELECTED FOR MALE INTERVIEW AND DOMESTIC VIOLENCE OF THE WOMEN QUESTIONNAIRE	YES = 1<br>NO = 2 ☐
HOUSEHOLD SELECTED FOR ANEMIA TEST AND ANTHROPOMETRIC MEASUREMENTS	YES = 1<br>NO = 2 ☐

INTERVIEWER VISITS

	1	2	3	FINAL VISIT
DATE	______	______	______	DAY ☐☐<br>MONTH ☐☐<br>YEAR 2 0 ☐☐
INTERVIEWER'S NAME	______	______	______	INT. NUMBER ☐☐☐
RESULT*	______	______	______	RESULT* ☐
NEXT VISIT: DATE	______	______		TOTAL NUMBER OF VISITS ☐
TIME	______	______		

*RESULT CODES:
1 COMPLETED
2 NO HOUSEHOLD MEMBER AT HOME OR NO COMPETENT RESPONDENT AT HOME AT TIME OF VISIT
3 ENTIRE HOUSEHOLD ABSENT FOR EXTENDED PERIOD OF TIME
4 POSTPONED
5 REFUSED
6 DWELLING VACANT OR ADDRESS NOT A DWELLING
7 DWELLING DESTROYED
8 DWELLING NOT FOUND
9 OTHER ______
(SPECIFY)

TOTAL PERSONS IN HOUSEHOLD ☐☐

TOTAL ELIGIBLE WOMEN ☐☐

TOTAL ELIGIBLE MEN ☐☐

LINE NO. OF RESPONDENT TO HOUSEHOLD QUESTIONNAIRE ☐☐

SUPERVISOR	FIELD EDITOR	OFFICE EDITOR	KEYED BY
NAME ______ ☐☐☐<br>DATE: ______	NAME ______ ☐☐☐<br>DATE: ______	☐☐	☐☐

THIS PAGE IS INTENTIONALLY BLANK

INTRODUCTION AND CONSENT

Hello. My name is ____. I am working with the ministry of health and ministry of planning. We are conducting a survey about health all over Cambodia. The information we collect will help the government to plan health services. Your household was selected for the survey. I would like to ask you some questions about your household. The questions usually take about 20 to 25 minutes. All of the answers you give will be confidential and will not be shared with anyone other than members of our survey team. You don't have to be in the survey, but we hope you will agree to answer the questions since your views are important. If I ask you any question you don't want to answer, just let me know and I will go on to the next question or you can stop the interview at any time.
In case you need more information about the survey, you may contact the person listed on this card.

GIVE CARD WITH CONTACT INFORMATION

Do you have any questions?
May I begin the interview now?

SIGNATURE OF INTERVIEWER: ________________________ DATE: ________________

RESPONDENT AGREES TO BE INTERVIEWED . . . 1 ↓ RESPONDENT DOES NOT AGREE TO BE INTERVIEWED . . . 2 → END

HOUSEHOLD SCHEDULE

							IF AGE 15 OR OLDER			
LINE NO.	USUAL RESIDENTS AND VISITORS	RELATIONSHIP TO HEAD OF HOUSEHOLD	SEX	RESIDENCE		AGE	MARITAL STATUS	ELIGIBILITY		
**1**	**2**	**3**	**4**	**5**	**6**	**7**	**8**	**9**	**10**	**11**
	Please give me the names of the persons who usually live in your household and guests of the household who stayed here last night, starting with the head of the household.<br><br>AFTER LISTING THE NAMES AND RECORDING THE RELATIONSHIP AND SEX FOR EACH PERSON, ASK QUESTIONS 2A-2C TO BE SURE THAT THE LISTING IS COMPLETE.<br><br>THEN ASK APPROPRIATE QUESTIONS IN COLUMNS 5-20 FOR EACH PERSON.	What is the relationship of (NAME) to the head of the household?<br><br>SEE CODES BELOW.	Is (NAME) male or female?	Does (NAME) usually live here?	Did (NAME) stay here last night?	How old is (NAME)?<br><br>IF 95 OR MORE, RECORD '95'.	What is (NAME)'s current marital status?<br><br>1 = MARRIED OR LIVING TOGETHER<br>2 = DIVORCED/ SEPARATED<br>3 = WIDOWED<br>4 = NEVER-MARRIED AND NEVER LIVED TOGETHER	CIRCLE LINE NUMBER OF ALL WOMEN AGE 15-49	CIRCLE LINE NUMBER OF ALL MEN AGE 15-49	CIRCLE LINE NUMBER OF ALL CHILDREN AGE 0-5
			M F	Y N	Y N	IN YEARS				
01		☐☐	1 2	1 2	1 2	☐☐	☐	01	01	01
02		☐☐	1 2	1 2	1 2	☐☐	☐	02	02	02
03		☐☐	1 2	1 2	1 2	☐☐	☐	03	03	03
04		☐☐	1 2	1 2	1 2	☐☐	☐	04	04	04
05		☐☐	1 2	1 2	1 2	☐☐	☐	05	05	05
06		☐☐	1 2	1 2	1 2	☐☐	☐	06	06	06
07		☐☐	1 2	1 2	1 2	☐☐	☐	07	07	07
08		☐☐	1 2	1 2	1 2	☐☐	☐	08	08	08
09		☐☐	1 2	1 2	1 2	☐☐	☐	09	09	09
10		☐☐	1 2	1 2	1 2	☐☐	☐	10	10	10

CODES FOR Q. 3: RELATIONSHIP TO HEAD OF HOUSEHOLD

01 = HEAD	08 = BROTHER OR SISTER
02 = WIFE OR HUSBAND	09 = OTHER RELATIVE
03 = SON OR DAUGHTER	10 = ADOPTED/FOSTER/ STEPCHILD
04 = SON-IN-LAW OR DAUGHTER-IN-LAW	11 = NOT RELATED
05 = GRANDCHILD	98 = DON'T KNOW
06 = PARENT	
07 = PARENT-IN-LAW	

	IF AGE 0-17 YEARS				IF AGE 3 YEARS OR OLDER		IF AGE 3-24 YEARS		IF AGE 0-4 YEARS
LINE NO.	SURVIVORSHIP AND RESIDENCE OF BIOLOGICAL PARENTS				EVER ATTENDED SCHOOL		CURRENT/RECENT SCHOOL ATTENDANCE		BIRTH REGIS-TRATION
	**12**	**13**	**14**	**15**	**16**	**17**	**18**	**19**	**20**
	Is (NAME)'s natural mother alive?	Does (NAME)'s natural mother usually live in this household or was she a guest last night?<br><br>IF YES: What is her name? RECORD MOTHER'S LINE NUMBER.<br><br>IF NO, RECORD '00'.	Is (NAME)'s natural father alive?	Does (NAME)'s natural father usually live in this household or was he a guest last night?<br><br>IF YES: What is his name? RECORD FATHER'S LINE NUMBER.<br><br>IF NO, RECORD '00'.	Has (NAME) ever attended school?	What is the highest level of school (NAME) has attended?<br><br>SEE CODES BELOW.<br><br>What is the highest grade (NAME) completed at that level?<br><br>SEE CODES BELOW.	Did (NAME) attend school at any time during the (2013-2014) school year?	During this/that school year, what level and grade [is/was] (NAME) attending?<br><br>SEE CODES BELOW.	Does (NAME) have a birth certificate?<br><br>IF NO, PROBE: Has (NAME)'s birth ever been registered with the civil authority?<br><br>1 = HAS CERTIFICATE<br>2 = REGISTERED<br>3 = NEITHER<br>8 = DON'T KNOW
	Y N DK		Y N DK		Y N	LEVEL GRADE	Y N	LEVEL GRADE	
01	1 2 8 (2 → GO TO 14)	☐☐	1 2 8 (2 → GO TO 16)	☐☐	1 2 (2 → GO TO 20)	☐ ☐☐	1 2 (2 → GO TO 20)	☐ ☐☐	☐
02	1 2 8 (2 → GO TO 14)	☐☐	1 2 8 (2 → GO TO 16)	☐☐	1 2 (2 → GO TO 20)	☐ ☐☐	1 2 (2 → GO TO 20)	☐ ☐☐	☐
03	1 2 8 (2 → GO TO 14)	☐☐	1 2 8 (2 → GO TO 16)	☐☐	1 2 (2 → GO TO 20)	☐ ☐☐	1 2 (2 → GO TO 20)	☐ ☐☐	☐
04	1 2 8 (2 → GO TO 14)	☐☐	1 2 8 (2 → GO TO 16)	☐☐	1 2 (2 → GO TO 20)	☐ ☐☐	1 2 (2 → GO TO 20)	☐ ☐☐	☐
05	1 2 8 (2 → GO TO 14)	☐☐	1 2 8 (2 → GO TO 16)	☐☐	1 2 (2 → GO TO 20)	☐ ☐☐	1 2 (2 → GO TO 20)	☐ ☐☐	☐
06	1 2 8 (2 → GO TO 14)	☐☐	1 2 8 (2 → GO TO 16)	☐☐	1 2 (2 → GO TO 20)	☐ ☐☐	1 2 (2 → GO TO 20)	☐ ☐☐	☐
07	1 2 8 (2 → GO TO 14)	☐☐	1 2 8 (2 → GO TO 16)	☐☐	1 2 (2 → GO TO 20)	☐ ☐☐	1 2 (2 → GO TO 20)	☐ ☐☐	☐
08	1 2 8 (2 → GO TO 14)	☐☐	1 2 8 (2 → GO TO 16)	☐☐	1 2 (2 → GO TO 20)	☐ ☐☐	1 2 (2 → GO TO 20)	☐ ☐☐	☐
09	1 2 8 (2 → GO TO 14)	☐☐	1 2 8 (2 → GO TO 16)	☐☐	1 2 (2 → GO TO 20)	☐ ☐☐	1 2 (2 → GO TO 20)	☐ ☐☐	☐
10	1 2 8 (2 → GO TO 14)	☐☐	1 2 8 (2 → GO TO 16)	☐☐	1 2 (2 → GO TO 20)	☐ ☐☐	1 2 (2 → GO TO 20)	☐ ☐☐	☐

CODES FOR Qs. 17 AND 19: EDUCATION

LEVEL	GRADE	
1 = PRIMARY	(01-06 FOR GRADE 1-6)	00 = LESS THAN 1 YEAR COMPLETED (USE '00' FOR Q. 17 ONLY. THIS CODE IS NOT ALLOWED FOR Q. 19)
2 = LOWER SECONDARY	(07-09 FOR GRADE 7-9)	
3 = UPPER SECONDARY	(10-12 FOR GRADE 10-12)	
4 = HIGHER	(01-08 FOR YEAR 1-8)	
5 = PRE-PRIMARY	(00 FOR ANY YEAR)	98 = DON'T KNOW
8 = DON'T KNOW		

DISABILITY

	IF AGE 5 YEARS OR OLDER					
LINE NO.	PROBLEM OF VISION	PROBLEM OF HEARING	PROBLEM OF WALKING OR CLIMBING	PROBLEM OF REMEMBERING OR CONCENTRATING	PROBLEM WITH SELF-CARE	PROBLEM OF COMMUNICATING
	21	22	23	24	25	26
	Does [NAME] have difficulty seeing, even if wearing glasses<br><br>IF NO, CIRCLE "1"<br>IF YES, PROB:<br>With some difficulty?<br>IF YES CIRCLE "2"<br>With a lot of difficulty?<br>IF YES CIRCLE "3"<br>Cannot see at all?<br>IF YES CIRCLE "4"<br>IF DON'T KNOW<br>CIRCLE :"5"	Does [NAME] have difficulty hearing, even if using a hearing aid?<br><br>IF NO, CIRCLE "1"<br>IF YES, PROB:<br>With some difficulty?<br>IF YES CIRCLE "2"<br>With a lot of difficulty?<br>IF YES CIRCLE "3"<br>Cannot hear at all?<br>IF YES CIRCLE "4"<br>IF DON'T KNOW<br>CIRCLE :"5"	Does [NAME] have difficulty walking or climbing steps?<br><br>IF NO, CIRCLE "1"<br>IF YES, PROB:<br>With some difficulty?<br>IF YES CIRCLE "2"<br>With a lot of difficulty?<br>IF YES CIRCLE "3"<br>Cannot walk or climb steps at all?<br>IF YES CIRCLE "4"<br>IF DON'T KNOW<br>CIRCLE :"5"	Does [NAME] have difficulty remembering or concentrating?<br><br>IF NO, CIRCLE "1"<br>IF YES, PROB:<br>With some difficulty?<br>IF YES CIRCLE "2"<br>With a lot of difficulty?<br>IF YES CIRCLE "3"<br>Cannot remember or concentrate at all?<br>IF YES CIRCLE "4"<br>IF DON'T KNOW<br>CIRCLE :"5"	Does [NAME] have difficulty with self-care such as washing all over or dressing?<br><br>IF NO, CIRCLE "1"<br>IF YES, PROB:<br>With some difficulty?<br>IF YES CIRCLE "2"<br>With a lot of difficulty?<br>IF YES CIRCLE "3"<br>Cannot do at all?<br>IF YES CIRCLE "4"<br>IF DON'T KNOW<br>CIRCLE :"5"	Because of a physical, mental or emotional health condition, does [NAME] have difficulty communicating, (for example understanding others or others understanding him/her?<br><br>IF NO, CIRCLE "1"<br>IF YES, PROB:<br>With some difficulty?<br>IF YES CIRCLE "2"<br>With a lot of difficulty?<br>IF YES CIRCLE "3"<br>Cannot communicating at all?<br>IF YES CIRCLE "4"<br>IF DON'T KNOW<br>CIRCLE :"5"
	N YS YA YT DK	N YS YA YT DK	N YS YA YT DK	N YS YA YT DK	N YS YA YT DK	N YS YA YT DK
01	1 2 3 4 5	1 2 3 4 5	1 2 3 4 5	1 2 3 4 5	1 2 3 4 5	1 2 3 4 5
02	1 2 3 4 5	1 2 3 4 5	1 2 3 4 5	1 2 3 4 5	1 2 3 4 5	1 2 3 4 5
03	1 2 3 4 5	1 2 3 4 5	1 2 3 4 5	1 2 3 4 5	1 2 3 4 5	1 2 3 4 5
04	1 2 3 4 5	1 2 3 4 5	1 2 3 4 5	1 2 3 4 5	1 2 3 4 5	1 2 3 4 5
05	1 2 3 4 5	1 2 3 4 5	1 2 3 4 5	1 2 3 4 5	1 2 3 4 5	1 2 3 4 5
06	1 2 3 4 5	1 2 3 4 5	1 2 3 4 5	1 2 3 4 5	1 2 3 4 5	1 2 3 4 5
07	1 2 3 4 5	1 2 3 4 5	1 2 3 4 5	1 2 3 4 5	1 2 3 4 5	1 2 3 4 5
08	1 2 3 4 5	1 2 3 4 5	1 2 3 4 5	1 2 3 4 5	1 2 3 4 5	1 2 3 4 5
09	1 2 3 4 5	1 2 3 4 5	1 2 3 4 5	1 2 3 4 5	1 2 3 4 5	1 2 3 4 5
10	1 2 3 4 5	1 2 3 4 5	1 2 3 4 5	1 2 3 4 5	1 2 3 4 5	1 2 3 4 5

N= **N**O
YS= **Y**ES **S**OME DIFFICULTY
YA= **Y**ES **A** LOT OF DIFFICULTY
YT= **Y**ES CANNOT … AT ALL (**T**OTALLY)
DK= **D**ON'T **K**NOW

							IF AGE 15 OR OLDER			
LINE NO.	USUAL RESIDENTS AND VISITORS	RELATIONSHIP TO HEAD OF HOUSEHOLD	SEX	RESIDENCE		AGE	MARITAL STATUS	ELIGIBILITY		
**1**	**2**	**3**	**4**	**5**	**6**	**7**	**8**	**9**	**10**	**11**
	Please give me the names of the persons who usually live in your household and guests of the household who stayed here last night, starting with the head of the household.<br><br>AFTER LISTING THE NAMES AND RECORDING THE RELATIONSHIP AND SEX FOR EACH PERSON, ASK QUESTIONS 2A-2C TO BE SURE THAT THE LISTING IS COMPLETE.<br><br>THEN ASK APPROPRIATE QUESTIONS IN COLUMNS 5-20 FOR EACH PERSON.	What is the relationship of (NAME) to the head of the household?<br><br>SEE CODES BELOW.	Is (NAME) male or female?	Does (NAME) usually live here?	Did (NAME) stay here last night?	How old is (NAME)?<br><br>IF 95 OR MORE, RECORD '95'.	What is (NAME)'s current marital status?<br><br>1 = MARRIED OR LIVING TOGETHER<br>2 = DIVORCED/ SEPARATED<br>3 = WIDOWED<br>4 = NEVER-MARRIED AND NEVER LIVED TOGETHER	CIRCLE LINE NUMBER OF ALL WOMEN AGE 15-49	CIRCLE LINE NUMBER OF ALL MEN AGE 15-49	CIRCLE LINE NUMBER OF ALL CHILDREN AGE 0-5
			M F	Y N	Y N	IN YEARS				
11		☐☐	1 2	1 2	1 2	☐☐	☐	11	11	11
12		☐☐	1 2	1 2	1 2	☐☐	☐	12	12	12
13		☐☐	1 2	1 2	1 2	☐☐	☐	13	13	13
14		☐☐	1 2	1 2	1 2	☐☐	☐	14	14	14
15		☐☐	1 2	1 2	1 2	☐☐	☐	15	15	15
16		☐☐	1 2	1 2	1 2	☐☐	☐	16	16	16
17		☐☐	1 2	1 2	1 2	☐☐	☐	17	17	17
18		☐☐	1 2	1 2	1 2	☐☐	☐	18	18	18
19		☐☐	1 2	1 2	1 2	☐☐	☐	19	19	19
20		☐☐	1 2	1 2	1 2	☐☐	☐	20	20	20

TICK HERE IF CONTINUATION SHEET USED ☐

2A) Just to make sure that I have a complete listing. Are there any other persons such as small children or infants that we have not listed? YES ☐ → ADD TO TABLE NO ☐

2B) Are there any other people who may not be members of your family, such as domestic servants, lodgers, or friends who usually live here? YES ☐ → ADD TO TABLE NO ☐

2C) Are there any guests or temporary visitors staying here, or anyone else who stayed here last night, who have not been listed? YES ☐ → ADD TO TABLE NO ☐

CODES FOR Q. 3: RELATIONSHIP TO HEAD OF HOUSEHOLD

01 = HEAD	08 = BROTHER OR SISTER
02 = WIFE OR HUSBAND	09 = OTHER RELATIVE
03 = SON OR DAUGHTER	10 = ADOPTED/FOSTER/ STEPCHILD
04 = SON-IN-LAW OR DAUGHTER-IN-LAW	11 = NOT RELATED
05 = GRANDCHILD	98 = DON'T KNOW
06 = PARENT	
07 = PARENT-IN-LAW	

	IF AGE 0-17 YEARS				IF AGE 3 YEARS OR OLDER		IF AGE 3-24 YEARS		IF AGE 0-4 YEARS
LINE NO.	SURVIVORSHIP AND RESIDENCE OF BIOLOGICAL PARENTS				EVER ATTENDED SCHOOL		CURRENT/RECENT SCHOOL ATTENDANCE		BIRTH REGIS-TRATION
	**12**	**13**	**14**	**15**	**16**	**17**	**18**	**19**	**20**
	Is (NAME)'s natural mother alive?	Does (NAME)'s natural mother usually live in this household or was she a guest last night?<br><br>IF YES: What is her name? RECORD MOTHER'S LINE NUMBER.<br><br>IF NO, RECORD '00'.	Is (NAME)'s natural father alive?	Does (NAME)'s natural father usually live in this household or was he a guest last night?<br><br>IF YES: What is his name? RECORD FATHER'S LINE NUMBER.<br><br>IF NO, RECORD '00'.	Has (NAME) ever attended school?	What is the highest level of school (NAME) has attended?<br><br>SEE CODES BELOW.<br><br>What is the highest grade (NAME) completed at that level?<br><br>SEE CODES BELOW.	Did (NAME) attend school at any time during the (2013-2014) school year?	During this/that school year, what level and grade [is/was] (NAME) attending?<br><br>SEE CODES BELOW.	Does (NAME) have a birth certificate?<br><br>IF NO, PROBE: Has (NAME)'s birth ever been registered with the civil authority?<br><br>1 = HAS CERTIFICATE<br>2 = REGISTERED<br>3 = NEITHER<br>8 = DON'T KNOW
	Y N DK		Y N DK		Y N	LEVEL GRADE	Y N	LEVEL GRADE	
11	1 2 8 GO TO 14		1 2 8 GO TO 16		1 2 GO TO 20		1 2 GO TO 20		
12	1 2 8 GO TO 14		1 2 8 GO TO 16		1 2 GO TO 20		1 2 GO TO 20		
13	1 2 8 GO TO 14		1 2 8 GO TO 16		1 2 GO TO 20		1 2 GO TO 20		
14	1 2 8 GO TO 14		1 2 8 GO TO 16		1 2 GO TO 20		1 2 GO TO 20		
15	1 2 8 GO TO 14		1 2 8 GO TO 16		1 2 GO TO 20		1 2 GO TO 20		
16	1 2 8 GO TO 14		1 2 8 GO TO 16		1 2 GO TO 20		1 2 GO TO 20		
17	1 2 8 GO TO 14		1 2 8 GO TO 16		1 2 GO TO 20		1 2 GO TO 20		
18	1 2 8 GO TO 14		1 2 8 GO TO 16		1 2 GO TO 20		1 2 GO TO 20		
19	1 2 8 GO TO 14		1 2 8 GO TO 16		1 2 GO TO 20		1 2 GO TO 20		
20	1 2 8 GO TO 14		1 2 8 GO TO 16		1 2 GO TO 20		1 2 GO TO 20		

CODES FOR Qs. 17 AND 19: EDUCATION

LEVEL	GRADE	
1 = PRIMARY	(01-06 FOR GRADE 1-6)	00 = LESS THAN 1 YEAR COMPLETED
2 = LOWER SECONDARY	(07-09 FOR GRADE 7-9)	(USE '00' FOR Q. 17 ONLY.
3 = UPPER SECONDARY	(10-12 FOR GRADE 10-12)	THIS CODE IS NOT ALLOWED
4 = HIGHER	(01-08 FOR YEAR 1-8)	FOR Q. 19)
5 = PRE-PRIMARY	(00 FOR ANY YEAR)	98 = DON'T KNOW
8 = DON'T KNOW		

	IF AGE 5 YEARS OR OLDER					
LINE NO.	PROBLEM OF VISION	PROBLEM OF HEARING	PROBLEM OF WALKING OR CLIMBING	PROBLEM OF REMEMBERING OR CONCENTRATING	PROBLEM WITH SELF-CARE	PROBLEM OF COMMUNICATING
	21	22	23	24	25	26
	Does [NAME] have difficulty seeing, even if wearing glasses<br><br>IF NO, CIRCLE "1"<br>IF YES, PROB:<br>With some difficulty?<br>IF YES CIRCLE "2"<br>With a lot of difficulty?<br>IF YES CIRCLE "3"<br>Cannot see at all?<br>IF YES CIRCLE "4"<br>IF DON'T KNOW<br>CIRCLE :"5"	Does [NAME] have difficulty hearing, even if using a hearing aid?<br><br>IF NO, CIRCLE "1"<br>IF YES, PROB:<br>With some difficulty?<br>IF YES CIRCLE "2"<br>With a lot of difficulty?<br>IF YES CIRCLE "3"<br>Cannot hear at all?<br>IF YES CIRCLE "4"<br>IF DON'T KNOW<br>CIRCLE :"5"	Does [NAME] have difficulty walking or climbing steps?<br><br>IF NO, CIRCLE "1"<br>IF YES, PROB:<br>With some difficulty?<br>IF YES CIRCLE "2"<br>With a lot of difficulty?<br>IF YES CIRCLE "3"<br>Cannot walk or climb steps at all?<br>IF YES CIRCLE "4"<br>IF DON'T KNOW<br>CIRCLE :"5"	Does [NAME] have difficulty remembering or concentrating?<br><br>IF NO, CIRCLE "1"<br>IF YES, PROB:<br>With some difficulty?<br>IF YES CIRCLE "2"<br>With a lot of difficulty?<br>IF YES CIRCLE "3"<br>Cannot remember or concentrate at all?<br>IF YES CIRCLE "4"<br>IF DON'T KNOW<br>CIRCLE :"5"	Does [NAME] have difficulty with self-care such as washing all over or dressing?<br><br>IF NO, CIRCLE "1"<br>IF YES, PROB:<br>With some difficulty?<br>IF YES CIRCLE "2"<br>With a lot of difficulty?<br>IF YES CIRCLE "3"<br>Cannot do at all?<br>IF YES CIRCLE "4"<br>IF DON'T KNOW<br>CIRCLE :"5"	Because of a physical, mental or emotional health condition, does [NAME] have difficulty communicating, (for example understanding others or others understanding him/her?<br><br>IF NO, CIRCLE "1"<br>IF YES, PROB:<br>With some difficulty?<br>IF YES CIRCLE "2"<br>With a lot of difficulty?<br>IF YES CIRCLE "3"<br>Cannot communicating at all?<br>IF YES CIRCLE "4"<br>IF DON'T KNOW<br>CIRCLE :"5"
	N YS YA YT DK	N YS YA YT DK	N YS YA YT DK	N YS YA YT DK	N YS YA YT DK	N YS YA YT DK
11	1 2 3 4 5	1 2 3 4 5	1 2 3 4 5	1 2 3 4 5	1 2 3 4 5	1 2 3 4 5
12	1 2 3 4 5	1 2 3 4 5	1 2 3 4 5	1 2 3 4 5	1 2 3 4 5	1 2 3 4 5
13	1 2 3 4 5	1 2 3 4 5	1 2 3 4 5	1 2 3 4 5	1 2 3 4 5	1 2 3 4 5
14	1 2 3 4 5	1 2 3 4 5	1 2 3 4 5	1 2 3 4 5	1 2 3 4 5	1 2 3 4 5
15	1 2 3 4 5	1 2 3 4 5	1 2 3 4 5	1 2 3 4 5	1 2 3 4 5	1 2 3 4 5
16	1 2 3 4 5	1 2 3 4 5	1 2 3 4 5	1 2 3 4 5	1 2 3 4 5	1 2 3 4 5
17	1 2 3 4 5	1 2 3 4 5	1 2 3 4 5	1 2 3 4 5	1 2 3 4 5	1 2 3 4 5
18	1 2 3 4 5	1 2 3 4 5	1 2 3 4 5	1 2 3 4 5	1 2 3 4 5	1 2 3 4 5
19	1 2 3 4 5	1 2 3 4 5	1 2 3 4 5	1 2 3 4 5	1 2 3 4 5	1 2 3 4 5
20	1 2 3 4 5	1 2 3 4 5	1 2 3 4 5	1 2 3 4 5	1 2 3 4 5	1 2 3 4 5

N= **N**O
YS= **Y**ES **S**OME DIFFICULTY
YA= **Y**ES **A** LOT OF DIFFICULTY
YT= **Y**ES CANNOT … AT ALL (**T**OTALLY)
DK= **D**ON'T **K**NOW

NO.	QUESTIONS AND FILTERS		CODING CATEGORIES
50	Was any person of your household injured or killed in an accident in the past 12 months?<br><br>IF YES, PROBE: How many?		YES . . . . 1<br>HOW MANY . . . . [ ][ ]<br>NO . . . . 2<br>(GO TO 65)
51	What is the name of the person(s) injured or killed?<br>ENTER THE NAME OF EACH PERSON INJURED OR KILLED IN Q52.<br>IF THERE ARE MORE THAN TWO PEOPLE, USE AN ADDITIONAL QUESTIONNAIRE.		
52	NAME INJURED/KILLED	NAME ____________	NAME ____________
53	Could you tell me in what type of accident (NAME) was injured or killed?	LANDMINE/UNEXPLODED BOMB (UXO) . . . . 01<br>GUN SHOT/WEAPON . . . . 02<br>ROAD ACCIDENT . . . . 03<br>FIRE/BURNING . . . . 04<br>SNAKE/ANIMAL BITE . . . . 05<br>FALL FROM TREE/BUILDING . . . . 06<br>DROWNING . . . . 07<br>POISONING (CHEMICAL) . . . . 08<br>VIOLENCE/ASSULT . . . . 09<br>OTHER ____________ 96<br>SPECIFY<br>DON'T KNOW 98	LANDMINE/UNEXPLODED BOMB (UXO) . . . . 01<br>GUN SHOT/WEAPON . . . . 02<br>ROAD ACCIDENT . . . . 03<br>FIRE/BURNING . . . . 04<br>SNAKE/ANIMAL BITE . . . . 05<br>FALL FROM TREE/BUILDING . . . . 06<br>DROWNING . . . . 07<br>POISONING (CHEMICAL) . . . . 08<br>VIOLENCE/ASSULT . . . . 09<br>OTHER ____________ 96<br>SPECIFY<br>DON'T KNOW 98
54	Is (NAME) still alive?	YES . . . . 1<br>NO . . . . 2<br>(GO TO 57)	YES . . . . 1<br>NO . . . . 2<br>(GO TO 57)
55	In your opinion, was (NAME)'s injury serious, moderate, or slight?	SERIOUS . . . . 1<br>MODERATE . . . . 2<br>SLIGHT . . . . 3<br>DON'T KNOW 8	SERIOUS . . . . 1<br>MODERATE . . . . 2<br>SLIGHT . . . . 3<br>DON'T KNOW 8
56	IF ALIVE: RECORD LINE NUMBER FROM COLUMN (1).	LINE NUMBER . . . . [ ][ ]<br>(GO TO 58)	LINE NUMBER . . . . [ ][ ]<br>(GO TO 58)
57	Was (NAME)'s death due to the accident?	YES . . . . 1<br>NO 2	YES . . . . 1<br>NO 2
58		GO BACK TO 52 IN NEXT COLUMN; OR, IF NO OTHER PERSON, GO TO 65.	GO TO 52 IN NEXT COLUMN OF ADDITIONAL QUESTIONNAIRE; OR, IF NO OTHER PERSON, GO TO 65.

NO.	QUESTIONS AND FILTERS	CODING CATEGORIES
65	Please tell me if any member of your household is sick, has an illness or an injury now or at any time in the last 30 days?<br><br>IF YES, PROBE: How many?	YES . . . . 1<br>HOW MANY . . . . [ ][ ]<br><br>NO . . . . 2<br>(GO TO 101)
66	Now I would like to ask you some questions about each person who is sick/injured now or at any time in the last 30 days. Could you tell me his/her/their name(s)? Then we will talk about one person at a time.<br><br>ENTER THE LINE NUMBER AND NAME OF EACH PERSON SICK/INJURED. ASK ALL QUESTIONS ABOUT ALL OF THESE PEOPLE. (IF THERE ARE MORE THAN 3 PEOPLE, USE ADDITIONAL QUESTIONNAIRE).	

NO.	QUESTIONS AND FILTERS			
67	LINE NUMBER AND NAME FROM COL. (1) AND (2).	LINE NUMBER . . . . [ ][ ]<br><br>NAME ________________	LINE NUMBER . . . . [ ][ ]<br><br>NAME ________________	LINE NUMBER . . . . [ ][ ]<br><br>NAME ________________
68	In your opinion, was (NAME)'s illness/injury serious, moderate, or slight?	SERIOUS . . . . 1<br>MODERATE . . . . 2<br>SLIGHT . . . . 3<br>DON'T KNOW 8	SERIOUS . . . . 1<br>MODERATE . . . . 2<br>SLIGHT . . . . 3<br>DON'T KNOW 8	SERIOUS . . . . 1<br>MODERATE . . . . 2<br>SLIGHT . . . . 3<br>DON'T KNOW 8
69	Was advice or treatment sought for (NAME)'s illness/injury?	YES . . . . 1<br>NO . . . . 2<br>(SKIP TO NEXT COLUMN OR TO 101)	YES . . . . 1<br>NO . . . . 2<br>(SKIP TO NEXT COLUMN OR TO 101)	YES . . . . 1<br>NO . . . . 2<br>(SKIP TO NEXT COLUMN OR TO 101)
70	Where was advice or treatment first sought for (NAME)'s illness/ injury?<br><br>IF "HOSPITAL", PROBE: Do you mean a permanent building where health workers are present every day?<br><br>IF "YES": Was it a Provincial Hospital, District Hospital, Health Center, or Private Hospital?<br><br>IF "HEALTH WORKER/ NURSE", PROBE: Did the health worker/ nurse visit you or did you go to his/her office/home?<br><br>CIRCLE THE APPROPRIATE CODE.	PUBLIC SECTOR<br>NATIONAL HOSP. (PP) . . . . 11<br>PROVINCIAL HOSP. (RH) . . . . . 12<br>DISTRICT H. (RH) . 13<br>HEALTH CENTER . 14<br>HEALTH POST . . . 15<br>OUTREACH . . . . 16<br>OTHER PUBLIC . . . 17<br><br>PRIVATE MEDICAL<br>PRIVATE HOSPITAL . . . . . 21<br>PRIVATE CLINIC . . . 22<br>PRIVATE PHARMACY . . . . . 23<br>HOME/OFFICE OF TRAINED HEALTH WORKER/ NURSE . . . . 24<br>VISIT OF TRAINED HLTH. WORKER/ NURSE . . . . 25<br>OTHER PRIVATE MEDICAL . . . . . 26<br>NOT MEDICAL SECTOR<br>SHOP SELLING DRUGS/MARKET . 31<br>KRU KHMER/ MAGICIAN . . . . . 32<br>MONK/RELIGIOUS LEADER . . . . 33<br>TRADITIONAL BIRTH ATTENDANT . . . 34<br>OUTSIDE OF THE COUNTRY . . . 41<br>OTHER 96	PUBLIC SECTOR<br>NATIONAL HOSP. (PP) . . . . 11<br>PROVINCIAL HOSP. (RH) . . . . . 12<br>DISTRICT H. (RH) . 13<br>HEALTH CENTER . 14<br>HEALTH POST . . . 15<br>OUTREACH . . . . 16<br>OTHER PUBLIC . . . 17<br><br>PRIVATE MEDICAL<br>PRIVATE HOSPITAL . . . . . 21<br>PRIVATE CLINIC . . . 22<br>PRIVATE PHARMACY . . . . . 23<br>HOME/OFFICE OF TRAINED HEALTH WORKER/ NURSE . . . . 24<br>VISIT OF TRAINED HLTH. WORKER/ NURSE . . . . 25<br>OTHER PRIVATE MEDICAL . . . . . 26<br>NOT MEDICAL SECTOR<br>SHOP SELLING DRUGS/MARKET . 31<br>KRU KHMER/ MAGICIAN . . . . . 32<br>MONK/RELIGIOUS LEADER . . . . 33<br>TRADITIONAL BIRTH ATTENDANT . . . 34<br>OUTSIDE OF THE COUNTRY . . . 41<br>OTHER 96	PUBLIC SECTOR<br>NATIONAL HOSP. (PP) . . . . 11<br>PROVINCIAL HOSP. (RH) . . . . . 12<br>DISTRICT H. (RH) . 13<br>HEALTH CENTER . 14<br>HEALTH POST . . . 15<br>OUTREACH . . . . 16<br>OTHER PUBLIC . . . 17<br><br>PRIVATE MEDICAL<br>PRIVATE HOSPITAL . . . . . 21<br>PRIVATE CLINIC . . . 22<br>PRIVATE PHARMACY . . . . . 23<br>HOME/OFFICE OF TRAINED HEALTH WORKER/ NURSE . . . . 24<br>VISIT OF TRAINED HLTH. WORKER/ NURSE . . . . 25<br>OTHER PRIVATE MEDICAL . . . . . 26<br>NOT MEDICAL SECTOR<br>SHOP SELLING DRUGS/MARKET . 31<br>KRU KHMER/ MAGICIAN . . . . . 32<br>MONK/RELIGIOUS LEADER . . . . 33<br>TRADITIONAL BIRTH ATTENDANT . . . 34<br>OUTSIDE OF THE COUNTRY . . . 41<br>OTHER 96

NO.	QUESTIONS AND FILTERS	CODING CATEGORIES		
71	How much in total was spent on transport to go to and return from (NAME OF PLACE FROM Q.70)?<br><br>RECORD IN RIELS OR IN DOLLARS.	RIELS . . . . . 1<br>DOLLARS . . . . . 2 0 0<br><br>FREE/NO COST 0000000<br>IN KIND . . . . . 9999996<br>DON'T KNOW . 9999998	RIELS . . . . . 1<br>DOLLARS . . . . . 2 0 0<br><br>FREE/NO COST 0000000<br>IN KIND . . . . . 9999996<br>DON'T KNOW . 9999998	RIELS . . . . . 1<br>DOLLARS . . . . . 2 0 0<br><br>FREE/NO COST 0000000<br>IN KIND . . . . . 9999996<br>DON'T KNOW . 9999998
72	How much in total was spent on (NAME)'s treatment at the (NAME (NAME OF PLACE FROM Q.70)?<br>IF LESS THAN 1,000,000 RIELS RECORD IN RIELS OR IN DOLLARS; IF 1,000,000 RIELS OR MORE, RECORD IN DOLLARS.	RIELS . . . . . 1<br>DOLLARS . . . . . 2 0 0<br><br>FREE/NO COST 0000000<br>IN KIND . . . . . 9999996<br>DON'T KNOW . 9999998	RIELS . . . . . 1<br>DOLLARS . . . . . 2 0 0<br><br>FREE/NO COST 0000000<br>IN KIND . . . . . 9999996<br>DON'T KNOW . 9999998	RIELS . . . . . 1<br>DOLLARS . . . . . 2 0 0<br><br>FREE/NO COST 0000000<br>IN KIND . . . . . 9999996<br>DON'T KNOW . 9999998
73	After the first visit to (NAME OF PLACE FROM Q.70), was there a second visit to this place or was advice or treatment sought anywhere else for (NAME)'s illness/injury?	YES . . . . 1<br>NO . . . . 2<br>(GO TO 80A)	YES . . . . 1<br>NO . . . . 2<br>(GO TO 80A)	YES . . . . 1<br>NO . . . . 2<br>(GO TO 80A)
74	For the second visit, where was advice or treatment sought for (NAME)'s illness/injury?<br><br>IF "HOSPITAL", PROBE: Do you mean a permanent building where health workers are present every day?<br><br>IF "YES": Was it a Provincial Hospital, District Hospital, Health Center, or Private Hospital?<br><br>IF "HEALTH WORKER/ NURSE", PROBE: Did the health worker/ nurse visit you or did you go to his/her office/home?<br><br>CIRCLE THE APPROPRIATE CODE.	PUBLIC SECTOR<br>NATIONAL HOSP. (PP) . . . . 11<br>PROVINCIAL HOSP. (RH) . . . . . 12<br>DISTRICT H. (RH) . 13<br>HEALTH CENTER . 14<br>HEALTH POST . . . 15<br>OUTREACH . . . . 16<br>OTHER PUBLIC . . . 17<br><br>PRIVATE MEDICAL<br>PRIVATE HOSPITAL . . . . . 21<br>PRIVATE CLINIC . . . 22<br>PRIVATE PHARMACY . . . . . 23<br>HOME/OFFICE OF TRAINED HEALTH WORKER/ NURSE . . . . 24<br>VISIT OF TRAINED HLTH. WORKER/ NURSE . . . . 25<br>OTHER PRIVATE MEDICAL . . . . . 26<br>NOT MEDICAL SECTOR<br>SHOP SELLING DRUGS/MARKET . 31<br>KRU KHMER/ MAGICIAN . . . . . 32<br>MONK/RELIGIOUS LEADER . . . . 33<br>TRADITIONAL BIRTH ATTENDANT . . . 34<br>OUTSIDE OF THE COUNTRY . . . 41<br>OTHER 96	PUBLIC SECTOR<br>NATIONAL HOSP. (PP) . . . . 11<br>PROVINCIAL HOSP. (RH) . . . . . 12<br>DISTRICT H. (RH) . 13<br>HEALTH CENTER . 14<br>HEALTH POST . . . 15<br>OUTREACH . . . . 16<br>OTHER PUBLIC . . . 17<br><br>PRIVATE MEDICAL<br>PRIVATE HOSPITAL . . . . . 21<br>PRIVATE CLINIC . . . 22<br>PRIVATE PHARMACY . . . . . 23<br>HOME/OFFICE OF TRAINED HEALTH WORKER/ NURSE . . . . 24<br>VISIT OF TRAINED HLTH. WORKER/ NURSE . . . . 25<br>OTHER PRIVATE MEDICAL . . . . . 26<br>NOT MEDICAL SECTOR<br>SHOP SELLING DRUGS/MARKET . 31<br>KRU KHMER/ MAGICIAN . . . . . 32<br>MONK/RELIGIOUS LEADER . . . . 33<br>TRADITIONAL BIRTH ATTENDANT . . . 34<br>OUTSIDE OF THE COUNTRY . . . 41<br>OTHER 96	PUBLIC SECTOR<br>NATIONAL HOSP. (PP) . . . . 11<br>PROVINCIAL HOSP. (RH) . . . . . 12<br>DISTRICT H. (RH) . 13<br>HEALTH CENTER . 14<br>HEALTH POST . . . 15<br>OUTREACH . . . . 16<br>OTHER PUBLIC . . . 17<br><br>PRIVATE MEDICAL<br>PRIVATE HOSPITAL . . . . . 21<br>PRIVATE CLINIC . . . 22<br>PRIVATE PHARMACY . . . . . 23<br>HOME/OFFICE OF TRAINED HEALTH WORKER/ NURSE . . . . 24<br>VISIT OF TRAINED HLTH. WORKER/ NURSE . . . . 25<br>OTHER PRIVATE MEDICAL . . . . . 26<br>NOT MEDICAL SECTOR<br>SHOP SELLING DRUGS/MARKET . 31<br>KRU KHMER/ MAGICIAN . . . . . 32<br>MONK/RELIGIOUS LEADER . . . . 33<br>TRADITIONAL BIRTH ATTENDANT . . . 34<br>OUTSIDE OF THE COUNTRY . . . 41<br>OTHER 96

NO.	QUESTIONS AND FILTERS	CODING CATEGORIES		
75	How much in total was spent on transport to go to and return from (NAME OF PLACE FROM Q.74)?<br><br>RECORD IN RIELS OR IN DOLLARS.	RIELS . . . . . 1 [ ][ ][ ][ ][ ][ ]<br>DOLLARS . . . . . 2 [0][0][ ][ ][ ][ ]<br><br>FREE/NO COST 0000000<br>IN KIND . . . . . 9999996<br>DON'T KNOW . 9999998	RIELS . . . . . 1 [ ][ ][ ][ ][ ][ ]<br>DOLLARS . . . . . 2 [0][0][ ][ ][ ][ ]<br><br>FREE/NO COST 0000000<br>IN KIND . . . . . 9999996<br>DON'T KNOW . 9999998	RIELS . . . . . 1 [ ][ ][ ][ ][ ][ ]<br>DOLLARS . . . . . 2 [0][0][ ][ ][ ][ ]<br><br>FREE/NO COST 0000000<br>IN KIND . . . . . 9999996<br>DON'T KNOW . 9999998
76	How much in total was spent on (NAME)'s treatment at the (NAME OF PLACE FROM Q.74)?<br>IF LESS THAN 1,000,000 RIELS RECORD IN RIELS OR IN DOLLARS; IF 1,000,000 RIELS OR MORE, RECORD IN DOLLARS.	RIELS . . . . . 1 [ ][ ][ ][ ][ ][ ]<br>DOLLARS . . . . . 2 [0][0][ ][ ][ ][ ]<br><br>FREE/NO COST 0000000<br>IN KIND . . . . . 9999996<br>DON'T KNOW . 9999998	RIELS . . . . . 1 [ ][ ][ ][ ][ ][ ]<br>DOLLARS . . . . . 2 [0][0][ ][ ][ ][ ]<br><br>FREE/NO COST 0000000<br>IN KIND . . . . . 9999996<br>DON'T KNOW . 9999998	RIELS . . . . . 1 [ ][ ][ ][ ][ ][ ]<br>DOLLARS . . . . . 2 [0][0][ ][ ][ ][ ]<br><br>FREE/NO COST 0000000<br>IN KIND . . . . . 9999996<br>DON'T KNOW . 9999998
77	After the second visit to (NAME OF PLACE FROM Q.74), was there a third visit to this place or was advice or treatment sought anywhere else for (NAME)'s illness/injury?	YES . . . . 1<br>NO . . . . 2<br>(GO TO 80A)	YES . . . . 1<br>NO . . . . 2<br>(GO TO 80A)	YES . . . . 1<br>NO . . . . 2<br>(GO TO 80A)
78	For the third visit, where was advice or treatment sought for (NAME)'s illness/injury?<br><br>IF "HOSPITAL", PROBE: Do you mean a permanent building where health workers are present every day?<br><br>IF "YES": Was it a Provincial Hospital, District Hospital, Health Center, or Private Hospital?<br><br>IF "HEALTH WORKER/ NURSE", PROBE: Did the health worker/ nurse visit you or did you go to his/her office/home?<br><br>CIRCLE THE APPROPRIATE CODE.	PUBLIC SECTOR<br>NATIONAL HOSP. (PP) . . . . 11<br>PROVINCIAL HOSP. (RH) . . . . . 12<br>DISTRICT H. (RH) . 13<br>HEALTH CENTER . 14<br>HEALTH POST . . . 15<br>OUTREACH . . . . 16<br>OTHER PUBLIC . . . 17<br><br>PRIVATE MEDICAL<br>PRIVATE HOSPITAL . . . . . 21<br>PRIVATE CLINIC . . . 22<br>PRIVATE PHARMACY . . . . . 23<br>HOME/OFFICE OF TRAINED HEALTH WORKER/ NURSE . . . . 24<br>VISIT OF TRAINED HLTH. WORKER/ NURSE . . . . 25<br>OTHER PRIVATE MEDICAL . . . . . 26<br>NOT MEDICAL SECTOR<br>SHOP SELLING DRUGS/MARKET . 31<br>KRU KHMER/ MAGICIAN . . . . . 32<br>MONK/RELIGIOUS LEADER . . . . 33<br>TRADITIONAL BIRTH ATTENDANT . . . 34<br>OUTSIDE OF THE COUNTRY . . . 41<br>OTHER 96	PUBLIC SECTOR<br>NATIONAL HOSP. (PP) . . . . 11<br>PROVINCIAL HOSP. (RH) . . . . . 12<br>DISTRICT H. (RH) . 13<br>HEALTH CENTER . 14<br>HEALTH POST . . . 15<br>OUTREACH . . . . 16<br>OTHER PUBLIC . . . 17<br><br>PRIVATE MEDICAL<br>PRIVATE HOSPITAL . . . . . 21<br>PRIVATE CLINIC . . . 22<br>PRIVATE PHARMACY . . . . . 23<br>HOME/OFFICE OF TRAINED HEALTH WORKER/ NURSE . . . . 24<br>VISIT OF TRAINED HLTH. WORKER/ NURSE . . . . 25<br>OTHER PRIVATE MEDICAL . . . . . 26<br>NOT MEDICAL SECTOR<br>SHOP SELLING DRUGS/MARKET . 31<br>KRU KHMER/ MAGICIAN . . . . . 32<br>MONK/RELIGIOUS LEADER . . . . 33<br>TRADITIONAL BIRTH ATTENDANT . . . 34<br>OUTSIDE OF THE COUNTRY . . . 41<br>OTHER 96	PUBLIC SECTOR<br>NATIONAL HOSP. (PP) . . . . 11<br>PROVINCIAL HOSP. (RH) . . . . . 12<br>DISTRICT H. (RH) . 13<br>HEALTH CENTER . 14<br>HEALTH POST . . . 15<br>OUTREACH . . . . 16<br>OTHER PUBLIC . . . 17<br><br>PRIVATE MEDICAL<br>PRIVATE HOSPITAL . . . . . 21<br>PRIVATE CLINIC . . . 22<br>PRIVATE PHARMACY . . . . . 23<br>HOME/OFFICE OF TRAINED HEALTH WORKER/ NURSE . . . . 24<br>VISIT OF TRAINED HLTH. WORKER/ NURSE . . . . 25<br>OTHER PRIVATE MEDICAL . . . . . 26<br>NOT MEDICAL SECTOR<br>SHOP SELLING DRUGS/MARKET . 31<br>KRU KHMER/ MAGICIAN . . . . . 32<br>MONK/RELIGIOUS LEADER . . . . 33<br>TRADITIONAL BIRTH ATTENDANT . . . 34<br>OUTSIDE OF THE COUNTRY . . . 41<br>OTHER 96

NO.	QUESTIONS AND FILTERS	CODING CATEGORIES		
79	How much in total was spent on transport to go to and return from (NAME OF PLACE FROM Q.78)?<br><br>RECORD IN RIELS OR IN DOLLARS.	RIELS . . . . . 1 [ ][ ][ ][ ][ ][ ]<br>DOLLARS . . . . . 2 [0][0][ ][ ][ ][ ]<br><br>FREE/NO COST 0000000<br>IN KIND . . . . . 9999996<br>DON'T KNOW . 9999998	RIELS . . . . . 1 [ ][ ][ ][ ][ ][ ]<br>DOLLARS . . . . . 2 [0][0][ ][ ][ ][ ]<br><br>FREE/NO COST 0000000<br>IN KIND . . . . . 9999996<br>DON'T KNOW . 9999998	RIELS . . . . . 1 [ ][ ][ ][ ][ ][ ]<br>DOLLARS . . . . . 2 [0][0][ ][ ][ ][ ]<br><br>FREE/NO COST 0000000<br>IN KIND . . . . . 9999996<br>DON'T KNOW . 9999998
80	How much in total was spent on (NAME)'s treatment at the (NAME OF PLACE FROM Q.78)?<br>IF LESS THAN 1,000,000 RIELS RECORD IN RIELS OR IN DOLLARS. IF 1,000,000 RIELS OR MORE, RECORD IN DOLLARS.	RIELS . . . . . 1 [ ][ ][ ][ ][ ][ ]<br>DOLLARS . . . . . 2 [0][0][ ][ ][ ][ ]<br><br>FREE/NO COST 0000000<br>IN KIND . . . . . 9999996<br>DON'T KNOW . 9999998	RIELS . . . . . 1 [ ][ ][ ][ ][ ][ ]<br>DOLLARS . . . . . 2 [0][0][ ][ ][ ][ ]<br><br>FREE/NO COST 0000000<br>IN KIND . . . . . 9999996<br>DON'T KNOW . 9999998	RIELS . . . . . 1 [ ][ ][ ][ ][ ][ ]<br>DOLLARS . . . . . 2 [0][0][ ][ ][ ][ ]<br><br>FREE/NO COST 0000000<br>IN KIND . . . . . 9999996<br>DON'T KNOW . 9999998
80A		GO BACK TO 68 IN NEXT COLUMN; OR, IF NO OTHER PERSON, GO TO 81.	GO BACK TO 68 IN NEXT COLUMN; OR, IF NO OTHER PERSON, GO TO 81.	GO BACK TO 68 IN NEXT COLUMN OF ADDITIONAL QUESTIONNAIRE; OR, IF NO OTHER PERSON, GO TO 81.
81	Where did the money come from to pay for transportation and treatment for the (two/three) member(s) of your household who had an illness/injury over the past 30 days?<br><br>RECORD ALL MENTIONED	SUBSIDY<br>HEALTH EQUITY FUND . . . . A<br>VOUCHER . . . . B<br>FEE EXEMPTION . . . . C<br>NGO . . . . D<br>INSURANCE SCHEMES<br>NATIONAL SOCIAL SECURITY FUND . . . E<br>COMMUNITY BASED HEALTH INSURANCE . . . . F<br>HEALTH INSURANCE THROUGH EMPLOYER . . . . G<br>OTHER PRIVATELY PURCHASED COMMERCIAL HEALTH INSURANCE .. H<br>OUT OF POCKET<br>WAGE/INCOME . . . . I<br>LOAN/TON TIN . . . . J<br>SALE OF ASSETS . . . . K<br>GIFT FROM RELATIVE . . . . L<br>SAVING . . . . M<br>OTHER ______________________ X<br>(Specify)		

HOUSEHOLD CHARACTERISTICS

NO.	QUESTIONS AND FILTERS	CODING CATEGORIES	SKIP
101	How often does anyone smoke inside your house?<br>Would you say daily, weekly, monthly, less than monthly, or never?	DAILY . . . . . 1<br>WEEKLY . . . . . 2<br>MONTHLY . . . . . 3<br>LESS THAN MONTHLY . . . . . 4<br>NEVER 5	
102	What is the main source of drinking water during the dry season for members of your household?	PIPED WATER<br>PIPED INTO DWELLING . . . . . 11<br>PIPED TO YARD/PLOT . . . . . 12<br>PUBLIC TAP/STANDPIPE . . . . . 13<br>TUBE WELL OR BOREHOLE . . . . . 21<br>DUG WELL<br>PROTECTED WELL . . . . . 31<br>UNPROTECTED WELL . . . . . 32<br>WATER FROM SPRING<br>PROTECTED SPRING . . . . . 41<br>UNPROTECTED SPRING . . . . . 42<br>RAINWATER . . . . . 51<br>TANKER TRUCK . . . . . 61<br>CART WITH SMALL TANK . . . . . 71<br>SURFACE WATER (RIVER/DAM/ LAKE/POND/STREAM/CANAL/ IRRIGATION CHANNEL) . . . . . 81<br>BOTTLED WATER . . . . . 91<br>OTHER ______ 96<br>(SPECIFY)	11, 12 → 104A<br>51 → 104A
103	Where is that water source located?	IN OWN DWELLING . . . . . 1<br>IN OWN YARD/PLOT . . . . . 2<br>ELSEWHERE 3	1, 2 → 104A
104	How long does it take to go there, get water, and come back?	MINUTES . . . . . [ ][ ][ ]<br>DON'T KNOW 998	
104A	During the wet season, is the main source of drinking water for members of your household the same as during the dry season?	YES . . . . . 1<br>NO 2	1 → 105
104B	What is the main source of drinking water during the wet season for members of your household?	PIPED WATER<br>PIPED INTO DWELLING . . . . . 11<br>PIPED TO YARD/PLOT . . . . . 12<br>PUBLIC TAP/STANDPIPE . . . . . 13<br>TUBE WELL OR BOREHOLE . . . . . 21<br>DUG WELL<br>PROTECTED WELL . . . . . 31<br>UNPROTECTED WELL . . . . . 32<br>WATER FROM SPRING<br>PROTECTED SPRING . . . . . 41<br>UNPROTECTED SPRING . . . . . 42<br>RAINWATER . . . . . 51<br>TANKER TRUCK . . . . . 61<br>CART WITH SMALL TANK . . . . . 71<br>SURFACE WATER (RIVER/DAM/ LAKE/POND/STREAM/CANAL/ IRRIGATION CHANNEL) . . . . . 81<br>BOTTLED WATER . . . . . 91<br>OTHER ______ 96<br>(SPECIFY)	11, 12 → 105<br>51 → 105

NO.	QUESTIONS AND FILTERS	CODING CATEGORIES	SKIP
104C	Where is that water source located?	IN OWN DWELLING . . . . 1<br>IN OWN YARD/PLOT . . . . 2<br>ELSEWHERE 3	1, 2 → 105
104D	How long does it take to go there, get water, and come back?	MINUTES . . . . [ ][ ][ ]<br>DON'T KNOW 998	
105	Do you do anything to the water to make it safer to drink?	YES, ALWAYS . . . . 1<br>YES, SOMETIME . . . . 2<br>NO . . . . 3<br>DON'T KNOW 8	3, 8 → 107
106	What do you usually do to make the water safer to drink?<br><br>Anything else?<br><br>RECORD ALL MENTIONED.	BOIL . . . . A<br>ADD BLEACH/CHLORINE . . . . B<br>STRAIN THROUGH A CLOTH . . . . C<br>USE WATER FILTER (CERAMIC/<br>SAND/COMPOSITE/ETC.) . . . . D<br>SOLAR DISINFECTION . . . . E<br>LET IT STAND AND SETTLE . . . . F<br><br>OTHER ______________________ X<br>(SPECIFY)<br>DON'T KNOW . . . . Z	

NO.	QUESTIONS AND FILTERS	CODING CATEGORIES	SKIP
107	What kind of toilet facility do members of your household usually use?	FLUSH OR POUR FLUSH TOILET<br>FLUSH TO PIPED SEWER<br>SYSTEM . . . . 11<br>FLUSH TO SEPTIC TANK . . . . 12<br>FLUSH TO PIT LATRINE . . . . 13<br>FLUSH TO SOMEWHERE ELSE . . . . 14<br>FLUSH, DON'T KNOW WHERE . . . . 15<br>PIT LATRINE<br>VENTILATED IMPROVED<br>PIT LATRINE . . . . 21<br>PIT LATRINE WITH SLAB . . . . 22<br>PIT LATRINE WITHOUT SLAB/<br>OPEN PIT . . . . 23<br>COMPOSTING TOILET . . . . 31<br>BUCKET TOILET . . . . 41<br>HANGING TOILET/HANGING<br>LATRINE . . . . 51<br>NO FACILITY/BUSH/FIELD . . . . 61<br>OTHER ______________________ 96<br>(SPECIFY)	 <br><br><br><br><br><br><br><br><br><br><br><br><br><br><br><br>→ 110
108	Do you share this toilet facility with other households?	YES . . . . 1<br>NO 2	 → 110
109	How many households use this toilet facility?	NO. OF HOUSEHOLDS<br>IF LESS THAN 10 . . . . [0][ ]<br>10 OR MORE HOUSEHOLDS . . . . 95<br>DON'T KNOW 98	

110	Does your household have:		YES	NO
	Electricity?	ELECTRICITY	1	2
	A radio?	RADIO	1	2
	A television?	TELEVISION	1	2
	A mobile telephone?	MOBILE TELEPHONE	1	2
	A non-mobile telephone?	NON-MOBILE TELEPHONE . . .	1	2
	A refrigerator?	REFRIGERATOR	1	2
	A wardrobe	WARDROBE	1	2
	A sewing machine or loom	SEWING MACHINE	1	2
	A CD/DVD player?	CD/DVD	1	2
	A generator/battery/solar panel	GENERATOR/BATTERY/SOLAR	1	2

NO.	QUESTIONS AND FILTERS	CODING CATEGORIES	SKIP
111	What type of fuel does your household mainly use for cooking?	ELECTRICITY . . . . 01<br>LPG . . . . 02<br>BIOGAS . . . . 03<br>KEROSENE . . . . 04<br>COAL, LIGNITE . . . . 05<br>CHARCOAL . . . . 06<br>WOOD . . . . 07<br>STRAW/SHRUBS/GRASS . . . . 08<br>AGRICULTURAL CROP . . . . 09<br>ANIMAL DUNG . . . . 10<br>NO FOOD COOKED<br>IN HOUSEHOLD . . . . 95<br>OTHER ______________________ 96<br>(SPECIFY)	 <br><br><br><br><br><br><br><br><br><br>→ 114

NO.	QUESTIONS AND FILTERS	CODING CATEGORIES	SKIP
112	Is the cooking usually done in the house, in a separate building, or outdoors?	IN THE HOUSE . . . . 1<br>IN A SEPARATE BUILDING . . . . 2<br>OUTDOORS . . . . 3<br><br>OTHER ______________ 6<br>(SPECIFY)	2, 3, 6 → 114
113	Do you have a separate room which is used as a kitchen?	YES . . . . 1<br>NO 2	
114	MAIN MATERIAL OF THE FLOOR.<br><br>RECORD OBSERVATION.	NATURAL FLOOR<br>EARTH/SAND/CLAY . . . . 11<br>DUNG . . . . 12<br>RUDIMENTARY FLOOR<br>WOOD PLANKS . . . . 21<br>PALM/BAMBOO . . . . 22<br>FINISHED FLOOR<br>PARQUET OR POLISHED<br>WOOD . . . . 31<br>VINYL OR ASPHALT STRIPS . . . . 32<br>CERAMIC TILES . . . . 33<br>CEMENT TILES . . . . 34<br>CEMENT . . . . 35<br>FLOATING HOUSE . . . . 41<br><br>OTHER ______________ 96<br>(SPECIFY)	
115	MAIN MATERIAL OF THE ROOF.<br><br>RECORD OBSERVATION.	NATURAL ROOFING<br>NO ROOF . . . . 11<br>BAMBOO/THATCH/PALM LEAF . . . . 12<br>RUDIMENTARY ROOFING<br>RUSTIC MAT . . . . 21<br>WOOD PLANKS . . . . 22<br>CARDBOARD . . . . 23<br>PLASTIC SHEET 24<br>FINISHED ROOFING<br>METAL . . . . 31<br>WOOD . . . . 32<br>CALAMINE/CEMENT FIBER . . . . 33<br>CERAMIC TILES . . . . 34<br>CLAY TILES . . . . 35<br>CEMENT . . . . 36<br><br>OTHER ______________ 96<br>(SPECIFY)	

NO.	QUESTIONS AND FILTERS	CODING CATEGORIES	SKIP
116	MAIN MATERIAL OF THE EXTERIOR WALLS.<br><br>RECORD OBSERVATION.	NATURAL WALLS<br>NO WALLS . . . . 11<br>PALM/BAMBOO/THACH . . . . . . 12<br>DIRT . . . . 13<br>RUDIMENTARY WALLS<br>BAMBOO WITH MUD . . . . . . 21<br>STRAW WITH MUD 22<br>STONE WITH MUD . . . . . . 23<br>UNCOVERED ADOBE . . . . . . 24<br>PLYWOOD . . . . 25<br>CARDBOARD . . . . 26<br>REUSED WOOD . . . . 27<br>METAL . . . . 28<br>FINISHED WALLS<br>CEMENT . . . . 31<br>STONE WITH LIME/CEMENT . . . . 32<br>BRICKS . . . . 33<br>CEMENT BLOCKS . . . . 34<br>COVERED ADOBE . . . . . . 35<br>WOOD PLANKS/SHINGLES . . . . 36<br><br>OTHER ______________ 96<br>(SPECIFY)	
117	How many rooms in this household are used for sleeping?	ROOMS . . . . [ ][ ]	
118	Does any member of this household own:<br><br>A watch?<br>A bicycle or cyclo?<br>A motorcycle or motor scooter?<br>A motorcycle-cart<br>A oxcart or horsecart?<br>A car or truck, tractor or van?<br>A boat with a motor?<br>A boat without a motor?	YES NO<br>WATCH . . . . . . 1 2<br>BICYCLE/CYCLO . . . . 1 2<br>MOTORCYCLE/SCOOTER . . . 1 2<br>MOTORCYCLE-CART . . . . 1 2<br>OXCART/HORSECART . . . . 1 2<br>CAR/TRUCK/TRACTOR/VAN . 1 2<br>BOAT WITH MOTOR . . . . 1 2<br>BOAT WITHOUT MOTOR . . . 1 2	
119	Does any member of this household own any agricultural land?	YES . . . . 1<br>NO 2	 → 121
120	How many hectares of agricultural land do members of this household own?	SQ. METER 1 [ ][ ][ ][ ].[ ]<br>A 2 [ ][ ][ ][ ].[ ]<br>HECTARES 3 [ ][ ][ ][ ].[ ]<br>RAY 4 [ ][ ][ ][ ].[ ]<br>KONG 5 [ ][ ][ ][ ].[ ]<br><br>DON'T KNOW 999998	
121	Does this household own any livestock, herds, other farm animals, or poultry?	YES . . . . 1<br>NO 2	 → 123

NO.	QUESTIONS AND FILTERS	CODING CATEGORIES	SKIP
122	How many of the following animals does this household own?<br><br>IF NONE, ENTER '00'.<br>IF 95 OR MORE, ENTER '95'.<br>IF UNKNOWN, ENTER '98'.<br><br>Water buffalo<br>Cows or bulls?<br>Horses, donkeys, or mules?<br>Goats/sheep?<br>Pigs?<br>Chickens or ducks?<br>Elephant?<br>Other ________ specify	WATER BUFFALO . . . . [ ][ ]<br>COWS/BULLS . . . . [ ][ ]<br>HORSES/DONKEYS/MULES . . . . . [ ][ ]<br>GOATS/SHEEPS . . . . [ ][ ]<br>PIGS . . . . [ ][ ]<br>CHICKENS/DUGS . . . . [ ][ ]<br>ELEPHANT . . . . [ ][ ]<br>OTHER . . . . [ ][ ]	
123	Does any member of this household have a bank account?	YES . . . . 1<br>NO 2	

NO.	QUESTIONS AND FILTERS	CODING CATEGORIES	SKIP
137	Please show me where members of your household most often wash their hands.	OBSERVED . . . . . 1<br>NOT OBSERVED,<br>NOT IN DWELLING/YARD/PLOT . . . . . 2<br>NOT OBSERVED,<br>NO PERMISSION TO SEE . . . . . 3<br>NOT OBSERVED, OTHER REASON 4	2, 3, 4 → 140
138	OBSERVATION ONLY:<br><br>OBSERVE PRESENCE OF WATER AT THE SPECIFIC PLACE FOR HANDWASHING.	WATER IS AVAILABLE . . . . . 1<br>WATER IS NOT AVAILABLE 2	
139	OBSERVATION ONLY:<br><br>OBSERVE PRESENCE OF SOAP, DETERGENT, OR OTHER CLEANSING AGENT.	SOAP OR DETERGENT<br>(BAR, LIQUID, POWDER, PASTE) . . . . . A<br>ASH, MUD, SAND . . . . . B<br>NONE C	
140	ASK RESPONDENT FOR A TEASPOONFUL OF COOKING SALT.<br><br>TEST SALT FOR IODINE.	IODINE PRESENT . . . . . 1<br>NO IODINE . . . . . 2<br><br>NO SALT IN HOUSEHOLD . . . . . 3<br><br>SALT NOT TESTED ________ 6<br>(SPECIFY REASON)	
141	ASK RESPONDENT TO SEE THE PACKAGE OR CAN OF THE COOKING SALT, AND CHECK THE LABEL.	LABELED IODIZED SALT . . . . . 1<br>NOT LABELED IODIZED SALT . . . . . 2<br><br>NO SALT PACKAGE/CAN IN<br>HOUSEHOLD 3	
142	Has this household been identified as poor through the Identification of Poor Households process conducted by village representatives, and been placed on the List of Poor Households or received an Equity Card or Priority Access Card?<br><br>ASK TO SEE THE EQUITY, PRIORITY ACCESS CARD AND OTHER CARD INCLUDE POST-IDENTIFICATION	(YES CARD SEEN / YES CARD NOT SEEN / NO)<br>A. EQUITY CARD: 1 / 2 / 3<br><br>B. PRIORITY ACCESS CARD/OTHER: 1 / 2 / 3	
143	Do members of this household receive free or subsidized health care that other people would normally have to pay for?	YES, FREE . . . . . 1<br>YES, SUBSIDIZED . . . . . 2<br>NO 3	3 → 145
144	What are free and/or subsidized health care that any member of this household received?	HEALTH EQUITY FUNDS . . . . . A<br>COMMUNITY BASED HEALTH<br>INSURANCE . . . . . B<br>MATERNITY VOUCHER . . . . . C<br>OTHER ________ D<br>SPECIFY	

145	**FOR HOUSEHOLD SELECTED FOR MALE INTERVIEW AND SECTION DV OF THE WOMEN QUESTIONNAIRE**

LOOK AT THE LAST DIGIT OF THE HOUSEHOLD QUESTIONNAIRE SERIAL NUMBER ON THE COVER PAGE. THIS IS THE ROW NUMBER YOU SHOULD GO TO. CHECK THE TOTAL NUMBER OF ELIGIBLE WOMEN (COLUMN 9) IN THE HOUSEHOLD SCHEDULE. THIS IS THE COLUMN NUMBER YOU SHOULD GO TO. FOLLOW THE SELECTED ROW AND COLUMN TO THE CELL WHERE THEY MEET AND CIRCLE THE NUMBER IN THE CELL. THIS IS THE NUMBER OF THE WOMAN SELECTED FOR THE DOMESTIC VIOLENCE QUESTIONS FROM THE LIST OF ELIGIBLE WOMEN IN COLUMN 9 OF THE HOUSEHOLD SCHEDULE. WRITE THE NAME AND LINE NUMBER OF THE SELECTED WOMAN IN THE SPACE BELOW THE TABLE.

EXAMPLE: THE HOUSEHOLD QUESTIONNAIRE SERIAL NUMBER IS ‘716’ AND THE HOUSEHOLD SCHEDULE COLUMN 9 SHOWS THAT THERE ARE THREE ELIGIBLE WOMEN AGE 15-49 IN THE HOUSEHOLD (LINE NUMBERS 02, 04, AND 05). SINCE THE LAST DIGIT OF THE HOUSEHOLD SERIAL NUMBER IS '6' GO TO ROW '6' AND SINCE THERE ARE THREE ELIGIBLE WOMEN IN THE HOUSEHOLD, GO TO COLUMN '3’. FOLLOW THE ROW AND COLUMN AND FIND THE NUMBER IN THE CELL WHERE THEY MEET (‘2’) AND CIRCLE THE NUMBER. NOW GO TO THE HOUSEHOLD SCHEDULE AND FIND THE SECOND WOMAN WHO IS ELIGIBLE FOR THE WOMAN'S INTERVIEW (LINE NUMBER '04' IN THIS EXAMPLE). WRITE HER NAME AND LINE NUMBER IN THE SPACE BELOW THE TABLE.

LAST DIGIT OF THE HOUSEHOLD QUESTIONNAIRE SERIAL NUMBER	TOTAL NUMBER OF ELIGIBLE WOMEN AGE 15-49 IN HOUSEHOLD SCHEDULE COLUMN 9							
	1	2	3	4	5	6	7	8
0	1	2	2	4	3	6	5	4
1	1	1	3	1	4	1	6	5
2	1	2	1	2	5	2	7	6
3	1	1	2	3	1	3	1	7
4	1	2	3	4	2	4	2	8
5	1	1	1	1	3	5	3	1
6	1	2	2	2	4	6	4	2
7	1	1	3	3	5	1	5	3
8	1	2	1	4	1	2	6	4
9	1	1	2	1	2	3	7	5

NAME OF SELECTED WOMAN ____________

HH LINE NUMBER OF SELECTED WOMAN . . . . [ ][ ]

CHECK COVER PAGE TO SEE IF HOUSEHOLD IS SELECTED FOR ANEMIA AND ANTHROPOMETRY

200	CHECK COVER PAGE OF HOUSEHOLD QUESTIONAIRE:<br>HOUSEHOLD SELECTED FOR ANEMIA TEST AND ANTHROPOMETRIC MEASUREMENTS ☐<br>HOUSEHOLD IS NOT SELECTED FOR ANEMIA TEST AND ANTHROPOMETRIC MEASUREMENTS ☐ → GO TO 242A

WEIGHT, HEIGHT AND HEMOGLOBIN MEASUREMENT FOR CHILDREN AGE 0-5

201	CHECK COLUMN 11 IN HOUSEHOLD SCHEDULE. RECORD THE LINE NUMBER AND NAME FOR ALL ELIGIBLE CHILDREN 0-5 YEARS IN QUESTION 202. IF MORE THAN SIX CHILDREN, USE ADDITIONAL QUESTIONNAIRE(S).			
		CHILD 1	CHILD 2	CHILD 3
202	LINE NUMBER FROM COLUMN 11<br><br>NAME FROM COLUMN 2	LINE NUMBER . . . . . ☐☐<br><br>NAME ________	LINE NUMBER . . . . . ☐☐<br><br>NAME ________	LINE NUMBER . . . . . ☐☐<br><br>NAME ________
203	IF MOTHER INTERVIEWED, COPY MONTH AND YEAR OF BIRTH FROM BIRTH HISTORY AND ASK DAY; IF MOTHER NOT INTERVIEWED, ASK:<br>What is (NAME's) birth date?	DAY . . . . ☐☐<br>MONTH . . . . . ☐☐<br>YEAR ☐☐☐☐	DAY . . . . ☐☐<br>MONTH . . . . . ☐☐<br>YEAR ☐☐☐☐	DAY . . . . ☐☐<br>MONTH . . . . . ☐☐<br>YEAR ☐☐☐☐
204	CHECK 203:<br>CHILD BORN IN JANUARY 2009 OR LATER?	YES . . . . 1<br>NO . . . . 2<br>(GO TO 203 FOR NEXT CHILD OR, IF NO MORE CHILDREN, GO TO 214)	YES . . . . 1<br>NO . . . . 2<br>(GO TO 203 FOR NEXT CHILD OR, IF NO MORE CHILDREN, GO TO 214)	YES . . . . 1<br>NO . . . . 2<br>(GO TO 203 FOR NEXT CHILD OR, IF NO MORE CHILDREN, GO TO 214)
205	WEIGHT IN KILOGRAMS	KG. ☐☐ . ☐☐<br><br>NOT PRESENT . . . 9994<br>REFUSED . . . . 9995<br>OTHER 9996	KG. ☐☐ . ☐☐<br><br>NOT PRESENT . . . 9994<br>REFUSED . . . . 9995<br>OTHER 9996	KG. ☐☐ . ☐☐<br><br>NOT PRESENT . . . 9994<br>REFUSED . . . . 9995<br>OTHER 9996
206	HEIGHT IN CENTIMETERS	CM. ☐☐☐ . ☐<br><br>NOT PRESENT . . . 9994<br>REFUSED . . . . 9995<br>OTHER 9996	CM. ☐☐☐ . ☐<br><br>NOT PRESENT . . . 9994<br>REFUSED . . . . 9995<br>OTHER 9996	CM. ☐☐☐ . ☐<br><br>NOT PRESENT . . . 9994<br>REFUSED . . . . 9995<br>OTHER 9996
207	MEASURED LYING DOWN OR STANDING UP?	LYING DOWN . . . . 1<br>STANDING UP . . . . 2<br>NOT MEASURED 3	LYING DOWN . . . . 1<br>STANDING UP . . . . 2<br>NOT MEASURED 3	LYING DOWN . . . . 1<br>STANDING UP . . . . 2<br>NOT MEASURED 3
208	CHECK 203:<br>IS CHILD AGE 0-5 MONTHS, I.E., WAS CHILD BORN IN MONTH OF INTERVIEW OR FIVE PREVIOUS MONTHS?	0-5 MONTHS . . . . 1<br>(GO TO 203 FOR NEXT CHILD OR, IF NO MORE CHILDREN, GO TO 214)<br>OLDER 2	0-5 MONTHS . . . . 1<br>(GO TO 203 FOR NEXT CHILD OR, IF NO MORE CHILDREN, GO TO 214)<br>OLDER 2	0-5 MONTHS . . . . 1<br>(GO TO 203 FOR NEXT CHILD OR, IF NO MORE CHILDREN, GO TO 214)<br>OLDER 2
209	LINE NUMBER OF PARENT/ OTHER ADULT RESPONSIBLE FOR THE CHILD (FROM COLUMN 1 OF HOUSEHOLD SCHEDULE.) RECORD '00' IF NOT LISTED.	LINE NUMBER . . . . . ☐☐	LINE NUMBER . . . . . ☐☐	LINE NUMBER . . . . . ☐☐

WEIGHT, HEIGHT AND HEMOGLOBIN MEASUREMENT FOR CHILDREN AGE 0-5

		CHILD 1	CHILD 2	CHILD 3
210	ASK CONSENT FOR ANEMIA TEST FROM PARENT/OTHER ADULT IDENTIFIED IN 209 AS RESPONSIBLE FOR CHILD.	As part of this survey, we are asking people all over the country to take an anemia test. Anemia is a serious health problem that usually results from poor nutrition, infection, or chronic disease. This survey will assist the government to develop programs to prevent and treat anemia.<br><br>We ask that all children born in 2009 or later take part in anemia testing in this survey and give a few drops of blood from a finger or heel. The equipment used to take the blood is clean and completely safe. It has never been used before and will be thrown away after each test.<br><br>The blood will be tested for anemia immediately, and the result will be told to you right away. The result will be kept strictly confidential and will not be shared with anyone other than members of our survey team.<br>Do you have any questions?<br>You can say yes to the test, or you can say no. It is up to you to decide.<br>Will you allow (NAME OF CHILD/NAMES OF CHILDREN) to participate in the anemia test?		
211	CIRCLE THE APPROPRIATE CODE AND SIGN YOUR NAME.	GRANTED . . . . 1<br>________ (SIGN)<br>REFUSED 2	GRANTED . . . . 1<br>________ (SIGN)<br>REFUSED 2	GRANTED . . . . 1<br>________ (SIGN)<br>REFUSED 2
212	RECORD HEMOGLOBIN LEVEL HERE AND IN THE ANEMIA PAMPHLET **(11)**.	G/DL [ ][ ].[ ]<br><br>NOT PRESENT . . . . .994<br>REFUSED . . . . .995<br>OTHER 996	G/DL [ ][ ].[ ]<br><br>NOT PRESENT . . . . .994<br>REFUSED . . . . .995<br>OTHER 996	G/DL [ ][ ].[ ]<br><br>NOT PRESENT . . . . .994<br>REFUSED . . . . .995<br>OTHER 996
213	GO BACK TO 203 IN NEXT COLUMN OF THIS QUESTIONNAIRE OR IN THE FIRST COLUMN OF THE NEXT PAGE; IF NO MORE CHILDREN, GO TO 214.			

WEIGHT, HEIGHT AND HEMOGLOBIN MEASUREMENT FOR CHILDREN AGE 0-5

		CHILD 4	CHILD 5	CHILD 6
202	LINE NUMBER FROM COLUMN 11<br><br>NAME FROM COLUMN 2	LINE NUMBER . . . . . ☐☐<br><br>NAME ________	LINE NUMBER . . . . . ☐☐<br><br>NAME ________	LINE NUMBER . . . . . ☐☐<br><br>NAME ________
203	IF MOTHER INTERVIEWED, COPY MONTH AND YEAR OF BIRTH FROM BIRTH HISTORY AND ASK DAY; IF MOTHER NOT INTERVIEWED, ASK:<br>What is (NAME'S) birth date?	DAY . . . . ☐☐<br>MONTH . . . . . ☐☐<br>YEAR ☐☐☐☐	DAY . . . . ☐☐<br>MONTH . . . . . ☐☐<br>YEAR ☐☐☐☐	DAY . . . . ☐☐<br>MONTH . . . . . ☐☐<br>YEAR ☐☐☐☐
204	CHECK 203:<br>CHILD BORN IN JANUARY 2009 OR LATER?	YES . . . . 1<br>NO . . . . 2<br>(GO TO 203 FOR NEXT CHILD OR, IF NO MORE CHILDREN, GO TO 214)	YES . . . . 1<br>NO . . . . 2<br>(GO TO 203 FOR NEXT CHILD OR, IF NO MORE CHILDREN, GO TO 214)	YES . . . . 1<br>NO . . . . 2<br>(GO TO 203 IN FIRST COLUMN OF NEW QUESTIONNAIRE; OR, IF NO MORE CHILDREN, GO TO 214)
205	WEIGHT IN KILOGRAMS	KG. ☐☐.☐☐<br><br>NOT PRESENT . . . . . 9994<br>REFUSED . . . . 9995<br>OTHER 9996	KG. ☐☐.☐☐<br><br>NOT PRESENT . . . . . 9994<br>REFUSED . . . . 9995<br>OTHER 9996	KG. ☐☐.☐☐<br><br>NOT PRESENT . . . . . 9994<br>REFUSED . . . . 9995<br>OTHER 9996
206	HEIGHT IN CENTIMETERS	CM. ☐☐☐.☐<br><br>NOT PRESENT . . . 9994<br>REFUSED . . . . 9995<br>OTHER 9996	CM. ☐☐☐.☐<br><br>NOT PRESENT . . . 9994<br>REFUSED . . . . 9995<br>OTHER 9996	CM. ☐☐☐.☐<br><br>NOT PRESENT . . . 9994<br>REFUSED . . . . 9995<br>OTHER 9996
207	MEASURED LYING DOWN OR STANDING UP?	LYING DOWN . . . . 1<br>STANDING UP . . . . 2<br>NOT MEASURED 3	LYING DOWN . . . . 1<br>STANDING UP . . . . 2<br>NOT MEASURED 3	LYING DOWN . . . . 1<br>STANDING UP . . . . 2<br>NOT MEASURED 3
208	CHECK 203:<br>IS CHILD AGE 0-5 MONTHS, I.E., WAS CHILD BORN IN MONTH OF INTERVIEW OR FIVE PREVIOUS MONTHS?	0-5 MONTHS . . . . 1<br>(GO TO 203 FOR NEXT CHILD OR, IF NO MORE CHILDREN, GO TO 214)<br><br>OLDER 2	0-5 MONTHS . . . . 1<br>(GO TO 203 FOR NEXT CHILD OR, IF NO MORE CHILDREN, GO TO 214)<br><br>OLDER 2	0-5 MONTHS . . . . 1<br>(GO TO 203 IN FIRST COLUMN OF NEW QUESTIONNAIRE; OR, IF NO MORE CHILDREN, GO TO 214)<br>OLDER 2
209	LINE NUMBER OF PARENT/ OTHER ADULT RESPONSIBLE FOR THE CHILD (FROM COLUMN 1 OF HOUSEHOLD SCHEDULE) RECORD '00' IF NOT LISTED.	LINE NUMBER . . . . . ☐☐	LINE NUMBER . . . . . ☐☐	LINE NUMBER . . . . . ☐☐

WEIGHT, HEIGHT AND HEMOGLOBIN MEASUREMENT FOR CHILDREN AGE 0-5

		CHILD 4	CHILD 5	CHILD 6
210	ASK CONSENT FOR ANEMIA TEST FROM PARENT/OTHER ADULT IDENTIFIED IN 209 AS RESPONSIBLE FOR CHILD.	As part of this survey, we are asking people all over the country to take an anemia test. Anemia is a serious health problem that usually results from poor nutrition, infection, or chronic disease. This survey will assist the government to develop programs to prevent and treat anemia.<br><br>We ask that all children born in 2009 or later take part in anemia testing in this survey and give a few drops of blood from a finger or heel. The equipment used to take the blood is clean and completely safe. It has never been used before and will be thrown away after each test.<br><br>The blood will be tested for anemia immediately, and the result told to you right away. The result will be kept strictly confidential and will not be shared with anyone other than members of our survey team.<br>Do you have any questions?<br>You can say yes to the test, or you can say no. It is up to you to decide.<br>Will you allow (NAME(S) OF CHILD(REN) to participate in the anemia test?		
211	CIRCLE THE APPROPRIATE CODE AND SIGN YOUR NAME.	GRANTED 1<br>________ (SIGN)<br>REFUSED 2	GRANTED 1<br>________ (SIGN)<br>REFUSED 2	GRANTED 1<br>________ (SIGN)<br>REFUSED 2
212	RECORD HEMOGLOBIN LEVEL HERE AND IN THE ANEMIA PAMPHLET.	G/DL [ ][ ].[ ]<br><br>NOT PRESENT . . . . . . .994<br>REFUSED . . . . .995<br>OTHER 996	G/DL [ ][ ].[ ]<br><br>NOT PRESENT . . . . . . .994<br>REFUSED . . . . .995<br>OTHER 996	G/DL [ ][ ].[ ]<br><br>NOT PRESENT . . . . . . .994<br>REFUSED . . . . .995<br>OTHER 996
213	GO BACK TO 203 IN NEXT COLUMN OF THIS QUESTIONNAIRE OR IN THE FIRST COLUMN OF AN ADDITIONAL QUESTIONNAIRE; IF NO MORE CHILDREN, GO TO 214.			

WEIGHT, HEIGHT, AND HEMOGLOBIN MEASUREMENT FOR WOMEN AGE 15-49

214	CHECK COLUMN 9 IN HOUSEHOLD SCHEDULE. RECORD THE LINE NUMBER AND NAME FOR ALL ELIGIBLE WOMEN IN 215.<br>IF THERE ARE MORE THAN THREE WOMEN, USE ADDITIONAL QUESTIONNAIRE(S).			
		WOMAN 1	WOMAN 2	WOMAN 3
215	LINE NUMBER FROM COLUMN 9<br>NAME FROM COLUMN 2	LINE NUMBER . . . . [ ][ ]<br>NAME ____	LINE NUMBER . . . . [ ][ ]<br>NAME ____	LINE NUMBER . . . . [ ][ ]<br>NAME ____
216	WEIGHT IN KILOGRAMS	KG. [ ][ ][ ].[ ][ ]<br>NOT PRESENT . . . . 99994<br>REFUSED . . . . 99995<br>OTHER 99996	KG. [ ][ ][ ].[ ][ ]<br>NOT PRESENT . . . . 99994<br>REFUSED . . . . 99995<br>OTHER 99996	KG. [ ][ ][ ].[ ][ ]<br>NOT PRESENT . . . . 99994<br>REFUSED . . . . 99995<br>OTHER 99996
217	HEIGHT IN CENTIMETERS	CM. [ ][ ][ ].[ ]<br>NOT PRESENT . . . . 9994<br>REFUSED . . . . 9995<br>OTHER 9996	CM. [ ][ ][ ].[ ]<br>NOT PRESENT . . . . 9994<br>REFUSED . . . . 9995<br>OTHER 9996	CM. [ ][ ][ ].[ ]<br>NOT PRESENT . . . . 9994<br>REFUSED . . . . 9995<br>OTHER 9996
218	AGE: CHECK COLUMN 7.	15-17 YEARS . . . . 1<br>18-49 YEARS . . . . 2<br>(GO TO 223)	15-17 YEARS . . . . 1<br>18-49 YEARS . . . . 2<br>(GO TO 223)	15-17 YEARS . . . . 1<br>18-49 YEARS . . . . 2<br>(GO TO 223)
219	MARITAL STATUS: CHECK COLUMN 8.	CODE 4 (NEVER IN UNION) . . . . . . 1<br>OTHER . . . . 2<br>(GO TO 223)	CODE 4 (NEVER IN UNION) . . . . . . 1<br>OTHER . . . . 2<br>(GO TO 223)	CODE 4 (NEVER IN UNION) . . . . . . 1<br>OTHER . . . . 2<br>(GO TO 223)
220	RECORD LINE NUMBER OF PARENT/OTHER ADULT RESPON-SIBLE FOR ADOLESCENT. RECORD '00' IF NOT LISTED.	LINE NUMBER OF PARENT OR OTHER RESPONSIBLE ADULT [ ][ ]	LINE NUMBER OF PARENT OR OTHER RESPONSIBLE ADULT [ ][ ]	LINE NUMBER OF PARENT OR OTHER RESPONSIBLE ADULT [ ][ ]
221	ASK CONSENT FOR ANEMIA TEST FROM PARENT/ OTHER ADULT IDENTIFIED IN 220 AS RESPONSIBLE FOR NEVER IN UNION WOMEN AGE 15-17.	As part of this survey, we are asking people all over the country to take an anemia test. Anemia is a serious health problem that usually results from poor nutrition, infection, or chronic disease. This survey will assist the government to develop programs to prevent and treat anemia. For the anemia testing, we will need a few drops of blood from a finger. The equipment used to take the blood is clean and completely safe. It has never been used before and will be thrown away after each test. The blood will be tested for anemia immediately, and the result will be told to you and (NAME OF ADOLESCENT) right away. The result will be kept strictly confidential and will not be shared with anyone other than members of our survey team.<br>Do you have any questions?<br>You can say yes to the test for (NAME OF ADOLESCENT), or you can say no. It is up to you to decide.<br>Will you allow (NAME OF ADOLESCENT) to take the anemia test?		
222	CIRCLE THE APPROPRIATE CODE AND SIGN YOUR NAME.	GRANTED . . . . 1<br>____ (SIGN)<br>PARENT/OTHER RESPONSIBLE ADULT REFUSED . . . . 2<br>(IF REFUSED, GO TO 242)	GRANTED . . . . 1<br>____ (SIGN)<br>PARENT/OTHER RESPONSIBLE ADULT REFUSED . . . . 2<br>(IF REFUSED, GO TO 242)	GRANTED . . . . 1<br>____ (SIGN)<br>PARENT/OTHER RESPONSIBLE ADULT REFUSED . . . . 2<br>(IF REFUSED, GO TO 242)

WEIGHT, HEIGHT, AND HEMOGLOBIN MEASUREMENT FOR WOMEN AGE 15-49

		WOMAN 1	WOMAN 2	WOMAN 3
223	ASK CONSENT FOR ANEMIA TEST FROM RESPONDENT.	As part of this survey, we are asking people all over the country to take an anemia test. Anemia is a serious health problem that usually results from poor nutrition, infection, or chronic disease. This survey will assist the government to develop programs to prevent and treat anemia. For the anemia testing, we will need a few drops of blood from a finger. The equipment used to take the blood is clean and completely safe. It has never been used before and will be thrown away after each test. The blood will be tested for anemia immediately, and the result will be told to you right away. The result will be kept strictly confidential and will not be shared with anyone other than members of our survey team.<br><br>Do you have any questions?<br>You can say yes to the test, or you can say no. It is up to you to decide.<br>Will you take the anemia test?		
224	CIRCLE THE APPROPRIATE CODE AND SIGN YOUR NAME.	GRANTED . . . . . 1<br>______ (SIGN)<br>RESPONDENT REFUSED . . . . . . 2<br>(IF REFUSED, GO TO 242)	GRANTED . . . . . 1<br>______ (SIGN)<br>RESPONDENT REFUSED . . . . . . 2<br>(IF REFUSED, GO TO 242)	GRANTED . . . . . 1<br>______ (SIGN)<br>RESPONDENT REFUSED . . . . . . 2<br>(IF REFUSED, GO TO 242)
225	PREGNANCY STATUS: CHECK 226 IN WOMAN'S QUESTIONNAIRE OR ASK: Are you pregnant?	YES . . . . . 1<br>NO . . . . . 2<br>DK 8	YES . . . . . 1<br>NO . . . . . 2<br>DK 8	YES . . . . . 1<br>NO . . . . . 2<br>DK 8
240	RECORD HEMO-GLOBIN LEVEL HERE AND IN ANEMIA PAMPHLET.	G/DL . . . . . [ ][ ].[ ]<br>NOT PRESENT . . . . . 994<br>REFUSED . . . . . 995<br>OTHER 996	G/DL . . . . . [ ][ ].[ ]<br>NOT PRESENT . . . . . 994<br>REFUSED . . . . . 995<br>OTHER 996	G/DL . . . . . [ ][ ].[ ]<br>NOT PRESENT . . . . . 994<br>REFUSED . . . . . 995<br>OTHER 996
242	GO BACK TO 216 IN NEXT COLUMN OF THIS QUESTIONNAIRE OR IN THE FIRST COLUMN OF AN ADDITIONAL QUESTIONNAIRE; IF NO MORE WOMEN GO TO 242A.			
242A	CHECK THE COVER PAGE IF THE HOUSEHOLD IS IN THE CLUSTER SELECTED FOR MICRONUTRIENT TEST.<br>HOUSEHOLD IS IN THE CLUSTER SELECTED FOR MICRONUTRIENT TEST [ ] → GO TO 243<br>HOUSEHOLD IS NOT IN THE CLUSTER SELECTED FOR MICRONUTRIENT TEST [ ] → END HOUSEHOLD INTERVIEW			

243	**PERMISSION TO REVISIT BY MICRONUTRIENT TEAM**

In the next few days, my colleagues who are working with the ministry of health would like to revisit your household to conduct a micronutrient study and will collect small quantity of blood, urine and stool samples from some members of your household. You don't have to permit the visit, but we hope you will agree since your household participation is very important.
In case you need more information about the revisit, you may contact the person listed on this card.

GIVE CARD WITH CONTACT INFORMATION

SIGNATURE OF INTERVIEWER:________________________ DATE: ______________

RESPONDENT AGREES TO BE REVISITED . . . 1

RESPONDENT DOES NOT AGREE TO BE REVISITED . . . 2 → END HOUSEHOLD INTERVIEW

COMPLETE COVERPAGE OF MICRONUTRIENT QUESTIONNAIRE USING HOUSEHOLD QUESTIONS	A
RECORDE THE INFORMATION ABOUT ALL ELIGIBLE CHILDREN **(251-256)** AND WOMEN **(270-274)** IN THE MICRONUTRIENT QUESTIONNAIRE	B
CONTACT THE MICRONUTRIENT COORDINATOR TO SEND A MICRONUTRIENT TEAM AND PROVIDE HER/HIM THE MICRONUTRIENT QUESTIONNAIRE	C

INTERVIEWER'S OBSERVATIONS

TO BE FILLED IN AFTER COMPLETING INTERVIEW

COMMENTS ABOUT RESPONDENT:

COMMENTS ON SPECIFIC QUESTIONS:

ANY OTHER COMMENTS:

SUPERVISOR'S OBSERVATIONS

NAME OF SUPERVISOR: ____________ DATE: ____________

CAMBODIA DEMOGRAPHIC AND HEALTH SURVEYS 2014
WOMAN'S QUESTIONNAIRE

MINISTRY OF PLANNING
NATIONAL INSTITUTE OF STATISTICS

MINISTRY OF HEALTH
GENERAL DIRECTORATE FOR HEALTH

IDENTIFICATION

DOMAIN . . . .

NAME OF HOUSEHOLD HEAD ____________

PROVINCE ____________

DISTRICT ____________

COMMUNE ____________

VILLAGE ____________

CLUSTER NUMBER . . . .

HOUSEHOLD NUMBER . . . .

NAME AND LINE NUMBER OF WOMAN ____________

INTERVIEWER VISITS

	1	2	3	FINAL VISIT
DATE	______	______	______	DAY<br>MONTH<br>YEAR 2 0
INTERVIEWER'S NAME	______	______	______	INT. NUMBER
RESULT*	______	______	______	RESULT*
NEXT VISIT: DATE	______	______		TOTAL NUMBER OF VISITS
TIME	______	______		

*RESULT CODES:

1	COMPLETED	4	REFUSED		
2	NOT AT HOME	5	PARTLY COMPLETED	7	OTHER ______ (SPECIFY)
3	POSTPONED	6	INCAPACITATED		

LANGUAGE OF QUESTIONNAIRE, LANGUAGE OF INTERVIEW, NATIVE :

KHMER LANGUAGE 1

OTHER LANGUAGE ______ 2
(SPECIFY)

USED TRANSLATOR:

YES 1

NO 2

SUPERVISOR	FIELD EDITOR	OFFICE EDITOR	KEYED BY
NAME ______	NAME ______		
DATE: ______	DATE: ______		

SECTION 1. RESPONDENT'S BACKGROUND

INTRODUCTION AND CONSENT

INFORMED CONSENT

Hello. My name is ______________________________________. I am working with the ministry of health and ministry of planning. We are conducting a survey about health all over Cambodia. The information we collect will help the government to plan health services. Your household was selected for the survey. The questions usually take about 30 to 60 minutes. All of the answers you give will be confidential and will not be shared with anyone other than members of our survey team. You don't have to be in the survey, but we hope you will agree to answer the questions since your views are important. If I ask you any question you don't want to answer, just let me know and I will go on to the next question or you can stop the interview at any time.

In case you need more information about the survey, you may contact the person listed on the card that has already been given to your household.

Do you have any questions? May I begin the interview now?

SIGNATURE OF INTERVIEWER: ______________________________ DATE: ________________

RESPONDENT AGREES TO BE INTERVIEWED . . . 1 ↓ RESPONDENT DOES NOT AGREE TO BE INTERVIEWED . . . 2 → END

NO.	QUESTIONS AND FILTERS	CODING CATEGORIES	SKIP
101	RECORD THE TIME.	HOUR . . . . [ ][ ]<br>MINUTES . . . . [ ][ ]	
102	In what month and year were you born?<br><br>IF RESPONDENT DOES NOT KNOW GREGORIAN MONTH AND YEAR OF BIRTH, ASK FOR KHMER MONTH AND YEAR. USE DATE CONVERSION CHART.<br><br>______________________________<br>(SPECIFY KHMER MONTH AND YEAR OF BIRTH)	GREGORIAN MONTH . . . . . [ ][ ]<br>DON'T KNOW MONTH . . . . 98<br>GREGORIAN YEAR [ ][ ][ ][ ]<br>DON'T KNOW YEAR 9998	
103	How old were you at your last birthday?<br><br>COMPARE AND CORRECT 102 AND/OR 103 IF INCONSISTENT.	AGE IN COMPLETED YEARS [ ][ ]	
104	Have you ever attended school?	YES . . . . 1<br>NO 2	 → 108
105	What is the highest level of school you attended: primary, secondary, or higher?	PRIMARY . . . . 1<br>LOWER SECONDARY . . . . 2<br>UPPER SECONDARY . . . . 3<br>HIGHER 4	
106	What is the highest (grade/form/year) you completed at that level?<br><br>IF COMPLETED LESS THAN ONE YEAR AT THAT LEVEL, RECORD '00'.	GRADE/FORM/YEAR . . . . [ ][ ]	

NO.	QUESTIONS AND FILTERS	CODING CATEGORIES	SKIP
107	CHECK 105:<br>PRIMARY ☐ ↓ SECONDARY OR HIGHER ☐		→ 110
108	Now I would like you to read this sentence to me.<br><br>SHOW CARD TO RESPONDENT.<br><br>IF RESPONDENT CANNOT READ WHOLE SENTENCE, PROBE:<br>Can you read any part of the sentence to me?	CANNOT READ AT ALL . . . . 1<br>ABLE TO READ ONLY PARTS OF SENTENCE . . . . 2<br>ABLE TO READ WHOLE SENTENCE 3<br>NO CARD WITH REQUIRED LANGUAGE ______ 4<br>(SPECIFY LANGUAGE)<br>BLIND/VISUALLY IMPAIRED 5	
109	CHECK 108:<br>CODE '2', '3' OR '4' CIRCLED ☐ ↓ CODE '1' OR '5' CIRCLED ☐		→ 111
110	Do you read a newspaper or magazine at least once a week, less than once a week or not at all?	AT LEAST ONCE A WEEK . . . . 1<br>LESS THAN ONCE A WEEK . . . . 2<br>NOT AT ALL 3	
111	Do you listen to the radio at least once a week, less than once a week or not at all?	AT LEAST ONCE A WEEK . . . . 1<br>LESS THAN ONCE A WEEK . . . . 2<br>NOT AT ALL 3	
112	Do you watch television at least once a week, less than once a week or not at all?	AT LEAST ONCE A WEEK . . . . 1<br>LESS THAN ONCE A WEEK . . . . 2<br>NOT AT ALL 3	
112A	Do you access to internet, at least once a week, less than once a week or not at all?	AT LEAST ONCE A WEEK . . . . 1<br>LESS THAN ONCE A WEEK . . . . 2<br>NOT AT ALL 3	
113	What is your religion?	BUDDHIST . . . . 1<br>MOSLEM . . . . 2<br>CHRISTIAN . . . . 3<br>OTHER 4	
115	In the last 12 months, how many times have you been away from home for one or more nights?	NUMBER OF TIMES . . . . ☐☐<br><br>NONE 00	 <br>→ 201
116	In the last 12 months, have you been away from home for more than one month at a time?	YES . . . . 1<br>NO 2	

SECTION 2. REPRODUCTION

NO.	QUESTIONS AND FILTERS	CODING CATEGORIES	SKIP
201	Now I would like to ask about all the births you have had during your life. Have you ever given birth?	YES . . . . . 1<br>NO 2	 → 206
202	Do you have any sons or daughters to whom you have given birth who are now living with you?	YES . . . . . 1<br>NO 2	 → 204
203	How many sons live with you?<br>And how many daughters live with you?<br>IF NONE, RECORD '00'.	SONS AT HOME . . . . . [ ][ ]<br>DAUGHTERS AT HOME . . . . . [ ][ ]	
204	Do you have any sons or daughters to whom you have given birth who are alive but do not live with you?	YES . . . . . 1<br>NO 2	 → 206
205	How many sons are alive but do not live with you?<br>And how many daughters are alive but do not live with you?<br>IF NONE, RECORD '00'.	SONS ELSEWHERE . . . . . [ ][ ]<br>DAUGHTERS ELSEWHERE [ ][ ]	
205A	Where do your sons or daughters who do not live with you live?	EXTENDED FAMILY/CAMP . . . . . A<br>NEIGHBOR . . . . . B<br>ORPHANAGE/NGO . . . . . C<br>TEMPLE (WAT) . . . . . D<br>OTHER ________ E<br>(SPECIFY)<br>DON'T KNOW X	
206	Have you ever given birth to a boy or girl who was born alive but later died?<br>IF NO, PROBE: Any baby who cried or showed signs of life but did not survive?	YES . . . . . 1<br>NO 2	 → 208
207	How many boys have died?<br>And how many girls have died?<br>IF NONE, RECORD '00'.	BOYS DEAD . . . . . [ ][ ]<br>GIRLS DEAD . . . . . [ ][ ]	
208	SUM ANSWERS TO 203, 205, AND 207, AND ENTER TOTAL.<br>IF NONE, RECORD '00'.	TOTAL BIRTHS . . . . . [ ][ ]	
209	CHECK 208:<br>Just to make sure that I have this right: you have had in TOTAL _____ births during your life. Is that correct?<br>YES [ ] ↓ NO [ ] → PROBE AND CORRECT 201-208 AS NECESSARY.		
210	CHECK 208:<br>ONE OR MORE BIRTHS [ ] ↓ NO BIRTHS [ ]		→ 226

211	Now I would like to record the names of all your births, whether still alive or not, starting with the first one you had.<br>RECORD NAMES OF ALL THE BIRTHS IN 212. RECORD TWINS AND TRIPLETS ON SEPARATE ROWS.<br>(IF THERE ARE MORE THAN 12 BIRTHS, USE AN ADDITIONAL QUESTIONNAIRE, STARTING WITH THE SECOND ROW).

212	213	214	215	216	217<br>IF ALIVE:	218<br>IF ALIVE:	219<br>IF ALIVE:	220<br>IF DEAD:	221
What name was given to your next baby?<br><br>(NAME)	Is (NAME) a boy or a girl?	Were any of these births twins?	In what month and year was (NAME) born?<br><br>PROBE:<br>When is his/her birthday?	Is (NAME) still alive?	How old was (NAME) at his/her last birthday?<br><br>RECORD AGE IN COM-PLETED YEARS.	Is (NAME) living with you?	RECORD HOUSE-HOLD LINE NUMBER OF CHILD (RECORD '00' IF CHILD NOT LISTED IN HOUSE-HOLD).	How old was (NAME) when he/she died?<br><br>IF '1 YR', PROBE:<br>How many months old was (NAME)?<br>RECORD DAYS IF LESS THAN 1 MONTH; MONTHS IF LESS THAN TWO YEARS; OR YEARS.	Were there any other live births between (NAME OF PREVIOUS BIRTH) and (NAME), including any children who died after birth?
01	BOY 1<br>GIRL 2	SING 1<br>MULT 2	MONTH<br>YEAR	YES . . 1<br>NO . . . 2<br>↓<br>220	AGE IN YEARS	YES . . . 1<br>NO 2	LINE NUMBER<br>↓<br>(NEXT BIRTH)	DAYS . . . 1<br>MONTHS 2<br>YEARS . . 3	
02	BOY 1<br>GIRL 2	SING 1<br>MULT 2	MONTH<br>YEAR	YES . . 1<br>NO . . . 2<br>↓<br>220	AGE IN YEARS	YES . . . 1<br>NO 2	LINE NUMBER<br>↓<br>(GO TO 221)	DAYS . . . 1<br>MONTHS 2<br>YEARS . . 3	YES . . . . 1<br>ADD BIRTH<br>NO . . . . . 2<br>NEXT BIRTH
03	BOY 1<br>GIRL 2	SING 1<br>MULT 2	MONTH<br>YEAR	YES . . 1<br>NO . . . 2<br>↓<br>220	AGE IN YEARS	YES . . . 1<br>NO 2	LINE NUMBER<br>↓<br>(GO TO 221)	DAYS . . . 1<br>MONTHS 2<br>YEARS . . 3	YES . . . . 1<br>ADD BIRTH<br>NO . . . . . 2<br>NEXT BIRTH
04	BOY 1<br>GIRL 2	SING 1<br>MULT 2	MONTH<br>YEAR	YES . . 1<br>NO . . . 2<br>↓<br>220	AGE IN YEARS	YES . . . 1<br>NO 2	LINE NUMBER<br>↓<br>(GO TO 221)	DAYS . . . 1<br>MONTHS 2<br>YEARS . . 3	YES . . . . 1<br>ADD BIRTH<br>NO . . . . . 2<br>NEXT BIRTH
05	BOY 1<br>GIRL 2	SING 1<br>MULT 2	MONTH<br>YEAR	YES . . 1<br>NO . . . 2<br>↓<br>220	AGE IN YEARS	YES . . . 1<br>NO 2	LINE NUMBER<br>↓<br>(GO TO 221)	DAYS . . . 1<br>MONTHS 2<br>YEARS . . 3	YES . . . . 1<br>ADD BIRTH<br>NO . . . . . 2<br>NEXT BIRTH
06	BOY 1<br>GIRL 2	SING 1<br>MULT 2	MONTH<br>YEAR	YES . . 1<br>NO . . . 2<br>↓<br>220	AGE IN YEARS	YES . . . 1<br>NO 2	LINE NUMBER<br>↓<br>(GO TO 221)	DAYS . . . 1<br>MONTHS 2<br>YEARS . . 3	YES . . . . 1<br>ADD BIRTH<br>NO . . . . . 2<br>NEXT BIRTH
07	BOY 1<br>GIRL 2	SING 1<br>MULT 2	MONTH<br>YEAR	YES . . 1<br>NO . . . 2<br>↓<br>220	AGE IN YEARS	YES . . . 1<br>NO 2	LINE NUMBER<br>↓<br>(GO TO 221)	DAYS . . . 1<br>MONTHS 2<br>YEARS . . 3	YES . . . . 1<br>ADD BIRTH<br>NO . . . . . 2<br>NEXT BIRTH

212	213	214	215	216	217 IF ALIVE:	218 IF ALIVE:	219 IF ALIVE:	220 IF DEAD:	221
What name was given to your first/next baby? (NAME)	Is (NAME) a boy or a girl?	Were any of these births twins?	In what month and year was (NAME) born? PROBE: When is his/her birthday?	Is (NAME) still alive?	How old was (NAME) at his/her last birthday? RECORD AGE IN COM-PLETED YEARS.	Is (NAME) living with you?	RECORD HOUSE-HOLD LINE NUMBER OF CHILD (RECORD '00' IF CHILD NOT LISTED IN HOUSE-HOLD).	How old was (NAME) when he/she died? IF '1 YR', PROBE: How many months old was (NAME)? RECORD DAYS IF LESS THAN 1 MONTH; MONTHS IF LESS THAN TWO YEARS; OR YEARS.	Were there any other live births between (NAME OF PREVIOUS BIRTH) and (NAME), including any children who died after birth?
08	BOY 1<br>GIRL 2	SING 1<br>MULT 2	MONTH<br>YEAR	YES . . 1<br>NO . . . 2 → 220	AGE IN YEARS	YES . . . 1<br>NO 2	LINE NUMBER<br>(GO TO 221)	DAYS . . . 1<br>MONTHS 2<br>YEARS . . 3	YES . . . . 1 ADD BIRTH<br>NO . . . . . 2 NEXT BIRTH
09	BOY 1<br>GIRL 2	SING 1<br>MULT 2	MONTH<br>YEAR	YES . . 1<br>NO . . . 2 → 220	AGE IN YEARS	YES . . . 1<br>NO 2	LINE NUMBER<br>(GO TO 221)	DAYS . . . 1<br>MONTHS 2<br>YEARS . . 3	YES . . . . 1 ADD BIRTH<br>NO . . . . . 2 NEXT BIRTH
10	BOY 1<br>GIRL 2	SING 1<br>MULT 2	MONTH<br>YEAR	YES . . 1<br>NO . . . 2 → 220	AGE IN YEARS	YES . . . 1<br>NO 2	LINE NUMBER<br>(GO TO 221)	DAYS . . . 1<br>MONTHS 2<br>YEARS . . 3	YES . . . . 1 ADD BIRTH<br>NO . . . . . 2 NEXT BIRTH
11	BOY 1<br>GIRL 2	SING 1<br>MULT 2	MONTH<br>YEAR	YES . . 1<br>NO . . . 2 → 220	AGE IN YEARS	YES . . . 1<br>NO 2	LINE NUMBER<br>(GO TO 221)	DAYS . . . 1<br>MONTHS 2<br>YEARS . . 3	YES . . . . 1 ADD BIRTH<br>NO . . . . . 2 NEXT BIRTH
12	BOY 1<br>GIRL 2	SING 1<br>MULT 2	MONTH<br>YEAR	YES . . 1<br>NO . . . 2 → 220	AGE IN YEARS	YES . . . 1<br>NO 2	LINE NUMBER<br>(GO TO 221)	DAYS . . . 1<br>MONTHS 2<br>YEARS . . 3	YES . . . . 1 ADD BIRTH<br>NO . . . . . 2 NEXT BIRTH

222	Have you had any live births since the birth of (NAME OF LAST BIRTH)? IF YES, RECORD BIRTH(S) IN TABLE.	YES . . . . 1<br>NO 2
223	COMPARE 208 WITH NUMBER OF BIRTHS IN HISTORY ABOVE AND MARK:<br>NUMBERS ARE SAME ☐ ↓ NUMBERS ARE DIFFERENT ☐ → (PROBE AND RECONCILE)	
224	CHECK 215:<br>ENTER THE NUMBER OF BIRTHS IN 2009 OR LATER.	NUMBER OF BIRTHS . . . . ☐<br>NONE . . . . 8 → 226

NO.	QUESTIONS AND FILTERS	CODING CATEGORIES	SKIP
225	C FOR EACH BIRTH SINCE **JANUARY 2009**, ENTER 'B' IN THE MONTH OF BIRTH IN THE CALENDAR. WRITE THE NAME OF THE CHILD TO THE LEFT OF THE 'B' CODE. FOR EACH BIRTH, ASK THE NUMBER OF MONTHS THE PREGNANCY LASTED AND RECORD 'P' IN EACH OF THE PRECEDING MONTHS ACCORDING TO THE DURATION OF PREGNANCY. (NOTE: THE NUMBER OF 'P's MUST BE ONE LESS THAN THE NUMBER OF MONTHS THAT THE PREGNANCY LASTED.)		
226	Are you pregnant now?	YES . . . . . 1<br>NO . . . . . 2<br>UNSURE 8	 NO/UNSURE → 230
227	How many months pregnant are you?<br>RECORD NUMBER OF COMPLETED MONTHS.<br>C ENTER 'P's IN THE CALENDAR, BEGINNING WITH THE MONTH OF INTERVIEW AND FOR THE TOTAL NUMBER OF COMPLETED MONTHS.	MONTHS . . . . . [ ][ ]	
228	When you got pregnant, did you want to get pregnant at that time?	YES . . . . . 1<br>NO 2	YES → 230
229	Did you want to have a baby later on or did you not want any (more) children?	LATER . . . . . 1<br>NO MORE 2	
230	Have you ever had a pregnancy that miscarried, was aborted, or ended in a stillbirth?	YES . . . . . 1<br>NO 2	 NO → 238
231	When did the last such pregnancy end?	MONTH . . . . . [ ][ ]<br>YEAR . . . . . [ ][ ][ ][ ]	
232	CHECK 231:<br>LAST PREGNANCY ENDED IN JAN. 2009 OR LATER [ ] ↓<br>LAST PREGNANCY ENDED BEFORE JAN. 2009 [ ] → 235A		235A
233	How many months pregnant were you when the last such pregnancy ended?<br>C RECORD NUMBER OF COMPLETED MONTHS. ENTER 'T' IN THE CALENDAR IN THE MONTH THAT THE PREGNANCY TERMINATED AND 'P' FOR THE REMAINING NUMBER OF COMPLETED MONTHS.	MONTHS . . . . . [ ][ ]	
233A	Did this pregnancy end in an induced abortion?	YES . . . . . 1<br>NO 2	 NO → 234
233B	What was the method used for that induced abortion	SURGICAL METHODS<br>VACUME ASPIRATION . . . . . A<br>CURETTAGE . . . . . B<br>DILATATION AND EVACUATION . . . C<br><br>MEDICAL METHODS<br>ORAL PILL/TABLET . . . . . D<br>VAGINAL PILL/TABLET . . . . . E<br>INJECTABLE . . . . . F<br>INTRAUTERINE . . . . . G<br><br>TRADITIONAL METHODS . . . . . H<br><br>OTHER METHODS X	

NO.	QUESTIONS AND FILTERS	CODING CATEGORIES	SKIP
233C	In the seven days after the abortion did you experience:<br>fever?<br>excessive bleeding?	YES NO DK<br>FEVER . . . . 1 2 8<br>BLEEDING 1 2 8	
233D	Did anyone help you to initiate the induced abortion?<br><br>IF YES: Who helped you to initiate the abortion?<br><br>Anyone else?<br><br>RECORD ALL PERSONS ASSISTING.	HEALTH PROFESSIONAL<br>DOCTOR/MEDICAL ASSISTANT . . . A<br>NURSE . . . . B<br>MIDWIFE . . . . C<br>OTHER HEALTH PROF. . . . . D<br><br>OTHER PERSON<br>TRADITIONAL BIRTH ATTENDANT . E<br>PHARMACIST . . . . F<br>KRU KHMER/MAGICIAN . . . . G<br>RELATIVE/FRIEND . . . . H<br><br>OTHER ______________________ X<br>(SPECIFY)<br>NO ONE . . . . Y	
233E	Where did the induced abortion take place?<br><br>IF HOSPITAL, PROBE:<br>Do you mean a permanent building where health workers are present everyday?<br><br>IF YES: Was it a provincial hospital, district hospital, health center, or private hospital?<br><br>WRITE THE NAME OF THE PLACE AND CIRCLE THE APPROPRIATE CODE.<br><br>______________________________<br>(NAME OF PLACE)	PUBLIC MEDICAL SECTOR<br>NATIONAL HOSPITAL (PP) . . . . 11<br>PROVINCIAL HOSPITAL (RH) . . . . . 12<br>DISTRICT HOSPITAL (RH) . . . . 13<br>HEALTH CENTER . . . . 14<br>HEALTH POST . . . . 15<br>MILITARY HOSPITAL . . . . 16<br>OTHER PUBLIC . . . . 17<br><br>PRIVATE MEDICAL SECTOR<br>PRIVATE HOSPITAL . . . . 21<br>PRIVATE CLINIC . . . . 22<br>OTHER PRIVATE MEDICAL . . . . . 26<br><br>HOME<br>YOUR HOME . . . . 31<br>OTHER HOME . . . . 32<br><br>OTHER PLACE ________________ 96<br>(SPECIFY)	
233F	Was anyone present to help you at the time of the abortion?<br><br>IF YES: Who was present to help you?<br><br>Anyone else?<br><br>RECORD ALL PERSONS ASSISTING.	HEALTH PROFESSIONAL<br>DOCTOR/MEDICAL ASSISTANT . . . A<br>NURSE . . . . B<br>MIDWIFE . . . . C<br>OTHER HEALTH PROF. . . . . D<br><br>OTHER PERSON<br>TRADITIONAL BIRTH ATTENDANT . E<br>PHARMACIST . . . . F<br>KRU KHMER/MAGICIAN . . . . G<br>RELATIVE/FRIEND . . . . H<br><br>OTHER PERSON ______________ X<br>(SPECIFY)<br>NO ONE . . . . Y	
234	Since January 2009 , have you had any other pregnancies that did not result in a live birth?	YES . . . . 1<br>NO 2	 → 236

NO.	QUESTIONS AND FILTERS	CODING CATEGORIES	SKIP
235	ASK THE DATE AND THE DURATION OF PREGNANCY FOR EACH EARLIER NON-LIVE BIRTH PREGNANCY BACK TO JANUARY 2009.<br>**C** ENTER 'T' IN THE CALENDAR IN THE MONTH THAT EACH PREGNANCY TERMINATED AND 'P' FOR THE REMAINING NUMBER OF COMPLETED MONTHS.		
235A	Since January 2009, how many induced abortion have you had ?	TOTAL NUMBER ABORTIONS SINCE JANUARY 2009	
236	Did you have any miscarriages, abortions or stillbirths that ended before 2009?	YES . . . . 1<br>NO 2	 → 238
237	When did the last such pregnancy that terminated before 2009 end?	MONTH . . . .<br>YEAR	
237A	In total, how many induced abortions have you had in your lifetime?	TOTAL NUMBER ABORTIONS IN LIFETIME	
238	When did your last menstrual period start?<br><br>____________________<br>(DATE, IF GIVEN)	DAYS AGO . . . . 1<br>WEEKS AGO . . . . 2<br>MONTHS AGO . . . . 3<br>YEARS AGO . . . . 4<br>IN MENOPAUSE/ HAS HAD HYSTERECTOMY . . . 994<br>BEFORE LAST BIRTH . . . . 995<br>NEVER MENSTRUATED 996	
239	From one menstrual period to the next, are there certain days when a woman is more likely to become pregnant?	YES . . . . 1<br>NO . . . . 2<br>DON'T KNOW 8	 → 241
240	Is this time just before her period begins, during her period, right after her period has ended, or halfway between two periods?	JUST BEFORE HER PERIOD BEGINS . . . . 1<br>DURING HER PERIOD . . . . 2<br>RIGHT AFTER HER PERIOD HAS ENDED . . . . 3<br>HALFWAY BETWEEN TWO PERIODS . . . . 4<br>OTHER ________________ 6<br>(SPECIFY)<br>DON'T KNOW 8	
241	Do you take iron tablet like this (or these) every week?<br>SHOW THE PICTURE OF IRON TABLET TO RESPONDENT	YES . . . . 1<br>NO 2	

SECTION 3. CONTRACEPTION

301	Now I would like to talk about family planning - the various ways or methods that a couple can use to delay or avoid a pregnancy.<br><br>Have you ever heard of (METHOD)?		
01	**Female Sterilization**. PROBE: Women can have an operation to avoid having any more children.	YES . . . . 1<br>NO 2	
02	**Male Sterilization**. PROBE: Men can have an operation to avoid having any more children.	YES . . . . 1<br>NO 2	
03	**IUD** PROBE: Women can have a loop or coil placed inside them by a doctor, midwife, or a nurse.	YES . . . . 1<br>NO 2	
04	**Injectables**. PROBE: Women can have an injection by a health provider that stops them from becoming pregnant for one or more months.	YES . . . . 1<br>NO 2	
05	**Implants**. PROBE: Women can have one or more small rods placed in their upper arm by a doctor, a midwife, or nurse which can prevent pregnancy for one or more years.	YES . . . . 1<br>NO 2	
06	**DAILY PILL Women can take a pill every day. PROBE:** Women can take a pill every day to avoid becoming pregnant.	YES . . . . 1<br>NO 2	
07	**MONTHLY PILL or CHINESE PILL. PROBE:** Women can take a pill once a month to avoid becoming pregnant.	YES . . . . 1<br>NO 2	
08	**Condom**. PROBE: Men can put a rubber sheath on their penis before sexual intercourse.	YES . . . . 1<br>NO 2	
09	**Female Condom**. PROBE: Women can place a sheath in their vagina before sexual intercourse.	YES . . . . 1<br>NO 2	
10	**Lactational Amenorrhea Method** (LAM)	YES . . . . 1<br>NO 2	
11	**Rhythm Method**. PROBE: Every month that a woman is sexually active she can avoid pregnancy by not having sexual intercourse on the days of the month she is most likely to get pregnant.	YES . . . . 1<br>NO 2	
12	**Withdrawal**. PROBE: Men can be careful and pull out before climax.	YES . . . . 1<br>NO 2	
13	**Emergency Contraception**. PROBE: As an emergency measure, within three days after they have unprotected sexual intercourse, women can take special pills to prevent pregnancy.	YES . . . . 1<br>NO 2	
14	Have you heard of any other ways or methods that women or men can use to avoid pregnancy?	YES . . . . 1<br>________<br>(SPECIFY)<br>________<br>(SPECIFY)<br>NO 2	
302	CHECK 226:<br>NOT PREGNANT OR UNSURE ☐ ↓ PREGNANT ☐		→ 311
303	Are you currently doing something or using any method to delay or avoid getting pregnant?	YES . . . . 1<br>NO 2	 → 311

NO.	QUESTIONS AND FILTERS	CODING CATEGORIES	SKIP
304	Which method are you using?<br><br>CIRCLE ALL MENTIONED.<br><br>IF MORE THAN ONE METHOD MENTIONED, FOLLOW SKIP INSTRUCTION FOR HIGHEST METHOD IN LIST.	FEMALE STERILIZATION . . . . A<br>MALE STERILIZATION . . . . B<br>IUD . . . . C<br>INJECTABLES . . . . D<br>IMPLANTS . . . . E<br>DAILY PILL . . . . F<br>MONTHLY METHOD (CHINESE PILL) . G<br>CONDOM . . . . H<br>FEMALE CONDOM . . . . I<br>DIAPHRAGM . . . . J<br>FOAM/JELLY . . . . K<br>LACTATIONAL AMEN. METHOD . . . . . L<br>RHYTHM METHOD . . . . M<br>WITHDRAWAL . . . . N<br>OTHER MODERN METHOD . . . . O<br>OTHER TRADITIONAL METHOD . . . Y	A–B → 307<br>C–E → 308A<br>H → 306<br>I–Y → 308A
305	What is the brand name of the pills you are using?<br><br>IF DON'T KNOW THE BRAND, ASK TO SEE THE PACKAGE.	SREY PICH . . . . 01<br>OK . . . . 02<br><br>OTHER ______________ 96<br>(SPECIFY)<br><br>DON'T KNOW 98	→ 308A
306	What is the brand name of the condoms you are using?<br><br>IF DON'T KNOW THE BRAND, ASK TO SEE THE PACKAGE.	NUMBER ONE . . . . 01<br>OK . . . . 02<br>NEAK KAPEAR/ NO NAME . . . . 03<br>OTHER ______________ 96<br>(SPECIFY)<br><br>DON'T KNOW 98	→ 308A
307	In what facility did the sterilization take place?<br><br>PROBE TO IDENTIFY THE TYPE OF SOURCE.<br><br>IF UNABLE TO DETERMINE IF PUBLIC OR PRIVATE SECTOR, WRITE THE NAME OF THE PLACE.<br><br>______________<br>(NAME OF PLACE)	PUBLIC SECTOR<br>NATIONAL HOSPITAL (PP) . . . . . 11<br>PROVINCIAL HOSPITAL (RH) . . . . . 12<br>DISTRICT HOSPITAL (RH) . . . . 13<br>HEALTH CENTER . . . . 14<br>HEALTH POST . . . . 15<br>MILITARY HOSPITAL . . . . 16<br>OTHER PUBLIC<br>SECTOR ______________ 17<br>(SPECIFY)<br><br>PRIVATE MEDICAL SECTOR<br>PRIVATE HOSPITAL . . . . . 21<br>PRIVATE CLINIC . . . . . 22<br>OTHER PRIVATE MEDICAL<br>SECTOR ______________ 23<br>(SPECIFY)<br><br>OTHER ______________ 96<br>(SPECIFY)<br>DON'T KNOW 98	

NO.	QUESTIONS AND FILTERS	CODING CATEGORIES	SKIP
308	In what month and year was the sterilization performed?		
308A	Since what month and year have you been using (CURRENT METHOD) without stopping?<br><br>PROBE: For how long have you been using (CURRENT METHOD) now without stopping?	MONTH . . . . [ ][ ]<br>YEAR . . . . [ ][ ][ ][ ]	
309	CHECK 308/308A, 215 AND 231:<br><br>ANY BIRTH OR PREGNANCY TERMINATION AFTER MONTH AND YEAR OF START OF USE OF CONTRACEPTION IN 308/308A — YES / NO<br><br>(YES →) GO BACK TO 308/308A, PROBE AND RECORD MONTH AND YEAR AT START OF CONTINUOUS USE OF CURRENT METHOD (MUST BE AFTER LAST BIRTH OR PREGNANCY TERMINATION).		
310	CHECK 308/308A:<br><br>YEAR IS 2009 OR LATER →<br>**C** ENTER CODE FOR METHOD USED IN MONTH OF INTERVIEW IN THE CALENDAR AND IN EACH MONTH BACK TO THE DATE STARTED USING.<br><br>YEAR IS 2008 OR EARLIER →<br>**C** ENTER CODE FOR METHOD USED IN MONTH OF INTERVIEW IN THE CALENDAR AND EACH MONTH BACK TO JANUARY 2009.<br><br>THEN SKIP TO → 322		
311	I would like to ask you some questions about the times you or your partner may have used a method to avoid getting pregnant during the last few years.<br><br>USE CALENDAR TO PROBE FOR EARLIER PERIODS OF USE AND NONUSE, STARTING WITH MOST RECENT USE, BACK TO JANUARY 2009.<br>USE NAMES OF CHILDREN, DATES OF BIRTH, AND PERIODS OF PREGNANCY AS REFERENCE POINTS.<br><br>**C** **IN COLUMN 1**, ENTER METHOD USE CODE OR '0' FOR NONUSE IN EACH BLANK MONTH.<br><br>ILLUSTRATIVE QUESTIONS:<br>* When was the last time you used a method? Which method was that?<br>* When did you start using that method? How long after the birth of (NAME)?<br>* How long did you use the method then?<br><br>**IN COLUMN 2**, ENTER CODES FOR DISCONTINUATION NEXT TO THE LAST MONTH OF USE. NUMBER OF CODES IN COLUMN 2 MUST BE SAME AS NUMBER OF INTERRUPTIONS OF METHOD USE IN COLUMN 1.<br><br>ASK WHY SHE STOPPED USING THE METHOD. IF A PREGNANCY FOLLOWED, ASK WHETHER SHE BECAME PREGNANT UNINTENTIONALLY WHILE USING THE METHOD OR DELIBERATELY STOPPED TO GET PREGNANT.<br><br>ILLUSTRATIVE QUESTIONS:<br>* Why did you stop using the (METHOD)? Did you become pregnant while using (METHOD), or did you stop to get pregnant, or did you stop for some other reason?<br>* IF DELIBERATELY STOPPED TO BECOME PREGNANT, ASK: How many months did it take you to get pregnant after you stopped using (METHOD)? AND ENTER ‘0’ IN EACH SUCH MONTH IN COLUMN 1.		

NO.	QUESTIONS AND FILTERS	CODING CATEGORIES	SKIP
312	CHECK THE CALENDAR FOR USE OF ANY CONTRACEPTIVE METHOD IN ANY MONTH<br><br>NO METHOD USED ☐ ↓<br>ANY METHOD USED ☐ → 314		→ 314
313	Have you ever used anything or tried in any way to delay or avoid getting pregnant?	YES . . . . 1<br>NO 2	 → 324
314	CHECK 304:<br><br>CIRCLE METHOD CODE:<br><br>IF MORE THAN ONE METHOD CODE CIRCLED IN 304, CIRCLE CODE FOR HIGHEST METHOD IN LIST.	NO CODE CIRCLED . . . . . 00<br>FEMALE STERILIZATION . . . . . 01<br>MALE STERILIZATION . . . . . 02<br>IUD . . . . 03<br>INJECTABLES . . . . 04<br>IMPLANTS . . . . 05<br>DAILY PILL . . . . 06<br>MONTHLY METHOD (CHINESE PILL) 07<br>CONDOM . . . . 08<br>FEMALE CONDOM . . . . 09<br>DIAPHRAGM . . . . 10<br>FOAM/JELLY . . . . 11<br>LACTATIONAL AMEN. METHOD . . . . . 12<br>RHYTHM METHOD . . . . 13<br>WITHDRAWAL . . . . 14<br>OTHER MODERN METHOD . . . . . 95<br>OTHER TRADITIONAL METHOD 96	→ 324<br>→ 317A<br>→ 326<br><br><br><br><br><br><br><br><br><br>⌐<br>→ 315A<br>⌐<br>→ 326<br>⌙
315<br><br><br><br><br><br><br><br><br><br>315A	You first started using (CURRENT METHOD) in (DATE FROM 308/308A). Where did you get it at that time?<br><br><br><br><br><br><br><br><br><br>Where did you learn how to use the rhythm/lactational amenorhea/withdrawal method?<br><br><br><br><br><br><br><br>PROBE TO IDENTIFY THE TYPE OF SOURCE.<br><br>IF UNABLE TO DETERMINE IF PUBLIC OR PRIVATE SECTOR, WRITE THE NAME OF THE PLACE.<br><br>____________________<br>(NAME OF PLACE)	PUBLIC SECTOR<br>NATIONAL HOSPITAL (PP) . . . . . 11<br>PROVINCIAL HOSPITAL (RH) . . . . . 12<br>DISTRICT HOSPITAL (RH) . . . . . 13<br>HEALTH CENTER . . . . . 14<br>HEALTH POST . . . . . 15<br>MILITARY HOSPITAL . . . . . 16<br>OTHER PUBLIC<br>SECTOR __________ 17<br>(SPECIFY)<br><br>PRIVATE MEDICAL SECTOR<br>PRIVATE HOSPITAL . . . . . 21<br>PRIVATE CLINIC . . . . . 22<br>PHARMACY . . . . . 23<br>OTHER PRIVATE MEDICAL<br>SECTOR __________ 24<br>(SPECIFY)<br><br>OTHER<br>SHOP . . . . . 31<br>COMMUNITY DISTRIBUTOR . . . . . 32<br>FRIEND/RELATIVE . . . . . 33<br><br>OTHER __________ 96<br>(SPECIFY)	

NO.	QUESTIONS AND FILTERS	CODING CATEGORIES	SKIP
316	CHECK 304:<br><br>CIRCLE METHOD CODE:<br><br>IF MORE THAN ONE METHOD CODE CIRCLED IN 304, CIRCLE CODE FOR HIGHEST METHOD IN LIST.	IUD . . . . . 03<br>INJECTABLES . . . . . 04<br>IMPLANTS . . . . . 05<br>DAILY PILL . . . . . 06<br>MONTHLY METHOD (CHINESE PILL) 07<br>CONDOM . . . . . 08<br>FEMALE CONDOM . . . . . 09<br>DIAPHRAGM . . . . . 10<br>FOAM/JELLY . . . . . 11<br>LACTATIONAL AMEN. METHOD . . . . . 12<br>RHYTHM METHOD 13	 <br><br><br><br>08 → 323<br>09, 10, 11 → 320<br><br><br>12 → 326<br>13 → 326
317<br><br><br>317A	At that time, were you told about side effects or problems you might have with the method?<br><br>When you got sterilized, were you told about side effects or problems you might have with the method?	YES . . . . . 1<br>NO 2	1 → 319
318	Were you ever told by a health or family planning worker about side effects or problems you might have with the method?	YES . . . . . 1<br>NO 2	2 → 320
319	Were you told what to do if you experienced side effects or problems?	YES . . . . . 1<br>NO 2	
320	CHECK 317:<br><br>CODE '1' CIRCLED ↓<br>At that time, were you told about other methods of family planning that you could use?<br><br>CODE '1' NOT CIRCLED ↓<br>When you obtained (CURRENT METHOD FROM 314) from (SOURCE OF METHOD FROM 307 OR 315), were you told about other methods of family planning that you could use?	YES . . . . . 1<br>NO 2	1 → 322
321	Were you ever told by a health or family planning worker about other methods of family planning that you could use?	YES . . . . . 1<br>NO 2	
322	CHECK 304:<br><br>CIRCLE METHOD CODE:<br><br>IF MORE THAN ONE METHOD CODE CIRCLED IN 304, CIRCLE CODE FOR HIGHEST METHOD IN LIST.	FEMALE STERILIZATION . . . . . 01<br>MALE STERILIZATION . . . . . 02<br>IUD . . . . . 03<br>INJECTABLES . . . . . 04<br>IMPLANTS . . . . . 05<br>DAILY PILL . . . . . 06<br>MONTHLY METHOD (CHINESE PILL) 07<br>CONDOM . . . . . 08<br>FEMALE CONDOM . . . . . 09<br>DIAPHRAGM . . . . . 10<br>FOAM/JELLY . . . . . 11<br>LACTATIONAL AMEN. METHOD . . . . . 12<br>RHYTHM METHOD . . . . . 13<br>WITHDRAWAL . . . . . 14<br>OTHER MODERN METHOD . . . . . 95<br>OTHER TRADITIONAL METHOD . . . 96	01, 02, 03 → 326<br><br><br><br>05 → 326<br><br><br><br><br><br><br>12, 13, 14, 95, 96 → 326

NO.	QUESTIONS AND FILTERS	CODING CATEGORIES	SKIP
323	Where did you obtain (CURRENT METHOD) the last time?<br><br>PROBE TO IDENTIFY THE TYPE OF SOURCE.<br><br>IF UNABLE TO DETERMINE IF PUBLIC OR PRIVATE SECTOR, WRITE THE NAME OF THE PLACE.<br><br>____________________<br>(NAME OF PLACE)	PUBLIC SECTOR<br>NATIONAL HOSPITAL (PP) . . . . . 11<br>PROVINCIAL HOSPITAL (RH) . . . . . 12<br>DISTRICT HOSPITAL (RH) . . . . 13<br>HEALTH CENTER . . . . 14<br>HEALTH POST . . . . 15<br>MILITARY HOSPITAL . . . . 16<br>OTHER PUBLIC<br>SECTOR ____________ 17<br>(SPECIFY)<br><br>PRIVATE MEDICAL SECTOR<br>PRIVATE HOSPITAL . . . . 21<br>PRIVATE CLINIC . . . . 22<br>PHARMACY . . . . 23<br>OTHER PRIVATE MEDICAL<br>SECTOR ____________ 24<br>(SPECIFY)<br><br>OTHER<br>SHOP . . . . 31<br>COMMUNITY DISTRIBUTOR . . . . . 32<br>FRIEND/RELATIVE . . . . 33<br><br>OTHER ____________ 96<br>(SPECIFY)	→ 326
324	Do you know of a place where you can obtain a method of family planning?	YES . . . . 1<br>NO 2	 → 326
325	Where is that?<br><br>Any other place?<br><br>PROBE TO IDENTIFY EACH TYPE OF SOURCE.<br><br>IF UNABLE TO DETERMINE IF PUBLIC OR PRIVATE SECTOR, WRITE THE NAME OF THE PLACE.<br><br>____________________<br>(NAME OF PLACE(S))	PUBLIC SECTOR<br>NATIONAL HOSPITAL (PP) . . . . . A<br>PROVINCIAL HOSPITAL (RH) . . . . . B<br>DISTRICT HOSPITAL (RH) . . . . C<br>HEALTH CENTER . . . . D<br>HEALTH POST . . . . E<br>MILITARY HOSPITAL . . . . F<br>OTHER PUBLIC<br>SECTOR ____________ G<br>(SPECIFY)<br><br>PRIVATE MEDICAL SECTOR<br>PRIVATE HOSPITAL . . . . H<br>PRIVATE CLINIC . . . . I<br>PHARMACY . . . . J<br>OTHER PRIVATE MEDICAL<br>SECTOR ____________ K<br>(SPECIFY)<br><br>OTHER<br>SHOP . . . . L<br>COMMUNITY DISTRIBUTOR . . . . . M<br>FRIEND/RELATIVE . . . . N<br><br>OTHER ____________ X<br>(SPECIFY)	

NO.	QUESTIONS AND FILTERS	CODING CATEGORIES	SKIP
326	In the last 12 months, were you visited by a fieldworker who talked to you about family planning?	YES . . . . 1<br>NO 2	
327	In the last 12 months, have you visited a health facility for care for yourself (or your children)?	YES . . . . 1<br>NO 2	 → 401
328	Did any staff member at the health facility speak to you about family planning methods?	YES . . . . 1<br>NO 2	

401	CHECK 224: ONE OR MORE BIRTHS IN 2009 OR LATER ☐ ↓ / NO BIRTHS IN 2009 OR LATER ☐				→ 556
402	CHECK 215: ENTER IN THE TABLE THE BIRTH HISTORY NUMBER, NAME, AND SURVIVAL STATUS OF EACH BIRTH IN 2009 OR LATER. ASK THE QUESTIONS ABOUT ALL OF THESE BIRTHS. BEGIN WITH THE LAST BIRTH. (IF THERE ARE MORE THAN 3 BIRTHS, USE LAST 2 COLUMNS OF ADDITIONAL QUESTIONNAIRES).<br><br>Now I would like to ask some questions about your children born in the last five years. (We will talk about each separately.)				
403	BIRTH HISTORY NUMBER FROM 212 IN BIRTH HISTORY	LAST BIRTH<br>BIRTH HISTORY NUMBER ☐☐	NEXT-TO-LAST BIRTH<br>BIRTH HISTORY NUMBER ☐☐	SECOND-FROM-LAST BIRTH<br>BIRTH HISTORY NUMBER ☐☐	
404	FROM 212 AND 216	NAME ________<br>LIVING ☐ DEAD ☐	NAME ________<br>LIVING ☐ DEAD ☐	NAME ________<br>LIVING ☐ DEAD ☐	
405	When you got pregnant with (NAME), did you want to get pregnant at that time?	YES . . . . 1<br>(SKIP TO 408)<br>NO 2	YES . . . . 1<br>(SKIP TO 430)<br>NO 2	YES . . . . 1<br>(SKIP TO 430)<br>NO 2	
406	Did you want to have a baby later on, or did you not want any (more) children?	LATER . . . . 1<br>NO MORE . . . . 2<br>(SKIP TO 408)	LATER . . . . 1<br>NO MORE . . . . 2<br>(SKIP TO 430)	LATER . . . . 1<br>NO MORE . . . . 2<br>(SKIP TO 430)	
407	How much longer did you want to wait?	MONTHS . .1 ☐☐<br>YEARS . . 2 ☐☐<br>DON'T KNOW . . . 998	MONTHS . .1 ☐☐<br>YEARS . . 2 ☐☐<br>DON'T KNOW . . . 998	MONTHS . .1 ☐☐<br>YEARS . . 2 ☐☐<br>DON'T KNOW . . . 998	
408	Did you see anyone for antenatal care for this pregnancy?	YES . . . . 1<br>NO . . . . 2<br>(SKIP TO 415)			
409	Whom did you see?<br><br>Anyone else?<br><br>PROBE TO IDENTIFY EACH TYPE OF PERSON AND RECORD ALL MENTIONED.	HEALTH PERSONNEL<br>DOCTOR/MEDICAL ASSISTANT . A<br>MIDWIFE . . . . B<br>NURSE . . . . . C<br>OTHER PERSON<br>TRADITIONAL BIRTH ATTENDANT . D<br>VILLAGE HEALT VOLUNTEER . . . E<br>OTHER ________ X<br>(SPECIFY)			

NO.	QUESTIONS AND FILTERS	LAST BIRTH<br>NAME ________	NEXT-TO-LAST BIRTH<br>NAME ________	SECOND-FROM-LAST BIRTH<br>NAME ________
410	Where did you receive antenatal care for this pregnancy?<br><br>Anywhere else?<br><br>PROBE TO IDENTIFY TYPE(S) OF SOURCE(S).<br><br>IF UNABLE TO DETERMINE IF PUBLIC OR PRIVATE SECTOR, WRITE THE NAME OF THE PLACE.<br><br>________<br>(NAME OF PLACE(S))	HOME<br>YOUR HOME . . . A<br>MIDWIFE/TBA HOME . . . . B<br>OTHER HOME . . . C<br><br>PUBLIC SECTOR<br>NATL HOSP (PP) D<br>PROV HOSP (RH) E<br>DIST HOSP (RH) F<br>HLTH CENTER . . . G<br>HLTH POST . . . . . H<br>OUTREACH . . . . . I<br>MILITARY HOSP J<br>OTHER PUBLIC SECTOR<br>________ K<br>(SPECIFY)<br>PRIVATE MED. SECTOR<br>PRIV. HOSP . . . L<br>PRIV. CLINIC . . . M<br>OTHER PRIVATE MED. SECTOR<br>________ N<br>(SPECIFY)<br><br>OTHER ________ X<br>(SPECIFY)		
411	How many months pregnant were you when you first received antenatal care for this pregnancy?	MONTHS . . . [ ][ ]<br><br>DON'T KNOW 98		
412	How many times did you receive antenatal care during this pregnancy?	NUMBER OF TIMES [ ][ ]<br><br>DON'T KNOW 98		
413	As part of your antenatal care during this pregnancy, were any of the following done at least once:<br><br>Were you weighed?<br>Was your height taken?<br>Was your blood pressure measured?<br>Did you give a urine sample?<br>Did you give a blood sample?<br>Did you received nutritional counseling?	YES NO<br><br>WEIGHT . . . 1 2<br>HEIGHT . . . 1 2<br>BP . . . . 1 2<br>URINE . . . . . 1 2<br>BLOOD . . . 1 2<br>NUTR COUN 1 2		
414	During (any of) your antenatal care visit(s), were you told about things to look out for that might suggest problems with the pregnancy?	YES . . . . 1<br>NO . . . . 2<br>DON'T KNOW 8		
415	During this pregnancy, were you given an injection in the arm to prevent the baby from getting tetanus, that is, convulsions after birth?	YES . . . . 1<br><br>NO . . . . 2<br>(SKIP TO 418) ←<br>DON'T KNOW 8		

NO.	QUESTIONS AND FILTERS	LAST BIRTH<br>NAME ________	NEXT-TO-LAST BIRTH<br>NAME ________	SECOND-FROM-LAST BIRTH<br>NAME ________
416	During this pregnancy, how many times did you get a tetanus injection?	TIMES . . . . [ ]<br>DON'T KNOW 8		
417	CHECK 416:	2 OR MORE TIMES [ ] (SKIP TO 421)<br>OTHER [ ]		
418	At any time before this pregnancy, did you receive any tetanus injections?	YES . . . . 1<br>NO . . . . 2<br>(SKIP TO 421)<br>DON'T KNOW . . . 8		
419	Before this pregnancy, how many times did you receive a tetanus injection?<br>IF 7 OR MORE TIMES, RECORD '7'.	TIMES . . . . [ ]<br>DON'T KNOW 8		
420	How many years ago did you receive the last tetanus injection before this pregnancy?	YEARS AGO . . . . . [ ][ ]		
421	During this pregnancy, were you given or did you buy any iron tablets or iron syrup?<br>SHOW TABLETS/SYRUP.	YES . . . . 1<br>NO . . . . 2<br>(SKIP TO 423)<br>DON'T KNOW 8		
422	During the whole pregnancy, for how many days did you take the tablets or syrup?<br>IF ANSWER IS NOT NUMERIC, PROBE FOR APPROXIMATE NUMBER OF DAYS.	DAYS [ ][ ][ ]<br>DON'T KNOW . . . 998		
423	During this pregnancy, did you take any drug for intestinal parasites?	YES . . . . 1<br>NO . . . . 2<br>DON'T KNOW 8		
423A	During this pregnancy, did you have difficulty with your vision during daylight?	YES . . . . 1<br>NO . . . . 2<br>DON'T KNOW 8		
423B	During this pregnancy, did you suffer from night blindness?	YES . . . . 1<br>NO . . . . 2<br>DON'T KNOW 8		

NO.	QUESTIONS AND FILTERS	LAST BIRTH<br>NAME ________	NEXT-TO-LAST BIRTH<br>NAME ________	SECOND-FROM-LAST BIRTH<br>NAME ________
424	During this pregnancy, did you take any drugs to keep you from getting malaria?	YES . . . . 1<br>NO . . . . 2<br>(SKIP TO 430)<br>DON'T KNOW 8		
425	What drugs did you take?<br><br>RECORD ALL MENTIONED.<br>IF TYPE OF DRUG IS NOT DETERMINED, SHOW TYPICAL ANTIMALARIAL DRUGS TO RESPONDENT.	SP/FANSIDAR . . . . . A<br>CHLOROQUINE . . . B<br>OTHER ________ X<br>(SPECIFY)<br>DON'T KNOW . . . . Z		
426	CHECK 425:<br><br>SP/FANSIDAR TAKEN FOR MALARIA PREVENTION.	CODE 'A' CIRCLED ☐<br>CODE 'A' NOT CIRCLED ☐<br>(SKIP TO 430)		
427	How many times did you take (SP/Fansidar) during this pregnancy?	TIMES . . . . . ☐☐		
428	CHECK 409:<br><br>ANTENATAL CARE FROM HEALTH PERSONNEL DURING THIS PREGNANCY	CODE 'A', B' OR 'C' CIRCLED ☐<br>OTHER ☐<br>(SKIP TO 430)		
429	Did you get the (SP/Fansidar) during any antenatal care visit, during another visit to a health facility or from another source?	ANTENATAL VISIT . . 1<br>ANOTHER FACILITY VISIT . . . . 2<br>OTHER SOURCE 6		
430	When (NAME) was born, was he/she very large, larger than average, average, smaller than average, or very small?	VERY LARGE . . . . . 1<br>LARGER THAN AVERAGE . . . . . 2<br>AVERAGE . . . . 3<br>SMALLER THAN AVERAGE . . . . . 4<br>VERY SMALL . . . . . 5<br>DON'T KNOW 8	VERY LARGE . . . . . 1<br>LARGER THAN AVERAGE . . . . . 2<br>AVERAGE . . . . 3<br>SMALLER THAN AVERAGE . . . . . 4<br>VERY SMALL . . . . . 5<br>DON'T KNOW 8	VERY LARGE . . . . . 1<br>LARGER THAN AVERAGE . . . . . 2<br>AVERAGE . . . . 3<br>SMALLER THAN AVERAGE . . . . . 4<br>VERY SMALL . . . . . 5<br>DON'T KNOW 8
431	Was (NAME) weighed at birth?	YES . . . . 1<br>NO . . . . 2<br>(SKIP TO 433)<br>DON'T KNOW 8	YES . . . . 1<br>NO . . . . 2<br>(SKIP TO 433)<br>DON'T KNOW 8	YES . . . . 1<br>NO . . . . 2<br>(SKIP TO 433)<br>DON'T KNOW 8
432	How much did (NAME) weigh?<br><br>RECORD WEIGHT IN KILOGRAMS FROM HEALTH CARD, IF AVAILABLE.	KG FROM CARD<br>1 ☐ . ☐☐☐<br>KG FROM RECALL<br>2 ☐ . ☐☐☐<br>DON'T KNOW 99998	KG FROM CARD<br>1 ☐ . ☐☐☐<br>KG FROM RECALL<br>2 ☐ . ☐☐☐<br>DON'T KNOW 99998	KG FROM CARD<br>1 ☐ . ☐☐☐<br>KG FROM RECALL<br>2 ☐ . ☐☐☐<br>DON'T KNOW 99998

NO.	QUESTIONS AND FILTERS	LAST BIRTH<br>NAME ________	NEXT-TO-LAST BIRTH<br>NAME ________	SECOND-FROM-LAST BIRTH<br>NAME ________
433	Who assisted with the delivery of (NAME)?<br><br>Anyone else?<br><br>PROBE FOR THE TYPE(S) OF PERSON(S) AND RECORD ALL MENTIONED.<br><br>IF RESPONDENT SAYS NO ONE ASSISTED, PROBE TO DETERMINE WHETHER ANY ADULTS WERE PRESENT AT THE DELIVERY.	HEALTH PERSONNEL<br>DOCTOR/MEDICAL ASSISTANT . A<br>MIDWIFE . . . . B<br>NURSE . . . . . C<br>OTHER PERSON<br>TRADITIONAL BIRTH ATTENDANT D<br>RELATIVE/FRIEND E<br>OTHER ________ X<br>(SPECIFY)<br>NO ONE ASSISTED Y	HEALTH PERSONNEL<br>DOCTOR/MEDICAL ASSISTANT . A<br>MIDWIFE . . . . B<br>NURSE . . . . . C<br>OTHER PERSON<br>TRADITIONAL BIRTH ATTENDANT D<br>RELATIVE/FRIEND E<br>OTHER ________ X<br>(SPECIFY)<br>NO ONE ASSISTED Y	HEALTH PERSONNEL<br>DOCTOR/MEDICAL ASSISTANT . A<br>MIDWIFE . . . . B<br>NURSE . . . . . C<br>OTHER PERSON<br>TRADITIONAL BIRTH ATTENDANT D<br>RELATIVE/FRIEND E<br>OTHER ________ X<br>(SPECIFY)<br>NO ONE ASSISTED Y
434	Where did you give birth to (NAME)?<br><br>PROBE TO IDENTIFY THE TYPE OF SOURCE.<br><br>IF UNABLE TO DETERMINE IF PUBLIC OR PRIVATE SECTOR, WRITE THE NAME OF THE PLACE.<br><br>(NAME OF PLACE)	HOME<br>YOUR HOME . . . 11<br>(SKIP TO 438)<br>OTHER HOME . . . 12<br>PUBLIC SECTOR<br>NATL HOSP (PP) 21<br>PROV HOSP (RH) 22<br>DIST HOSP (RH) 23<br>HLTH CENTER 24<br>HLTH POST 25<br>MILITARY HOSP 26<br>OTHER PUBLIC SECTOR ________ 27<br>(SPECIFY)<br>PRIVATE MED. SECTOR<br>PRIV. HOSP . 31<br>PF CLINIC . . . . 32<br>OTHER PRIVATE MED. SECTOR ________ 33<br>(SPECIFY)<br><br>OTHER ________ 96<br>(SPECIFY)<br>(SKIP TO 438)	HOME<br>YOUR HOME . . . 11<br>(SKIP TO 448)<br>OTHER HOME . . . 12<br>PUBLIC SECTOR<br>NATL HOSP (PP) 21<br>PROV HOSP (RH) 22<br>DIST HOSP (RH) 23<br>HLTH CENTER 24<br>HLTH POST 25<br>MILITARY HOSP 26<br>OTHER PUBLIC SECTOR ________ 27<br>(SPECIFY)<br>PRIVATE MED. SECTOR<br>PRIV. HOSP . 31<br>PF CLINIC . . . . 32<br>OTHER PRIVATE MED. SECTOR ________ 33<br>(SPECIFY)<br><br>OTHER ________ 96<br>(SPECIFY)<br>(SKIP TO 448)	HOME<br>YOUR HOME . . . 11<br>(SKIP TO 448)<br>OTHER HOME . . . 12<br>PUBLIC SECTOR<br>NATL HOSP (PP) 21<br>PROV HOSP (RH) 22<br>DIST HOSP (RH) 23<br>HLTH CENTER 24<br>HLTH POST 25<br>MILITARY HOSP 26<br>OTHER PUBLIC SECTOR ________ 27<br>(SPECIFY)<br>PRIVATE MED. SECTOR<br>PRIV. HOSP . 31<br>PF CLINIC . . . . 32<br>OTHER PRIVATE MED. SECTOR ________ 33<br>(SPECIFY)<br><br>OTHER ________ 96<br>(SPECIFY)<br>(SKIP TO 448)
435	Was (NAME) delivered by caesarean, that is, did they cut your belly open to take the baby out?	YES, ELECTIVE . . . 1<br>YES, EMERGENCY/ MEDIC INDICATED 2<br><br>NO 3	YES, ELECTIVE . . . 1<br>YES, EMERGENCY/ MEDIC INDICATED 2<br><br>NO 3	YES, ELECTIVE . . . 1<br>YES, EMERGENCY/ MEDIC INDICATED 2<br><br>NO 3
436	After you gave birth to (NAME), did anyone check on your health while you were still in the facility?	YES . . . . 1<br>(SKIP TO 439)<br>NO 2		
437	Did anyone check on your health after you left the facility?	YES . . . . 1<br>(SKIP TO 439)<br>NO . . . . 2<br>(SKIP TO 446)		
438	After you gave birth to (NAME), did anyone check on your health?	YES . . . . 1<br>NO . . . . 2<br>(SKIP TO 442)		

NO.	QUESTIONS AND FILTERS	LAST BIRTH<br>NAME ________	NEXT-TO-LAST BIRTH<br>NAME ________	SECOND-FROM-LAST BIRTH<br>NAME ________
439	Who checked on your health at that time?<br><br>PROBE FOR MOST QUALIFIED PERSON.	HEALTH PERSONNEL<br>DOCTOR/MEDICAL ASSISTANT . 11<br>MIDWIFE . . . . 12<br>NURSE . . . . . 13<br>OTHER PERSON<br>TRADITIONAL BIRTH ATTENDANT 21<br>OTHER ________ 96<br>(SPECIFY)		
440	How long after delivery did the first check take place?<br><br>IF LESS THAN ONE DAY, RECORD HOURS.<br>IF LESS THAN ONE WEEK, RECORD DAYS.	HOURS 1 [ ][ ]<br>DAYS 2 [ ][ ]<br>WEEKS 3 [ ][ ]<br>DON'T KNOW . . . 998		
441	In the six week after delivery, how many times did you received a check on your health?	TIMES [ ][ ]<br>DON'T KNOW . . . 98		
442	In the six weeks after (NAME) was born, did any health care provider or a traditional birth attendant check on his/her health?	YES . . . . 1<br>NO . . . . 2<br>(SKIP TO 446)<br>DON'T KNOW 8		
443	How many hours, days or weeks after the birth of (NAME) did the first check take place?<br><br>IF LESS THAN ONE DAY, RECORD HOURS.<br>IF LESS THAN ONE WEEK, RECORD DAYS.	HRS AFTER BIRTH . . 1 [ ][ ]<br>DAYS AFTER BIRTH . . 2 [ ][ ]<br>WKS AFTER BIRTH . . 3 [ ][ ]<br>DON'T KNOW . . . 998		
444	Who checked on (NAME)'s health at that time?<br><br>PROBE FOR MOST QUALIFIED PERSON.	HEALTH PERSONNEL<br>DOCTOR/MEDICAL ASSISTANT . 11<br>MIDWIFE . . . . 12<br>NURSE . . . . . 13<br>OTHER PERSON<br>TRADITIONAL BIRTH ATTENDANT 21<br>OTHER ________ 96<br>(SPECIFY)		

NO.	QUESTIONS AND FILTERS	LAST BIRTH<br>NAME ________	NEXT-TO-LAST BIRTH<br>NAME ________	SECOND-FROM-LAST BIRTH<br>NAME ________
445	Where did this first check of (NAME) take place?<br><br>PROBE TO IDENTIFY THE TYPE OF SOURCE AND CIRCLE THE APPROPRIATE CODE.<br><br>IF UNABLE TO DETERMINE IF A HOSPITAL, HEALTH CENTER, OR CLINIC IS PUBLIC OR PRIVATE MEDICAL, WRITE THE THE NAME OF THE PLACE.<br><br>________<br>(NAME OF PLACE)	HOME<br>YOUR HOME . . . 11<br>OTHER HOME . . . 12<br><br>PUBLIC SECTOR<br>NATL HOSP (PP) 21<br>PROV HOSP (RH) 22<br>DIST HOSP (RH) 23<br>HLTH CENTER 24<br>HLTH POST 25<br>MILITARY HOSP 26<br>OTHER PUBLIC SECTOR<br>________ 27<br>(SPECIFY)<br>PRIVATE MED. SECTOR<br>PRIV. HOSP . 31<br>PI CLINIC . . . . 32<br>OTHER PRIVATE MED. SECTOR<br>________ 33<br>(SPECIFY)<br><br>OTHER________ 96<br>(SPECIFY)		
445A	In the six week after (NAME) was born, many times did a health care provider or a traditional birth attendant check on his/her health?	TIMES [ ][ ]<br><br>DON'T KNOW . . . 98		
446	In the first six weeks after delivery, did you receive<br><br>A deworming tablet (this/these)?<br>An advice on contraception?<br><br>A counseling for newborn care?<br>An iron tablet (like this/these)?<br><br>SHOW COMMON TYPES OF AMPULES/CAPSULES/SYRUPS.	YES NO<br><br>DEWORMING 1 2<br>CONTRA-CEPTION 1 2<br>COUN NEWB 1 2<br>IRON . . . . . 1 2<br><br>(SKIP TO 447)←		
446A	In the first six weeks after delivery, how many days did you receive iron tablets ?	DAYS [ ][ ]<br><br>DON'T KNOW . . . 98		
447	Has your menstrual period returned since the birth of (NAME)?	YES . . . . 1<br>(SKIP TO 449)←<br>NO . . . . 2<br>(SKIP TO 450)←		
448	Did your period return between the birth of (NAME) and your next pregnancy?		YES . . . . 1<br>NO . . . . 2<br>(SKIP TO 452)←	YES . . . . 1<br>NO . . . . 2<br>(SKIP TO 452)←
449	For how many months after the birth of (NAME) did you not have a period?	MONTHS . . . [ ][ ]<br><br>DON'T KNOW 98	MONTHS . . . [ ][ ]<br><br>DON'T KNOW 98	MONTHS . . . [ ][ ]<br><br>DON'T KNOW 98

NO.	QUESTIONS AND FILTERS	LAST BIRTH<br>NAME ________	NEXT-TO-LAST BIRTH<br>NAME ________	SECOND-FROM-LAST BIRTH<br>NAME ________
450	CHECK 226:<br><br>IS RESPONDENT PREGNANT?	NOT PREG-NANT ☐ ↓<br>PREGNANT OR UNSURE ☐ (SKIP TO 452)		
451	Have you had sexual intercourse since the birth of (NAME)?	YES . . . . 1<br>NO . . . . 2<br>(SKIP TO 453)		
452	For how many months after the birth of (NAME) did you not have sexual intercourse?	MONTHS . . . ☐☐<br><br>DON'T KNOW 98	MONTHS . . . ☐☐<br><br>DON'T KNOW 98	MONTHS . . . ☐☐<br><br>DON'T KNOW 98
453	Did you ever breastfeed (NAME)?	YES . . . . 1<br>(SKIP TO 455)<br>NO 2	YES . . . . 1<br><br>NO 2	YES . . . . 1<br><br>NO 2
454	CHECK 404:<br><br>IS CHILD LIVING?	LIVING ☐ (SKIP TO 460)<br>DEAD ☐ (GO BACK TO 405 IN NEXT COLUMN; OR IF NO MORE BIRTHS, GO TO 501)		
455	How long after birth did you first put (NAME) to the breast?<br><br>IF LESS THAN 1 HOUR, RECORD '00' HOURS.<br>IF LESS THAN 24 HOURS, RECORD HOURS.<br>OTHERWISE, RECORD DAYS.	IMMEDIATELY . . . 000<br><br>HOURS 1 ☐☐<br><br>DAYS 2 ☐☐		
456	In the first three days after delivery, was (NAME) given anything to drink other than breast milk such as chheu em?	YES . . . . 1<br>NO . . . . 2<br>(SKIP TO 458)		
457	What was (NAME) given to drink?<br><br>Anything else?<br><br>RECORD ALL LIQUIDS MENTIONED.	MILK (OTHER THAN BREAST MILK) A<br>PLAIN WATER . . . B<br>SUGAR OR HONEY WATER . . . C<br>SUGAR-SALT-WATER SOLUTION . . . . . D<br>COCONUT/FRUIT JUICE . . . . . E<br>INFANT FORMULA F<br>HERBAL TEA . . . G<br><br>OTHER ________ X<br>(SPECIFY)		

NO.	QUESTIONS AND FILTERS	LAST BIRTH<br>NAME ________________	NEXT-TO-LAST BIRTH<br>NAME ________________	SECOND-FROM-LAST BIRTH<br>NAME ________________
458	CHECK 404:<br><br>IS CHILD LIVING?	LIVING ☐ ↓ DEAD ☐ (GO BACK TO 405 IN NEXT COLUMN; OR, IF NO MORE BIRTHS, GO TO 501)	LIVING ☐ ↓ DEAD ☐ (GO BACK TO 405 IN NEXT COLUMN; OR, IF NO MORE BIRTHS, GO TO 501)	LIVING ☐ ↓ DEAD ☐ (GO BACK TO 405 IN NEXT-TO-LAST COLUMN OF NEW QUESTIONNAIRE; OR, IF NO MORE BIRTHS, GO TO 501)
459	Are you still breastfeeding (NAME)?	YES . . . . 1<br>NO 2		
460	Did (NAME) drink anything from a bottle with a nipple yesterday or last night?	YES . . . . 1<br>NO . . . . 2<br>DON'T KNOW 8	YES . . . . 1<br>NO . . . . 2<br>DON'T KNOW 8	YES . . . . 1<br>NO . . . . 2<br>DON'T KNOW 8
461		GO BACK TO 405 IN NEXT COLUMN; OR, IF NO MORE BIRTHS, GO TO 501.	GO BACK TO 405 IN NEXT COLUMN; OR, IF NO MORE BIRTHS, GO TO 501.	GO BACK TO 405 IN NEXT-TO-LAST COLUMN OF NEW QUESTIONNAIRE; OR, IF NO MORE BIRTHS, GO TO 501.

SECTION 5. CHILD IMMUNIZATION, HEALTH AND NUTRITION

501	ENTER IN THE TABLE THE BIRTH HISTORY NUMBER, NAME, AND SURVIVAL STATUS OF EACH BIRTH IN 2009 OR LATER. ASK THE QUESTIONS ABOUT ALL OF THESE BIRTHS. BEGIN WITH THE LAST BIRTH. (IF THERE ARE MORE THAN 3 BIRTHS, USE LAST 2 COLUMNS OF ADDITIONAL QUESTIONNAIRES).			
502	BIRTH HISTORY NUMBER FROM 212 IN BIRTH HISTORY	LAST BIRTH<br>BIRTH HISTORY NUMBER	NEXT-TO-LAST BIRTH<br>BIRTH HISTORY NUMBER	SECOND-FROM-LAST BIRTH<br>BIRTH HISTORY NUMBER . . . . . .
503	FROM 212 AND 216	NAME ______<br>LIVING / DEAD<br>(GO TO 503 IN NEXT COLUMN OR, IF NO MORE BIRTHS, GO TO 553)	NAME ______<br>LIVING / DEAD<br>(GO TO 503 IN NEXT COLUMN OR, IF NO MORE BIRTHS, GO TO 553)	NAME ______<br>LIVING / DEAD<br>(GO TO 503 IN NEXT-TO-LAST COLUMN OF NEW QUESTIONNAIRE, OR IF NO MORE BIRTHS, GO TO 553)
504	Do you have a card where (NAME)'s vaccinations are written down?<br>IF YES:<br>May I see it please?	YES, SEEN . . . . 1<br>(SKIP TO 506)<br>YES, NOT SEEN . . . . 2<br>(SKIP TO 509)<br>NO CARD 3	YES, SEEN . . . . 1<br>(SKIP TO 506)<br>YES, NOT SEEN . . . . 2<br>(SKIP TO 509)<br>NO CARD 3	YES, SEEN . . . . 1<br>(SKIP TO 506)<br>YES, NOT SEEN . . . . . . 2<br>(SKIP TO 509)<br>NO CARD 3
505	Did you ever have a vaccination card for (NAME)?	YES . . . . 1<br>(SKIP TO 509)<br>NO 2	YES . . . . 1<br>(SKIP TO 509)<br>NO 2	YES . . . . 1<br>(SKIP TO 509)<br>NO 2

506 (1) COPY DATES FROM THE CARD.
(2) WRITE '44' IN 'DAY' COLUMN IF CARD SHOWS THAT A DOSE WAS GIVEN, BUT NO DATE IS RECORDED.

	LAST BIRTH DAY	MONTH	YEAR		NEXT-TO-LAST BIRTH DAY	MONTH	YEAR		SECOND-FROM-LAST BIRTH DAY	MONTH	YEAR
BCG				BCG				BCG			
HB 0 (HEPATITIS B GIVEN AT BIRTH)				HB0				HB0			
OPV 1				OPV1				OPV1			
OPV 2				OPV2				OPV2			
OPV 3				OPV3				OPV3			
TETRA /PENTAVALENT 1				T/P1				T/P1			
TETRA /PENTAVALENT 2				T/P2				T/P2			
TETRA /PENTAVALENT 3				T/P3				T/P3			
MEASLES				MEA				MEA			
VITAMIN A (MOST RECENT)				VIT A				VIT A			

507	CHECK 506:	BCG TO MEASLES ALL RECORDED (GO TO 511) / OTHER	BCG TO MEASLES ALL RECORDED (GO TO 511) / OTHER	BCG TO MEASLES ALL RECORDED (GO TO 511) / OTHER

NO.	QUESTIONS AND FILTERS	LAST BIRTH<br>NAME ________	NEXT-TO-LAST BIRTH<br>NAME ________	SECOND-FROM-LAST BIRTH<br>NAME ________
508	Has (NAME) had any vaccinations that are not recorded on this card, including vaccinations given in a national immunization day campaign?<br><br>RECORD 'YES' ONLY IF THE RESPONDENT MENTIONS AT LEAST ONE OF THE VACCINATIONS IN 506 THAT ARE NOT RECORDED AS HAVING BEEN GIVEN.	YES . . . . 1<br>(PROBE FOR VACCINATIONS AND WRITE '66' IN THE CORRESPONDING DAY COLUMN IN 506)<br>(SKIP TO 511)<br>NO . . . . 2<br>(SKIP TO 511)<br>DON'T KNOW 8	YES . . . . 1<br>(PROBE FOR VACCINATIONS AND WRITE '66' IN THE CORRESPONDING DAY COLUMN IN 506)<br>(SKIP TO 511)<br>NO . . . . 2<br>(SKIP TO 511)<br>DON'T KNOW 8	YES . . . . 1<br>(PROBE FOR VACCINATIONS AND WRITE '66' IN THE CORRESPONDING DAY COLUMN IN 506)<br>(SKIP TO 511)<br>NO . . . . 2<br>(SKIP TO 511)<br>DON'T KNOW 8
509	Did (NAME) ever have any vaccinations to prevent him/her from getting diseases, including vaccinations received in a national immunization day campaign?	YES . . . . 1<br>NO . . . . 2<br>(SKIP TO 511)<br>DON'T KNOW 8	YES . . . . 1<br>NO . . . . 2<br>(SKIP TO 511)<br>DON'T KNOW 8	YES . . . . 1<br>NO . . . . 2<br>(SKIP TO 511)<br>DON'T KNOW 8
510	Please tell me if (NAME) had any of the following vaccinations:			
510A	A BCG vaccination against tuberculosis, that is, an injection in the left arm or shoulder that usually causes a scar?	YES . . . . 1<br>NO . . . . 2<br>DON'T KNOW 8	YES . . . . 1<br>NO . . . . 2<br>DON'T KNOW 8	YES . . . . 1<br>NO . . . . 2<br>DON'T KNOW 8
510B	Polio vaccine, that is, drops in the mouth?	YES . . . . 1<br>NO . . . . 2<br>(SKIP TO 510E)<br>DON'T KNOW 8	YES . . . . 1<br>NO . . . . 2<br>(SKIP TO 510E)<br>DON'T KNOW 8	YES . . . . 1<br>NO . . . . 2<br>(SKIP TO 510E)<br>DON'T KNOW 8
510D	How many times was the polio vaccine given?	NUMBER OF TIMES . . . . . ☐	NUMBER OF TIMES . . . . . ☐	NUMBER OF TIMES . . . . . ☐
510E	A Tetravalent/Pentavalent vaccination, that is, an injection given in the thigh, sometimes at the same time as polio drops?	YES . . . . 1<br>NO . . . . 2<br>(SKIP TO 510G)<br>DON'T KNOW 8	YES . . . . 1<br>NO . . . . 2<br>(SKIP TO 510G)<br>DON'T KNOW 8	YES . . . . 1<br>NO . . . . 2<br>(SKIP TO 510G)<br>DON'T KNOW 8
510F	How many times was the Tetravalent/Pentavalent vaccination given?	NUMBER OF TIMES . . . . . ☐	NUMBER OF TIMES . . . . . ☐	NUMBER OF TIMES . . . . . ☐
510G	A measles injection - that is, a shot in the arm at the age of 9-11 months - to prevent him/her from getting measles?	YES . . . . 1<br>NO . . . . 2<br>DON'T KNOW 8	YES . . . . 1<br>NO . . . . 2<br>DON'T KNOW 8	YES . . . . 1<br>NO . . . . 2<br>DON'T KNOW 8
510H	A hepatitis B vaccination against hepatitis, that is, an injection given in the right thigh in the first weeks after birth?	YES . . . . 1<br>NO . . . . 2<br>DON'T KNOW 8	YES . . . . 1<br>NO . . . . 2<br>DON'T KNOW 8	YES . . . . 1<br>NO . . . . 2<br>DON'T KNOW 8

NO.	QUESTIONS AND FILTERS	LAST BIRTH<br>NAME ________	NEXT-TO-LAST BIRTH<br>NAME ________	SECOND-FROM-LAST BIRTH<br>NAME ________
511	Within the last six months, was (NAME) given a vitamin A dose like (this/any of these)?<br><br>SHOW COMMON TYPES OF AMPULES/CAPSULES/SYRUPS.	YES . . . . 1<br>NO . . . . 2<br>DON'T KNOW 8	YES . . . . 1<br>NO . . . . 2<br>DON'T KNOW 8	YES . . . . 1<br>NO . . . . 2<br>DON'T KNOW 8
512	In the last seven days, was (NAME) given micrinutrient powder like (this/any of these)?<br><br>SHOW PACKAGE OF MICRONUTRIENT POWDER	YES . . . . 1<br>NO . . . . 2<br>DON'T KNOW 8	YES . . . . 1<br>NO . . . . 2<br>DON'T KNOW 8	YES . . . . 1<br>NO . . . . 2<br>DON'T KNOW 8
513	Was (NAME) given any drug for intestinal worms in the last six months?	YES . . . . 1<br>NO . . . . 2<br>DON'T KNOW 8	YES . . . . 1<br>NO . . . . 2<br>DON'T KNOW 8	YES . . . . 1<br>NO . . . . 2<br>DON'T KNOW 8
514	Has (NAME) had diarrhea in the last 2 weeks?	YES . . . . 1<br>NO . . . . 2<br>(SKIP TO 525)←<br>DON'T KNOW 8	YES . . . . 1<br>NO . . . . 2<br>(SKIP TO 525)←<br>DON'T KNOW 8	YES . . . . 1<br>NO . . . . 2<br>(SKIP TO 525)←<br>DON'T KNOW 8
515	Was there any blood in the stools?	YES . . . . 1<br>NO . . . . 2<br>DON'T KNOW 8	YES . . . . 1<br>NO . . . . 2<br>DON'T KNOW 8	YES . . . . 1<br>NO . . . . 2<br>DON'T KNOW 8
516	Now I would like to know how much (NAME) was given to drink during the diarrhea (including breastmilk).<br><br>Was he/she given less than usual to drink, about the same amount, or more than usual to drink?<br><br>IF LESS, PROBE: Was he/she given much less than usual to drink or somewhat less?	MUCH LESS . . . . . 1<br>SOMEWHAT LESS 2<br>ABOUT THE SAME 3<br>MORE . . . . 4<br>NOTHING TO DRINK 5<br>DON'T KNOW 8	MUCH LESS . . . . . 1<br>SOMEWHAT LESS 2<br>ABOUT THE SAME 3<br>MORE . . . . 4<br>NOTHING TO DRINK 5<br>DON'T KNOW 8	MUCH LESS . . . . . 1<br>SOMEWHAT LESS 2<br>ABOUT THE SAME 3<br>MORE . . . . 4<br>NOTHING TO DRINK 5<br>DON'T KNOW 8
517	When (NAME) had diarrhea, was he/she given less than usual to eat, about the same amount, more than usual, or nothing to eat?<br><br>IF LESS, PROBE: Was he/she given much less than usual to eat or somewhat less?	MUCH LESS . . . . . 1<br>SOMEWHAT LESS 2<br>ABOUT THE SAME 3<br>MORE . . . . 4<br>STOPPED FOOD 5<br>NEVER GAVE FOOD 6<br>DON'T KNOW 8	MUCH LESS . . . . . 1<br>SOMEWHAT LESS 2<br>ABOUT THE SAME 3<br>MORE . . . . 4<br>STOPPED FOOD 5<br>NEVER GAVE FOOD 6<br>DON'T KNOW 8	MUCH LESS . . . . . 1<br>SOMEWHAT LESS 2<br>ABOUT THE SAME 3<br>MORE . . . . 4<br>STOPPED FOOD 5<br>NEVER GAVE FOOD 6<br>DON'T KNOW 8
518	Did you seek advice or treatment for the diarrhea from any source?	YES . . . . 1<br>NO . . . . 2<br>(SKIP TO 522)←	YES . . . . 1<br>NO . . . . 2<br>(SKIP TO 522)←	YES . . . . 1<br>NO . . . . 2<br>(SKIP TO 522)←

NO.	QUESTIONS AND FILTERS	LAST BIRTH<br>NAME ________	NEXT-TO-LAST BIRTH<br>NAME ________	SECOND-FROM-LAST BIRTH<br>NAME ________
519	Where did you seek advice or treatment?<br><br>Anywhere else?<br><br>PROBE TO IDENTIFY EACH TYPE OF SOURCE.<br><br>IF UNABLE TO DETERMINE IF PUBLIC OR PRIVATE SECTOR, WRITE THE NAME OF THE PLACE.<br><br>________<br>(NAME OF PLACE(S))	PUBLIC SECTOR<br>NATL HOSP (PP) A<br>PROV HOSP (RH) B<br>DIST HOSP (RH) C<br>HLTH CENTER . D<br>HLTH POST . . . E<br>OUTREACH . . . F<br>MILITARY HOSP G<br>OTHER PUBLIC SECTOR . . .<br>________ H<br>(SPECIFY)<br><br>PRIVATE MEDICAL SECTOR<br>PRIV. HOSP . . . I<br>PRIV. CLINIC . . . J<br>PHARMACY . . . K<br>HOME OF TRAINED HEALTH WORKER . . . L<br>VISIT OF TRAINED HEALTH WORKER . . . M<br>OTHER PRIVATE MED. SECTOR<br>________ N<br>(SPECIFY)<br><br>OTHER SOURCE<br>SHOP . . . . O<br>TRADITIONAL PRACTITIONER P<br><br>OTHER ________ X<br>(SPECIFY)	PUBLIC SECTOR<br>NATL HOSP (PP) A<br>PROV HOSP (RH) B<br>DIST HOSP (RH) C<br>HLTH CENTER . D<br>HLTH POST . . . E<br>OUTREACH . . . F<br>MILITARY HOSP G<br>OTHER PUBLIC SECTOR . . .<br>________ H<br>(SPECIFY)<br><br>PRIVATE MEDICAL SECTOR<br>PRIV. HOSP . . . I<br>PRIV. CLINIC . . . J<br>PHARMACY . . . K<br>HOME OF TRAINED HEALTH WORKER . . . L<br>VISIT OF TRAINED HEALTH WORKER . . . M<br>OTHER PRIVATE MED. SECTOR<br>________ N<br>(SPECIFY)<br><br>OTHER SOURCE<br>SHOP . . . . O<br>TRADITIONAL PRACTITIONER P<br><br>OTHER ________ X<br>(SPECIFY)	PUBLIC SECTOR<br>NATL HOSP (PP) A<br>PROV HOSP (RH) B<br>DIST HOSP (RH) C<br>HLTH CENTER . D<br>HLTH POST . . . E<br>OUTREACH . . . F<br>MILITARY HOSP G<br>OTHER PUBLIC SECTOR . . .<br>________ H<br>(SPECIFY)<br><br>PRIVATE MEDICAL SECTOR<br>PRIV. HOSP . . . I<br>PRIV. CLINIC . . . J<br>PHARMACY . . . K<br>HOME OF TRAINED HEALTH WORKER . . . L<br>VISIT OF TRAINED HEALTH WORKER . . . M<br>OTHER PRIVATE MED. SECTOR<br>________ N<br>(SPECIFY)<br><br>OTHER SOURCE<br>SHOP . . . . O<br>TRADITIONAL PRACTITIONER P<br><br>OTHER ________ X<br>(SPECIFY)
520	CHECK 519:	TWO OR MORE CODES CIRCLED ☐<br>ONLY ONE CODE CIRCLED ☐<br>(SKIP TO 522)	TWO OR MORE CODES CIRCLED ☐<br>ONLY ONE CODE CIRCLED ☐<br>(SKIP TO 522)	TWO OR MORE CODES CIRCLED ☐<br>ONLY ONE CODE CIRCLED ☐<br>(SKIP TO 522)
521	Where did you first seek advice or treatment?<br><br>USE LETTER CODE FROM 519.	FIRST PLACE . . . ☐	FIRST PLACE . . . ☐	FIRST PLACE . . . ☐
522	Was he/she given any of the following to drink at any time since he/she started having the diarrhea:<br><br>a) A fluid made from a special packet called Oralyte?<br><br>b) A ORS Tablet called Oralyte?	YES NO DK<br><br>FLUID FROM ORS PKT 1 2 8<br><br>ORS TAB 1 2 8	YES NO DK<br><br>FLUID FROM ORS PKT 1 2 8<br><br>ORS TAB 1 2 8	YES NO DK<br><br>FLUID FROM ORS PKT 1 2 8<br><br>ORS TAB 1 2 8

NO.	QUESTIONS AND FILTERS	LAST BIRTH<br>NAME ________	NEXT-TO-LAST BIRTH<br>NAME ________	SECOND-FROM-LAST BIRTH<br>NAME ________
523	Was anything (else) given to treat the diarrhea?	YES . . . . 1<br>NO . . . . 2<br>(SKIP TO 525)<br>DON'T KNOW 8	YES . . . . 1<br>NO . . . . 2<br>(SKIP TO 525)<br>DON'T KNOW 8	YES . . . . 1<br>NO . . . . 2<br>(SKIP TO 525)<br>DON'T KNOW 8
524	What (else) was given to treat the diarrhea?<br><br>Anything else?<br><br>RECORD ALL TREATMENTS GIVEN.	PILL OR SYRUP<br>ANTIBIOTIC . . . . . A<br>ANTIMOTILITY B<br>ZINC . . . . C<br>OTHER (NOT ANTI-BIOTIC, ANTI-MOTILITY, OR ZINC) . . . . D<br>UNKNOWN PILL OR SYRUP . . . E<br><br>INJECTION<br>ANTIBIOTIC . . . . . F<br>NON-ANTIBIOTIC G<br>UNKNOWN INJECTION . . . H<br><br>(IV) INTRAVENOUS I<br><br>HOME REMEDY/ HERBAL MED-ICINE . . . . J<br><br>OTHER ________ X<br>(SPECIFY)	PILL OR SYRUP<br>ANTIBIOTIC . . . . . A<br>ANTIMOTILITY B<br>ZINC . . . . C<br>OTHER (NOT ANTI-BIOTIC, ANTI-MOTILITY, OR ZINC) . . . . D<br>UNKNOWN PILL OR SYRUP . . . E<br><br>INJECTION<br>ANTIBIOTIC . . . . . F<br>NON-ANTIBIOTIC G<br>UNKNOWN INJECTION . . . H<br><br>(IV) INTRAVENOUS I<br><br>HOME REMEDY/ HERBAL MED-ICINE . . . . J<br><br>OTHER ________ X<br>(SPECIFY)	PILL OR SYRUP<br>ANTIBIOTIC . . . . . A<br>ANTIMOTILITY B<br>ZINC . . . . C<br>OTHER (NOT ANTI-BIOTIC, ANTI-MOTILITY, OR ZINC) . . . . D<br>UNKNOWN PILL OR SYRUP . . . E<br><br>INJECTION<br>ANTIBIOTIC . . . . . F<br>NON-ANTIBIOTIC G<br>UNKNOWN INJECTION . . . H<br><br>(IV) INTRAVENOUS I<br><br>HOME REMEDY/ HERBAL MED-ICINE . . . . J<br><br>OTHER ________ X<br>(SPECIFY)
525	Has (NAME) been ill with a fever at any time in the last 2 weeks?	YES . . . . 1<br>NO . . . . 2<br>(SKIP TO 527)<br>DON'T KNOW 8	YES . . . . 1<br>NO . . . . 2<br>(SKIP TO 527)<br>DON'T KNOW 8	YES . . . . 1<br>NO . . . . 2<br>(SKIP TO 527)<br>DON'T KNOW 8
526	At any time during the illness, did (NAME) have blood taken from his/her finger or heel for testing?	YES . . . . 1<br>NO . . . . 2<br>DON'T KNOW 8	YES . . . . 1<br>NO . . . . 2<br>DON'T KNOW 8	YES . . . . 1<br>NO . . . . 2<br>DON'T KNOW 8
527	Has (NAME) had an illness with a cough at any time in the last 2 weeks?	YES . . . . 1<br>NO . . . . 2<br>(SKIP TO 530)<br>DON'T KNOW 8	YES . . . . 1<br>NO . . . . 2<br>(SKIP TO 530)<br>DON'T KNOW 8	YES . . . . 1<br>NO . . . . 2<br>(SKIP TO 530)<br>DON'T KNOW 8
528	When (NAME) had an illness with a cough, did he/she breathe faster than usual with short, rapid breaths or have difficulty breathing?	YES . . . . 1<br>NO . . . . 2<br>(SKIP TO 531)<br>DON'T KNOW 8	YES . . . . 1<br>NO . . . . 2<br>(SKIP TO 531)<br>DON'T KNOW 8	YES . . . . 1<br>NO . . . . 2<br>(SKIP TO 531)<br>DON'T KNOW 8
529	Was the fast or difficult breathing due to a problem in the chest or to a blocked or runny nose?	CHEST ONLY . . . 1<br>NOSE ONLY . . . . . 2<br>BOTH . . . . 3<br>OTHER ________ 6<br>(SPECIFY)<br>DON'T KNOW . . . . . 8<br>(SKIP TO 531)	CHEST ONLY . . . 1<br>NOSE ONLY . . . . . 2<br>BOTH . . . . 3<br>OTHER ________ 6<br>(SPECIFY)<br>DON'T KNOW . . . . . 8<br>(SKIP TO 531)	CHEST ONLY . . . 1<br>NOSE ONLY . . . . . 2<br>BOTH . . . . 3<br>OTHER ________ 6<br>(SPECIFY)<br>DON'T KNOW . . . . . 8<br>(SKIP TO 531)

NO.	QUESTIONS AND FILTERS	LAST BIRTH<br>NAME ________	NEXT-TO-LAST BIRTH<br>NAME ________	SECOND-FROM-LAST BIRTH<br>NAME ________
530	CHECK 525:<br><br>HAD FEVER?	YES ☐ ↓ NO OR DK ☐ → (GO BACK TO 503 IN NEXT COLUMN; OR, IF NO MORE BIRTHS, GO TO 553)	YES ☐ ↓ NO OR DK ☐ → (GO BACK TO 503 IN NEXT COLUMN; OR, IF NO MORE BIRTHS, GO TO 553)	YES ☐ ↓ NO OR DK ☐ → (GO TO 503 IN NEXT-TO-LAST COLUMN OF NEW QUESTIONNAIRE; OR, IF NO MORE BIRTHS, GO TO 553)
531	Now I would like to know how much (NAME) was given to drink (including breastmilk) during the illness with a (fever/cough).<br>Was he/she given less than usual to drink, about the same amount, or more than usual to drink?<br><br>IF LESS, PROBE: Was he/she given much less than usual to drink or somewhat less?	MUCH LESS ..... 1<br>SOMEWHAT LESS 2<br>ABOUT THE SAME 3<br>MORE .... 4<br>NOTHING TO DRINK 5<br>DON'T KNOW 8	MUCH LESS ..... 1<br>SOMEWHAT LESS 2<br>ABOUT THE SAME 3<br>MORE .... 4<br>NOTHING TO DRINK 5<br>DON'T KNOW 8	MUCH LESS ..... 1<br>SOMEWHAT LESS 2<br>ABOUT THE SAME 3<br>MORE .... 4<br>NOTHING TO DRINK 5<br>DON'T KNOW 8
532	When (NAME) had a (fever/cough), was he/she given less than usual to eat, about the same amount, more than usual, or nothing to eat?<br><br>IF LESS, PROBE: Was he/she given much less than usual to eat or somewhat less?	MUCH LESS ..... 1<br>SOMEWHAT LESS 2<br>ABOUT THE SAME 3<br>MORE .... 4<br>STOPPED FOOD 5<br>NEVER GAVE FOOD 6<br>DON'T KNOW 8	MUCH LESS ..... 1<br>SOMEWHAT LESS 2<br>ABOUT THE SAME 3<br>MORE .... 4<br>STOPPED FOOD 5<br>NEVER GAVE FOOD 6<br>DON'T KNOW 8	MUCH LESS ..... 1<br>SOMEWHAT LESS 2<br>ABOUT THE SAME 3<br>MORE .... 4<br>STOPPED FOOD 5<br>NEVER GAVE FOOD 6<br>DON'T KNOW 8
533	Did you seek advice or treatment for the illness from any source?	YES .... 1<br>NO .... 2<br>(SKIP TO 537)←	YES .... 1<br>NO .... 2<br>(SKIP TO 537)←	YES .... 1<br>NO .... 2<br>(SKIP TO 537)←

NO.	QUESTIONS AND FILTERS	LAST BIRTH<br>NAME ______	NEXT-TO-LAST BIRTH<br>NAME ______	SECOND-FROM-LAST BIRTH<br>NAME ______
534	Where did you seek advice or treatment?<br><br>Anywhere else?<br><br>PROBE TO IDENTIFY EACH TYPE OF SOURCE.<br><br>IF UNABLE TO DETERMINE IF PUBLIC OR PRIVATE SECTOR, WRITE THE NAME OF THE PLACE.<br><br>______<br>(NAME OF PLACE(S))	PUBLIC SECTOR<br>NATL HOSP (PP) A<br>PROV HOSP (RH) B<br>DIST HOSP (RH) C<br>HLTH CENTER . D<br>HLTH POST . . . E<br>OUTREACH . . . F<br>MILITARY HOSP G<br>OTHER PUBLIC SECTOR . . .<br>______ H<br>(SPECIFY)<br><br>PRIVATE MEDICAL SECTOR<br>PRIV. HOSP . . . I<br>PRIV. CLINIC . . . J<br>PHARMACY . . . K<br>HOME OF TRAINED HEALTH WORKER . . . L<br>VISIT OF TRAINED HEALTH WORKER . . . M<br>OTHER PRIVATE MED. SECTOR<br>______ N<br>(SPECIFY)<br><br>OTHER SOURCE<br>SHOP . . . . O<br>TRADITIONAL PRACTITIONER P<br><br>OTHER ______ X<br>(SPECIFY)	PUBLIC SECTOR<br>NATL HOSP (PP) A<br>PROV HOSP (RH) B<br>DIST HOSP (RH) C<br>HLTH CENTER . D<br>HLTH POST . . . E<br>OUTREACH . . . F<br>MILITARY HOSP G<br>OTHER PUBLIC SECTOR . . .<br>______ H<br>(SPECIFY)<br><br>PRIVATE MEDICAL SECTOR<br>PRIV. HOSP . . . I<br>PRIV. CLINIC . . . J<br>PHARMACY . . . K<br>HOME OF TRAINED HEALTH WORKER . . . L<br>VISIT OF TRAINED HEALTH WORKER . . . M<br>OTHER PRIVATE MED. SECTOR<br>______ N<br>(SPECIFY)<br><br>OTHER SOURCE<br>SHOP . . . . O<br>TRADITIONAL PRACTITIONER P<br><br>OTHER ______ X<br>(SPECIFY)	PUBLIC SECTOR<br>NATL HOSP (PP) A<br>PROV HOSP (RH) B<br>DIST HOSP (RH) C<br>HLTH CENTER . D<br>HLTH POST . . . E<br>OUTREACH . . . F<br>MILITARY HOSP G<br>OTHER PUBLIC SECTOR . . .<br>______ H<br>(SPECIFY)<br><br>PRIVATE MEDICAL SECTOR<br>PRIV. HOSP . . . I<br>PRIV. CLINIC . . . J<br>PHARMACY . . . K<br>HOME OF TRAINED HEALTH WORKER . . . L<br>VISIT OF TRAINED HEALTH WORKER . . . M<br>OTHER PRIVATE MED. SECTOR<br>______ N<br>(SPECIFY)<br><br>OTHER SOURCE<br>SHOP . . . . O<br>TRADITIONAL PRACTITIONER P<br><br>OTHER ______ X<br>(SPECIFY)
535	CHECK 534:	☐ TWO OR MORE CODES CIRCLED<br>☐ ONLY ONE CODE CIRCLED (SKIP TO 537)	☐ TWO OR MORE CODES CIRCLED<br>☐ ONLY ONE CODE CIRCLED (SKIP TO 537)	☐ TWO OR MORE CODES CIRCLED<br>☐ ONLY ONE CODE CIRCLED (SKIP TO 537)
536	Where did you first seek advice or treatment?<br><br>USE LETTER CODE FROM 534.	FIRST PLACE . . . ☐	FIRST PLACE . . . ☐	FIRST PLACE . . . ☐
537	At any time during the illness, did (NAME) take any drugs for the illness?	YES . . . . 1<br>NO . . . . 2<br>(GO BACK TO 503 IN NEXT COLUMN; OR, IF NO MORE BIRTHS, GO TO 553)<br>DON'T KNOW 8	YES . . . . 1<br>NO . . . . 2<br>(GO BACK TO 503 IN NEXT COLUMN; OR, IF NO MORE BIRTHS, GO TO 553)<br>DON'T KNOW 8	YES . . . . 1<br>NO . . . . 2<br>(GO TO 503 IN NEXT-TO-LAST COLUMN OF NEW QUESTIONNAIRE; OR, IF NO MORE BIRTHS, GO TO 553)<br>DON'T KNOW 8

NO.	QUESTIONS AND FILTERS	LAST BIRTH<br>NAME ________	NEXT-TO-LAST BIRTH<br>NAME ________	SECOND-FROM-LAST BIRTH<br>NAME ________
538	What drugs did (NAME) take?<br><br>Any other drugs?<br><br>RECORD ALL MENTIONED.	ANTIMALARIAL DRUGS<br>SP/FANSIDAR . . . A<br>CHLOROQUINE B<br>QUININE . . . . C<br>MALARINE . . . . . D<br>A+M (2, 3, 4) . . . E<br>MEFLOQUINE . . . F<br>ARTEMISININ . . . G<br>ARTESUNATE TABLET . . . . . H<br>ARTESUNATE SUPPOSITORY I<br>ARTEKINE . . . . . J<br>COTEXIN . . . . . K<br>OTHER ANTI-MALARIAL ________ . . . L<br>(SPECIFY)<br><br>ANTIBIOTIC DRUGS<br>PILL/SYRUP . . . M<br>INJECTION . . . N<br><br>OTHER DRUGS<br>ASPIRIN . . . . O<br>ACETA-MINOPHEN . . . P<br>IBUPROFEN . . . Q<br>DRUG COCKTAIL R<br>MULTIVITAMIN . S<br><br>OTHER ________ X<br>(SPECIFY)<br>DON'T KNOW . . . . . Z	ANTIMALARIAL DRUGS<br>SP/FANSIDAR . . . A<br>CHLOROQUINE B<br>QUININE . . . . C<br>MALARINE . . . . . D<br>A+M (2, 3, 4) . . . E<br>MEFLOQUINE . . . F<br>ARTEMISININ . . . G<br>ARTESUNATE TABLET . . . . . H<br>ARTESUNATE SUPPOSITORY I<br>ARTEKINE . . . . . J<br>COTEXIN . . . . . K<br>OTHER ANTI-MALARIAL ________ . . . L<br>(SPECIFY)<br><br>ANTIBIOTIC DRUGS<br>PILL/SYRUP . . . M<br>INJECTION . . . N<br><br>OTHER DRUGS<br>ASPIRIN . . . . O<br>ACETA-MINOPHEN . . . P<br>IBUPROFEN . . . Q<br>DRUG COCKTAIL R<br>MULTIVITAMIN . S<br><br>OTHER ________ X<br>(SPECIFY)<br>DON'T KNOW . . . . . Z	ANTIMALARIAL DRUGS<br>SP/FANSIDAR . . . A<br>CHLOROQUINE B<br>QUININE . . . . C<br>MALARINE . . . . . D<br>A+M (2, 3, 4) . . . E<br>MEFLOQUINE . . . F<br>ARTEMISININ . . . G<br>ARTESUNATE TABLET . . . . . H<br>ARTESUNATE SUPPOSITORY I<br>ARTEKINE . . . . . J<br>COTEXIN . . . . . K<br>OTHER ANTI-MALARIAL ________ . . . L<br>(SPECIFY)<br><br>ANTIBIOTIC DRUGS<br>PILL/SYRUP . . . M<br>INJECTION . . . N<br><br>OTHER DRUGS<br>ASPIRIN . . . . O<br>ACETA-MINOPHEN . . . P<br>IBUPROFEN . . . Q<br>DRUG COCKTAIL R<br>MULTIVITAMIN . S<br><br>OTHER ________ X<br>(SPECIFY)<br>DON'T KNOW . . . . . Z
552		GO BACK TO 503 IN NEXT COLUMN; OR, IF NO MORE BIRTHS, GO TO 553.	GO BACK TO 503 IN NEXT COLUMN; OR, IF NO MORE BIRTHS, GO TO 553.	GO TO 503 IN NEXT-TO-LAST COLUMN OF NEW QUESTIONNAIRE; OR, IF NO MORE BIRTHS, GO TO 553.

NO.	QUESTIONS AND FILTERS	CODING CATEGORIES	SKIP
553	CHECK 215 AND 218, ALL ROWS:<br><br>NUMBER OF CHILDREN BORN IN 2009 OR LATER LIVING WITH THE RESPONDENT<br><br>ONE OR MORE ☐ ↓ NONE ☐ →<br><br>RECORD NAME OF YOUNGEST CHILD LIVING WITH HER AND CONTINUE WITH 554<br><br>________________<br>(NAME)		556
554	The last time (NAME FROM 553) passed stools, what was done to dispose of the stools?	CHILD USED TOILET OR LATRINE . . . 01<br>PUT/RINSED<br>INTO TOILET OR LATRINE . . . . 02<br>PUT/RINSED<br>INTO DRAIN OR DITCH . . . . 03<br>THROWN INTO GARBAGE . . . . 04<br>BURIED . . . . 05<br>LEFT IN THE OPEN . . . . 06<br>OTHER ________________ 96<br>(SPECIFY)	
555	CHECK 522(a) AND 522(b), ALL COLUMNS:<br><br>NO CHILD RECEIVED FLUID FROM ORS PACKET OR PRE-PACKAGED ORS LIQUID ☐ ↓<br><br>ANY CHILD RECEIVED FLUID FROM ORS PACKET OR PRE-PACKAGED ORS LIQUID ☐ →		557
556	Have you ever heard of a special product called Oralyte/Orasel you can get for the treatment of diarrhea?	YES . . . . 1<br>NO 2	
557	CHECK 215 AND 218, ALL ROWS:<br><br>NUMBER OF CHILDREN BORN IN 2012 OR LATER LIVING WITH THE RESPONDENT<br><br>ONE OR MORE ☐ ↓ NONE ☐ →<br><br>RECORD NAME OF YOUNGEST CHILD LIVING WITH HER AND CONTINUE WITH 558<br><br>________________<br>(NAME)		563

NO.	QUESTIONS AND FILTERS		CODING CATEGORIES				SKIP
558	Now I would like to ask you about liquids or foods that (NAME FROM 557) had yesterday during the day or at night. I am interested in whether your child had the item I mention even if it was combined with other foods.						
	Did (NAME FROM 557) (drink/eat):			YES	NO	DK	
	a)	Plain water?	**a)**	1	2	8	
	b)	Juice or juice drinks?	**b)**	1	2	8	
	c)	Soup?	**c)**	1	2	8	
	d)	Milk such as tinned, powdered, or fresh animal milk?	**d)**	1	2	8	
		IF YES: How many times did (NAME) drink milk?<br>IF 7 OR MORE TIMES, RECORD '7'.	NUMBER OF TIMES<br>DRANK MILK	☐			
	e)	Infant formula?	**e)**	1	2	8	
		IF YES: How many times did (NAME) drink infant formula?<br>IF 7 OR MORE TIMES, RECORD '7'.	NUMBER OF TIMES<br>DRANK FORMULA	☐			
	f)	Any other liquids?	**f)**	1	2	8	
	g)	Yogurt?	**g)**	1	2	8	
		IF YES: How many times did (NAME) eat yogurt?<br>IF 7 OR MORE TIMES, RECORD '7'.	NUMBER OF TIMES<br>ATE YOGURT	☐			
	h)	Any [BRAND NAME OF COMMERCIALLY FORTIFIED BABY FOOD, E.G., Cerelac]?	**h)**	1	2	8	
	i)	Bread, rice, noodles, porridge, or other foods made from grains?	**i)**	1	2	8	
	j)	Pumpkin, carrots, squash or sweet potatoes that are yellow or orange inside?	**j)**	1	2	8	
	k)	White potatoes, white yams, manioc, cassava, or any other foods made from roots?	**k)**	1	2	8	
	l)	Any dark green, leafy vegetables?	**l)**	1	2	8	
	m)	Ripe mangoes, papayas or [INSERT ANY OTHER LOCALLY AVAILABLE VITAMIN A-RICH FRUITS]?	**m)**	1	2	8	
	n)	Any other fruits or vegetables?	**n)**	1	2	8	
	o)	Liver, kidney, heart or other organ meats?	**o)**	1	2	8	
	p)	Any meat, such as beef, pork, lamb, goat, chicken, or duck?	**p)**	1	2	8	
		IF YES: How many times did (NAME) eat meat?<br>IF 7 OR MORE TIMES, RECORD '7'.	NUMBER OF TIMES<br>ATE MEAT	☐			
	q)	Eggs?	**q)**	1	2	8	
	r)	Fresh or dried fish or shellfish?	**r)**	1	2	8	
	s)	Any foods made from beans, peas, lentils, or nuts?	**s)**	1	2	8	
	t)	Cheese or other food made from milk?	**t)**	1	2	8	
	u)	Any foods made with oil, fat, or butter?	**u)**	1	2	8	
	v)	Any snake, snail, frog, rat, or insects?	**v)**	1	2	8	
	w)	Any sugary foods such as pastry, cakes, chocolates, sweets or candies ?	**w)**	1	2	8	
	x)	Any other solid, semi-solid, or soft food?	**x)**	1	2	8	

NO.	QUESTIONS AND FILTERS	CODING CATEGORIES	SKIP
559	CHECK 558 (CATEGORIES "g" THROUGH "x"):<br>ALL "NO" ☐ ↓<br>AT LEAST ONE "YES" OR ALL DKs ☐ → 561		561
560	Did (NAME) eat any solid, semi-solid, or soft foods yesterday during the day or at night?<br><br>IF 'YES' PROBE: What kind of solid, semi-solid or soft foods did (NAME) eat?	YES . . . . 1<br>(GO BACK TO 558 TO RECORD FOOD EATEN YESTERDAY)<br><br>NO 2	 <br><br>563
561	How many times did (NAME FROM 557) eat solid, semisolid, or soft foods yesterday during the day or at night?<br><br>IF 7 OR MORE TIMES, RECORD '7'.	NUMBER OF TIMES . . . . ☐<br><br>DON'T KNOW 8	

NO.	QUESTIONS AND FILTERS	CODINGS CATEGORIES	SKIP
563	CHECK Q.217 AND Q.218, ALL ROW:<br>AT LEAST ONE CHILD AGED 0-5 YEARS OLD AND LIVE WITH THE RESPONDENT<br>YES ☐ NO ☐		601
564	CHECK Q.217<br>SELECT THE YOUNGEST CHILD AGED 0-5 YEARS OLD, RECORD THE CHILD NAME AND LINE NUMBER<br>NAME OF THE YOUNGEST CHILD FROM Q.212 ________	LINE NUMBER OF THE YOUNGEST CHILD (Q.219) ☐☐	
565	Now I would like to ask you about (NAME); your youngest child that is 0-5 years old		
566	How many children's books or picture books do you have for (NAME)?	NONE . . . . . 00<br>NUMBER OF CHILDREN'S BOOKS . . 0 ☐<br>TEN OR MORE BOOKS 10	
567	I am interested in learning about the things that (name) plays with when he/she is at home.<br>Does he/she play with:<br>a) Homemade toys (such as dolls, cars, or other toys made at home)?<br>b) Toys from a shop or manufactured toys?<br>c) Household objects (such as bowls or pots) or objects found outside (such as sticks, rocks, animal shells or leaves)?<br>IF THE RESPONDENT SAYS "YES" TO THE CATEGORIES ABOVE, THEN PROBE TO LEARN SPECIFICALLY WHAT THE CHILD PLAYS WITH TO ASCERTAIN THE RESPONSE.	OUI NON NSP<br>HOMEMADE TOYS . . . . . 1 2 8<br>TOYS FROM SHOP . . . . . 1 2 8<br>HOUSEHOLD OBJECTS OR OUTSIDE OBJECTS 1 2 8	
568	Sometimes adults taking care of children have to leave the house to go shopping, wash clothes, or for other reasons and have to leave young children.<br>On how many days in the past week was (name):<br>a) Left alone for more than an hour?<br>b) Left in the care of another child, that is, someone less than 10 years old, for more than an hour?<br>IF 'NONE' ENTER' 0'. IF 'DON'T KNOW' ENTER'8'	NUMBER OF DAYS LEFT ALONE MORE THAN AN HOURS . ☐<br>NUMBER OF DAYS LEFT WITH ANOTHER CHILD FOR MORE THAN AN HOURS ☐	
569	CHECK Q.217 ET 218:<br>A CHILD AGED 3, 4 OR 5 YEARS OLD; LIVE IN THIS HOUSEHOLD (Q.217=3 OR 4 AND Q.218=1)?<br>YES ☐ NO ☐		601
570	CHECK Q.217:<br>SELECT THE YOUNGEST CHILD AGED 3 , 4 OR 5 YEARS OLD. RECORD THE CHILD'S NAME AND LINE NUMBER<br>NAME OF THE YOUGEST CHILD 3, 4 OR 5 YEARS OLD (Q.212) ________	LINE NUMBER OF THE YOUNGEST CHILD (Q.219) ☐☐	
571	Now I would to ask some questions regarding (NAME), your youngest child aged 3-5 years old.		

572	Does (NAME) attend any organized learning or early childhood education programme, such as a private or government facility, including kindergarten or community child care?	YES . . . . 1<br>NO . . . . 2<br>DON'T KNOW 8	2, 8 → 574
573	In the past 7 days, about how many hours did (NAME) go to that place:	NUMBER OF HOURS . . . . [ ][ ]	
574	In the past 3 days, did you or any household member age 15 or over engage in any of the following activities with (NAME):<br><br>ENCERCLEZ TOUT CE QUI EST MENTIONNÉ.<br><br>a) Read books to or looked at picture with (NAME)?<br>b) Told stories to (NAME)?<br>c) Sang songs to (NAME) or with (NAME), including lullabies?<br>d) Took (NAME) outside the home, compound, yard or enclosure?<br>e) Played with (NAME)<br>f) Named, counted, or drew things to or with (NAME)?	MOM / DAD / OTHER / NO ONE<br>READB OOKS . . . . A B X Y<br>TOLD STORIES . . . . A B X Y<br>SANG SONGS . . . . A B X Y<br>TOOK OUTSIDE . . . . A B X Y<br>PLAYED . . . . A B X Y<br>NAMED/COUNTED . . . A B X Y	
575	I would like to ask you some questions about the health and development of (NAME). Children do not all develop and learn at the same rate. For example, some walk earlier than others. These questions are related to several aspects of (NAME)'s development.<br><br>Can (NAME) identify or name at least ten letters of the alphabet?	YES . . . . 1<br>NO . . . . 2<br>DK 8	
576	Can (NAME) read at least four simple, popular words?	YES . . . . 1<br>NO . . . . 2<br>DK 8	
577	Does (NAME) know the name and recognize the symbol of all numbers from 1 to 10?	YES . . . . 1<br>NO . . . . 2<br>DK 8	
578	Can (NAME) pick up a small object with two fingers, like a stick or a rock from the ground?	YES . . . . 1<br>NO . . . . 2<br>DK 8	
579	Is (NAME) sometimes too sick to play?	YES . . . . 1<br>NO . . . . 2<br>DK 8	
580	Does (NAME) follow simple directions on how to do something correctly?	YES . . . . 1<br>NO . . . . 2<br>DK 8	
581	When given something to do, is (NAME) able to do it independently?	YES . . . . 1<br>NO . . . . 2<br>DK 8	
582	Does (NAME) get along well with other children?	YES . . . . 1<br>NO . . . . 2<br>DK 8	
583	Does (NAME) kick, bite, or hit other children or adults?	YES . . . . 1<br>NO . . . . 2<br>DK 8	
584	Does (NAME) get distracted easily?	YES . . . . 1<br>NO . . . . 2<br>DK 8	

SECTION 6. MARRIAGE AND SEXUAL ACTIVITY

NO.	QUESTIONS AND FILTERS	CODING CATEGORIES	SKIP
601	Are you currently married or living together with a man as if married?	YES, CURRENTLY MARRIED . . . . 1<br>YES, LIVING WITH A MAN . . . . 2<br>NO, NOT IN UNION 3	1, 2 → 604
602	Have you ever been married or lived together with a man as if married?	YES, FORMERLY MARRIED . . . . 1<br>YES, LIVED WITH A MAN . . . . 2<br>NO 3	3 → 612
603	What is your marital status now: are you widowed, divorced, or separated?	WIDOWED . . . . 1<br>DIVORCED . . . . 2<br>SEPARATED 3	→ 609
604	Is your (husband/partner) living with you now or is he staying elsewhere?	LIVING WITH HER . . . . 1<br>STAYING ELSEWHERE 2	
605	RECORD THE HUSBAND'S/PARTNER'S NAME AND LINE NUMBER FROM THE HOUSEHOLD QUESTIONNAIRE.<br>IF HE IS NOT LISTED IN THE HOUSEHOLD, RECORD '00'.	NAME ____________________<br>LINE NO. . . . . [ ][ ]	
606	Does your (husband/partner) have other wives or does he live with other women as if married?	YES . . . . 1<br>NO . . . . 2<br>DON'T KNOW 8	2, 8 → 609
607	Including yourself, in total, how many wives or live-in partners does he have?	TOTAL NUMBER OF WIVES AND LIVE-IN PARTNERS . . . . . [ ][ ]<br>DON'T KNOW 98	
608	Are you the first, second, … wife?	RANK . . . . [ ][ ]	
609	Have you been married or lived with a man only once or more than once?	ONLY ONCE . . . . 1<br>MORE THAN ONCE 2	
610	CHECK 609:<br>MARRIED/ LIVED WITH A MAN ONLY ONCE [ ] ↓<br>In what month and year did you start living with your (husband/partner)?<br>MARRIED/ LIVED WITH A MAN MORE THAN ONCE [ ] ↓<br>Now I would like to ask about your first (husband/partner). In what month and year did you start living with him?<br>IF RESPONDENT DOES NOT KNOW GREGORIAN DATE, ASK FOR KHMER DATE OF MARRIAGE.<br>USE DATE CONVERSION CHART TO FIND GREGORIAN MONTH AND YEAR.<br>______________________________<br>(SPECIFY KHMER MONTH AND YEAR OF MARRIAGE)	GREGORIAN MONTH . . . . [ ][ ]<br>DON'T KNOW MONTH . . . . 98<br>GREGORIAN YEAR [ ][ ][ ][ ]<br>DON'T KNOW YEAR 9998	→ 612
611	How old were you when you first started living with him?	AGE . . . . [ ][ ]	
612	CHECK FOR THE PRESENCE OF OTHERS. BEFORE CONTINUING, MAKE EVERY EFFORT TO ENSURE PRIVACY.		
613	Now I would like to ask some questions about sexual activity in order to gain a better understanding of some important life issues.<br>How old were you when you had sexual intercourse for the very first time?	NEVER HAD SEXUAL INTERCOURSE . . . . .00<br>AGE IN YEARS . . . . [ ][ ]<br>FIRST TIME WHEN STARTED LIVING WITH (FIRST) HUSBAND/PARTNER 95	00 → 628

NO.	QUESTIONS AND FILTERS	CODING CATEGORIES	SKIP
614	Now I would like to ask you some questions about your recent sexual activity. Let me assure you again that your answers are completely confidential and will not be told to anyone. If we should come to any question that you don't want to answer, just let me know and we will go to the next question.		
615	When was the last time you had sexual intercourse?<br><br>IF LESS THAN 12 MONTHS, ANSWER MUST BE RECORDED IN DAYS, WEEKS OR MONTHS.<br>IF 12 MONTHS (ONE YEAR) OR MORE, ANSWER MUST BE RECORDED IN YEARS.	DAYS AGO . . . . 1 ☐☐<br>WEEKS AGO . . . . 2 ☐☐<br>MONTHS AGO . . . . 3 ☐☐<br>YEARS AGO . . . . 4 ☐☐	 <br><br>→ 627

		LAST SEXUAL PARTNER	SECOND-TO-LAST SEXUAL PARTNER	THIRD-TO-LAST SEXUAL PARTNER
616	When was the last time you had sexual intercourse with this person?		DAYS AGO 1 [ ][ ]<br>WEEKS AGO 2 [ ][ ]<br>MONTHS AGO 3 [ ][ ]	DAYS AGO 1 [ ][ ]<br>WEEKS AGO 2 [ ][ ]<br>MONTHS AGO 3 [ ][ ]
617	The last time you had sexual intercourse (with this second/third person), was a condom used?	YES . . . . 1<br>NO . . . . 2<br>(SKIP TO 619)	YES . . . . 1<br>NO . . . . 2<br>(SKIP TO 619)	YES . . . . 1<br>NO . . . . 2<br>(SKIP TO 619)
618	Was a condom used every time you had sexual intercourse with this person in the last 12 months?	YES . . . . 1<br>NO 2	YES . . . . 1<br>NO 2	YES . . . . 1<br>NO 2
619	What was your relationship to this person with whom you had sexual intercourse?<br><br>IF BOYFRIEND:<br>Were you living together as if married?<br>IF YES, CIRCLE '2'.<br>IF NO, CIRCLE '3'.	HUSBAND . . . . 1<br>LIVE-IN PARTNER . . . 2<br>BOYFRIEND NOT LIVING WITH RESPONDENT . . . 3<br>CASUAL ACQUAINTANCE . . . 4<br>PROSTITUTE . . . . 5<br>OTHER ____________ 6<br>(SPECIFY)<br>(SKIP TO 622)	HUSBAND . . . . 1<br>LIVE-IN PARTNER . . . 2<br>BOYFRIEND NOT LIVING WITH RESPONDENT . . . 3<br>CASUAL ACQUAINTANCE . . . 4<br>PROSTITUTE . . . . 5<br>OTHER ____________ 6<br>(SPECIFY)<br>(SKIP TO 622)	HUSBAND . . . . 1<br>LIVE-IN PARTNER . . . 2<br>BOYFRIEND NOT LIVING WITH RESPONDENT . . . 3<br>CASUAL ACQUAINTANCE . . . 4<br>PROSTITUTE . . . . 5<br>OTHER ____________ 6<br>(SPECIFY)<br>(SKIP TO 622)
620	CHECK 609:	MARRIED ONLY ONCE [ ]<br>MARRIED MORE THAN ONCE [ ] (SKIP TO 622)	MARRIED ONLY ONCE [ ]<br>MARRIED MORE THAN ONCE [ ] (SKIP TO 622)	MARRIED ONLY ONCE [ ]<br>MARRIED MORE THAN ONCE [ ] (SKIP TO 622)
621	CHECK 613:	FIRST TIME WHEN STARTED LIVING WITH FIRST HUSBAND (SKIP TO 623)<br>OTHER [ ]	FIRST TIME WHEN STARTED LIVING WITH FIRST HUSBAND (SKIP TO 623)<br>OTHER [ ]	FIRST TIME WHEN STARTED LIVING WITH FIRST HUSBAND (SKIP TO 623)<br>OTHER [ ]
622	How long ago did you first have sexual intercourse with this (second/third) person?	DAYS AGO 1 [ ][ ]<br>WEEKS AGO 2 [ ][ ]<br>MONTHS AGO 3 [ ][ ]<br>YEARS AGO 4 [ ][ ]	DAYS AGO 1 [ ][ ]<br>WEEKS AGO 2 [ ][ ]<br>MONTHS AGO 3 [ ][ ]<br>YEARS AGO 4 [ ][ ]	DAYS AGO 1 [ ][ ]<br>WEEKS AGO 2 [ ][ ]<br>MONTHS AGO 3 [ ][ ]<br>YEARS AGO 4 [ ][ ]
623	How many times during the last 12 months did you have sexual intercourse with this person?	NUMBER OF TIMES [ ][ ]	NUMBER OF TIMES [ ][ ]	NUMBER OF TIMES [ ][ ]
624	How old is this person?	AGE OF PARTNER [ ][ ]<br>DON'T KNOW 98	AGE OF PARTNER [ ][ ]<br>DON'T KNOW 98	AGE OF PARTNER [ ][ ]<br>DON'T KNOW 98
625	Apart from (this person/these two people), have you had sexual intercourse with any other person in the last 12 months?	YES . . . . 1<br>(GO BACK TO 616 IN NEXT COLUMN)<br>NO . . . . 2<br>(SKIP TO 627)	YES . . . . 1<br>(GO BACK TO 616 IN NEXT COLUMN)<br>NO . . . . 2<br>(SKIP TO 627)	
626	In total, with how many different people have you had sexual intercourse in the last 12 months?<br>IF NON-NUMERIC ANSWER, PROBE TO GET AN ESTIMATE.<br>IF NUMBER OF PARTNERS IS 95 OR MORE, WRITE '95'.			NUMBER OF PARTNERS LAST 12 MONTHS . . . [ ][ ]<br>DON'T KNOW . . . 98

NO.	QUESTIONS AND FILTERS	CODING CATEGORIES	SKIP
627	In total, with how many different people have you had sexual intercourse in your lifetime?<br><br>IF NON-NUMERIC ANSWER, PROBE TO GET AN ESTIMATE.<br><br>IF NUMBER OF PARTNERS IS 95 OR MORE, WRITE '95'.	NUMBER OF PARTNERS IN LIFETIME . . . . [ ][ ]<br><br>DON'T KNOW 98	
628	PRESENCE OF OTHERS DURING THIS SECTION	YES NO<br>CHILDREN <10 . . . . 1 2<br>MALE ADULTS . . . . 1 2<br>FEMALE ADULTS 1 2	
629	Do you know of a place where a person can get condoms?	YES . . . . 1<br>NO 2	 → 632
630	Where is that?<br><br>Any other place?<br><br>PROBE TO IDENTIFY EACH TYPE OF SOURCE.<br><br>IF UNABLE TO DETERMINE IF PUBLIC OR PRIVATE SECTOR, WRITE THE NAME OF THE PLACE.<br><br>____________________<br>(NAME OF PLACE(S))	PUBLIC SECTOR<br>NATIONAL HOSPITAL (PP) . . . . A<br>PROVINCIAL HOSP (RH) . . . . B<br>DISTRICT HOSPITAL (RH) . . . . C<br>HEALTH CENTER . . . . D<br>HEALTH POST . . . . E<br>OUTREACH . . . . F<br>MILITARY HOSPITAL . . . . G<br>OTHER PUBLIC<br>SECTOR ____________ H<br>(SPECIFY)<br><br>PRIVATE MEDICAL SECTOR<br>PRIVATE HOSPITAL . . . . I<br>PRIVATE CLINIC . . . . J<br>PHARMACY . . . . K<br>OTHER PRIVATE MEDICAL<br>SECTOR ____________ L<br>(SPECIFY)<br><br>OTHER SOURCE<br>SHOP . . . . M<br>COMMUNITY DISTRIBUTOR . . . . . N<br>FRIENDS/RELATIVES . . . . O<br><br>OTHER ____________ X<br>(SPECIFY)	
631	If you wanted to, could you yourself get a condom?	YES . . . . 1<br>NO . . . . 2<br>DON'T KNOW/UNSURE 8	
632	Do you know of a place where a person can get female condoms?	YES . . . . 1<br>NO 2	 → 701

NO.	QUESTIONS AND FILTERS	CODING CATEGORIES	SKIP
633	Where is that?<br><br>Any other place?<br><br>PROBE TO IDENTIFY EACH TYPE OF SOURCE.<br><br>IF UNABLE TO DETERMINE IF PUBLIC OR PRIVATE SECTOR, WRITE THE NAME OF THE PLACE.<br><br>________________<br>(NAME OF PLACE(S))	PUBLIC SECTOR<br>NATIONAL HOSPITAL (PP) . . . . A<br>PROVINCIAL HOSP (RH) . . . . B<br>DISTRICT HOSPITAL (RH) . . . . C<br>HEALTH CENTER . . . . D<br>HEALTH POST . . . . E<br>OUTREACH . . . . F<br>MILITARY HOSPITAL . . . . G<br>OTHER PUBLIC<br>SECTOR __________ H<br>(SPECIFY)<br><br>PRIVATE MEDICAL SECTOR<br>PRIVATE HOSPITAL . . . . I<br>PRIVATE CLINIC . . . . J<br>PHARMACY . . . . K<br>OTHER PRIVATE MEDICAL<br>SECTOR __________ L<br>(SPECIFY)<br><br>OTHER SOURCE<br>SHOP . . . . M<br>COMMUNITY DISTRIBUTOR . . . . . N<br>FRIENDS/RELATIVES . . . . O<br><br>OTHER __________ X<br>(SPECIFY)	
634	If you wanted to, could you yourself get a female condom?	YES . . . . 1<br>NO . . . . 2<br>DON'T KNOW/UNSURE 8	

SECTION 7. FERTILITY PREFERENCES

NO.	QUESTIONS AND FILTERS	CODING CATEGORIES	SKIP
701	CHECK 304:<br>NEITHER STERILIZED ☐ ↓ HE OR SHE STERILIZED ☐		→ 712
702	CHECK 226:<br>PREGNANT ☐ ↓ NOT PREGNANT OR UNSURE ☐		→ 704
703	Now I have some questions about the future. After the child you are expecting now, would you like to have another child, or would you prefer not to have any more children?	HAVE ANOTHER CHILD . . . . 1<br>NO MORE . . . . 2<br>UNDECIDED/DON'T KNOW 8	→ 705<br><br>→ 711
704	Now I have some questions about the future. Would you like to have (a/another) child, or would you prefer not to have any (more) children?	HAVE (A/ANOTHER) CHILD . . . . 1<br>NO MORE/NONE . . . . 2<br>SAYS SHE CAN'T GET PREGNANT . 3<br>UNDECIDED/DON'T KNOW 8	 → 707<br>→ 712<br>→ 710
705	CHECK 226:<br>NOT PREGNANT OR UNSURE ☐ ↓<br>How long would you like to wait from now before the birth of (a/another) child?<br>PREGNANT ☐ ↓<br>After the birth of the child you are expecting now, how long would you like to wait before the birth of another child?	MONTHS . . . . 1 ☐☐<br>YEARS . . . . 2 ☐☐<br>SOON/NOW . . . . 993<br>SAYS SHE CAN'T GET PREGNANT 994<br>AFTER MARRIAGE . . . . 995<br>OTHER ______________________ 996<br>(SPECIFY)<br>DON'T KNOW 998	 <br>→ 710<br>→ 712<br><br>→ 710
706	CHECK 226:<br>NOT PREGNANT OR UNSURE ☐ ↓ PREGNANT ☐		→ 711
707	CHECK 303: USING A CONTRACEPTIVE METHOD?<br>NOT CURRENTLY USING ☐ ↓ CURRENTLY USING ☐		→ 712
708	CHECK 705:<br>NOT ASKED ☐ ↓ 24 OR MORE MONTHS OR 02 OR MORE YEARS ☐ ↓ 00-23 MONTHS OR < 02 YEARS ☐		→ 711

NO.	QUESTIONS AND FILTERS	CODING CATEGORIES	SKIP
709	CHECK 703 AND 704:<br><br>WANTS TO HAVE A/ANOTHER CHILD ☐ ↓<br>You have said that you do not want (a/another) child soon.<br>Can you tell me why you are not using a method to prevent pregnancy?<br>Any other reason?<br><br>WANTS NO MORE/NONE ☐ ↓<br>You have said that you do not want any (more) children.<br>Can you tell me why you are not using a method to prevent pregnancy?<br>Any other reason?<br><br>RECORD ALL REASONS MENTIONED.	NOT MARRIED . . . . A<br><br>FERTILITY-RELATED REASONS<br>NOT HAVING SEX . . . . B<br>INFREQUENT SEX . . . . C<br>MENOPAUSAL/HYSTERECTOMY D<br>CAN'T GET PREGNANT . . . . E<br>NOT MENSTRUATED SINCE LAST BIRTH . . . . F<br>BREASTFEEDING . . . . G<br>UP TO GOD/FATALISTIC . . . . H<br><br>OPPOSITION TO USE<br>RESPONDENT OPPOSED . . . . I<br>HUSBAND/PARTNER OPPOSED . . . J<br>OTHERS OPPOSED . . . . K<br>RELIGIOUS PROHIBITION . . . . L<br><br>LACK OF KNOWLEDGE<br>KNOWS NO METHOD . . . . M<br>KNOWS NO SOURCE . . . . N<br><br>METHOD-RELATED REASONS<br>SIDE EFFECTS/HEALTH CONCERNS . . . . O<br>LACK OF ACCESS/TOO FAR . . . . . P<br>COSTS TOO MUCH . . . . Q<br>PREFERRED METHOD NOT AVAILABLE . . . . R<br>NO METHOD AVAILABLE . . . . S<br>INCONVENIENT TO USE . . . . T<br>INTERFERES WITH BODY'S NORMAL PROCESSES . . . . U<br><br>OTHER ______________________ X<br>(SPECIFY)<br>DON'T KNOW . . . . Z	
710	CHECK 303: USING A CONTRACEPTIVE METHOD?<br>NOT ASKED ☐ ↓ NO, NOT CURRENTLY USING ☐ ↓ YES, CURRENTLY USING ☐		→ 712
711	Do you think you will use a contraceptive method to delay or avoid pregnancy at any time in the future?	YES . . . . 1<br>NO . . . . 2<br>DON'T KNOW 8	
712	CHECK 216:<br><br>HAS LIVING CHILDREN ☐ ↓<br>If you could go back to the time you did not have any children and could choose exactly the number of children to have in your whole life, how many would that be?<br><br>NO LIVING CHILDREN ☐ ↓<br>If you could choose exactly the number of children to have in your whole life, how many would that be?<br><br>PROBE FOR A NUMERIC RESPONSE.	NONE . . . . 00<br><br>NUMBER . . . . ☐☐<br><br>OTHER ______________________ 96<br>(SPECIFY)	→ 714<br><br><br><br>→ 714

NO.	QUESTIONS AND FILTERS	CODING CATEGORIES	SKIP
713	How many of these children would you like to be boys, how many would you like to be girls and for how many would it not matter if it's a boy or a girl?	BOYS GIRLS EITHER<br>NUMBER [ ][ ] [ ][ ] [ ][ ]<br>OTHER ______________ 96<br>(SPECIFY)	
714	In the last few months have you:<br>Heard about family planning on the radio?<br>Seen anything about family planning on the television?<br>Read about family planning in a newspaper or magazine?<br>Through family or friends?<br>From community council?<br>Billboards, posters, or leaflets?<br>Local campaign for family planing?	YES NO<br>RADIO . . . . 1 2<br>TELEVISION . . . . 1 2<br>NEWSPAPER OR MAGAZINE . . . 1 2<br>FAMILY/FRIENDS . . . . 1 2<br>COMMUNITY COUNCIL . . . . 1 2<br>BILLBOARDS/POSTERS . . . . . 1 2<br>LOCAL CAMPAIGN 1 2	
716	CHECK 601:<br>YES, CURRENTLY MARRIED [ ] ↓ YES, LIVING WITH A MAN [ ] ↓ NO, NOT IN UNION [ ] →		801
717	CHECK 303: USING A CONTRACEPTIVE METHOD?<br>CURRENTLY USING [ ] ↓ NOT CURRENTLY USING OR NOT ASKED [ ] →		720
718	Would you say that using contraception is mainly your decision, mainly your (husband's/partner's) decision, or did you both decide together?	MAINLY RESPONDENT . . . . 1<br>MAINLY HUSBAND/PARTNER . . . . . 2<br>JOINT DECISION . . . . 3<br>OTHER ______________ 6<br>(SPECIFY)	
719	CHECK 304:<br>NEITHER STERILIZED [ ] ↓ HE OR SHE STERILIZED [ ] →		801
720	Does your (husband/partner) want the same number of children that you want, or does he want more or fewer than you want?	SAME NUMBER . . . . 1<br>MORE CHILDREN . . . . 2<br>FEWER CHILDREN . . . . 3<br>DON'T KNOW 8	

SECTION 8. HUSBAND'S BACKGROUND AND WOMAN'S WORK

NO.	QUESTIONS AND FILTERS	CODING CATEGORIES	SKIP
801	CHECK 601 AND 602:<br>CURRENTLY MARRIED/ LIVING WITH A MAN [ ]↓<br>FORMERLY MARRIED/ LIVED WITH A MAN [ ] → 803<br>NEVER MARRIED AND NEVER LIVED WITH A MAN [ ] → 807		803<br>807
802	How old was your (husband/partner) on his last birthday?	AGE IN COMPLETED YEARS [ ][ ]	
803	Did your (last) (husband/partner) ever attend school?	YES . . . 1<br>NO . . . 2	 → 806
804	What was the highest level of school he attended: primary, lower secondary, or higher?	PRIMARY . . . 1<br>LOWER SECONDARY . . . 2<br>UPPER SECONDARY . . . 3<br>HIGHER . . . 4<br>DON'T KNOW . . . 8	 <br><br><br>→ 806
805	What was the highest (grade/form/year) he completed at that level?<br>IF COMPLETED LESS THAN ONE YEAR AT THAT LEVEL, RECORD '00'.	GRADE . . . [ ][ ]<br>DON'T KNOW . . . 98	
806	CHECK 801:<br>CURRENTLY MARRIED/ LIVING WITH A MAN [ ]↓<br>What is your (husband's/ partner's) occupation? That is, what kind of work does he mainly do?<br>FORMERLY MARRIED/ LIVED WITH A MAN [ ]↓<br>What was your (last) (husband's/ partner's) occupation? That is, what kind of work did he mainly do?	____________ [ ][ ]<br>____________<br>____________	
807	Aside from your own housework, have you done any work in the last seven days?	YES . . . 1<br>NO . . . 2	→ 811
808	As you know, some women take up jobs for which they are paid in cash or kind. Others sell things, have a small business or work on the family farm or in the family business.<br>In the last seven days, have you done any of these things or any other work?	YES . . . 1<br>NO . . . 2	→ 811
809	Although you did not work in the last seven days, do you have any job or business from which you were absent for leave, illness, vacation, maternity leave, or any other such reason?	YES . . . 1<br>NO . . . 2	→ 811
810	Have you done any work in the last 12 months?	YES . . . 1<br>NO . . . 2	 → 815
811	What is your occupation, that is, what kind of work do you mainly do?	____________ [ ][ ]<br>____________<br>____________	
812	Do you do this work for a member of your family, for someone else, or are you self-employed?	FOR FAMILY MEMBER . . . 1<br>FOR SOMEONE ELSE . . . 2<br>SELF-EMPLOYED . . . 3	

NO.	QUESTIONS AND FILTERS	CODING CATEGORIES	SKIP
813	Do you usually work throughout the year, or do you work seasonally, or only once in a while?	THROUGHOUT THE YEAR . . . . 1<br>SEASONALLY/PART OF THE YEAR 2<br>ONCE IN A WHILE 3	
814	Are you paid in cash or kind for this work or are you not paid at all?	CASH ONLY . . . . 1<br>CASH AND KIND . . . . 2<br>IN KIND ONLY . . . . 3<br>NOT PAID 4	
815	CHECK 601:<br>CURRENTLY MARRIED/LIVING WITH A MAN ☐ ↓<br>NOT IN UNION ☐ → 823		823
816	CHECK 814:<br>CODE 1 OR 2 CIRCLED ☐ ↓<br>OTHER ☐ → 819		819
817	Who usually decides how the money you earn will be used: you, your (husband/partner), or you and your (husband/partner) jointly?	RESPONDENT . . . . 1<br>HUSBAND/PARTNER . . . . 2<br>RESPONDENT AND<br>HUSBAND/PARTNER JOINTLY . . . 3<br>OTHER ______________ 6<br>(SPECIFY)	
817A	Have you ever given up/refused a job for money because your husband/partner did not want you to work?	YES . . . . 1<br>NO . . . . 2<br>DON'T KNOW 8	
817B	Has your husband/partner's ever disrupted your work or other income-generating activities?	NO . . . . 1<br>INTERRUPTED . . . . 2<br>DISTURBED . . . . 3<br>CANNOT WORK/STOP WORKING . . . 4<br>LOST CONFIDENCE . . . . 5<br>DON'T KNOW 8	
818	Would you say that the money that you earn is more than what your (husband/partner) earns, less than what he earns, or about the same?	MORE THAN HIM . . . . 1<br>LESS THAN HIM . . . . 2<br>ABOUT THE SAME . . . . 3<br>HUSBAND/PARTNER DOESN'T<br>BRING IN ANY MONEY . . . . 4<br>DON'T KNOW 8	 <br><br><br>→ 820
819	Who usually decides how your (husband's/partner's) earnings will be used: you, your (husband/partner), or you and your (husband/partner) jointly?	RESPONDENT . . . . 1<br>HUSBAND/PARTNER . . . . 2<br>RESPONDENT AND<br>HUSBAND/PARTNER JOINTLY . . . 3<br>HUSBAND/PARTNER HAS<br>NO EARNINGS . . . . 4<br>OTHER ______________ 6<br>(SPECIFY)	
820	Who usually makes decisions about health care for yourself: you, your (husband/partner), you and your (husband/partner) jointly, or someone else?	RESPONDENT . . . . 1<br>HUSBAND/PARTNER . . . . 2<br>RESPONDENT AND<br>HUSBAND/PARTNER JOINTLY . . . 3<br>SOMEONE ELSE . . . . 4<br>OTHER 6	

NO.	QUESTIONS AND FILTERS	CODING CATEGORIES	SKIP
821	Who usually makes decisions about making major household purchases?	RESPONDENT . . . . 1<br>HUSBAND/PARTNER . . . . 2<br>RESPONDENT AND<br>HUSBAND/PARTNER JOINTLY . . . 3<br>SOMEONE ELSE . . . . 4<br>OTHER 6	
822	Who usually makes decisions about visits to your family or relatives?	RESPONDENT . . . . 1<br>HUSBAND/PARTNER . . . . 2<br>SOMEONE ELSE<br>HUSBAND/PARTNER JOINTLY . . . 3<br>SOMEONE ELSE . . . . 4<br>OTHER 6	
823	Do you own this or any other house either alone or jointly with someone else?	ALONE ONLY . . . . 1<br>JOINTLY ONLY . . . . 2<br>BOTH ALONE AND JOINTLY . . . . 3<br>DOES NOT OWN 4	
824	Do you own any land either alone or jointly with someone else?	ALONE ONLY . . . . 1<br>JOINTLY ONLY . . . . 2<br>BOTH ALONE AND JOINTLY . . . . 3<br>DOES NOT OWN 4	

NO.	QUESTIONS AND FILTERS		PRES./ LISTEN.	PRES./ NOT LISTEN.	NOT PRES.	SKIP
825	PRESENCE OF OTHERS AT THIS POINT (PRESENT AND LISTENING, PRESENT BUT NOT LISTENING, OR NOT PRESENT)	CHILDREN < 10	1	2	3	
		HUSBAND	1	2	3	
		OTHER MALES	1	2	3	
		OTHER FEMALES . . .	1	2	3	

NO.	QUESTIONS AND FILTERS		YES	NO	DK	SKIP
826	In your opinion, is a husband justified in hitting or beating his wife in the following situations:					
	If she goes out without telling him?	GOES OUT	1	2	8	
	If she neglects the children?	NEGL. CHILDREN . . .	1	2	8	
	If she argues with him?	ARGUES	1	2	8	
	If she refuses to have sex with him?	REFUSES SEX	1	2	8	
	If she burns the food?	BURNS FOOD	1	2	8	
	If she ask him to use condom?	ASK USE CONDOM .	1	2	8	
827	In your opinion, is a parent justified in hitting or beating his son for the following reasons:					
	If he disobeys?	DISOBEY	1	2	8	
	If he impolite?	IMPOLITE	1	2	8	
	If he has embarrassed the family?	EMBARR. FAMILY . . .	1	2	8	
	If he does not do the housework or cooking?	HOUSEWORK . . .	1	2	8	
	If he does not take care of younger sibling ?	CARE SIBLING . . .	1	2	8	
828	In your opinion, is a parent justified in hitting or beating his daughter for the following reasons:					
	If she disobeys?	DISOBEY	1	2	8	
	If she impolite?	IMPOLITE	1	2	8	
	If she has embarrassed the family?	EMBARR. FAMILY . . .	1	2	8	
	If she does not do the housework or cooking?	HOUSEWORK . . .	1	2	8	
	If she does not take care of younger sibling ?	CARE SIBLING . . .	1	2	8	

SECTION 9. HIV/AIDS

NO.	QUESTIONS AND FILTERS	CODING CATEGORIES	SKIP
901	Now I would like to talk about something else. Have you ever heard of an illness called AIDS?	YES . . . 1<br>NO . . . 2	 → 937
902	Can people reduce their chance of getting the AIDS virus by having just one uninfected sex partner who has no other sex partners?	YES . . . 1<br>NO . . . 2<br>DON'T KNOW . . . 8	
903	Can people get the AIDS virus from mosquito bites?	YES . . . 1<br>NO . . . 2<br>DON'T KNOW . . . 8	
904	Can people reduce their chance of getting the AIDS virus by using a condom every time they have sex?	YES . . . 1<br>NO . . . 2<br>DON'T KNOW . . . 8	
905	Can people get the AIDS virus by sharing food with a person who has AIDS?	YES . . . 1<br>NO . . . 2<br>DON'T KNOW . . . 8	
906	Can people get the AIDS virus because of witchcraft or other supernatural means?	YES . . . 1<br>NO . . . 2<br>DON'T KNOW . . . 8	
907	Is it possible for a healthy-looking person to have the AIDS virus?	YES . . . 1<br>NO . . . 2<br>DON'T KNOW . . . 8	
908	Can the virus that causes AIDS be transmitted from a mother to her baby:<br>During pregnancy?<br>During delivery?<br>By breastfeeding?	YES NO DK<br>DURING PREG. . . . . . 1 2 8<br>DURING DELIVERY . . . 1 2 8<br>BREASTFEEDING . . . 1 2 8	
909	CHECK 908:<br>AT LEAST ONE 'YES' ☐ ↓ OTHER ☐		→ 911
910	Are there any special drugs that a doctor or a nurse can give to a woman infected with the AIDS virus to reduce the risk of transmission to the baby?	YES . . . 1<br>NO . . . 2<br>DON'T KNOW . . . 8	
911	CHECK 208 AND 215:<br>NO BIRTHS ☐<br>LAST BIRTH SINCE JANUARY 2012 ☐ ↓ LAST BIRTH BEFORE JANUARY 2012 ☐		→ 926<br>→ 926
912	CHECK 408 FOR LAST BIRTH:<br>HAD ANTENATAL CARE ☐ ↓ NO ANTENATAL CARE ☐		→ 920
913	CHECK FOR PRESENCE OF OTHERS. BEFORE CONTINUING, MAKE EVERY EFFORT TO ENSURE PRIVACY.		
914	During any of the antenatal visits for your last birth were you given any information about:<br>Babies getting the AIDS virus from their mother?<br>Things that you can do to prevent getting the AIDS virus?<br>Getting tested for the AIDS virus?	YES NO DK<br>AIDS FROM MOTHER 1 2 8<br>THINGS TO DO 1 2 8<br>TESTED FOR AIDS 1 2 8	

NO.	QUESTIONS AND FILTERS	CODING CATEGORIES	SKIP
915	Were you offered a test for the AIDS virus as part of your antenatal care?	YES . . . . . 1<br>NO 2	
916	I don't want to know the results, but were you tested for the AIDS virus as part of your antenatal care?	YES . . . . . 1<br>NO 2	 2 → 920
917	Where was the test done?<br><br>PROBE TO IDENTIFY THE TYPE OF SOURCE AND CIRCLE THE APPROPRIATE CODE.<br><br>IF UNABLE TO DETERMINE IF PUBLIC OR PRIVATE SECTOR, WRITE THE NAME OF THE PLACE.<br><br>__________<br>(NAME OF PLACE)	PUBLIC SECTOR<br>NATIONAL HOSPITAL (PP) . . . . . 11<br>PROVINCIAL HOSP (RH) . . . . . 12<br>DISTRICT HOSPITAL (RH) . . . 13<br>HEALTH CENTER . . . . . 14<br>HEALTH POST . . . . . 15<br>OUTREACH . . . . . 16<br>MILITARY HOSPITAL . . . . . 17<br>VCCT CENTER . . . . . 18<br>PMTCT SITE . . . . . 19<br>OTHER PUBLIC SECTOR _____ 20<br>(SPECIFY)<br>PRIVATE MEDICAL SECTOR<br>PRIVATE HOSPITAL . . . . . 21<br>PRIVATE CLINIC . . . . . 22<br>PRIVATE LABORATORY . . . . . 23<br>OTHER PRIVATE MEDICAL SECTOR<br>_____ 24<br>(SPECIFY)<br><br>OTHER _____ 96<br>(SPECIFY)	
918	I don't want to know the results, but did you get the results of the test?	YES . . . . . 1<br>NO 2	 2 → 924
919	All women are supposed to receive counseling after being tested. After you were tested, did you receive counseling?	YES . . . . . 1<br>NO . . . . . 2<br>DON'T KNOW 8	1, 2, 8 → 924
920	CHECK 434 FOR LAST BIRTH:<br>ANY CODE 21-33 CIRCLED ☐ ↓ OTHER ☐		OTHER → 926
921	Between the time you went for delivery but before the baby was born, were you offered a test for the AIDS virus?	YES . . . . . 1<br>NO 2	
922	I don't want to know the results, but were you tested for the AIDS virus at that time?	YES . . . . . 1<br>NO 2	 2 → 926
923	I don't want to know the results, but did you get the results of the test?	YES . . . . . 1<br>NO 2	
924	Have you been tested for the AIDS virus since that time you were tested during your pregnancy?	YES . . . . . 1<br>NO 2	1 → 927
925	How many months ago was your most recent HIV test?	MONTHS AGO . . . . . ☐☐<br><br>TWO OR MORE YEARS 96	→ 932

NO.	QUESTIONS AND FILTERS	CODING CATEGORIES	SKIP
926	I don't want to know the results, but have you ever been tested to see if you have the AIDS virus?	YES . . . . . 1<br>NO 2	 → 930
927	How many months ago was your most recent HIV test?	MONTHS AGO . . . . . [ ][ ]<br><br>TWO OR MORE YEARS 96	
928	I don't want to know the results, but did you get the results of the test?	YES . . . . . 1<br>NO 2	
929	Where was the test done?<br><br>PROBE TO IDENTIFY THE TYPE OF SOURCE.<br><br>IF UNABLE TO DETERMINE IF PUBLIC OR PRIVATE SECTOR, WRITE THE NAME OF THE PLACE.<br><br>____________________<br>(NAME OF PLACE)	PUBLIC SECTOR<br>NATIONAL HOSPITAL (PP) . . . . . 11<br>PROVINCIAL HOSP (RH) . . . . . 12<br>DISTRICT HOSPITAL (RH) . . . 13<br>HEALTH CENTER . . . . . 14<br>HEALTH POST . . . . . 15<br>OUTREACH . . . . . 16<br>MILITARY HOSPITAL . . . . . 17<br>VCCT CENTER . . . . . 18<br>PMTCT SITE . . . . . 19<br>OTHER PUBLIC<br>SECTOR __________ 20<br>(SPECIFY)<br>PRIVATE MEDICAL SECTOR<br>PRIVATE HOSPITAL . . . . . 21<br>PRIVATE CLINIC . . . . . 22<br>PRIVATE LABORATORY . . . . . 23<br>OTHER PRIVATE<br>MEDICAL SECTOR<br>__________ 24<br>(SPECIFY)<br><br>OTHER __________ 96<br>(SPECIFY)	→ 932
930	Do you know of a place where people can go to get tested for the AIDS virus?	YES . . . . . 1<br>NO 2	 → 932

NO.	QUESTIONS AND FILTERS	CODING CATEGORIES	SKIP
931	Where is that?<br><br>Any other place?<br><br>PROBE TO IDENTIFY EACH TYPE OF SOURCE.<br><br>IF UNABLE TO DETERMINE IF PUBLIC OR PRIVATE SECTOR, WRITE THE NAME OF THE PLACE.<br><br>_______________<br>(NAME OF PLACE(S))	PUBLIC SECTOR<br>NATIONAL HOSPITAL (PP) . . . . A<br>PROVINCIAL HOSP (RH) . . . . B<br>DISTRICT HOSPITAL (RH) . . . C<br>HEALTH CENTER . . . . D<br>HEALTH POST . . . . E<br>OUTREACH . . . . F<br>MILITARY HOSPITAL . . . . G<br>VCCT CENTER . . . . H<br>PMTCT SITE . . . . I<br>OTHER PUBLIC<br>SECTOR _______________ J<br>(SPECIFY)<br>PRIVATE MEDICAL SECTOR<br>PRIVATE HOSPITAL . . . . K<br>PRIVATE CLINIC . . . . L<br>PRIVATE LABORATORY . . . . . M<br>OTHER PRIVATE<br>MEDICAL SECTOR<br>_______________ N<br>(SPECIFY)<br><br>OTHER _______________ X<br>(SPECIFY)	
932	Would you buy fresh vegetables from a shopkeeper or vendor if you knew that this person had the AIDS virus?	YES . . . . 1<br>NO . . . . 2<br>DON'T KNOW 8	
933	If a member of your family got infected with the AIDS virus, would you want it to remain a secret or not?	YES, REMAIN A SECRET . . . . 1<br>NO . . . . 2<br>DK/NOT SURE/DEPENDS 8	
934	If a member of your family became sick with AIDS, would you be willing to care for her or him in your own household?	YES . . . . 1<br>NO . . . . 2<br>DK/NOT SURE/DEPENDS 8	
935	In your opinion, if a female teacher has the AIDS virus but is not sick, should she be allowed to continue teaching in the school?	SHOULD BE ALLOWED . . . . 1<br>SHOULD NOT BE ALLOWED . . . . 2<br>DK/NOT SURE/DEPENDS 8	
936	Do you personally know someone who has been denied health services in the last 12 months because he or she is suspected to have the AIDS virus or has the AIDS virus?	YES . . . . 1<br>NO . . . . 2<br>DK ANYONE WITH AIDS 8	
936A	Do you agree or disagree with the following statement: People with the AIDS virus should be ashamed of themselves.	AGREE . . . . 1<br>DISAGREE . . . . 2<br>DON'T KNOW/NO OPINION 8	
936B	Do you agree or disagree with the following statement: People with the AIDS virus should be blamed for bringing the disease into the community?	AGREE . . . . 1<br>DISAGREE . . . . 2<br>DON'T KNOW/NO OPINION 8	
937	CHECK 901:<br>HEARD ABOUT AIDS ☐ ↓<br>Apart from AIDS, have you heard about other infections that can be transmitted through sexual contact?<br><br>NOT HEARD ABOUT AIDS ☐ ↓<br>Have you heard about infections that can be transmitted through sexual contact?	YES . . . . 1<br>NO 2	

NO.	QUESTIONS AND FILTERS	CODING CATEGORIES	SKIP
938	CHECK 613:<br>HAS HAD SEXUAL INTERCOURSE ☐ ↓<br>NEVER HAD SEXUAL INTERCOURSE ☐ → 946		946
939	CHECK 937: HEARD ABOUT OTHER SEXUALLY TRANSMITTED INFECTIONS?<br>YES ☐ ↓<br>NO ☐ → 941		941
940	Now I would like to ask you some questions about your health in the last 12 months. During the last 12 months, have you had a disease which you got through sexual contact?	YES . . . . 1<br>NO . . . . 2<br>DON'T KNOW 8	
941	Sometimes women experience a bad-smelling abnormal genital discharge.<br>During the last 12 months, have you had a bad smelling abnormal genital discharge?	YES . . . . 1<br>NO . . . . 2<br>DON'T KNOW 8	
942	Sometimes women have a genital sore or ulcer. During the last 12 months, have you had a genital sore or ulcer?	YES . . . . 1<br>NO . . . . 2<br>DON'T KNOW 8	
943	CHECK 940, 941, AND 942:<br>HAS HAD AN INFECTION (ANY 'YES') ☐ ↓<br>HAS NOT HAD AN INFECTION OR DOES NOT KNOW ☐ → 946		946
944	The last time you had (PROBLEM FROM 940/941/942), did you seek any kind of advice or treatment?	YES . . . . 1<br>NO 2	 → 946
945	Where did you go?<br><br>Any other place?<br><br>PROBE TO IDENTIFY EACH TYPE OF SOURCE.<br><br>IF UNABLE TO DETERMINE IF PUBLIC OR PRIVATE SECTOR, WRITE THE NAME OF THE PLACE.<br><br>________________<br>(NAME OF PLACE(S))	PUBLIC SECTOR<br>NATIONAL HOSPITAL (PP) . . . . A<br>PROVINCIAL HOSP (RH) . . . . B<br>DISTRICT HOSPITAL (RH) . . . C<br>HEALTH CENTER . . . . D<br>HEALTH POST . . . . E<br>FIELDWORKER . . . . F<br>OUTREACH . . . . G<br>MILITARY HOSPITAL . . . . H<br>VCCT CENTER . . . . I<br>PMTCT SITE . . . . J<br>OTHER PUBLIC ________ K<br>(SPECIFY)<br><br>PRIVATE MEDICAL SECTOR<br>PRIVATE HOSPITAL . . . . L<br>PRIVATE CLINIC . . . . M<br>PRIVATE LABORATORY . . . . N<br>OTHER PRIVATE MEDICAL SECTOR ________ O<br>(SPECIFY)<br><br>OTHER SOURCE<br>OTHER ________ X<br>(SPECIFY)	
946	If a wife knows her husband has a disease that she can get during sexual intercourse, is she justified in asking that they use a condom when they have sex?	YES . . . . 1<br>NO . . . . 2<br>DON'T KNOW 8	
947	Is a wife justified in refusing to have sex with her husband when she knows he has sex with other women?	YES . . . . 1<br>NO . . . . 2<br>DON'T KNOW 8	

NO.	QUESTIONS AND FILTERS	CODING CATEGORIES	SKIP
948	CHECK 601:<br>CURRENTLY MARRIED/ LIVING WITH A MAN ☐ ↓<br>NOT IN UNION ☐		→ 1001
949	Can you say no to your (husband/partner) if you do not want to have sexual intercourse?	YES . . . . 1<br>NO . . . . 2<br>DEPENDS/NOT SURE 8	
950	Could you ask your (husband/partner) to use a condom if you wanted him to?	YES . . . . 1<br>NO . . . . 2<br>DEPENDS/NOT SURE 8	

SECTION 10. OTHER HEALTH ISSUES

NO.	QUESTIONS AND FILTERS	CODING CATEGORIES	SKIP
1001	Now I would like to ask you some other questions relating to health matters. Have you had an injection for any reason in the last 12 months?<br>IF YES: How many injections have you had?<br>IF NUMBER OF INJECTIONS IS 90 OR MORE, OR DAILY FOR 3 MONTHS OR MORE, RECORD '90'.<br>IF NON-NUMERIC ANSWER, PROBE TO GET AN ESTIMATE.	NUMBER OF INJECTIONS . . . [ ][ ]<br>NONE 00	 → 1004
1002	Among these injections, how many were administered by a doctor, a nurse, a pharmacist, a dentist, or any other health worker?<br>IF NUMBER OF INJECTIONS IS 90 OR MORE, OR DAILY FOR 3 MONTHS OR MORE, RECORD '90'.<br>IF NON-NUMERIC ANSWER, PROBE TO GET AN ESTIMATE.	NUMBER OF INJECTIONS . . . [ ][ ]<br>NONE 00	 → 1004
1003	The last time you got an injection from a health worker, did he/she take the syringe and needle from a new, unopened package?	YES . . . . 1<br>NO . . . . 2<br>DON'T KNOW 8	
1004	Do you currently smoke cigarettes?	YES . . . . 1<br>NO 2	 → 1006
1005	In the last 24 hours, how many cigarettes did you smoke?	NUMBER OF CIGARETTES . . . . [ ][ ]	
1006	Do you currently smoke or use any (other) type of tobacco?	YES . . . . 1<br>NO 2	 → 1008
1007	What (other) type of tobacco do you currently smoke or use?<br>RECORD ALL MENTIONED.	PIPE . . . . A<br>CHEWING TOBACCO . . . . B<br>SNUFF . . . . C<br>OTHER ______________________ X<br>(SPECIFY)	
1008	Many different factors can prevent women from getting medical advice or treatment for themselves. When you are sick and want to get medical advice or treatment, is each of the following a big problem or not?<br>Getting permission to go to the doctor?<br>Getting money needed for advice or treatment?<br>The distance to the health facility?<br>Not wanting to go alone?	BIG PROB-LEM / NOT A BIG PROB-LEM<br>PERMISSION TO GO . . . 1 2<br>GETTING MONEY . . . . . 1 2<br>DISTANCE . . . . 1 2<br>GO ALONE 1 2	
1009	Are you covered by any health insurance?	YES . . . . 1<br>NO 2	 → 1101

NO.	QUESTIONS AND FILTERS	CODING CATEGORIES	SKIP
1010	What type of health insurance are you covered by?<br><br>RECORD ALL MENTIONED.	HEALTH EQUITY FUND . . . . A<br>MATERNAL HEALTH VOUCHER . . . . . B<br>COMMUNITY-BASED HEALTH<br>INSURANCE . . . . C<br>HEALTH INSURANCE THROUGH<br>EMPLOYER . . . . D<br>OTHER PRIVATELY PURCHASED<br>COMMERCIAL HEALTH INSURANCE E<br>OTHER ______________________ X<br>(SPECIFY)	

SECTION 11. MATERNAL MORTALITY

NO.	QUESTIONS AND FILTERS	CODING CATEGORIES	SKIP
1101	Now I would like to ask you some questions about your brothers and sisters, that is, all of the children born to your natural mother, including those who are living with you, those living elsewhere and those who have died. How many children did your mother give birth to, including you?	NUMBER OF BIRTHS TO NATURAL MOTHER . . . . [ ][ ]	
1102	CHECK 1101: TWO OR MORE BIRTHS [ ] ↓ ONLY ONE BIRTH (RESPONDENT ONLY) [ ]		→ 1114
1103	How many of these births did your mother have before you were born?	NUMBER OF PRECEDING BIRTHS . . . . [ ][ ]	

NO.	QUESTION	(1)	(2)	(3)	(4)	(5)	(6)
1104	What was the name given to your oldest (next oldest) brother or sister?	______	______	______	______	______	______
1105	Is (NAME) male or female?	MALE 1<br>FEMALE 2	MALE 1<br>FEMALE 2	MALE 1<br>FEMALE 2	MALE 1<br>FEMALE 2	MALE 1<br>FEMALE 2	MALE 1<br>FEMALE 2
1106	Is (NAME) still alive?	YES . . . 1<br>NO . . . 2<br>GO TO 1108<br>DK . . . 8<br>GO TO (2)	YES . . . 1<br>NO . . . 2<br>GO TO 1108<br>DK . . . 8<br>GO TO (3)	YES . . . 1<br>NO . . . 2<br>GO TO 1108<br>DK . . . 8<br>GO TO (4)	YES . . . 1<br>NO . . . 2<br>GO TO 1108<br>DK . . . 8<br>GO TO (5)	YES . . . 1<br>NO . . . 2<br>GO TO 1108<br>DK . . . 8<br>GO TO (6)	YES . . . 1<br>NO . . . 2<br>GO TO 1108<br>DK . . . 8<br>GO TO (7)
1107	How old is (NAME)?	[ ][ ]<br>GO TO (2)	[ ][ ]<br>GO TO (3)	[ ][ ]<br>GO TO (4)	[ ][ ]<br>GO TO (5)	[ ][ ]<br>GO TO (6)	[ ][ ]<br>GO TO (7)
1108	How many years ago did (NAME) die?	[ ][ ]	[ ][ ]	[ ][ ]	[ ][ ]	[ ][ ]	[ ][ ]
1109	How old was (NAME) when he/she died?	[ ][ ]<br>IF MALE OR DIED BEFORE 12 YEARS OF AGE GO TO (2)	[ ][ ]<br>IF MALE OR DIED BEFORE 12 YEARS OF AGE GO TO (3)	[ ][ ]<br>IF MALE OR DIED BEFORE 12 YEARS OF AGE GO TO (4)	[ ][ ]<br>IF MALE OR DIED BEFORE 12 YEARS OF AGE GO TO (5)	[ ][ ]<br>IF MALE OR DIED BEFORE 12 YEARS OF AGE GO TO (6)	[ ][ ]<br>IF MALE OR DIED BEFORE 12 YEARS OF AGE GO TO (7)
1110	Was (NAME) pregnant when she died?	YES . . . 1<br>GO TO 1112A<br>NO . . . 2	YES . . . 1<br>GO TO 1112A<br>NO . . . 2	YES . . . 1<br>GO TO 1112A<br>NO . . . 2	YES . . . 1<br>GO TO 1112A<br>NO . . . 2	YES . . . 1<br>GO TO 1112A<br>NO . . . 2	YES . . . 1<br>GO TO 1112A<br>NO . . . 2
1111	Did (NAME) die during childbirth?	YES . . . 1<br>GO TO 1113<br>NO . . . 2	YES . . . 1<br>GO TO 1113<br>NO . . . 2	YES . . . 1<br>GO TO 1113<br>NO . . . 2	YES . . . 1<br>GO TO 1113<br>NO . . . 2	YES . . . 1<br>GO TO 1113<br>NO . . . 2	YES . . . 1<br>GO TO 1113<br>NO . . . 2
1112	Did (NAME) die within six weeks after the end of a pregnancy or childbirth?	YES . . . 1<br>NO . . . 2<br>GO TO 1113	YES . . . 1<br>NO . . . 2<br>GO TO 1113	YES . . . 1<br>NO . . . 2<br>GO TO 1113	YES . . . 1<br>NO . . . 2<br>GO TO 1113	YES . . . 1<br>NO . . . 2<br>GO TO 1113	YES . . . 1<br>NO . . . 2<br>GO TO 1113
1112A	Was the death of (NAME) related to accident such as traffic accident?	YES . . . 1<br>NO . . . 2	YES . . . 1<br>NO . . . 2	YES . . . 1<br>NO . . . 2	YES . . . 1<br>NO . . . 2	YES . . . 1<br>NO . . . 2	YES . . . 1<br>NO . . . 2
1113	How many live born children did (NAME) give birth to during her lifetime (before this pregnancy)?	[ ][ ]	[ ][ ]	[ ][ ]	[ ][ ]	[ ][ ]	[ ][ ]

GO BACK TO 1004 IN NEXT COLUMN, OR, IF NO MORE BROTHERS OR SISTERS, END.

1104	What was the name given to your oldest (next oldest) brother or sister?	(7)	(8)	(9)	(10)	(11)	(12)
1105	Is (NAME) male or female?	MALE 1<br>FEMALE 2	MALE 1<br>FEMALE 2	MALE 1<br>FEMALE 2	MALE 1<br>FEMALE 2	MALE 1<br>FEMALE 2	MALE 1<br>FEMALE 2
1106	Is (NAME) still alive?	YES . . . 1<br>NO . . . 2<br>GO TO 1108<br>DK . . . 8<br>GO TO (8)	YES . . . 1<br>NO . . . 2<br>GO TO 1108<br>DK . . . 8<br>GO TO (9)	YES . . . 1<br>NO . . . 2<br>GO TO 1108<br>DK . . . 8<br>GO TO (10)	YES . . . 1<br>NO . . . 2<br>GO TO 1108<br>DK . . . 8<br>GO TO (11)	YES . . . 1<br>NO . . . 2<br>GO TO 1108<br>DK . . . 8<br>GO TO (12)	YES . . . 1<br>NO . . . 2<br>GO TO 1108<br>DK . . . 8<br>GO TO (13)
1107	How old is (NAME)?	GO TO (8)	GO TO (9)	GO TO (10)	GO TO (11)	GO TO (12)	GO TO (13)
1108	How many years ago did (NAME) die?						
1109	How old was (NAME) when he/she died?	IF MALE OR DIED BEFORE 12 YEARS OF AGE GO TO (8)	IF MALE OR DIED BEFORE 12 YEARS OF AGE GO TO (9)	IF MALE OR DIED BEFORE 12 YEARS OF AGE GO TO (10)	IF MALE OR DIED BEFORE 12 YEARS OF AGE GO TO (11)	IF MALE OR DIED BEFORE 12 YEARS OF AGE GO TO (12)	IF MALE OR DIED BEFORE 12 YEARS OF AGE GO TO (13)
1110	Was (NAME) pregnant when she died?	YES . . . 1<br>GO TO 1112A<br>NO . . . 2	YES . . . 1<br>GO TO 1112A<br>NO . . . 2	YES . . . 1<br>GO TO 1112A<br>NO . . . 2	YES . . . 1<br>GO TO 1112A<br>NO . . . 2	YES . . . 1<br>GO TO 1112A<br>NO . . . 2	YES . . . 1<br>GO TO 1112A<br>NO . . . 2
1111	Did (NAME) die during childbirth?	YES . . . 1<br>GO TO 1113<br>NO . . . 2	YES . . . 1<br>GO TO 1113<br>NO . . . 2	YES . . . 1<br>GO TO 1113<br>NO . . . 2	YES . . . 1<br>GO TO 1113<br>NO . . . 2	YES . . . 1<br>GO TO 1113<br>NO . . . 2	YES . . . 1<br>GO TO 1113<br>NO . . . 2
1112	Did (NAME) die within six weeks after the end of a pregnancy or childbirth?	YES . . . 1<br>NO . . . 2<br>GO TO 1113	YES . . . 1<br>NO . . . 2<br>GO TO 1113	YES . . . 1<br>NO . . . 2<br>GO TO 1113	YES . . . 1<br>NO . . . 2<br>GO TO 1113	YES . . . 1<br>NO . . . 2<br>GO TO 1113	YES . . . 1<br>NO . . . 2<br>GO TO 1113
1112A	Was the death of (NAME) related to accident such as traffic accident?	YES . . . 1<br>NO . . . 2	YES . . . 1<br>NO . . . 2	YES . . . 1<br>NO . . . 2	YES . . . 1<br>NO . . . 2	YES . . . 1<br>NO . . . 2	YES . . . 1<br>NO . . . 2
1113	How many live born children did (NAME) give birth to during her lifetime (before this pregnancy)?						

GO BACK TO 1004 IN NEXT COLUMN, OR, IF NO MORE BROTHERS OR SISTERS, END.

1114	RECORD THE TIME.	HOUR . . . .<br>MINUTES . . . .

SECTION 12. DOMESTIC VIOLENCE MODULE

NO.	QUESTIONS AND FILTERS	CODING CATEGORIES	SKIP
DV00	CHECK COVER PAGE HOUSEHOLD QUESTIONNAIRE: HOUSEHOLD SELECTED FOR MALE INTERVIEW AND DOMESTIC VIOLENCE OF THE WOMEN	YES . . . . 1<br>NO 2	 → END
DV00A	CHECK HOUSEHOLD QUESTIONNAIRE, Q.145<br>WOMAN SELECTED FOR THIS SECTION ☐ ↓ WOMAN NOT SELECTED ☐ →		END
DV01	CHECK FOR PRESENCE OF OTHERS:<br>DO NOT CONTINUE UNTIL PRIVACY IS ENSURED.<br>PRIVACY OBTAINED ☐ ↓ PRIVACY NOT POSSIBLE ☐ →		DV32
	READ TO THE RESPONDENT<br>Now I would like to ask you questions about some other important aspects of a woman's life. You may find some of these questions very personal. However, your answers are crucial for helping to understand the condition of women in [COUNTRY]. Let me assure you that your answers are completely confidential and will not be told to anyone and no one else in your household will know that you were asked these questions.		
DV02	CHECK 601 AND 602:<br>CURRENTLY MARRIED/ LIVING WITH A MAN ☐ ↓ FORMERLY MARRIED/ LIVED WITH A MAN (READ IN PAST TENSE AND USE 'LAST' WITH HUSBAND/PARTNER') ☐ ↓ NEVER MARRIED/ NEVER LIVED WITH A MAN ☐ →		DV16

DV03 First, I am going to ask you about some situations which happen to some women. Please tell me if these apply to your relationship with your (last) (husband/partner)?

Question	Coding category	YES	NO	DK
a) He (is/was) jealous or angry if you (talk/talked) to other men?	a) JEALOUS	1	2	8
b) He frequently (accuses/accused) you of being unfaithful?	b) ACCUSES	1	2	8
c) He (does/did) not permit you to meet your female friends?	c) NOT MEET FRIENDS . . .	1	2	8
d) He (tries/tried) to limit your contact with your family?	d) NO FAMILY	1	2	8
e) He (insists/insisted) on knowing where you (are/were) at all times?	e) WHERE YOU ARE	1	2	8
f) He (does/did) not give you money to cover the household expenses?	f) GIVE MONEY	1	2	8
g) He (does/did) not trust you with money?	g) TRUST WITH MONEY	1	2	8

DV04 Now I need to ask some more questions about your relationship with your (last) (husband/partner).

A Did your (last) (husband/partner) ever:

B How often did this happen during the last 12 months: often, only sometimes, or not at all?

		EVER	OFTEN	SOME-TIMES	NOT IN LAST 12 MONTHS
a)	say or do something to humiliate you in front of others?	a) YES 1 →<br>NO 2 ↓	1	2	3
b)	threaten to hurt or harm you or someone you care about?	b) YES 1 →<br>NO 2 ↓	1	2	3
c)	insult you or make you feel bad about yourself?	c) YES 1 →<br>NO 2 ↓	1	2	3

NO.	QUESTIONS AND FILTERS	CODING CATEGORIES	SKIP
DV05	A. Did your (last) (husband/partner) ever do any of the following things to you:	B. How often did this happen during the last 12 months: often, only sometimes, or not at all?	

	A.	EVER	OFTEN	SOME-TIMES	NOT IN LAST 12 MONTHS
a)	push you, shake you, or throw something at you?	a) YES 1 → NO 2 ↓	1	2	3
b)	slap you?	b) YES 1 → NO 2 ↓	1	2	3
c)	twist your arm or pull your hair?	c) YES 1 → NO 2 ↓	1	2	3
d)	punch you with his fist or with something that could hurt you?	d) YES 1 → NO 2 ↓	1	2	3
e)	kick you, drag you, or beat you up?	e) YES 1 → NO 2 ↓	1	2	3
f)	try to choke you or burn you on purpose?	f) YES 1 → NO 2 ↓	1	2	3
g)	threaten or attack you with a knife, gun, or other weapon?	g) YES 1 → NO 2 ↓	1	2	3
h)	physically force you to have sexual intercourse with him when you did not want to?	h) YES 1 → NO 2 ↓	1	2	3
i)	physically force you to perform any other sexual acts you did not want to?	i) YES 1 → NO 2 ↓	1	2	3
j)	force you with threats or in any other way to perform sexual acts you did not want to?	j) YES 1 → NO 2 ↓	1	2	3

NO.	QUESTIONS AND FILTERS	CODING CATEGORIES	SKIP
DV06	CHECK DV05A (a-j):<br>AT LEAST ONE 'YES' ☐ ↓<br>NOT A SINGLE 'YES' ☐ →		DV09
DV07	How long after you first (got married/started living together) with your (last) (husband/partner) did (this/any of these things) first happen?<br><br>IF LESS THAN ONE YEAR, RECORD '00'.	NUMBER OF YEARS . . . . ☐☐<br><br>BEFORE MARRIAGE/BEFORE LIVING TOGETHER 95	
DV08	Did the following ever happen as a result of what your (last) (husband/partner) did to you:		
	a) You had cuts, bruises, or aches?	a) YES . . . . 1<br>NO 2	
	b) You had eye injuries, sprains, dislocations, or burns?	b) YES . . . . 1<br>NO 2	
	c) You had deep wounds, broken bones, broken teeth, or any other serious injury?	c) YES . . . . 1<br>NO 2	

NO.	QUESTIONS AND FILTERS	CODING CATEGORIES	SKIP
DV09	Have you ever hit, slapped, kicked, or done anything else to physically hurt your (last) (husband/partner) at times when he was not already beating or physically hurting you?	YES . . . . . 1<br>NO 2	 → DV11
DV10	In the last 12 months, how often have you done this to your (last) (husband/partner): often, only sometimes, or not at all?	OFTEN . . . . . 1<br>SOMETIMES . . . . . 2<br>NOT AT ALL 3	
DV11	Does (did) your (last) (husband/partner) drink alcohol?	YES . . . . . 1<br>NO 2	 → DV13
DV12	How often does (did) he get drunk: often, only sometimes, or never?	OFTEN . . . . . 1<br>SOMETIMES . . . . . 2<br>NEVER 3	
DV13	Are (Were) you afraid of your (last) (husband/partner): most of the time, sometimes, or never?	MOST OF THE TIME AFRAID . . . . . 1<br>SOMETIMES AFRAID . . . . . 2<br>NEVER AFRAID 3	
DV14	CHECK 609:<br>MARRIED MORE THAN ONCE ☐ ↓ MARRIED ONLY ONCE ☐ →		DV16
DV15	A So far we have been talking about the behavior of your (current/last) (husband/partner). Now I want to ask you about the behavior of any previous (husband/partner).	B How long ago did this last happen?	

	EVER	0 - 11 MONTHS AGO	12+ MONTHS AGO	DON'T REMEMBER
a) Did any previous (husband/partner) ever hit, slap, kick, or do anything else to hurt you physically?	a) YES 1 →<br>NO 2 ↓	1	2	3
b) Did any previous (husband/partner) physically force you to have intercourse or perform any other sexual acts against your will?	b) YES 1 →<br>NO 2	1	2	3

NO.	QUESTIONS AND FILTERS	CODING CATEGORIES	SKIP
DV16	CHECK 601 AND 602:<br>EVER MARRIED/EVER LIVED WITH A MAN ☐↓<br>From the time you were 15 years old has anyone other than (your/any) (husband/partner) hit you, slapped you, kicked you, or done anything else to hurt you physically?<br>NEVER MARRIED/NEVER LIVED WITH A MAN ☐↓<br>From the time you were 15 years old has anyone hit you, slapped you, kicked you, or done anything else to hurt you physically?	YES . . . . 1<br>NO . . . . 2<br>REFUSED TO ANSWER/ NO ANSWER 3	 2, 3 → DV19
DV17	Who has hurt you in this way?<br>Anyone else?<br>RECORD ALL MENTIONED.	MOTHER/STEP-MOTHER . . . . A<br>FATHER/STEP-FATHER . . . . B<br>SISTER/BROTHER . . . . C<br>DAUGHTER/SON . . . . D<br>OTHER RELATIVE . . . . E<br>CURRENT BOYFRIEND . . . . F<br>FORMER BOYFRIEND . . . . G<br>MOTHER-IN-LAW . . . . H<br>FATHER-IN-LAW . . . . I<br>OTHER IN-LAW . . . . J<br>TEACHER . . . . K<br>EMPLOYER/SOMEONE AT WORK . . . . L<br>POLICE/SOLDIER . . . . M<br>OTHER ________ X<br>(SPECIFY)	
DV18	In the last 12 months, how often has (this person/have these persons) physically hurt you: often, only sometimes, or not at all?	OFTEN . . . . 1<br>SOMETIMES . . . . 2<br>NOT AT ALL 3	
DV19	CHECK 201, 226, AND 230:<br>EVER BEEN PREGNANT (YES ON 201 OR 226 OR 230) ☐↓<br>NEVER BEEN PREGNANT ☐ →		DV22
DV20	Has any one ever hit, slapped, kicked, or done anything else to hurt you physically while you were pregnant?	YES . . . . 1<br>NO 2	 → DV22
DV21	Who has done any of these things to physically hurt you while you were pregnant?<br>Anyone else?<br>RECORD ALL MENTIONED.	CURRENT HUSBAND/PARTNER . . . . A<br>MOTHER/STEP-MOTHER . . . . B<br>FATHER/STEP-FATHER . . . . C<br>SISTER/BROTHER . . . . D<br>DAUGHTER/SON . . . . E<br>OTHER RELATIVE . . . . F<br>FORMER HUSBAND/PARTNER . . . . G<br>CURRENT BOYFRIEND . . . . H<br>FORMER BOYFRIEND . . . . I<br>MOTHER-IN-LAW . . . . J<br>FATHER-IN-LAW . . . . K<br>OTHER IN-LAW . . . . L<br>TEACHER . . . . M<br>EMPLOYER/SOMEONE AT WORK . . . . N<br>POLICE/SOLDIER . . . . O<br>OTHER ________ X<br>(SPECIFY)	

NO.	QUESTIONS AND FILTERS	CODING CATEGORIES	SKIP
DV22	CHECK 601 AND 602:<br><br>EVER MARRIED/EVER LIVED WITH A MAN ☐ ↓ NEVER MARRIED/NEVER LIVED WITH A MAN ☐ →		DV22B
DV22A	Now I want to ask you about things that may have been done to you by someone other than (your/any) (husband/partner).<br><br>At any time in your life, as a child or as an adult, has anyone ever forced you in any way to have sexual intercourse or perform any other sexual acts when you did not want to?	YES . . . . . 1<br>NO . . . . . 2<br>REFUSED TO ANSWER/<br>NO ANSWER 3	1 → DV23<br>2, 3 → DV24A
DV22B	At any time in your life, as a child or as an adult, has anyone ever forced you in any way to have sexual intercourse or perform any other sexual acts when you did not want to?	YES . . . . . 1<br>NO . . . . . 2<br>REFUSED TO ANSWER/<br>NO ANSWER 3	2, 3 → DV26
DV23	Has you ever been forced in any way to have sexual intercourse or perform any other sexual acts when you did not want to by several person/group of person/gang?	YES . . . . . 1<br>NO . . . . . 2<br>REFUSED TO ANSWER/<br>NO ANSWER 3	2, 3 → DV24
DV23A	Who was the person who was forcing you the very first time this happened?	CURRENT HUSBAND/PARTNER . . . . . 01<br>FORMER HUSBAND/PARTNER . . . . . 02<br>CURRENT/FORMER BOYFRIEND . . . . . 03<br>FATHER/STEP-FATHER . . . . . 04<br>BROTHER/STEP-BROTHER . . . . . 05<br>OTHER RELATIVE . . . . . 06<br>IN-LAW . . . . . 07<br>OWN FRIEND/ACQUAINTANCE . . . . . 08<br>FAMILY FRIEND . . . . . 09<br>TEACHER . . . . . 10<br>EMPLOYER/SOMEONE AT WORK . . . . . 11<br>POLICE/SOLDIER . . . . . 12<br>PRIEST/RELIGIOUS LEADER . . . . . 13<br>STRANGER . . . . . 14<br><br>OTHER ______ 96<br>(SPECIFY)	
DV24	CHECK 601 AND 602:<br><br>EVER MARRIED/EVER LIVED WITH A MAN ☐ ↓<br>In the last 12 months, has anyone other than (your/any) (husband/partner) physically forced you to have sexual intercourse when you did not want to?<br><br>NEVER MARRIED/NEVER LIVED WITH A MAN ☐ ↓<br>In the last 12 months has anyone physically forced you to have sexual intercourse when you did not want to?	YES . . . . . 1<br>NO 2	1, 2 → DV25
DV24A	CHECK DV05A (h-j) and DV15A(b)<br><br>AT LEAST ONE 'YES' ☐ ↓ NOT A SINGLE 'YES' ☐ →		DV26

NO.	QUESTIONS AND FILTERS	CODING CATEGORIES	SKIP
DV25	CHECK 601 AND 602:<br><br>EVER MARRIED/EVER LIVED WITH A MAN ☐<br>How old were you the first time you were forced to have sexual intercourse or perform any other sexual acts by anyone, including (your/any) husband/partner?<br><br>NEVER MARRIED/NEVER LIVED WITH A MAN ☐<br>How old were you the first first time you were forced to have sexual intercourse or perform any other sexual acts?	AGE IN COMPLETED YEARS [ ][ ]<br><br>DON'T KNOW 98	
DV26	CHECK DV05A (a-j), DV15A (a,b), DV16, DV20, DV22A, AND DV22B:<br><br>AT LEAST ONE 'YES' ☐<br>NOT A SINGLE 'YES' ☐ → DV30		DV30
DV27	Thinking about what you yourself have experienced among the different things we have been talking about, have you ever tried to seek help?	YES . . . . 1<br>NO 2	 → DV29
DV28	From whom have you sought help?<br><br>Anyone else?<br><br>RECORD ALL MENTIONED.	OWN FAMILY . . . . A<br>HUSBAND'S/PARTNER'S FAMILY . . . . . B<br>CURRENT/FORMER<br>HUSBAND/PARTNER . . . . C<br>CURRENT/FORMER BOYFRIEND . . . . . D<br>FRIEND . . . . E<br>NEIGHBOR . . . . F<br>RELIGIOUS LEADER . . . . G<br>DOCTOR/MEDICAL PERSONNEL . . . . . H<br>POLICE . . . . I<br>LAWYER . . . . J<br>SOCIAL SERVICE ORGANIZATION . . . . . K<br><br>OTHER ______________________ X<br>(SPECIFY)	→ DV30
DV29	Have you ever told any one about this?	YES . . . . 1<br>NO 2	
DV30	As far as you know, did your father ever beat your mother?	YES . . . . 1<br>NO . . . . 2<br>DON'T KNOW 8	

THANK THE RESPONDENT FOR HER COOPERATION AND REASSURE HER ABOUT THE CONFIDENTIALITY OF HER ANSWERS. FILL OUT THE QUESTIONS BELOW WITH REFERENCE TO THE DOMESTIC VIOLENCE MODULE ONLY.

NO.	QUESTION		YES ONCE	YES, MORE THAN ONCE	NO
DV31	DID YOU HAVE TO INTERRUPT THE INTERVIEW BECAUSE SOME ADULT WAS TRYING TO LISTEN, OR CAME INTO THE ROOM, OR INTERFERED IN ANY OTHER WAY?	HUSBAND	1	2	3
		OTHER MALE ADULT . . .	1	2	3
		FEMALE ADULT	1	2	3

DV32 INTERVIEWER'S COMMENTS / EXPLANATION FOR NOT COMPLETING THE DOMESTIC VIOLENCE MODULE

____

____

____

INTERVIEWER'S OBSERVATIONS

TO BE FILLED IN AFTER COMPLETING INTERVIEW

COMMENTS ABOUT RESPONDENT:

COMMENTS ON SPECIFIC QUESTIONS:

ANY OTHER COMMENTS:

SUPERVISOR'S OBSERVATIONS

NAME OF SUPERVISOR: ______________________ DATE: ______________

EDITOR'S OBSERVATIONS

NAME OF EDITOR: ______________________ DATE: ______________

INSTRUCTIONS:
ONLY ONE CODE SHOULD APPEAR IN ANY BOX.
COLUMN 1 REQUIRES A CODE IN EVERY MONTH.

INFORMATION TO BE CODED FOR EACH COLUMN

COLUMN 1: BIRTHS, PREGNANCIES, CONTRACEPTIVE USE**

- B BIRTHS
- P PREGNANCIES
- T TERMINATIONS

- 0 NO METHOD
- 1 FEMALE STERILIZATION
- 2 MALE STERILIZATION
- 3 IUD
- 4 INJECTABLES
- 5 IMPLANTS
- 6 PILL
- 7 MONTHLY PILL
- 8 CONDOM
- 9 FEMALE CONDOM
- D DIAPHRAGM
- J FOAM OR JELLY
- K LACTATIONAL AMENORRHEA METHOD
- L RHYTHM METHOD
- M WITHDRAWAL
- X OTHER MODERN METHOD
- Y OTHER TRADITIONAL METHOD

COLUMN 2: DISCONTINUATION OF CONTRACEPTIVE USE

- 0 INFREQUENT SEX/HUSBAND AWAY
- 1 BECAME PREGNANT WHILE USING
- 2 WANTED TO BECOME PREGNANT
- 3 HUSBAND/PARTNER DISAPPROVED
- 4 WANTED MORE EFFECTIVE METHOD
- 5 SIDE EFFECTS/HEALTH CONCERNS
- 6 LACK OF ACCESS/TOO FAR
- 7 COSTS TOO MUCH
- 8 INCONVENIENT TO USE
- F UP TO GOD/FATALISTIC
- A DIFFICULT TO GET PREGNANT/MENOPAUSAL
- D MARITAL DISSOLUTION/SEPARATION
- X OTHER ______________________ (SPECIFY)
- Z DON'T KNOW

				1	2	
	12	DEC	01			
	11	NOV	02			
	10	OCT	03			
	09	SEP	04			
**2**	08	AUG	05			**2**
**0**	07	JUL	06			**0**
**1**	06	JUN	07			**1**
**4**	05	MAY	08			**4**
*	04	APR	09			*
	03	MAR	10			
	02	FEB	11			
	01	JAN	12			
	12	DEC	13			
	11	NOV	14			
	10	OCT	15			
	09	SEP	16			
**2**	08	AUG	17			**2**
**0**	07	JUL	18			**0**
**1**	06	JUN	19			**1**
**3**	05	MAY	20			**3**
*	04	APR	21			*
	03	MAR	22			
	02	FEB	23			
	01	JAN	24			
	12	DEC	25			
	11	NOV	26			
	10	OCT	27			
	09	SEP	28			
2	08	AUG	29			2
0	07	JUL	30			0
1	06	JUN	31			1
2	05	MAY	32			2
*	04	APR	33			*
	03	MAR	34			
	02	FEB	35			
	01	JAN	36			
	12	DEC	37			
	11	NOV	38			
	10	OCT	39			
	09	SEP	40			
2	08	AUG	41			2
0	07	JUL	42			0
1	06	JUN	43			1
1	05	MAY	44			1
*	04	APR	45			*
	03	MAR	46			
	02	FEB	47			
	01	JAN	48			
	12	DEC	49			
	11	NOV	50			
	10	OCT	51			
	09	SEP	52			
2	08	AUG	53			2
0	07	JUL	54			0
1	06	JUN	55			1
0	05	MAY	56			0
*	04	APR	57			*
	03	MAR	58			
	02	FEB	59			
	01	JAN	60			
	12	DEC	61			
	11	NOV	62			
	10	OCT	63			
	09	SEP	64			
2	08	AUG	65			2
0	07	JUL	66			0
0	06	JUN	67			0
9	05	MAY	68			9
*	04	APR	69			*
	03	MAR	70			
	02	FEB	71			
	01	JAN	72			

CAMBODIA DEMOGRAPHIC AND HEALTH SURVEYS 2014
MEN QUESTIONNAIRE

MINISTRY OF PLANNING
NATIONAL INSTITUTE OF STATISTICS

MINISTRY OF HEALTH
DIRECTORATE FOR GENERAL HEALTH

IDENTIFICATION

DOMAIN . . . .

NAME OF HOUSEHOLD HEAD ________

PROVINCE ________

DISTRICT ________

COMMUNE ________

VILLAGE ________

CLUSTER NUMBER . . . .

HOUSEHOLD NUMBER . . . .

NAME AND LINE NUMBER OF MAN ________

INTERVIEWER VISITS

	1	2	3	FINAL VISIT
DATE	________	________	________	DAY<br>MONTH<br>YEAR 2 0
INTERVIEWER'S NAME	________	________	________	INT. NUMBER
RESULT*	________	________	________	RESULT*
NEXT VISIT: DATE	________	________		TOTAL NUMBER OF VISITS
TIME	________	________		

*RESULT CODES:
1 COMPLETED
2 NOT AT HOME
3 POSTPONED
4 REFUSED
5 PARTLY COMPLETED
6 INCAPACITATED
7 OTHER ________ (SPECIFY)

LANGUAGE OF QUESTIONNAIRE, LANGUAGE OF INTERVIEW, NATIVE :

KHMER LANGUAGE 1
OTHER LANGUAGE________ 2
(SPECIFY)

USED TRANSLATOR:

YES 1
NO 2

SUPERVISOR	FIELD EDITOR	OFFICE EDITOR	KEYED BY
NAME ________	NAME ________		
DATE: ________	DATE: ________		

SECTION 1. RESPONDENT'S BACKGROUND

INTRODUCTION AND CONSENT

INFORMED CONSENT

Hello. My name is ____. I am working with the ministry of planning and ministry of health. We are conducting a survey about health all over Cambodia. The information we collect will help the government to plan health services. Your household was selected for the survey. The questions usually take about 20 minutes. All of the answers you give will be confidential and will not be shared with anyone other than members of our survey team. You don't have to be in the survey, but we hope you will agree to answer the questions since your views are important. If I ask you any question you don't want to answer, just let me know and I will go on to the next question or you can stop the interview at any time.

In case you need more information about the survey, you may contact the person listed on the card that has already been given to your household.

Do you have any questions? May I begin the interview now?

SIGNATURE OF INTERVIEWER: ______________________________ DATE: __________________

RESPONDENT AGREES TO BE INTERVIEWED . . . . . 1 ↓ RESPONDENT DOES NOT AGREE TO BE INTERVIEWED . . . 2 → END

NO.	QUESTIONS AND FILTERS	CODING CATEGORIES	SKIP
101	RECORD THE TIME.	HOUR . . . . ☐☐<br>MINUTES . . . . ☐☐	
102	In what month and year were you born?<br><br>IF RESPONDENT DOES NOT KNOW GREGORIAN MONTH AND YEAR OF BIRTH, ASK FOR KHMER MONTH AND YEAR. USE DATE CONVERSION CHART.<br><br>____________________________<br>(SPECIFY KHMER MONTH AND YEAR OF BIRTH)	GREGORIAN MONTH . . . . . ☐☐<br>DON'T KNOW MONTH . . . . 98<br>GREGORIAN YEAR ☐☐☐☐<br>DON'T KNOW YEAR 9998	
103	How old were you at your last birthday?<br><br>COMPARE AND CORRECT 102 AND/OR 103 IF INCONSISTENT.	AGE IN COMPLETED YEARS ☐☐	
104	Have you ever attended school?	YES . . . . 1<br>NO 2	 → 108
105	What is the highest level of school you attended: primary, secondary, or higher?	PRIMARY . . . . 1<br>LOWER SECONDARY . . . . 2<br>UPPER SECONDARY . . . . 3<br>HIGHER 4	
106	What is the highest (grade/form/year) you completed at that level?<br><br>IF COMPLETED LESS THAN ONE YEAR AT THAT LEVEL, RECORD '00'.	GRADE/FORM/YEAR . . . . ☐☐	

NO.	QUESTIONS AND FILTERS	CODING CATEGORIES	SKIP
107	CHECK 105:<br>PRIMARY ☐ ↓ SECONDARY OR HIGHER ☐		→ 110
108	Now I would like you to read this sentence to me.<br><br>SHOW CARD TO RESPONDENT.<br><br>IF RESPONDENT CANNOT READ WHOLE SENTENCE, PROBE: Can you read any part of the sentence to me?	CANNOT READ AT ALL . . . . 1<br>ABLE TO READ ONLY PARTS OF SENTENCE . . . . 2<br>ABLE TO READ WHOLE SENTENCE 3<br>NO CARD WITH REQUIRED LANGUAGE ______ 4<br>(SPECIFY LANGUAGE)<br>BLIND/VISUALLY IMPAIRED 5	
109	CHECK 108:<br>CODE '2', '3' OR '4' CIRCLED ☐ ↓ CODE '1' OR '5' CIRCLED ☐		→ 111
110	Do you read a newspaper or magazine, at least once a week, less than once a week or not at all?	AT LEAST ONCE A WEEK . . . . 1<br>LESS THAN ONCE A WEEK . . . . 2<br>NOT AT ALL 3	
111	Do you listen to the radio, at least once a week, less than once a week or not at all?	AT LEAST ONCE A WEEK . . . . 1<br>LESS THAN ONCE A WEEK . . . . 2<br>NOT AT ALL 3	
112	Do you watch television, at least once a week, less than once a week or not at all?	AT LEAST ONCE A WEEK . . . . 1<br>LESS THAN ONCE A WEEK . . . . 2<br>NOT AT ALL 3	
112A	Do you access to internet, at least once a week, less than once a week or not at all?	AT LEAST ONCE A WEEK . . . . 1<br>LESS THAN ONCE A WEEK . . . . 2<br>NOT AT ALL 3	
113	What is your religion?	BUDDHIST . . . . 1<br>MOSLEM . . . . 2<br>CHRISTIAN . . . . 3<br>OTHER 4	
115	In the last 12 months, how many times have you been away from home for one or more nights?	NUMBER OF TIMES . . . . ☐☐<br>NONE 00	 → 201
116	In the last 12 months, have you been away from home for more than one month at a time?	YES . . . . 1<br>NO 2	

SECTION 2. REPRODUCTION

NO.	QUESTIONS AND FILTERS	CODING CATEGORIES	SKIP
201	Now I would like to ask about any children you have had during your life. I am interested in all of the children that are biologically yours, even if they are not legally yours or do not have your last name.<br><br>Have you ever fathered any children with any woman?	YES . . . . . 1<br>NO . . . . . 2<br>DON'T KNOW 8	 NO/DON'T KNOW → 206
202	Do you have any sons or daughters that you have fathered who are now living with you?	YES . . . . . 1<br>NO 2	 → 204
203	How many sons live with you?<br><br>And how many daughters live with you?<br><br>IF NONE, RECORD '00'.	SONS AT HOME . . . . . [ ][ ]<br>DAUGHTERS AT HOME . . . . . [ ][ ]	
204	Do you have any sons or daughters that you have fathered who are alive but do not live with you?	YES . . . . . 1<br>NO 2	 → 206
205	How many sons are alive but do not live with you?<br><br>And how many daughters are alive but do not live with you?<br><br>IF NONE, RECORD '00'.	SONS ELSEWHERE . . . . . [ ][ ]<br>DAUGHTERS ELSEWHERE . . . [ ][ ]	
205A	Where do your sons or daughters who do not live with you live?	EXTENDED FAMILY/CAMP . . . . . A<br>NEIGHBOR . . . . . B<br>ORPHANAGE/NGO . . . . . C<br>TEMPLE (WAT) . . . . . D<br>OTHER ________ E<br>(SPECIFY)<br>DON'T KNOW X	
206	Have you ever fathered a son or a daughter who was born alive but later died?<br><br>IF NO, PROBE: Any baby who cried or showed signs of life but did not survive?	YES . . . . . 1<br>NO . . . . . 2<br>DON'T KNOW 8	 NO/DON'T KNOW → 208
207	How many boys have died?<br><br>And how many girls have died?<br><br>IF NONE, RECORD '00'.	BOYS DEAD . . . . . [ ][ ]<br>GIRLS DEAD . . . . . [ ][ ]	
208	SUM ANSWERS TO 203, 205, AND 207, AND ENTER TOTAL.<br><br>IF NONE, RECORD '00'.	TOTAL CHILDREN . . . . . [ ][ ]	
209	CHECK 208:<br>HAS HAD MORE THAN ONE CHILD [ ] ↓<br>HAS HAD ONLY ONE CHILD [ ] → 212<br>HAS NOT HAD ANY CHILDREN [ ] → 401		212<br>401
210	Did all of the children you have fathered have the same biological mother?	YES . . . . . 1<br>NO 2	YES → 212
211	In all, how many women have you fathered children with?	NUMBER OF WOMEN . . . . . [ ][ ]	
212	How old were you when your (first) child was born?	AGE IN YEARS . . . . . [ ][ ]	

NO.	QUESTIONS AND FILTERS	CODING CATEGORIES	SKIP
213	CHECK 203 AND 205:<br>AT LEAST ONE LIVING CHILD ☐ ↓ NO LIVING CHILDREN ☐ → 401		401
214	How old is your (youngest) child?	AGE IN YEARS . . . . ☐☐	
215	CHECK 214:<br>(YOUNGEST) CHILD IS AGE 0-2 YEARS ☐ ↓ OTHER ☐ → 401		401
216	What is the name of your (youngest) child?<br>WRITE NAME OF (YOUNGEST) CHILD<br>________________<br>(NAME OF (YOUNGEST) CHILD)		
217	When (NAME)'s mother was pregnant with (NAME), did she have any antenatal check-ups?	YES . . . . 1<br>NO . . . . 2<br>DON'T KNOW 8	NO, DON'T KNOW → 219
218	Were you ever present during any of those antenatal check-ups?	PRESENT . . . . 1<br>NOT PRESENT 2	
219	Was (NAME) born in a hospital or health facility?	HOSPITAL/HEALTH FACILITY . . . . . 1<br>OTHER 2	
220	When a child has diarrhea, how much should he or she be given to drink: more than usual, about the same as usual, less than usual, or nothing to drink at all?	MORE THAN USUAL . . . . 1<br>ABOUT THE SAME . . . . 2<br>LESS THAN USUAL . . . . 3<br>NOTHING TO DRINK . . . . 4<br>DON'T KNOW 8	

SECTION 4. MARRIAGE AND SEXUAL ACTIVITY

NO.	QUESTIONS AND FILTERS	CODING CATEGORIES	SKIP
401	Are you currently married or living together with a woman as if married?	YES, CURRENTLY MARRIED . . . . 1<br>YES, LIVING WITH A WOMAN . . . . . 2<br>NO, NOT IN UNION 3	404
402	Have you ever been married or lived together with a woman as if married?	YES, FORMERLY MARRIED . . . . 1<br>YES, LIVED WITH A WOMAN . . . . 2<br>NO 3	413
403	What is your marital status now: are you widowed, divorced, or separated?	WIDOWED . . . . 1<br>DIVORCED . . . . 2<br>SEPARATED 3	410
404	Is your (wife/partner) living with you now or is she staying elsewhere?	LIVING WITH HIM . . . . 1<br>STAYING ELSEWHERE 2	
405	Do you have other wives or do you live with other women as if married?	YES (MORE THAN ONE) . . . . 1<br>NO (ONLY ONE) 2	
409	Please tell me the name of the (your wife/the woman you are living with)<br><br>RECORD THE NAME AND THE LINE NUMBER FROM THE HOUSEHOLD QUESTIONNAIRE FOR SPOUSE OR LIVE-IN PARTNER IF THE PERSON IS NOT LISTED IN THE HOUSEHOLD, RECODE '00'.	NAME ________________<br><br>LINE NUMBER . . . . [ ][ ]	
410	Have you been married or lived with a woman only once or more than once?	ONLY ONCE . . . . 1<br>MORE THAN ONCE 2	411A

NO.	QUESTIONS AND FILTERS	CODING CATEGORIES	SKIP
411<br><br>411A	In what month and year did you start living with your (wife/partner)?<br><br>Now I would like to ask about your first (wife/partner). In what month and year did you start living with her?<br><br>IF RESPONDENT DOES NOT KNOW GREGORIAN DATE ASK FOR KHMER MONTH AND YEAR OF MARRIAGE. USE DATE CONVERSION CHART TO FIND GREGORIAN MONTH AND YEAR.<br><br>________________________<br>(SPECIFY KHMER MONTH AND YEAR OF MARRIAGE)	GREGORIAN MONTH . . . . [ ][ ]<br><br>DON'T KNOW MONTH . . . . 98<br><br>GREGORIAN YEAR [ ][ ][ ][ ]<br><br>DON'T KNOW YEAR 9998	 <br><br><br>413
412	How old were you when you first started living with her?	AGE . . . . [ ][ ]	
413	CHECK FOR THE PRESENCE OF OTHERS.<br><br>BEFORE CONTINUING, MAKE EVERY EFFORT TO ENSURE PRIVACY.		
414	Now I would like to ask some questions about sexual activity in order to gain a better understanding of some important life issues.<br><br>How old were you when you had sexual intercourse for the very first time?	NEVER HAD SEXUAL INTERCOURSE . . . . .00<br><br>AGE IN YEARS . . . . [ ][ ]<br><br>FIRST TIME WHEN STARTED LIVING WITH (FIRST) WIFE/PARTNER 95	501
415	Now I would like to ask you some questions about your recent sexual activity. Let me assure you again that your answers are completely confidential and will not be told to anyone. If we should come to any question that you don't want to answer, just let me know and we will go to the next question.		
416	When was the <u>last</u> time you had sexual intercourse?<br><br>IF LESS THAN 12 MONTHS, ANSWER MUST BE RECORDED IN DAYS, WEEKS OR MONTHS.<br><br>IF 12 MONTHS (ONE YEAR) OR MORE, ANSWER MUST BE RECORDED IN YEARS.	DAYS AGO . . . . 1 [ ][ ]<br><br>WEEKS AGO . . . . 2 [ ][ ]<br><br>MONTHS AGO . . . . 3 [ ][ ]<br><br>YEARS AGO . . . . 4 [ ][ ]	 <br><br><br><br><br>430

		LAST SEXUAL PARTNER	SECOND-TO-LAST SEXUAL PARTNER	THIRD-TO-LAST SEXUAL PARTNER
417	When was the last time you had sexual intercourse with this person?		DAYS AGO 1 [ ][ ]<br>WEEKS AGO 2 [ ][ ]<br>MONTHS AGO 3 [ ][ ]	DAYS AGO 1 [ ][ ]<br>WEEKS AGO 2 [ ][ ]<br>MONTHS AGO 3 [ ][ ]
418	The last time you had sexual intercourse (with this second/third person), was a condom used?	YES . . . . 1<br>NO . . . . 2<br>(SKIP TO 420)←	YES . . . . 1<br>NO . . . . 2<br>(SKIP TO 420)←	YES . . . . 1<br>NO . . . . 2<br>(SKIP TO 420)←
419	Was a condom used every time you had sexual intercourse with this person in the last 12 months?	YES . . . . 1<br>NO 2	YES . . . . 1<br>NO 2	YES . . . . 1<br>NO 2
420	What was your relationship to this person with whom you had sexual intercourse?<br><br>IF GIRLFRIEND:<br>Were you living together as if married?<br><br>IF YES, CIRCLE '2'.<br>IF NO, CIRCLE '3'.	WIFE . . . . 1<br>LIVE-IN PARTNER . . . . 2<br>GIRLFRIEND NOT LIVING WITH RESPONDENT . . . . 3<br>CASUAL ACQUAINTANCE . . . 4<br>PROSTITUTE . . . . . 5<br>OTHER ___________ 6<br>(SPECIFY)<br>(SKIP TO 422A) ←	WIFE . . . . 1<br>LIVE-IN PARTNER . . . . 2<br>GIRLFRIEND NOT LIVING WITH RESPONDENT . . . . 3<br>CASUAL ACQUAINTANCE . . . 4<br>PROSTITUTE . . . . . 5<br>OTHER ___________ 6<br>(SPECIFY)<br>(SKIP TO 422A) ←	WIFE . . . . 1<br>LIVE-IN PARTNER . . . . 2<br>GIRLFRIEND NOT LIVING WITH RESPONDENT . . . . 3<br>CASUAL ACQUAINTANCE . . . 4<br>PROSTITUTE . . . . . . 5<br>OTHER ___________ 6<br>(SPECIFY)<br>(SKIP TO 422A) ←
421	CHECK 410:	MARRIED ONLY ONCE [ ]↓<br>MARRIED MORE THAN ONCE [ ] (SKIP TO 423)	MARRIED ONLY ONCE [ ]↓<br>MARRIED MORE THAN ONCE [ ] (SKIP TO 423)	MARRIED ONLY ONCE [ ]↓<br>MARRIED MORE THAN ONCE [ ] (SKIP TO 423)
422	CHECK 414:	FIRST TIME WHEN STARTED LIVING WITH FIRST WIFE (SKIP TO 424)<br>OTHER [ ]↓ (SKIP TO 423)	FIRST TIME WHEN STARTED LIVING WITH FIRST WIFE (SKIP TO 424)<br>OTHER [ ]↓ (SKIP TO 423)	FIRST TIME WHEN STARTED LIVING WITH FIRST WIFE (SKIP TO 424)<br>OTHER [ ]↓ (SKIP TO 423)
422A	Who was the person that you had sexual intercourse in the last 12 months?	BROTHEL-BASED SEX WORKER . . . 01<br>KARAOKE WORKER 02<br>BEER PROMOTER . 03<br>BAR/NIGHT CLUB WORKER . . . . 04<br>MASSEUSE . . . . 05<br>RESTAURANT WORKER . . . . 06<br>STREET-BASED SEX WORKER . . . 07<br>BARBER 08<br>OTHER___________ 98<br>(SPECIFY)	BROTHEL-BASED SEX WORKER . . . 01<br>KARAOKE WORKER 02<br>BEER PROMOTER . 03<br>BAR/NIGHT CLUB WORKER . . . . 04<br>MASSEUSE . . . . 05<br>RESTAURANT WORKER . . . . 06<br>STREET-BASED SEX WORKER . . . 07<br>BARBER 08<br>OTHER___________ 98<br>(SPECIFY)	BROTHEL-BASED SEX WORKER . . . . 01<br>KARAOKE WORKER 02<br>BEER PROMOTER . 03<br>BAR/NIGHT CLUB WORKER . . . . 04<br>MASSEUSE . . . . 05<br>RESTAURANT WORKER . . . . 06<br>STREET-BASED SEX WORKER . . . . 07<br>BARBER 08<br>OTHER___________ 98<br>(SPECIFY)

		LAST SEXUAL PARTNER	SECOND-TO-LAST SEXUAL PARTNER	THIRD-TO-LAST SEXUAL PARTNER
423	How long ago did you first have sexual intercourse with this (second/third) person?	DAYS AGO 1 [ ][ ]<br>WEEKS AGO 2 [ ][ ]<br>MONTHS AGO 3 [ ][ ]<br>YEARS AGO 4 [ ][ ]	DAYS AGO 1 [ ][ ]<br>WEEKS AGO 2 [ ][ ]<br>MONTHS AGO 3 [ ][ ]<br>YEARS AGO 4 [ ][ ]	DAYS AGO 1 [ ][ ]<br>WEEKS AGO 2 [ ][ ]<br>MONTHS AGO 3 [ ][ ]<br>YEARS AGO 4 [ ][ ]
424	How many times during the last 12 months did you have sexual intercourse with this person?	NUMBER OF TIMES [ ][ ]	NUMBER OF TIMES [ ][ ]	NUMBER OF TIMES [ ][ ]
425	How old is this person?	AGE OF PARTNER [ ][ ]<br>DON'T KNOW 98	AGE OF PARTNER [ ][ ]<br>DON'T KNOW 98	AGE OF PARTNER [ ][ ]<br>DON'T KNOW 98
426	Apart from (this person/these two people), have you had sexual intercourse with any other person in the last 12 months?	YES . . . . 1<br>(GO BACK TO 417 IN NEXT COLUMN)<br>NO . . . . 2<br>(SKIP TO 427A)	YES . . . . 1<br>(GO BACK TO 417 IN NEXT COLUMN)<br>NO . . . . 2<br>(SKIP TO 427A)	
427	In total, with how many different people have you had sexual intercourse in the last 12 months?<br>IF NON-NUMERIC ANSWER, PROBE TO GET AN ESTIMATE.<br>IF NUMBER OF PARTNERS IS 95 OR MORE, WRITE '95'.			NUMBER OF PARTNERS LAST 12 MONTHS . . . [ ][ ]<br>DON'T KNOW . . . 98

NO.	QUESTIONS AND FILTERS	CODING CATEGORIES	SKIP
427A	Have you heard of men having sex with men?	YES . . . . 1<br>NO 2	 → 428
427B	Have you ever had sex with a man?	YES . . . . 1<br>NO 2	

NO.	QUESTIONS AND FILTERS	CODING CATEGORIES	SKIP
428	CHECK 420 (ALL COLUMNS):<br>AT LEAST ONE PARTNER IS PROSTITUTE ☐ ↓<br>NO PARTNERS ARE PROSTITUTES ☐ → 430		430
429	CHECK 420 AND 418 (ALL COLUMNS):<br>CONDOM USED WITH EVERY PROSTITUTE ☐ → 433<br>OTHER ☐ → 434		433<br>434
430	In the last 12 months, did you pay anyone in exchange for having sexual intercourse?	YES . . . . . 1<br>NO 2	1 → 432
431	Have you ever paid anyone in exchange for having sexual intercourse?	YES . . . . . 1<br>NO 2	→ 434
432	The last time you paid someone in exchange for having sexual intercourse, was a condom used?	YES . . . . . 1<br>NO 2	2 → 434
433	Was a condom used during sexual intercourse every time you paid someone in exchange for having sexual intercourse in the last 12 months?	YES . . . . . 1<br>NO . . . . . 2<br>DON'T KNOW 8	
434	In the last 12 months, did you ever give money, gifts or favors to anyone in exchange for having sexual intercourse?	YES . . . . . 1<br>NO 2	
434A	In total, with how many different people have you had sexual intercourse in your lifetime?<br>IF NON-NUMERIC ANSWER, PROBE TO GET AN ESTIMATE.<br>IF NUMBER OF PARTNERS IS 95 OR MORE, WRITE '95'.	NUMBER OF PARTNERS IN LIFETIME . . . . . ☐☐<br>DON'T KNOW 98	
435	CHECK 418, MOST RECENT PARTNER (FIRST COLUMN):<br>NOT ASKED ☐ → 438<br>CONDOM USED ☐ ↓<br>NO CONDOM USED ☐ → 438		438<br>438
436	You told me that a condom was used the last time you had sex. What is the brand name of the condom used at that time?<br>IF BRAND NOT KNOWN, ASK TO SEE THE PACKAGE.	OK . . . . . 01<br>NUMBER ONE . . . . . 02<br>NEAK KAPEAR/NO NAME . . . . . 03<br>OTHER _____ (SPECIFY) 96<br>DON'T KNOW 98	
436A	You told me that a condom was used the last time you had sex. How much did you pay for that condom?	RIELS . . . . . ☐☐☐☐☐<br>FREE . . . . . 00000<br>IN KIND . . . . . 99996<br>DON'T KNOW 99998	

NO.	QUESTIONS AND FILTERS	CODING CATEGORIES	SKIP
437	From where did you obtain the condom the last time?<br><br>PROBE TO IDENTIFY TYPE OF SOURCE.<br><br>IF UNABLE TO DETERMINE IF PUBLIC OR PRIVATE SECTOR, WRITE THE NAME OF THE PLACE.<br><br>________________<br>(NAME OF PLACE)	PUBLIC SECTOR<br>NATIONAL HOSPITAL (PP) . . . . 11<br>PROVINCIAL HOSP (RH) . . . . 12<br>DISTRICT HOSPITAL (RH) . . . . 13<br>HEALTH CENTEF . . . . 14<br>HEALTH POST . . . . 15<br>OUTREACH . . . . 16<br>MILITARY HOSPITAL . . . . 17<br>OTHER PUBLIC<br>SECTOR ________ 18<br>(SPECIFY)<br><br>PRIVATE MEDICAL SECTOR<br>PRIVATE HOSPITAL . . . . . 21<br>PRIVATE CLINIC . . . . 22<br>PHARMACY . . . . 23<br>OTHER PRIVATE MEDICAL<br>SECTOR ________ 24<br>(SPECIFY)<br><br>OTHER SOURCE<br>SHOP . . . . 31<br>COMMUNITY DISTRIBUTOR . . . . . 32<br>FRIEND/RELATIVE . . . . 33<br><br>OTHER ________ 36<br>(SPECIFY)	
438	The last time you had sex did you or your partner use any method (other than a condom) to avoid or prevent a pregnancy?	YES . . . . 1<br>NO . . . . 2<br>DON'T KNOW 8	 2, 8 → 501
439	What method did you or your partner use?<br><br>PROBE:<br>Did you or your partner use any other method to prevent pregnancy?<br><br>RECORD ALL MENTIONED.	FEMALE STERILIZATION . . . . A<br>MALE STERILIZATION . . . . B<br>IUD . . . . C<br>INJECTABLES . . . . D<br>IMPLANTS . . . . E<br>PILL . . . . F<br>FEMALE CONDOM . . . . G<br>DIAPHRAGM . . . . H<br>FOAM/JELLY . . . . I<br>LAM . . . . J<br>RHYTHM METHOD . . . . K<br>WITHDRAWAL . . . . L<br>OTHER MODERN METHOD . . . . X<br>OTHER TRADITIONAL METHOD . . . . . Y	

SECTION 5. FERTILITY PREFERENCES

NO.	QUESTIONS AND FILTERS	CODING CATEGORIES	SKIP
501	CHECK 401:<br>CURRENTLY MARRIED OR LIVING WITH A PARTNER ☐ ↓<br>NOT CURRENTLY MARRIED AND NOT LIVING WITH A PARTNER ☐ → 509		509
502	CHECK 439:<br>MAN NOT STERILIZED ☐ ↓<br>MAN STERILIZED ☐ → 509		509
503	(Is your (wife/partner)/Are any of your (wives/partners)) currently pregnant?	YES . . . . . 1<br>NO . . . . . 2<br>DON'T KNOW 8	 NO, DON'T KNOW → 505
504	Now I have some questions about the future. After the (child/children) you and your (wife(wives)/partner(s)) are expecting now, would you like to have another child, or would you prefer not have any more children?	HAVE ANOTHER CHILD . . . . . 1<br>NO MORE/NONE . . . . . 2<br>UNDECIDED/DON'T KNOW 8	1 → 506<br>2, 8 → 509
505	Now I have some questions about the future. Would you like to have (a/another) child, or would you prefer not to have any (more) children?	HAVE (A/ANOTHER) CHILD . . . . . 1<br>NO MORE/NONE . . . . . 2<br>SAYS COUPLE CAN'T GET PREGNANT . . . . . 3<br>WIFE (WIVES)/PARTNER(S) STERILIZED . . . . . 4<br>UNDECIDED/DON'T KNOW 8	2, 3, 4, 8 → 509
506	CHECK 405:<br>ONE WIFE/ PARTNER ☐ ↓<br>MORE THAN ONE WIFE/ PARTNER ☐ → 508		508
507	CHECK 503:<br>WIFE/PARTNER NOT PREGNANT OR DON'T KNOW ☐ ↓<br>How long would you like to wait from now before the birth of (a/another) child?<br>WIFE/PARTNER PREGNANT ☐ ↓<br>After the birth of the child you are expecting now, how long would you like to wait before the birth of another child?	MONTHS . . . . . 1 ☐☐<br>YEARS . . . . . 2 ☐☐<br>SOON/NOW . . . . . 993<br>COUPLE INFECUND . . . . . 994<br>OTHER ______ 996<br>(SPECIFY)<br>DON'T KNOW 998	→ 509
508	How long would you like to wait from now before the birth of (a/another) child?	MONTHS . . . . . 1 ☐☐<br>YEARS . . . . . 2 ☐☐<br>SOON/NOW . . . . . 993<br>HE/ALL HIS WIVES/PARTNERS ARE INFECUND . . . . . 994<br>OTHER ______ 996<br>(SPECIFY)<br>DON'T KNOW 998	

NO.	QUESTIONS AND FILTERS	CODING CATEGORIES	SKIP
509	CHECK 203 AND 205:<br>HAS LIVING CHILDREN ☐ ↓<br>If you could go back to the time you did not have any children and could choose exactly the number of children to have in your whole life, how many would that be?<br>NO LIVING CHILDREN ☐ ↓<br>If you could choose exactly the number of children to have in your whole life, how many would that be?<br>PROBE FOR A NUMERIC RESPONSE.	NONE . . . . 00<br>NUMBER . . . . ☐☐<br>OTHER ______________________ 96<br>(SPECIFY)	→ 601<br><br>→ 601
510	How many of these children would you like to be boys, how many would you like to be girls and for how many would it not matter if it's a boy or a girl?	BOYS GIRLS EITHER<br>NUMBER ☐☐ ☐☐ ☐☐<br>OTHER ______________________ 96<br>(SPECIFY)	

SECTION 6. EMPLOYMENT AND GENDER ROLES

NO.	QUESTIONS AND FILTERS	CODING CATEGORIES	SKIP
601	Have you done any work in the last seven days?	YES . . . . 1<br>NO 2	1 → 604
602	Although you did not work in the last seven days, do you have any job or business from which you were absent for leave, illness, vacation, or any other such reason?	YES . . . . 1<br>NO 2	1 → 604
603	Have you done any work in the last 12 months?	YES . . . . 1<br>NO 2	2 → 610
604	What is your occupation, that is, what kind of work do you mainly do?	____________ [ ][ ]<br>____________<br>____________	
605	Do you usually work throughout the year, or do you work seasonally, or only once in a while?	THROUGHOUT THE YEAR . . . . 1<br>SEASONALLY/PART OF THE YEAR 2<br>ONCE IN A WHILE 3	
606	Are you paid in cash or kind for this work or are you not paid at all?	CASH ONLY . . . . 1<br>CASH AND KIND . . . . 2<br>IN KIND ONLY . . . . 3<br>NOT PAID 4	
607	CHECK 401:<br>CURRENTLY MARRIED OR LIVING WITH A PARTNER [ ] ↓<br>NOT CURRENTLY MARRIED AND NOT LIVING WITH A PARTNER [ ] → 612		612
608	CHECK 606:<br>CODE 1 OR 2 CIRCLED [ ] ↓<br>OTHER [ ] → 610		610
609	Who usually decides how the money you earn will be used: you, your (wife/partner), or you and your (wife/partner) jointly?	RESPONDENT . . . . 1<br>WIFE/PARTNER . . . . 2<br>RESPONDENT AND WIFE/ PARTNER JOINTLY . . . . 3<br>OTHER ____________ 6<br>(SPECIFY)	
610	Who usually makes decisions about health care for yourself: you, your (wife/partner), you and your (wife/partner) jointly, or someone else?	RESPONDENT . . . . 1<br>WIFE/PARTNER . . . . 2<br>RESPONDENT AND WIFE/ PARTNER JOINTLY . . . . 3<br>SOMEONE ELSE . . . . 4<br>OTHER ____________ 6<br>(SPECIFY)	
611	Who usually makes decisions about making major household purchases?	RESPONDENT . . . . 1<br>WIFE/PARTNER . . . . 2<br>RESPONDENT AND WIFE/ PARTNER JOINTLY . . . . 3<br>SOMEONE ELSE . . . . 4<br>OTHER ____________ 6<br>(SPECIFY)	

NO.	QUESTIONS AND FILTERS	CODING CATEGORIES	SKIP
612	Do you own this or any other house either alone or jointly with someone else?	ALONE ONLY . . . . 1<br>JOINTLY ONLY . . . . 2<br>BOTH ALONE AND JOINTLY . . . . . 3<br>DOES NOT OWN 4	
613	Do you own any land either alone or jointly with someone else?	ALONE ONLY . . . . 1<br>JOINTLY ONLY . . . . 2<br>BOTH ALONE AND JOINTLY . . . . . 3<br>DOES NOT OWN 4	
614	In your opinion, is a husband justified in hitting or beating his wife in the following situations:<br><br>If she goes out without telling him?<br>If she neglects the children?<br>If she argues with him?<br>If she refuses to have sex with him?<br>If she burns the food?<br>If she ask him to use condom?	YES NO DK<br><br>GOES OUT . . . . 1 2 8<br>NEGL. CHILDREN . . . 1 2 8<br>ARGUES . . . . 1 2 8<br>REFUSES SEX . . . . . 1 2 8<br>BURNS FOOD . . . . 1 2 8<br>ASK USE CONDOM . 1 2 8	
615	In your opinion, is a parent justified in hitting or beating his son for the following reasons:<br><br>If he disobeys?<br>If he impolite?<br>If he has embarrassed the family?<br>If he does not do the housework or cooking?<br>If he does not take care of younger sibling ?	YES NO DK<br><br>DISOBEY . . . . 1 2 8<br>IMPOLITE . . . . 1 2 8<br>EMBARR. FAMILY . . . 1 2 8<br>HOUSEWORK . . . . 1 2 8<br>CARE SIBLING . . . 1 2 8	
616	In your opinion, is a parent justified in hitting or beating his daughter for the following reasons:<br><br>If she disobeys?<br>If she impolite?<br>If she has embarrassed the family?<br>If she does not do the housework or cooking?<br>If she does not take care of younger sibling ?	YES NO DK<br><br>DISOBEY . . . . 1 2 8<br>IMPOLITE . . . . 1 2 8<br>EMBARR. FAMILY . . . 1 2 8<br>HOUSEWORK . . . . 1 2 8<br>CARE SIBLING . . . 1 2 8	

<u>SECTION 7. HIV/AIDS</u>

NO.	QUESTIONS AND FILTERS	CODING CATEGORIES	SKIP
701	Now I would like to talk about something else. Have you ever heard of an illness called AIDS?	YES . . . 1<br>NO . . . 2	 → 723
702	Can people reduce their chances of getting the AIDS virus by having just one uninfected sex partner who has no other sex partners?	YES . . . 1<br>NO . . . 2<br>DON'T KNOW . . . 8	
703	Can people get the AIDS virus from mosquito bites?	YES . . . 1<br>NO . . . 2<br>DON'T KNOW . . . 8	
704	Can people reduce their chance of getting the AIDS virus by using a condom every time they have sex?	YES . . . 1<br>NO . . . 2<br>DON'T KNOW . . . 8	
705	Can people get the AIDS virus by sharing food with a person who has AIDS?	YES . . . 1<br>NO . . . 2<br>DON'T KNOW . . . 8	
706	Can people get the AIDS virus because of witchcraft or other supernatural means?	YES . . . 1<br>NO . . . 2<br>DON'T KNOW . . . 8	
707	Is it possible for a healthy-looking person to have the AIDS virus?	YES . . . 1<br>NO . . . 2<br>DON'T KNOW . . . 8	
708	Can the virus that causes AIDS be transmitted from a mother to her baby:<br>During pregnancy?<br>During delivery?<br>By breastfeeding?	YES NO DK<br>DURING PREG. . . . 1 2 8<br>DURING DELIVERY . . . 1 2 8<br>BREASTFEEDING . . . 1 2 8	
709	CHECK 708:<br>AT LEAST ONE 'YES' ☐ ↓ OTHER ☐		→ 711
710	Are there any special drugs that a doctor or a nurse can give to a woman infected with the AIDS virus to reduce the risk of transmission to the baby?	YES . . . 1<br>NO . . . 2<br>DON'T KNOW . . . 8	
711	CHECK FOR PRESENCE OF OTHERS. BEFORE CONTINUING, MAKE EVERY EFFORT TO ENSURE PRIVACY.		
712	I don't want to know the results, but have you ever been tested to see if you have the AIDS virus?	YES . . . 1<br>NO . . . 2	 → 716
713	How many months ago was your most recent HIV test?	MONTHS AGO . . . ☐☐<br>TWO OR MORE YEARS . . . 96	
714	I don't want to know the results, but did you get the results of the test?	YES . . . 1<br>NO . . . 2	
714A	How often did you have had an HIV test?	ONLY ONCE . . . 1<br>EVERY YEAR . . . 2<br>EVERY 6 MONTHS . . . 3<br>EVERY 3 MONTHS . . . 4<br>OTHER ______ 6<br>SPECIFY	

NO.	QUESTIONS AND FILTERS	CODING CATEGORIES	SKIP
715	Where was the test done?<br><br>PROBE TO IDENTIFY THE TYPE OF SOURCE.<br><br>IF UNABLE TO DETERMINE IF PUBLIC OR PRIVATE SECTOR, WRITE THE NAME OF THE PLACE.<br><br>________________________<br>(NAME OF PLACE)	PUBLIC SECTOR<br>NATIONAL HOSPITAL (PP) . . . . 11<br>PROVINCIAL HOSP (RH) . . . . 12<br>DISTRICT HOSPITAL (RH) . . . 13<br>HEALTH CENTER . . . . 14<br>HEALTH POST . . . . 15<br>OUTREACH . . . . 16<br>MILITARY HOSPITAL . . . . 17<br>VCCT CENTER 18<br>PMTCT SITE 19<br>OTHER PUBLIC<br>SECTOR ____________ 20<br>(SPECIFY)<br><br>PRIVATE MEDICAL SECTOR<br>PRIVATE HOSPITAL . . . . 21<br>PRIVATE CLINIC . . . . 22<br>PRIVATE LABORATORY . . . 23<br>OTHER PRIVATE MEDICAL<br>SECTOR ____________ 24<br>(SPECIFY)<br><br>OTHER ____________ 96<br>(SPECIFY)	→ 718
716	Do you know of a place where people can go to get tested for the AIDS virus?	YES . . . . 1<br>NO 2	 → 718
717	Where is that?<br><br>Any other place?<br><br>PROBE TO IDENTIFY EACH TYPE OF SOURCE.<br><br>IF UNABLE TO DETERMINE IF PUBLIC OR PRIVATE SECTOR, WRITE THE NAME OF THE PLACE.<br><br>________________________<br>(NAME OF PLACE)	PUBLIC SECTOR<br>NATIONAL HOSPITAL (PP) . . . . A<br>PROVINCIAL HOSP (RH) . . . . B<br>DISTRICT HOSPITAL (RH) . . . . C<br>HEALTH CENTEF . . . . D<br>HEALTH POST . . . . E<br>OUTREACH . . . . F<br>MILITARY HOSPITAL . . . . G<br>VCT CENTER . . . . H<br>PMTCT SITE . . . . I<br>OTHER PUBLIC ____________ J<br>(SPECIFY)<br><br>PRIVATE MEDICAL SECTOR<br>PRIVATE HOSPITAL . . . . . K<br>PRIVATE CLIN . . . . L<br>PRIVATE LABORAT . . . . M<br>OTHER PRIVATE MEDICAL<br>SECTOR ____________ N<br>(SPECIFY)<br><br>OTHER ____________ X<br>(SPECIFY)	
718	Would you buy fresh vegetables from a shopkeeper or vendor if you knew that this person had the AIDS virus?	YES . . . . 1<br>NO . . . . 2<br>DON'T KNOW 8	

NO.	QUESTIONS AND FILTERS	CODING CATEGORIES	SKIP
719	If a member of your family got infected with the AIDS virus, would you want it to remain a secret or not?	YES, REMAIN A SECRET . . . . 1<br>NO . . . . 2<br>DK/NOT SURE/DEPENDS 8	
720	If a member of your family became sick with AIDS, would you be willing to care for her or him in your own household?	YES . . . . 1<br>NO . . . . 2<br>DK/NOT SURE/DEPENDS 8	
721	In your opinion, if a female teacher has the AIDS virus but is not sick, should she be allowed to continue teaching in the school?	SHOULD BE ALLOWED . . . . 1<br>SHOULD NOT BE ALLOWED . . . . 2<br>DK/NOT SURE/DEPENDS 8	
722	Do you personally know someone who has been denied health services in the last 12 months because he or she is suspected to have the AIDS virus or has the AIDS virus?	YES . . . . 1<br>NO . . . . 2<br>DK ANYONE WITH AIDS 8	
722A	Do you agree or disagree with the following statement: People with the AIDS virus should be ashamed of themselves.	AGREE . . . . 1<br>DISAGREE . . . . 2<br>DON'T KNOW/NO OPINION 8	
722B	Do you agree or disagree with the following statement: People with the AIDS virus should be ashamed of themselves.	AGREE . . . . 1<br>DISAGREE . . . . 2<br>DON'T KNOW/NO OPINION 8	
723	CHECK 701:<br>HEARD ABOUT AIDS ☐ ↓<br>Apart from AIDS, have you heard about other infections that can be transmitted through sexual contact?<br>NOT HEARD ABOUT AIDS ☐ ↓<br>Have you heard about infections that can be transmitted through sexual contact?	YES . . . . 1<br>NO 2	
724	CHECK 414:<br>HAS HAD SEXUAL INTERCOURSE ☐ ↓<br>HAS NOT HAD SEXUAL INTERCOURSE ☐ →		732
725	CHECK 723: HEARD ABOUT OTHER SEXUALLY TRANSMITTED INFECTIONS?<br>YES ☐ ↓<br>NO ☐ →		727
726	Now I would like to ask you some questions about your health in the last 12 months. During the last 12 months, have you had a disease which you got through sexual contact?	YES . . . . 1<br>NO . . . . 2<br>DON'T KNOW 8	
727	Sometimes men experience an abnormal discharge from their penis.<br>During the last 12 months, have you had an abnormal discharge from your penis?	YES . . . . 1<br>NO . . . . 2<br>DON'T KNOW 8	
728	Sometimes men have a sore or ulcer near their penis. During the last 12 months, have you had a sore or ulcer near your penis?	YES . . . . 1<br>NO . . . . 2<br>DON'T KNOW 8	
729	CHECK 726, 727, AND 728:<br>HAS HAD AN INFECTION (ANY 'YES') ☐ ↓<br>HAS NOT HAD AN INFECTION OR DOES NOT KNOW ☐ →		732
730	The last time you had (PROBLEM FROM 726/727/728), did you seek any kind of advice or treatment?	YES . . . . 1<br>NO 2	NO → 732

NO.	QUESTIONS AND FILTERS	CODING CATEGORIES	SKIP
731	Where did you go?<br><br>Any other place?<br><br>PROBE TO IDENTIFY EACH TYPE OF SOURCE.<br><br>IF UNABLE TO DETERMINE IF PUBLIC OR PRIVATE SECTOR, WRITE THE NAME OF THE PLACE.<br><br>____________________<br>(NAME OF PLACE(S))	PUBLIC SECTOR<br>NATIONAL HOSPITAL (PP) . . . . A<br>PROVINCIAL HOSP (RH) . . . . B<br>DISTRICT HOSPITAL (RH) . . . . C<br>HEALTH CENTER . . . . D<br>HEALTH POST . . . . E<br>OUTREACH . . . . F<br>MILITARY HOSPITAL . . . . G<br>OTHER PUBLIC . . . .<br>SECTOR ____________ H<br>(SPECIFY)<br><br>PRIVATE MEDICAL SECTOR<br>PRIVATE HOSPITAL . . . . . I<br>PRIVATE CLINIC . . . . J<br>PHARMACY . . . . K<br>OTHER PRIVATE MEDICAL<br>SECTOR ____________ L<br>(SPECIFY)<br><br>OTHER SOURCE<br>SHOP . . . . M<br>FRIEND/RELATIVE . . . . N<br><br>OTHER ____________ X<br>(SPECIFY)	
732	If a wife knows her husband has a disease that she can get during sexual intercourse, is she justified in asking that they use a condom when they have sex?	YES . . . . 1<br>NO . . . . 2<br>DON'T KNOW 8	
733	Is a wife justified in refusing to have sex with her husband when she knows her husband has sex with other women?	YES . . . . 1<br>NO . . . . 2<br>DON'T KNOW 8	

SECTION 8. OTHER HEALTH ISSUES

NO.	QUESTIONS AND FILTERS	CODING CATEGORIES	SKIP
805	Now I would like to ask you some other questions relating to health matters. Have you had an injection for any reason in the last 12 months?<br>IF YES: How many injections have you had?<br>IF NUMBER OF INJECTIONS IS 90 OR MORE, OR DAILY FOR 3 MONTHS OR MORE, RECORD '90'.<br>IF NON-NUMERIC ANSWER, PROBE TO GET AN ESTIMATE.	NUMBER OF INJECTIONS . . . [ ][ ]<br>NONE 00	 → 808
806	Among these injections, how many were administered by a doctor, a nurse, a pharmacist, a dentist, or any other health worker?<br>IF NUMBER OF INJECTIONS IS 90 OR MORE, OR DAILY FOR 3 MONTHS OR MORE, RECORD '90'.<br>IF NON-NUMERIC ANSWER, PROBE TO GET AN ESTIMATE.	NUMBER OF INJECTIONS . . . [ ][ ]<br>NONE 00	 → 808
807	The last time you got an injection from a health worker, did he/she take the syringe and needle from a new, unopened package?	YES . . . . 1<br>NO . . . . 2<br>DON'T KNOW 8	
808	Do you currently smoke cigarettes?	YES . . . . 1<br>NO 2	 → 810
809	In the last 24 hours, how many cigarettes did you smoke?	NUMBER OF CIGARETTES . . . . [ ][ ]	
810	Do you currently smoke or use any (other) type of tobacco?	YES . . . . 1<br>NO 2	 → 812
811	What (other) type of tobacco do you currently smoke or use?<br>RECORD ALL MENTIONED.	PIPE . . . . A<br>CHEWING TOBACCO . . . . B<br>SNUFF . . . . C<br>OTHER ______________________ X<br>(SPECIFY)	

NO.	QUESTIONS AND FILTERS	CODING CATEGORIES	SKIP
812	Are you covered by any health insurance?	YES . . . . 1<br>NO 2	 → 814
813	What type of health insurance are you covered by?<br><br>RECORD ALL MENTIONED.	HEALTH EQUITY FUND . . . . A<br>COMMUNITY-BASED HEALTH<br>INSURANCE . . . . B<br>HEALTH INSURANCE THROUGH<br>EMPLOYER . . . . C<br>OTHER PRIVATELY PURCHASED<br>COMMERCIAL HEALTH INSURANCE D<br>OTHER ______________ X<br>(SPECIFY)	
814	RECORD THE TIME.	HOUR . . . . [ ][ ]<br>MINUTES . . . . [ ][ ]	

INTERVIEWER'S OBSERVATIONS

TO BE FILLED IN AFTER COMPLETING INTERVIEW

COMMENTS ABOUT RESPONDENT:

COMMENTS ON SPECIFIC QUESTIONS:

ANY OTHER COMMENTS:

SUPERVISOR'S OBSERVATIONS

NAME OF SUPERVISOR: ____________________ DATE: ____________________

EDITOR'S OBSERVATIONS

NAME OF EDITOR: ____________________ DATE: ____________________

CAMBODIA DEMOGRAPHIC AND HEALTH SURVEYS 2014
MICRONUTRIENT QUESTIONNAIRE

MINISTRY OF PLANNING
NATIONAL INSTITUTE OF STATISTICS

MINISTRY OF HEALTH
DIRECTORATE FOR GENERAL HEALTH

IDENTIFICATION	
DOMAIN	
NAME OF HOUSEHOLD HEAD ______	
PROVINCE ______	
DISTRICT ______	
COMMUNE ______	
VILLAGE ______	
CLUSTER NUMBER	
HOUSEHOLD NUMBER	
ARE THERE ELIGIBLE CHILDREN FOR MICRONUTRIENT TEST?	YES = 1<br>NO = 2
ARE THERE ELIGIBLE WOMEN FOR MICRONUTRIENT TEST?	YES = 1<br>NO = 2
RESPONDENT AGREES TO BE REVISITED ?	YES = 1<br>NO = 2
ID OF HOUSEHOLD INTERVIEWER	
NAME OF MICRONUTRIENT INTERVIEWER ______	

THIS PAGE IS INTENTIONALLY BLANK

MICRONUTRIENTS FOR CHILDREN AGE 6-59 MONTH

251	CHECK COLUMN 11 IN HOUSEHOLD SCHEDULE. RECORD THE LINE NUMBER AND NAME FOR ALL ELIGIBLE CHILDREN 0-5 YEARS IN QUESTION 252. IF MORE THAN SIX CHILDREN, USE ADDITIONAL QUESTIONNAIRE(S).			
		CHILD 1	CHILD 2	CHILD 3
252	LINE NUMBER FROM COLUMN 11<br><br>NAME FROM COLUMN 2	LINE NUMBER . . . . . ☐☐<br><br>NAME ________	LINE NUMBER . . . . . ☐☐<br><br>NAME ________	LINE NUMBER . . . . . ☐☐<br><br>NAME ________
253	IF MOTHER INTERVIEWED, COPY MONTH AND YEAR OF BIRTH FROM BIRTH HISTORY AND ASK DAY; IF MOTHER NOT INTERVIEWED, ASK:<br>What is (NAME's) birth date?	DAY . . . . ☐☐<br>MONTH . . . . . ☐☐<br>YEAR ☐☐☐☐	DAY . . . . ☐☐<br>MONTH . . . . . ☐☐<br>YEAR ☐☐☐☐	DAY . . . . ☐☐<br>MONTH . . . . . ☐☐<br>YEAR ☐☐☐☐
254	CHECK 253:<br>CHILD BORN IN JANUARY 2009 OR LATER?	YES . . . . 1<br>NO . . . . 2<br>(GO TO 253 FOR NEXT CHILD OR, IF NO MORE CHILDREN, GO TO 270)	YES . . . . 1<br>NO . . . . 2<br>(GO TO 253 FOR NEXT CHILD OR, IF NO MORE CHILDREN, GO TO 270)	YES . . . . 1<br>NO . . . . 2<br>(GO TO 253 FOR NEXT CHILD OR, IF NO MORE CHILDREN, GO TO 270)
255	CHECK 253:<br>IS CHILD AGE 0-5 MONTHS, I.E., WAS CHILD BORN IN MONTH OF INTERVIEW OR FIVE PREVIOUS MONTHS?	0-5 MONTHS . . . . 1<br>(GO TO 253 FOR NEXT CHILD OR, IF NO MORE CHILDREN, GO TO 270)<br>OLDER 2	0-5 MONTHS . . . . 1<br>(GO TO 253 FOR NEXT CHILD OR, IF NO MORE CHILDREN, GO TO 270)<br>OLDER 2	0-5 MONTHS . . . . 1<br>(GO TO 253 FOR NEXT CHILD OR, IF NO MORE CHILDREN, GO TO 270)<br>OLDER 2
256	LINE NUMBER OF PARENT/ OTHER ADULT RESPONSIBLE FOR THE CHILD (FROM COLUMN 1 OF HOUSEHOLD SCHEDULE.)<br>RECORD '00' IF NOT LISTED.	LINE NUMBER . . . . . ☐☐	LINE NUMBER . . . . . ☐☐	LINE NUMBER . . . . . ☐☐
257	ASK CONSENT FOR VENOUS BLOOD FOR MICRONUTRIENT TESTS FROM PARENT/OTHER ADULT IDENTIFIED IN 256 AS RESPONSIBLE FOR CHILD.	As part of this survey, we are asking people all over the country to take micronutrient tests. Micronutrient deficiency is a serious health problem that usually results from poor nutrition, infection, or chronic disease. This survey will assist the government to develop programs to prevent and treat micronutrient deficiency.<br><br>We ask that all children born in 2009 or later take part in micronutrient testing in this survey and give 4 ml of venous blood. The blood will be tested for iron, zinc, calcium, vitamin A, vitamin B9, vitamin D, inflammatory factors, and hemoglobinopathy.The equipment used to take the blood is clean and completely safe. It has never been used before and will be thrown away after each blood draw.<br><br>The blood will be tranported to the central laboratory, and the result will not be available to you. No one else will be able to know (NAME OF CHILD/NAME OF CHILDREN) test results either<br><br>Do you have any questions?<br>You can say yes to the test, or you can say no. It is up to you to decide.<br>Will you allow (NAME OF CHILD/NAMES OF CHILDREN) to participate in the micronutrient tests?		
258	CIRCLE THE APPROPRIATE CODE AND SIGN YOUR NAME.	GRANTED . . . . 1<br>________ (SIGN)<br>REFUSED 2	GRANTED . . . . 1<br>________ (SIGN)<br>REFUSED 2	GRANTED . . . . 1<br>________ (SIGN)<br>REFUSED 2
259	ASK CONSENT FOR IODINE TEST FROM PARENT/OTHER ADULT IDENTIFIED IN 256 AS RESPONSIBLE FOR CHILD.	As part of this survey, we are asking people all over the country to take iodine test. Iodine deficiency is a serious health problem that usually results from poor nutrition, infection, or chronic disease. This survey will assist the government to develop programs to prevent and treat anemia.<br><br>We ask that all children born in 2009 or later take part in iodine testing in this survey and give 4 ml of urine. The urine will be tested for iodine.The equipment used to take the urine is clean and completely safe.<br><br>The urine will be tranported to the central laboratory, and the result will not be available to you. No one else will be able to know (NAME OF CHILD/NAME OF CHILDREN) test results either<br><br>Do you have any questions?<br>You can say yes to the test, or you can say no. It is up to you to decide.<br>Will you allow (NAME OF CHILD/NAMES OF CHILDREN) to participate in the iodine test?		

MICRONUTRIENTS FOR CHILDREN AGE 6-59 MONTH

		CHILD 1	CHILD 2	CHILD 3
260	CIRCLE THE APPROPRIATE CODE AND SIGN YOUR NAME.	GRANTED . . . . 1<br>________ (SIGN)<br>REFUSED 2	GRANTED . . . . 1<br>________ (SIGN)<br>REFUSED 2	GRANTED . . . . 1<br>________ (SIGN)<br>REFUSED 2
261	ASK CONSENT FOR INTESTINAL PARASITE TEST FROM PARENT/OTHER ADULT IDENTIFIED IN 256 AS RESPONSIBLE FOR CHILD.	As part of this survey, we are asking people all over the country to take part in the tests for intertinal parasite. Intestinal parasite is a serious health problem. This survey will assist the government to develop programs to prevent and treat anemia.<br><br>We ask that all children born in 2009 or later take part in Intestinal parasite testing in this survey and give 1 gram of stool. The stool will be tested for intestinal paraside.The equipment used to take the stool is clean and completely safe.<br><br>The stool will be tranported to the central laboratory, and the result will not be available to you. No one else will be able to know (NAME OF CHILD/NAME OF CHILDREN) test results either<br><br>Do you have any questions?<br>You can say yes to the test, or you can say no. It is up to you to decide.<br>Will you allow (NAME OF CHILD/NAMES OF CHILDREN) to participate in the test?		
262	CIRCLE THE APPROPRIATE CODE AND SIGN YOUR NAME.	GRANTED . . . . 1<br>________ (SIGN)<br>REFUSED 2	GRANTED . . . . 1<br>________ (SIGN)<br>REFUSED 2	GRANTED . . . . 1<br>________ (SIGN)<br>REFUSED 2
263	CHECK Q.258<br>PUT THE 2ND LABEL ON VAVUTAINER TUBE.<br>PUT THE 3RD LABEL ON THE BLOOD TRANSMITTAL FORM	AGREE FOR BLOOD TEST<br>PUT THE 1ST BARCODE LABEL HERE<br>NOT PRESENT . . . 99994<br>REFUSED . . . . 99995<br>OTHER 99996	AGREE FOR BLOOD TEST<br>PUT THE 1ST BARCODE LABEL HERE<br>NOT PRESENT . . . 99994<br>REFUSED . . . . 99995<br>OTHER 99996	AGREE FOR BLOOD TEST<br>PUT THE 1ST BARCODE LABEL HERE<br>NOT PRESENT . . . 99994<br>REFUSED . . . . 99995<br>OTHER 99996
263A	CHECK Q.260<br>PUT THE 5TH LABEL ON URINE CONTAINER<br>PUT THE 6TH LABEL ON THE URINE TRANSMITTAL FORM	AGREE FOR URINE TEST<br>PUT THE 4TH BARCODE LABEL HERE<br>NOT PRESENT . . . 99994<br>REFUSED . . . . 99995<br>OTHER 99996	AGREE FOR URINE TEST<br>PUT THE 4TH BARCODE LABEL HERE<br>NOT PRESENT . . . 99994<br>REFUSED . . . . 99995<br>OTHER 99996	AGREE FOR URINE TEST<br>PUT THE 4TH BARCODE LABEL HERE<br>NOT PRESENT . . . 99994<br>REFUSED . . . . 99995<br>OTHER 99996
263B	CHECK Q.262<br>PUT THE 8TH LABEL ON STOOL CONTAINER<br>PUT THE 9TH LABEL ON THE STOOL TRANSMITTAL FORM	AGREE FOR STOOL TEST<br>PUT THE 7TH BARCODE LABEL HERE<br>NOT PRESENT . . . 99994<br>REFUSED . . . . 99995<br>OTHER 99996	AGREE FOR STOOL TEST<br>PUT THE 7TH BARCODE LABEL HERE<br>NOT PRESENT . . . 99994<br>REFUSED . . . . 99995<br>OTHER 99996	AGREE FOR STOOL TEST<br>PUT THE 7TH BARCODE LABEL HERE<br>NOT PRESENT . . . 99994<br>REFUSED . . . . 99995<br>OTHER 99996
264	GO BACK TO 253 IN NEXT COLUMN OF THIS QUESTIONNAIRE OR IN THE FIRST COLUMN OF THE NEXT PAGE; IF NO MORE CHILDREN, GO TO 270.			

MICRONUTRIENTS FOR CHILDREN AGE 6-59 MONTH

		CHILD 4	CHILD 5	CHILD 6
252	LINE NUMBER FROM COLUMN 11<br><br>NAME FROM COLUMN 2	LINE NUMBER . . . . . [ ][ ]<br><br>NAME ________	LINE NUMBER . . . . . [ ][ ]<br><br>NAME ________	LINE NUMBER . . . . . [ ][ ]<br><br>NAME ________
253	IF MOTHER INTERVIEWED, COPY MONTH AND YEAR OF BIRTH FROM BIRTH HISTORY AND ASK DAY; IF MOTHER NOT INTERVIEWED, ASK:<br>What is (NAME's) birth date?	DAY . . . . [ ][ ]<br>MONTH . . . . . [ ][ ]<br>YEAR [ ][ ][ ][ ]	DAY . . . . [ ][ ]<br>MONTH . . . . . [ ][ ]<br>YEAR [ ][ ][ ][ ]	DAY . . . . [ ][ ]<br>MONTH . . . . . [ ][ ]<br>YEAR [ ][ ][ ][ ]
254	CHECK 253:<br>CHILD BORN IN JANUARY 2009 OR LATER?	YES . . . . 1<br>NO . . . . 2<br>(GO TO 253 FOR NEXT CHILD OR, IF NO MORE CHILDREN, GO TO 270)	YES . . . . 1<br>NO . . . . 2<br>(GO TO 253 FOR NEXT CHILD OR, IF NO MORE CHILDREN, GO TO 270)	YES . . . . 1<br>NO . . . . 2<br>(GO TO 253 FOR NEXT CHILD OR, IF NO MORE CHILDREN, GO TO 270)
255	CHECK 253:<br>IS CHILD AGE 0-5 MONTHS, I.E., WAS CHILD BORN IN MONTH OF INTERVIEW OR FIVE PREVIOUS MONTHS?	0-5 MONTHS . . . . 1<br>(GO TO 253 FOR NEXT CHILD OR, IF NO MORE CHILDREN, GO TO 270)<br>OLDER 2	0-5 MONTHS . . . . 1<br>(GO TO 253 FOR NEXT CHILD OR, IF NO MORE CHILDREN, GO TO 270)<br>OLDER 2	0-5 MONTHS . . . . 1<br>(GO TO 253 FOR NEXT CHILD OR, IF NO MORE CHILDREN, GO TO 270)<br>OLDER 2
256	LINE NUMBER OF PARENT/ OTHER ADULT RESPONSIBLE FOR THE CHILD (FROM COLUMN 1 OF HOUSEHOLD SCHEDULE.)<br>RECORD '00' IF NOT LISTED.	LINE NUMBER . . . . . [ ][ ]	LINE NUMBER . . . . . [ ][ ]	LINE NUMBER . . . . . [ ][ ]
257	ASK CONSENT FOR VENOUS BLOOD FOR MICRONUTRIENT TESTS FROM PARENT/OTHER ADULT IDENTIFIED IN 256 AS RESPONSIBLE FOR CHILD.	As part of this survey, we are asking people all over the country to take micronutrient tests. Micronutrient deficiency is a serious health problem that usually results from poor nutrition, infection, or chronic disease. This survey will assist the government to develop programs to prevent and treat micronutrient deficiency.<br><br>We ask that all children born in 2009 or later take part in micronutrient testing in this survey and give 4 ml of venous blood. The blood will be tested for iron, zinc, calcium, vitamin A, vitamin B9, vitamin D, inflammatory factors, and hemoglobinopathy.The equipment used to take the blood is clean and completely safe. It has never been used before and will be thrown away after each blood draw.<br><br>The blood will be tranported to the central laboratory, and the result will not be available to you. No one else will be able to know (NAME OF CHILD/NAME OF CHILDREN) test results either<br><br>Do you have any questions?<br>You can say yes to the test, or you can say no. It is up to you to decide.<br>Will you allow (NAME OF CHILD/NAMES OF CHILDREN) to participate in the micronutrient tests?		
258	CIRCLE THE APPROPRIATE CODE AND SIGN YOUR NAME.	GRANTED . . . . 1<br>________ (SIGN)<br>REFUSED 2	GRANTED . . . . 1<br>________ (SIGN)<br>REFUSED 2	GRANTED . . . . 1<br>________ (SIGN)<br>REFUSED 2
259	ASK CONSENT FOR IODINE TEST FROM PARENT/OTHER ADULT IDENTIFIED IN 256 AS RESPONSIBLE FOR CHILD.	As part of this survey, we are asking people all over the country to take iodine test. Iodine deficiency is a serious health problem that usually results from poor nutrition, infection, or chronic disease. This survey will assist the government to develop programs to prevent and treat anemia.<br><br>We ask that all children born in 2009 or later take part in iodine testing in this survey and give 4 ml of urine. The urine will be tested for iodine.The equipment used to collect the urine is clean and completely safe.<br><br>The urine will be tranported to the central laboratory, and the result will not be available to you. No one else will be able to know (NAME OF CHILD/NAME OF CHILDREN) test results either<br><br>Do you have any questions?<br>You can say yes to the test, or you can say no. It is up to you to decide.<br>Will you allow (NAME OF CHILD/NAMES OF CHILDREN) to participate in the iodine test?		
260	CIRCLE THE APPROPRIATE CODE AND SIGN YOUR NAME.	GRANTED . . . . 1<br>________ (SIGN)<br>REFUSED 2	GRANTED . . . . 1<br>________ (SIGN)<br>REFUSED 2	GRANTED . . . . 1<br>________ (SIGN)<br>REFUSED 2

MICRONUTRIENTS FOR CHILDREN AGE 6-59 MONTH

		CHILD 4	CHILD 5	CHILD 6
261	ASK CONSENT FOR INTESTINAL PARASITE TEST FROM PARENT/OTHER ADULT IDENTIFIED IN 256 AS RESPONSIBLE FOR CHILD.	As part of this survey, we are asking people all over the country to take part in the tests for intertinal parasite. Intestinal parasite is a serious health problem. This survey will assist the government to develop programs to prevent and treat anemia.<br><br>We ask that all children born in 2009 or later take part in Intestinal parasite testing in this survey and give 1 gram of stool. The stool will be tested for intestinal paraside.The equipment used to collect the stool is clean and completely safe.<br><br>The stool will be tranported to the central laboratory, and the result will not be available to you. No one else will be able to know (NAME OF CHILD/NAME OF CHILDREN) test results either<br><br>Do you have any questions?<br>You can say yes to the test, or you can say no. It is up to you to decide.<br>Will you allow (NAME OF CHILD/NAMES OF CHILDREN) to participate in the test?		
262	CIRCLE THE APPROPRIATE CODE AND SIGN YOUR NAME.	GRANTED . . . . 1<br>(SIGN)<br>REFUSED 2	GRANTED . . . . 1<br>(SIGN)<br>REFUSED 2	GRANTED . . . . 1<br>(SIGN)<br>REFUSED 2
263	CHECK Q.258<br>PUT THE 2ND LABEL ON VAVUTAINER TUBE.<br>PUT THE 3RD LABEL ON THE BLOOD TRANSMITTAL FORM	AGREE FOR BLOOD TEST<br>PUT THE 1ST BARCODE LABEL HERE<br>NOT PRESENT . . . 99994<br>REFUSED . . . . 99995<br>OTHER 99996	AGREE FOR BLOOD TEST<br>PUT THE 1ST BARCODE LABEL HERE<br>NOT PRESENT . . . 99994<br>REFUSED . . . . 99995<br>OTHER 99996	AGREE FOR BLOOD TEST<br>PUT THE 1ST BARCODE LABEL HERE<br>NOT PRESENT . . . 99994<br>REFUSED . . . . 99995<br>OTHER 99996
263A	CHECK Q.260<br>PUT THE 5TH LABEL ON URINE CONTAINER<br>PUT THE 6TH LABEL ON THE URINE TRANSMITTAL FORM	AGREE FOR URINE TEST<br>PUT THE 4TH BARCODE LABEL HERE<br>NOT PRESENT . . . 99994<br>REFUSED . . . . 99995<br>OTHER 99996	AGREE FOR URINE TEST<br>PUT THE 4TH BARCODE LABEL HERE<br>NOT PRESENT . . . 99994<br>REFUSED . . . . 99995<br>OTHER 99996	AGREE FOR URINE TEST<br>PUT THE 4TH BARCODE LABEL HERE<br>NOT PRESENT . . . 99994<br>REFUSED . . . . 99995<br>OTHER 99996
263B	CHECK Q.262<br>PUT THE 8TH LABEL ON STOOL CONTAINER<br>PUT THE 9TH LABEL ON THE STOOL TRANSMITTAL FORM	AGREE FOR STOOL TEST<br>PUT THE 7TH BARCODE LABEL HERE<br>NOT PRESENT . . . 99994<br>REFUSED . . . . 99995<br>OTHER 99996	AGREE FOR STOOL TEST<br>PUT THE 7TH BARCODE LABEL HERE<br>NOT PRESENT . . . 99994<br>REFUSED . . . . 99995<br>OTHER 99996	AGREE FOR STOOL TEST<br>PUT THE 7TH BARCODE LABEL HERE<br>NOT PRESENT . . . 99994<br>REFUSED . . . . 99995<br>OTHER 99996
264	GO BACK TO 253 IN NEXT COLUMN OF THIS QUESTIONNAIRE OR IN THE FIRST COLUMN OF THE NEXT PAGE; IF NO MORE CHILDREN, GO TO 270.			

MICRONUTRIENT FOR WOMEN AGE 15-49

	CHECK COLUMN 9 IN HOUSEHOLD SCHEDULE. RECORD THE LINE NUMBER AND NAME FOR ALL ELIGIBLE WOMEN IN 270.<br>IF THERE ARE MORE THAN THREE WOMEN, USE ADDITIONAL QUESTIONNAIRE(S).			
		WOMAN 1	WOMAN 2	WOMAN 3
270	LINE NUMBER FROM COLUMN 9<br>NAME FROM COLUMN 2	LINE NUMBER . . . . [ ][ ]<br>NAME ____________	LINE NUMBER . . . . [ ][ ]<br>NAME ____________	LINE NUMBER . . . . [ ][ ]<br>NAME ____________
271	CHECK 224: WOMEN QUESTIONNAIRES	ONE OR MORE BIRTHS IN 2009 OR LATER [ ]<br>NO BIRTHS IN 2009 OR LATER [ ] → GO TO 271 IN NEXT COLUMN; IF NO MORE WOMEN GO TO THE LAST SECTION	ONE OR MORE BIRTHS IN 2009 OR LATER [ ]<br>NO BIRTHS IN 2009 OR LATER [ ] → GO TO 271 IN NEXT COLUMN; IF NO MORE WOMEN GO TO THE LAST SECTION	ONE OR MORE BIRTHS IN 2009 OR LATER [ ]<br>NO BIRTHS IN 2009 OR LATER [ ] → GO TO 271 IN THE FIRST COLUMN OF AN ADDITIONAL QUESTIONNAIRE; IF NO MORE WOMEN GO TO THE LAST SECTION
272	AGE: CHECK COLUMN 7.	15-17 YEARS . . . . 1<br>18-49 YEARS . . . . 2<br>(GO TO 277)	15-17 YEARS . . . . 1<br>18-49 YEARS . . . . 2<br>(GO TO 277)	15-17 YEARS . . . . 1<br>18-49 YEARS . . . . 2<br>(GO TO 277)
273	MARITAL STATUS: CHECK COLUMN 8.	CODE 4 (NEVER IN UNION) . . . . . 1<br>OTHER . . . . 2<br>(GO TO 277)	CODE 4 (NEVER IN UNION) . . . . . 1<br>OTHER . . . . 2<br>(GO TO 277)	CODE 4 (NEVER IN UNION) . . . . . 1<br>OTHER . . . . 2<br>(GO TO 277)
274	RECORD LINE NUMBER OF PARENT/OTHER ADULT RESPON-SIBLE FOR ADOLESCENT. RECORD '00' IF NOT LISTED.	LINE NUMBER OF PARENT OR OTHER RESPONSIBLE ADULT [ ][ ]<br>NAME: ____________	LINE NUMBER OF PARENT OR OTHER RESPONSIBLE ADULT [ ][ ]<br>NAME: ____________	LINE NUMBER OF PARENT OR OTHER RESPONSIBLE ADULT [ ][ ]<br>NAME: ____________
275	ASK CONSENT FOR MICRONUTRIENT TEST FROM PARENT/ OTHER ADULT IDENTIFIED IN 274 AS RESPONSIBLE FOR NEVER IN UNION WOMEN AGE 15-17.	As part of this survey, we are asking people all over the country to take micronutrient tests. Micronutrient deficiency is a serious health problem that usually results from poor nutrition, infection, or chronic disease. This survey will assist the government to develop programs to prevent and treat micrnutrient deficiency. For the micronutrient testing, we will need 6 ml of blood from a venous puncture. The blood will be tested for iron, zinc, calcium, vitamins A, D, and B9, inflammatory factors, and hemoglobinopathy. The equipment used to take the blood is clean and completely safe. It has never been used before and will be thrown away after each test. The blood will be transported to the central laboratory, and the result will not be available to you. No one else will be able to know (NAME OF ADOLESCENT) test results either.<br><br>Do you have any questions?<br>You can say yes to the test for (NAME OF ADOLESCENT), or you can say no. It is up to you to decide.<br>Will you allow (NAME OF ADOLESCENT) to take the micronutrient tests?		
276	CIRCLE THE APPROPRIATE CODE AND SIGN YOUR NAME.	GRANTED . . . . 1<br>____________ (SIGN)<br>PARENT/OTHER RESPONSIBLE ADULT REFUSED . . . . 2<br>(IF REFUSED, GO TO 279)	GRANTED . . . . 1<br>____________ (SIGN)<br>PARENT/OTHER RESPONSIBLE ADULT REFUSED . . . . 2<br>(IF REFUSED, GO TO 279)	GRANTED . . . . 1<br>____________ (SIGN)<br>PARENT/OTHER RESPONSIBLE ADULT REFUSED . . . . 2<br>(IF REFUSED, GO TO 279)
277	ASK CONSENT FOR MICRONUTRIENT TEST FROM RESPONDENT.	As part of this survey, we are asking people all over the country to take micronutrient tests. Micronutrient deficiency is a serious health problem that usually results from poor nutrition, infection, or chronic disease. This survey will assist the government to develop programs to prevent and treat micrnutrient deficiency. For the micronutrient testing, we will need 6 ml of blood from a venous puncture. The blood will be tested for iron, zinc, calcium, vitamins A, D, and B9, inflammatory factors, and hemoglobinopathy. The equipment used to take the blood is clean and completely safe. It has never been used before and will be thrown away after each test. The blood will be transported to the central laboratory, and the result will not be available to you. No one else will be able to know your test results either.<br><br>Do you have any questions?<br>You can say yes to the test, or you can say no. It is up to you to decide.<br>Will you take the micronutrient tests?		
278	CIRCLE THE APPROPRIATE CODE AND SIGN YOUR NAME.	GRANTED . . . . 1<br>____________ (SIGN)<br>RESPONDENT REFUSED 2	GRANTED . . . . 1<br>____________ (SIGN)<br>RESPONDENT REFUSED 2	GRANTED . . . . 1<br>____________ (SIGN)<br>RESPONDENT REFUSED 2

MICRONUTRIENT FOR WOMEN AGE 15-49

		WOMAN 1	WOMAN 2	WOMAN 3
279	CHECK:<br><br>272 AND 273	272 : 15-17 YEARS<br>273 : OTHER → 282<br>272 : 18-49 YEARS → 282<br>272 : 15-17 YEARS<br>273 : CODE 4 (NEVER IN UNION) ↓	272 : 15-17 YEARS<br>273 : OTHER → 282<br>272 : 18-49 YEARS → 282<br>272 : 15-17 YEARS<br>273 : CODE 4 (NEVER IN UNION) ↓	272 : 15-17 YEARS<br>273 : OTHER → 282<br>272 : 18-49 YEARS → 282<br>272 : 15-17 YEARS<br>273 : CODE 4 (NEVER IN UNION) ↓
280	ASK CONSENT FOR IODINE TEST FROM PARENT/ OTHER ADULT IDENTIFIED IN 274 AS RESPONSIBLE FOR NEVER IN UNION WOMEN AGE 15-17.	As part of this survey, we are asking people all over the country to take an iodine test. Iodine deficiency is a serious health problem that usually results from poor nutrition, infection, or chronic disease. This survey will assist the government to develop programs to prevent and treat iodine deficiency. For the iodine testing, we will need 2 ml of urine. The equipment used to collect the urine is clean and completely safe. It has never been used before and will be thrown away after each test. The urine will be transported to the central laboratory, and the result will not be available to you. No one else will be able to know (NAME OF ADOLESCENT) test results either.<br><br>Do you have any questions?<br>You can say yes to the test for (NAME OF ADOLESCENT), or you can say no. It is up to you to decide.<br>Will you allow (NAME OF ADOLESCENT) to take the iodine test?		
281	CIRCLE THE APPROPRIATE CODE AND SIGN YOUR NAME.	GRANTED . . . . 1<br>__________<br>(SIGN)<br>PARENT/OTHER RESPONSIBLE ADULT REFUSED . . . . 2<br>(IF REFUSED, GO TO 284)	GRANTED . . . . 1<br>__________<br>(SIGN)<br>PARENT/OTHER RESPONSIBLE ADULT REFUSED . . . . 2<br>(IF REFUSED, GO TO 284)	GRANTED . . . . 1<br>__________<br>(SIGN)<br>PARENT/OTHER RESPONSIBLE ADULT REFUSED . . . . 2<br>(IF REFUSED, GO TO 284)
282	ASK CONSENT FOR IODINE TEST FROM RESPONDENT.	As part of this survey, we are asking people all over the country to take an iodine test. Iodine deficiency is a serious health problem that usually results from poor nutrition, infection, or chronic disease. This survey will assist the government to develop programs to prevent and treat iodine deficiency. For the iodine testing, we will need 2 ml of urine. The equipment used to collect urine is clean and completely safe. It has never been used before and will be thrown away after each test. The urine will be transported to the central laboratory, and the result will not be available to you. No one else will be able to know your test results either.<br><br>Do you have any questions?<br>You can say yes to the test, or you can say no. It is up to you to decide.<br>Will you take the iodine test?		
283	CIRCLE THE APPROPRIATE CODE AND SIGN YOUR NAME.	GRANTED . . . . 1<br>__________<br>(SIGN)<br>RESPONDENT REFUSED 2	GRANTED . . . . 1<br>__________<br>(SIGN)<br>RESPONDENT REFUSED 2	GRANTED . . . . 1<br>__________<br>(SIGN)<br>RESPONDENT REFUSED 2
284	CHECK:<br><br>272 AND 273	272 : 15-17 YEARS<br>273 : OTHER → 287<br>272 : 18-49 YEARS → 287<br>272 : 15-17 YEARS<br>273 : CODE 4 (NEVER IN UNION) ↓	272 : 15-17 YEARS<br>273 : OTHER → 287<br>272 : 18-49 YEARS → 287<br>272 : 15-17 YEARS<br>273 : CODE 4 (NEVER IN UNION) ↓	272 : 15-17 YEARS<br>273 : OTHER → 287<br>272 : 18-49 YEARS → 287<br>272 : 15-17 YEARS<br>273 : CODE 4 (NEVER IN UNION) ↓
285	ASK CONSENT FOR INTESTINAL PARASITE FROM PARENT/OTHER ADULTS IDENTIFIED IN 274 AS RESPONSIBLE FOR NEVER IN UNION WOMEN AGE 15-17.	As part of this survey, we are asking people all over the country to take a test for intestinal parasite. Intestinal parasite is a serious health problem. This survey will assist the government to develop programs to prevent and treat intestinal parasite. For the intestinal parasite testing, we will need 1 gram of stool. The equipment used to collect the stool is clean and completely safe. It has never been used before and will be thrown away after each test. The stool will be transported to the central laboratory, and the result will not be available to you. No one else will be able to know (NAME OF ADOLESCENT) test results either.<br><br>Do you have any questions?<br>You can say yes to the test for (NAME OF ADOLESCENT), or you can say no. It is up to you to decide.<br>Will you allow (NAME OF ADOLESCENT) to take the test for intestinal parasite?		
286	CIRCLE THE APPROPRIATE CODE AND SIGN YOUR NAME.	GRANTED . . . . 1<br>__________<br>(SIGN)<br>PARENT/OTHER RESPONSIBLE ADULT REFUSED . . . . 2<br>(IF REFUSED, GO TO 289)	GRANTED . . . . 1<br>__________<br>(SIGN)<br>PARENT/OTHER RESPONSIBLE ADULT REFUSED . . . . 2<br>(IF REFUSED, GO TO 289)	GRANTED . . . . 1<br>__________<br>(SIGN)<br>PARENT/OTHER RESPONSIBLE ADULT REFUSED . . . . 2<br>(IF REFUSED, GO TO 289)

MICRONUTRIENT FOR WOMEN AGE 15-49

		WOMAN 1	WOMAN 2	WOMAN 3
287	ASK CONSENT FOR INTESTINAL PARASITE TEST FROM RESPONDENT.	As part of this survey, we are asking people all over the country to take a test for intestinal parasite. Intestinal parasite is a serious health problem. This survey will assist the government to develop programs to prevent and treat intestinal parasite. For the test for intestinal parasite, we will need 1 gram of stool. The equipment used to collect stool is clean and completely safe. It has never been used before and will be thrown away after each test. The stool will be transported to the central laboratory, and the result will not be available to you. No one else will be able to know your test results either.<br><br>Do you have any questions?<br>You can say yes to the test, or you can say no. It is up to you to decide.<br>Will you take the test for intestinal parasite ?		
288	CIRCLE THE APPROPRIATE CODE AND SIGN YOUR NAME.	GRANTED . . . . 1<br>______ (SIGN)<br>RESPONDENT REFUSED 2	GRANTED . . . . 1<br>______ (SIGN)<br>RESPONDENT REFUSED 2	GRANTED . . . . 1<br>______ (SIGN)<br>RESPONDENT REFUSED 2
289	CHECK Q.276 = 1 AND Q.278 = 1<br><br>PUT 2ND BARCODE ON VACUTAINER TUBE<br>PUT 3RD BARCODE ON BLOOD TRASMITTAL FORM	AGREE FOR BLOOD TEST<br>PUT THE 1ST BARCODE LABEL HERE<br>NOT PRESENT . . . . 99994<br>REFUSED . . . . 99995<br>OTHER 99996	AGREE FOR BLOOD TEST<br>PUT THE 1ST BARCODE LABEL HERE<br>NOT PRESENT . . . . 99994<br>REFUSED . . . . 99995<br>OTHER 99996	AGREE FOR BLOOD TEST<br>PUT THE 1ST BARCODE LABEL HERE<br>NOT PRESENT . . . . 99994<br>REFUSED . . . . 99995<br>OTHER 99996
290	CHECK Q.281 = 1 AND Q.283 = 1<br><br>PUT 5TH BARCODE ON VACUTAINER TUBE<br>PUT 6TH BARCODE ON BLOOD TRASMITTAL FORM	AGREE FOR URINE TEST<br>PUT THE 4TH BARCODE LABEL HERE<br>NOT PRESENT . . . . 99994<br>REFUSED . . . . 99995<br>OTHER 99996	AGREE FOR URINE TEST<br>PUT THE 4TH BARCODE LABEL HERE<br>NOT PRESENT . . . . 99994<br>REFUSED . . . . 99995<br>OTHER 99996	AGREE FOR URINE TEST<br>PUT THE 4TH BARCODE LABEL HERE<br>NOT PRESENT . . . . 99994<br>REFUSED . . . . 99995<br>OTHER 99996
291	CHECK Q.286 = 1 AND Q.288 = 1<br><br>PUT 8TH BARCODE ON VACUTAINER TUBE<br>PUT 9TH BARCODE ON BLOOD TRASMITTAL FORM	AGREE FOR STOOL TEST<br>PUT THE 7TH BARCODE LABEL HERE<br>NOT PRESENT . . . . 99994<br>REFUSED . . . . 99995<br>OTHER 99996	AGREE FOR STOOL TEST<br>PUT THE 7TH BARCODE LABEL HERE<br>NOT PRESENT . . . . 99994<br>REFUSED . . . . 99995<br>OTHER 99996	AGREE FOR STOOL TEST<br>PUT THE 7TH BARCODE LABEL HERE<br>NOT PRESENT . . . . 99994<br>REFUSED . . . . 99995<br>OTHER 99996
292	GO BACK TO 271 IN NEXT COLUMN OF THIS QUESTIONNAIRE OR IN THE FIRST COLUMN OF AN ADDITIONAL QUESTIONNAIRE; IF NO MORE WOMEN, END INTERVIEW.			
