# Supplementary material for: Factors influencing unmet need for contraception amongst adolescent girls and women in Cambodia
Source: PeerJ. 2020 Oct 7;8:e10065. doi: 10.7717/peerj.10065 (PMC7547592; doi:10.7717/peerj.10065)
Supplement: Supplemental Information 13 [file peerj-08-10065-s013.pdf]

Cambodia - Demographic and Health Survey 2014

National Institute of Statistics (NIS) - Ministry of Planning, Royal Government of Cambodia, Directorate General for Health (DGH) - Ministry of Health, Royal Government of Cambodia

Report generated on: November 9, 2015

Visit our data catalog at: http://microdata.worldbank.org

Sampling

Sampling Procedure

The 2014 CDHS sample is a nationally representative sample of women and men between age 15 and 49 who completed interviews. To achieve a balance between the ability to provide estimates at the subnational level and limiting the sample size, 19 sampling domains were defined, 14 of which correspond to individual provinces and 5 of which correspond to grouped provinces:
• Fourteen individual provinces: Banteay Meanchey, Kampong Cham, Kampong Chhnang, Kampong Speu, Kampong Thom, Kandal, Kratie, Phnom Penh, Prey Veng, Pursat, Siem Reap, Svay Rieng, Takeo, and Otdar Meanchey
• Five groups of provinces: Battambang and Pailin, Kampot and Kep, Preah Sihanouk and Koh Kong, Preah Vihear and Stung Treng, and Mondul Kiri and Ratanak Kiri

The sample of households was allocated to the sampling domains in such a way that estimates of indicators could be produced with precision at the national level, as well as separately for urban and rural areas of the country and for each of the 19 sampling domains.

The sampling frame used for the 2014 CDHS was derived from the list of all enumeration areas (EAs) created for the 2008 Cambodia General Population Census (GPC), provided by NIS. The list had been updated in 2012, and it excluded 241 EAs that are special settlement areas and not ordinary residential areas. It included 28,455 EAs for the entire country. The GPC also created maps that delimited the boundaries of each EA. Overall, 4,245 EAs were designated as urban and 24,210 as rural, with an average size of 99 households per EA.

The survey used a stratified sample selected in two stages. Stratification was achieved by separating every reporting domain into urban and rural areas. Thus, the 19 domains were stratified into a total of 38 sampling strata. Samples were selected independently in every stratum through a two-stage selection process. Implicit stratifications were achieved at each of the lower geographical or administrative levels by sorting the sampling frame according to geographical/administrative order before sample selection and by using a probability proportional to size selection strategy at the first stage of selection.

For further details on sample selection, see Appendix A of the final report.

Response Rate

All of the 611 clusters selected for the sample were surveyed in the 2014 CDHS. A total of 16,356 households were selected, of which 15,937 were found to be occupied during data collection. Among these households, 15,825 completed the Household Questionnaire, yielding a response rate of 99 percent.

In these interviewed households, 18,012 women were identified as eligible for the individual interview. Interviews were completed with 98 percent of these women. Of the 5,484 eligible men identified in every third household, 95 percent were successfully interviewed. There was little variation in response rates by urban-rural residence.

Weighting

Due to the nonproportional allocation of the sample to the different domains and to urban and rural areas, along with possible differences in response rates, sampling weights are required for any analysis using the 2014 CDHS data to ensure the actual representativeness of the survey results at the national as well as the regional level. Since the 2014 CDHS sample was a two-stage stratified cluster sample, sampling weights were calculated based on sampling probabilities separately for each sampling stage and for each cluster.

Sampling weights were adjusted for household nonresponse as well as for individual nonresponse in order to calculate the survey weights. A spreadsheet containing all sampling parameters and selection probabilities was prepared to facilitate the calculation of survey weights. Several sets of survey weights were calculated:
• one set for all households and for women’s individual surveys
• one set for households selected for the male survey and for men’s individual surveys
• one set for women selected for the domestic violence survey
• one set for households in the clusters selected for the micronutrient survey

The differences between the household weights and the individual weights are due to individual nonresponse. The domestic

violence survey weight takes the number of eligible women in the household into account because of the selection of only one woman per household. The final survey weights were normalized so that the total number of weighted cases was equal to the total number of unweighted cases at the national level, for both household weights and individual women’s and men’s weights. The normalized weights are relative weights that are valid for estimating means, proportions, and ratios but are not valid for estimating population totals or pooled data.

Questionnaires

Overview

Four questionnaires were used in the 2014 CDHS: the Household Questionnaire, the Woman's Questionnaire, the Man's Questionnaire, and the Micronutrient Questionnaire. These questionnaires are based on the questionnaires developed by the worldwide Demographic and Health Surveys (DHS) Program and on the questionnaires used during the 2010 CDHS survey. To reflect relevant population and health issues in Cambodia, the questionnaires were adapted during a series of technical meetings with various stakeholders from government ministries and agencies, nongovernmental organizations, and international donors. The final drafts of the questionnaires were discussed at a stakeholders' meeting organized by the National Institute of Statistics. The adapted questionnaires were translated from English into Khmer and pretested in February and March 2014.

The Household Questionnaire was used to list all of the usual members and visitors in the selected households. Basic information was collected on the characteristics of each person listed, including age, sex, education, and relationship to the head of the household. For children under age 18, parents' survival status was determined. The Household Questionnaire was also used to identify women and men eligible for an individual interview.

The Woman's Questionnaire was used to collect information from all women age 15-49 and the Man's Questionnaire was administered to all men age 15-49 living in one-third of the households in the CDHS sample.

The Micronutrient Questionnaire was implemented in a subsample of one-sixth of the sampled clusters for the collection of micronutrient specimens among eligible women and children. Specimens collected included venous blood, urine, and stool samples.

Data Collection

Data Collection Dates

Start	End	Cycle
2014-06	2014-12	N/A

Data Collection Mode

Face-to-face [f2f]

DATA COLLECTION NOTES

TRAINING AND FIELDWORK
The goal of training was to create 19 field teams capable of collecting data for the 2014 CDHS. Each team was responsible for data collection in one of the 19 survey domains (comprising the 23 provinces and the capital city of Phnom Penh). Field teams were composed of five people (5 teams) or six people (14 teams): a team leader, a field editor, two or three female interviewers, and one male interviewer. Nineteen fully staffed field teams would require 114 field personnel, and at the end of training 109 field personnel were retained. Twenty-six days of training included four days of field practice in Kandal province. Data processing personnel (3 data processing supervisors, 10 office editors/coders, 19 data entry operators, and 5 reserves) also attended classroom training.

Training began with the Household Questionnaire and was followed by the Woman's Questionnaire. Additional time was spent reviewing the Household Questionnaire, including consent statements for hemoglobin testing, and conversion of ages and dates of birth from the Khmer calendar to the Gregorian calendar. One week was devoted to additional activities, including the Man's Questionnaire, measurement of women's and children's height and weight, sample implementation and household selection, testing of household salt for iodine, and organization of documents and materials for return to the head office. After completion of training, including field practice, fieldwork was launched and teams disbursed to their assigned provinces.

During the training period, the 19 CDHS team leaders were provided with the cluster information for the provinces in which they would be working so that they could devise a data collection sequence for their sample points. Team leaders were best equipped to perform this task because they hailed from their own provinces. They also conducted the CDHS household listing operation (described in Appendix A) and therefore were well acquainted with the areas in which they would be working. The progression of fieldwork by geographic location had to take into account weather conditions during the rainy season.

Fieldwork supervision was carried out regularly by three CDHS survey coordinators from NIS and MOH along with an ICF Macro consultant. Supervision visits were conducted throughout the six months of data collection and included retrieval of questionnaires from the field. In addition, a quality control program was run by the data processing team to detect key data collection errors for each team. These data checks were used to provide regular feedback to each team based on its specific performance. Data collection was conducted from June 2 to December 12, 2014.

The training and fieldwork for collection of stool, urine, and venous blood samples were conducted separately by UNICEF in collaboration with the Institut de Recherche pour le Dveloppement (France) and Cambodia's Ministry of Agriculture, Forestry, and Fisheries. Details are provided in the micronutrient chapter.

Data Collectors

Name	Abbreviation	Affiliation
National Institute of Statistics	NIS	Ministry of Planning, Royal Government of Cambodia

Data Processing

Data Editing

Completed questionnaires were returned from the field to NIS headquarters, where they were entered and edited by data processing personnel who were specially trained for this task and had also attended questionnaire training of field staff. Data processing personnel included a data processing chief, two assistants, four secondary editors and coordinators, 25 entry operators, and eight office editors.

Data processing for the 2014 CDHS began on 25 personal computers on July 6, 2014, five weeks after the first interviews were conducted. Processing the data concurrently with data collection allowed for regular monitoring of team performance and data quality. Field check tables were generated regularly during the data processing to check various data quality parameters. As a result, feedback was given on a regular basis, encouraging teams to continue in areas of high quality and to correct areas of needed improvement. Feedback was individually tailored to each team. Data entry, which included 100 percent double entry to minimize keying errors, and data editing were completed on January 8, 2015. Data cleaning and finalization were completed on January 23, 2015.

Data Appraisal

Estimates of Sampling Error

The estimates from a sample survey are affected by two types of errors: (1) nonsampling errors and (2) sampling errors. Nonsampling errors are the results of mistakes made in implementing data collection and data processing, such as failure to locate and interview the correct household, misunderstanding of the questions on the part of either the interviewer or the respondent, and data entry errors. Although numerous efforts were made during the implementation of the 2014 Cambodia Demographic and Health Survey (CDHS) to minimize this type of error, nonsampling errors are impossible to avoid and difficult to evaluate statistically.

Sampling errors, on the other hand, can be evaluated statistically. The sample of respondents selected in the 2014 CDHS is only one of many samples that could have been selected from the same population, using the same design and identical size. Each of these samples would yield results that differ somewhat from the results of the actual sample selected. Sampling errors are a measure of the variability between all possible samples. Although the degree of variability is not known exactly, it can be estimated from the survey results.

A sampling error is usually measured in terms of the standard error for a particular statistic (mean, percentage, etc.), which is the square root of the variance. The standard error can be used to calculate confidence intervals within which the true value for the population can reasonably be assumed to fall. For example, for any given statistic calculated from a sample survey, the value of that statistic will fall within a range of plus or minus two times the standard error of that statistic in 95 percent of all possible samples of identical size and design.

If the sample of respondents had been selected as a simple random sample, it would have been possible to use straightforward formulas for calculating sampling errors. However, the 2014 CDHS sample is the result of a multistage stratified design, and, consequently, it was necessary to use more complex formulae. The computer software used to calculate sampling errors for the 2014 CDHS is an SAS program. This program used the Taylor linearization method for variance estimation for survey estimates that are means or proportions. The Jackknife repeated replication method is used for variance estimation of more complex statistics such as fertility and mortality rates.

The Taylor linearization method treats any percentage or average as a ratio estimate, $r = y/x$, where y represents the total sample value for variable y, and x represents the total number of cases in the group or subgroup under consideration.

Note: A more detailed description of estimate of sampling error is presented in APPENDIX B of the survey report.

Other forms of Data Appraisal

Data Quality Tables
- Household age distribution
- Age distribution of eligible and interviewed women
- Age distribution of eligible and interviewed men
- Completeness of reporting
- Births by calendar years
- Reporting of age at death in days
- Reporting of age at death in months
- Nutritional status of children based on the NCHS/CDC/WHO International Reference Population

Note: See detailed data quality tables in APPENDIX C of the report.

File Description

Variable List

Related Materials

Questionnaires

Cambodia Demographic and Health Survey 2014, Household Questionnaire

Title	Cambodia Demographic and Health Survey 2014, Household Questionnaire
Author(s)	National Institute of Statistics (NIS), Ministry of Planning, Royal Government of Cambodia Directorate General for Health (DGH), Ministry of Planning, Royal Government of Cambodia
Country	Cambodia
Language	English
Filename	KHM_2014_DHS_hh_questionnaire.pdf

Cambodia Demographic and Health Survey 2014, Woman's Questionnaire

Title	Cambodia Demographic and Health Survey 2014, Woman's Questionnaire
Author(s)	National Institute of Statistics (NIS), Ministry of Planning, Royal Government of Cambodia Directorate General for Health (DGH), Ministry of Planning, Royal Government of Cambodia
Country	Cambodia
Language	English
Filename	KHM_2014_DHS_women_questionnaire.pdf

Cambodia Demographic and Health Survey 2014, Man's Questionnaire

Title	Cambodia Demographic and Health Survey 2014, Man's Questionnaire
Author(s)	National Institute of Statistics (NIS), Ministry of Planning, Royal Government of Cambodia Directorate General for Health (DGH), Ministry of Planning, Royal Government of Cambodia
Country	Cambodia
Language	English
Filename	KHM_2014_DHS_men_questionnaire.pdf

Cambodia Demographic and Health Survey 2014, Micronutrient Questionnaire

Title	Cambodia Demographic and Health Survey 2014, Micronutrient Questionnaire
Author(s)	National Institute of Statistics (NIS), Ministry of Planning, Royal Government of Cambodia Directorate General for Health (DGH), Ministry of Planning, Royal Government of Cambodia
Country	Cambodia
Language	English
Filename	KHM_2014_DHS_micronutrient_questionnaire.pdf

Reports

Cambodia Demographic and Health Survey 2014, Report

Title	Cambodia Demographic and Health Survey 2014, Report
Author(s)	National Institute of Statistics, Ministry of Planning, Phnom Penh, Cambodia Directorate General for Health, Ministry of Health, Phnom Penh, Cambodia The DHS Program, ICF International, Rockville, Maryland, USA
Date	2015-09-01
Country	Cambodia
Language	English

Table of contents

TABLES AND FIGURES ... ix
FOREWORD ... xvii
ACKNOWLEDGMENTS ... xix
MAP OF CAMBODIA ... xx

1 INTRODUCTION ... 1
1.1 Geodemography, History, and Economy ... 1
1.1.1 Geodemography ... 1
1.1.2 History ... 2
1.1.3 Economy ... 2
1.2 Health Status and Policy ... 2
1.3 Objective and Survey Organization ... 4
1.4 Sample Design ... 4
1.5 Questionnaires ... 5
1.6 Training and Fieldwork ... 7
1.7 Biomarker Testing ... 7
1.7.1 Anthropometric Measurement ... 7
1.7.2 Hemoglobin Testing... 8
1.7.3 Micronutrient Testing ... 8
1.8 Data Processing ... 8
1.9 Sample Coverage ... 8

2 HOUSEHOLD POPULATION AND HOUSING CHARACTERISTICS ... 11
2.1 Characteristics of the Household Population ... 11
2.1.1 Age and Sex Composition ... 11
2.1.2 Household Composition ... 13
2.2 Education of the Household Population ... 14
2.3 Housing Characteristics ... 17
2.3.1 Water Supply ... 18
2.3.2 Sanitation Facilities ... 20
2.3.3 Hand Washing ... 21
2.3.4 Flooring Material and Cooking Arrangements ... 22
2.4 Household Possessions ... 23
2.5 Household Wealth ... 23
2.6 Birth Registration ... 24
2.7 Children's Living Arrangements, Orphanhood, and School Attendance by Survivorship of Parents 25
2.7.1 Children's Living Arrangements and Orphanhood ... 25
2.7.2 School Attendance by Survivorship of Parents ... 27

3 UTILIZATION OF HEALTH SERVICES FOR ACCIDENT, ILLNESS, OR INJURY 29
3.1 Accidental Death or Injury ... 29
3.1.1 Frequency of Accidental Death or Injury ... 29
3.1.2 Type of Accident ... 30
3.2 Prevalence and Severity of Illness or Injury ... 32
3.3 Treatment Sought for Illness or Injury ... 33
3.4 Utilization of Health Care Facilities ... 34
3.5 Cost for Health Care ... 36
3.5.1 Distribution of Cost for Health Care ... 36
3.5.2 Expenditures for Health Care ... 36
3.5.3 Sources of Money for Health Care Expenditures ... 38

4 DISABILITY ... 41
4.1 Disability among the General Household Population ... 41
4.2 Disability among Ill or Injured Household Members ... 43
4.3 Disability and Employment ... 44

5 RESPONDENT CHARACTERISTICS ... 47
5.1 Characteristics of Survey Respondents ... 47
5.2 Educational Attainment and Literacy ... 49
5.3 Access to Mass Media ... 52
5.4 Employment ... 55
5.4.1 Employment Status ... 55
5.4.2 Occupation ... 57
5.4.3 Earnings, Employers, and Continuity of Employment ... 59
5.5 Health Insurance ... 60
5.6 Use of Tobacco ... 62

6 FERTILITY ... 67
6.1 Current Fertility Levels and Differentials ... 67
6.2 Fertility Trends ... 70
6.2.1 Comparison of Current and Cumulative Fertility Levels ... 70
6.2.2 Retrospective Data ... 70
6.2.3 Comparison with Previous CDHS ... 71
6.3 Children Ever Born and Living ... 72
6.4 Birth Intervals ... 73
6.5 Age at First Birth ... 75
6.6 Teenage Pregnancy and Motherhood ... 76

7 PRACTICE OF ABORTION ... 79
7.1 Number of Lifetime Induced Abortions ... 79
7.2 Practice of Abortion in the Past Five Years ... 81
7.2.1 Pregnancy Duration at the Time of Abortion ... 81
7.2.2 Place of Abortion ... 82
7.2.3 Persons Who Helped with the Abortion ... 83
7.2.4 Method Used for the Abortion ... 83

8 FAMILY PLANNING ... 85
8.1 Knowledge of Contraceptive Methods ... 85
8.2 Current Use of Contraceptive Methods ... 86
8.3 Use of Social Marketing Brands ... 89
8.4 Knowledge of Fertile Period ... 90
8.5 Timing of Sterilization... 91
8.6 Source of Family Planning Methods ... 91
8.7 Informed Choice ... 92
8.8 Future Use of Contraception ... 93
8.9 Exposure to Family Planning Messages ... 94
8.10 Contact of Nonusers with Family Planning Providers ... 95

9 OTHER PROXIMATE DETERMINANTS OF FERTILITY ... 97
9.1 Marital Status ... 97
9.2 Polygamy ... 98
9.3 Age at First Union ... 99
9.4 Age at First Sexual Intercourse ... 102
9.5 Recent Sexual Activity ... 105
9.6 Postpartum Amenorrhea, Abstinence, and Insusceptibility ... 108
9.7 Termination of Exposure to Pregnancy ... 109

10 FERTILITY PREFERENCES ... 111
10.1 Desire for More Children ... 111
10.2 Need and Demand for Family Planning Services ... 114
10.3 Ideal Family Size ... 116
10.4 Fertility Planning ... 118

11 ADULT AND MATERNAL MORTALITY ... 121
11.1 Data Quality Issues ... 121
11.2 Adult Mortality ... 123
11.3 Maternal Mortality ... 123

12 INFANT AND CHILD MORTALITY ... 127
12.1 Assessment of Data Quality ... 128
12.2 Levels and Trends in Childhood Mortality ... 128
12.3 Socioeconomic Differentials in Childhood Mortality ... 129
12.4 Demographic Differentials in Mortality ... 131
12.5 Perinatal Mortality ... 132
12.6 High-Risk Fertility Behavior ... 133

13 MATERNAL HEALTH ... 137
13.1 Antenatal Care ... 137
13.1.1 Source of Antenatal Care ... 137
13.1.2 Components of Antenatal Care ... 139
13.1.3 Tetanus Toxoid Vaccinations ... 141
13.2 Childbirth and Delivery ... 141
13.2.1 Place of Delivery... 142
13.2.2 Assistance at Delivery ... 143
13.3 Postnatal Care and Practices ... 144
13.4 Perceived Problems in Accessing Women's Health Care ... 149

14 CHILD HEALTH ... 151
14.1 Child's Size at Birth ... 151
14.2 Immunization of Children ... 152
14.3 Acute Respiratory Infection ... 155
14.4 Fever ... 157
14.5 Diarrhea ... 158
14.6 Feeding Practices ... 161
14.7 Knowledge of ORS Packets ... 163
14.8 Stool Disposal ... 163

15 EARLY CHILDHOOD EDUCATION AND DEVELOPMENT ... 165
15.1 Early Childhood Education and Learning ... 165
15.2 Adequate Care for Young Children ... 169
15.3 Early Childhood Development ... 170

16 NUTRITION OF CHILDREN AND WOMEN ... 173
16.1 Nutritional Status of Children ... 174
16.1.1 Measurement of Nutritional Status among Young Children ... 174
16.1.2 Measures of Child Nutritional Status ... 175
16.1.3 Trends in Children's Nutritional Status ... 177
16.2 Initiation of Breastfeeding ... 178
16.3 Breastfeeding Status by Age ... 180
16.4 Duration of Breastfeeding ... 181
16.5 Types of Complementary Foods ... 183
16.6 Infant and Young Child Feeding (IYCF) Practices ... 184
16.7 Prevalence of Anemia in Children ... 187
16.8 Micronutrient Intake among Children ... 189
16.9 Use of Iodized Salt ... 191
16.10 Nutritional Status of Women ... 192
16.11 Prevalence of Anemia in Women ... 194
16.12 Micronutrient Intake among Mothers ... 196

17 MICRONUTRIENTS ... 199
17.1 Coverage of Micronutrient Testing ... 200
17.2 Iron, Hemoglobin, and Parasitic Infections ... 200
17.2.1 Anemia and Iron Status in Mothers ... 201
17.2.2 Anemia and Iron Status in Children ... 201
17.2.3 Intestinal Parasite Infection ... 203
17.3 Vitamin and Calcium Deficiency ... 203
17.3.1 Vitamin and Calcium Deficiency among Mothers ... 203
17.3.2 Vitamin and Calcium Deficiency among Children ... 203
17.4 Urine Iodine Concentration ... 205

18 HIV/AIDS-RELATED KNOWLEDGE, ATTITUDES, AND BEHAVIOR ... 207
18.1 Knowledge of HIV/AIDS and of Transmission and Prevention Methods... 207
18.1.1 Awareness of AIDS ... 207
18.1.2 HIV Prevention Methods ... 208
18.1.3 Knowledge about Transmission ... 210
18.1.4 Knowledge of Mother-to-Child Transmission ... 213
18.2 Stigma Associated with AIDS and Attitudes Related to HIV/AIDS ... 215
18.3 Attitudes towards Negotiating Safer Sex ... 217
18.4 Multiple Sexual Partnerships ... 218
18.5 Testing for HIV ... 223
18.6 Reports of Recent Sexually Transmitted Infections ... 227
18.7 Injections ... 229
18.8 HIV/AIDS-Related Knowledge and Behavior among Youth ... 230
18.8.1 Knowledge about HIV/AIDS and Source for Condoms ... 231
18.8.2 Age at First Sex and Condom Use at First Sexual Intercourse ... 232
18.8.3 Recent Sexual Activity ... 234
18.8.4 Multiple Sexual Partnerships ... 235
18.8.5 HIV Testing ... 236

19 WOMEN'S EMPOWERMENT AND DEMOGRAPHIC AND HEALTH OUTCOMES ... 239
19.1 Employment and Forms of Earnings ... 239
19.2 Control over Women's and Men's Earnings ... 240
19.3 Participation in Household Decision Making ... 243
19.4 Ownership of Assets ... 247
19.5 Attitudes towards Wife Beating ... 249
19.6 Women's Empowerment Indicators ... 252
19.7 Current Use of Contraception by Women's Status ... 253
19.8 Ideal Family Size and Unmet Need by Women's Status ... 254
19.9 Reproductive Health Care and Women's Empowerment Status ... 255

20 DOMESTIC VIOLENCE ... 257
20.1 Measurement of Violence ... 257
20.1.1 Use of Valid Measures of Violence ... 257
20.1.2 Ethical Considerations in the 2014 CDHS ... 258
20.1.3 Subsample for the Violence Module ... 259
20.2 Experience of Physical Violence ... 259
20.3 Perpetrators of Physical Violence ... 261
20.4 Experience of Sexual Violence ... 262
20.5 Perpetrators of Sexual Violence ... 262
20.6 Age at First Experience of Sexual Violence ... 263
20.7 Experience of Different Forms of Violence ... 263
20.8 Violence during Pregnancy ... 264
20.9 Marital Control by Spouse ... 266
20.10 Forms of Spousal Violence ... 268
20.11 Spousal Violence by Background Characteristics ... 269
20.12 Violence by Spousal Characteristics and Women's Empowerment Indicators ... 271
20.13 Recent Spousal Violence by Any Husband or Partner ... 273
20.14 Onset of Spousal Violence ... 273
20.15 Physical Consequences of Spousal Violence ... 274
20.16 Violence by Women against Their Husband ... 274
20.17 Help-seeking Behavior by Women Who Experience Violence ... 276

REFERENCES ... 279
APPENDIX A: SAMPLE IMPLEMENTATION ... 281
A.1 Introduction ... 281
A.2 Sampling Frame ... 281
A.3 Sampling Methodology and Procedure ... 282
A.4 Sampling Probabilities ... 284
APPENDIX B: ESTIMATES OF SAMPLING ERRORS ... 291
APPENDIX C: DATA QUALITY TABLES ... 317
APPENDIX D: PERSONS INVOLVED IN THE 2014 CAMBODIA DEMOGRAPHIC AND HEALTH SURVEY 323
APPENDIX E: QUESTIONNAIRES ... 327

Filename http://dhsprogram.com/pubs/pdf/FR312/FR312.pdf

Cambodia 2014 Demographic and Health Survey, Key Findings

Title	Cambodia 2014 Demographic and Health Survey, Key Findings
Author(s)	The DHS Program
Date	2015-09-01
Country	Cambodia
Language	English
Description	This report summarises the findings of the 2014 Ghana Demographic and Health Survey (2014 GDHS), implemented by the Ghana Statistical Service (GSS), the Ghana Health Service (GHS), and the National Public Health and Reference Laboratory (NPHRL) of the GHS.
Filename	http://dhsprogram.com/pubs/pdf/SR226/SR226.pdf

Fast Facts from The 2014 Cambodia Demographic and Health Survey

Title	Fast Facts from The 2014 Cambodia Demographic and Health Survey
Author(s)	The DHS Program
Date	2015-09-01
Country	Cambodia
Language	English
Filename	http://dhsprogram.com/pubs/pdf/DM73/DM73.pdf

Cambodia 2014 Demographic and Health Survey, Fact Sheet

Title	Cambodia 2014 Demographic and Health Survey, Fact Sheet
Author(s)	The DHS Program
Date	2015-09-01
Country	Cambodia
Language	English
Filename	http://dhsprogram.com/pubs/pdf/GF33/GF33.pdf

Other materials

Reading and Understanding Tables from the 2014 Cambodia Demographic and Health Survey (CDHS)

Title	Reading and Understanding Tables from the 2014 Cambodia Demographic and Health Survey (CDHS)
Author(s)	The DHS Program
Date	2015-09-01
Country	Cambodia
Language	English
Filename	http://dhsprogram.com/pubs/pdf/DM68/DM68.pdf
