# Supplementary material for: Factors influencing unmet need for contraception amongst adolescent girls and women in Cambodia
Source: PeerJ. 2020 Oct 7;8:e10065. doi: 10.7717/peerj.10065 (PMC7547592; doi:10.7717/peerj.10065)
Supplement: Supplemental Information 14 [file peerj-08-10065-s014.docx]

**ARCHIVE WHERE STUDY IS ORIGINALLY STORED**

The DHS Program
<http://dhsprogram.com/data/available-datasets.cfm>

**ACCESS AUTHORITY**

| **Name** | **Email** | **URL** |
| --- | --- | --- |
| The DHS Program | [archive@dhsprogram.com](mailto:archive@dhsprogram.com) | [http://www.DHSprogram.com](http://www.dhsprogram.com/) |

**CONTACTS**

| **Name** | **Affiliation** | **Email** | **URL** |
| --- | --- | --- | --- |
| Information about The DHS Program | The DHS Program | [reports@DHSprogram.com](mailto:reports@DHSprogram.com) | [http://www.DHSprogram.com](http://www.dhsprogram.com/) |
| General Inquiries | The DHS Program | [info@dhsprogram.com](mailto:info@dhsprogram.com) | [http://www.DHSprogram.com](http://www.dhsprogram.com/) |
| Data and Data Related Resources | The DHS Program | [archive@dhsprogram.com](mailto:archive@dhsprogram.com) | [http://www.DHSprogram.com](http://www.dhsprogram.com/) |
