# Supplementary material for: Factors influencing unmet need for contraception amongst adolescent girls and women in Cambodia
Source: PeerJ. 2020 Oct 7;8:e10065. doi: 10.7717/peerj.10065 (PMC7547592; doi:10.7717/peerj.10065)
Supplement: Supplemental Information 15 [file peerj-08-10065-s015.docx]

Variable names

| v394 | Visited health facility in last 12 months |
| --- | --- |
| V467d | Distance to health facility for getting medical help for self |
| regionnew | Urban/rural region |
| agenew | Age groups |
| v201 | Total children even born (parity) |
| newwhc | Person who decides on respondent’s health care |
| newwhhp | Person who decides on large household purchases |
| v714 | Respondent currently working |
| v621 | Husband’s desire for children |
| V384a | Heard about family planning on radio in the last few months |
| V384b | Heard about family planning on TV in the last few months |
| v394 | Visited health facility in the last 12 months |

use "D:\Farwa analysis\data 2014 DHS Cambodia\KH DHS\khir72dt\KHIR72FL.DTA", clear

gen wgt = v005/1000000

tab v025 [iweight = wgt]

generate hadsex = v525

recode hadsex (0=0) (2/96=1) (*=.)

label define hadsex 0 "had no sex" 1 "had sex"

tab hadsex

drop if hadsex==0

tab hadsex

save "D:\Farwa analysis\data analysis 11042018.dta", replace

generate regionnew = v025

recode regionnew (2=0) (1=1)

label define regionnew 0 "rural" 1 "urban"

tab regionnew

fre regionnew

label values regionnew regionnew

generate agenew = v012

drop if (agenew >29)

fre agenew

recode agenew (15/19 = 1) (20/24 =2) (25/29 =0)

label define agenew 1 "15-19" 2 "20-24" 0 "25-29"

label values agenew agenew

***total unmet need for contraception , new coding***

gen unmetneedtotal=1 if v626a==1 | v626a==2

replace unmetneedtotal=0 if (v626a>2 & v626a~=.)

***women's autonomy coding-to be used ***

generate newwhc = v743a

recode newwhc (1=0) (2=1) (4=2) (5/6=3)

label define newwhc 0 "respondant alone" 1 "together" 2 "husband alone" 3 "someone else"

label values newwhc newwhc

generate newwhhp = v743b

recode newwhhp (1=0) (2=1) (4=2) (5/6=3)

label define newwhhp 0 "respondant alone" 1 "together" 2 "husband alone" 3 "someone else"

label values newwhhp newwhhp

**wealth index***

generate wealthindex = v190

replace wealthindex=. if v190==7

recode wealthindex (1=4)(2=3)(3=2)(4=1)(5=0)(7=.)

label define wealthindex 0 "richest" 1 "richer" 2 "middle" 3 "poorer" 4 "poorest"

label values wealthindex wealthindex

***education***

generate education = v106

fre education

recode education (3=0)(2=1) (1=2)(0=3)

label define education 0 "higher" 1 "secondary" 2"primary" 3 "no education"

label values education education

***parity v201***

generate parity=v201

label define parity 0 "no children" 1 "1-2 children" 2 "3 or more children"

recode parity (0=0) (1/2=1) (3/7=2)

label values parity parity

fre parity

***for descriptive analyses and bivariate analyses (Chi square), choose one variable at a time***

tab unmetneedtotal agenew, col

tab unmetneedtotal agenew, chi

***surveyweights***

svyset [pw=wgt],psu (v021) strata (v022)

***FINAL MODEL with PARITY as categorical var***

svy: logistic unmetneedtotal i.regionnew i.agenew i.parity i.newwhc i.newwhhp i.v714 i.v621 i.v384a i.v384b i.v394 i.v467d, base

estat gof, group (10)
